# Nesting of colon and ovarian cancer cells in the endothelial niche is associated with alterations in glycan and lipid metabolism

**DOI:** 10.1038/srep39999

**Published:** 2017-01-04

**Authors:** Anna Halama, Bella S. Guerrouahen, Jennifer Pasquier, Noothan J. Satheesh, Karsten Suhre, Arash Rafii

**Affiliations:** 1 Department of Physiology and Biophysics, Weill Cornell Medicine-Qatar, Qatar-Foundation, P.O. Box 24144, Doha, Qatar; 2 Stem Cell and Microenvironment Laboratory, Weill Cornell Medicine-Qatar, Education City, Qatar Foundation, Doha, Qatar; 3 Department of Genetic Medicine, Weill Cornell Medicine-Qatar, New York, NY 10065, USA; 4 Translational Medicine Division-Research Department, Sidra Medical and Research Center, P.O. Box 26999, Doha, Qatar; 5 Institute of Bioinformatics and Systems Biology, Helmholtz Zentrum München, German Research Center for Environmental Health, Neuherberg, Germany; 6 Department of Genetic Medicine and Obstetrics and Gynecology, Weill Cornell Medical College, Stem Cell and Microenvironment Laboratory, Weill Cornell Medical College in Qatar, Qatar-Foundation, P.O. Box 24144, Doha, Qatar

## Abstract

The metabolic phenotype of a cancer cell is determined by its genetic makeup and microenvironment, which dynamically modulates the tumor landscape. The endothelial cells provide both a promoting and protective microenvironment – a niche for cancer cells. Although metabolic alterations associated with cancer and its progression have been fairly defined, there is a significant gap in our understanding of cancer metabolism in context of its microenvironment. We deployed an *in vitro* co-culture system based on direct contact of cancer cells with endothelial cells (E4^+^EC), mimicking the tumor microenvironment. Metabolism of colon (HTC15 and HTC116) and ovarian (OVCAR3 and SKOV3) cancer cell lines was profiled with non-targeted metabolic approaches at different time points in the first 48 hours after co-culture was established. We found significant, coherent and non-cell line specific changes in fatty acids, glycerophospholipids and carbohydrates over time, induced by endothelial cell contact. The metabolic patterns pinpoint alterations in hexosamine biosynthetic pathway, glycosylation and lipid metabolism as crucial for cancer – endothelial cells interaction. We demonstrated that “Warburg effect” is not modulated in the initial stage of nesting of cancer cell in the endothelial niche. Our study provides novel insight into cancer cell metabolism in the context of the endothelial microenvironment.

Cancer cells are evolving in and interacting with a complex environment composed of numerous different cell types including fibroblasts, epithelial and endothelial cells, pericytes, myofibroblasts and infiltrating cells of the immune system, which together shape the cancer microenvironment^1^. Dynamic changes in the tumor landscape are associated with a bidirectional communication between the cancer cells and non-malignant cells in their vicinity. Increased nutritional demands of metabolically active cancer cells requires growth of new blood vessels, which serves on one hand to supply the essential molecules and oxygen and on the other, to remove the toxic byproducts of cancer cell metabolism^2^. To achieve this need, cancer cells stimulate new blood vessel formation and growth (angiogenesis) through activation of pro-angiogenic signaling pathways, which is commonly accepted as a hallmark of cancer^3^. The success of these interactions with neighboring cells and tissues plays a critical role in promoting cancer growth, its invasiveness and formation of metastatic lesions^4^.

Recently, modulatory effect of tumor microenvironment on cancer cell metabolism was reported^5^, as well as metabolic alterations associated with metastasis^6^, which suggests tight regulation of tumor invasiveness by the microenvironment *via* metabolic - oncogenic signaling crosstalk’s. The abnormal glycolitic activity assocaited with lactate production, was recognized as specific characteristics of cancer metabolism by Otto Warburg in the late 1920s^7^. Proliferating tumor cells rely on increased aerobic glycolysis to produce energy and to enable the supply of building blocks that are essential for highly proliferating cells. It has been shown that abnormal vascularization of the tumor is promoting hypoxic conditions, which in turn can cause an increase in glucose uptake and lactate production^8^. The “Warburg effect” is one of the numerous of metabolic switches, identified in wide variaty of *in vitro*^9^ and clinical studies^10^, which may potentially be regulated by the tumor microenvironment. Those interactions, although highly important from the perspective of identification of novel druggable targets and/or recognition of causal resistance mechanisms, are so far poorly defined.

The monitoring of interactions between cancer cells and their microenvironment is limited *in vivo* due to the numerous of systemic (organismal) features, which might dominate the signal. We previously established an *in vitro* model consisting of co-culture of cancer and endothelial cells^11^,^12^,^13^. We selected endothelial cells E4^+^EC previously created by transfection of the Primary Endothelial Cells (PECs) with the adenoviral *E4ORF1* gene^14^. The E4^+^EC cells exhibit chronic, low activation of Akt signaling^14^, which is a known feature of tumor endothelial cells^15^. Using this model we can avoid the supplementation of the media with serum and cytokines in our co-culture experiments, which is an essential condition to unbiased approach to metabolomics changes.

In the present study we used a co-culture system to investigate the impact of endothelial niche on cancer cell metabolism. In a previous study, we observed significant metabolic differences between colon and ovarian cancer cells^16^. Here, we ask whether the endothelial environment modulates cancer cell metabolism in a consistant manner, independent of the cell line specific features. We deployed non-targeted metabolomics platforms of Metabolon providing a broad coverage of metabolites from eight main metabolic pathways including amino acid, carbohydrate, cofactors and vitamins, energy, lipid, nucleotide, peptide and xenobiotics. The metabolic alterations were monitored in four different cancer cell lines, including two from colon and two from ovarian origin over a period of two days at different time points (6 h, 18 h, 24 h and 48 h) after establishing co-culture with endothelial cells. We identified metabolites displaying coherent and non-cell line specific changes over time pointing toward glycerophospholipid, fatty acid metabolism and glycosylation as pathways impacted by the endothelial niche and involved in cancer-endothelium interactions. Our findings reveal a new insight into cancer cell metabolism in context of its microenvironment.

## Materials and Methods

### Cell culture

Established cancer cell lines were obtained from the American Type Culture Collection (ATCC, Manassas, VA, USA). The human ovarian adenocarcinoma cell lines SKOV3 (HTB-77) and OVCAR3 (HTB-161) were grown in Dulbecco’s modified Eagle medium high glucose (Hyclone, Thermo Scientific) supplemented with 10% fetal bovine serum (FBS; Hyclone, Thermo Scientific), 1% penicillin–streptomycin solution (Sigma), 2 mM L-glutamine (Sigma) and 1X non-essential amino acids (Hyclone, Thermo Scientific). The human colorectal adenocarcinoma cell lines HCT15 (ATCC CCL 225) and HCT116 (CCL247) were maintained in McCoy’s 5 A medium supplemented with 10% heat-inactivated FBS, 2 mM L-glutamine, and 1% penicillin–streptomycin solution, as previously described^16^. The endothelial cells E4ORF1+ECs (E4+ECs) were generated by lentiviral introduction of the adenoviral E4ORF1 gene into the primary endothelial cells, which enables maintenance of long-term survival^14^. The E4^+^ECs express green fluorescence protein (GFP). Cell were grown in M199 medium (Gibco, Life Technologies) supplemented with 20% FBS, 1% penicillin–streptomycin solution (Sigma), 4 mM L-glutamine (Sigma), 20 Units/mL heparin (Sigma) and 20 μg/mL β Endothelial Cell Growth Factor (βECGF, Sigma).

### Co-culture and sample processing

**Figure 1:**
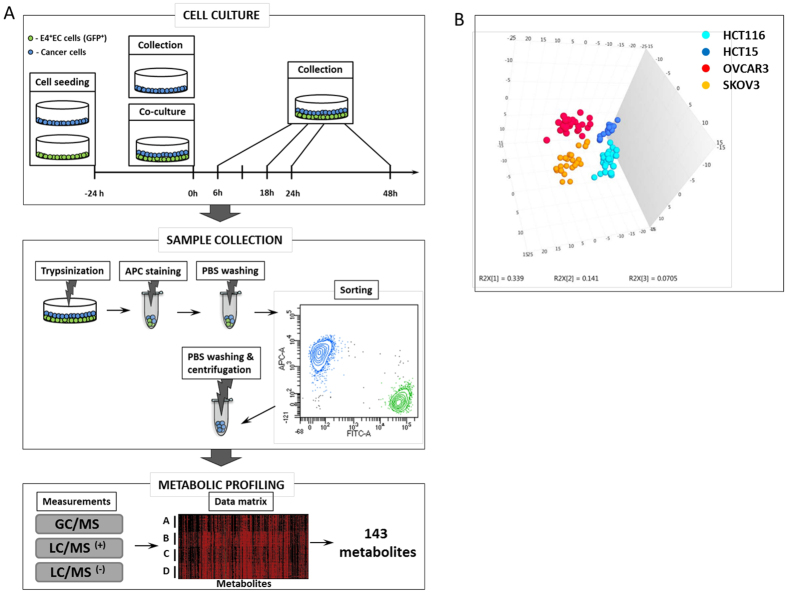
The overview of experimental settings. (**A**) Experimental design. Cancer cells from ovarian (SKOV3 and OVCAR3) and colon (HCT15 and HCT116) were co-cultured with GFP^+^ endothelial cells (E4^+^EC) for 6, 18, 24 and 48 hours. Cells were trypsinized, stained with APC-EpCAM and separated by sorting. The control cancer cells at time 0, which were not cultivated with E4^+^EC, were also sorted. The metabolite intensities were measured on Metabolon platform. Only metabolites, which were present in all cell lines, were used for further analysis. (**B**) PCA plot is showing strong separation reflecting cancer cell line.

For the experiments, cells were trypsinized, washed with PBS and cultivated in M199 medium supplemented with 1% penicillin–streptomycin solution (Sigma), 4 mM L-glutamine (Sigma), 50 μg/mL heparin (Sigma) and 20 μg/mL βECGF (Sigma), to avoid the effect of culture milieu on metabolic profile. The E4^+^EC cells were seeded in the T25 flask 24 h prior the co-culture with cancer cells, to form a dense monolayer. The cancer cells were seeded on the top of E4+EC monolayer. Samples were collected by trypsinization at different time points (6 h, 18 h, 24 h, and 48 h) and washed with PBS. Cells were resuspended in 50 μL of staining media (PBS1X/FBS 5%/EDTA 2 mM) and incubated with fluorochrome-conjugated antibody (CD326, EpCAM, alexa fluor, Biolegend) for 30 minutes at 4 °C and washed with PBS. The E4^+^EC and cancer cell suspension was separated using Fluorescence Activated Cell Sorting (FACS). Cancer cells cultivated in the absence of E4^+^EC, were used as control (T0). Five replicates for each conditions were prepared. The experimental design is illustrated in Fig. 1A.

### Cell sorting

Separation of cancer cells from E4^+^EC was performed using Activated Cell Sorting (FACS) on an SORP FACSAria II (BD Biosciences). The cancer cells were separated from the E4^+^ECs based on AF 647 fluorescence (APC) by 640 nm red laser and 670/14 nm emission and GFP fluorescent by 488 nm blue laser and 525/50 nm emission. Doublets were excluded by FSC-W × FSC-H and SSC-W × SSC-H analysis; Data were processed with FACSDiva 7 software (BD Biosciences). Fluorescent minus one (FMO), showing the level of background that is present in a particular fluorescent detector, was used for gating. In total 100 samples were collected (25 samples/cell line).

### Metabolic profiling

The sample processing and metabolite measurements were performed as previously described^16^. Briefly, 100 of frozen pellets were sent to Metabolon Inc. (Durham, NC, USA) for metabolic profiling based on ultra-high-performance liquid chromatography-mass spectrometry (UPLC-MS) and gas chromatography-mass spectrometry (GC-MS) approaches^17^. The cell pellets were extracted using a series of organic and aqueous solvents to remove the protein fraction. Each sample extract was divided into equal parts to be measured using two methods - UPLC-MS and GC-MS. Samples were evaporated, frozen and vacuum-dried.

#### Sample measurements on UPLC-MS platform

Before measurement with UPLC-MS, samples were reconstituted in an acidic or basic solution compatible with LC- solvents. The samples were assayed using a Waters ACQUITY UPLC system and a Thermo Fisher Scientific Orbitrap Elite high resolution/accurate mass spectrometercomposed of a heated electrospray ionization (HESI) source and an Orbitrap mass analyzer operating at 30,000 mass resolution. Two different extract reconstitutions were performed for sample measurements in positive and negative mode. Extracts reconstituted in acidic conditions were gradient eluted using water/methanol solvent containing 0.1% formic acid followed by measurements in acidic positive ion optimized conditions. Extracts reconstituted in basic conditions were gradient eluted using water/methanol solvent containing 6.5 mM ammonium bicarbonate followed by measurements in basic negative ion optimized conditions.

#### Sample measurements on GC-MS platform

Before measurement with GC–MS samples extracts were re-dried under vacuum for at least 24 h and derivatized using N, O-Bis(trimethylsilyl)trifluoroacetamide (BSTFA) under dried nitrogen conditions. The samples were assayed using Thermo-Finnigan Trace DSQ fast-scanning single-quadrupole mass spectrometer instrument. Sample separation was performed on a 5% diphenyl/95% dimethyl polysiloxane GC column, with helium as the carrier gas. The electron impact ionization was performed prior to mass spectrometric analysis and detection. The instrument was tuned and calibrated daily for mass resolution and mass accuracy.

#### Metabolomics data

The metabolite identification was performed as described previously^16^. Briefly, the metabolite peaks obtained after measurements from UPLC-MS and GC-MS platform were identified using Metabolon’s propriety peak integration software. Identification of metabolites was performed by comparison of the obtained data to the library entries of purified standards or unknown recurrent entities. The combination of chromatographic properties and mass spectra provided an indication of a match to the specific compound on known or unknown entity as well as isobaric entity. The unknown metabolites, labeled as “X – number” (for example X – 14577), are reproducibly detectable molecules, for which the chemical structure has not been identified yet. The isobaric entity is representing the total ion counts of components having the same retention time, mass-to-charge ratio (m/z), and GC-MS or UPLC-MS spectra, that the individual contributions of the compounds to the peak cannot be determined. The series of quality control (QC) and curation processes were conducted to ensure data quality. Library matches for each compound were checked for each sample, and manually corrected if necessary. Sample measurements were obtained during a 5-day period, and data was normalized to correct variations resulting from inter-day tuning differences in the instrument. Each compound was corrected in a run-day.

#### Quality check of metabolomics data

We applied principal component analysis (PCA), an unsupervised statistical analysis that converts high-dimensional data into fewer dimensions with maintenance of the variance from the original data, prior to the statistical data analysis to inspect the quality of the metabolic data. The PCA enables visual examination of sample distribution in the PC space. The PCA was carried out using software SIMCA version 14. The 100 samples clustered into four groups based on the cell line, among which we identified two outliers in SKOV3 cell line, clustered with HCT15 cell line as indicated in supplementary Figure S1. The outliers were excluded from the dataset prior statistical analysis.

### Statistical analysis

Statistical analysis was performed in R version 3.1.3 and R-Studio version 0.97.551. We applied a mixed effects linear regression model for each metabolite, with dependent variable metabolite intensity and fixed effect time, and a random effect identifying the cell line. Dependent variables (metabolite intensities) were log-scaled and then *z*-scored before computing the statistics. Metabolites with >20% missing values in at least one cell line were removed from the dataset. In total 143 metabolites were used for the analysis. Significance was defined at a stringent Bonferroni level of p < 3.49 × 10^−4^ (p < 0.05/143).

We tested two different models of time-dependence: (1) treating time as a quantitative independent variable and (2) pair-wise comparisons of all time-points post-co-culture to time point zero.

We showed previously that including all ratios between metabolites into the analysis can reduce the overall variance and allow for the identification of biologically relevant pathways, provided conservative p-gain statistics are used^18^. We therefore also examined the time-resolved effect of endothelial cells on metabolite ratios, using mixed effects linear regression model for each pair of metabolites, time as well as the fixed effects and cell line. In total, we calculated 10,153 metabolic ratios (143 * 142/2). We considered metabolite ratios as significantly altered if they matched following conditions: p-value < 4.92 × 10^−06^ (p < 0.05/10,153) and simultaneously p-gain > 1.01 × 10^05^ (p-gain > 10 * 10,153).

## Results

### Metabotype of cancer cell remains unique after contact with endothelial cells

We identified 143 metabolites present in all four cancer cell lines including two from ovarian (OVCAR3 and SKOV3) and two from colon (HCT15 and HCT116) cancer. The PCA results showed clear separation into four groups reflecting four cell lines as illustrated in the Fig. 1B. This observation suggests a dominant effect of the cell line on metabotype compared to a time-resolved contact with endothelial cells. We further ask whether the endothelial cells trigger metabolic changes in cancer cells which would be common among all the cell lines.

### Endothelial cells are triggering coherent and non-cell line specific changes in cancer cell metabolism

**Figure 2:**
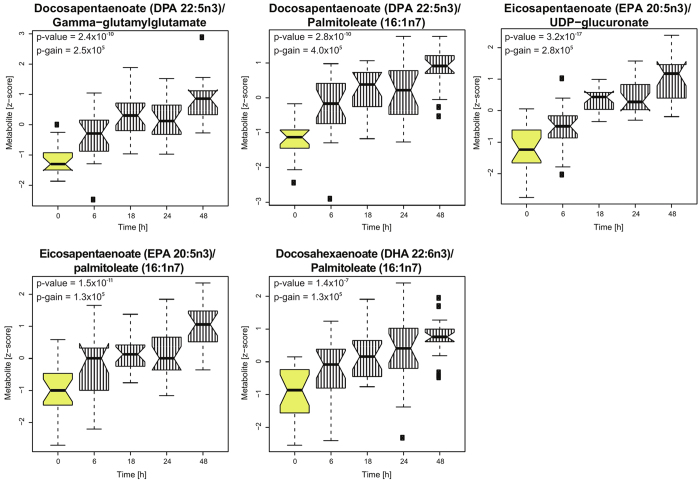
Metabolic ratios showing significant alterations in all examined cancer cell lines due to the contact with endothelial cells. The box plot presents a significant increase in the metabolic ratios, which was observed over the entire experimental period in all cancer cell lines. The yellow color indicates control (cancer cells at time point 0, not co-cultured) and striped pattern indicates cancer cells co-cultured with endothelial cells at different time points.

We applied mixed effect linear regression model that enables testing for metabolic alterations occuring over the entire experimental period, which will be coherent for all four cancer cell lines. Five metabolites, including two polyunsaturated fatty acids (PUFA) (docosapentaenoate (DPA 22:5n3) and eicosapentaenoate (EPA 20:5n3)), one dipeptide (gamma-glutamylglutamate) and two nucleotide sugars (isobar: UDP-acetylglucosamine, UDP-Acetylgalactosamine and UDP-glucuronate) were significantly altered in all cancer cell lines (Table 1). The PUFA’s and gamma-glutamylglutamate were increased and nucleotide sugars decreased over the experimental period. Detailed information on metabolic patterns for all metabolites and cell lines are presented in Figure S1, and the computed *p-values* in Table S1A. Additionally, significant increase in five pairs of metabolites was observed over the whole experimental period (Fig. 2), when we used computed metabolic ratios (p-value ≤ 2.4*10^−6^ and p-gain ≥ 1.02*10^5^). The significantly altered metabolic pairs are predominantly fatty acids (DPA, EPA, DHA and palmitoleate). The full set of associations for metabolic ratios is provided in Table S2.

This data demonstrate that endothelial cells are triggering changes in cancer cell metabolism coherently and independently of the cancer cell line origin.

### Cancer cells exhibit dynamic metabolic responses to endothelial niche

We further ask whether cancer cells display time-specific metabolic responses by applying pair-wise comparisons of all time-points (6 h, 18 h, 24 h and 48 h) post-co-culture to time point zero. Already six hours after contact with endothelial cells, significant alterations were observed in thirteen metabolites out of which seven, including cytidine 5′-monophosphate (5′-CMP), N-acetylneuraminate, gamma-glutamylglutamate, glycerophosphorylcholine (GPC), lactose, pantothenate and unknown X14577, were up-regulated only at this time point. The PUFA’s including DPA, EPA and arachidonate (AA) together with cytidine 5′-diphosphocholine begin to raise at 6 h and remain elevated over the experiment. The patterns of metabolic alterations change at eighteen hours; the uniquely altered metabolites at 18 h include dihomo-linolenate (20:3n3), N-acetylglutamate and unknown metabolite X – 11583. The temporal increase was observed in dihomo-linoleate (6 h and 18 h), 5-methylthioadenosine (MTA), glutathione reduced (GSH) and linoleate (18 h and 24 h) as well as choline (6 h, 18 h and 24 h). At time point 18 h cancer cells exhibited increase in N-acetylglutamate and decrease in isobar: UDP-acetylglucosamine, UDP-acetylgalactosamine, which persisted over all following time points. All seven metabolites identified as significantly altered 48 hours after co-culture were found either in the group of metabolites modified significantly over the entire experiment or at other time points. The full set of metabolic patterns and computed p-values for all time points and cell lines is provided in Figures S2,S3,S4,S5,S6 and Table S1B–E. This results show that endothelial cells modulate cancer cell metabolism in time-dependent manner.

We further analyzed the metabolic alterations in the context of pathways to elucidate those metabolites which are crucial for the interactions between cancer and endothelial cells. The metabolic profiles of cancer cells after co-culture with endothelial cells suggest alterations in pathways described below. Data interpretation for the metabolites altered as single compounds on the pathway is provided in the supplementary material (Figure S7).

### Hexosamine biosynthetic pathway but not “Warburg effect” is altered due to the contact with endothelial cells

**Figure 3:**
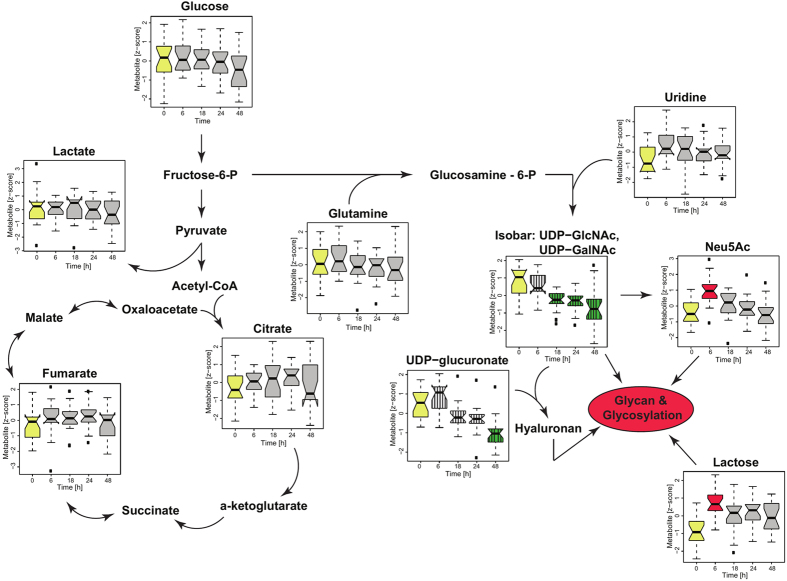
Impact of endothelial cells on glycolysis, TCA cycle and glycosylation. The glucose intake and lactate production same as TCA cycle metabolism were not significantly altered in all cancer cell lines. The significant metabolic alterations can be linked with modification in glycosylation patterns. Red reflects significant increase observed in all cancer cell lines at given time points in comparison with control. Green reflects significant decrease observed in all cancer cell lines in comparison with control (time point 0, highlighted in yellow). Gray indicates no significant changes observed for all cancer cell lines. The striped pattern reflects alterations identified as significant over the entire experiment. The significance level was p ≤ 3.49 × 10^−04^ (p ≤ 0.05/143).

Previous reports suggested regulatory role of tumor microenvironment on Warburg effect. In our studies neither levels of glucose nor lactate were significantly altered in cancer cells after culture with endothelial cells. The alterations in TCA cycle molecules were also not observed in all the cell lines. The individual cell line levels of lactate remain unchanged in all cell lines and glucose exhibit minor cell line specific decrease over the entire experiment (Figure S1). This results show that in cancer cells, metabolites associated with “Warburg effect” are not modulated by the endothelial cells in the early stage of niche formation. In turn, the endothelial cells induce significant alteration in carbohydrates level (Table 1), which can be synthetized in the hexamine biosynthetic pathway (UDP-GlcNAc, UDP-GalNac, UDP-glucuronate and Neu5Ac). All carbohydrates exhibiting significant alteration can serve as substrates for glycoconjugates formation and glycosylation. Figure 3 illustrates alteration patterns as well as potential interactions between the molecules in metabolic network related to hexosamine biosynthetic pathway. The UDP-GlcNAc can be utilized as a substrate for Neu5Ac synthesis or together with the UDP-glucoronate can serve as monosaccharide building blocks for hyaluronan synthesis, which is glycosaminoglycan, composed of 2,000–25,000 disacharides of glucuronic acid and N-acetylglucosamine^19^. Increase in lactose can also be linked to changes in glycosylation patterns, since glucose together with galactose are involved in assembling lactosylceramide^20^, ubiquitously present glycosphingolipid structures in mammals involved in cell proliferation, adhesion, migration and angiogenesis^21^. These results suggest potential involvement of hexamine biosynthetic pathway and glycosylation in the formation of cancer niche.

### Endothelial cells modulate glycerophospholipids metabolism of cancer cells

**Figure 4:**
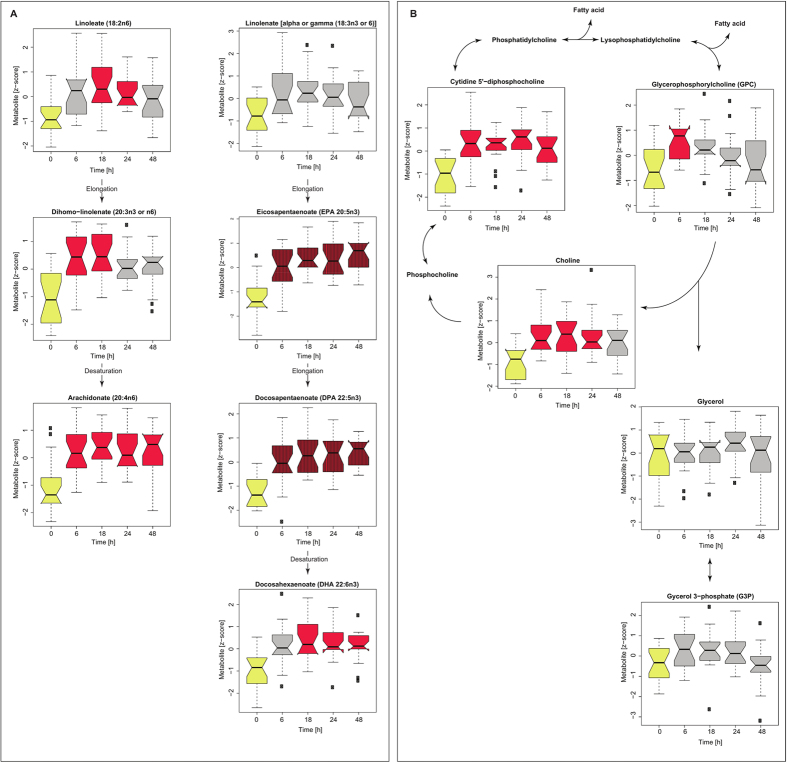
The impact of an endothelial niche on fatty acid and glycerophospholipids metabolism in ovarian and colon cancer cell lines. (**A**) Alteration patterns of PUFA’s (n-6 and n-3), representing all cancer cell lines (OVCAR3, SKOV3, HCT15 and HCT116) are shown as box plots. (**B**) Alteration patterns of glycerophospholipids, representing all cancer cell lines (OVCAR3, SKOV3, HCT15 and HCT116) are shown as box plots. Red reflects significant increase observed in all cancer cell lines at given time point in comparison with control (time point 0, highlighted in yellow). Gray indicates no significant changes observed for all cancer cell lines. The striped pattern reflects alterations identified as significant over the entire experiment. The significance level was p ≤ 3.49 × 10^−04^ (p ≤ 0.05/143).

Among the 21 significantly altered metabolites we identified 9 lipids including three omega-3 (n-3 FA) and three omega-6 (n-6 FA) fatty acids as well as choline and its intermediates. These metabolites are closely connected, since glycerophospholipids such as phosphatidylcholines or lysophosphatidylcholines are composed of fatty acid chains (one or two), a phosphate group, and choline molecule. The n-3 and n-6 FA cannot be synthetized *de novo* in mammals, but can be modified (elongated or desaturated), once the primary molecule of n-3 (α-linolenic acid) or n-6 FA (linoleic acid (LA)) are delivered into the cell. As shown in Fig. 4A linoleic acid significantly increases and α-linolenic acid exhibits a trend of increase after the co-culture was established. These molecules can serve as a building blocks for the remaining n-6 FA’s and n-3 FA’s molecules. Alternatively, n-6 FA’s and n-3 FA’s can be released from the cellular membrane; simultaneous significant increase in choline and its intermediates (Fig. 4B) as well as increase of EPA and DPA over the entire experiment followed by the increase of DHA after 18 h, might suggest possible release of EPA and its elongation and desaturation to form DHA. These results indicate that contact with endothelial cells induces time resolved alteration in glycerophospholipids metabolism as reflected by the alterations in fatty acid as well as choline and its intermediates.

## Discussion

In this study we characterized metabolic changes associated with nesting of cancer cell in the endothelial niche by deploying *in vitro* co-culture system. Although the cell line specific metabotype exhibited dominant signal, we identified coherent metabolic alterations in all cancer cell lines triggered by the contact with endothelial cells. While our study shows that glycerophospholipid metabolism, hexosamine biosynthetic pathway, and glycosylation have significant role in the first 48 hours of the co-culture establishment, we do not observe alteration in the “Warburg effect” as well as TCA cycle metabolism.

The elevated “Warburg effect”, is considered as one of the metabolic features of cancer metabolism regulated by the microenvironment, as it is a a consequence of hypoxic conditions caused by the abnormal vascularization^8^. In this study the levels of metabolites associated with “Warburg effect” remained unchanged at different time points within the first 48 hours after the co-culture was established. This, suggest that “Warburg effect” is not a critical metabolic event in the initial phase of co-culture formation, and the alteration initially reported as caused by microenvironment^8^, might be rather associated with cancer proliferation. Generally, increase in glucose metabolism and lactate production correlates with the increased proliferation rate of the cancer cells. Previously we reported that endothelial cells enhance proliferation capacity of cancer cells 4 days after co-culture was established^12^. Accordingly, the metabolic alterations identified in the first 48 hours might be solely associated with the cancer–endothelial-cells interactions and not with proliferation of cancer cells.

The significant alterations in metabolites linked to glycosylation, was a common response to the co-culture for all tested cancer cell lines. The remodeling of glycan structures in cancer has been already associated with cross-talk between cancer cells and their microenvironment, which in turn supports its progression, invasiveness, and metastasis^20^. A direct impact of glycosylation on the cell-cell adhesion, contributing to cancer invasiveness and metastasis was reported in several studies^22^,^23^,^24^,^25^. For instance, significantly altered UDP-glucuronate and UDP-glucosamine can be deployed for glycosylation as separate units or can serve as substrates for hyaluronan biosynthesis, which also is involved in cellular membrane glycosylation^26^. Furthermore, increased synthesis of hyaluronan was associated with malignant progression in several cancers, including ovarian and colon cancer^27^; its role in tumor growth and progression *in vivo* as well as in malignant transformation was previously reported^28^. Thus, modifications in glycosylation patterns triggered by the endothelium might be considered as a metabolic switch crucial for both: implanting of cancer cell in the endothelial niche as well as its progression and transition towards more aggressive phenotype.

The phosphatidylcholine and phosphatidylethanolamine, which can be *de novo* synthetized in the Kennedy pathway^29^, are the major building blocks of the cellular membrane and have direct impact on membrane structure, its integrity and signaling function^30^. Thus, the significant increase observed in both glycerophospholipid metabolism and PUFA’s could be associated with remodeling of cancer cellular membrane. These changes, induced by the endothelial cells, might improve cellular cross-talk or modulate signaling pathways promoting cancer progression^31^. The composition of the fatty acid chains in the glycerophospholipid residue can be rearranged to modulate membrane fluidity and permeability in order to meet cellular demands^32^. Furthermore, increase in choline metabolism was associated with malignant transformation^33^. Increased choline and cytidine-5′-diphosphocholine levels can be linked with the choline kinase (ChoK) displaying oncogenic activities^34^. It has been shown that increase in choline metabolism is strongly regulated by the RAS and PI3K-Akt oncogenic pathways^33^,^35^. This observation may suggest that endothelial niche is modulating malignant phenotype subsequent to RAS and PI3K-Akt pathway activation, resulting in increased choline metabolism.

Increase in n-6 PUFA’s such as AA is associated with metastatic behaviors in ovarian cancer^36^, as well as in colon cancer^37^. Additionally, in accordance to our study, increase in lipid metabolism over the metabolism of carbohydrates and amino acids was associated with tumor metastasis^6^. The increased metastatic potential of tumor cells associated with AA can be directly linked with its derivatives namely eicosanoids (prostaglandins, leucotrines, hydroxyeicosatraenoic acids), promoting angiogenesis, and subsequently metastatic spread^38^. These might suggest that PUFA’s are released from the membrane to stimulate endothelial cell proliferation, which in turn underscores the cross-talk between cancer and endothelial cells previously showed in our studies^13^. In contrast, n-3 PUFA’s were associated with anti-inflammatory, anti-carcinogenic and anti-angiogenic effects^39^. It has been shown that n-3 PUFA’s compete with n-6 PUFA’s for incorporation in cellular membrane as well as for the COX-2 and 5-LOX enzymes, involved in eicosanoid production^39^. Thus, the simultaneous elevation of both n-3 and n-6 PUFA’s may suggest tight regulation of angiogenesis stimulated by cancer cells.

## Conclusions

The metabolic events accompanying the implantation of cancer cell into the endothelial niche are poorly understood. Using the *in vitro* co-culture system, mimicking cancer–endothelial-cell interaction, we identified metabolic alterations coherent in all cancer cell lines (independent of cancer origin), which were triggered by the endothelial cells. We have shown that contact with endothelial cells has a significant impact on the cellular membrane composition as pointed by the alterations in glycerophospholipid and fatty acid metabolism. Significant alteration in the metabolites involved in glycoconjugates formation and glycosylation might be associated with the cellular adhesion crucial for cancer – endothelial cells interaction as well as malignant transformation. Additionally, increased PUFA’s level, involved in angiogenesis modulation, may suggest that cancer cells tightly control the endothelial cells progression. We demonstrated that “Warburg effect” is not modulated by the endothelial cells in the initial stage of cancer – endothelial cell interaction. Our study provides a novel input into the cancer cell metabolism in the context of endothelial microenvironment, which was poorly defined so far. However, further studies monitoring the impact of endothelial cells on cancer cell metabolism over increased time period and with larger cell line number and cancer origin would be required to provide a broad overview on formation, preservation and progression of cancer cell in endothelial niche.

## Additional Information

**How to cite this article**: Halama, A. *et al*. Nesting of colon and ovarian cancer cells in the endothelial niche is associated with alterations in glycan and lipid metabolism. *Sci. Rep.* **7**, 39999; doi: 10.1038/srep39999 (2017).

**Publisher's note:** Springer Nature remains neutral with regard to jurisdictional claims in published maps and institutional affiliations.

## Supplementary Material

Supplementary Materials

Supplementary Table 1

Supplementary Table 2

Supplementary Figure 1

Supplementary Figure 2

Supplementary Figure 3

Supplementary Figure 4

Supplementary Figure 5

Supplementary Figure 6

Supplementary Figure 7

## Figures and Tables

**Table 1:** List of metabolites showing significant alterations in cancer cells induced by endothelial cells at the different time points independent of cell line and cancer origin.

Metabolite	Pathway	Time	0–6 h	0–18 h	0–24 h	0–48 h	
5-methylthioadenosine (MTA)	Amino acid			1.2 × 10^−6^↑	3.6 × 10^−6^↑		
Glutathione reduced (GSH)				3.4 × 10^−6^↑	3.4 × 10^−6^↑		
N-acetylglutamate				1.4 × 10^−06^↑			
Lactose	Carbohydrates		2.9 × 10^−6^↑				
N-acetylneuraminate			2.3 × 10^−5^↑				
Isobar: UDP-acetylglucosamine, UDP-Acetylgalactosamine		5.3 × 10^−8^↓		6.7 × 10^−5^↓	1.6 × 10^−4^↓	1.1 × 10^−4^↓	
UDP-glucuronate		9.3 × 10^−12^↓				8.8 × 10^−7^↓	
Pantothenate	Co&V		8.6 × 10^−5^↑				
Arachidonate (20:4n6)	Lipid		1.2 × 10^−4^↑	1.1 × 10^−5^↑	7.4 × 10^−5^↑	1.9 × 10^−4^↑	
Choline			6.5 × 10^−5^↑	1.8 × 10^−4^↑	1.8 × 10^−4^↑		
Cytidine 5′-diphosphocholine			1.3 × 10^−5^↑	2.0 × 10^−5^↑	5.0 × 10^−6^↑	3.1 × 10^−4^↑	
Dihomo-linolenate (20:3n3)			1.8 × 10^−4^↑	1.3 × 10^−5^↑			
Docosapentaenoate (DPA 22:5n3)		1.1 × 10^−4^↑	3.4 × 10^−4^↑	1.2 × 10^−6^↑	3.2 × 10^−6^↑	3.2 × 10^−6^↑	
Eicosapentaenoate (EPA 20:5n3)		2.0 × 10^−6^↑	1.0 × 10^−4^↑	3.3 × 10^−8^↑	1.3 × 10^−6^↑	5.6 × 10^−8^↑	
Glycerophosphorylcholine (GPC)			2.4 × 10^−4^↑				
Docosahexaenoate (DHA 22:6n3)				7.9 × 10^−5^↑	1.3 × 10^−4^↑	3.2 × 10^−4^↑	
Linoleate (18:2n6)				1.7 × 10^−4^↑	2.3 × 10^−4^↑		
Cytidine 5′-monophosphate (5′-CMP)	Nucleotide		2.0 × 10^−4^↑				
Gamma-glutamylglutamate	Polypeptide	1.4 × 10^−4^↑	2.3 × 10^−6^↑				
X – 14577	Unknown		9.8 × 10^−6^↑		1.9 × 10^−4^↑		
X – 11583				1.5 × 10^−4^↑			

The direction of metabolic alterations is represented by an arrow as follow: ↑ - increase; ↓ - decrease. “Co&V” indicates cofactor and vitamin. “Time” and time intervals (**0–6 h**, **18 h**, **24 h**, **48 h)** reflect metabolites showing significant alteration over the entire experimental period and specific time points, respectively.
