# Supplementary material for: Nesting of colon and ovarian cancer cells in the endothelial niche is associated with alterations in glycan and lipid metabolism
Source: Sci Rep. 2017 Jan 4;7:39999. doi: 10.1038/srep39999 (PMC5209689; doi:10.1038/srep39999)
Supplement: Supplementary Materials [file srep39999-s1.pdf]

Nesting of colon and ovarian cancer cells in the endothelial niche is associated with alterations in glycan and lipid metabolism.

Anna Halama[1†], Bella S Guerrouahen[2,3,4†], Jennifer Pasquier[2,3], Noothan J. Satheesh[1†], Karsten Suhre [1,5*] and Arash Rafii [2,3,6*].

†Contributed equally
1. Department of Physiology and Biophysics, Weill Cornell Medicine-Qatar, Qatar-Foundation, P.O. Box 24144, Doha, Qatar.
2. Stem Cell and Microenvironment Laboratory, Weill Cornell Medicine-Qatar, Education City, Qatar Foundation, Doha, Qatar.
3. Department of Genetic Medicine, Weill Cornell Medicine-Qatar, New York, NY 10065, USA.
4. Translational Medicine Division-Research Department, Sidra Medical and Research Center, PO Box 26999, Doha, Qatar.
5. Institute of Bioinformatics and Systems Biology, Helmholtz Zentrum München, German Research Center for Environmental Health, Neuherberg, Germany.
6. Department of Genetic Medicine and Obstetrics and Gynecology, Weill Cornell Medical College, Stem Cell and Microenvironment Laboratory, Weill Cornell Medical College in Qatar, Qatar-Foundation, P.O. Box 24144, Doha, Qatar.

Corresponding authors:
Dr. Karsten Suhre: Department of Physiology and Biophysics, Weill Cornell Medicine-Qatar, Doha, Qatar Weill Cornell Medical College in Qatar, Qatar-Foundation, P.O Box: 24144, Doha, Qatar. Phone: +974 4492 8482. Fax: +974 4492 8422. Email: kas2049@qatar-med.cornell.edu

Dr. Arash Rafii: Department of Genetic Medicine and Obstetrics and Gynecology, Weill Cornell Medicine-Qatar, Stem cell and microenvironment laboratory Weill Cornell Medical College in Qatar, Qatar-Foundation, P.O Box: 24144, Doha, Qatar. Phone: +974 3313 5828. Fax: +974 4492 8422. Email: jat2021@qatar-med.cornell.edu

Supplementary materials

Supplementary Table 1 A-E. List of metabolites present in all cancer cell lines together with the p-values reflecting significance level of metabolic alterations over the entire experiment **TableS1A** and at different time points **TableS1B-E.**

Supplementary Table 2. List of metabolic ratios together with the p-values and p-gain values reflecting significance level of metabolic ratio alterations over the entire experiment.

Supplementary Figure 1. Identification of outliers using 3D-PCA.

The PCA score plots generated by SIMCA software show distinct clustering of HCT15 (dark blue), HCT116 (light blue), OVCAR3 (red), SKOV3 (orange). To sample outliers were identified in the SKOV3 cell line and were removed from the analysis.

Supplementary Figure 2 – 6. Patters of all metabolic alterations plotted for all cell lines and for each cell line individually over the entire experiment and at different time points.

Supplementary Figure 7. **Alterations in glutathione, gamma-glutamylglutamate, N-acetylglutamate (NAG) and 5-methylthioadenosine (MTA) in cancer cells induced by contact with endothelial cells might be associated with modification in: A) Gamma-glutamyl cycle**. Elevated level of GSH, the primary antioxidant of the cell, was observed in several different cancer types including colon and ovarian cancer and was linked with resistance to therapy and poor prognosis[1,2]. The gamma-glutamyl cycle is playing a crucial role in the synthesis/salvage of GSH, since it serves as a donor of cysteine, which has limited availability in the cell due to its toxicity [3]. Elevated levels of GSH and gamma-glutamylglutamate might suggest impact of endothelial cells on this pathway. **B) Synthesis and degradation of N-acetylglutamate**. Increased level of NAG suggests alterations in its synthesis which could be directly linked to changes in the urea cycle, especially since NAG is an essential activator of carbamoyl phosphate synthetase (CPS1) catalyzing the first step of ammonia detoxification to urea [4]. The synthesis of NAG could be triggered by arginine, known to be elevated in colon cancer tissues [5]. Thus, based on the increases in NAG it could be hypothesized that endothelial cells might impact the urea cycle and arginine metabolism. **C) Methionine salvage pathway.** MTA is a byproduct of polyamine synthesis as well as a substrate for methionine recovery [6]. The elevated level of MTA might suggest that endothelial cells are modulating alterations in methionine as well as spermine and spermidine metabolism of cancer cells.

It has to be noted that only pathway components depicted in red reflect the significant increase observed in all cancer cell lines at a given time point in comparison with control (time point 0, highlighted in yellow) and gray indicates no significant changes.

1 Barranco, S. C. *et al.* Relationship between colorectal cancer glutathione levels and patient survival: early results. *Dis Colon Rectum* **43**, 1133-1140 (2000).

2 Hanigan, M. H., Frierson, H. F., Jr., Brown, J. E., Lovell, M. A. & Taylor, P. T. Human ovarian tumors express gamma-glutamyl transpeptidase. *Cancer Res* **54**, 286-290 (1994).

3 Lu, S. C. Regulation of glutathione synthesis. *Mol Aspects Med* **30**, 42-59, 10.1016/j.mam.2008.05.005 (2009).

4 de Cima, S. *et al.* Structure of human carbamoyl phosphate synthetase: deciphering the on/off switch of human ureagenesis. *Sci Rep* **5**, 16950, 10.1038/srep16950 (2015).

5 Lu, Y. *et al.* Overexpression of arginine transporter CAT-1 is associated with accumulation of L-arginine and cell growth in human colorectal cancer tissue. *PLoS One* **8**, e73866, 10.1371/journal.pone.0073866 (2013).

6 Pirkov, I., Norbeck, J., Gustafsson, L. & Albers, E. A complete inventory of all enzymes in the eukaryotic methionine salvage pathway. *FEBS J* **275**, 4111-4120, 10.1111/j.1742-4658.2008.06552.x (2008).
