# Supplementary material for: Nesting of colon and ovarian cancer cells in the endothelial niche is associated with alterations in glycan and lipid metabolism
Source: Sci Rep. 2017 Jan 4;7:39999. doi: 10.1038/srep39999 (PMC5209689; doi:10.1038/srep39999)
Supplement: Supplementary Figure 1 [file srep39999-s4.pdf]

Nesting of colon and ovarian cancer cells in the endothelial niche is associated with alterations in glycan and lipid metabolism.

Anna Halama[1†], Bella S Guerrouahen[2,3,4†], Jennifer Pasquier[2,3], Noothan J. Satheesh[1†], Karsten Suhre [1,5*] and Arash Rafii [2,3,6*].

†Contributed equally
1. Department of Physiology and Biophysics, Weill Cornell Medicine-Qatar, Qatar-Foundation, P.O. Box 24144, Doha, Qatar.
2. Stem Cell and Microenvironment Laboratory, Weill Cornell Medicine-Qatar, Education City, Qatar Foundation, Doha, Qatar.
3. Department of Genetic Medicine, Weill Cornell Medicine-Qatar, New York, NY 10065, USA.
4. Translational Medicine Division-Research Department, Sidra Medical and Research Center, PO Box 26999, Doha, Qatar.
5. Institute of Bioinformatics and Systems Biology, Helmholtz Zentrum München, German Research Center for Environmental Health, Neuherberg, Germany.
6. Department of Genetic Medicine and Obstetrics and Gynecology, Weill Cornell Medical College, Stem Cell and Microenvironment Laboratory, Weill Cornell Medical College in Qatar, Qatar-Foundation, P.O. Box 24144, Doha, Qatar.

Corresponding authors:
Dr. Karsten Suhre: Department of Physiology and Biophysics, Weill Cornell Medicine-Qatar, Doha, Qatar Weill Cornell Medical College in Qatar, Qatar-Foundation, P.O Box: 24144, Doha, Qatar. Phone: +974 4492 8482. Fax: +974 4492 8422. Email: kas2049@qatar-med.cornell.edu

Dr. Arash Rafii: Department of Genetic Medicine and Obstetrics and Gynecology, Weill Cornell Medicine-Qatar, Stem cell and microenvironment laboratory Weill Cornell Medical College in Qatar, Qatar-Foundation, P.O Box: 24144, Doha, Qatar. Phone: +974 3313 5828. Fax: +974 4492 8422. Email: jat2021@qatar-med.cornell.edu

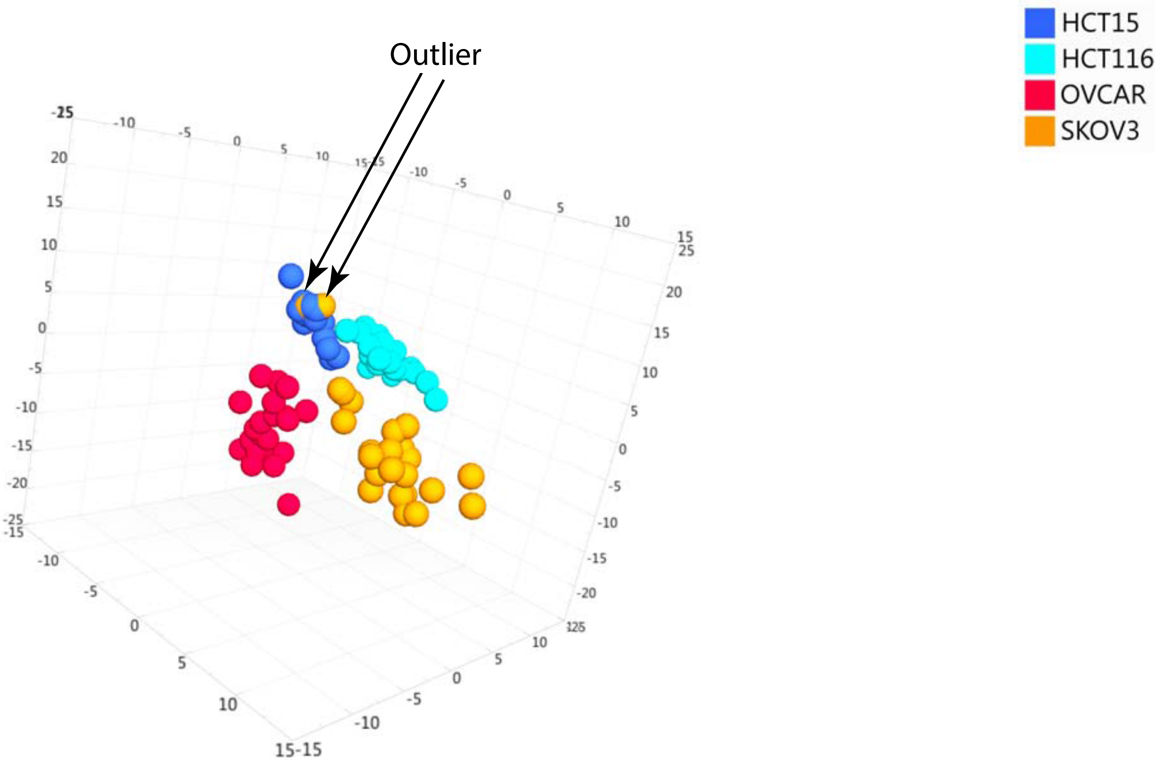
Outlier
HCT15
HCT116
OVCAR
SKOV3
