# Supplementary material for: Nesting of colon and ovarian cancer cells in the endothelial niche is associated with alterations in glycan and lipid metabolism
Source: Sci Rep. 2017 Jan 4;7:39999. doi: 10.1038/srep39999 (PMC5209689; doi:10.1038/srep39999)
Supplement: Supplementary Figure 2 [file srep39999-s5.pdf]

Nesting of colon and ovarian cancer cells in the endothelial niche is associated with alterations in glycan and lipid metabolism.

Anna Halama[1†], Bella S Guerrouahen[2,3,4†], Jennifer Pasquier[2,3], Noothan J. Satheesh[1†], Karsten Suhre [1,5*] and Arash Rafii [2,3,6*].

†Contributed equally
1. Department of Physiology and Biophysics, Weill Cornell Medicine-Qatar, Qatar-Foundation, P.O. Box 24144, Doha, Qatar.
2. Stem Cell and Microenvironment Laboratory, Weill Cornell Medicine-Qatar, Education City, Qatar Foundation, Doha, Qatar.
3. Department of Genetic Medicine, Weill Cornell Medicine-Qatar, New York, NY 10065, USA.
4. Translational Medicine Division-Research Department, Sidra Medical and Research Center, PO Box 26999, Doha, Qatar.
5. Institute of Bioinformatics and Systems Biology, Helmholtz Zentrum München, German Research Center for Environmental Health, Neuherberg, Germany.
6. Department of Genetic Medicine and Obstetrics and Gynecology, Weill Cornell Medical College, Stem Cell and Microenvironment Laboratory, Weill Cornell Medical College in Qatar, Qatar-Foundation, P.O. Box 24144, Doha, Qatar.

Corresponding authors:
Dr. Karsten Suhre: Department of Physiology and Biophysics, Weill Cornell Medicine-Qatar, Doha, Qatar Weill Cornell Medical College in Qatar, Qatar-Foundation, P.O Box: 24144, Doha, Qatar. Phone: +974 4492 8482. Fax: +974 4492 8422. Email: kas2049@qatar-med.cornell.edu

Dr. Arash Rafii: Department of Genetic Medicine and Obstetrics and Gynecology, Weill Cornell Medicine-Qatar, Stem cell and microenvironment laboratory Weill Cornell Medical College in Qatar, Qatar-Foundation, P.O Box: 24144, Doha, Qatar. Phone: +974 3313 5828. Fax: +974 4492 8422. Email: jat2021@qatar-med.cornell.edu

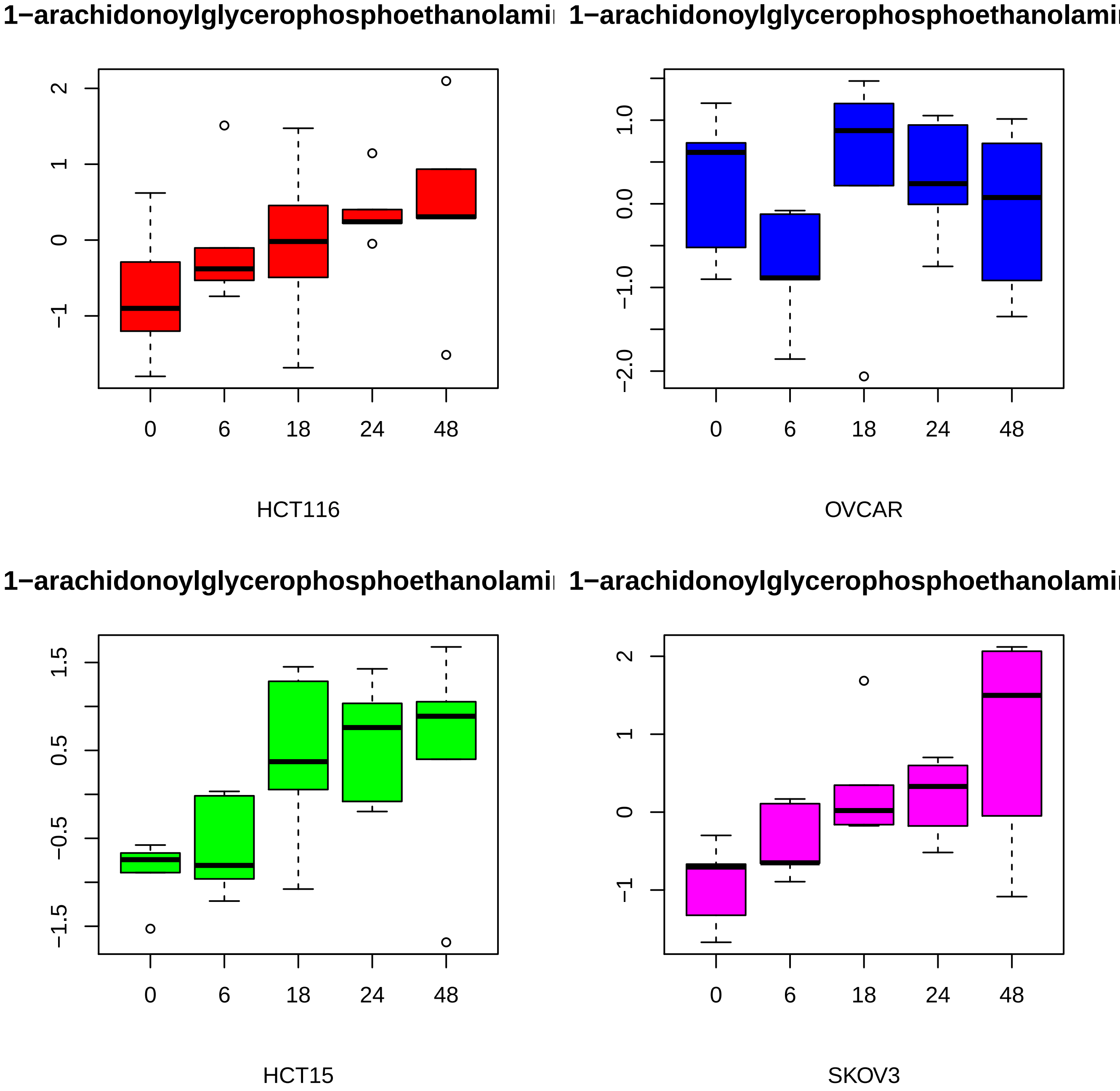

1−arachidonoylglycerophosphoethanolami
−1
0
1
2
0
6
18
24
48
HCT116
1−arachidonoylglycerophosphoethanolami
−2.0
−1.0
0.0
1.0
0
6
18
24
48
OVCAR
1−arachidonoylglycerophosphoethanolami
−1.5
−0.5
0.5
1.5
0
6
18
24
48
HCT15
1−arachidonoylglycerophosphoethanolami
−1
0
1
2
0
6
18
24
48
SKOV3

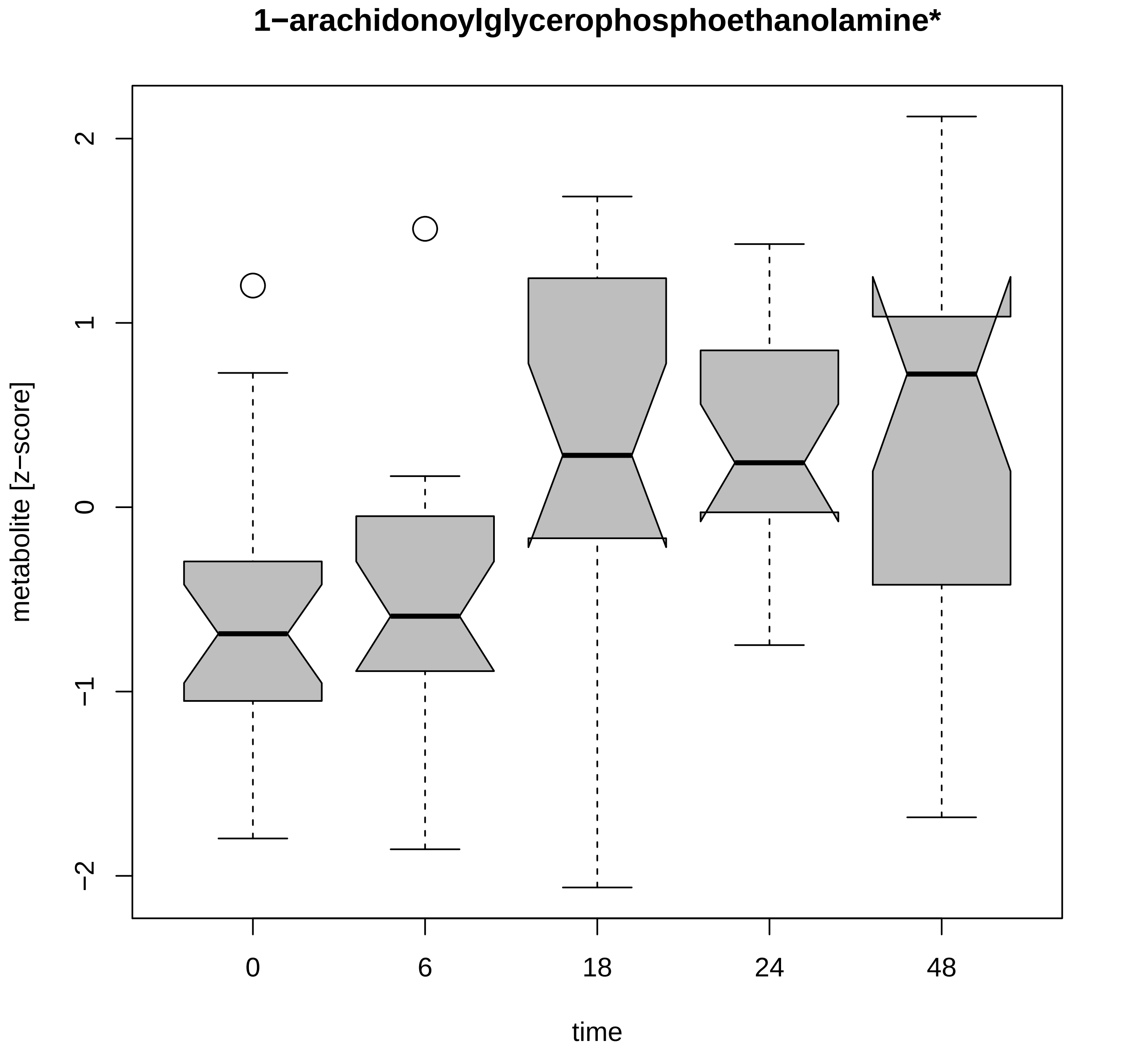

1−arachidonoylglycerophosphoethanolamine*
metabolite [z−score]
2
1
0
−1
−2
0
6
18
24
48
time

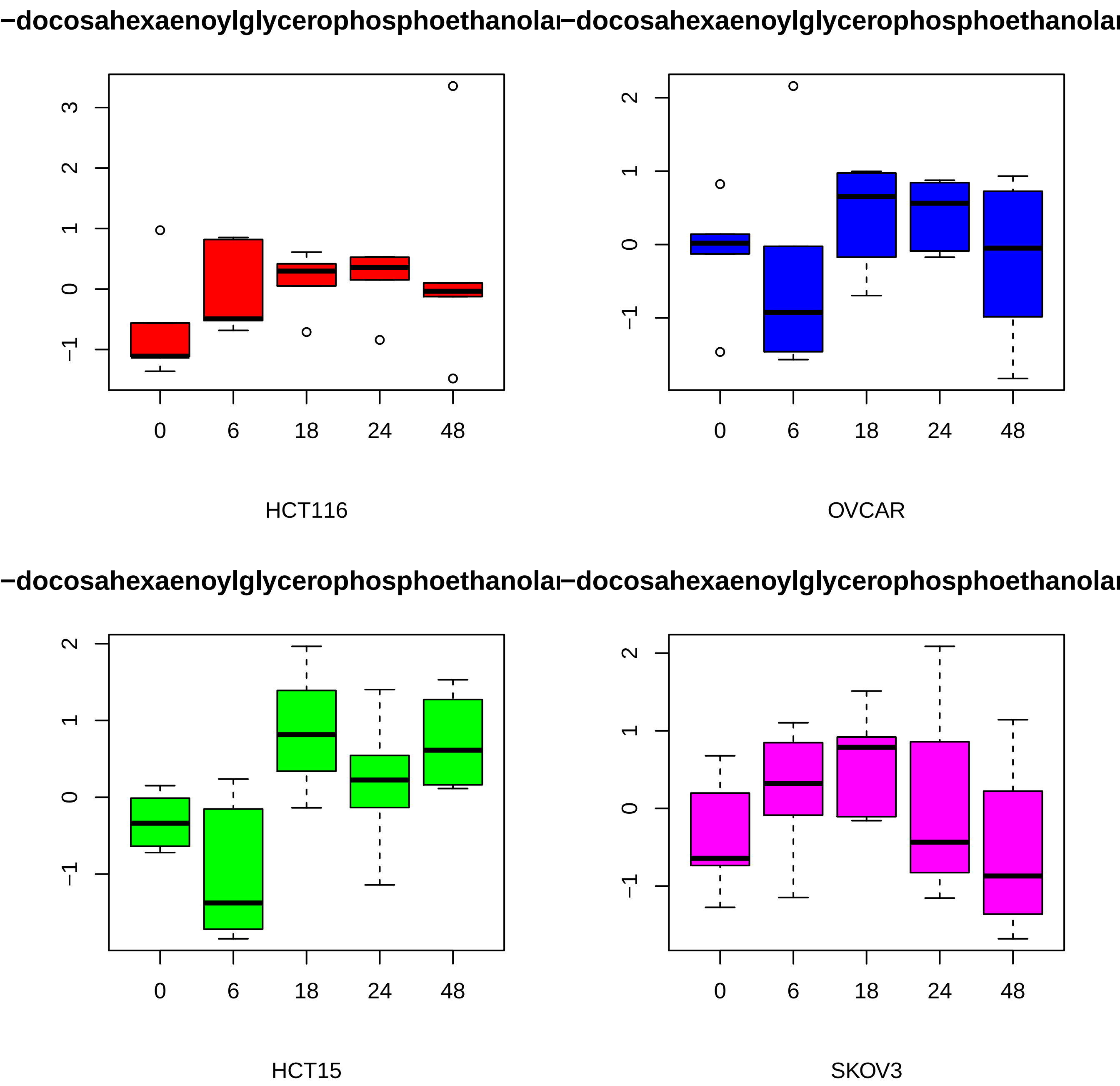
−docosahexaenoylglycerophosphoethanola
HCT116
−docosahexaenoylglycerophosphoethanola
OVCAR
−docosahexaenoylglycerophosphoethanola
HCT15
−docosahexaenoylglycerophosphoethanola
SKOV3
0
6
18
24
48

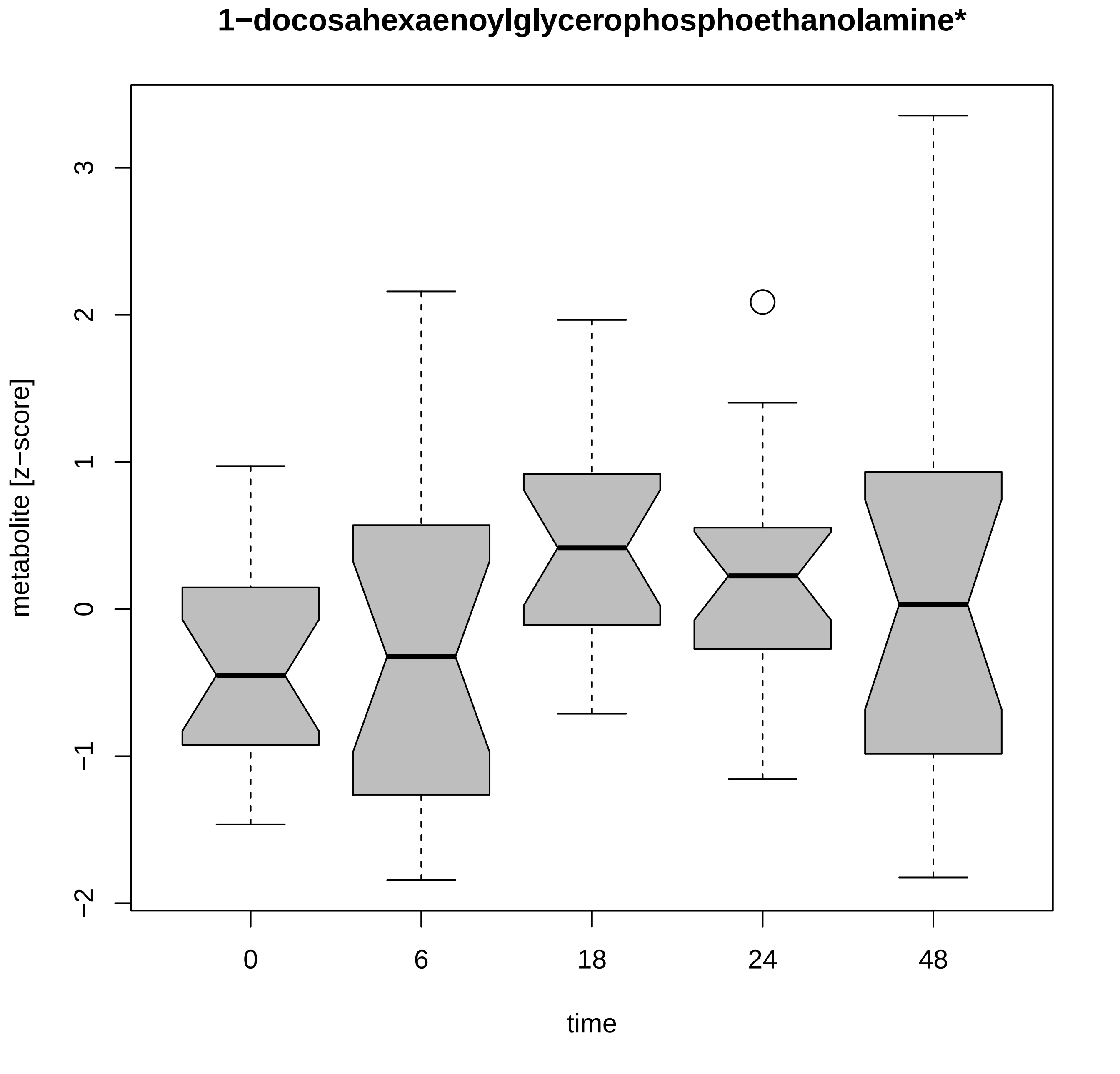

1−docosahexaenoylglycerophosphoethanolamine*
metabolite [z−score]
−2
−1
0
1
2
3
0
6
18
24
48
time

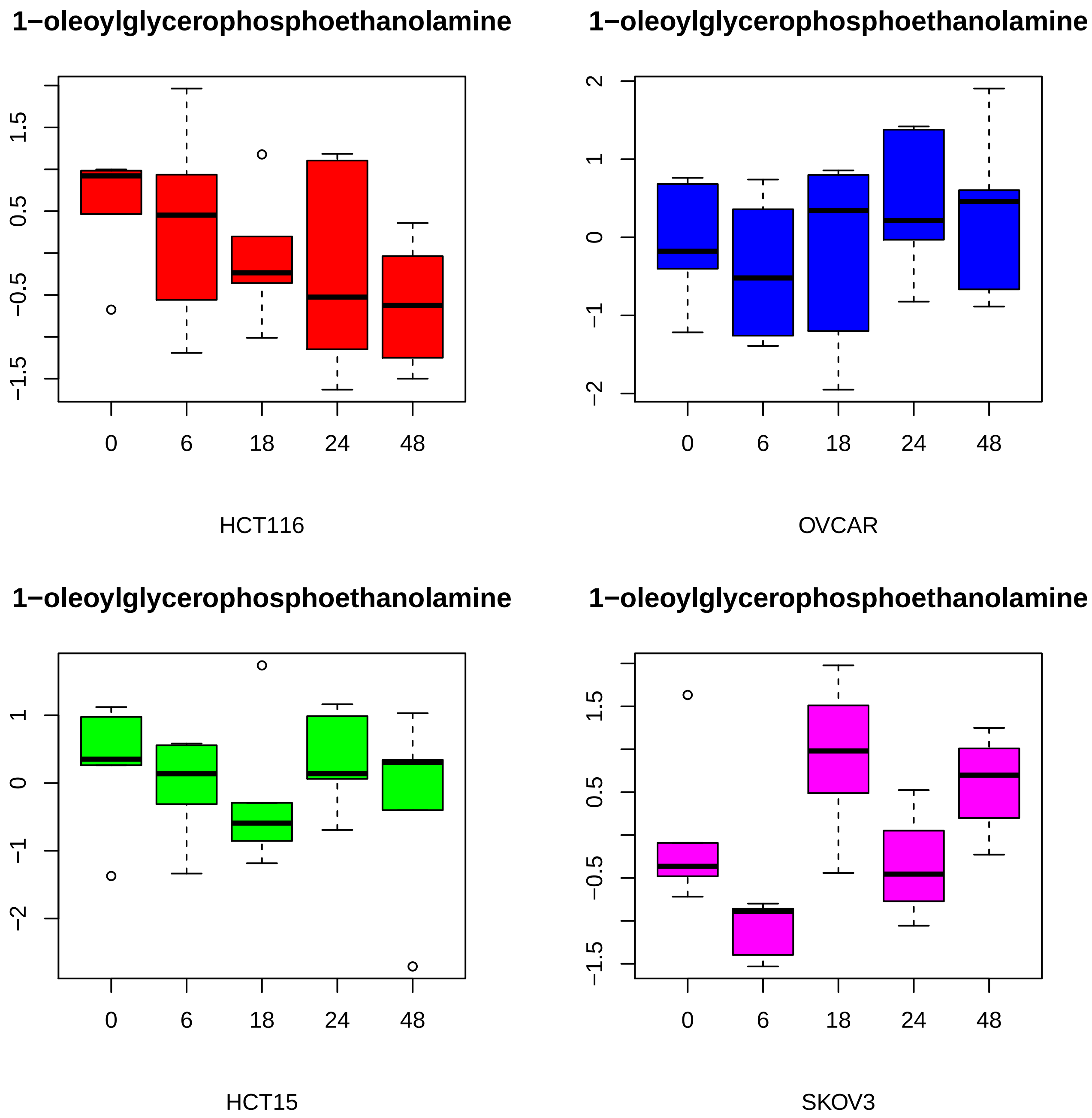
1−oleoylglycerophosphoethanolamine
−1.5
−0.5
0.5
1.5
0
6
18
24
48
HCT116
1−oleoylglycerophosphoethanolamine
−2
−1
0
1
2
0
6
18
24
48
OVCAR
1−oleoylglycerophosphoethanolamine
−2
−1
0
1
0
6
18
24
48
HCT15
1−oleoylglycerophosphoethanolamine
−1.5
−0.5
0.5
1.5
0
6
18
24
48
SKOV3

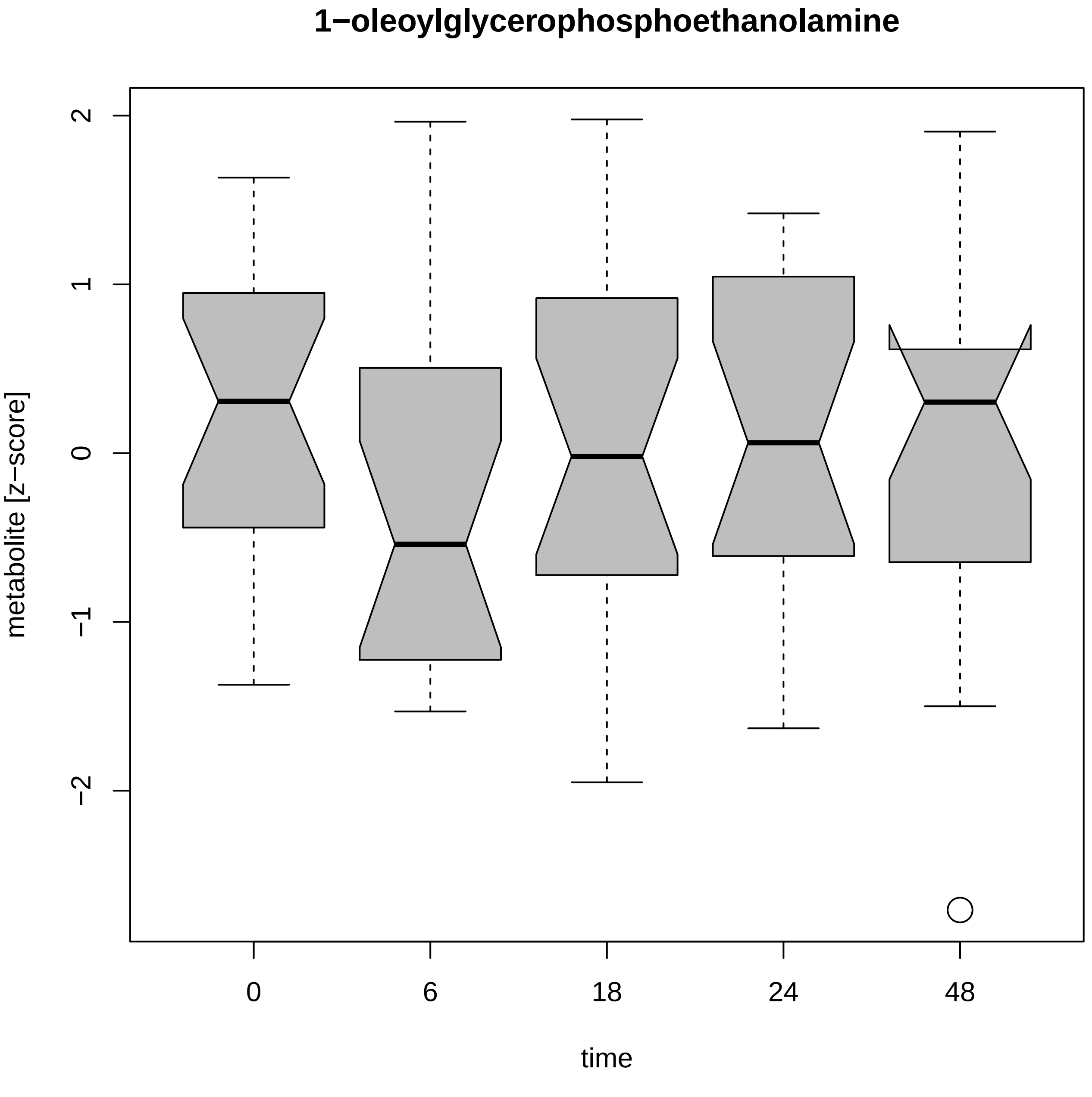

1−oleoylglycerophosphoethanolamine
metabolite [z−score]
2
1
0
−1
−2
0
6
18
24
48
time

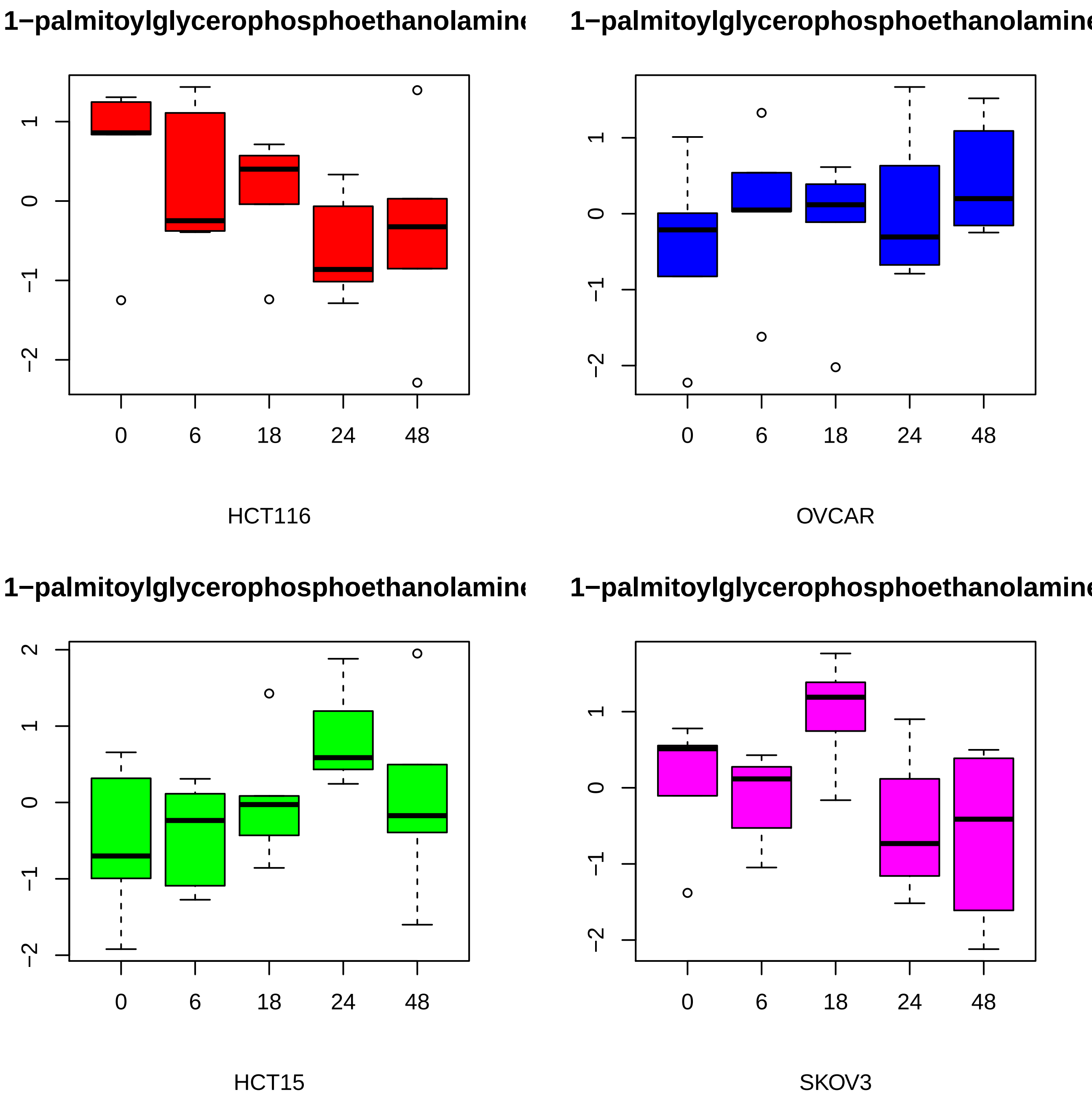
1−palmitoylglycerophosphoethanolamine
0
6
18
24
48
HCT116
1−palmitoylglycerophosphoethanolamine
0
6
18
24
48
OVCAR
1−palmitoylglycerophosphoethanolamine
0
6
18
24
48
HCT15
1−palmitoylglycerophosphoethanolamine
0
6
18
24
48
SKOV3

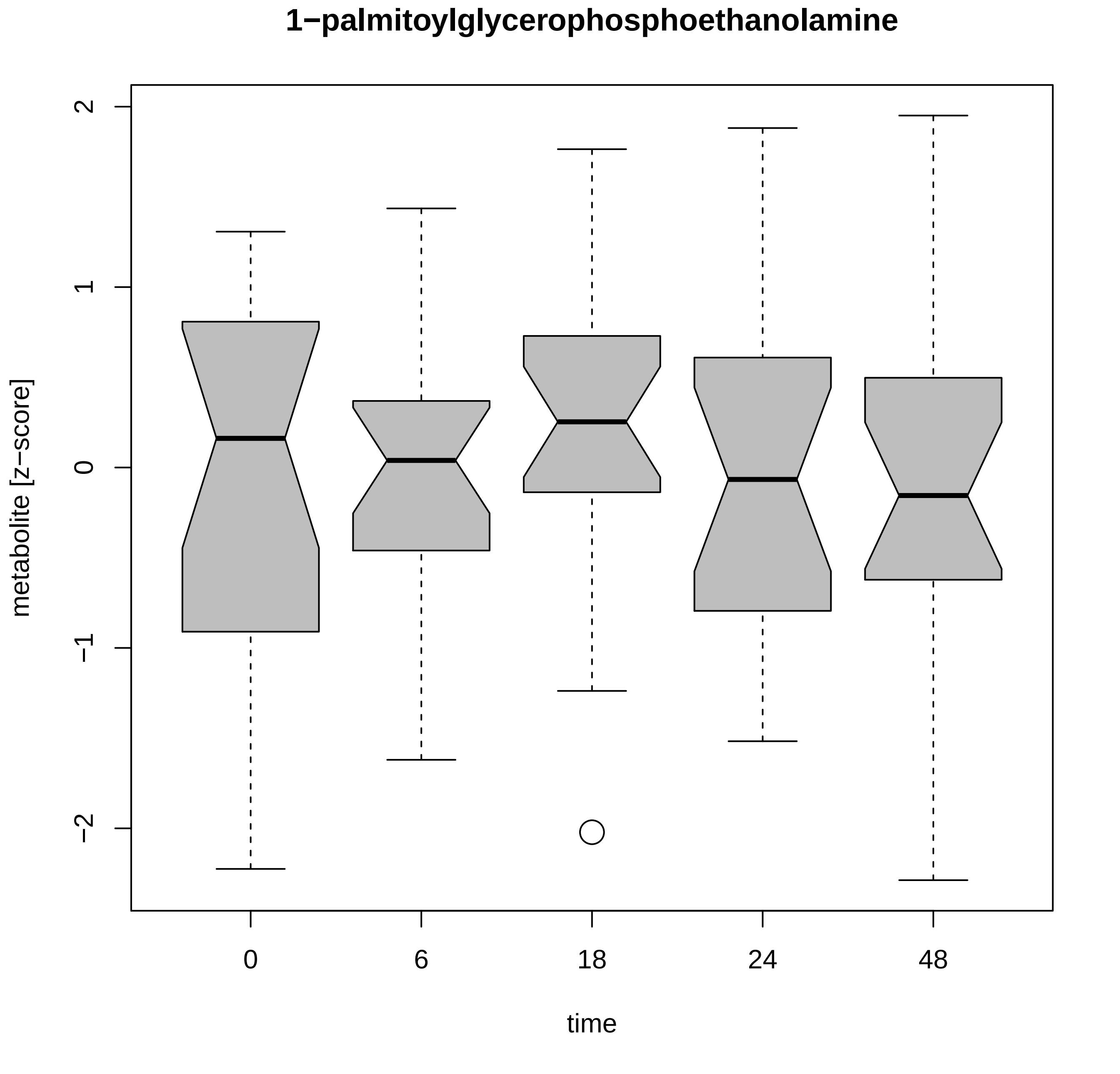
1−palmitoylglycerophosphoethanolamine
metabolite [z−score]
2
1
0
−1
−2
0
6
18
24
48
time

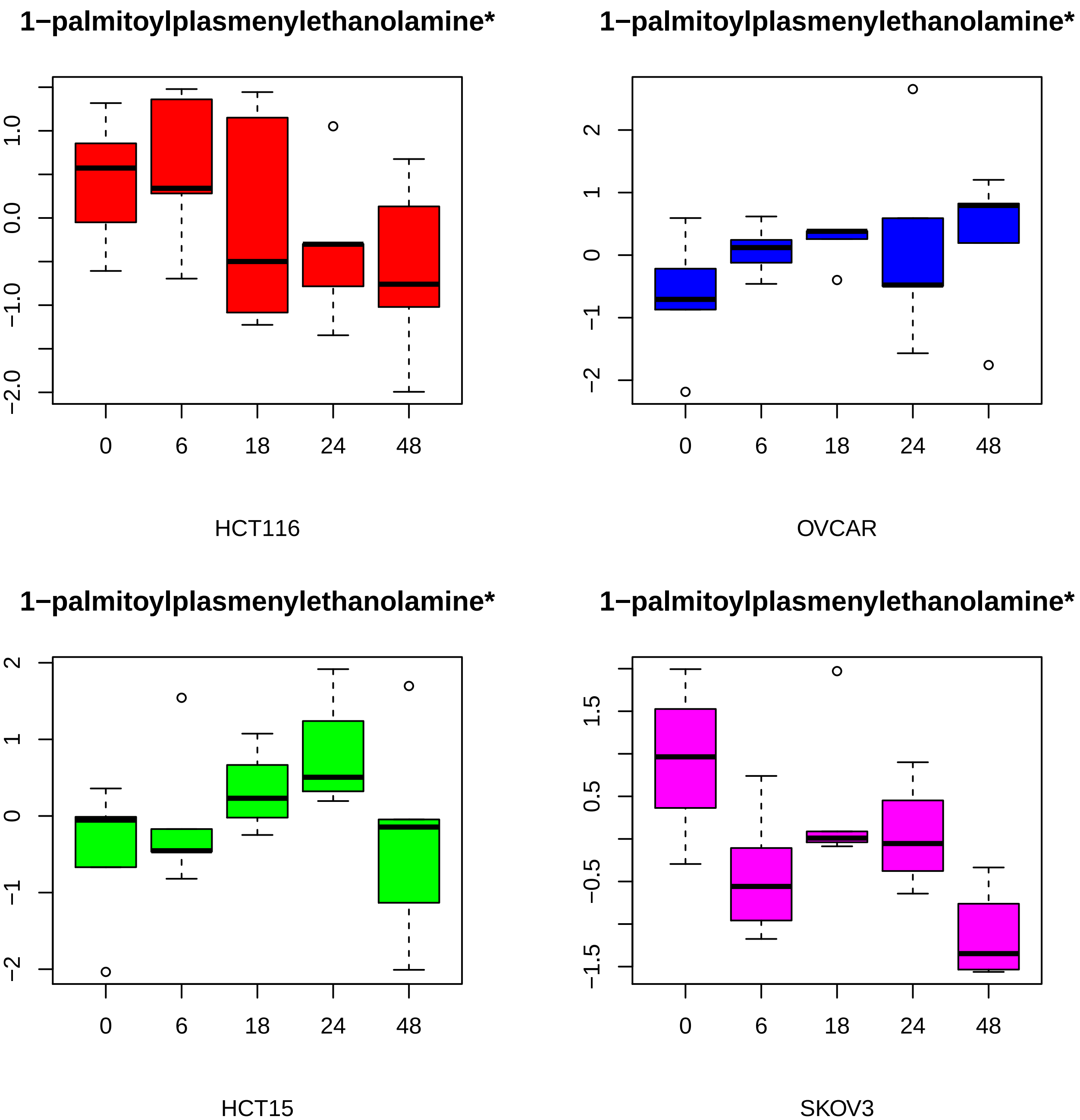
1−palmitoylplasmenylethanolamine*
1.0
0.0
−1.0
−2.0
0
6
18
24
48
HCT116
1−palmitoylplasmenylethanolamine*
2
1
0
−1
−2
0
6
18
24
48
OVCAR
1−palmitoylplasmenylethanolamine*
2
1
0
−1
−2
0
6
18
24
48
HCT15
1−palmitoylplasmenylethanolamine*
1.5
0.5
−0.5
−1.5
0
6
18
24
48
SKOV3

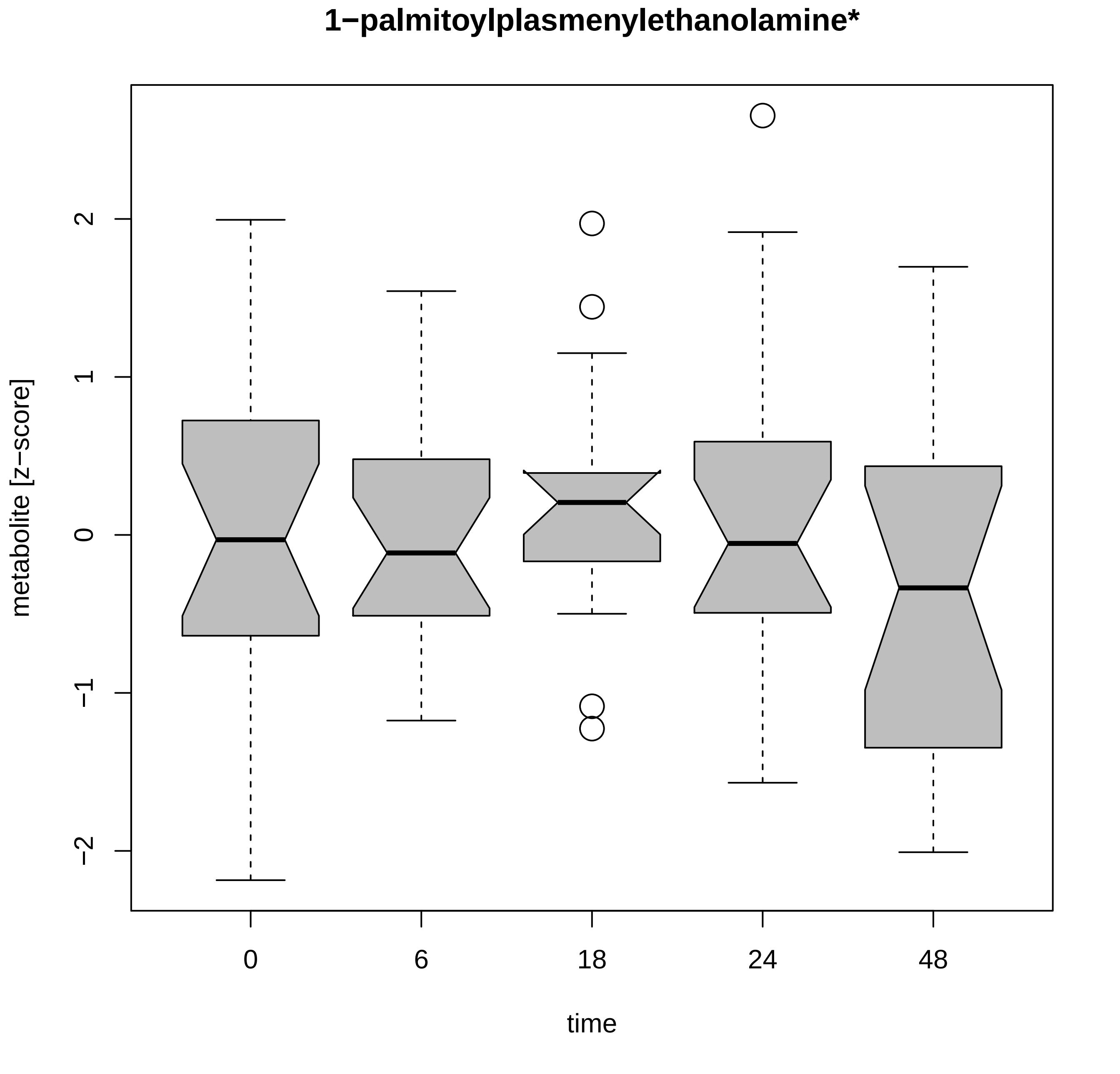
1−palmitoylplasmenylethanolamine*
metabolite [z−score]
−2
−1
0
1
2
0
6
18
24
48
time

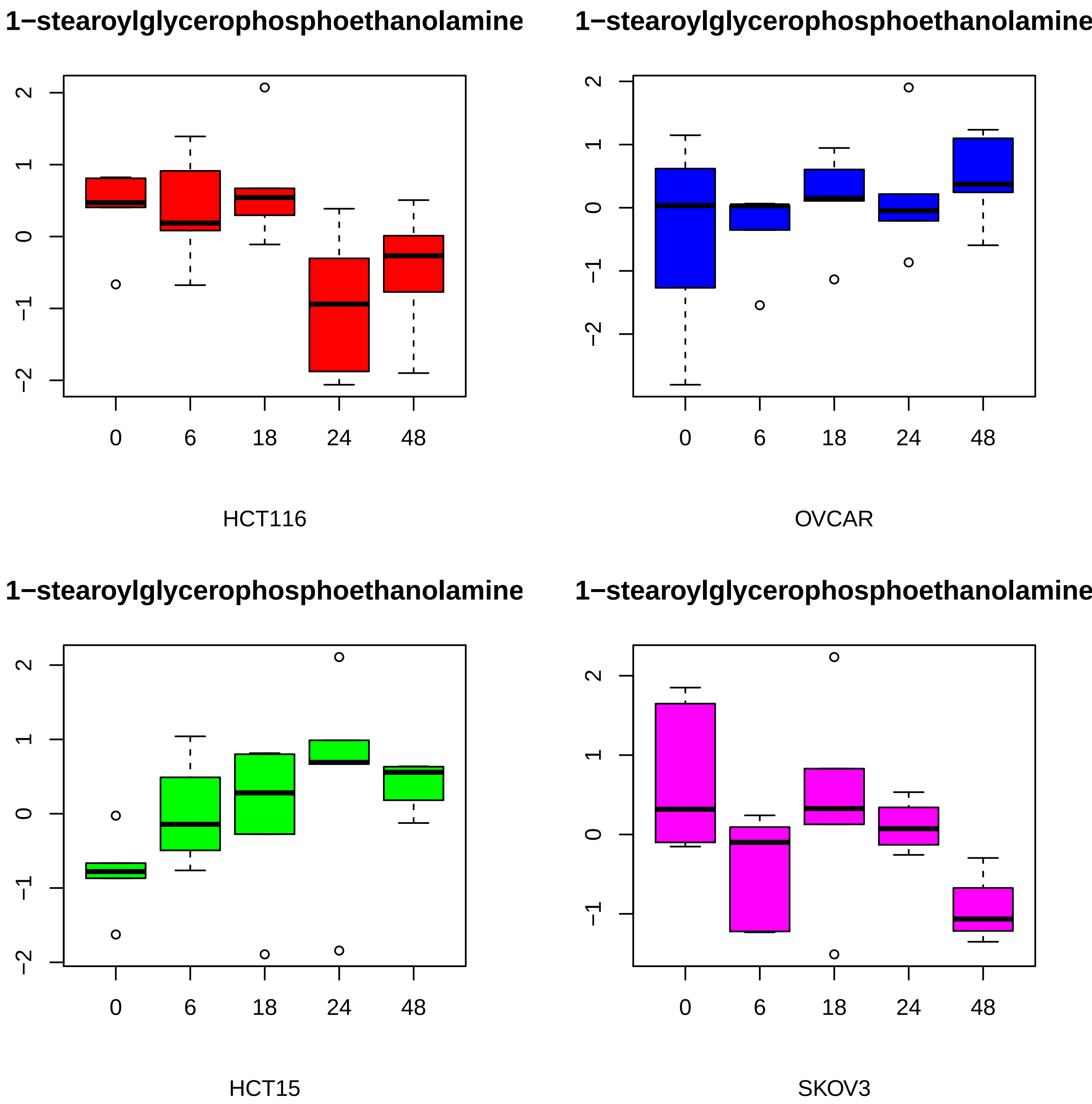
1−stearoylglycerophosphoethanolamine
HCT116
1−stearoylglycerophosphoethanolamine
OVCAR
1−stearoylglycerophosphoethanolamine
HCT15
1−stearoylglycerophosphoethanolamine
SKOV3
0
6
18
24
48

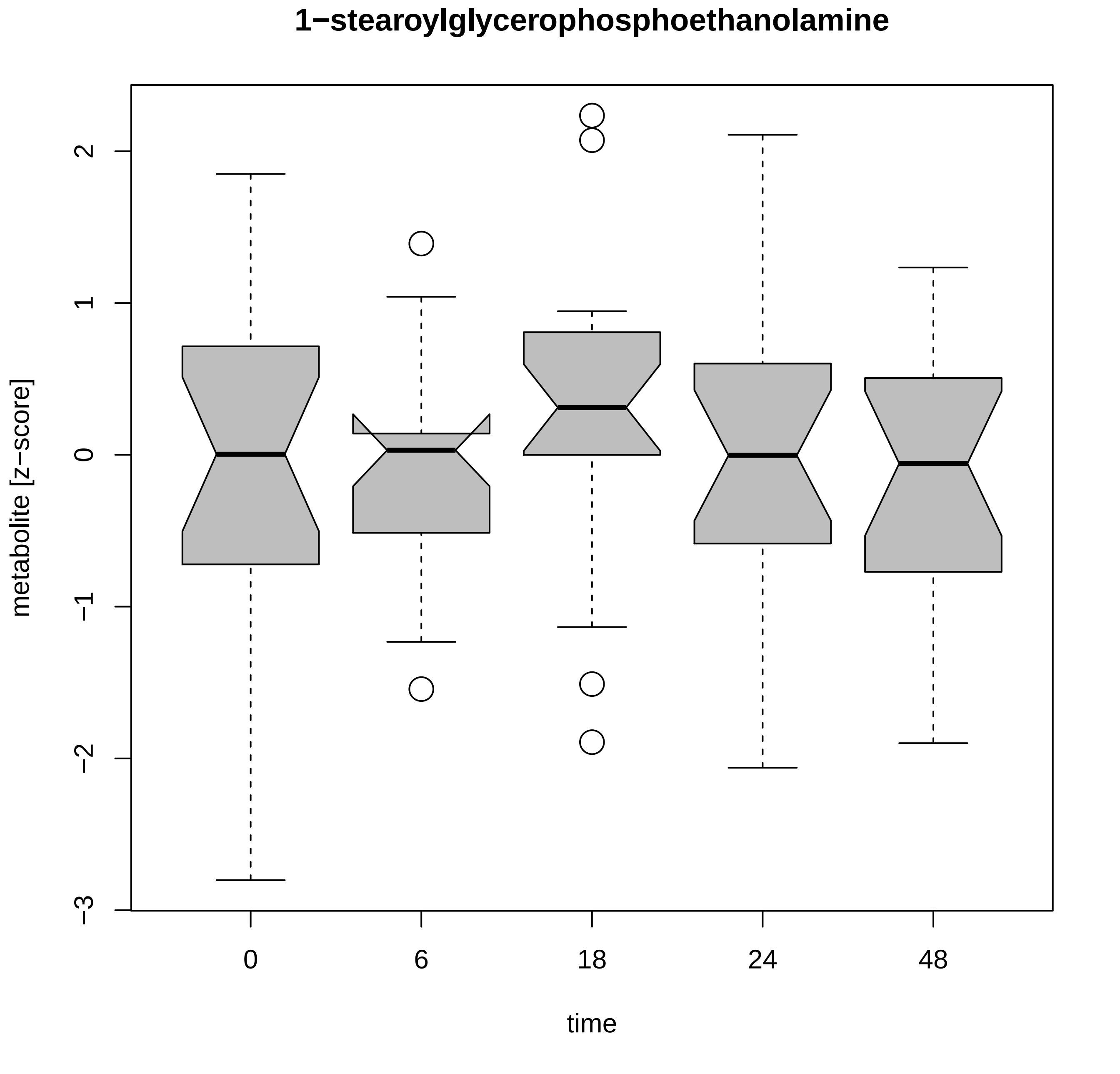
1−stearoylglycerophosphoethanolamine
metabolite [z−score]
2
1
0
−1
−2
−3
0
6
18
24
48
time

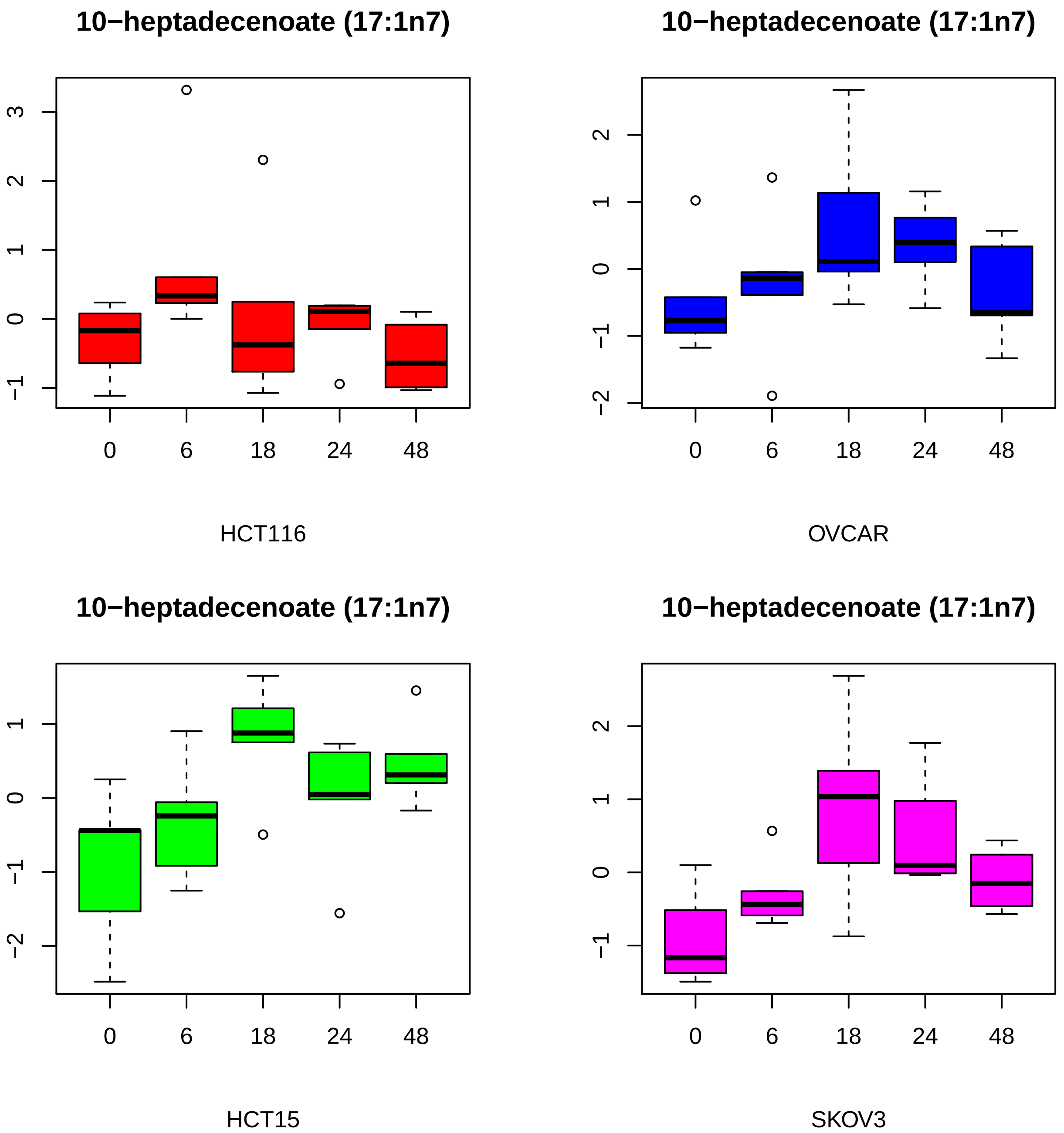
10−heptadecenoate (17:1n7)
HCT116
10−heptadecenoate (17:1n7)
OVCAR
10−heptadecenoate (17:1n7)
HCT15
10−heptadecenoate (17:1n7)
SKOV3
0
6
18
24
48
−2
−1
0
1
2
3

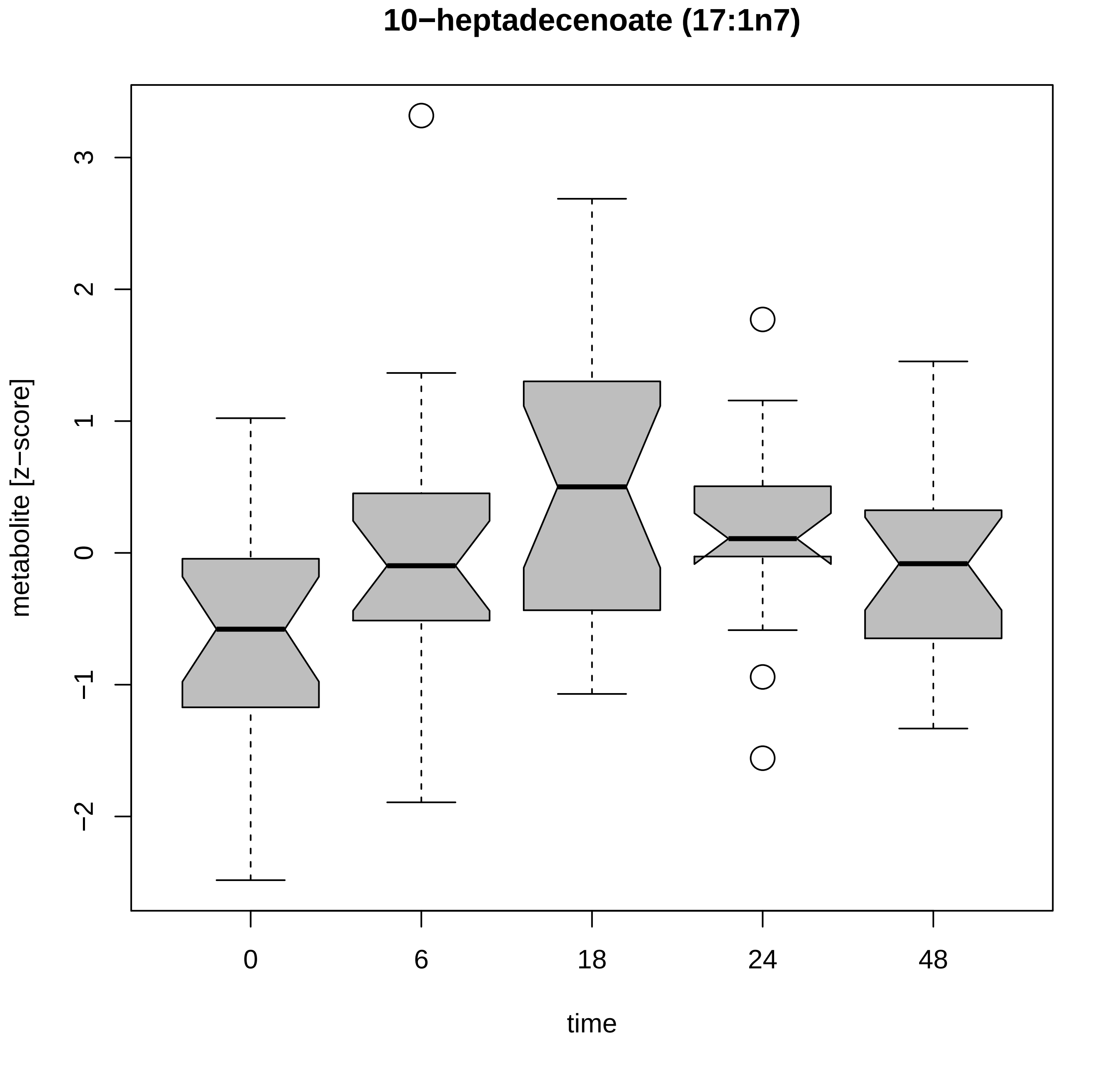
10−heptadecenoate (17:1n7)
metabolite [z−score]
time
3
2
1
0
−1
−2
0
6
18
24
48

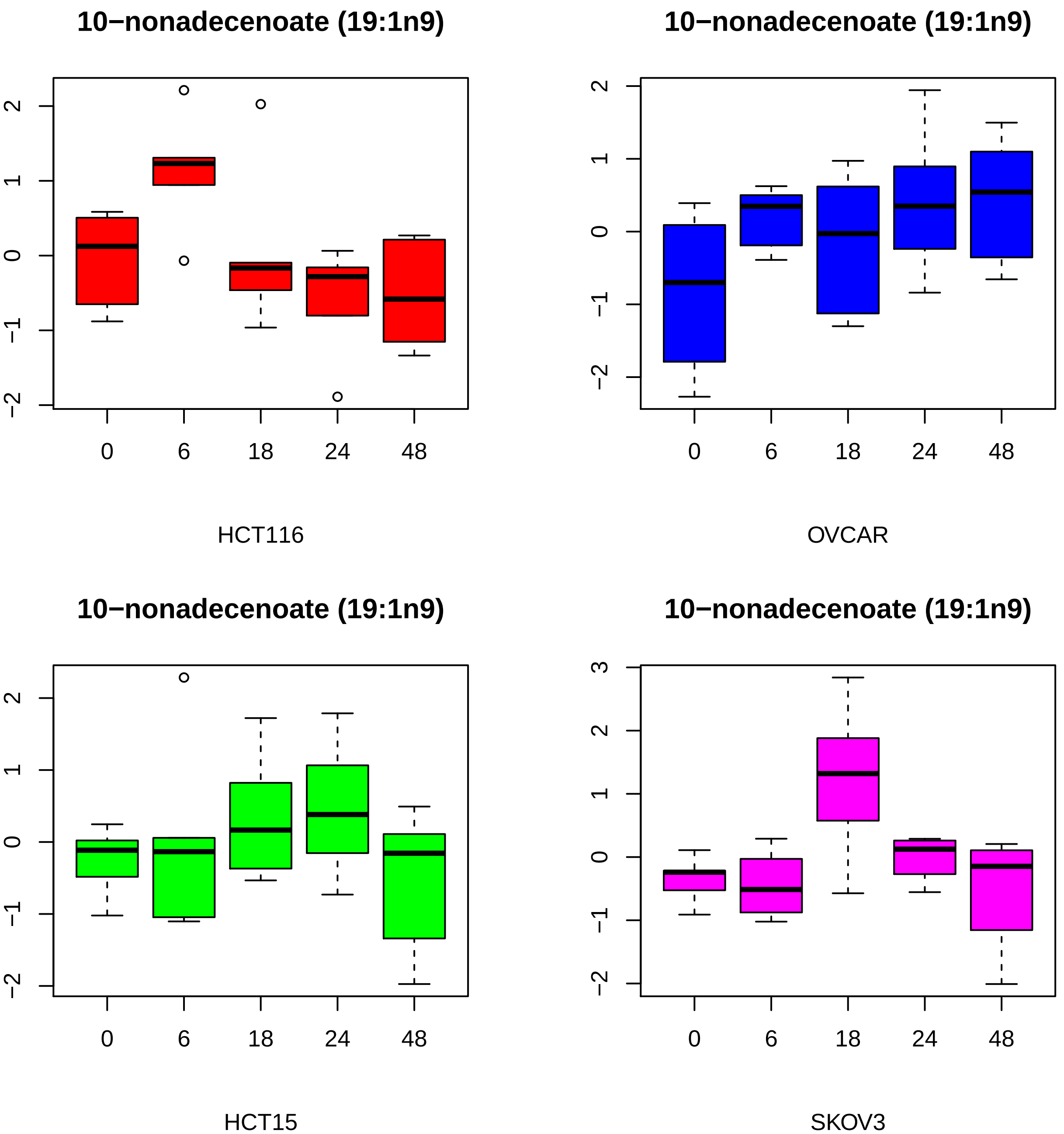
10−nonadecenoate (19:1n9)
−2
−1
0
1
2
0
6
18
24
48
HCT116
10−nonadecenoate (19:1n9)
−2
−1
0
1
2
0
6
18
24
48
OVCAR
10−nonadecenoate (19:1n9)
−2
−1
0
1
2
0
6
18
24
48
HCT15
10−nonadecenoate (19:1n9)
−2
−1
0
1
2
3
0
6
18
24
48
SKOV3

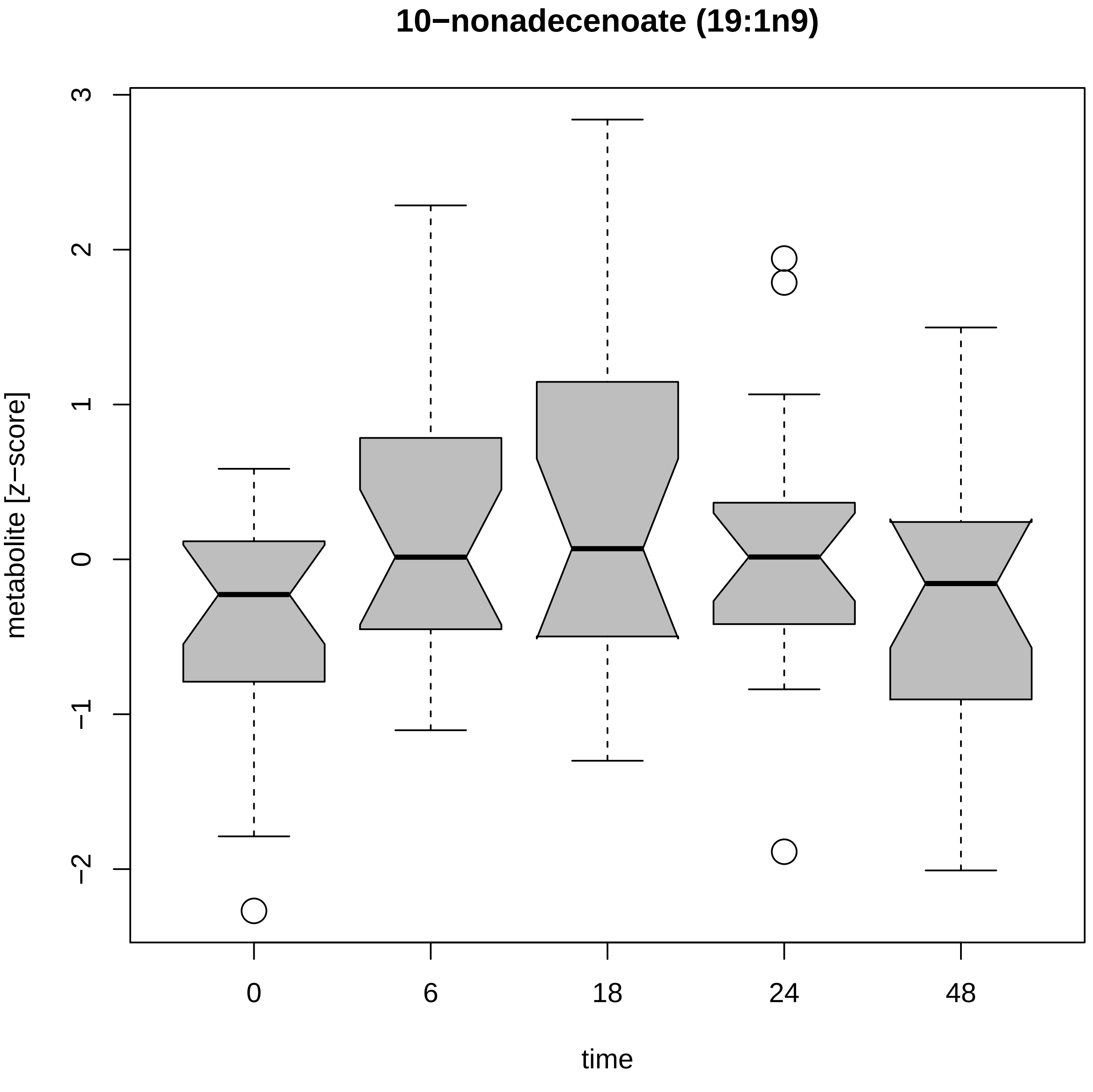

10−nonadecenoate (19:1n9)
metabolite [z−score]
−2
−1
0
1
2
3
0
6
18
24
48
time

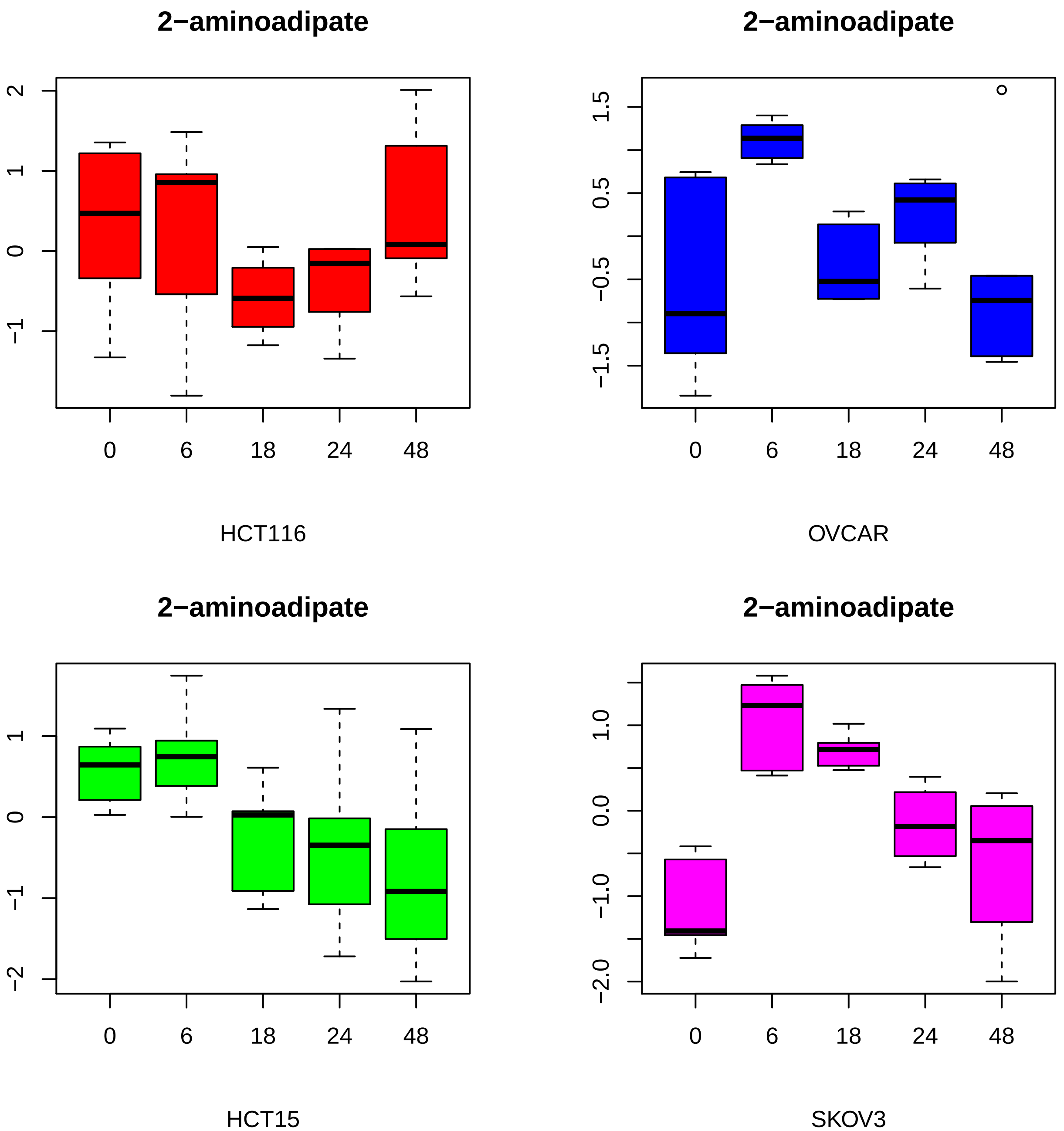
2−aminoadipate
−1
0
1
2
0
6
18
24
48
HCT116
2−aminoadipate
−1.5
−0.5
0.5
1.5
0
6
18
24
48
OVCAR
2−aminoadipate
−2
−1
0
1
0
6
18
24
48
HCT15
2−aminoadipate
−2.0
−1.0
0.0
1.0
0
6
18
24
48
SKOV3

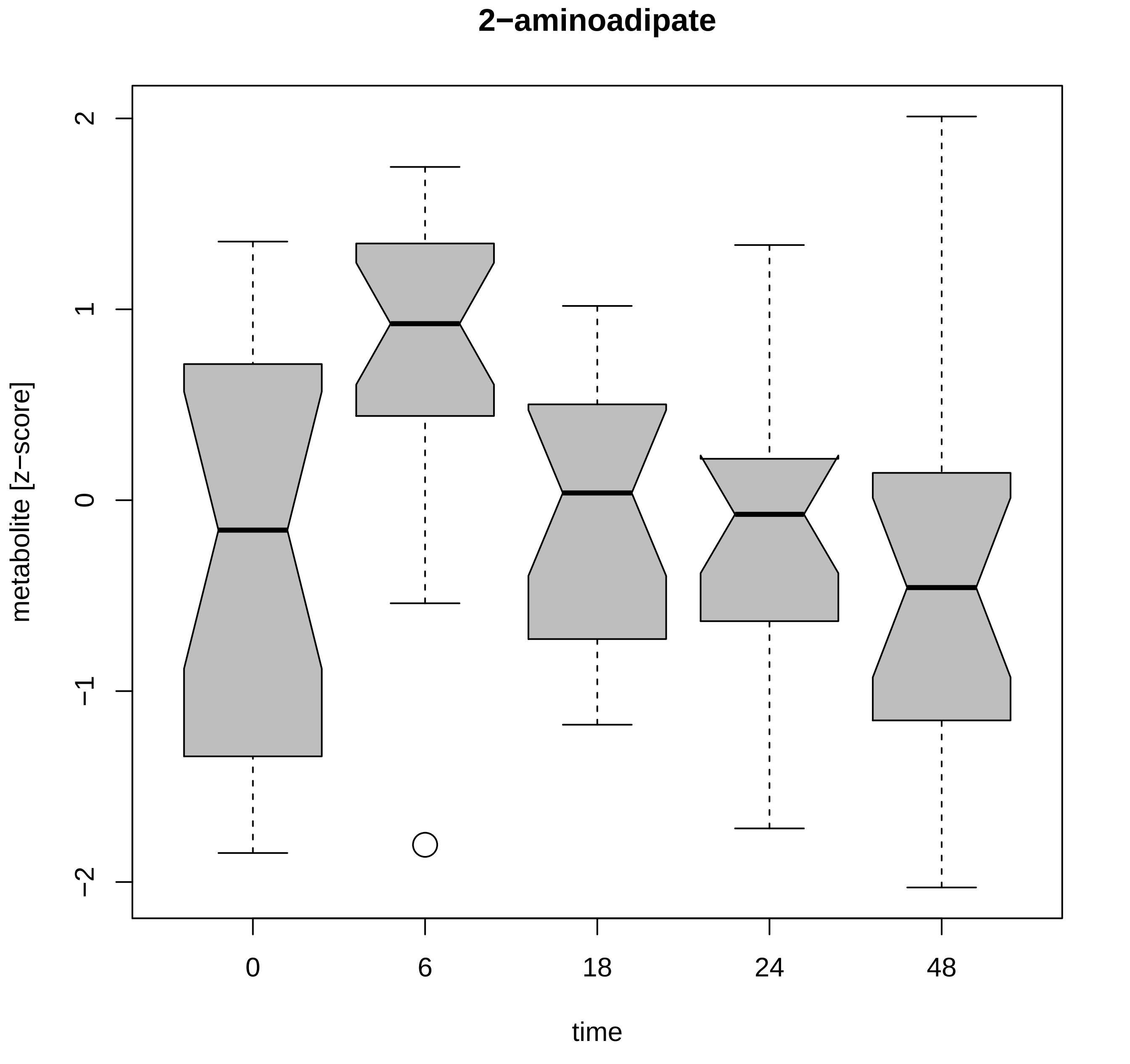
2−aminoadipate
metabolite [z−score]
time
2
1
0
−1
−2
0
6
18
24
48

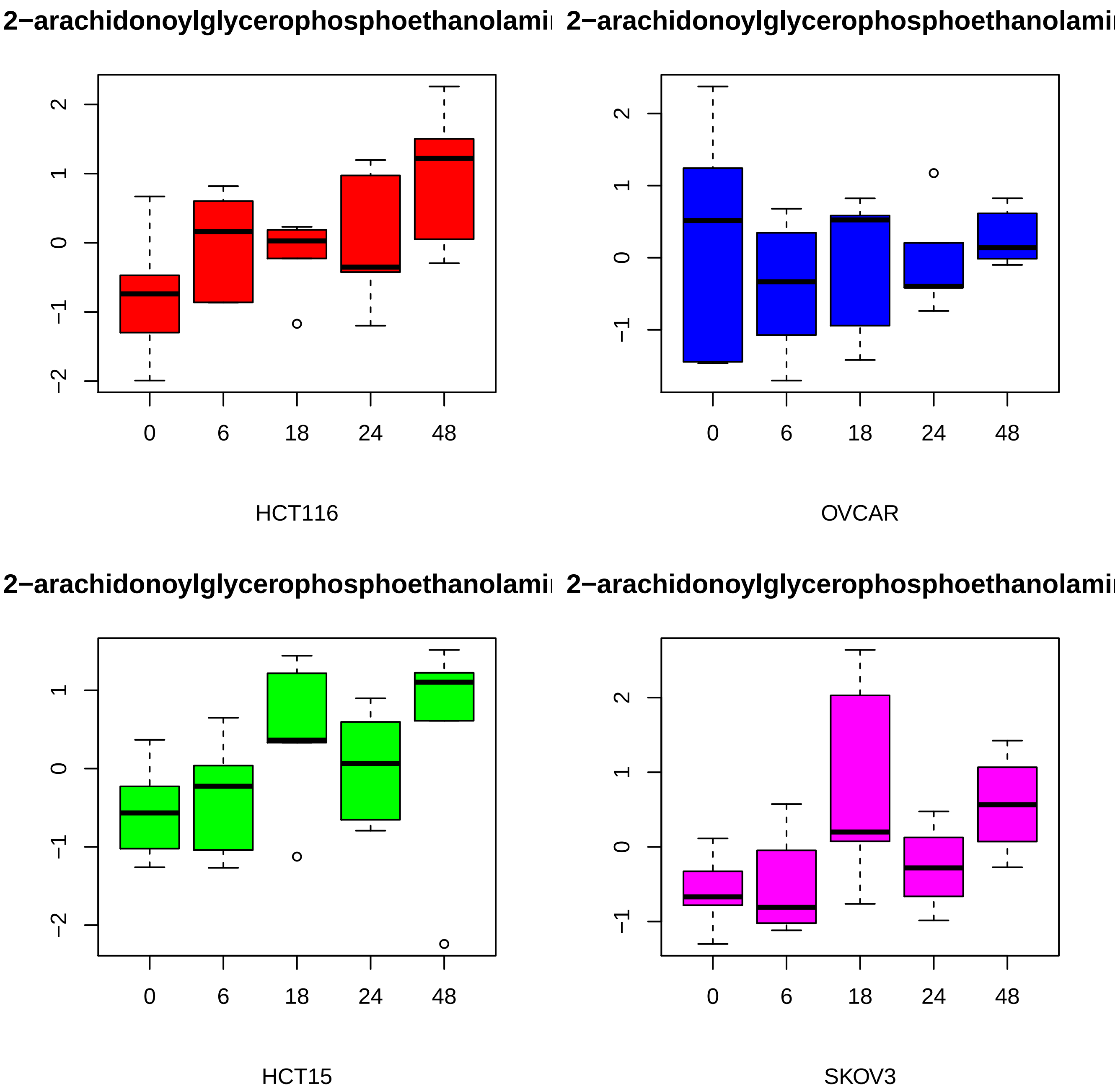
2−arachidonoylglycerophosphoethanolami
2−arachidonoylglycerophosphoethanolami
2−arachidonoylglycerophosphoethanolami
2−arachidonoylglycerophosphoethanolami
−2
−1
0
1
2
0
6
18
24
48
HCT116
OVCAR
HCT15
SKOV3

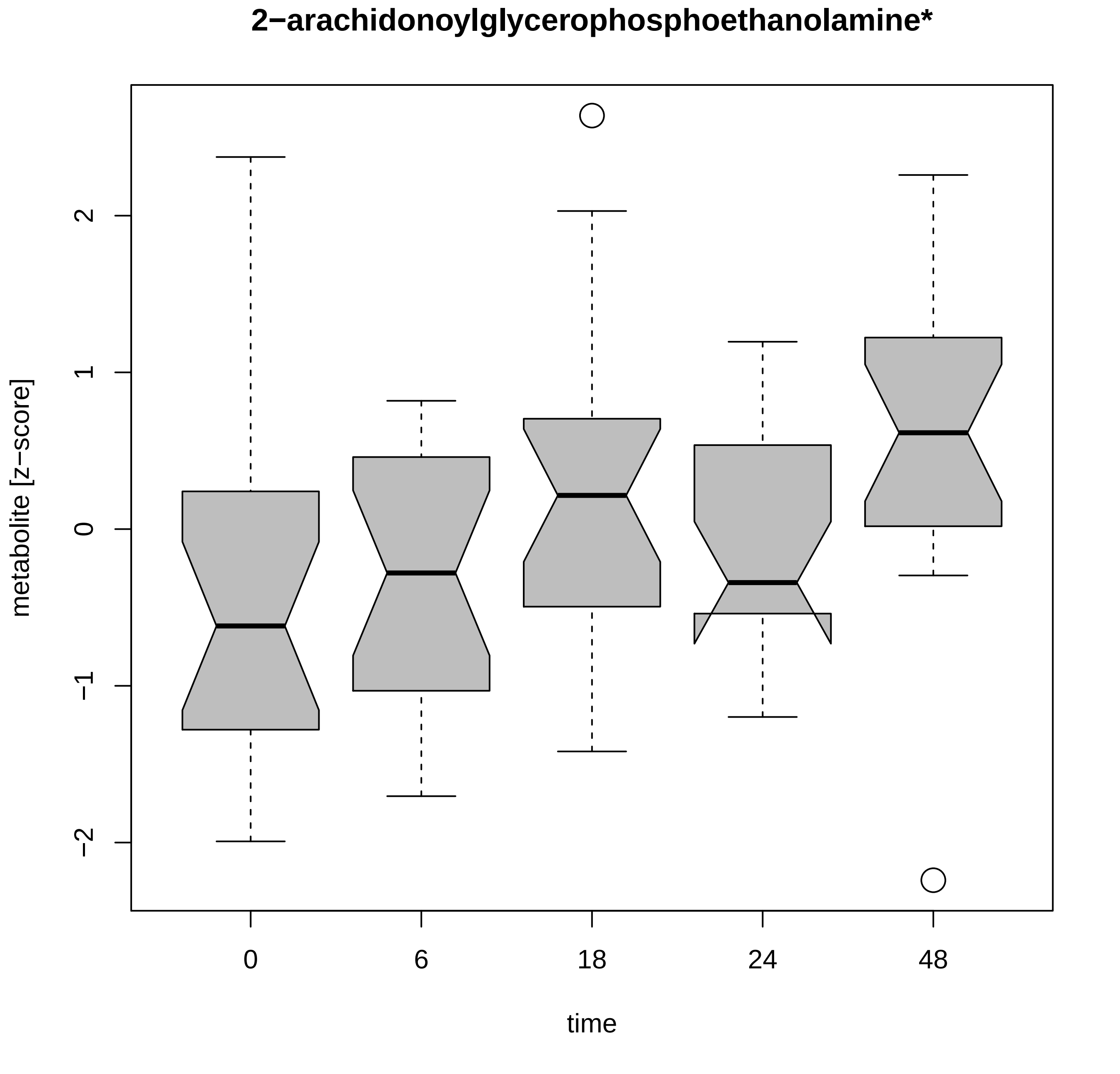
2−arachidonoylglycerophosphoethanolamine*
metabolite [z−score]
−2
−1
0
1
2
0
6
18
24
48
time

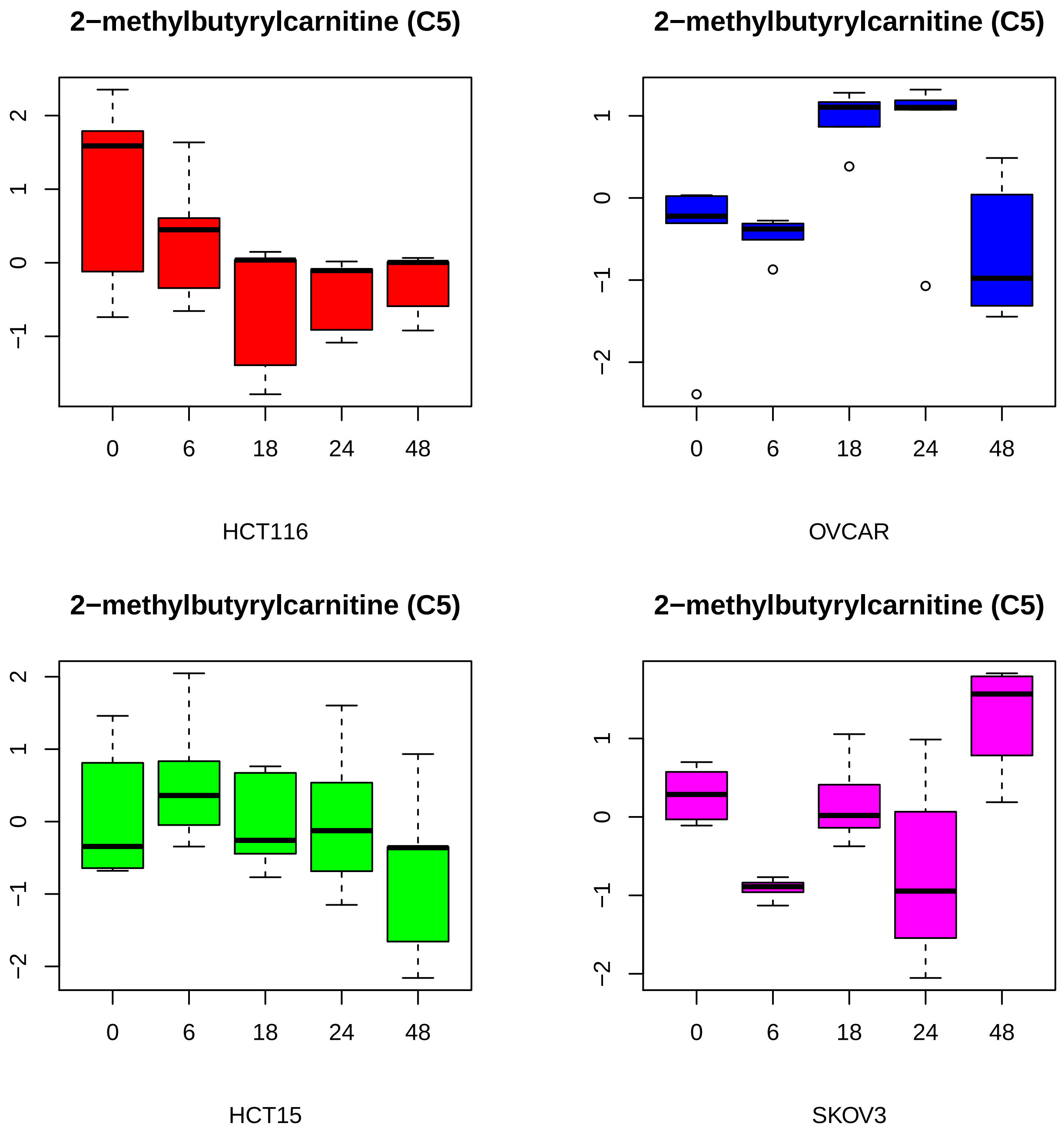
2−methylbutyrylcarnitine (C5)
HCT116
2−methylbutyrylcarnitine (C5)
OVCAR
2−methylbutyrylcarnitine (C5)
HCT15
2−methylbutyrylcarnitine (C5)
SKOV3
0
6
18
24
48

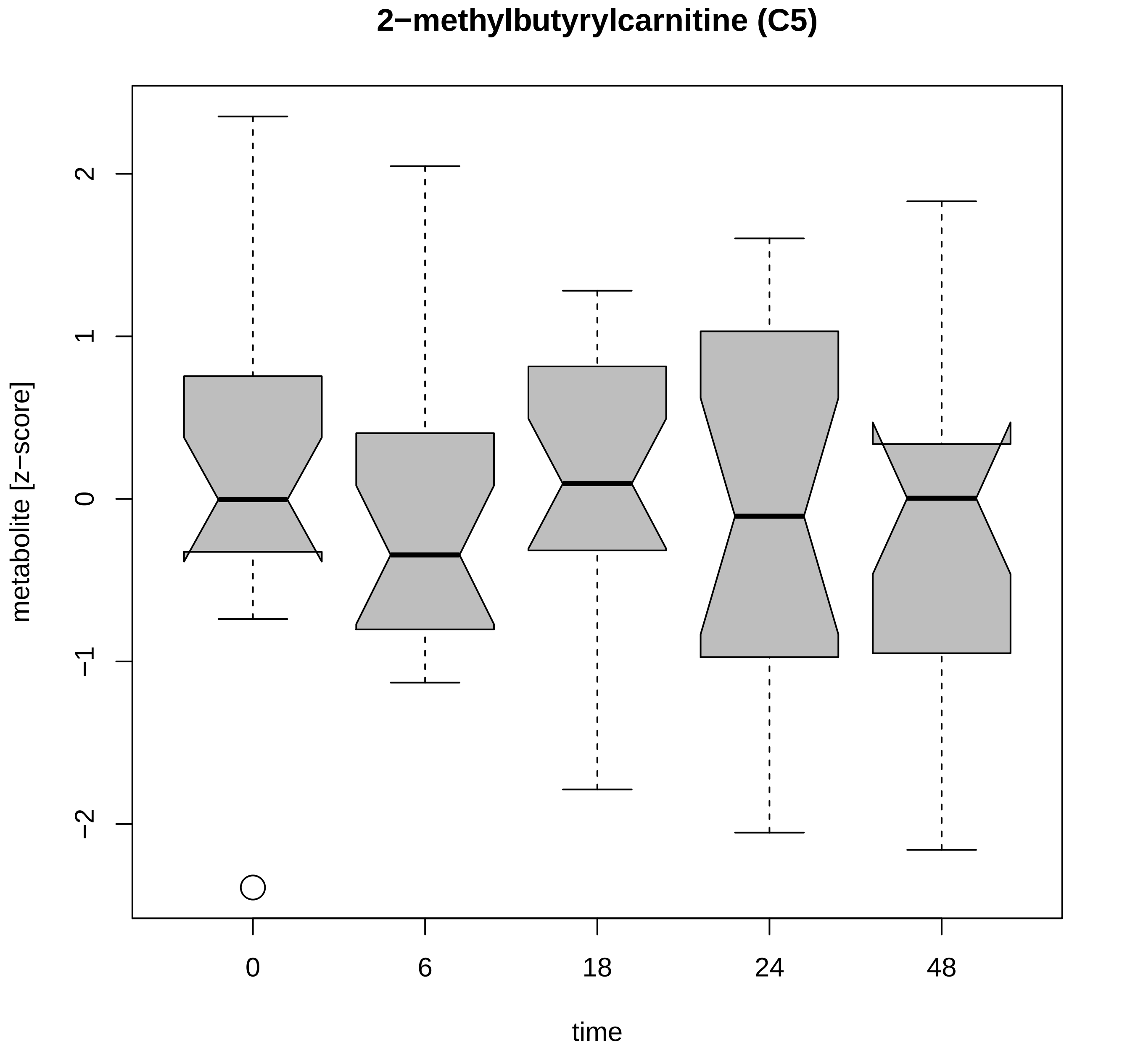
2−methylbutyrylcarnitine (C5)
metabolite [z−score]
2
1
0
−1
−2
0
6
18
24
48
time

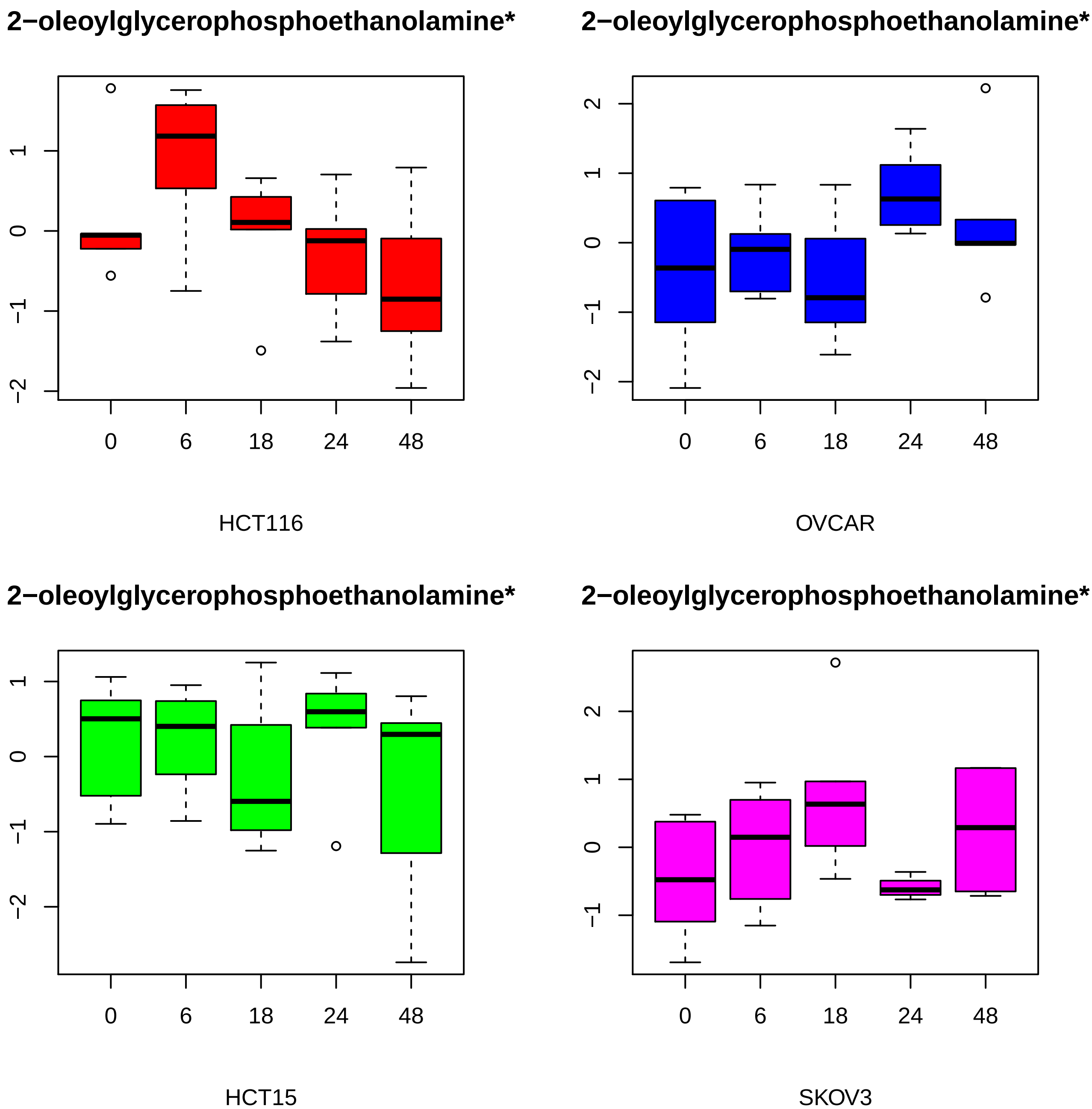

2−oleoylglycerophosphoethanolamine*
HCT116
2−oleoylglycerophosphoethanolamine*
OVCAR
2−oleoylglycerophosphoethanolamine*
HCT15
2−oleoylglycerophosphoethanolamine*
SKOV3
0
6
18
24
48

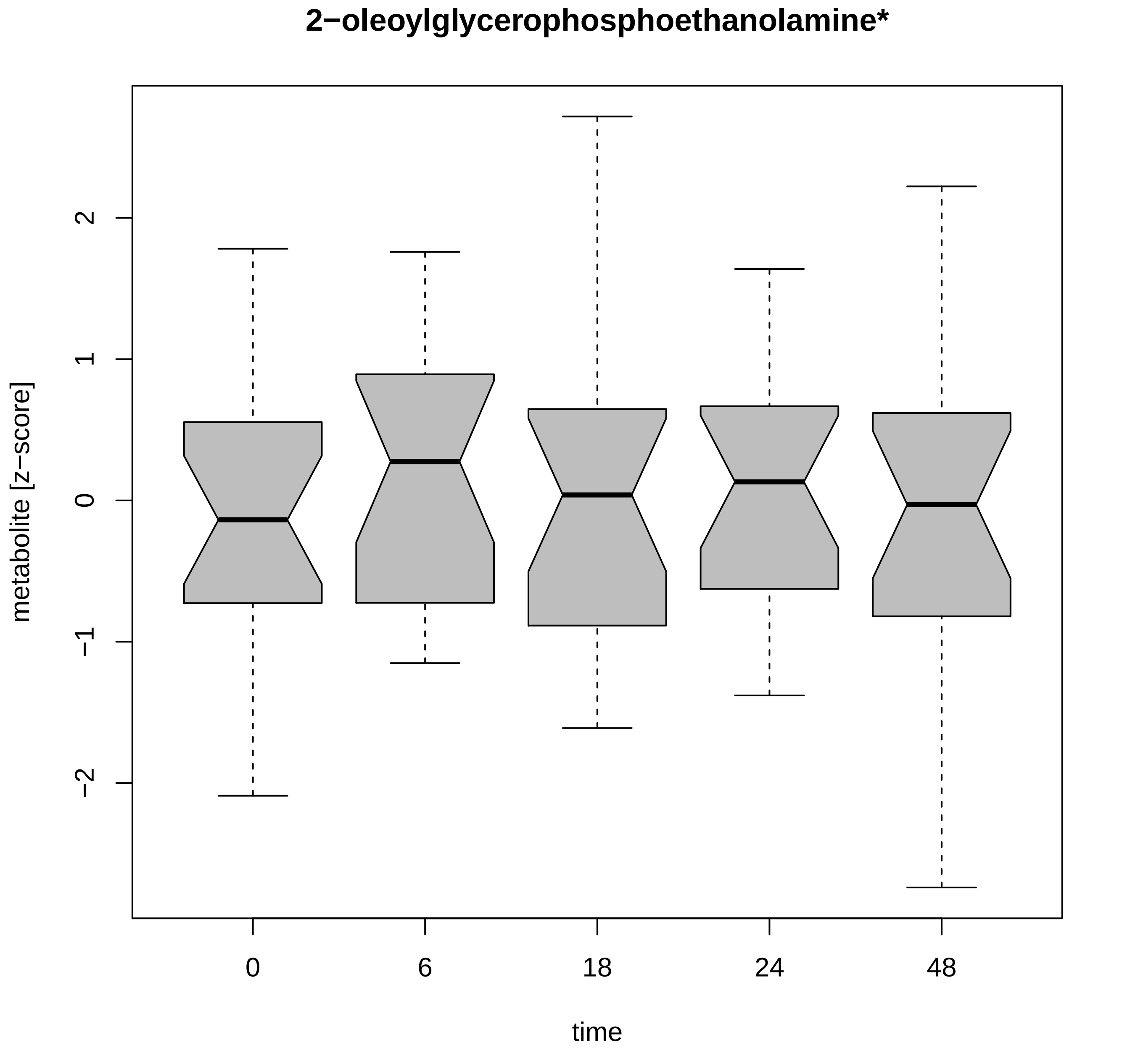

2−oleoylglycerophosphoethanolamine*
metabolite [z−score]
2
1
0
−1
−2
0
6
18
24
48
time

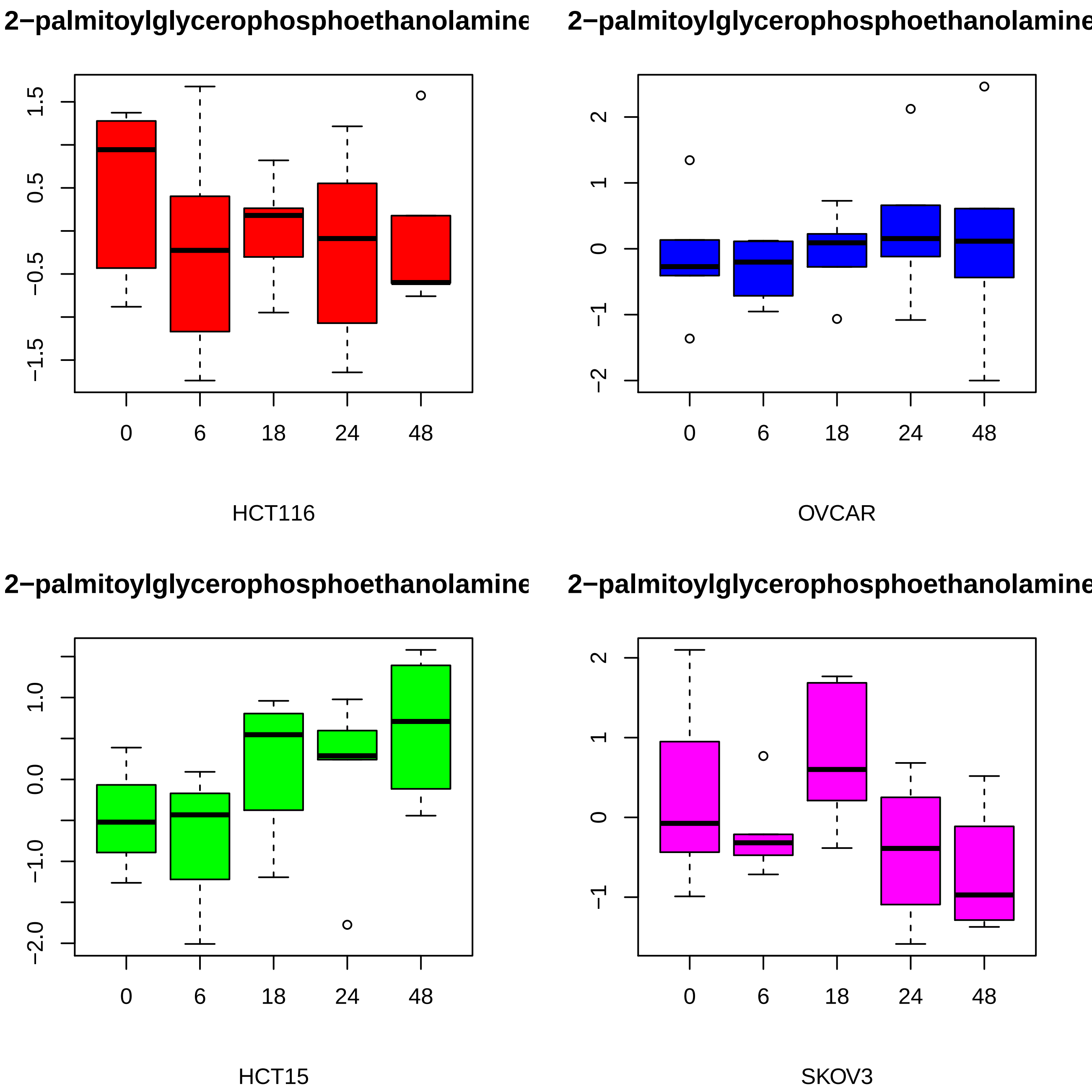

2−palmitoylglycerophosphoethanolamine
1.5
0.5
−0.5
−1.5
0
6
18
24
48
HCT116
2−palmitoylglycerophosphoethanolamine
2
1
0
−1
−2
0
6
18
24
48
OVCAR
2−palmitoylglycerophosphoethanolamine
1.0
0.0
−1.0
−2.0
0
6
18
24
48
HCT15
2−palmitoylglycerophosphoethanolamine
2
1
0
−1
0
6
18
24
48
SKOV3

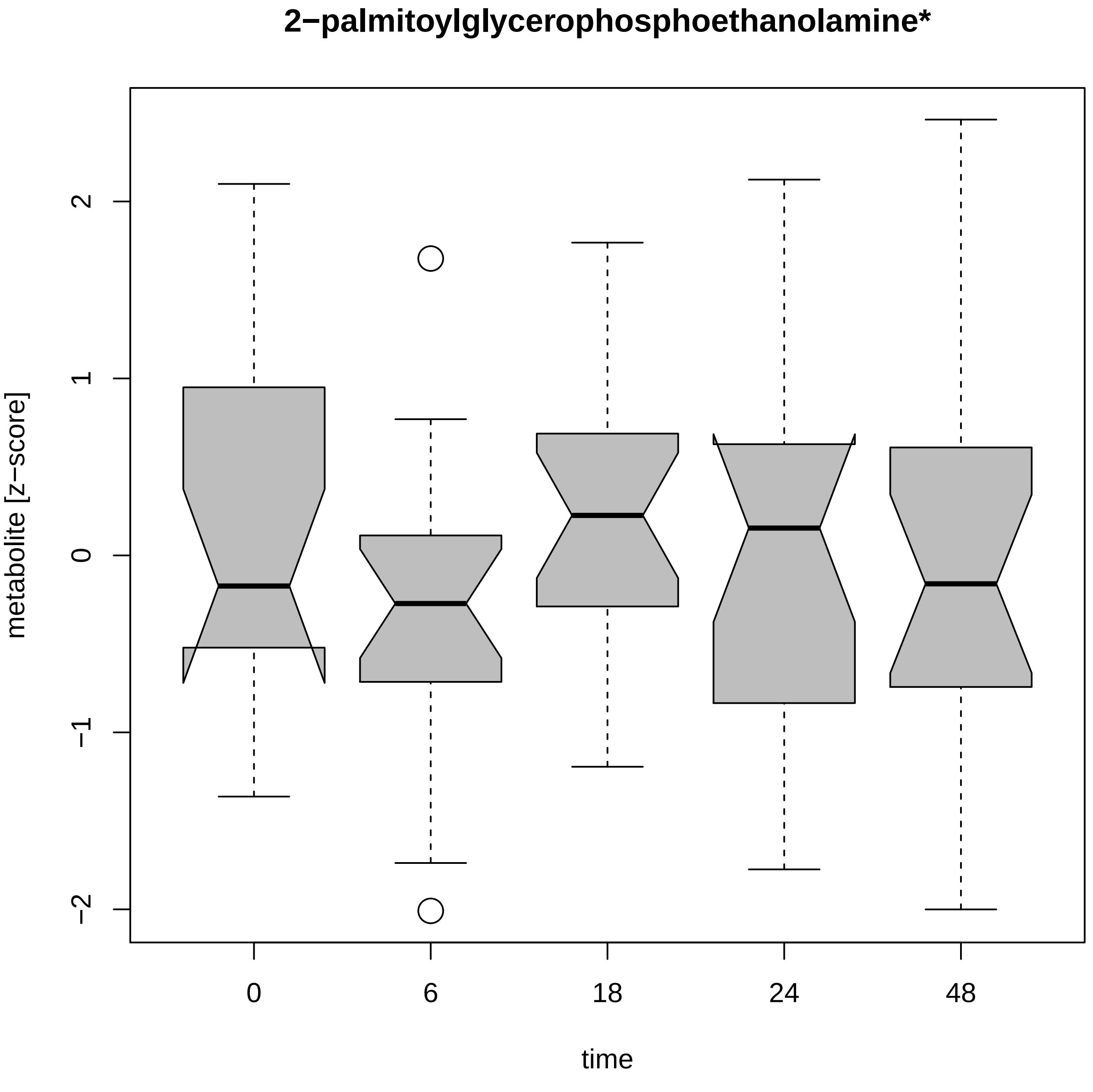
2−palmitoylglycerophosphoethanolamine*
metabolite [z−score]
−2
−1
0
1
2
0
6
18
24
48
time

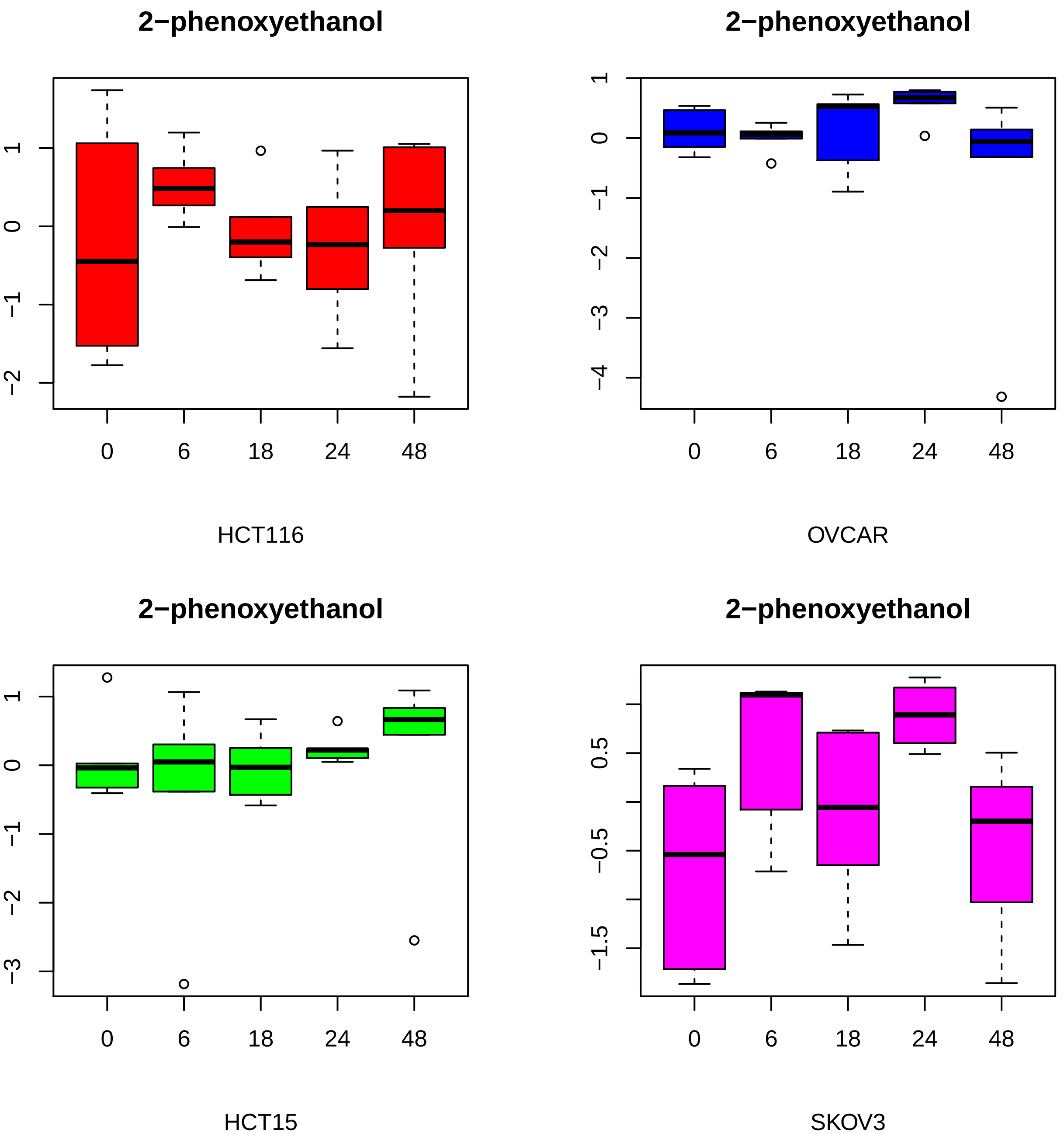
2−phenoxyethanol
1
0
−1
−2
0
6
18
24
48
HCT116
2−phenoxyethanol
1
0
−1
−2
−3
−4
0
6
18
24
48
OVCAR
2−phenoxyethanol
1
0
−1
−2
−3
0
6
18
24
48
HCT15
2−phenoxyethanol
0.5
−0.5
−1.5
0
6
18
24
48
SKOV3

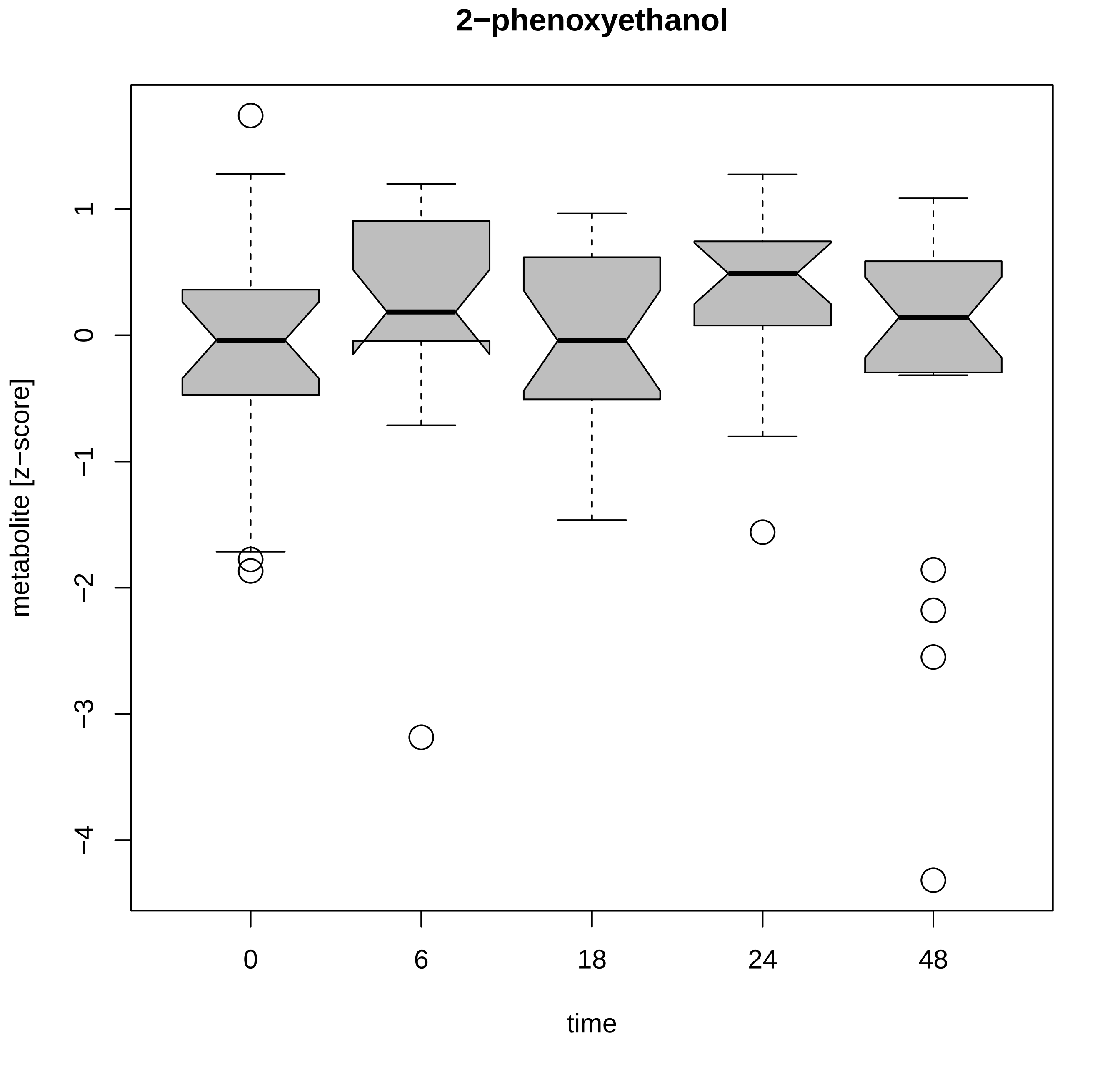
2−phenoxyethanol
metabolite [z−score]
1
0
−1
−2
−3
−4
0
6
18
24
48
time

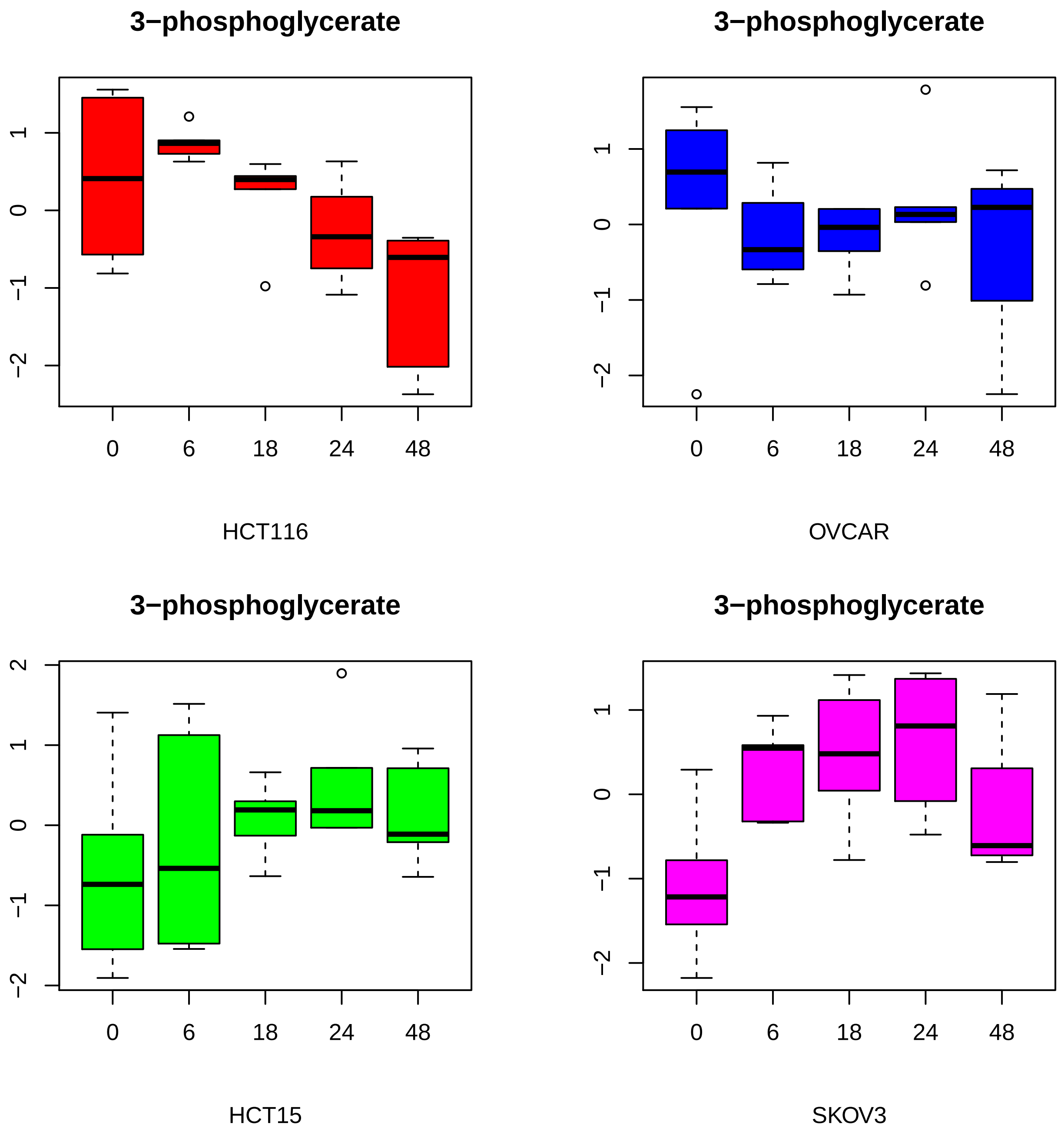
3−phosphoglycerate
HCT116
3−phosphoglycerate
OVCAR
3−phosphoglycerate
HCT15
3−phosphoglycerate
SKOV3
0
6
18
24
48
−2
−1
0
1
2

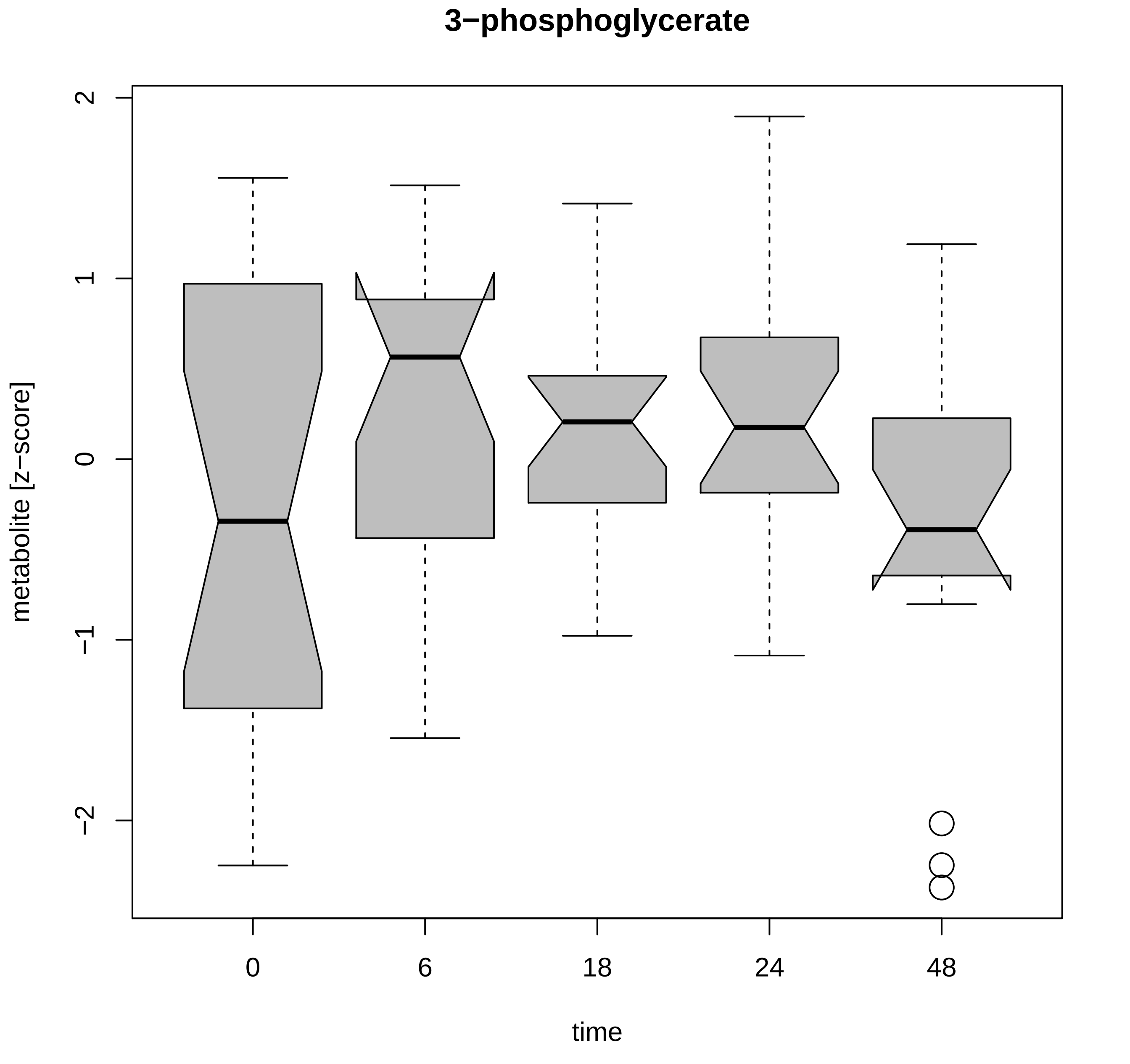
3−phosphoglycerate
metabolite [z−score]
2
1
0
−1
−2
0
6
18
24
48
time

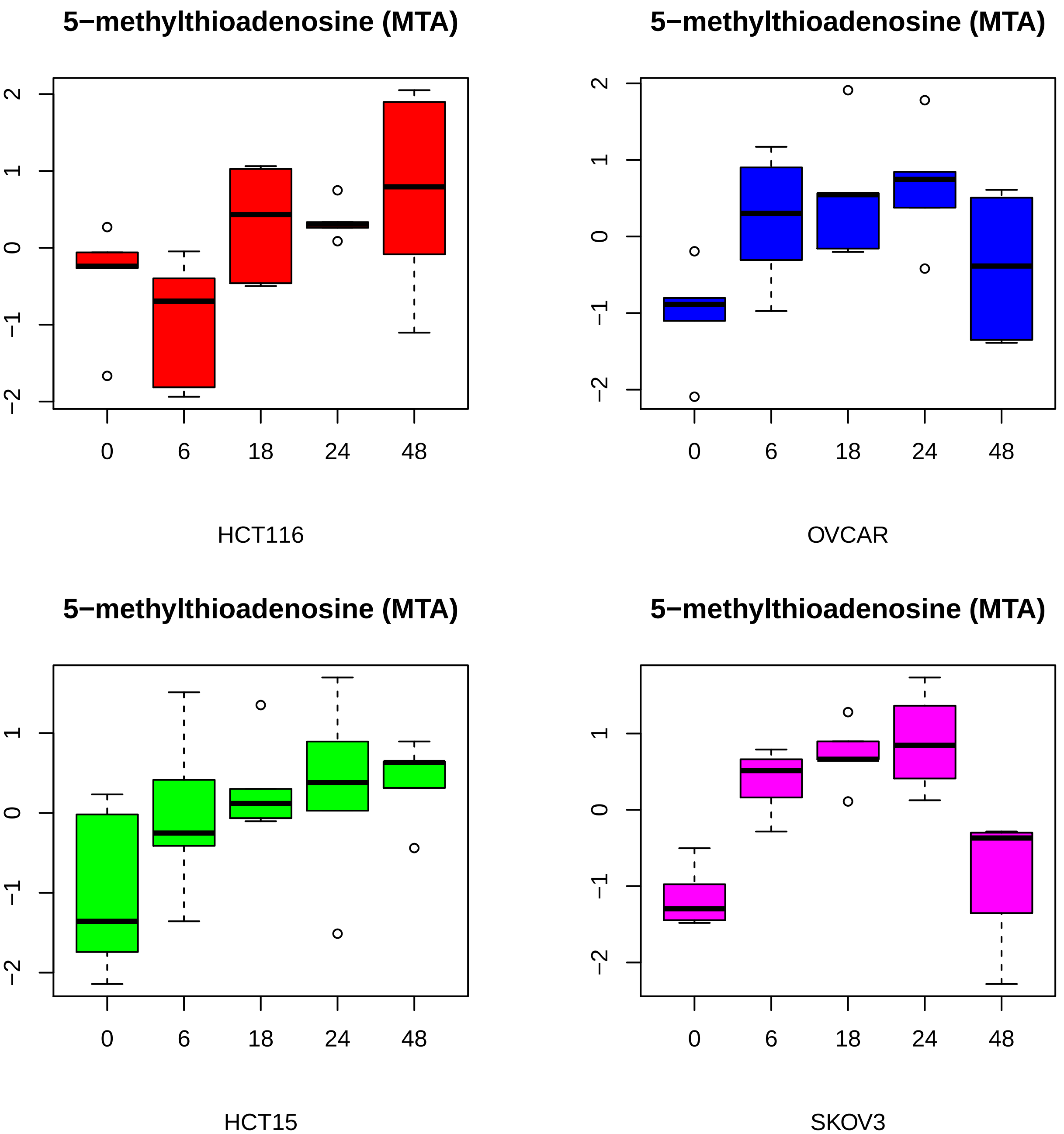
5−methylthioadenosine (MTA)
HCT116
OVCAR
HCT15
SKOV3
0
6
18
24
48
−2
−1
0
1
2

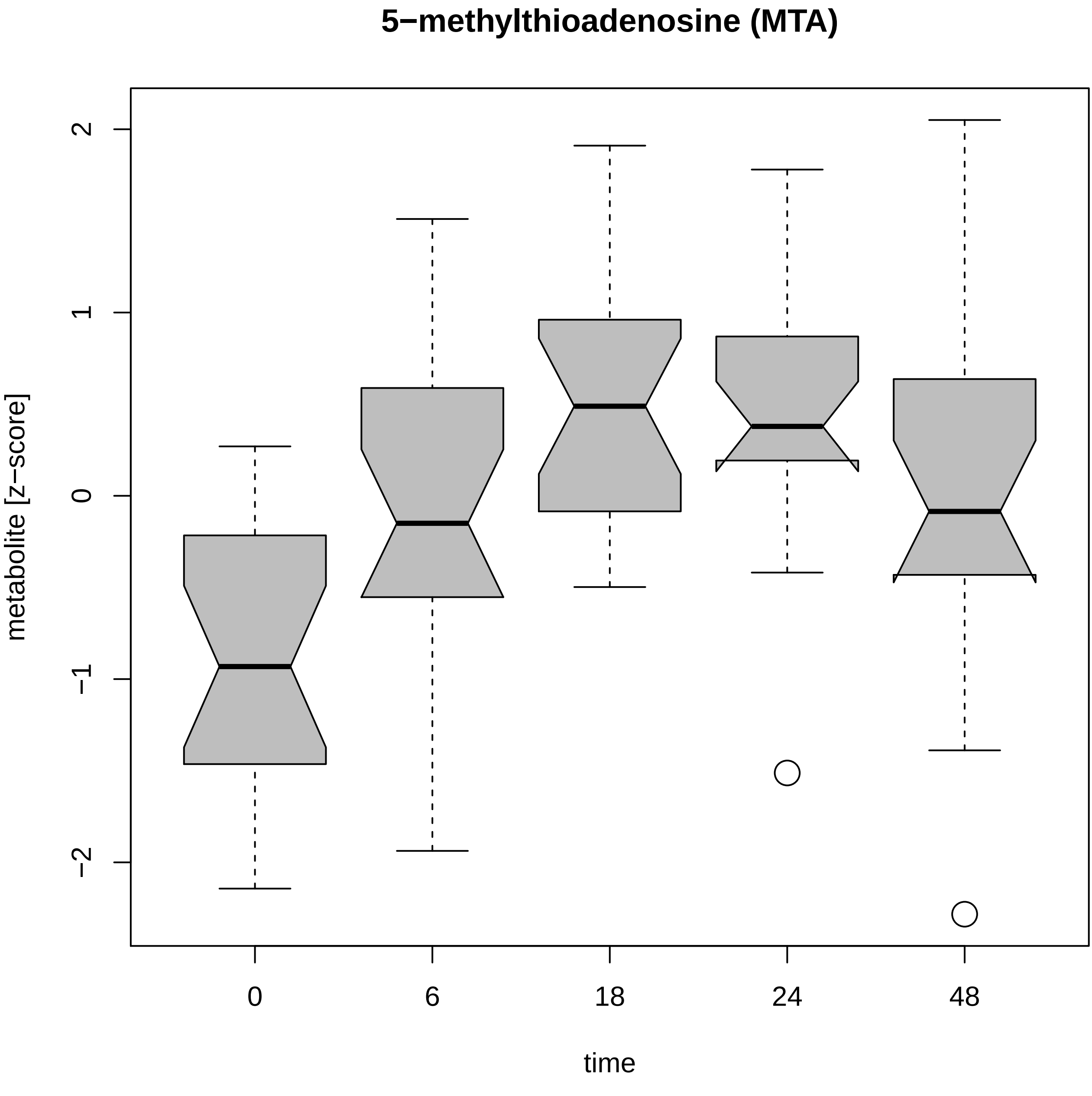

5−methylthioadenosine (MTA)
metabolite [z−score]
2
1
0
−1
−2
0
6
18
24
48
time

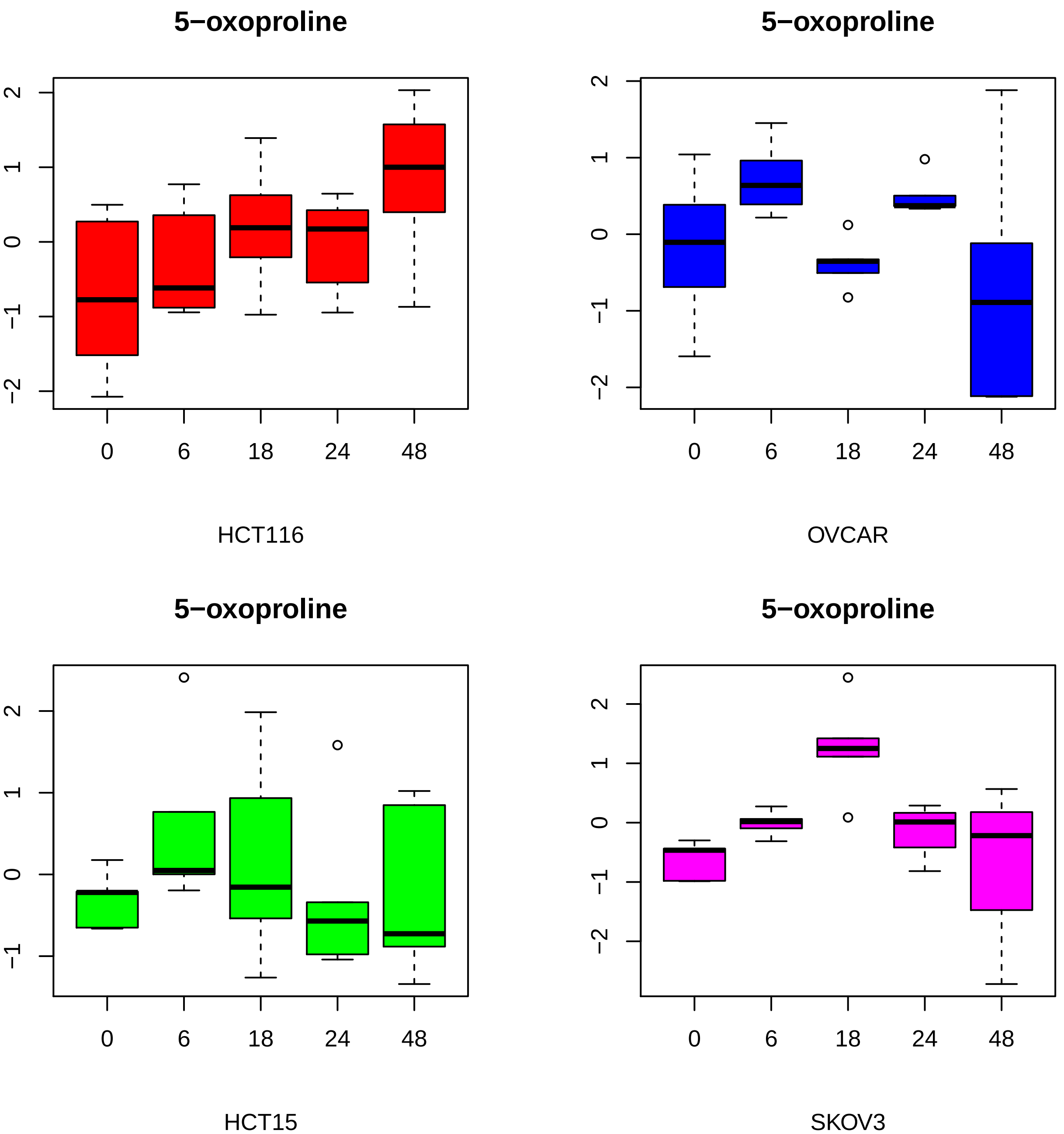
5−oxoproline
2
1
0
−1
−2
0
6
18
24
48
HCT116
5−oxoproline
2
1
0
−1
−2
0
6
18
24
48
OVCAR
5−oxoproline
2
1
0
−1
0
6
18
24
48
HCT15
5−oxoproline
2
1
0
−1
−2
0
6
18
24
48
SKOV3

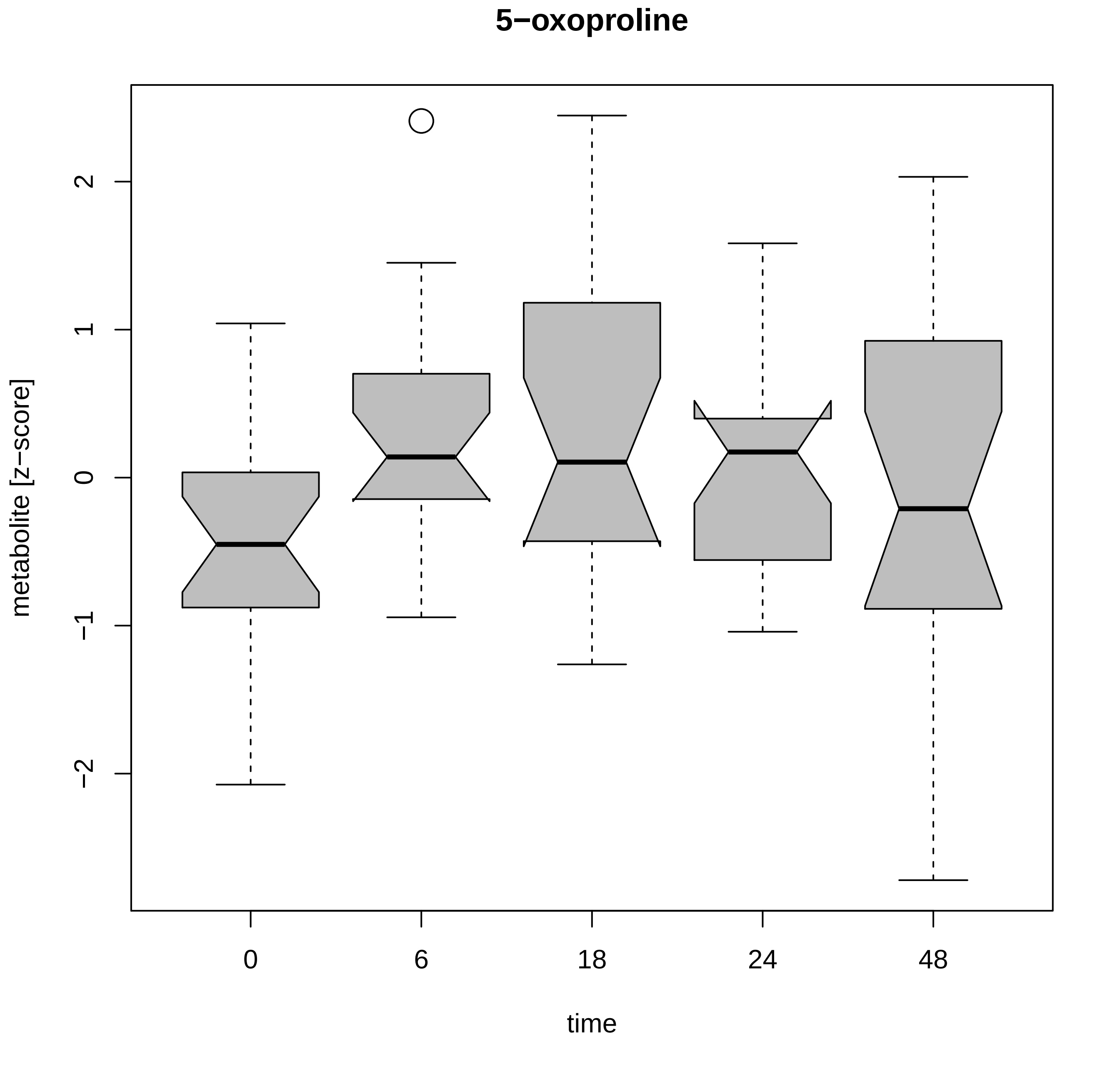
5−oxoproline
metabolite [z−score]
2
1
0
−1
−2
0
6
18
24
48
time

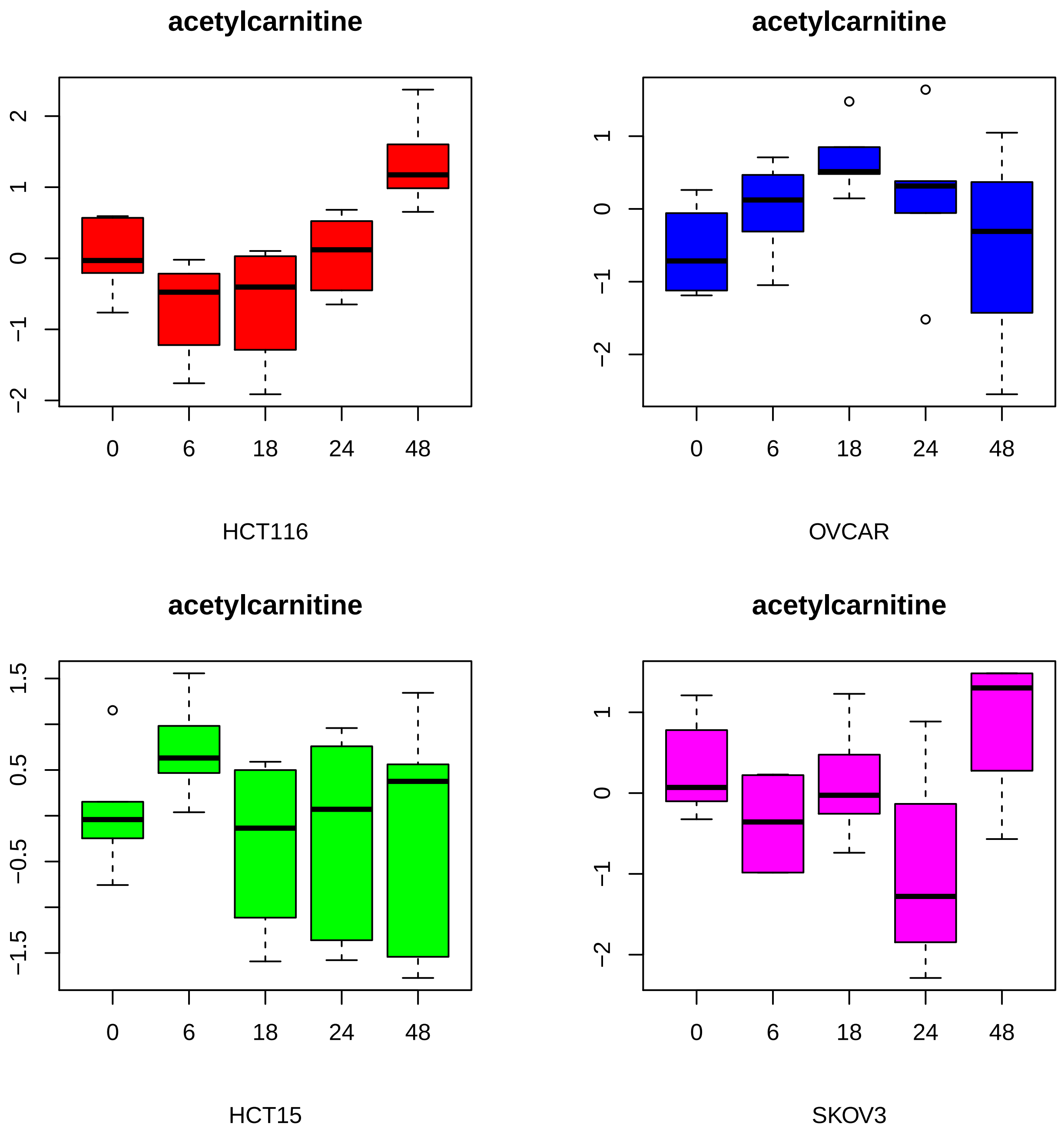
acetylcarnitine
2
1
0
−1
−2
0
6
18
24
48
HCT116
acetylcarnitine
1
0
−1
−2
0
6
18
24
48
OVCAR
acetylcarnitine
1.5
0.5
−0.5
−1.5
0
6
18
24
48
HCT15
acetylcarnitine
1
0
−1
−2
0
6
18
24
48
SKOV3

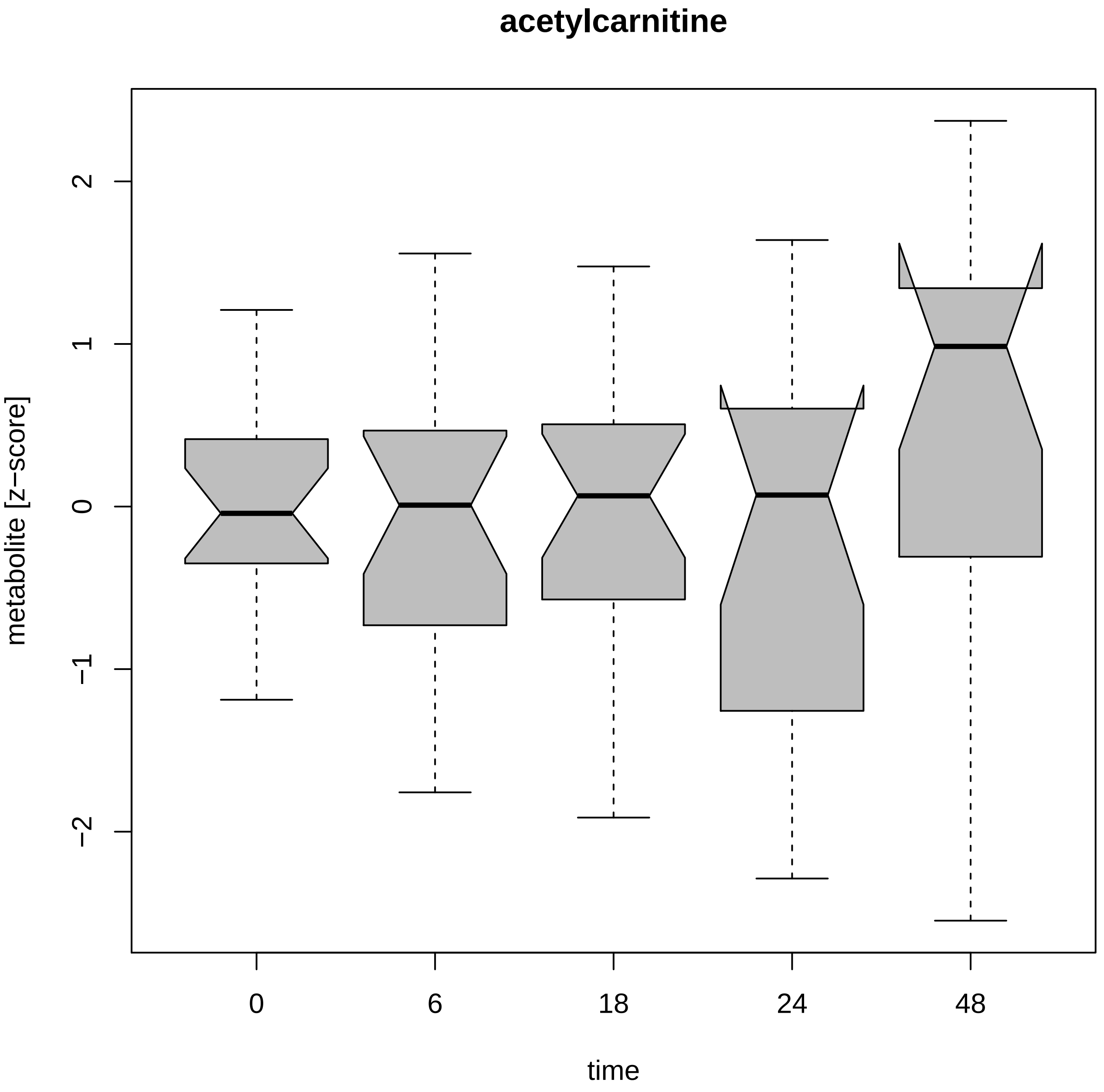
acetylcarnitine
metabolite [z−score]
2
1
0
−1
−2
0
6
18
24
48
time

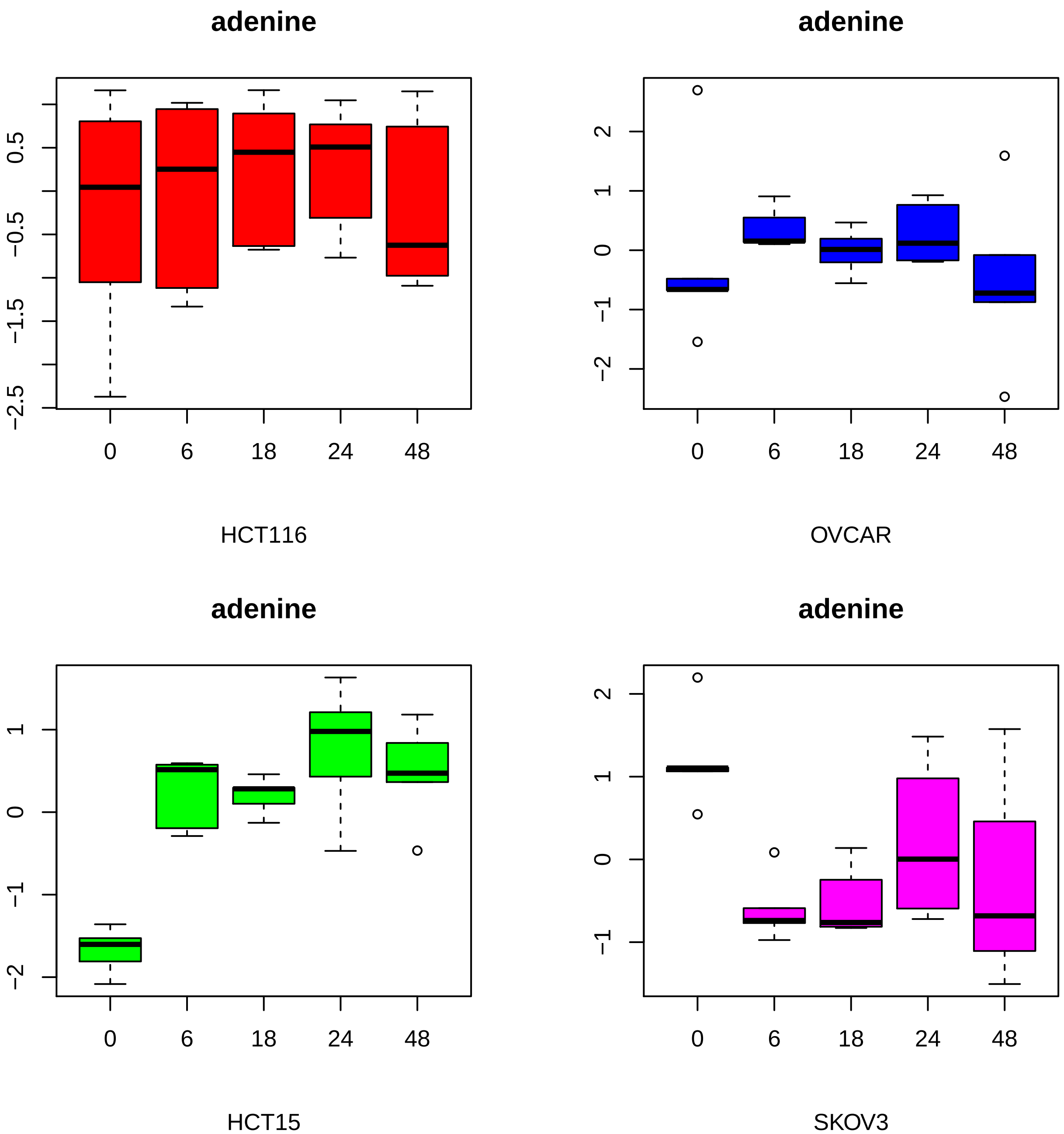
adenine
0.5
−0.5
−1.5
−2.5
0
6
18
24
48
HCT116
adenine
2
1
0
−1
−2
0
6
18
24
48
OVCAR
adenine
1
0
−1
−2
0
6
18
24
48
HCT15
adenine
2
1
0
−1
0
6
18
24
48
SKOV3

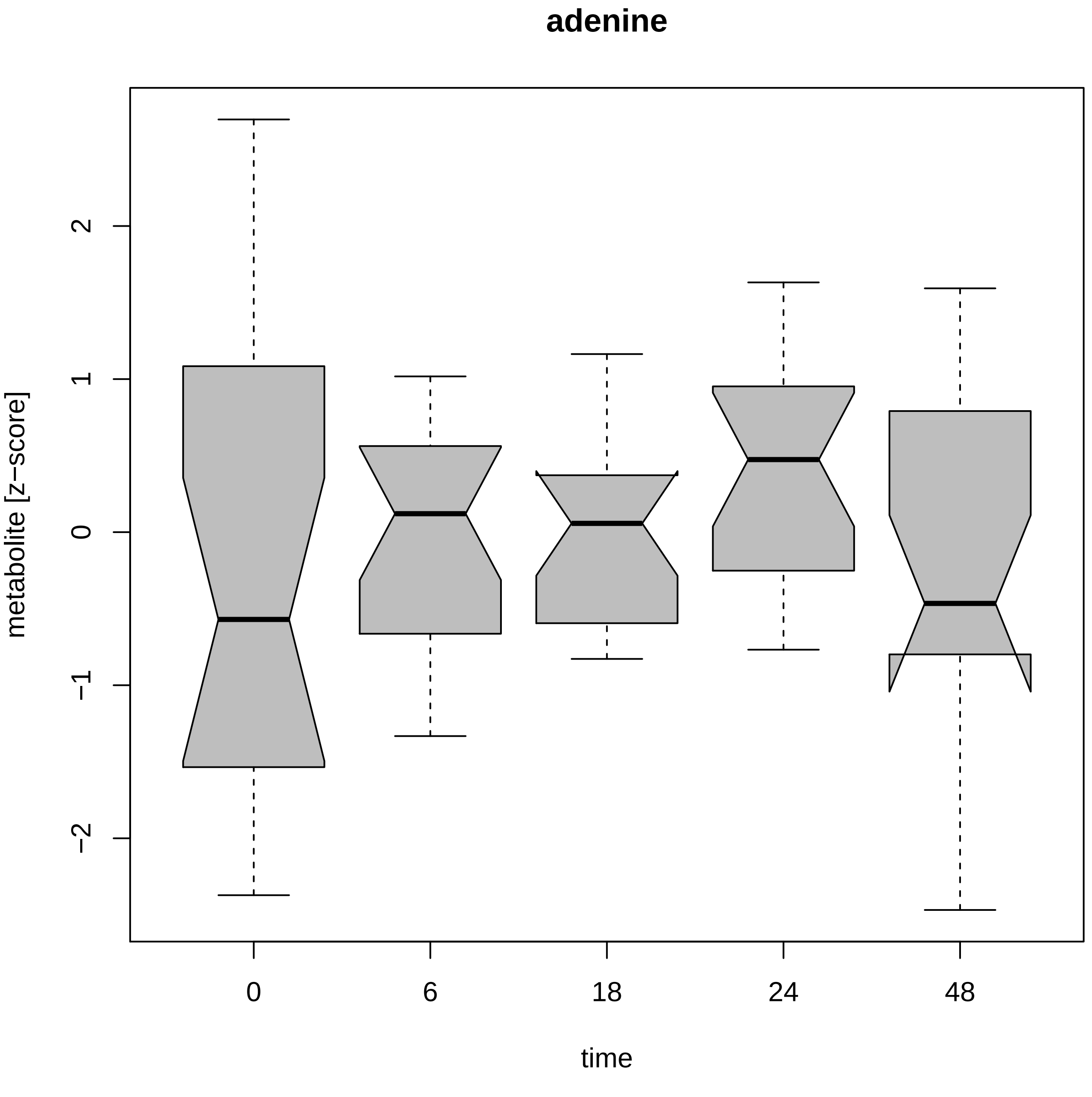

adenine
metabolite [z−score]
2
1
0
−1
−2
0
6
18
24
48
time

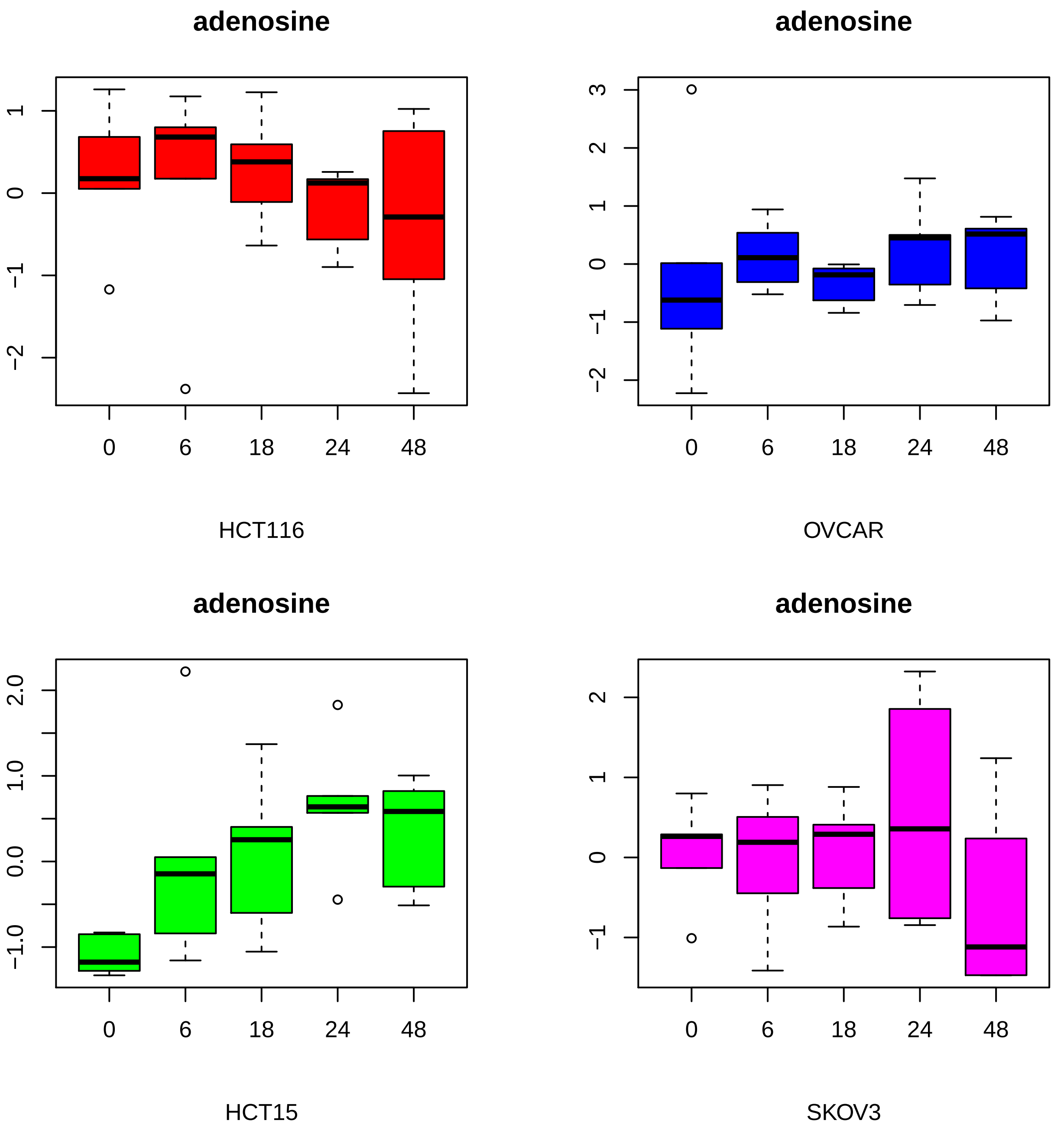
adenosine
HCT116
adenosine
OVCAR
adenosine
HCT15
adenosine
SKOV3
0
6
18
24
48
−2
−1
0
1
2
3
−1.0
0.0
1.0
2.0

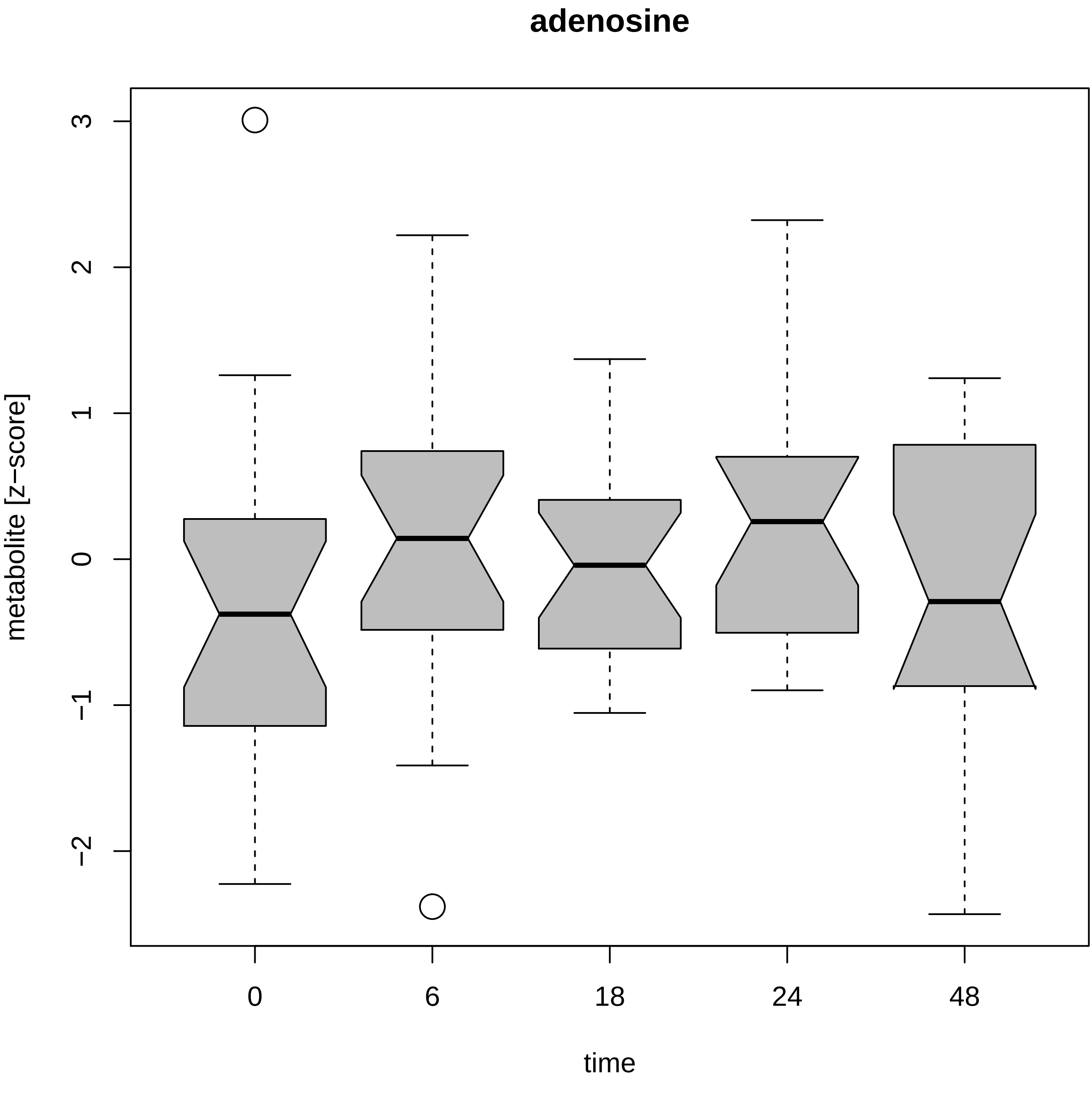
adenosine
3
2
1
0
−1
−2
metabolite [z−score]
0
6
18
24
48
time

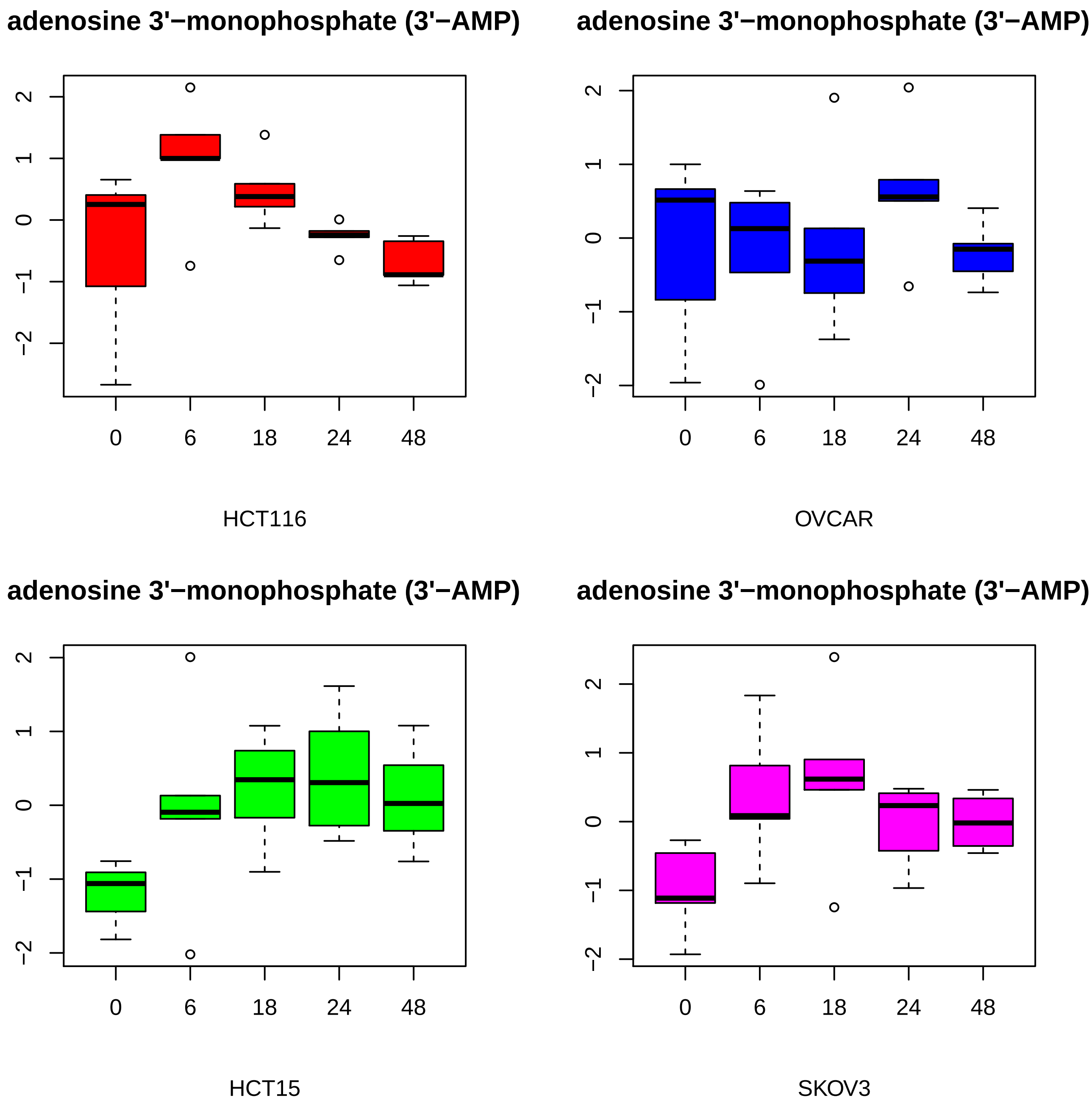

adenosine 3'−monophosphate (3'−AMP)
HCT116
adenosine 3'−monophosphate (3'−AMP)
OVCAR
adenosine 3'−monophosphate (3'−AMP)
HCT15
adenosine 3'−monophosphate (3'−AMP)
SKOV3
0
6
18
24
48

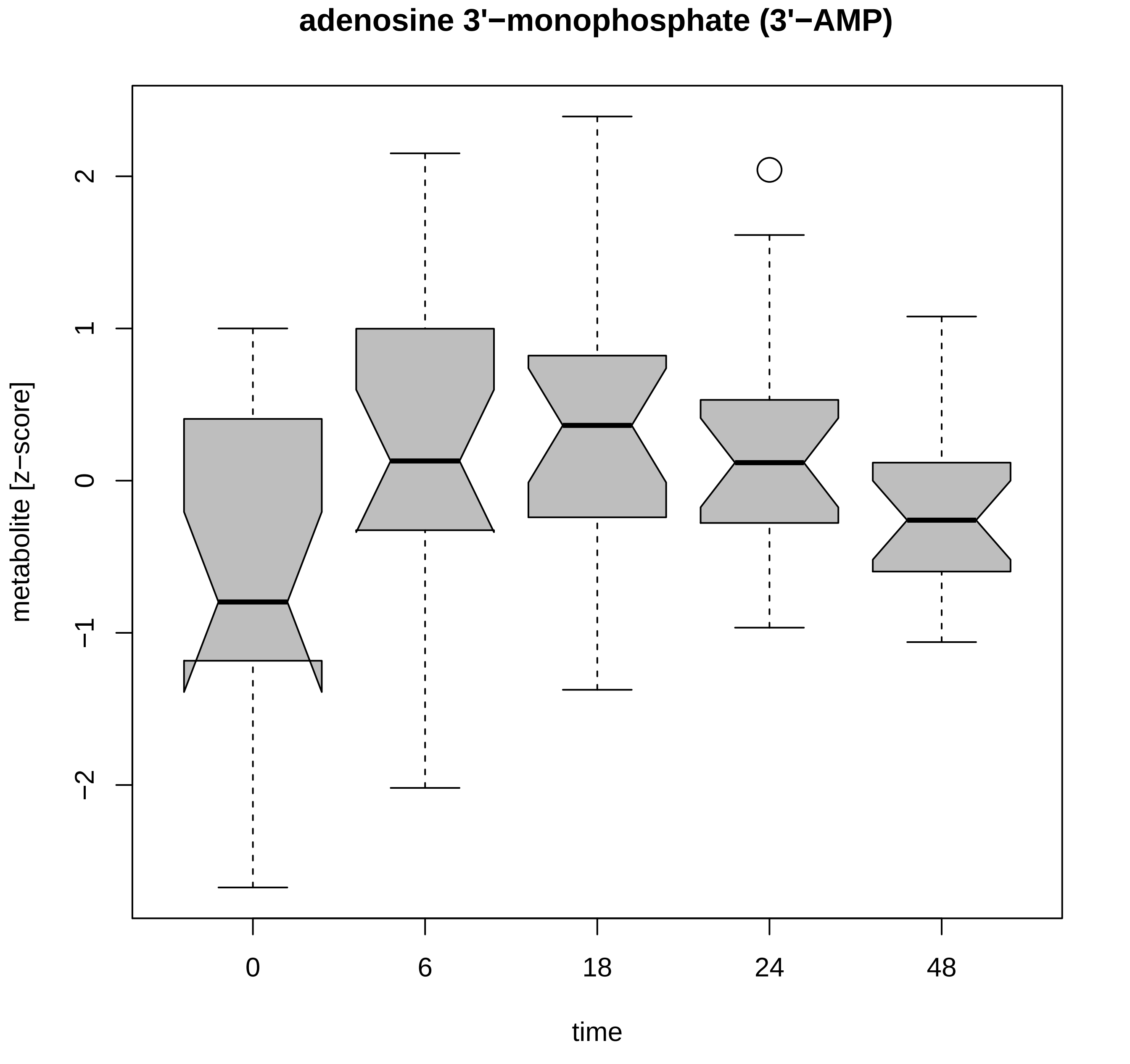
adenosine 3'−monophosphate (3'−AMP)
metabolite [z−score]
−2
−1
0
1
2
0
6
18
24
48
time

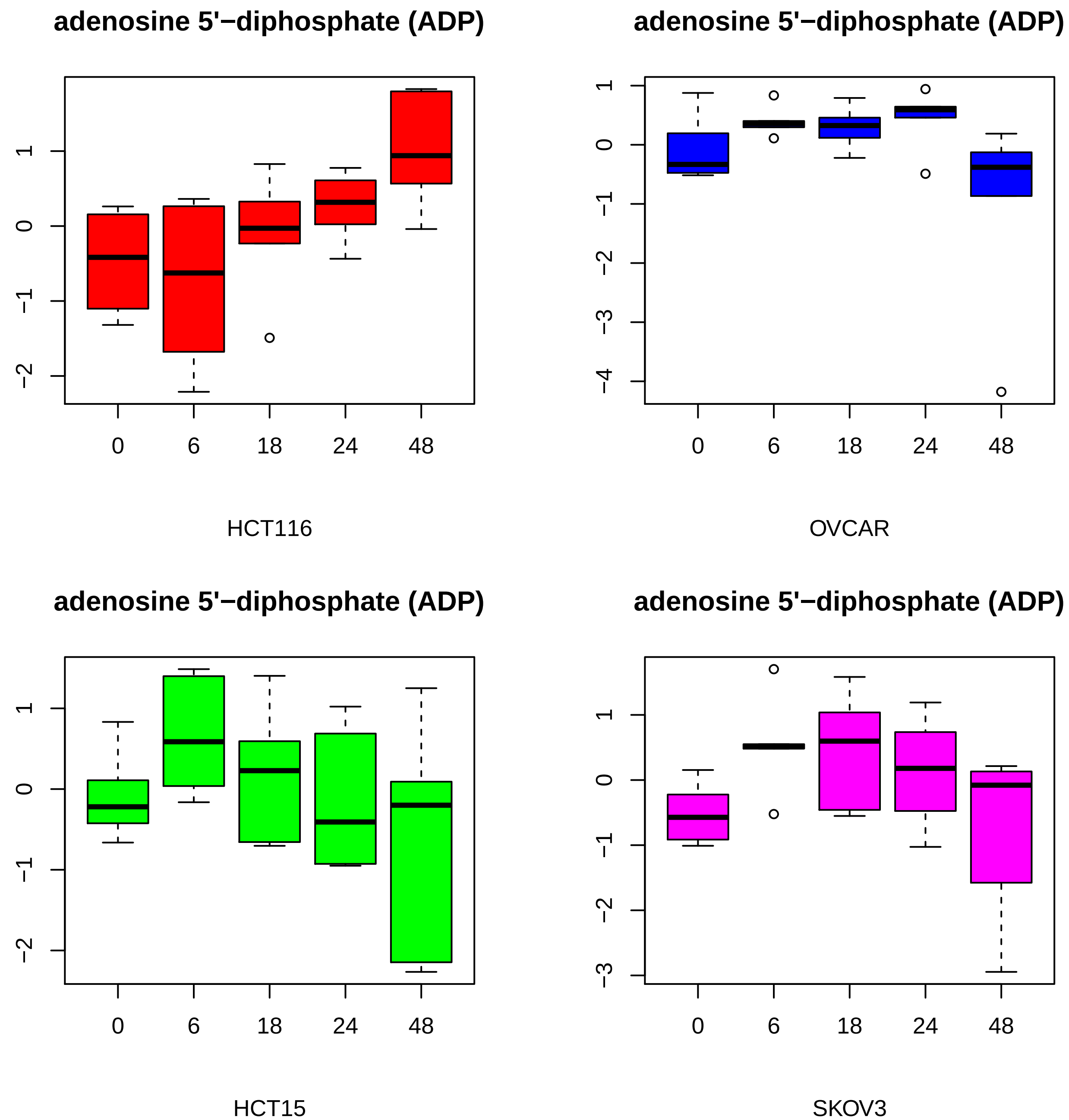
adenosine 5'−diphosphate (ADP)
HCT116
0
6
18
24
48
−2
−1
0
1
adenosine 5'−diphosphate (ADP)
OVCAR
0
6
18
24
48
−4
−3
−2
−1
0
1
adenosine 5'−diphosphate (ADP)
HCT15
0
6
18
24
48
−2
−1
0
1
adenosine 5'−diphosphate (ADP)
SKOV3
0
6
18
24
48
−3
−2
−1
0
1

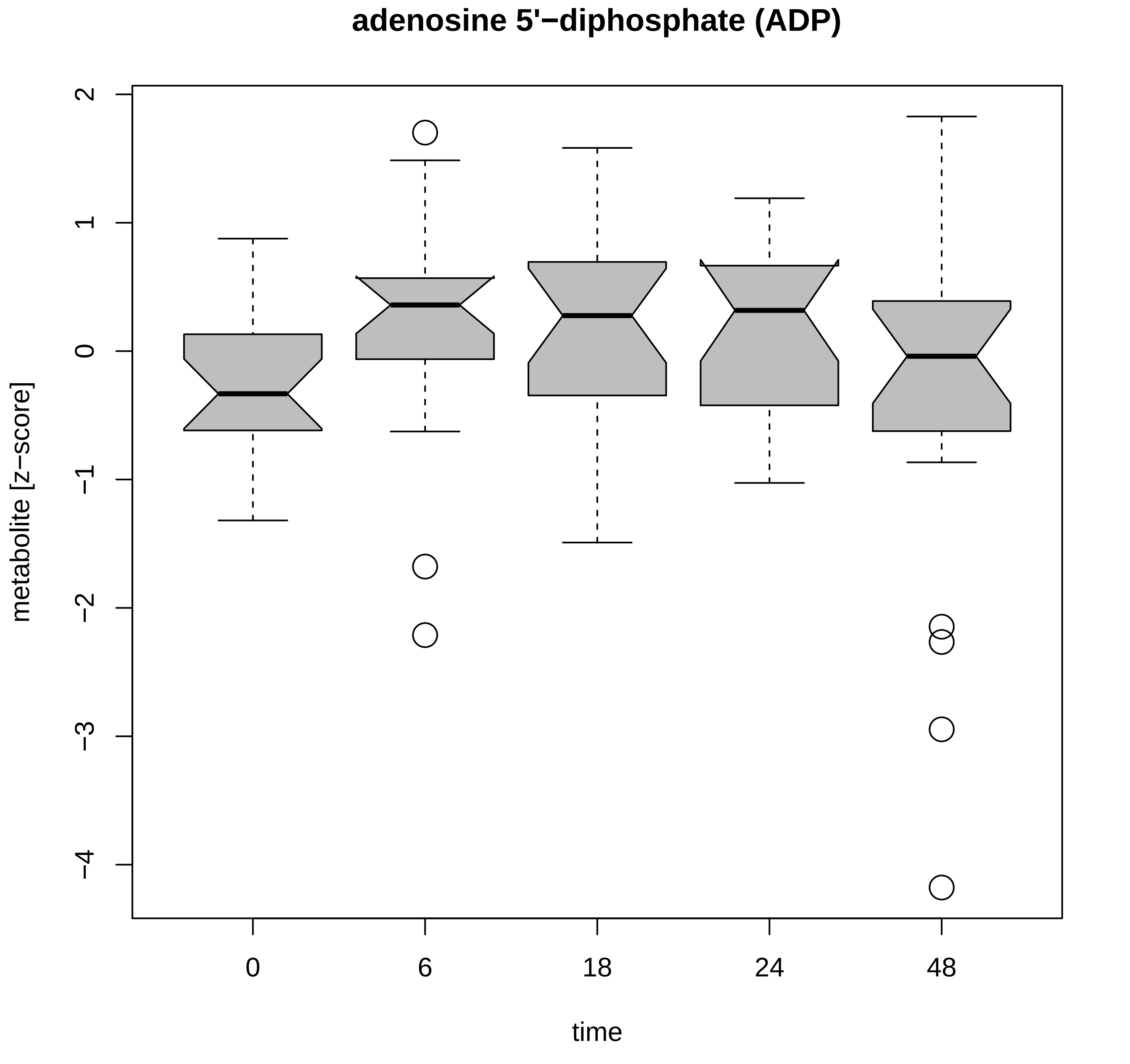

adenosine 5'−diphosphate (ADP)
metabolite [z−score]
2
1
0
−1
−2
−3
−4
0
6
18
24
48
time

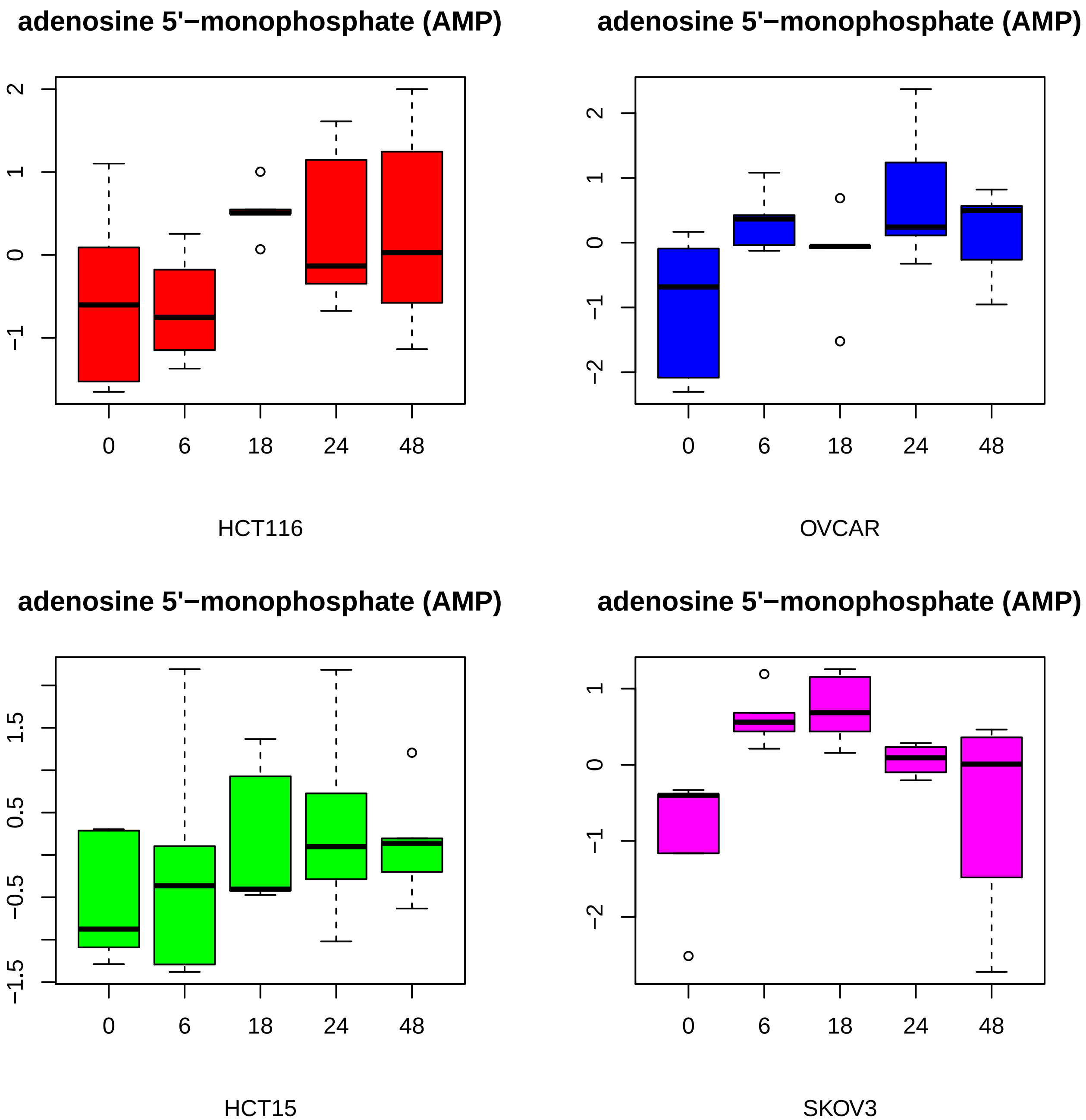

adenosine 5'−monophosphate (AMP)
HCT116
adenosine 5'−monophosphate (AMP)
OVCAR
adenosine 5'−monophosphate (AMP)
HCT15
adenosine 5'−monophosphate (AMP)
SKOV3
0
6
18
24
48

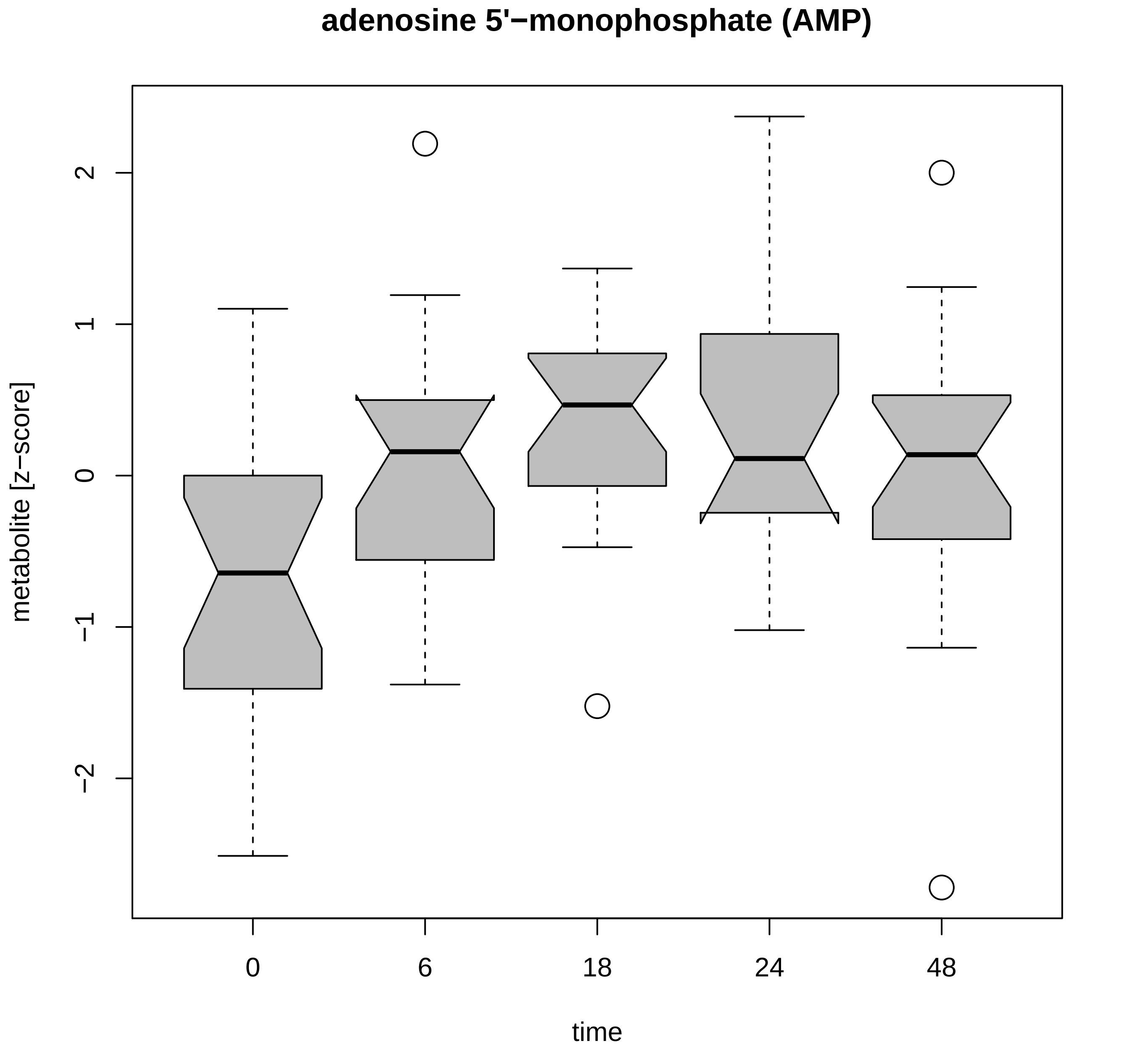

adenosine 5'−monophosphate (AMP)
metabolite [z−score]
2
1
0
−1
−2
0
6
18
24
48
time

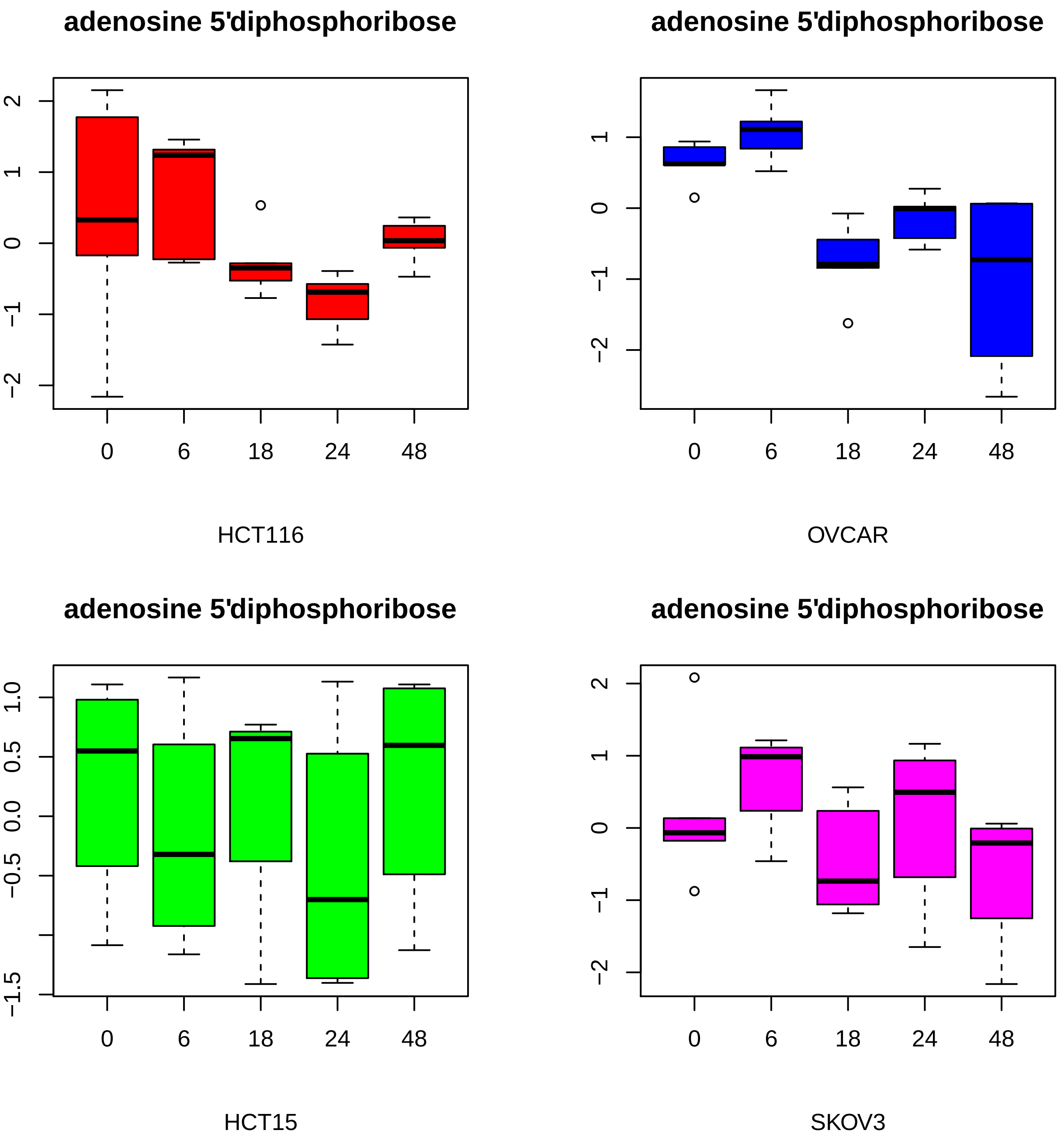
adenosine 5'diphosphoribose
2
1
0
−1
−2
0
6
18
24
48
HCT116
adenosine 5'diphosphoribose
1
0
−1
−2
0
6
18
24
48
OVCAR
adenosine 5'diphosphoribose
1.0
0.5
0.0
−0.5
−1.5
0
6
18
24
48
HCT15
adenosine 5'diphosphoribose
2
1
0
−1
−2
0
6
18
24
48
SKOV3

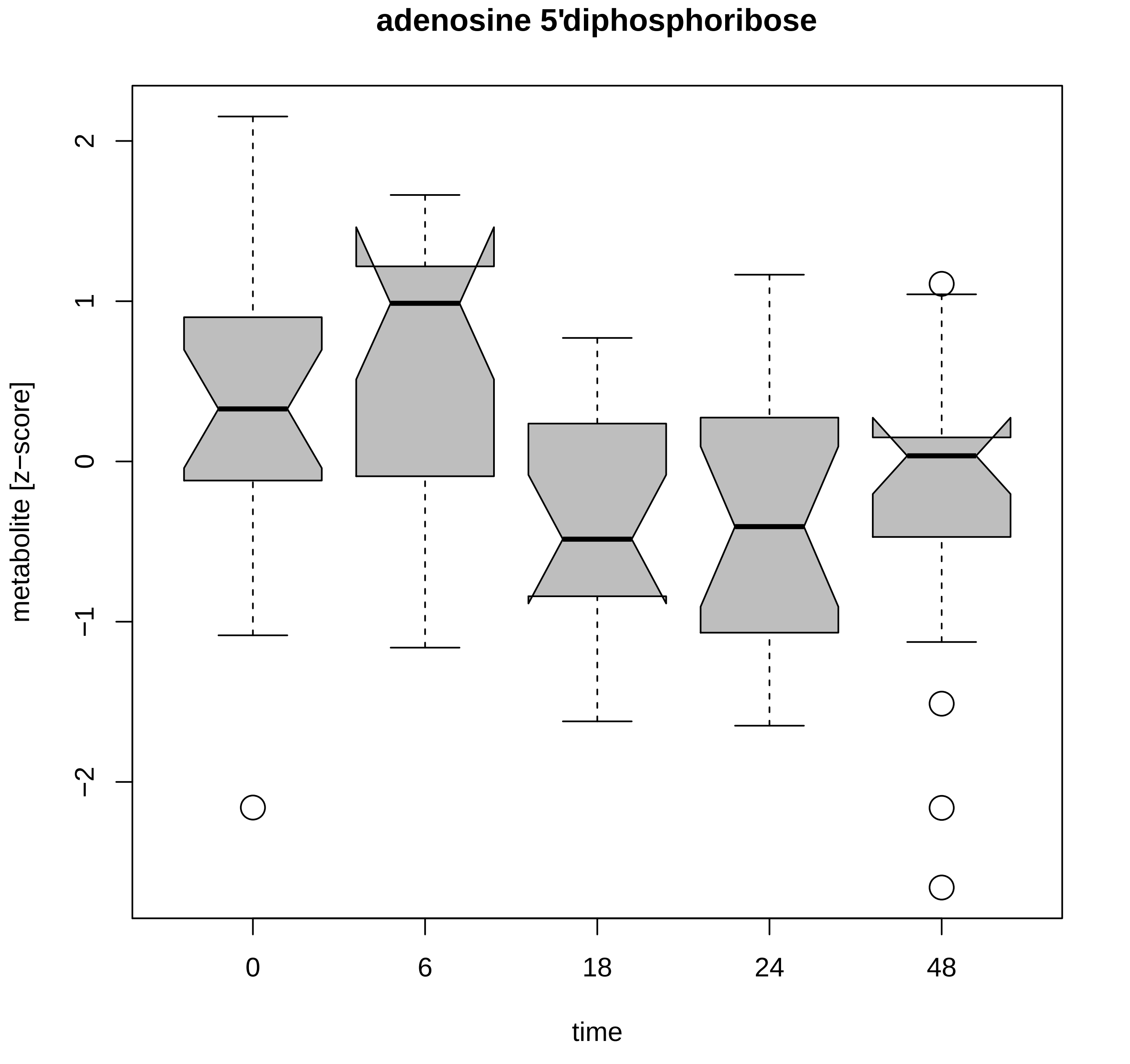

adenosine 5'diphosphoribose
metabolite [z−score]
2
1
0
−1
−2
0
6
18
24
48
time

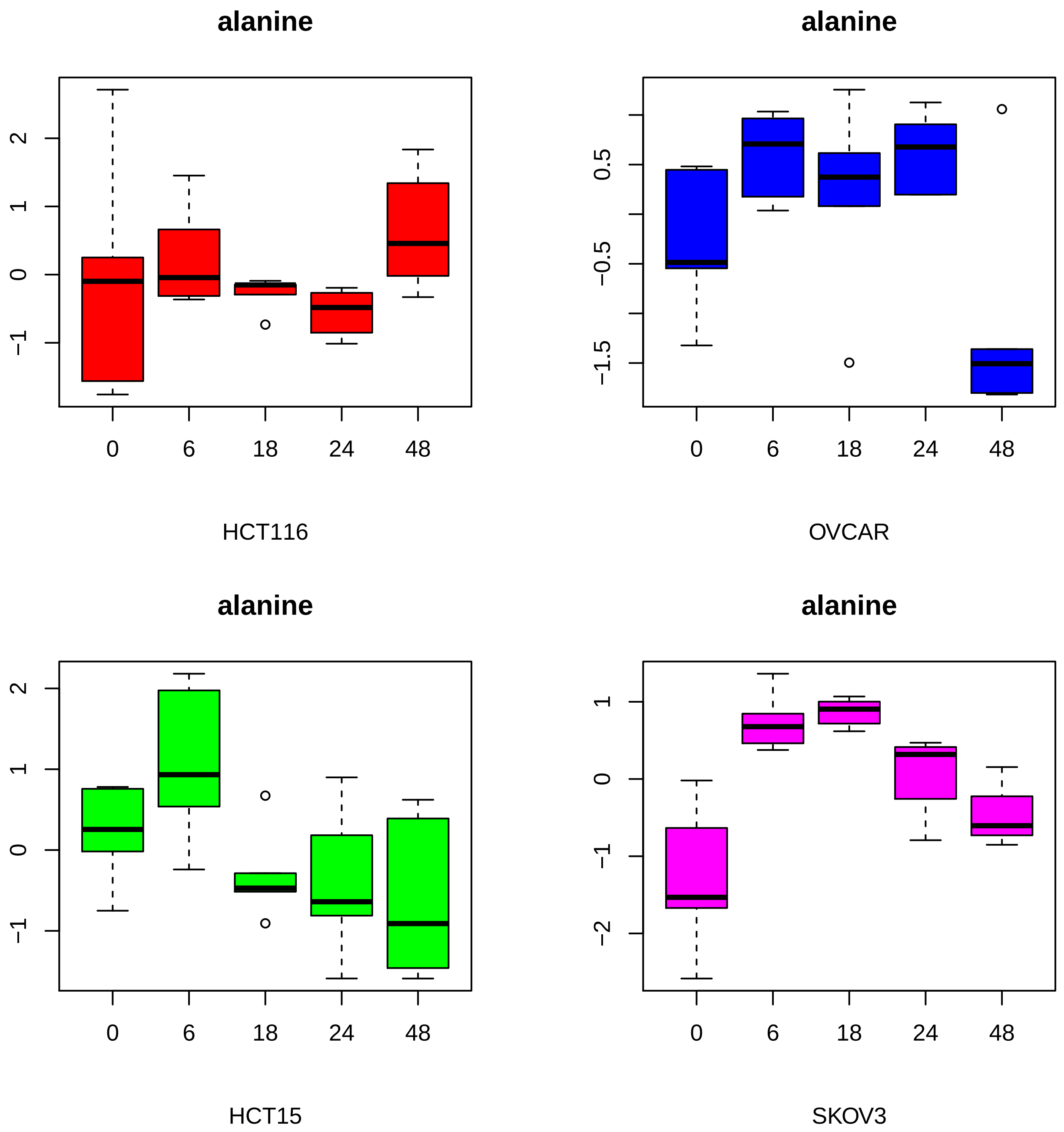
alanine
2
1
0
−1
0
6
18
24
48
HCT116
alanine
0.5
−0.5
−1.5
0
6
18
24
48
OVCAR
alanine
2
1
0
−1
0
6
18
24
48
HCT15
alanine
1
0
−1
−2
0
6
18
24
48
SKOV3

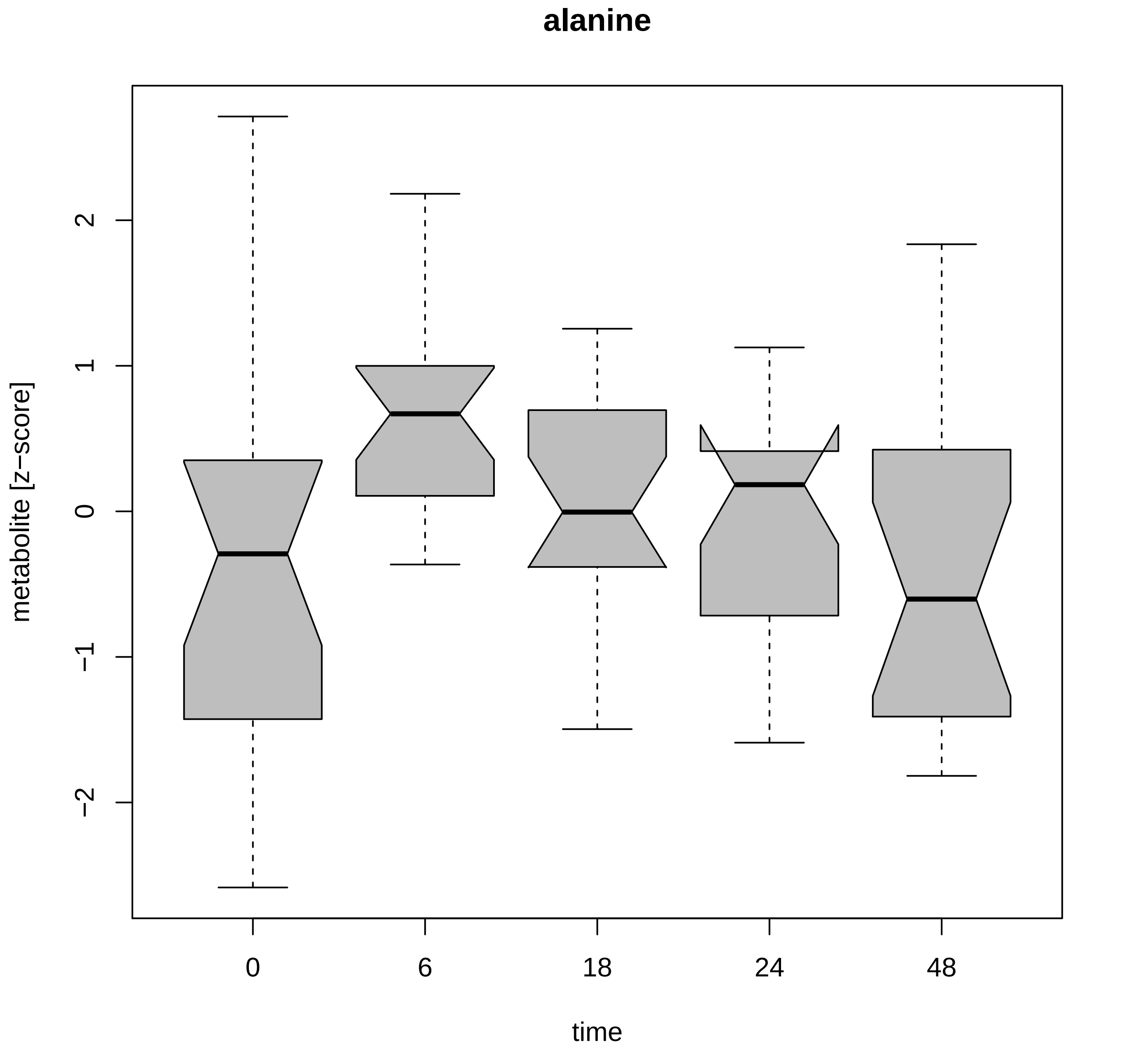
alanine
metabolite [z−score]
2
1
0
−1
−2
0
6
18
24
48
time

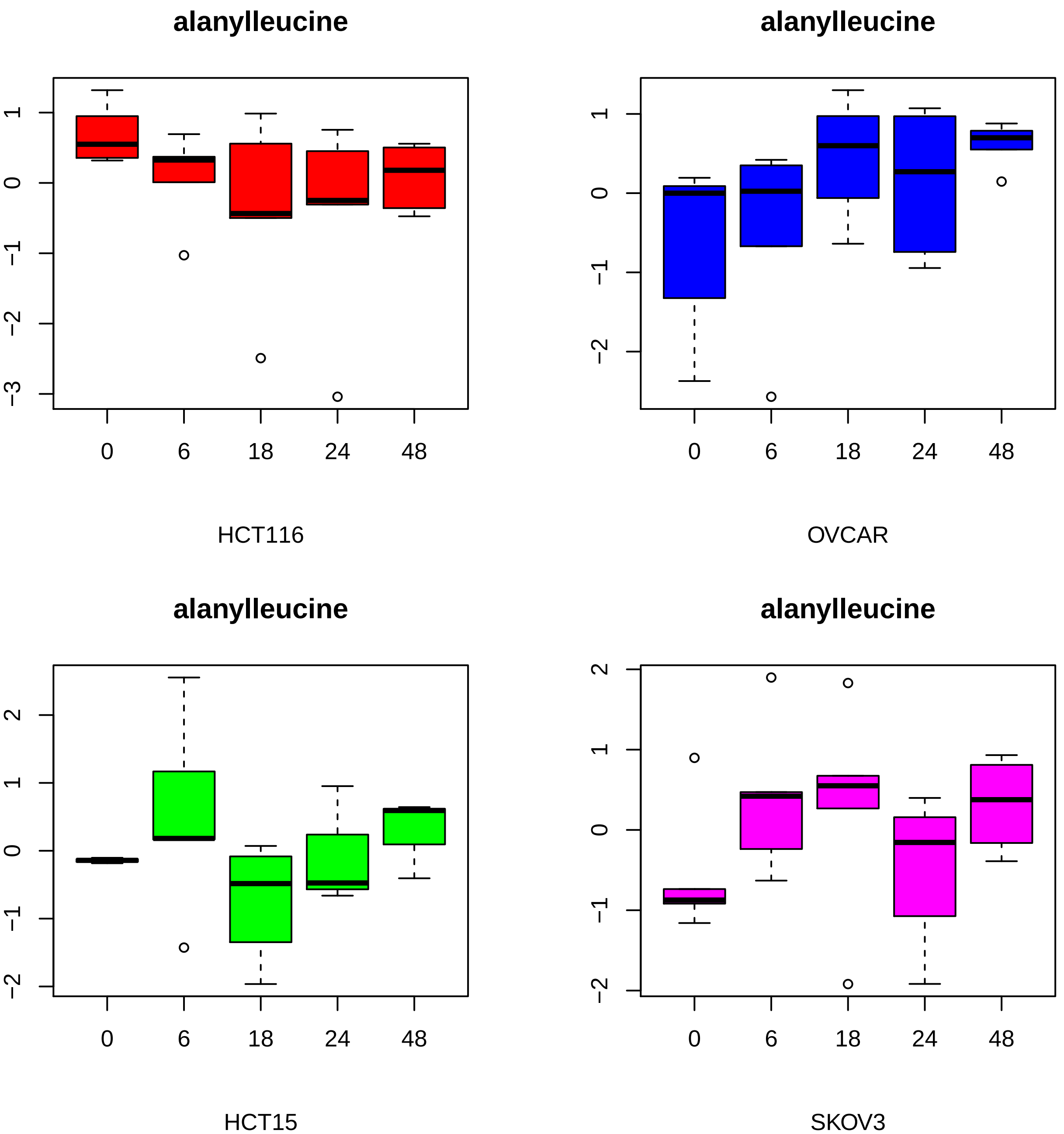
alanylleucine
1
0
−1
−2
−3
0
6
18
24
48
HCT116
alanylleucine
1
0
−1
−2
0
6
18
24
48
OVCAR
alanylleucine
2
1
0
−1
−2
0
6
18
24
48
HCT15
alanylleucine
2
1
0
−1
−2
0
6
18
24
48
SKOV3

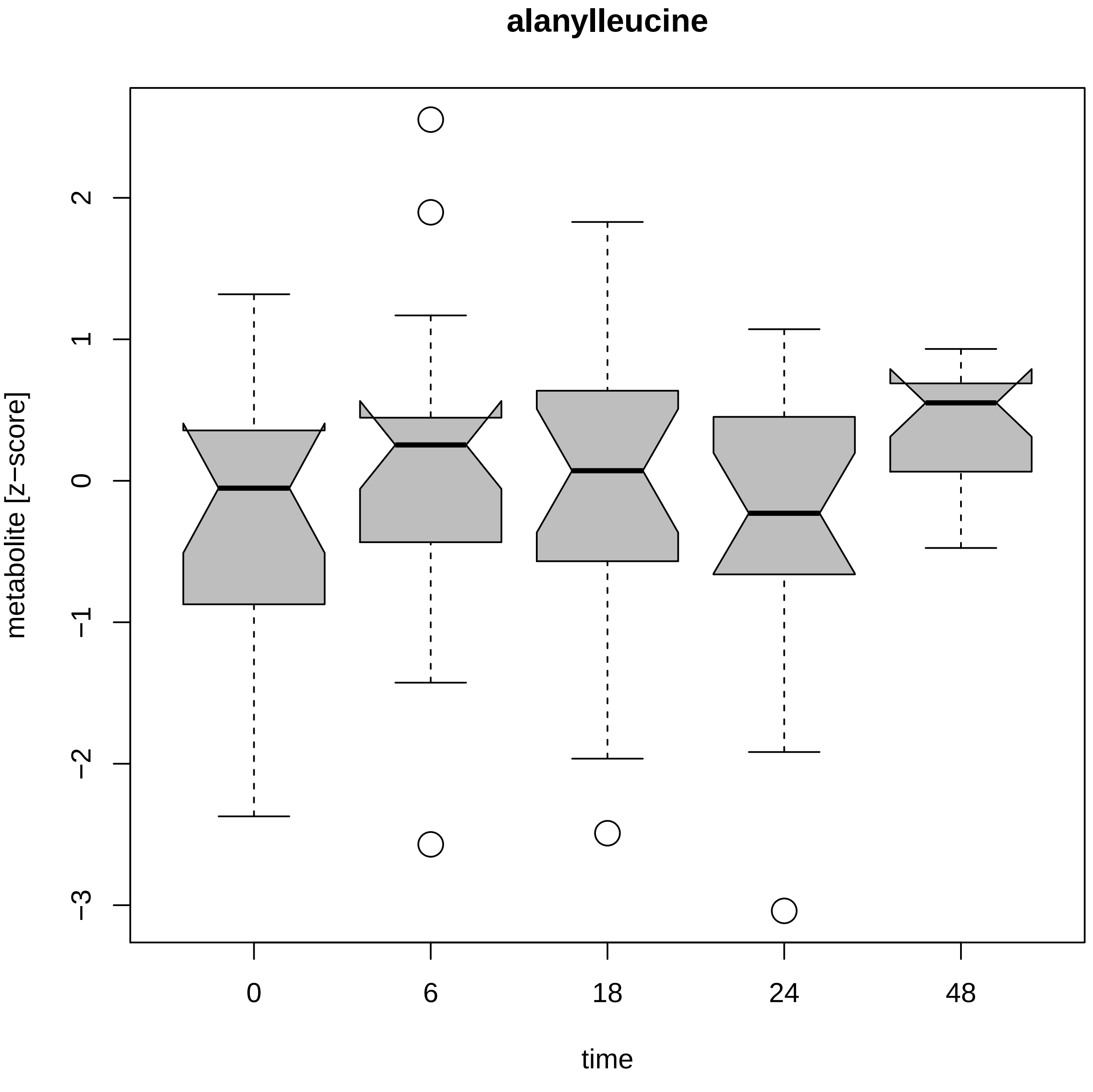
alanylleucine
metabolite [z−score]
time
0
6
18
24
48
−3
−2
−1
0
1
2

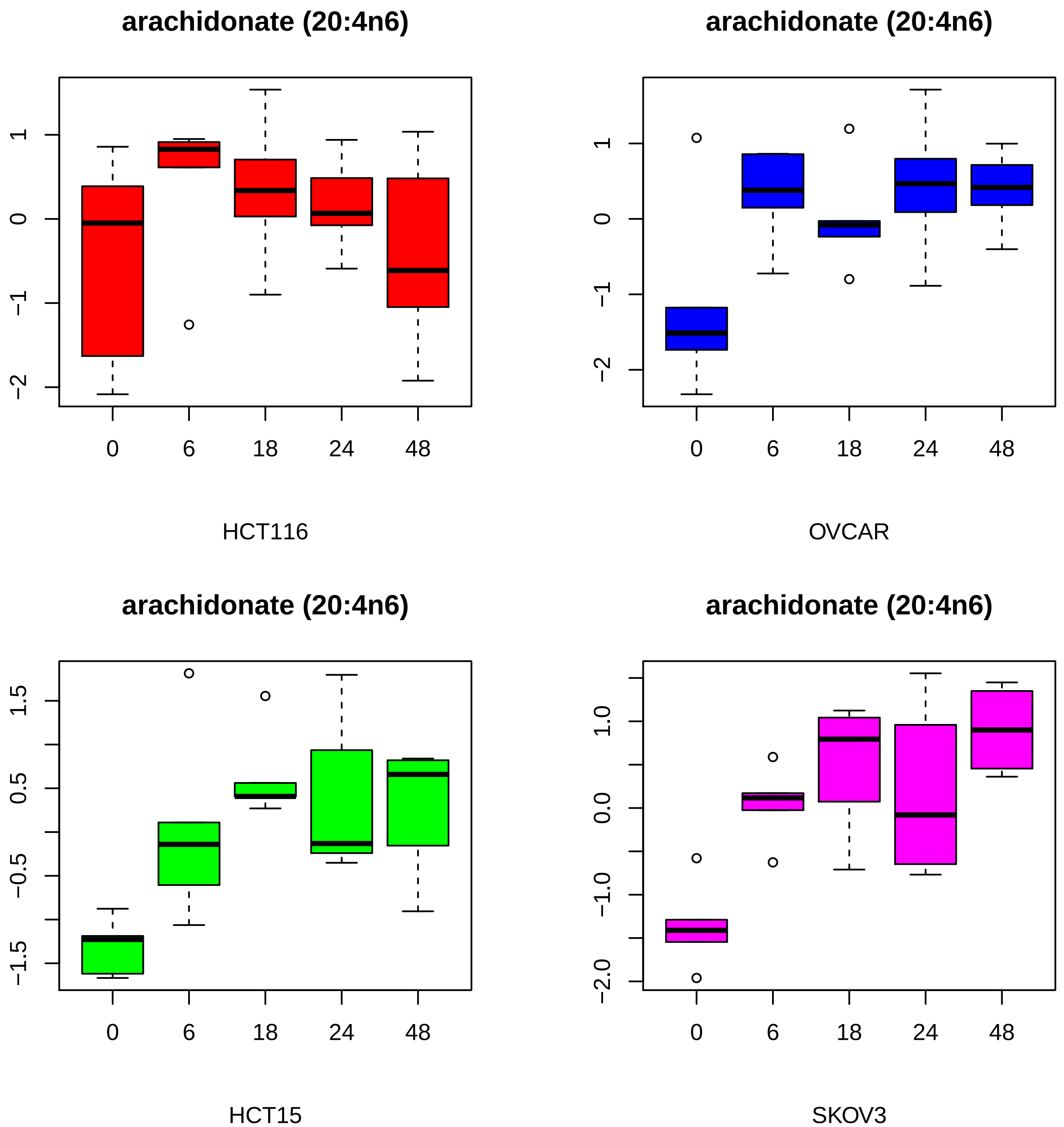

arachidonate (20:4n6)
1
0
−1
−2
0
6
18
24
48
HCT116
arachidonate (20:4n6)
1
0
−1
−2
0
6
18
24
48
OVCAR
arachidonate (20:4n6)
1.5
0.5
−0.5
−1.5
0
6
18
24
48
HCT15
arachidonate (20:4n6)
1.0
0.0
−1.0
−2.0
0
6
18
24
48
SKOV3

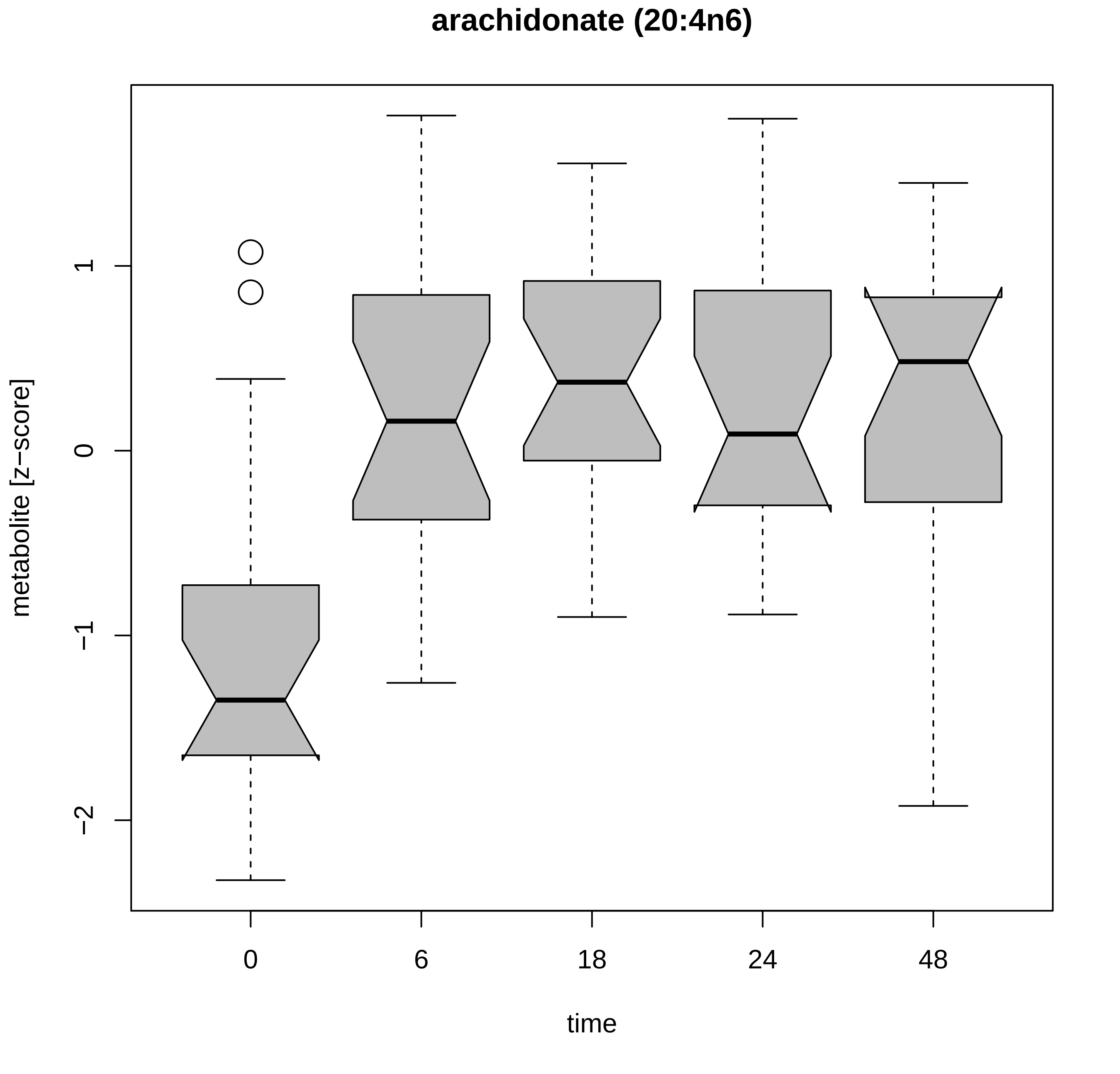
arachidonate (20:4n6)
metabolite [z−score]
−2
−1
0
1
0
6
18
24
48
time

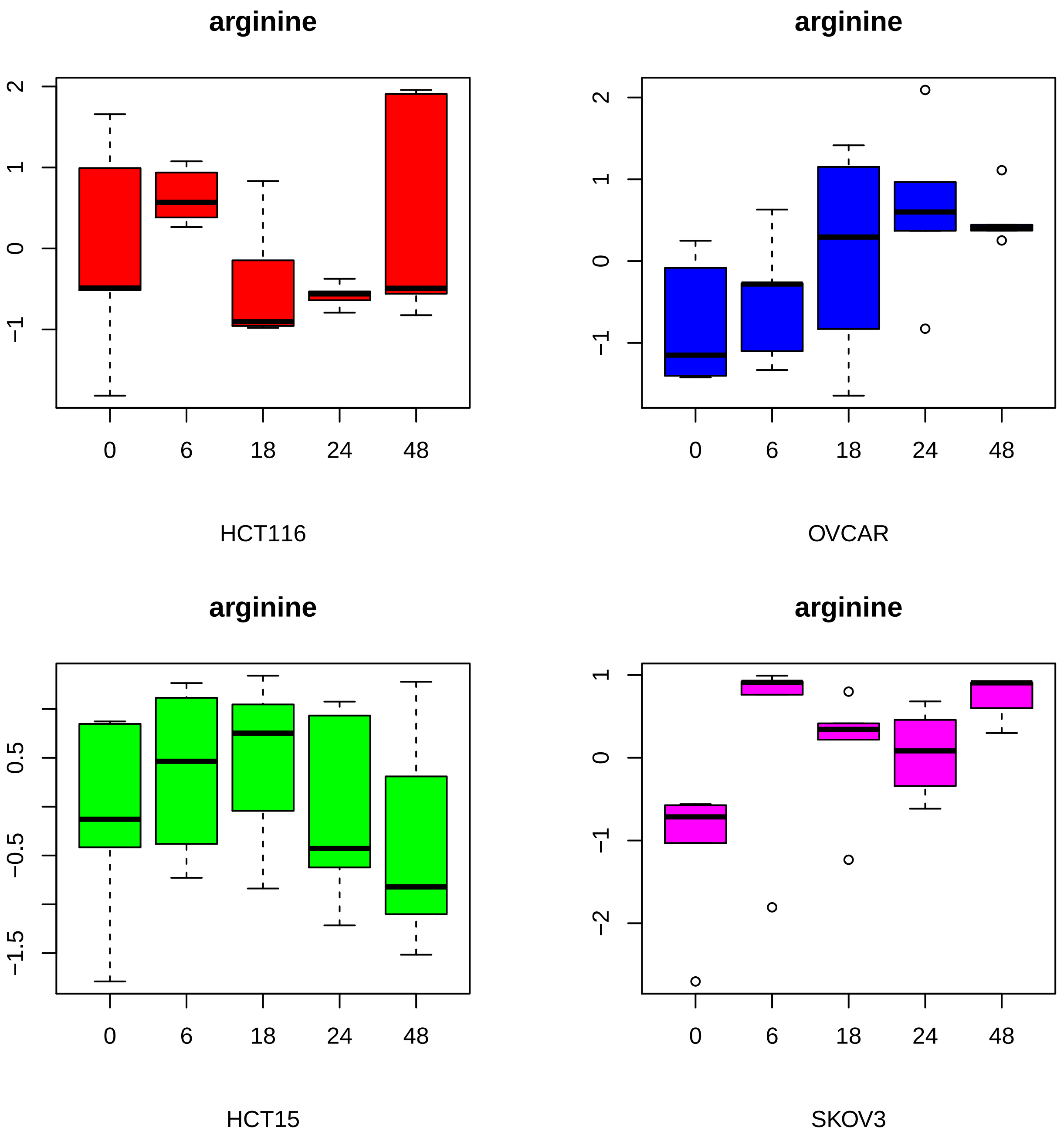

arginine
HCT116
0
6
18
24
48
2
1
0
−1
arginine
OVCAR
2
1
0
−1
arginine
HCT15
0.5
−0.5
−1.5
arginine
SKOV3
1
0
−1
−2

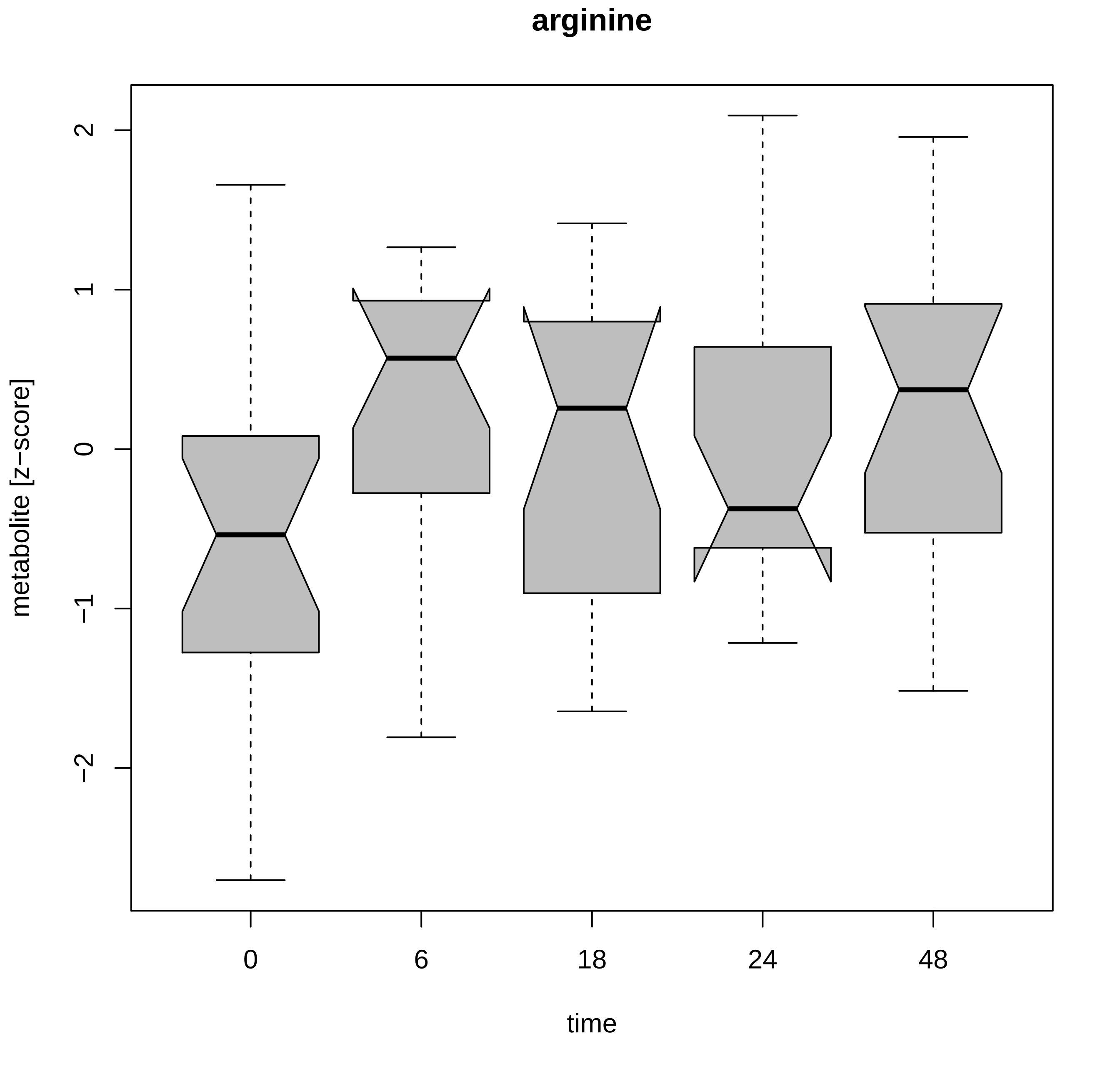
arginine
metabolite [z−score]
2
1
0
−1
−2
0
6
18
24
48
time

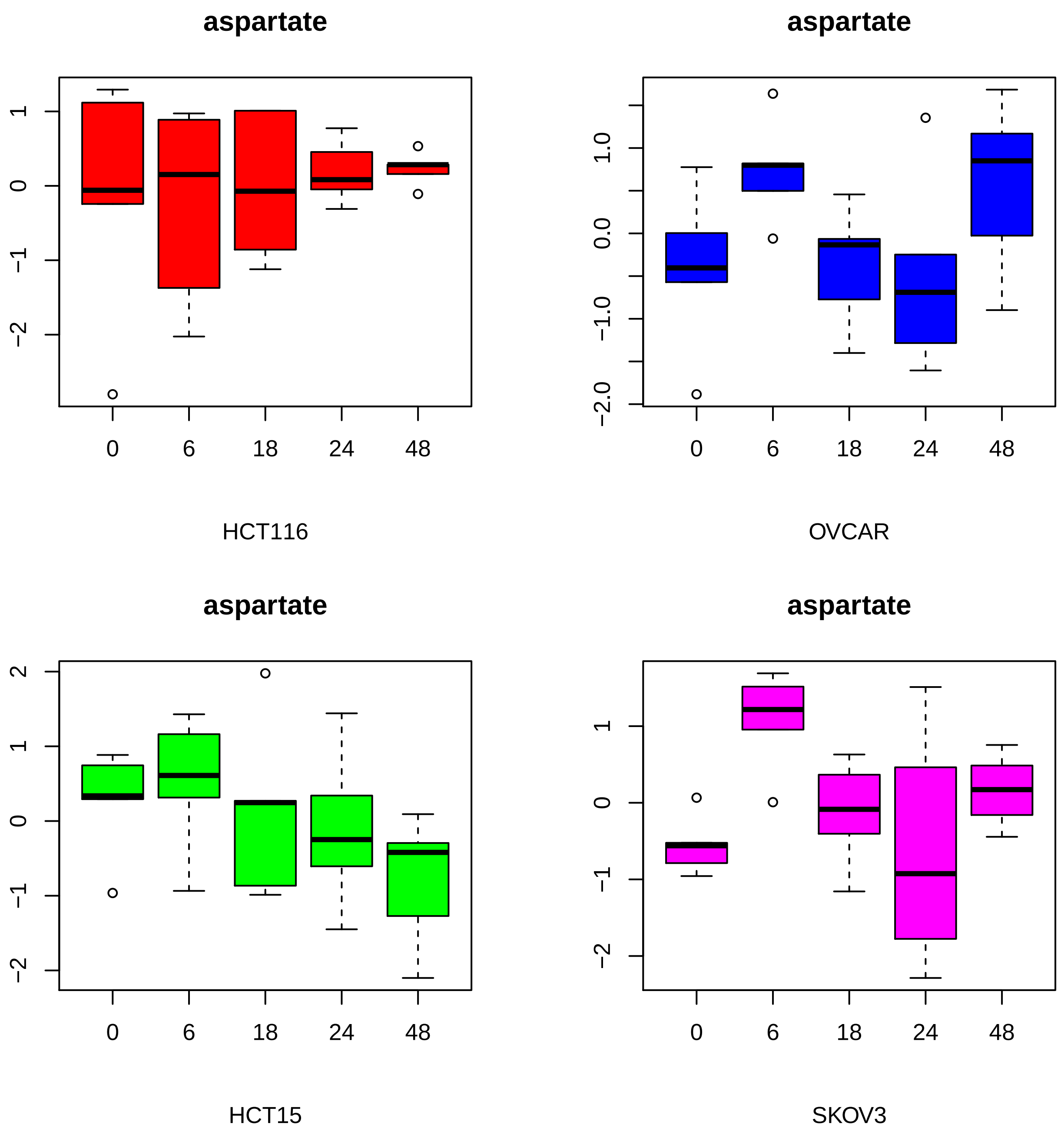
aspartate
1
0
−1
−2
0
6
18
24
48
HCT116
aspartate
1.0
0.0
−1.0
−2.0
0
6
18
24
48
OVCAR
aspartate
2
1
0
−1
−2
0
6
18
24
48
HCT15
aspartate
1
0
−1
−2
0
6
18
24
48
SKOV3

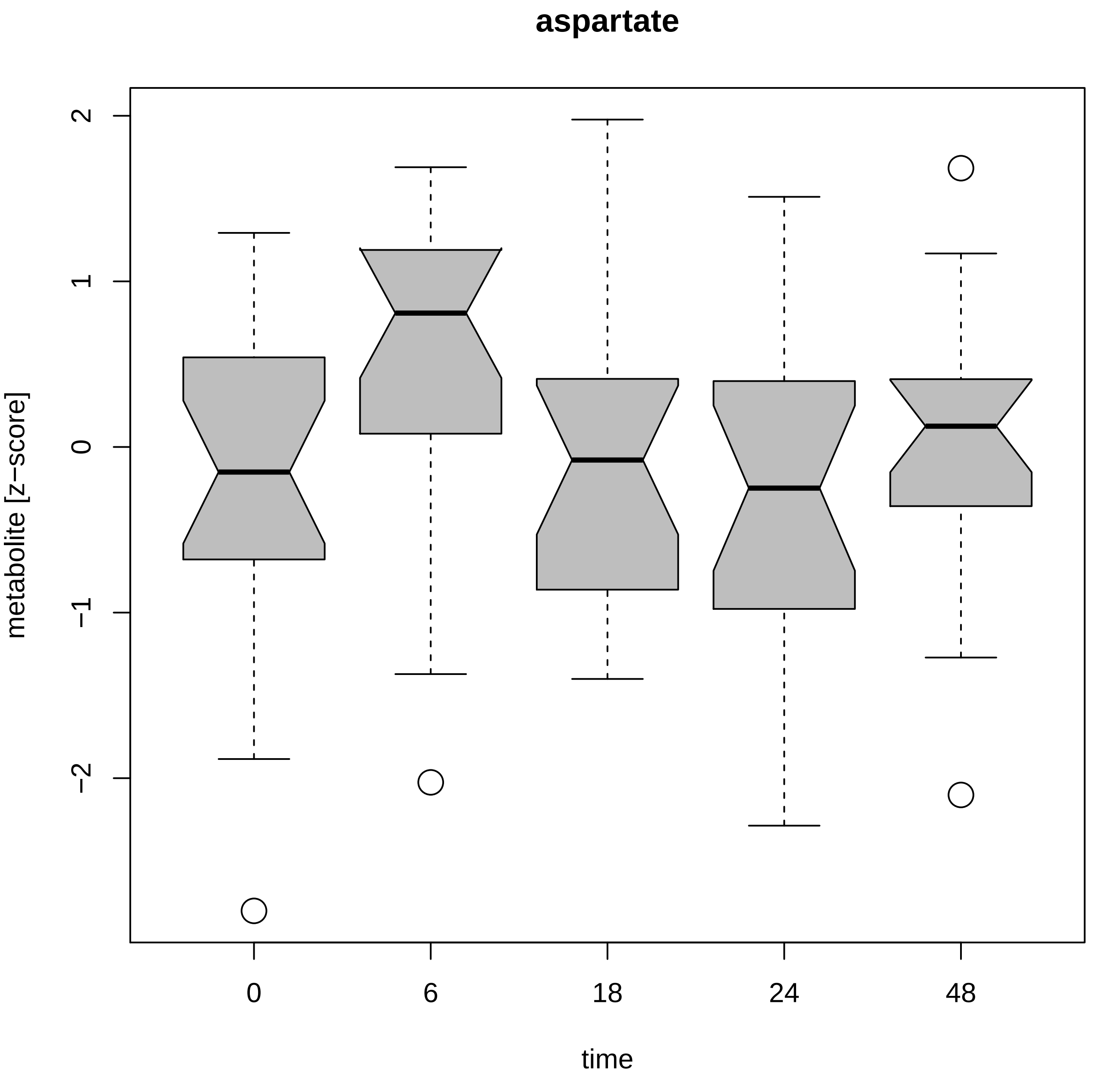

aspartate
metabolite [z−score]
2
1
0
−1
−2
0
6
18
24
48
time

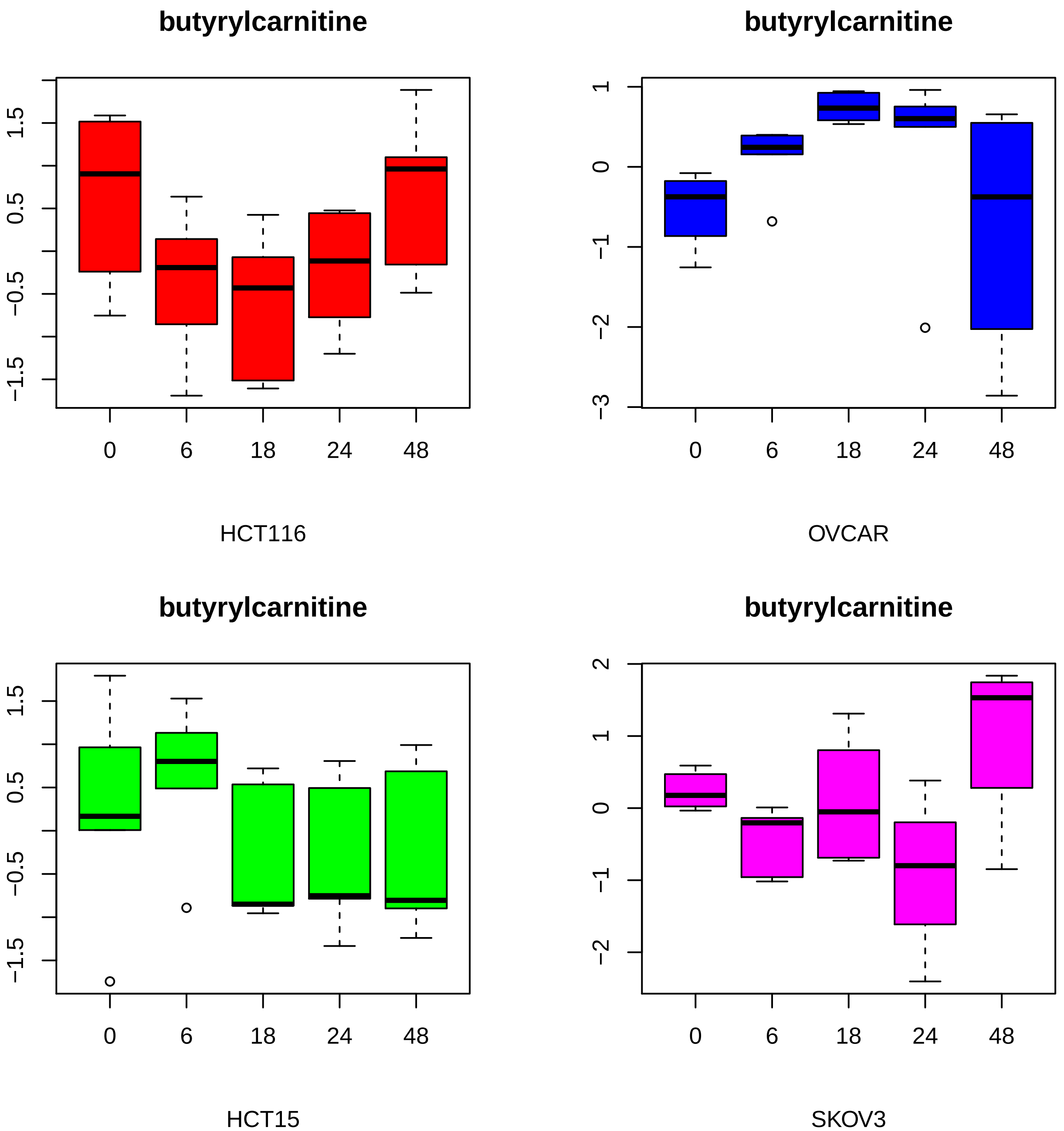
butyrylcarnitine
1.5
0.5
−0.5
−1.5
0
6
18
24
48
HCT116
butyrylcarnitine
1
0
−1
−2
−3
0
6
18
24
48
OVCAR
butyrylcarnitine
1.5
0.5
−0.5
−1.5
0
6
18
24
48
HCT15
butyrylcarnitine
2
1
0
−1
−2
0
6
18
24
48
SKOV3

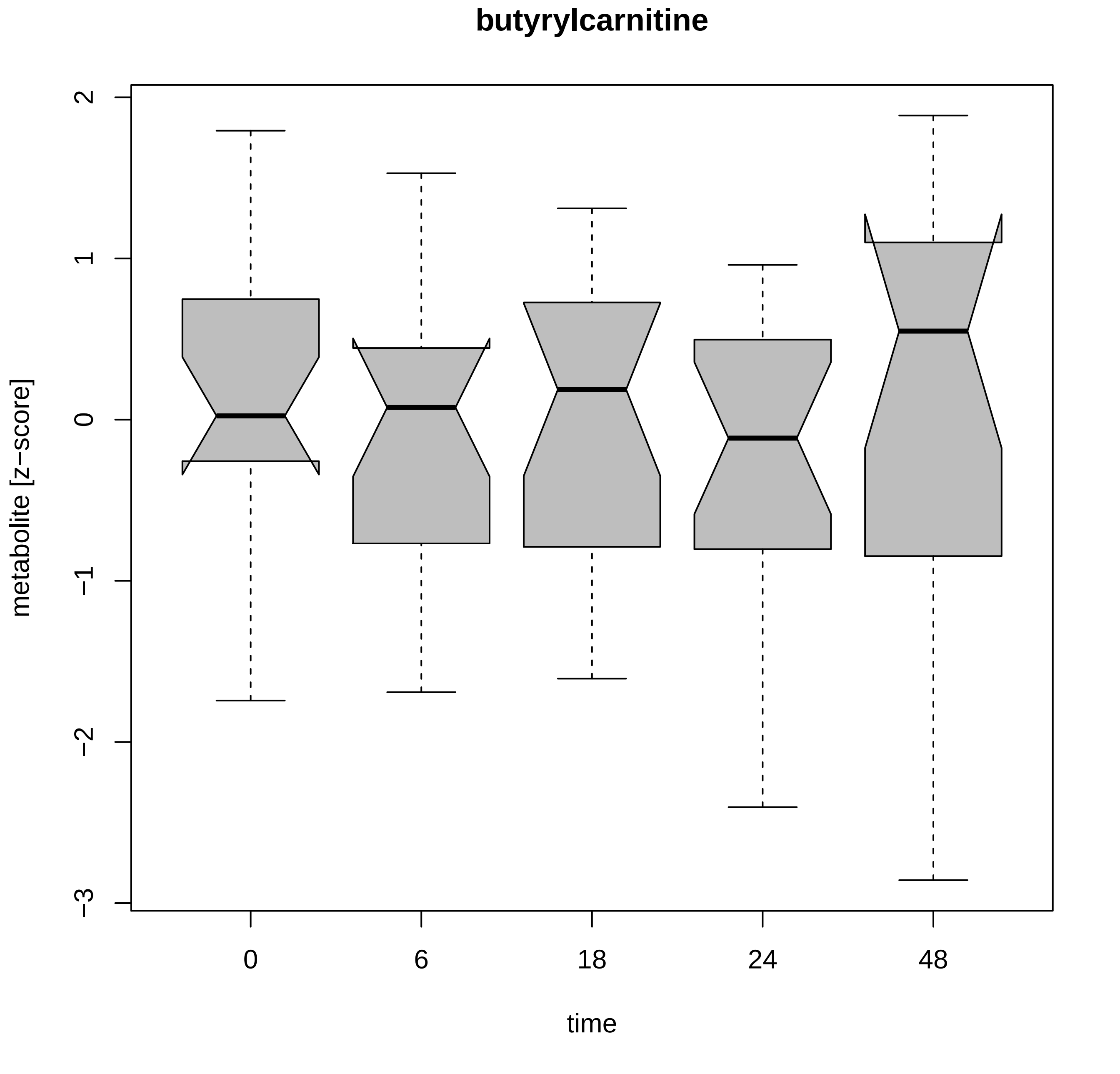

butyrylcarnitine
metabolite [z−score]
2
1
0
−1
−2
−3
0
6
18
24
48
time

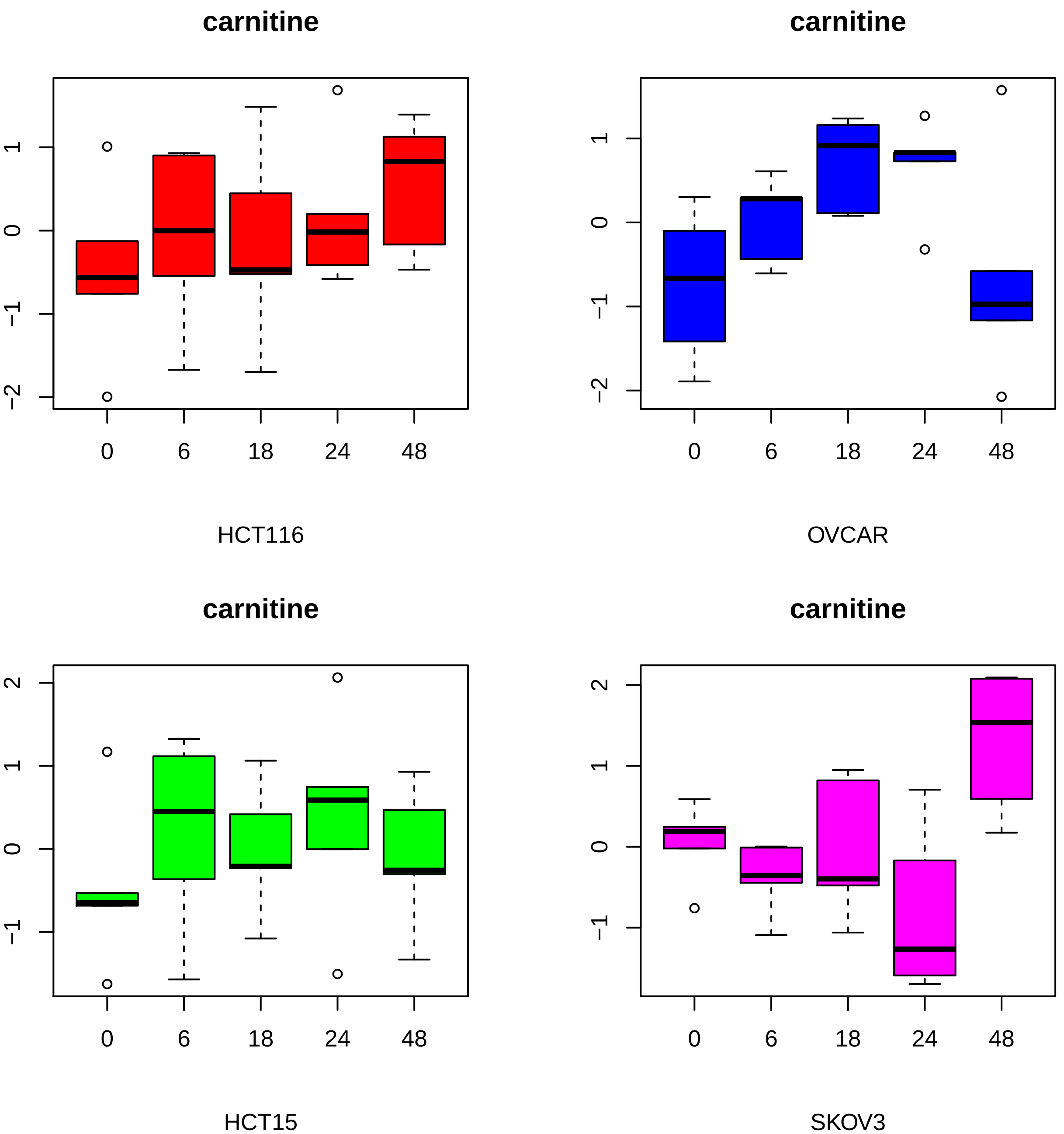
carnitine
HCT116
carnitine
OVCAR
carnitine
HCT15
carnitine
SKOV3
0
6
18
24
48

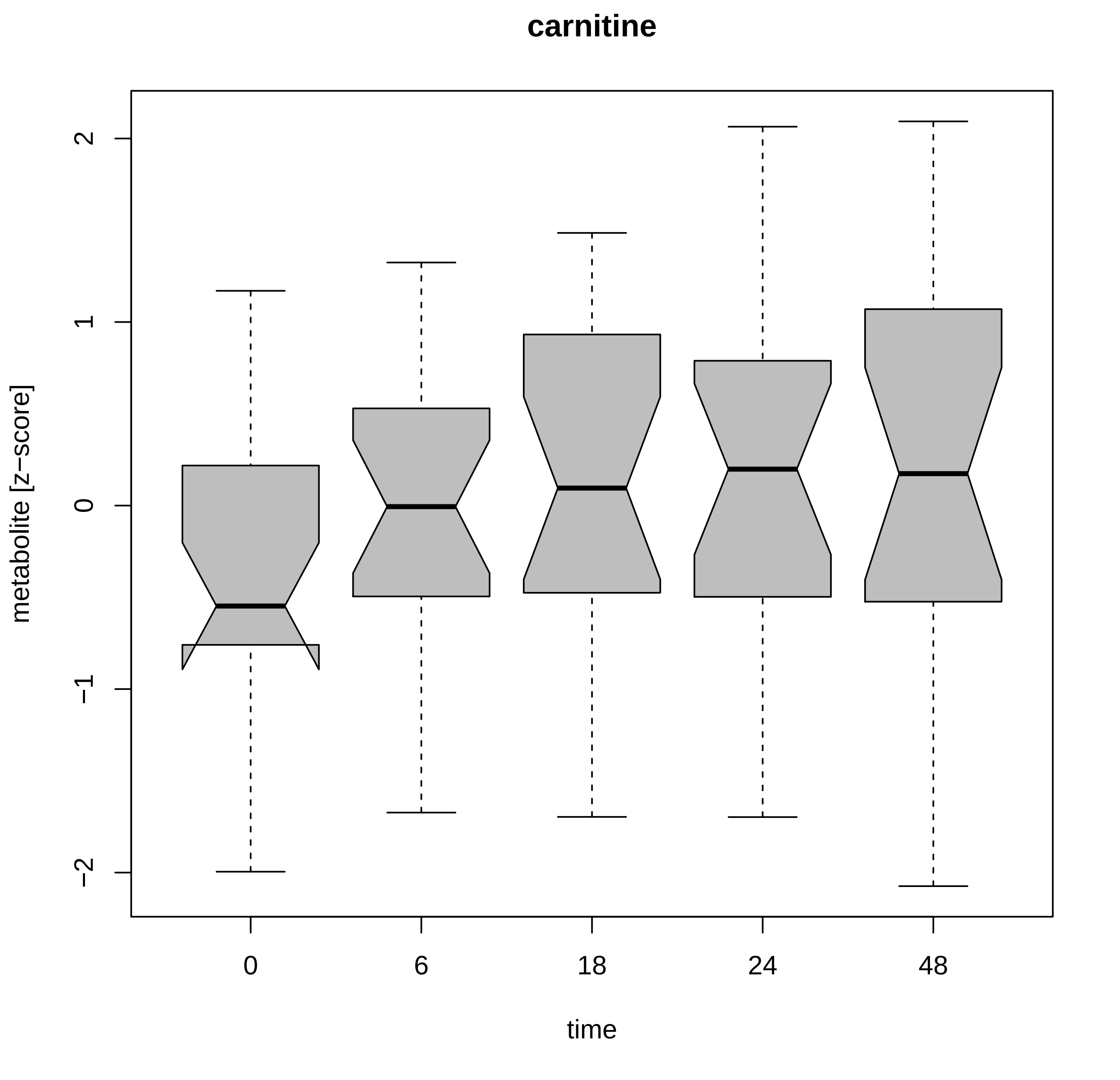

carnitine
metabolite [z−score]
2
1
0
−1
−2
0
6
18
24
48
time

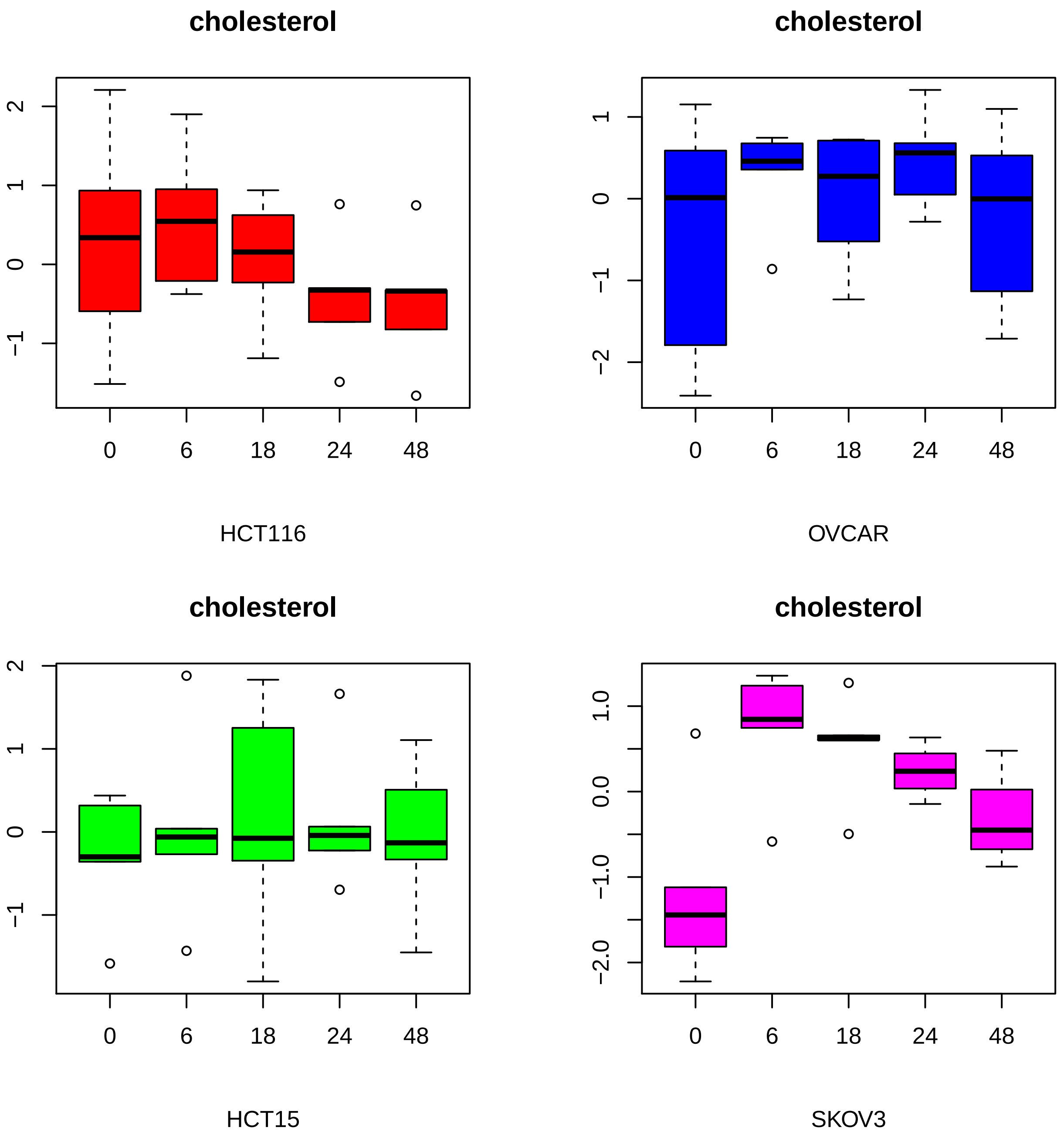

cholesterol
2
1
0
−1
0
6
18
24
48
HCT116
cholesterol
1
0
−1
−2
0
6
18
24
48
OVCAR
cholesterol
2
1
0
−1
0
6
18
24
48
HCT15
cholesterol
1.0
0.0
−1.0
−2.0
0
6
18
24
48
SKOV3

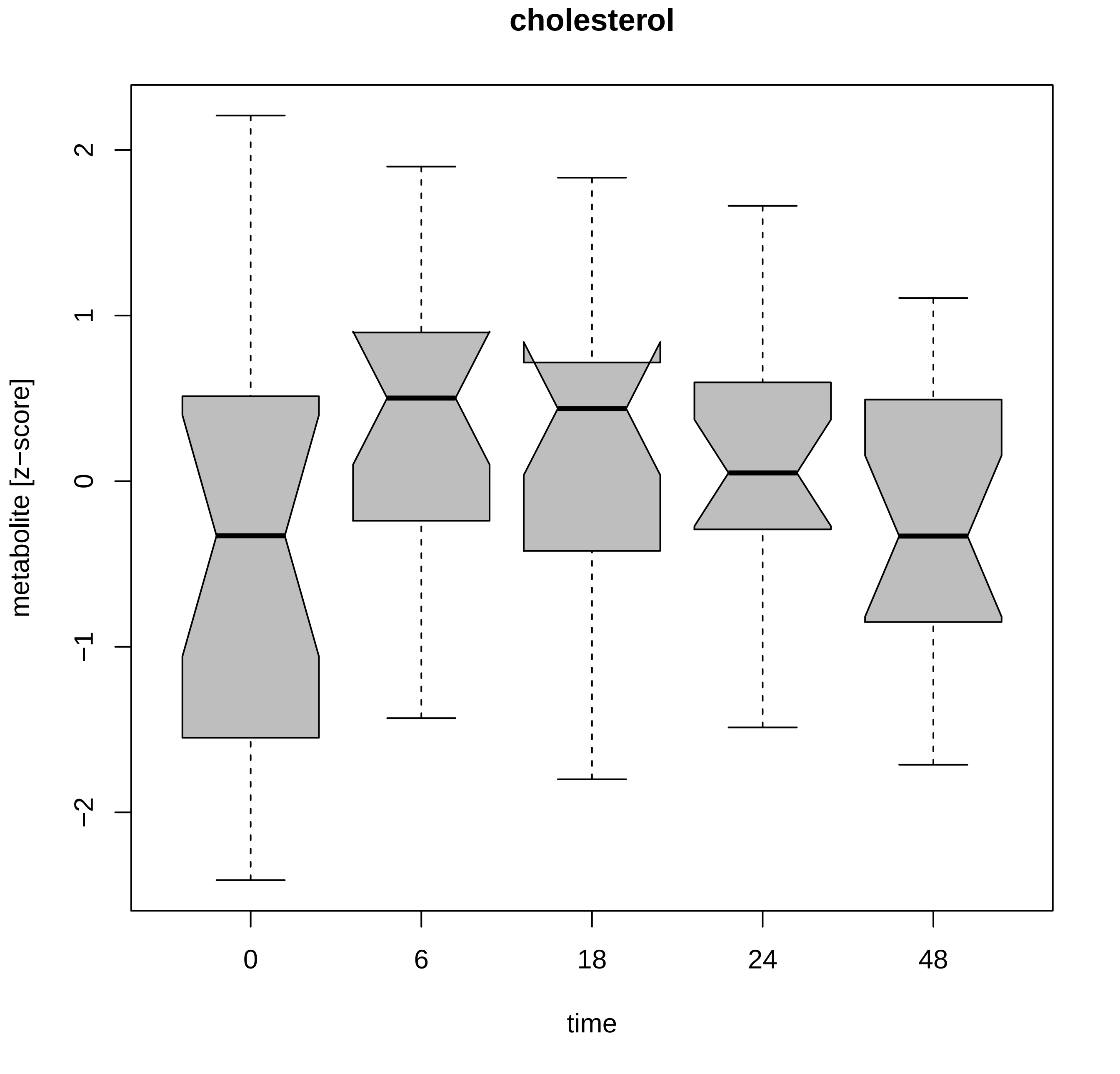

cholesterol
2
1
0
−1
−2
metabolite [z−score]
0
6
18
24
48
time

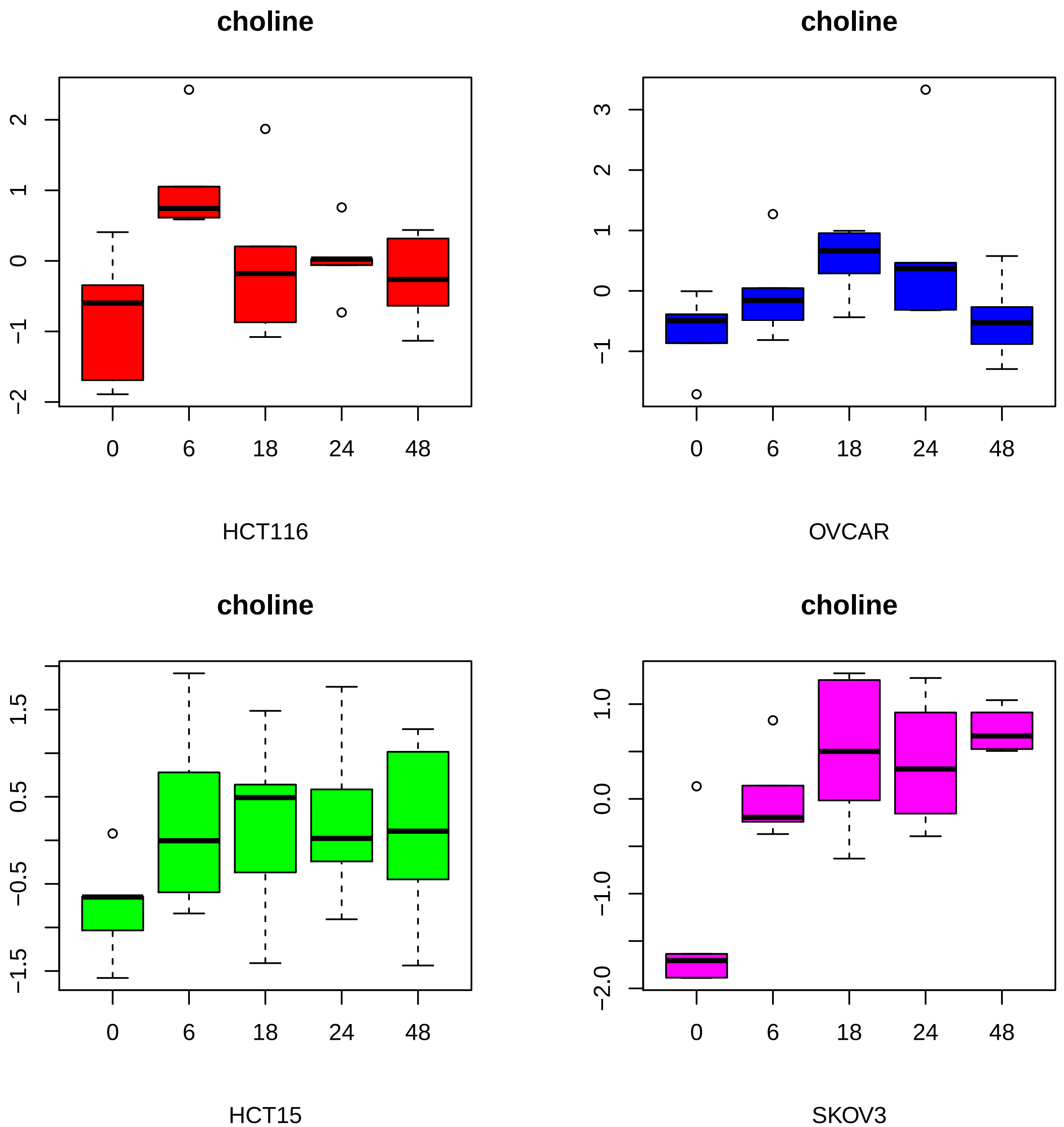

choline
HCT116
choline
OVCAR
choline
HCT15
choline
SKOV3
0
6
18
24
48

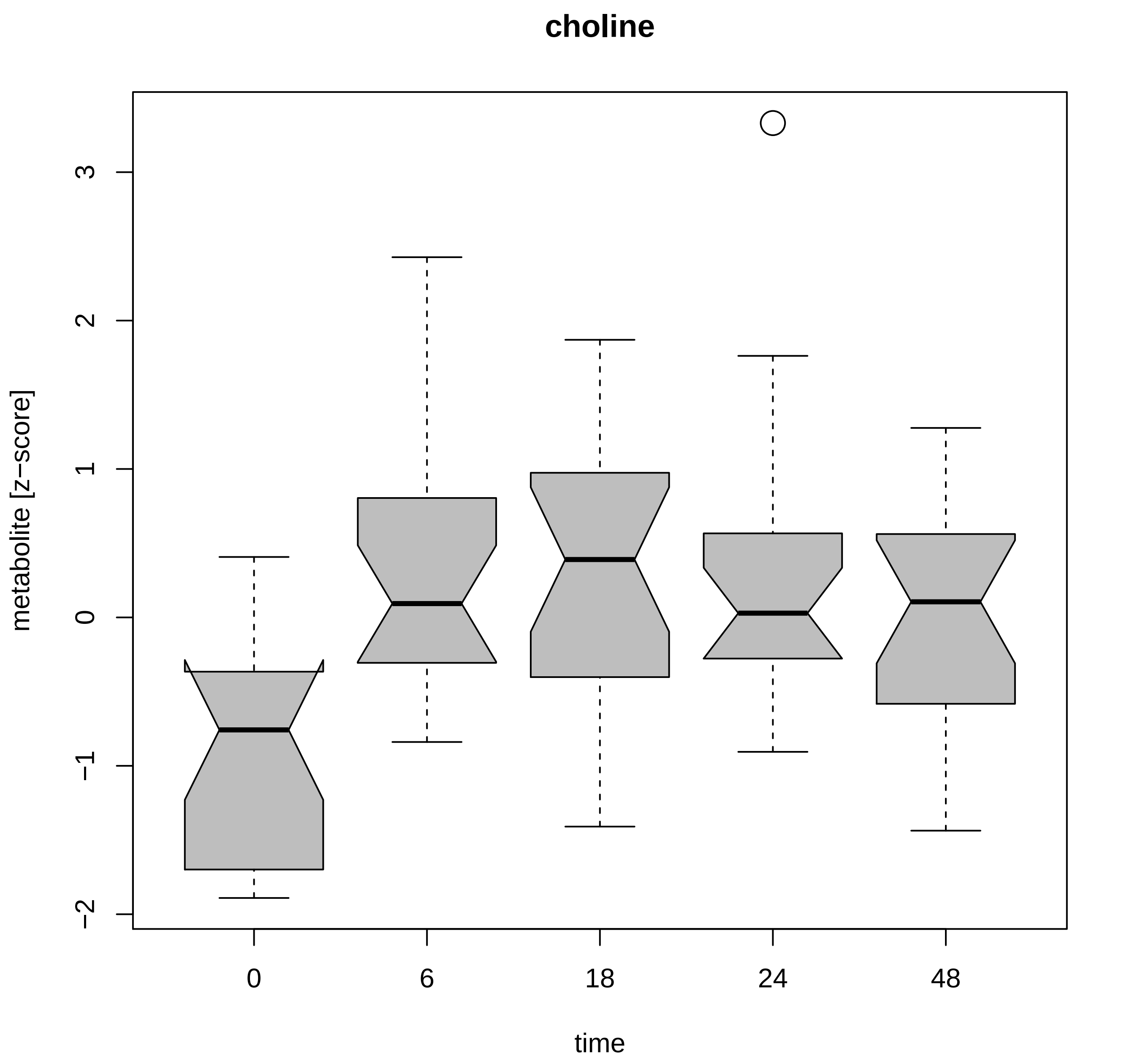
choline
metabolite [z−score]
3
2
1
0
−1
−2
0
6
18
24
48
time

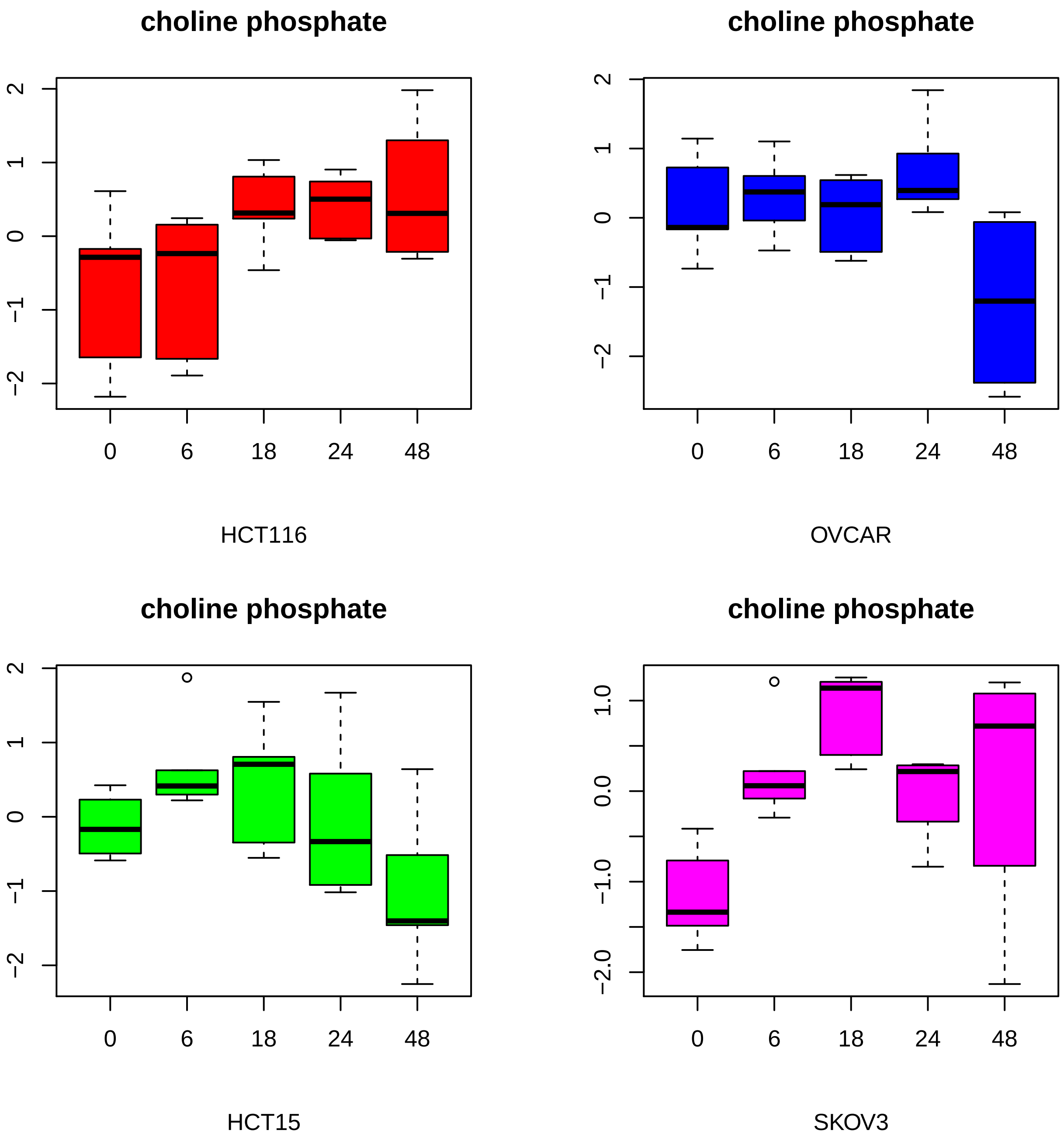
choline phosphate
HCT116
choline phosphate
OVCAR
choline phosphate
HCT15
choline phosphate
SKOV3
0
6
18
24
48

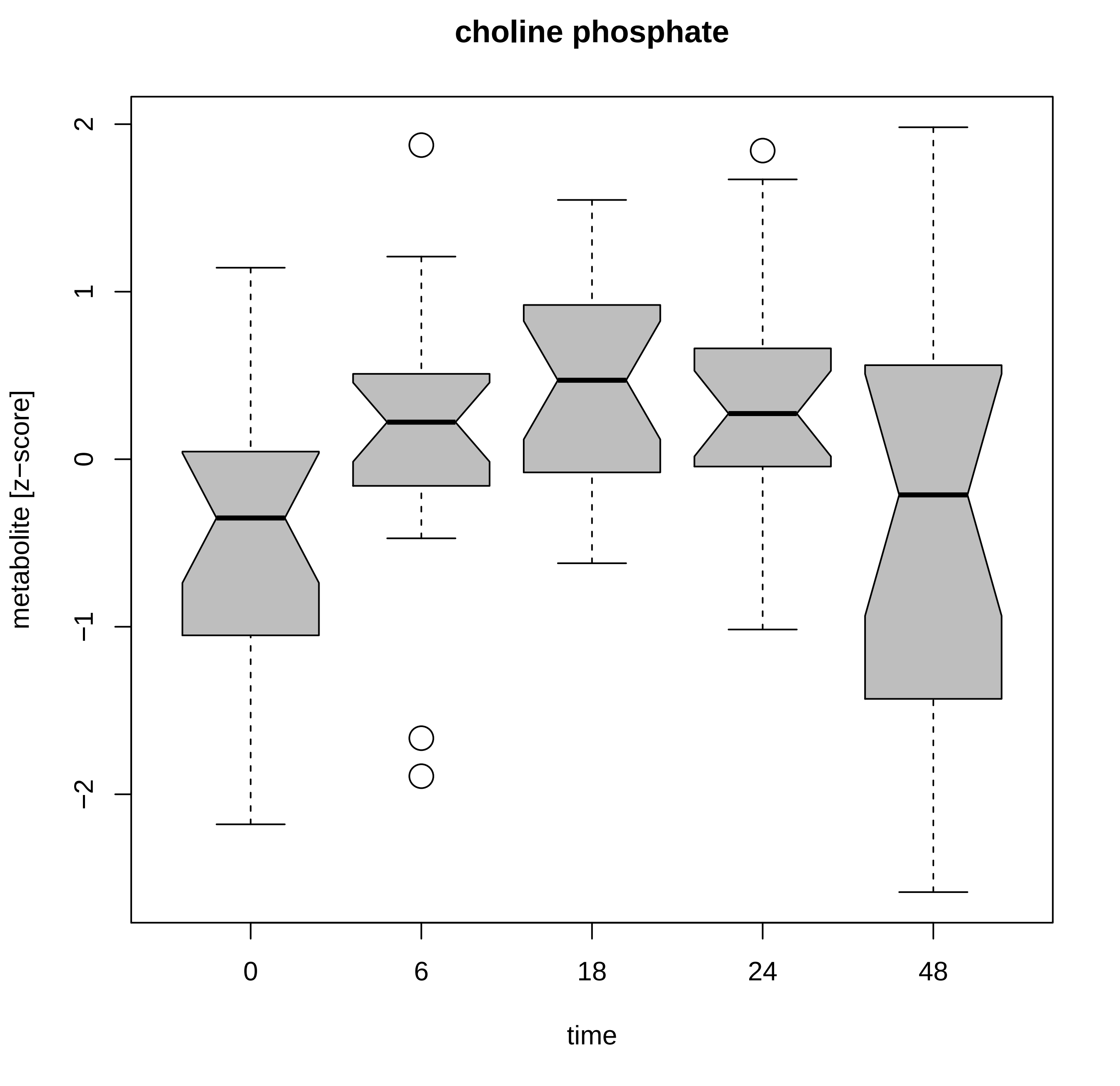

choline phosphate
metabolite [z−score]
time
2
1
0
−1
−2
0
6
18
24
48

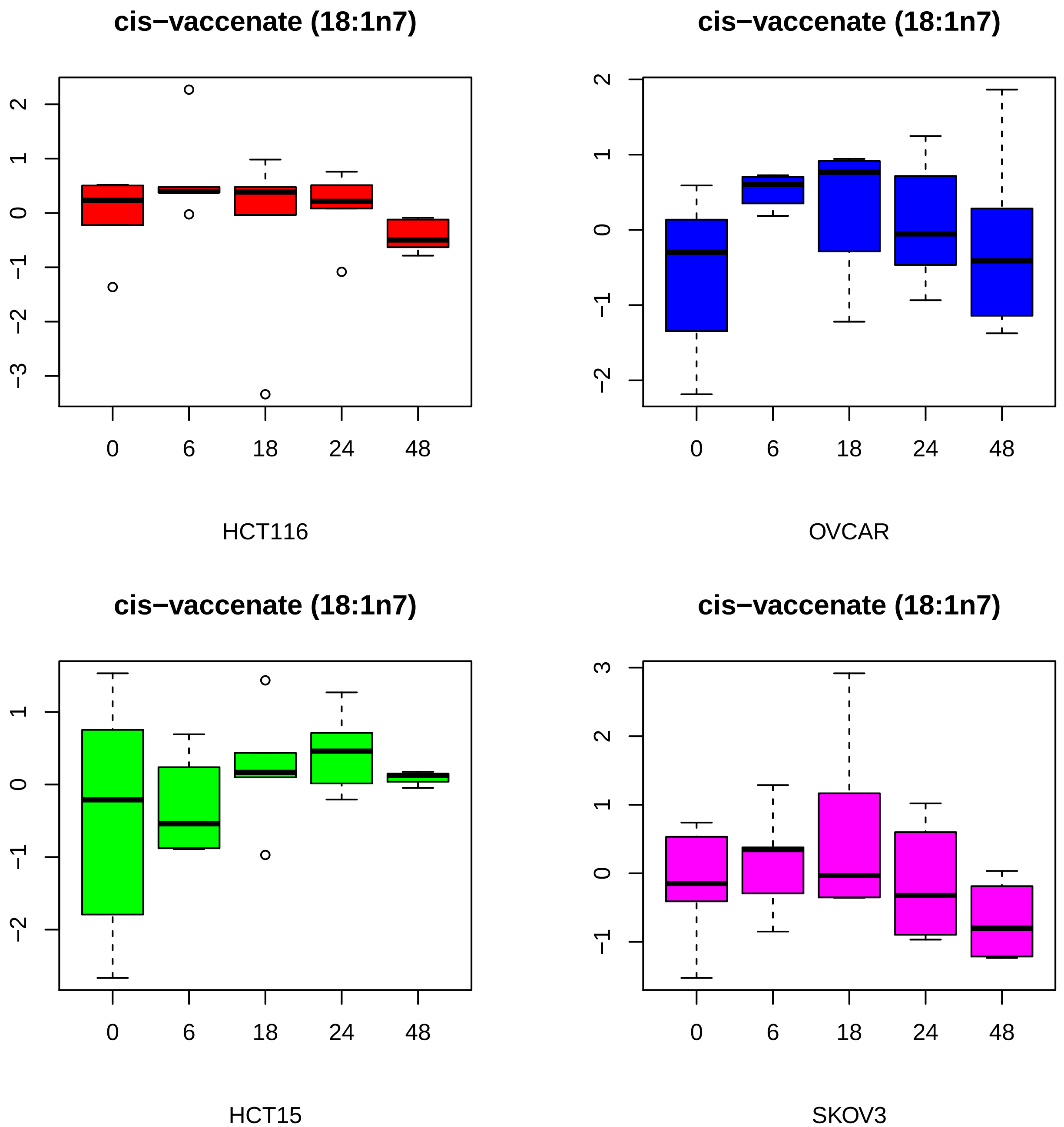

cis−vaccenate (18:1n7)
HCT116
cis−vaccenate (18:1n7)
OVCAR
cis−vaccenate (18:1n7)
HCT15
cis−vaccenate (18:1n7)
SKOV3
0
6
18
24
48

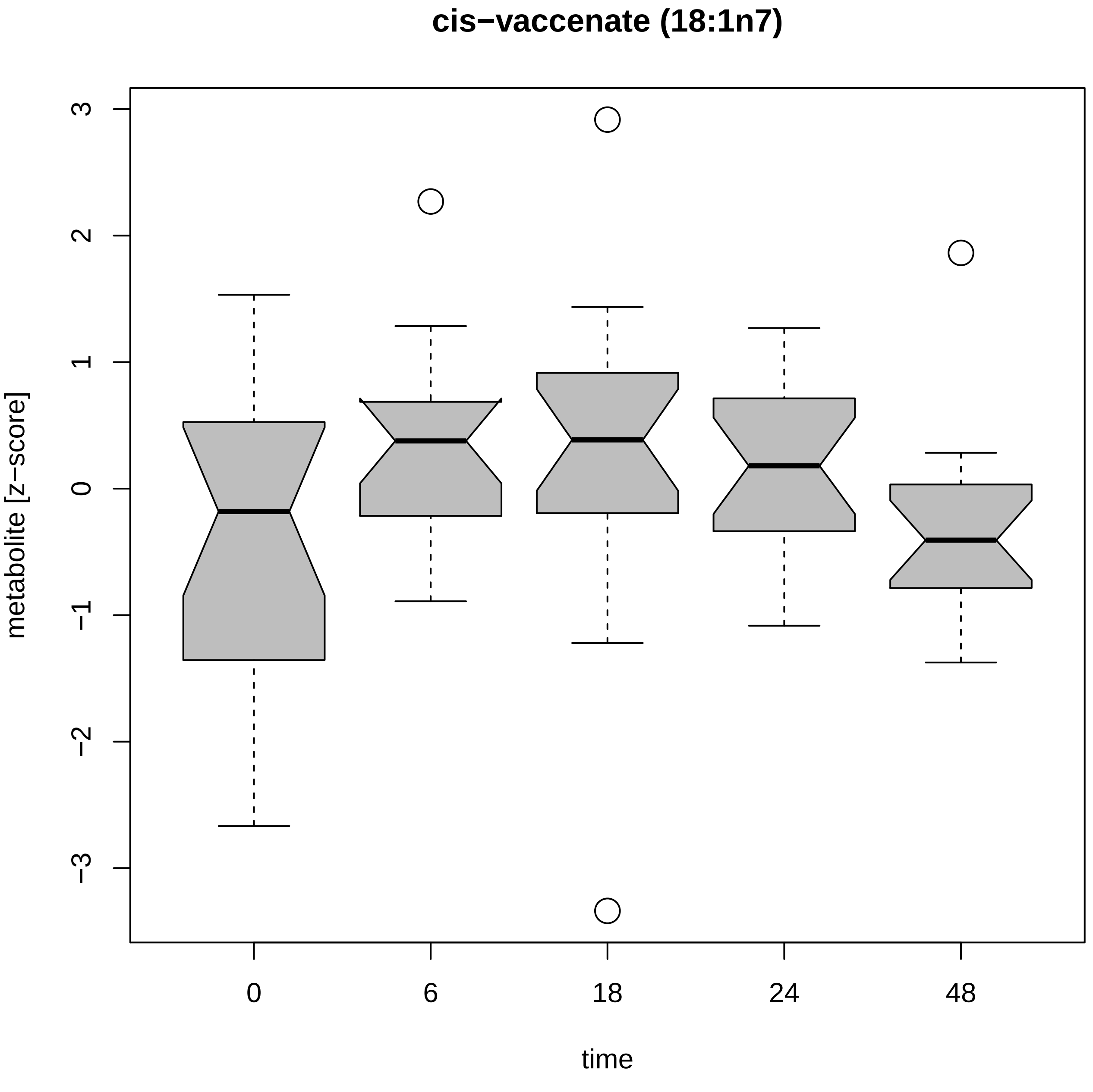

cis−vaccenate (18:1n7)
metabolite [z−score]
3
2
1
0
−1
−2
−3
0
6
18
24
48
time

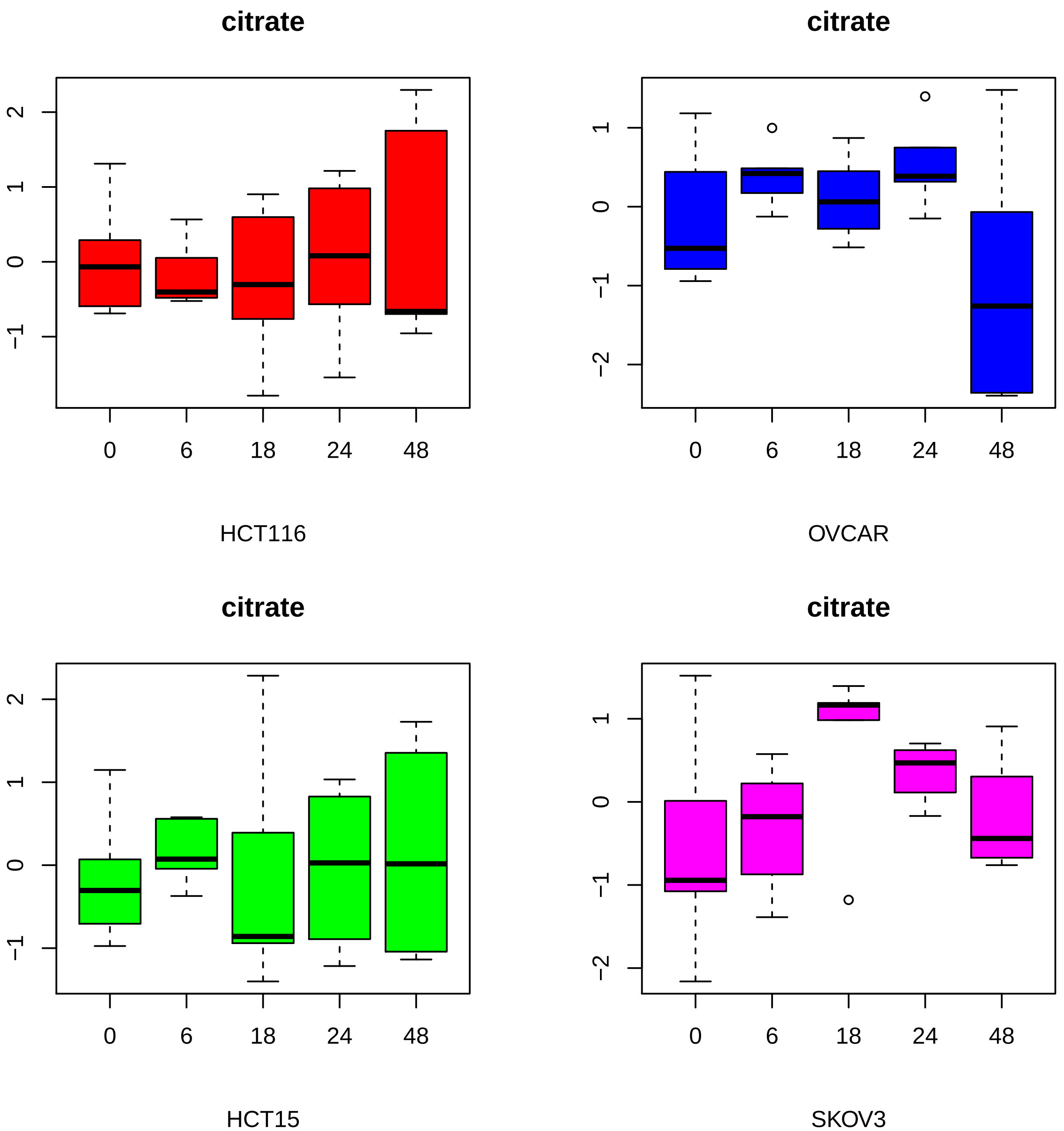

citrate
2
1
0
−1
0
6
18
24
48
HCT116
citrate
1
0
−1
−2
0
6
18
24
48
OVCAR
citrate
2
1
0
−1
0
6
18
24
48
HCT15
citrate
1
0
−1
−2
0
6
18
24
48
SKOV3

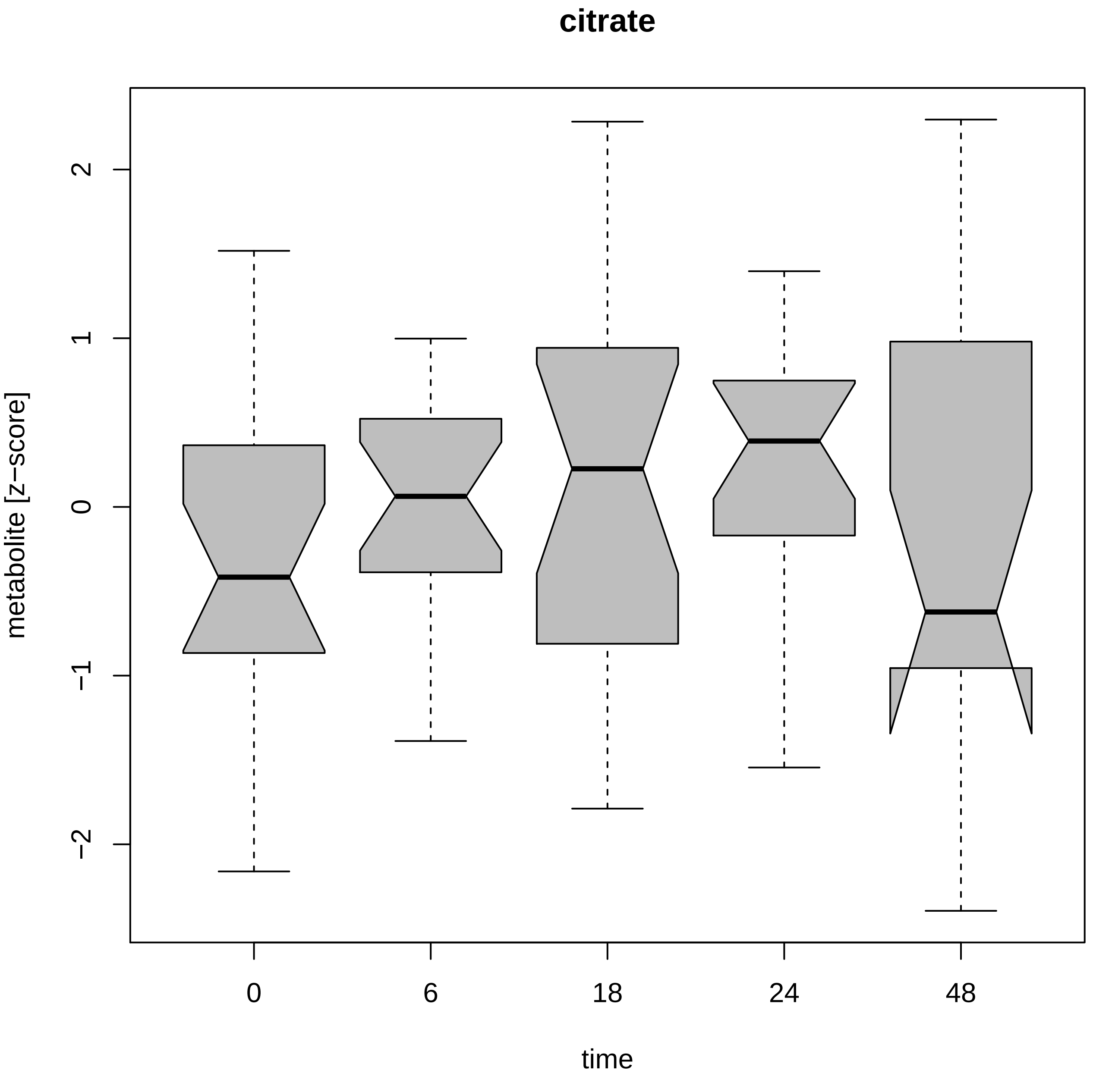
citrate
metabolite [z−score]
2
1
0
−1
−2
0
6
18
24
48
time

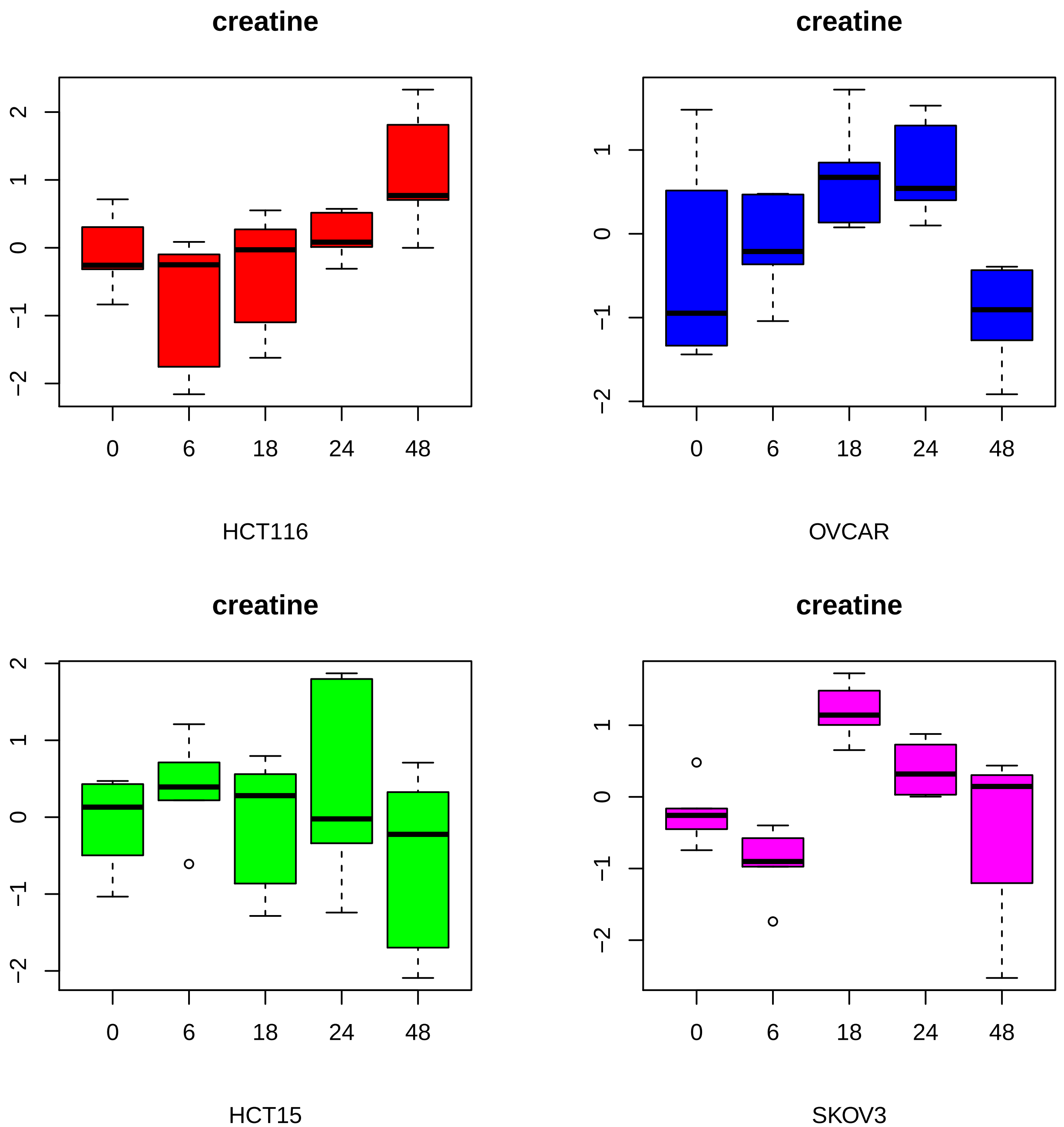
creatine
HCT116
OVCAR
HCT15
SKOV3
0
6
18
24
48

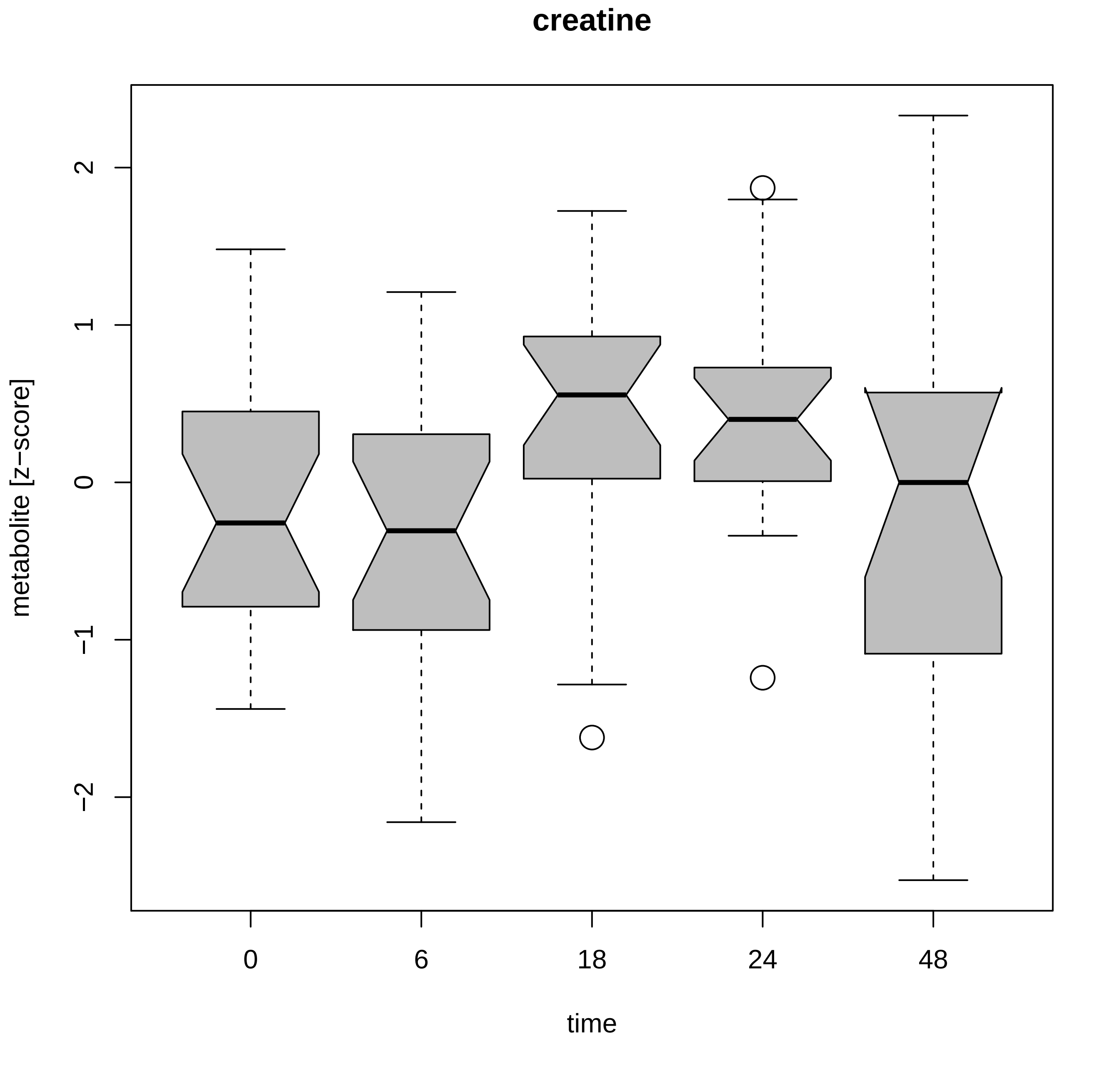
creatine
metabolite [z−score]
2
1
0
−1
−2
0
6
18
24
48
time

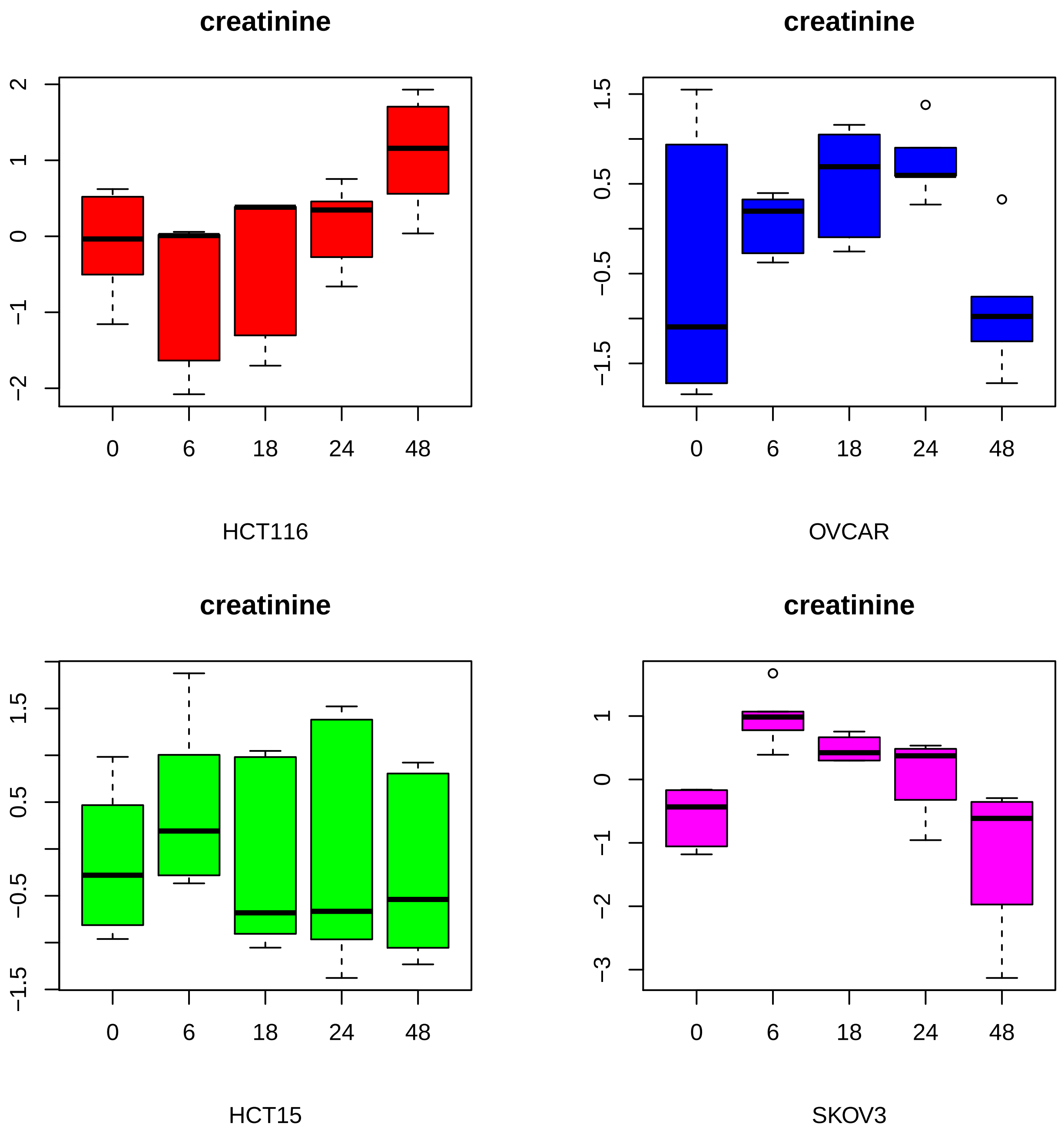
creatinine
HCT116
creatinine
OVCAR
creatinine
HCT15
creatinine
SKOV3
0
6
18
24
48

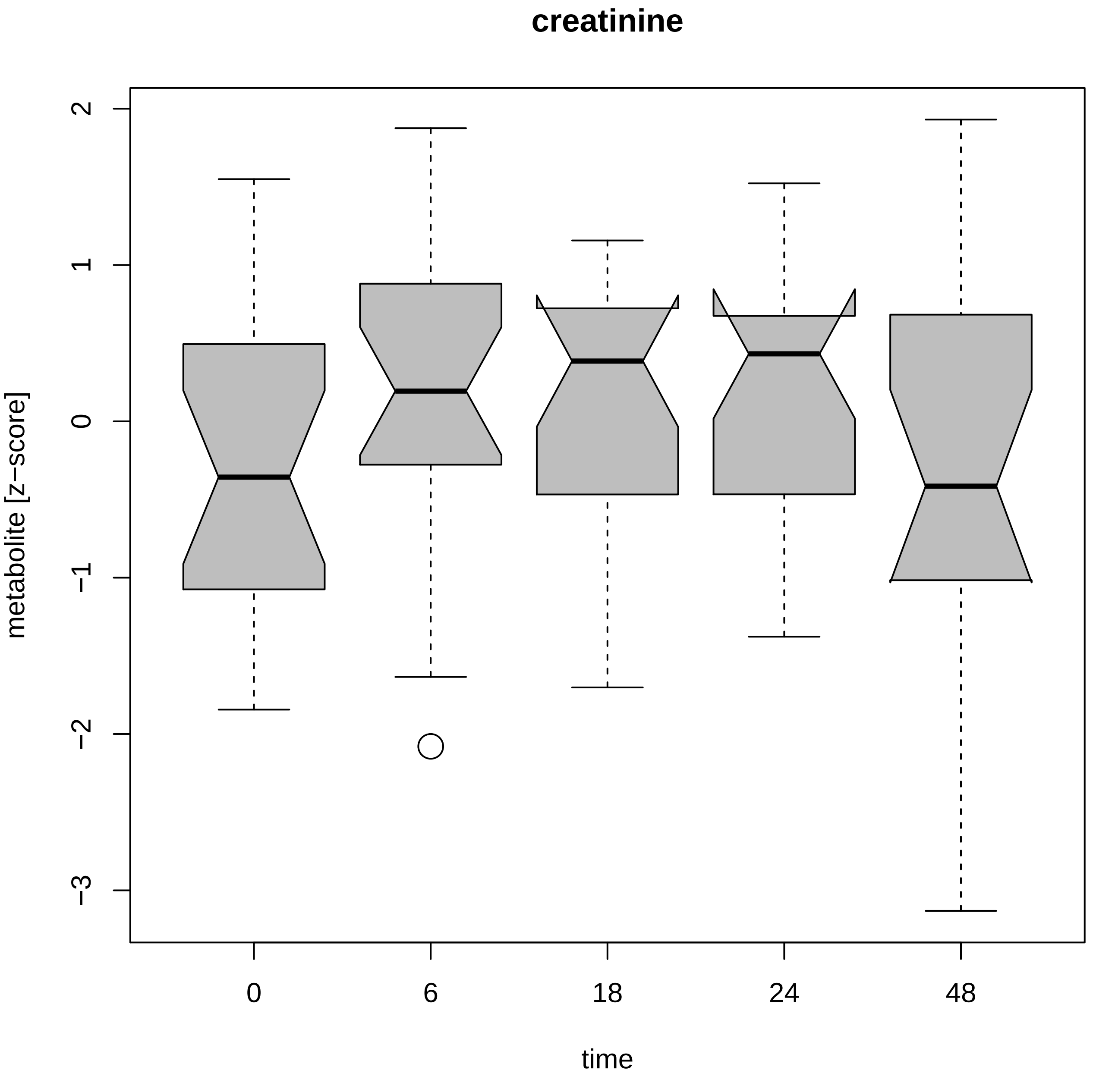

creatinine
2
1
0
−1
−2
−3
metabolite [z−score]
0
6
18
24
48
time

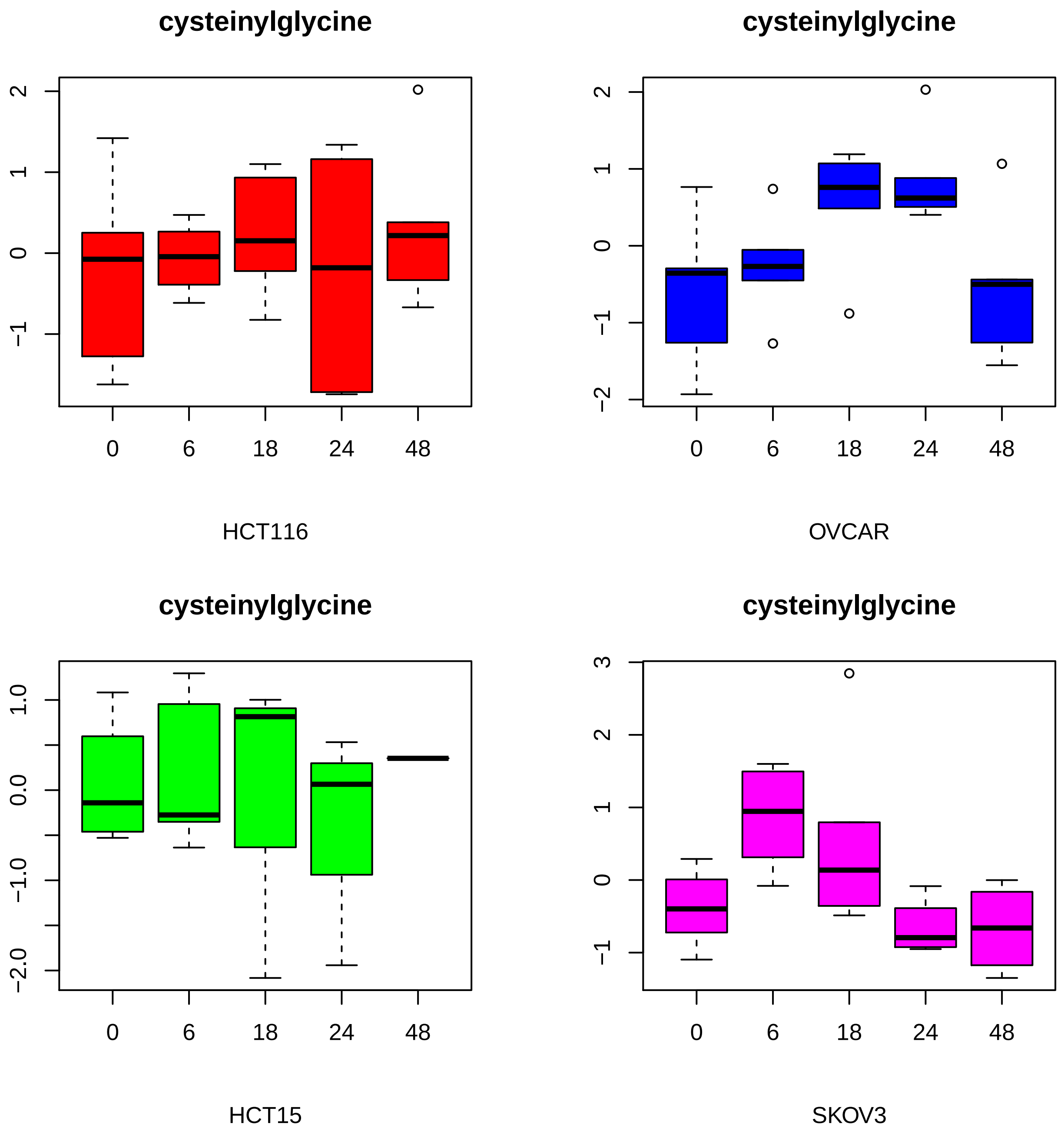
cysteinylglycine
HCT116
OVCAR
HCT15
SKOV3
0
6
18
24
48
−2
−1
0
1
2
3
−2.0
−1.0
0.0
1.0

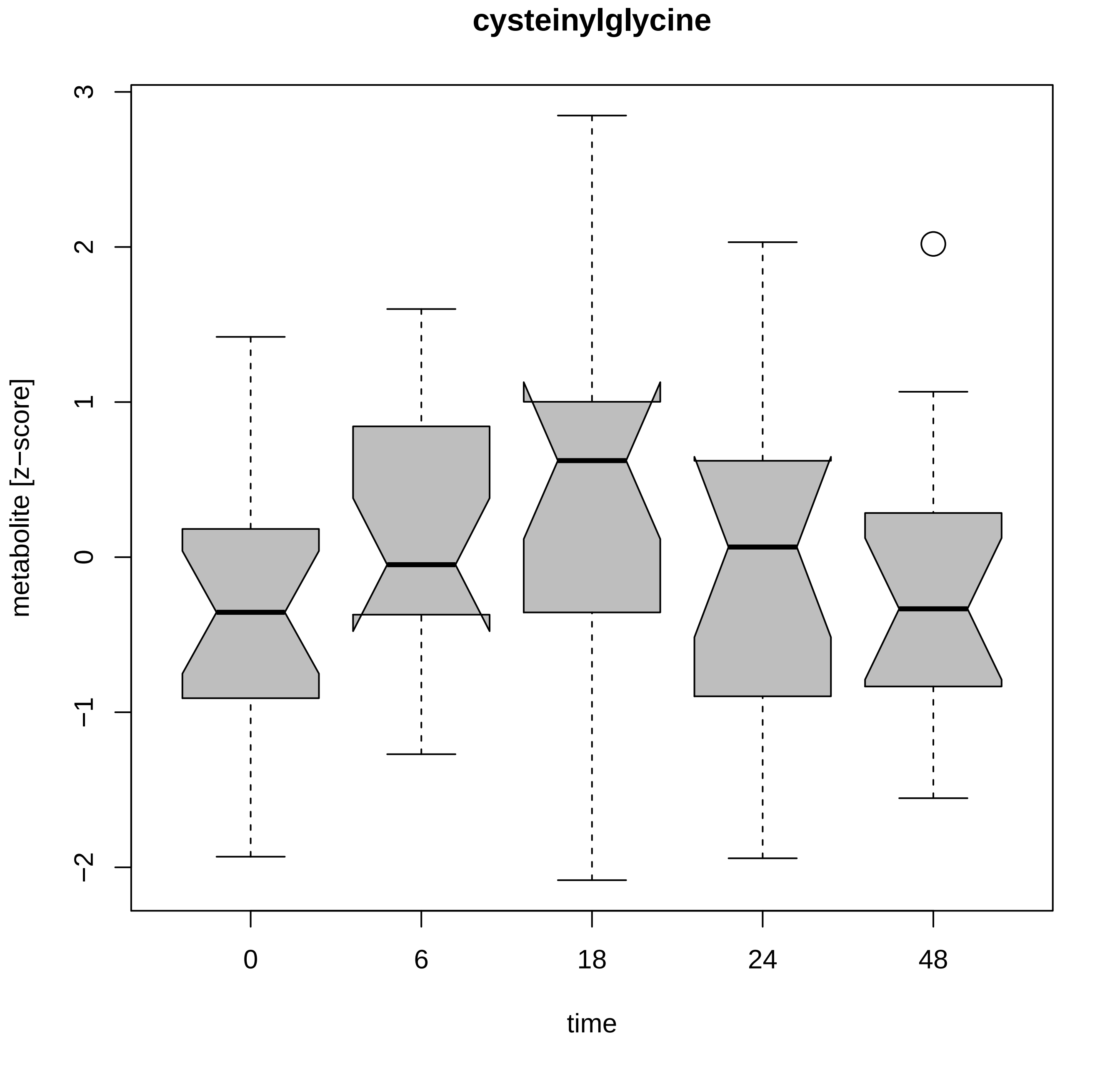
cysteinylglycine
metabolite [z−score]
3
2
1
0
−1
−2
0
6
18
24
48
time

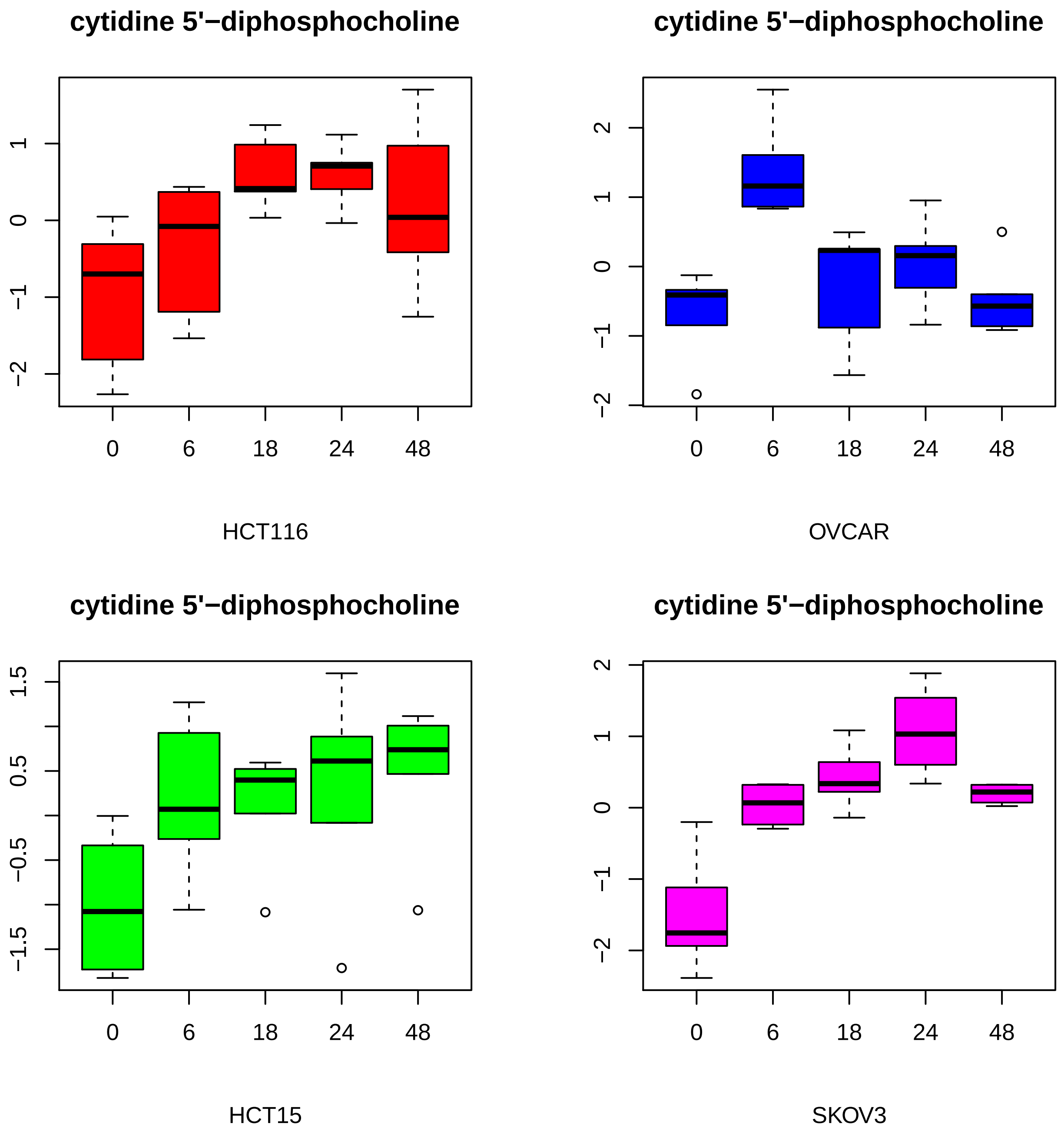
cytidine 5'−diphosphocholine
1
0
−1
−2
0
6
18
24
48
HCT116
cytidine 5'−diphosphocholine
2
1
0
−1
−2
0
6
18
24
48
OVCAR
cytidine 5'−diphosphocholine
1.5
0.5
−0.5
−1.5
0
6
18
24
48
HCT15
cytidine 5'−diphosphocholine
2
1
0
−1
−2
0
6
18
24
48
SKOV3

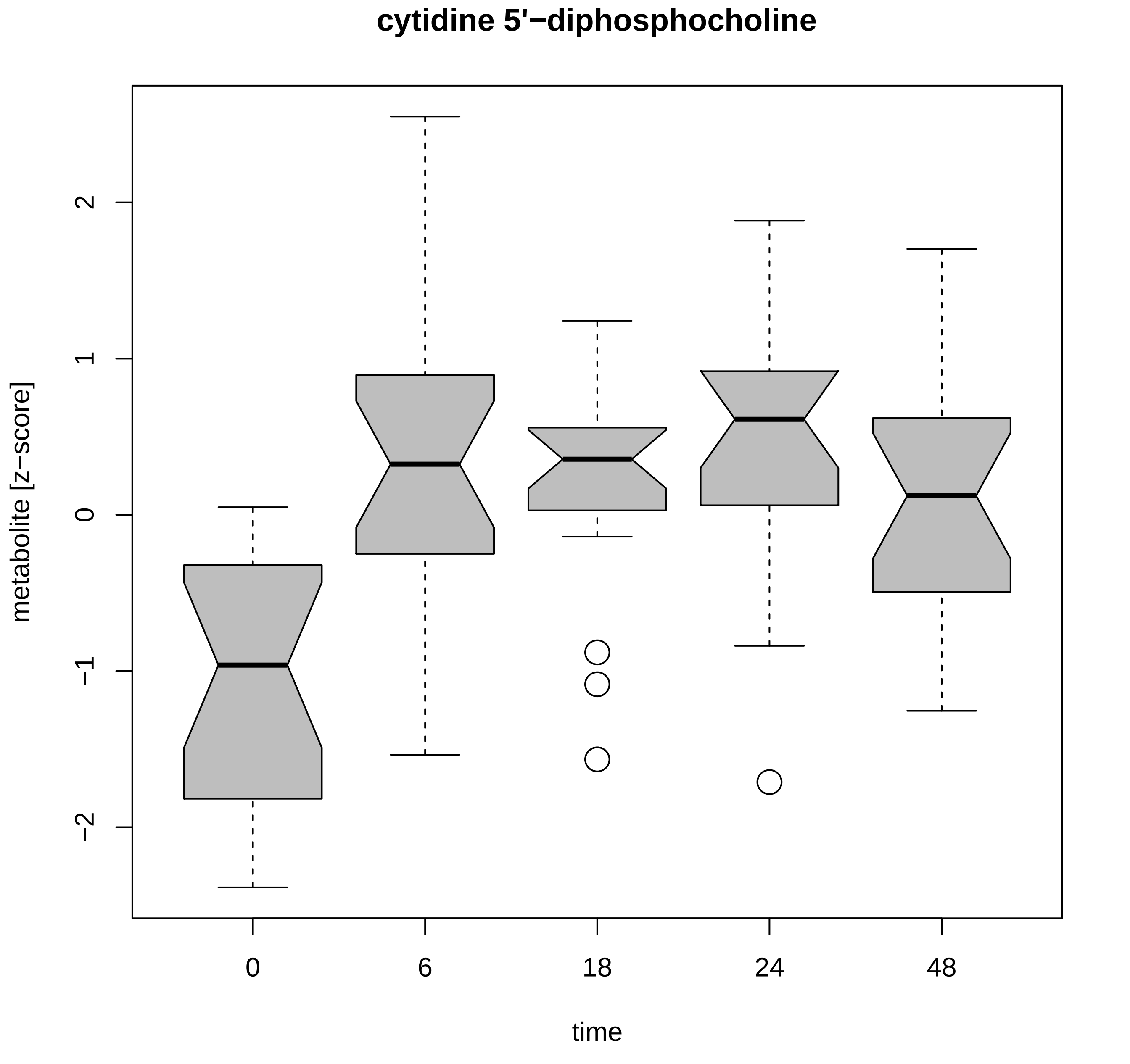
cytidine 5'−diphosphocholine
metabolite [z−score]
2
1
0
−1
−2
0
6
18
24
48
time

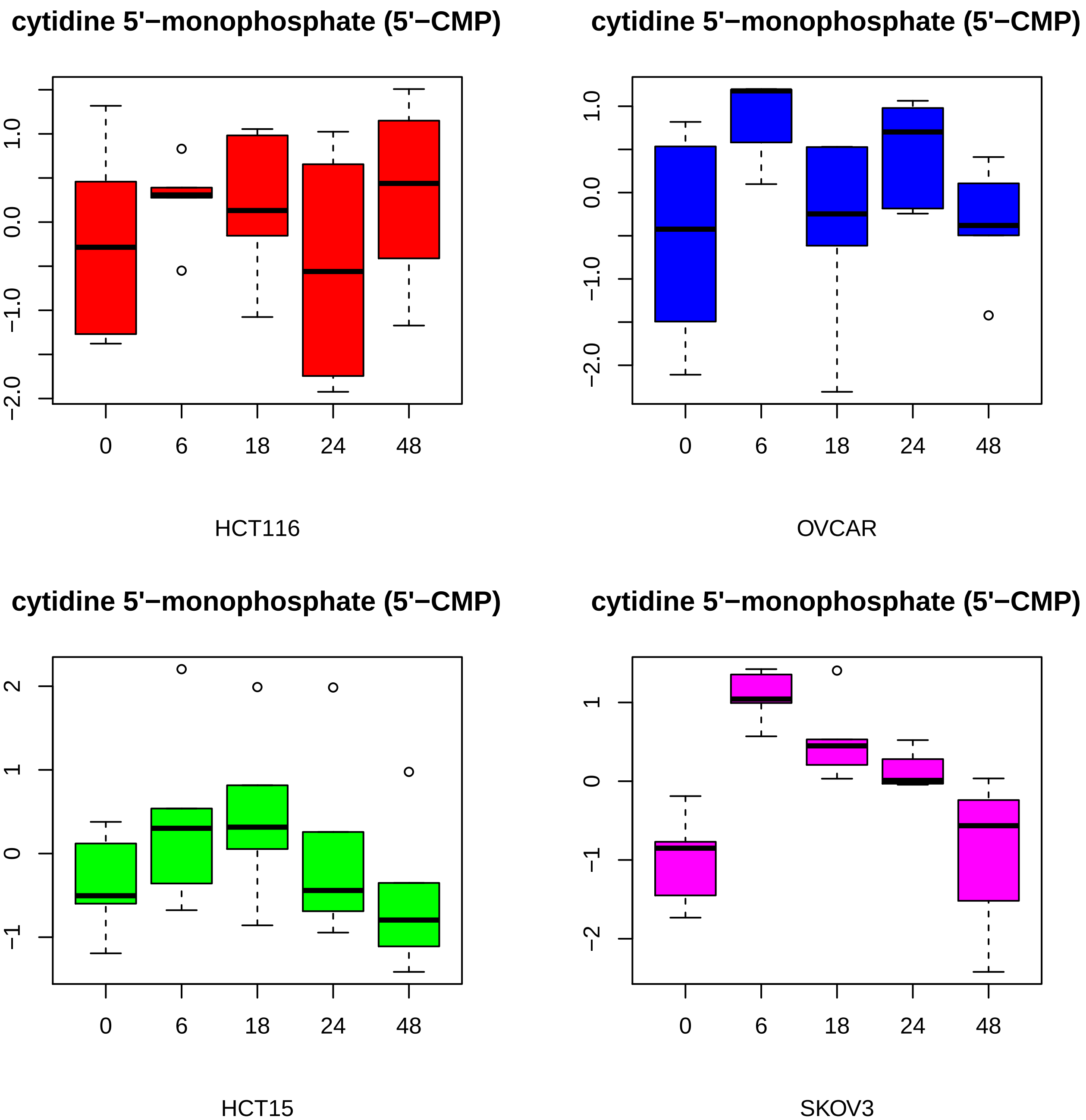

cytidine 5'−monophosphate (5'−CMP)
−2.0
−1.0
0.0
1.0
0
6
18
24
48
HCT116
cytidine 5'−monophosphate (5'−CMP)
−2.0
−1.0
0.0
1.0
0
6
18
24
48
OVCAR
cytidine 5'−monophosphate (5'−CMP)
−1
0
1
2
0
6
18
24
48
HCT15
cytidine 5'−monophosphate (5'−CMP)
−2
−1
0
1
0
6
18
24
48
SKOV3

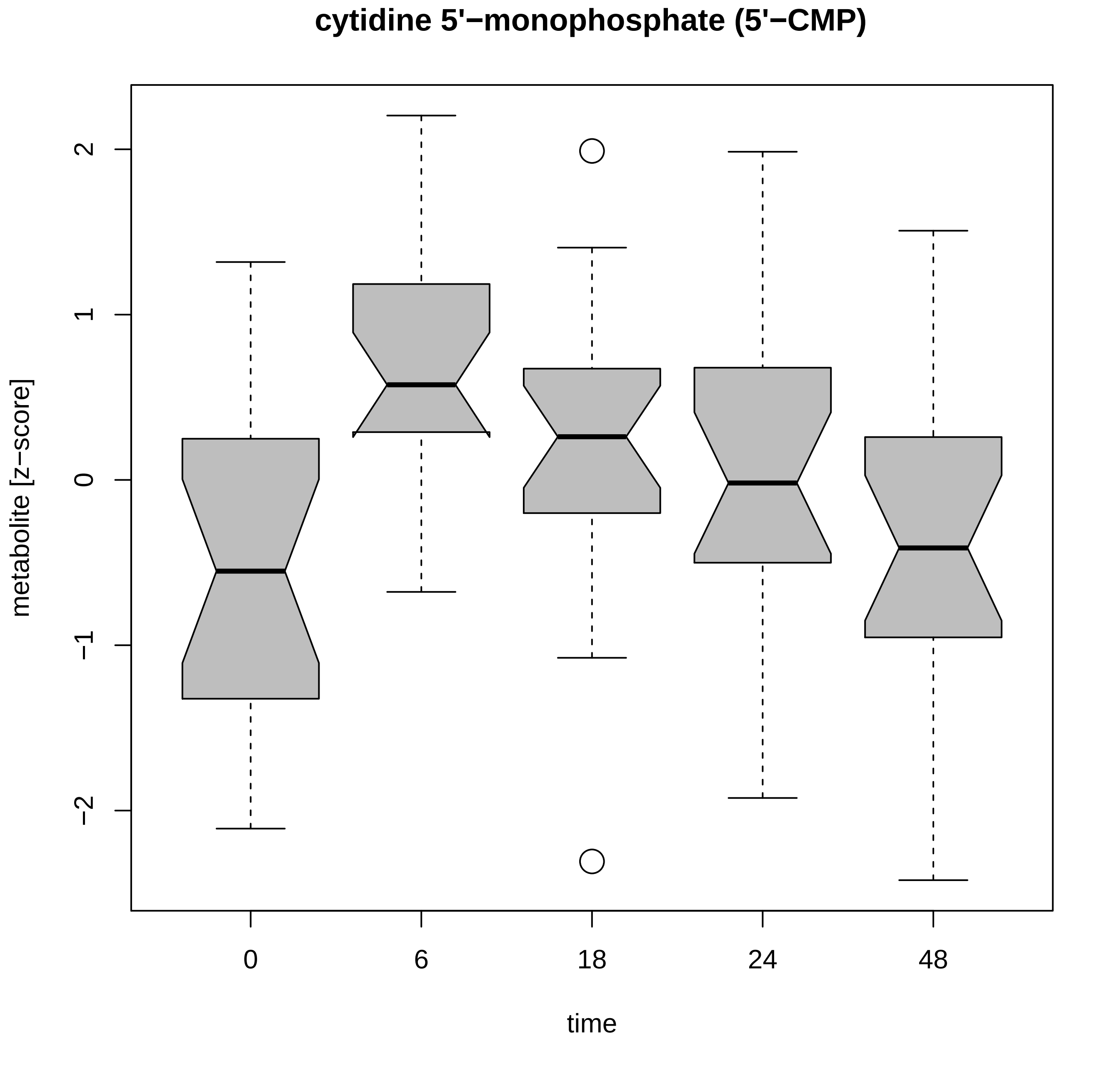
cytidine 5'−monophosphate (5'−CMP)
metabolite [z−score]
time
0
6
18
24
48
−2
−1
0
1
2

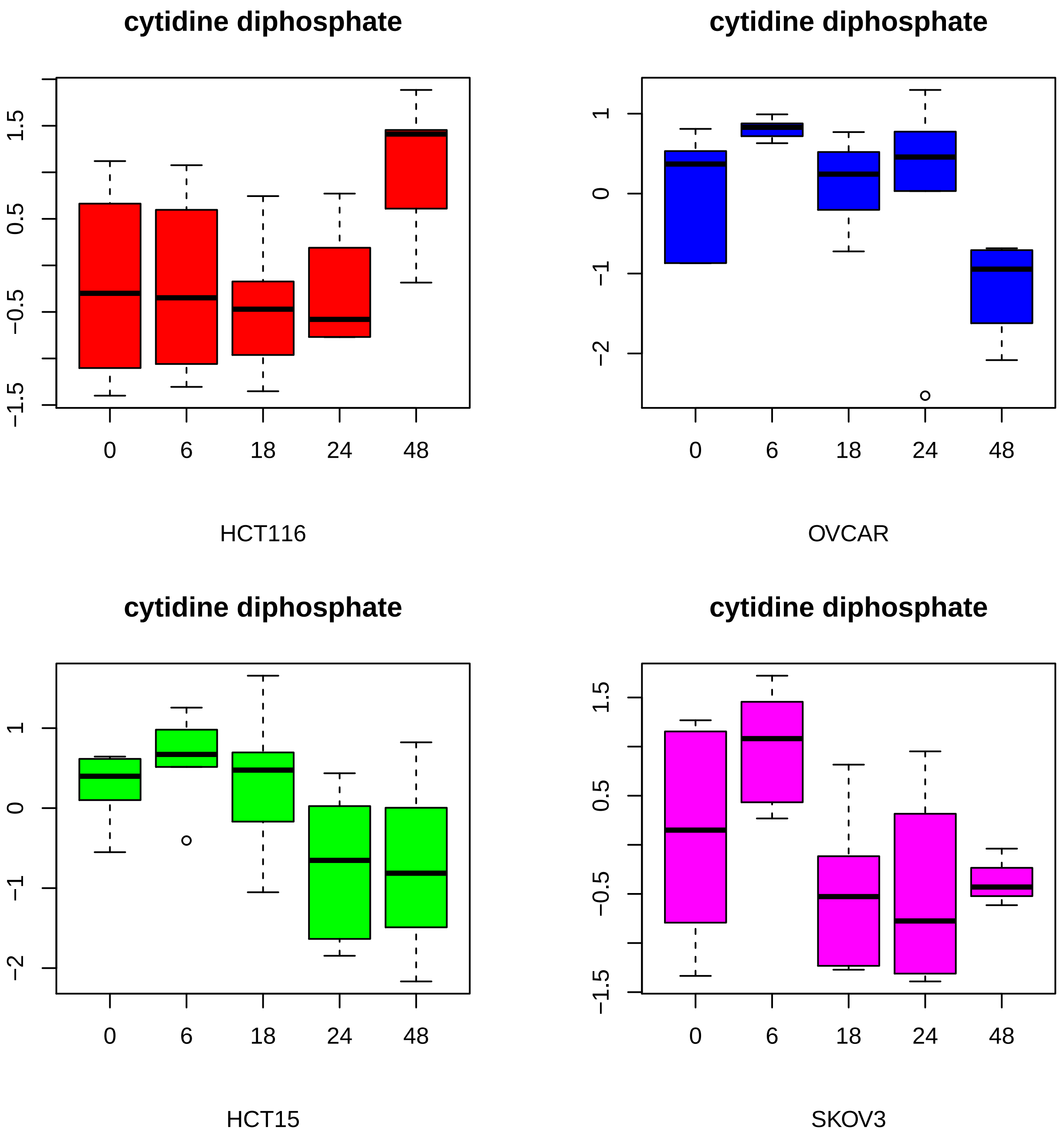
cytidine diphosphate
HCT116
0
6
18
24
48
1.5
0.5
−0.5
−1.5
cytidine diphosphate
OVCAR
1
0
−1
−2
cytidine diphosphate
HCT15
cytidine diphosphate
SKOV3

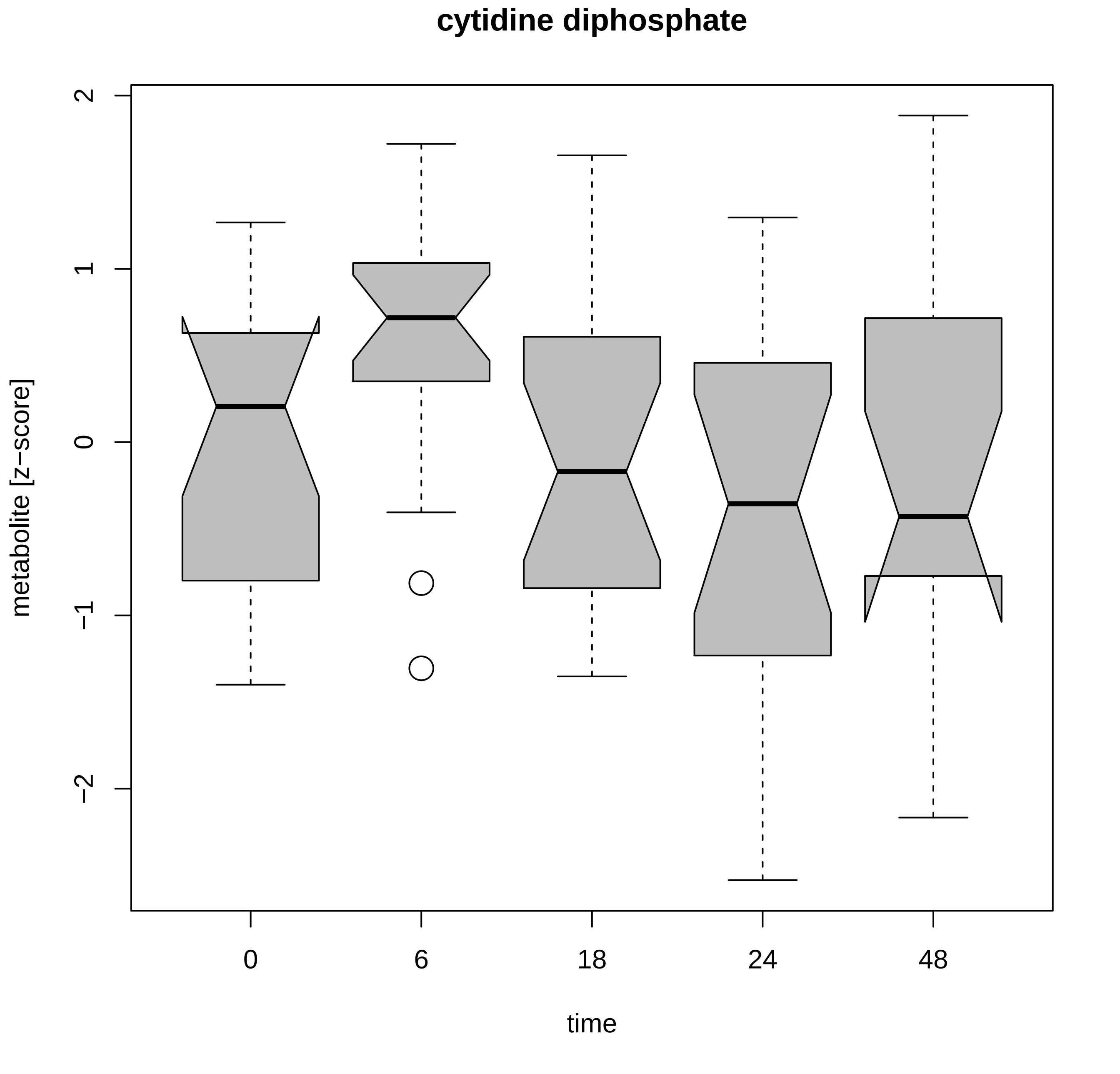
cytidine diphosphate
metabolite [z−score]
2
1
0
−1
−2
0
6
18
24
48
time

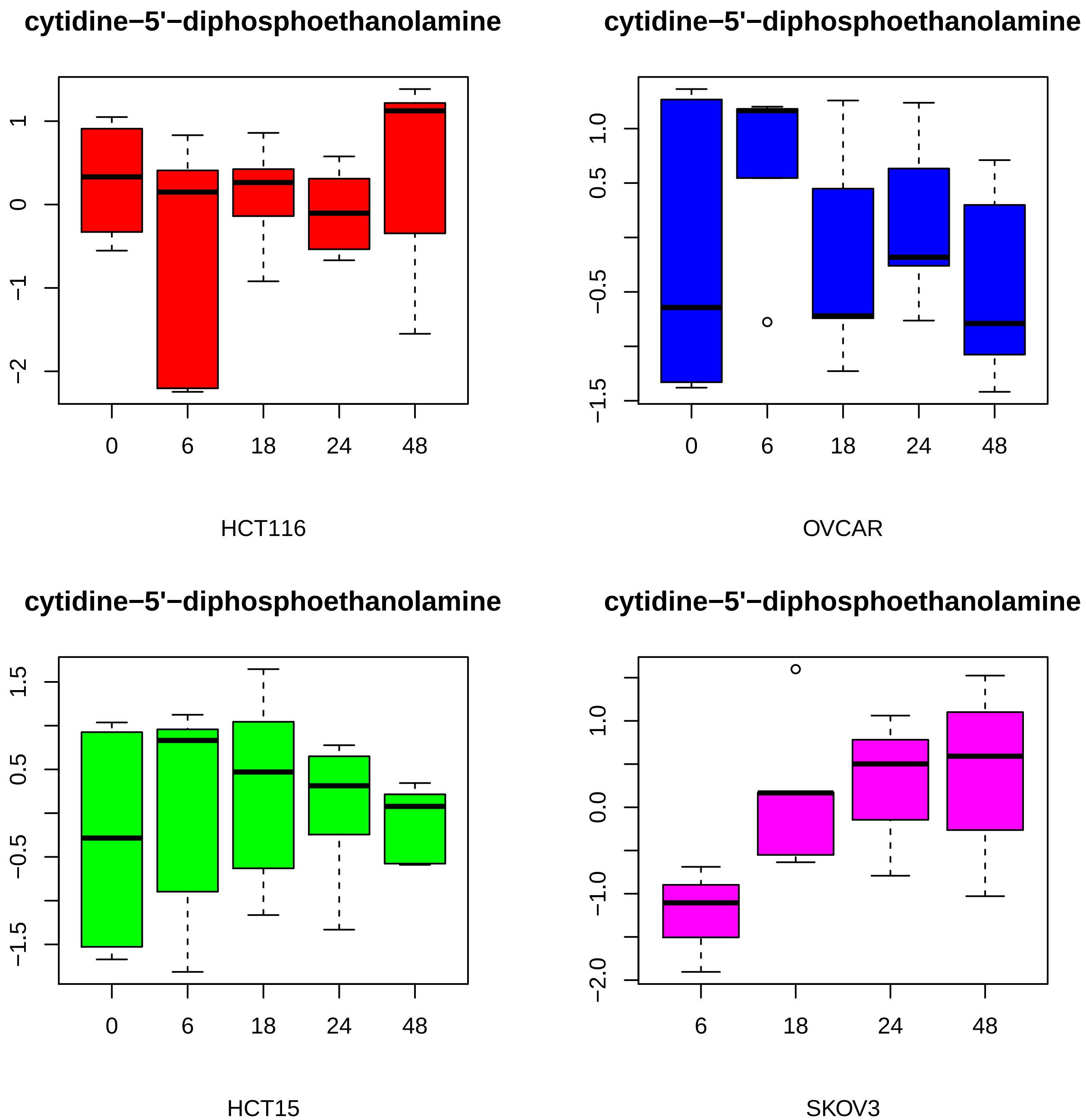
cytidine−5'−diphosphoethanolamine
HCT116
0
6
18
24
48
1
0
−1
−2
cytidine−5'−diphosphoethanolamine
OVCAR
1.0
0.5
−0.5
−1.5
cytidine−5'−diphosphoethanolamine
HCT15
1.5
0.5
−0.5
−1.5
cytidine−5'−diphosphoethanolamine
SKOV3
1.0
0.0
−1.0
−2.0

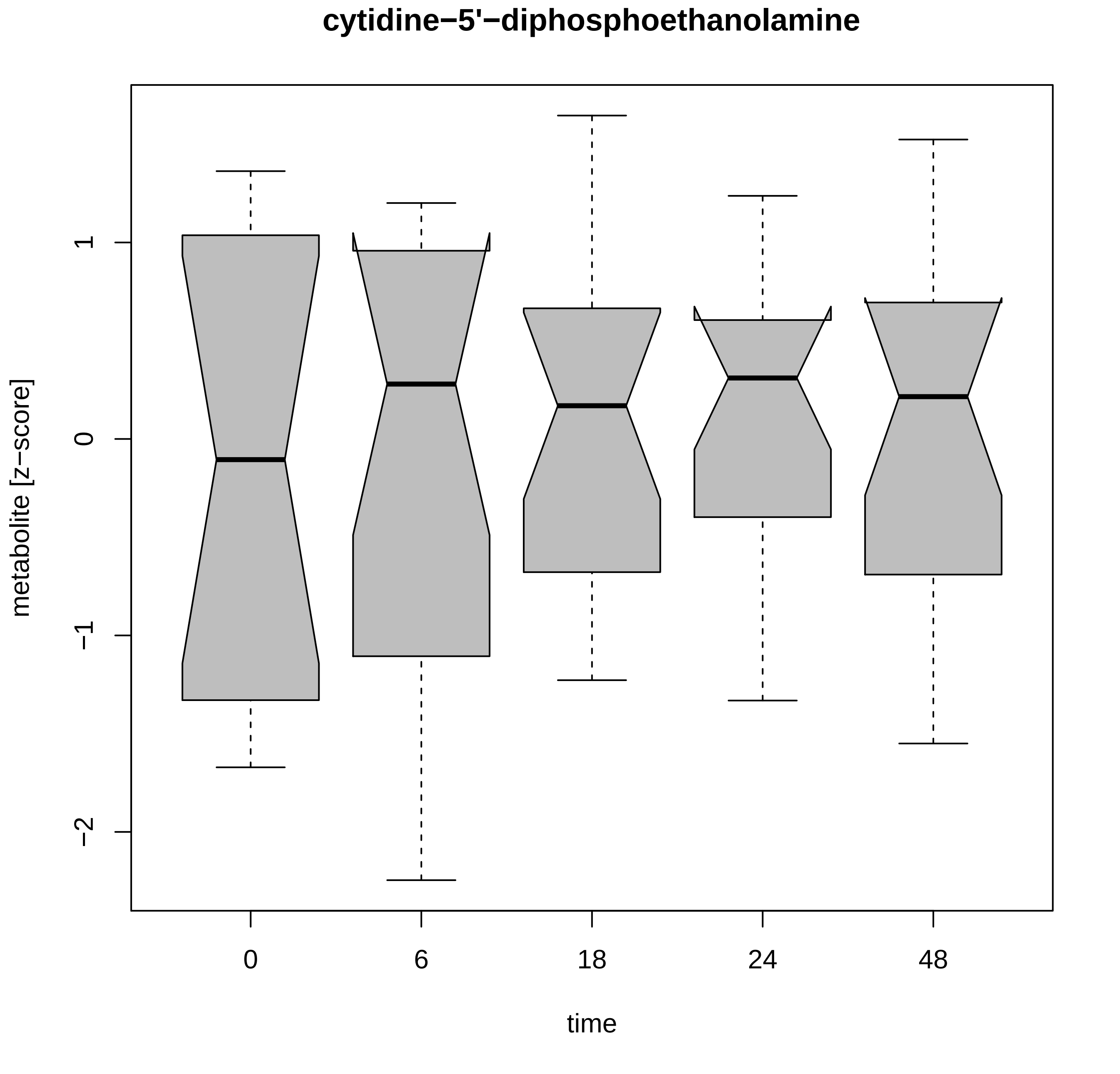

cytidine−5'−diphosphoethanolamine
metabolite [z−score]
1
0
−1
−2
0
6
18
24
48
time

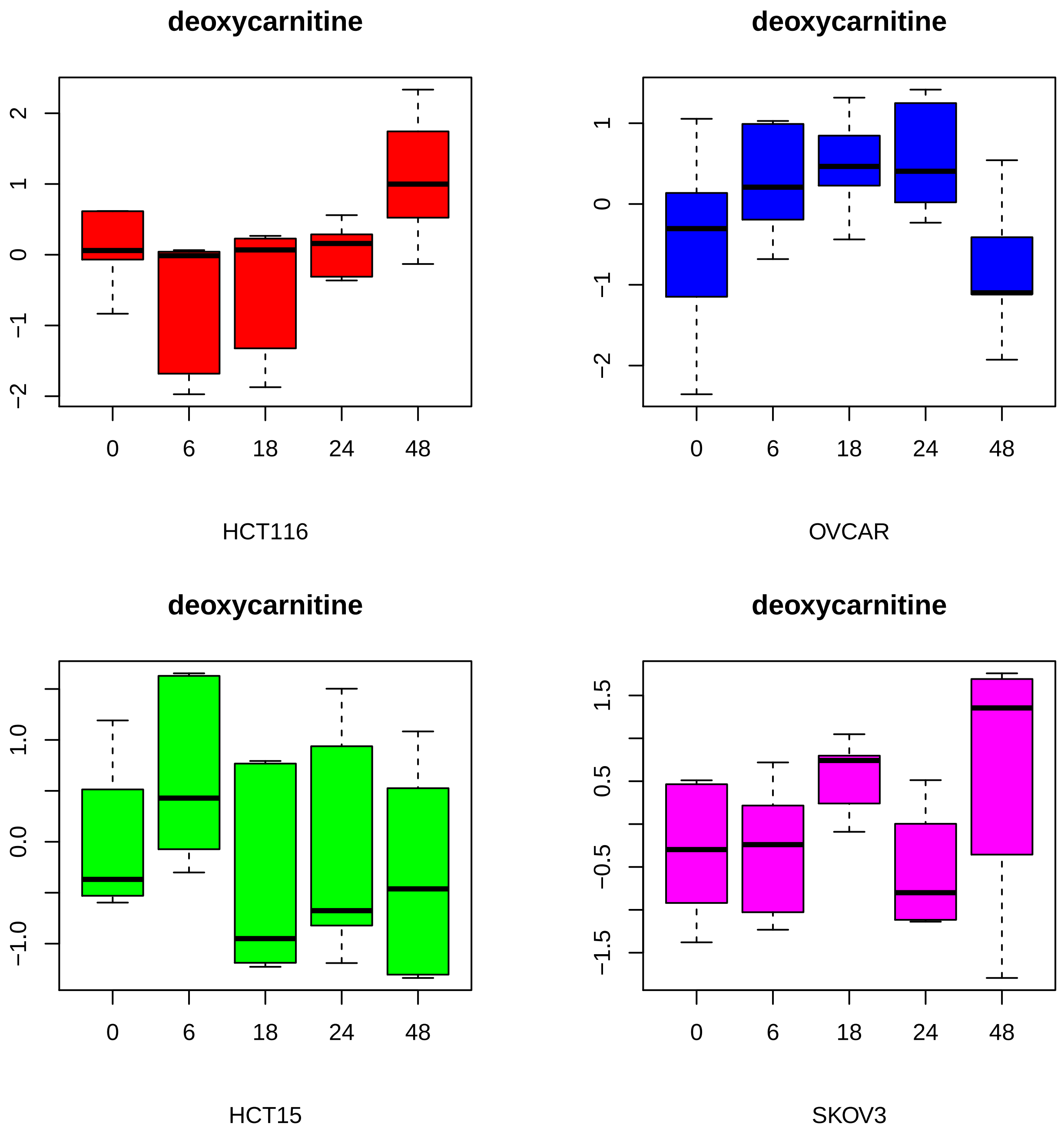
deoxycarnitine
2
1
0
−1
−2
0
6
18
24
48
HCT116
deoxycarnitine
1
0
−1
−2
0
6
18
24
48
OVCAR
deoxycarnitine
1.0
0.0
−1.0
0
6
18
24
48
HCT15
deoxycarnitine
1.5
0.5
−0.5
−1.5
0
6
18
24
48
SKOV3

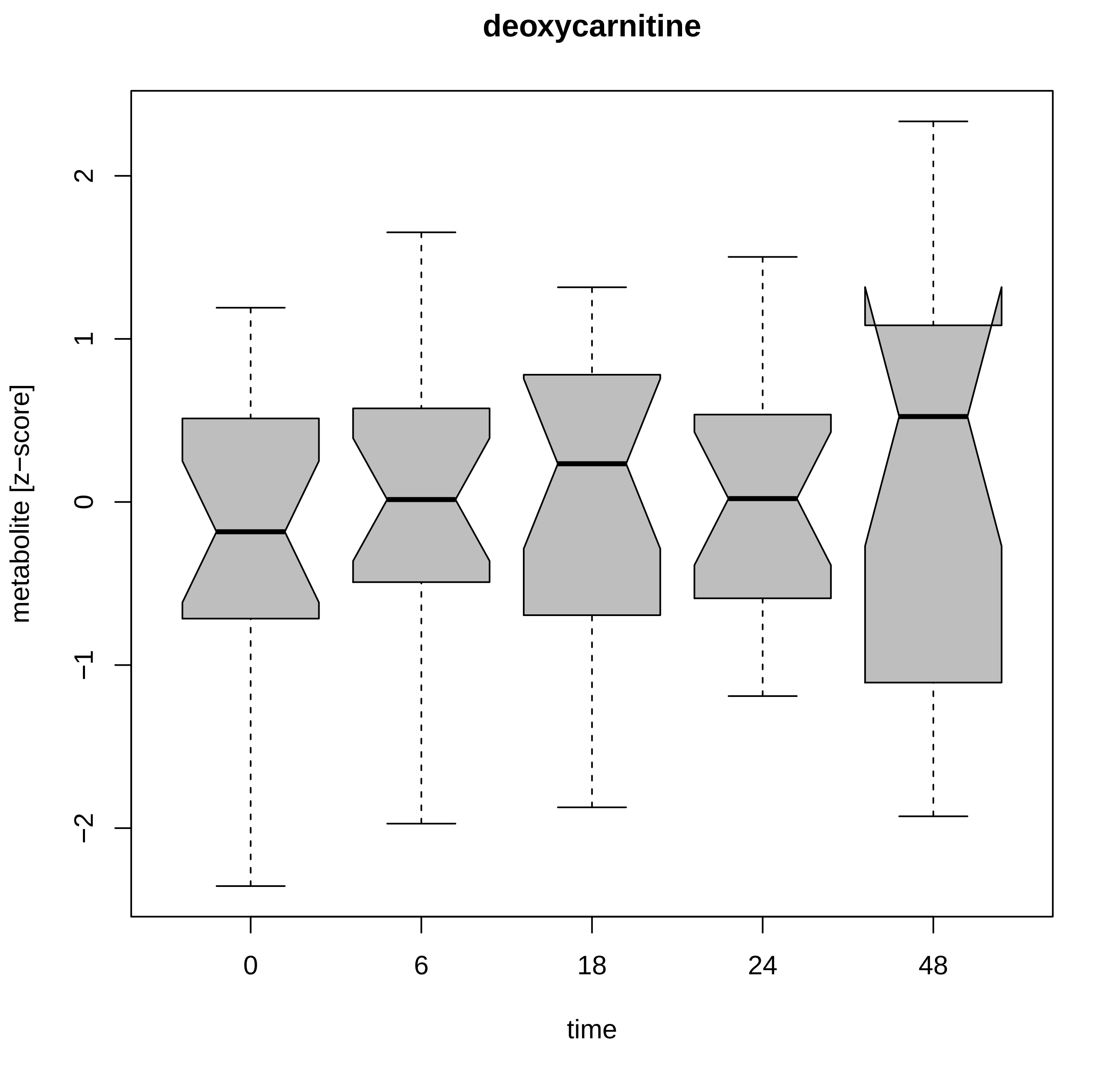
deoxycarnitine
metabolite [z−score]
2
1
0
−1
−2
0
6
18
24
48
time

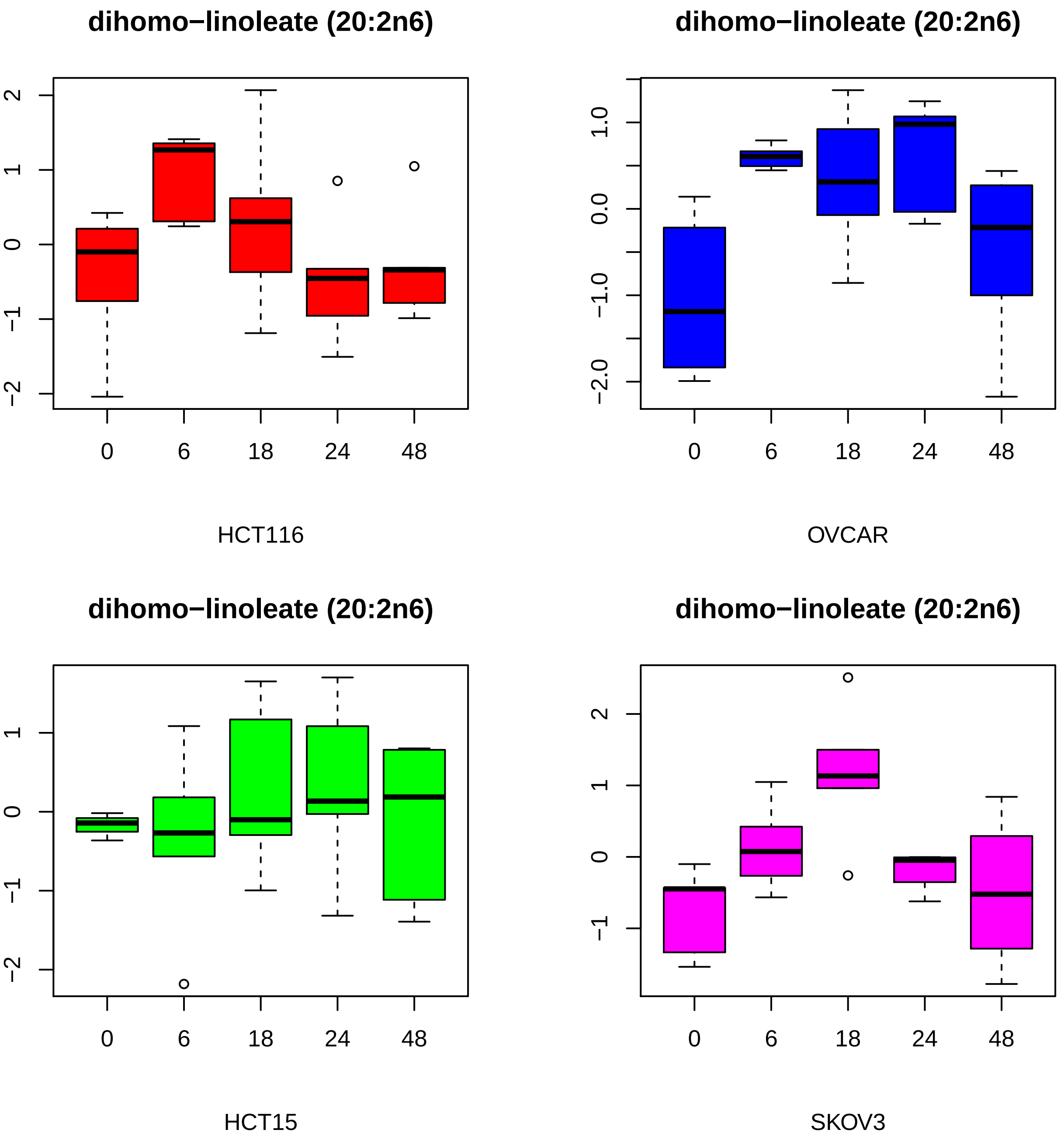
dihomo−linoleate (20:2n6)
HCT116
OVCAR
HCT15
SKOV3
0
6
18
24
48
−2
−1
0
1
2
−2.0
−1.0
0.0
1.0

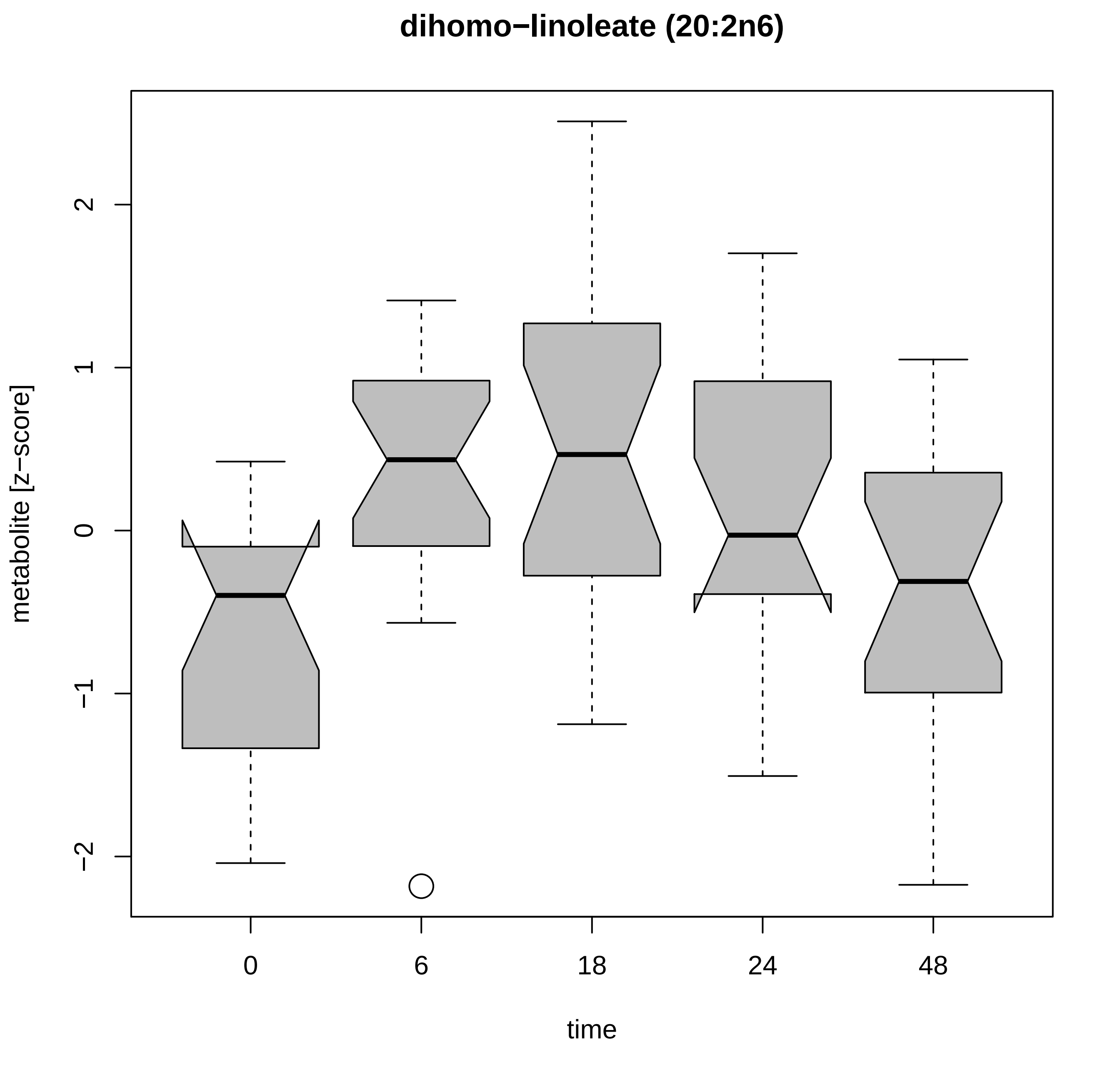
dihomo−linoleate (20:2n6)
metabolite [z−score]
−2
−1
0
1
2
0
6
18
24
48
time

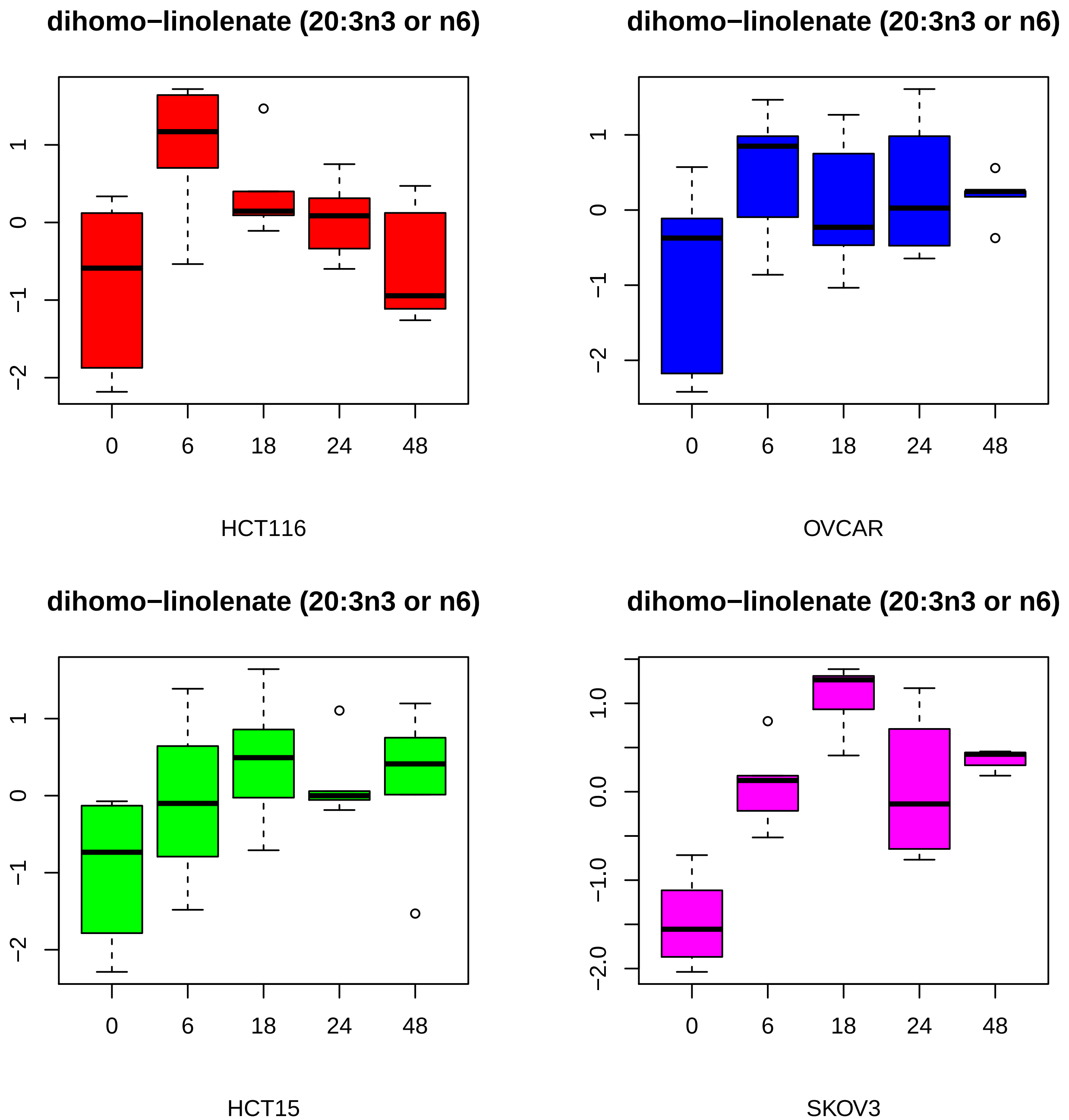

dihomo−linolenate (20:3n3 or n6)
HCT116
OVCAR
HCT15
SKOV3
0
6
18
24
48

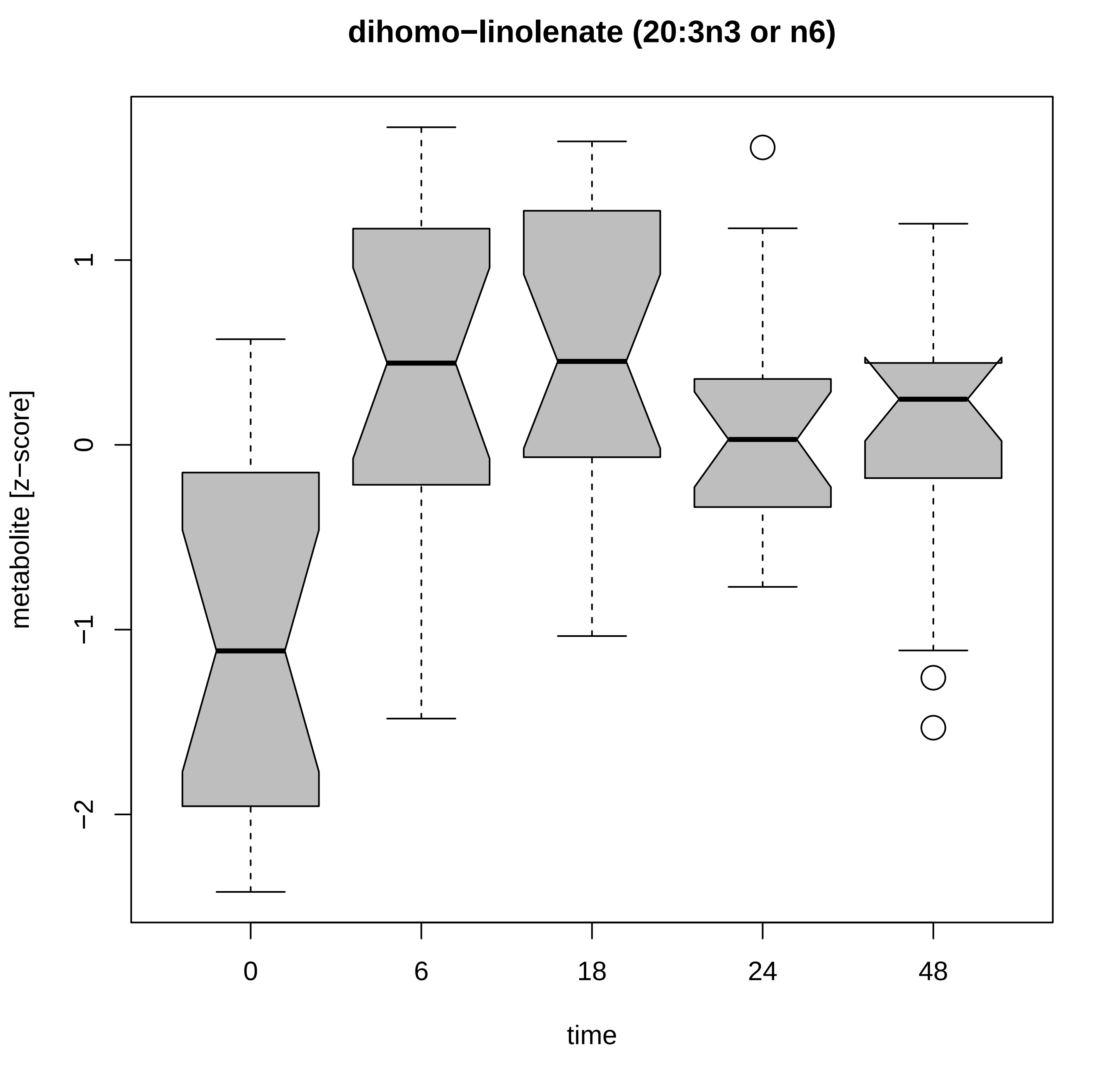

dihomo−linolenate (20:3n3 or n6)
metabolite [z−score]
−2
−1
0
1
0
6
18
24
48
time

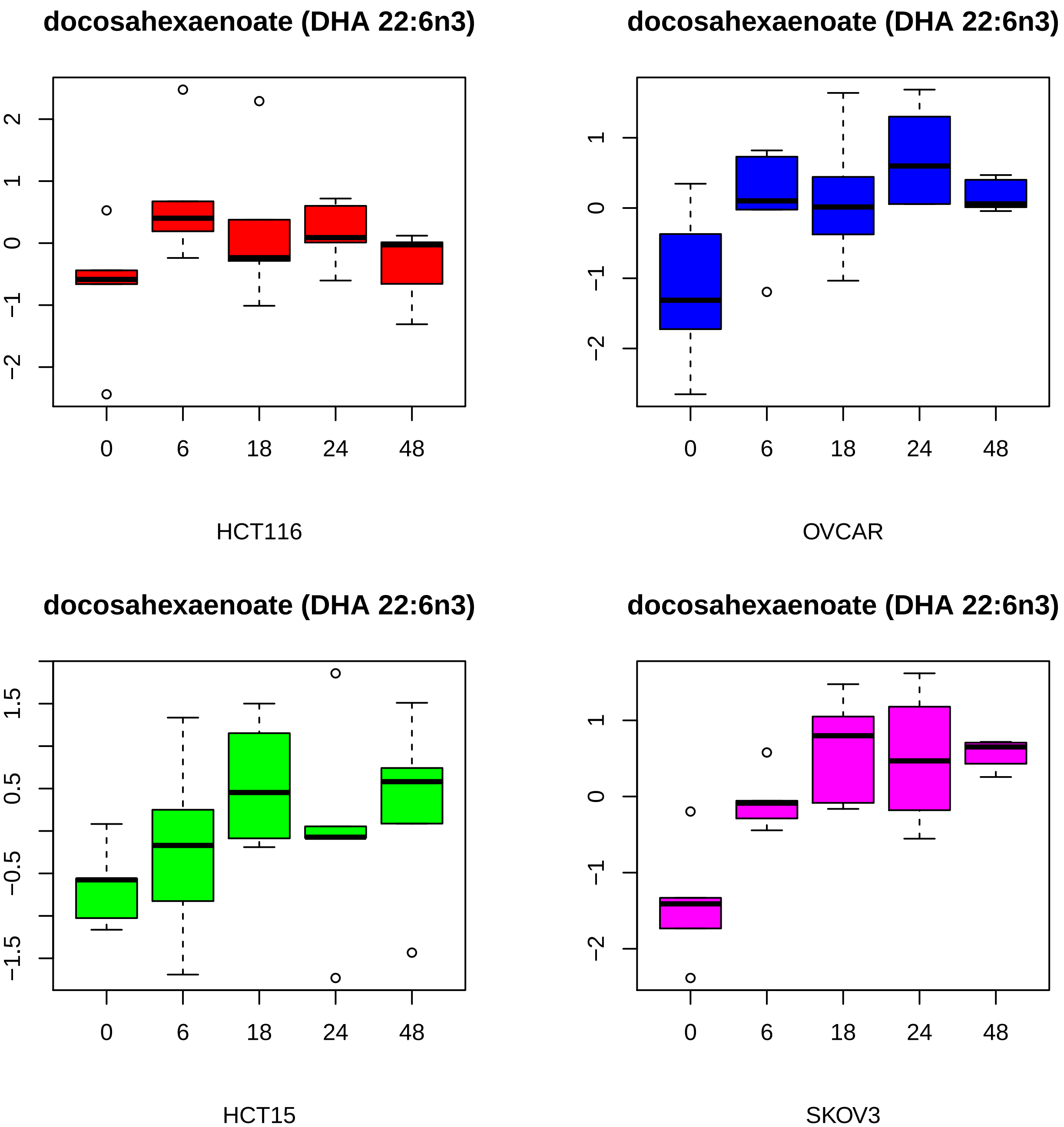
docosahexaenoate (DHA 22:6n3)
HCT116
OVCAR
HCT15
SKOV3
0
6
18
24
48
2
1
0
−1
−2
1.5
0.5
−0.5
−1.5

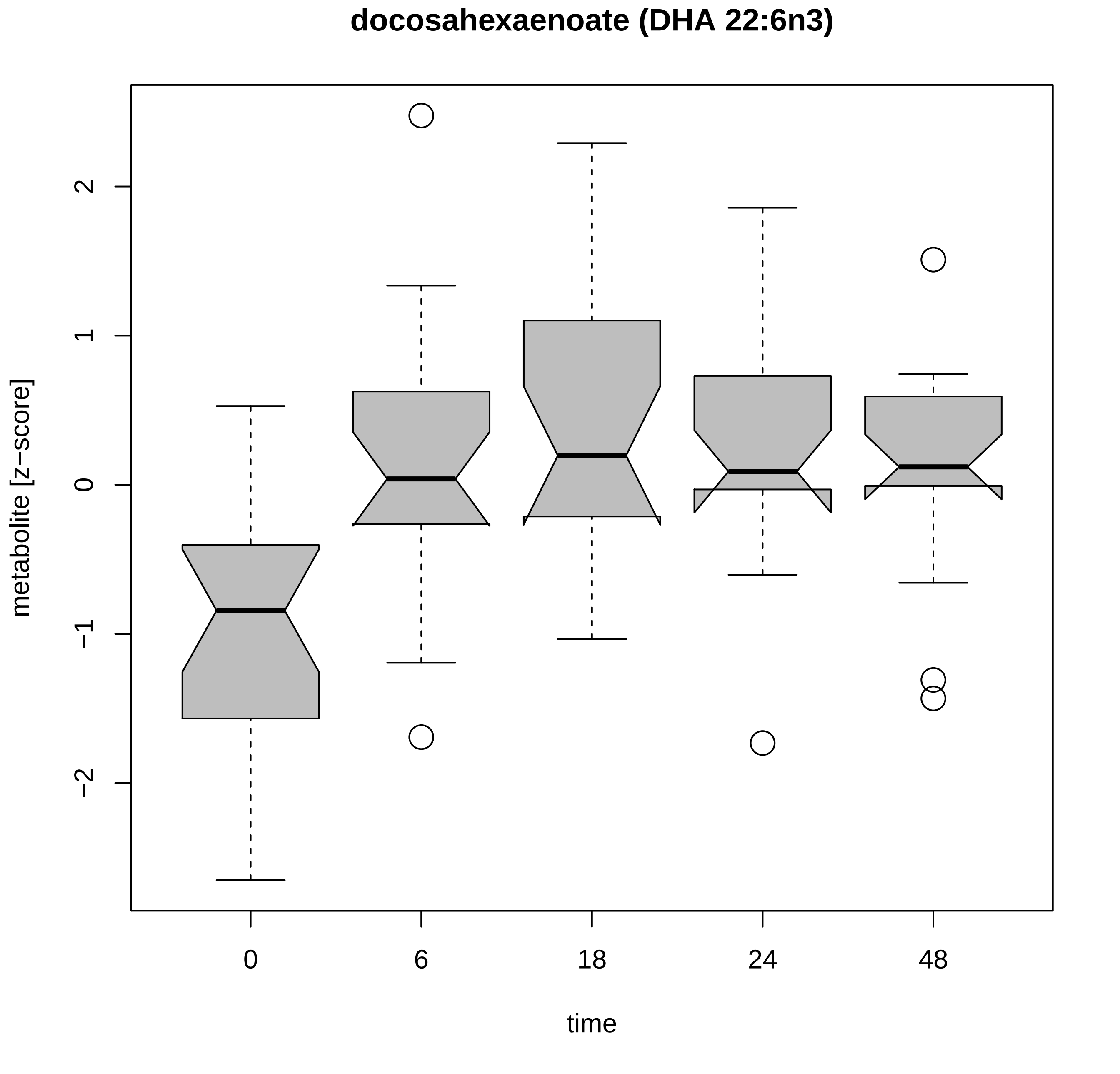
docosahexaenoate (DHA 22:6n3)
metabolite [z−score]
time
−2
−1
0
1
2
0
6
18
24
48

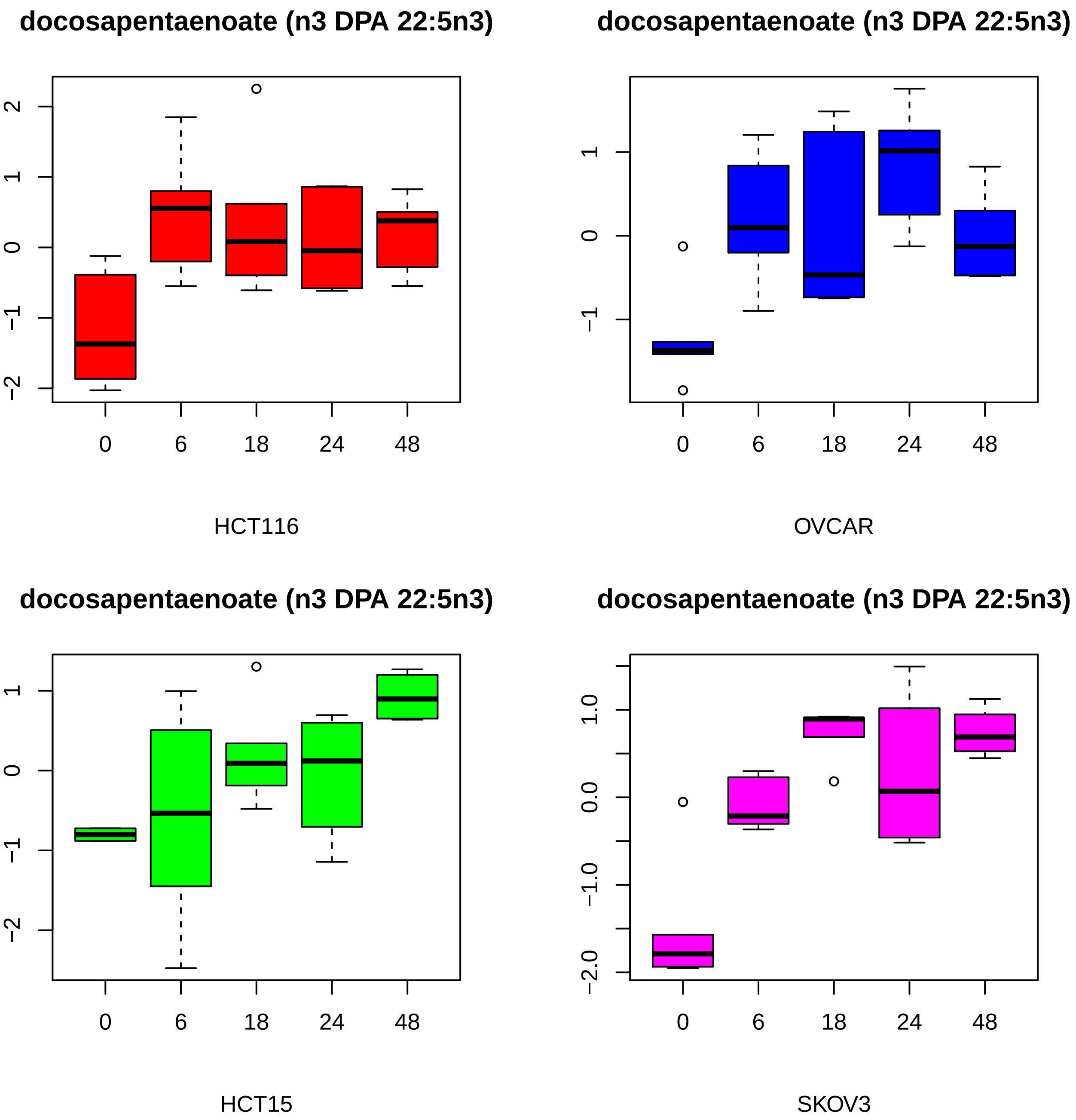
docosapentaenoate (n3 DPA 22:5n3)
HCT116
docosapentaenoate (n3 DPA 22:5n3)
OVCAR
docosapentaenoate (n3 DPA 22:5n3)
HCT15
docosapentaenoate (n3 DPA 22:5n3)
SKOV3
0
6
18
24
48

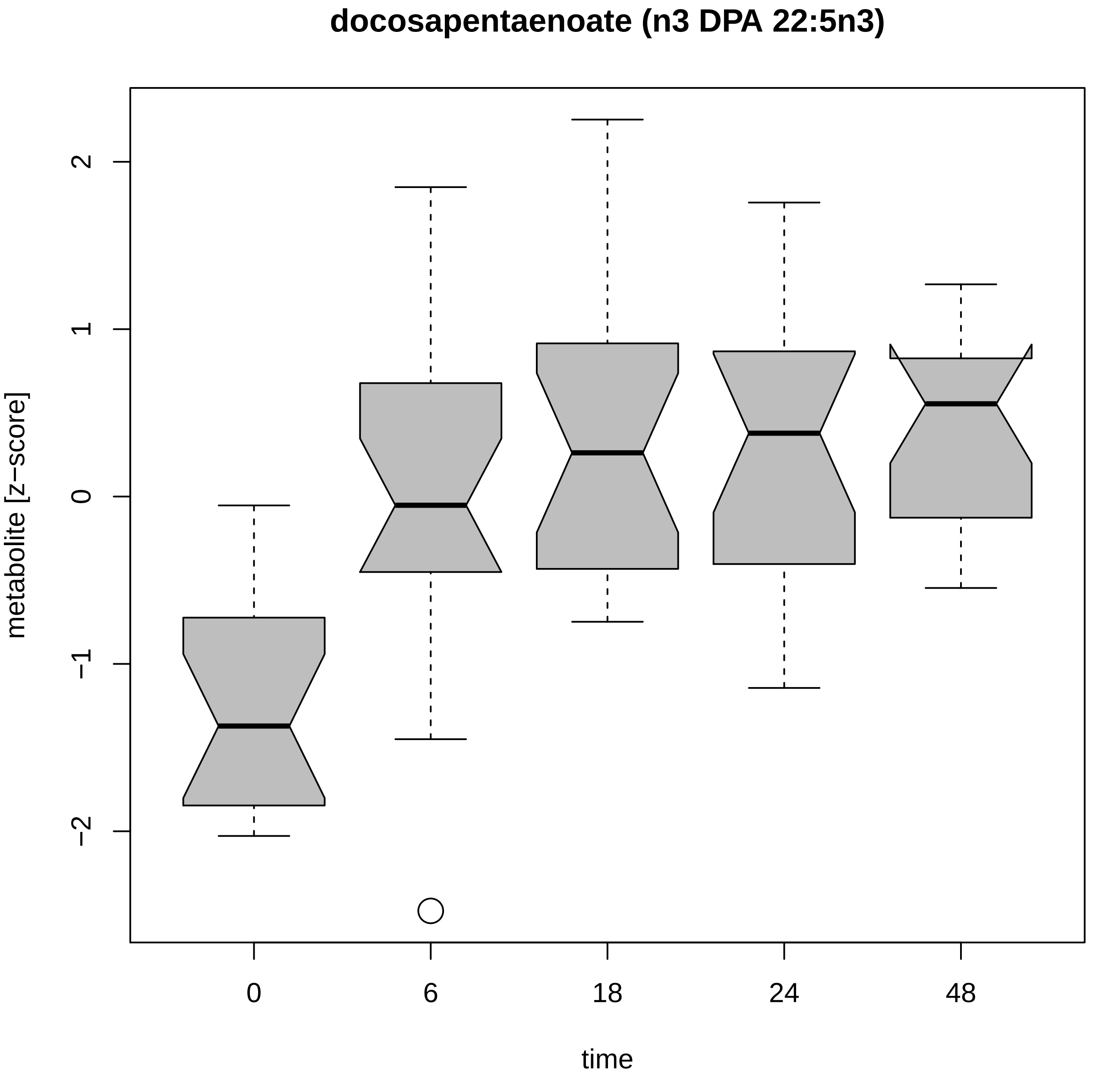
docosapentaenoate (n3 DPA 22:5n3)
metabolite [z−score]
2
1
0
−1
−2
0
6
18
24
48
time

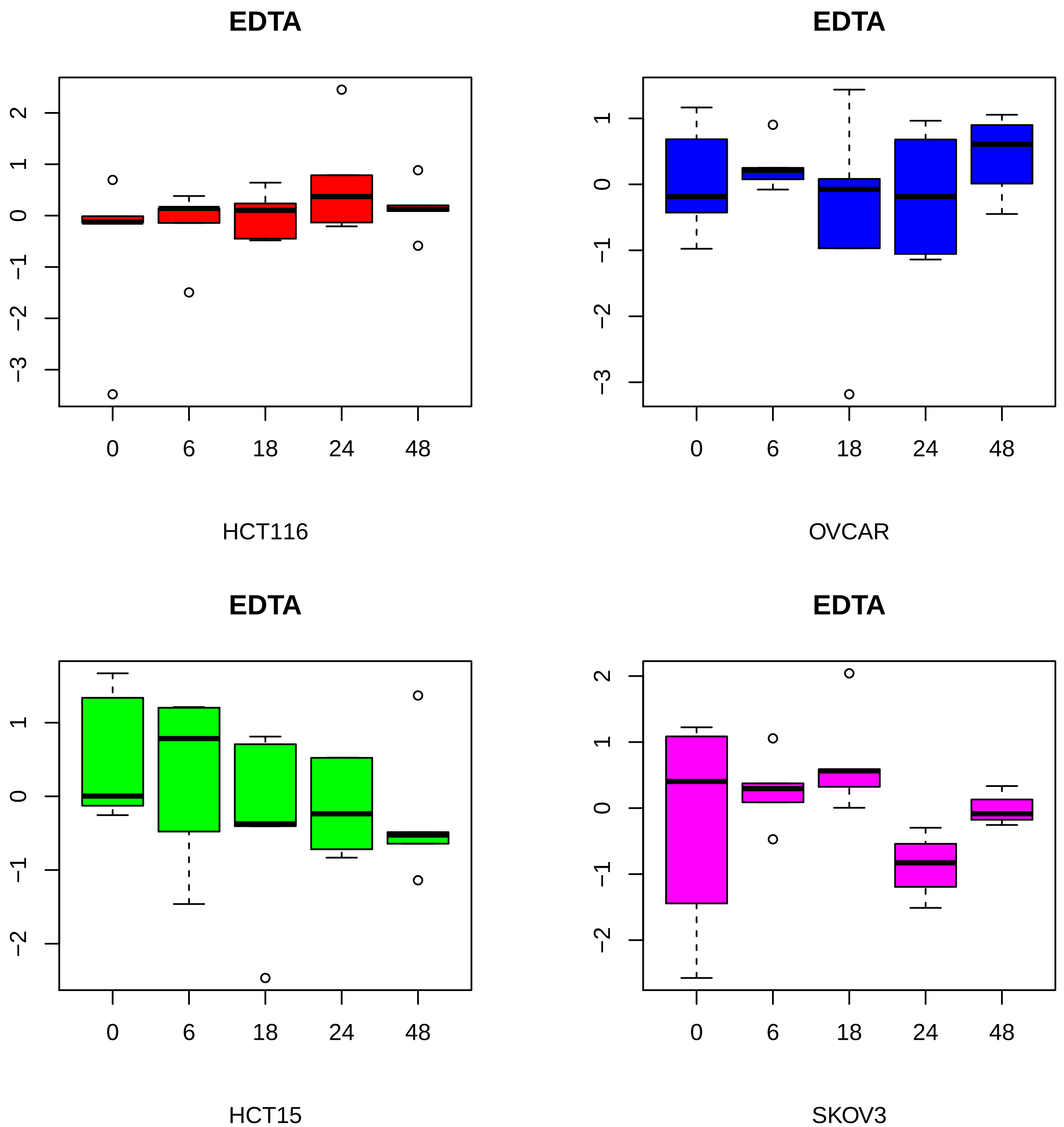

EDTA
HCT116
EDTA
OVCAR
EDTA
HCT15
EDTA
SKOV3
0
6
18
24
48

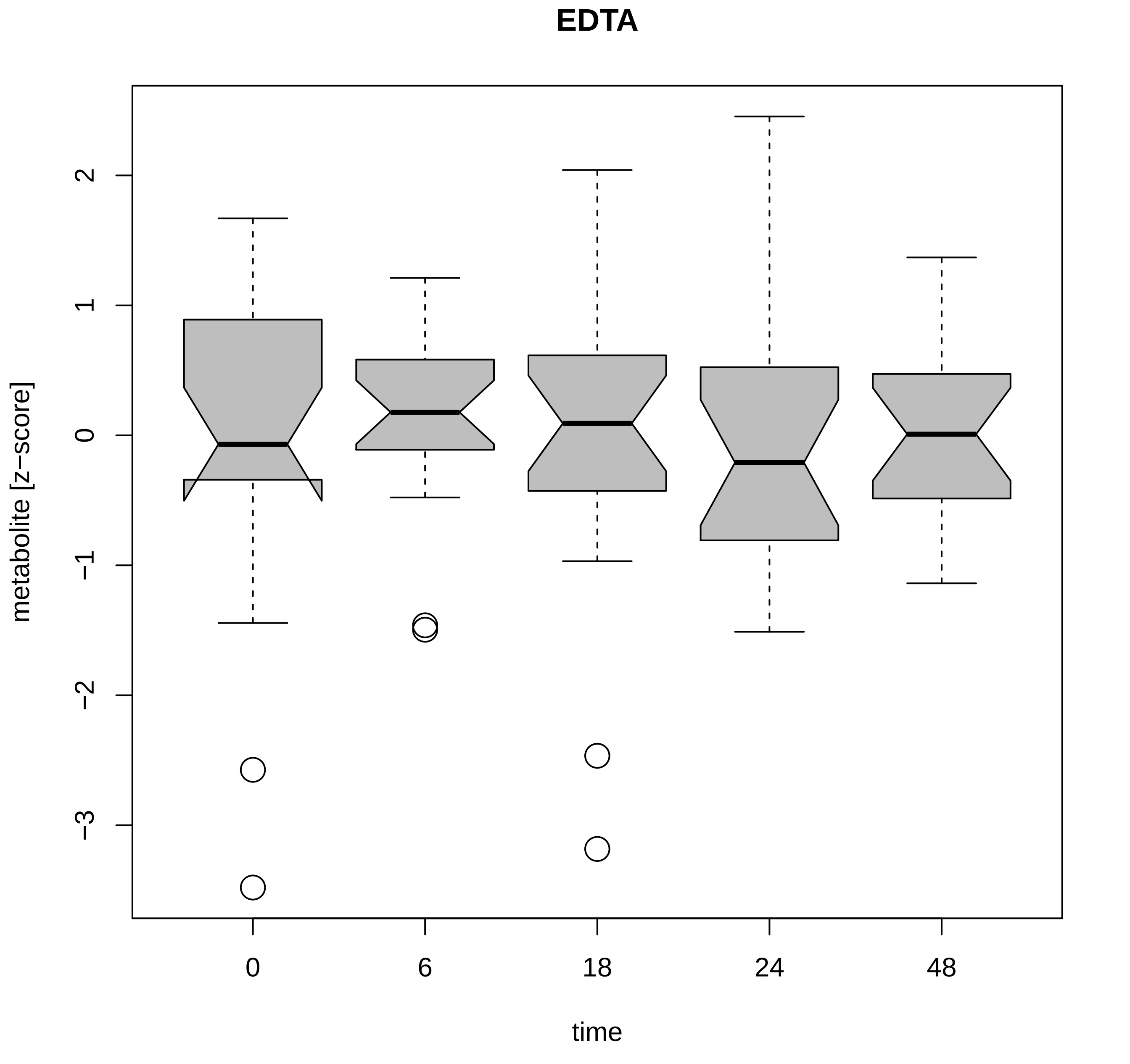
EDTA
metabolite [z−score]
2
1
0
−1
−2
−3
0
6
18
24
48
time

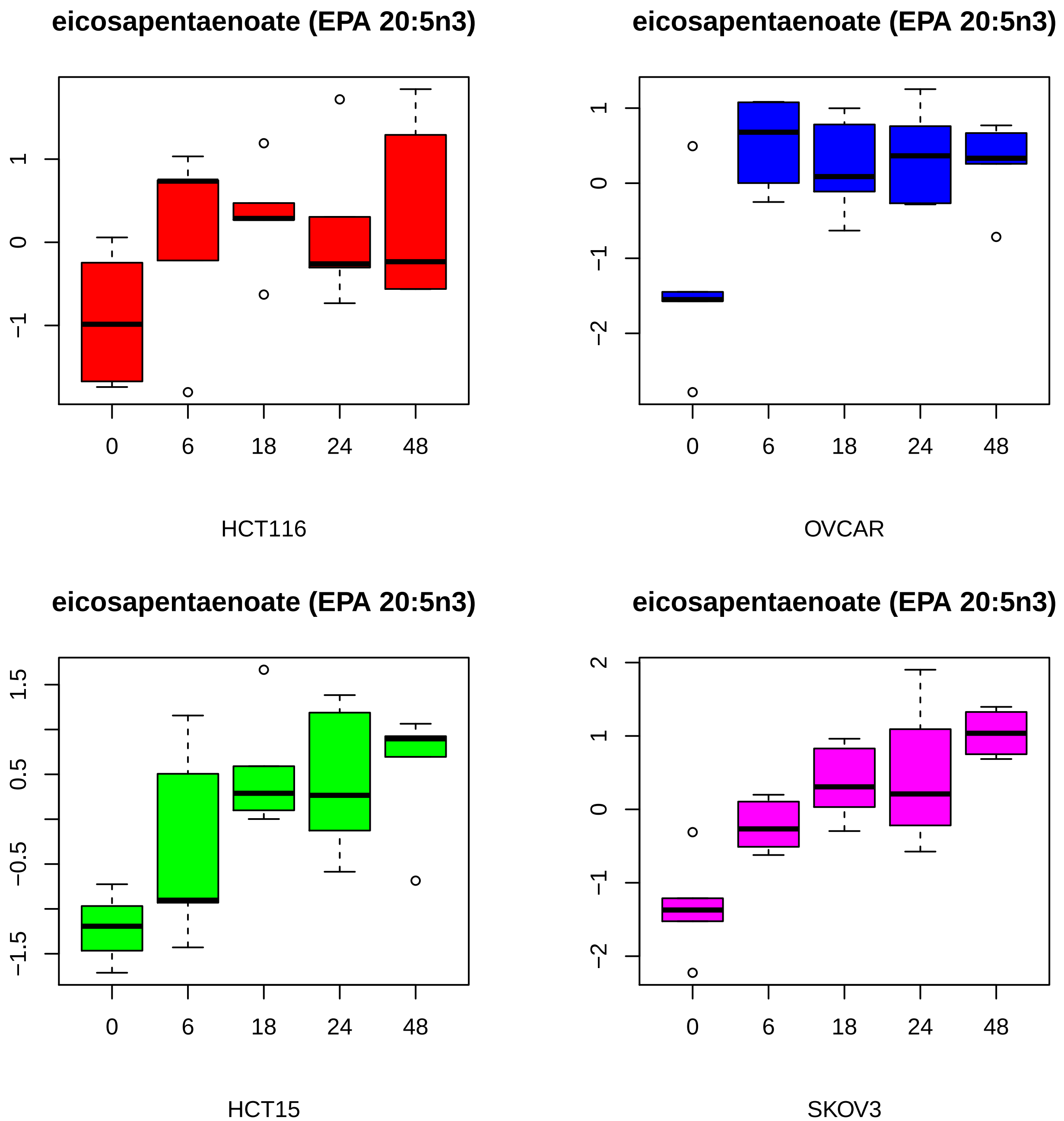

eicosapentaenoate (EPA 20:5n3)
HCT116
eicosapentaenoate (EPA 20:5n3)
OVCAR
eicosapentaenoate (EPA 20:5n3)
HCT15
eicosapentaenoate (EPA 20:5n3)
SKOV3
0
6
18
24
48

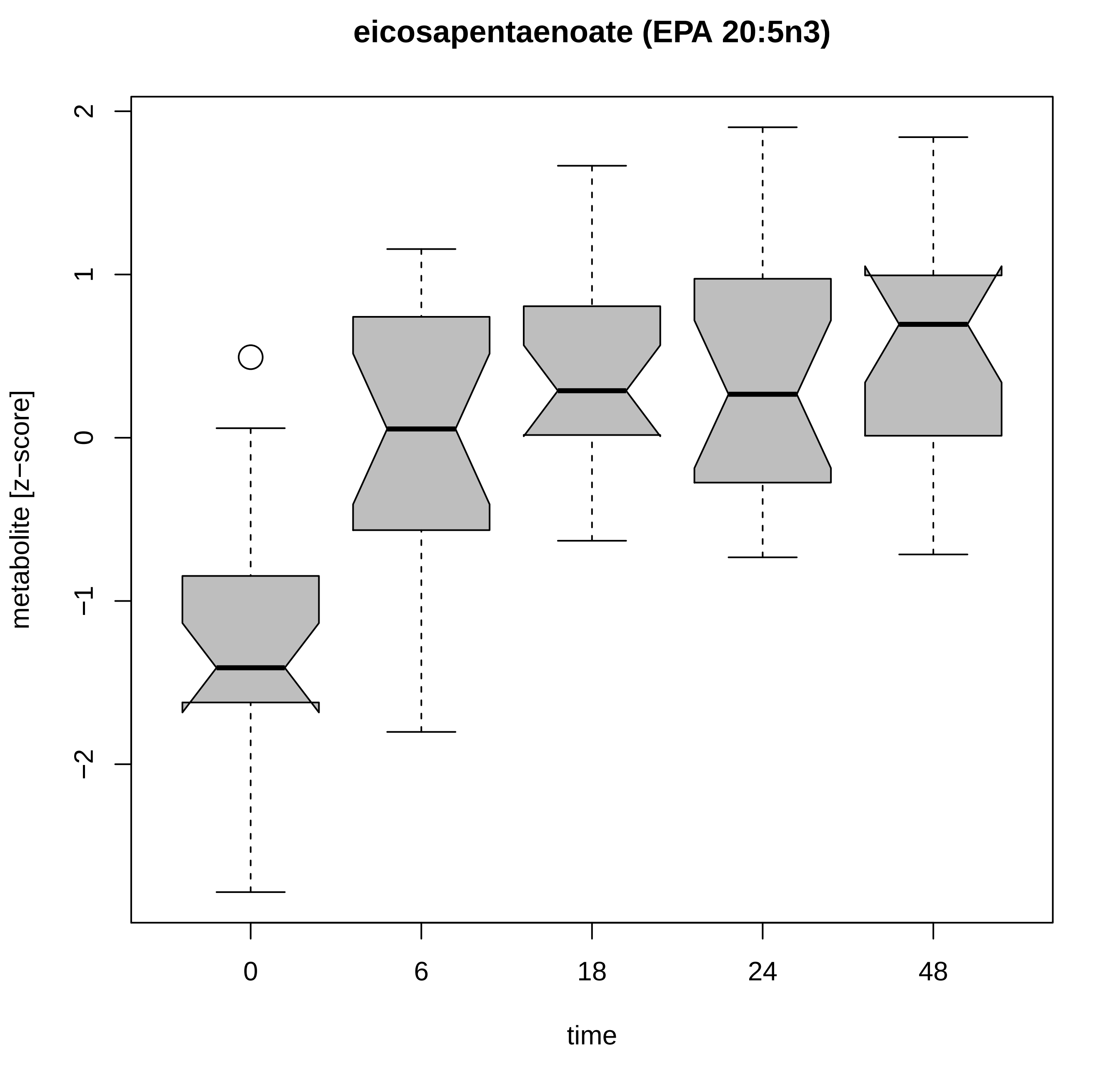
eicosapentaenoate (EPA 20:5n3)
metabolite [z−score]
2
1
0
−1
−2
0
6
18
24
48
time

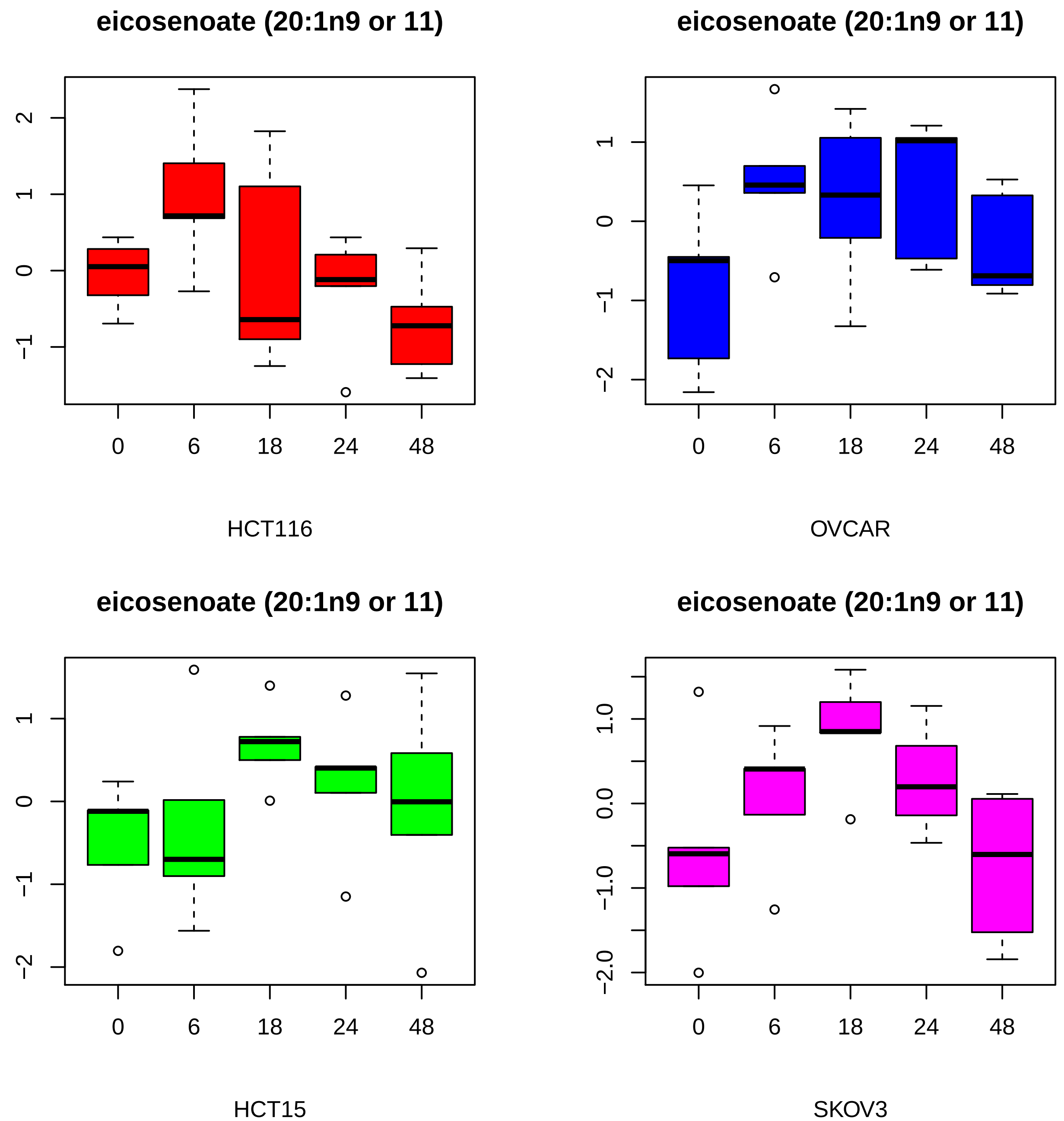
eicosenoate (20:1n9 or 11)
HCT116
eicosenoate (20:1n9 or 11)
OVCAR
eicosenoate (20:1n9 or 11)
HCT15
eicosenoate (20:1n9 or 11)
SKOV3
0
6
18
24
48

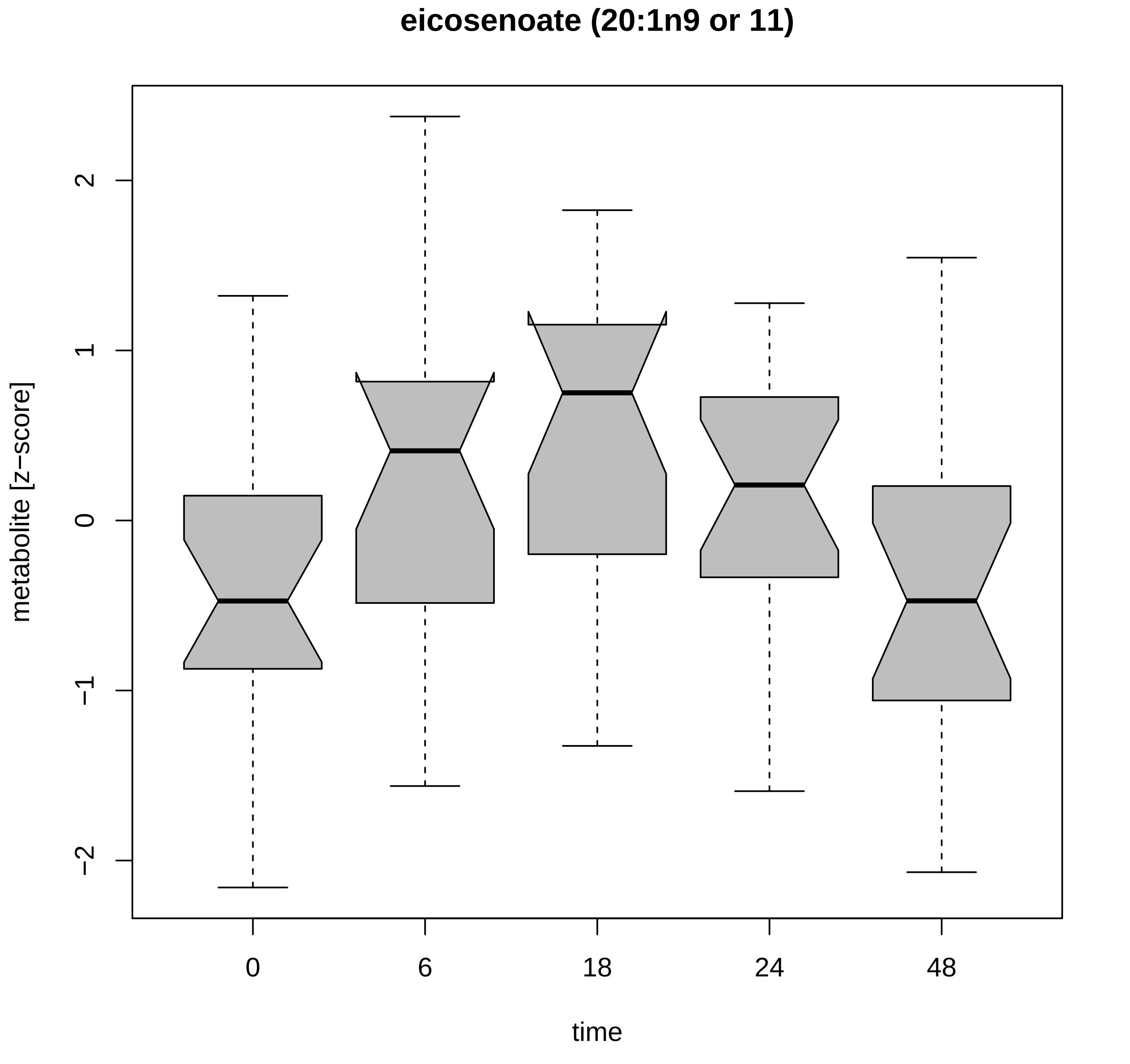

eicosenoate (20:1n9 or 11)
metabolite [z−score]
−2
−1
0
1
2
0
6
18
24
48
time

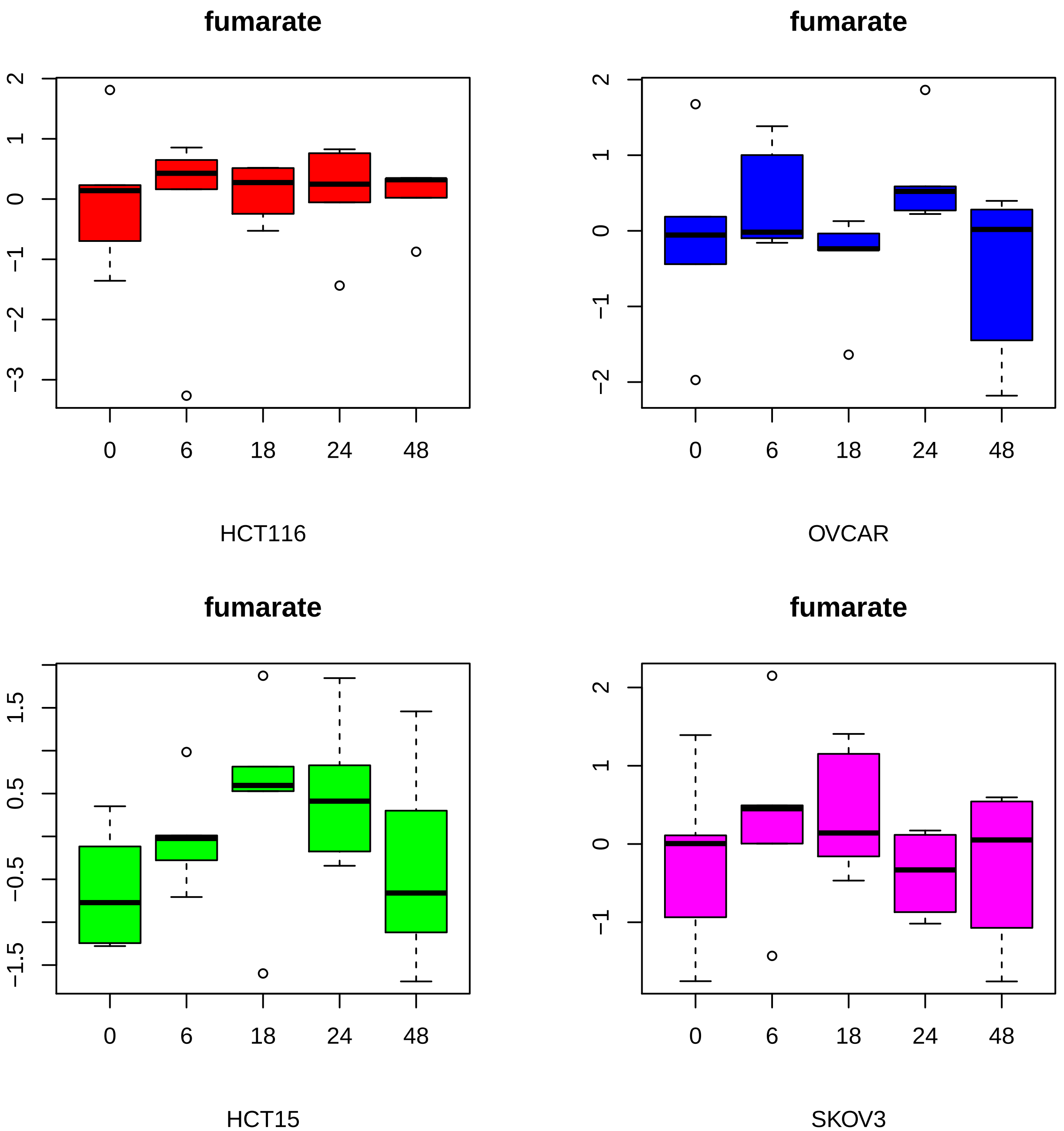

fumarate
HCT116
fumarate
OVCAR
fumarate
HCT15
fumarate
SKOV3
0
6
18
24
48

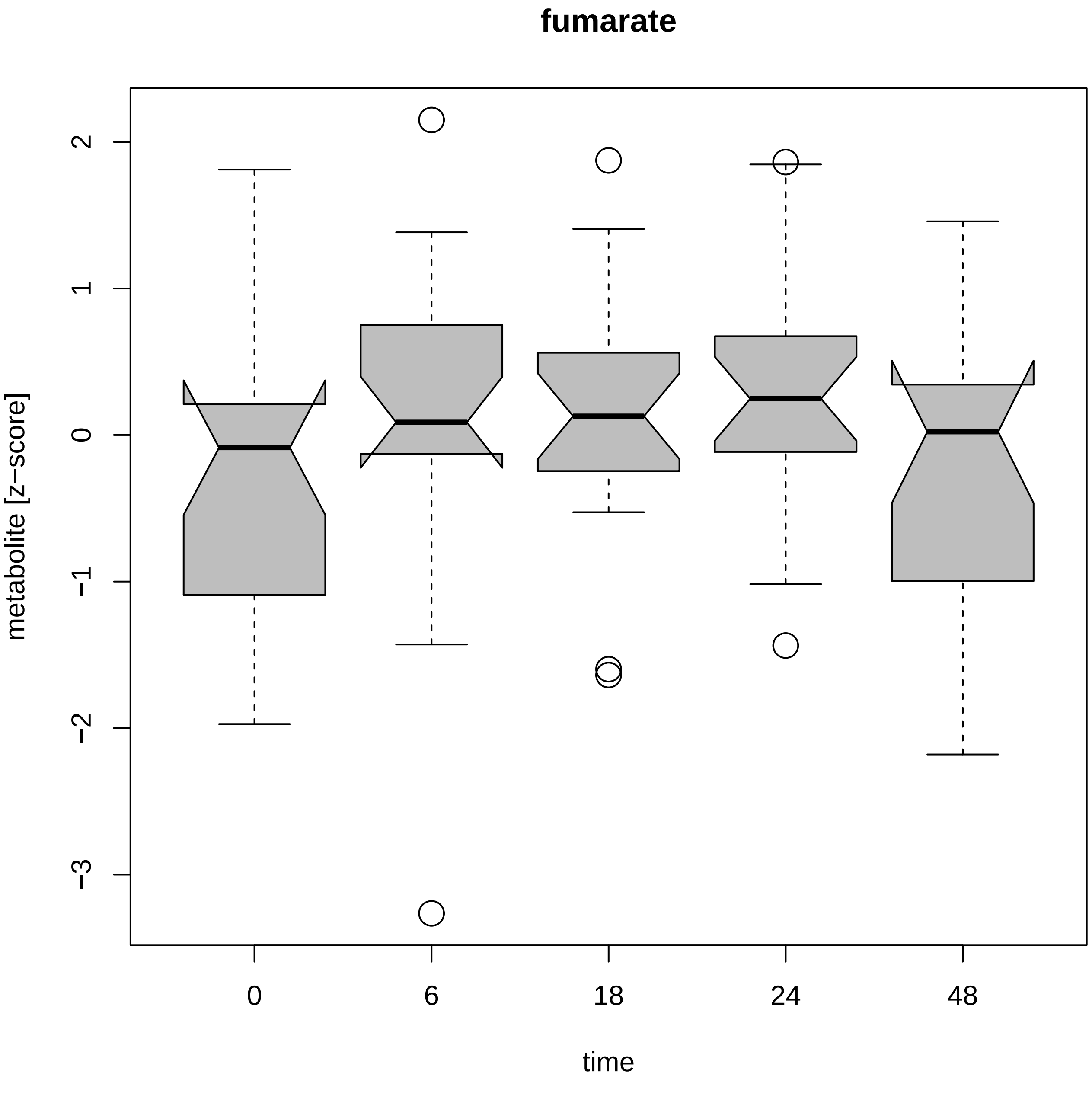
fumarate
metabolite [z−score]
time
0
6
18
24
48
2
1
0
−1
−2
−3

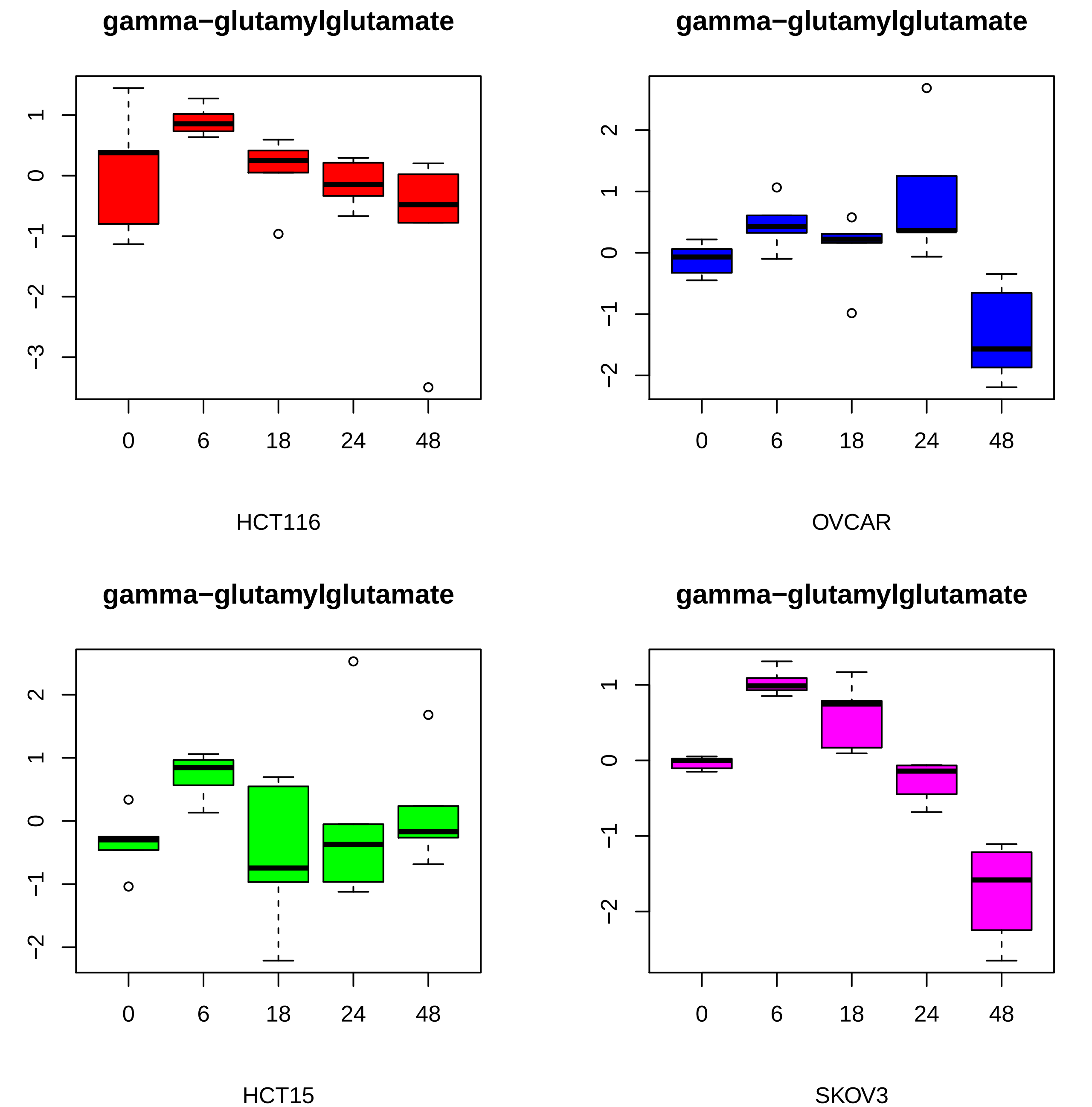
gamma−glutamylglutamate
HCT116
gamma−glutamylglutamate
OVCAR
gamma−glutamylglutamate
HCT15
gamma−glutamylglutamate
SKOV3
0
6
18
24
48

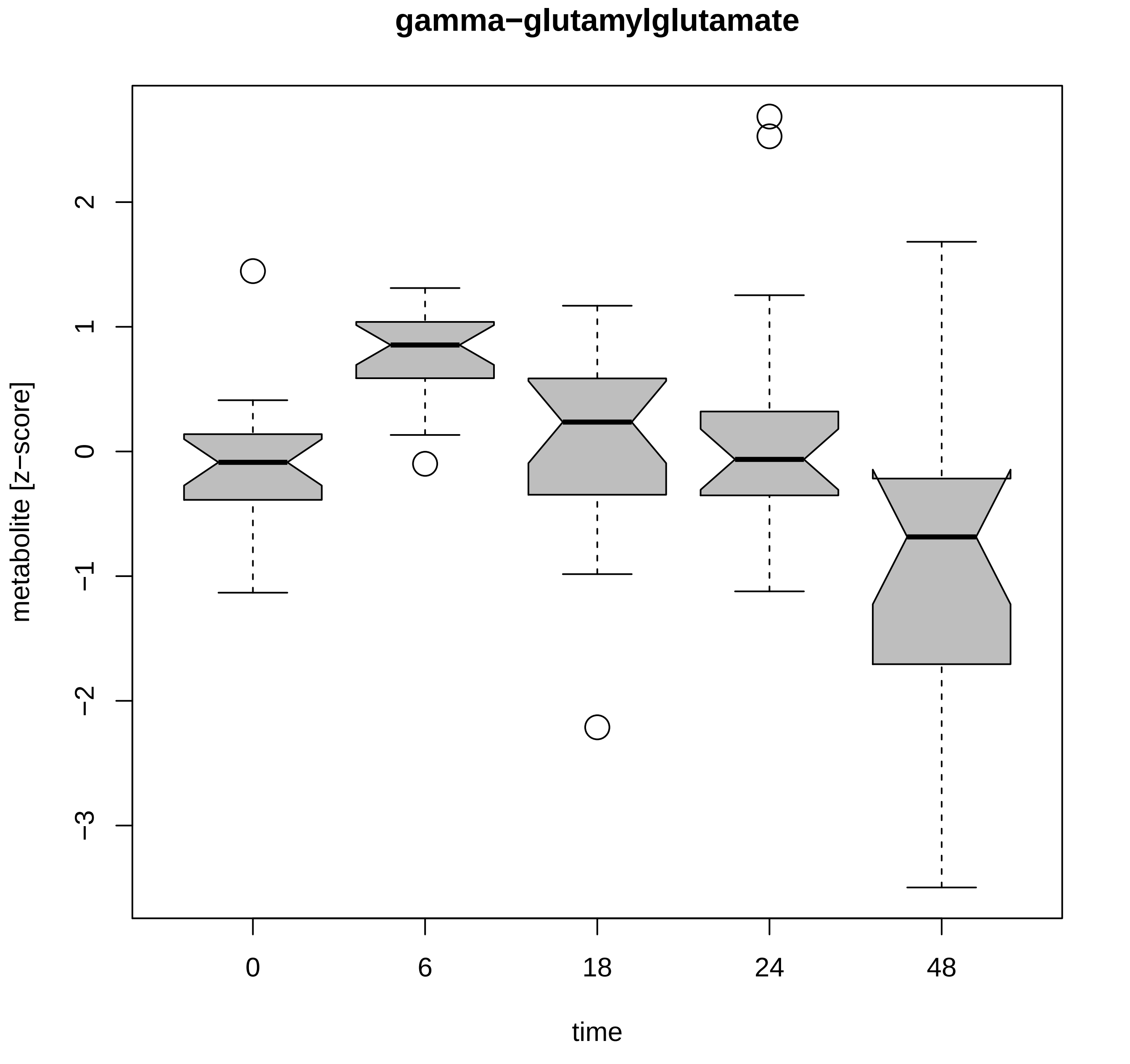
gamma−glutamylglutamate
metabolite [z−score]
2
1
0
−1
−2
−3
0
6
18
24
48
time

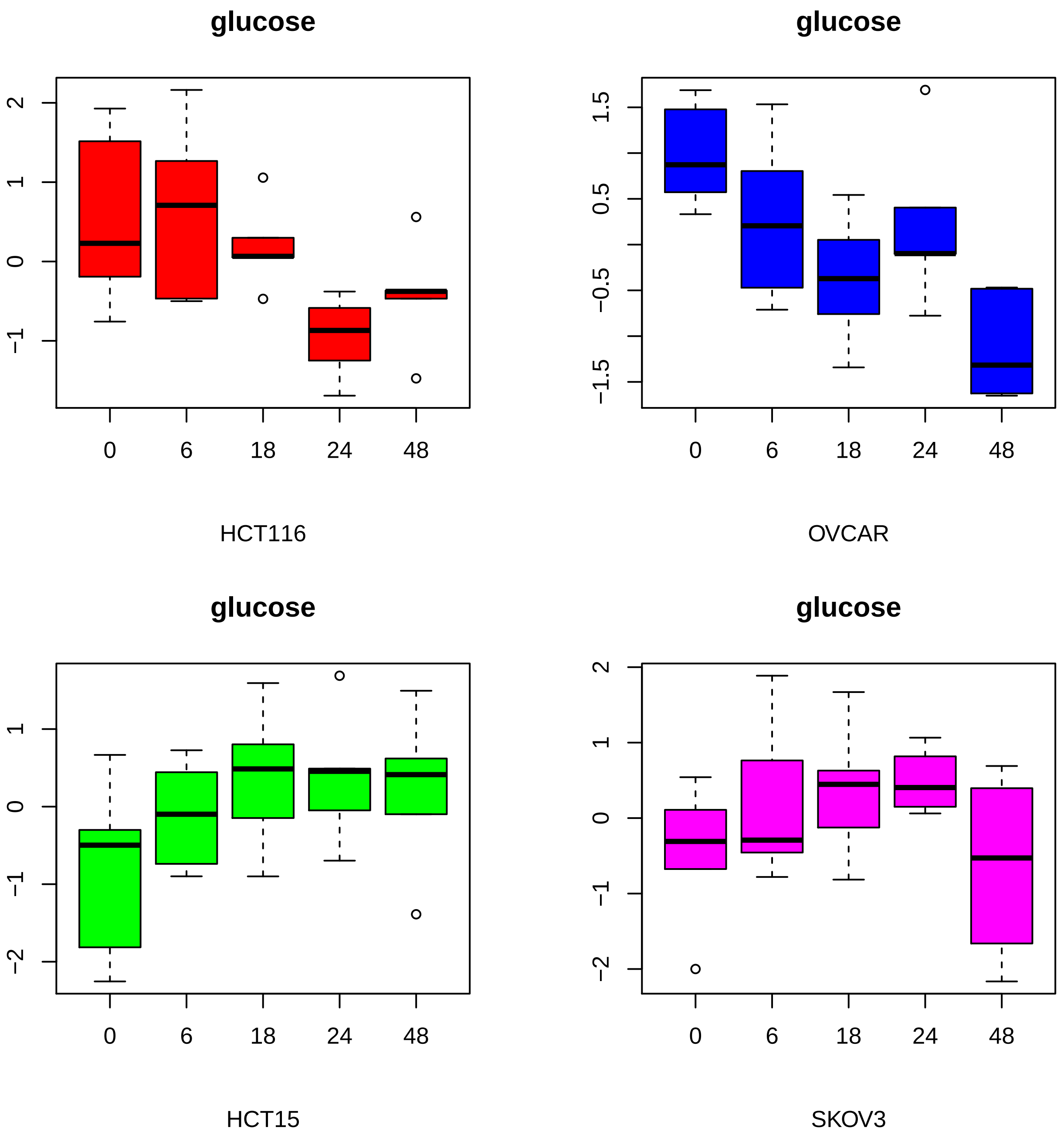
glucose
2
1
0
−1
0
6
18
24
48
HCT116
glucose
1.5
0.5
−0.5
−1.5
0
6
18
24
48
OVCAR
glucose
1
0
−1
−2
0
6
18
24
48
HCT15
glucose
2
1
0
−1
−2
0
6
18
24
48
SKOV3

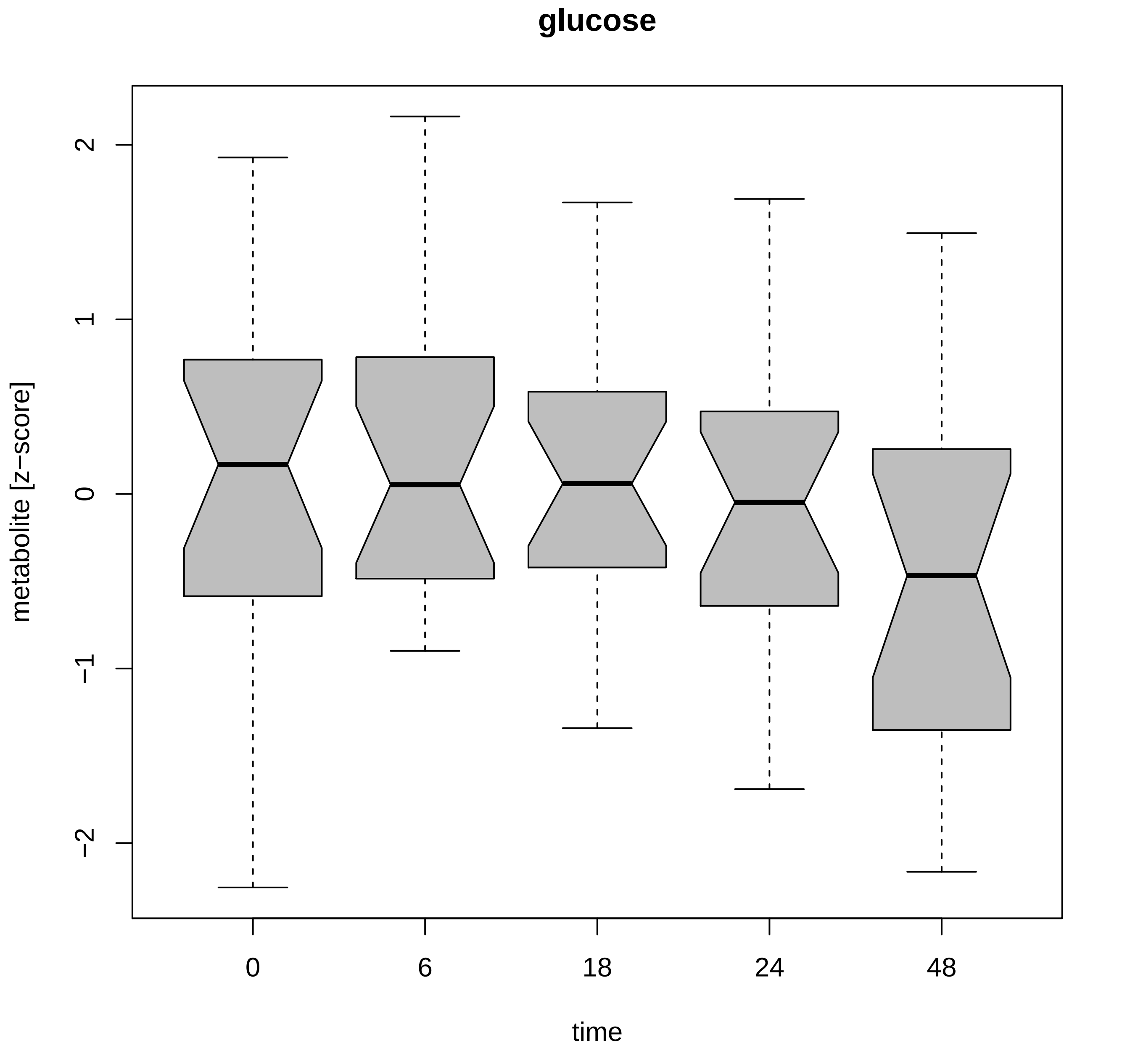
glucose
metabolite [z−score]
time
2
1
0
−1
−2
0
6
18
24
48

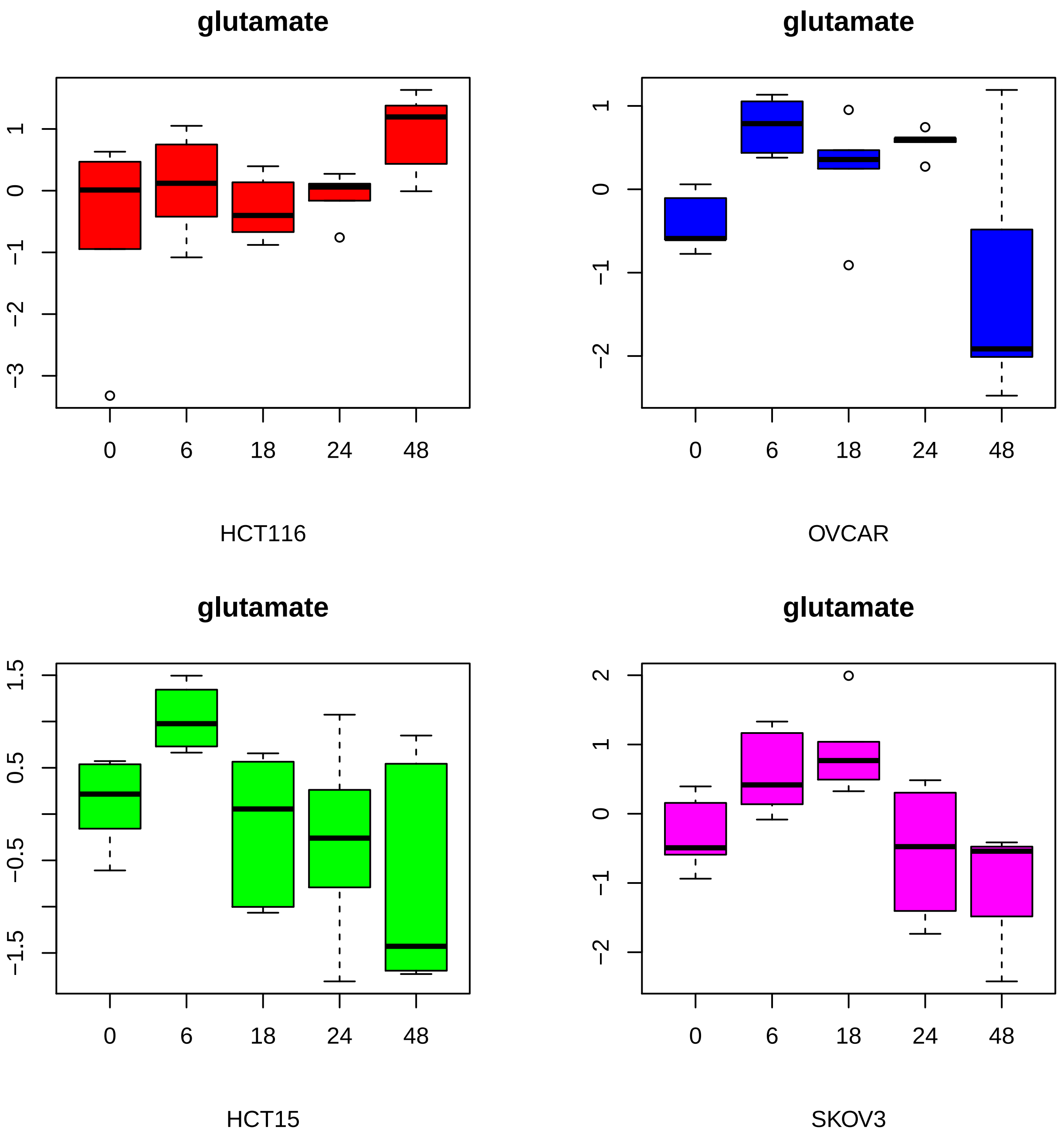
glutamate
1
0
−1
−2
−3
0
6
18
24
48
HCT116
glutamate
1
0
−1
−2
0
6
18
24
48
OVCAR
glutamate
1.5
0.5
−0.5
−1.5
0
6
18
24
48
HCT15
glutamate
2
1
0
−1
−2
0
6
18
24
48
SKOV3

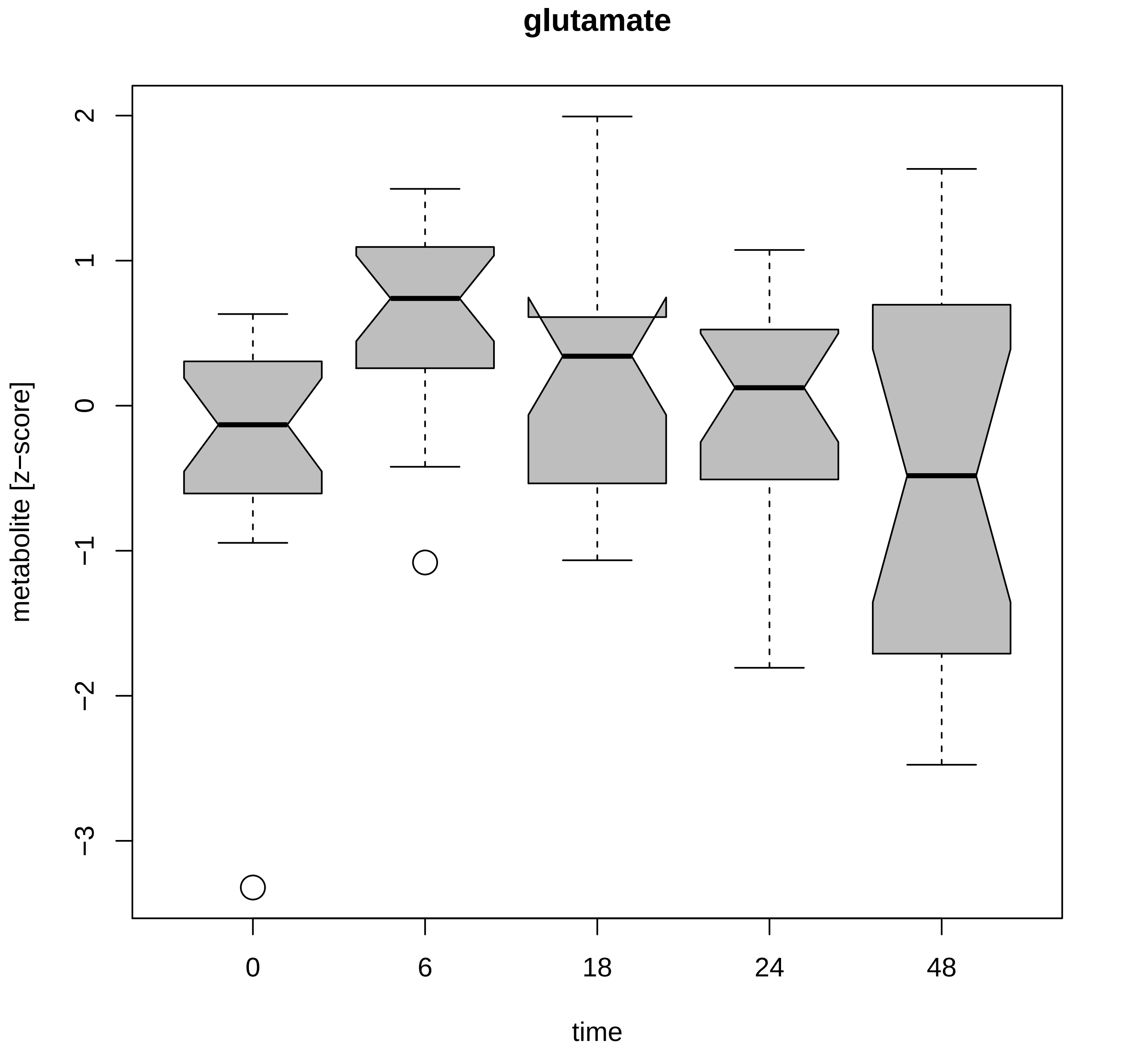
glutamate
metabolite [z−score]
2
1
0
−1
−2
−3
0
6
18
24
48
time

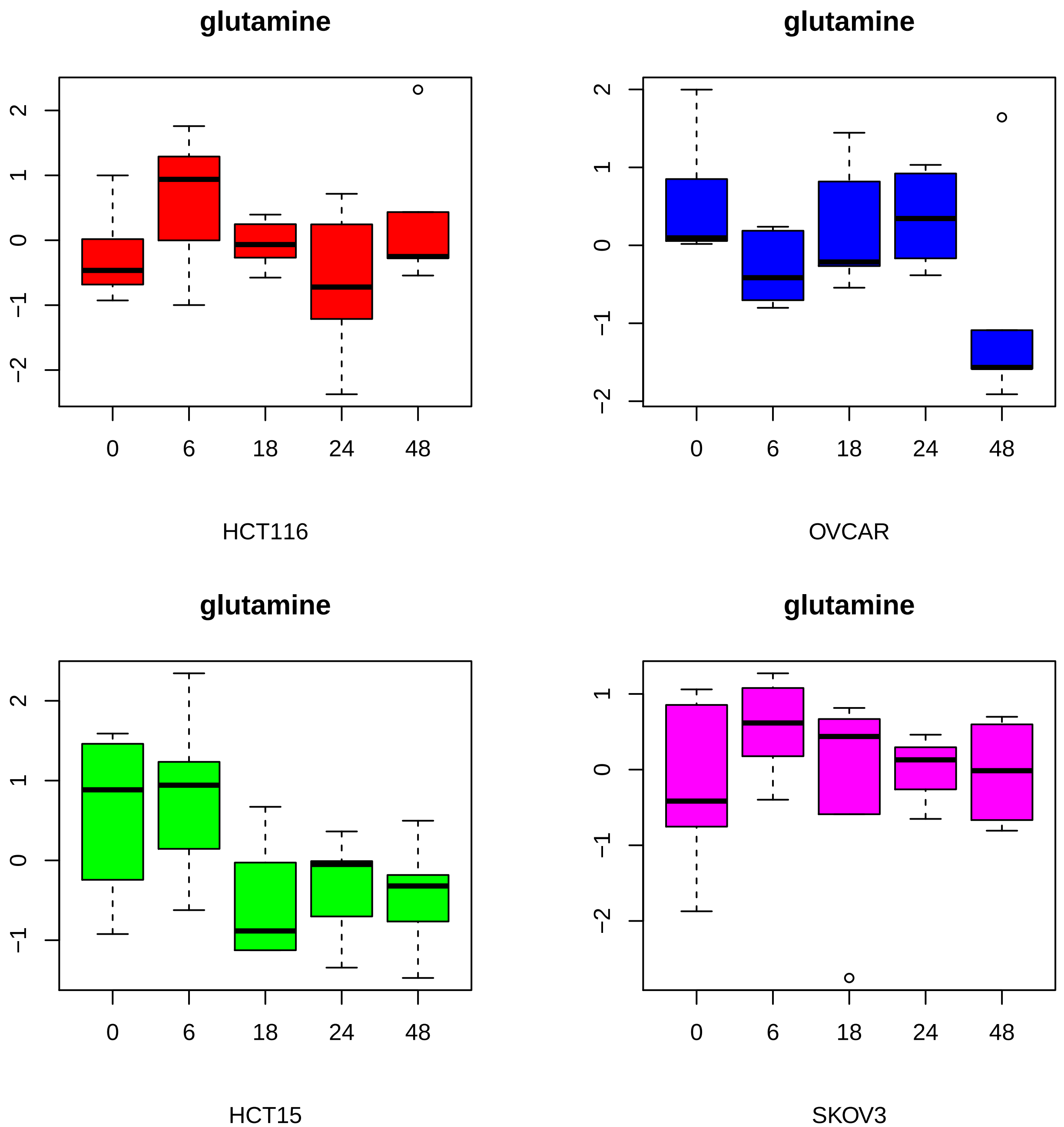
glutamine
HCT116
OVCAR
HCT15
SKOV3
0
6
18
24
48

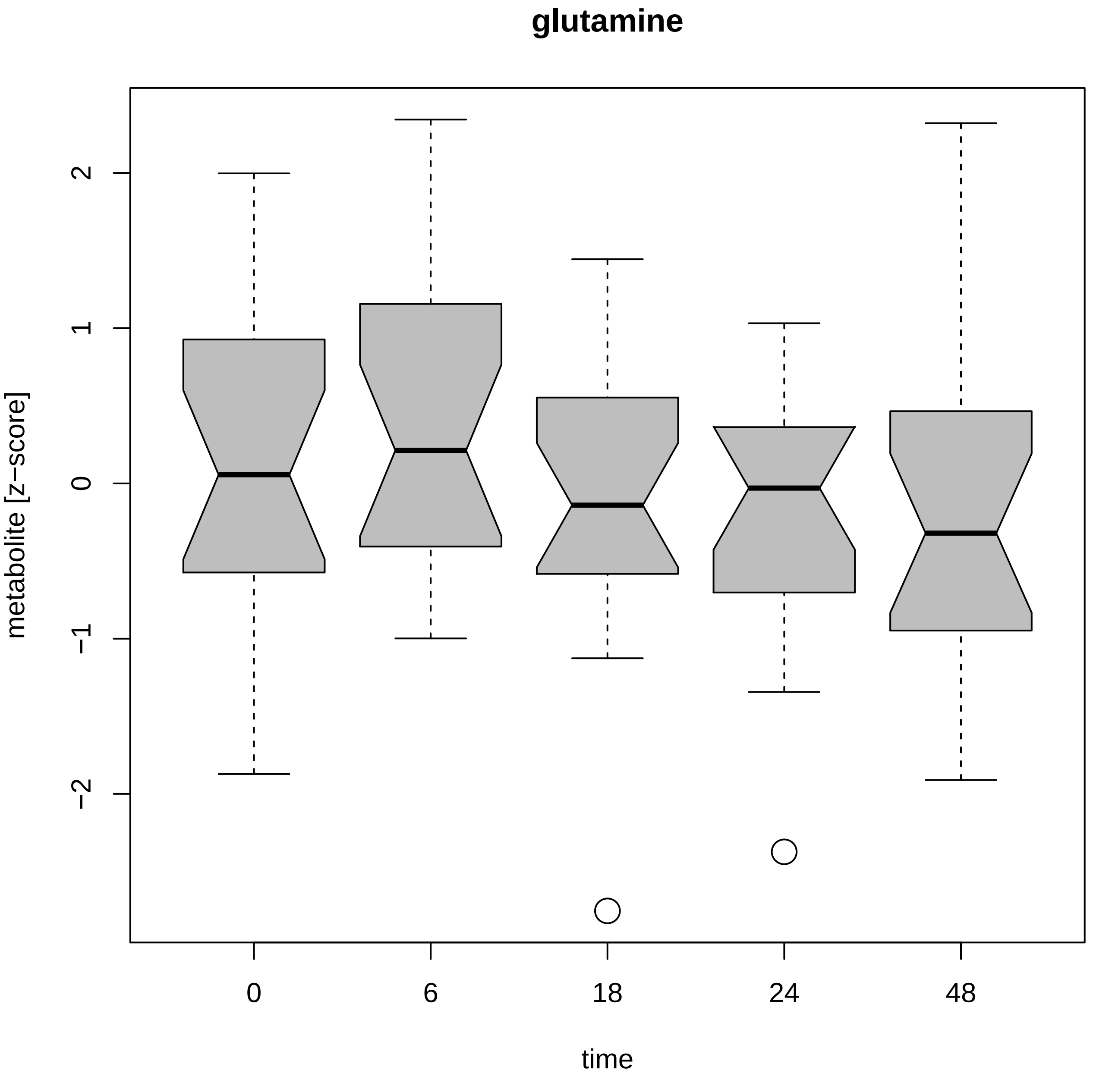
glutamine
metabolite [z−score]
time
2
1
0
−1
−2
0
6
18
24
48

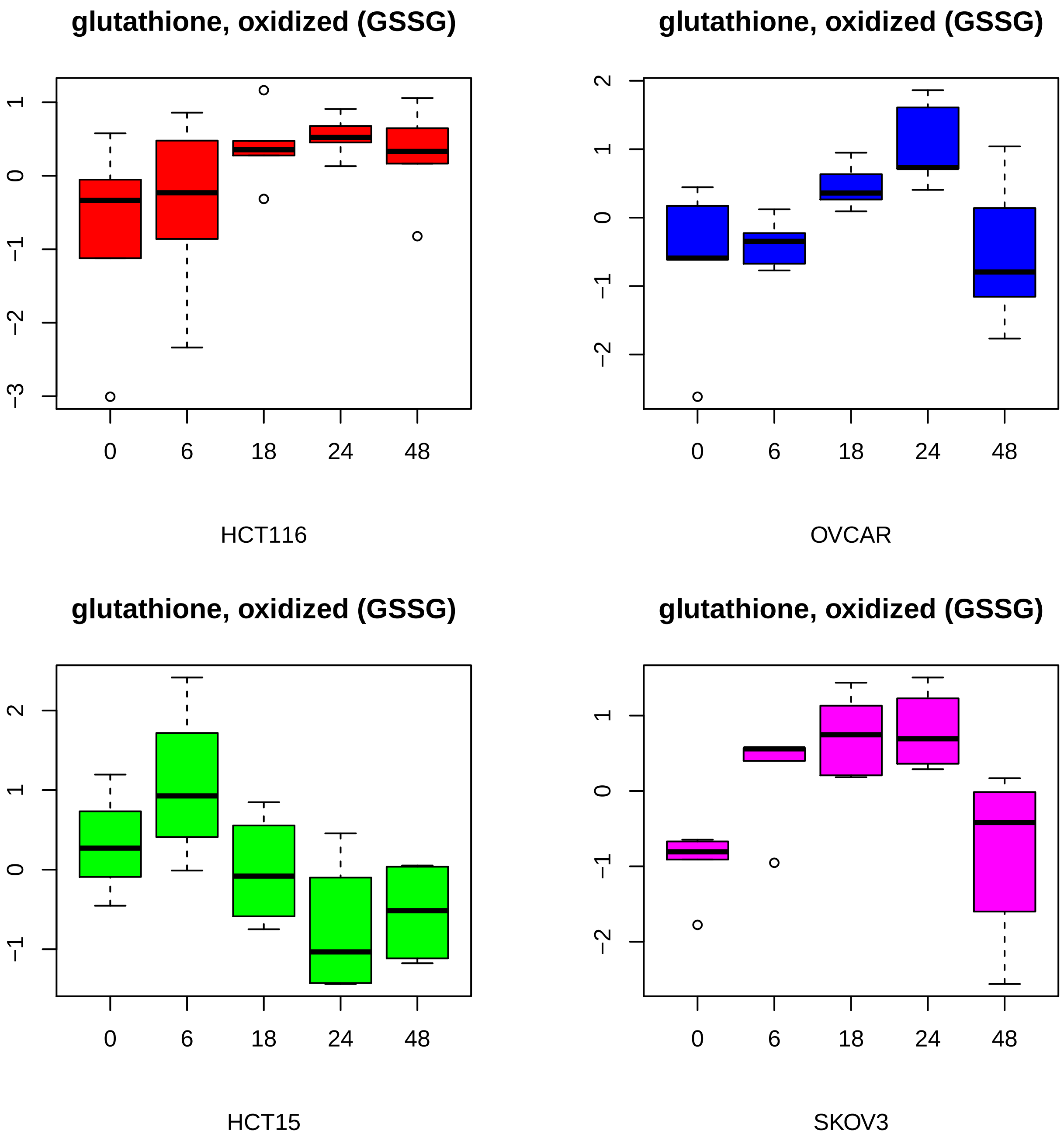
glutathione, oxidized (GSSG)
HCT116
glutathione, oxidized (GSSG)
OVCAR
glutathione, oxidized (GSSG)
HCT15
glutathione, oxidized (GSSG)
SKOV3
0
6
18
24
48

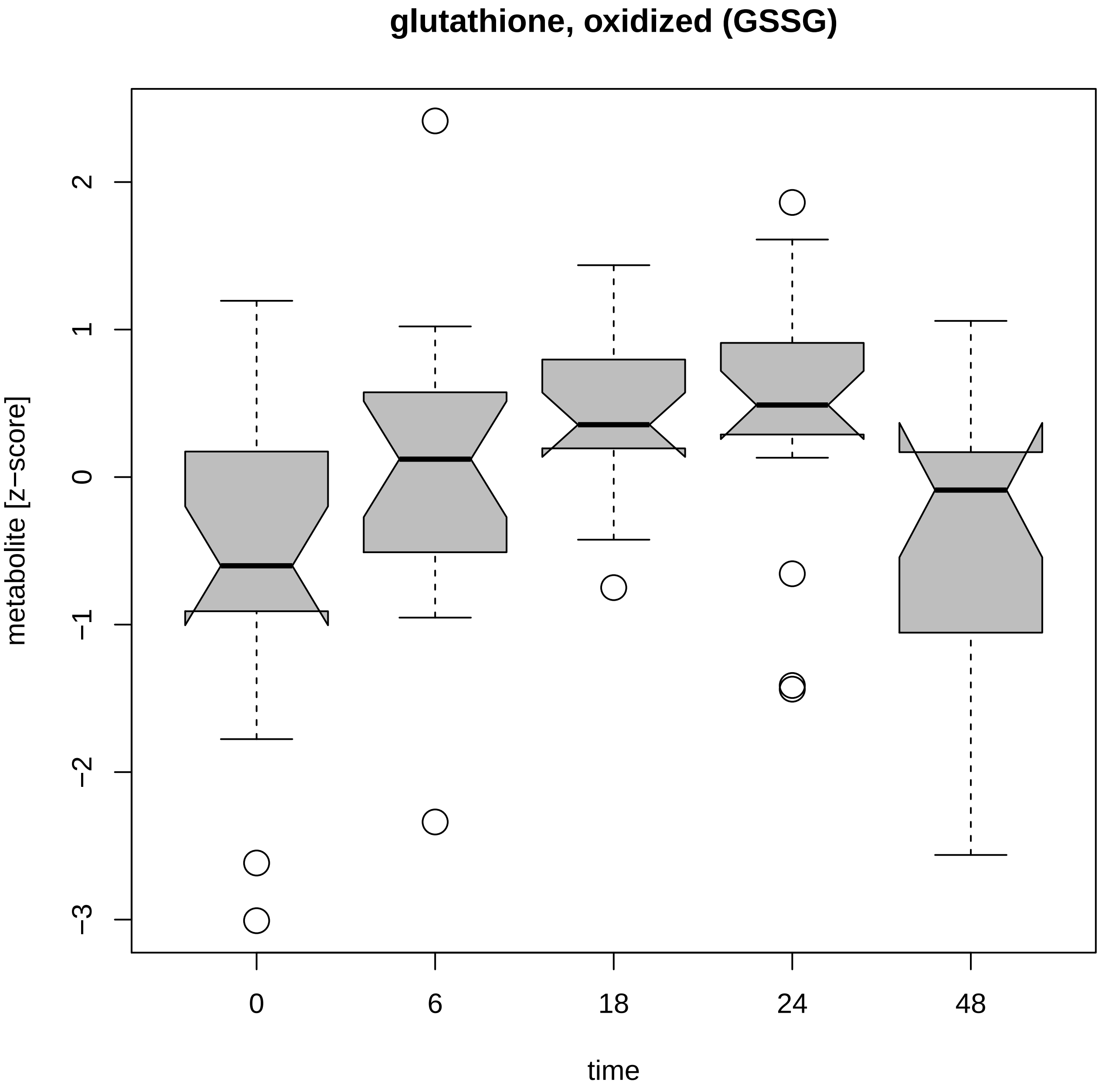
glutathione, oxidized (GSSG)
metabolite [z−score]
2
1
0
−1
−2
−3
0
6
18
24
48
time

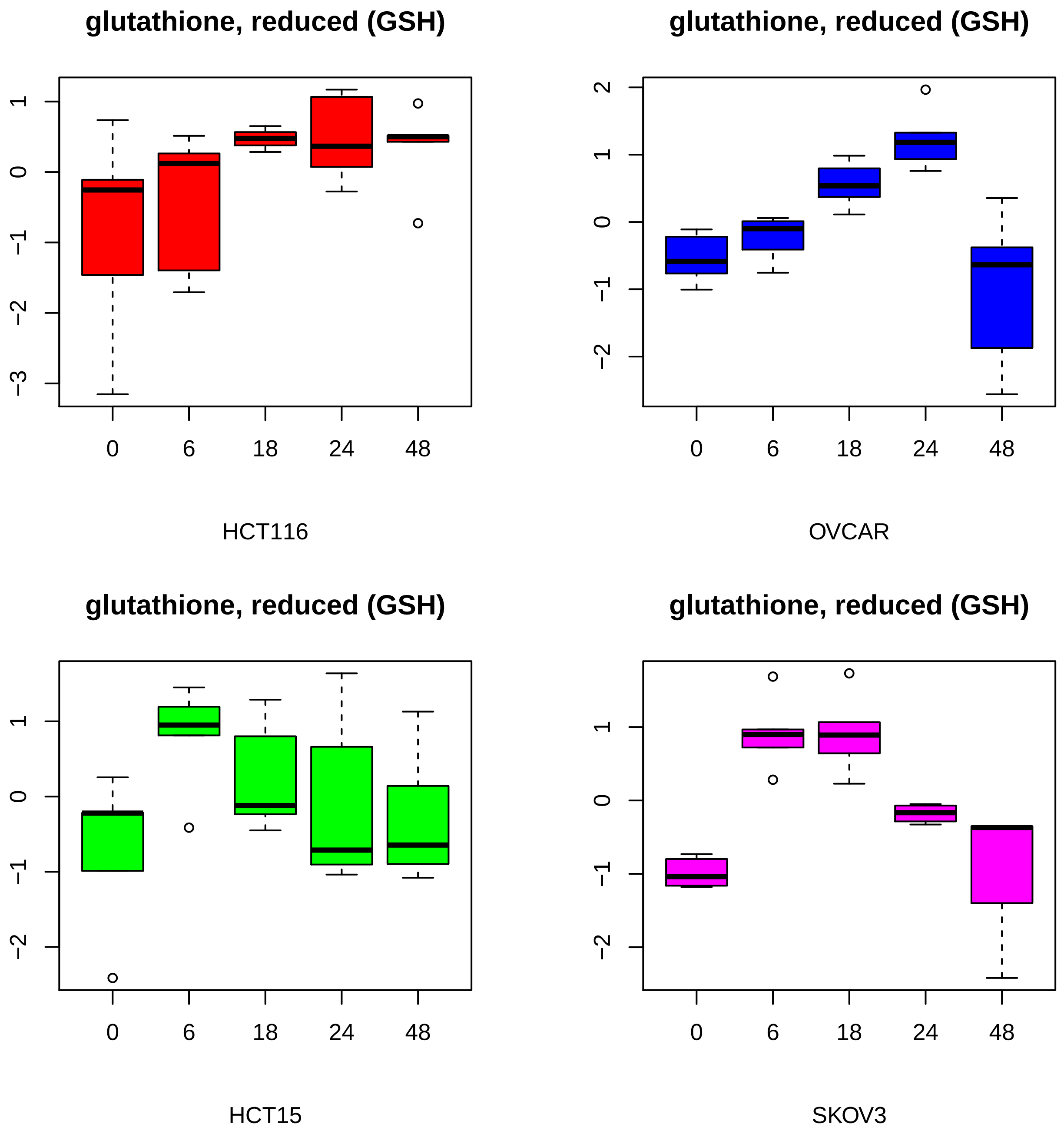

glutathione, reduced (GSH)
1
0
−1
−2
−3
0
6
18
24
48
HCT116
glutathione, reduced (GSH)
2
1
0
−1
−2
0
6
18
24
48
OVCAR
glutathione, reduced (GSH)
1
0
−1
−2
0
6
18
24
48
HCT15
glutathione, reduced (GSH)
1
0
−1
−2
0
6
18
24
48
SKOV3

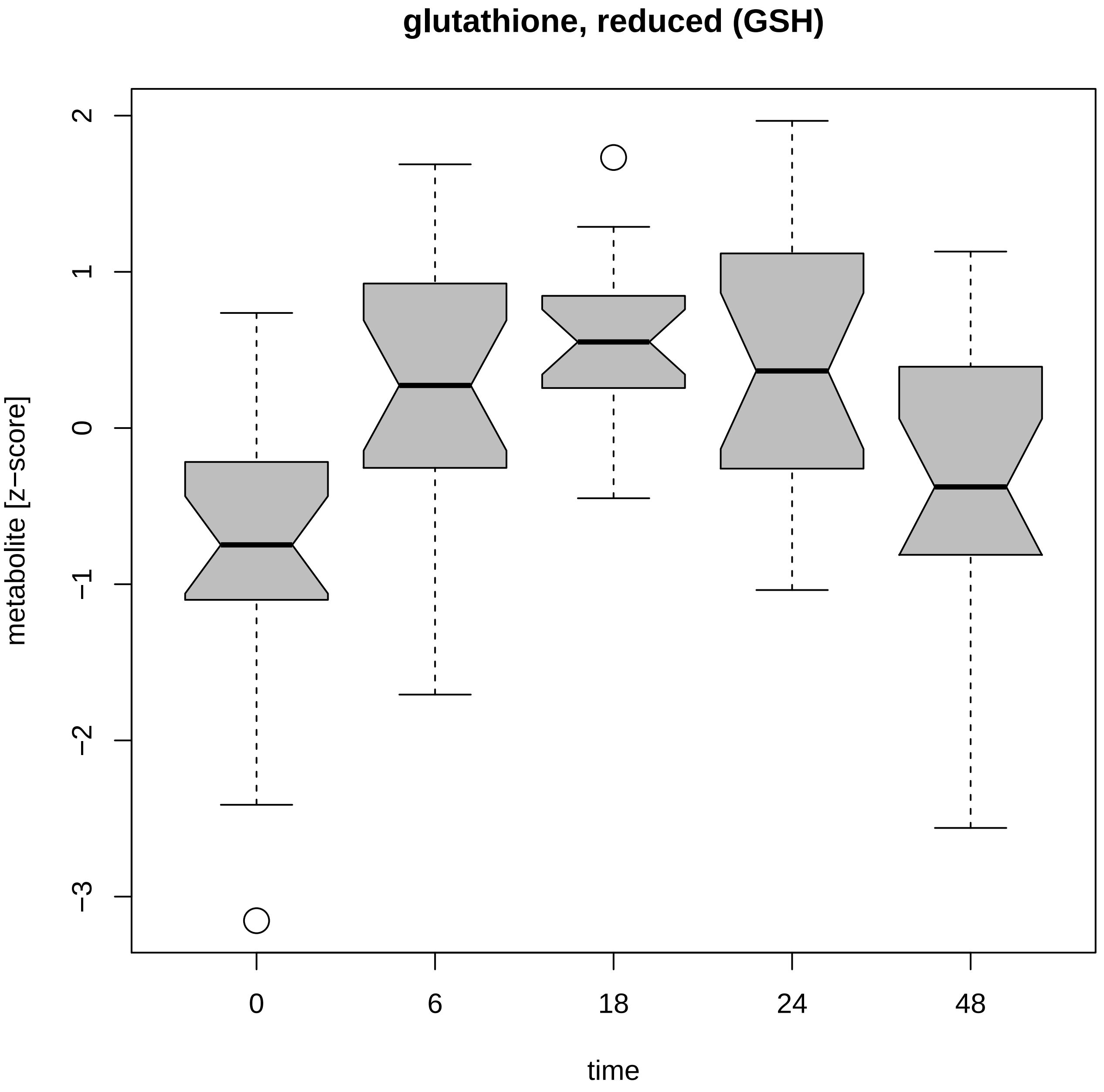
glutathione, reduced (GSH)
metabolite [z−score]
2
1
0
−1
−2
−3
0
6
18
24
48
time

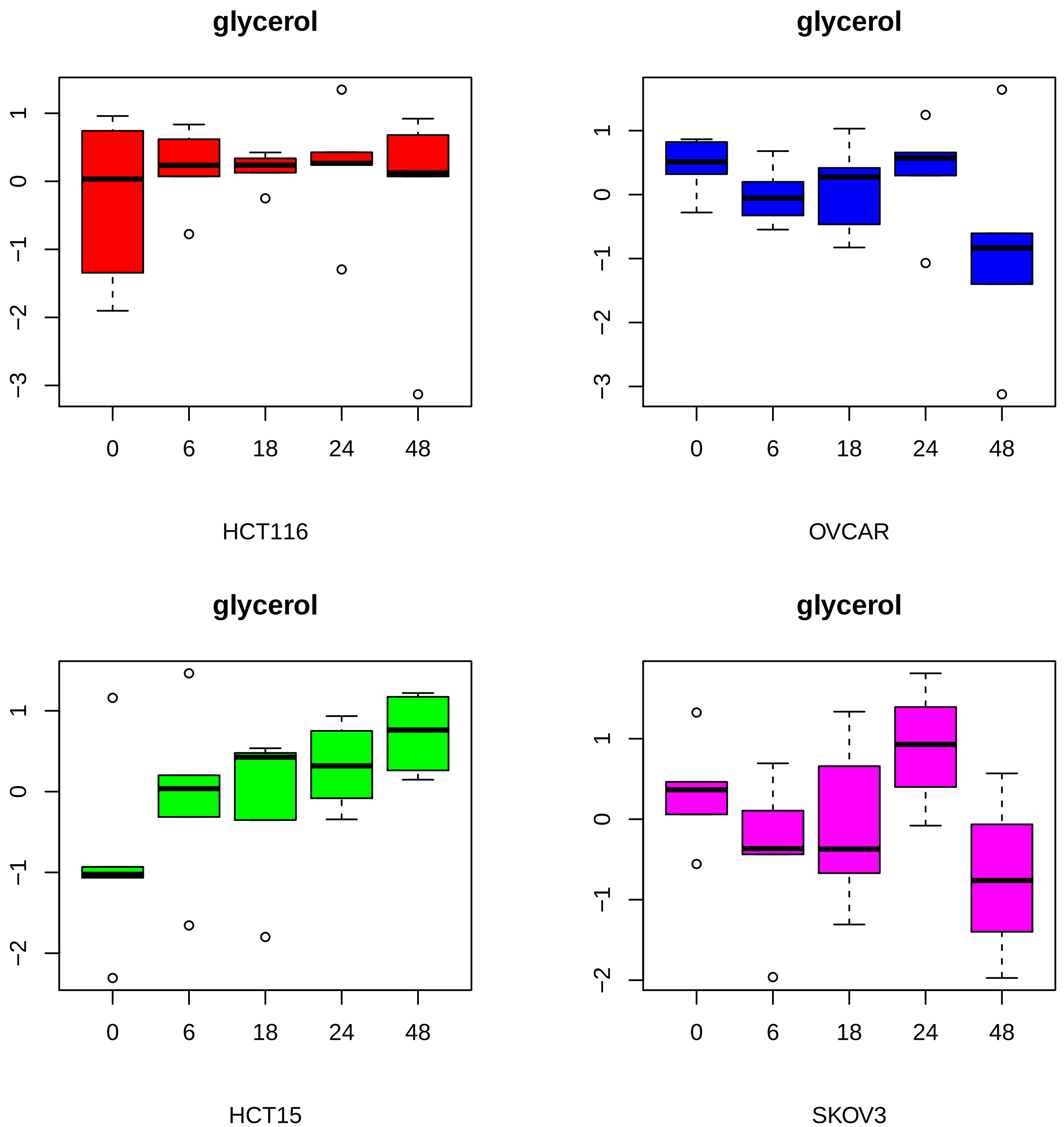

glycerol
1
0
−1
−2
−3
0
6
18
24
48
HCT116
glycerol
1
0
−1
−2
−3
0
6
18
24
48
OVCAR
glycerol
1
0
−1
−2
0
6
18
24
48
HCT15
glycerol
1
0
−1
−2
0
6
18
24
48
SKOV3

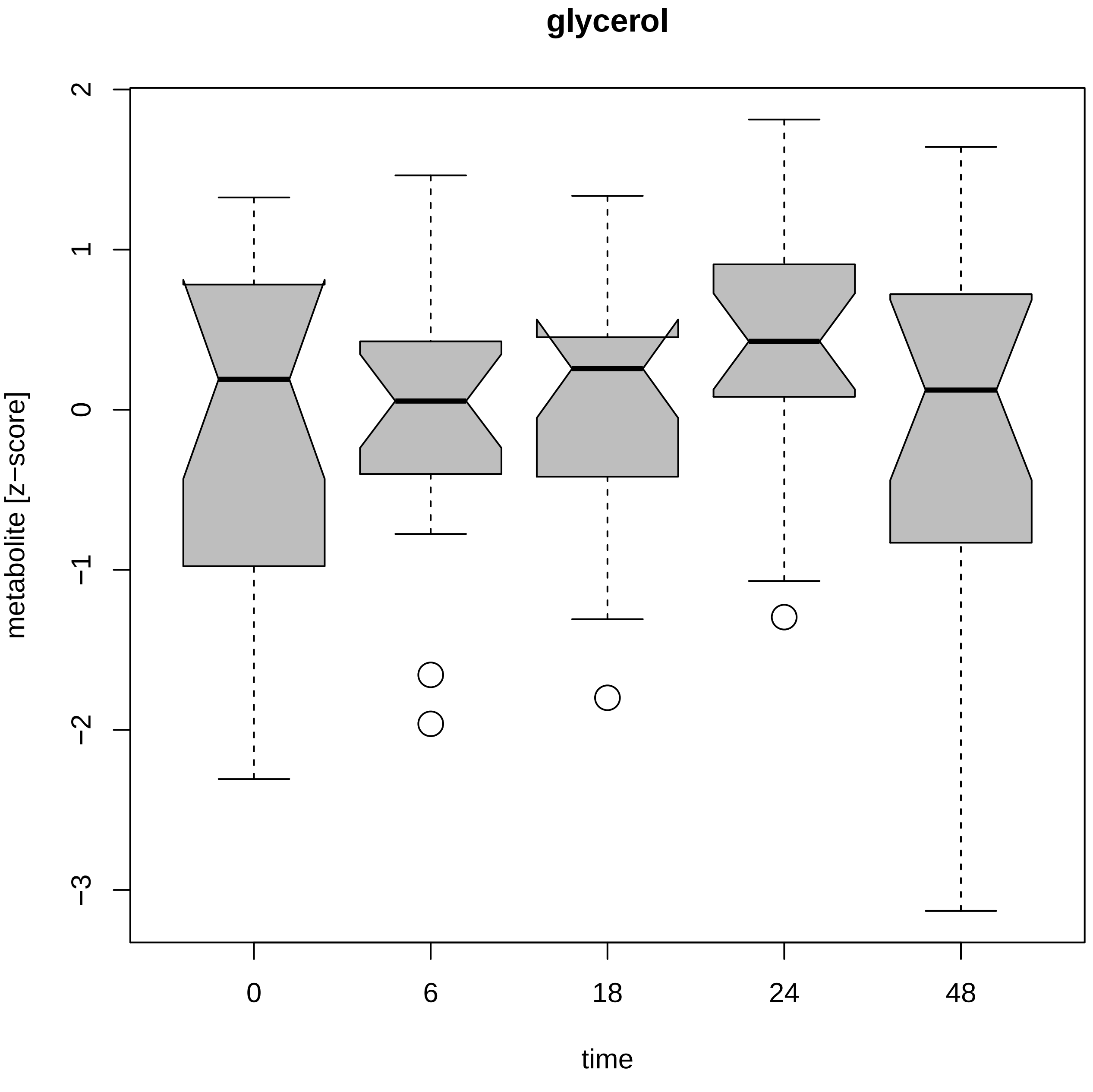

glycerol
metabolite [z−score]
2
1
0
−1
−2
−3
0
6
18
24
48
time

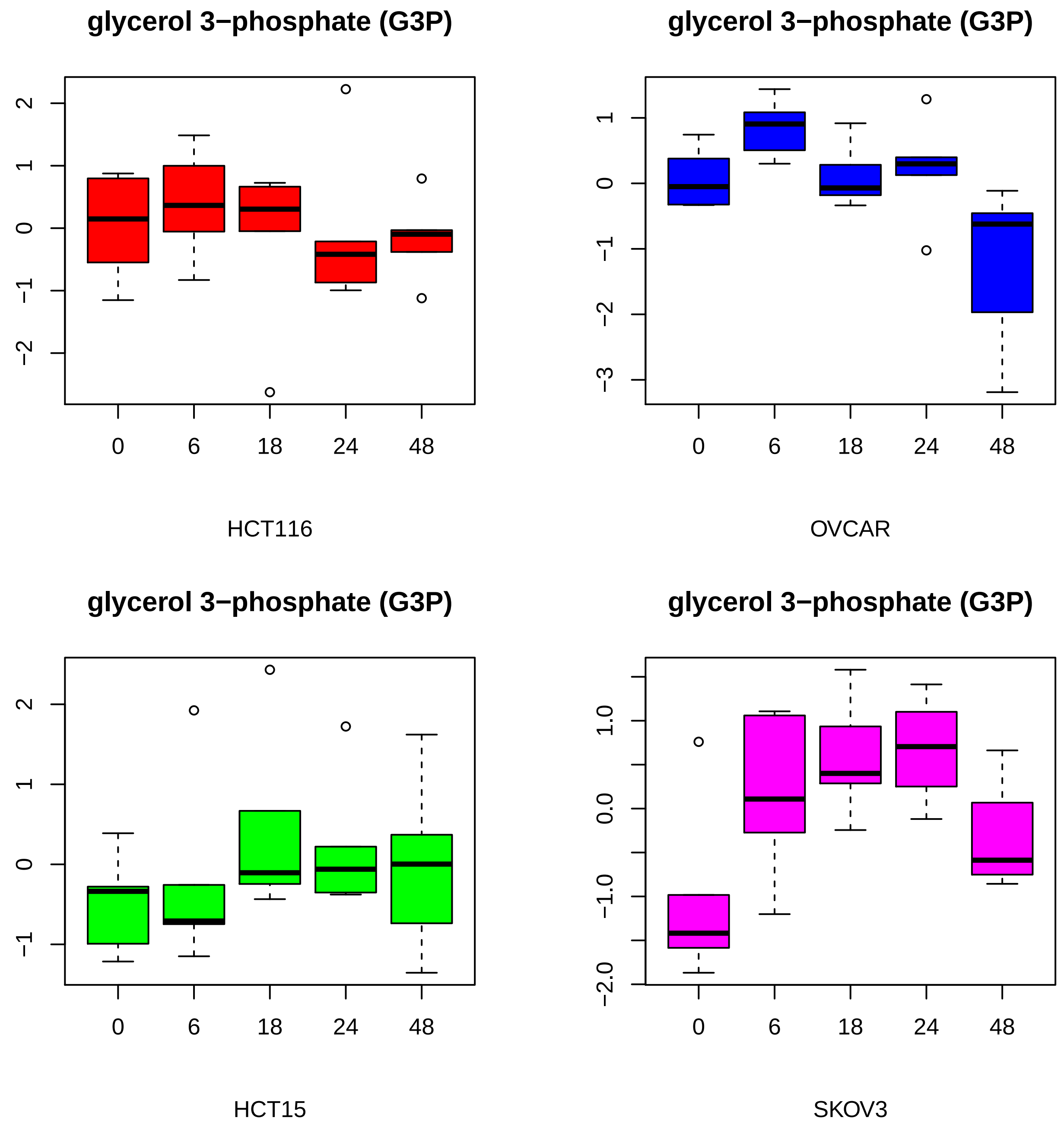
glycerol 3−phosphate (G3P)
HCT116
0 6 18 24 48
−2 −1 0 1 2
glycerol 3−phosphate (G3P)
OVCAR
0 6 18 24 48
−3 −2 −1 0 1
glycerol 3−phosphate (G3P)
HCT15
0 6 18 24 48
−1 0 1 2
glycerol 3−phosphate (G3P)
SKOV3
0 6 18 24 48
−2.0 −1.0 0.0 1.0

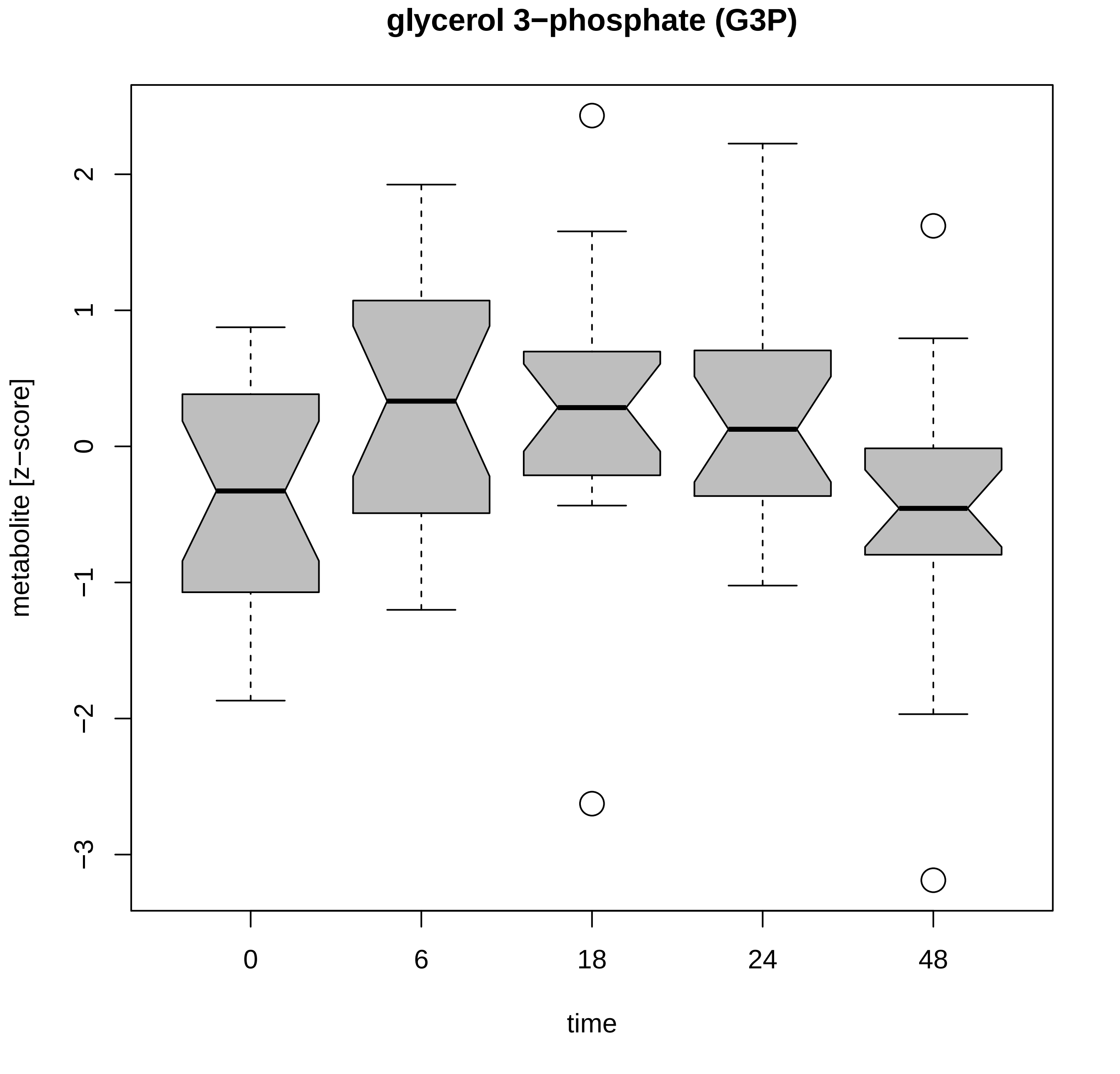

glycerol 3−phosphate (G3P)
metabolite [z−score]
time
2
1
0
−1
−2
−3
0
6
18
24
48

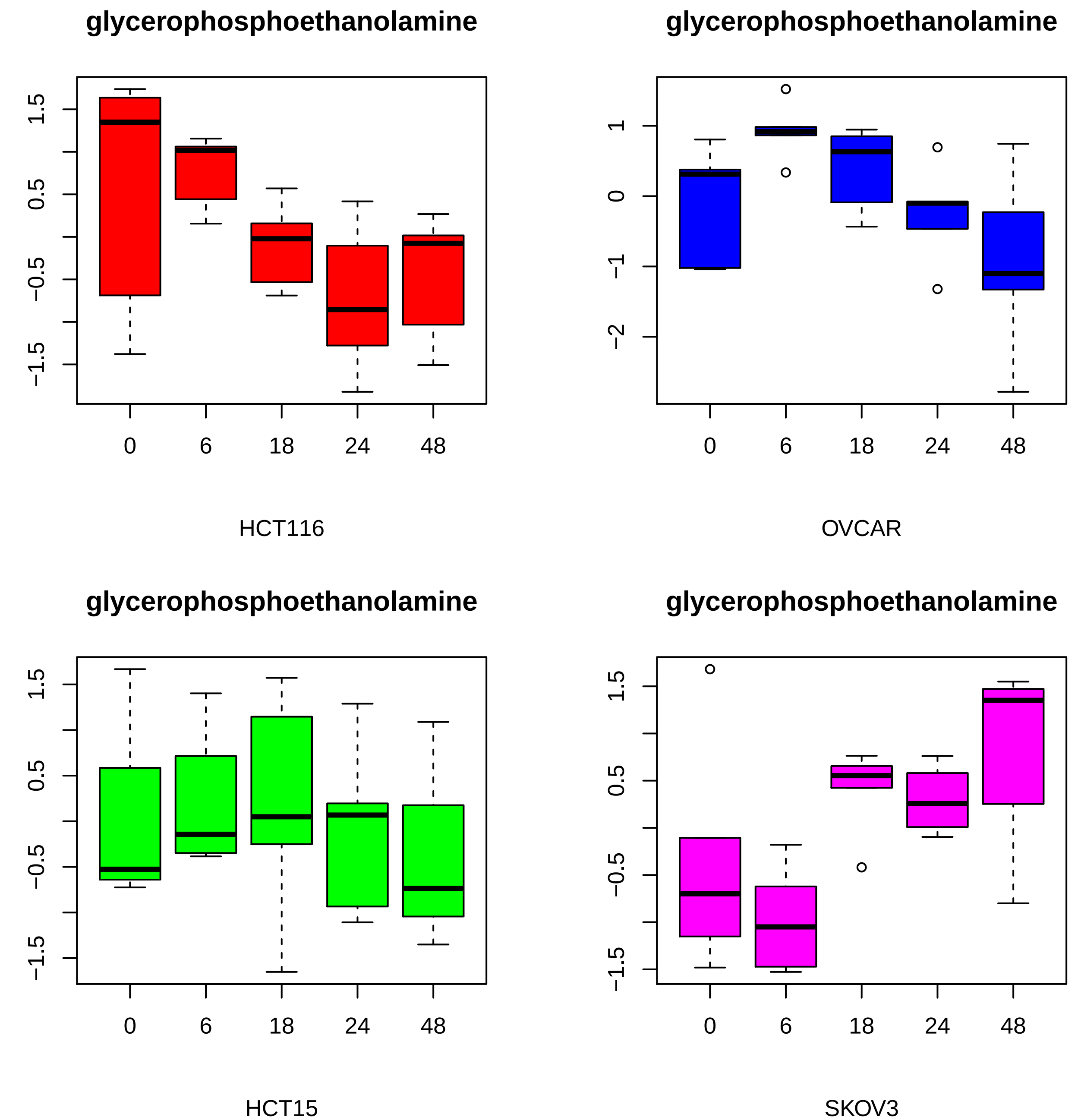
glycerophosphoethanolamine
1.5
0.5
−0.5
−1.5
0
6
18
24
48
HCT116
glycerophosphoethanolamine
1
0
−1
−2
0
6
18
24
48
OVCAR
glycerophosphoethanolamine
1.5
0.5
−0.5
−1.5
0
6
18
24
48
HCT15
glycerophosphoethanolamine
1.5
0.5
−0.5
−1.5
0
6
18
24
48
SKOV3

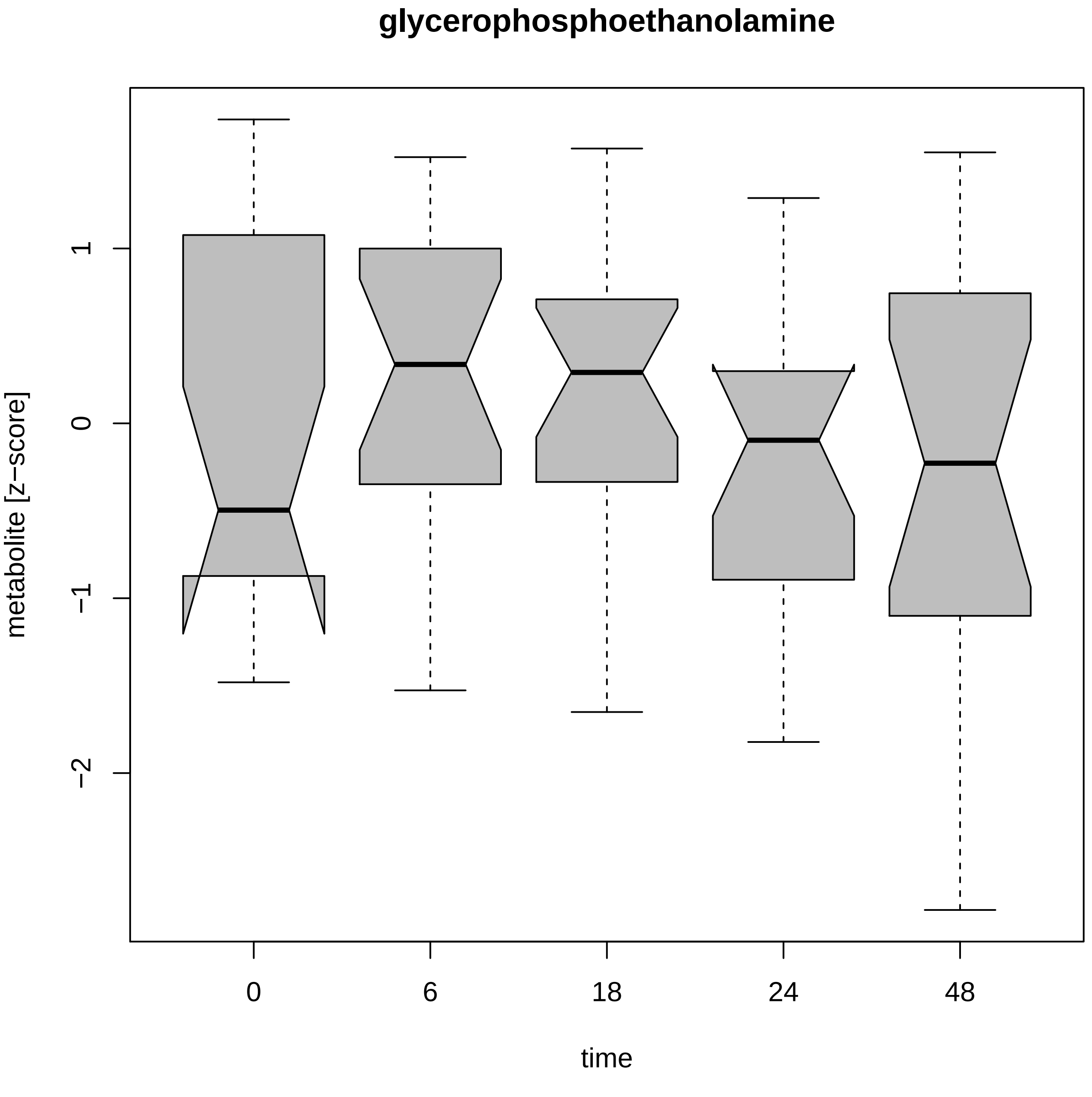
glycerophosphoethanolamine
metabolite [z−score]
1
0
−1
−2
0
6
18
24
48
time

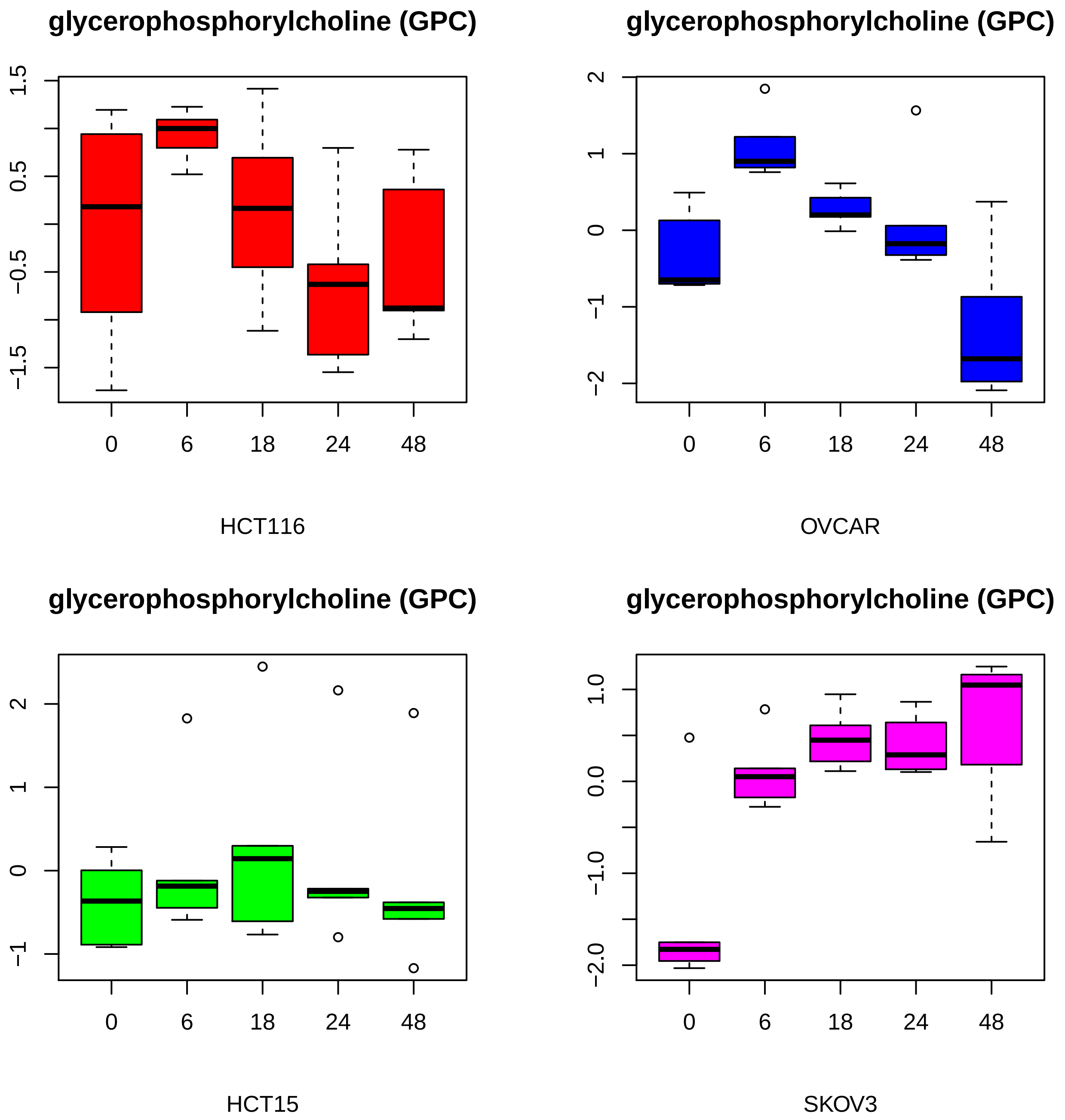
glycerophosphorylcholine (GPC)
1.5
0.5
−0.5
−1.5
0
6
18
24
48
HCT116
glycerophosphorylcholine (GPC)
2
1
0
−1
−2
0
6
18
24
48
OVCAR
glycerophosphorylcholine (GPC)
2
1
0
−1
0
6
18
24
48
HCT15
glycerophosphorylcholine (GPC)
1.0
0.0
−1.0
−2.0
0
6
18
24
48
SKOV3

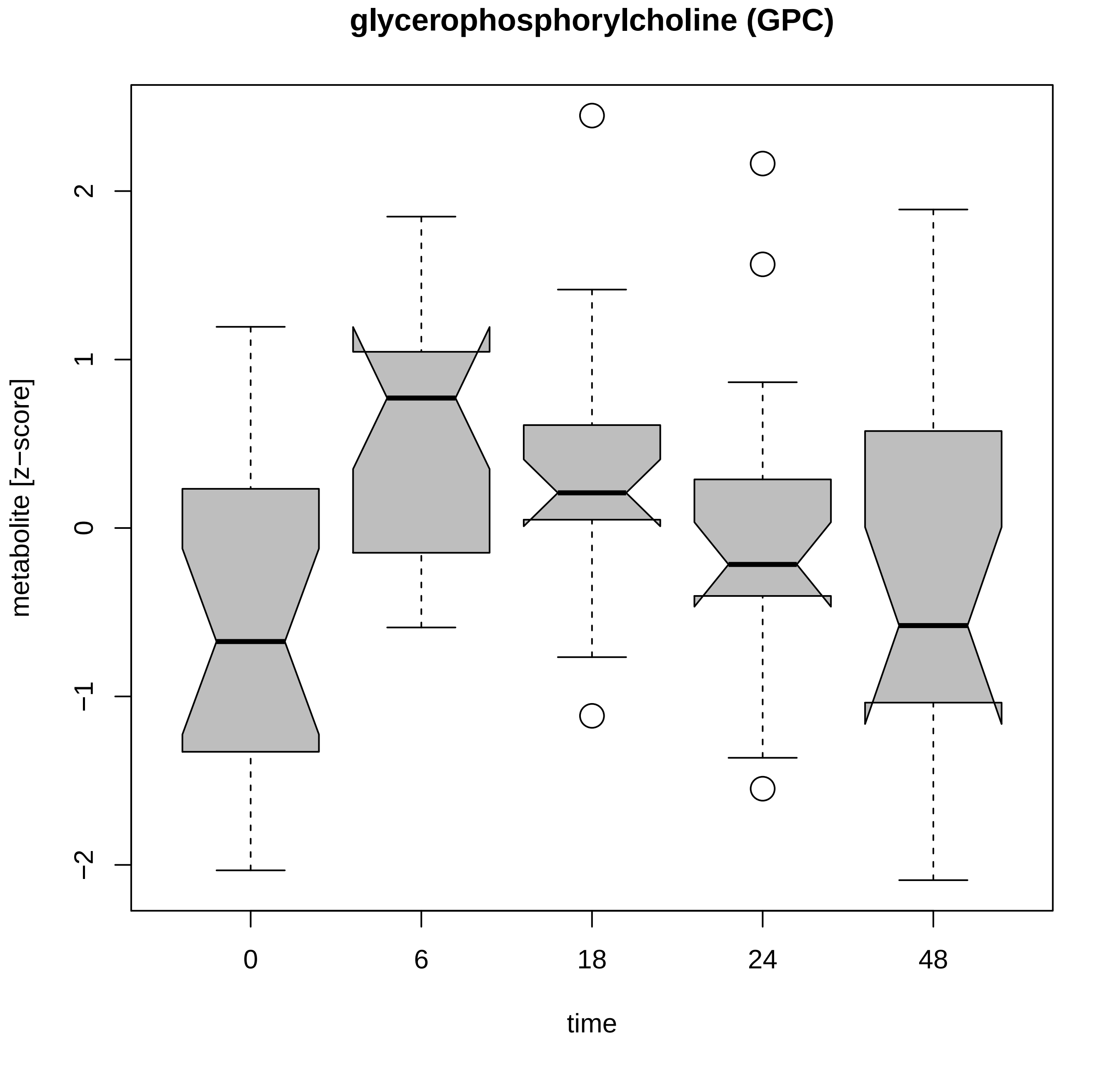

glycerophosphorylcholine (GPC)
metabolite [z−score]
2
1
0
−1
−2
0
6
18
24
48
time

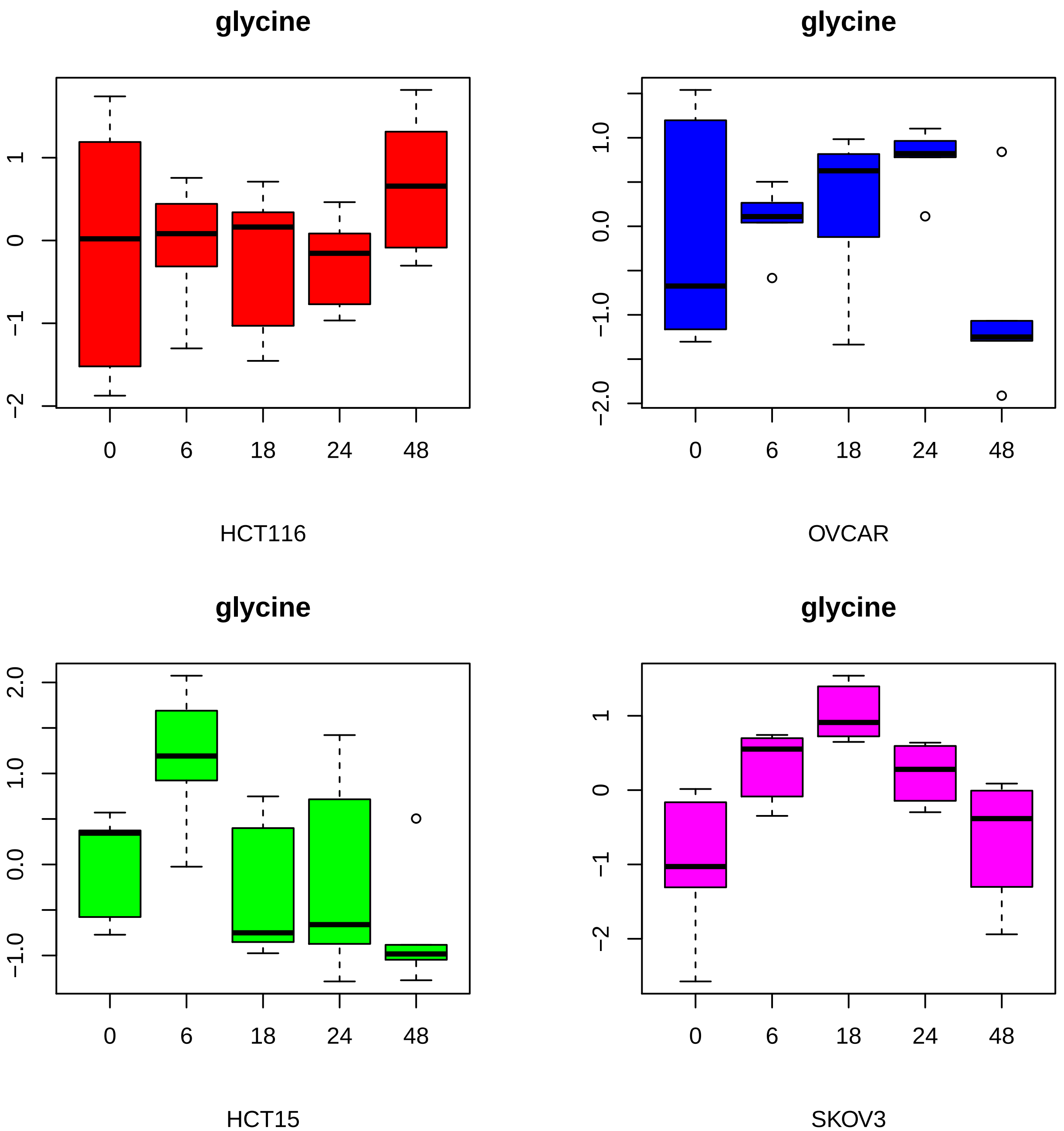
glycine
HCT116
glycine
OVCAR
glycine
HCT15
glycine
SKOV3
0
6
18
24
48

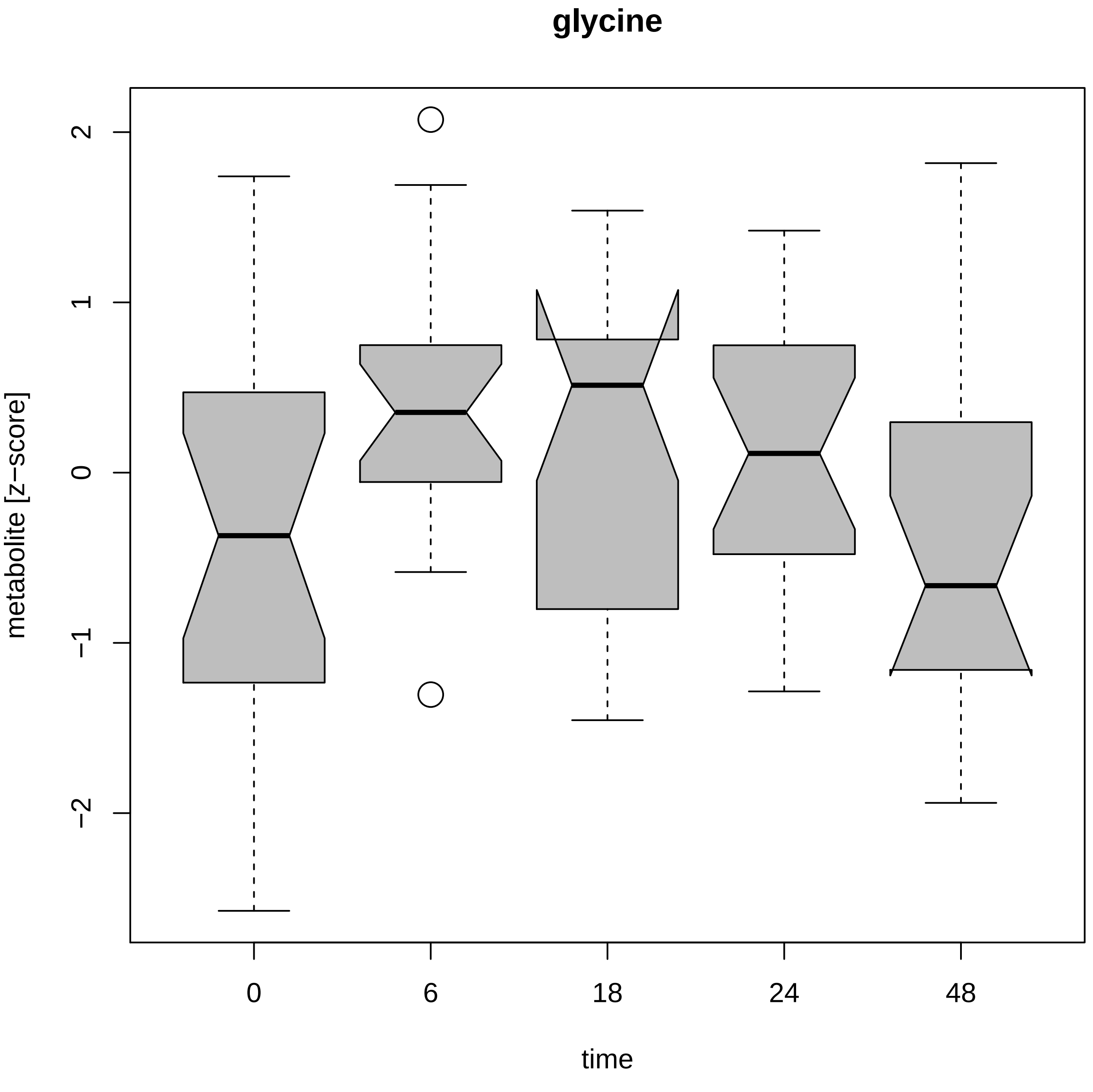
glycine
metabolite [z−score]
2
1
0
−1
−2
0
6
18
24
48
time

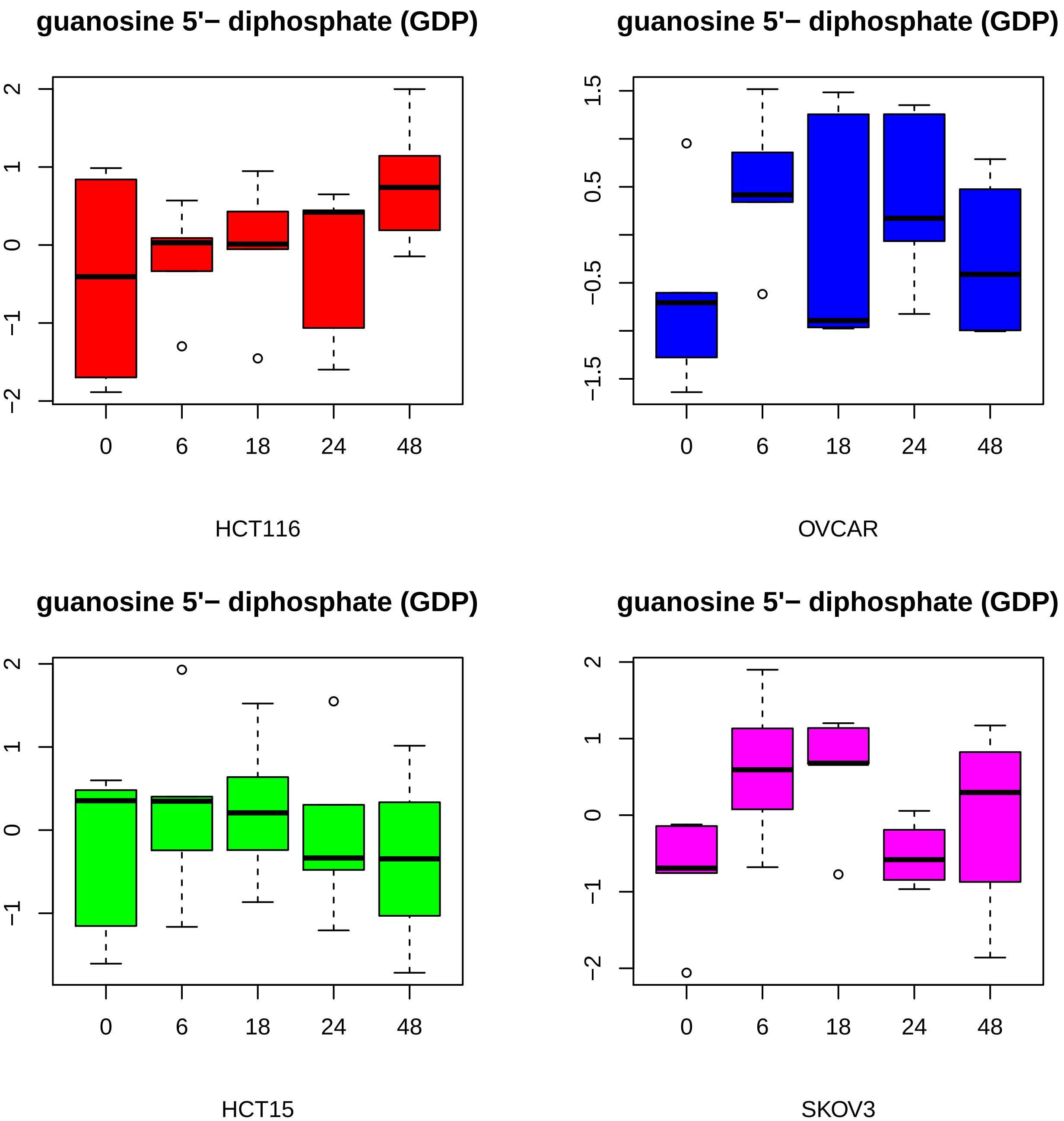

guanosine 5'− diphosphate (GDP)
2
1
0
−1
−2
0
6
18
24
48
HCT116
guanosine 5'− diphosphate (GDP)
1.5
0.5
−0.5
−1.5
0
6
18
24
48
OVCAR
guanosine 5'− diphosphate (GDP)
2
1
0
−1
0
6
18
24
48
HCT15
guanosine 5'− diphosphate (GDP)
2
1
0
−1
−2
0
6
18
24
48
SKOV3

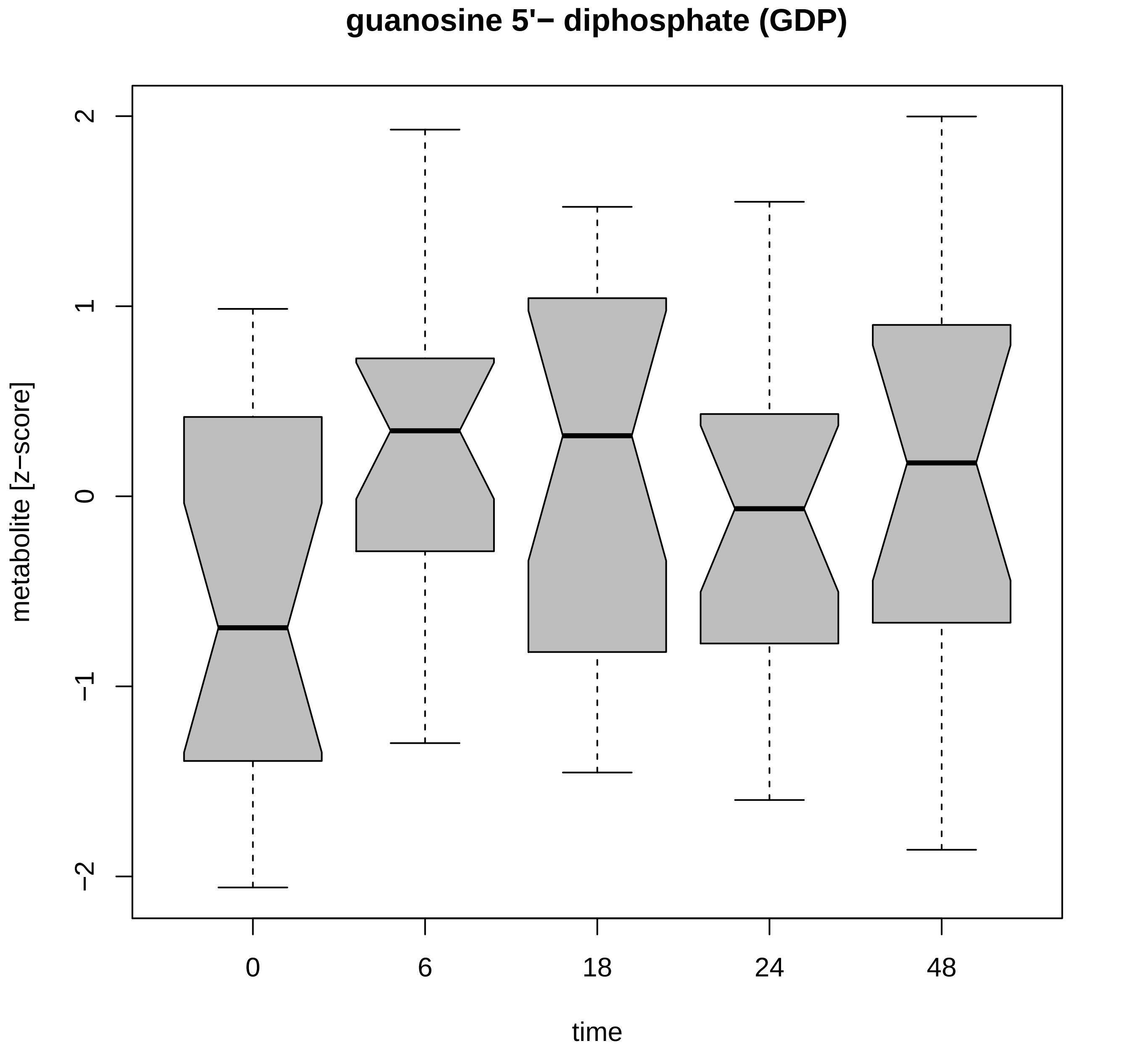
guanosine 5'− diphosphate (GDP)
metabolite [z−score]
2
1
0
−1
−2
0
6
18
24
48
time

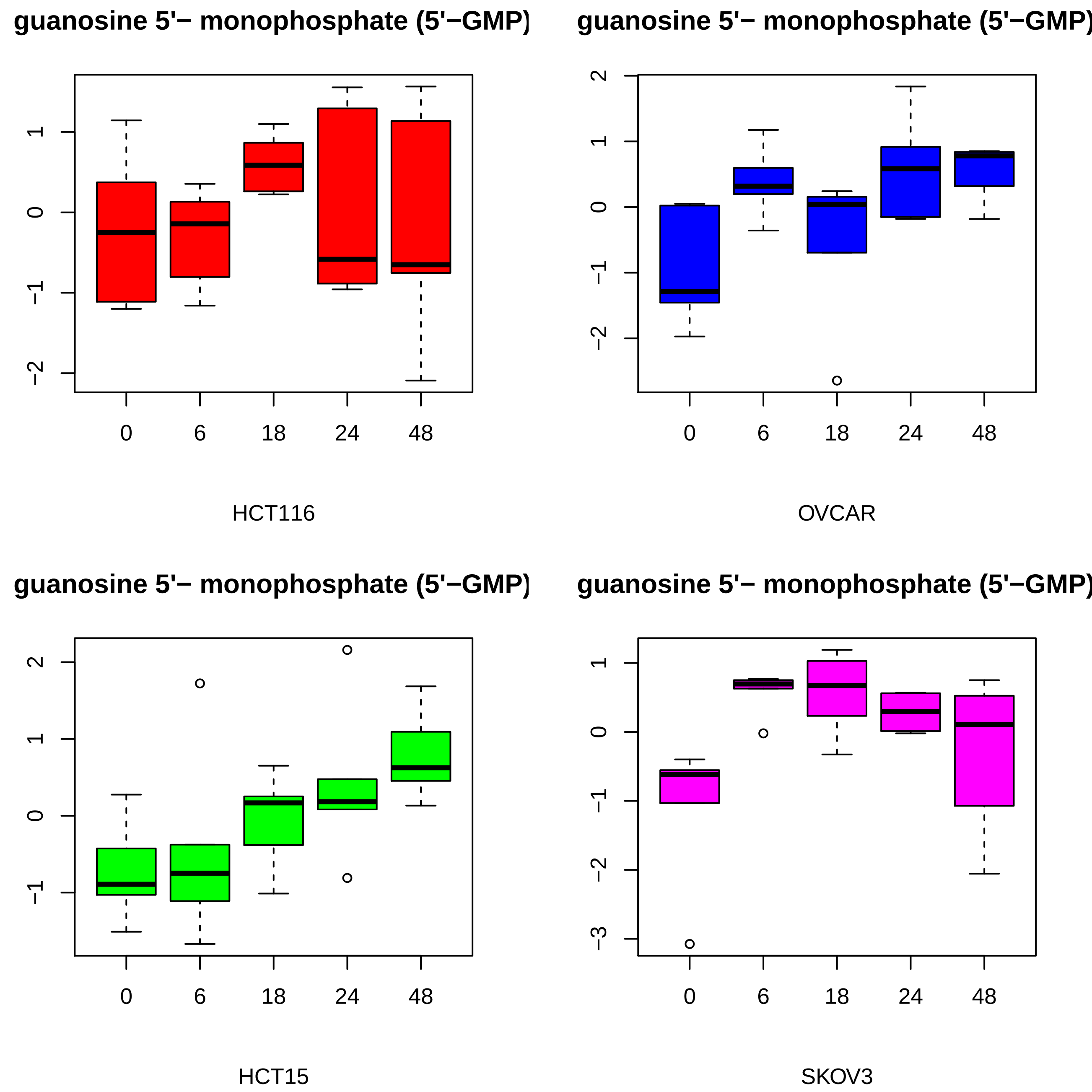
guanosine 5'− monophosphate (5'−GMP)
HCT116
0
6
18
24
48
1
0
−1
−2
guanosine 5'− monophosphate (5'−GMP)
OVCAR
2
1
0
−1
−2
guanosine 5'− monophosphate (5'−GMP)
HCT15
guanosine 5'− monophosphate (5'−GMP)
SKOV3
−3

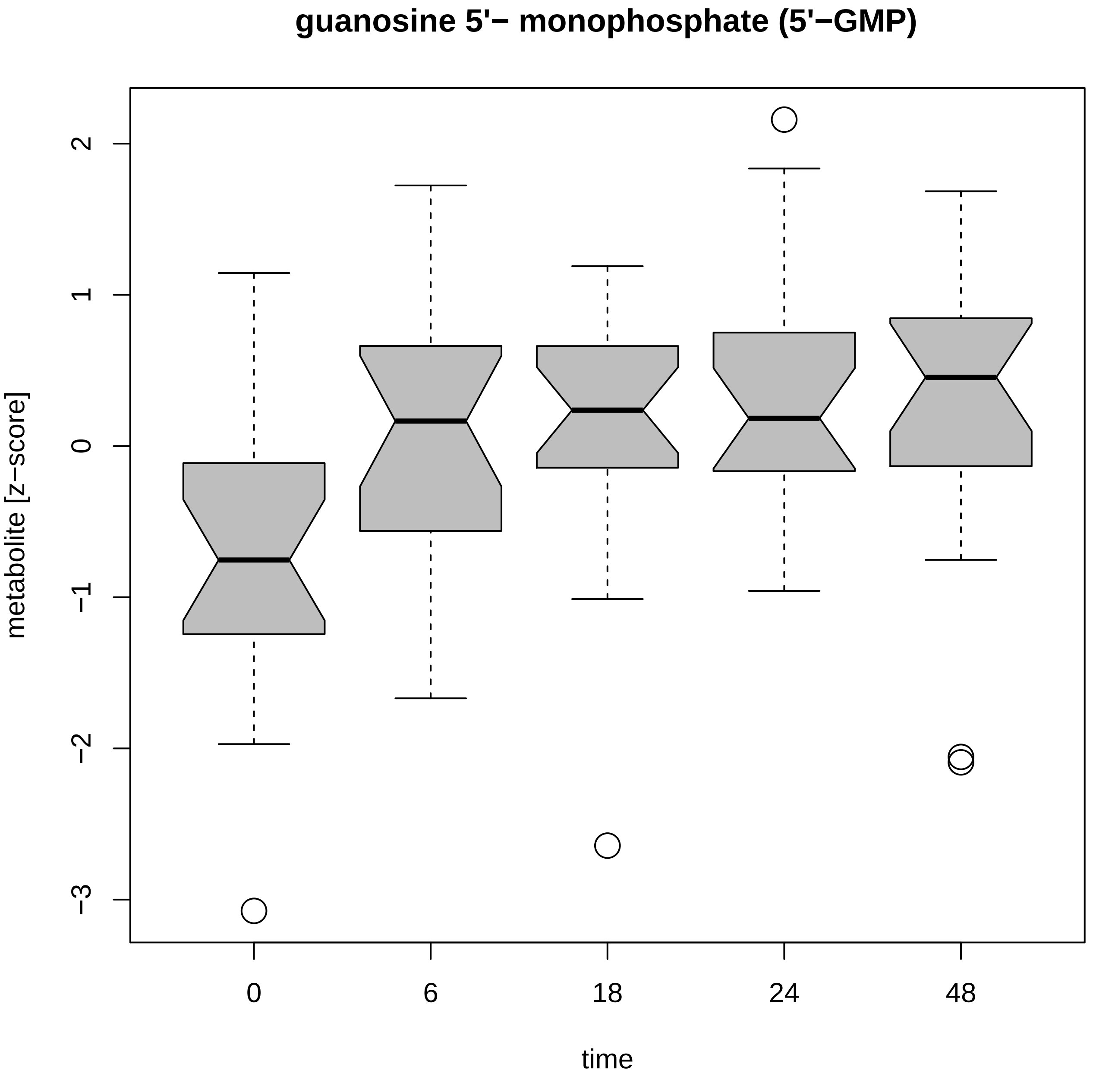

guanosine 5'− monophosphate (5'−GMP)
metabolite [z−score]
2
1
0
−1
−2
−3
0
6
18
24
48
time

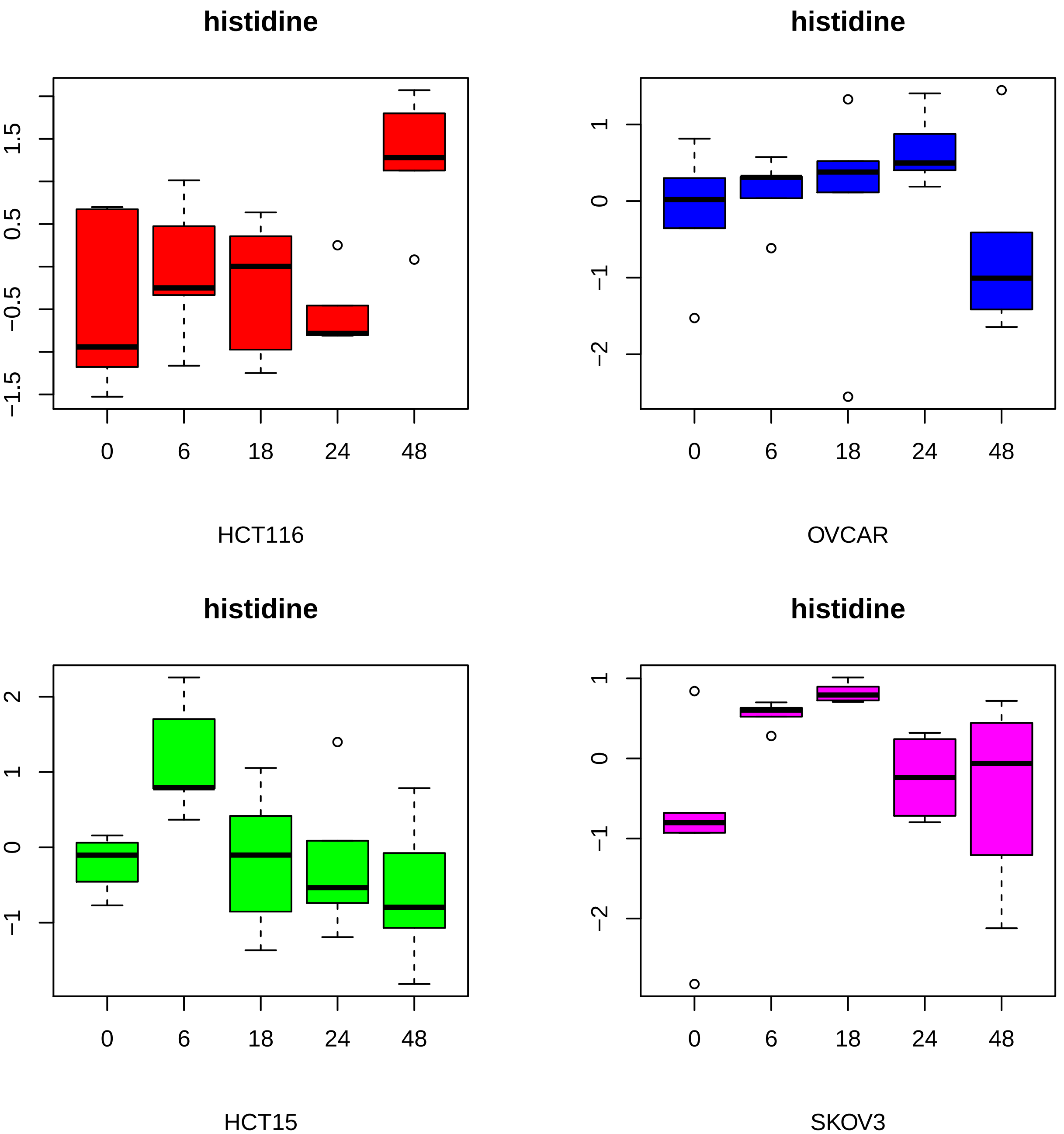
histidine
HCT116
0
6
18
24
48
−1.5
−0.5
0.5
1.5
histidine
OVCAR
−2
−1
0
1
histidine
HCT15
−1
0
1
2
histidine
SKOV3
−2
−1
0
1

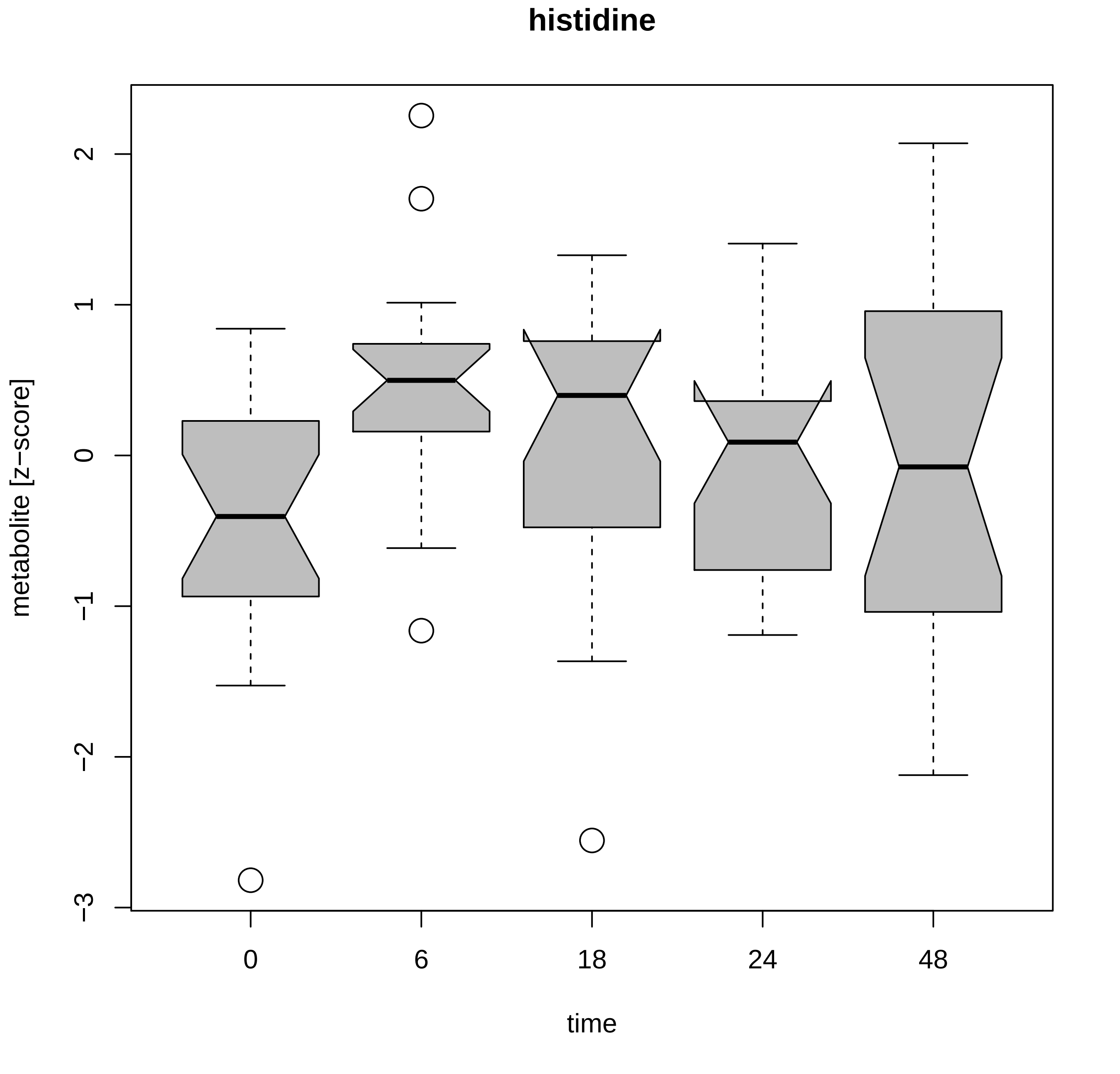
histidine
metabolite [z−score]
time
2
1
0
−1
−2
−3
0
6
18
24
48

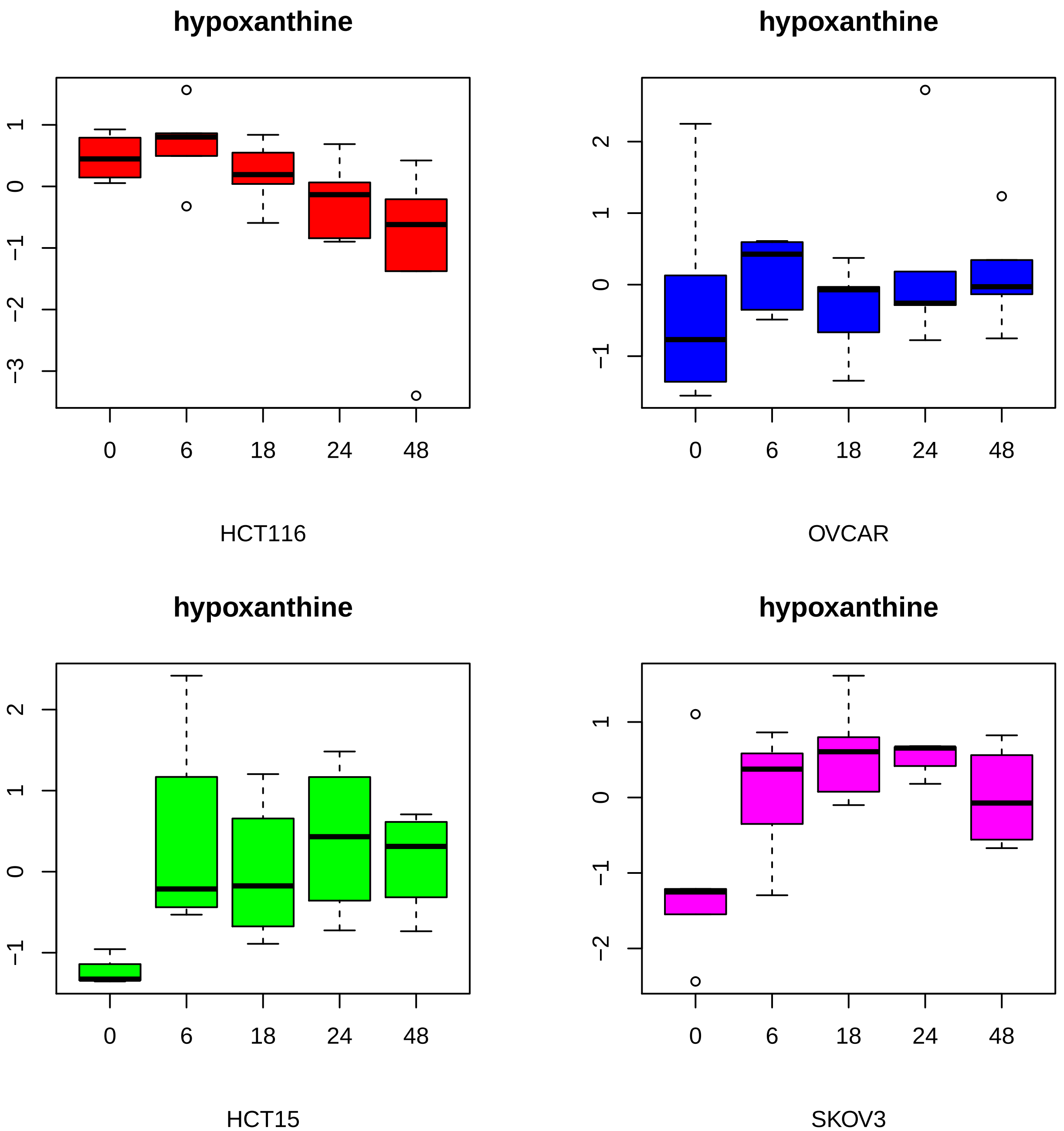
hypoxanthine
1
0
−1
−2
−3
0
6
18
24
48
HCT116
hypoxanthine
2
1
0
−1
0
6
18
24
48
OVCAR
hypoxanthine
2
1
0
−1
0
6
18
24
48
HCT15
hypoxanthine
1
0
−1
−2
0
6
18
24
48
SKOV3

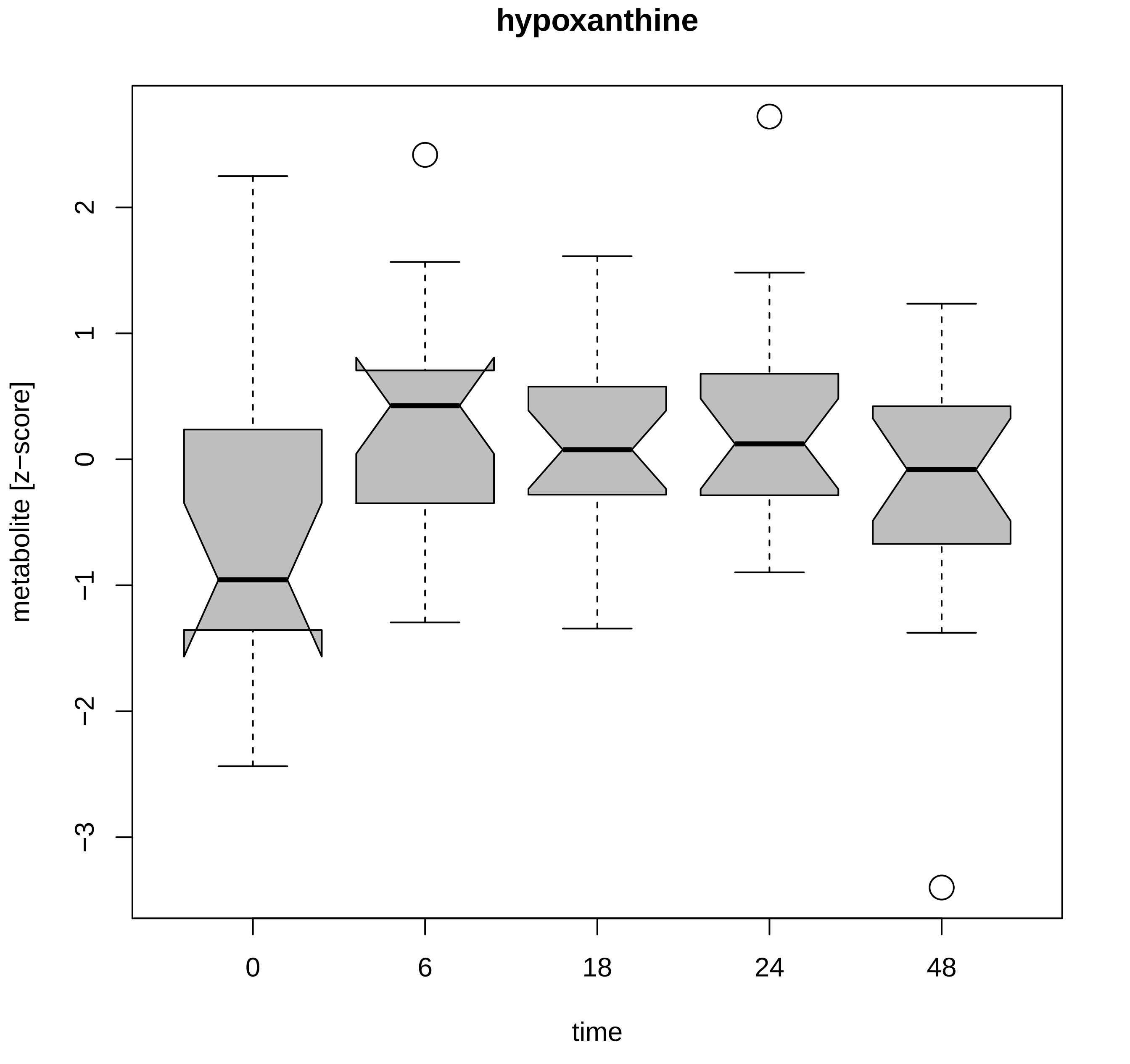
hypoxanthine
metabolite [z−score]
2
1
0
−1
−2
−3
0
6
18
24
48
time

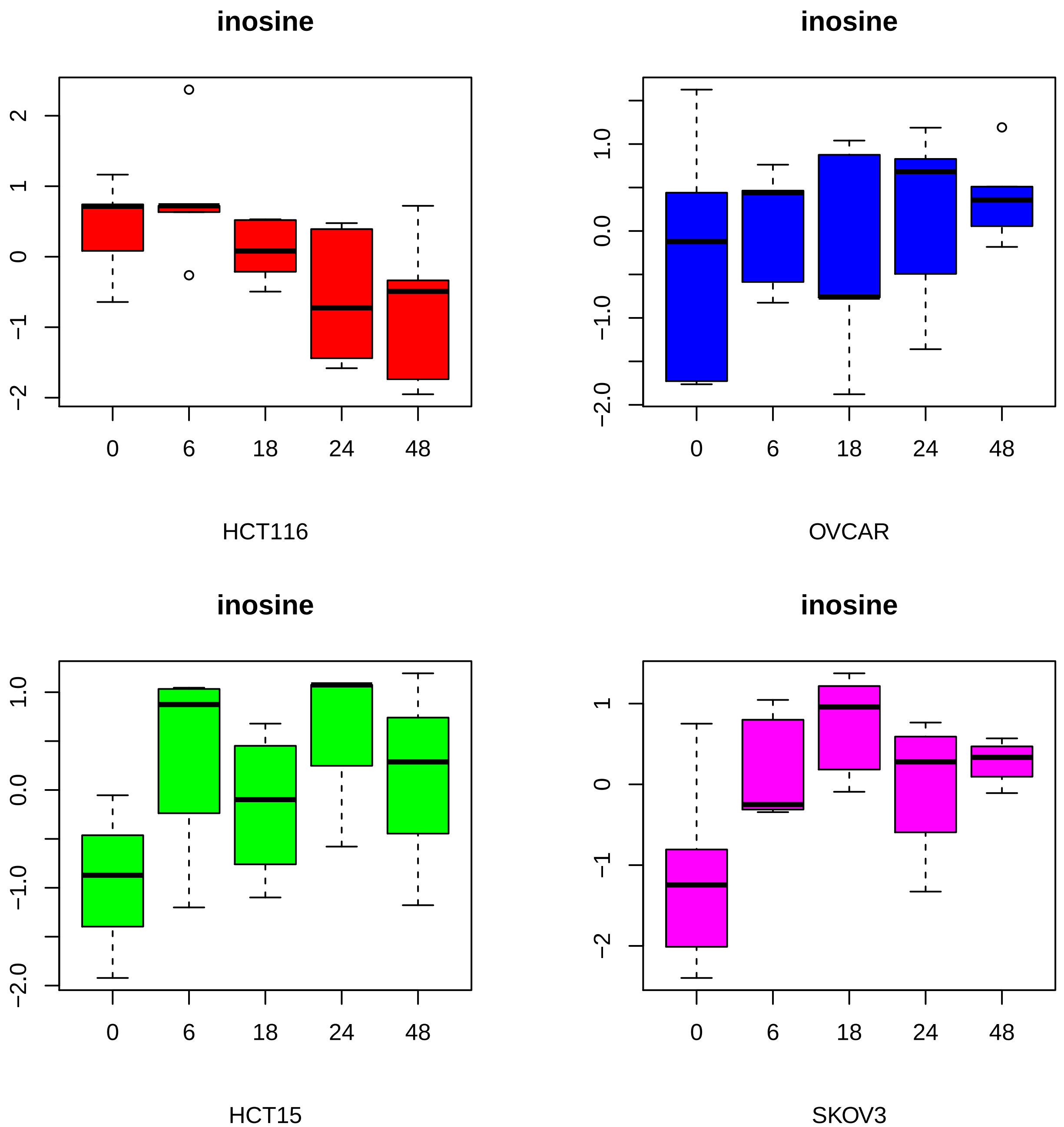
inosine
2
1
0
−1
−2
0
6
18
24
48
HCT116
inosine
1.0
0.0
−1.0
−2.0
0
6
18
24
48
OVCAR
inosine
1.0
0.0
−1.0
−2.0
0
6
18
24
48
HCT15
inosine
1
0
−1
−2
0
6
18
24
48
SKOV3

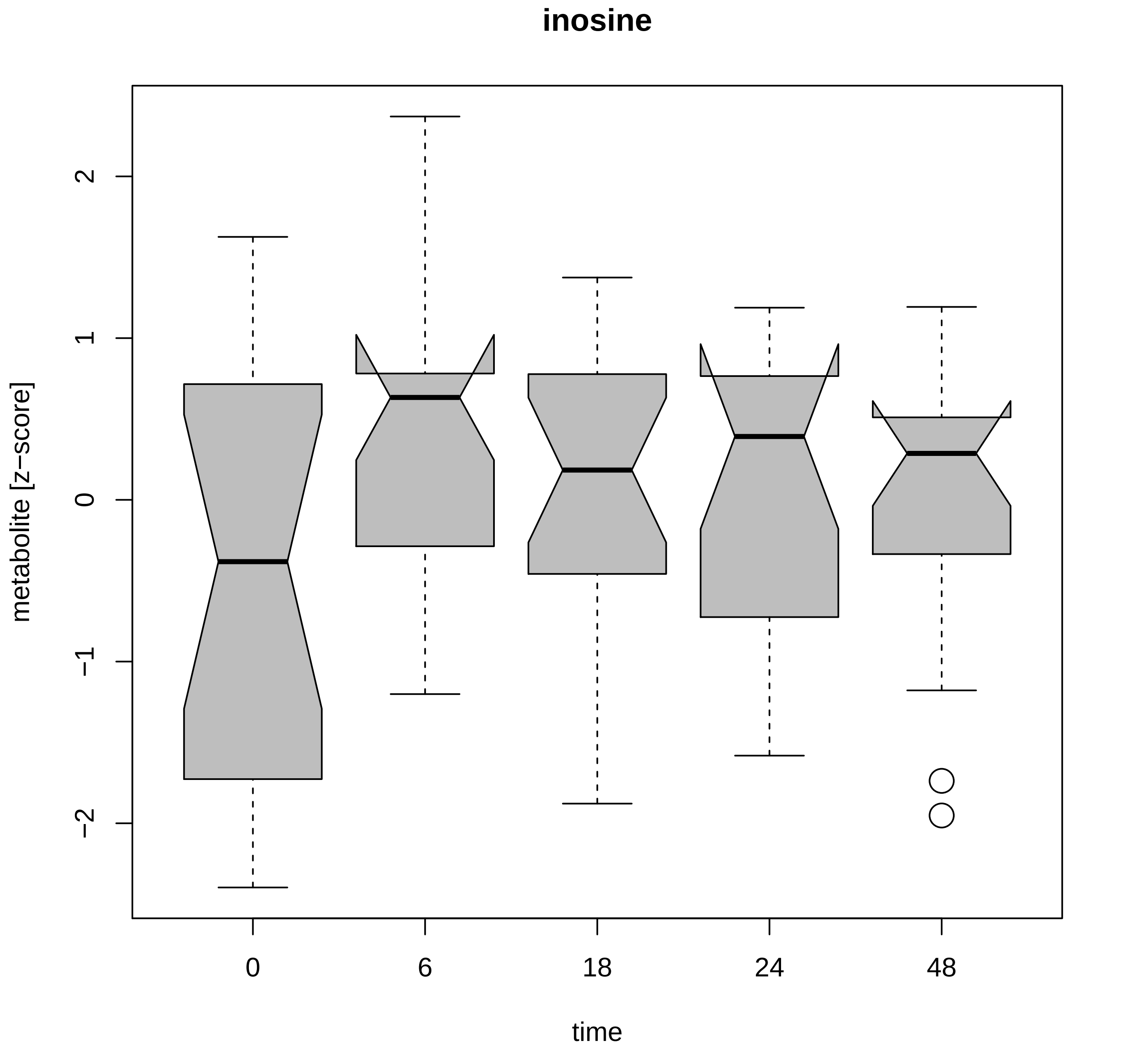

inosine
metabolite [z−score]
2
1
0
−1
−2
0
6
18
24
48
time

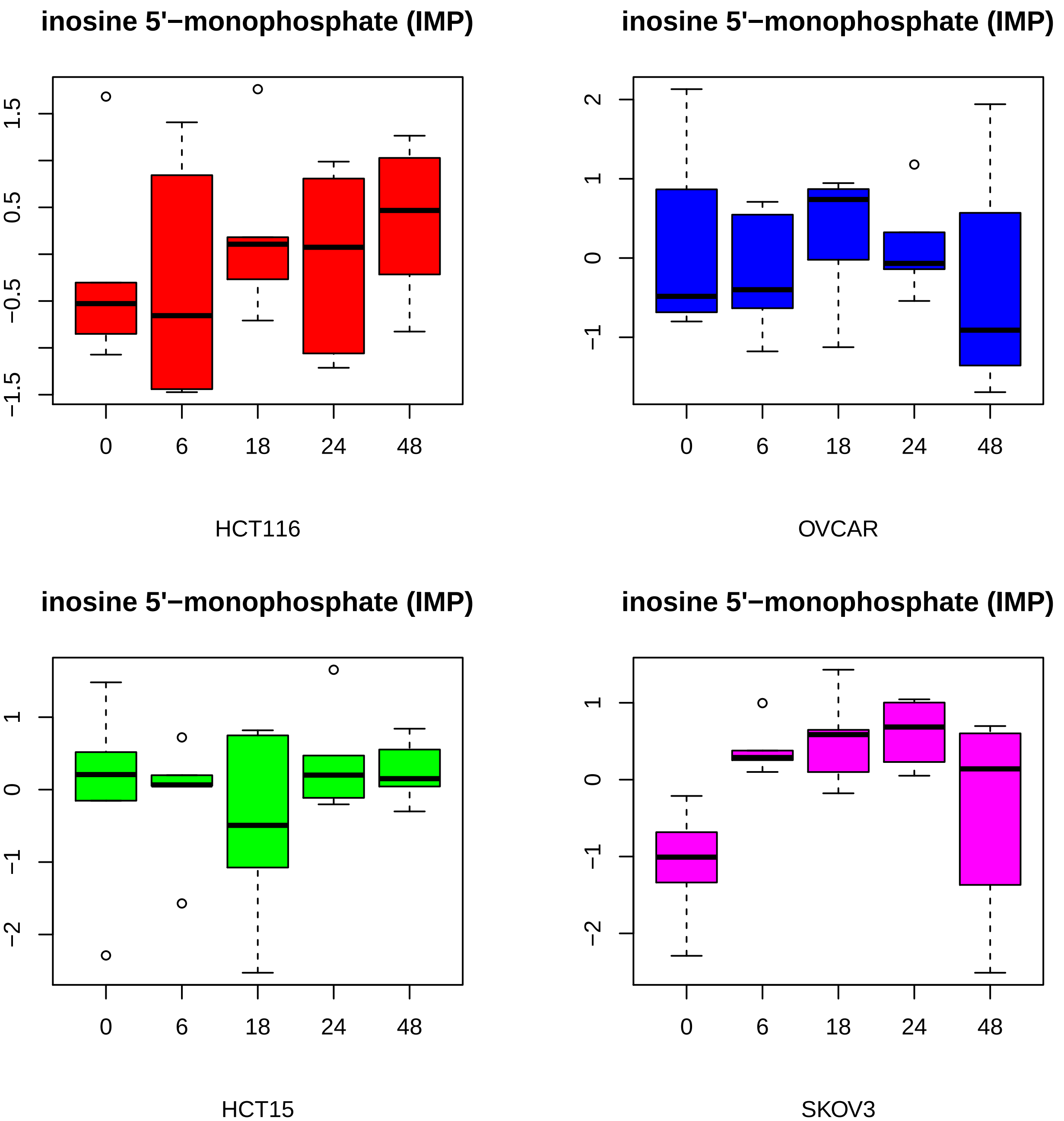
inosine 5'−monophosphate (IMP)
−1.5
−0.5
0.5
1.5
0
6
18
24
48
HCT116
inosine 5'−monophosphate (IMP)
−1
0
1
2
0
6
18
24
48
OVCAR
inosine 5'−monophosphate (IMP)
−2
−1
0
1
0
6
18
24
48
HCT15
inosine 5'−monophosphate (IMP)
−2
−1
0
1
0
6
18
24
48
SKOV3

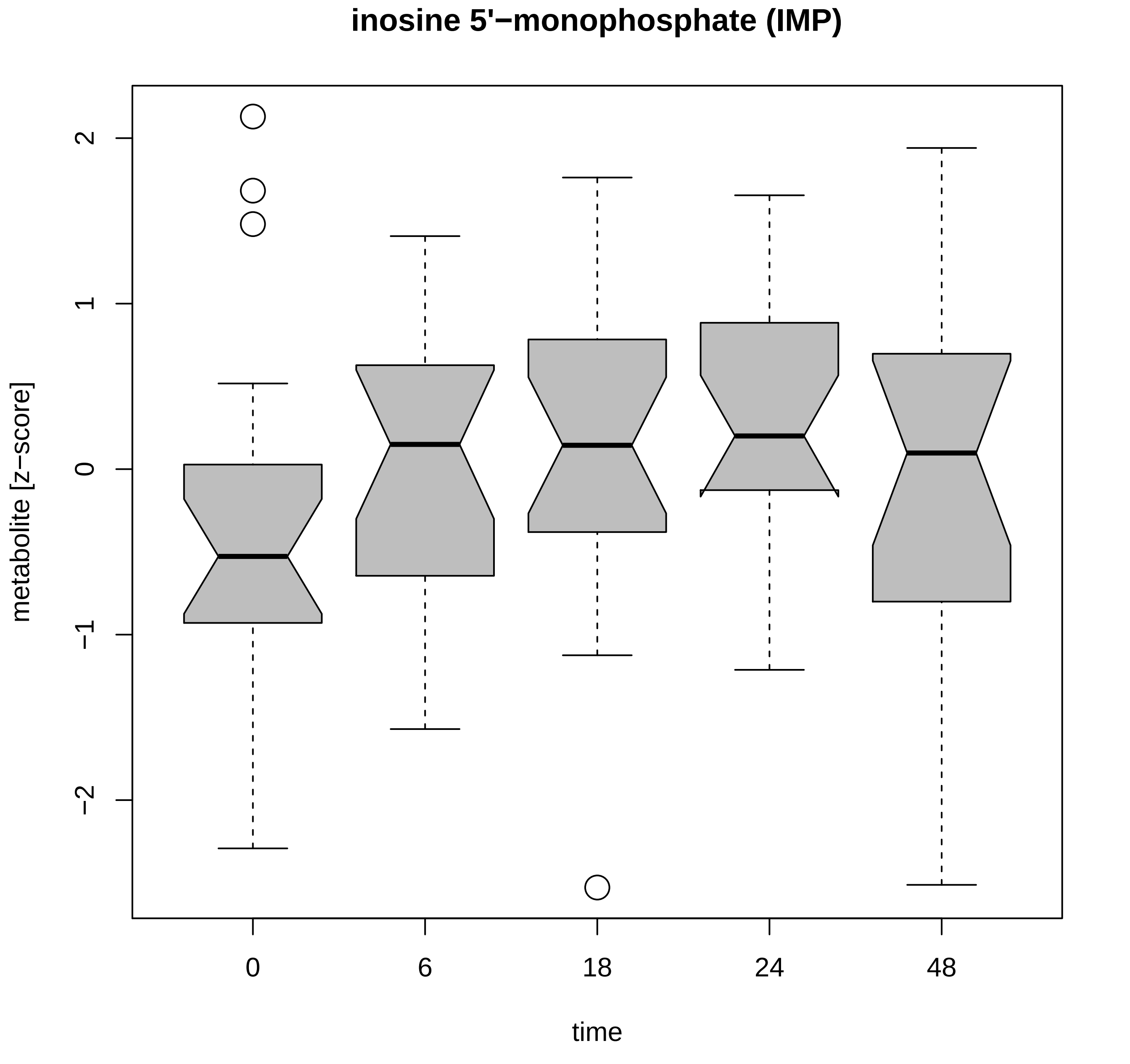
inosine 5'−monophosphate (IMP)
metabolite [z−score]
−2
−1
0
1
2
0
6
18
24
48
time

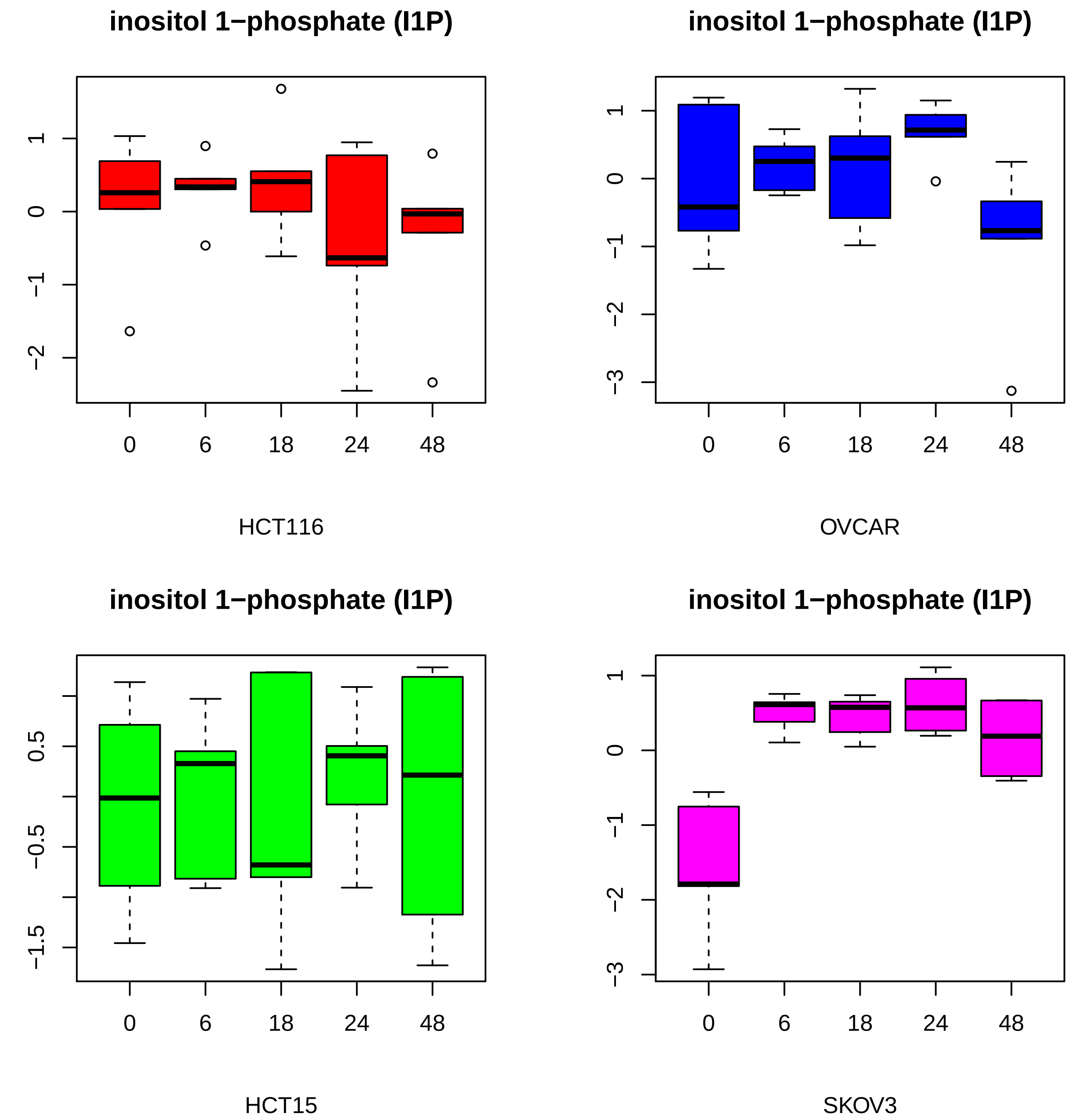
inositol 1−phosphate (I1P)
HCT116
inositol 1−phosphate (I1P)
OVCAR
inositol 1−phosphate (I1P)
HCT15
inositol 1−phosphate (I1P)
SKOV3
0
6
18
24
48
1
0
−1
−2
−3
0.5
−0.5
−1.5

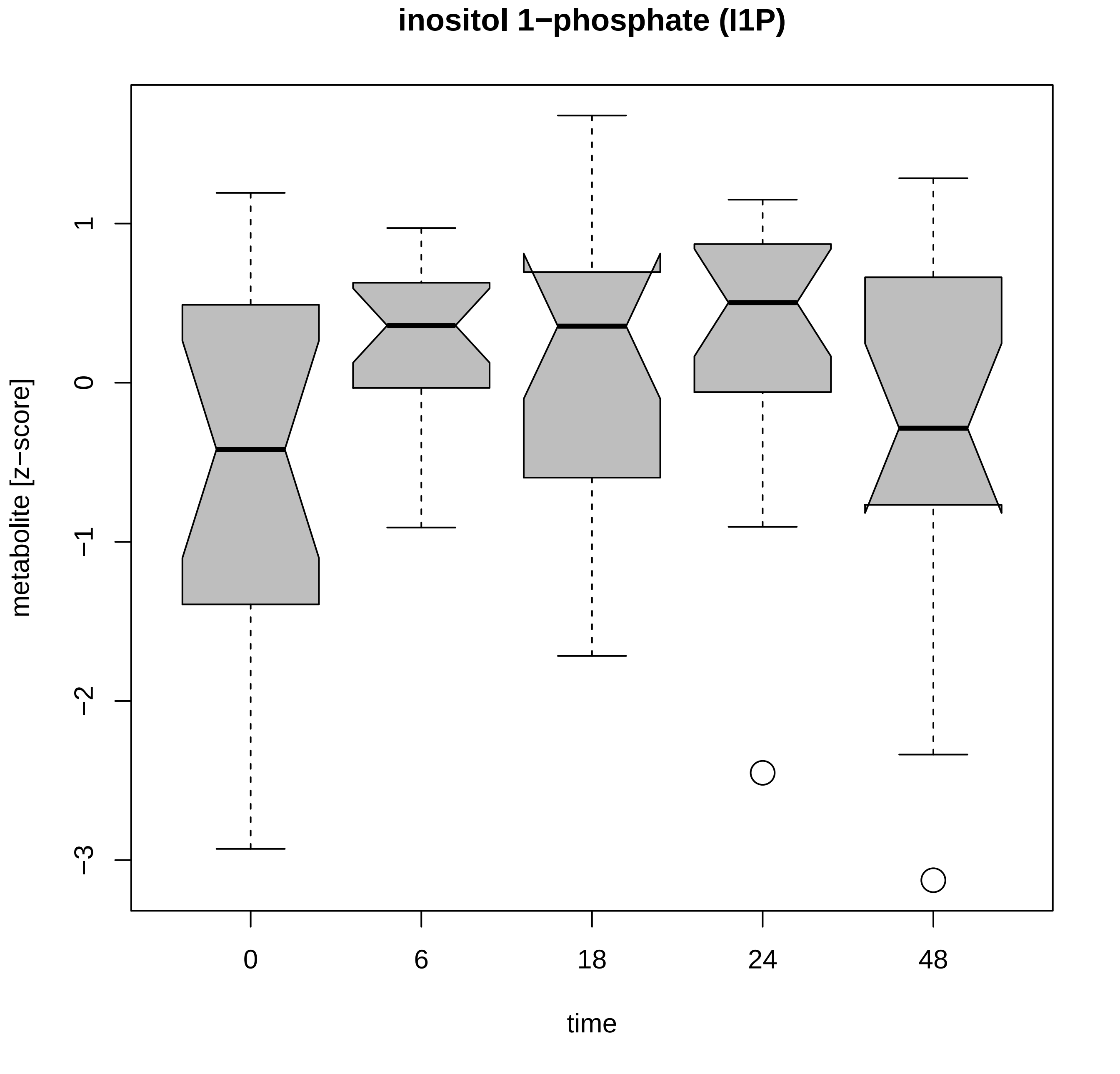

inositol 1−phosphate (I1P)
metabolite [z−score]
1
0
−1
−2
−3
0
6
18
24
48
time

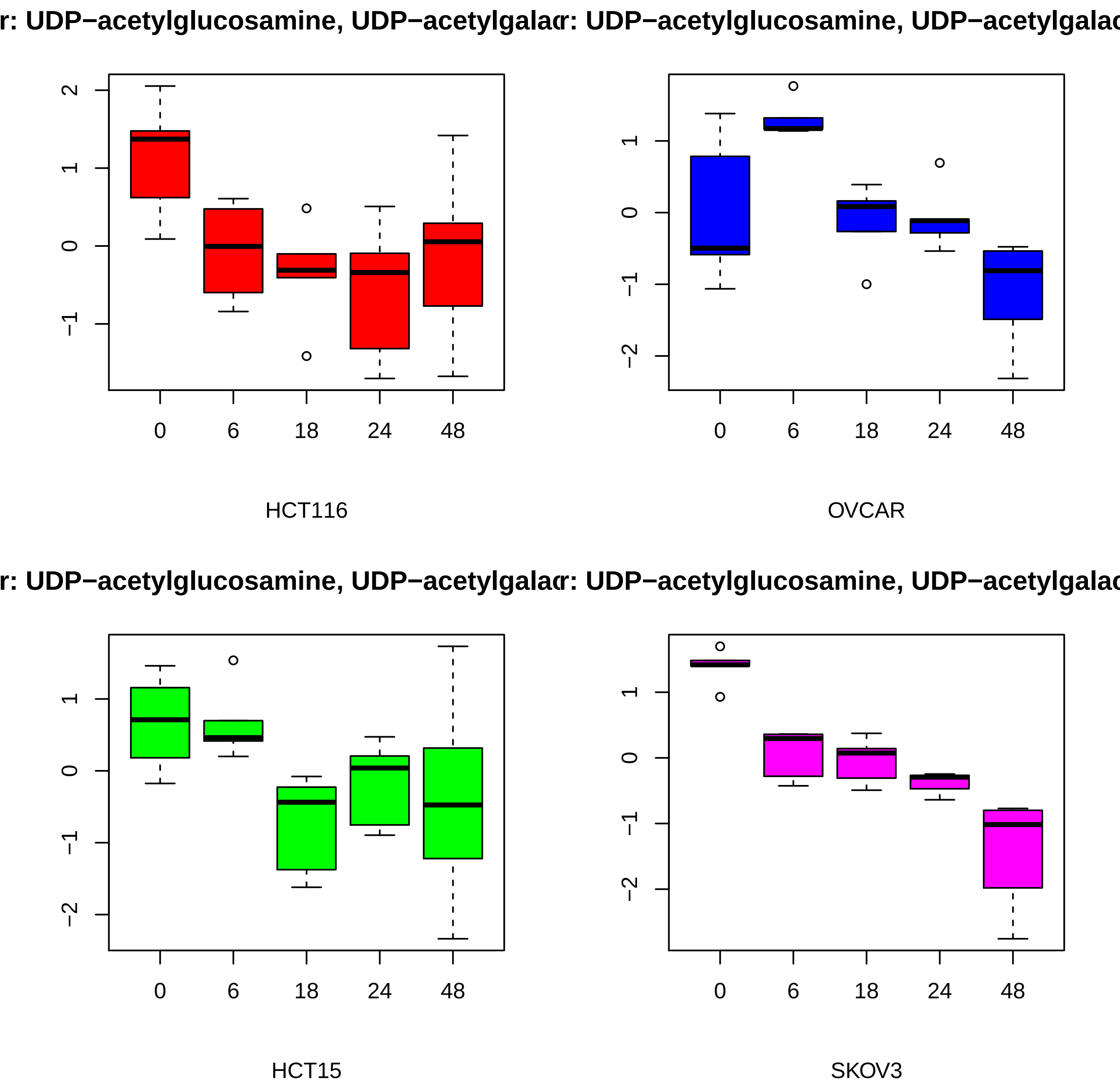

r: UDP−acetylglucosamine, UDP−acetylgalac
r: UDP−acetylglucosamine, UDP−acetylgalac
2
1
0
−1
0
6
18
24
48
HCT116
1
0
−1
−2
0
6
18
24
48
OVCAR
r: UDP−acetylglucosamine, UDP−acetylgalac
r: UDP−acetylglucosamine, UDP−acetylgalac
1
0
−1
−2
0
6
18
24
48
HCT15
1
0
−1
−2
0
6
18
24
48
SKOV3

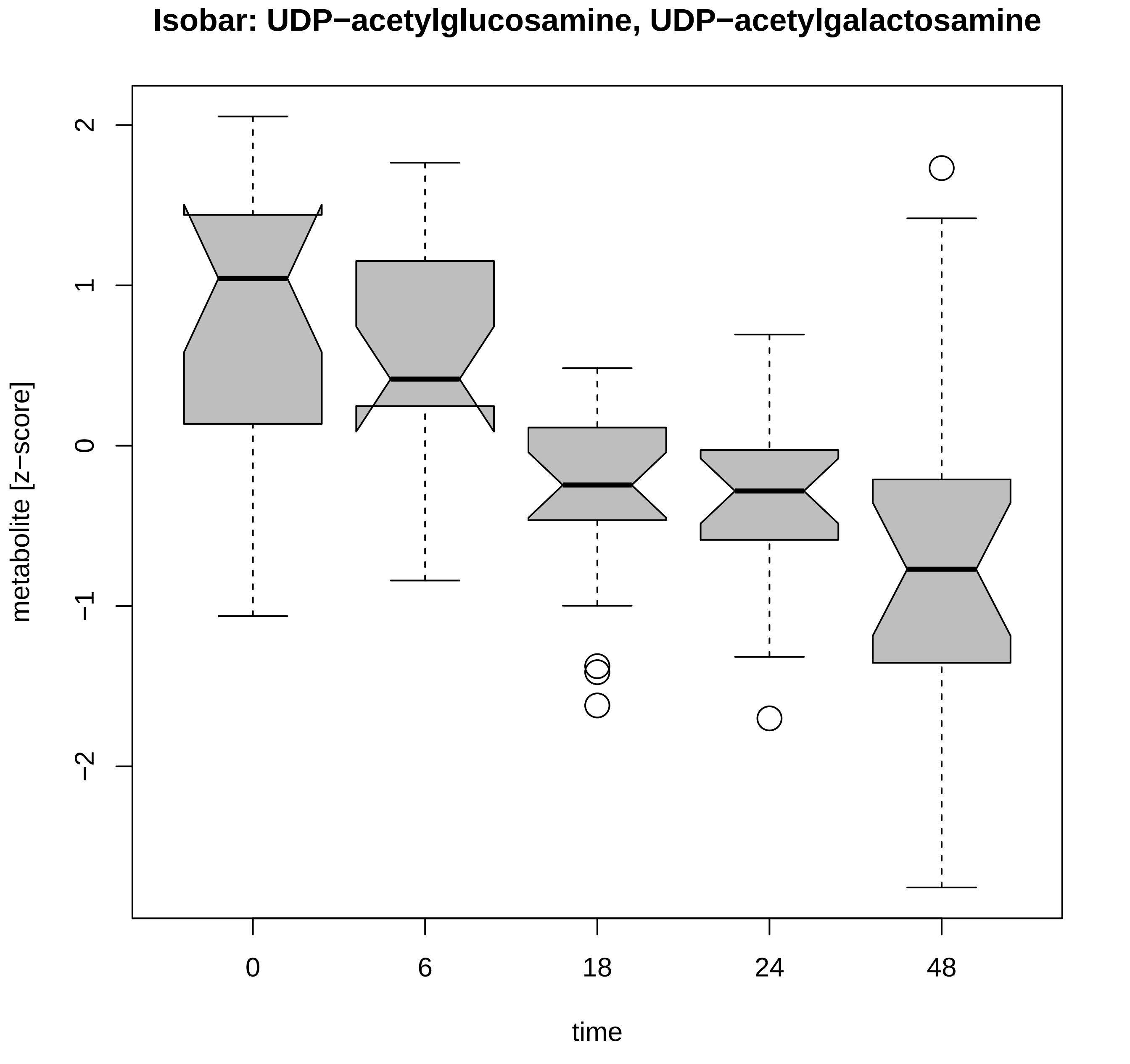

Isobar: UDP−acetylglucosamine, UDP−acetylgalactosamine
metabolite [z−score]
2
1
0
−1
−2
0
6
18
24
48
time

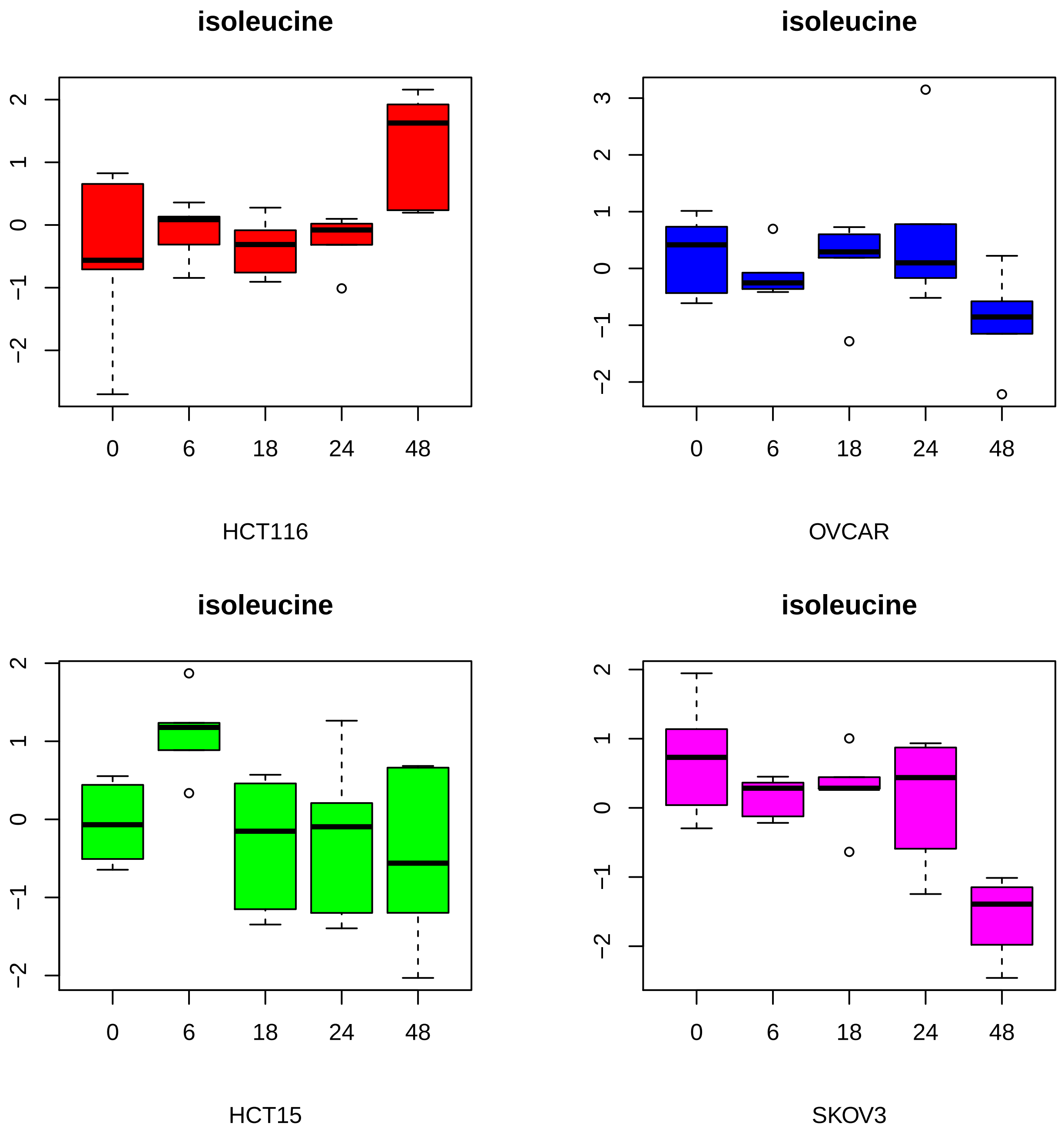
isoleucine
isoleucine
isoleucine
isoleucine
HCT116
OVCAR
HCT15
SKOV3
0
6
18
24
48

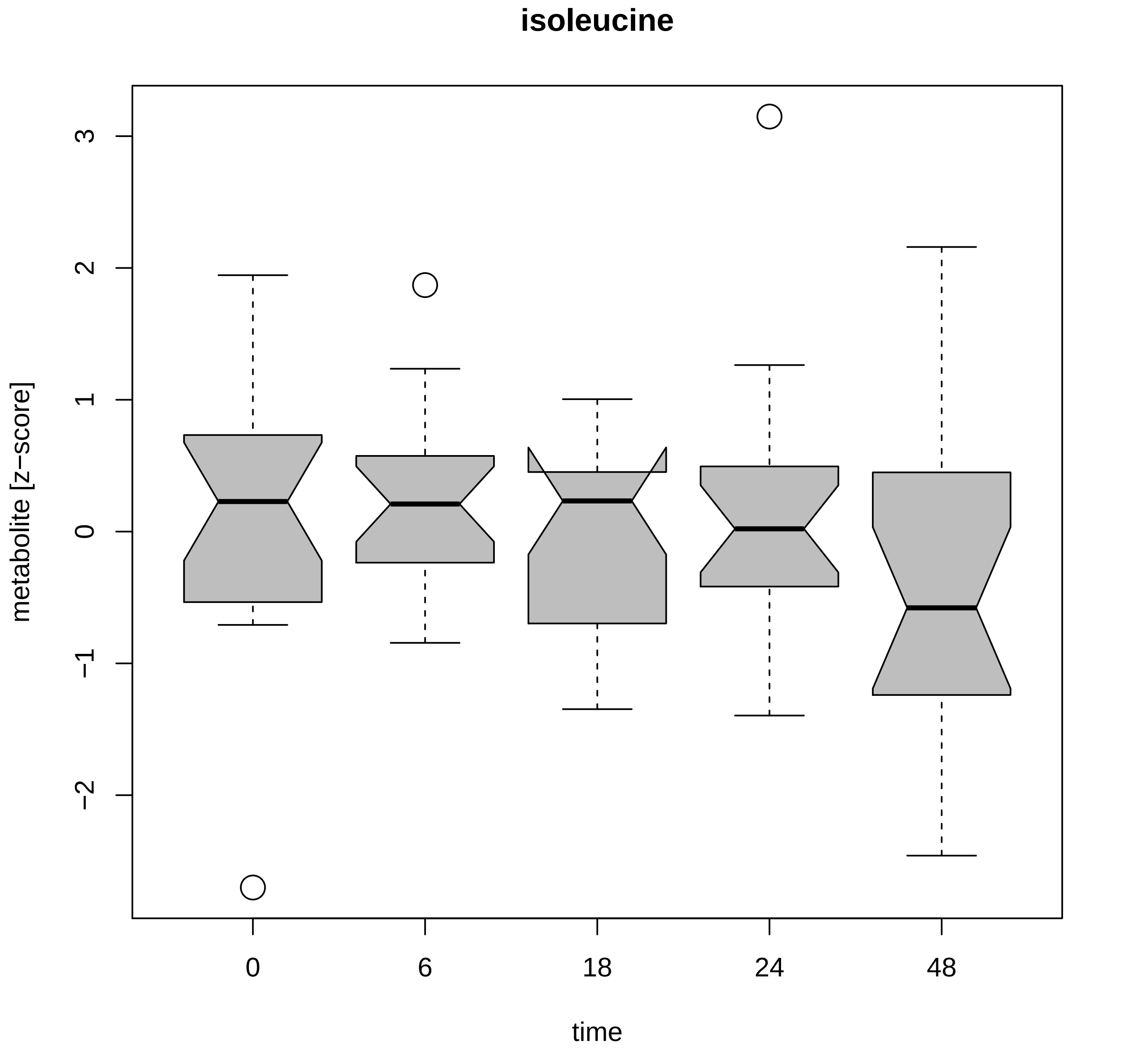

isoleucine
metabolite [z−score]
3
2
1
0
−1
−2
0
6
18
24
48
time

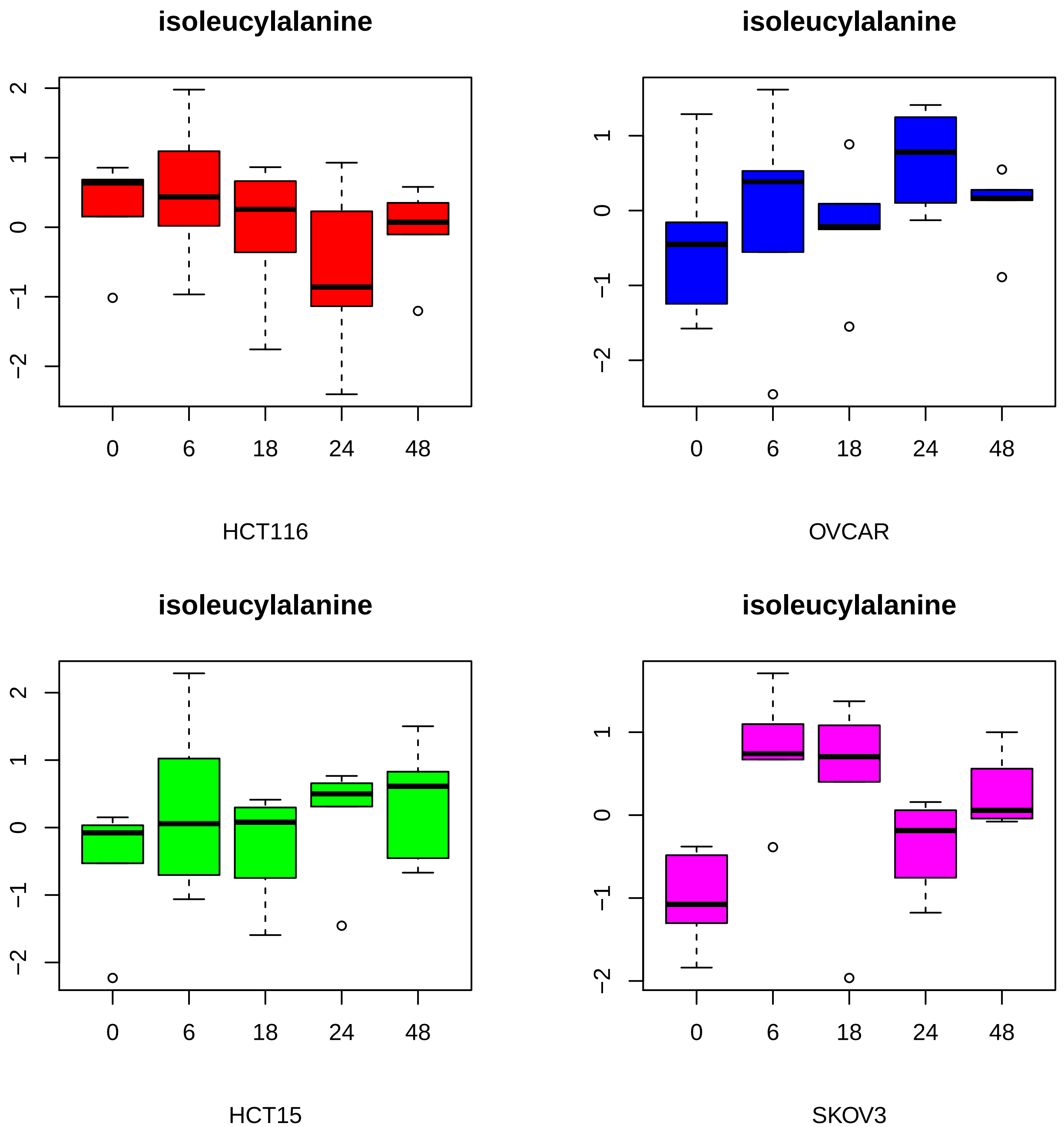
isoleucylalanine
2
1
0
−1
−2
0
6
18
24
48
HCT116
isoleucylalanine
1
0
−1
−2
0
6
18
24
48
OVCAR
isoleucylalanine
2
1
0
−1
−2
0
6
18
24
48
HCT15
isoleucylalanine
1
0
−1
−2
0
6
18
24
48
SKOV3

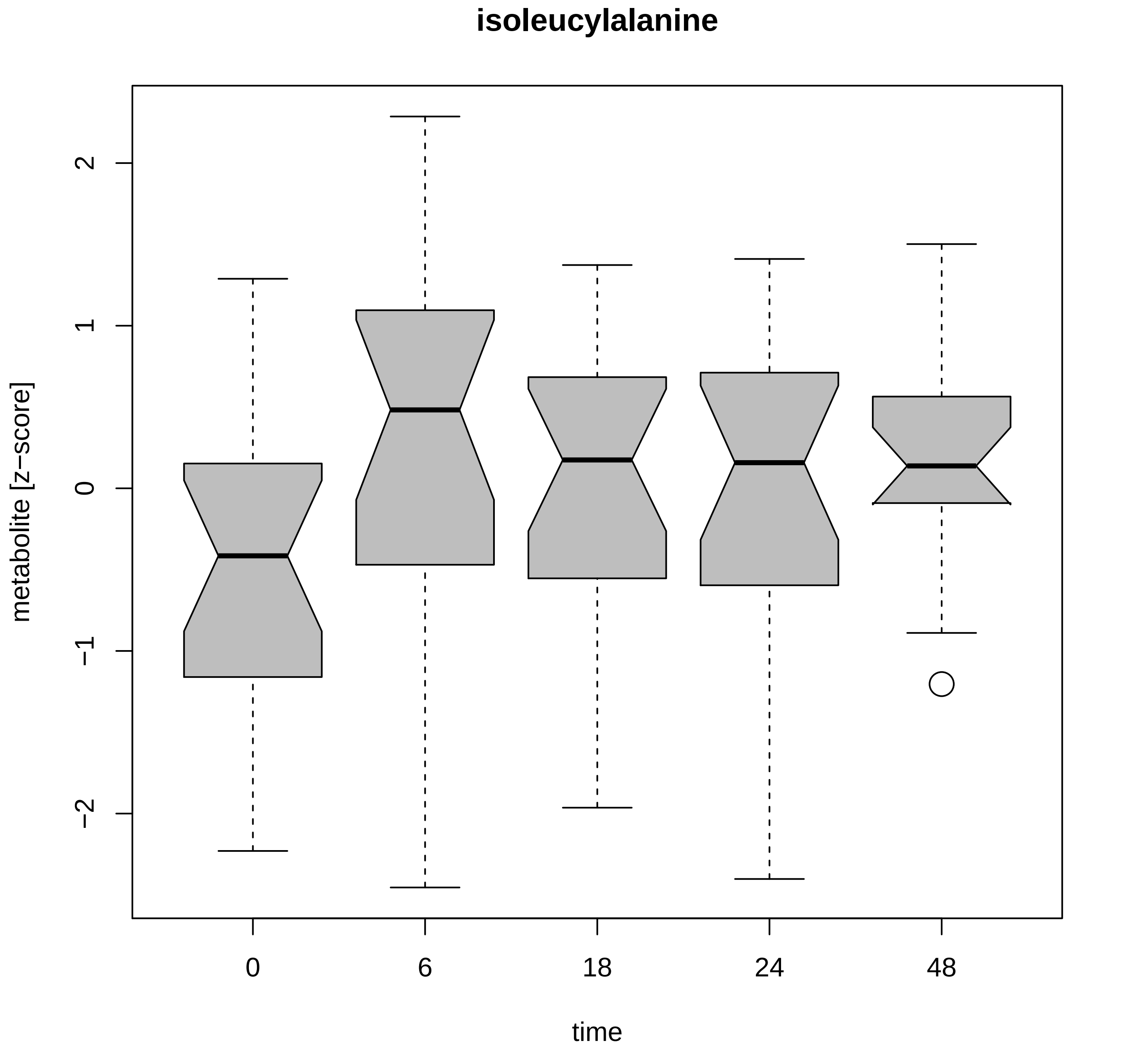
isoleucylalanine
metabolite [z−score]
−2
−1
0
1
2
0
6
18
24
48
time

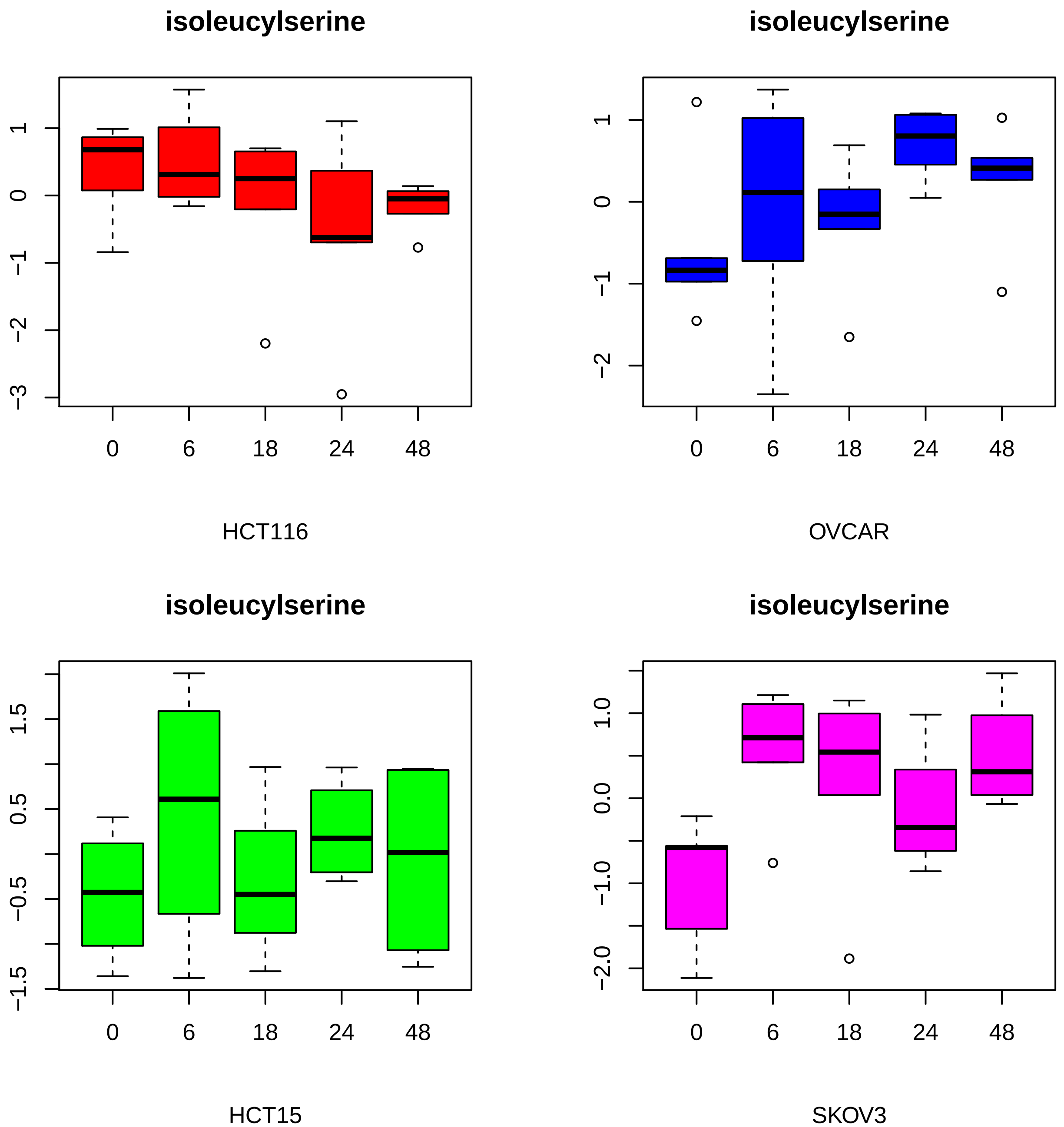
isoleucylserine
1
0
−1
−2
−3
0
6
18
24
48
HCT116
isoleucylserine
1
0
−1
−2
0
6
18
24
48
OVCAR
isoleucylserine
1.5
0.5
−0.5
−1.5
0
6
18
24
48
HCT15
isoleucylserine
1.0
0.0
−1.0
−2.0
0
6
18
24
48
SKOV3

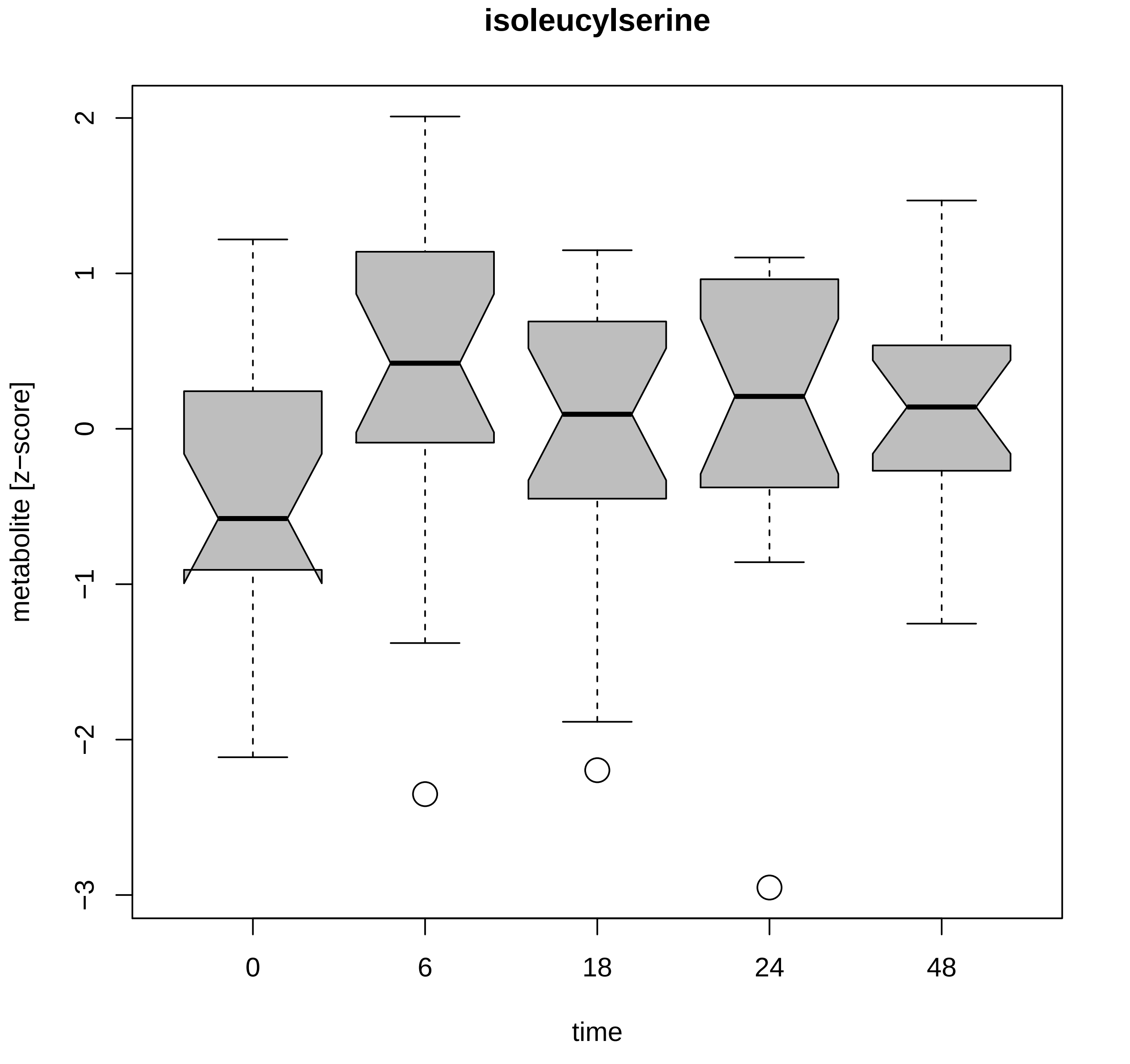
isoleucylserine
metabolite [z−score]
time
2
1
0
−1
−2
−3
0
6
18
24
48

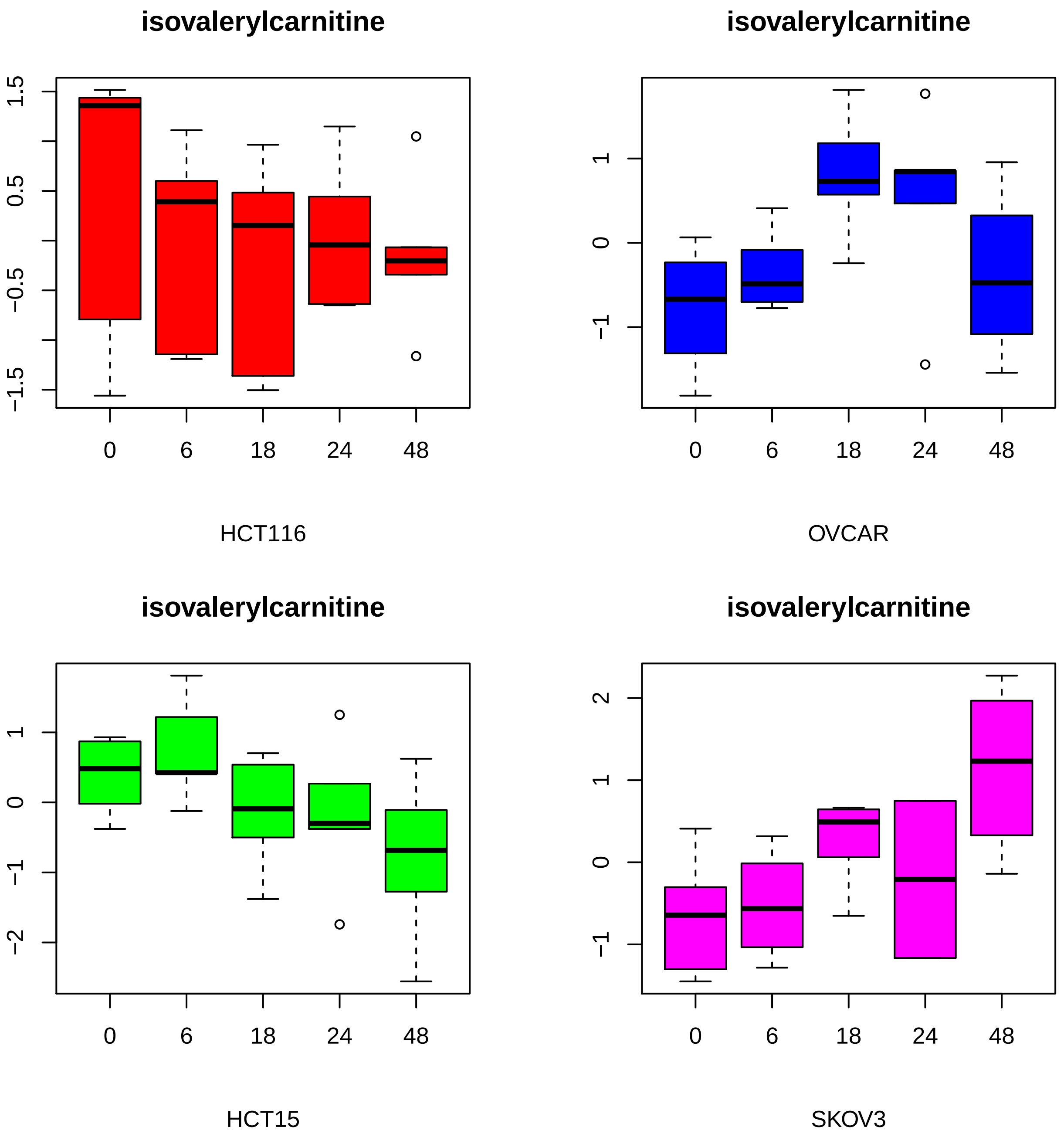

isovalerylcarnitine
1.5
0.5
−0.5
−1.5
0
6
18
24
48
HCT116
isovalerylcarnitine
1
0
−1
0
6
18
24
48
OVCAR
isovalerylcarnitine
1
0
−1
−2
0
6
18
24
48
HCT15
isovalerylcarnitine
2
1
0
−1
0
6
18
24
48
SKOV3

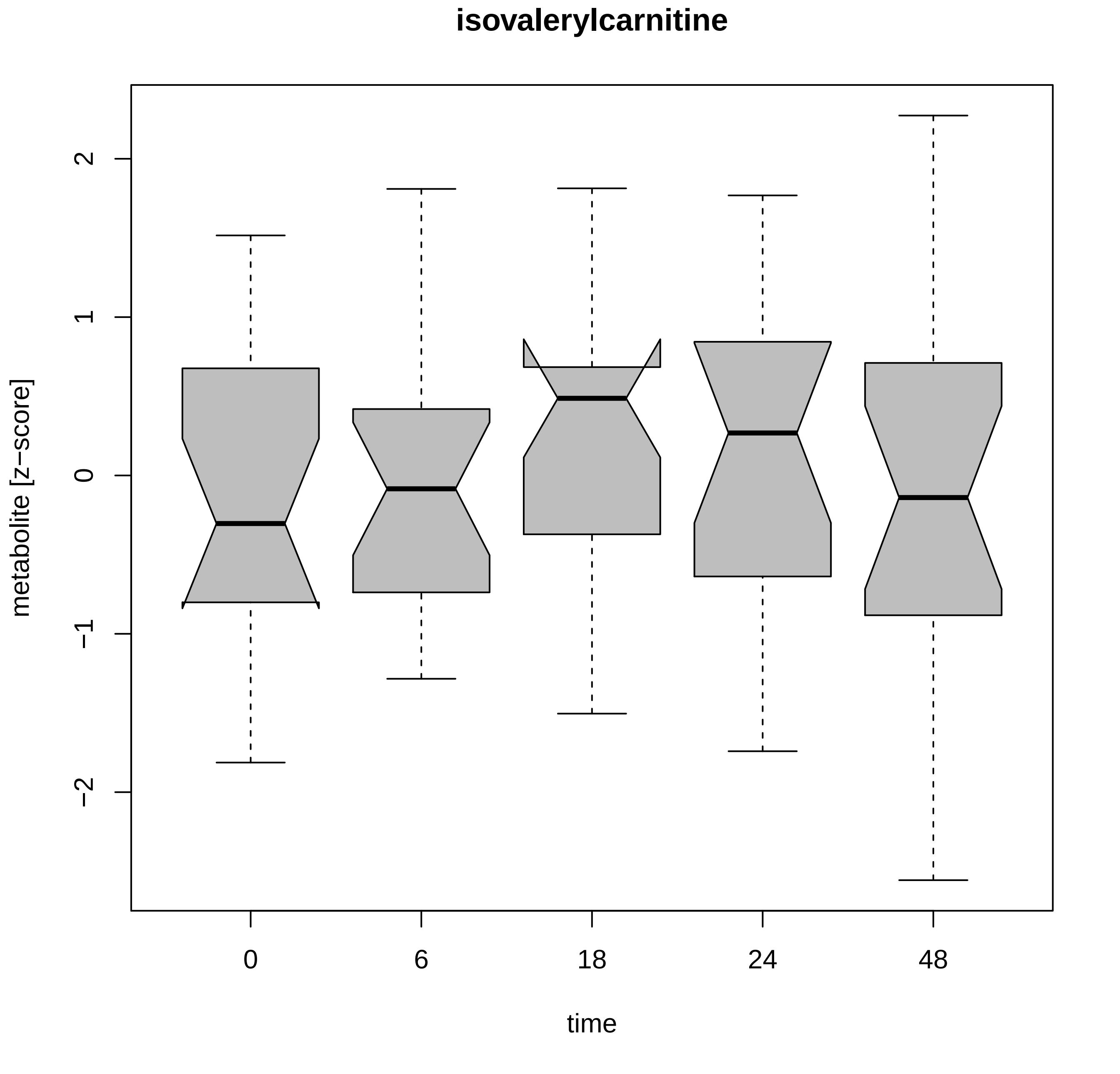

isovalerylcarnitine
metabolite [z−score]
2
1
0
−1
−2
0
6
18
24
48
time

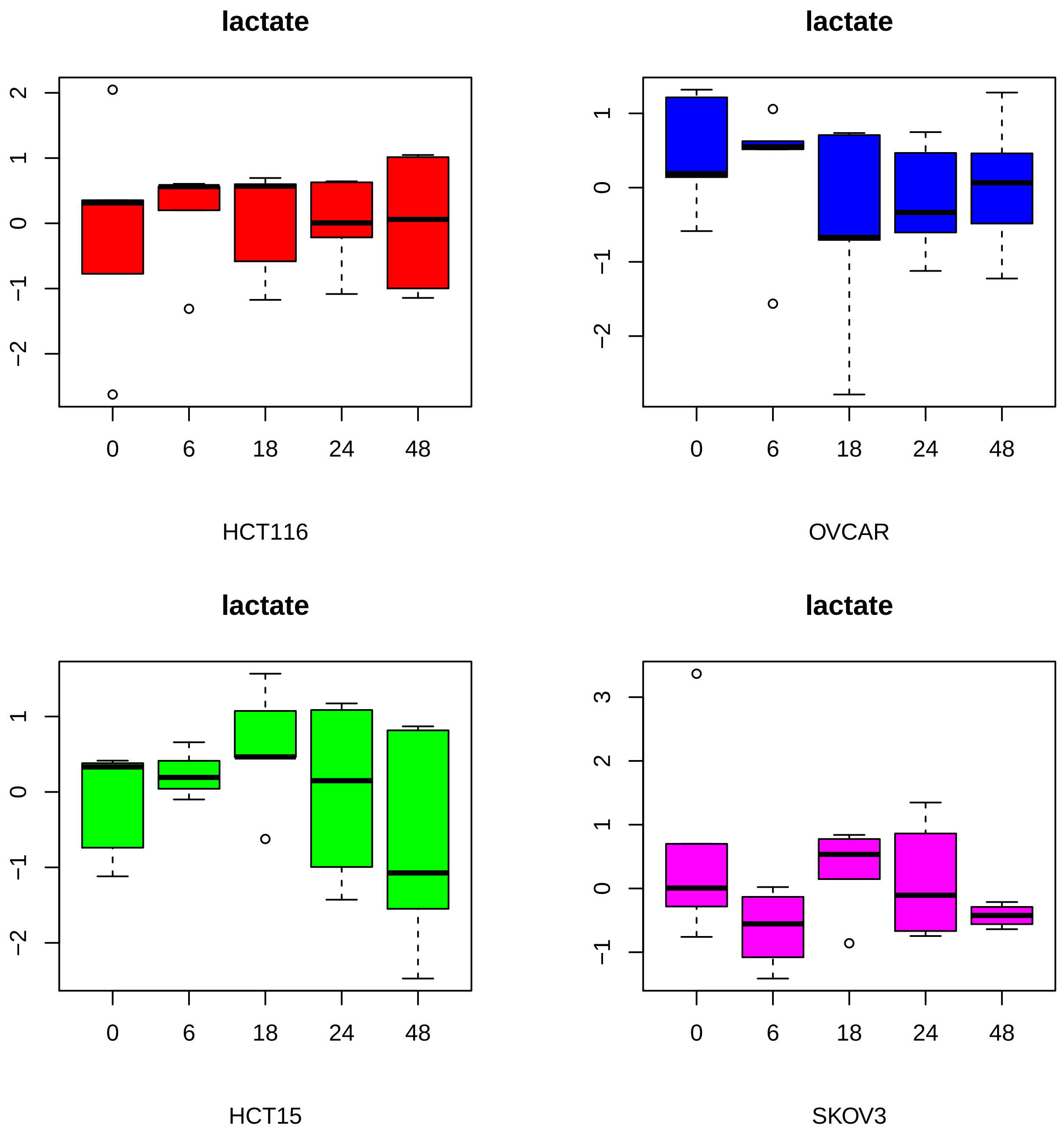

lactate
2
1
0
−1
−2
0
6
18
24
48
HCT116
lactate
1
0
−1
−2
0
6
18
24
48
OVCAR
lactate
1
0
−1
−2
0
6
18
24
48
HCT15
lactate
3
2
1
0
−1
0
6
18
24
48
SKOV3

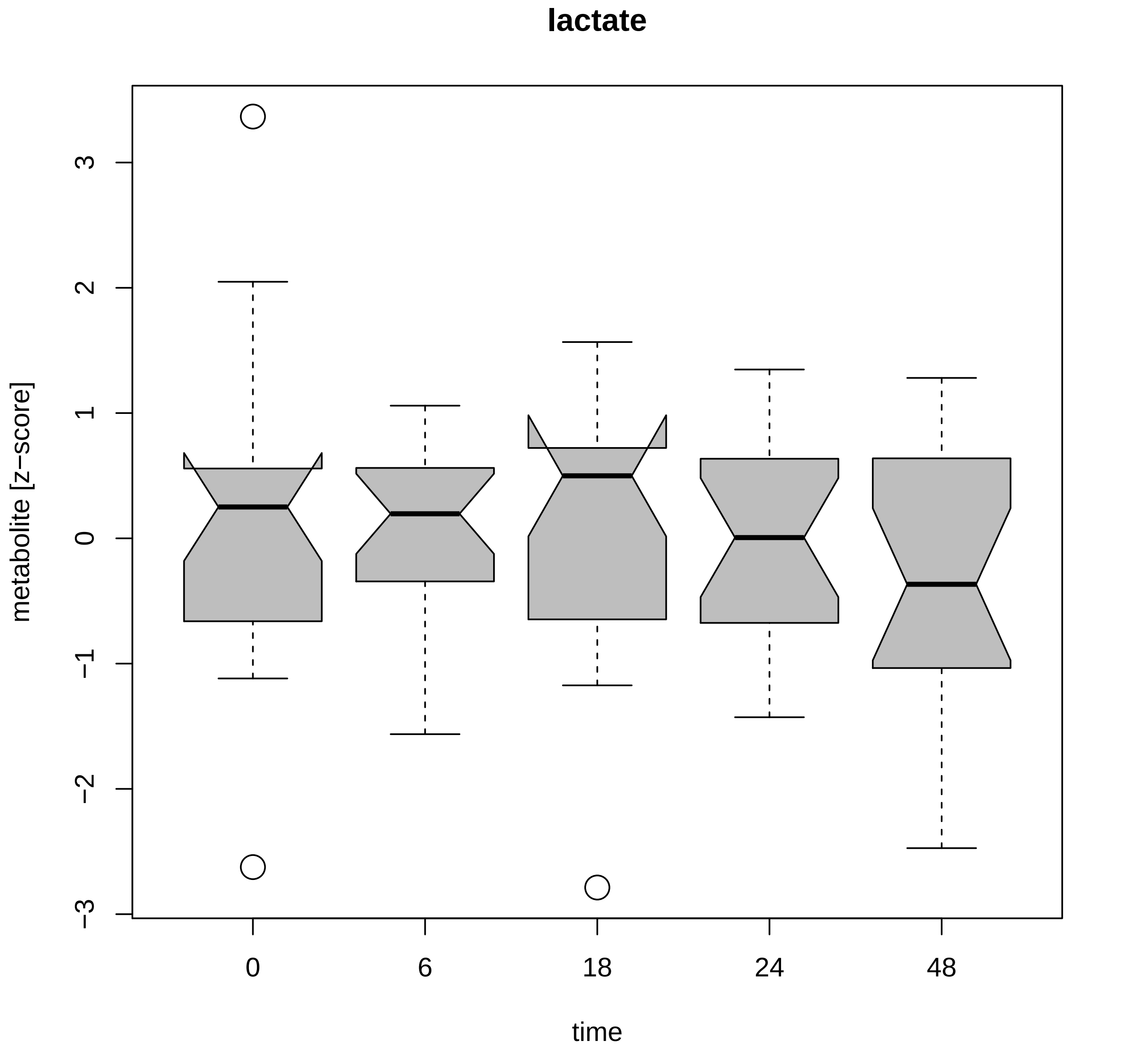

lactate
metabolite [z−score]
3
2
1
0
−1
−2
−3
0
6
18
24
48
time

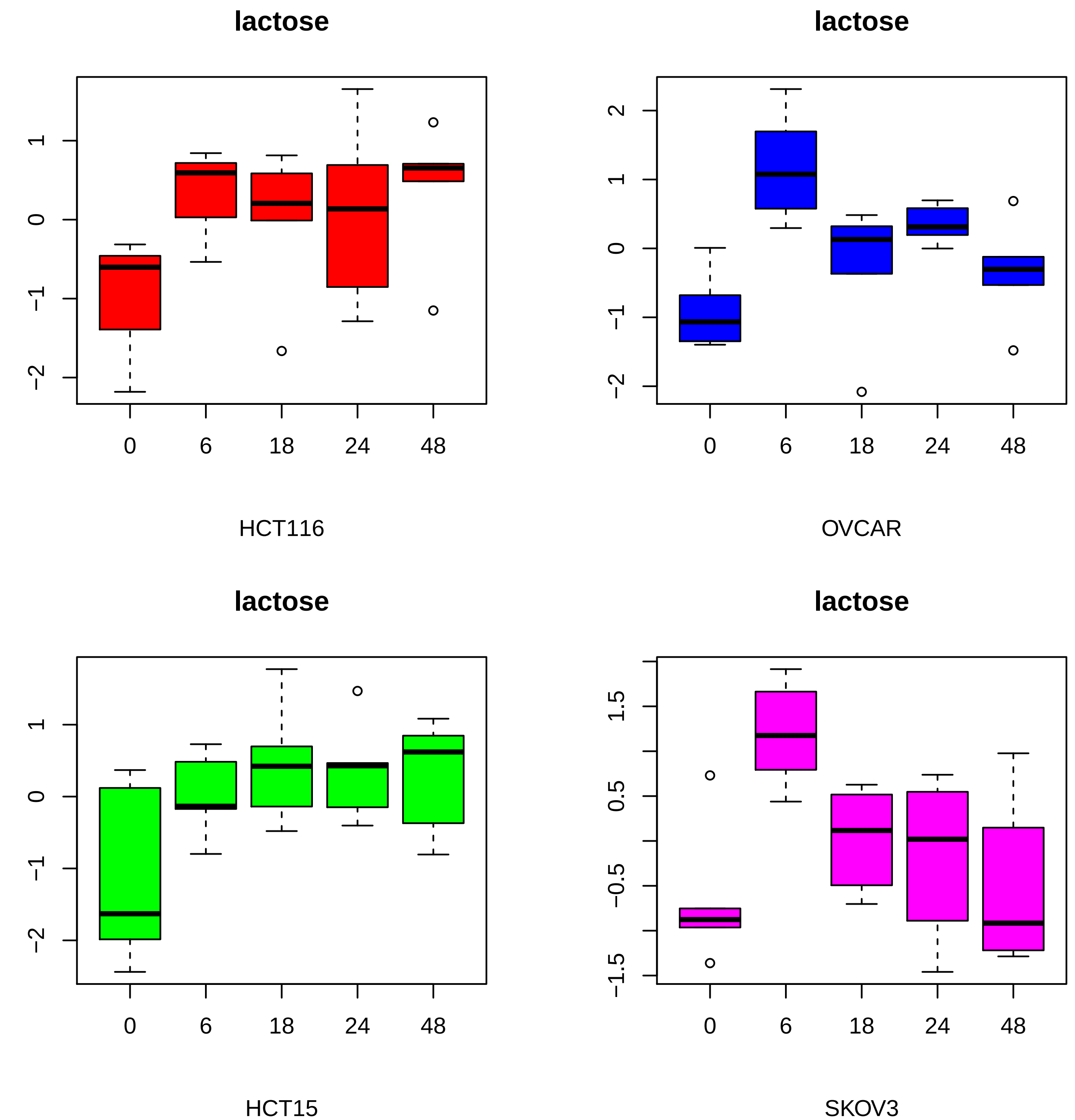
lactose
HCT116
lactose
OVCAR
lactose
HCT15
lactose
SKOV3
0
6
18
24
48

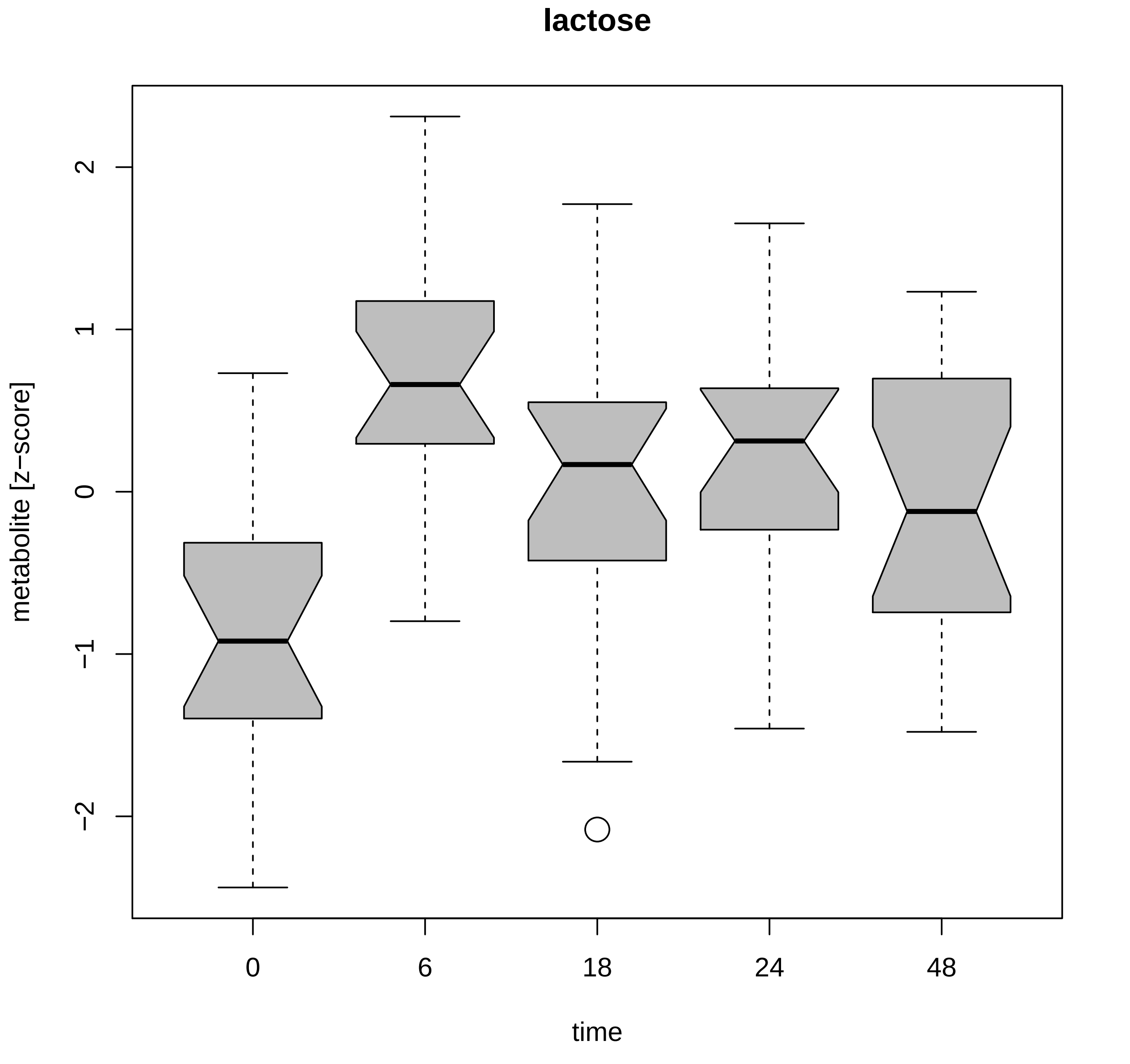
lactose
metabolite [z−score]
2
1
0
−1
−2
0
6
18
24
48
time

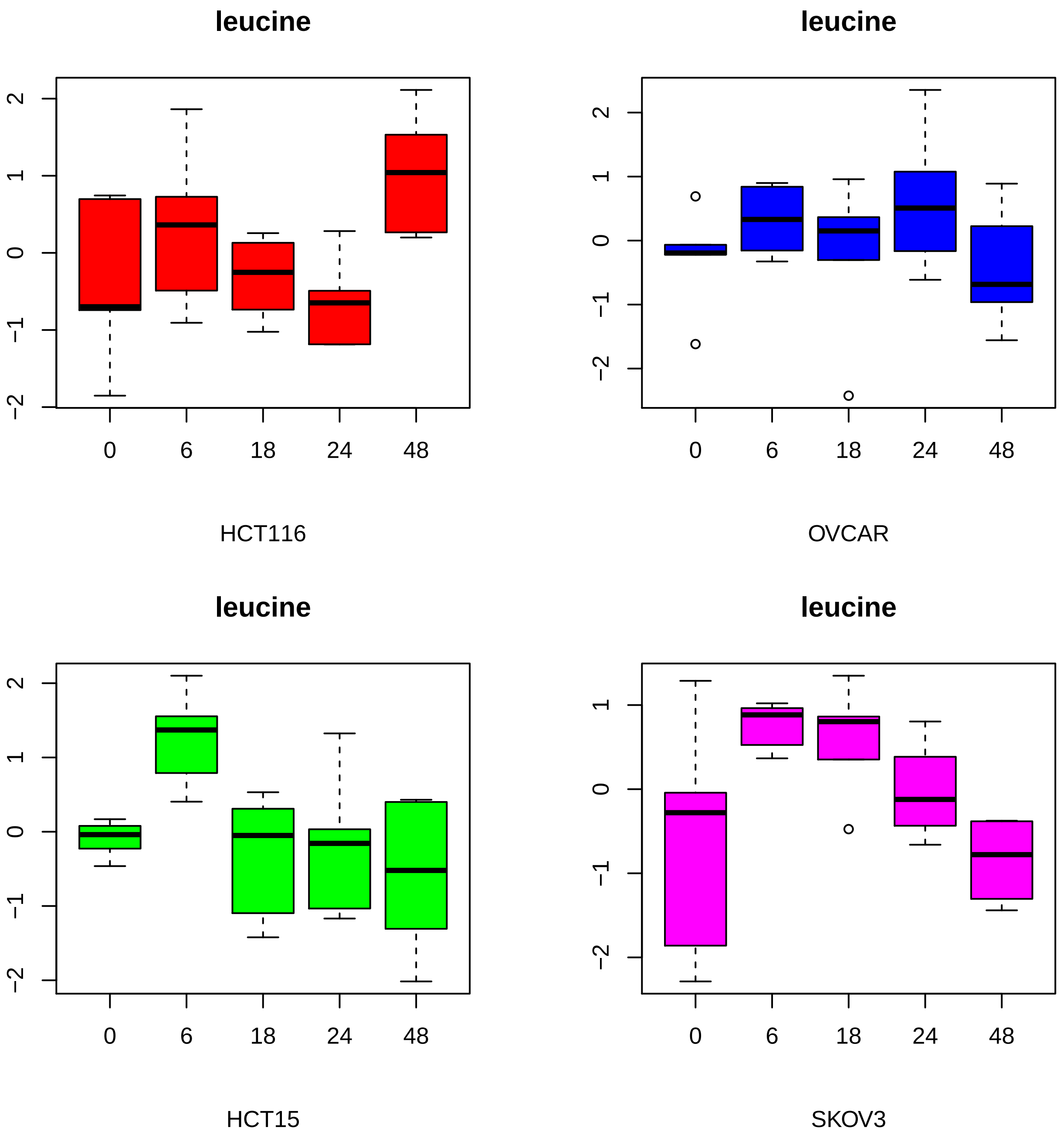
leucine
2
1
0
−1
−2
0
6
18
24
48
HCT116
leucine
2
1
0
−1
−2
0
6
18
24
48
OVCAR
leucine
2
1
0
−1
−2
0
6
18
24
48
HCT15
leucine
1
0
−1
−2
0
6
18
24
48
SKOV3

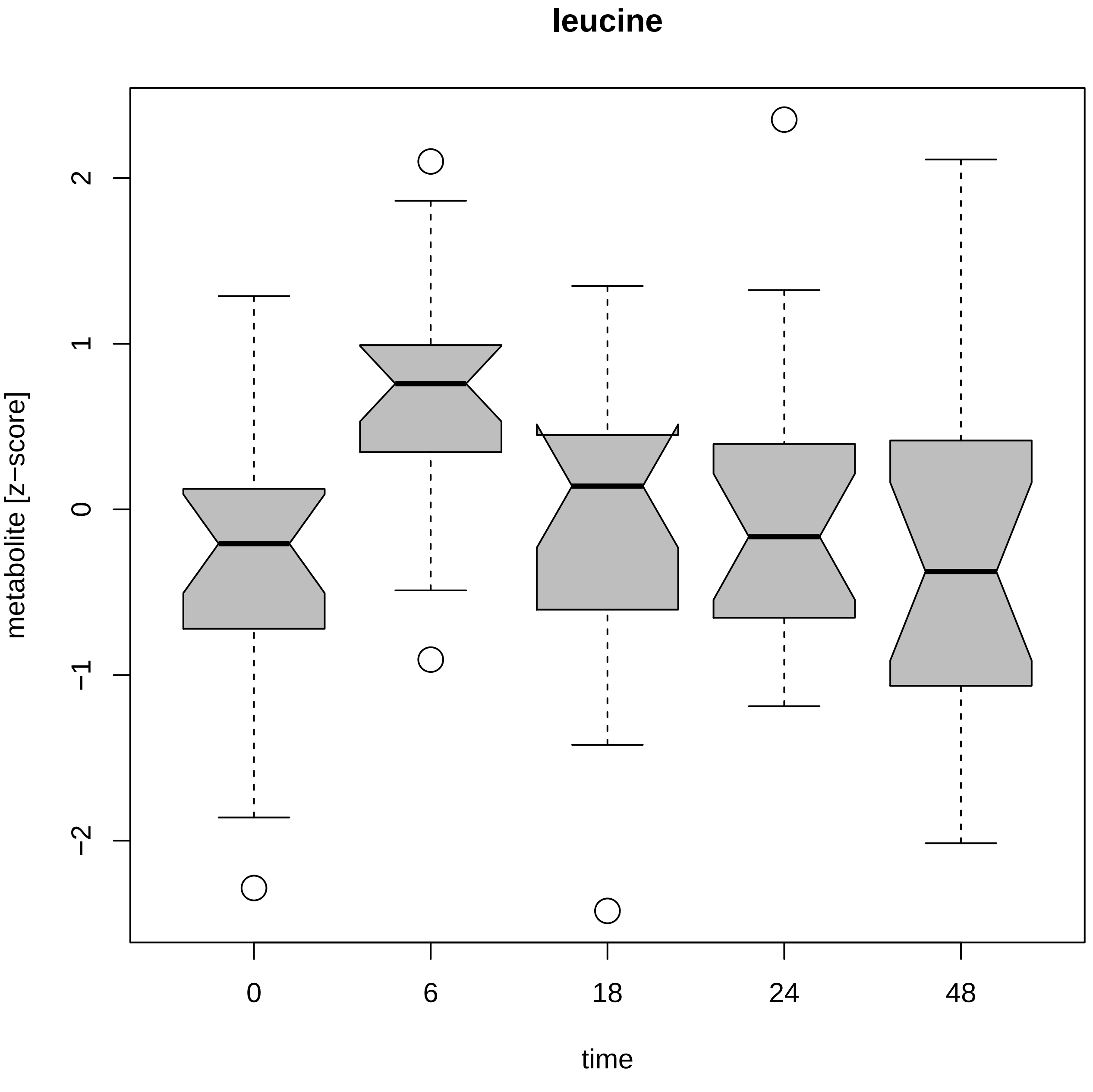

leucine
metabolite [z−score]
2
1
0
−1
−2
0
6
18
24
48
time

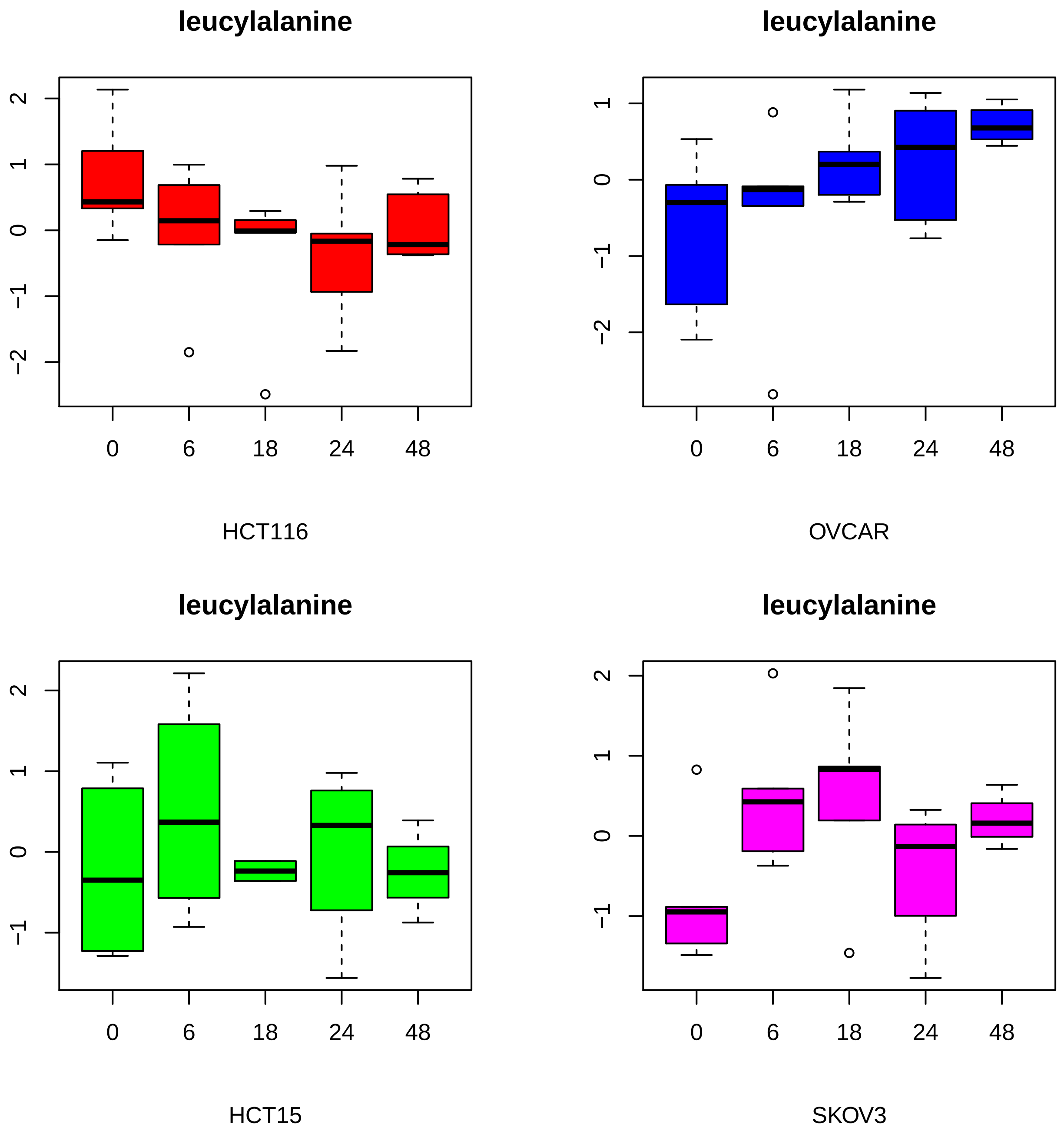
leucylalanine
HCT116
OVCAR
HCT15
SKOV3
0
6
18
24
48

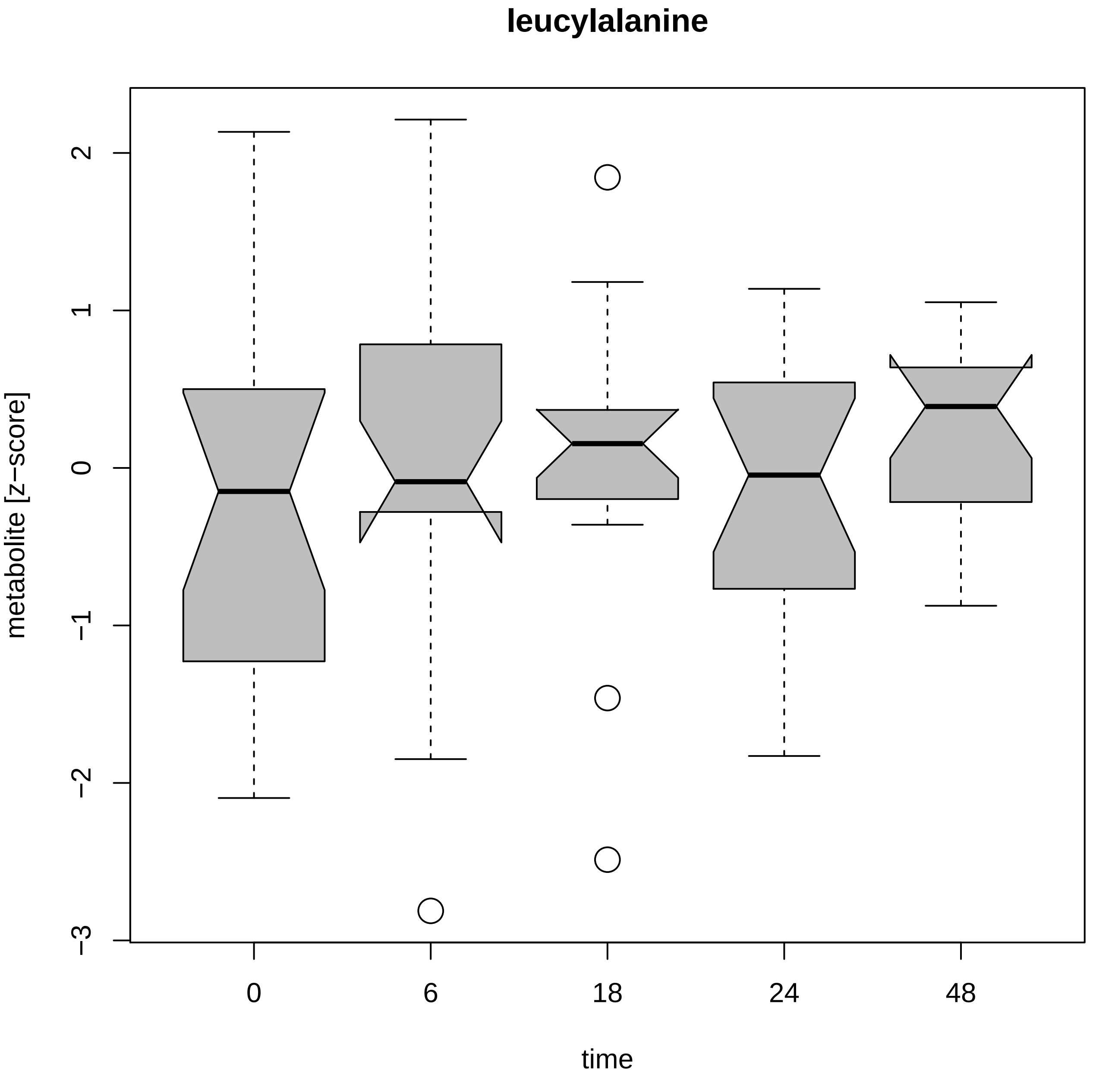

leucylalanine
metabolite [z−score]
2
1
0
−1
−2
−3
0
6
18
24
48
time

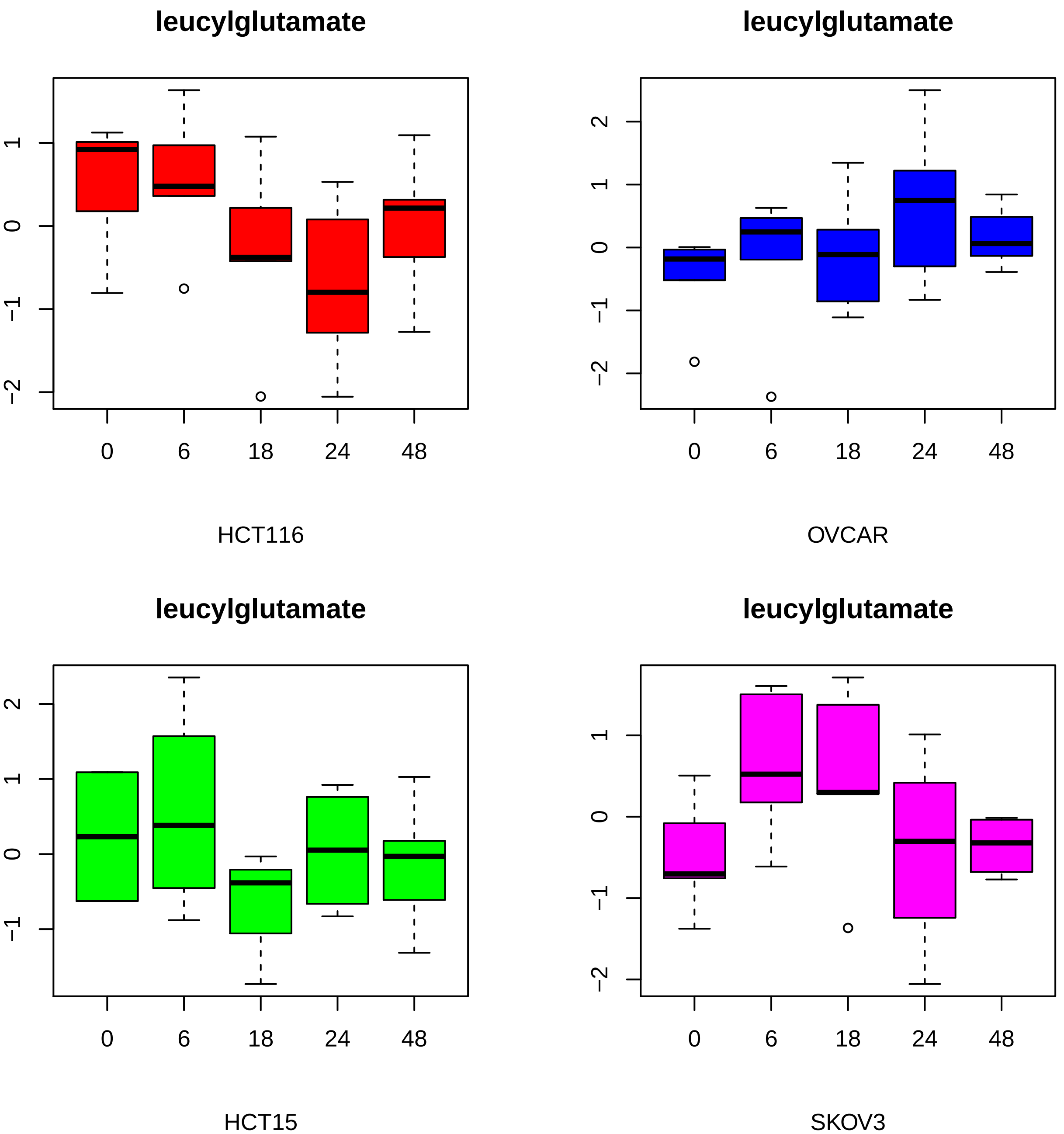
leucylglutamate
1
0
−1
−2
0
6
18
24
48
HCT116
leucylglutamate
2
1
0
−1
−2
0
6
18
24
48
OVCAR
leucylglutamate
2
1
0
−1
0
6
18
24
48
HCT15
leucylglutamate
1
0
−1
−2
0
6
18
24
48
SKOV3

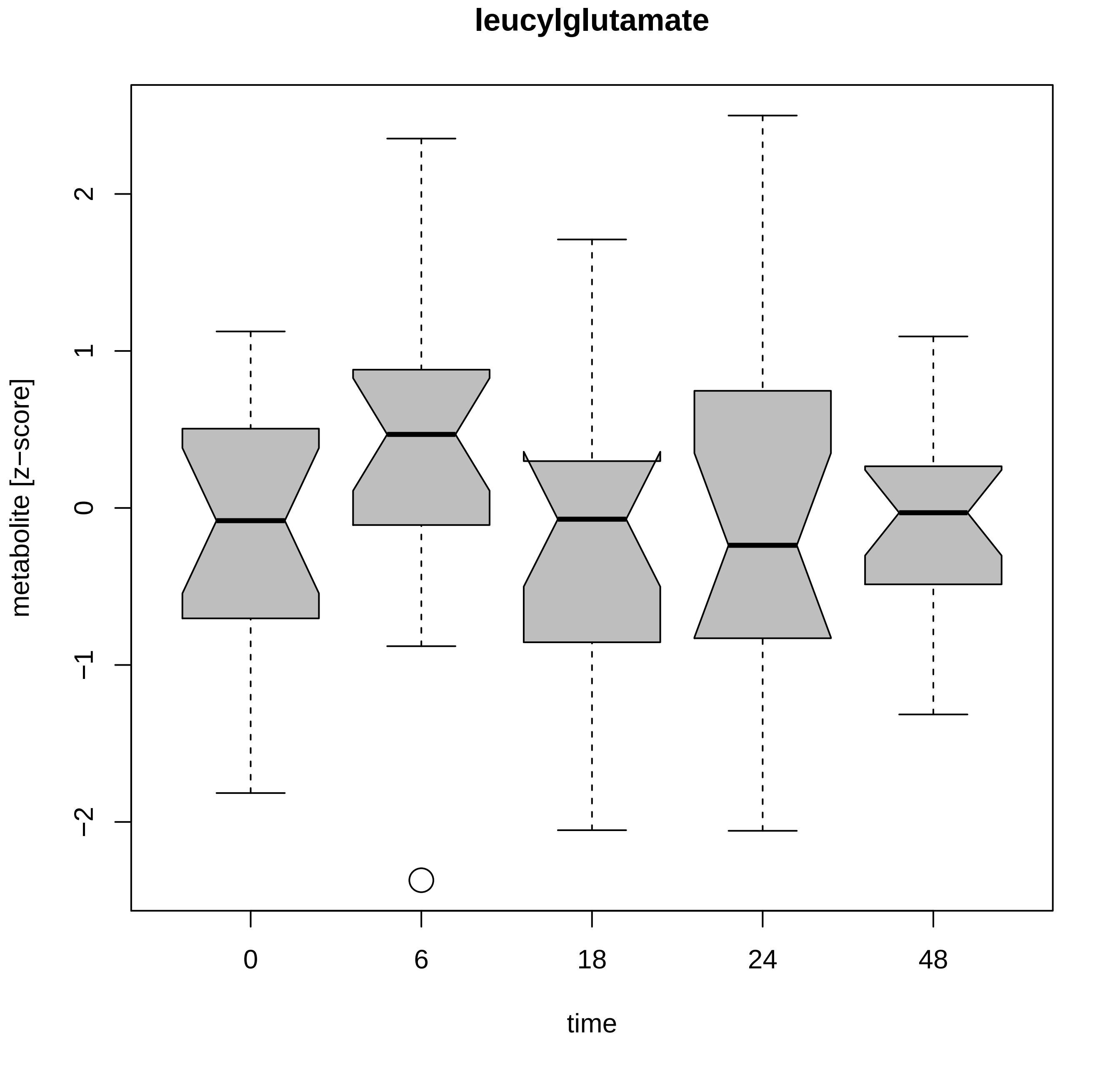
leucylglutamate
metabolite [z−score]
time
0
6
18
24
48
−2
−1
0
1
2

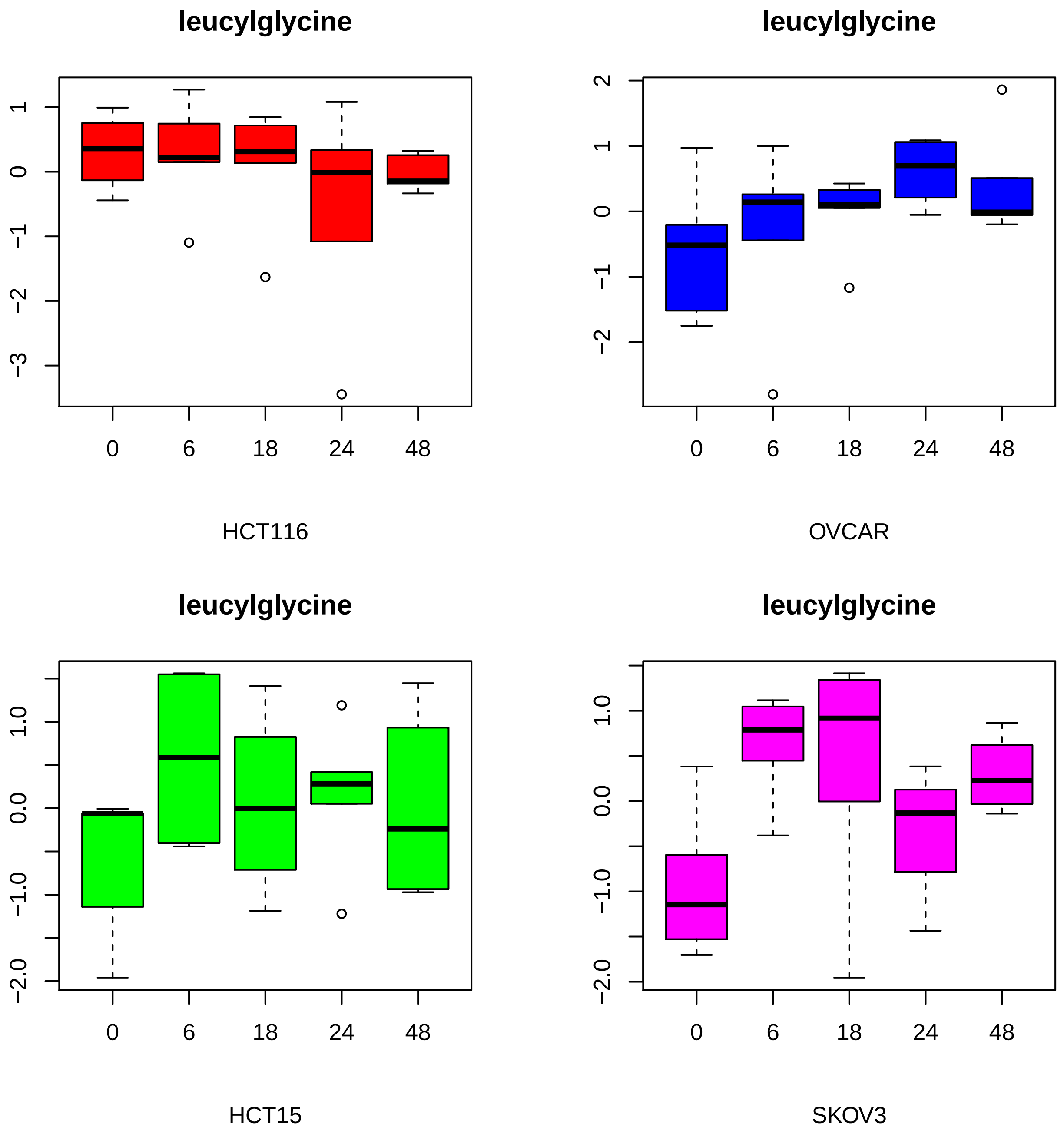

leucylglycine
1
0
−1
−2
−3
0
6
18
24
48
HCT116
leucylglycine
2
1
0
−1
−2
0
6
18
24
48
OVCAR
leucylglycine
1.0
0.0
−1.0
−2.0
0
6
18
24
48
HCT15
leucylglycine
1.0
0.0
−1.0
−2.0
0
6
18
24
48
SKOV3

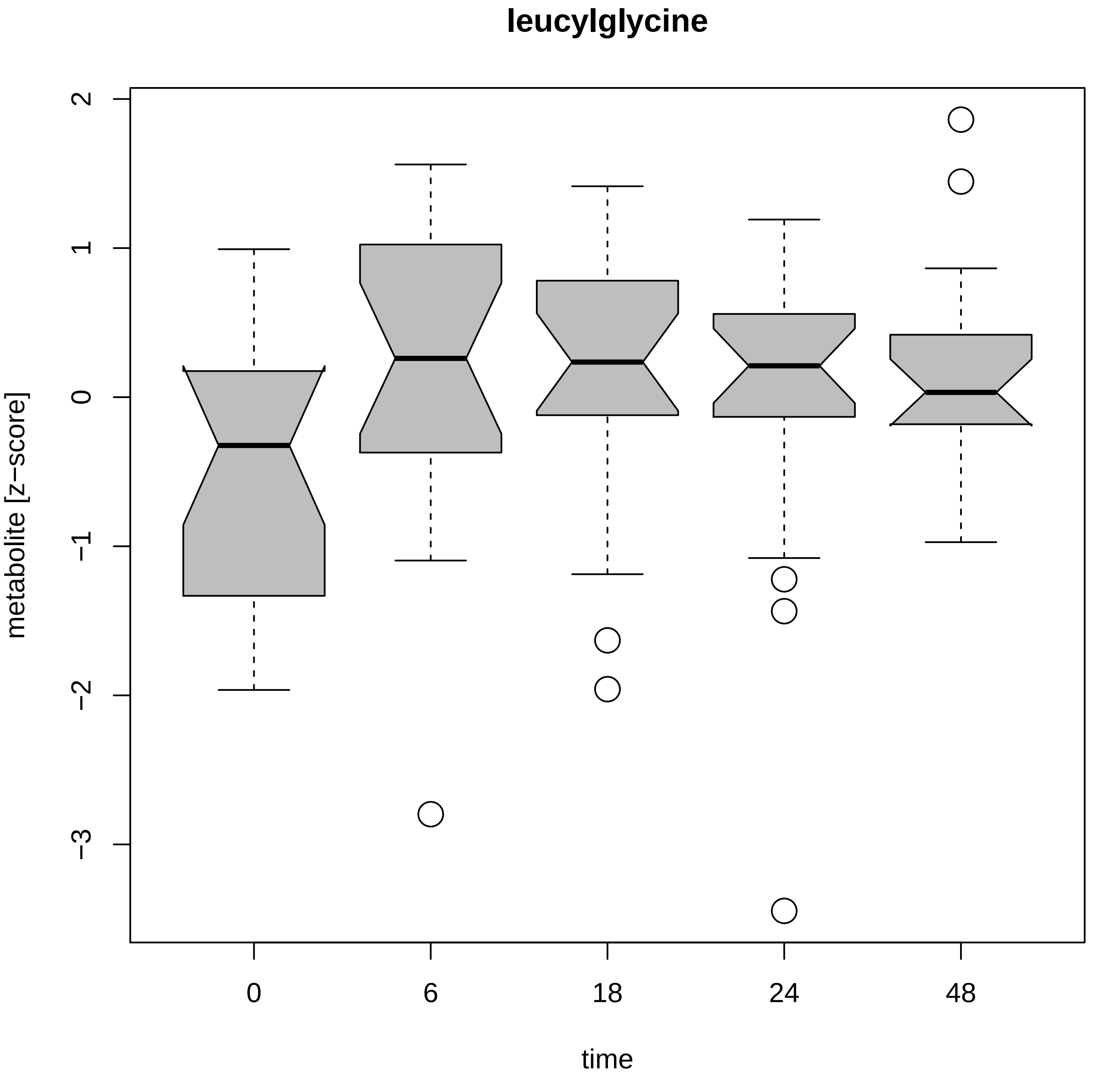
leucylglycine
2
1
0
−1
−2
−3
metabolite [z−score]
0
6
18
24
48
time

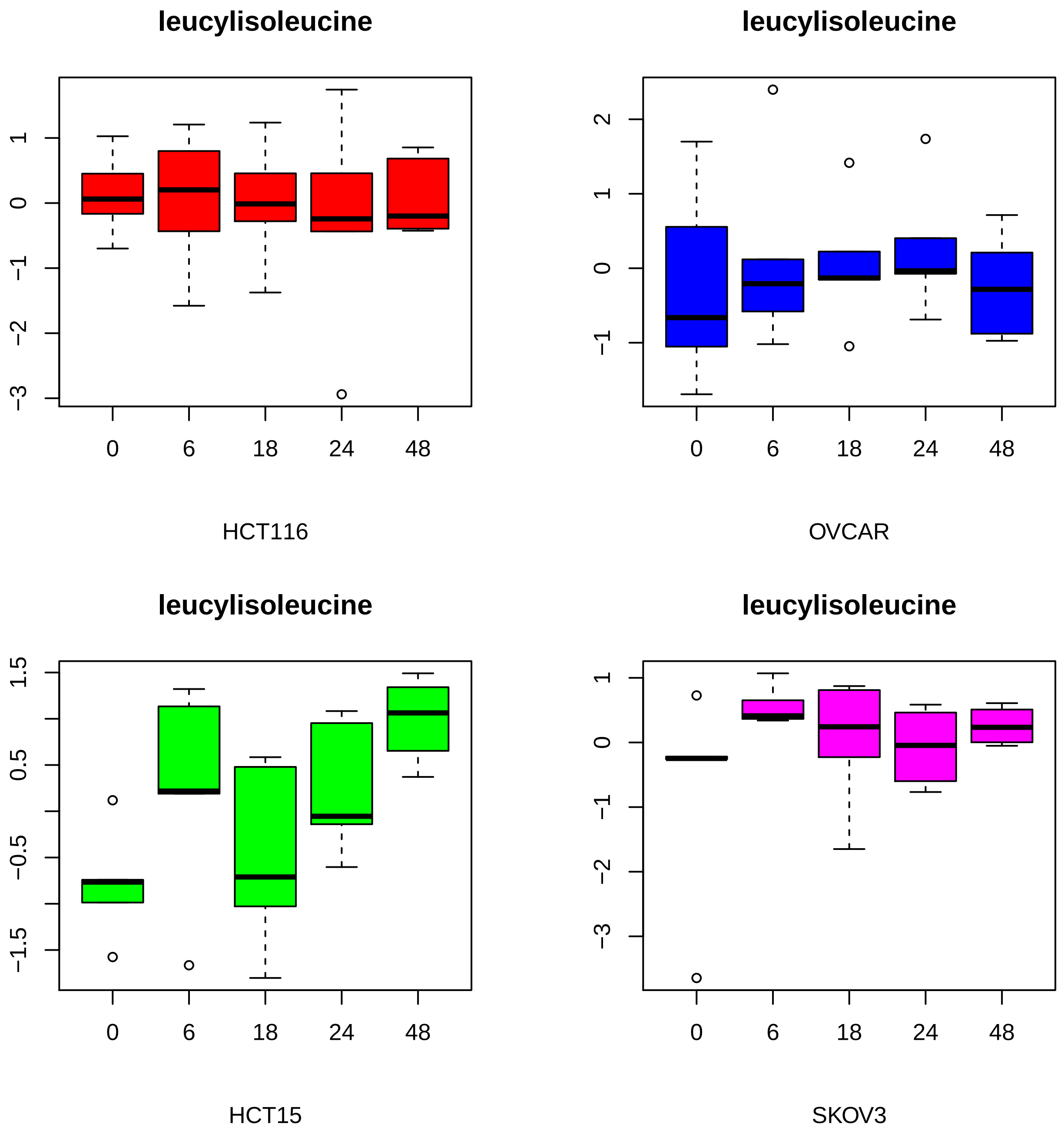

leucylisoleucine
1
0
−1
−2
−3
0
6
18
24
48
HCT116
leucylisoleucine
2
1
0
−1
0
6
18
24
48
OVCAR
leucylisoleucine
1.5
0.5
−0.5
−1.5
0
6
18
24
48
HCT15
leucylisoleucine
1
0
−1
−2
−3
0
6
18
24
48
SKOV3

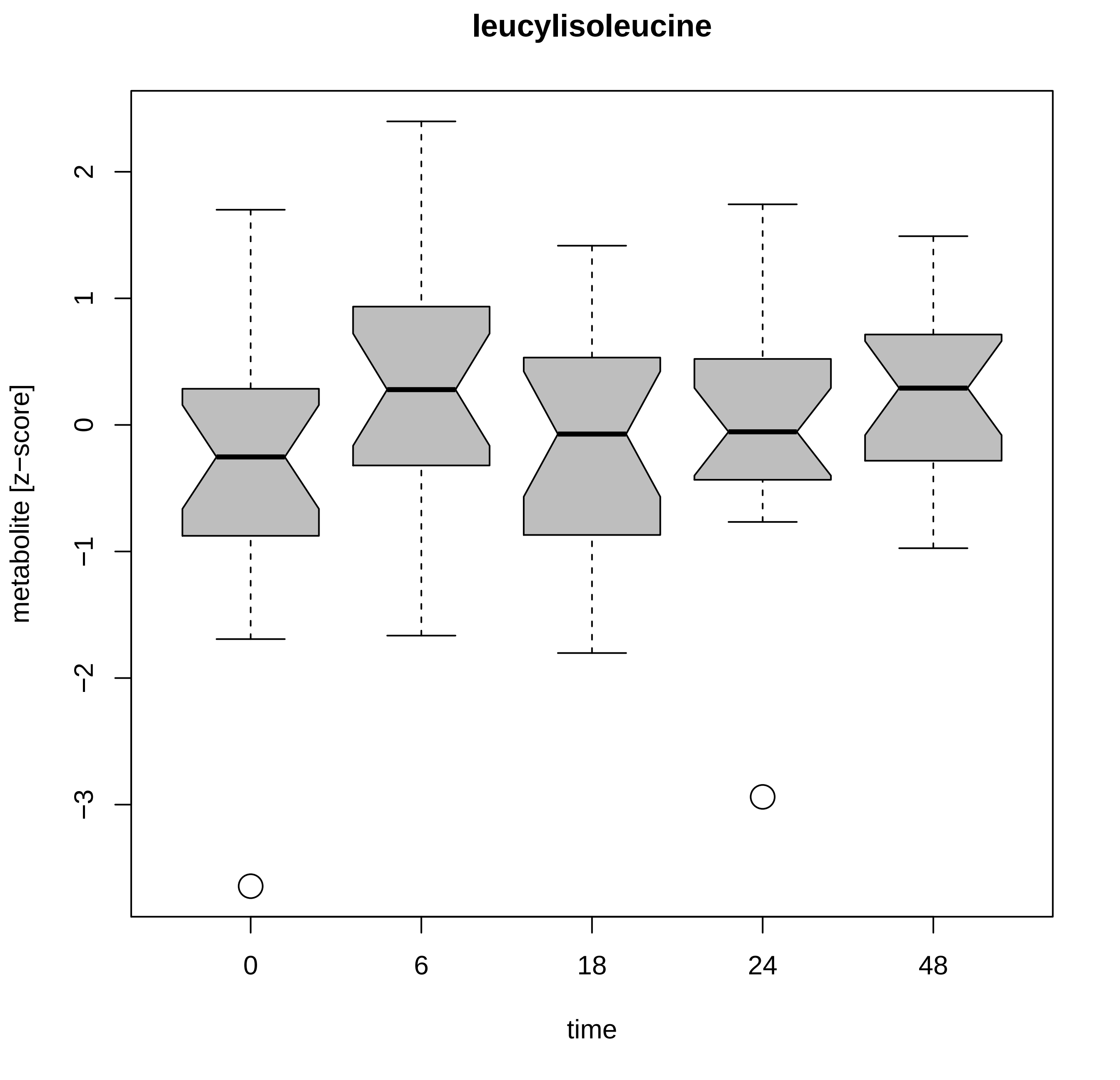

leucylisoleucine
metabolite [z−score]
2
1
0
−1
−2
−3
0
6
18
24
48
time

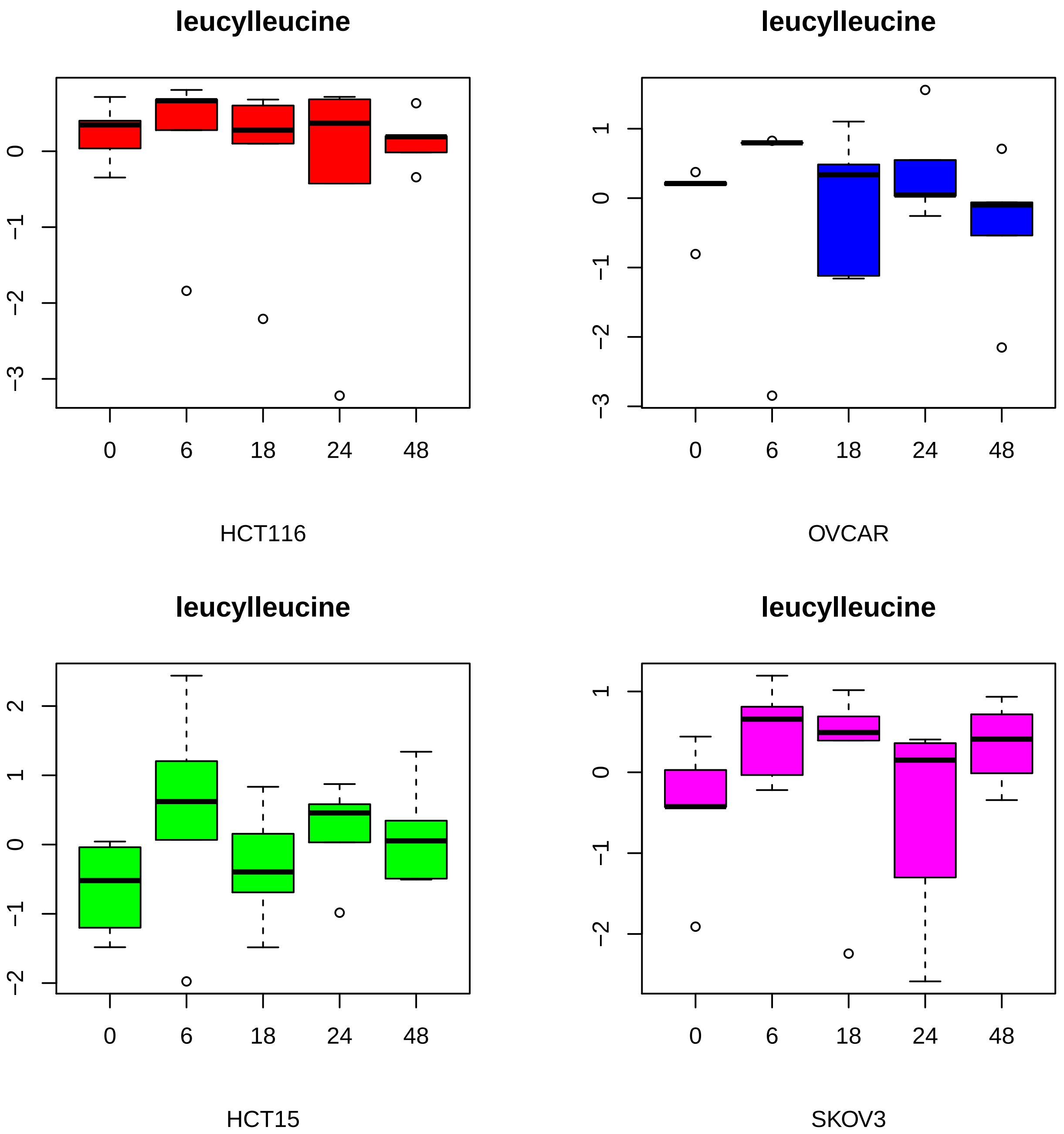
leucylleucine
0
−1
−2
−3
0
6
18
24
48
HCT116
leucylleucine
1
0
−1
−2
−3
0
6
18
24
48
OVCAR
leucylleucine
2
1
0
−1
−2
0
6
18
24
48
HCT15
leucylleucine
1
0
−1
−2
0
6
18
24
48
SKOV3

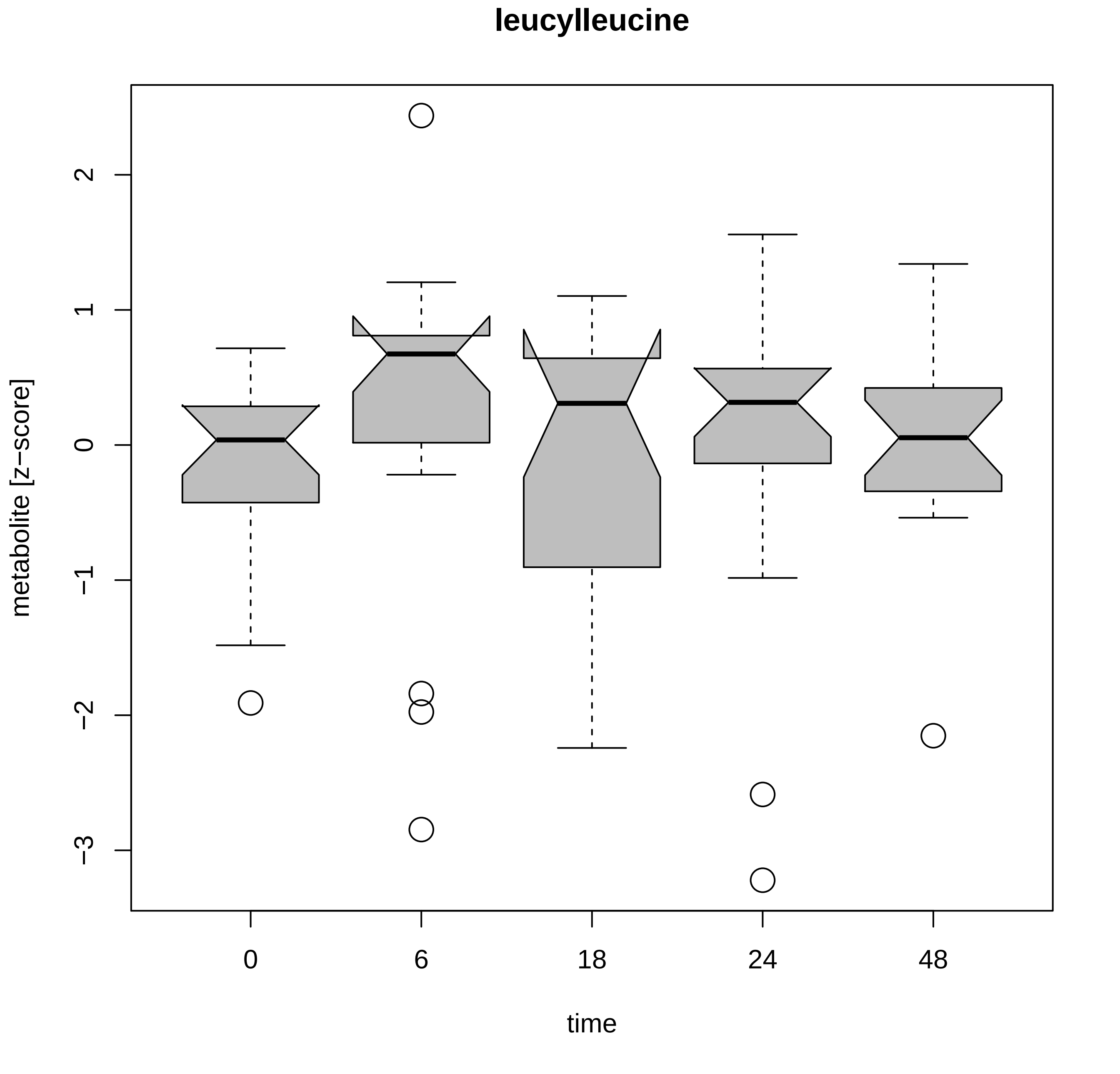

leucylleucine
metabolite [z−score]
2
1
0
−1
−2
−3
0
6
18
24
48
time

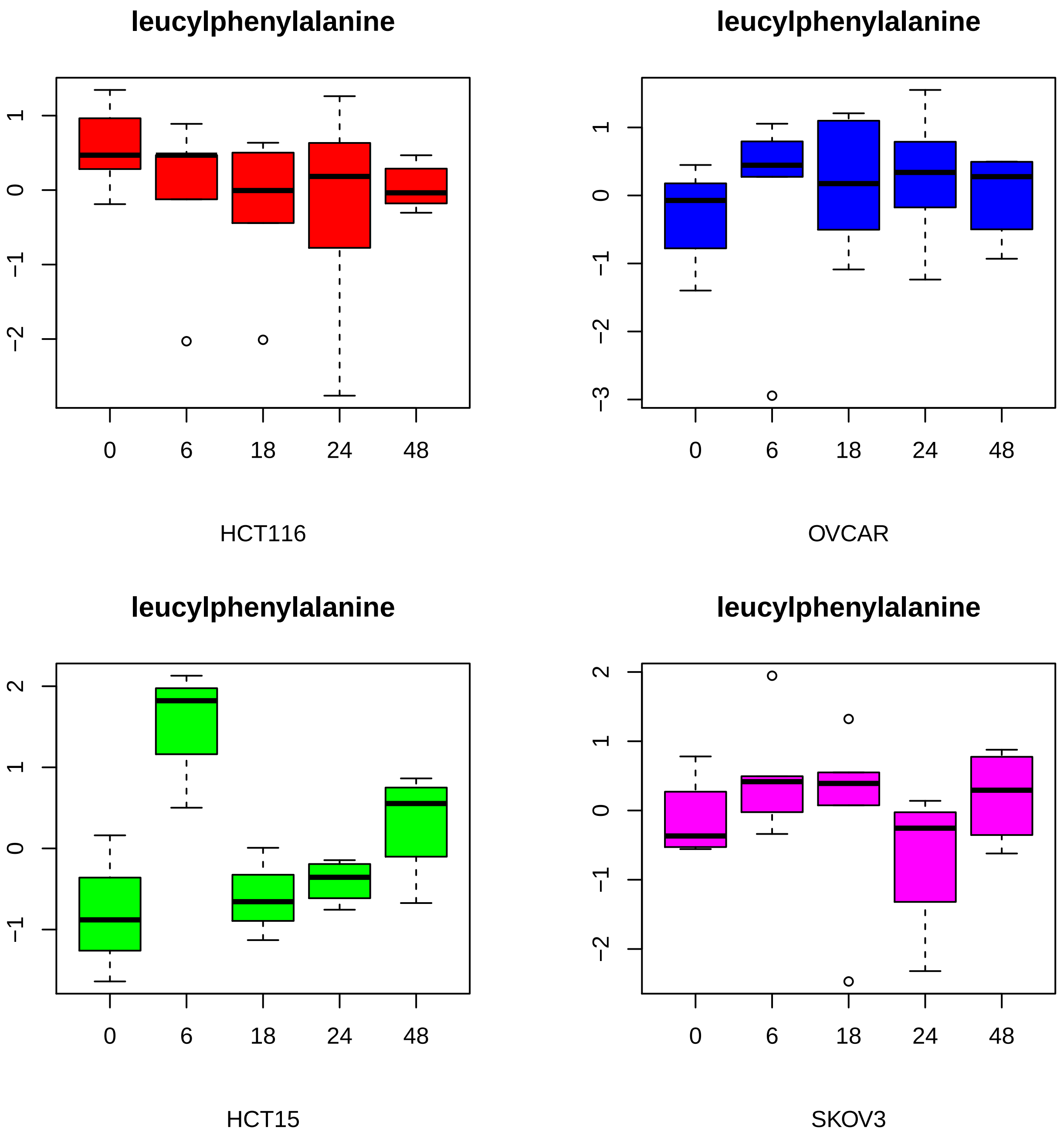
leucylphenylalanine
1
0
−1
−2
0
6
18
24
48
HCT116
leucylphenylalanine
1
0
−1
−2
−3
0
6
18
24
48
OVCAR
leucylphenylalanine
2
1
0
−1
0
6
18
24
48
HCT15
leucylphenylalanine
2
1
0
−1
−2
0
6
18
24
48
SKOV3

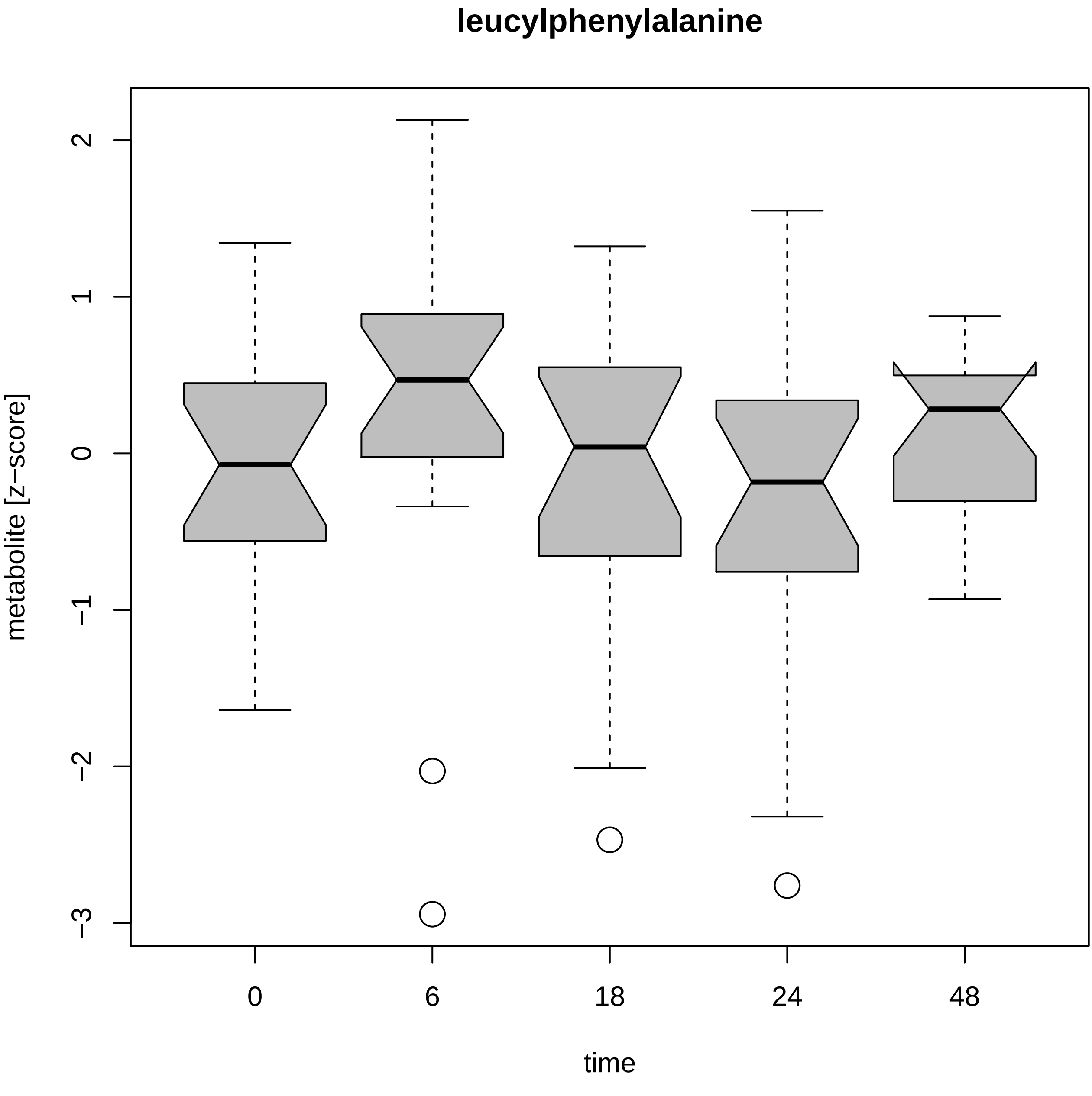
leucylphenylalanine
metabolite [z−score]
2
1
0
−1
−2
−3
0
6
18
24
48
time

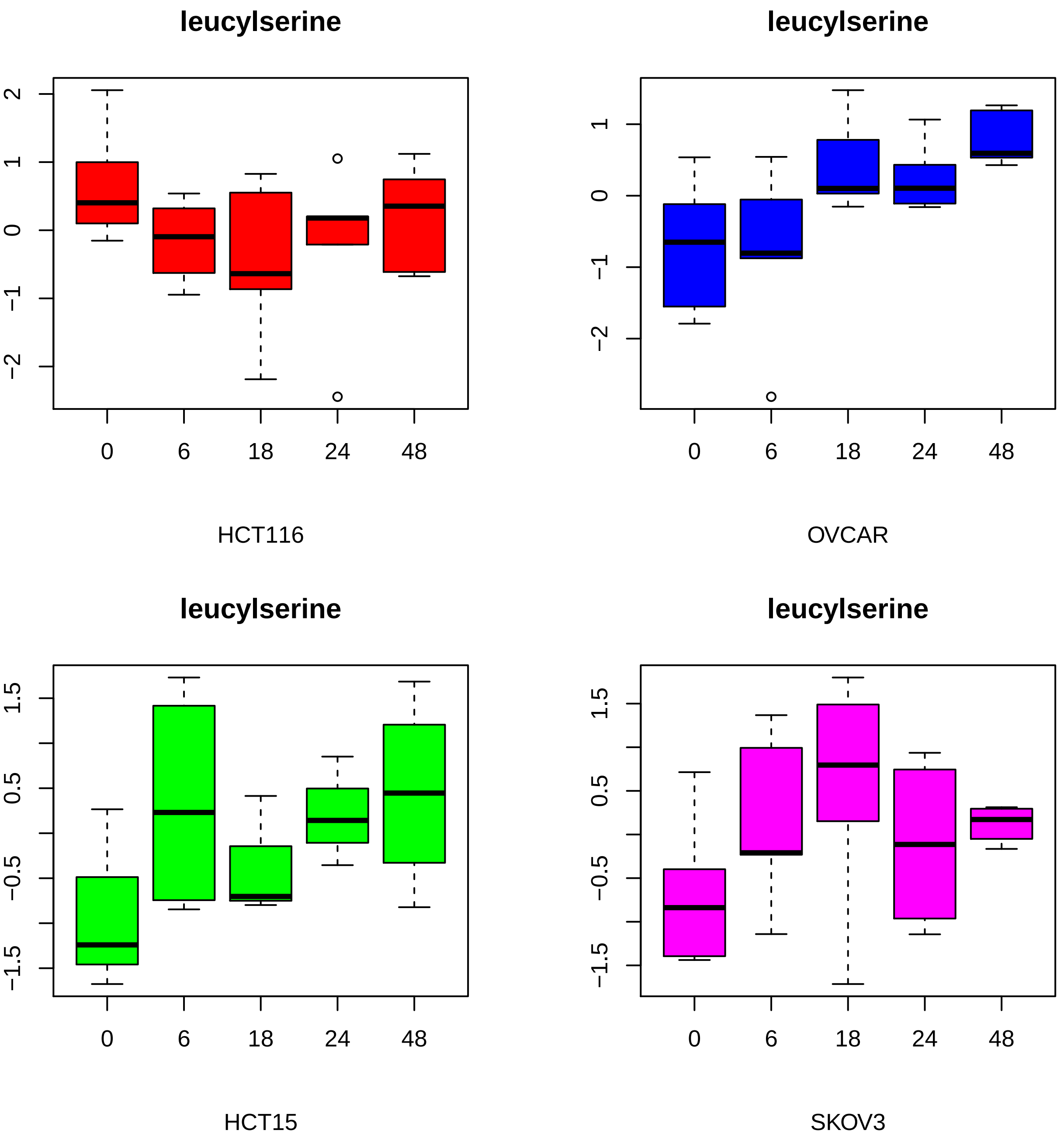

leucylserine
HCT116
leucylserine
OVCAR
leucylserine
HCT15
leucylserine
SKOV3
0
6
18
24
48

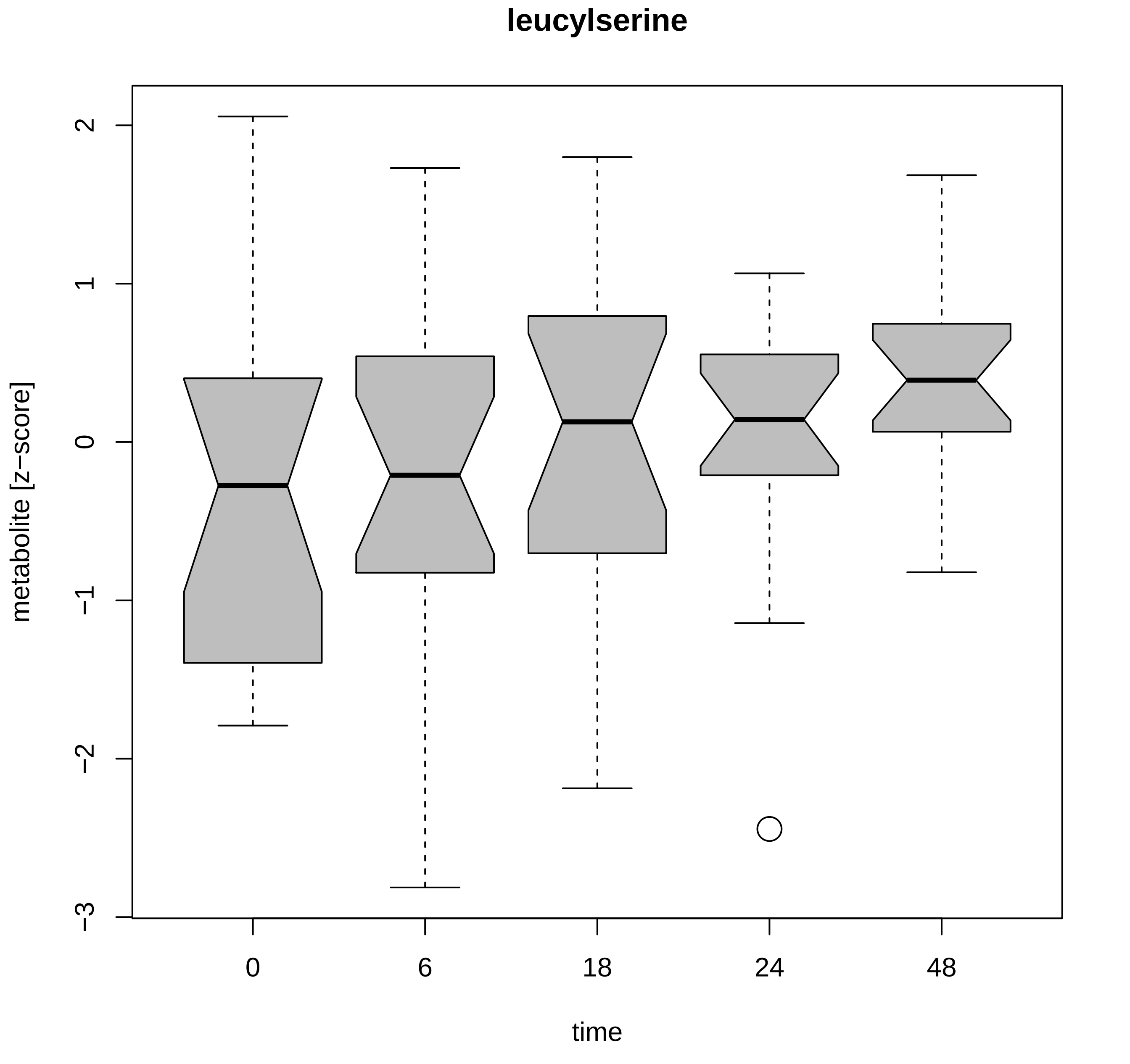

leucylserine
metabolite [z−score]
2
1
0
−1
−2
−3
0
6
18
24
48
time

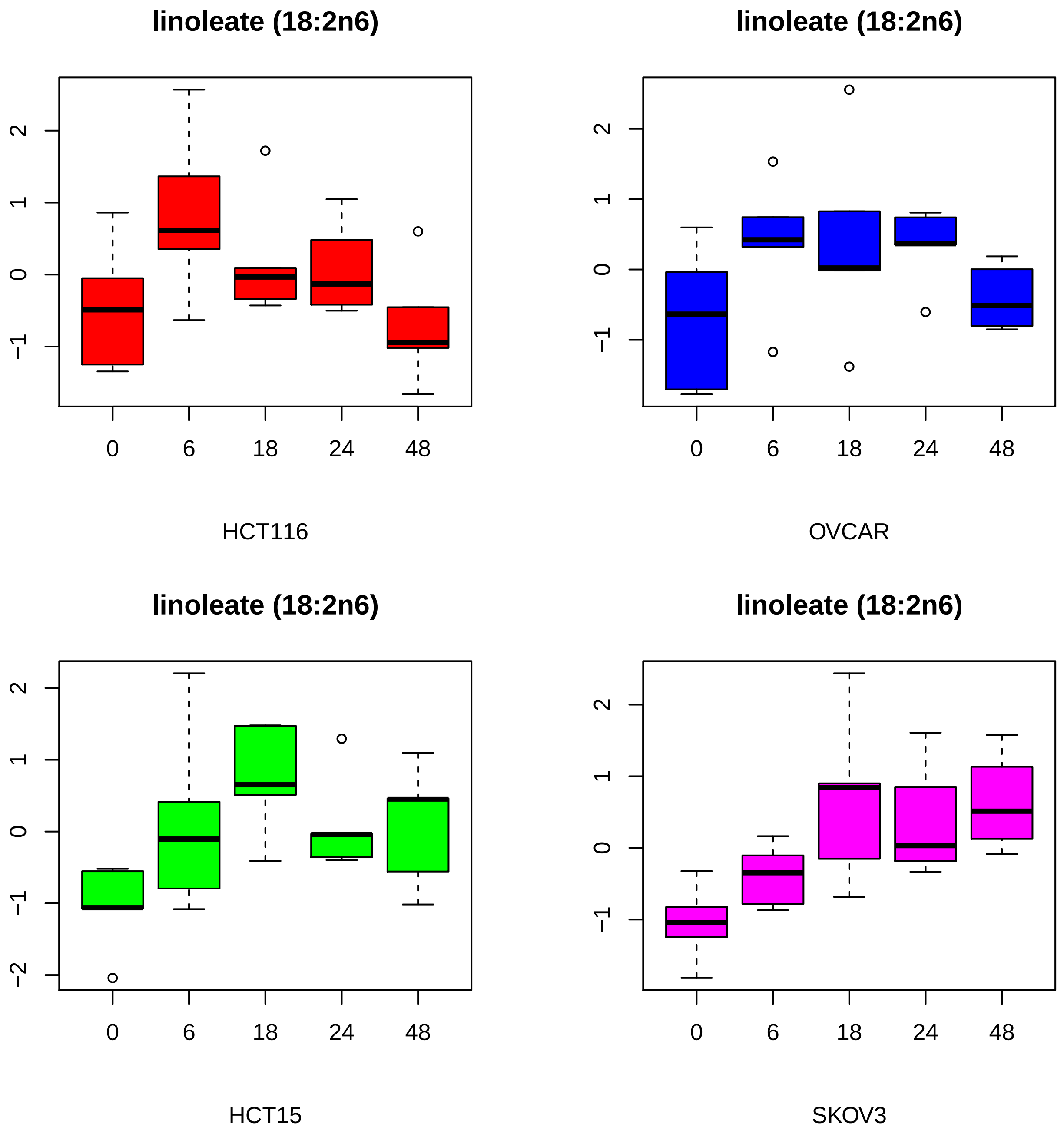
linoleate (18:2n6)
2
1
0
−1
0
6
18
24
48
HCT116
linoleate (18:2n6)
2
1
0
−1
0
6
18
24
48
OVCAR
linoleate (18:2n6)
2
1
0
−1
−2
0
6
18
24
48
HCT15
linoleate (18:2n6)
2
1
0
−1
0
6
18
24
48
SKOV3

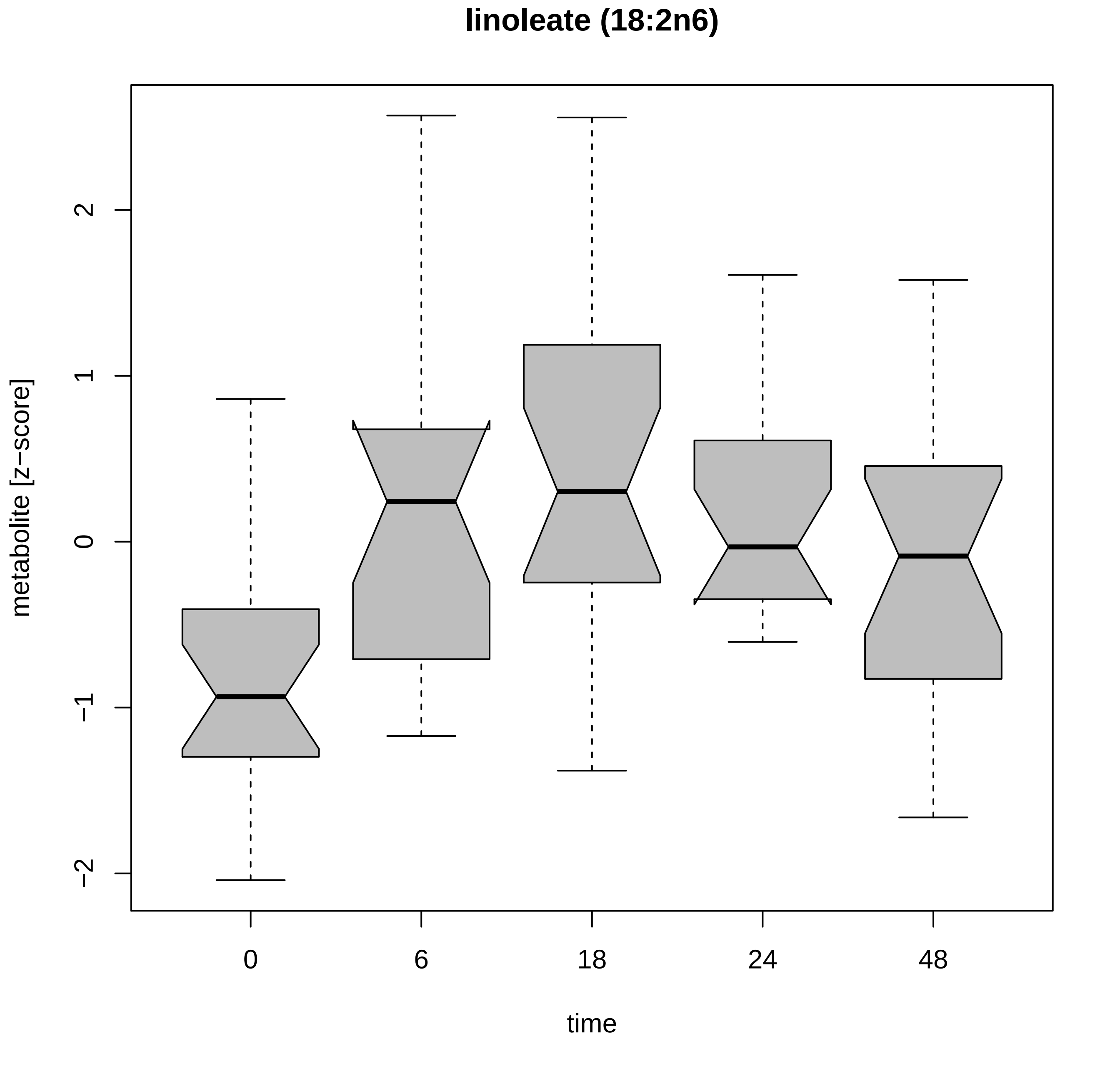

linoleate (18:2n6)
metabolite [z−score]
2
1
0
−1
−2
0
6
18
24
48
time

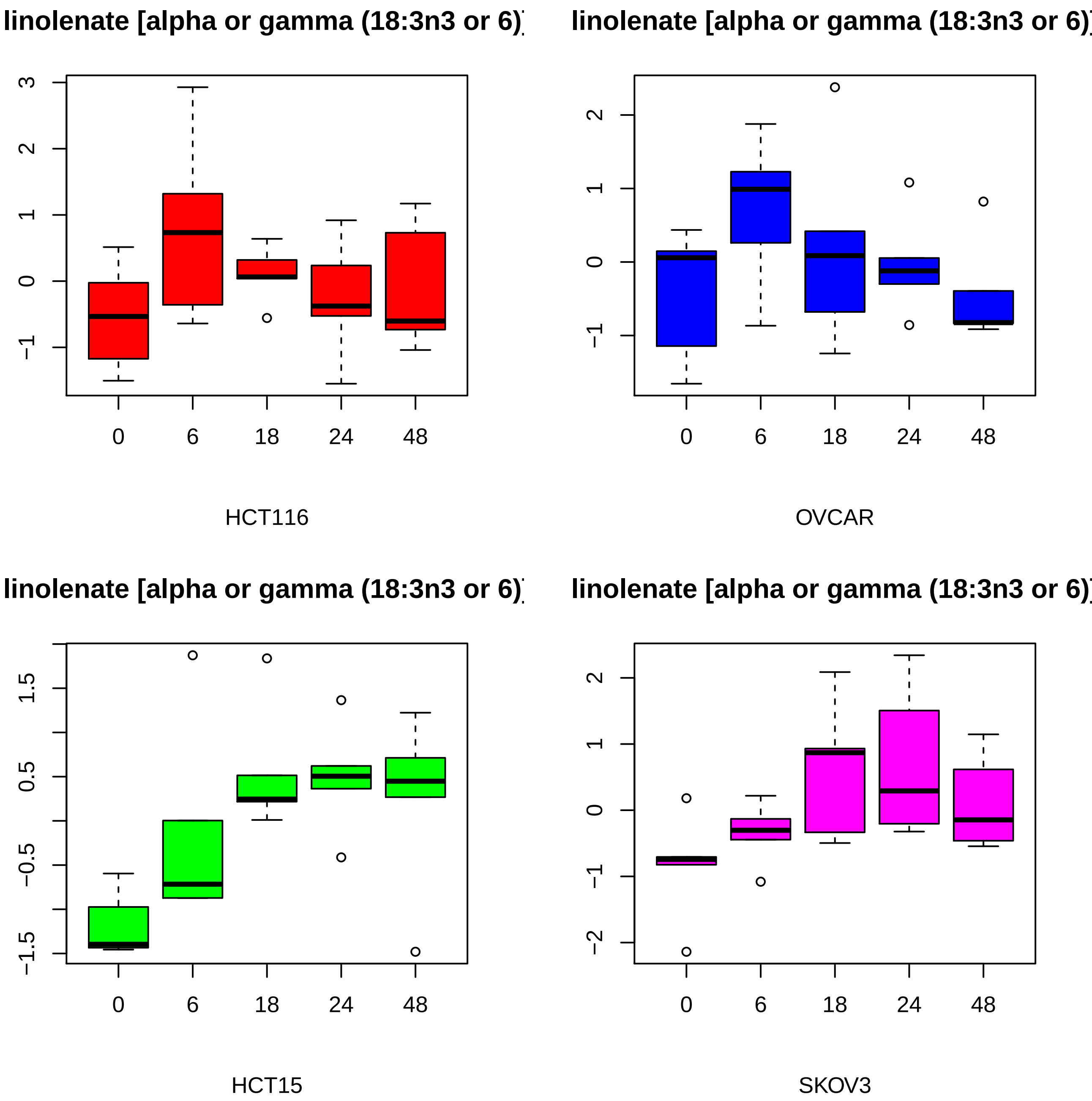
linolenate [alpha or gamma (18:3n3 or 6)]
3
2
1
0
−1
0
6
18
24
48
HCT116
linolenate [alpha or gamma (18:3n3 or 6)]
2
1
0
−1
0
6
18
24
48
OVCAR
linolenate [alpha or gamma (18:3n3 or 6)]
1.5
0.5
−0.5
−1.5
0
6
18
24
48
HCT15
linolenate [alpha or gamma (18:3n3 or 6)]
2
1
0
−1
−2
0
6
18
24
48
SKOV3

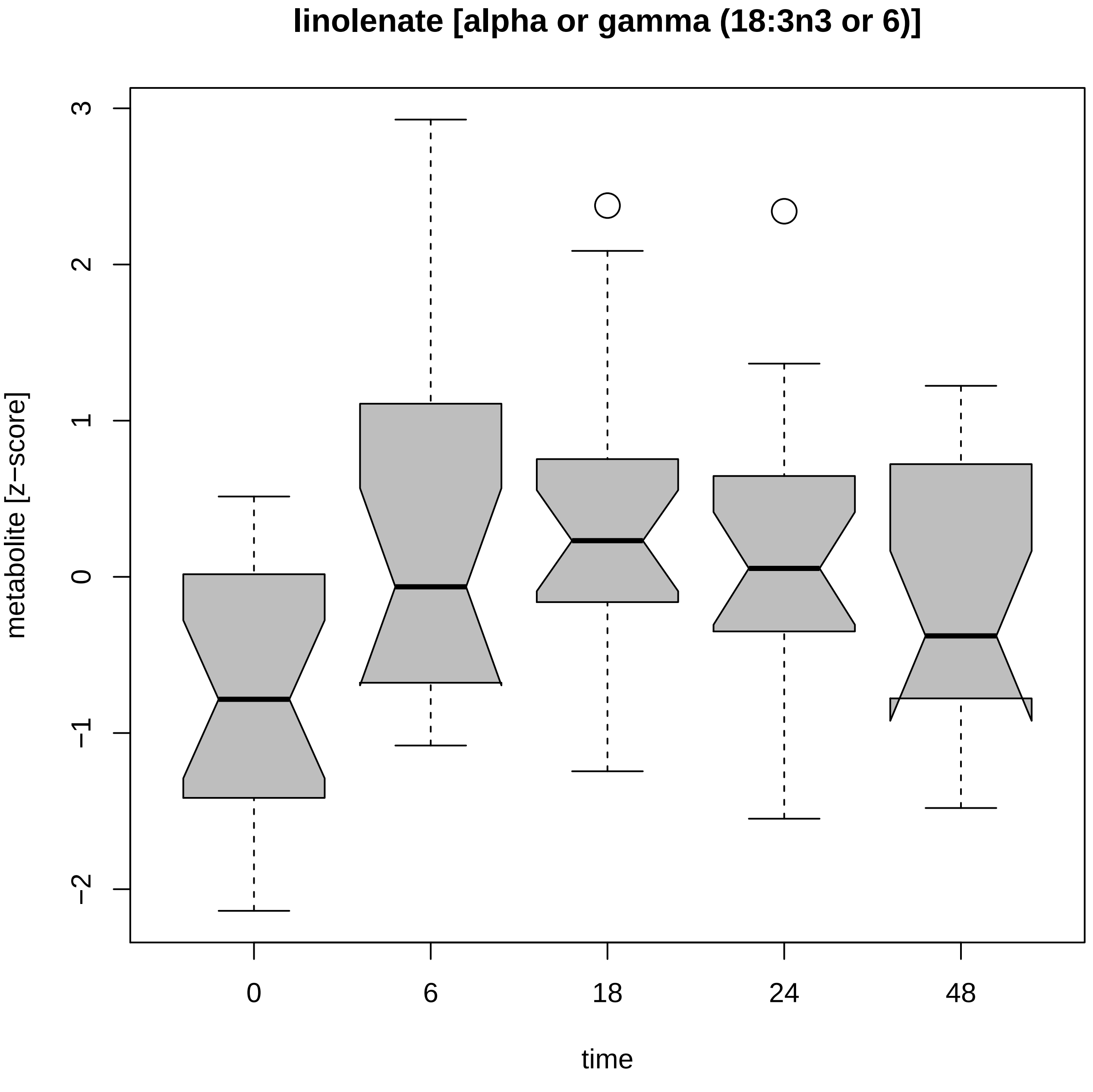

linolenate [alpha or gamma (18:3n3 or 6)]
metabolite [z−score]
3
2
1
0
−1
−2
0
6
18
24
48
time

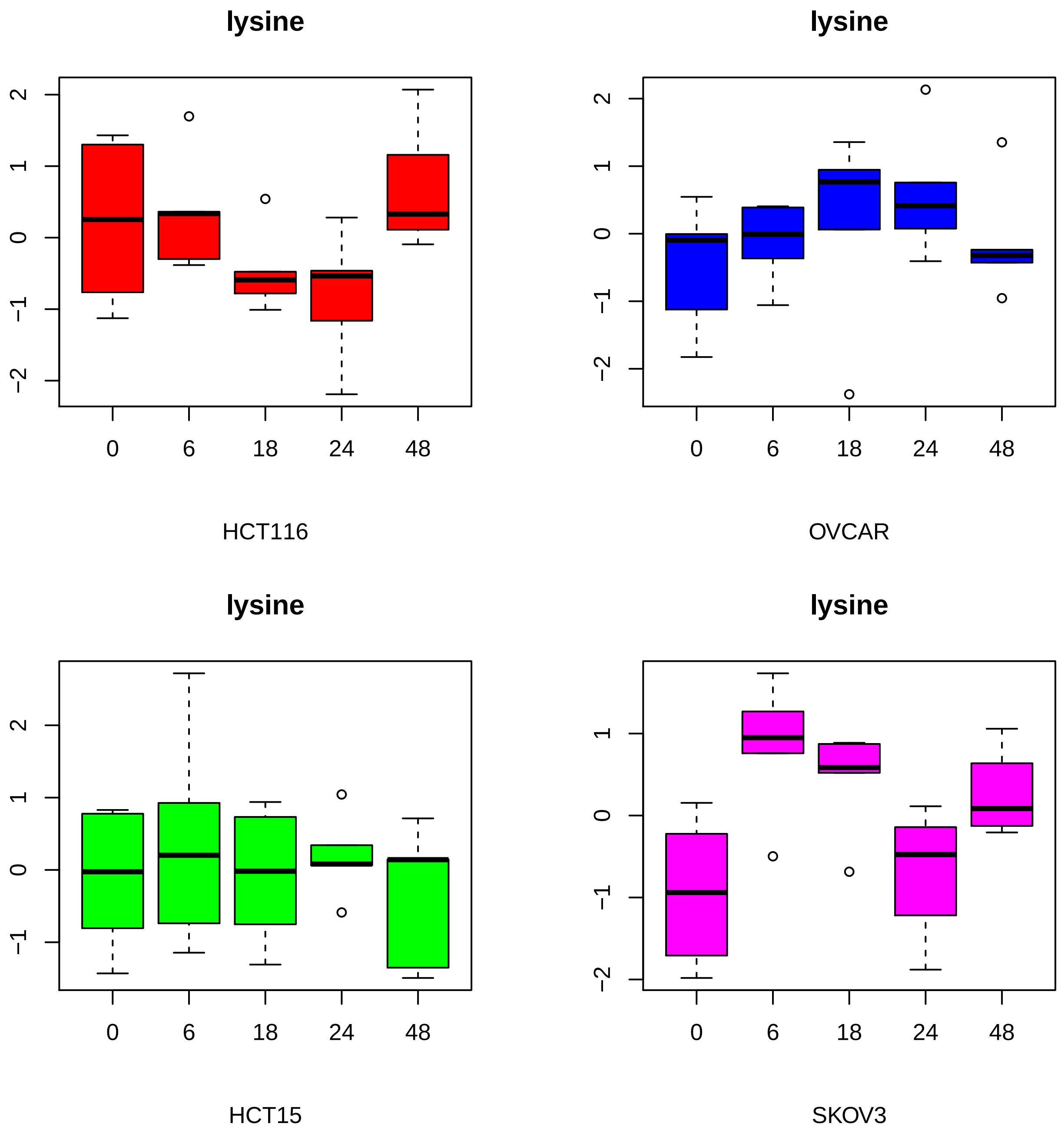
lysine
2
1
0
−1
−2
0
6
18
24
48
HCT116
lysine
2
1
0
−1
−2
0
6
18
24
48
OVCAR
lysine
2
1
0
−1
0
6
18
24
48
HCT15
lysine
1
0
−1
−2
0
6
18
24
48
SKOV3

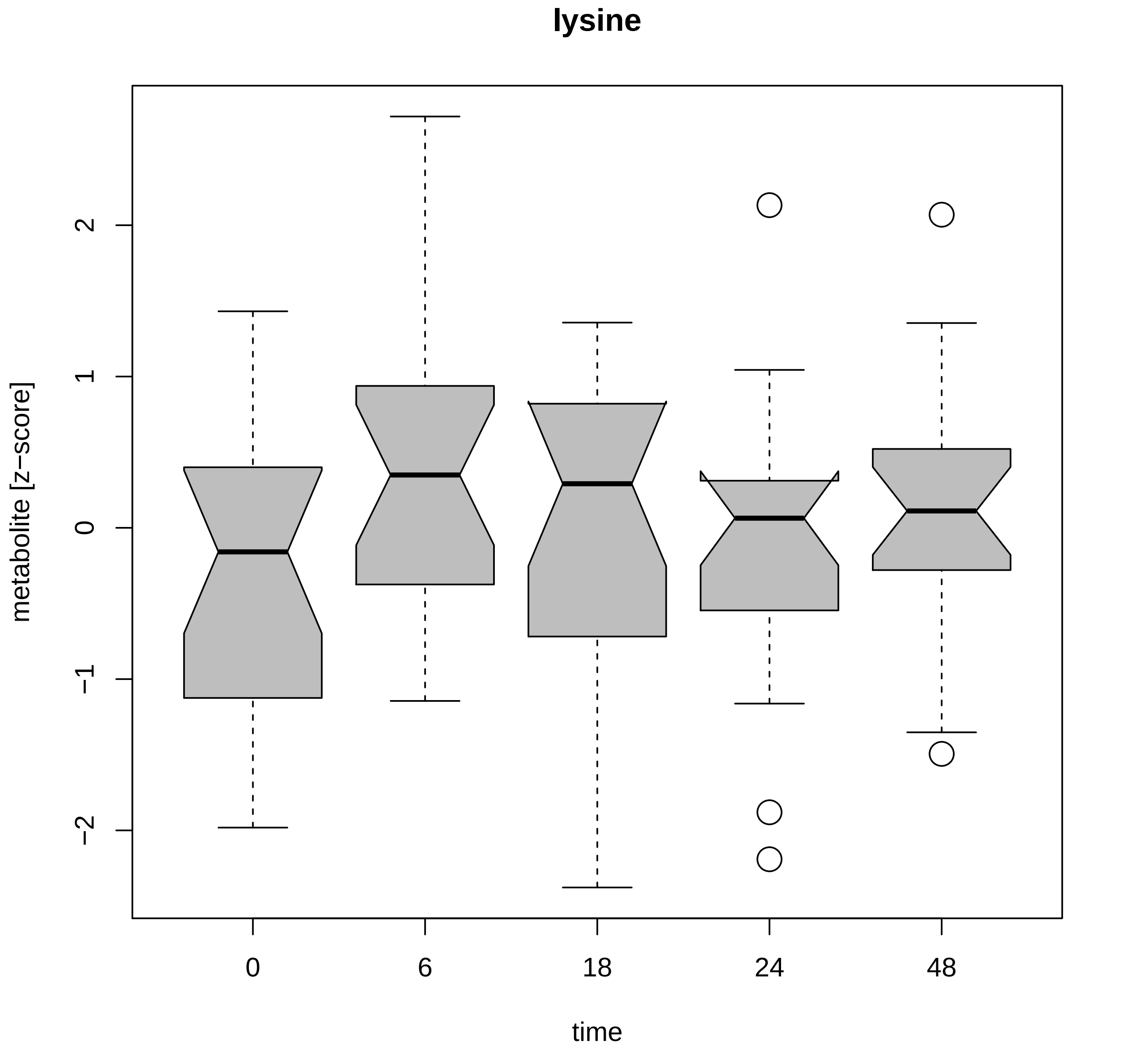

lysine
metabolite [z−score]
2
1
0
−1
−2
0
6
18
24
48
time

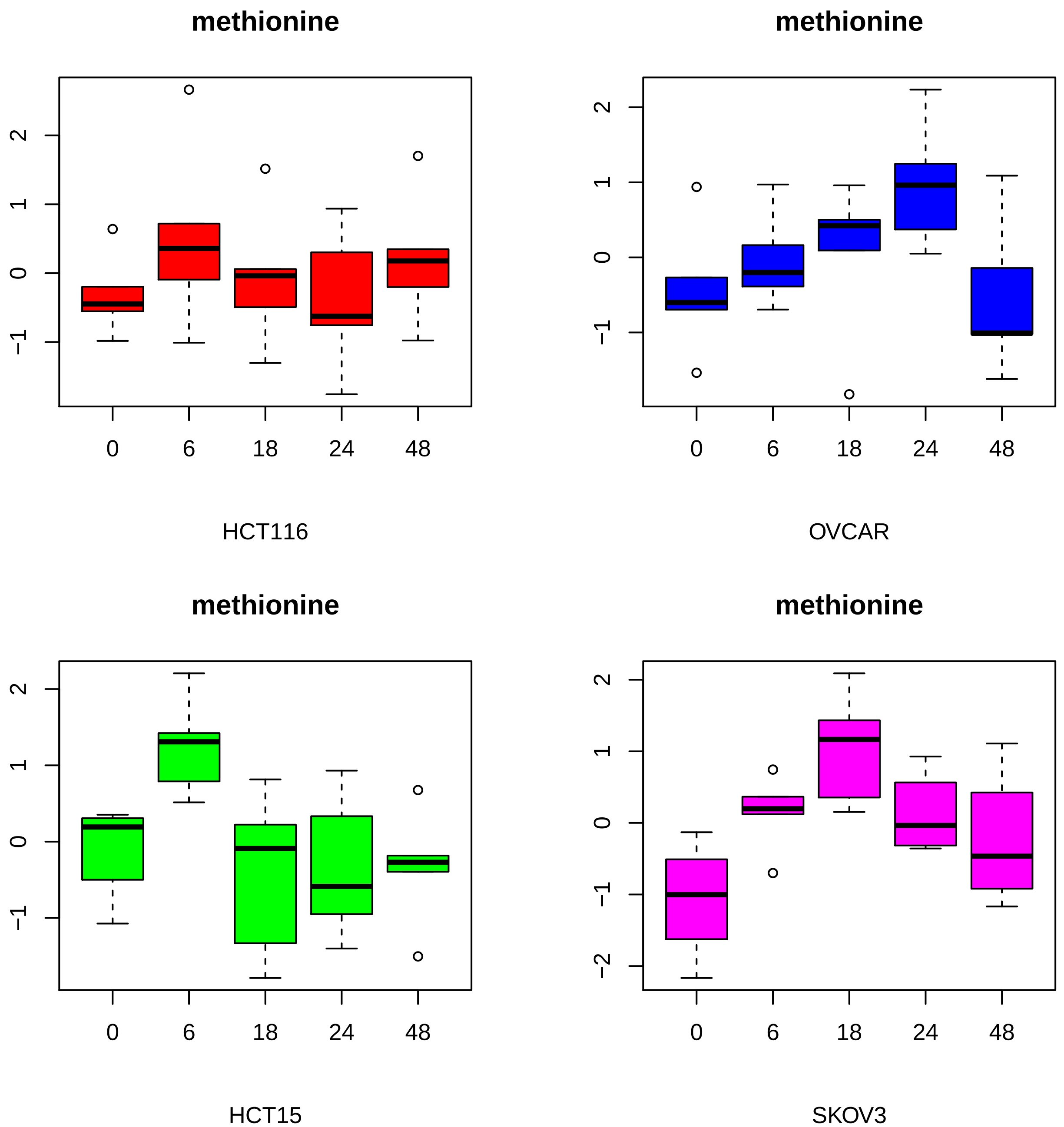
methionine
2
1
0
−1
0
6
18
24
48
HCT116
methionine
2
1
0
−1
0
6
18
24
48
OVCAR
methionine
2
1
0
−1
0
6
18
24
48
HCT15
methionine
2
1
0
−1
−2
0
6
18
24
48
SKOV3

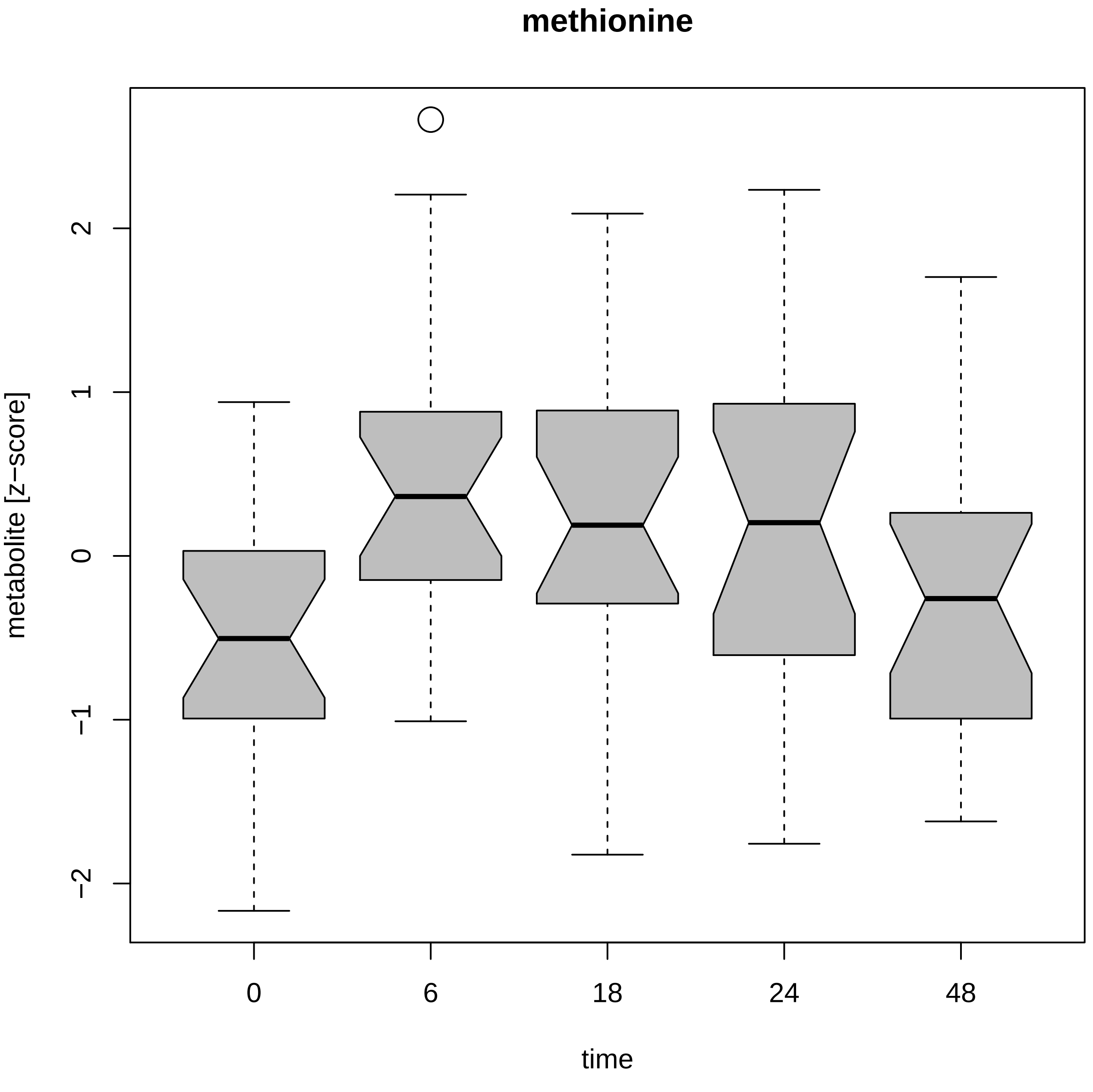

methionine
2
1
0
−1
−2
metabolite [z−score]
0
6
18
24
48
time

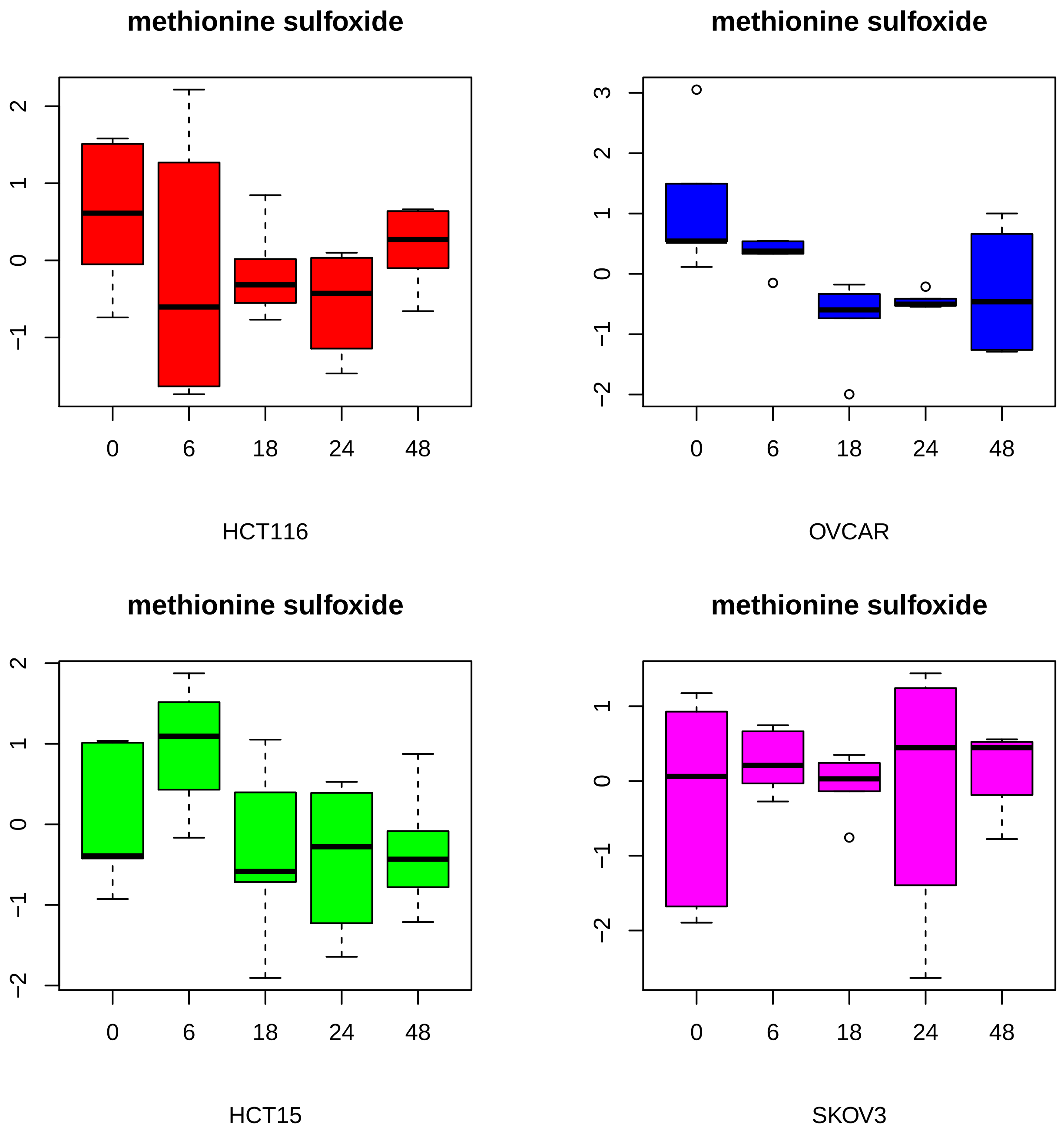
methionine sulfoxide
HCT116
methionine sulfoxide
OVCAR
methionine sulfoxide
HCT15
methionine sulfoxide
SKOV3
0
6
18
24
48

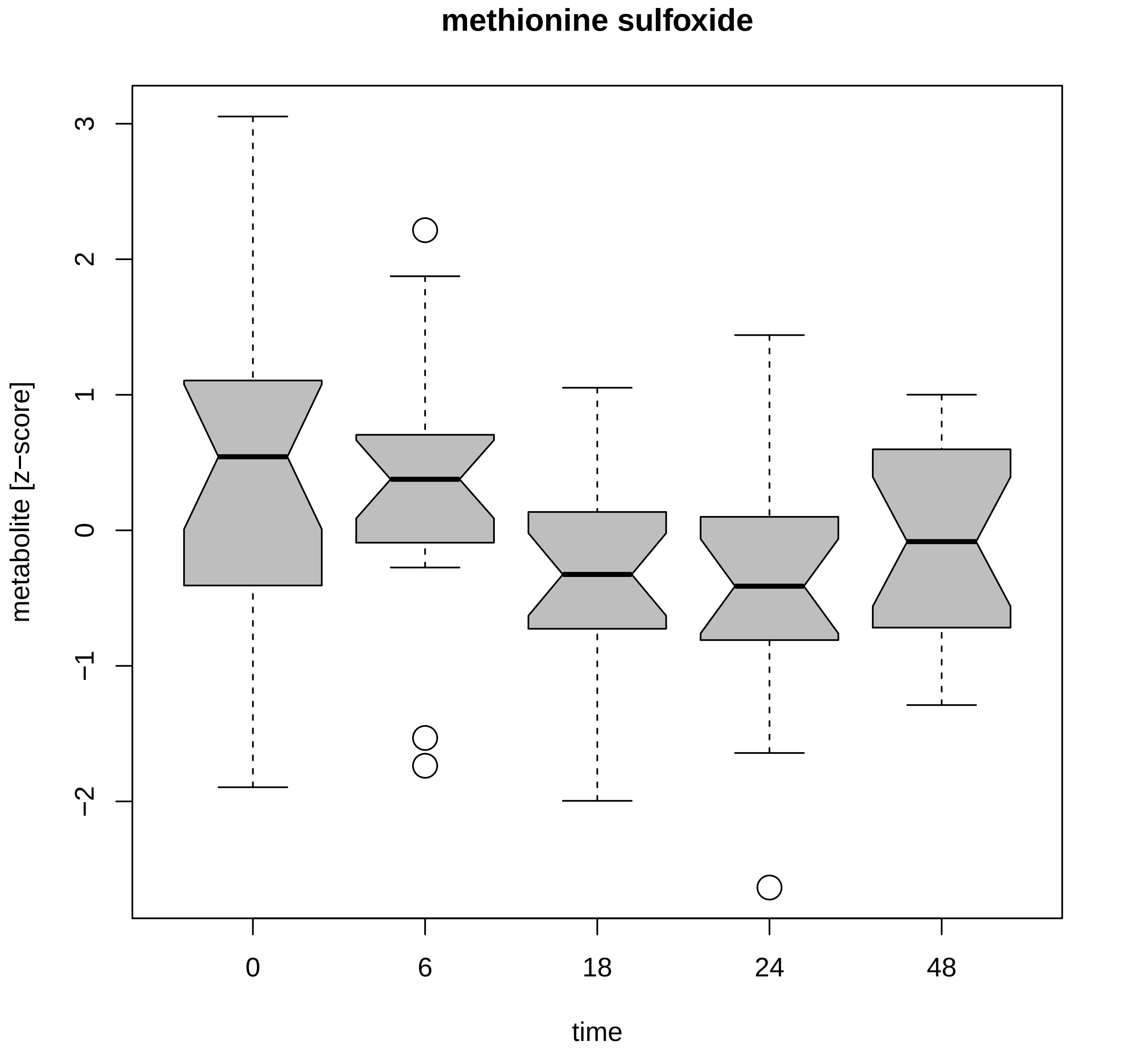

methionine sulfoxide
metabolite [z−score]
3
2
1
0
−1
−2
0
6
18
24
48
time

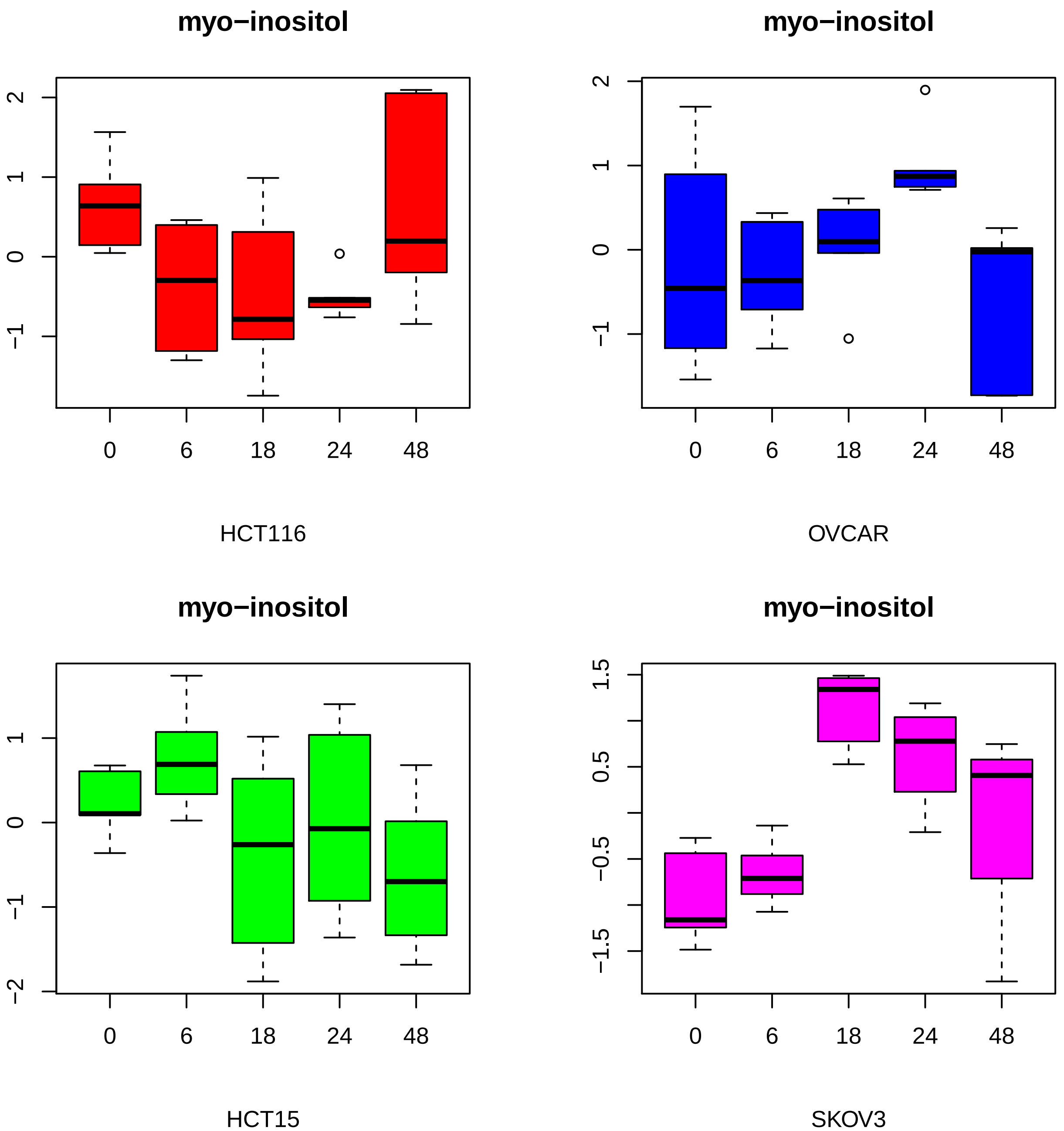

myo−inositol
2
1
0
−1
0
6
18
24
48
HCT116
myo−inositol
2
1
0
−1
0
6
18
24
48
OVCAR
myo−inositol
1
0
−1
−2
0
6
18
24
48
HCT15
myo−inositol
1.5
0.5
−0.5
−1.5
0
6
18
24
48
SKOV3

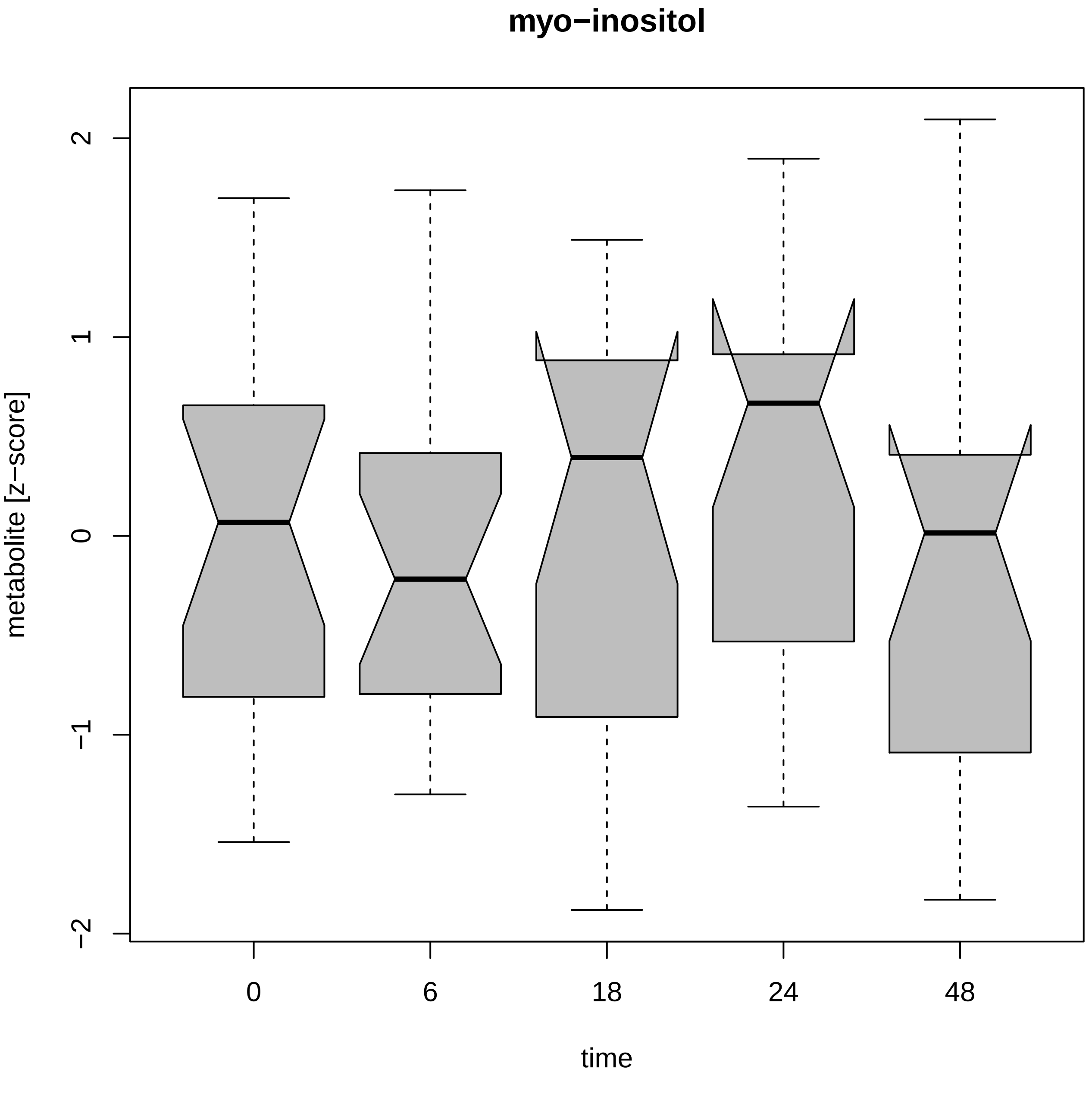

myo−inositol
metabolite [z−score]
2
1
0
−1
−2
0
6
18
24
48
time

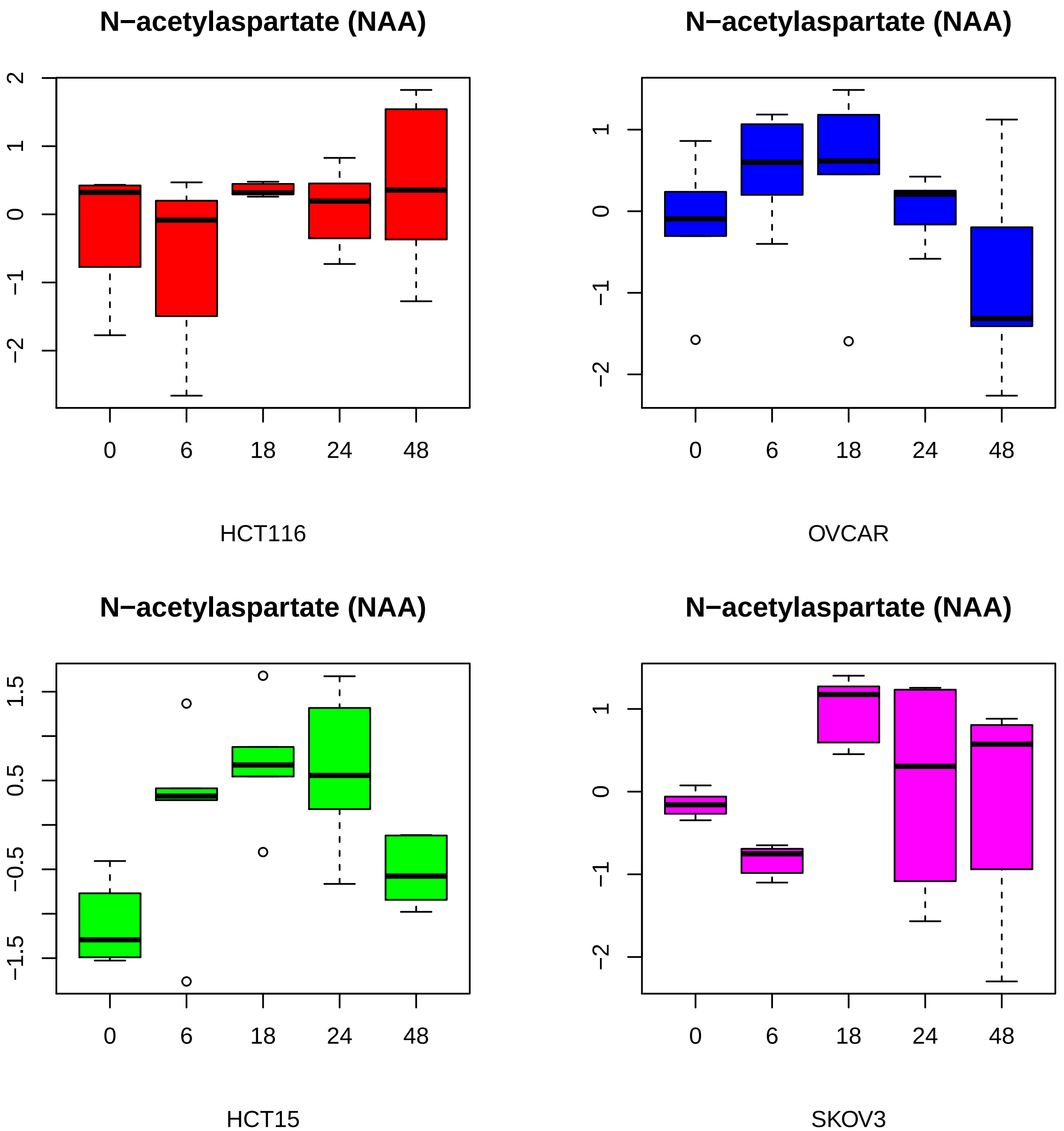
N−acetylaspartate (NAA)
2
1
0
−1
−2
0
6
18
24
48
HCT116
N−acetylaspartate (NAA)
1
0
−1
−2
0
6
18
24
48
OVCAR
N−acetylaspartate (NAA)
1.5
0.5
−0.5
−1.5
0
6
18
24
48
HCT15
N−acetylaspartate (NAA)
1
0
−1
−2
0
6
18
24
48
SKOV3

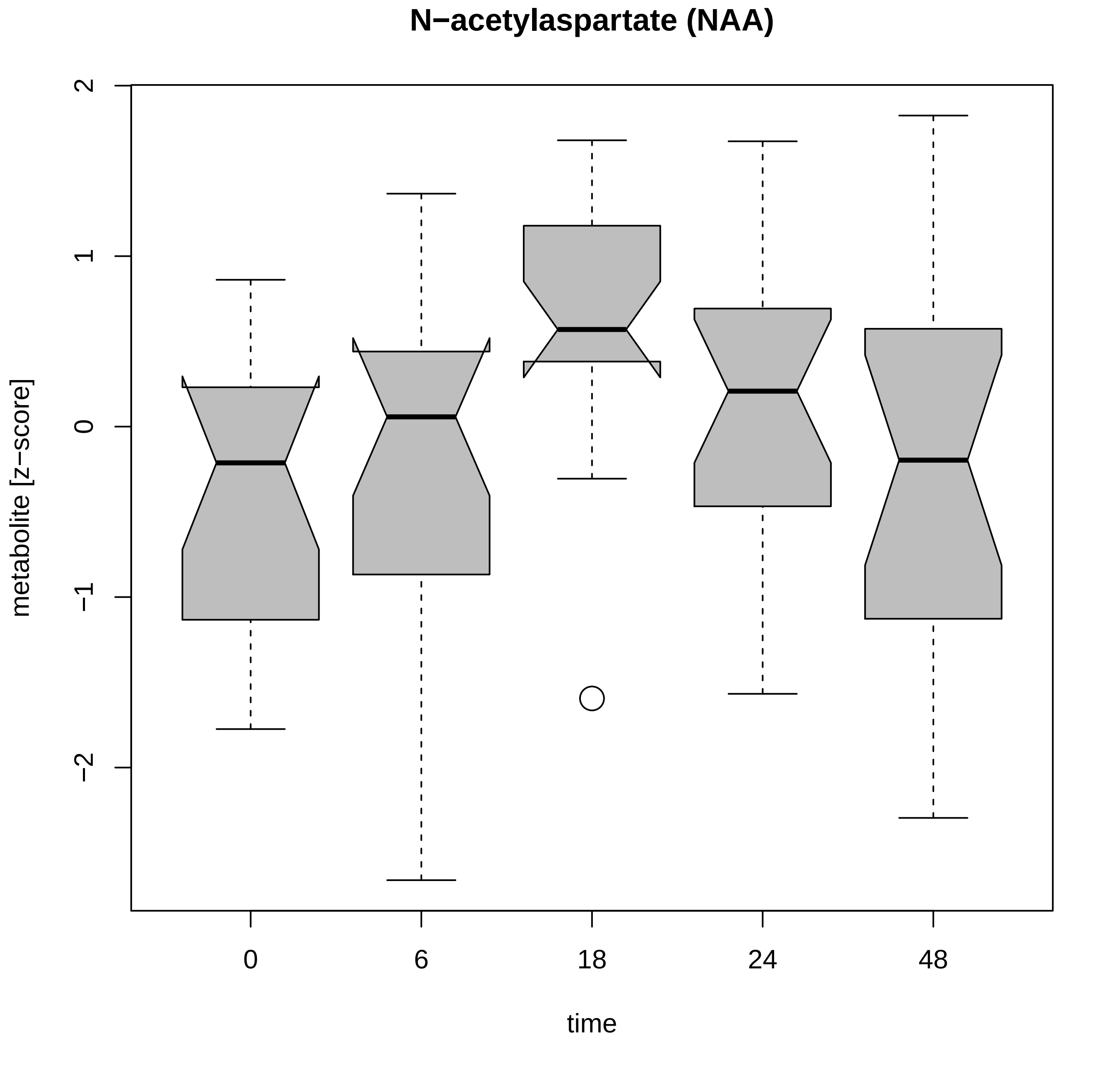
N−acetylaspartate (NAA)
metabolite [z−score]
time
0
6
18
24
48
−2
−1
0
1
2

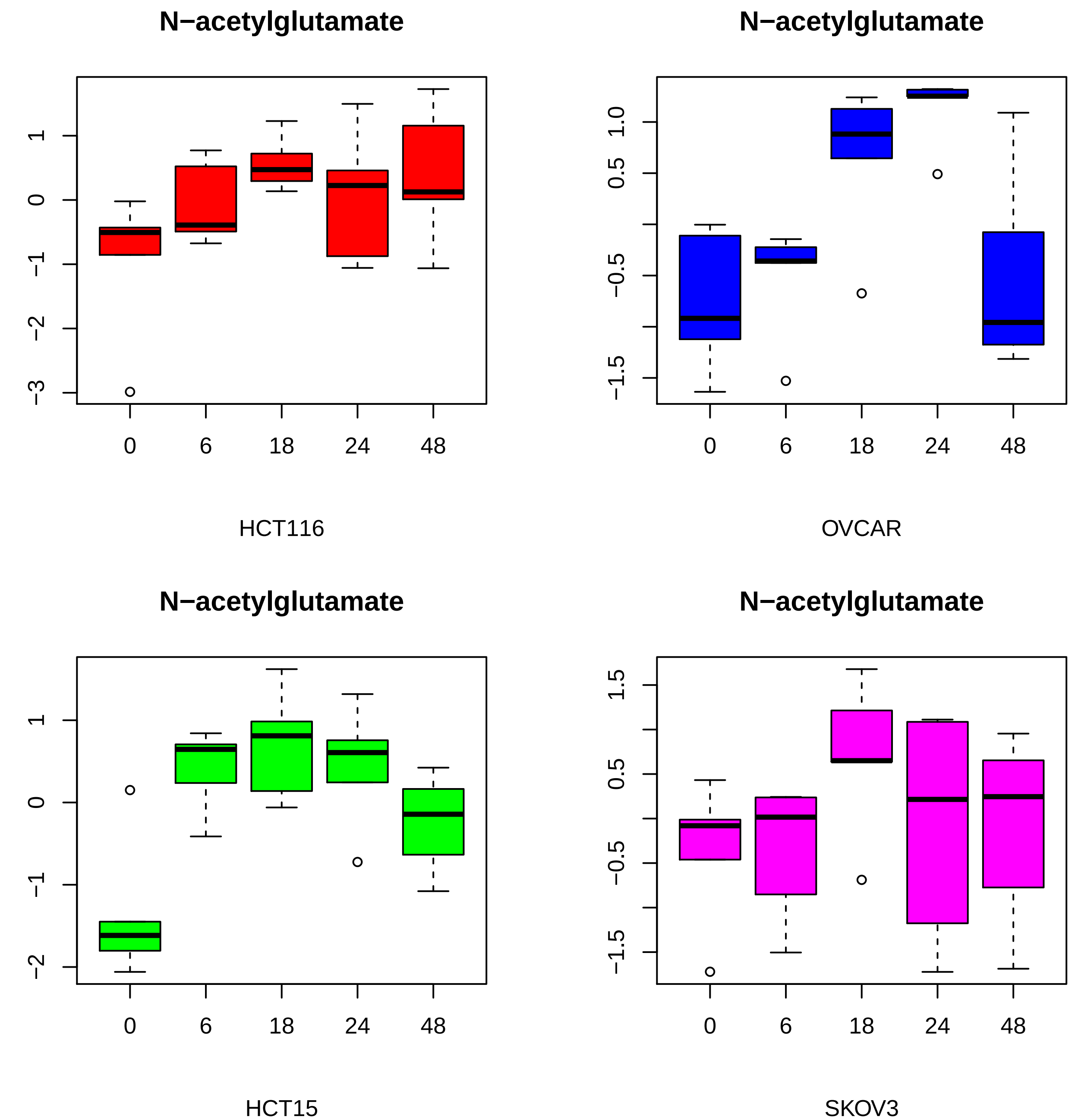
N−acetylglutamate
HCT116
N−acetylglutamate
OVCAR
N−acetylglutamate
HCT15
N−acetylglutamate
SKOV3
0
6
18
24
48
−3
−2
−1
0
1
−1.5
−0.5
0.5
1.0
1.5

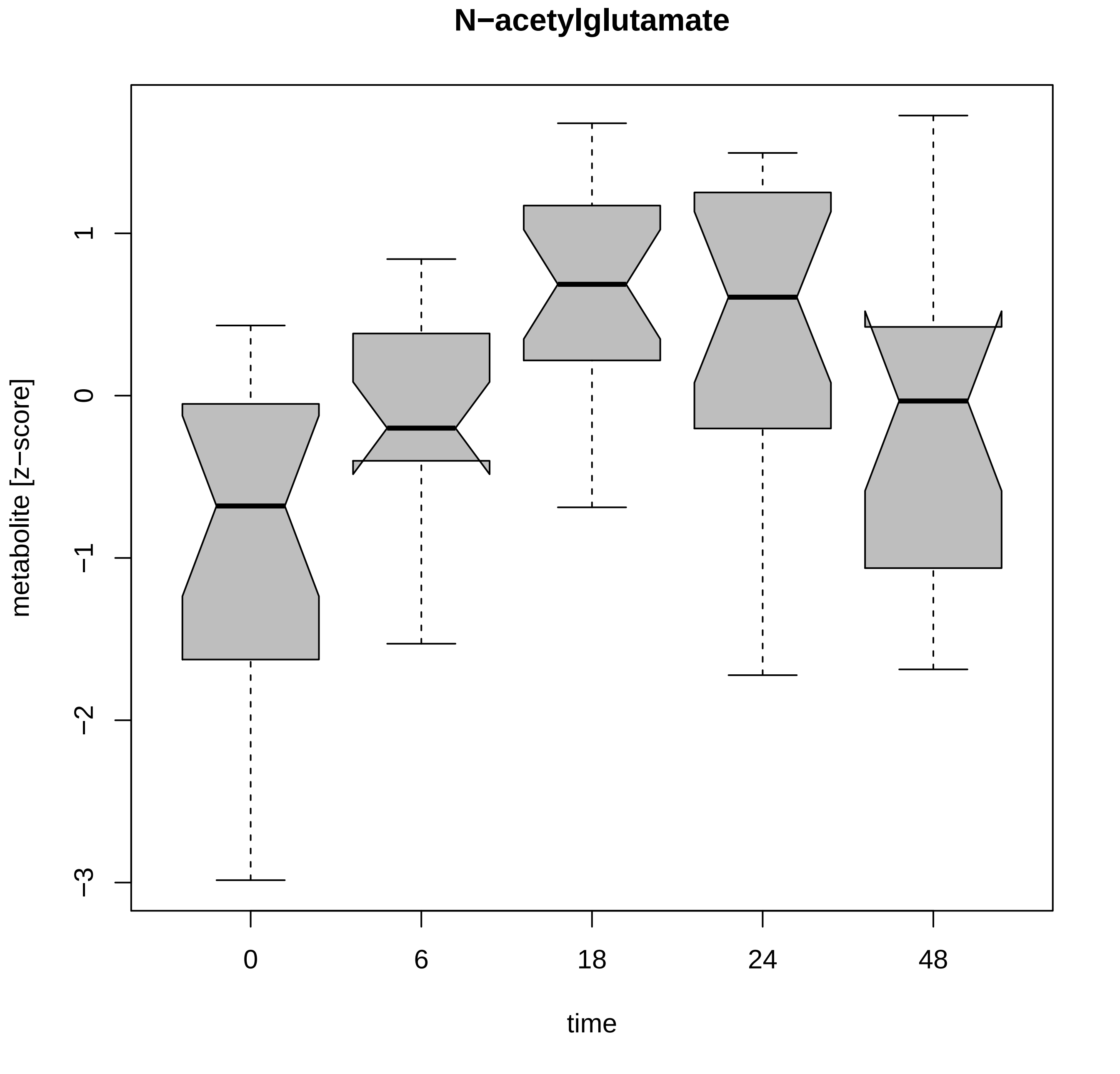

N−acetylglutamate
metabolite [z−score]
−3
−2
−1
0
1
0
6
18
24
48
time

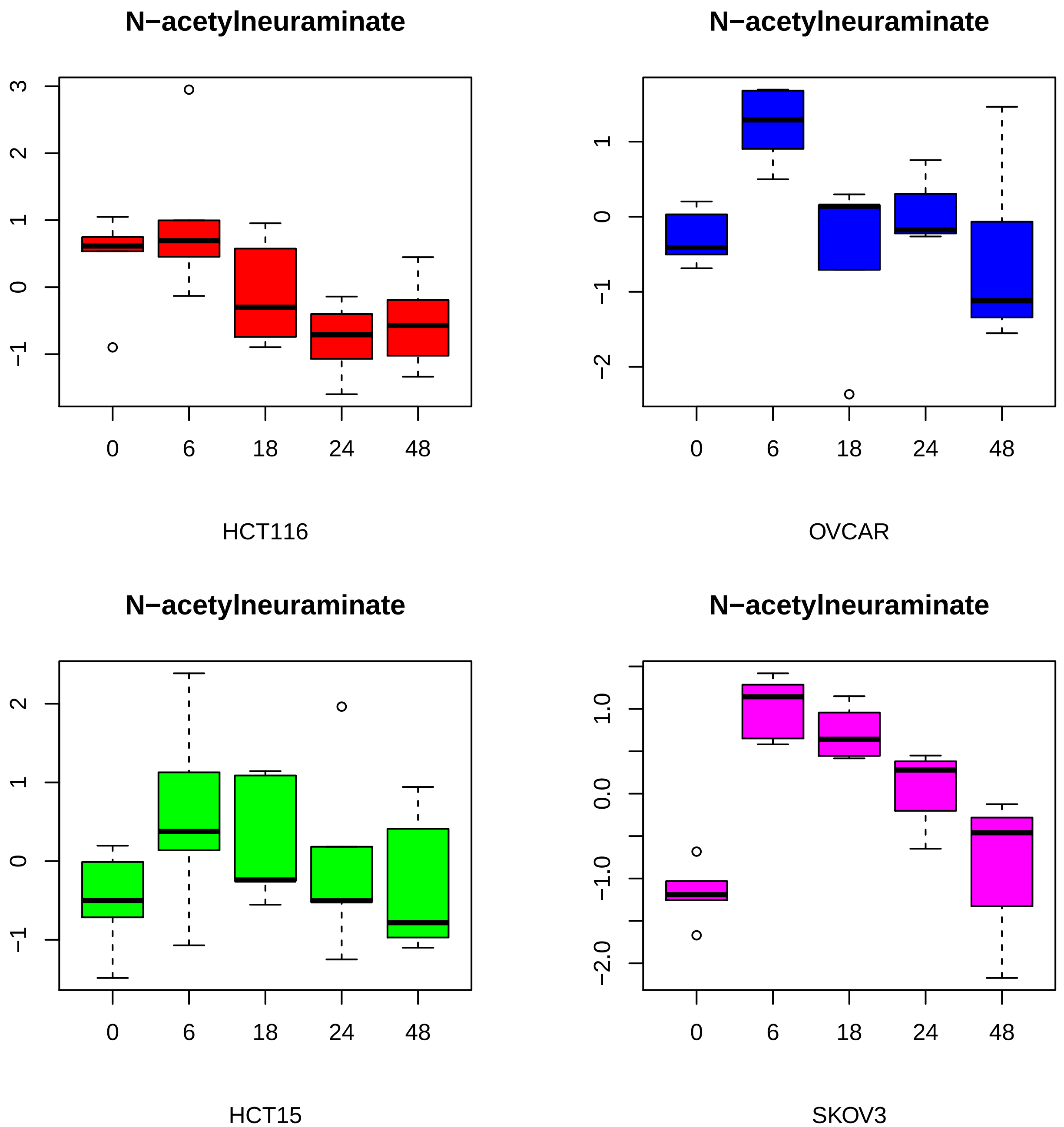

N−acetylneuraminate
HCT116
N−acetylneuraminate
OVCAR
N−acetylneuraminate
HCT15
N−acetylneuraminate
SKOV3
0
6
18
24
48

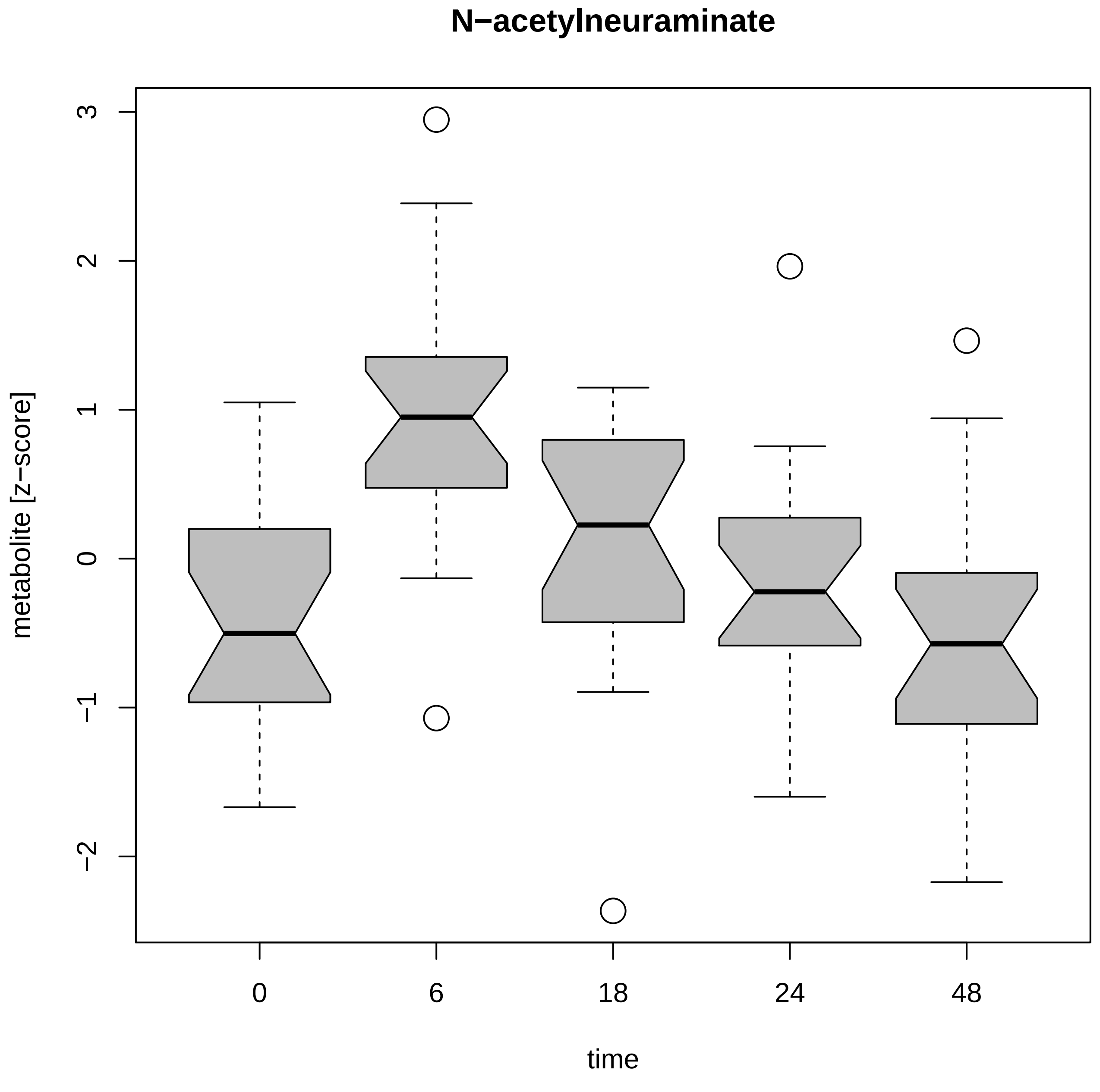
N−acetylneuraminate
metabolite [z−score]
3
2
1
0
−1
−2
0
6
18
24
48
time

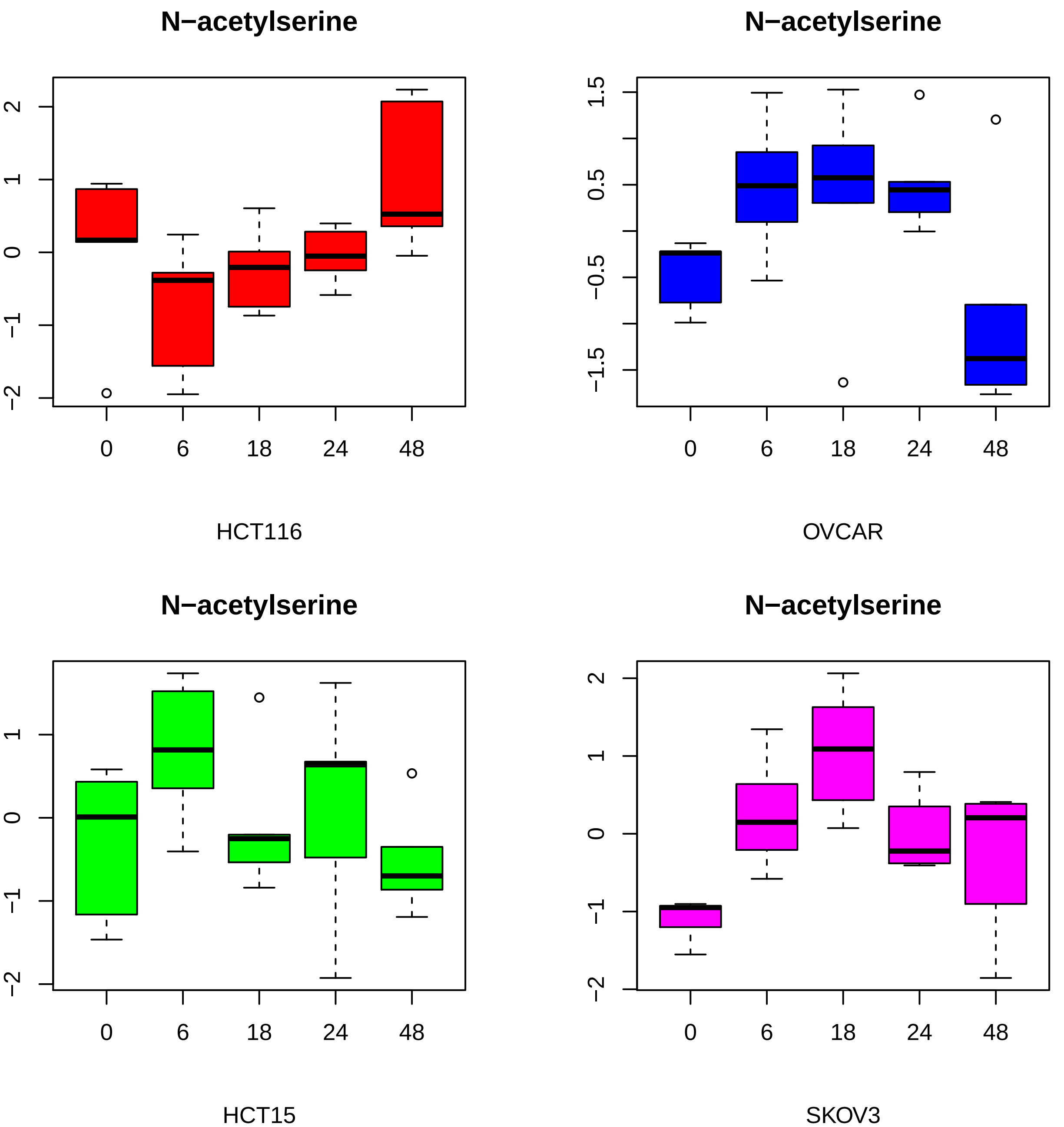
N−acetylserine
2
1
0
−1
−2
0
6
18
24
48
HCT116
N−acetylserine
1.5
0.5
−0.5
−1.5
0
6
18
24
48
OVCAR
N−acetylserine
1
0
−1
−2
0
6
18
24
48
HCT15
N−acetylserine
2
1
0
−1
−2
0
6
18
24
48
SKOV3

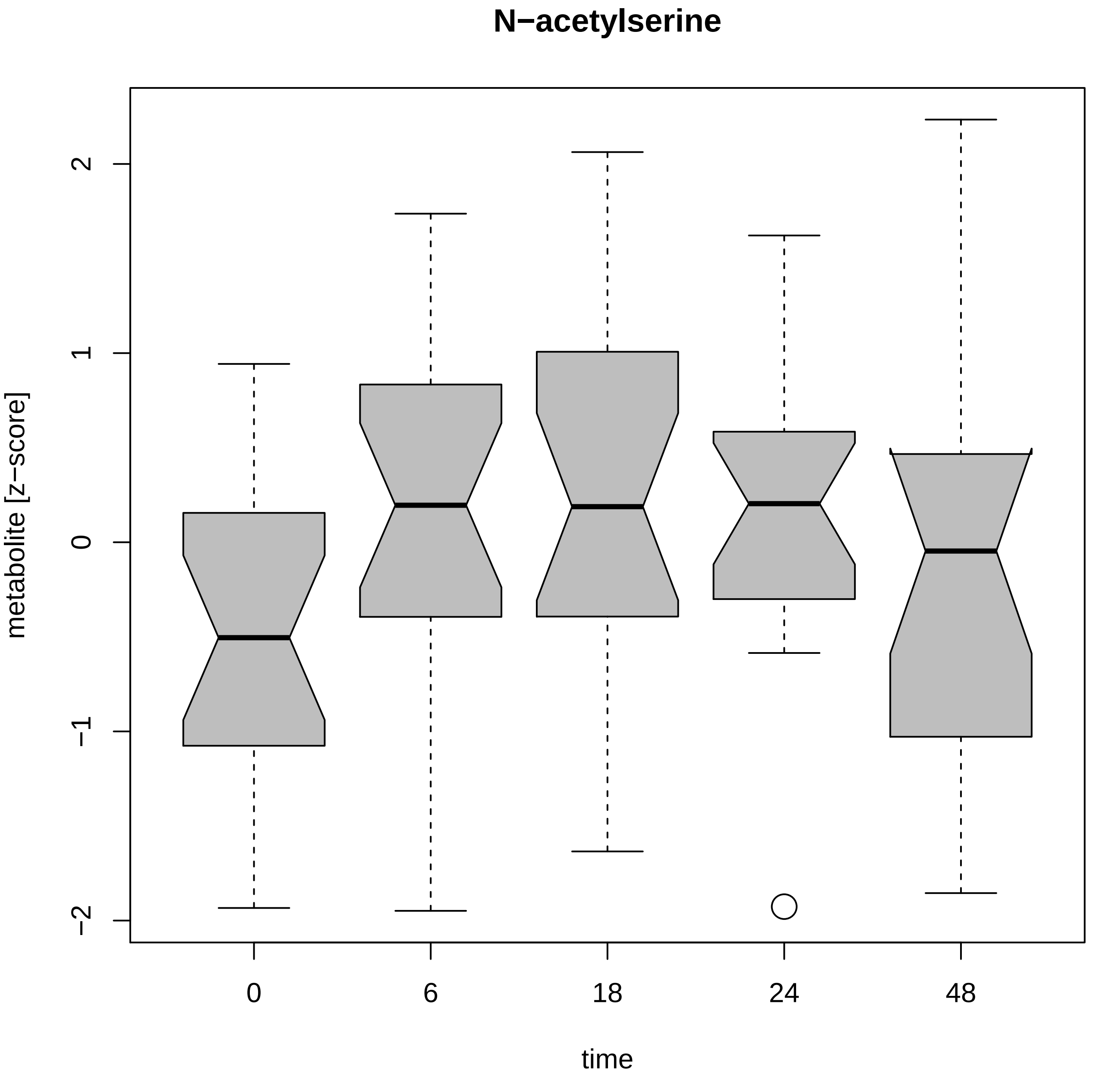
N−acetylserine
metabolite [z−score]
2
1
0
−1
−2
0
6
18
24
48
time

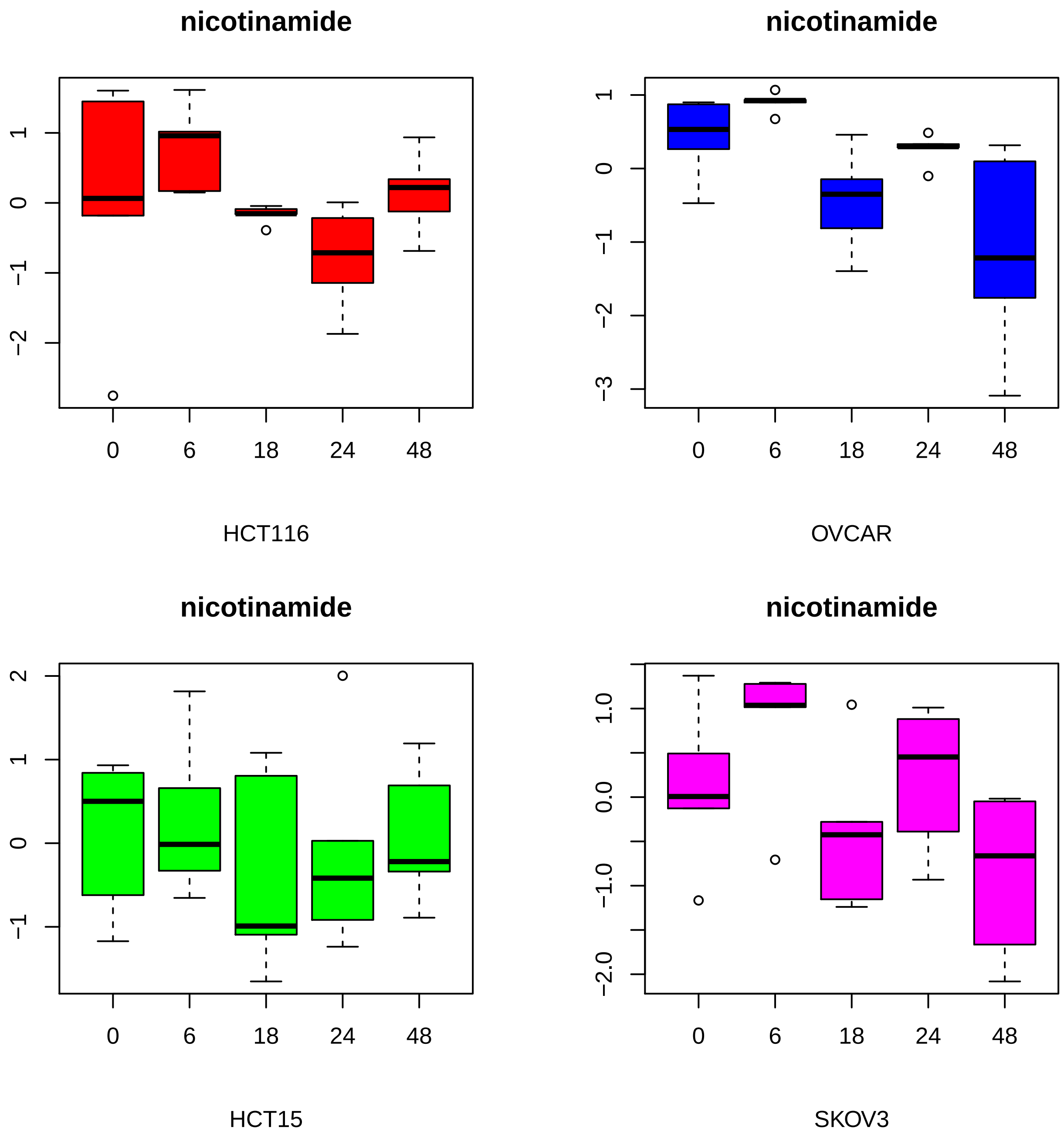
nicotinamide
nicotinamide
nicotinamide
nicotinamide
1
0
−1
−2
0
6
18
24
48
HCT116
1
0
−1
−2
−3
OVCAR
2
1
0
−1
HCT15
1.0
0.0
−1.0
−2.0
SKOV3

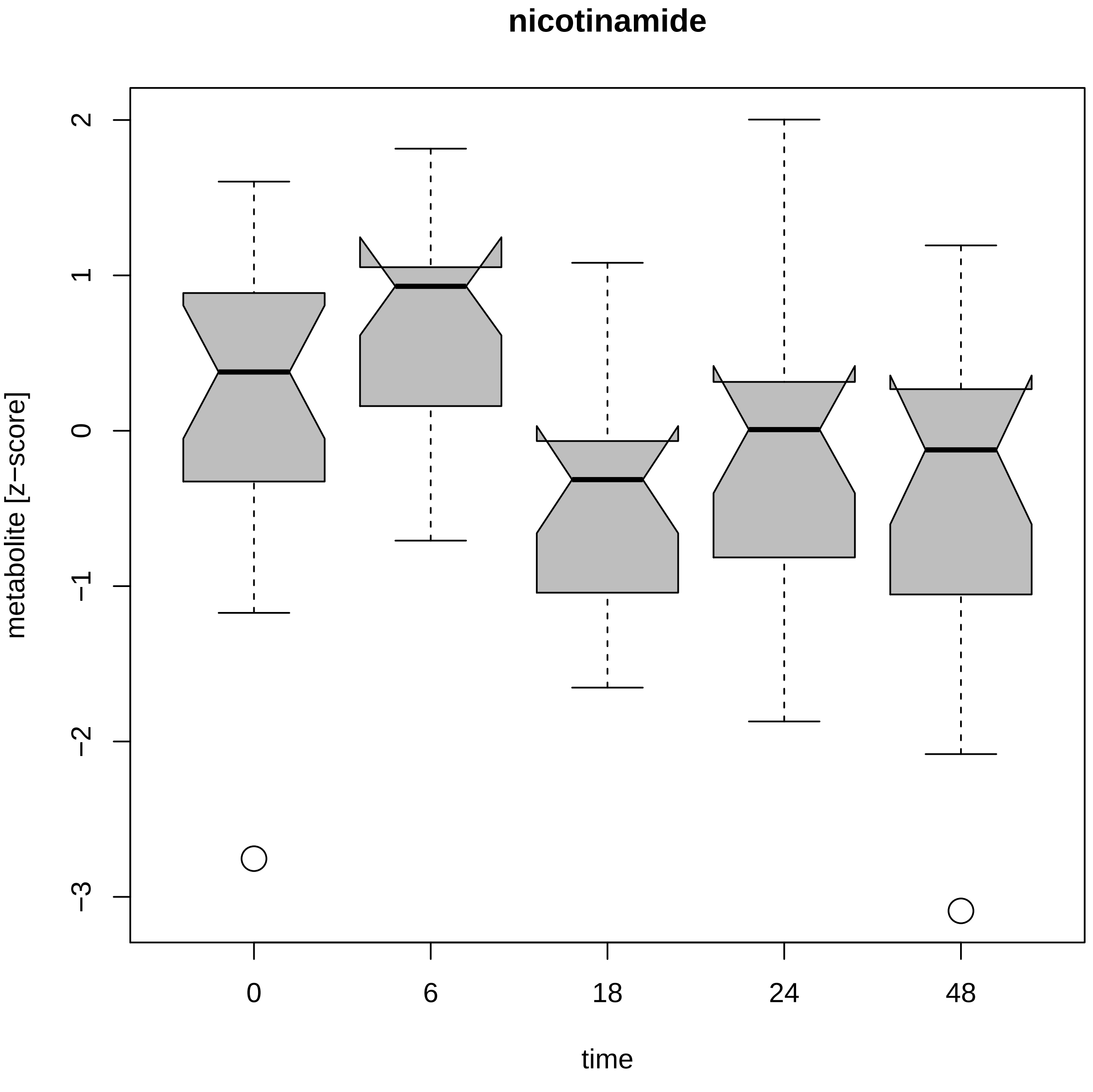
nicotinamide
metabolite [z−score]
time
2
1
0
−1
−2
−3
0
6
18
24
48

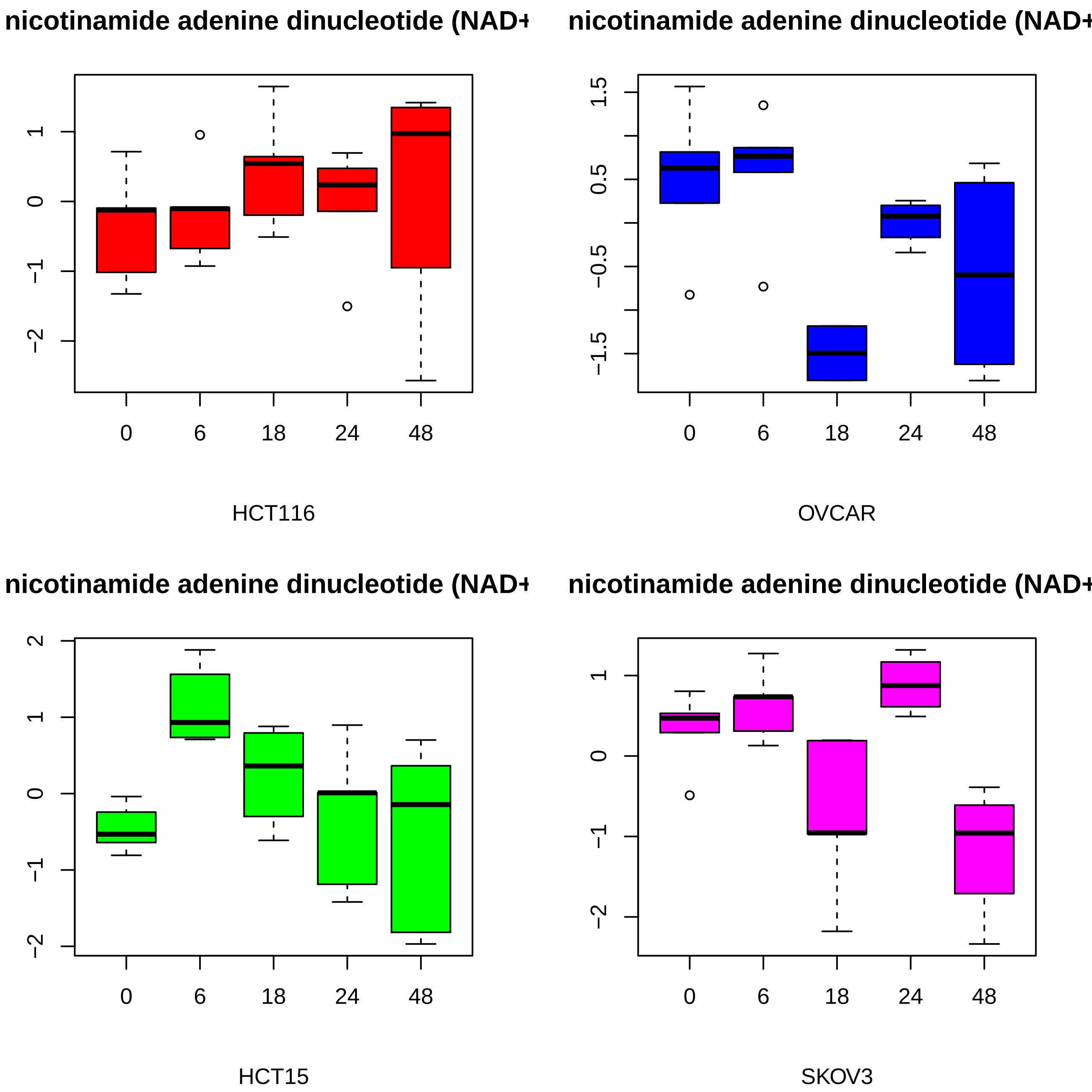
nicotinamide adenine dinucleotide (NAD+
1
0
−1
−2
0
6
18
24
48
HCT116
nicotinamide adenine dinucleotide (NAD+
1.5
0.5
−0.5
−1.5
0
6
18
24
48
OVCAR
nicotinamide adenine dinucleotide (NAD+
2
1
0
−1
−2
0
6
18
24
48
HCT15
nicotinamide adenine dinucleotide (NAD+
1
0
−1
−2
0
6
18
24
48
SKOV3

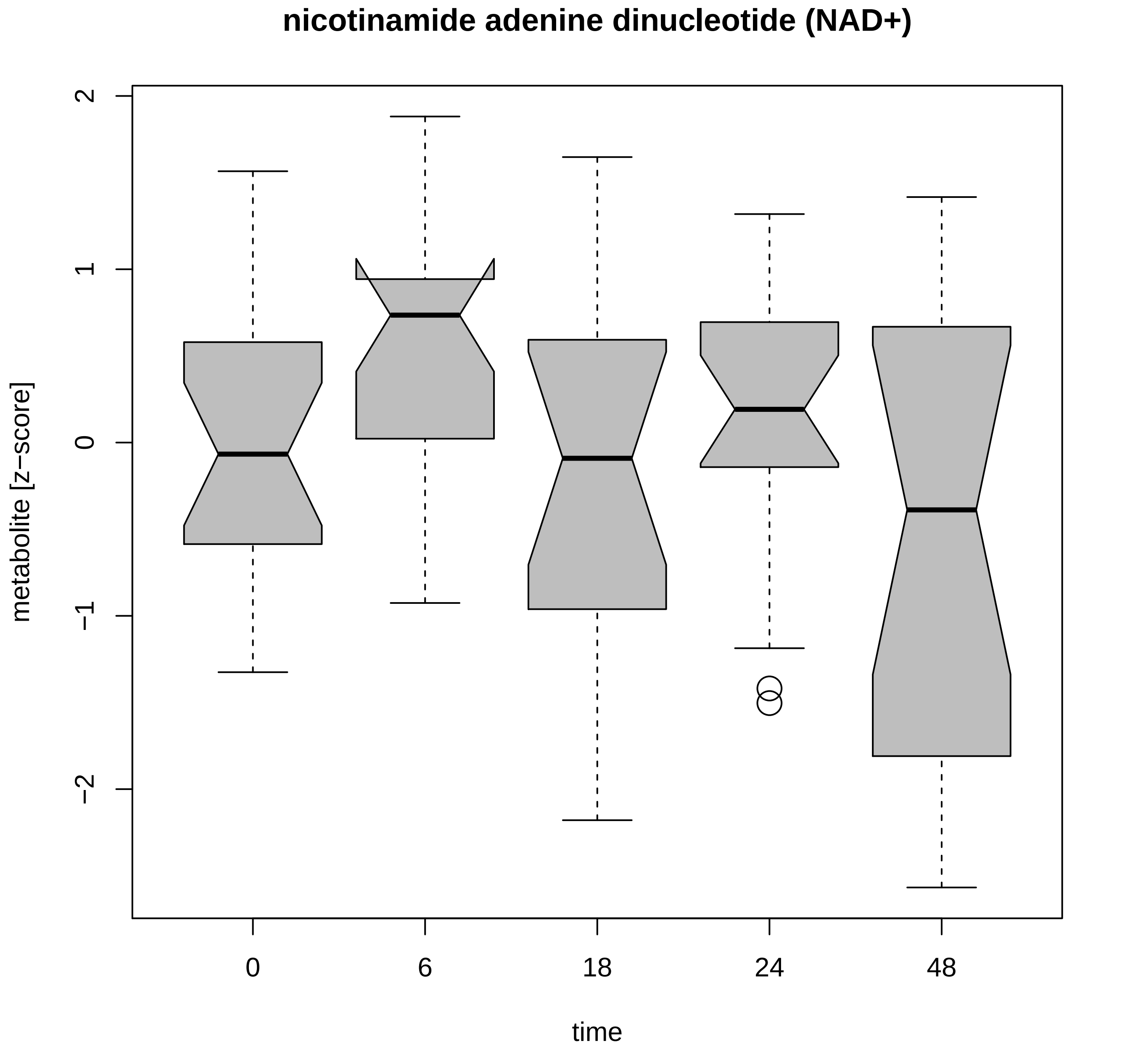
nicotinamide adenine dinucleotide (NAD+)
metabolite [z−score]
2
1
0
−1
−2
0
6
18
24
48
time

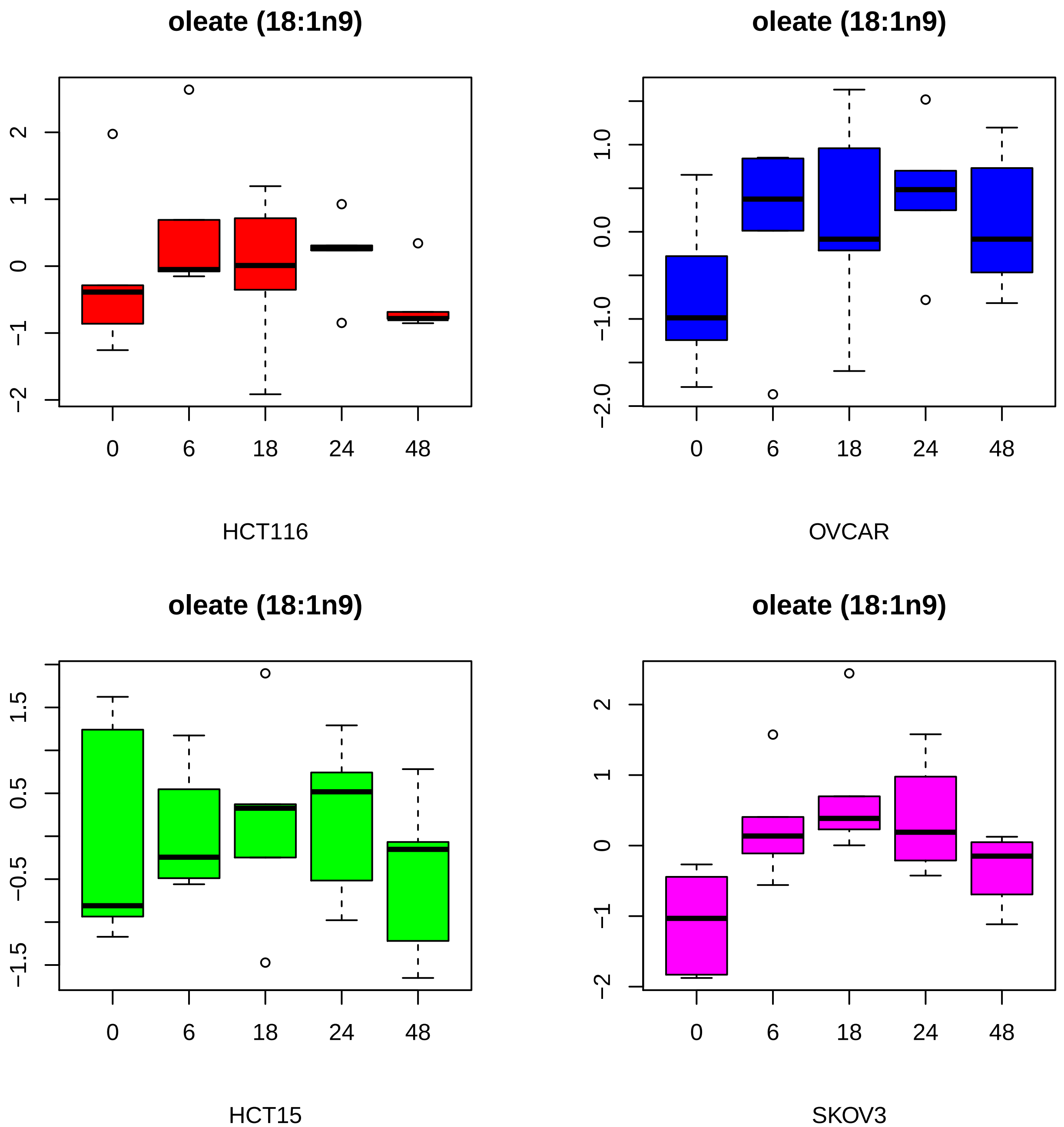

oleate (18:1n9)
HCT116
oleate (18:1n9)
OVCAR
oleate (18:1n9)
HCT15
oleate (18:1n9)
SKOV3
0
6
18
24
48

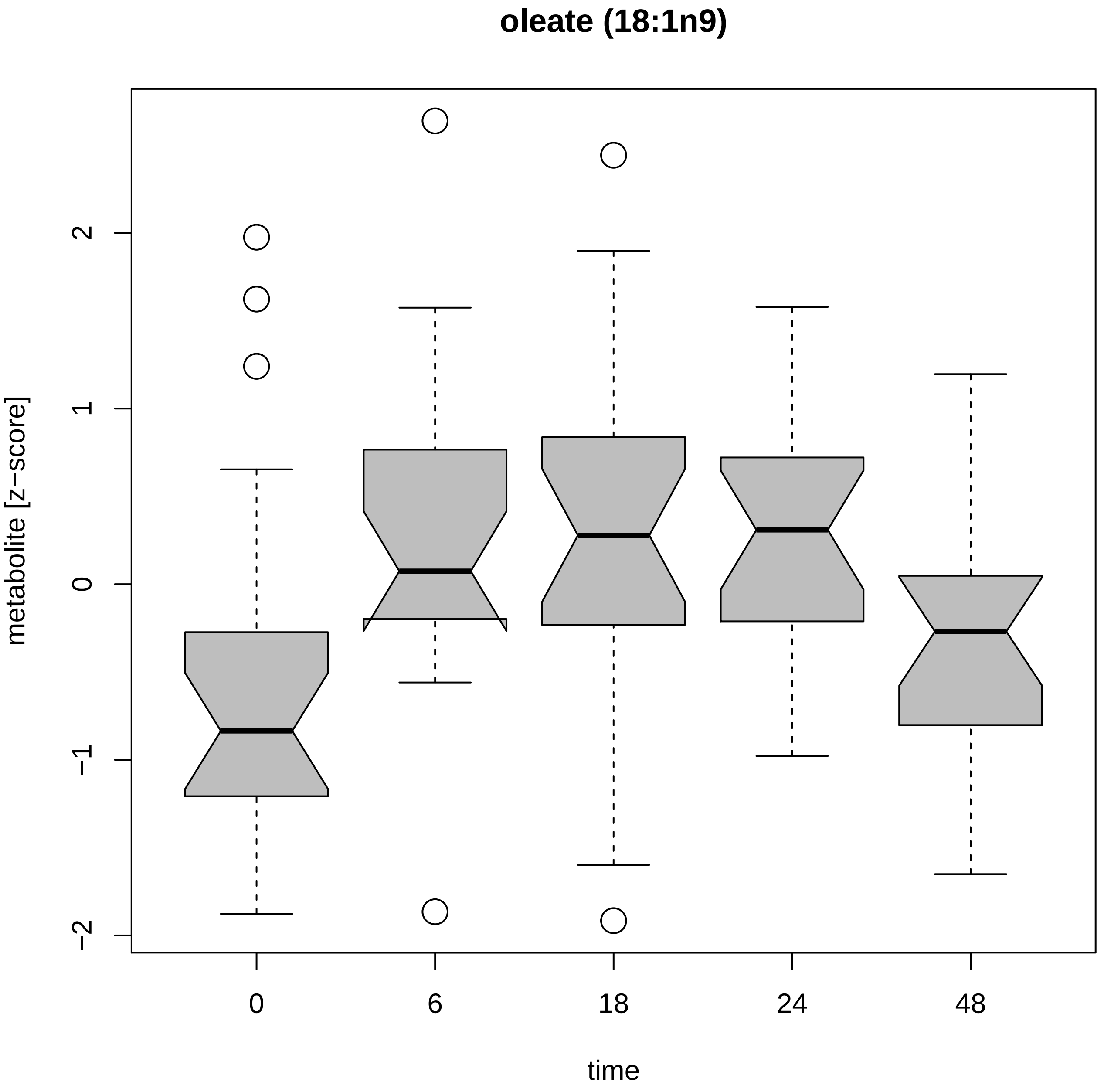

oleate (18:1n9)
metabolite [z−score]
2
1
0
−1
−2
0
6
18
24
48
time

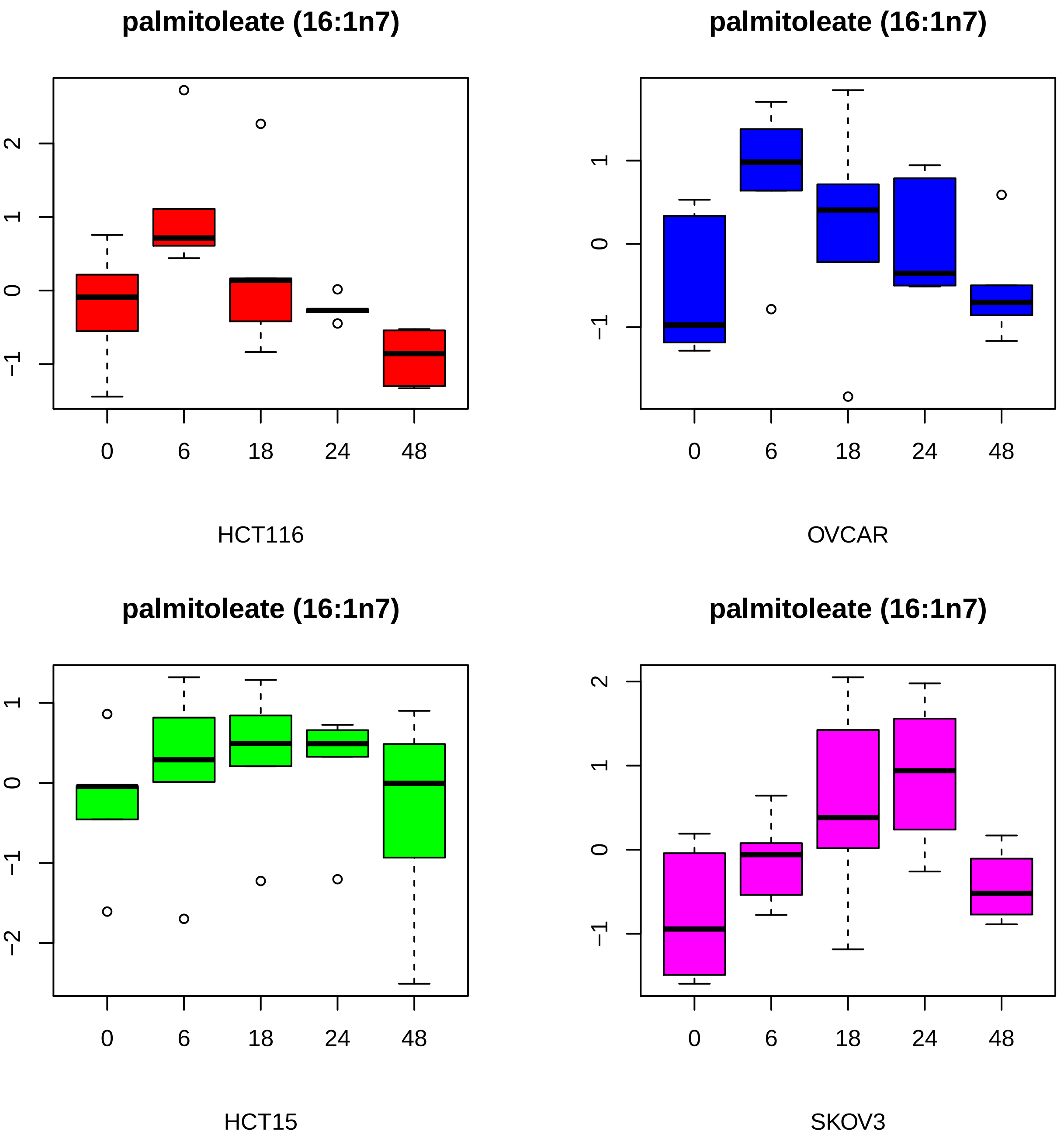
palmitoleate (16:1n7)
HCT116
palmitoleate (16:1n7)
OVCAR
palmitoleate (16:1n7)
HCT15
palmitoleate (16:1n7)
SKOV3
0
6
18
24
48

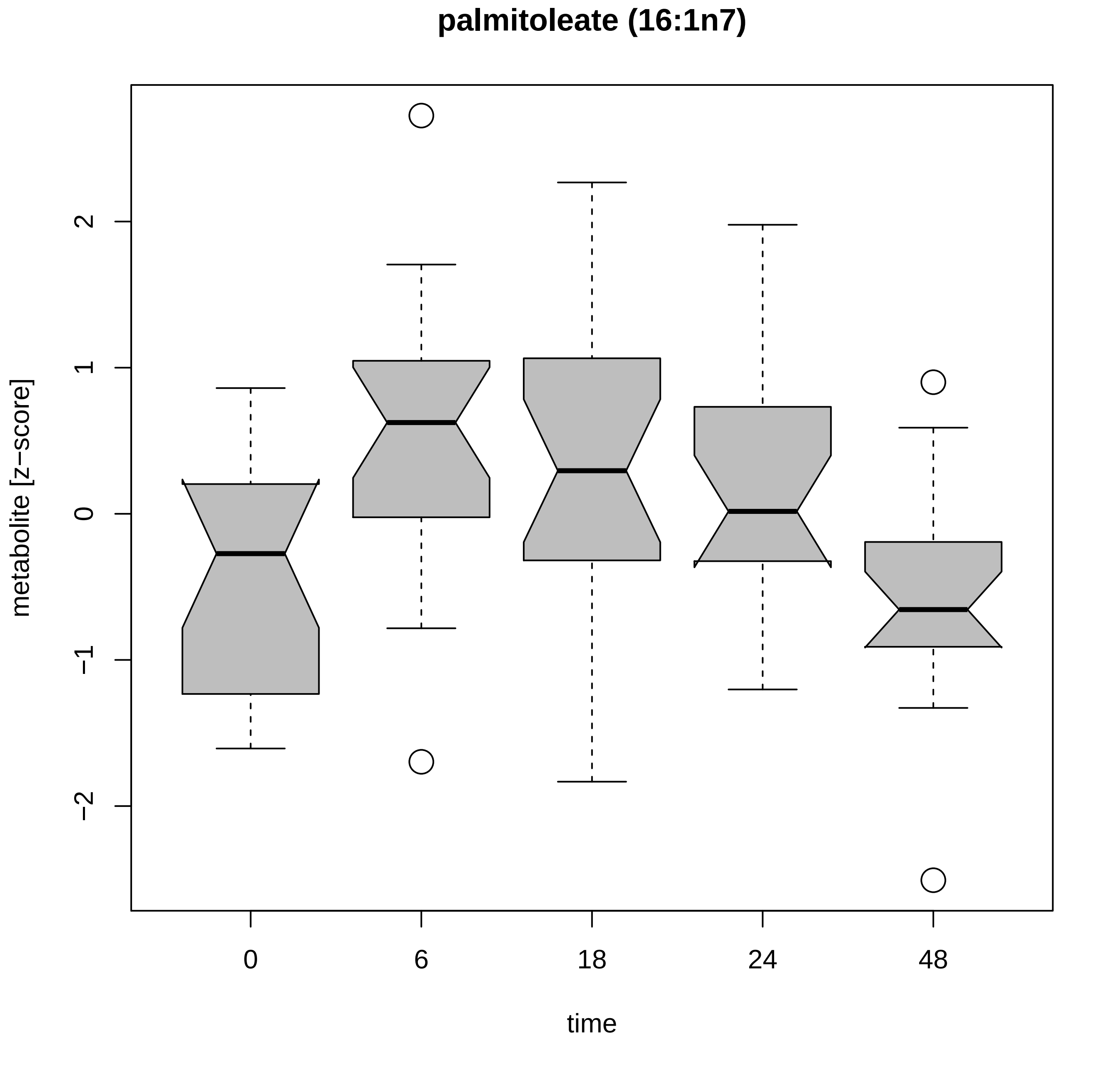

palmitoleate (16:1n7)
metabolite [z−score]
2
1
0
−1
−2
0
6
18
24
48
time

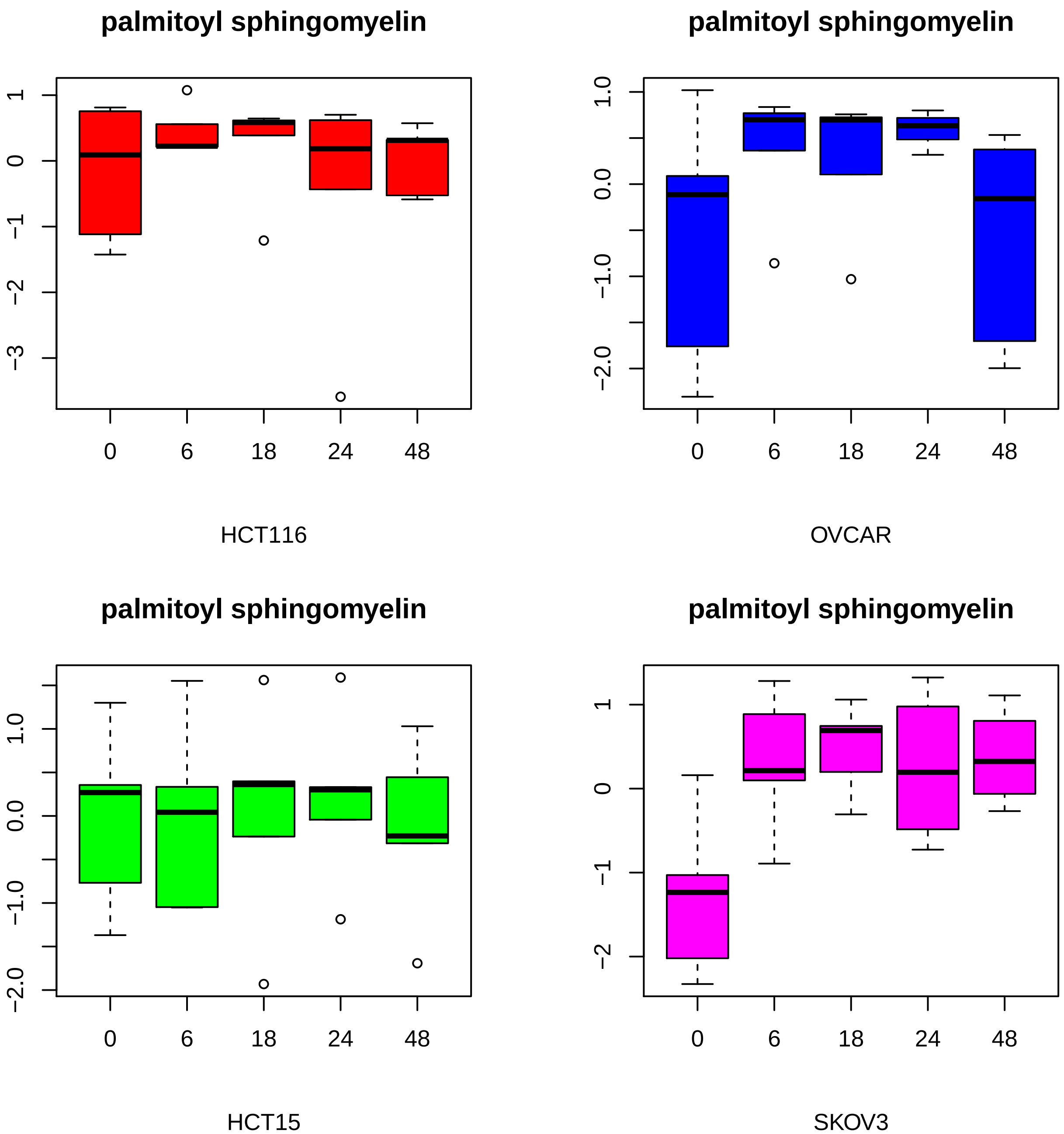

palmitoyl sphingomyelin
1
0
−1
−2
−3
0
6
18
24
48
HCT116
palmitoyl sphingomyelin
1.0
0.0
−1.0
−2.0
0
6
18
24
48
OVCAR
palmitoyl sphingomyelin
1.0
0.0
−1.0
−2.0
0
6
18
24
48
HCT15
palmitoyl sphingomyelin
1
0
−1
−2
0
6
18
24
48
SKOV3

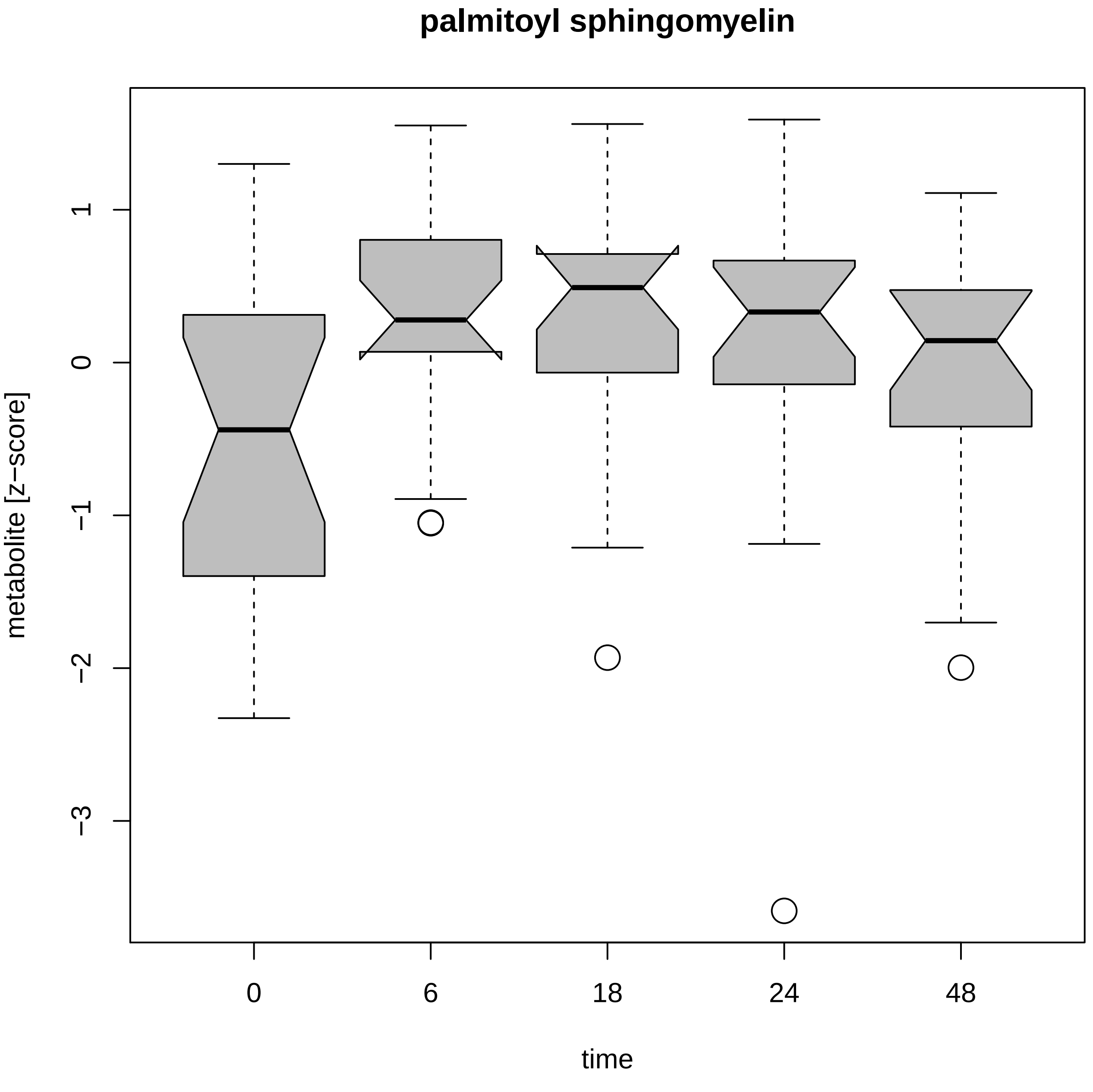
palmitoyl sphingomyelin
metabolite [z−score]
1
0
−1
−2
−3
0
6
18
24
48
time

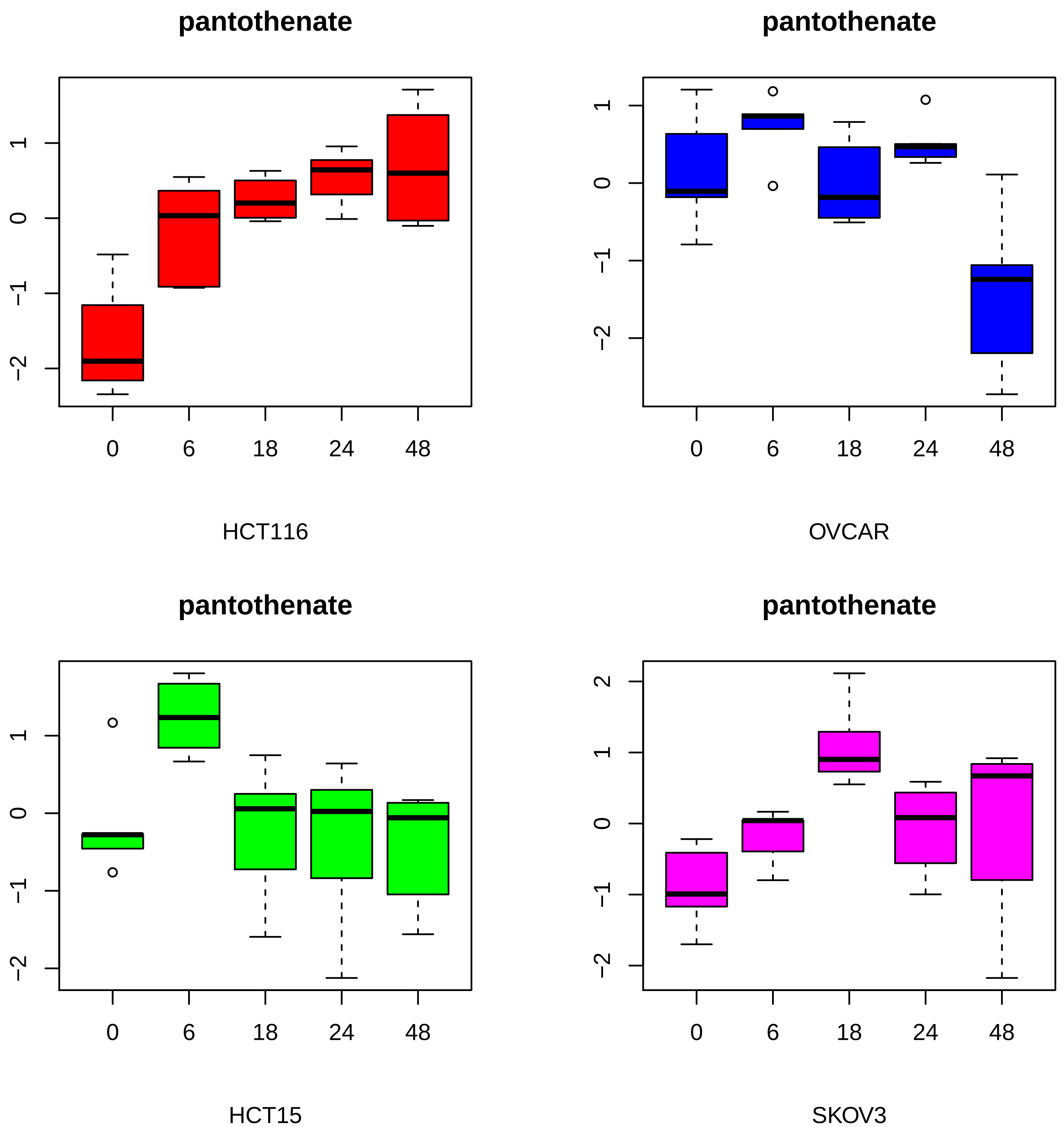
pantothenate
1
0
−1
−2
0
6
18
24
48
HCT116
pantothenate
1
0
−1
−2
0
6
18
24
48
OVCAR
pantothenate
1
0
−1
−2
0
6
18
24
48
HCT15
pantothenate
2
1
0
−1
−2
0
6
18
24
48
SKOV3

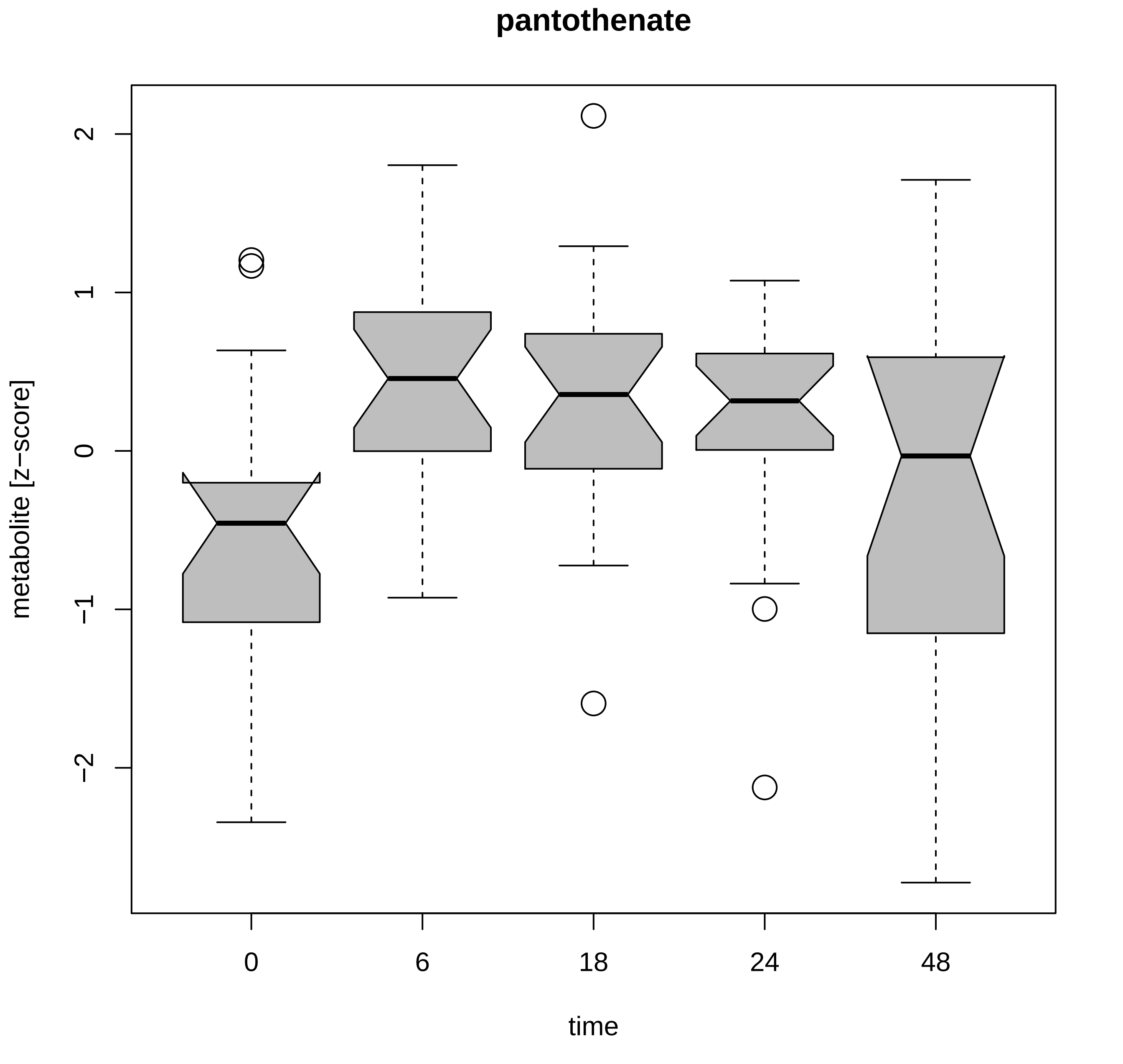

pantothenate
metabolite [z−score]
2
1
0
−1
−2
0
6
18
24
48
time

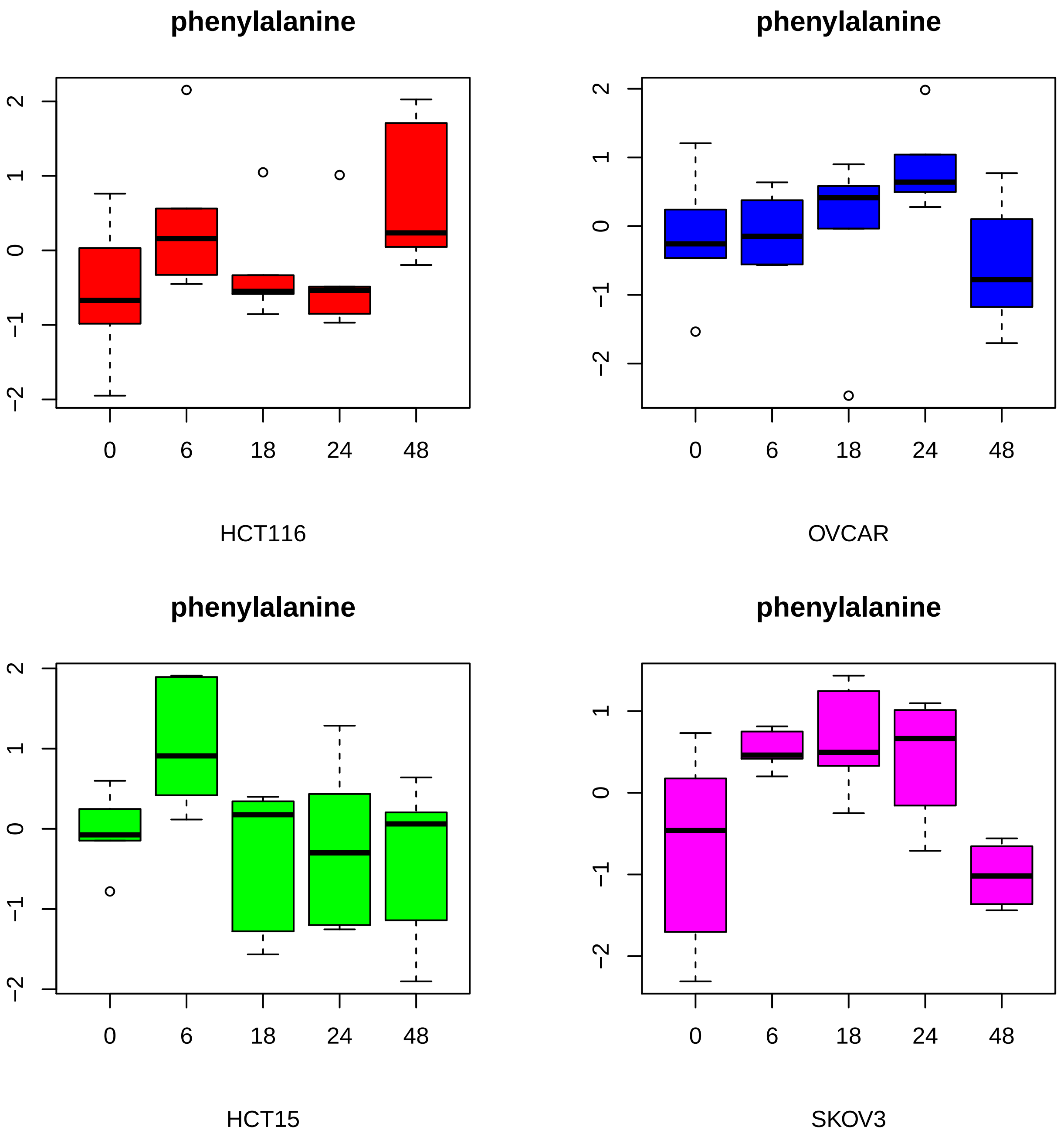
phenylalanine
2
1
0
−1
−2
0
6
18
24
48
HCT116
phenylalanine
OVCAR
phenylalanine
HCT15
phenylalanine
SKOV3

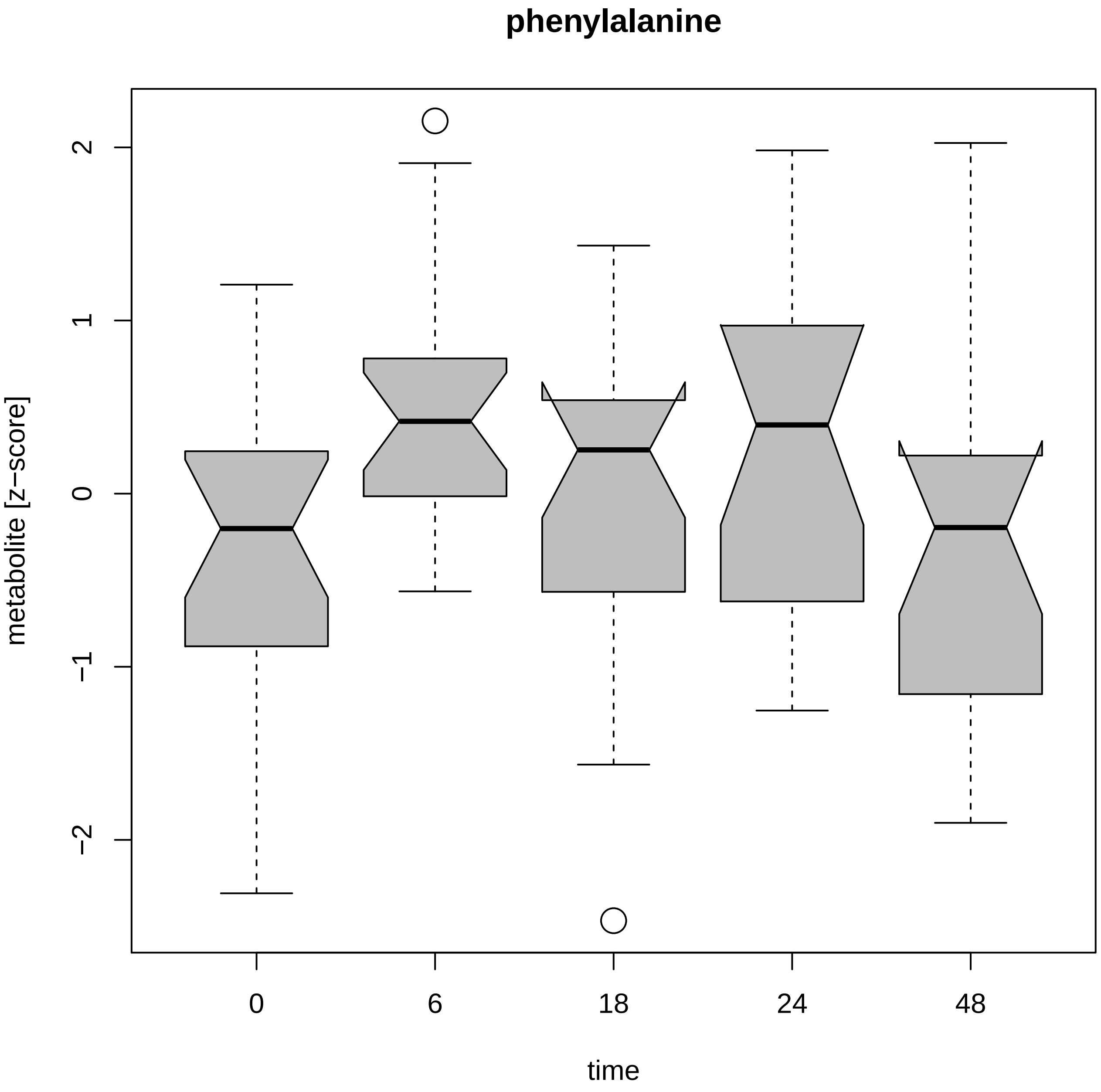
phenylalanine
metabolite [z−score]
2
1
0
−1
−2
0
6
18
24
48
time

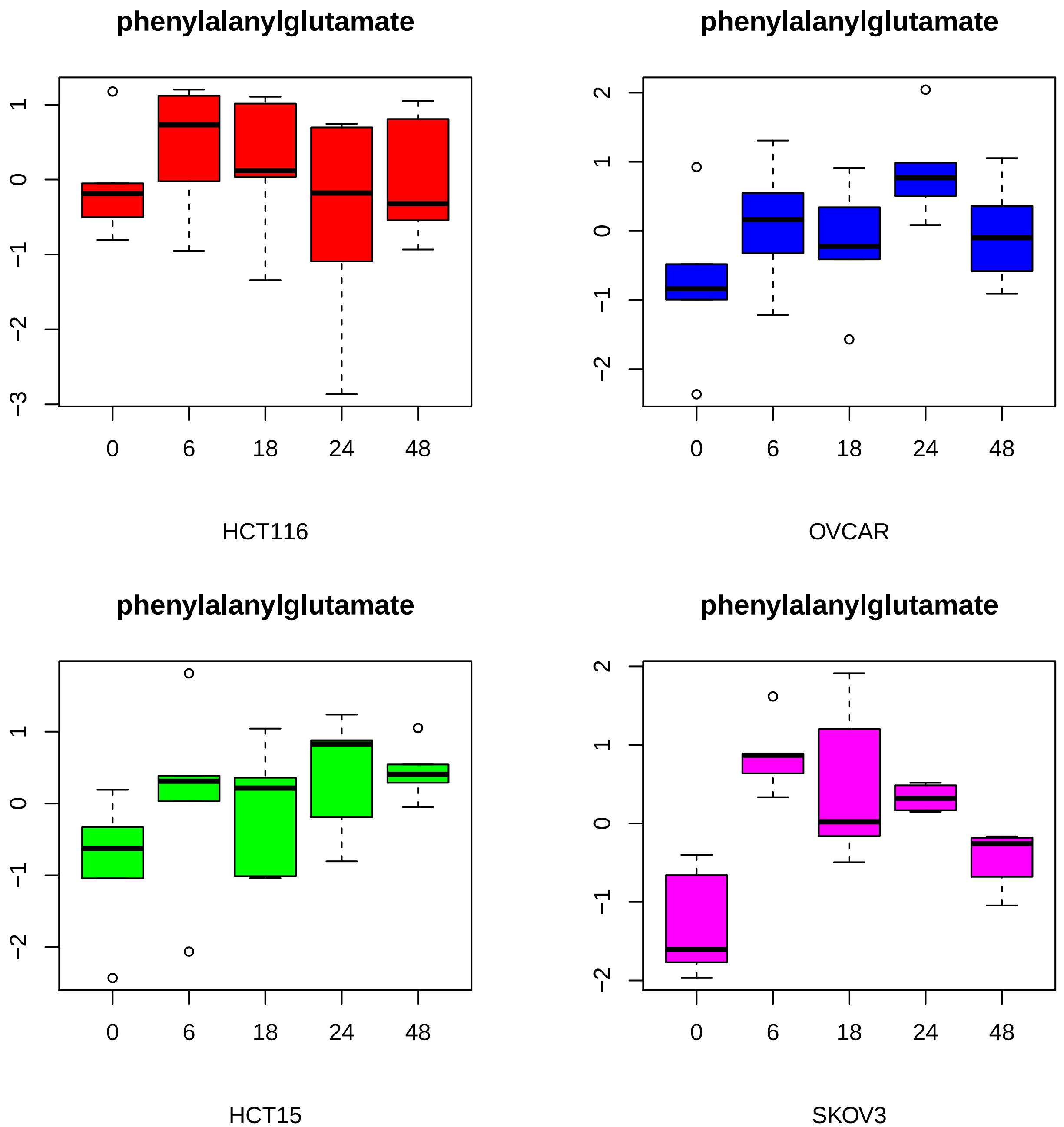
phenylalanylglutamate
HCT116
phenylalanylglutamate
OVCAR
phenylalanylglutamate
HCT15
phenylalanylglutamate
SKOV3
0
6
18
24
48

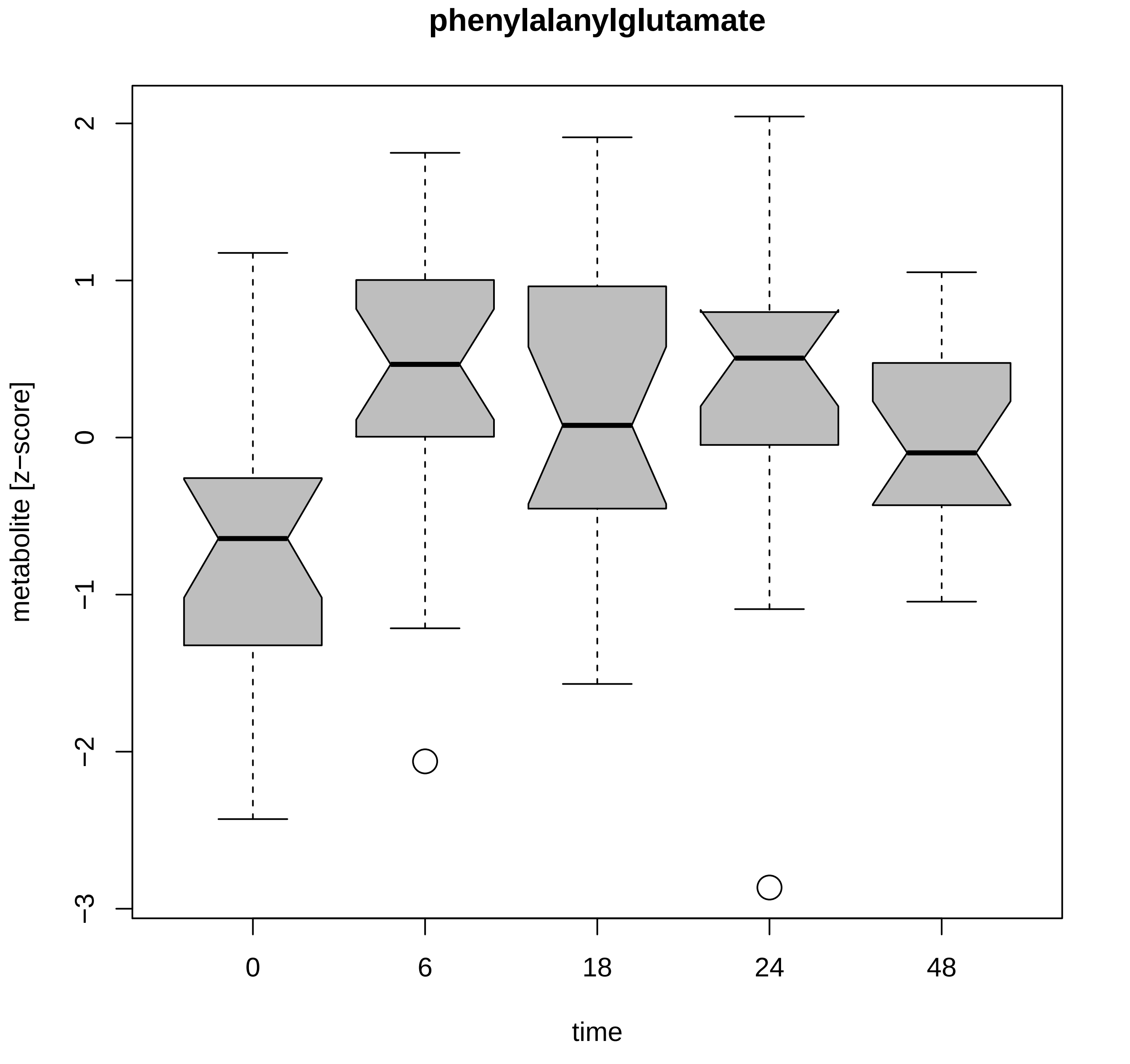
phenylalanylglutamate
metabolite [z−score]
2
1
0
−1
−2
−3
0
6
18
24
48
time

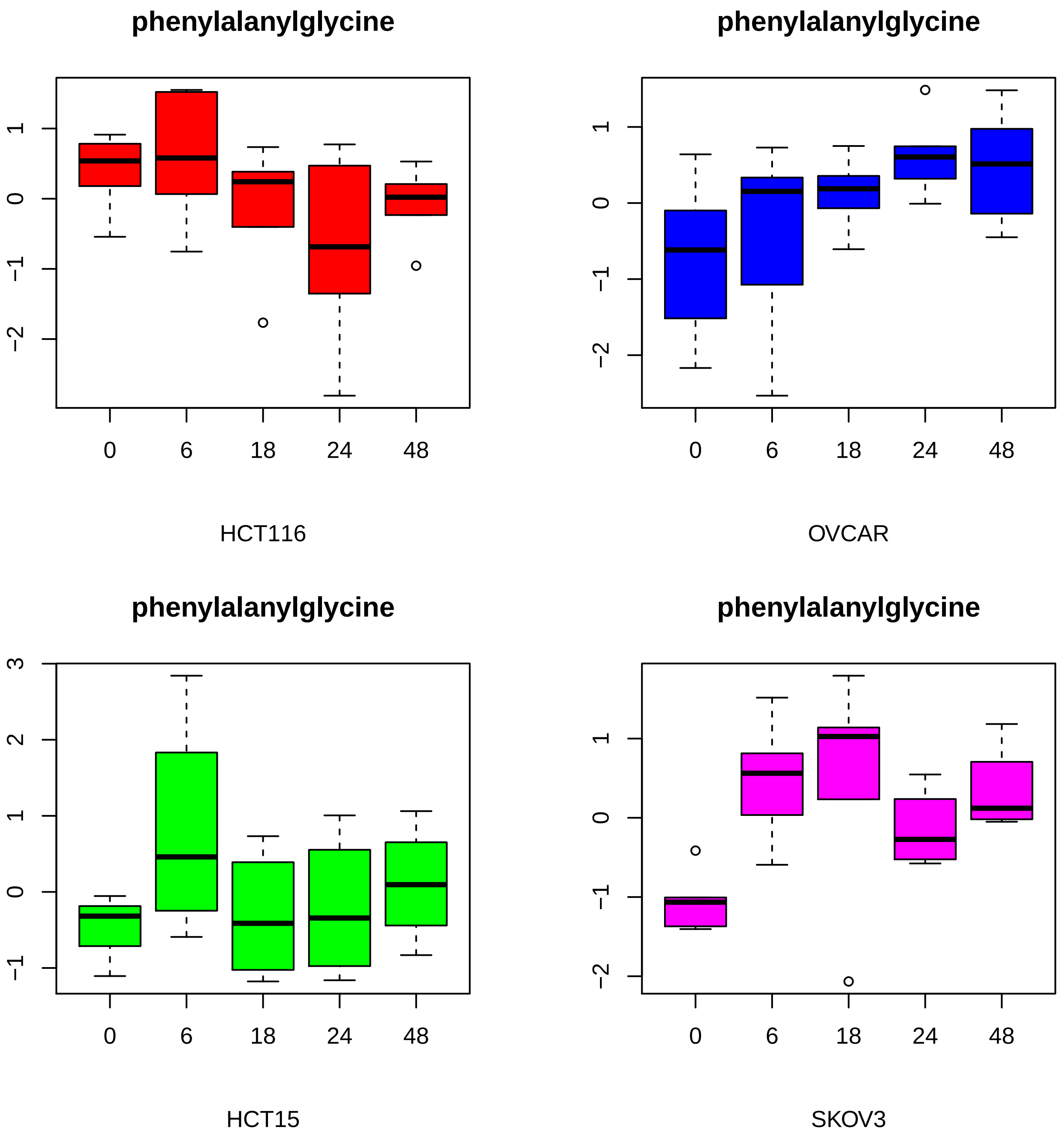

phenylalanylglycine
1
0
−1
−2
0
6
18
24
48
HCT116
phenylalanylglycine
1
0
−1
−2
0
6
18
24
48
OVCAR
phenylalanylglycine
3
2
1
0
−1
0
6
18
24
48
HCT15
phenylalanylglycine
1
0
−1
−2
0
6
18
24
48
SKOV3

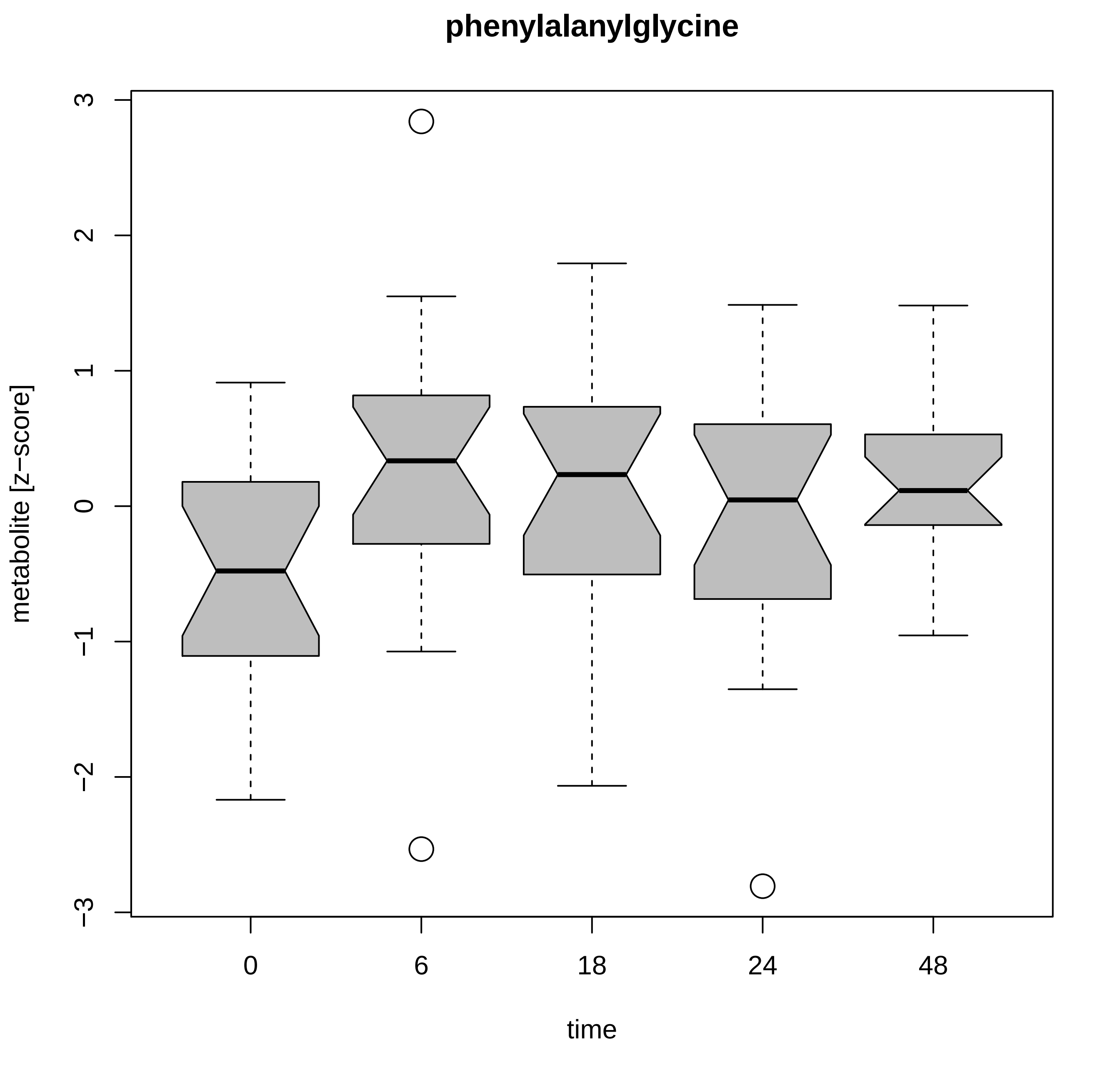
phenylalanylglycine
metabolite [z−score]
3
2
1
0
−1
−2
−3
0
6
18
24
48
time

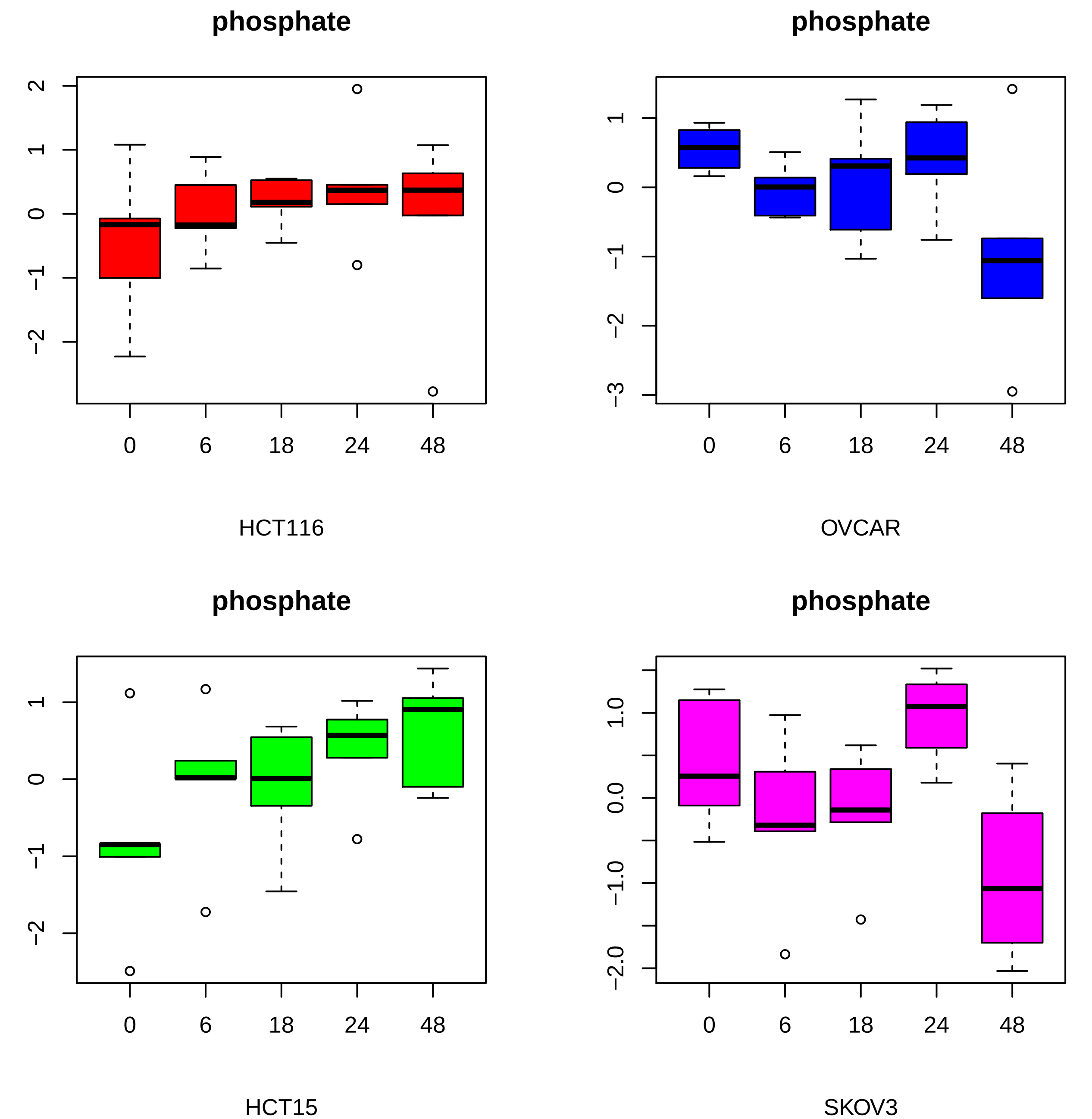
phosphate
2
1
0
−1
−2
0
6
18
24
48
HCT116
phosphate
1
0
−1
−2
−3
0
6
18
24
48
OVCAR
phosphate
1
0
−1
−2
0
6
18
24
48
HCT15
phosphate
1.0
0.0
−1.0
−2.0
0
6
18
24
48
SKOV3

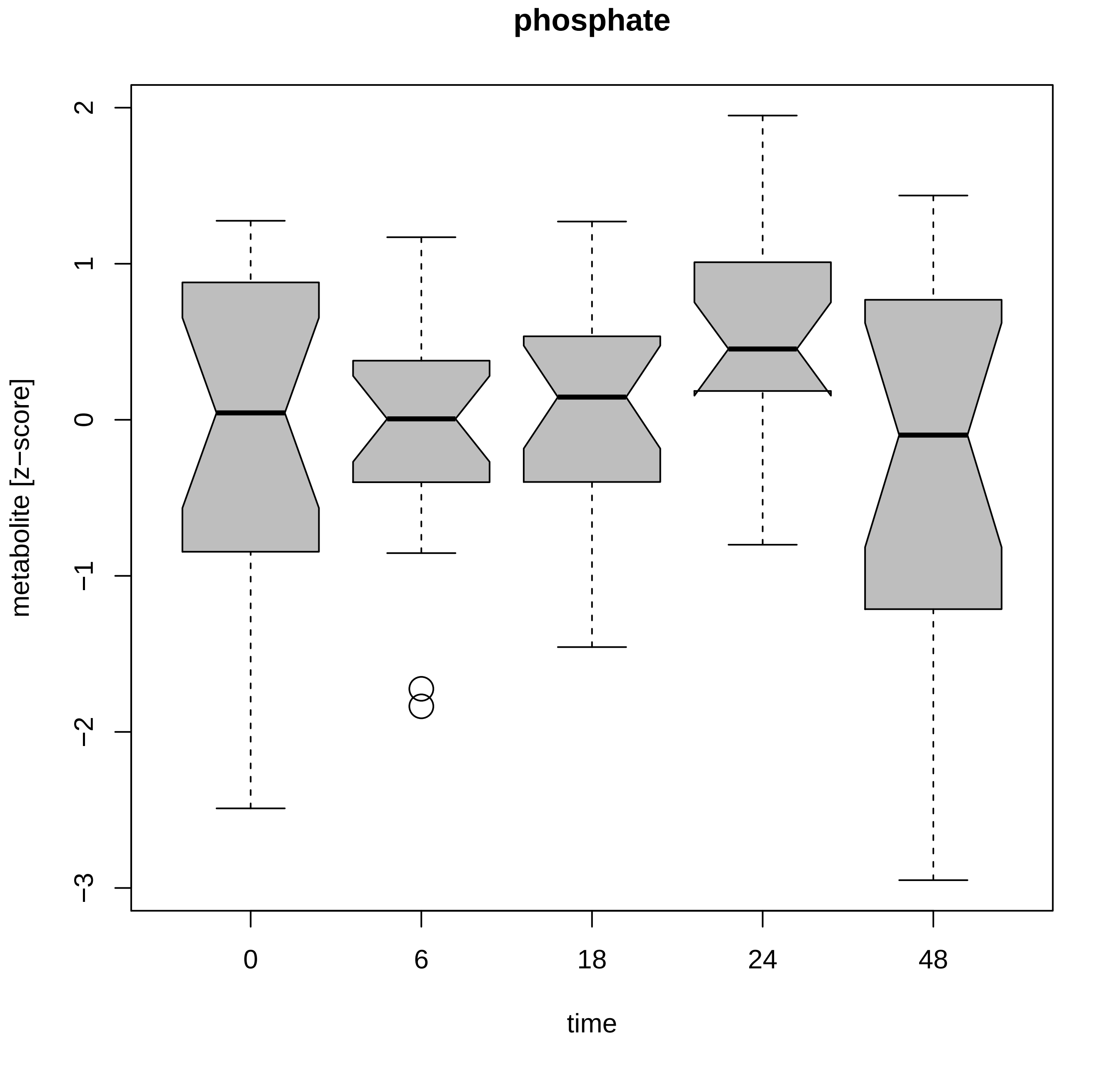
phosphate
metabolite [z−score]
2
1
0
−1
−2
−3
0
6
18
24
48
time

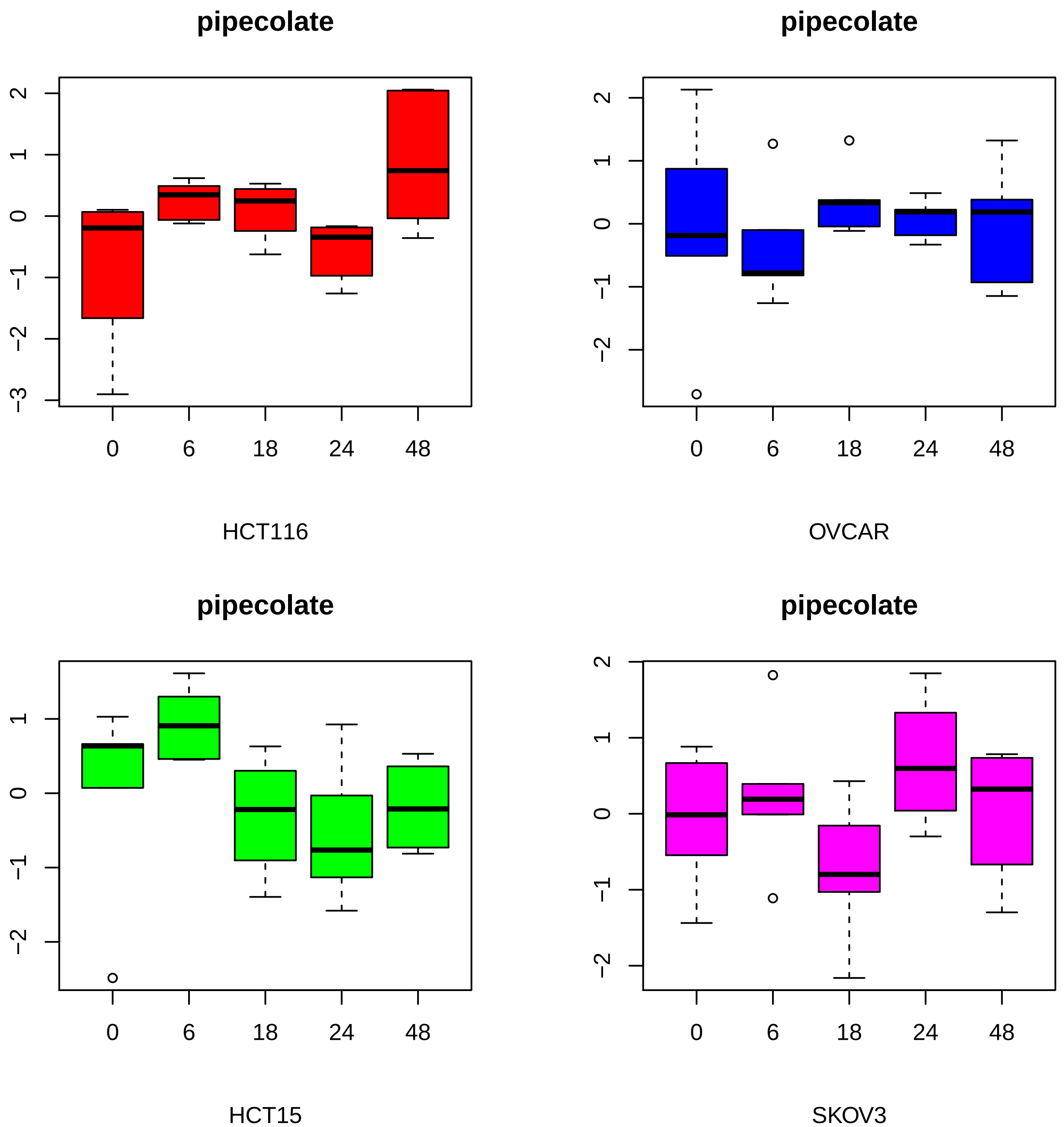
pipecolate
HCT116
pipecolate
OVCAR
pipecolate
HCT15
pipecolate
SKOV3
0
6
18
24
48
2
1
0
−1
−2
−3

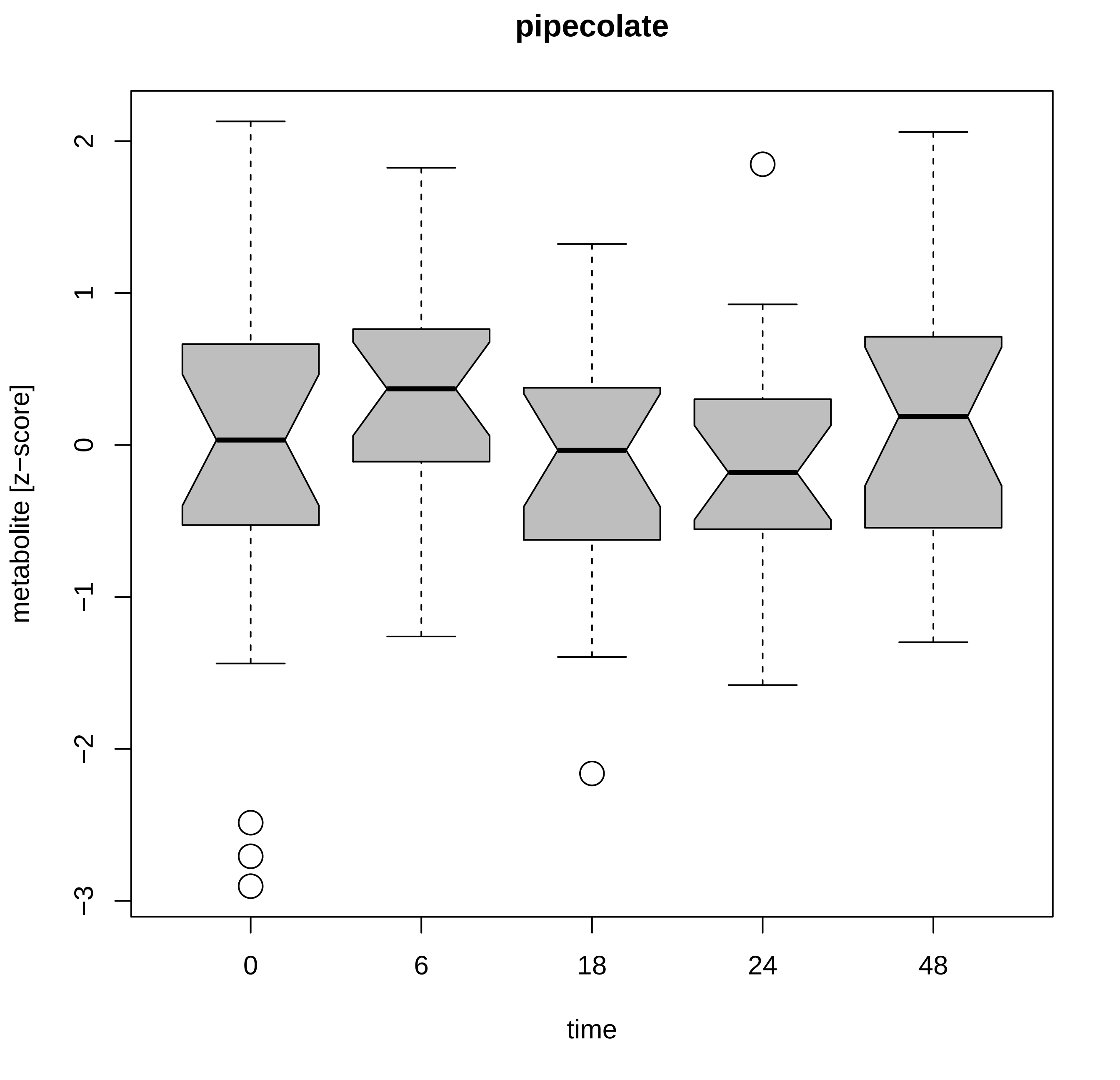
pipecolate
metabolite [z−score]
2
1
0
−1
−2
−3
0
6
18
24
48
time

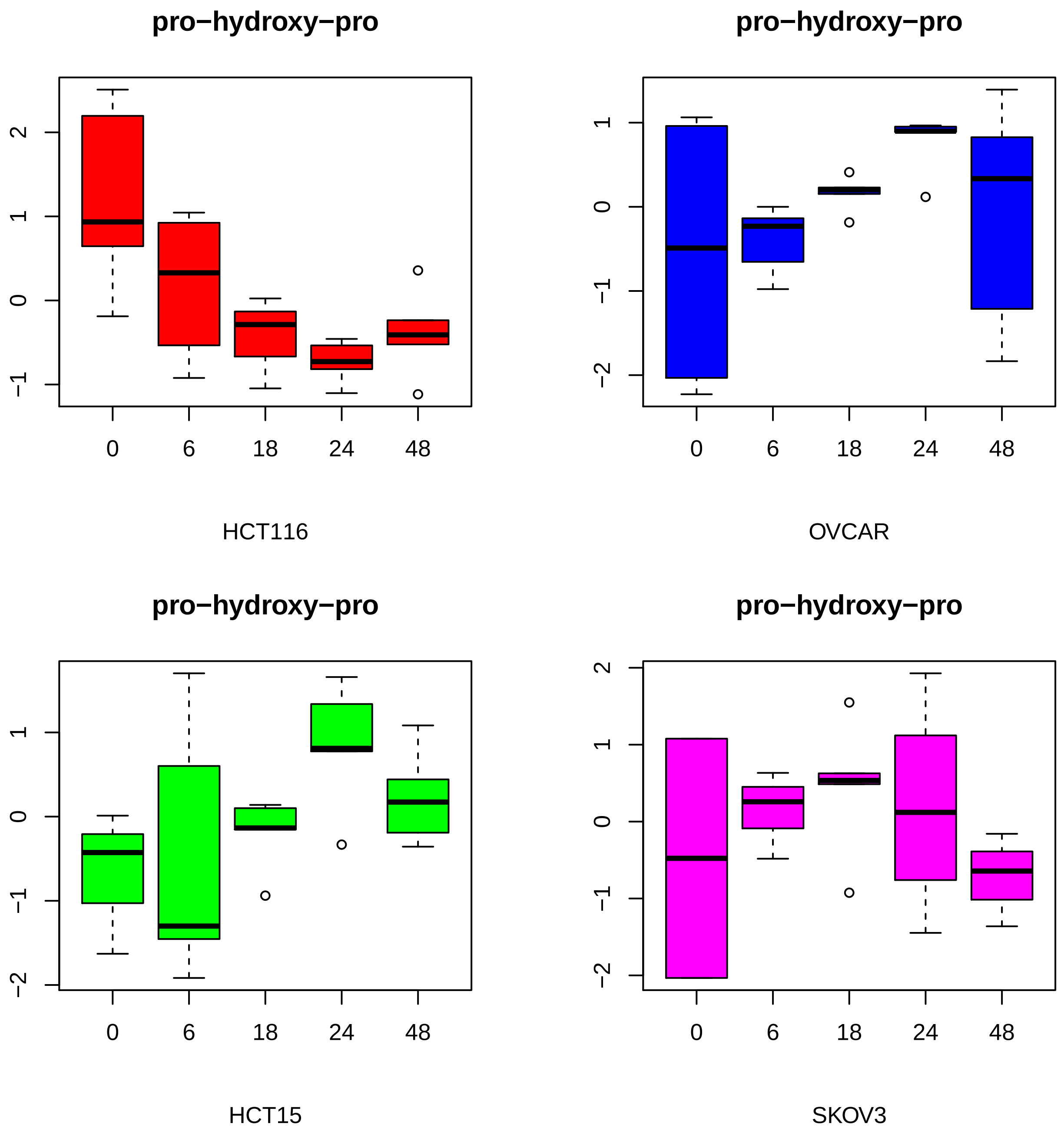
pro−hydroxy−pro
2
1
0
−1
0
6
18
24
48
HCT116
pro−hydroxy−pro
1
0
−1
−2
0
6
18
24
48
OVCAR
pro−hydroxy−pro
1
0
−1
−2
0
6
18
24
48
HCT15
pro−hydroxy−pro
2
1
0
−1
−2
0
6
18
24
48
SKOV3

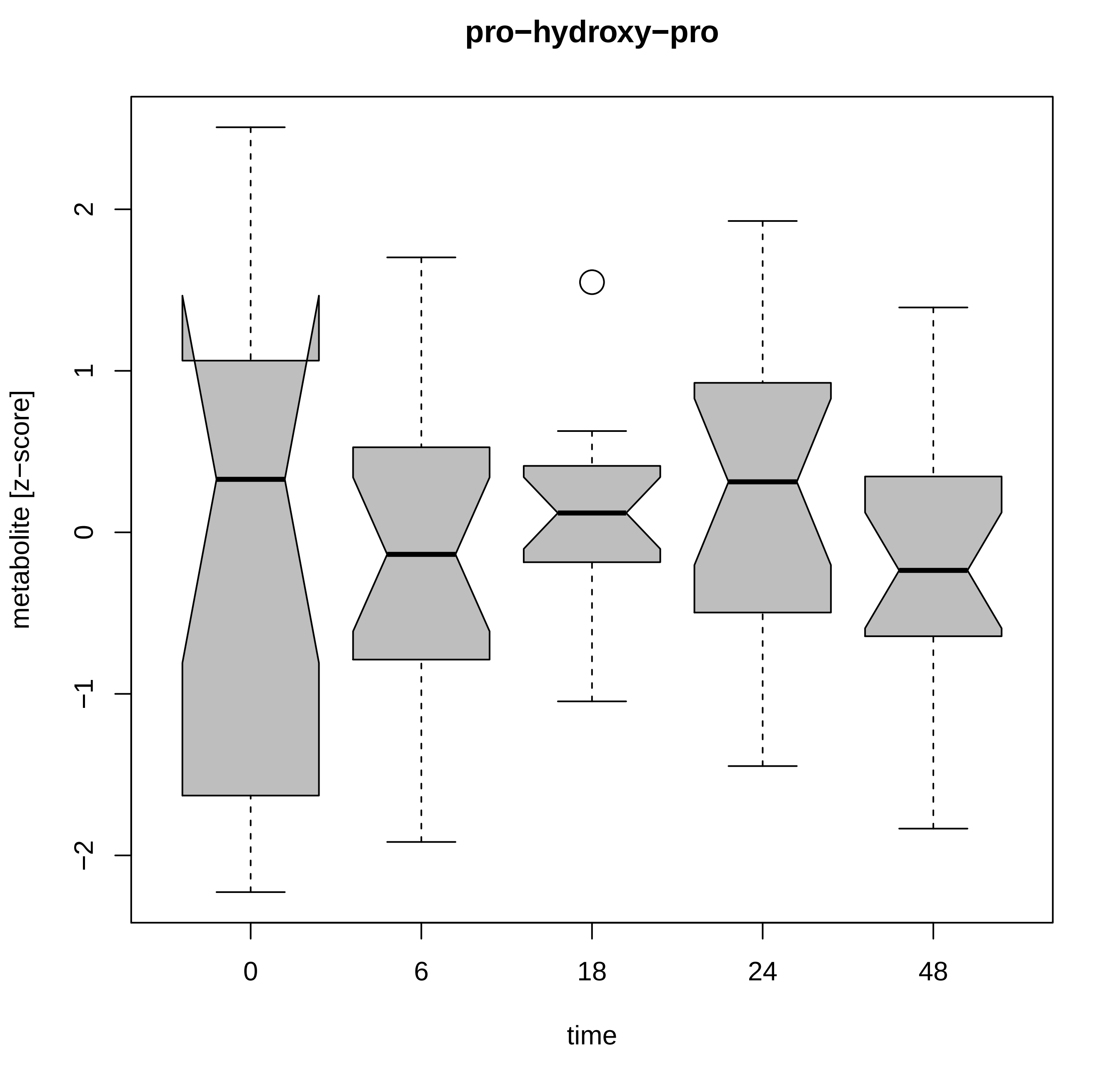

pro−hydroxy−pro
metabolite [z−score]
−2
−1
0
1
2
0
6
18
24
48
time

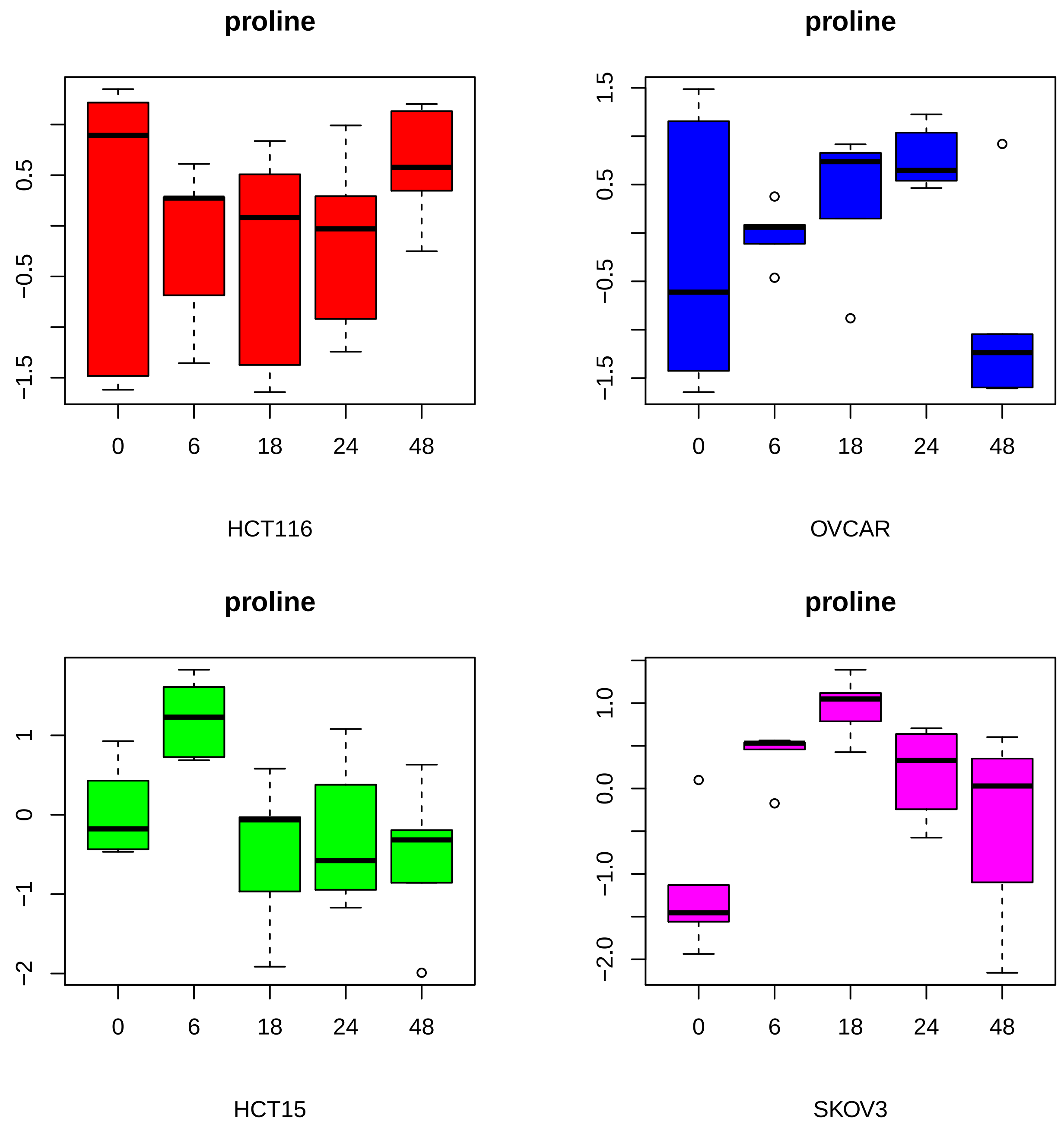
proline
0.5
−0.5
−1.5
0
6
18
24
48
HCT116
proline
1.5
0.5
−0.5
−1.5
0
6
18
24
48
OVCAR
proline
1
0
−1
−2
0
6
18
24
48
HCT15
proline
1.0
0.0
−1.0
−2.0
0
6
18
24
48
SKOV3

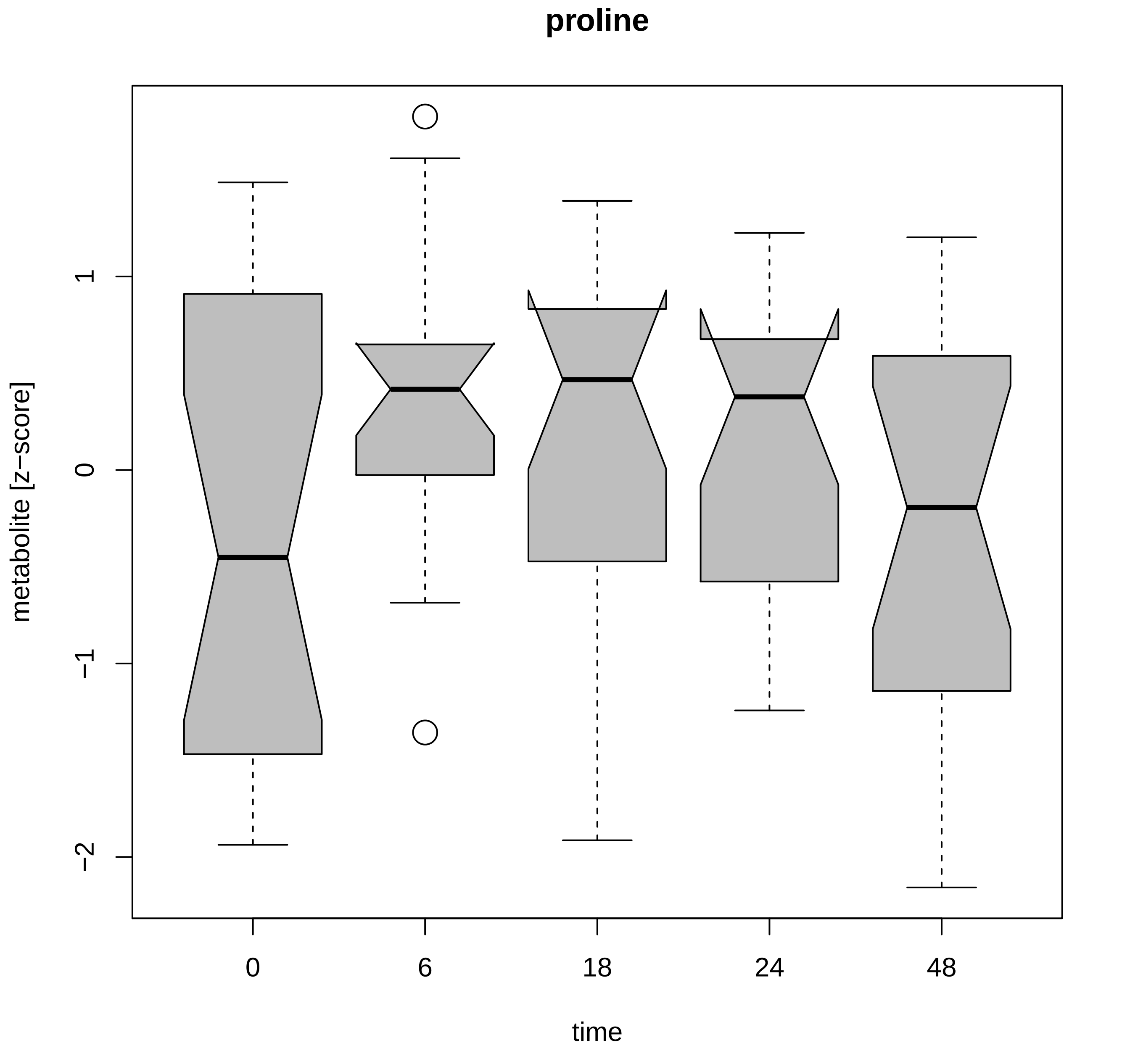
proline
metabolite [z−score]
1
0
−1
−2
0
6
18
24
48
time

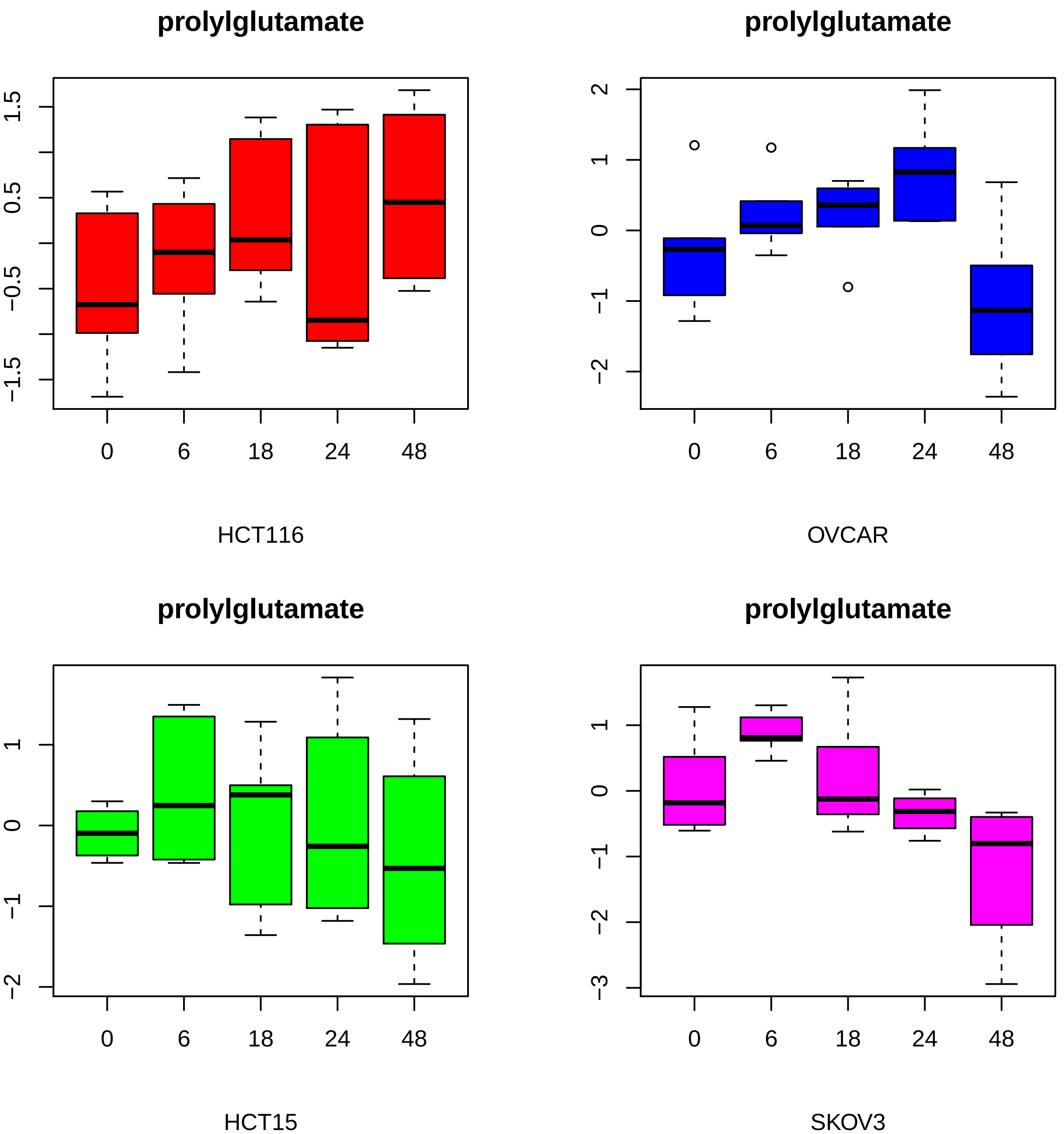

prolylglutamate
1.5
0.5
−0.5
−1.5
0
6
18
24
48
HCT116
prolylglutamate
2
1
0
−1
−2
0
6
18
24
48
OVCAR
prolylglutamate
1
0
−1
−2
0
6
18
24
48
HCT15
prolylglutamate
1
0
−1
−2
−3
0
6
18
24
48
SKOV3

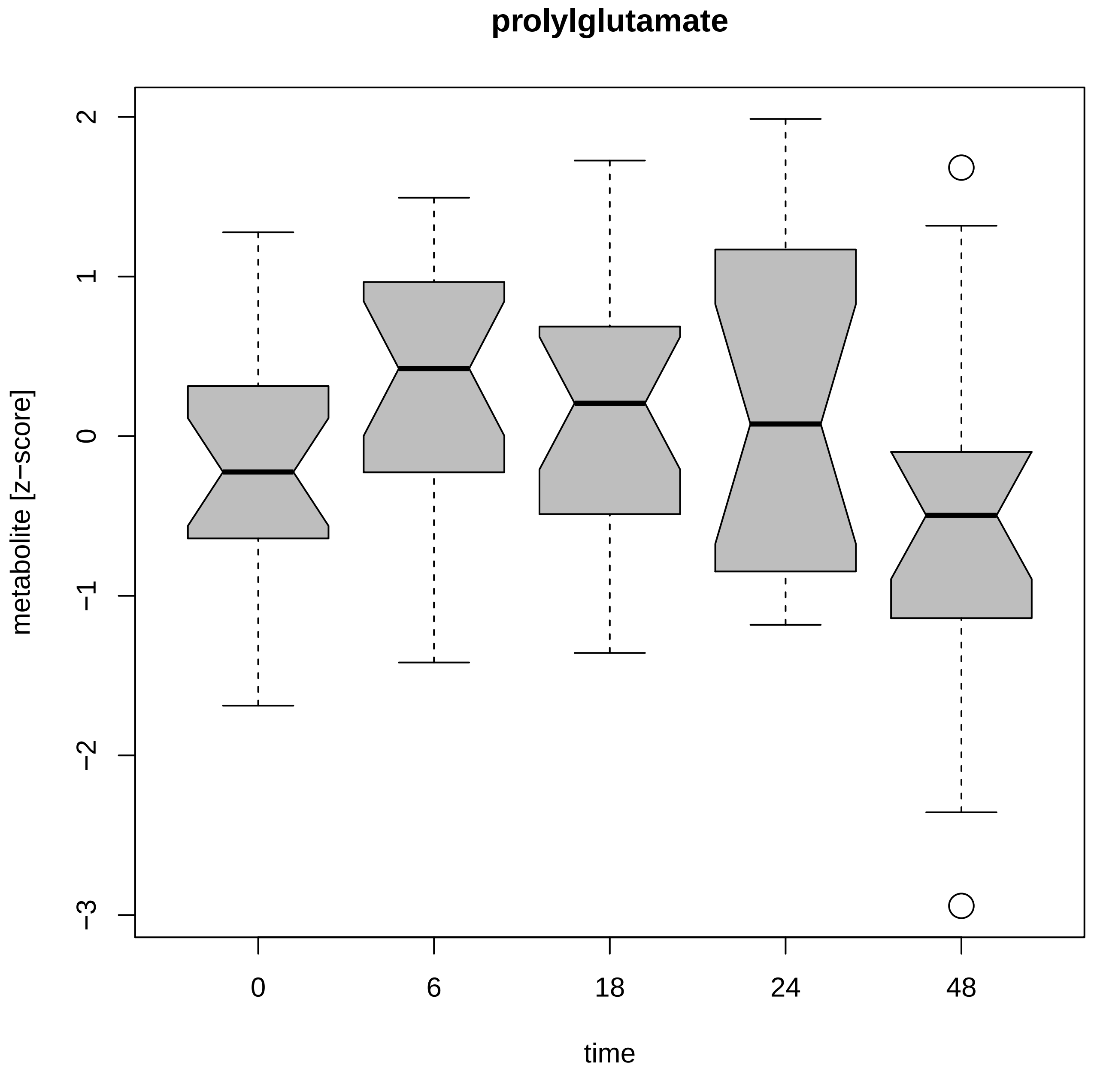
prolylglutamate
metabolite [z−score]
time
2
1
0
−1
−2
−3
0
6
18
24
48

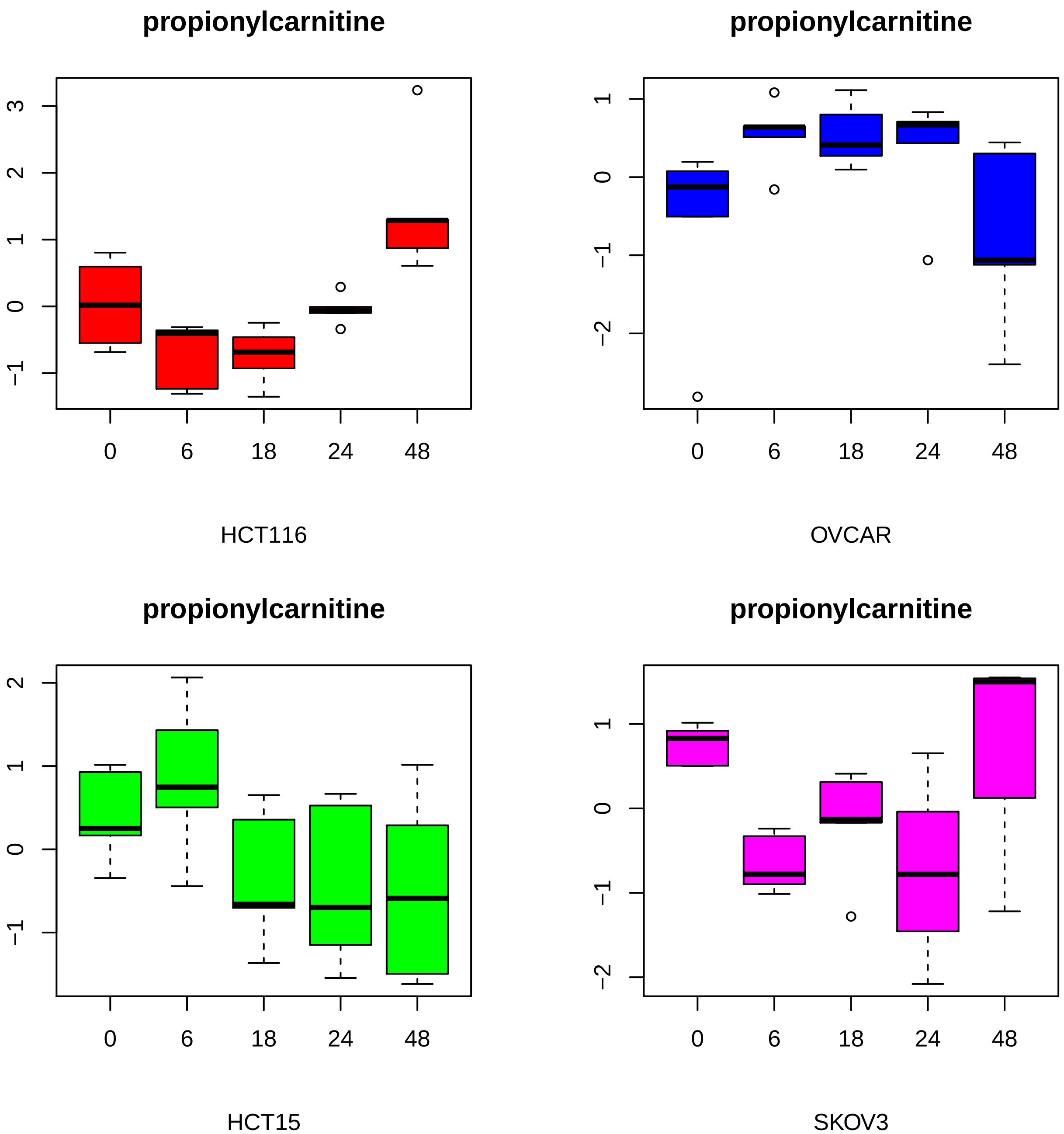
propionylcarnitine
HCT116
OVCAR
HCT15
SKOV3
0
6
18
24
48

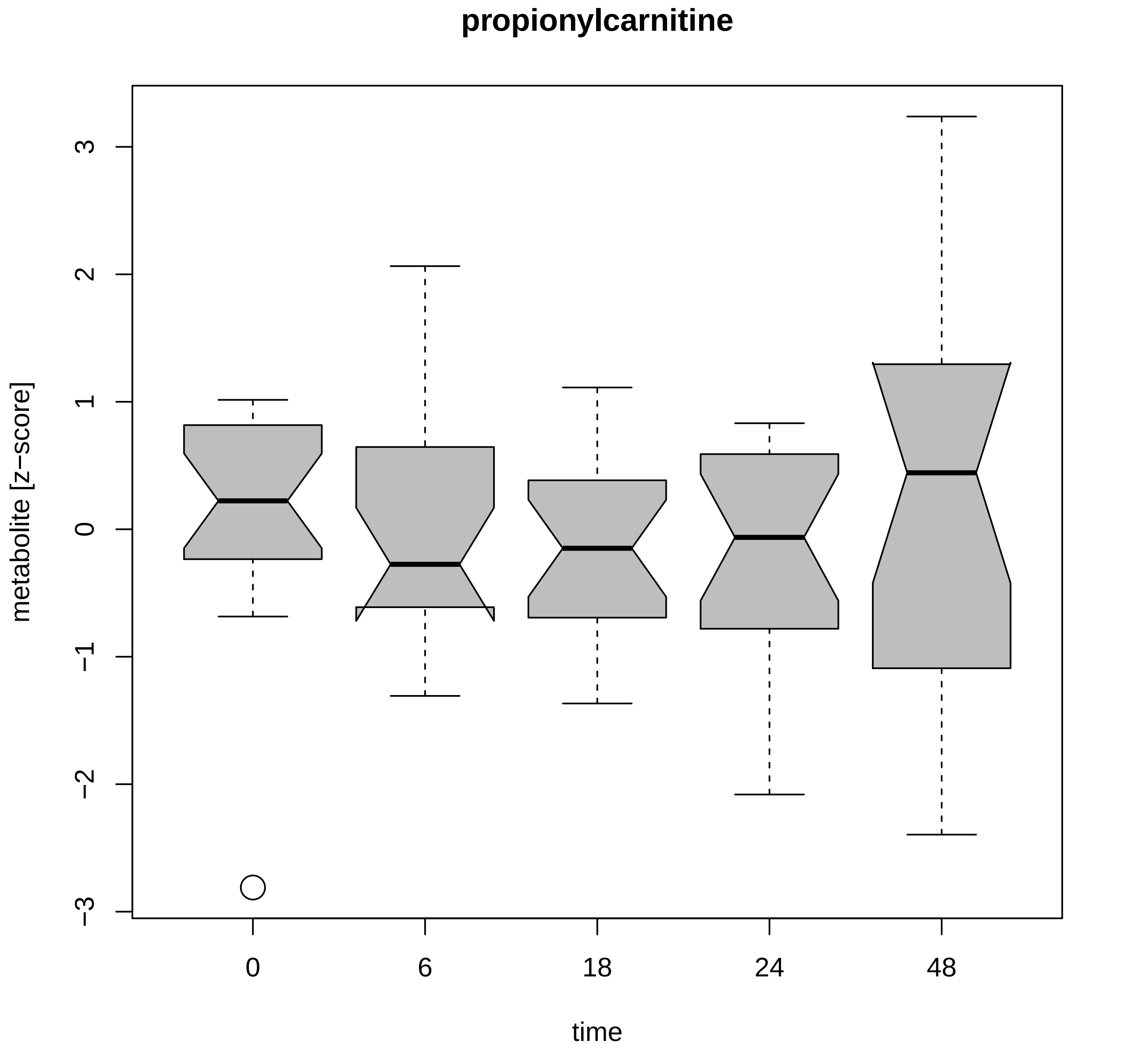
propionylcarnitine
metabolite [z−score]
3
2
1
0
−1
−2
−3
0
6
18
24
48
time

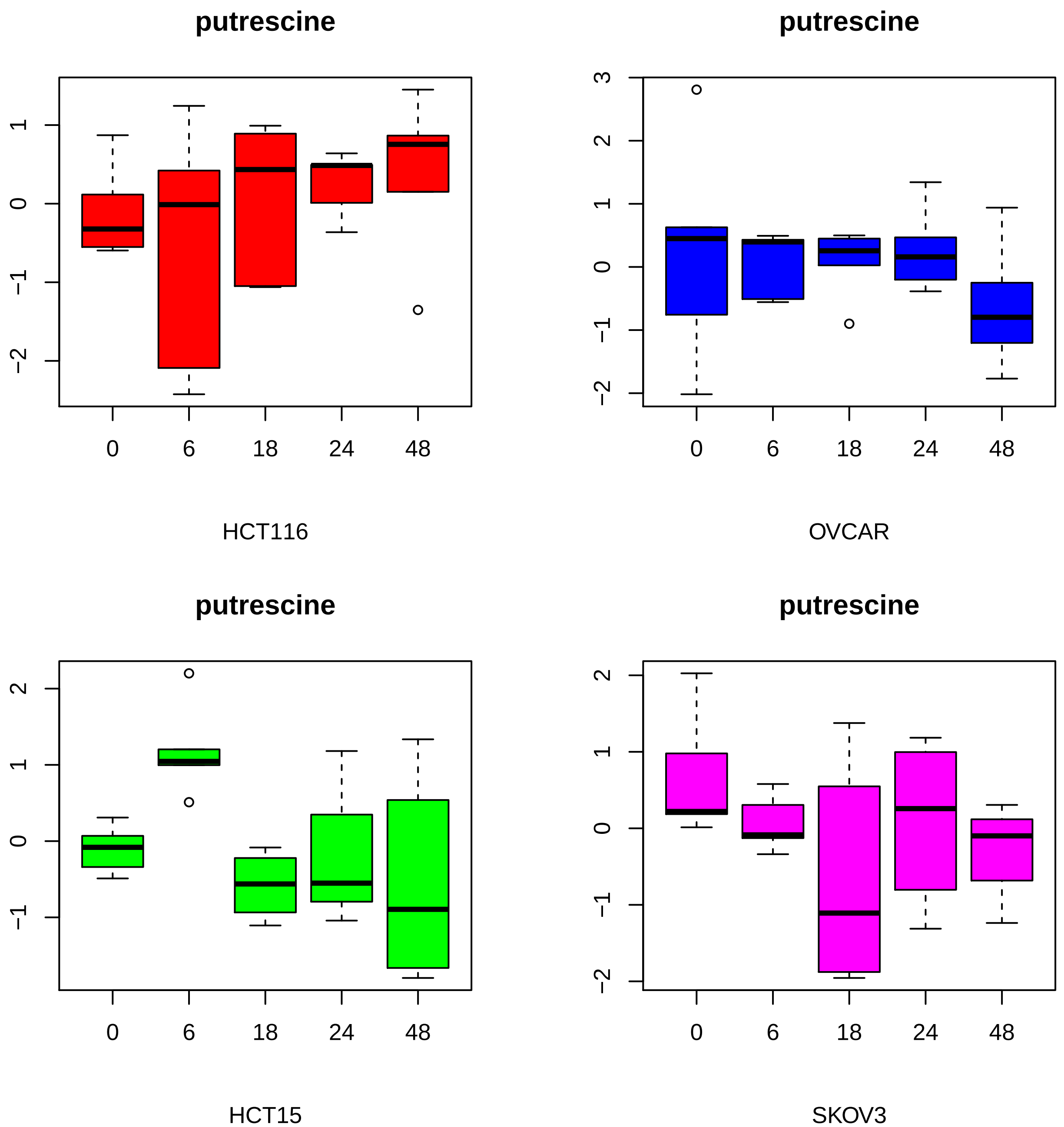
putrescine
HCT116
putrescine
OVCAR
putrescine
HCT15
putrescine
SKOV3
0
6
18
24
48

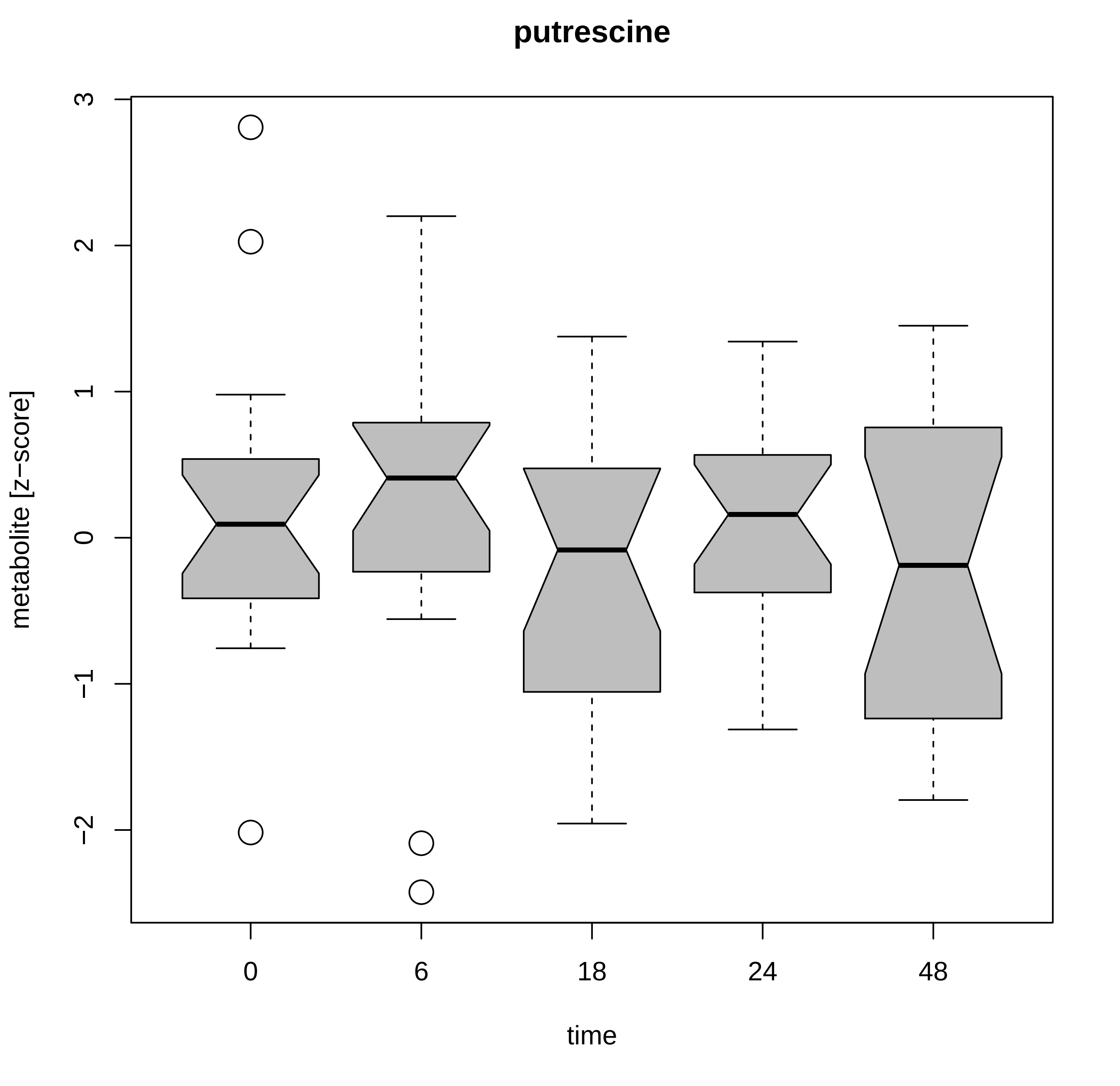

putrescine
metabolite [z−score]
3
2
1
0
−1
−2
0
6
18
24
48
time

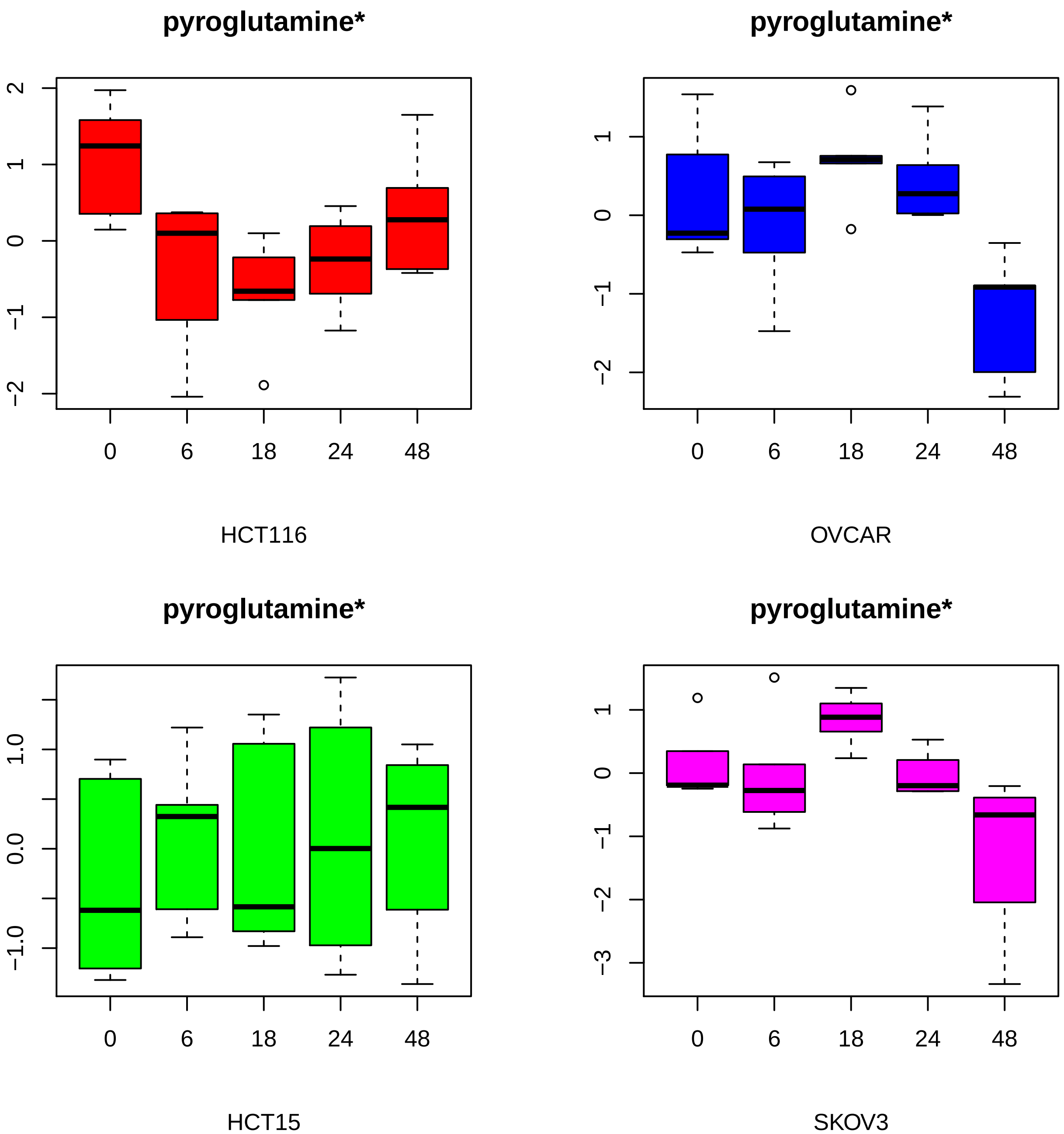
pyroglutamine*
2
1
0
−1
−2
0
6
18
24
48
HCT116
pyroglutamine*
1
0
−1
−2
0
6
18
24
48
OVCAR
pyroglutamine*
1.0
0.0
−1.0
0
6
18
24
48
HCT15
pyroglutamine*
1
0
−1
−2
−3
0
6
18
24
48
SKOV3

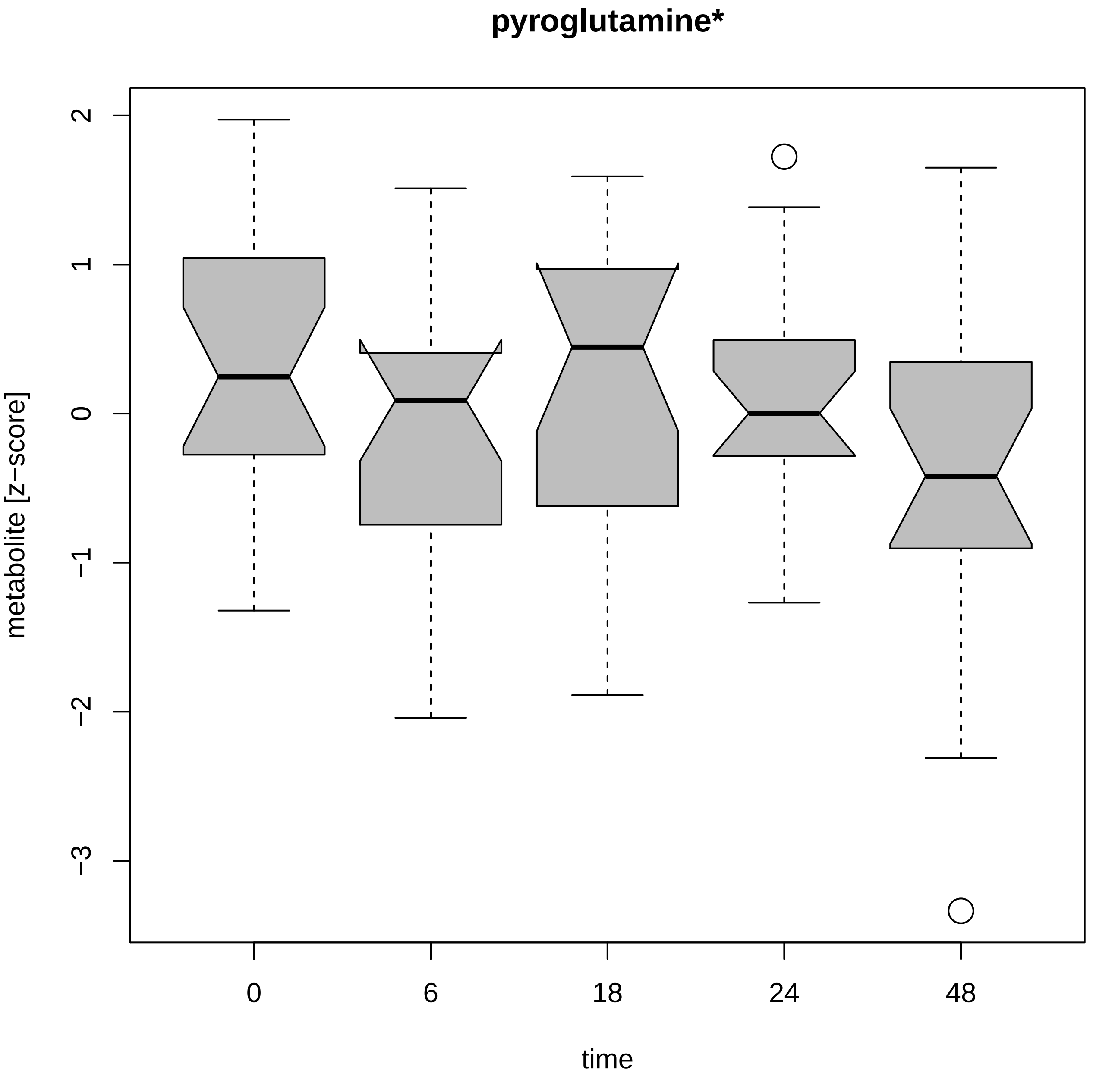
pyroglutamine*
metabolite [z−score]
2
1
0
−1
−2
−3
0
6
18
24
48
time

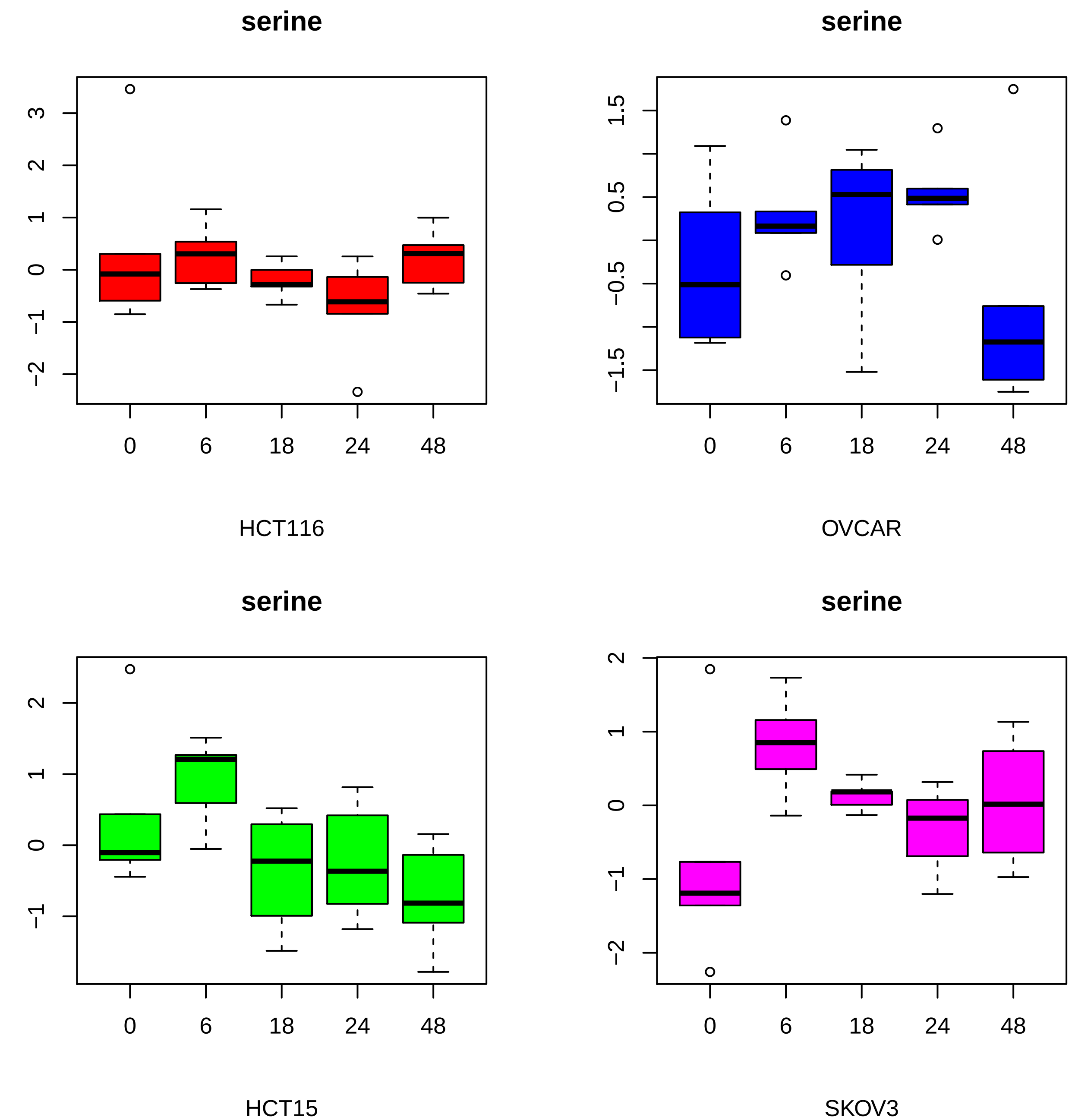

serine
HCT116
serine
OVCAR
serine
HCT15
serine
SKOV3
0
6
18
24
48

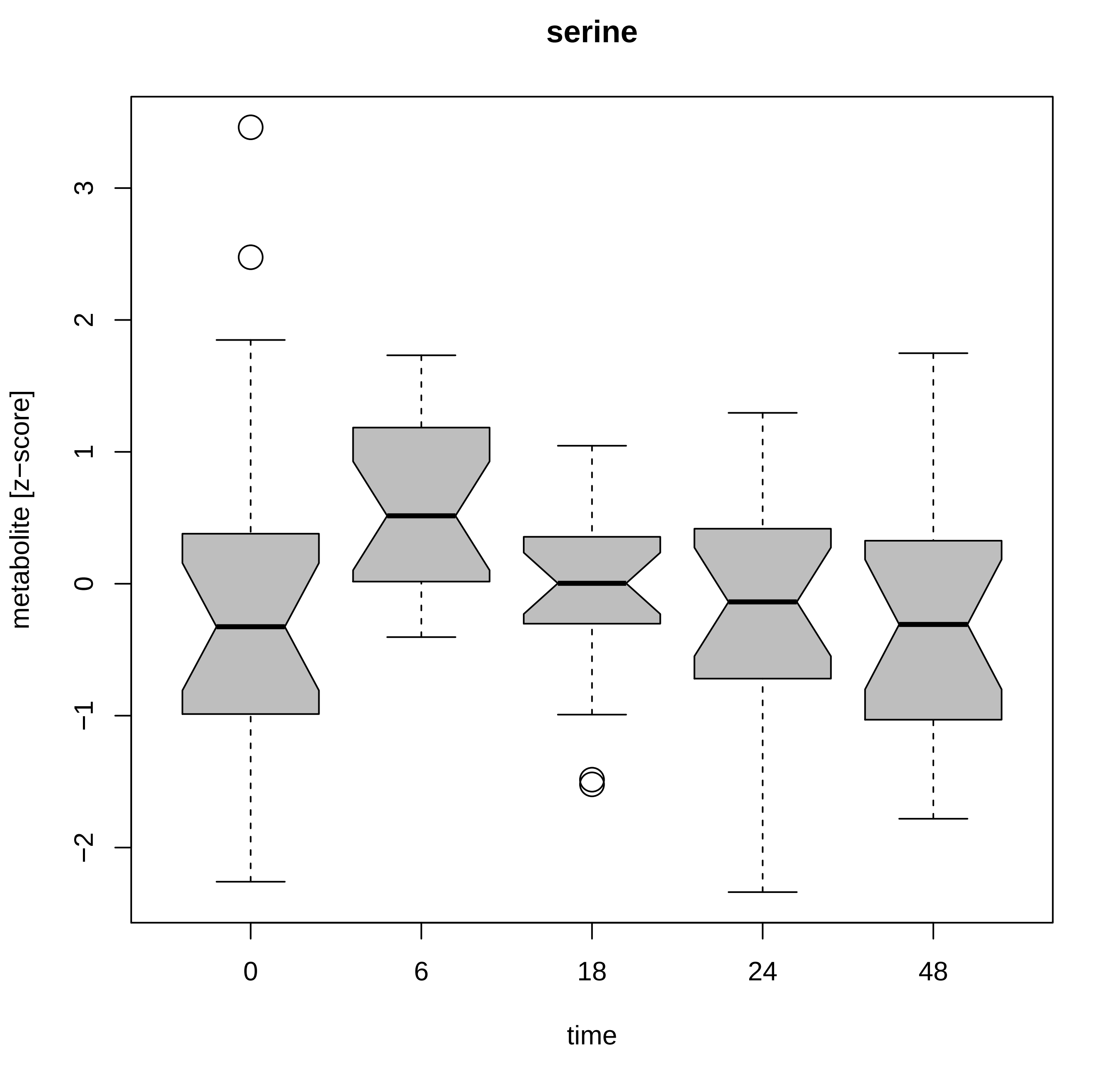
serine
metabolite [z−score]
3
2
1
0
−1
−2
0
6
18
24
48
time

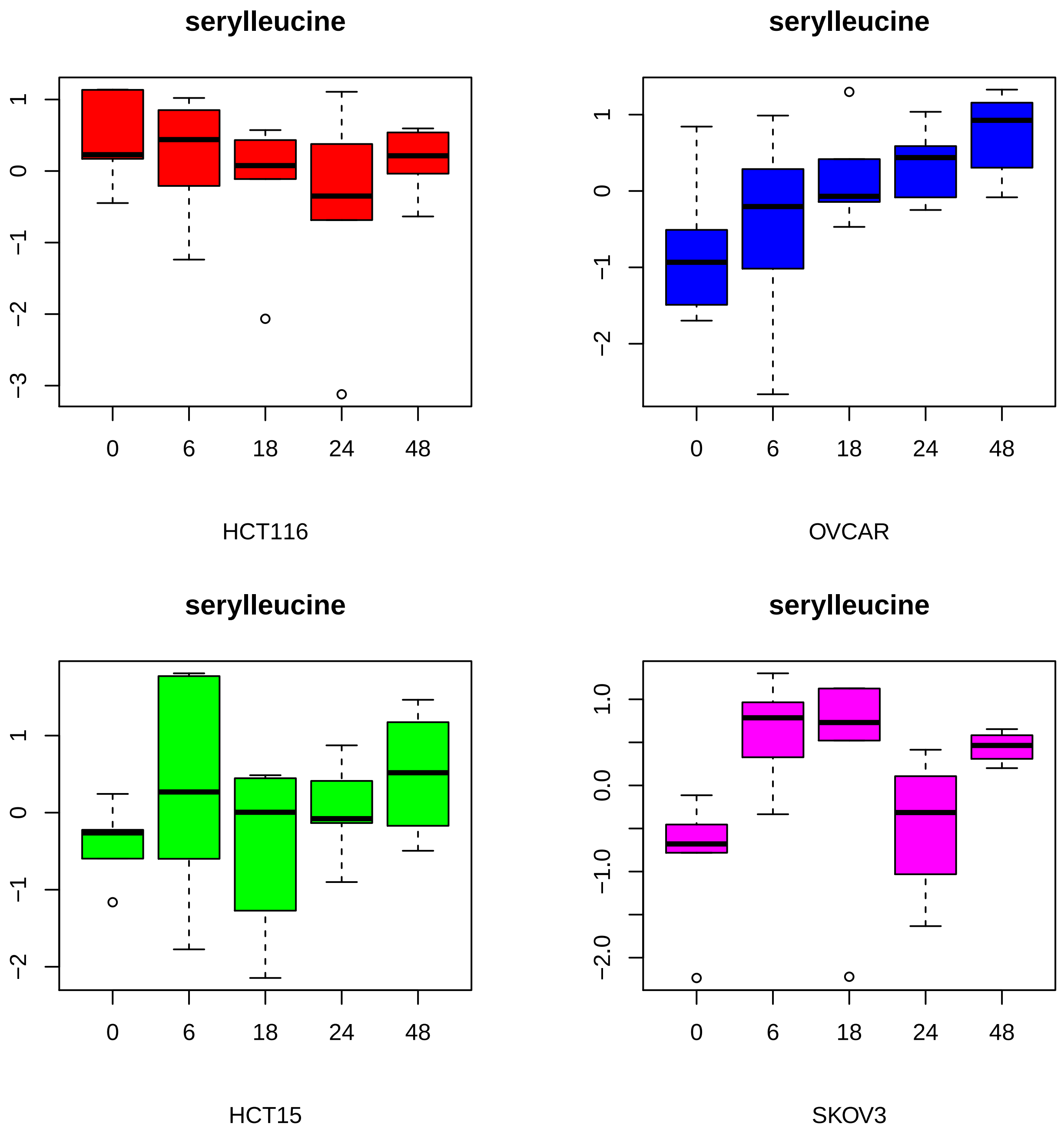
serylleucine
HCT116
serylleucine
OVCAR
serylleucine
HCT15
serylleucine
SKOV3
0
6
18
24
48

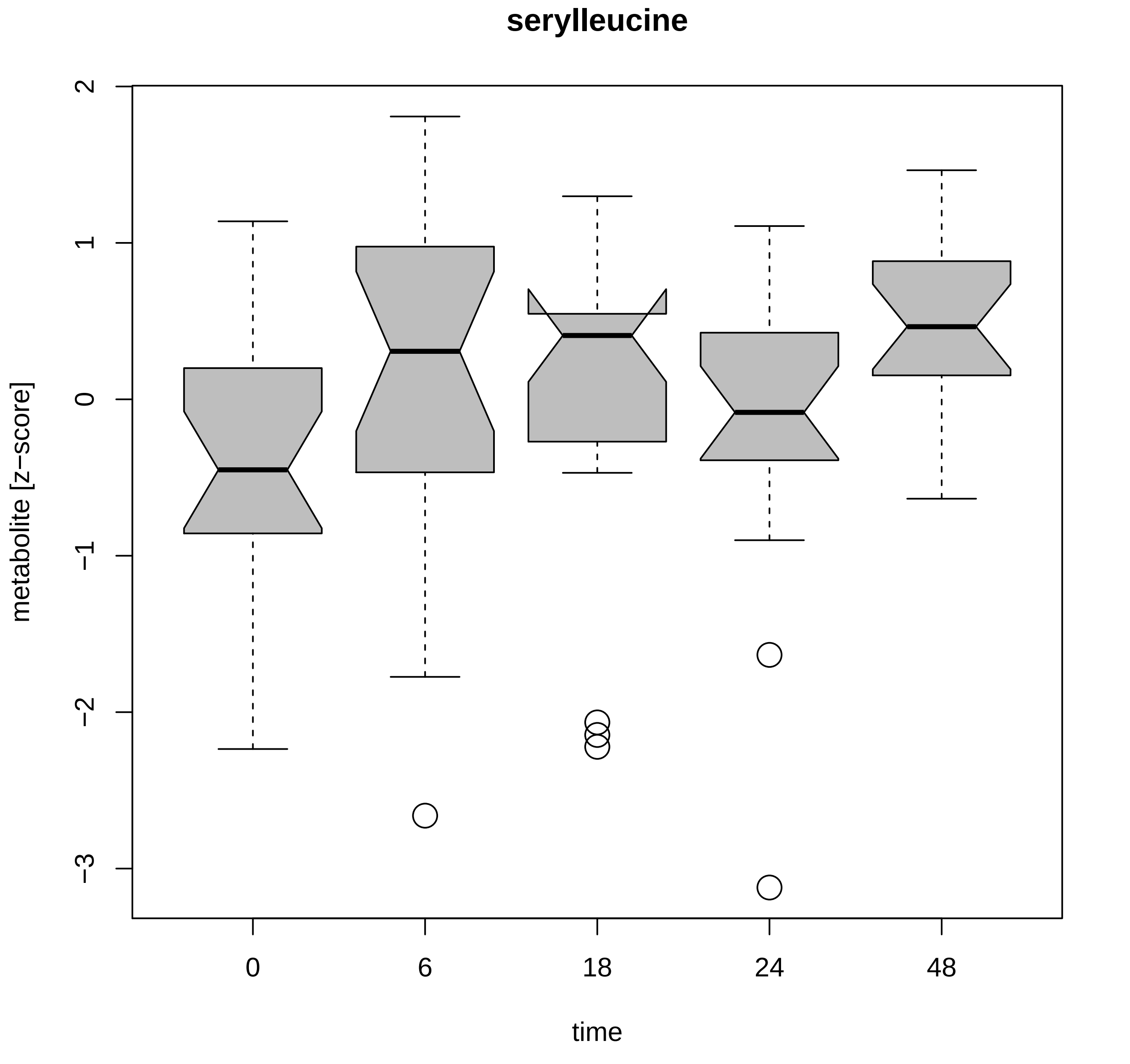

serylleucine
metabolite [z−score]
2
1
0
−1
−2
−3
0
6
18
24
48
time

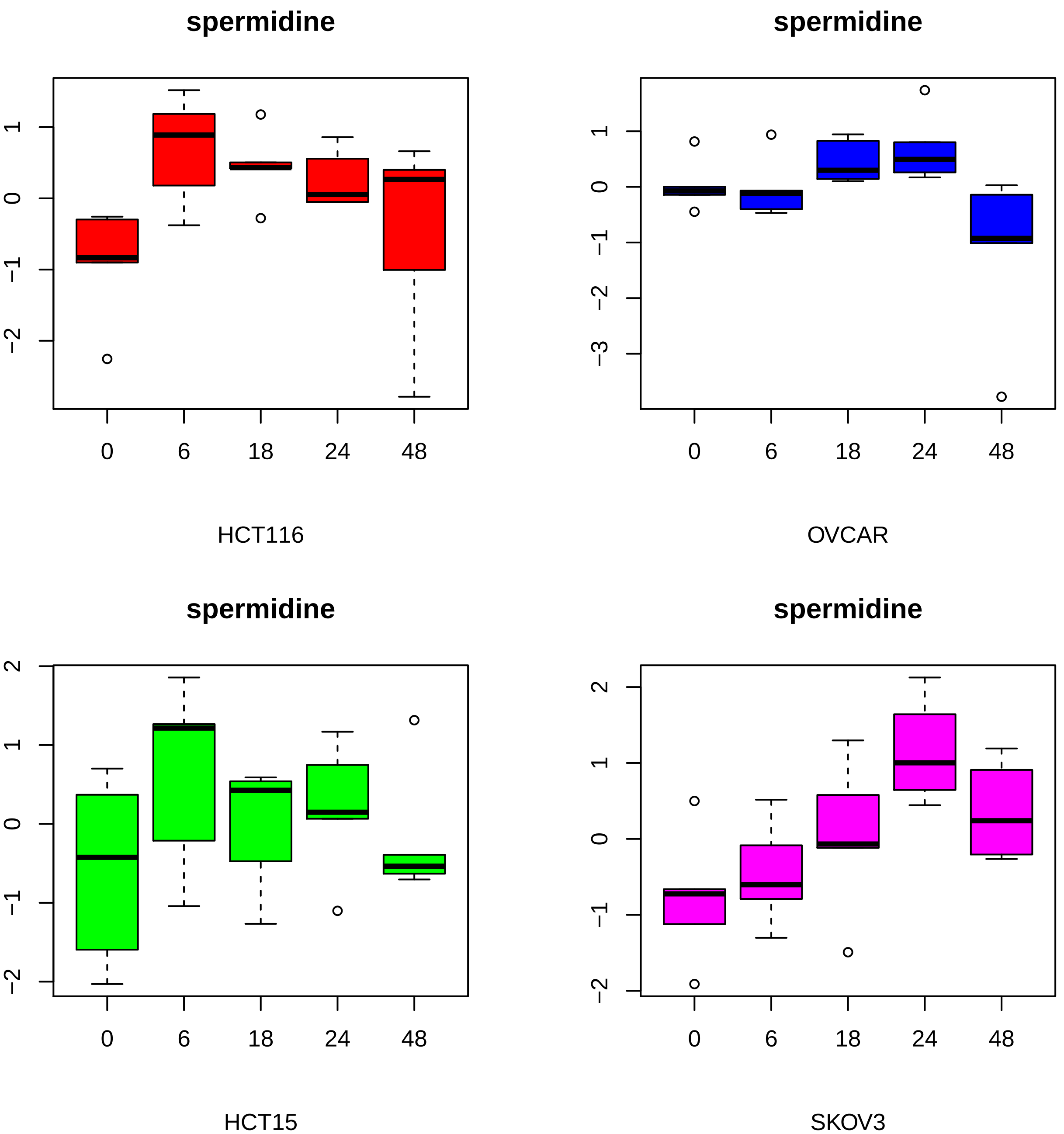

spermidine
spermidine
spermidine
spermidine
0
6
18
24
48
HCT116
OVCAR
HCT15
SKOV3

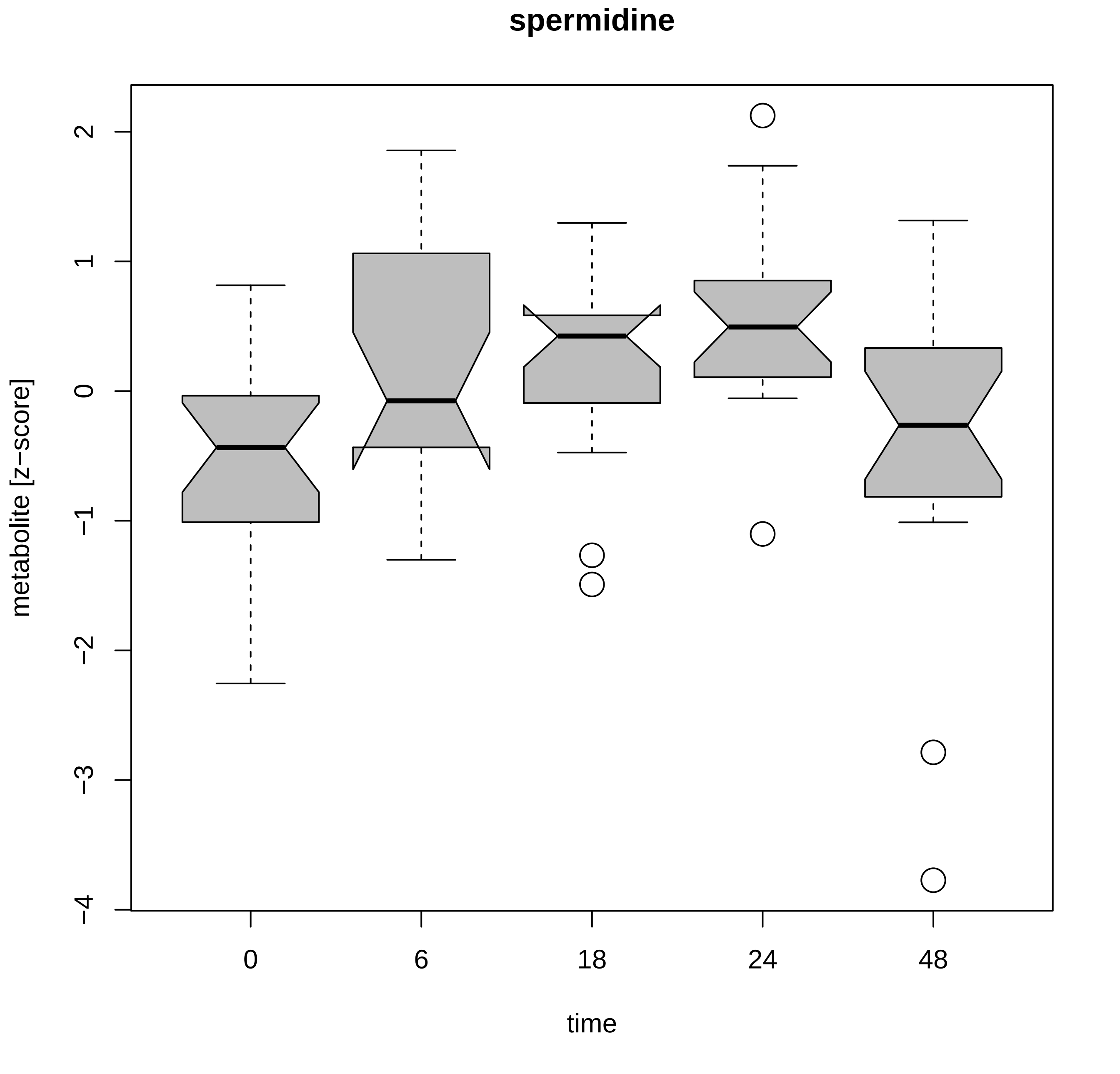
spermidine
metabolite [z−score]
2
1
0
−1
−2
−3
−4
0
6
18
24
48
time

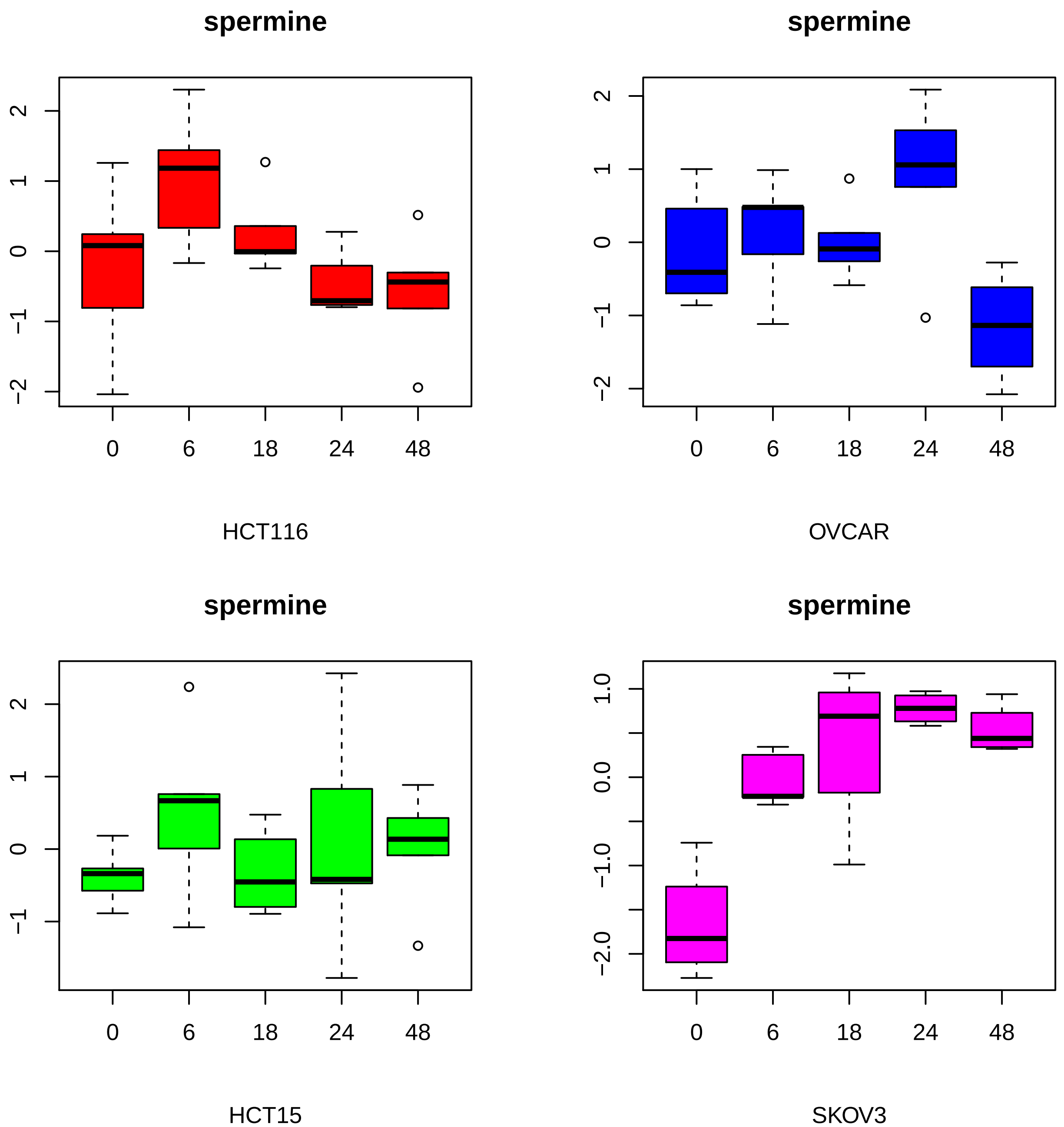
spermine
2
1
0
−1
−2
0
6
18
24
48
HCT116
spermine
2
1
0
−1
−2
0
6
18
24
48
OVCAR
spermine
2
1
0
−1
0
6
18
24
48
HCT15
spermine
1.0
0.0
−1.0
−2.0
0
6
18
24
48
SKOV3

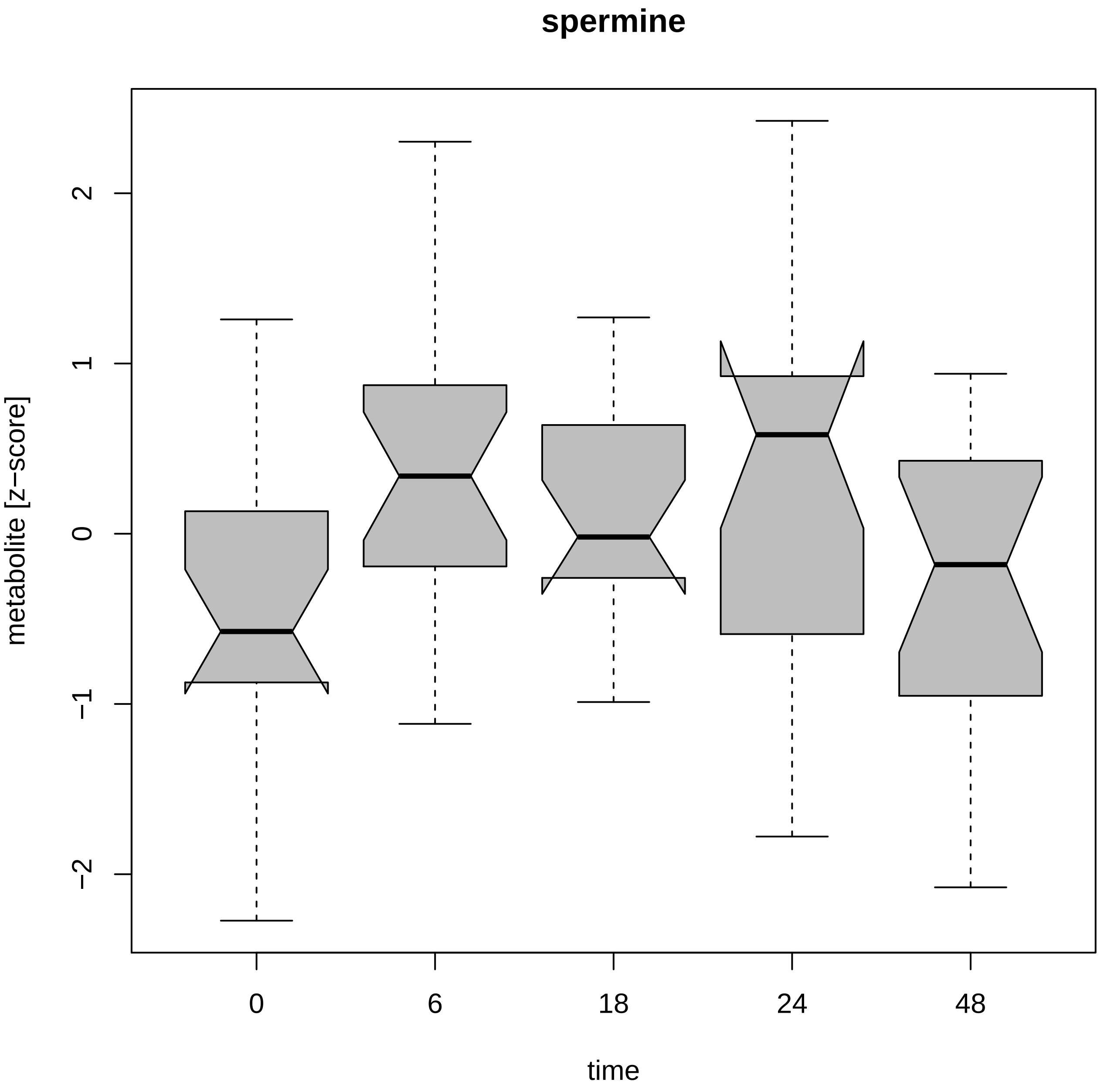

spermine
metabolite [z−score]
−2
−1
0
1
2
0
6
18
24
48
time

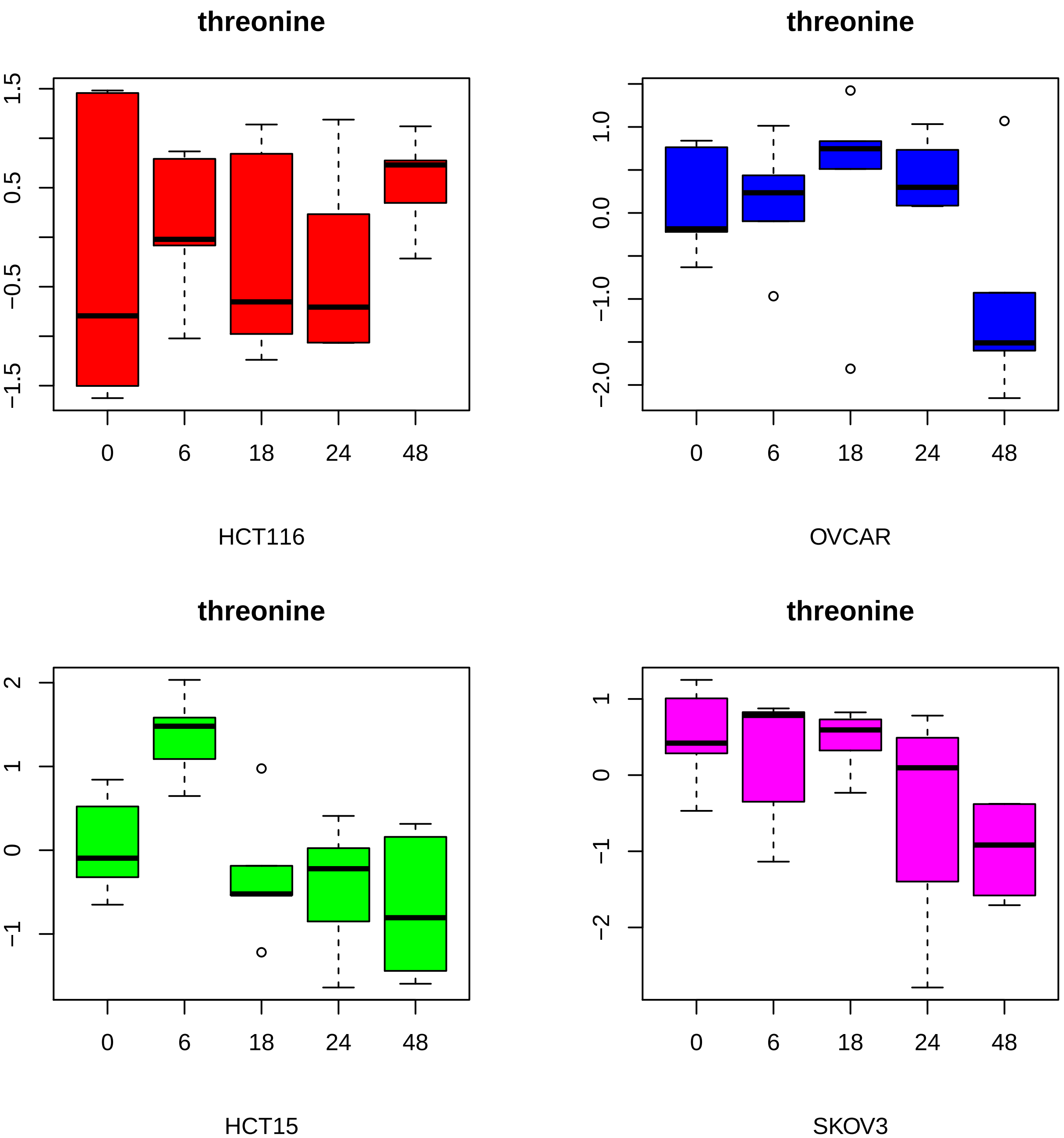
threonine
1.5
0.5
−0.5
−1.5
0
6
18
24
48
HCT116
threonine
1.0
0.0
−1.0
−2.0
0
6
18
24
48
OVCAR
threonine
2
1
0
−1
0
6
18
24
48
HCT15
threonine
1
0
−1
−2
0
6
18
24
48
SKOV3

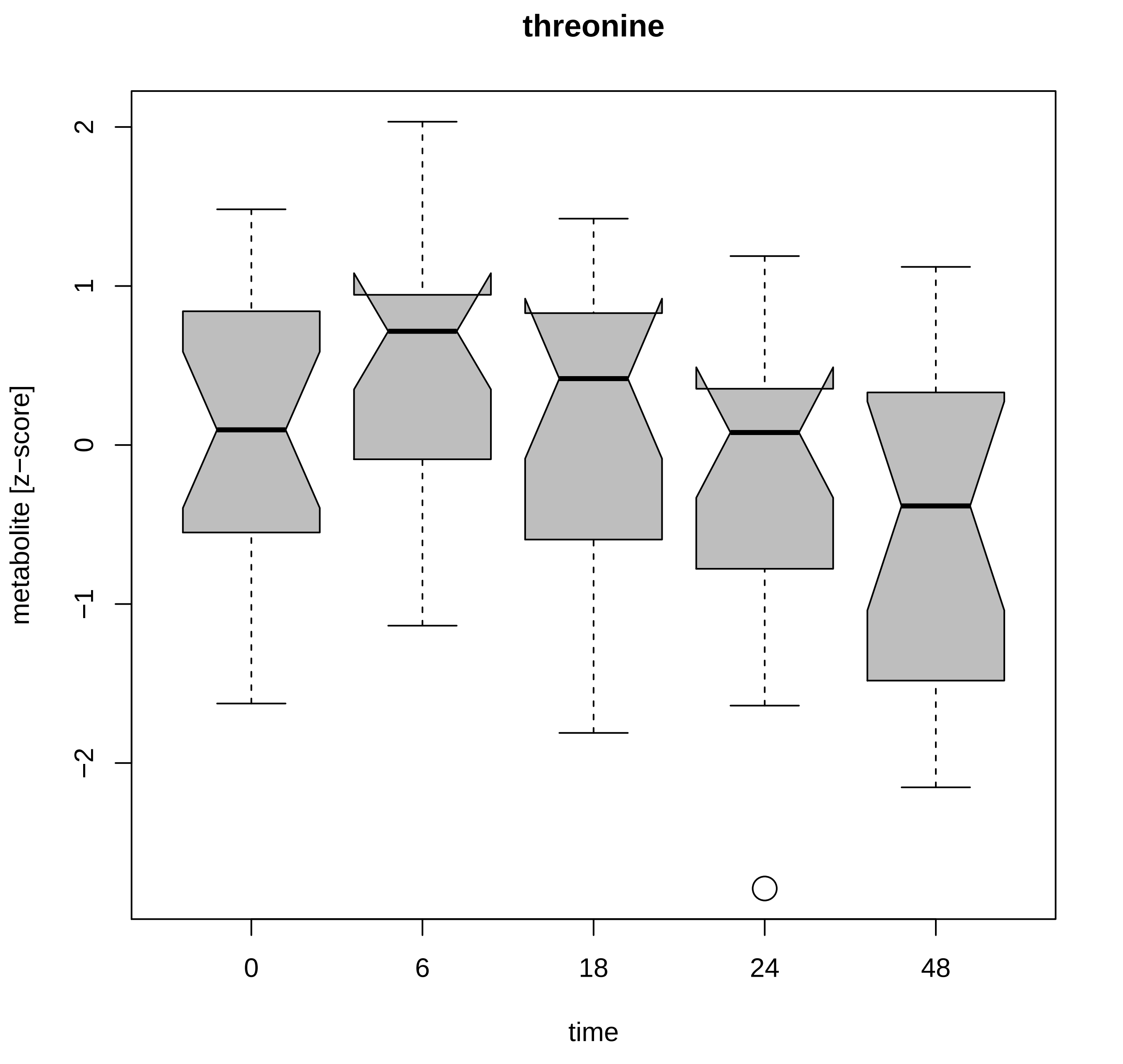

threonine
metabolite [z−score]
2
1
0
−1
−2
0
6
18
24
48
time

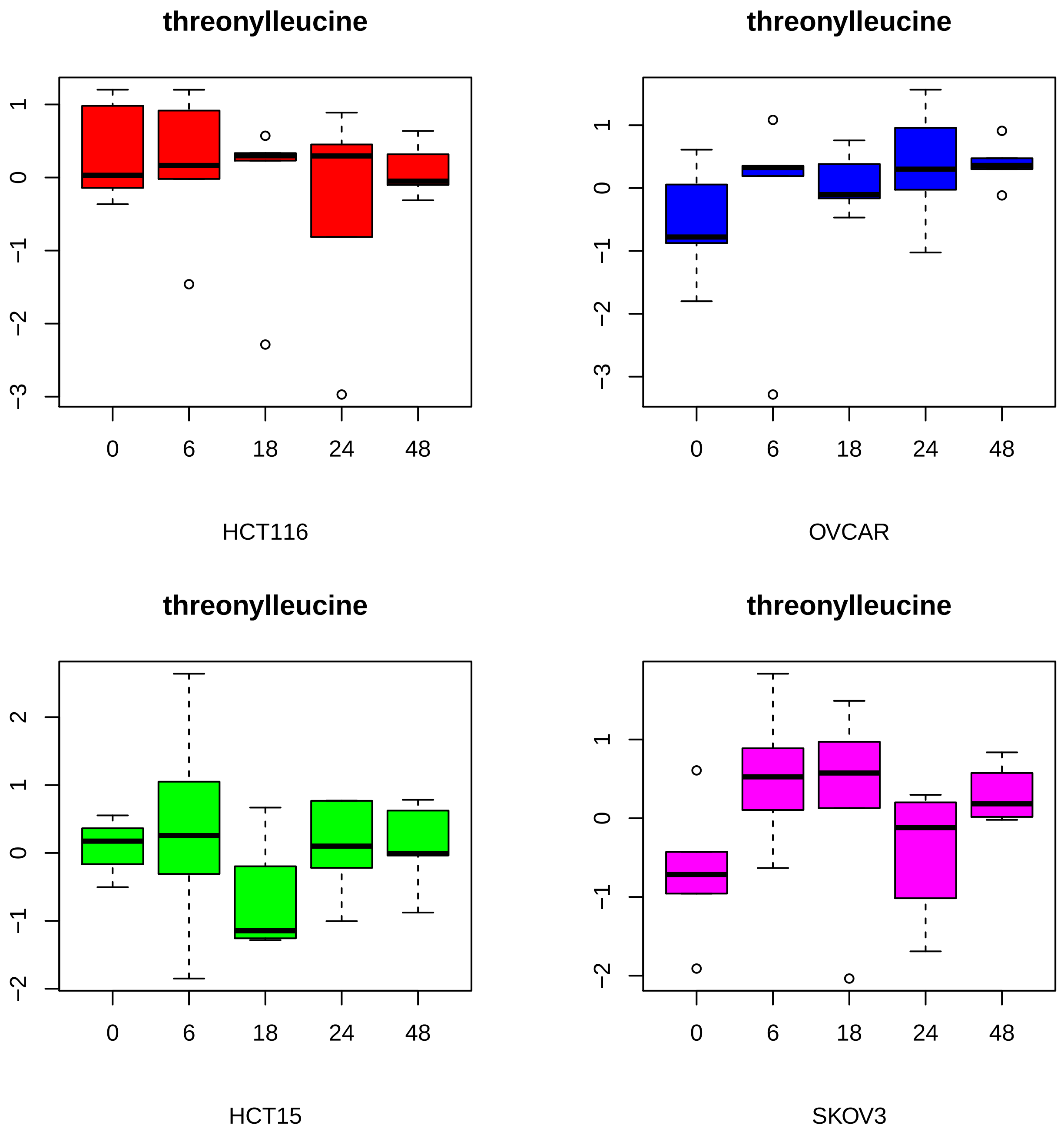
threonylleucine
HCT116
threonylleucine
OVCAR
threonylleucine
HCT15
threonylleucine
SKOV3
0
6
18
24
48
1
0
−1
−2
−3
2

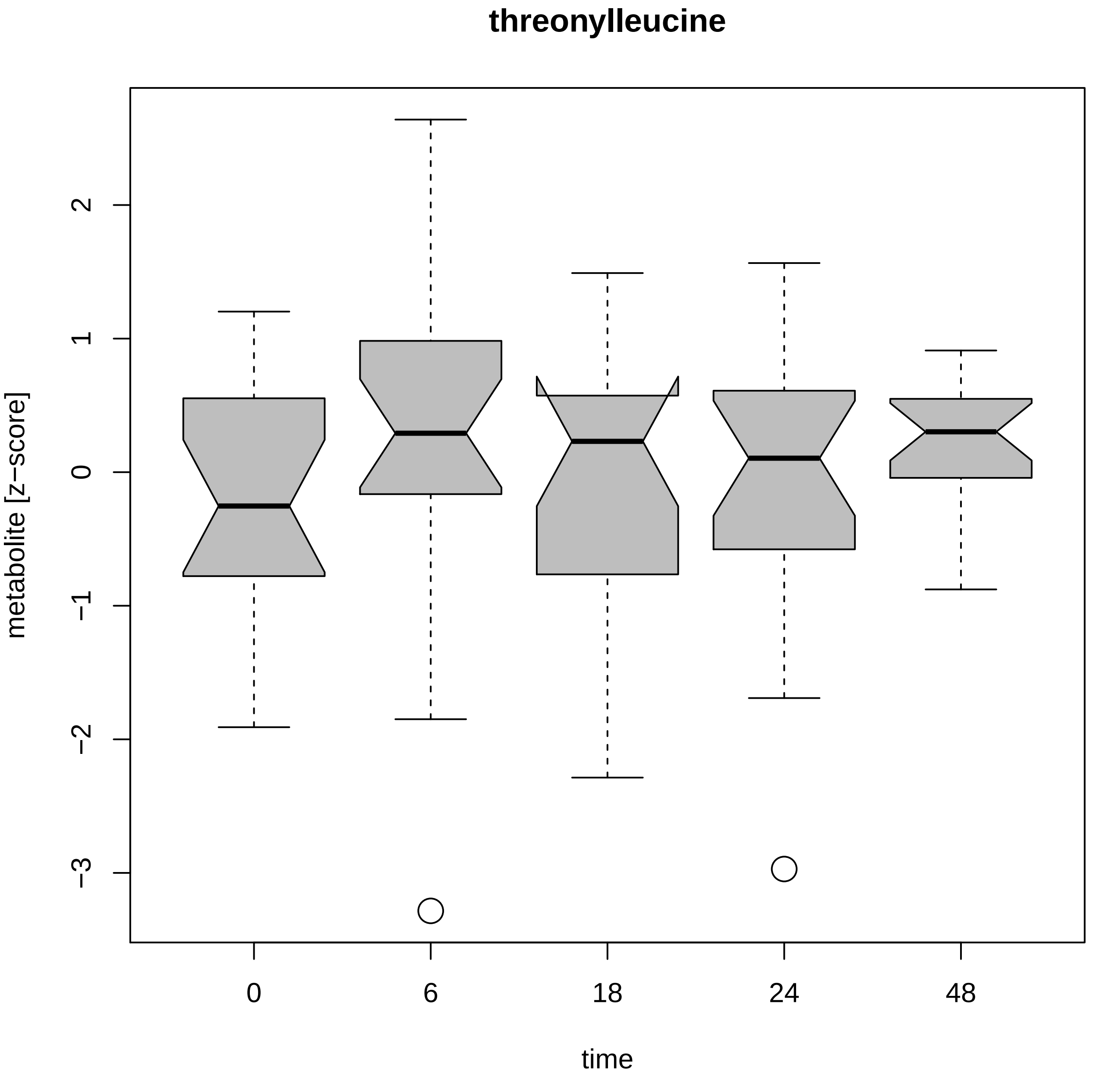
threonylleucine
metabolite [z−score]
2
1
0
−1
−2
−3
0
6
18
24
48
time

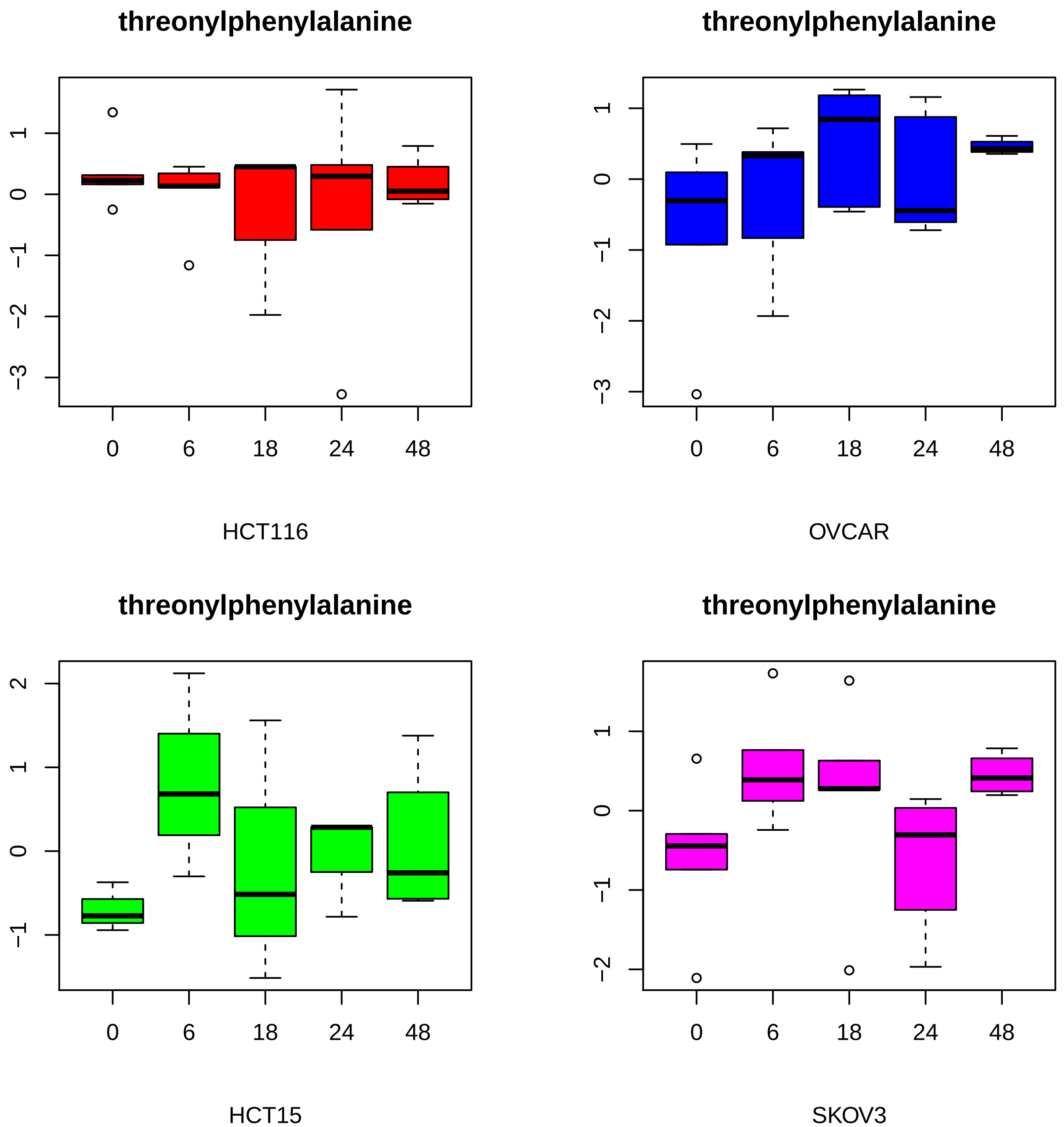
threonylphenylalanine
1
0
−1
−2
−3
0
6
18
24
48
HCT116
threonylphenylalanine
1
0
−1
−2
−3
0
6
18
24
48
OVCAR
threonylphenylalanine
2
1
0
−1
0
6
18
24
48
HCT15
threonylphenylalanine
1
0
−1
−2
0
6
18
24
48
SKOV3

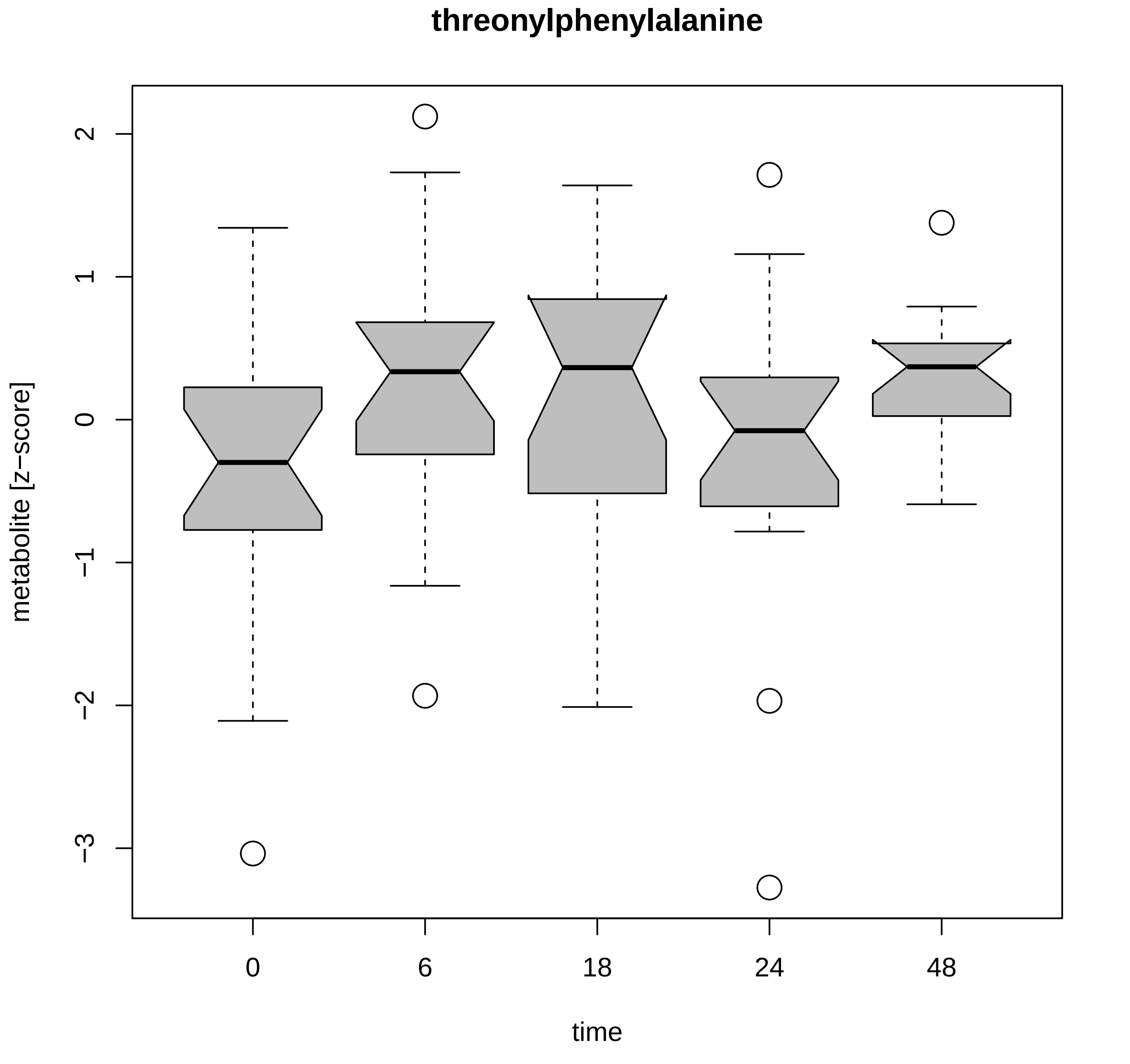

threonylphenylalanine
metabolite [z−score]
2
1
0
−1
−2
−3
0
6
18
24
48
time

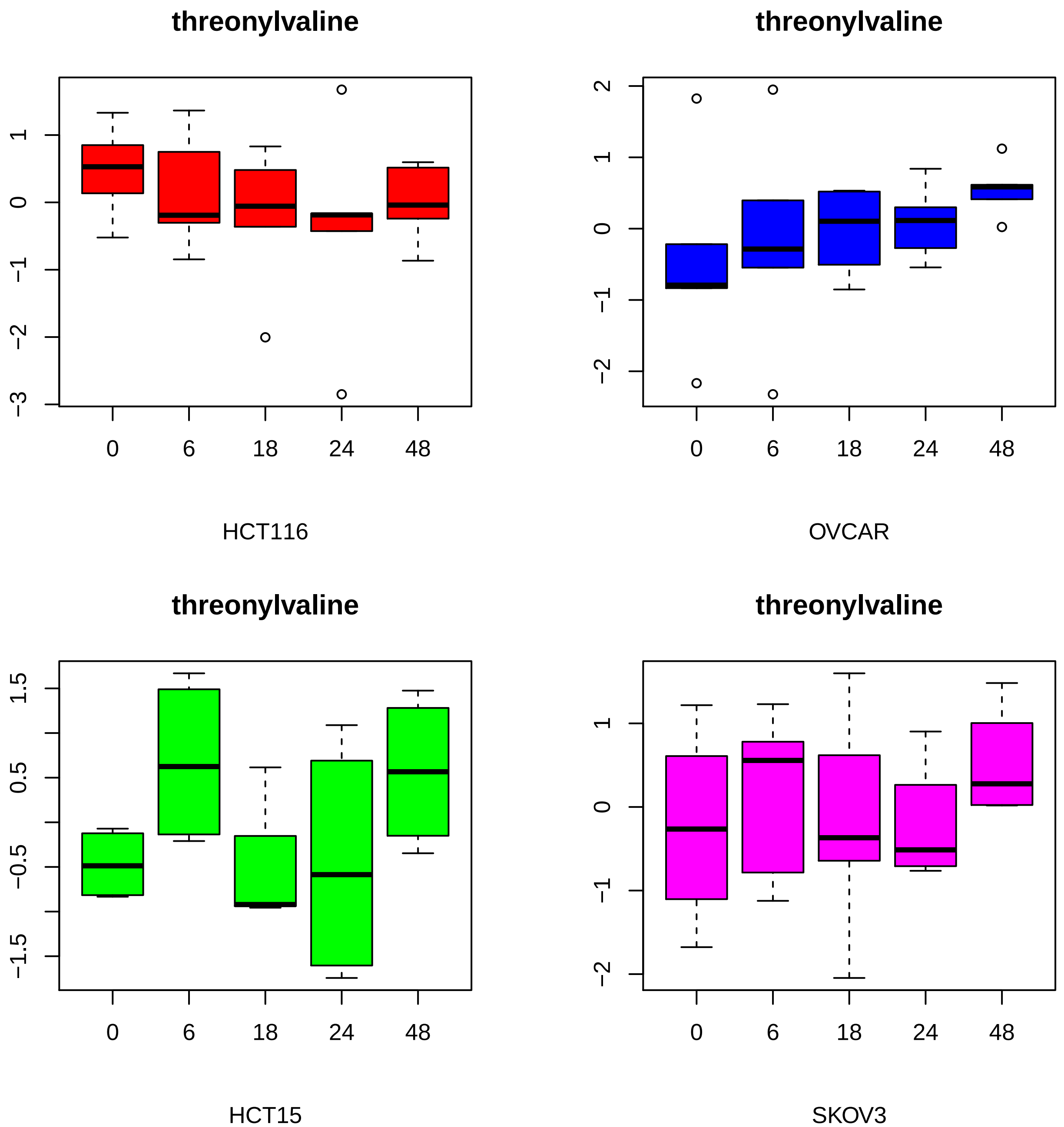
threonylvaline
HCT116
threonylvaline
OVCAR
threonylvaline
HCT15
threonylvaline
SKOV3
0
6
18
24
48

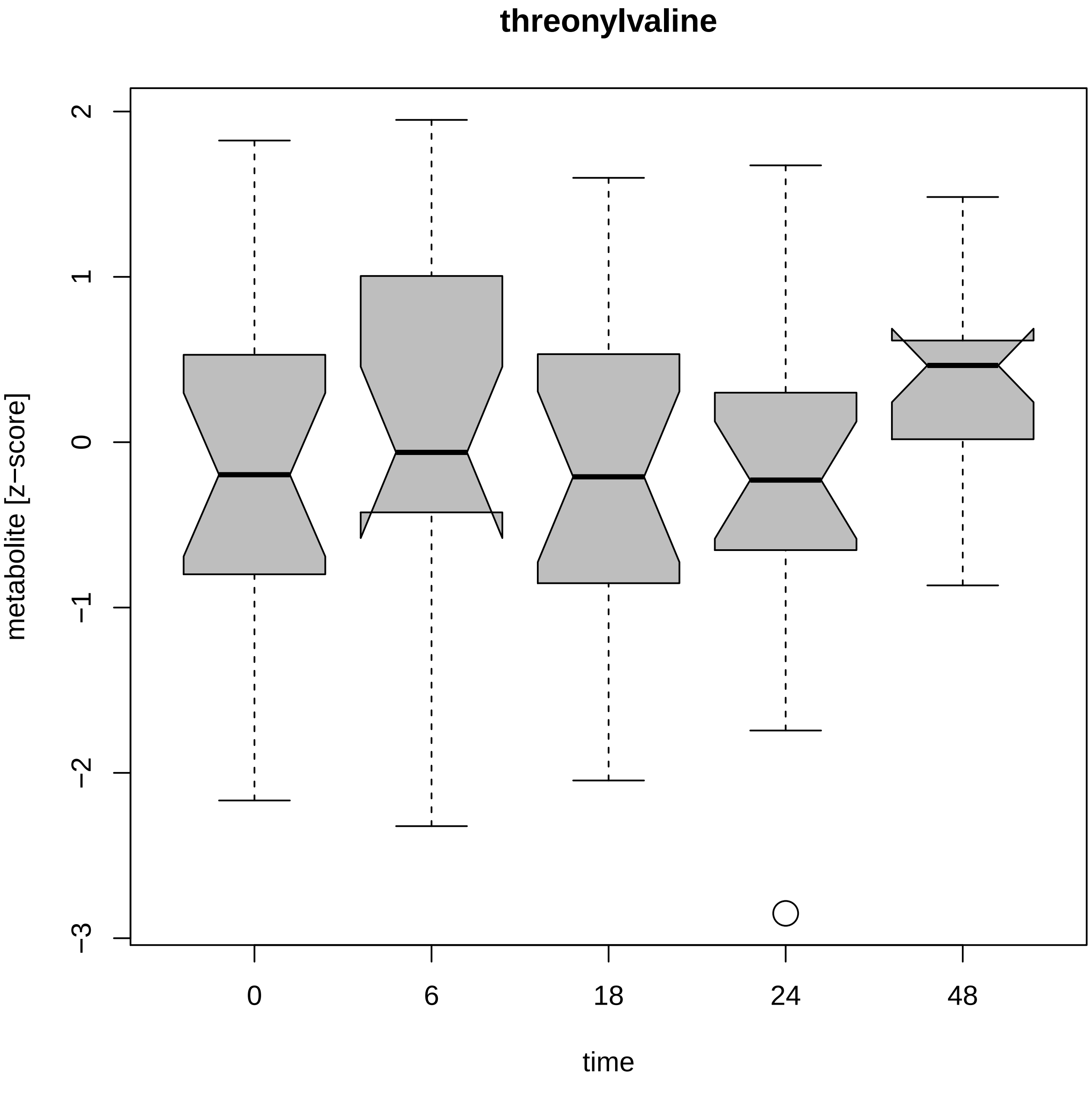
threonylvaline
metabolite [z−score]
2
1
0
−1
−2
−3
0
6
18
24
48
time

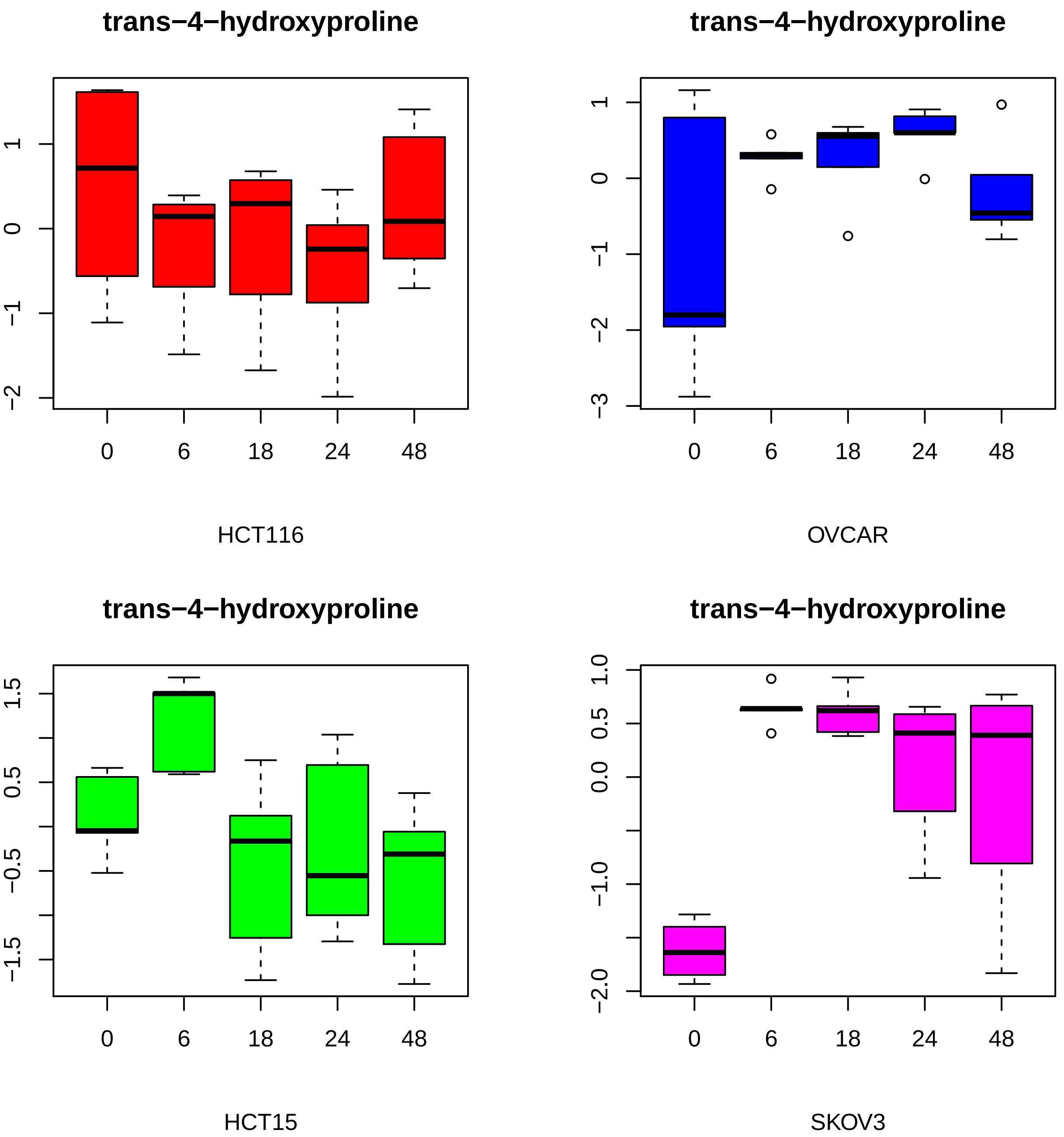
trans−4−hydroxyproline
HCT116
0
6
18
24
48
1
0
−1
−2
trans−4−hydroxyproline
OVCAR
1
0
−1
−2
−3
trans−4−hydroxyproline
HCT15
1.5
0.5
−0.5
−1.5
trans−4−hydroxyproline
SKOV3
1.0
0.5
0.0
−1.0
−2.0

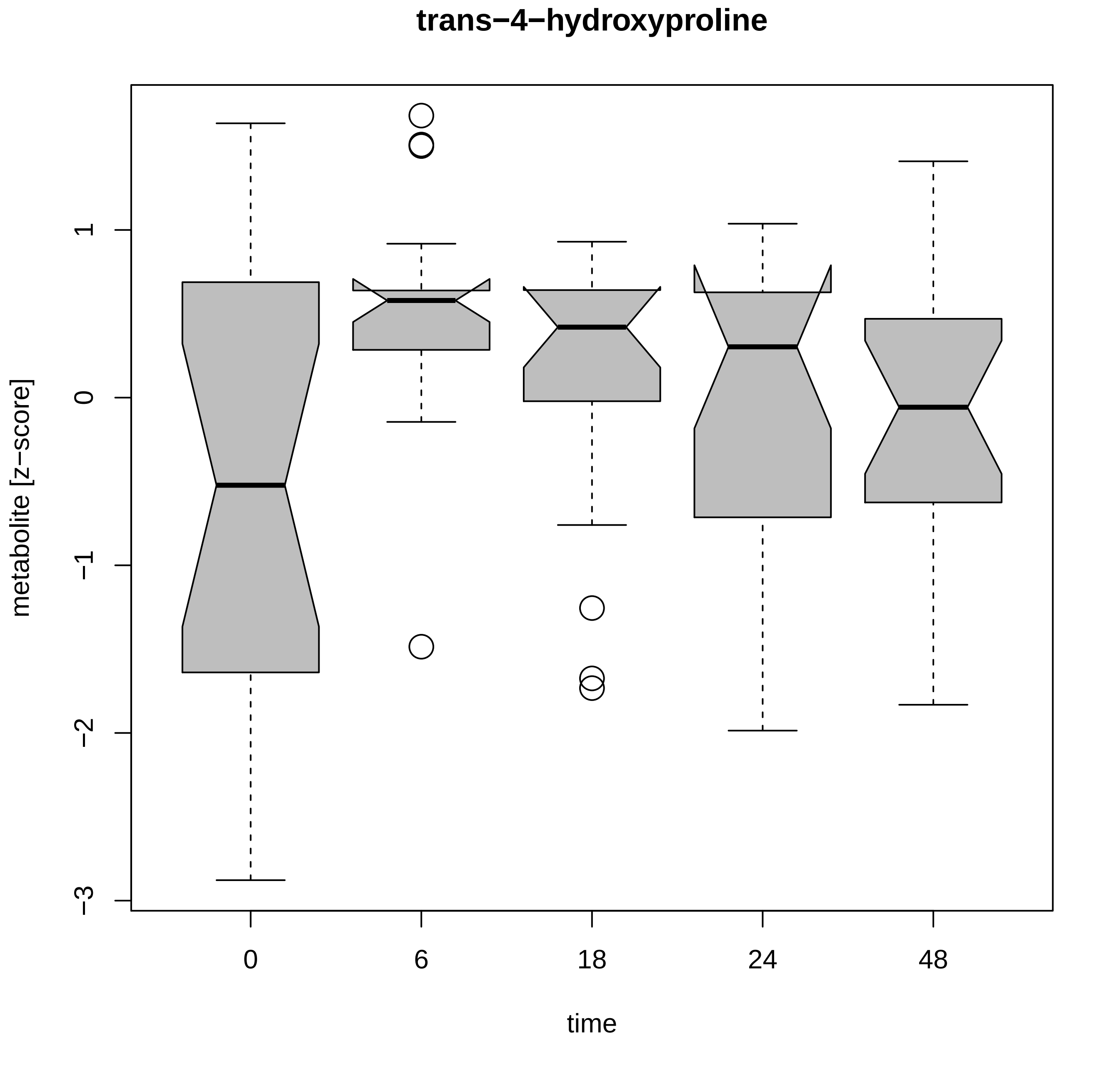

trans−4−hydroxyproline
metabolite [z−score]
1
0
−1
−2
−3
0
6
18
24
48
time

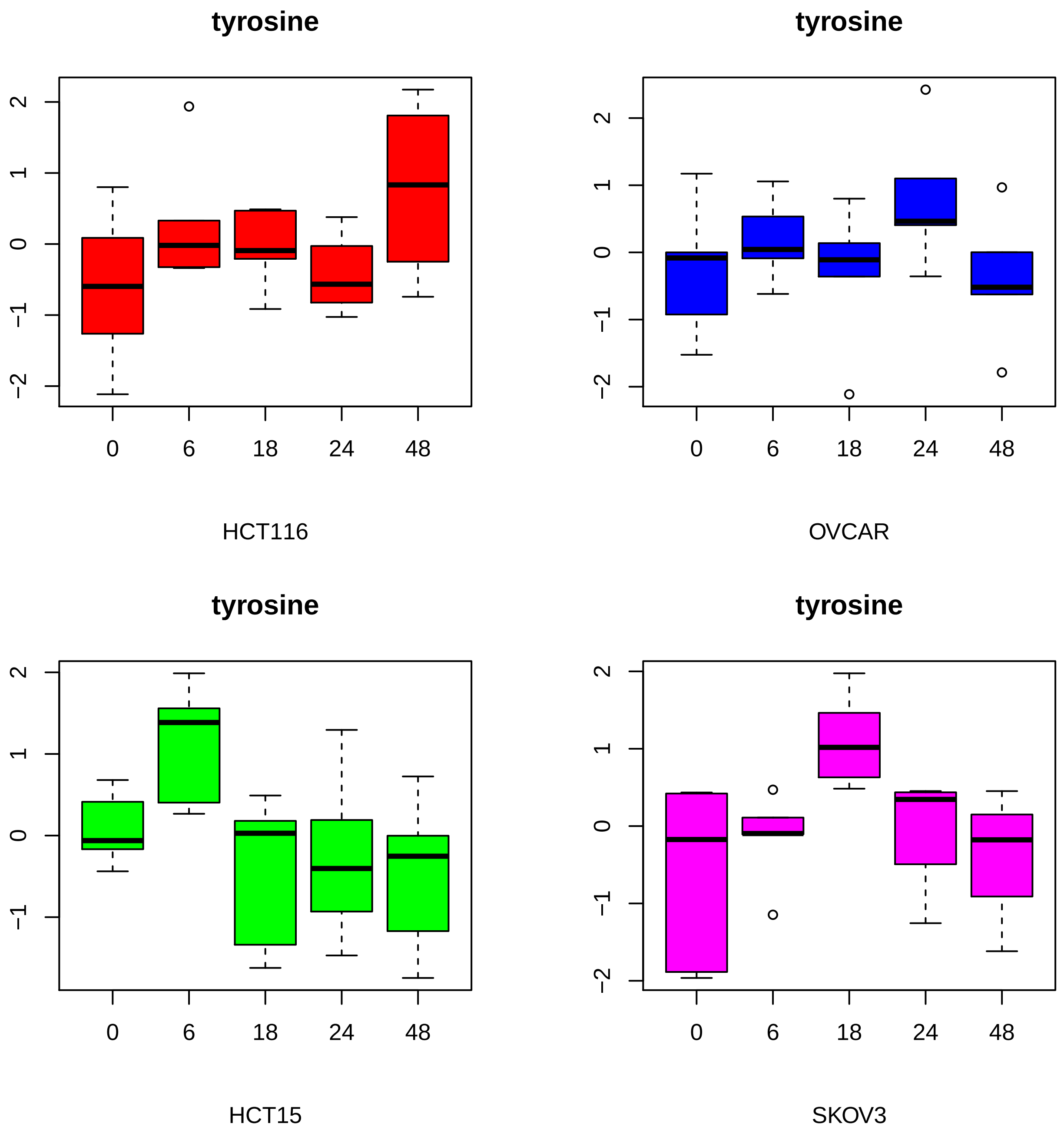
tyrosine
HCT116
tyrosine
OVCAR
tyrosine
HCT15
tyrosine
SKOV3
0
6
18
24
48

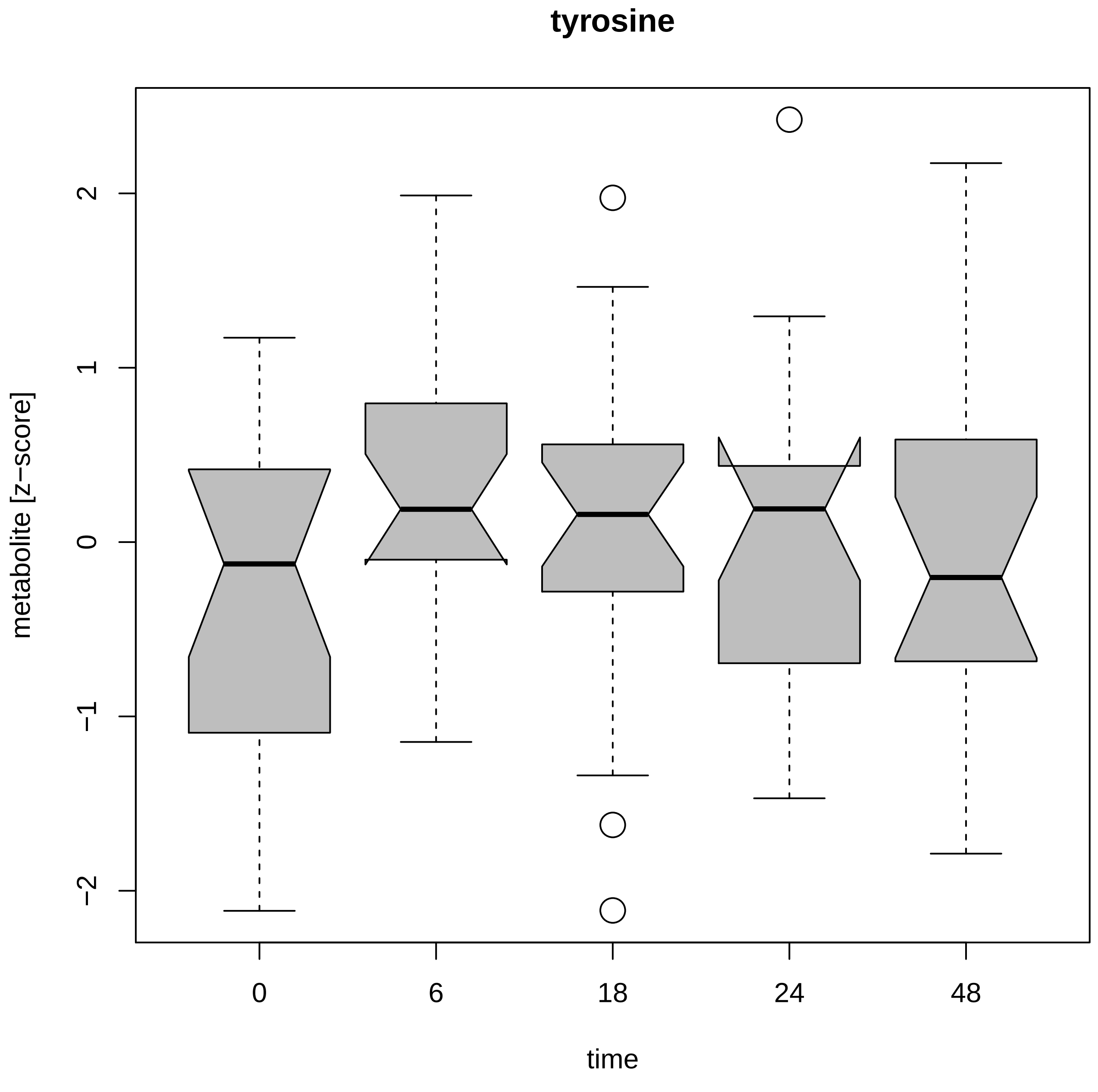

tyrosine
metabolite [z−score]
2
1
0
−1
−2
0
6
18
24
48
time

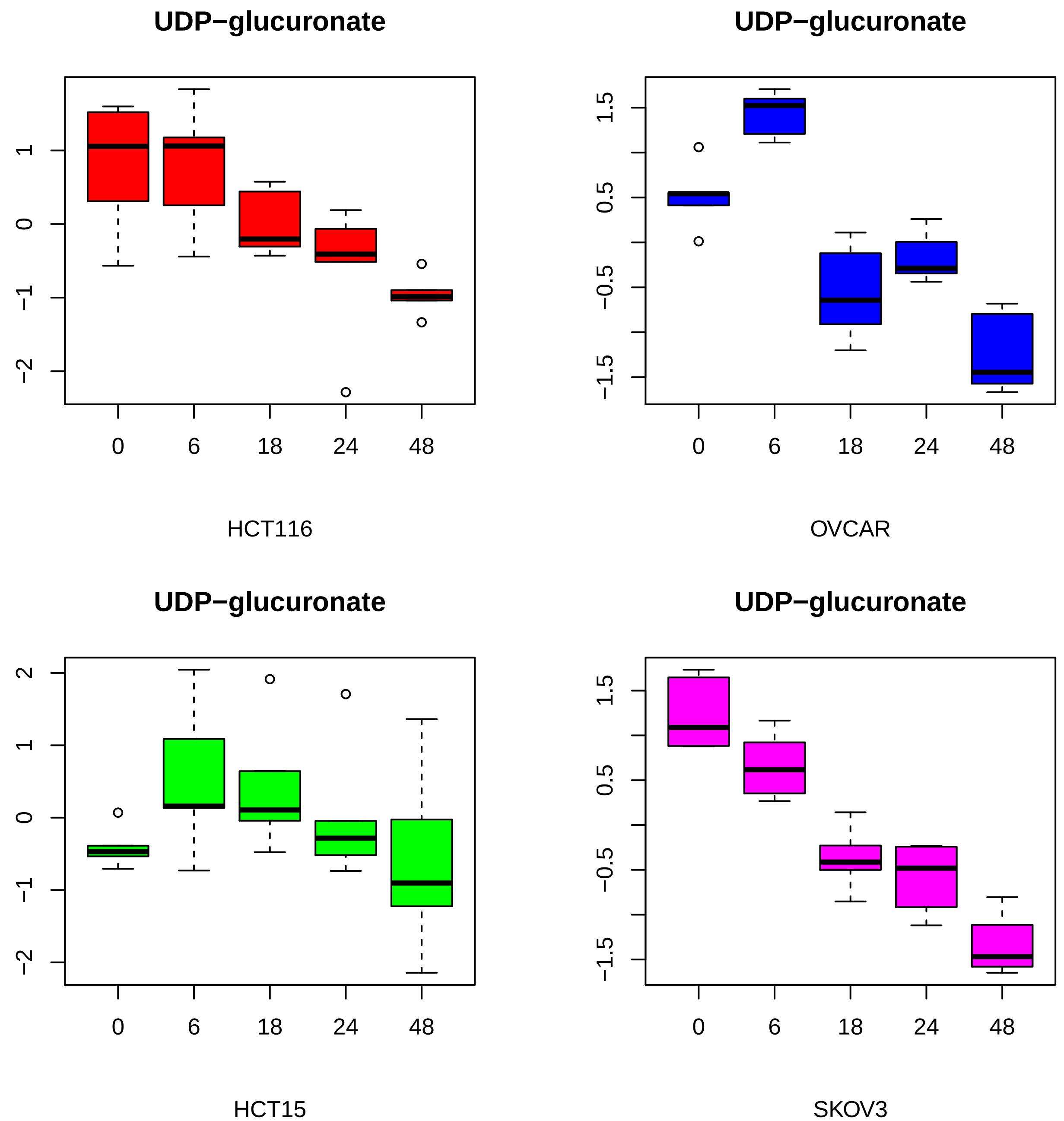
UDP−glucuronate
UDP−glucuronate
UDP−glucuronate
UDP−glucuronate
1
0
−1
−2
0
6
18
24
48
HCT116
1.5
0.5
−0.5
−1.5
OVCAR
2
HCT15
SKOV3

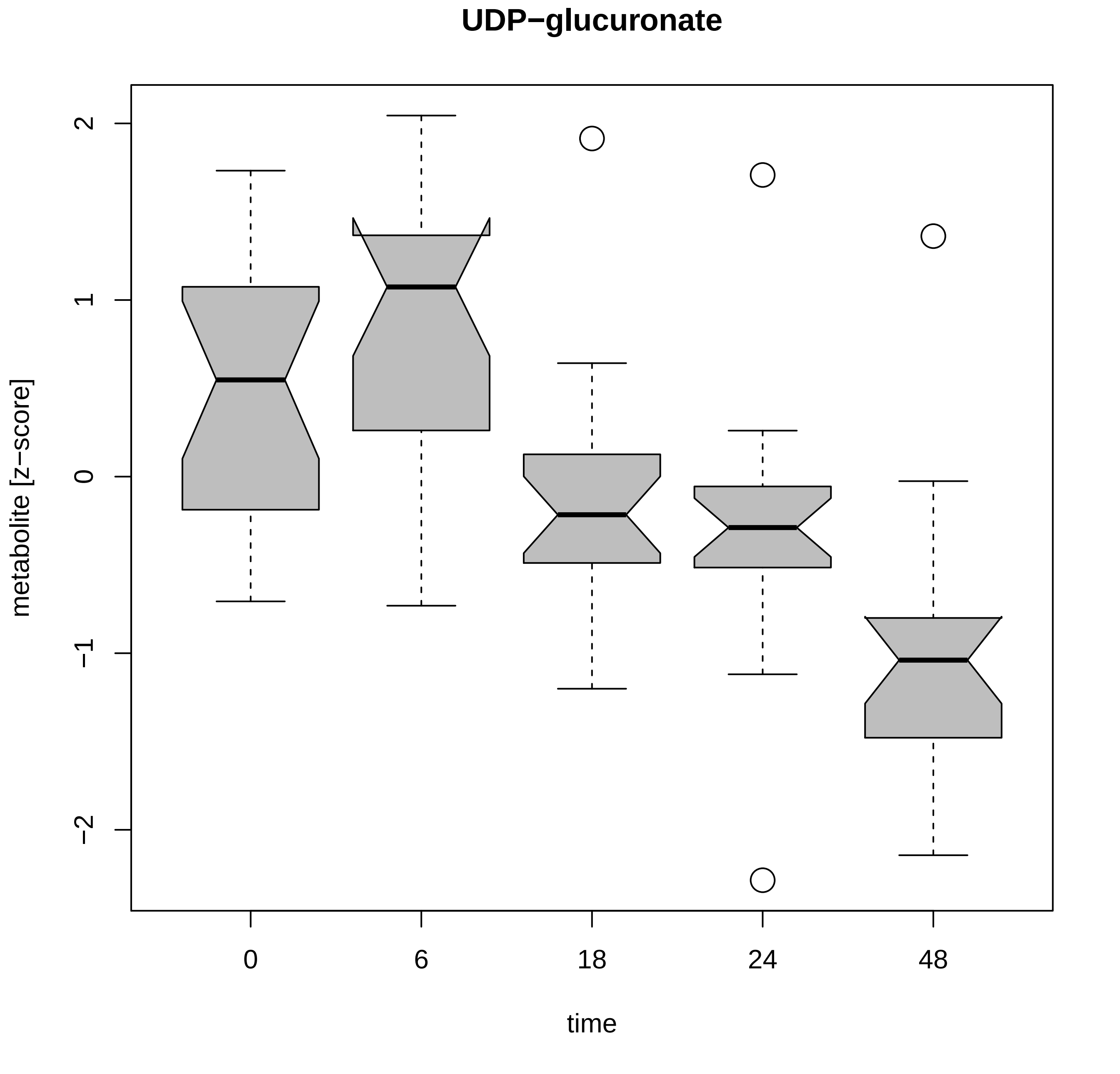

UDP−glucuronate
metabolite [z−score]
2
1
0
−1
−2
0
6
18
24
48
time

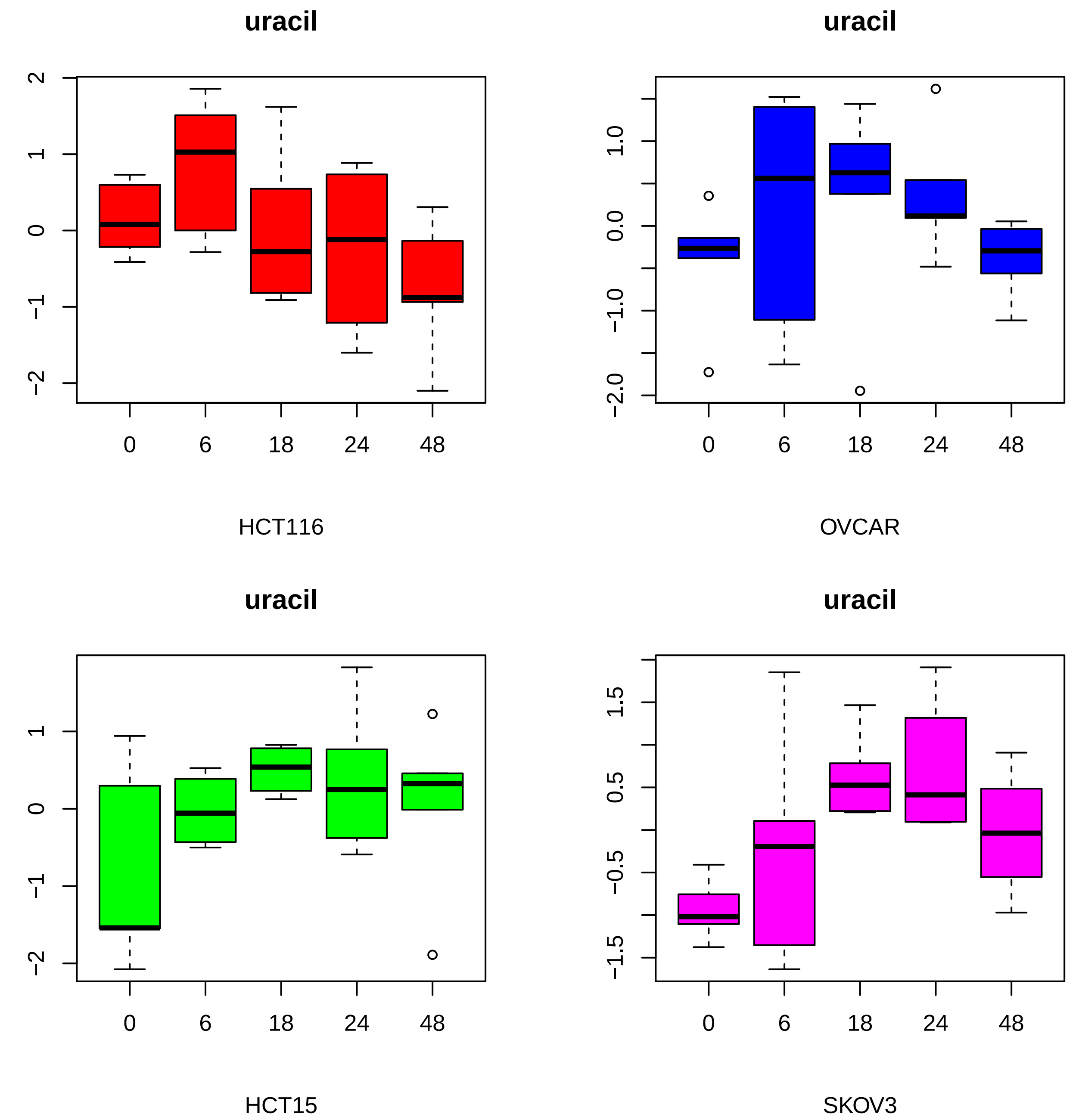
uracil
2
1
0
−1
−2
0
6
18
24
48
HCT116
uracil
1.0
0.0
−1.0
−2.0
0
6
18
24
48
OVCAR
uracil
1
0
−1
−2
0
6
18
24
48
HCT15
uracil
1.5
0.5
−0.5
−1.5
0
6
18
24
48
SKOV3

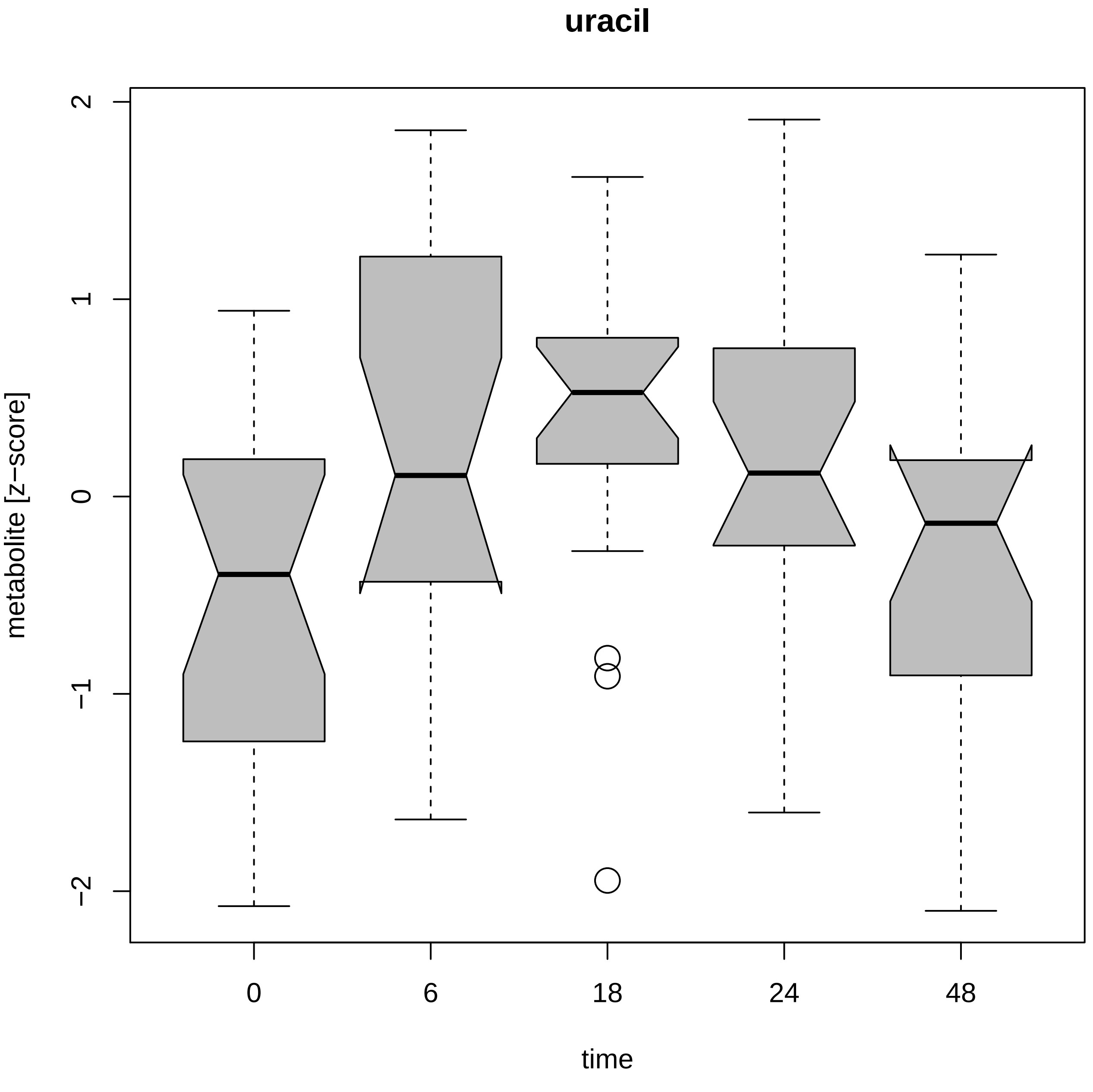

uracil
metabolite [z−score]
2
1
0
−1
−2
0
6
18
24
48
time

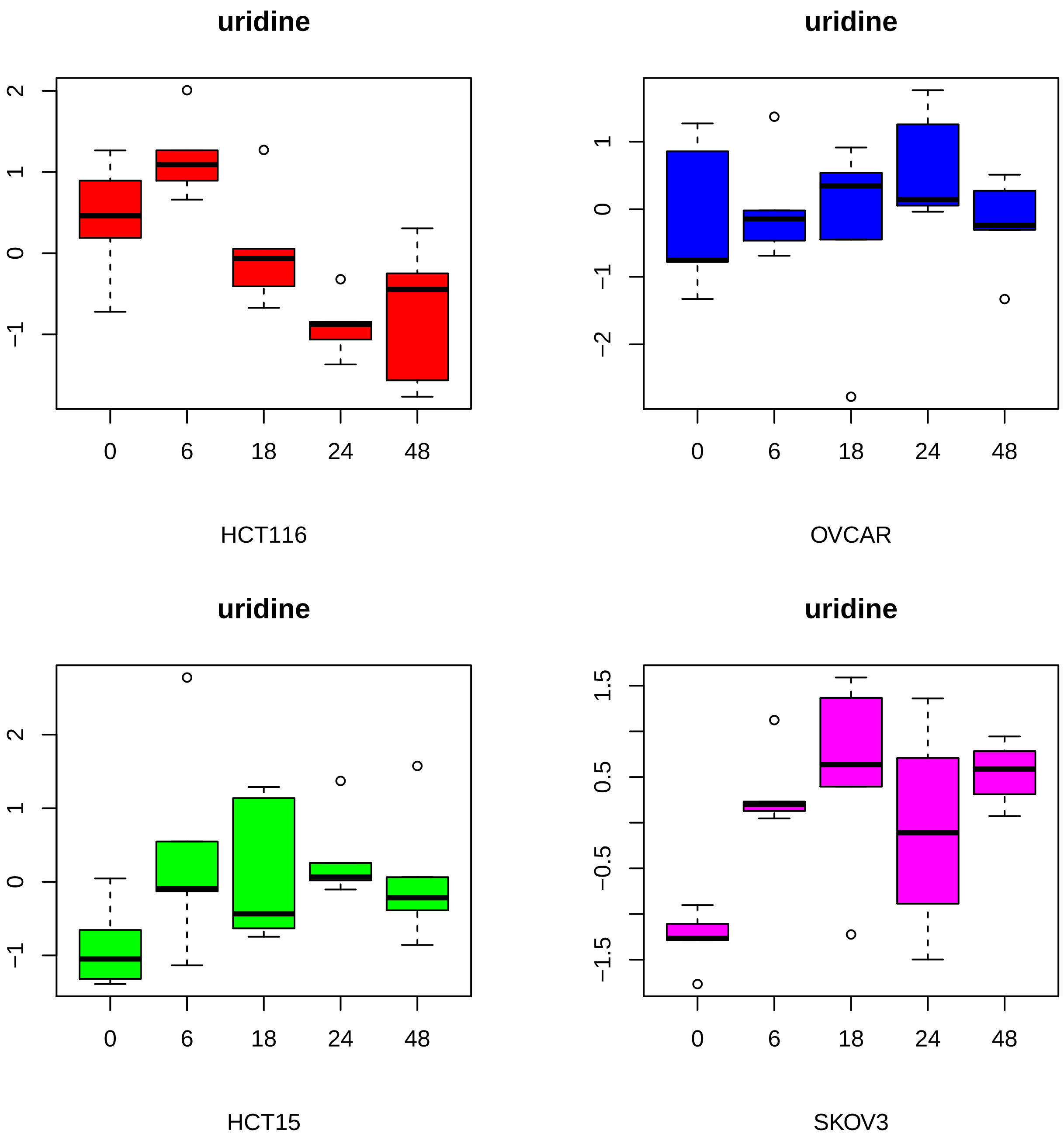

uridine
2
1
0
−1
0
6
18
24
48
HCT116
uridine
1
0
−1
−2
0
6
18
24
48
OVCAR
uridine
2
1
0
−1
0
6
18
24
48
HCT15
uridine
1.5
0.5
−0.5
−1.5
0
6
18
24
48
SKOV3

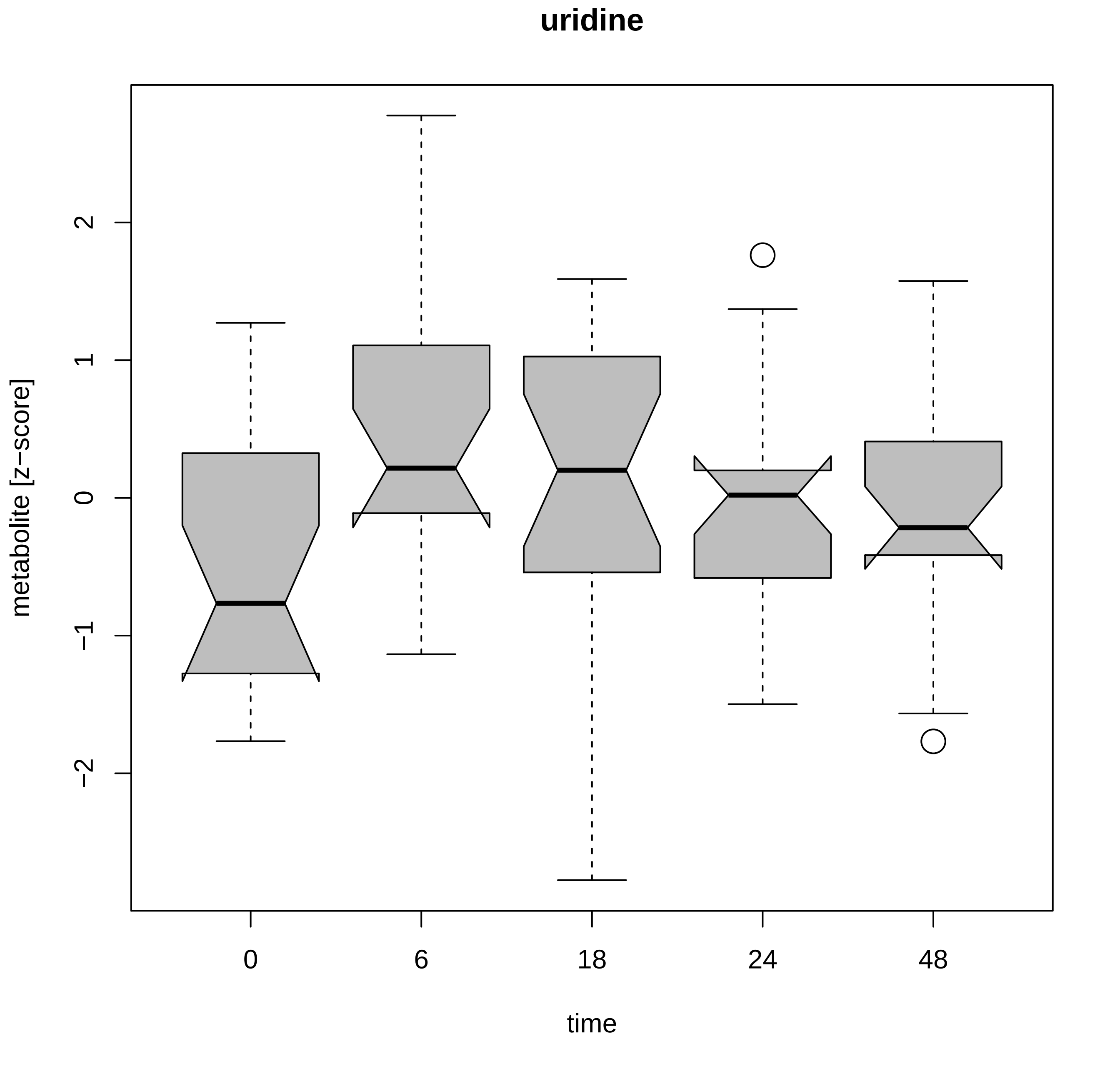
uridine
metabolite [z−score]
2
1
0
−1
−2
0
6
18
24
48
time

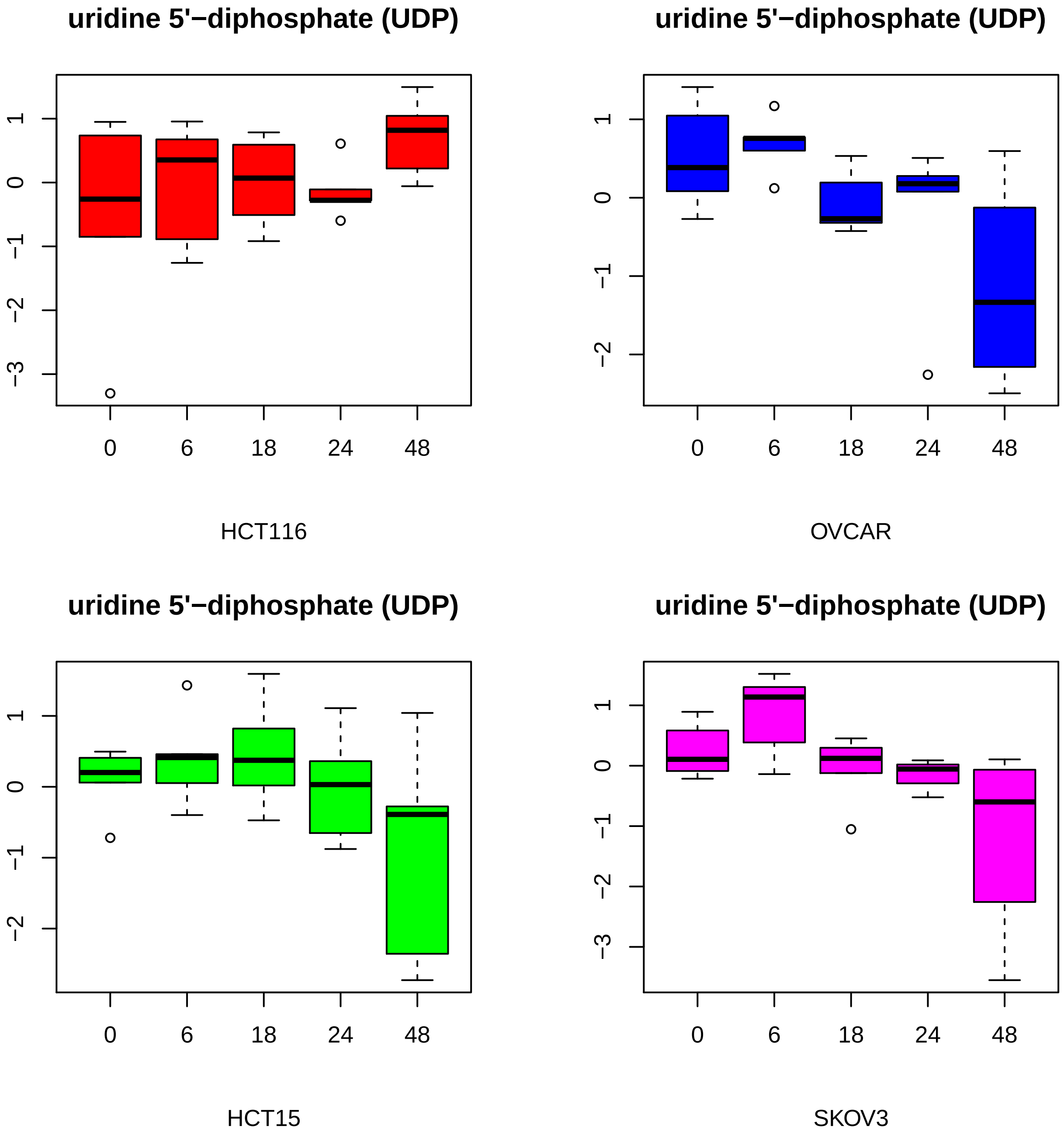
uridine 5'−diphosphate (UDP)
1
0
−1
−2
−3
0
6
18
24
48
HCT116
uridine 5'−diphosphate (UDP)
1
0
−1
−2
0
6
18
24
48
OVCAR
uridine 5'−diphosphate (UDP)
1
0
−1
−2
0
6
18
24
48
HCT15
uridine 5'−diphosphate (UDP)
1
0
−1
−2
−3
0
6
18
24
48
SKOV3

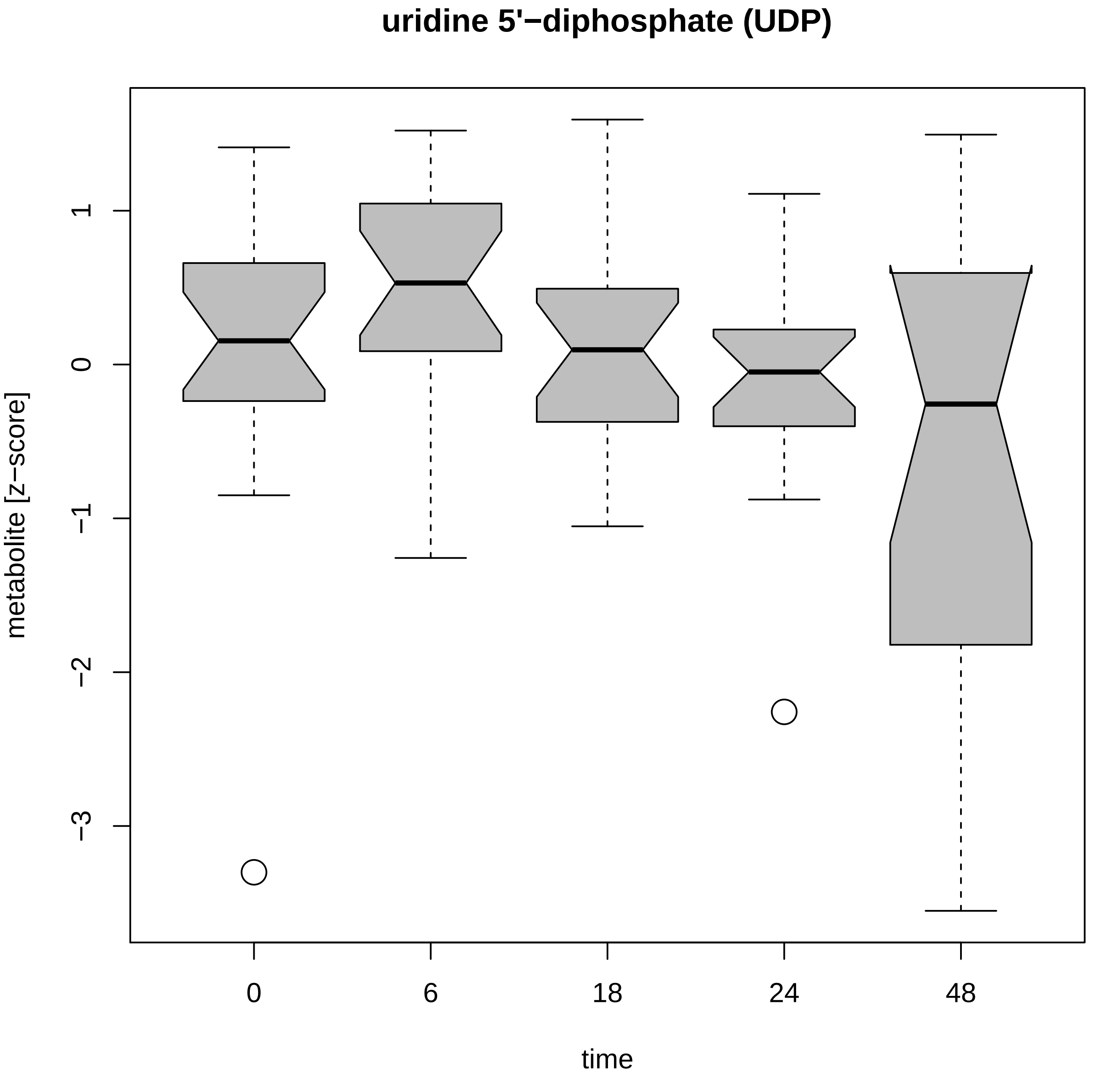
uridine 5'−diphosphate (UDP)
metabolite [z−score]
1
0
−1
−2
−3
0
6
18
24
48
time

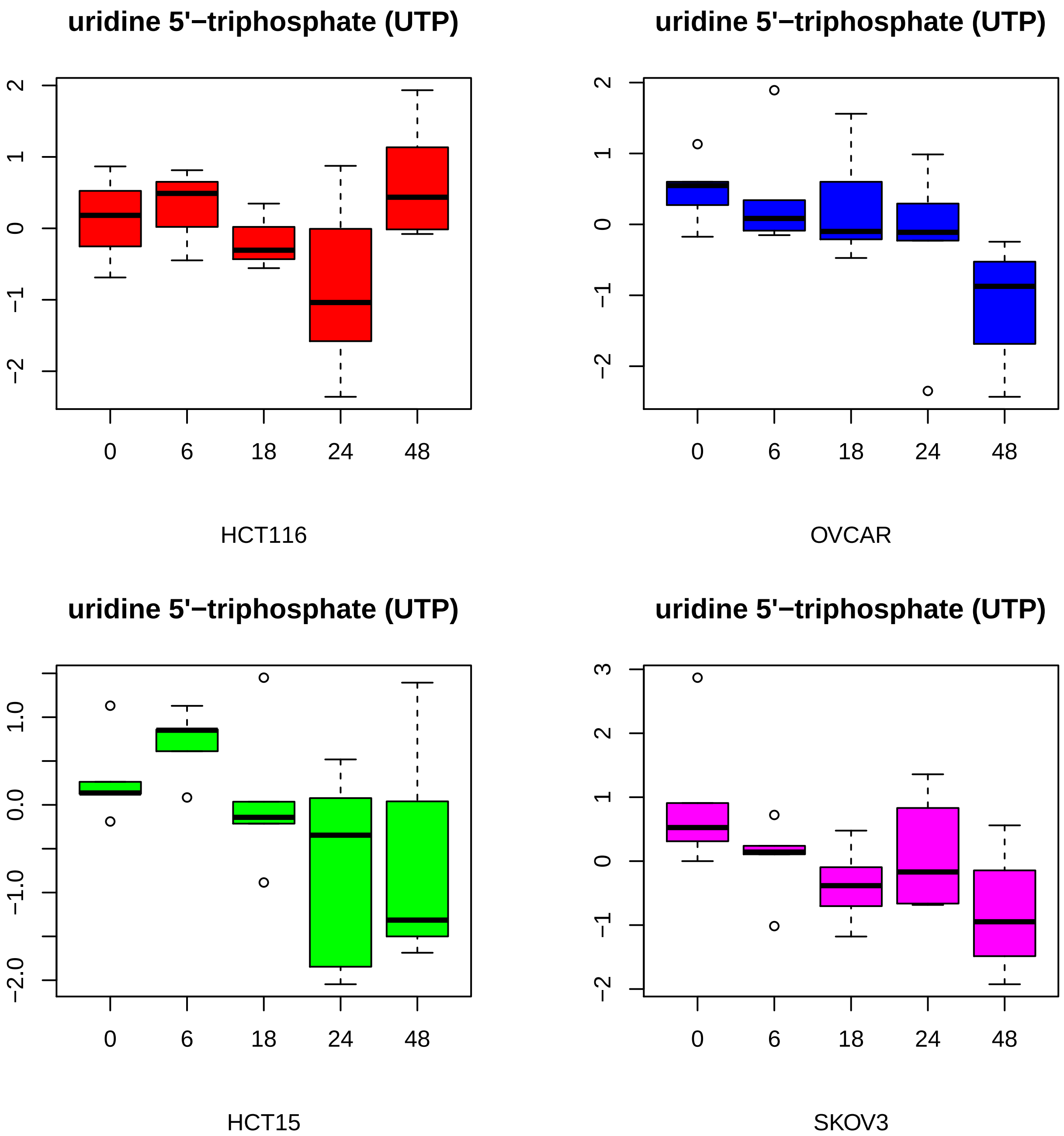
uridine 5'−triphosphate (UTP)
HCT116
uridine 5'−triphosphate (UTP)
OVCAR
uridine 5'−triphosphate (UTP)
HCT15
uridine 5'−triphosphate (UTP)
SKOV3
0
6
18
24
48

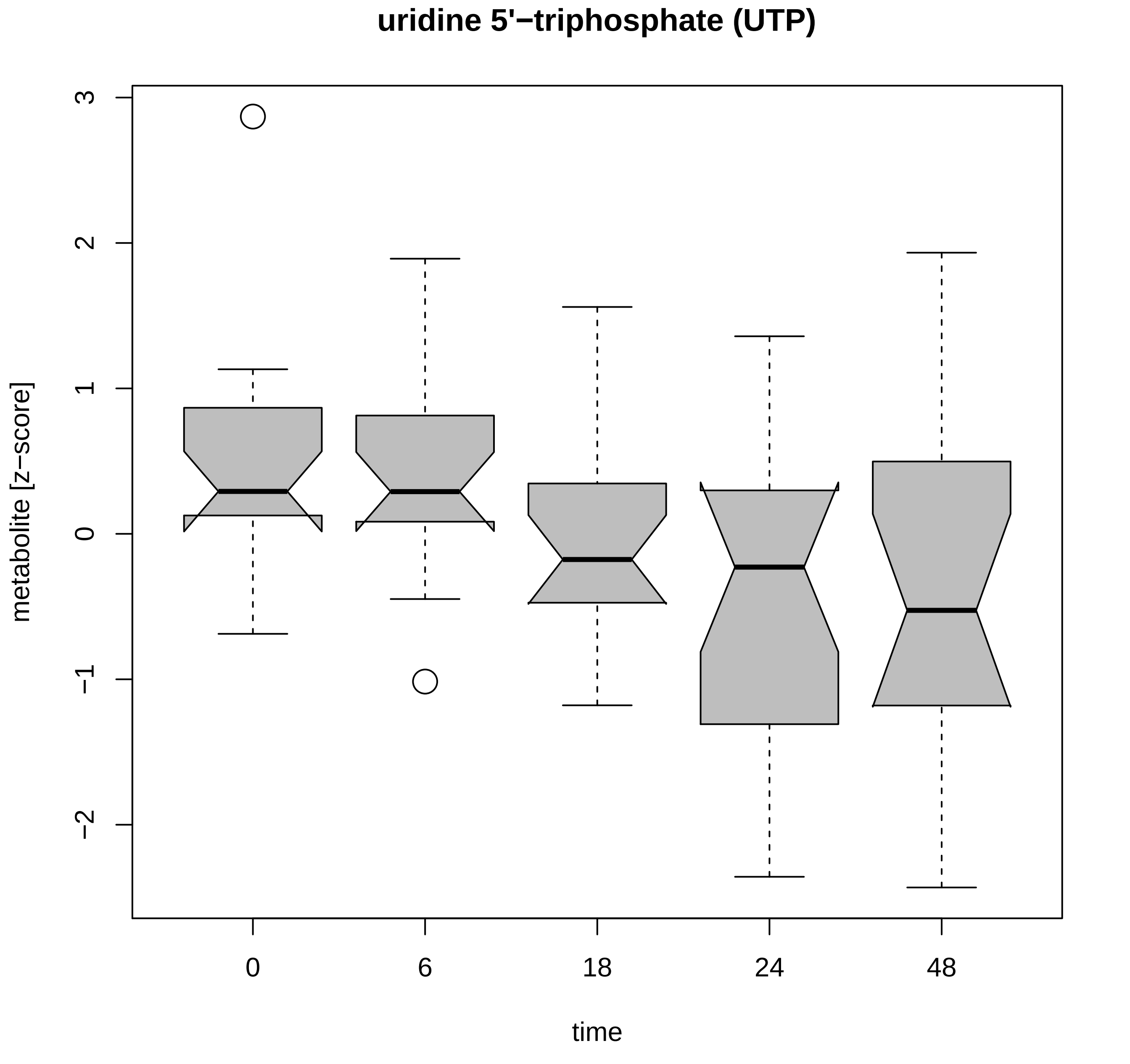

uridine 5'−triphosphate (UTP)
metabolite [z−score]
3
2
1
0
−1
−2
0
6
18
24
48
time

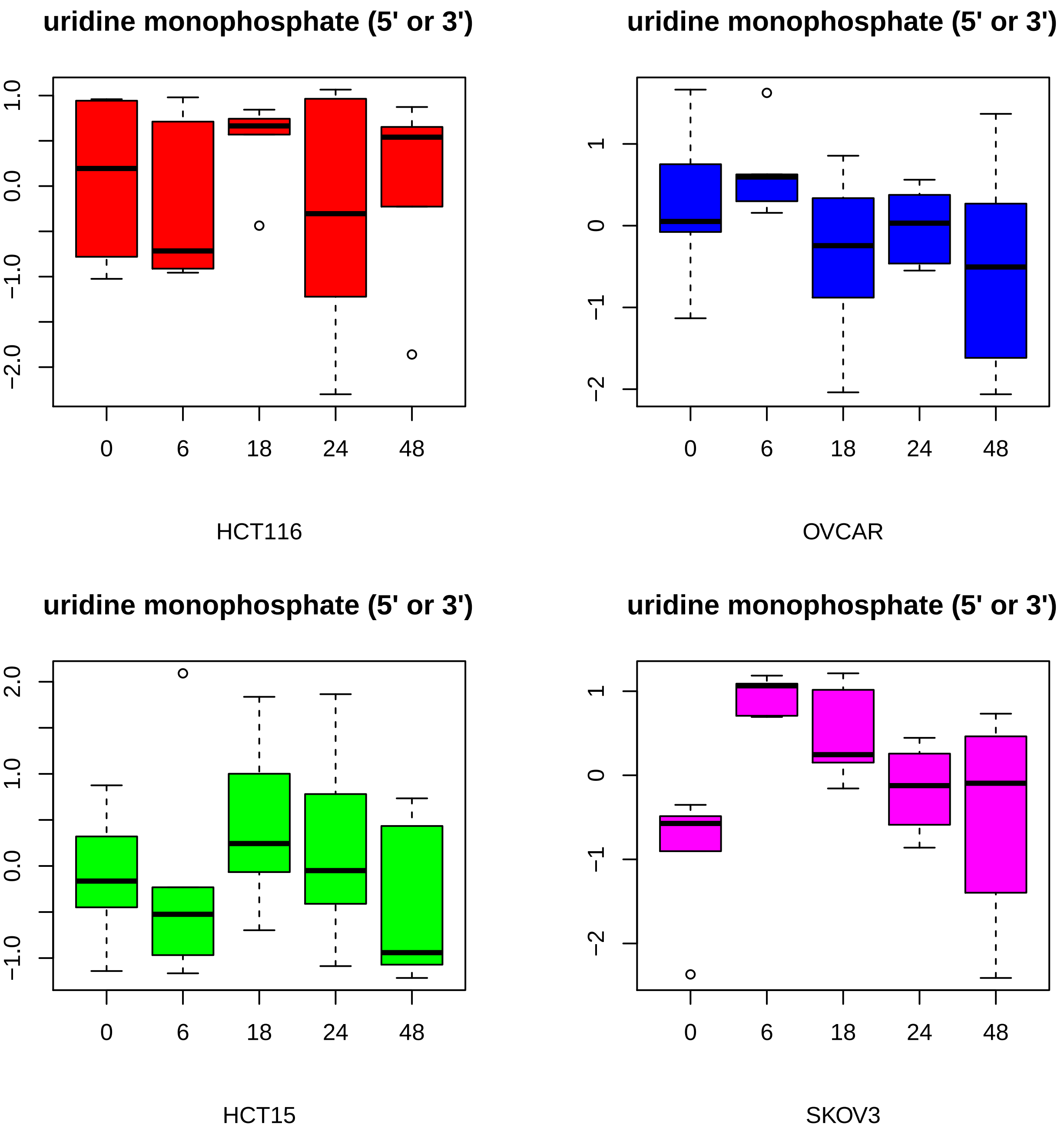
uridine monophosphate (5' or 3')
1.0
0.0
−1.0
−2.0
0
6
18
24
48
HCT116
uridine monophosphate (5' or 3')
1
0
−1
−2
0
6
18
24
48
OVCAR
uridine monophosphate (5' or 3')
2.0
1.0
0.0
−1.0
0
6
18
24
48
HCT15
uridine monophosphate (5' or 3')
1
0
−1
−2
0
6
18
24
48
SKOV3

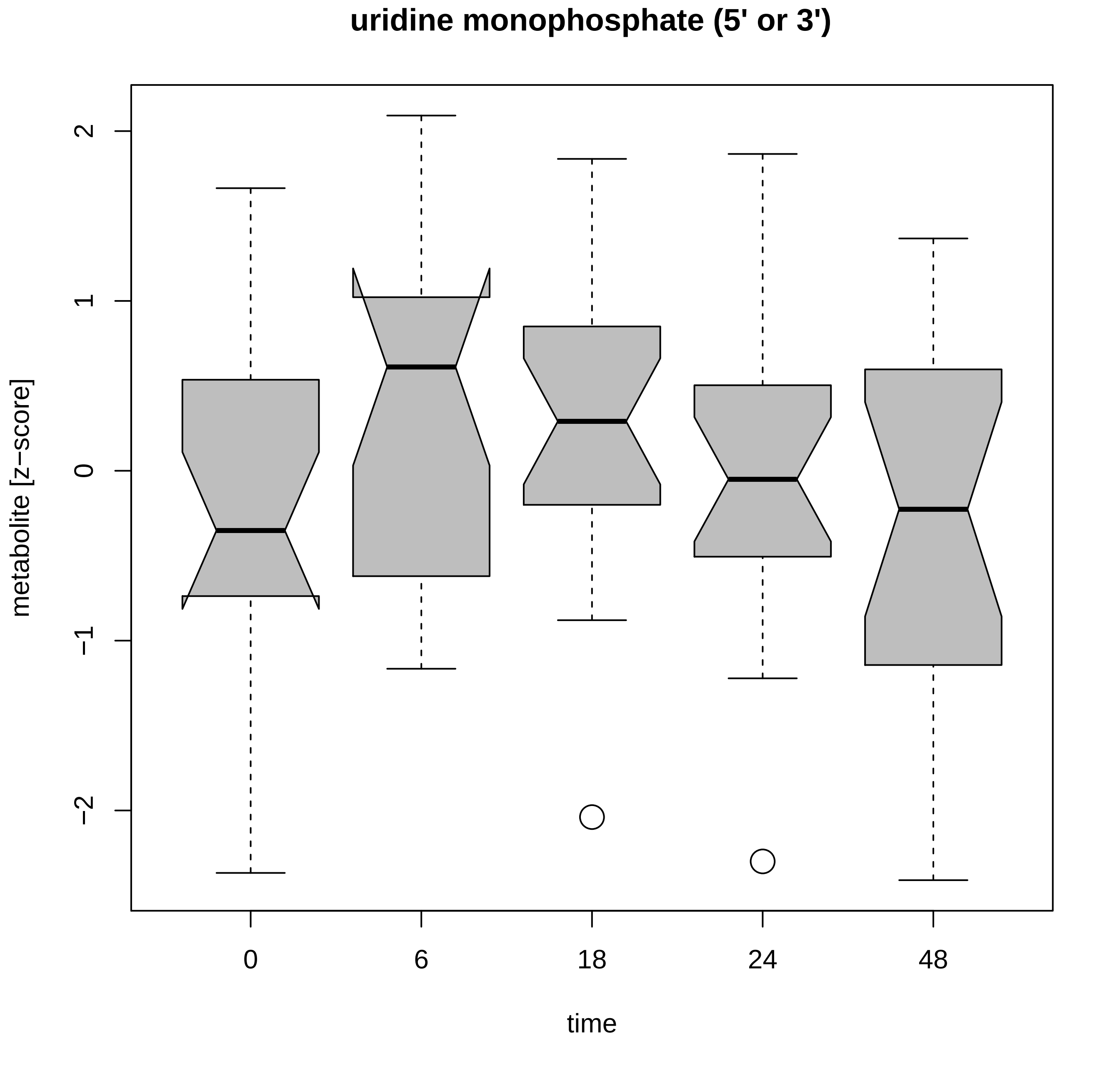

uridine monophosphate (5' or 3')
metabolite [z−score]
2
1
0
−1
−2
0
6
18
24
48
time

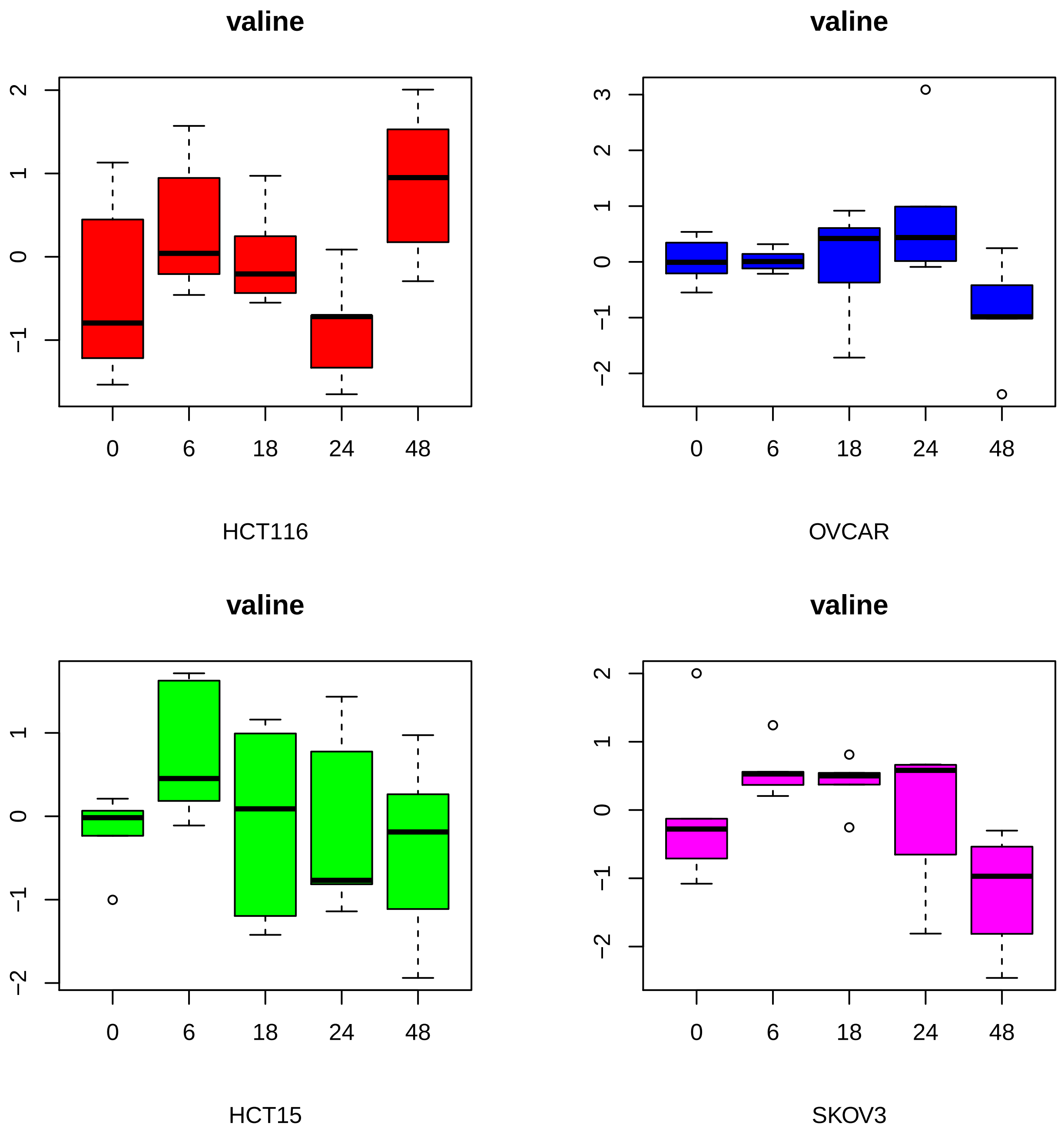
valine
HCT116
valine
OVCAR
valine
HCT15
valine
SKOV3
0
6
18
24
48

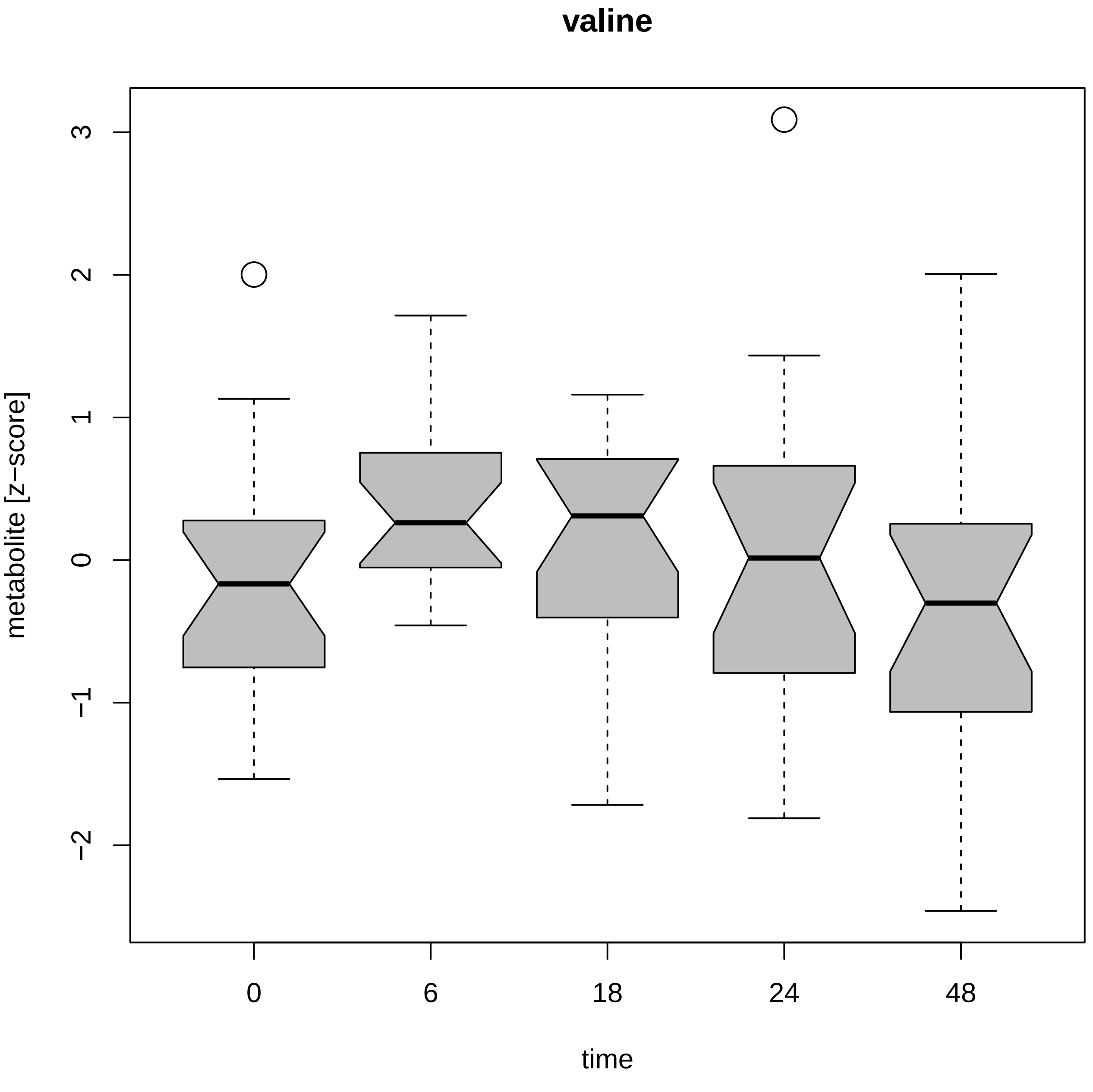
valine
metabolite [z−score]
3
2
1
0
−1
−2
0
6
18
24
48
time

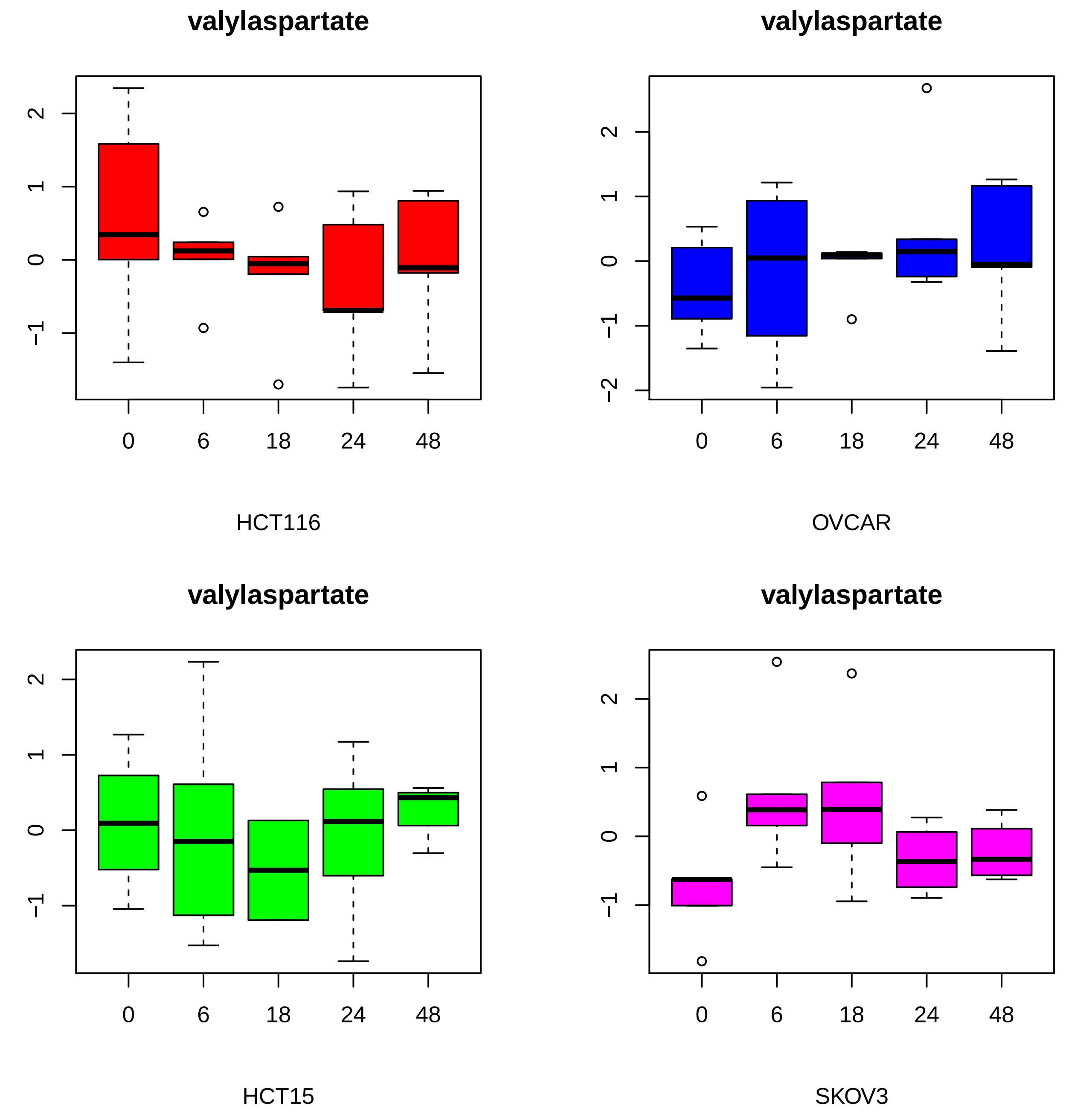

valylaspartate
HCT116
valylaspartate
OVCAR
valylaspartate
HCT15
valylaspartate
SKOV3
0
6
18
24
48
2
1
0
−1
−2

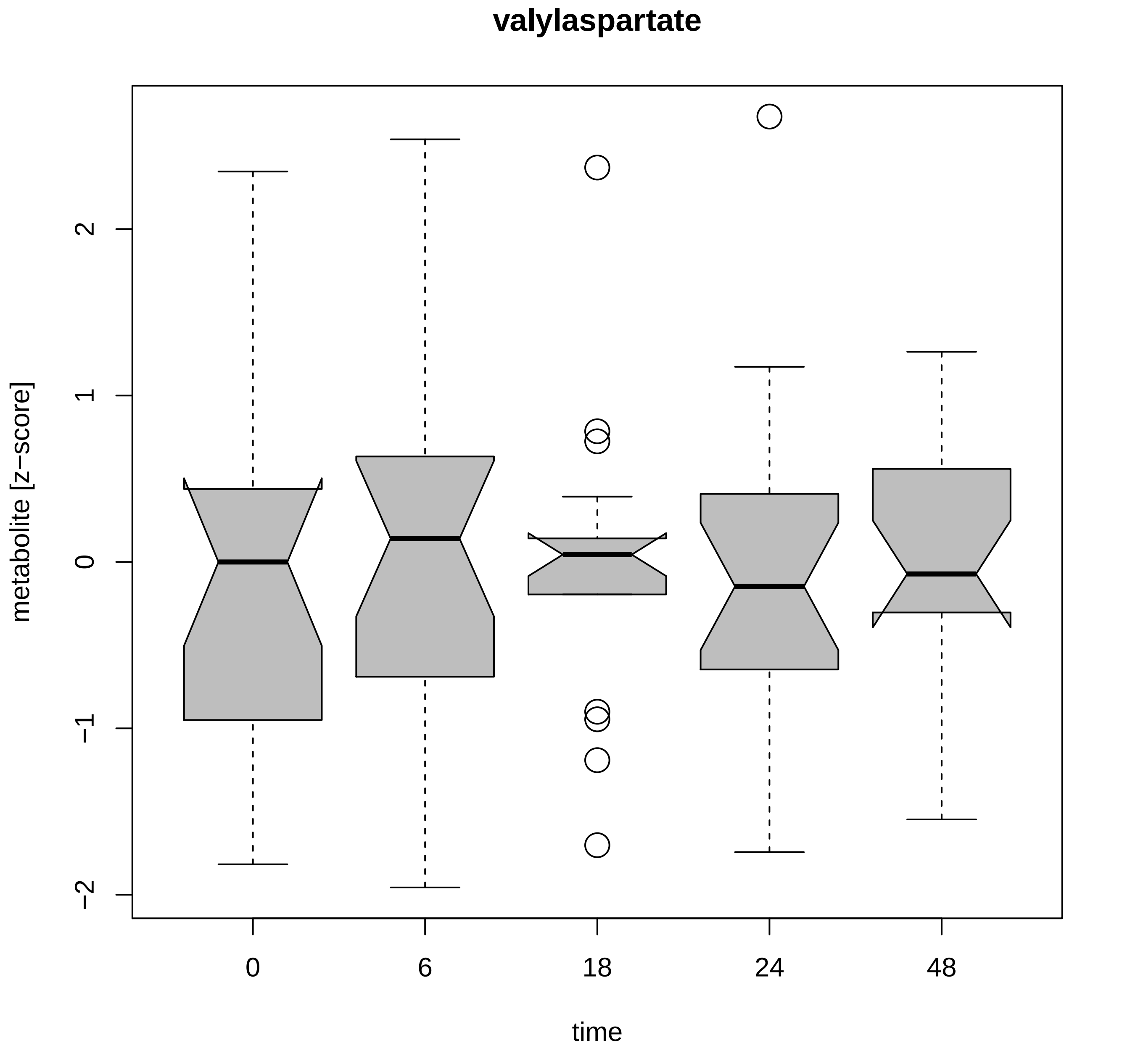
valylaspartate
metabolite [z−score]
2
1
0
−1
−2
0
6
18
24
48
time

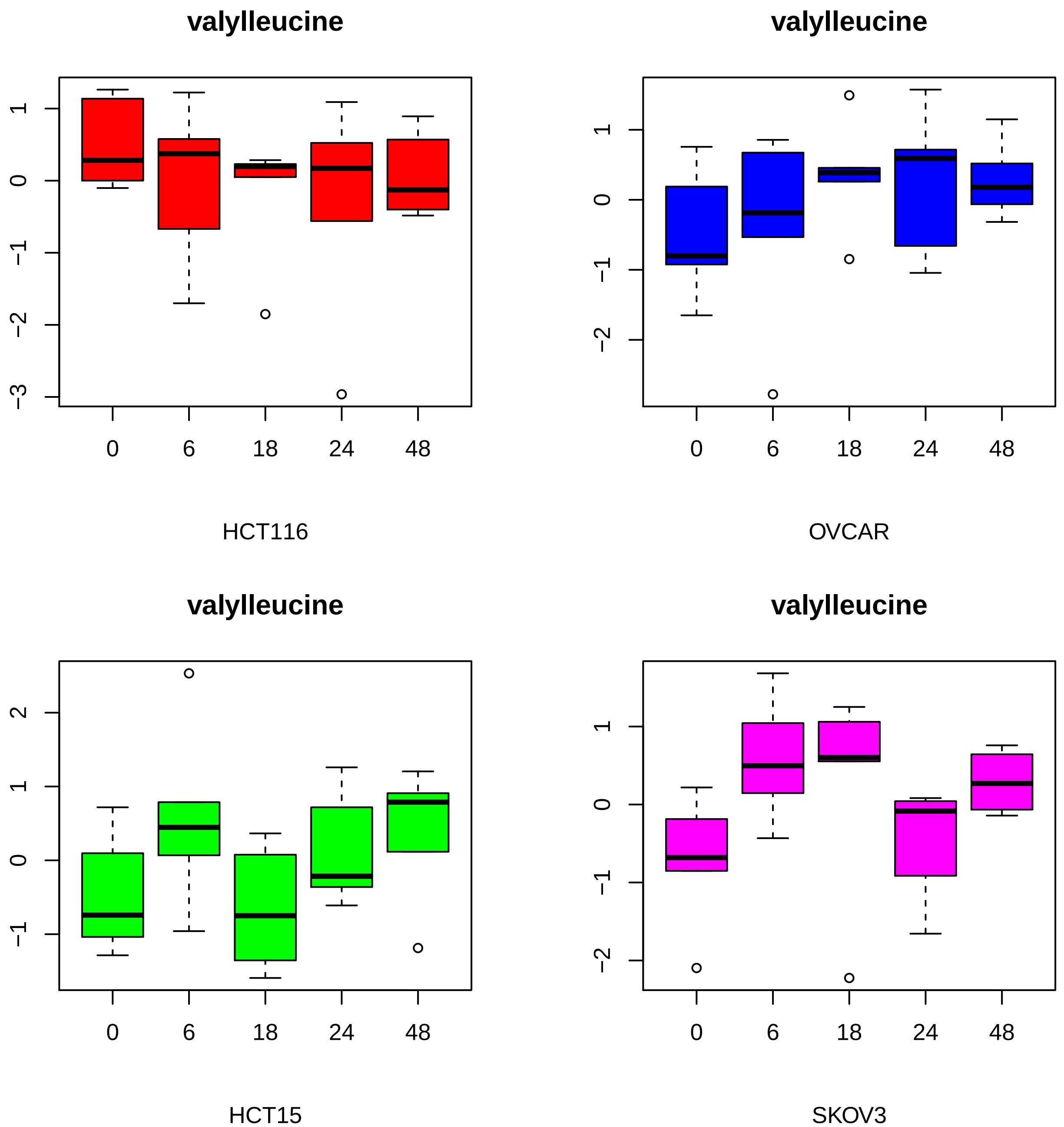
valylleucine
0
6
18
24
48
HCT116
valylleucine
OVCAR
valylleucine
HCT15
valylleucine
SKOV3

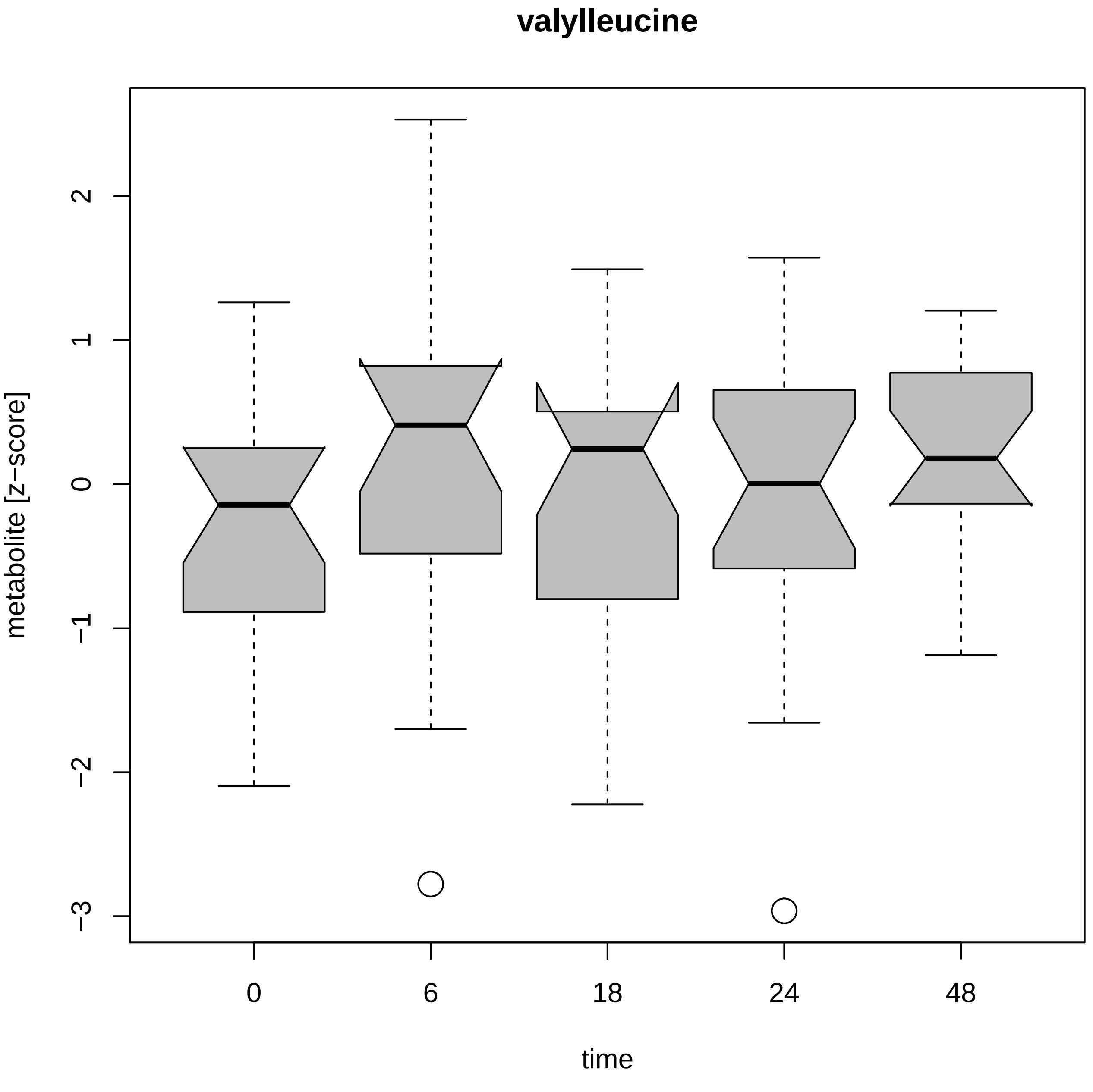

valylleucine
metabolite [z−score]
2
1
0
−1
−2
−3
0
6
18
24
48
time

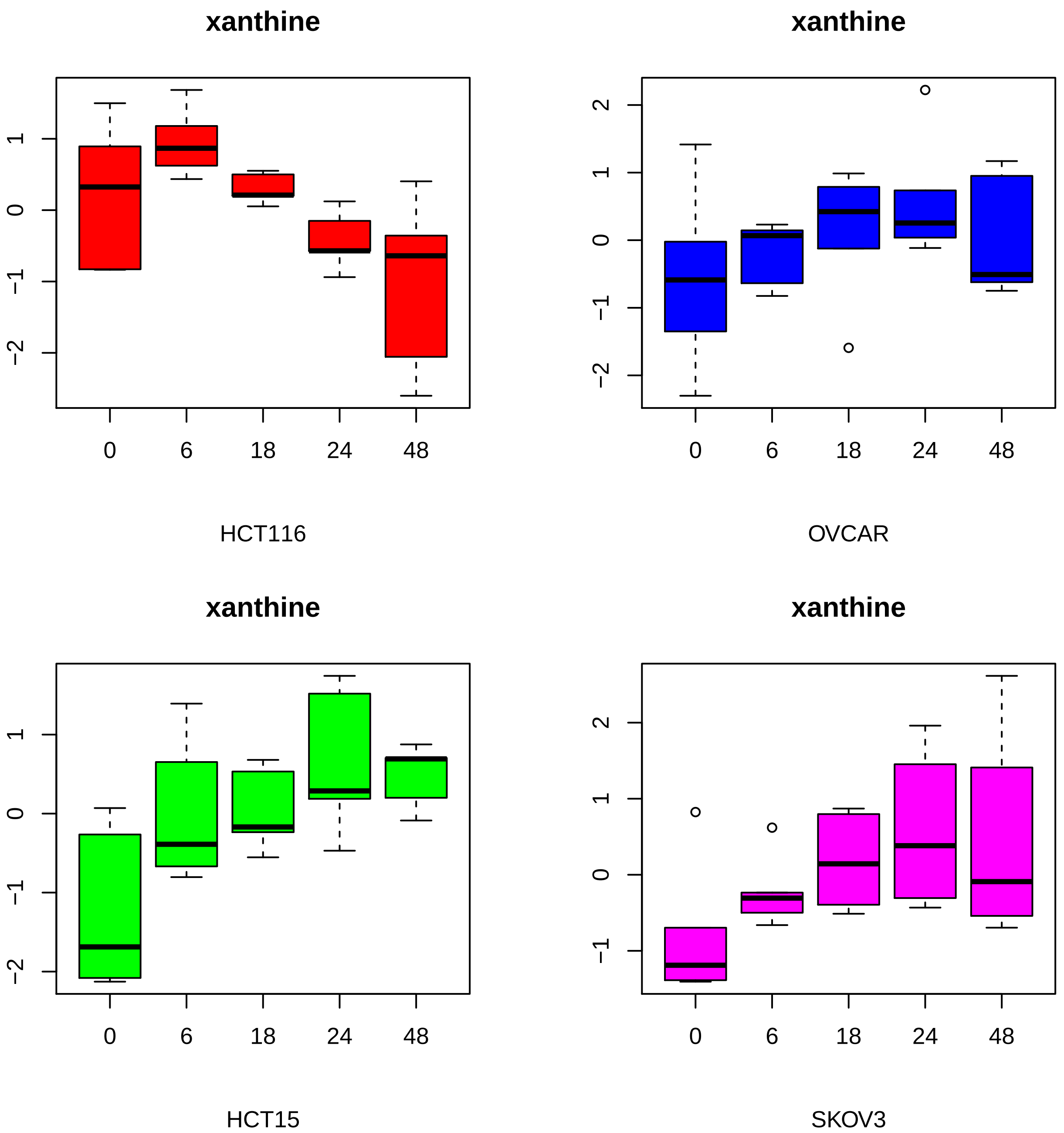
xanthine
1
0
−1
−2
0
6
18
24
48
HCT116
xanthine
2
1
0
−1
−2
0
6
18
24
48
OVCAR
xanthine
1
0
−1
−2
0
6
18
24
48
HCT15
xanthine
2
1
0
−1
0
6
18
24
48
SKOV3

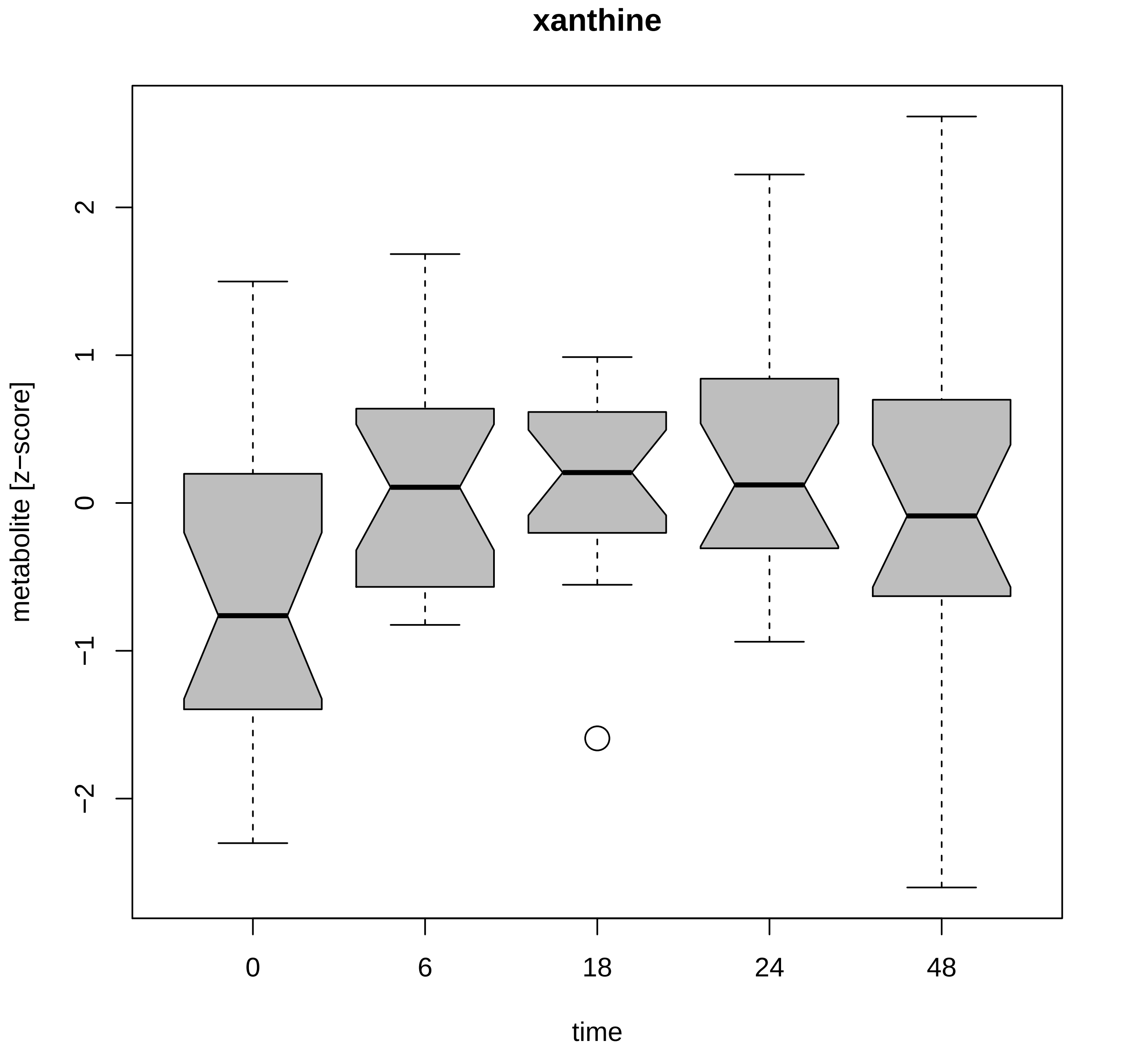
xanthine
metabolite [z−score]
2
1
0
−1
−2
0
6
18
24
48
time

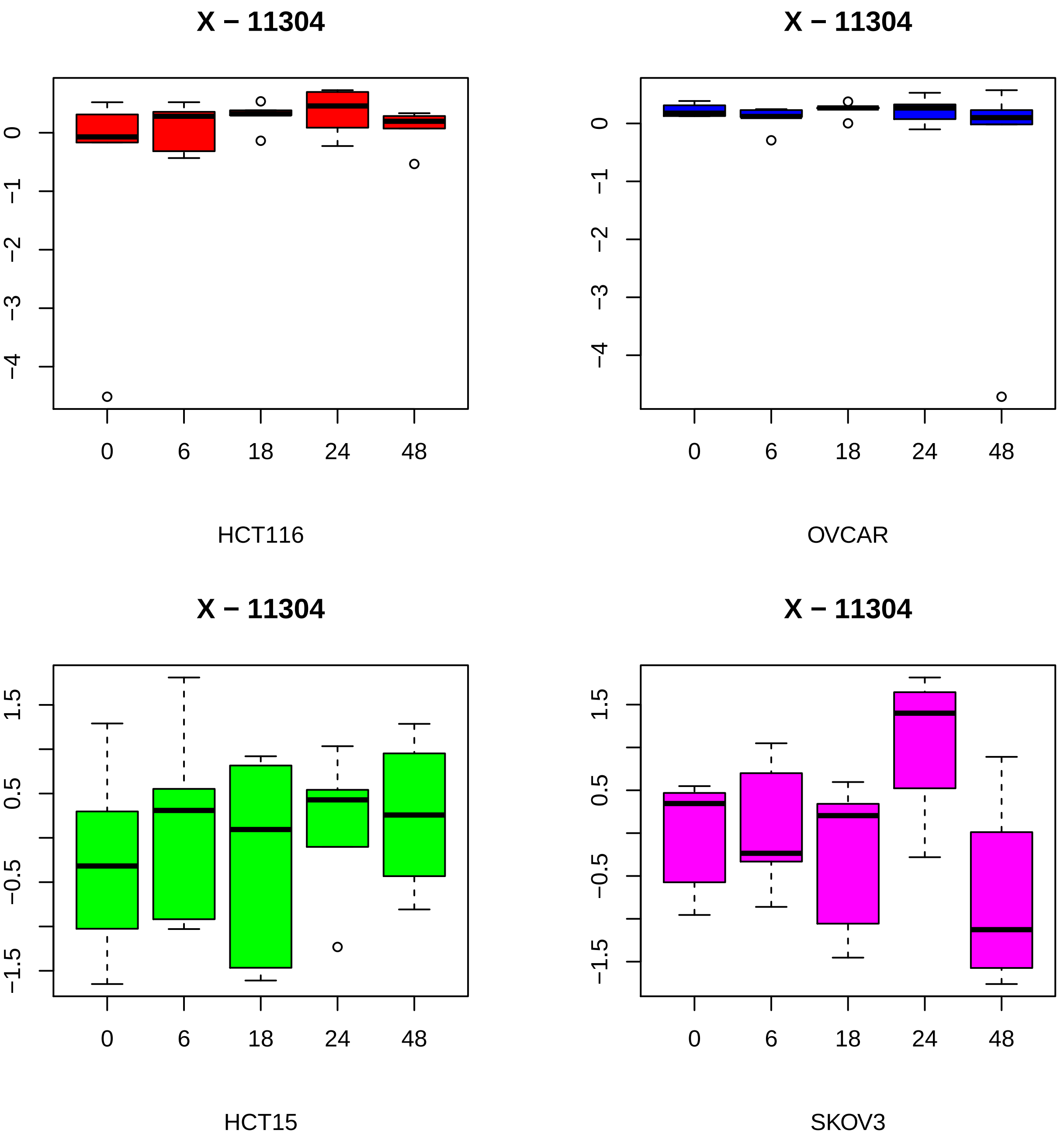
X − 11304
0
−1
−2
−3
−4
0
6
18
24
48
HCT116
X − 11304
0
−1
−2
−3
−4
0
6
18
24
48
OVCAR
X − 11304
1.5
0.5
−0.5
−1.5
0
6
18
24
48
HCT15
X − 11304
1.5
0.5
−0.5
−1.5
0
6
18
24
48
SKOV3

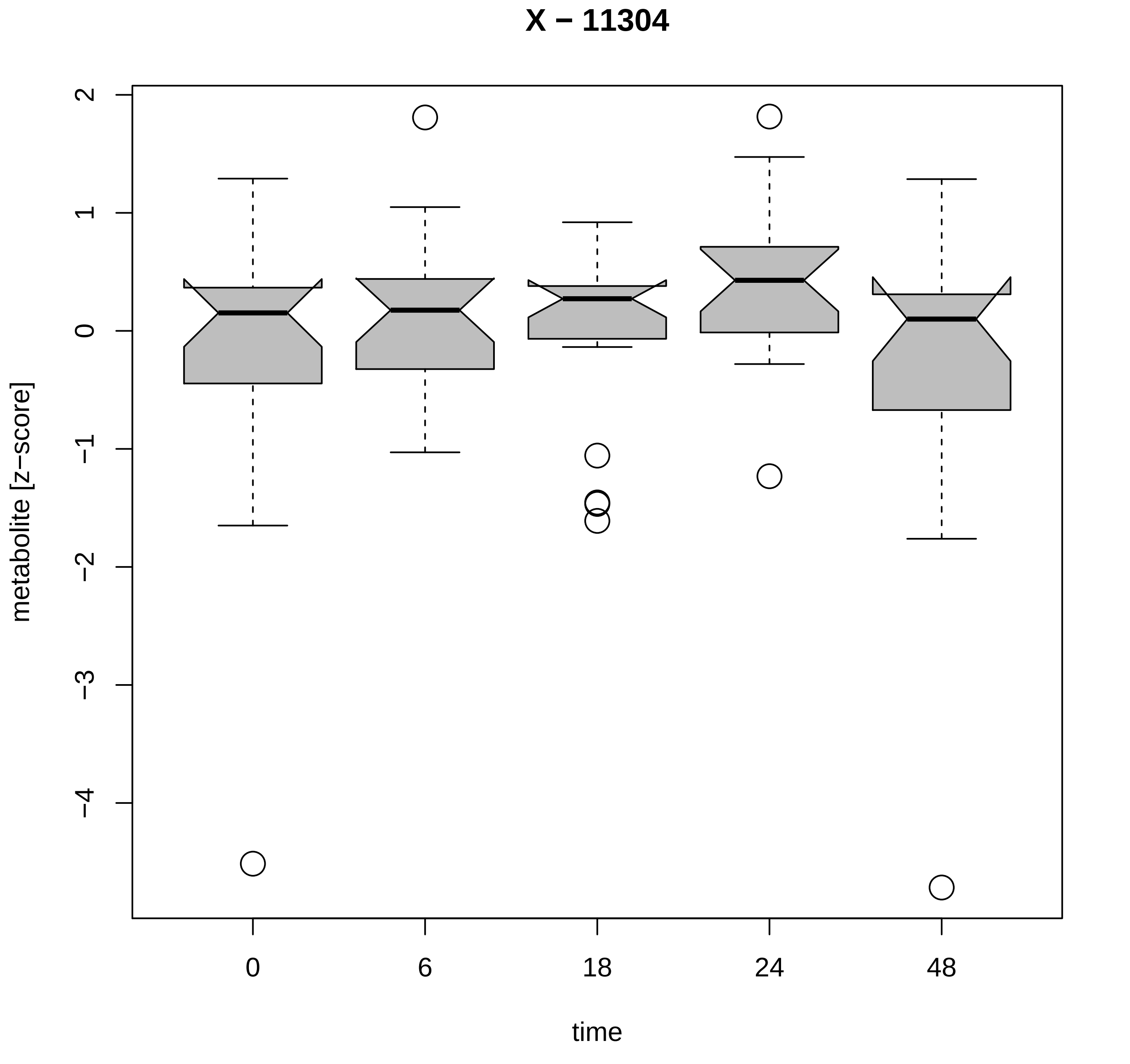
X − 11304
metabolite [z−score]
time
2
1
0
−1
−2
−3
−4
0
6
18
24
48

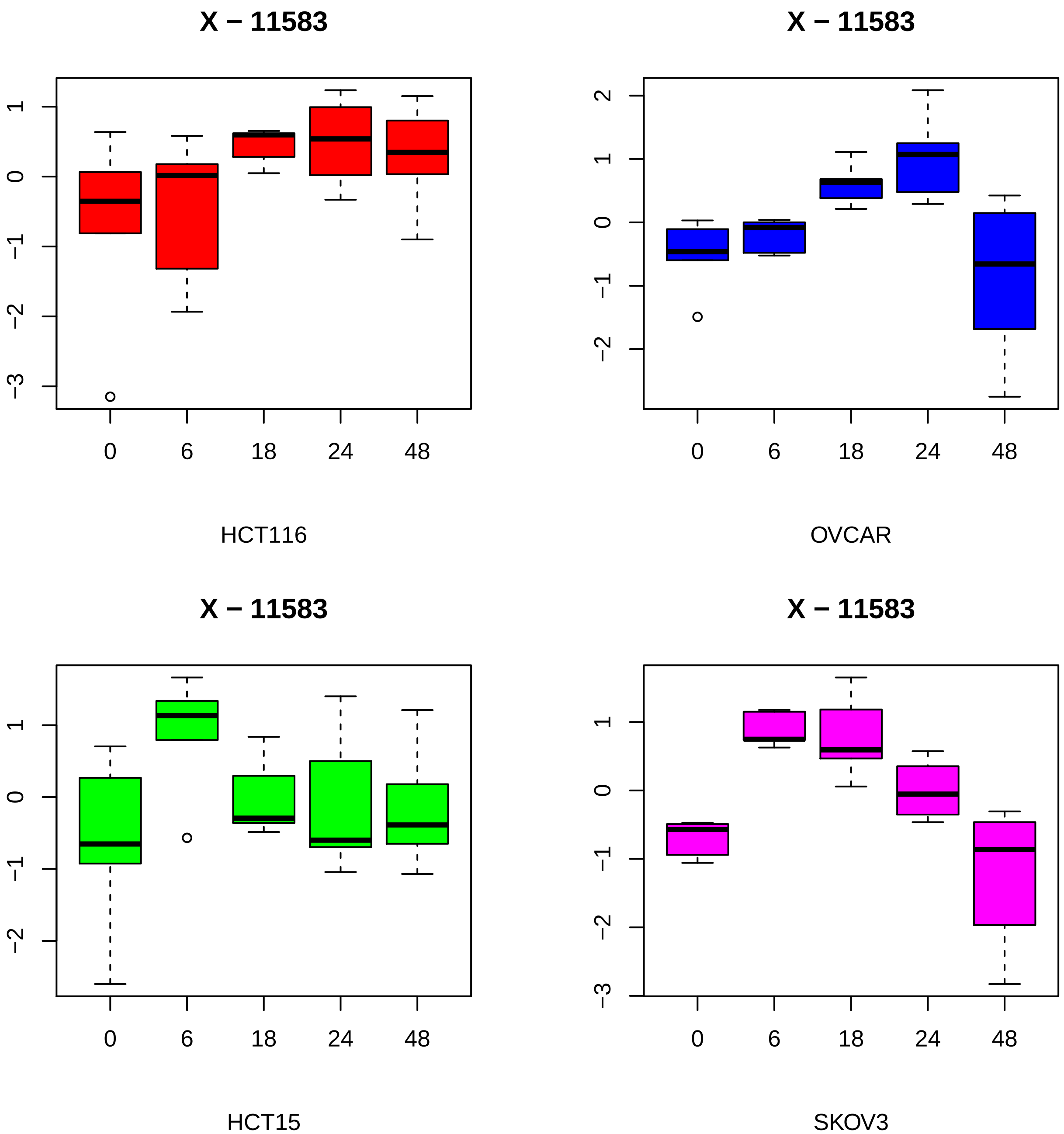
X − 11583
HCT116
X − 11583
OVCAR
X − 11583
HCT15
X − 11583
SKOV3
0
6
18
24
48

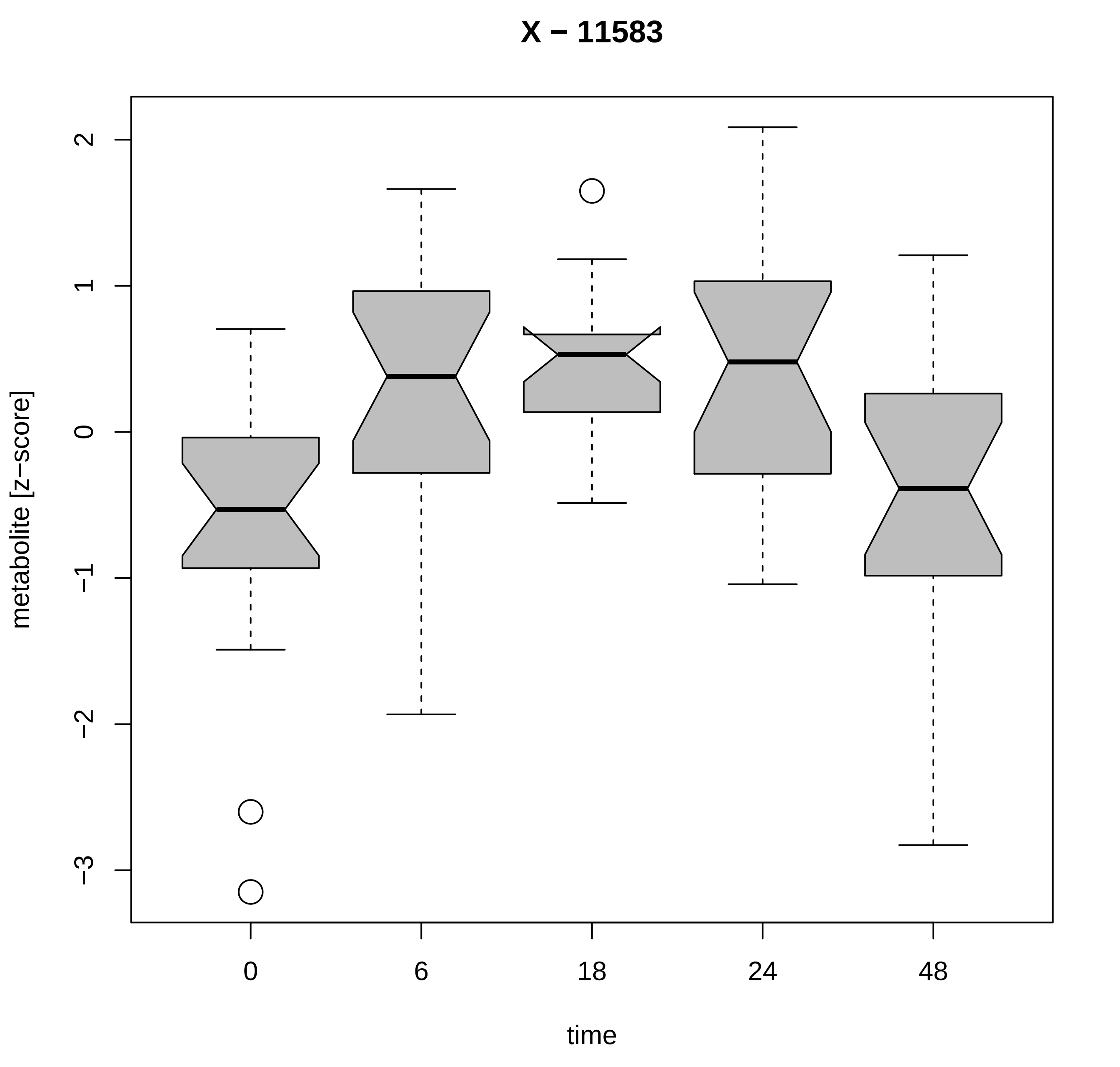
X − 11583
metabolite [z−score]
2
1
0
−1
−2
−3
0
6
18
24
48
time

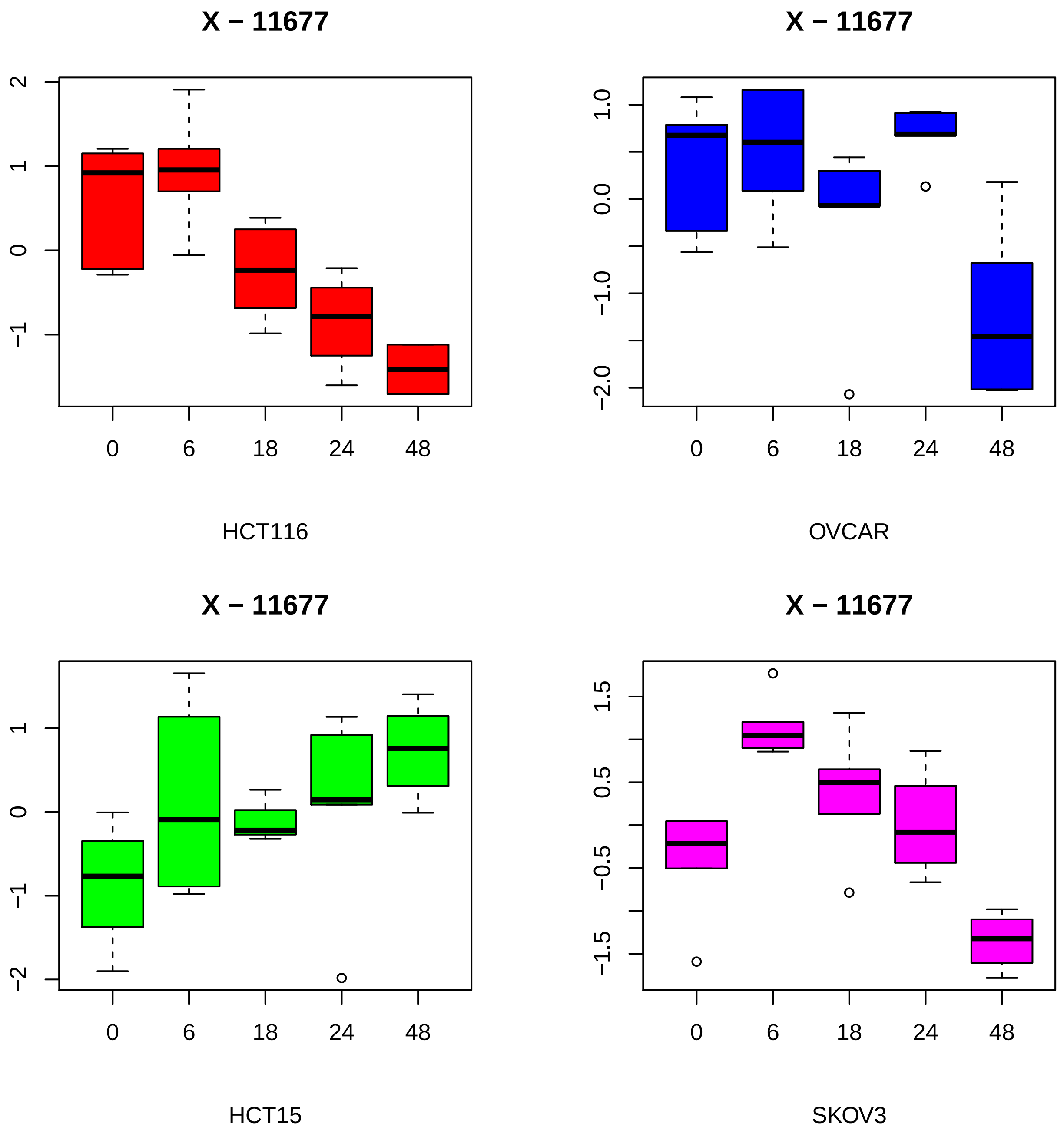

X − 11677
HCT116
X − 11677
OVCAR
X − 11677
HCT15
X − 11677
SKOV3
0
6
18
24
48

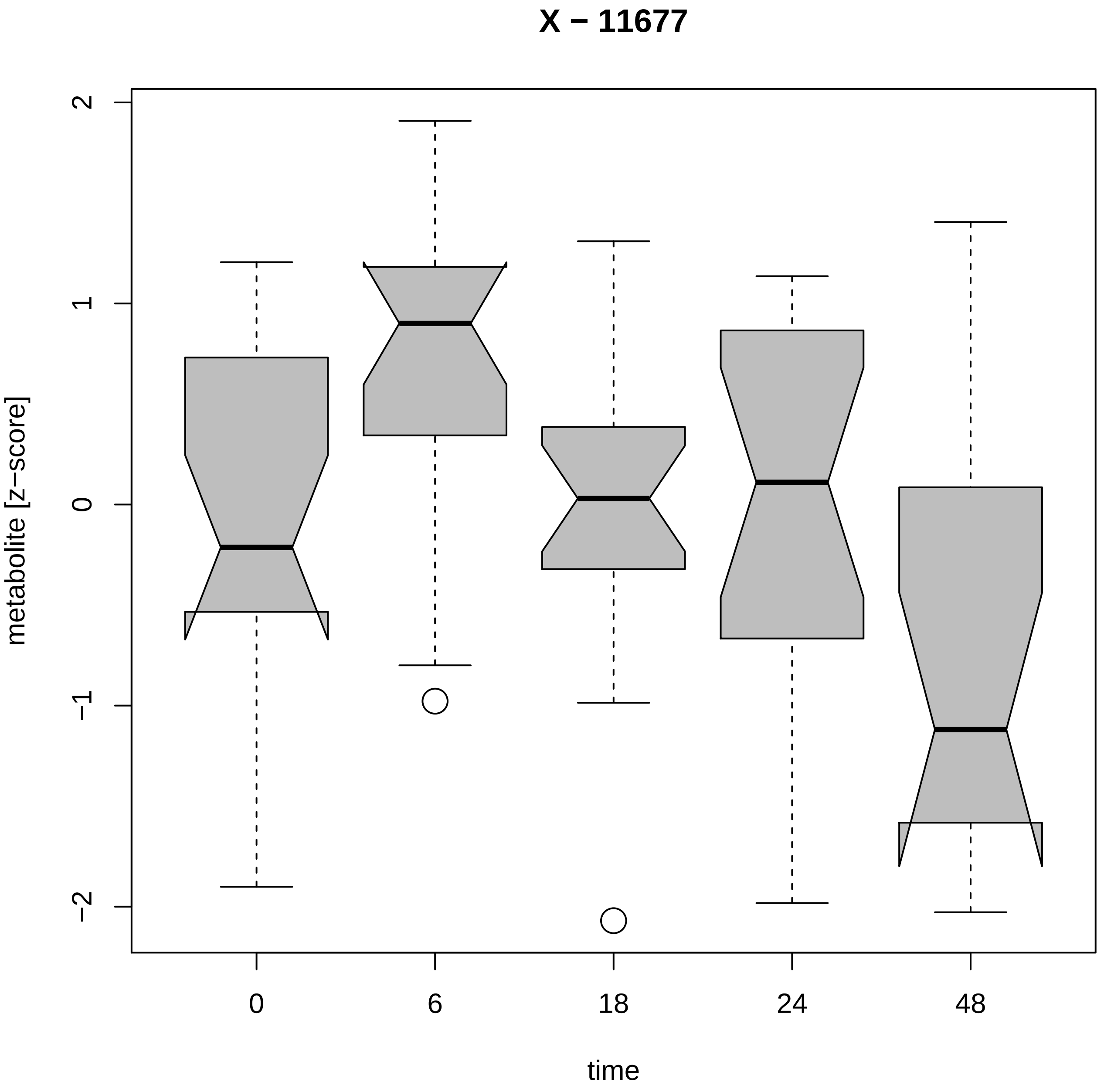

X − 11677
metabolite [z−score]
2
1
0
−1
−2
0
6
18
24
48
time

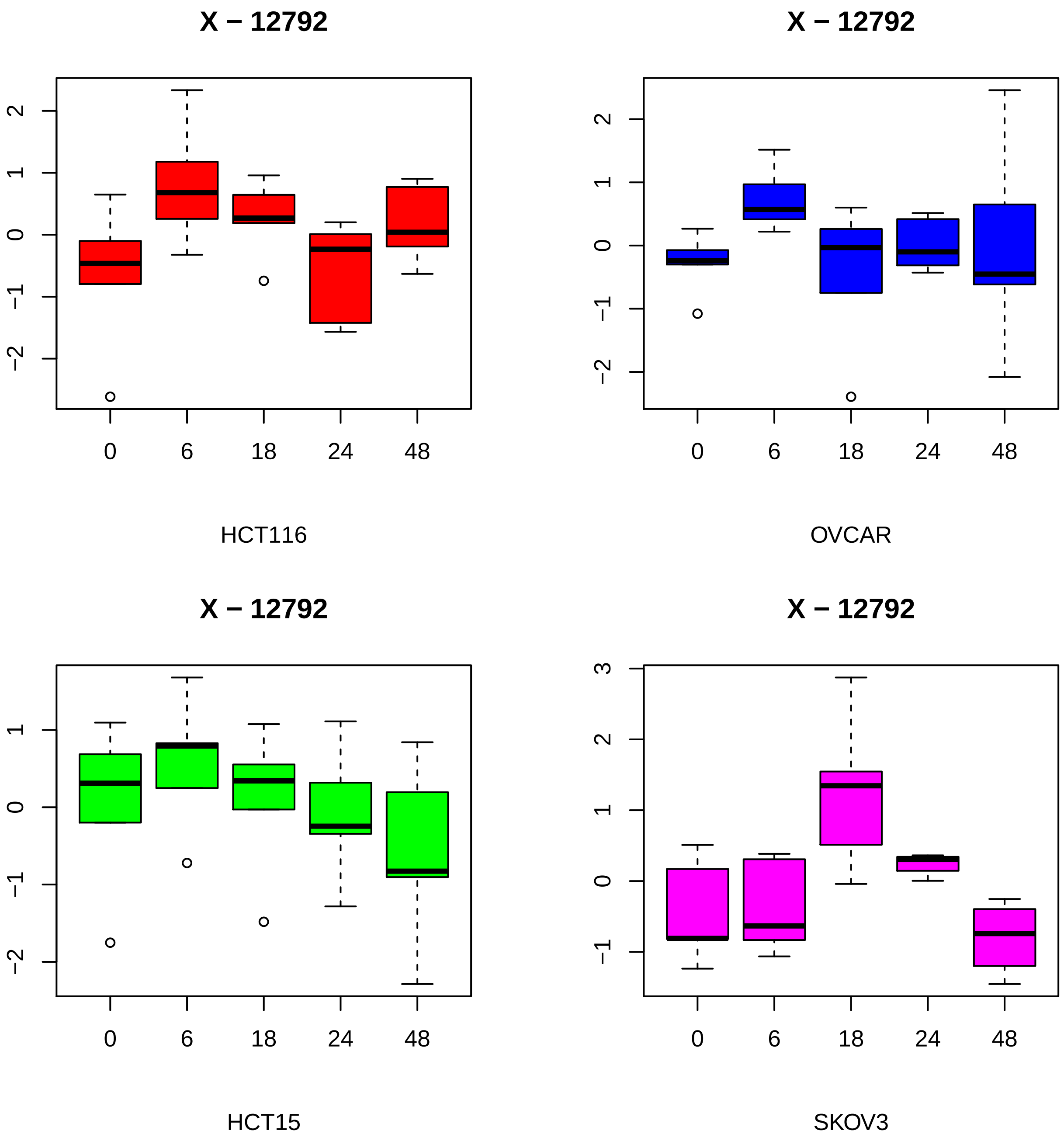
X − 12792
HCT116
X − 12792
OVCAR
X − 12792
HCT15
X − 12792
SKOV3
0
6
18
24
48
−2
−1
0
1
2
3

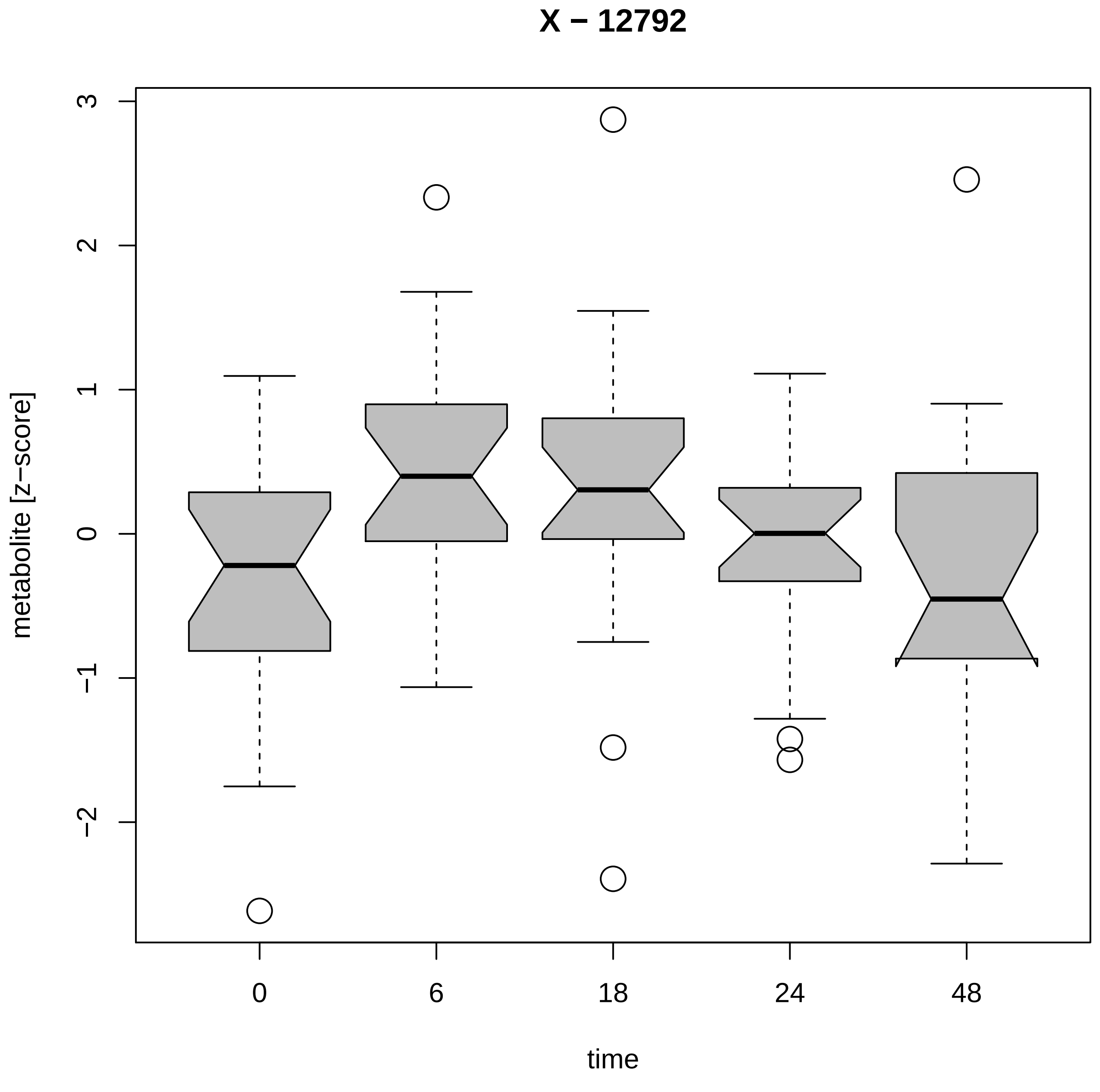

X − 12792
metabolite [z−score]
3
2
1
0
−1
−2
0
6
18
24
48
time

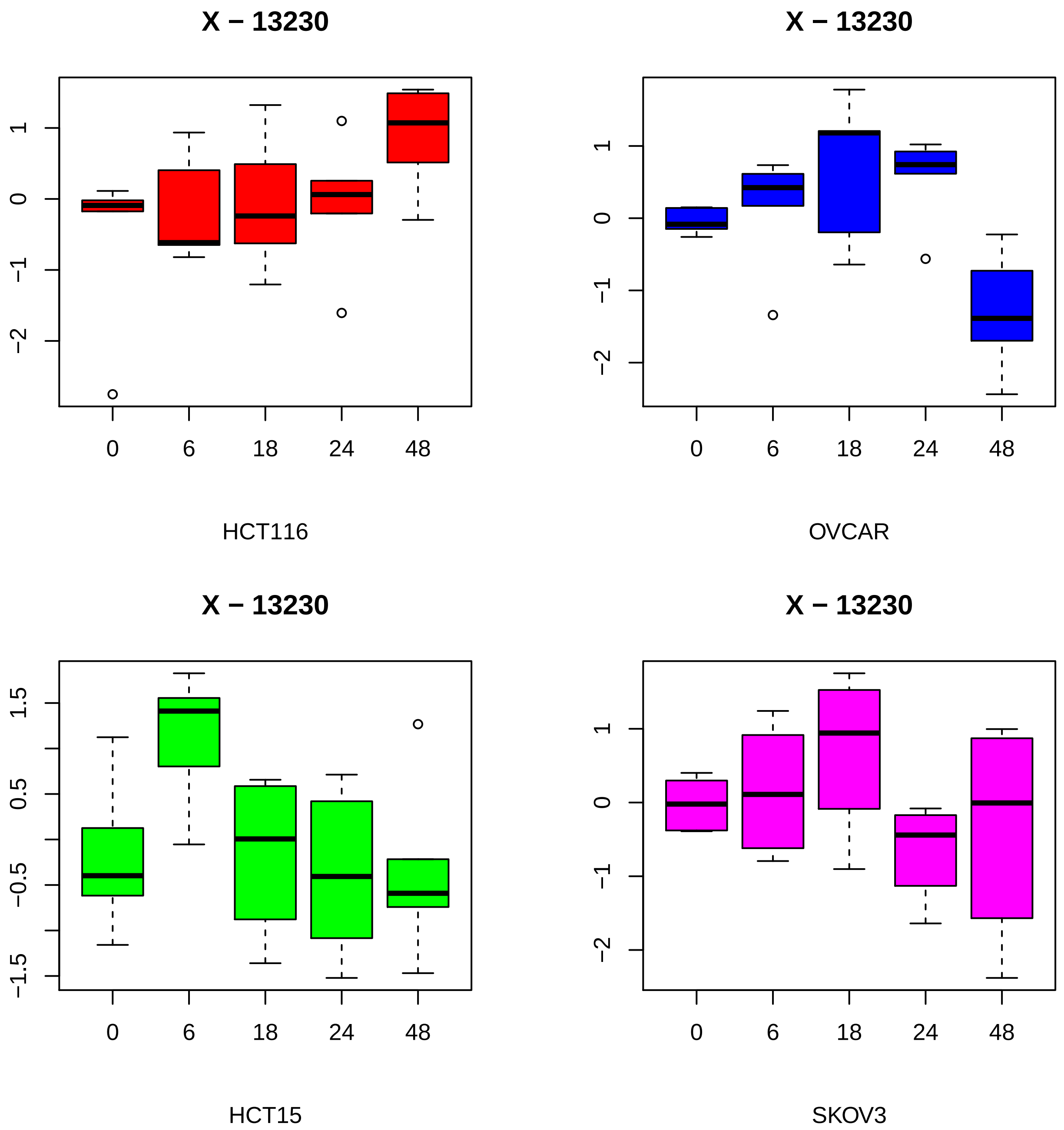
X − 13230
HCT116
OVCAR
HCT15
SKOV3
0
6
18
24
48
1
0
−1
−2
1.5
0.5
−0.5
−1.5

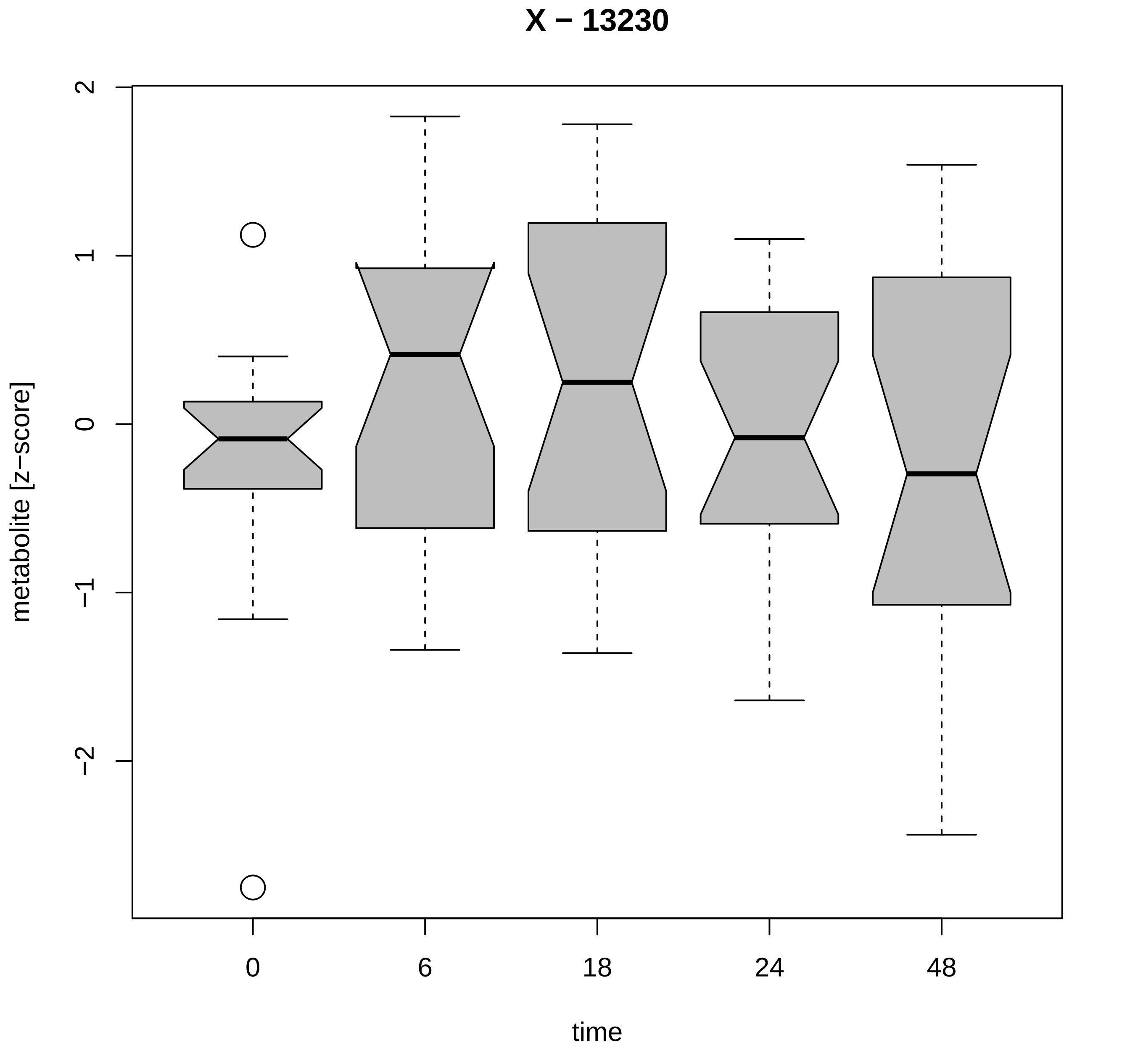

X − 13230
metabolite [z−score]
2
1
0
−1
−2
0
6
18
24
48
time

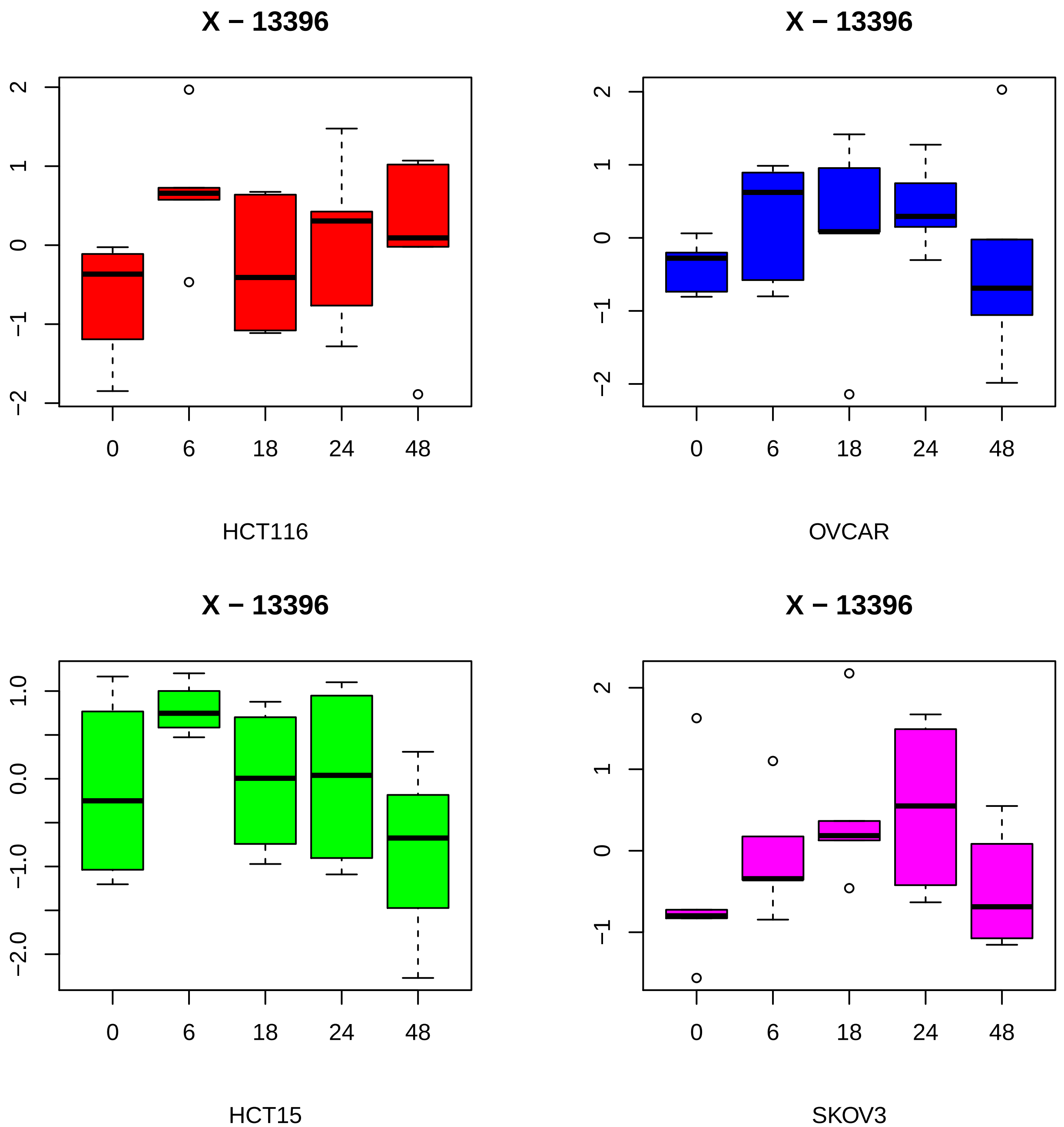

X − 13396
HCT116
X − 13396
OVCAR
X − 13396
HCT15
X − 13396
SKOV3
0
6
18
24
48

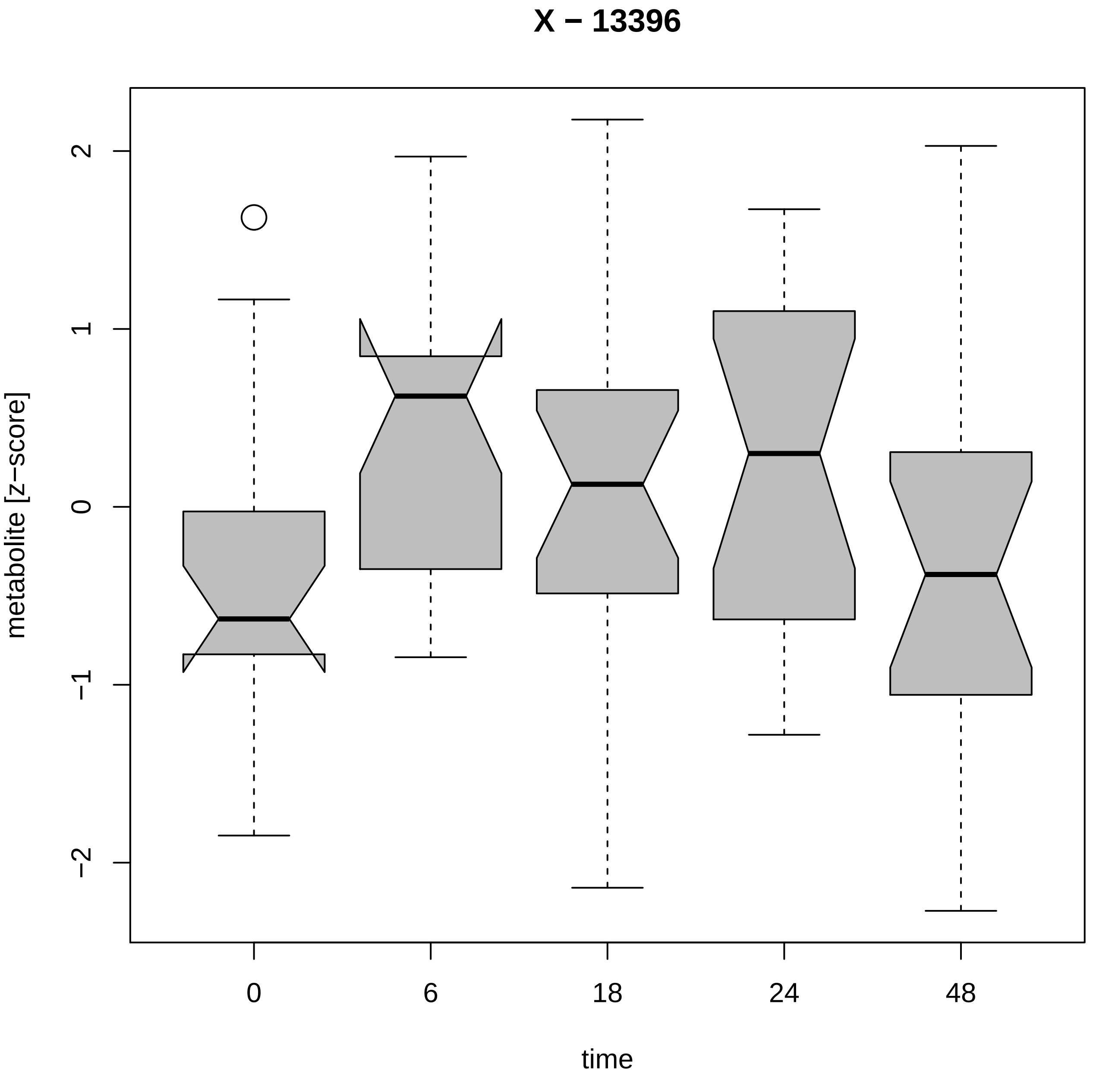
X − 13396
metabolite [z−score]
2
1
0
−1
−2
0
6
18
24
48
time

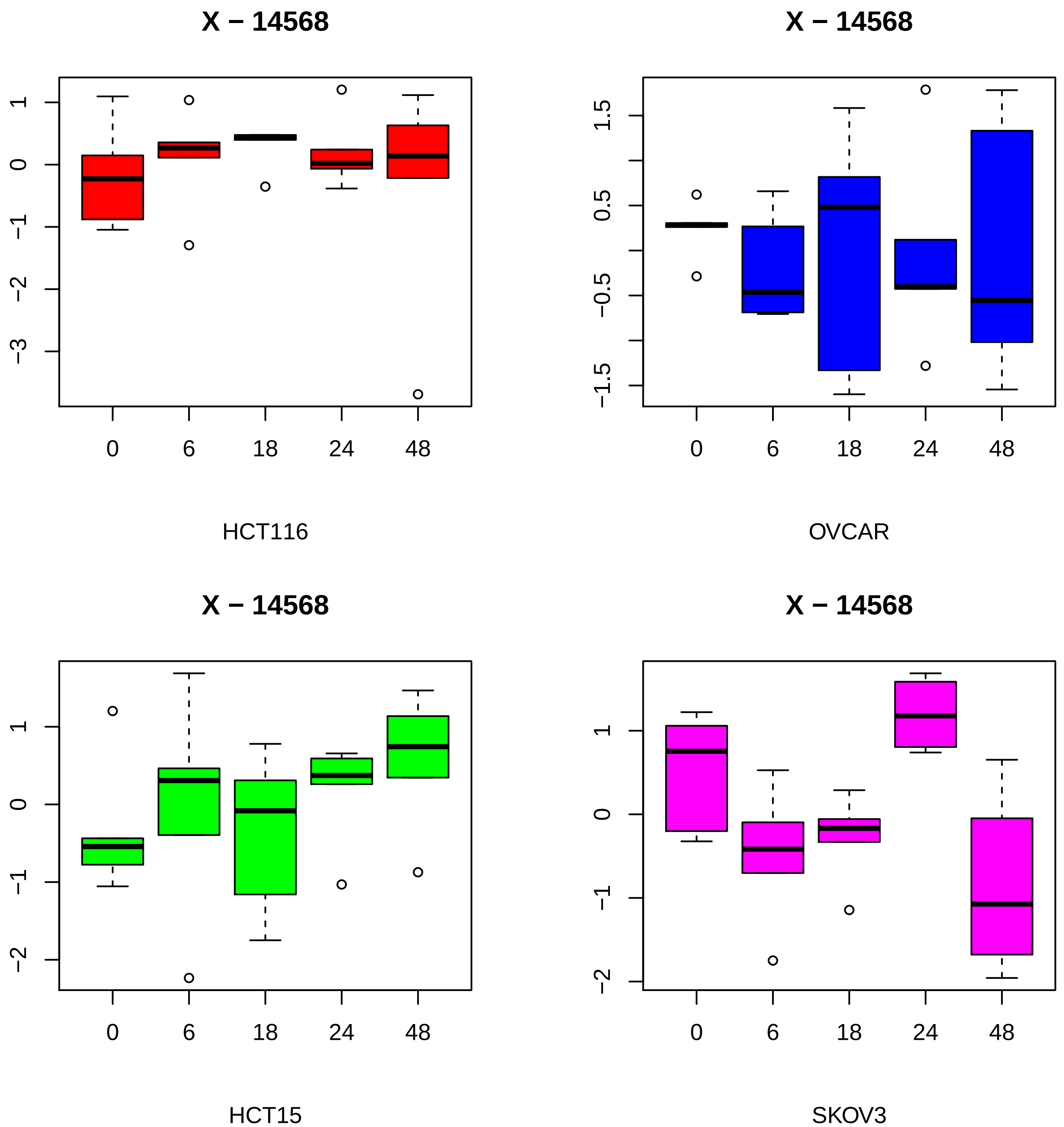

X − 14568
1
0
−1
−2
−3
0
6
18
24
48
HCT116
X − 14568
1.5
0.5
−0.5
−1.5
0
6
18
24
48
OVCAR
X − 14568
1
0
−1
−2
0
6
18
24
48
HCT15
X − 14568
1
0
−1
−2
0
6
18
24
48
SKOV3

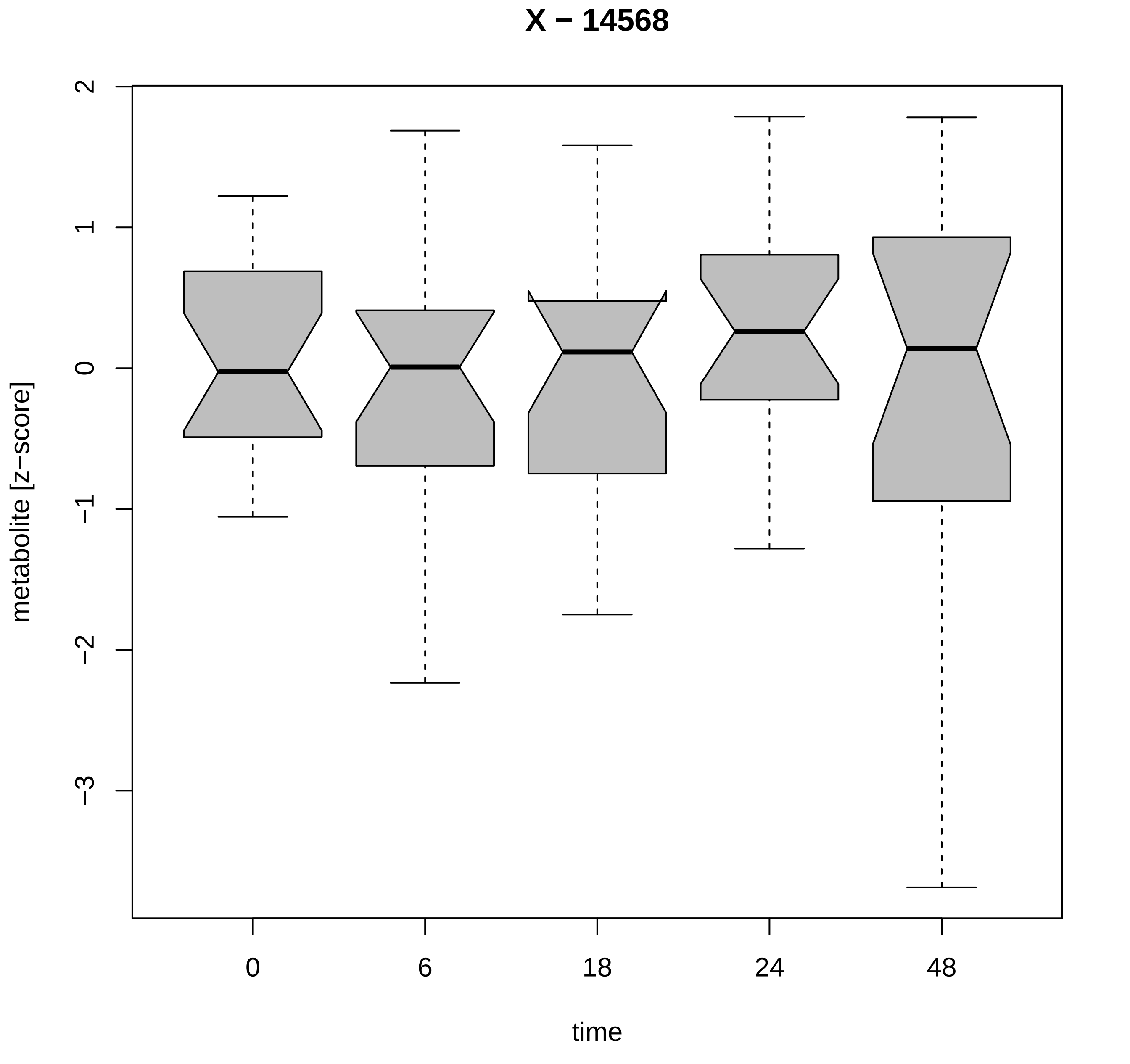

X − 14568
metabolite [z−score]
2
1
0
−1
−2
−3
0
6
18
24
48
time

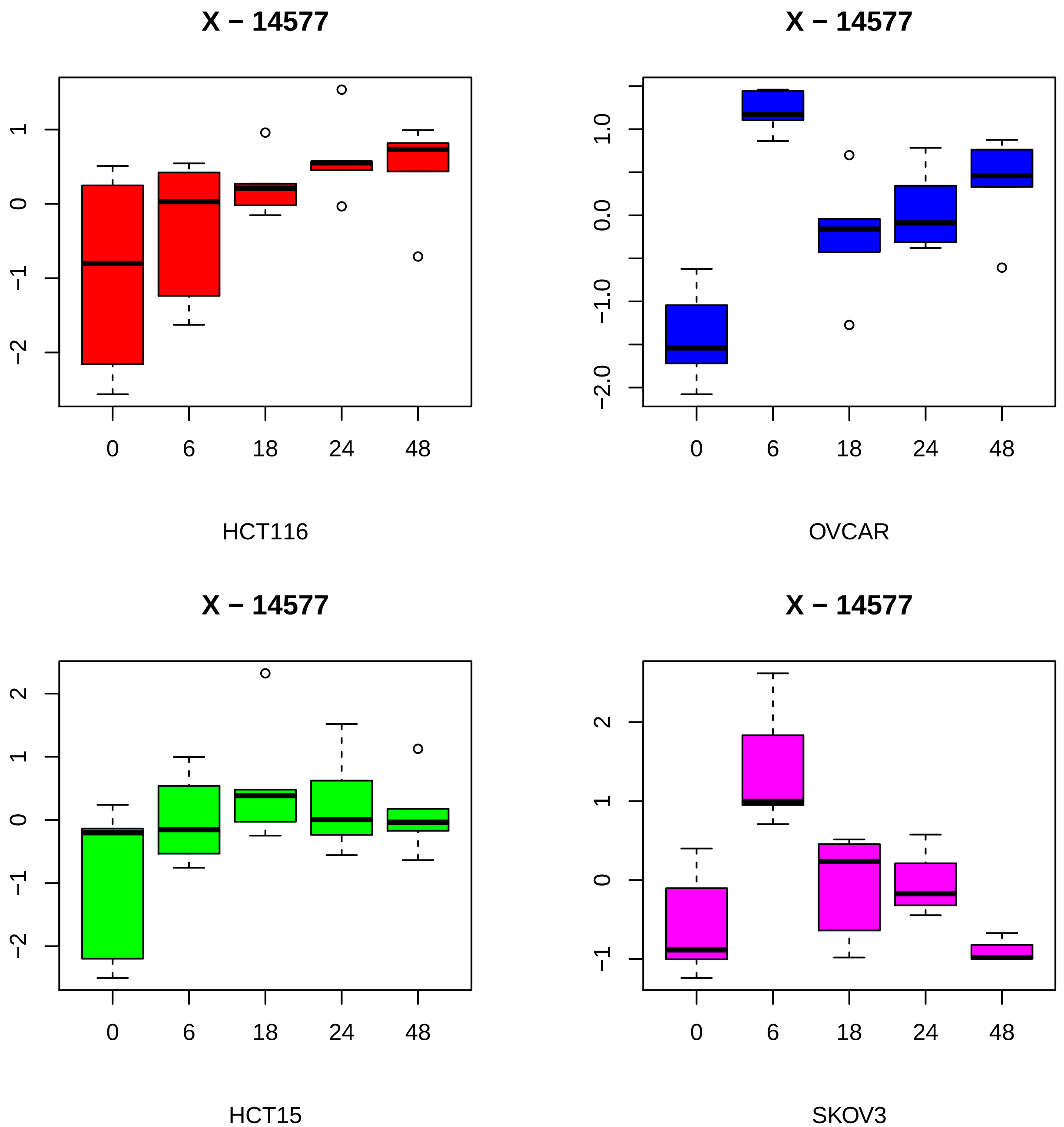
X − 14577
1
0
−1
−2
0
6
18
24
48
HCT116
X − 14577
1.0
0.0
−1.0
−2.0
0
6
18
24
48
OVCAR
X − 14577
2
1
0
−1
−2
0
6
18
24
48
HCT15
X − 14577
2
1
0
−1
0
6
18
24
48
SKOV3

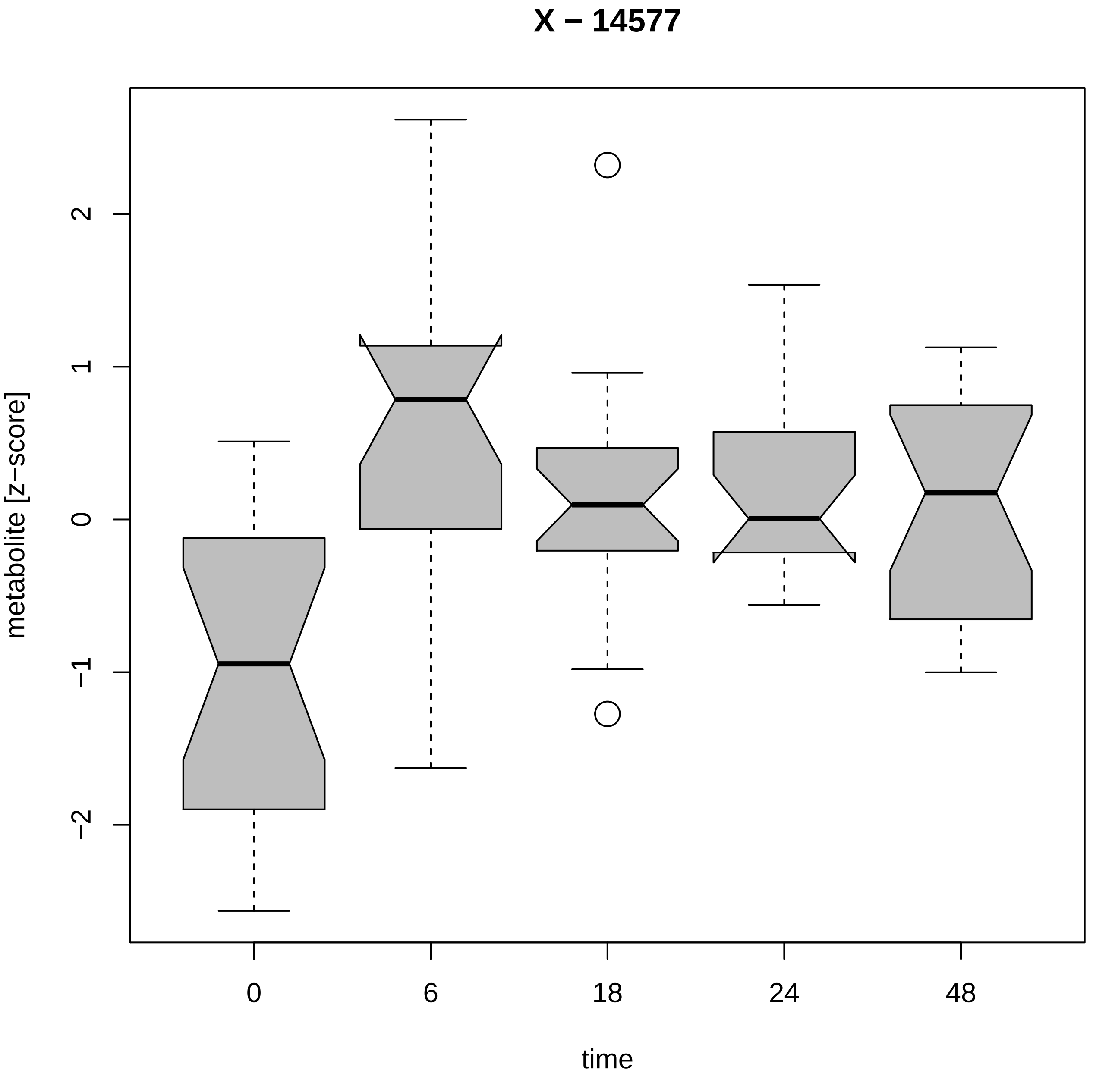

X – 14577
metabolite [z–score]
2
1
0
–1
–2
0
6
18
24
48
time

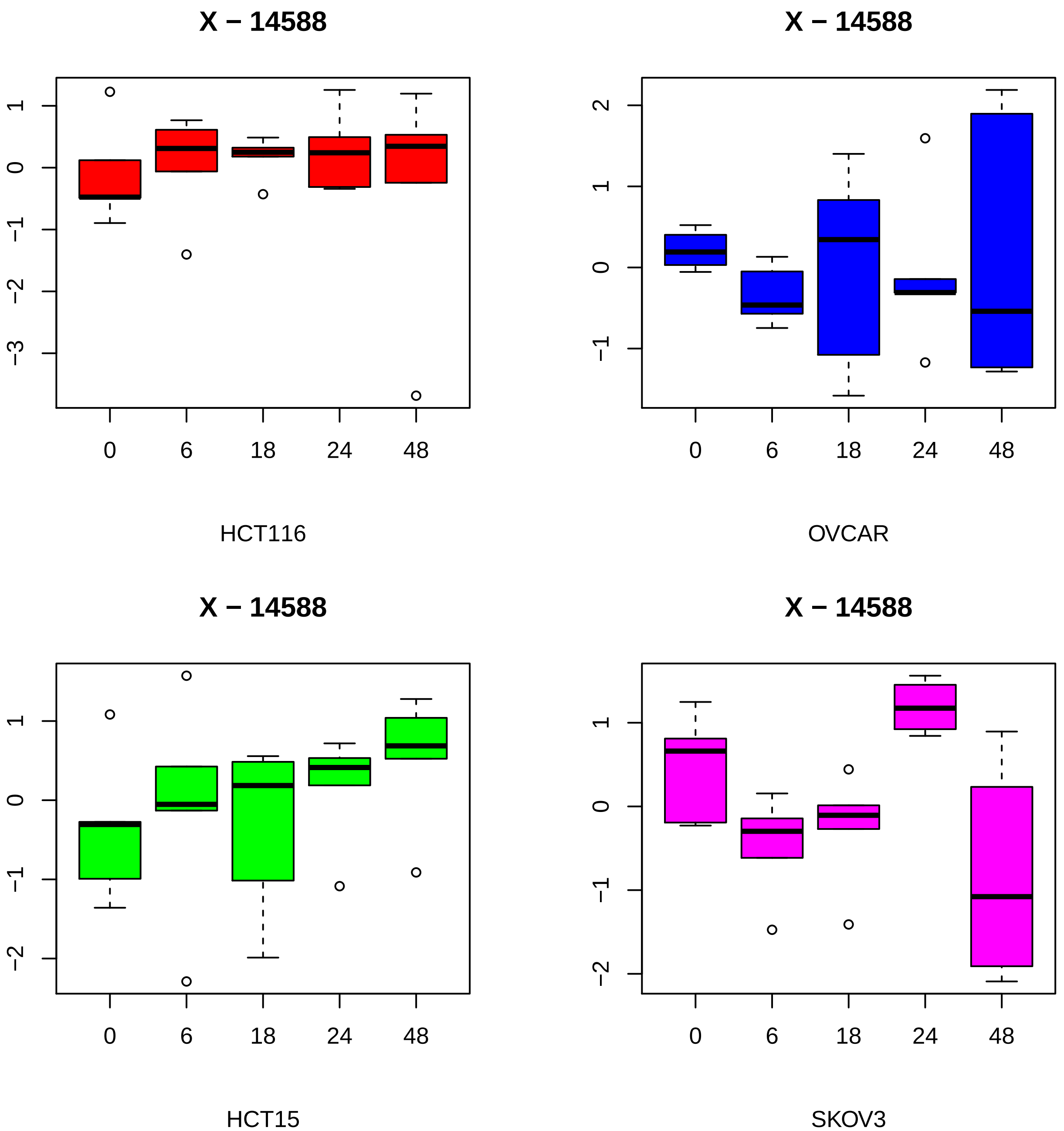
X − 14588
1
0
−1
−2
−3
0
6
18
24
48
HCT116
X − 14588
2
1
0
−1
0
6
18
24
48
OVCAR
X − 14588
1
0
−1
−2
0
6
18
24
48
HCT15
X − 14588
1
0
−1
−2
0
6
18
24
48
SKOV3

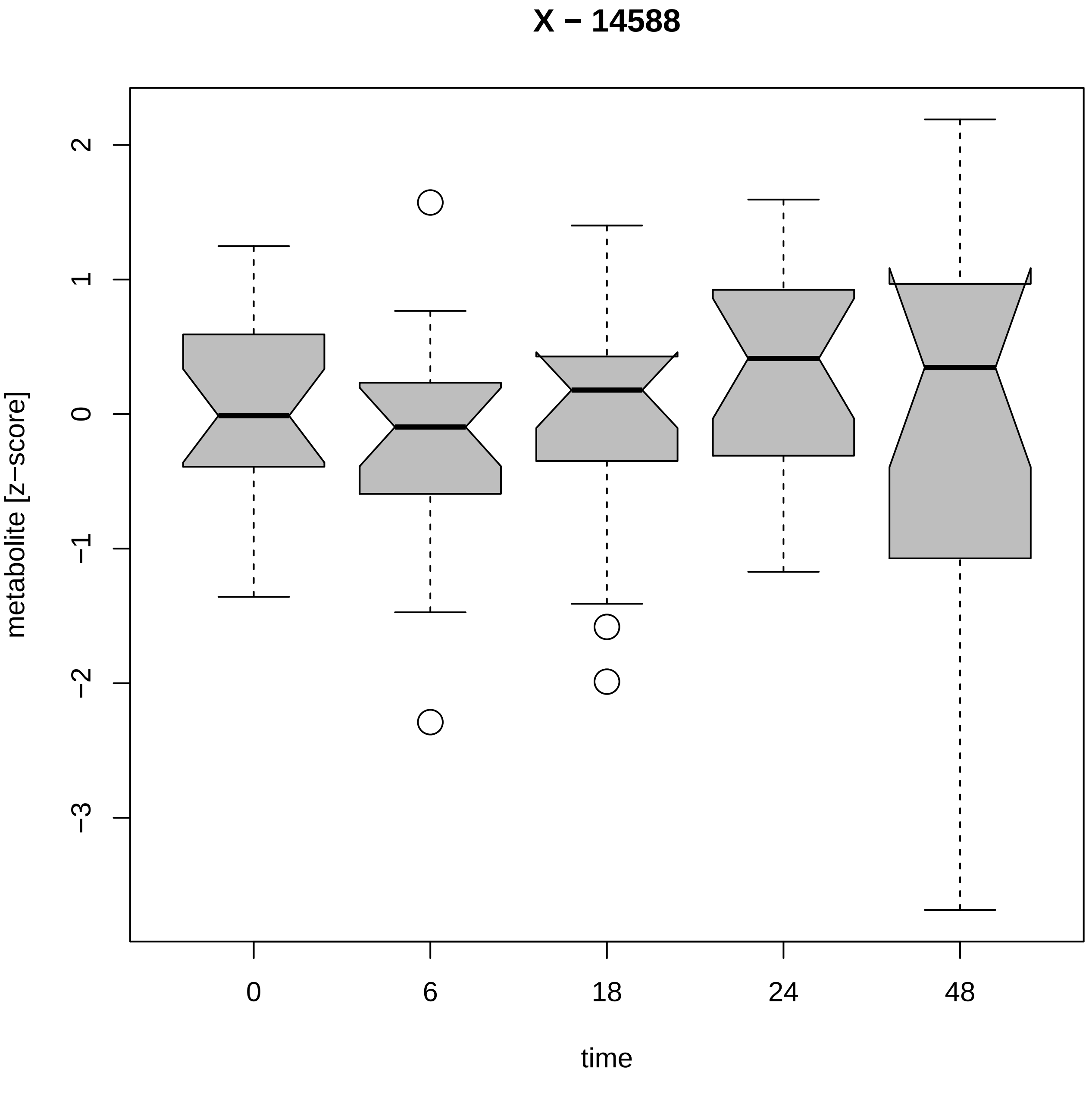
X − 14588
metabolite [z−score]
2
1
0
−1
−2
−3
0
6
18
24
48
time

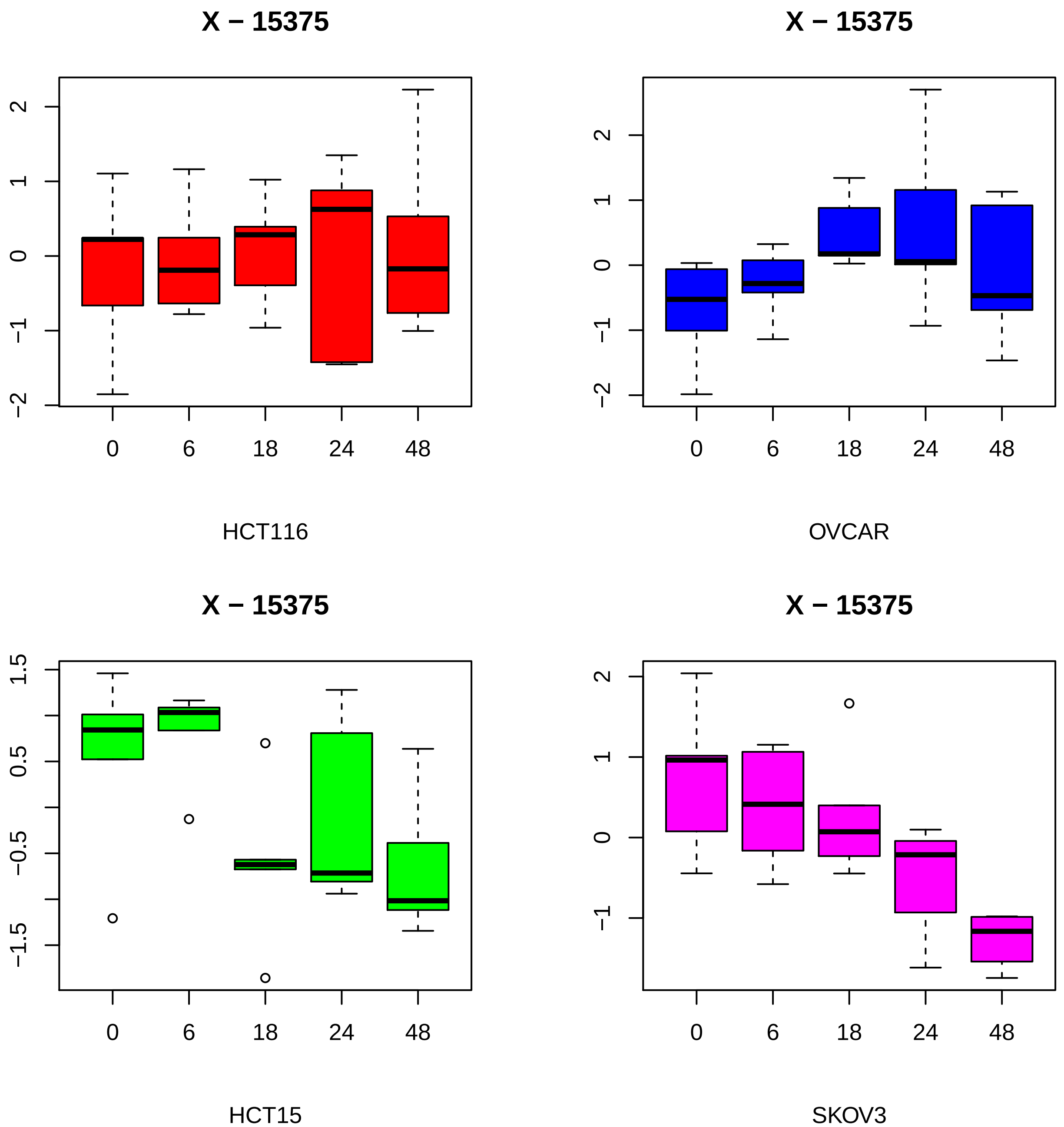
X − 15375
2
1
0
−1
−2
0
6
18
24
48
HCT116
X − 15375
2
1
0
−1
−2
0
6
18
24
48
OVCAR
X − 15375
1.5
0.5
−0.5
−1.5
0
6
18
24
48
HCT15
X − 15375
2
1
0
−1
0
6
18
24
48
SKOV3

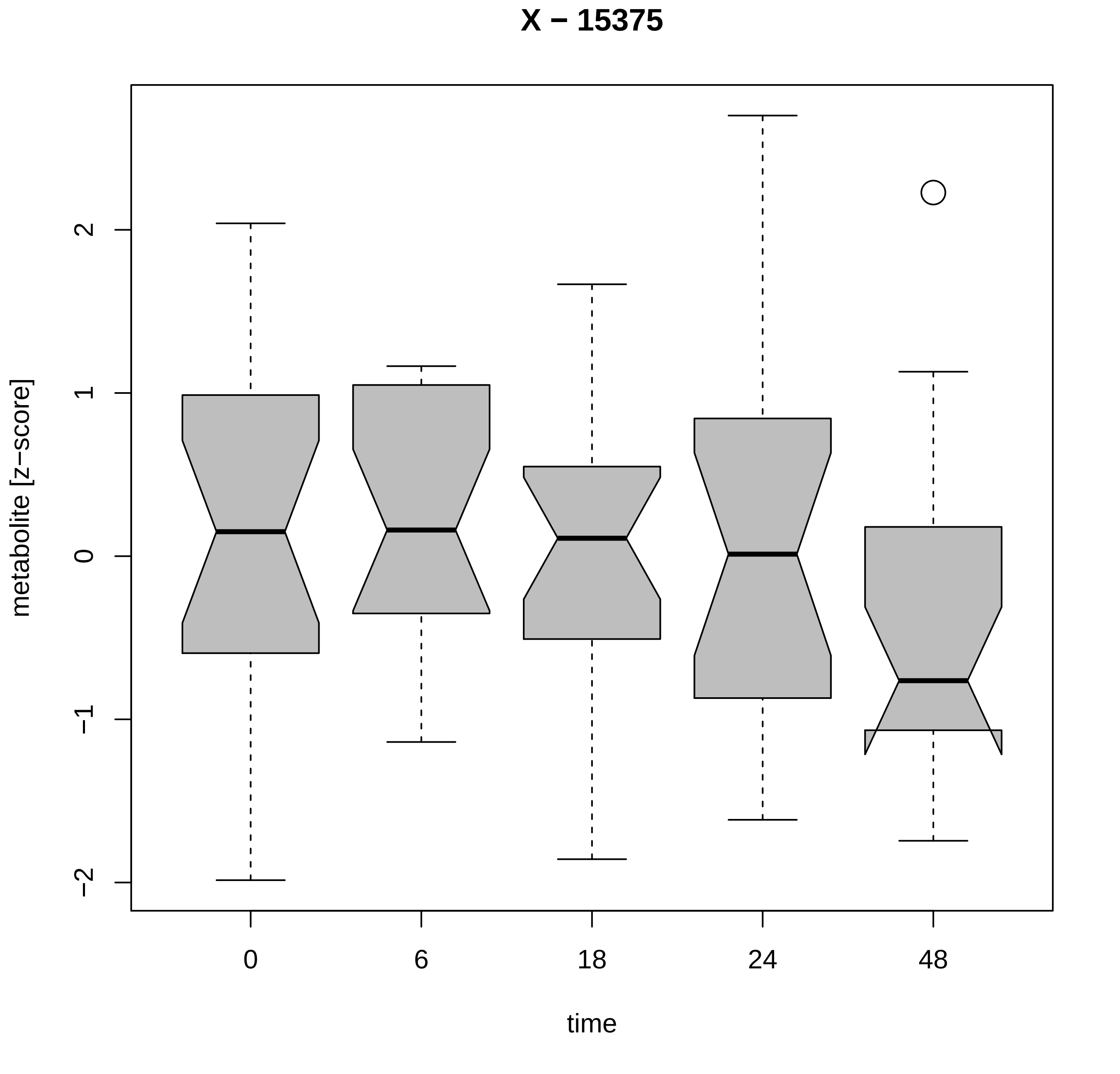
X − 15375
metabolite [z−score]
−2
−1
0
1
2
0
6
18
24
48
time

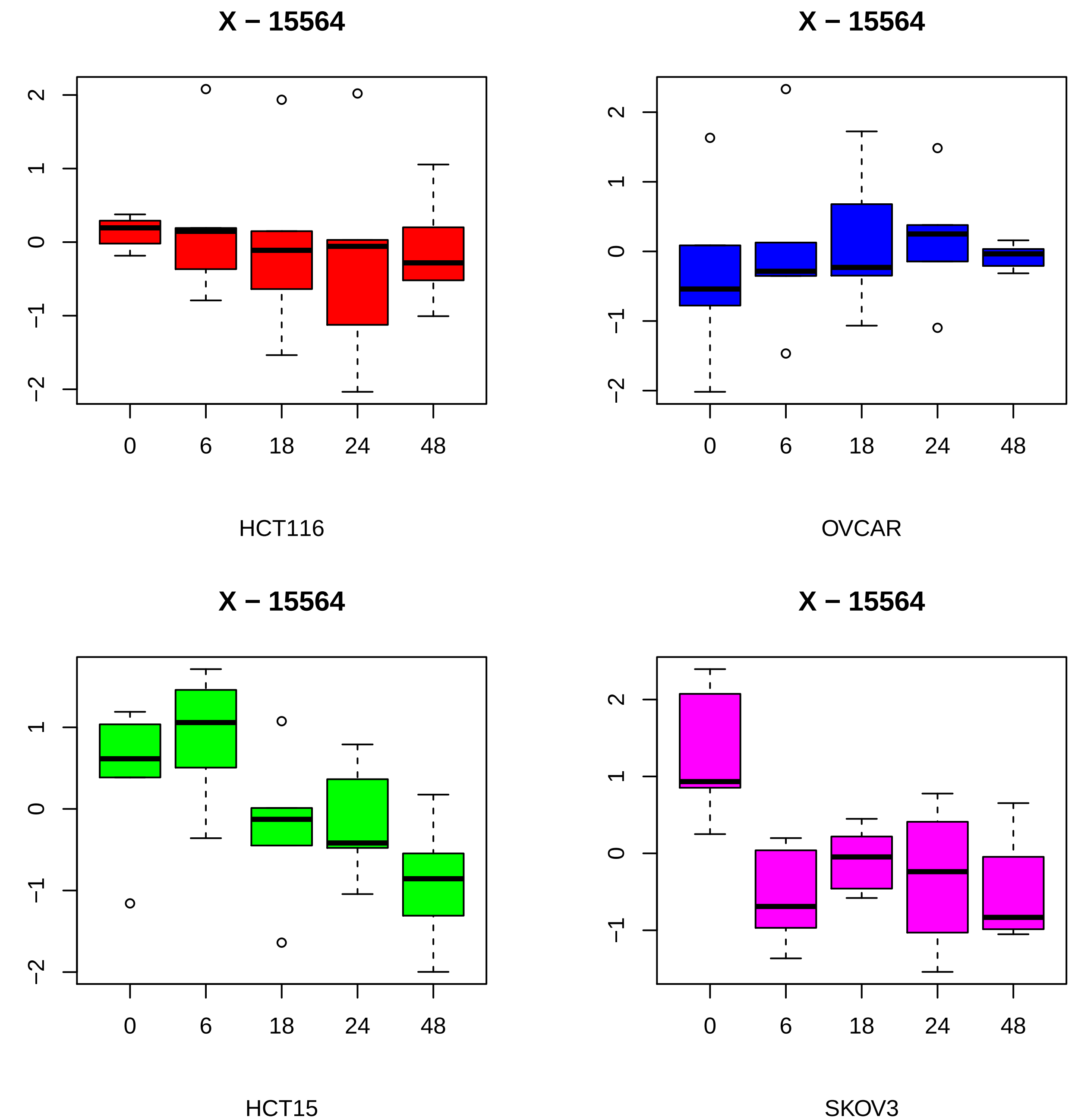
X − 15564
2
1
0
−1
−2
0
6
18
24
48
HCT116
X − 15564
2
1
0
−1
−2
0
6
18
24
48
OVCAR
X − 15564
1
0
−1
−2
0
6
18
24
48
HCT15
X − 15564
2
1
0
−1
0
6
18
24
48
SKOV3

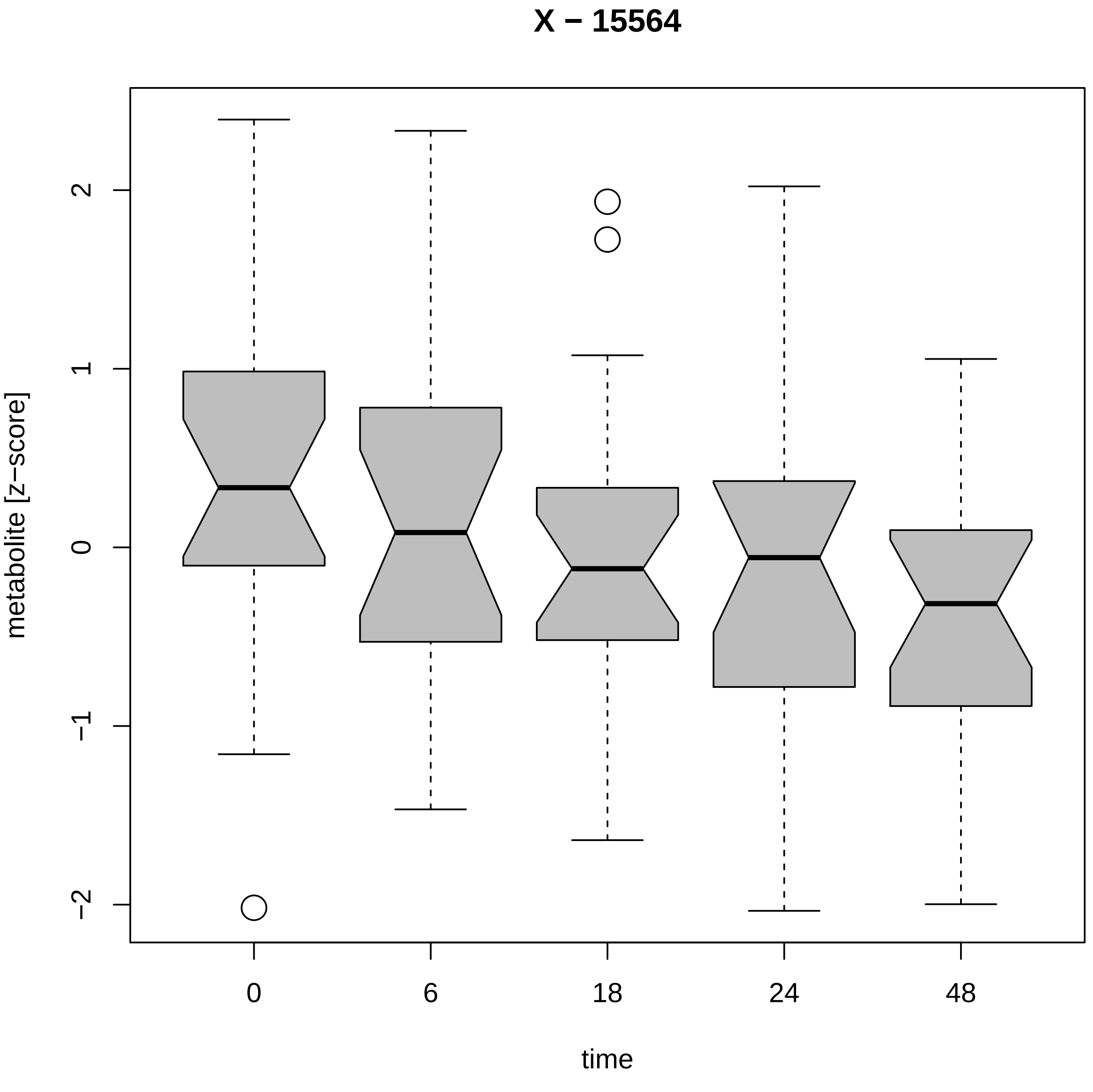
X − 15564
metabolite [z−score]
2
1
0
−1
−2
0
6
18
24
48
time
