# Supplementary material for: Nesting of colon and ovarian cancer cells in the endothelial niche is associated with alterations in glycan and lipid metabolism
Source: Sci Rep. 2017 Jan 4;7:39999. doi: 10.1038/srep39999 (PMC5209689; doi:10.1038/srep39999)
Supplement: Supplementary Figure 3 [file srep39999-s6.pdf]

Nesting of colon and ovarian cancer cells in the endothelial niche is associated with alterations in glycan and lipid metabolism.

Anna Halama[1†], Bella S Guerrouahen[2,3,4†], Jennifer Pasquier[2,3], Noothan J. Satheesh[1†], Karsten Suhre [1,5*] and Arash Rafii [2,3,6*].

†Contributed equally
1. Department of Physiology and Biophysics, Weill Cornell Medicine-Qatar, Qatar-Foundation, P.O. Box 24144, Doha, Qatar.
2. Stem Cell and Microenvironment Laboratory, Weill Cornell Medicine-Qatar, Education City, Qatar Foundation, Doha, Qatar.
3. Department of Genetic Medicine, Weill Cornell Medicine-Qatar, New York, NY 10065, USA.
4. Translational Medicine Division-Research Department, Sidra Medical and Research Center, PO Box 26999, Doha, Qatar.
5. Institute of Bioinformatics and Systems Biology, Helmholtz Zentrum München, German Research Center for Environmental Health, Neuherberg, Germany.
6. Department of Genetic Medicine and Obstetrics and Gynecology, Weill Cornell Medical College, Stem Cell and Microenvironment Laboratory, Weill Cornell Medical College in Qatar, Qatar-Foundation, P.O. Box 24144, Doha, Qatar.

Corresponding authors:
Dr. Karsten Suhre: Department of Physiology and Biophysics, Weill Cornell Medicine-Qatar, Doha, Qatar Weill Cornell Medical College in Qatar, Qatar-Foundation, P.O Box: 24144, Doha, Qatar. Phone: +974 4492 8482. Fax: +974 4492 8422. Email: kas2049@qatar-med.cornell.edu

Dr. Arash Rafii: Department of Genetic Medicine and Obstetrics and Gynecology, Weill Cornell Medicine-Qatar, Stem cell and microenvironment laboratory Weill Cornell Medical College in Qatar, Qatar-Foundation, P.O Box: 24144, Doha, Qatar. Phone: +974 3313 5828. Fax: +974 4492 8422. Email: jat2021@qatar-med.cornell.edu

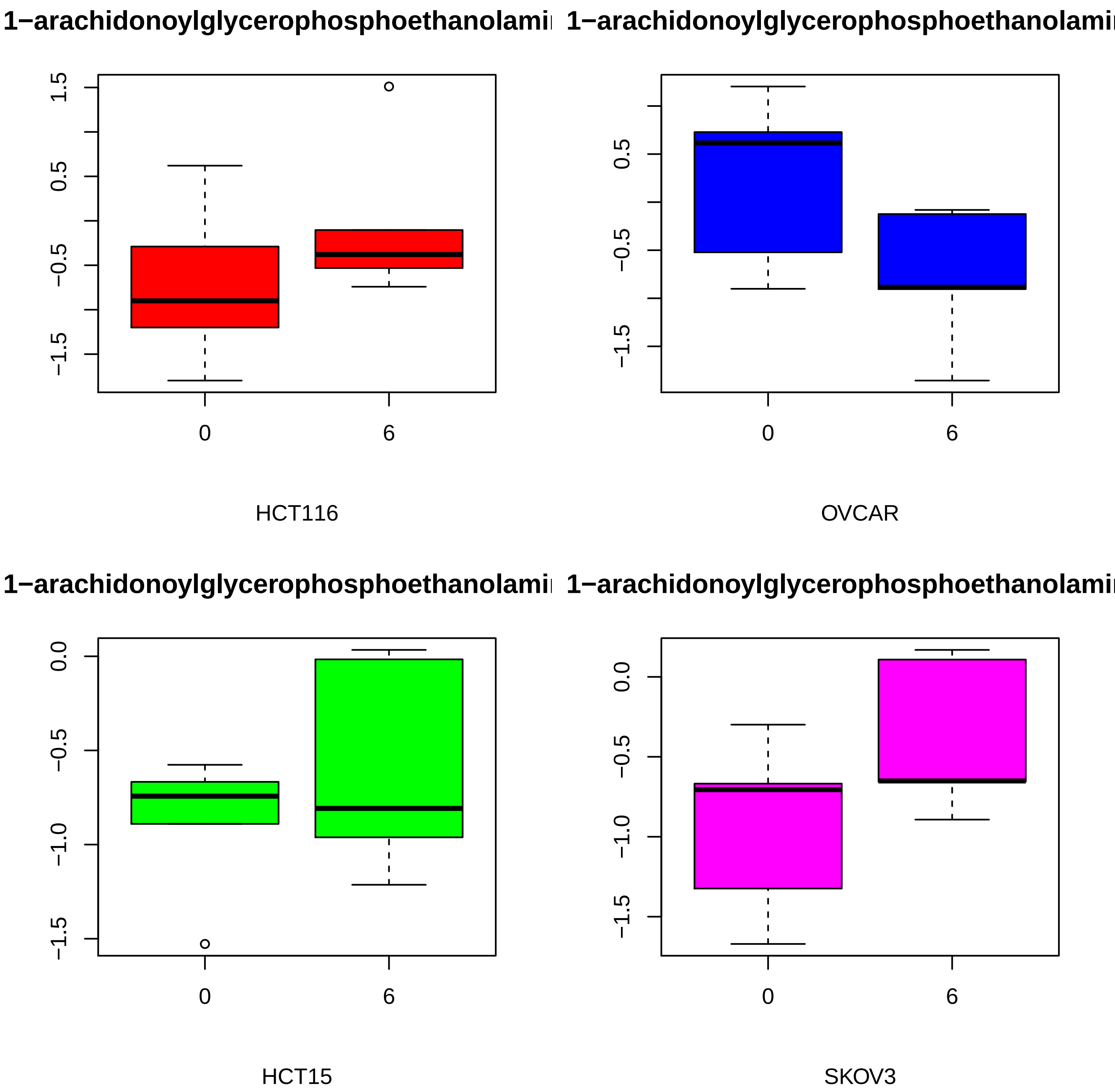
1−arachidonoylglycerophosphoethanolami
1.5
0.5
−0.5
−1.5
0
6
HCT116
1−arachidonoylglycerophosphoethanolami
0.5
−0.5
−1.5
0
6
OVCAR
1−arachidonoylglycerophosphoethanolami
0.0
−0.5
−1.0
−1.5
0
6
HCT15
1−arachidonoylglycerophosphoethanolami
0.0
−0.5
−1.0
−1.5
0
6
SKOV3

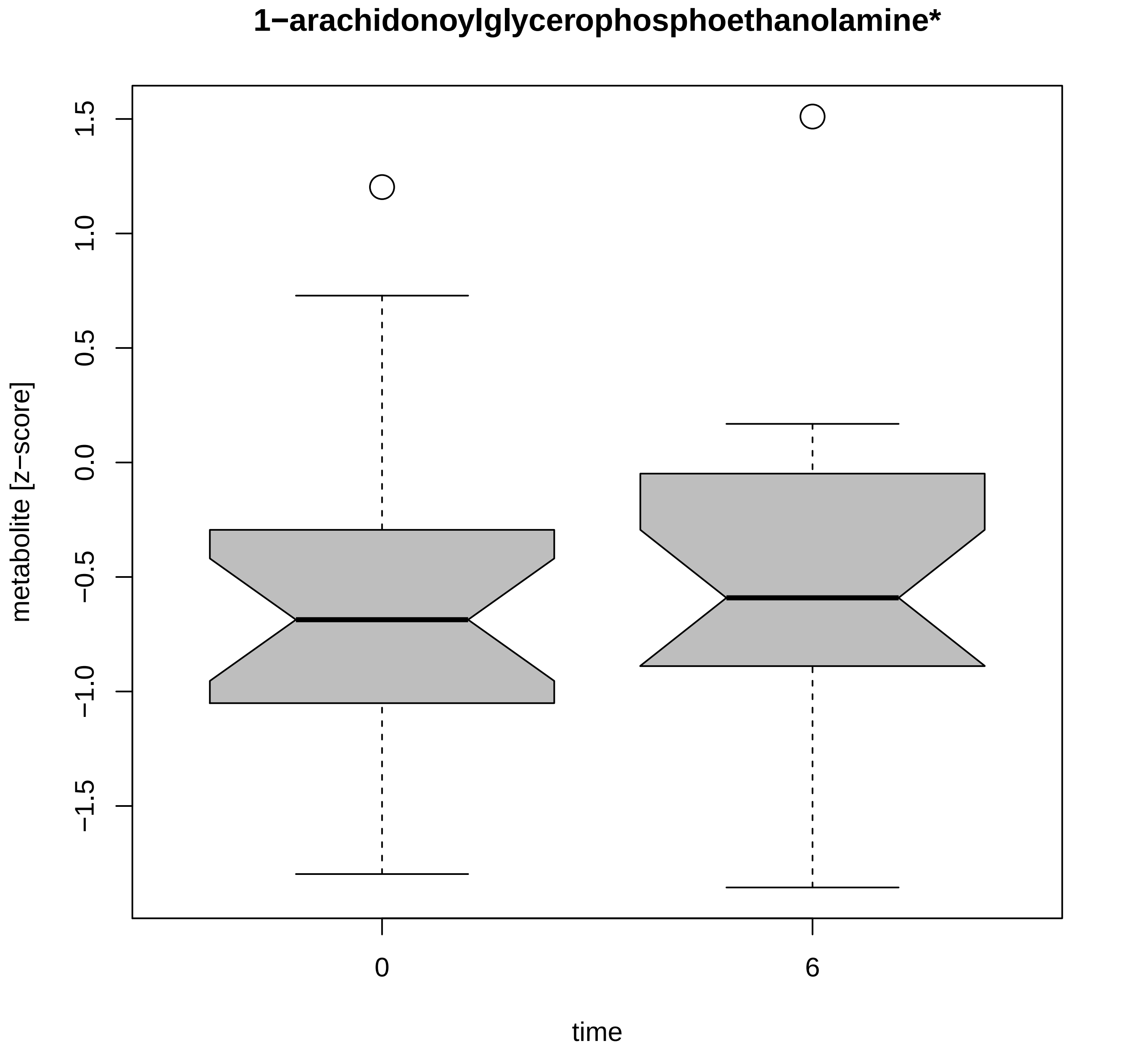
1−arachidonoylglycerophosphoethanolamine*
metabolite [z−score]
1.5
1.0
0.5
0.0
−0.5
−1.0
−1.5
0
6
time

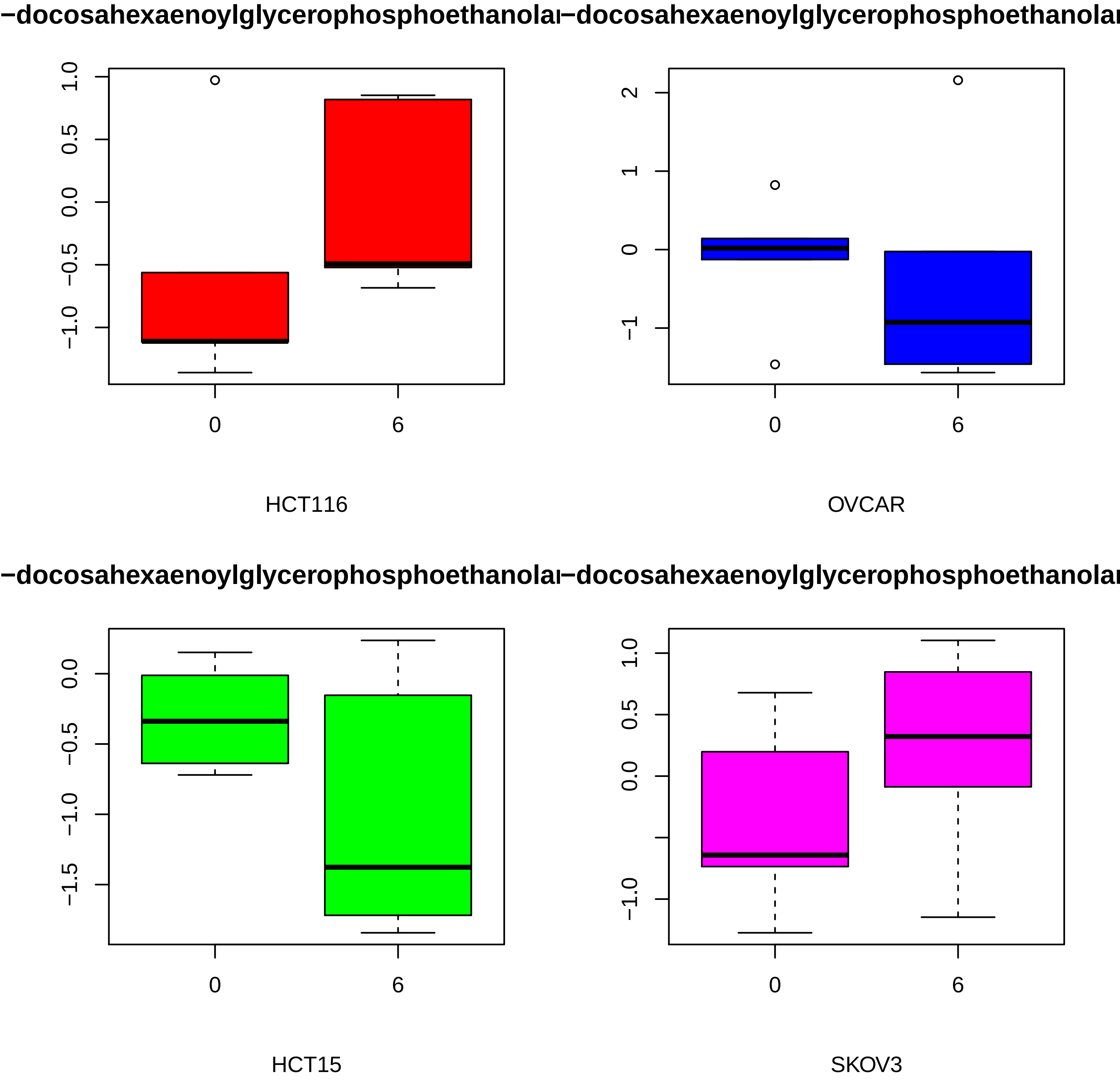
−docosahexaenoylglycerophosphoethanola
−docosahexaenoylglycerophosphoethanola
1.0
0.5
0.0
−0.5
−1.0
0
6
HCT116
2
1
0
−1
0
6
OVCAR
−docosahexaenoylglycerophosphoethanola
−docosahexaenoylglycerophosphoethanola
0.0
−0.5
−1.0
−1.5
0
6
HCT15
1.0
0.5
0.0
−1.0
0
6
SKOV3

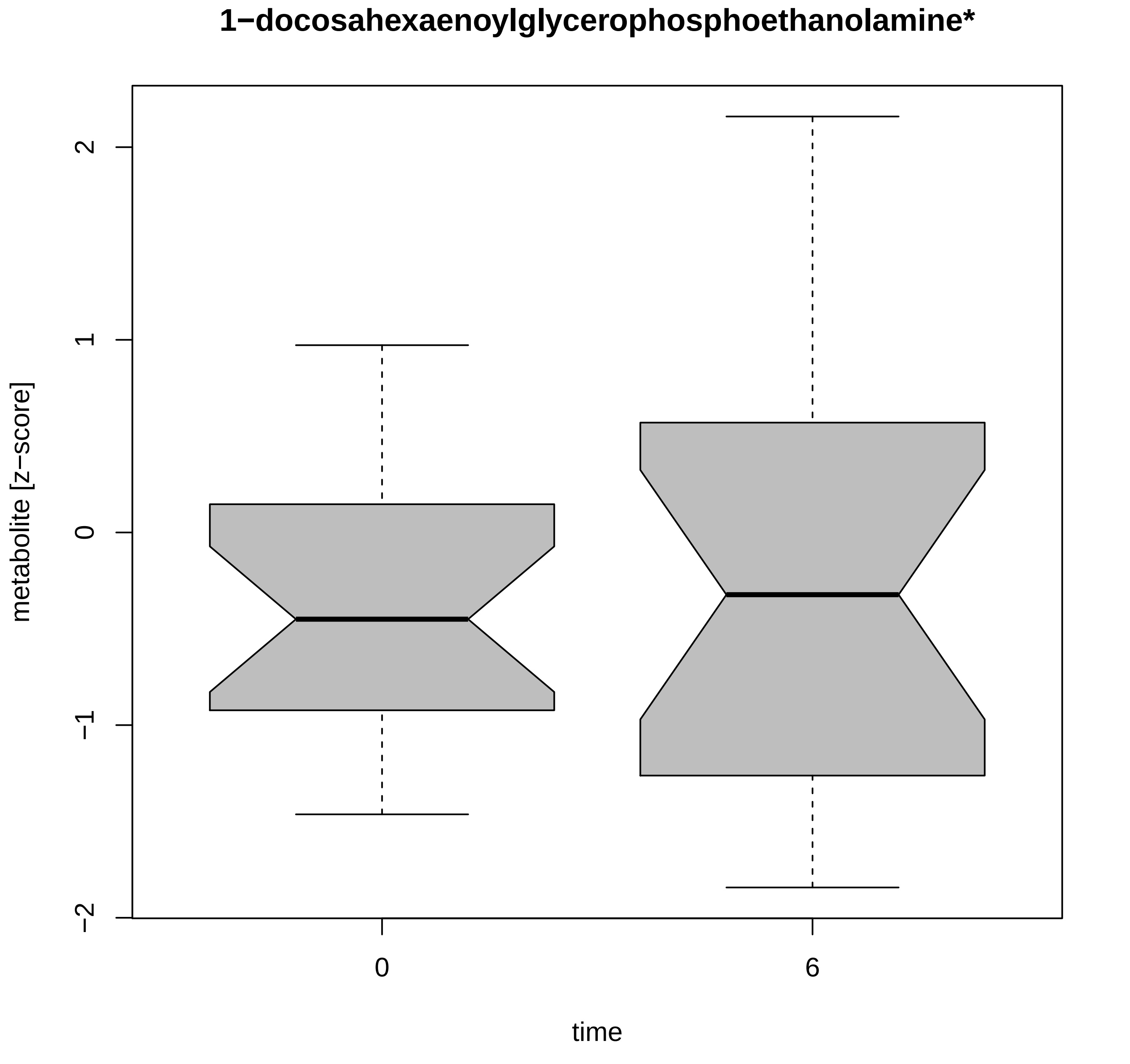
1−docosahexaenoylglycerophosphoethanolamine*
metabolite [z−score]
2
1
0
−1
−2
0
6
time

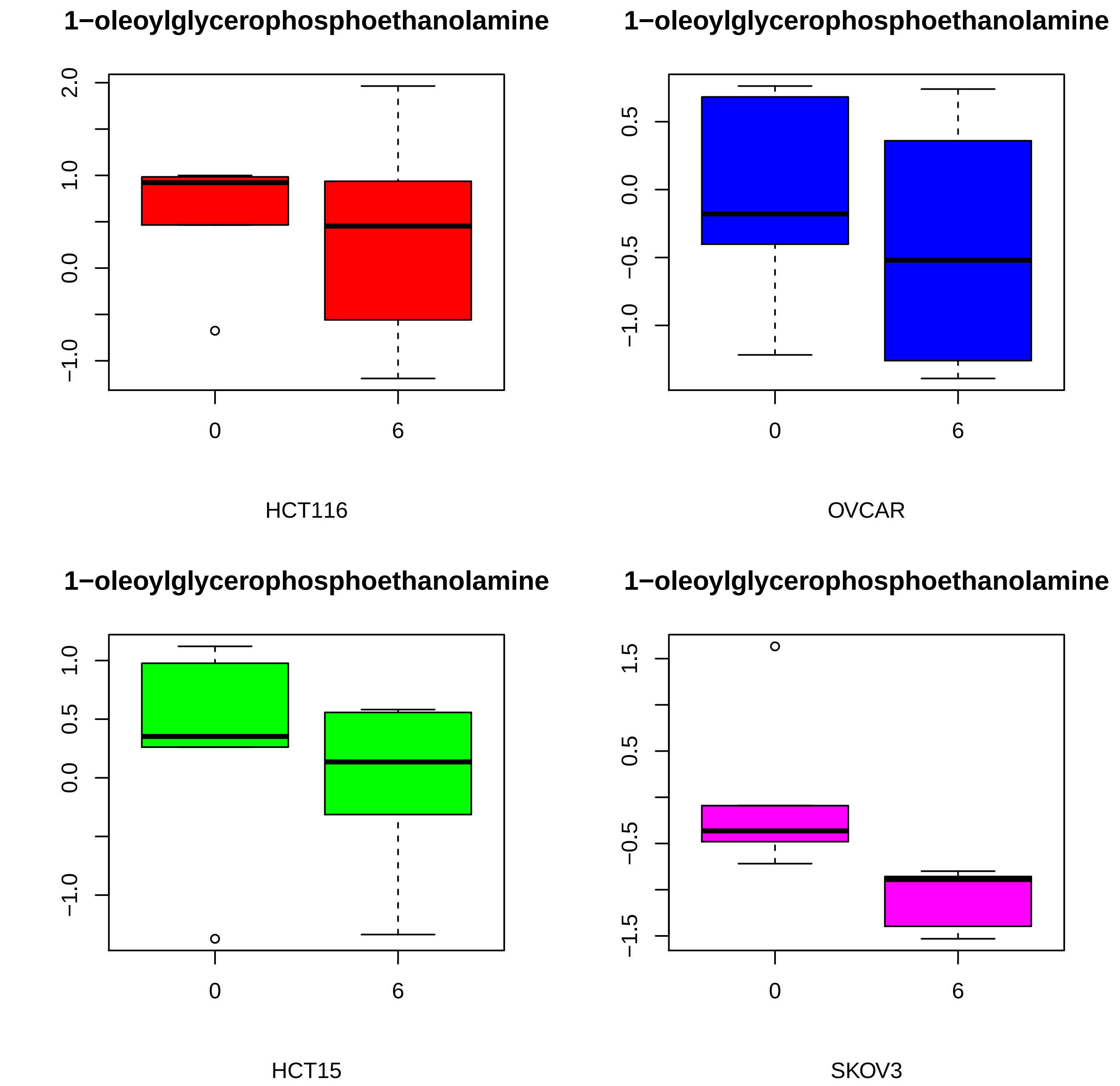
1−oleoylglycerophosphoethanolamine
2.0
1.0
0.0
−1.0
0
6
HCT116
1−oleoylglycerophosphoethanolamine
0.5
0.0
−0.5
−1.0
0
6
OVCAR
1−oleoylglycerophosphoethanolamine
1.0
0.5
0.0
−1.0
0
6
HCT15
1−oleoylglycerophosphoethanolamine
1.5
0.5
−0.5
−1.5
0
6
SKOV3

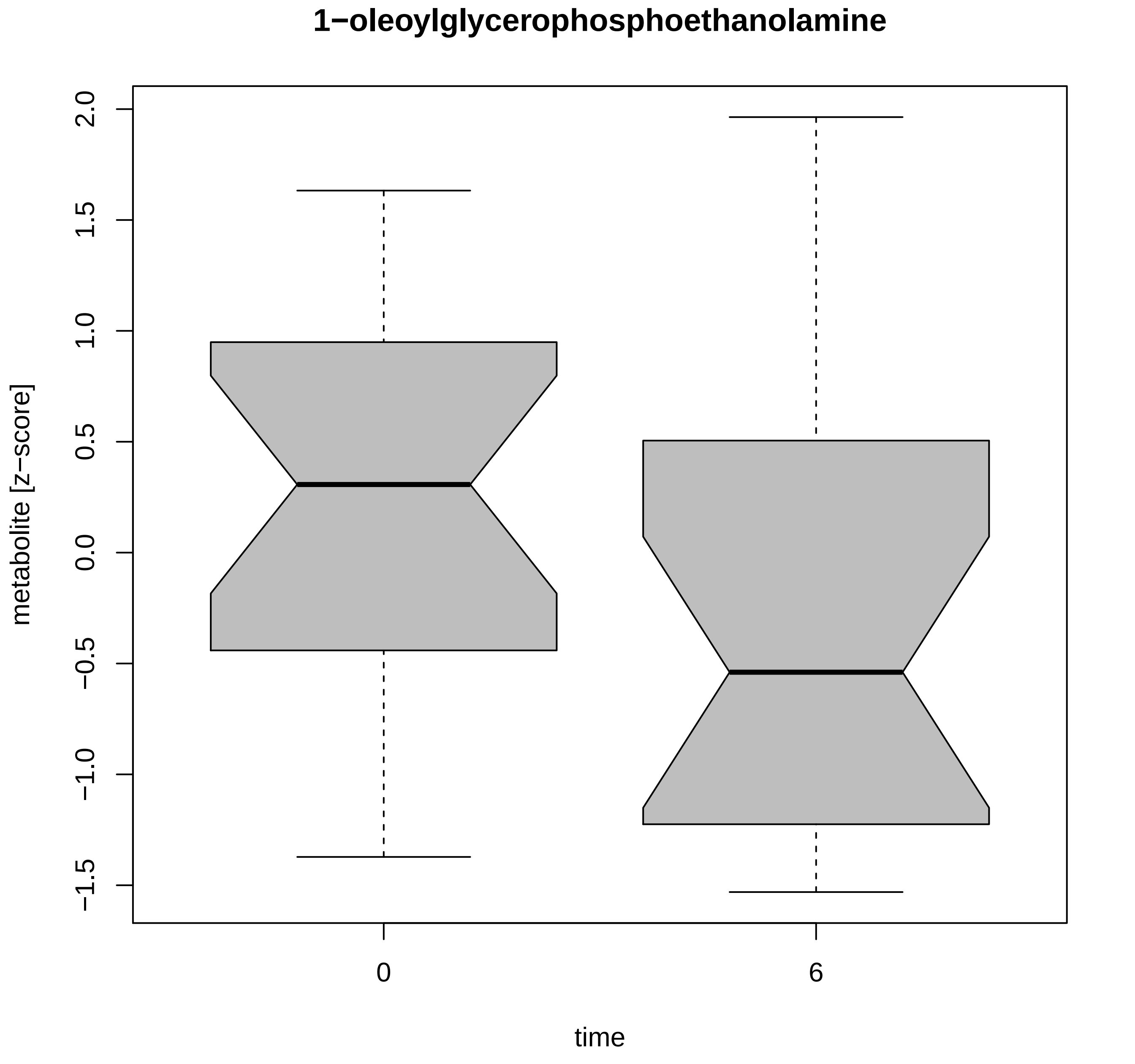
1−oleoylglycerophosphoethanolamine
metabolite [z−score]
2.0
1.5
1.0
0.5
0.0
−0.5
−1.0
−1.5
0
6
time

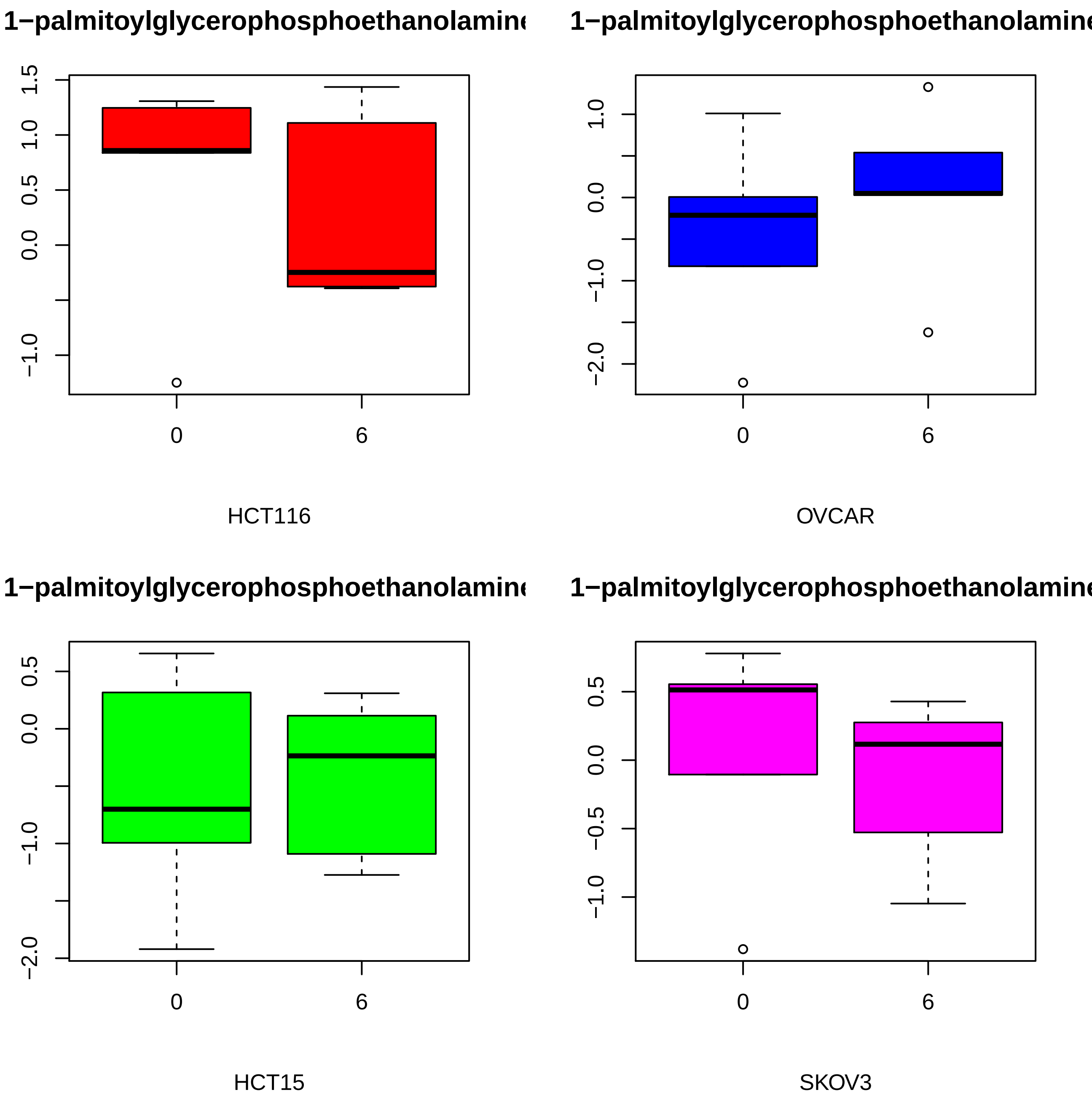

1−palmitoylglycerophosphoethanolamine
1.5
1.0
0.5
0.0
−1.0
0
6
HCT116
1−palmitoylglycerophosphoethanolamine
1.0
0.0
−1.0
−2.0
0
6
OVCAR
1−palmitoylglycerophosphoethanolamine
0.5
0.0
−1.0
−2.0
0
6
HCT15
1−palmitoylglycerophosphoethanolamine
0.5
0.0
−0.5
−1.0
0
6
SKOV3

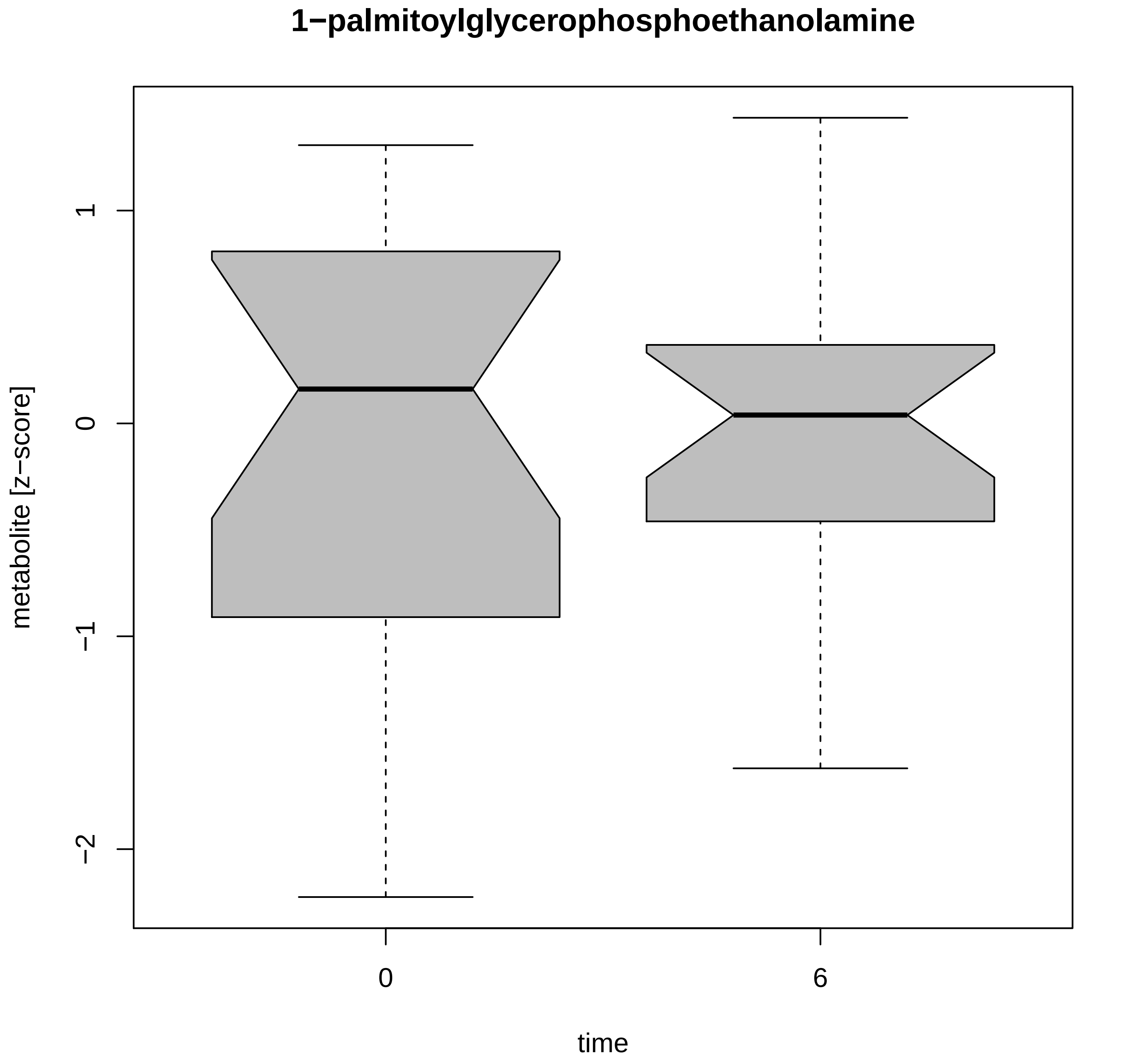
1−palmitoylglycerophosphoethanolamine
metabolite [z−score]
1
0
−1
−2
0
6
time

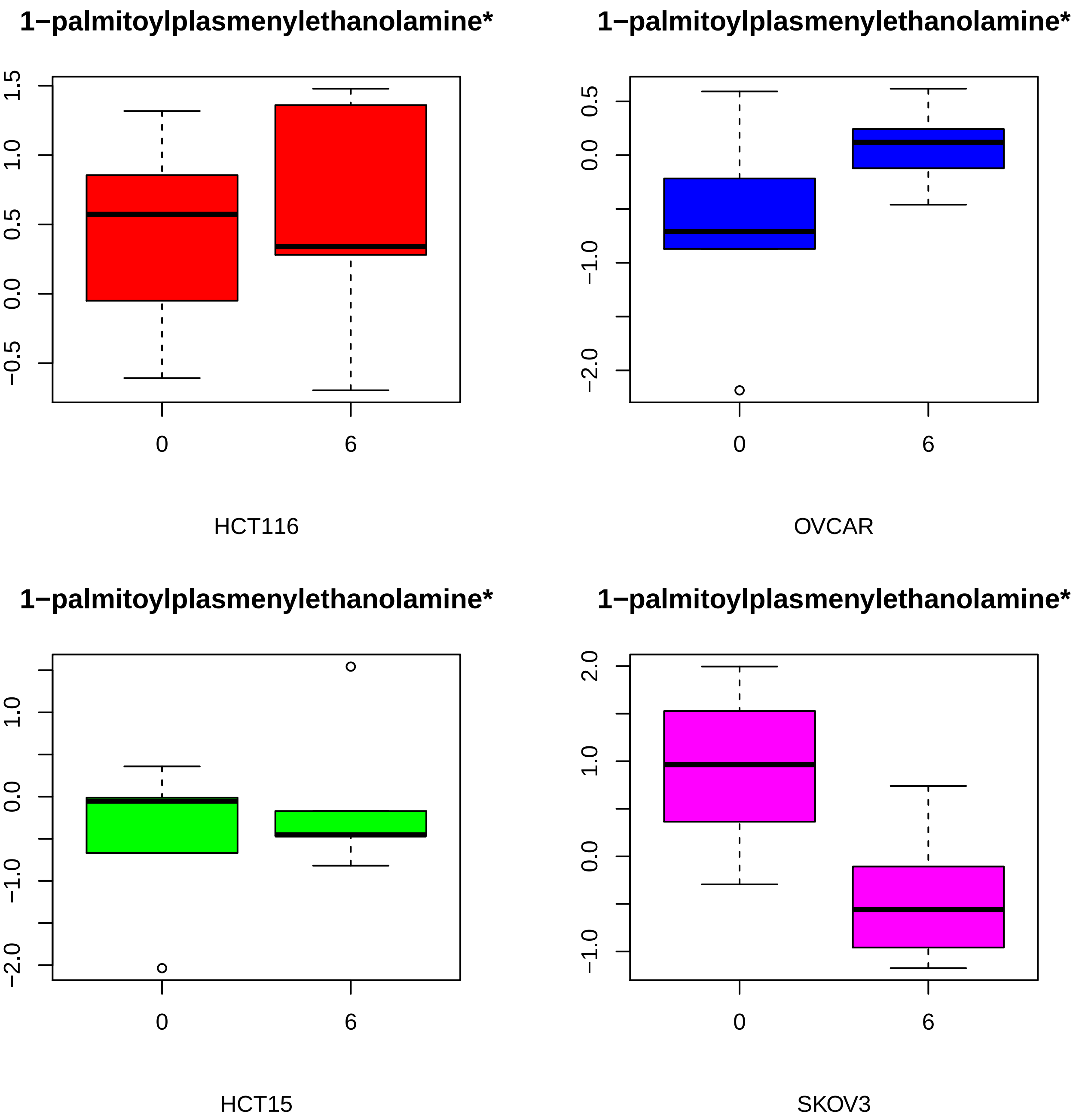
1−palmitoylplasmenylethanolamine*
1.5
1.0
0.5
0.0
−0.5
0
6
HCT116
1−palmitoylplasmenylethanolamine*
0.5
0.0
−1.0
−2.0
0
6
OVCAR
1−palmitoylplasmenylethanolamine*
1.0
0.0
−1.0
−2.0
0
6
HCT15
1−palmitoylplasmenylethanolamine*
2.0
1.0
0.0
−1.0
0
6
SKOV3

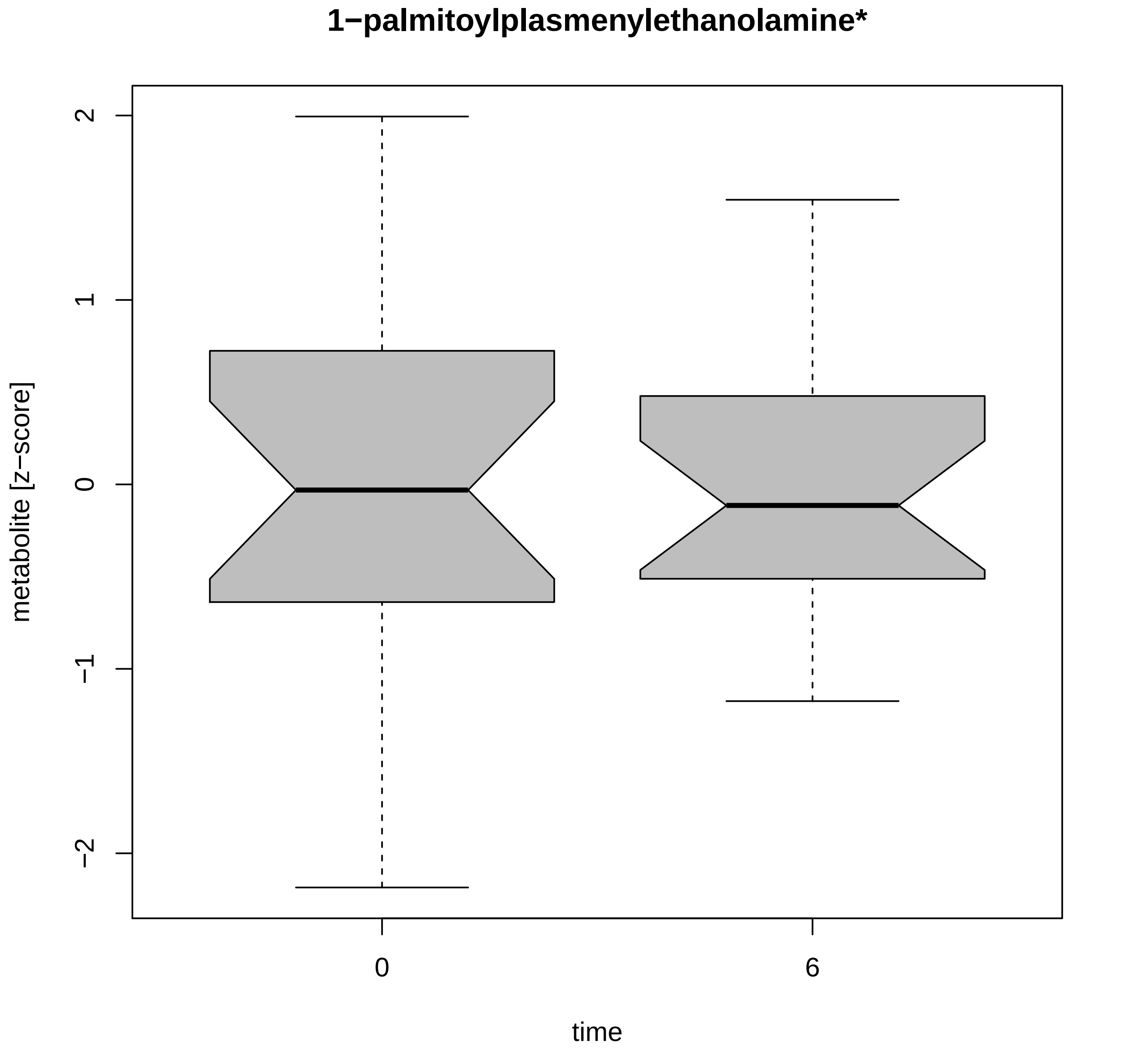
1−palmitoylplasmenylethanolamine*
metabolite [z−score]
2
1
0
−1
−2
0
6
time

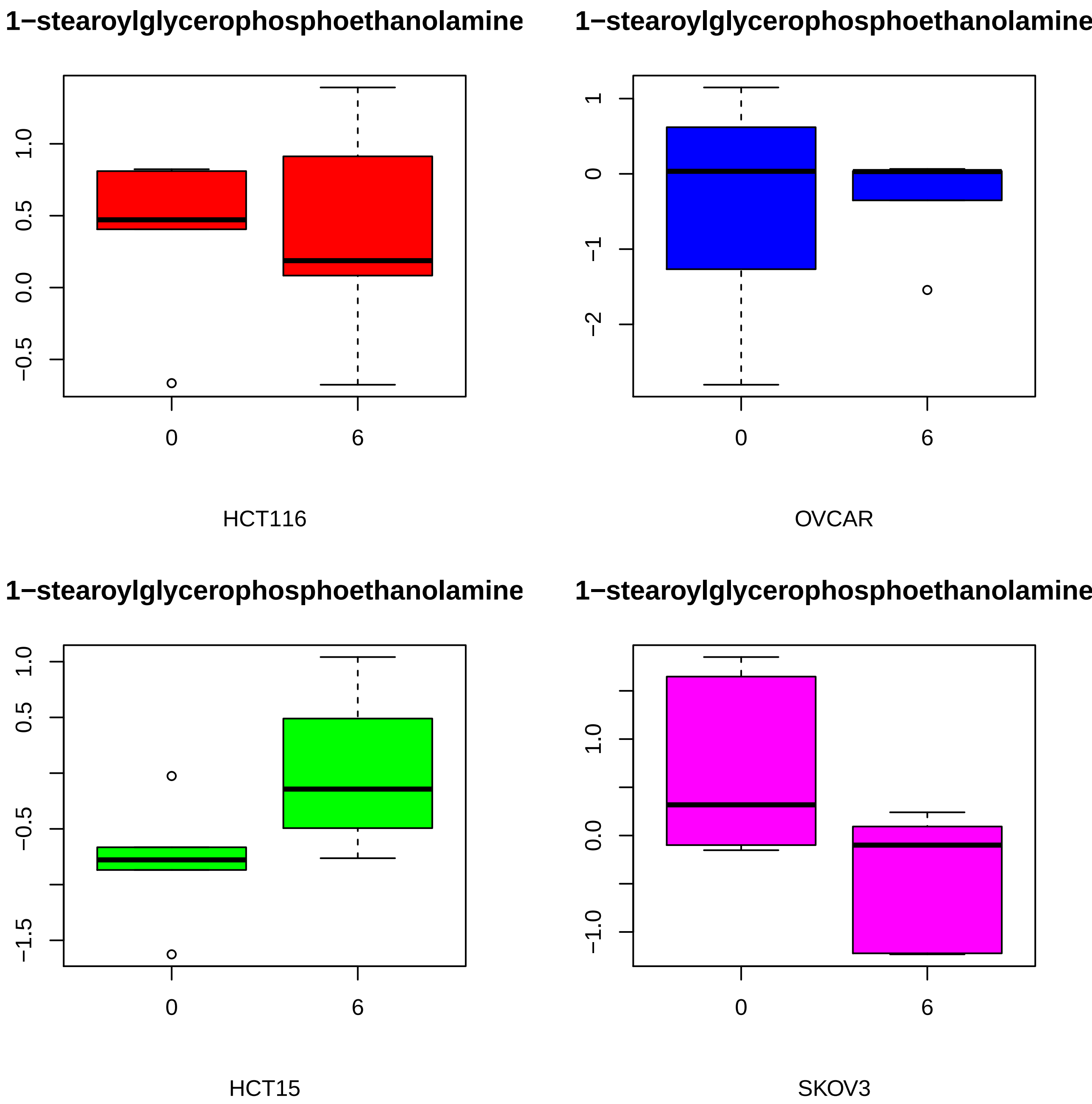
1−stearoylglycerophosphoethanolamine
1.0
0.5
0.0
−0.5
0
6
HCT116
1−stearoylglycerophosphoethanolamine
1
0
−1
−2
0
6
OVCAR
1−stearoylglycerophosphoethanolamine
1.0
0.5
−0.5
−1.5
0
6
HCT15
1−stearoylglycerophosphoethanolamine
1.0
0.0
−1.0
0
6
SKOV3

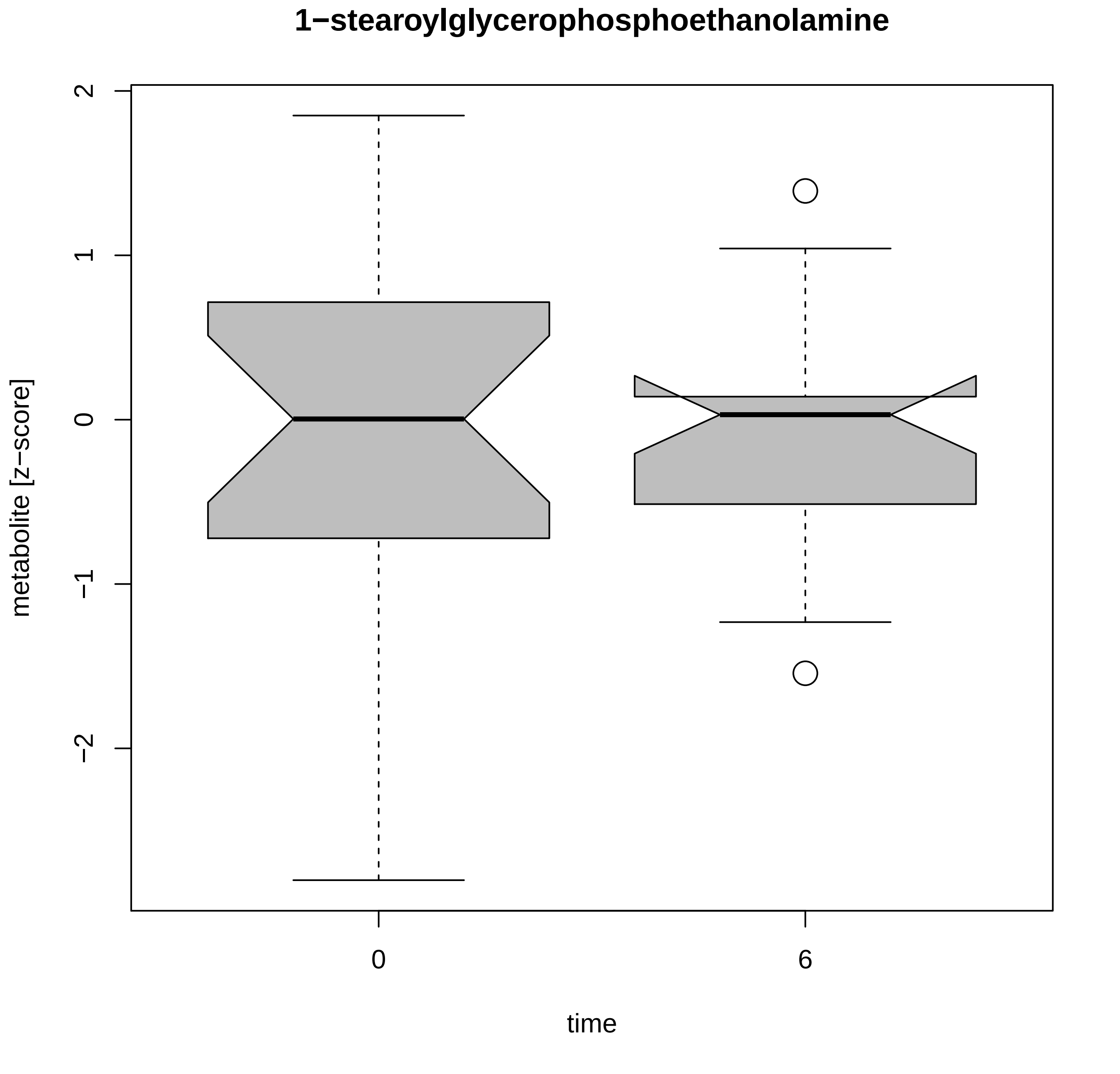
1−stearoylglycerophosphoethanolamine
metabolite [z−score]
2
1
0
−1
−2
0
6
time

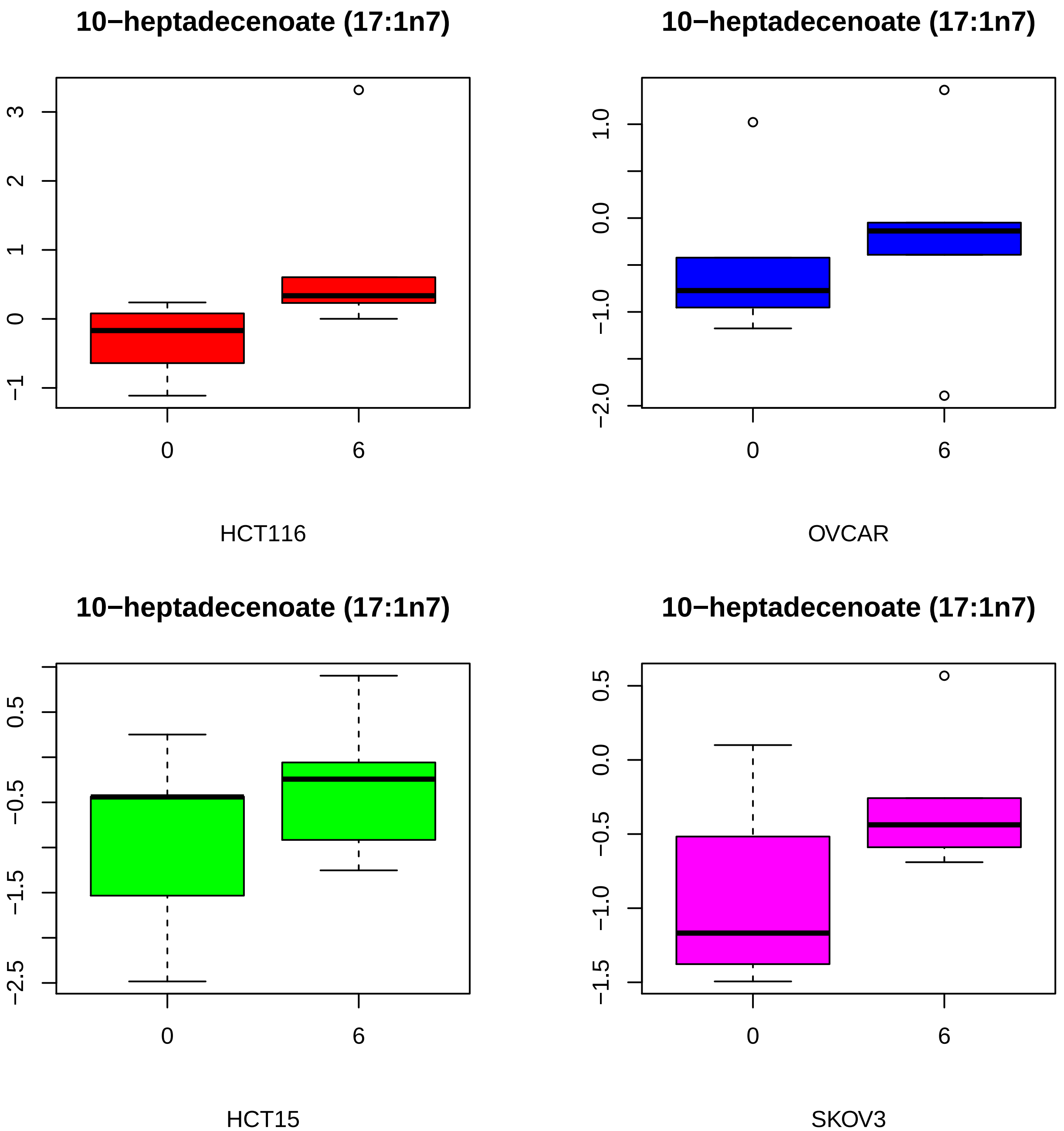
10−heptadecenoate (17:1n7)
HCT116
10−heptadecenoate (17:1n7)
OVCAR
10−heptadecenoate (17:1n7)
HCT15
10−heptadecenoate (17:1n7)
SKOV3
0
6

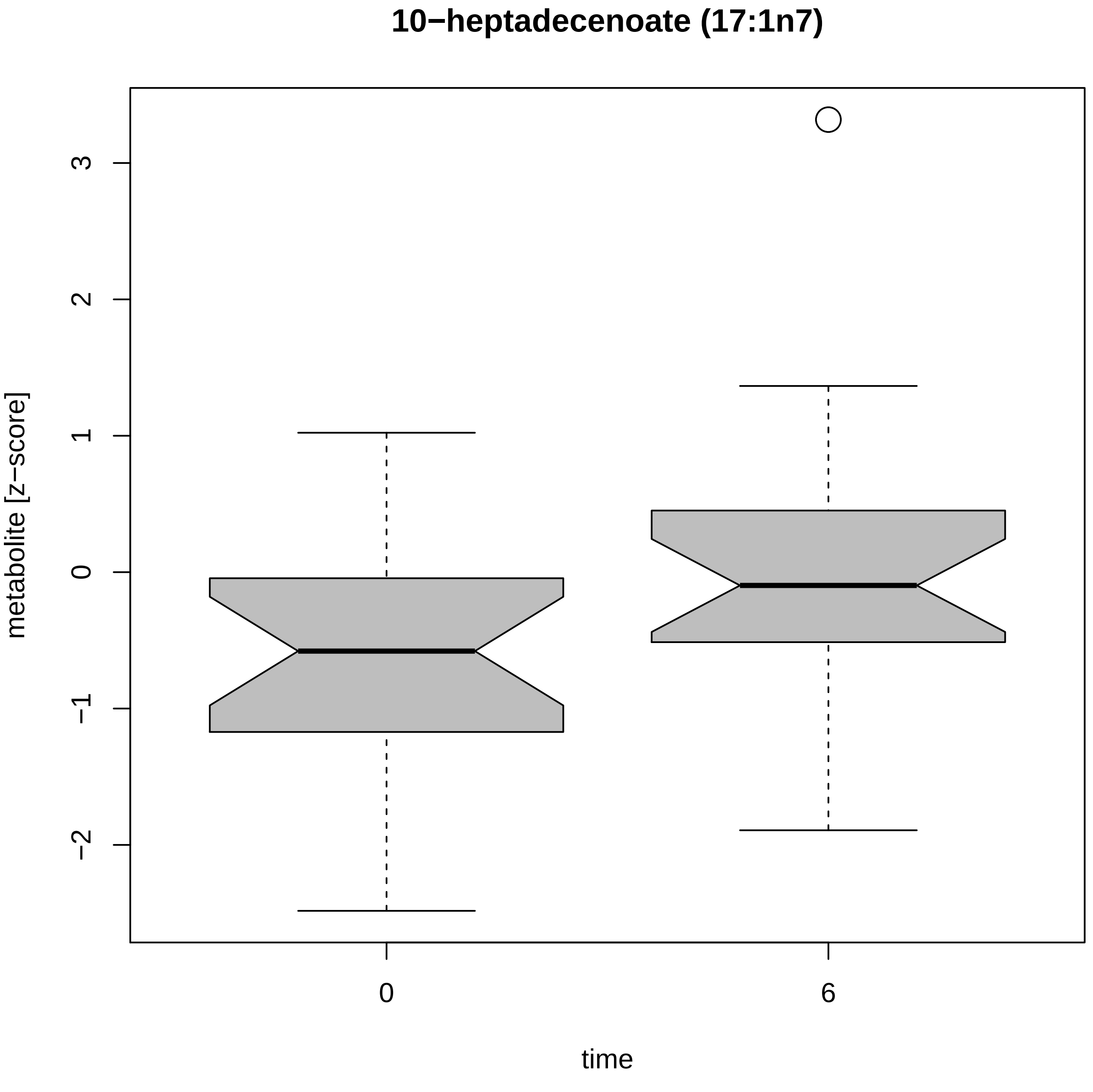
10−heptadecenoate (17:1n7)
metabolite [z−score]
3
2
1
0
−1
−2
0
6
time

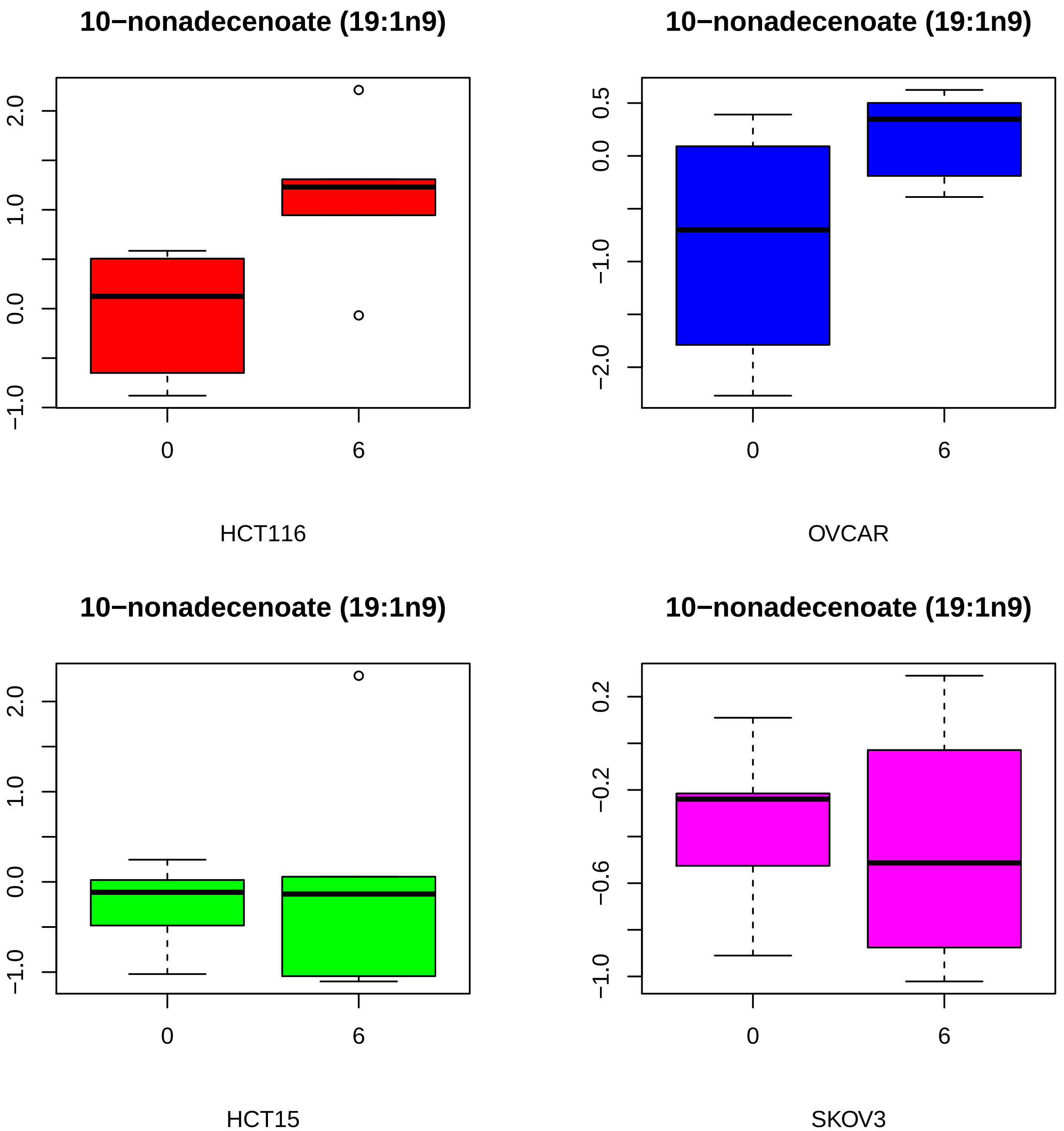
10−nonadecenoate (19:1n9)
−1.0
0.0
1.0
2.0
0
6
HCT116
10−nonadecenoate (19:1n9)
−2.0
−1.0
0.0
0.5
0
6
OVCAR
10−nonadecenoate (19:1n9)
−1.0
0.0
1.0
2.0
0
6
HCT15
10−nonadecenoate (19:1n9)
−1.0
−0.6
−0.2
0.2
0
6
SKOV3

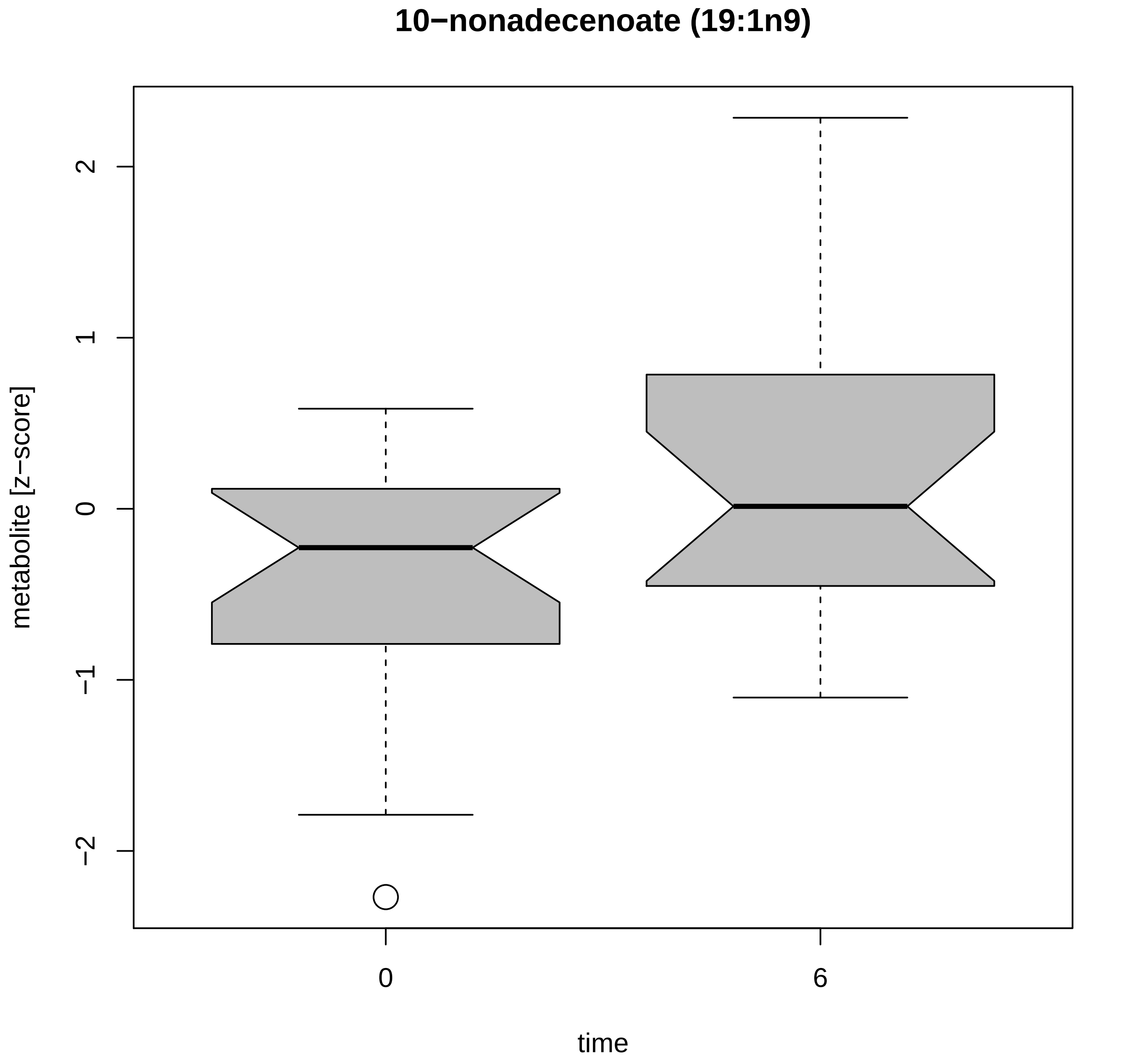
10−nonadecenoate (19:1n9)
metabolite [z−score]
2
1
0
−1
−2
0
6
time

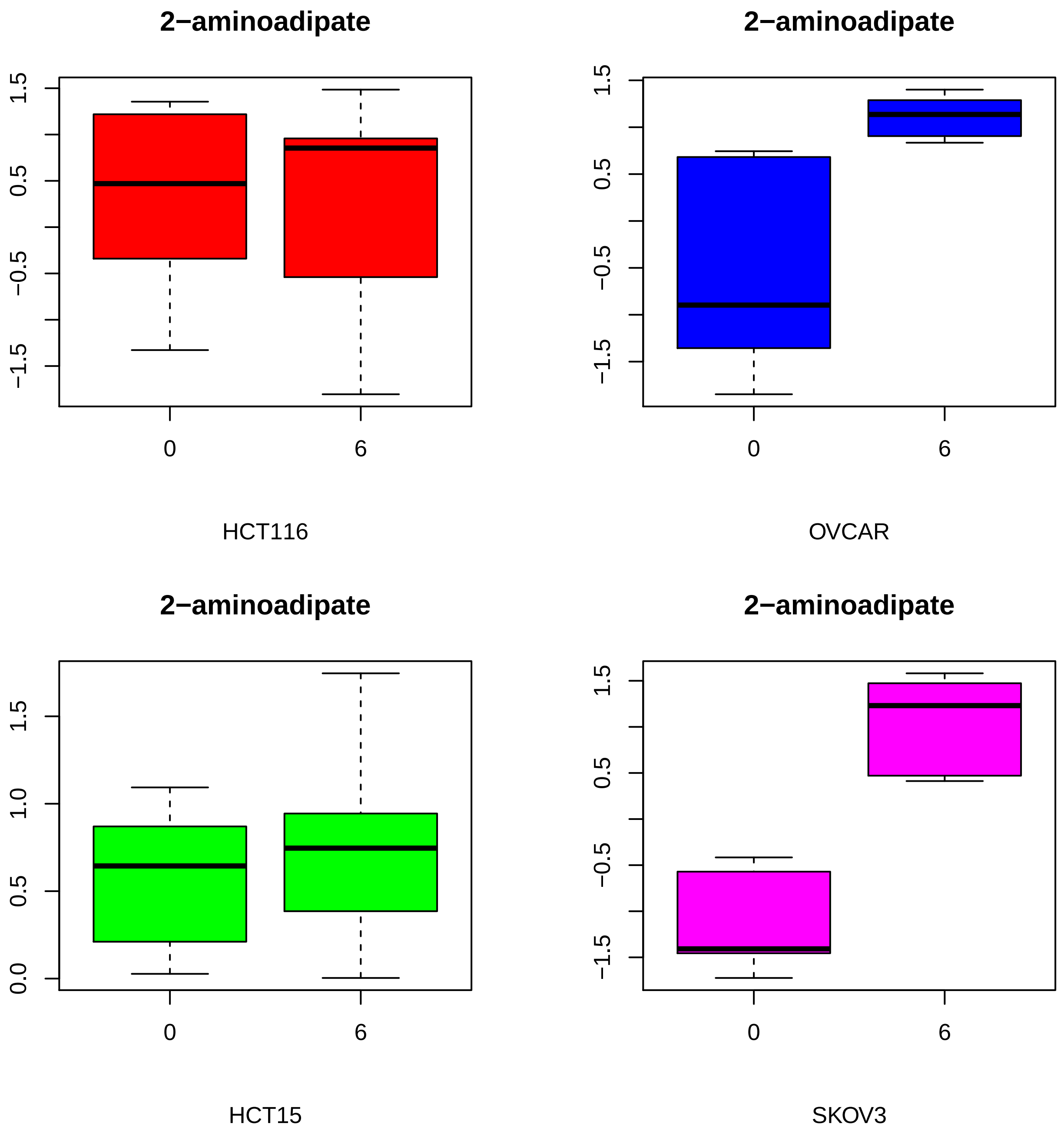

2−aminoadipate
1.5
0.5
−0.5
−1.5
0
6
HCT116
2−aminoadipate
1.5
0.5
−0.5
−1.5
0
6
OVCAR
2−aminoadipate
1.5
1.0
0.5
0.0
0
6
HCT15
2−aminoadipate
1.5
0.5
−0.5
−1.5
0
6
SKOV3

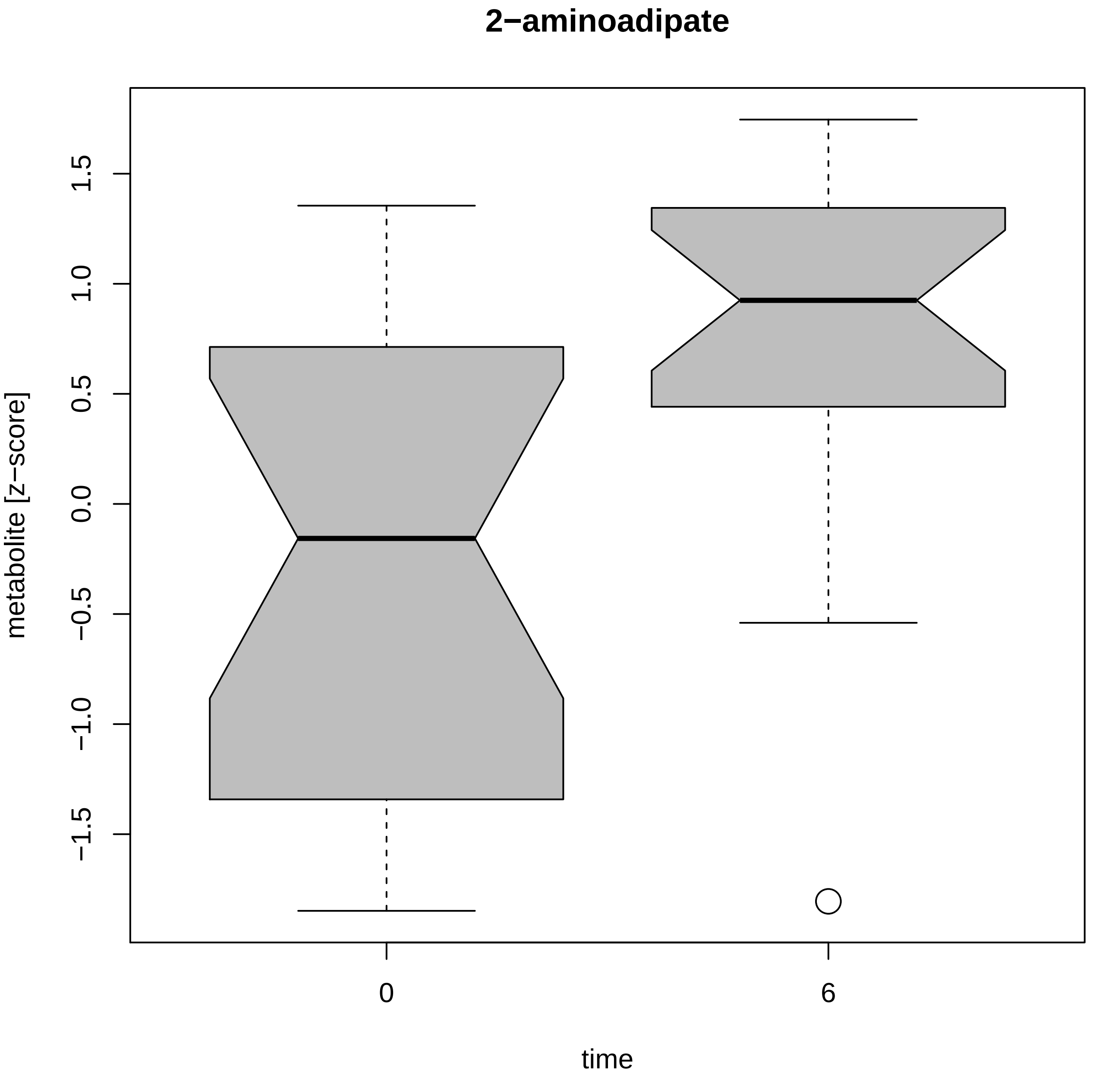
2−aminoadipate
metabolite [z−score]
1.5
1.0
0.5
0.0
−0.5
−1.0
−1.5
0
6
time

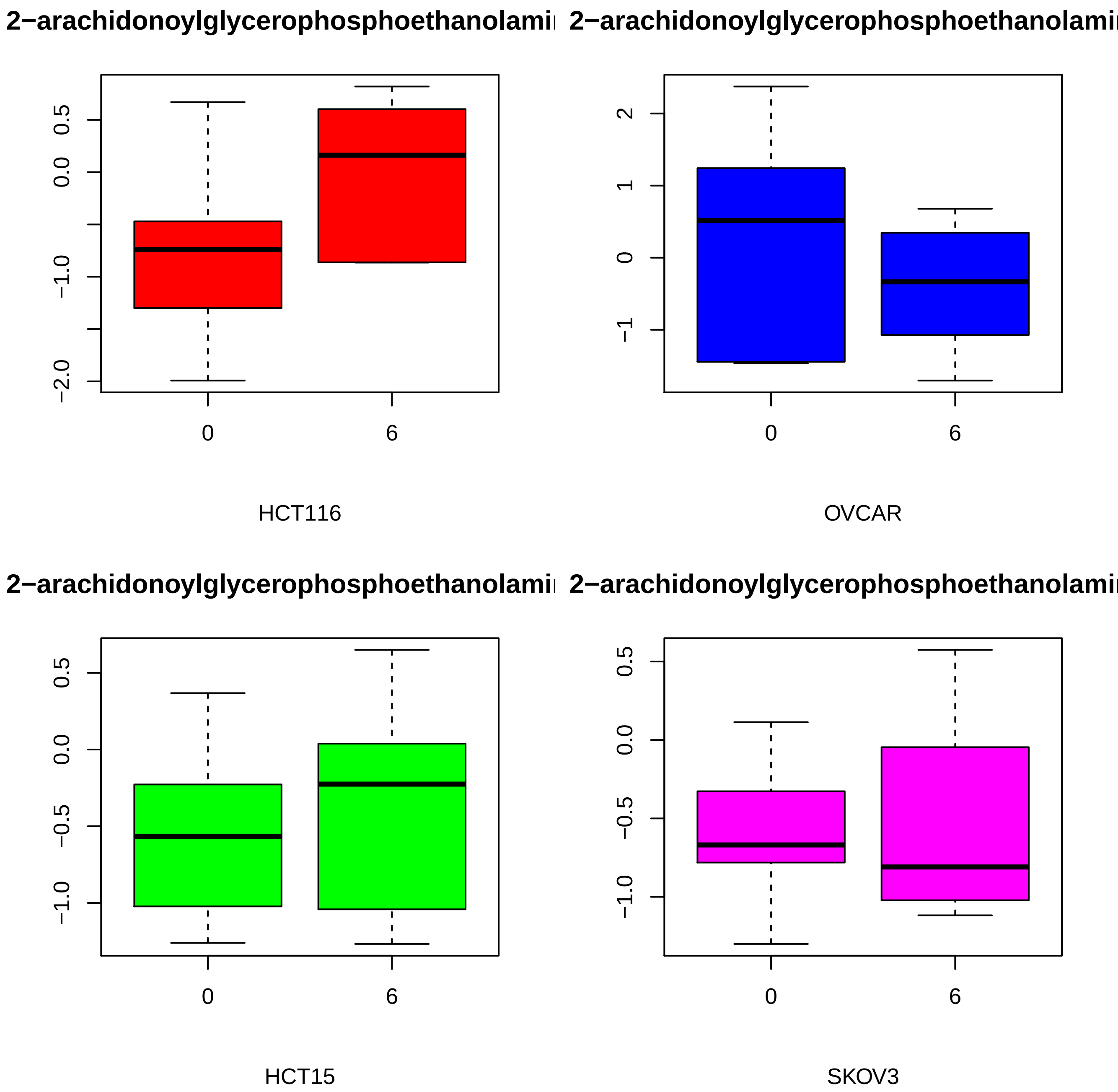
2−arachidonoylglycerophosphoethanolami
0.5
0.0
−1.0
−2.0
0
6
HCT116
2−arachidonoylglycerophosphoethanolami
2
1
0
−1
0
6
OVCAR
2−arachidonoylglycerophosphoethanolami
0.5
0.0
−0.5
−1.0
0
6
HCT15
2−arachidonoylglycerophosphoethanolami
0.5
0.0
−0.5
−1.0
0
6
SKOV3

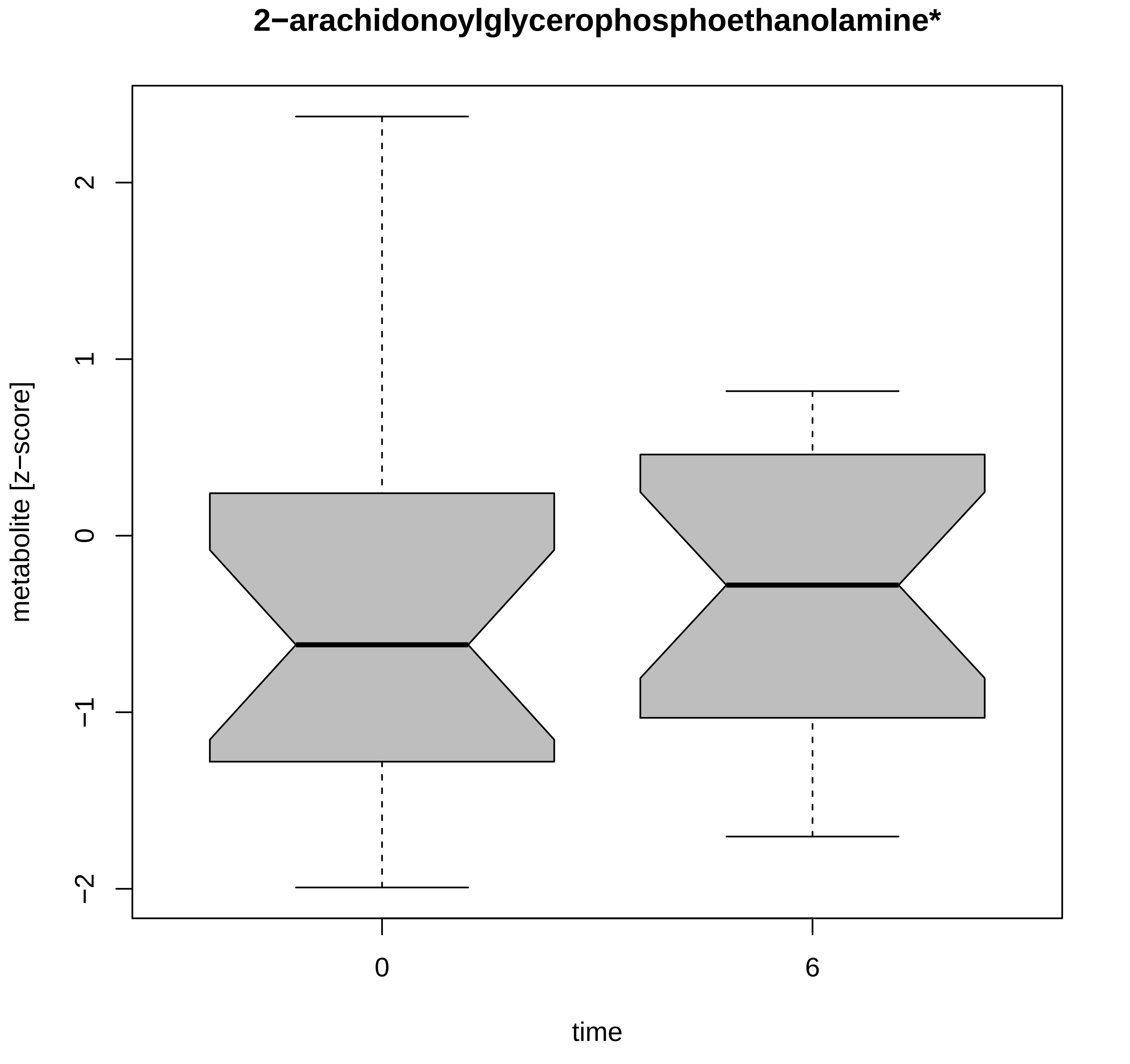
2−arachidonoylglycerophosphoethanolamine*
metabolite [z−score]
2
1
0
−1
−2
0
6
time

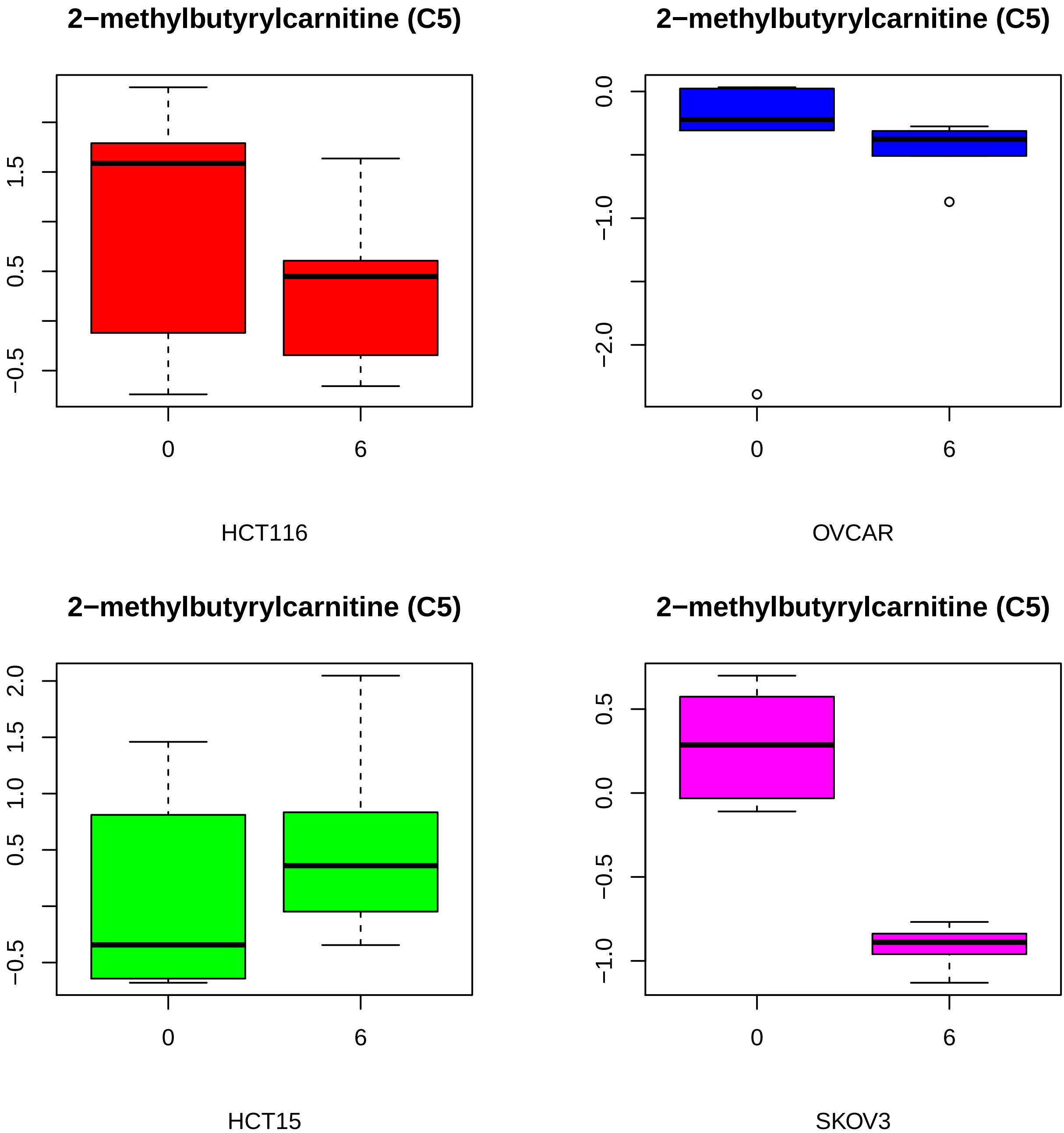
2−methylbutyrylcarnitine (C5)
−0.5
0.5
1.5
0
6
HCT116
2−methylbutyrylcarnitine (C5)
−2.0
−1.0
0.0
0
6
OVCAR
2−methylbutyrylcarnitine (C5)
−0.5
0.5
1.0
1.5
2.0
0
6
HCT15
2−methylbutyrylcarnitine (C5)
−1.0
−0.5
0.0
0.5
0
6
SKOV3

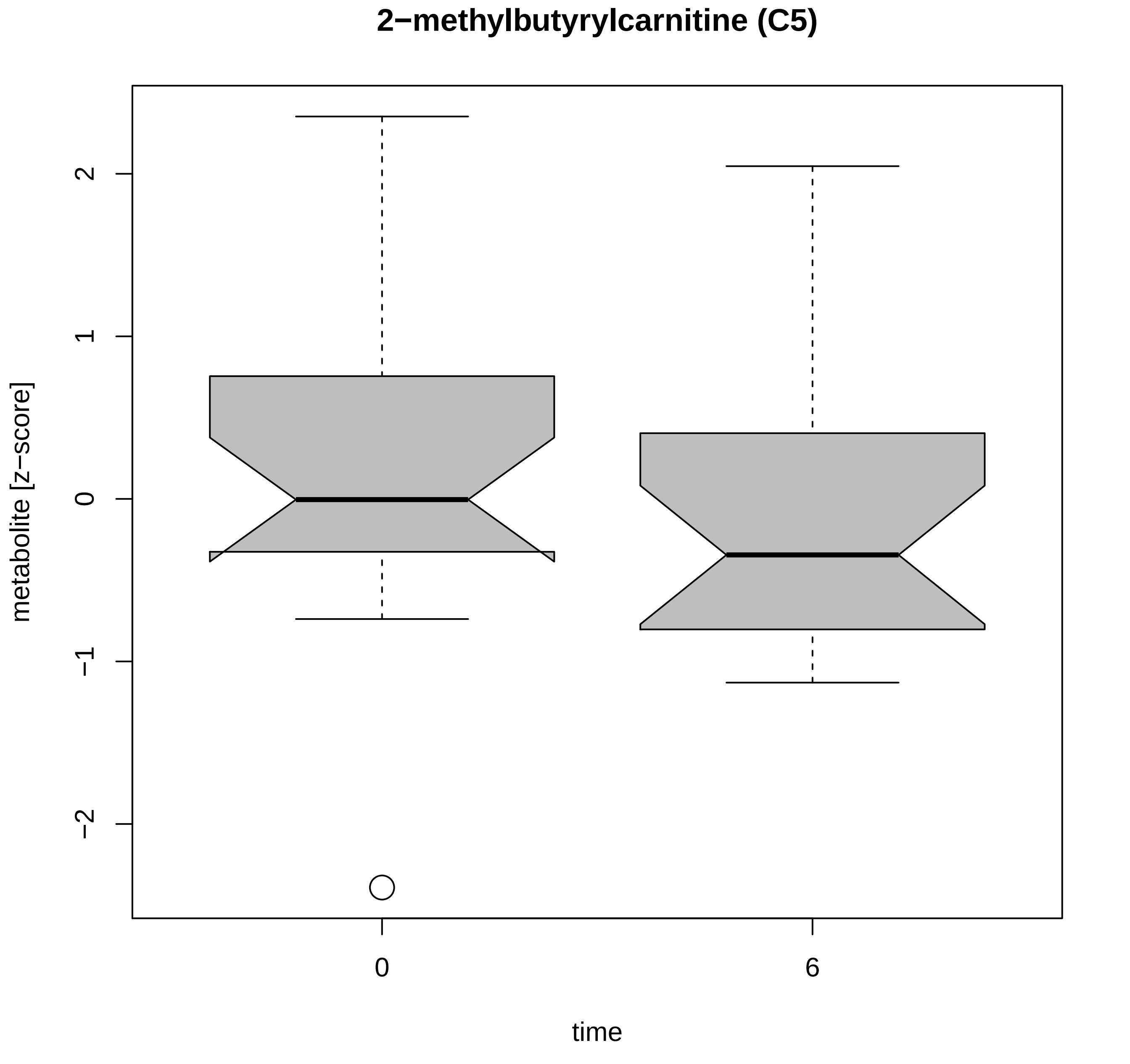

2−methylbutyrylcarnitine (C5)
metabolite [z−score]
2
1
0
−1
−2
0
6
time

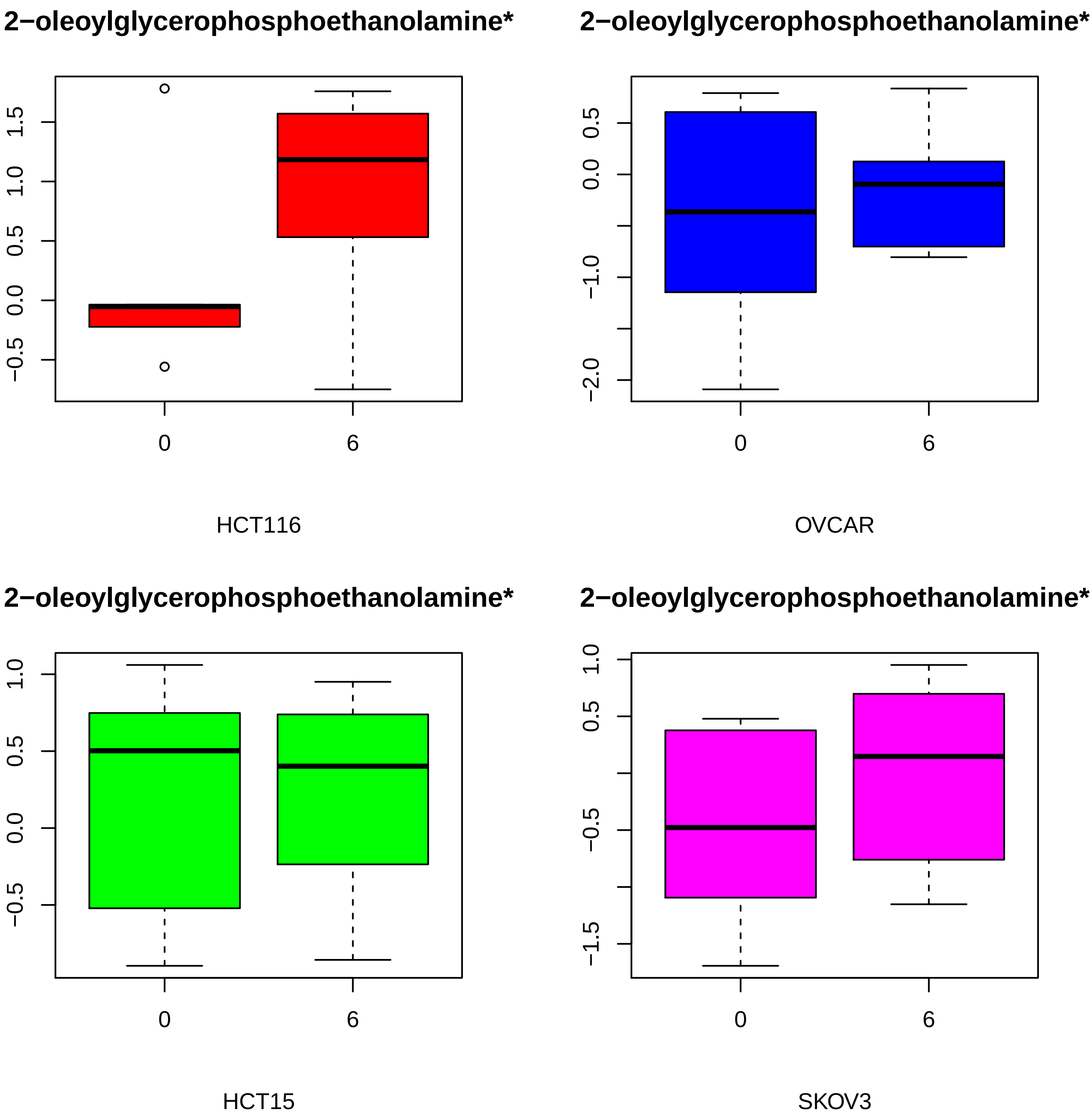

2−oleoylglycerophosphoethanolamine*
1.5
1.0
0.5
0.0
−0.5
0
6
HCT116
2−oleoylglycerophosphoethanolamine*
0.5
0.0
−1.0
−2.0
0
6
OVCAR
2−oleoylglycerophosphoethanolamine*
1.0
0.5
0.0
−0.5
0
6
HCT15
2−oleoylglycerophosphoethanolamine*
1.0
0.5
−0.5
−1.5
0
6
SKOV3

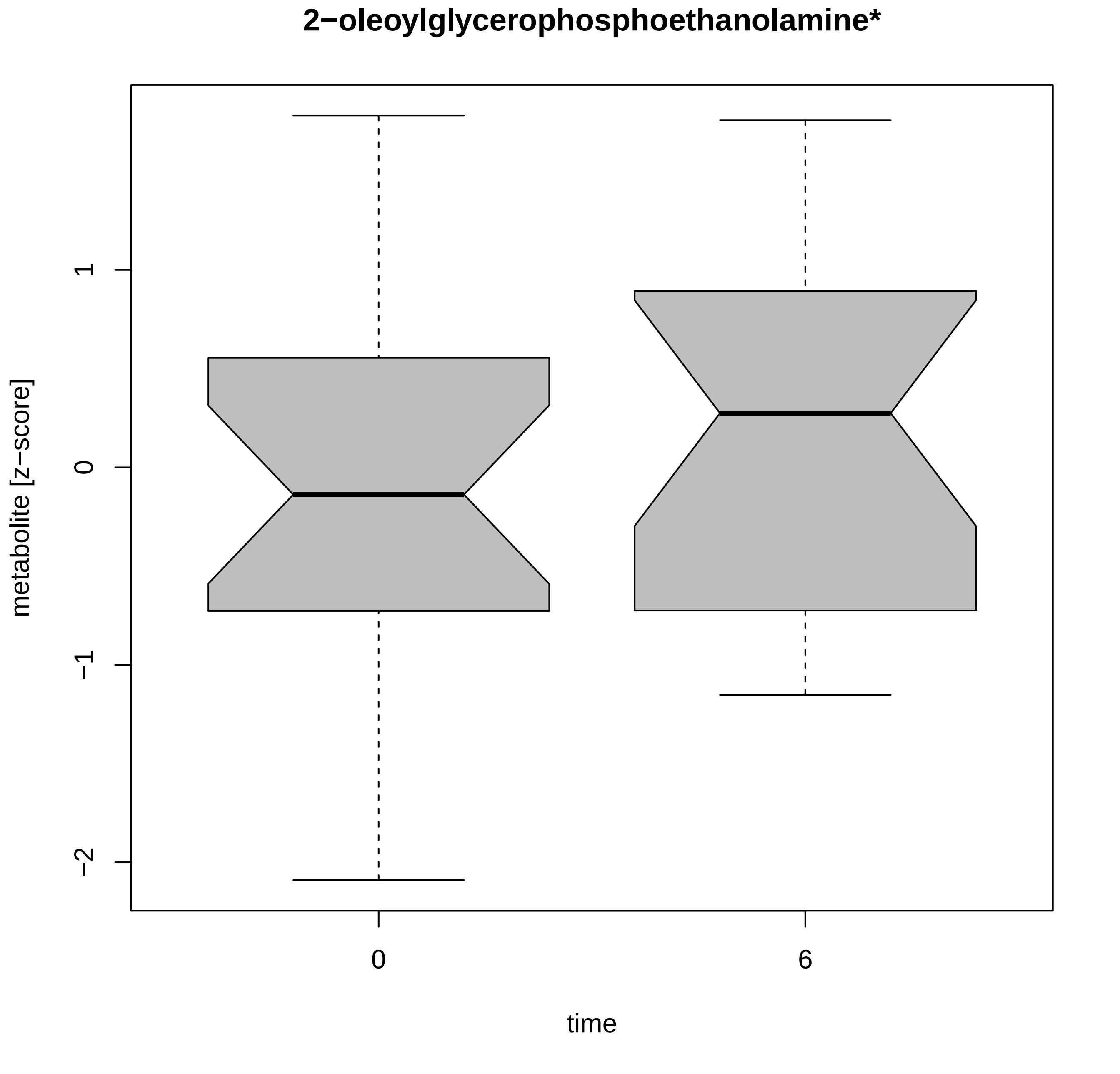

2−oleoylglycerophosphoethanolamine*
metabolite [z−score]
1
0
−1
−2
0
6
time

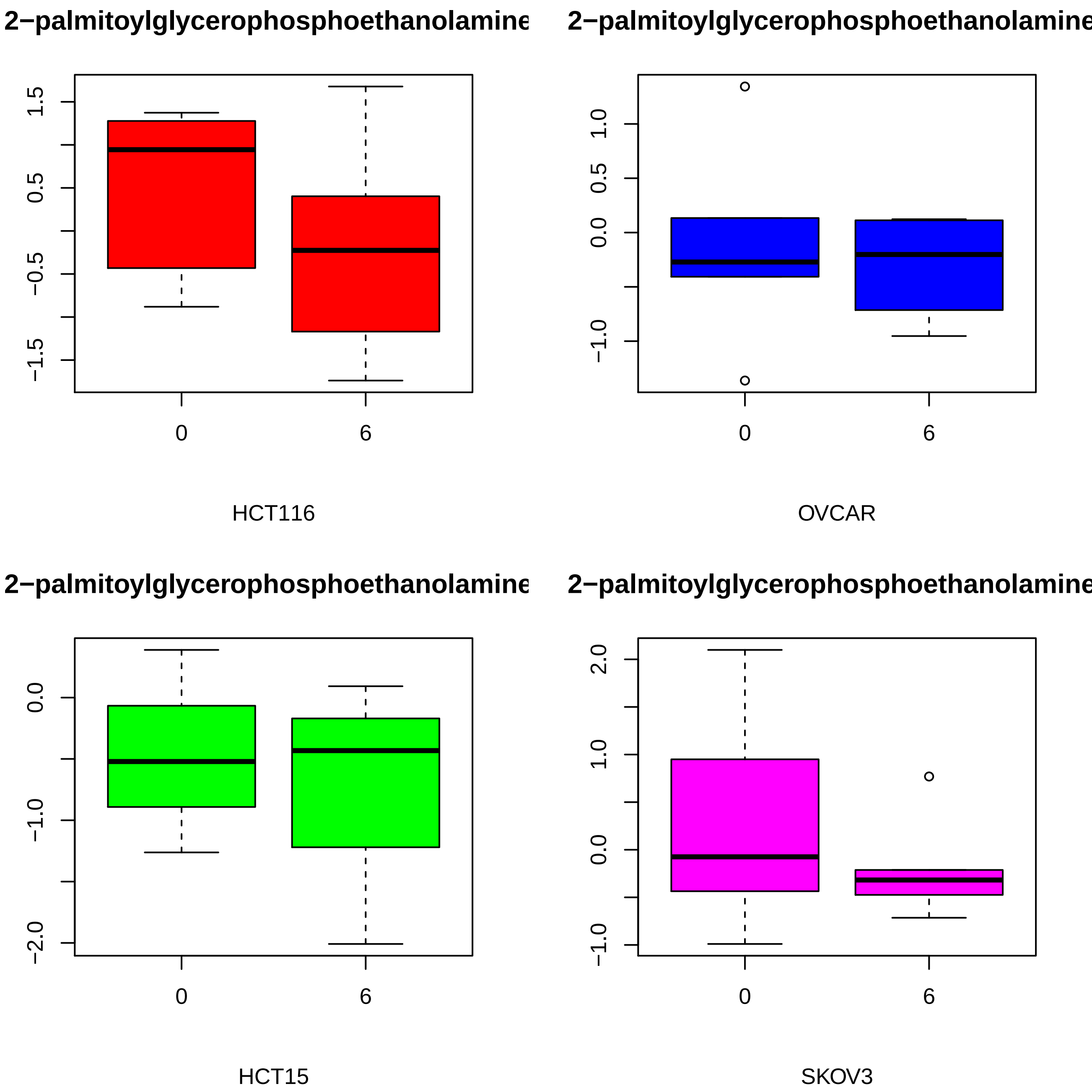
2−palmitoylglycerophosphoethanolamine
1.5
0.5
−0.5
−1.5
0
6
HCT116
2−palmitoylglycerophosphoethanolamine
1.0
0.5
0.0
−1.0
0
6
OVCAR
2−palmitoylglycerophosphoethanolamine
0.0
−1.0
−2.0
0
6
HCT15
2−palmitoylglycerophosphoethanolamine
2.0
1.0
0.0
−1.0
0
6
SKOV3

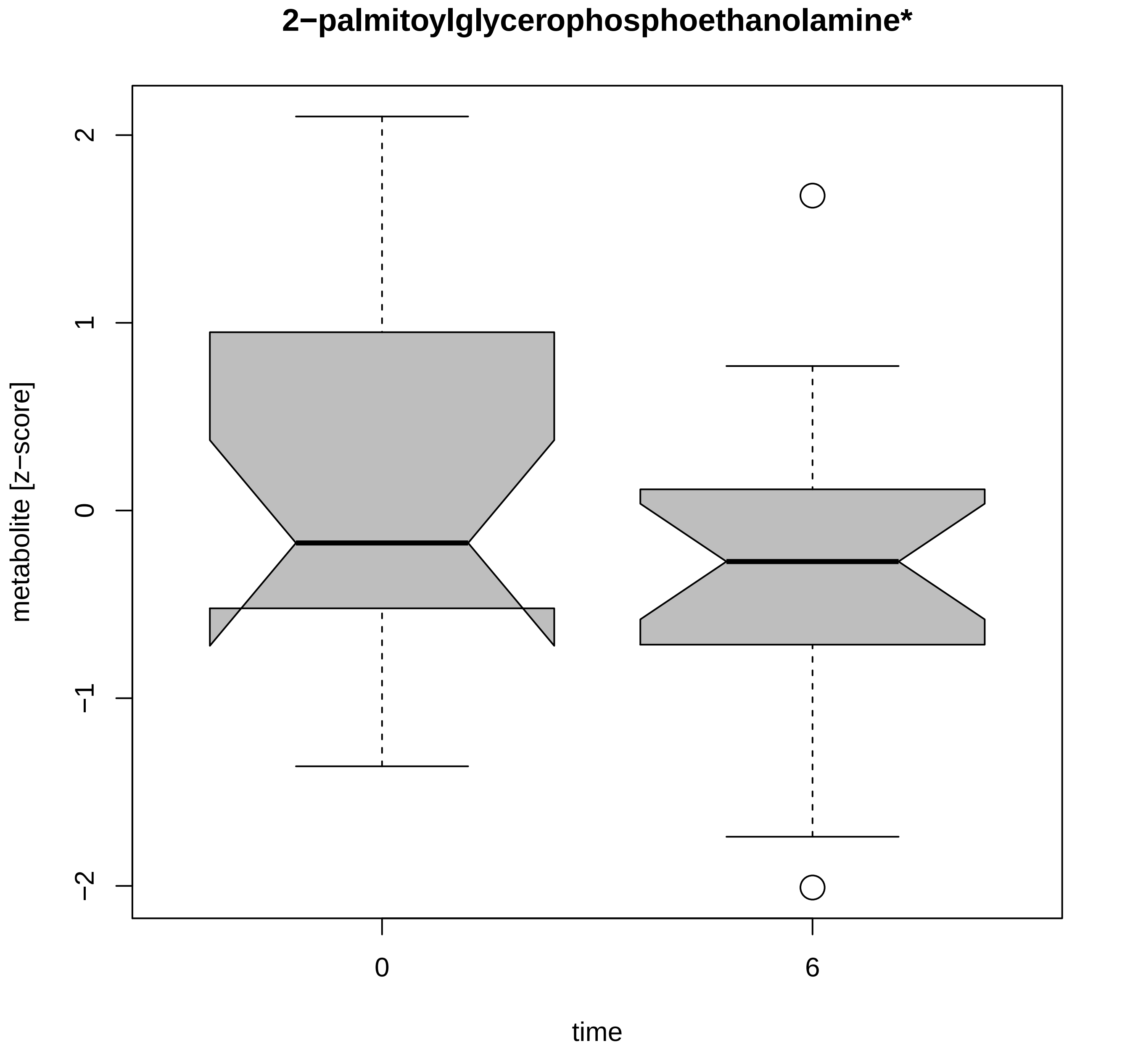
2−palmitoylglycerophosphoethanolamine*
metabolite [z−score]
2
1
0
−1
−2
0
6
time

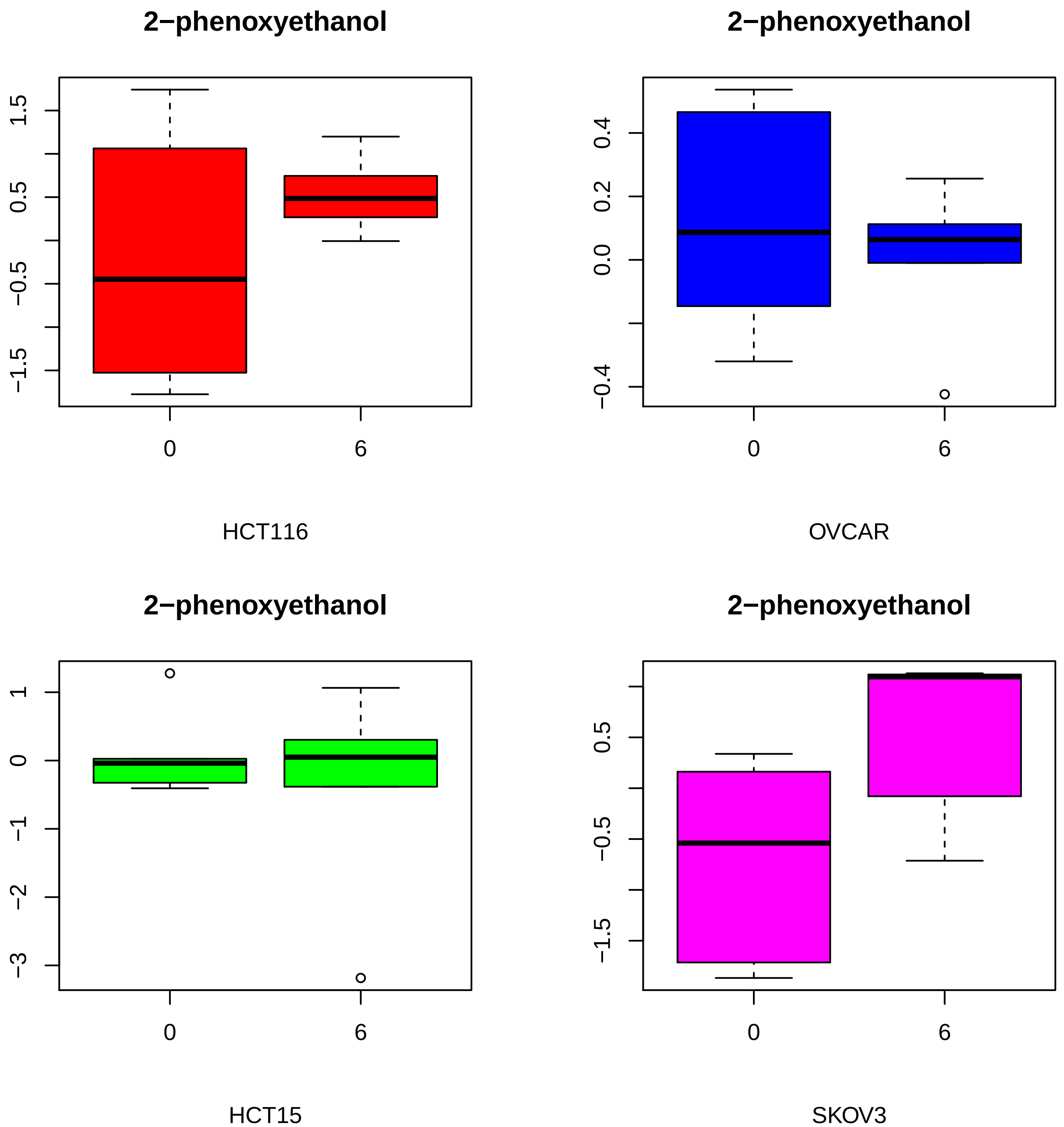
2−phenoxyethanol
1.5
0.5
−0.5
−1.5
0
6
HCT116
2−phenoxyethanol
0.4
0.2
0.0
−0.4
0
6
OVCAR
2−phenoxyethanol
1
0
−1
−2
−3
0
6
HCT15
2−phenoxyethanol
0.5
−0.5
−1.5
0
6
SKOV3

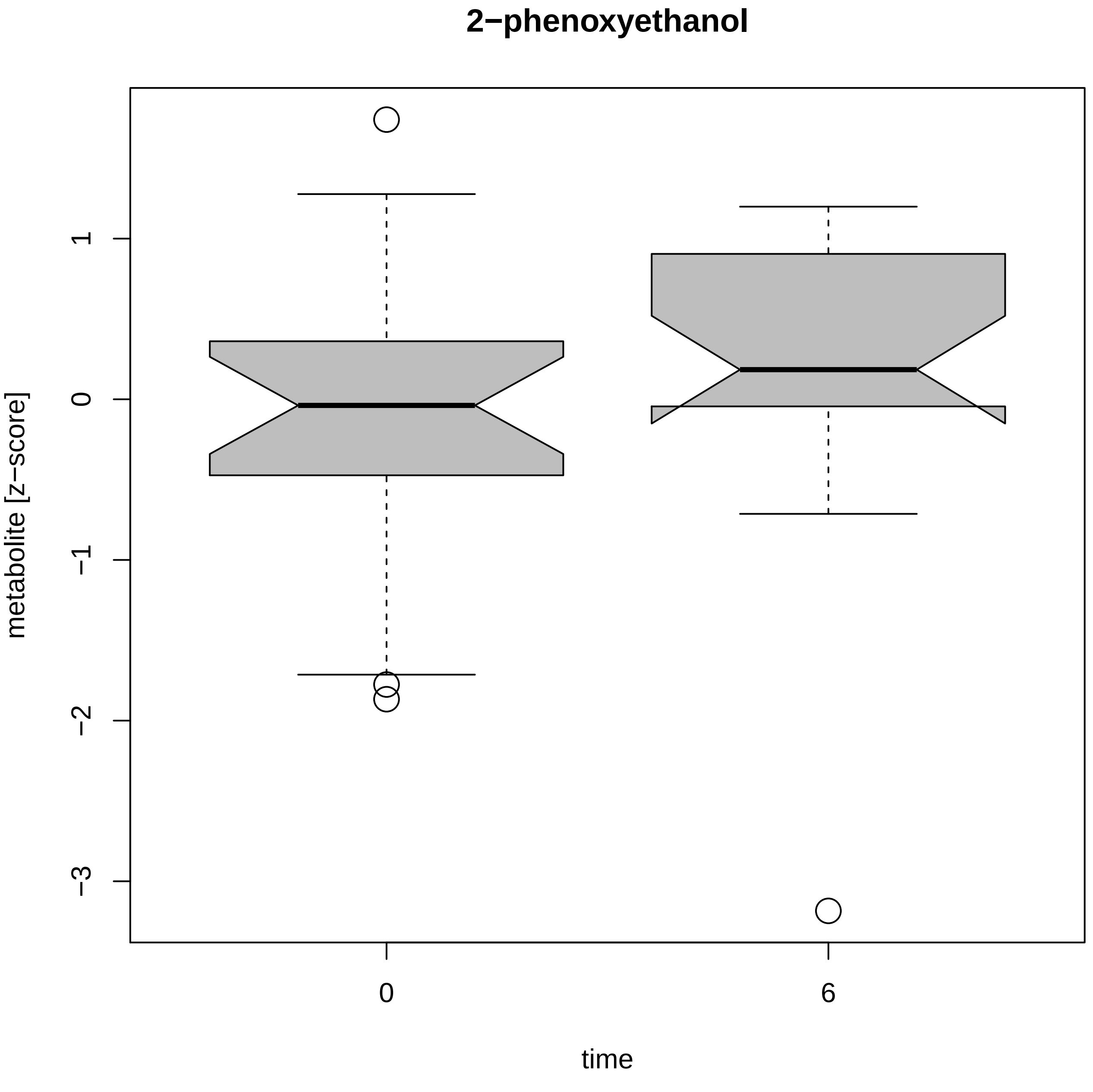

2−phenoxyethanol
metabolite [z−score]
1
0
−1
−2
−3
0
6
time

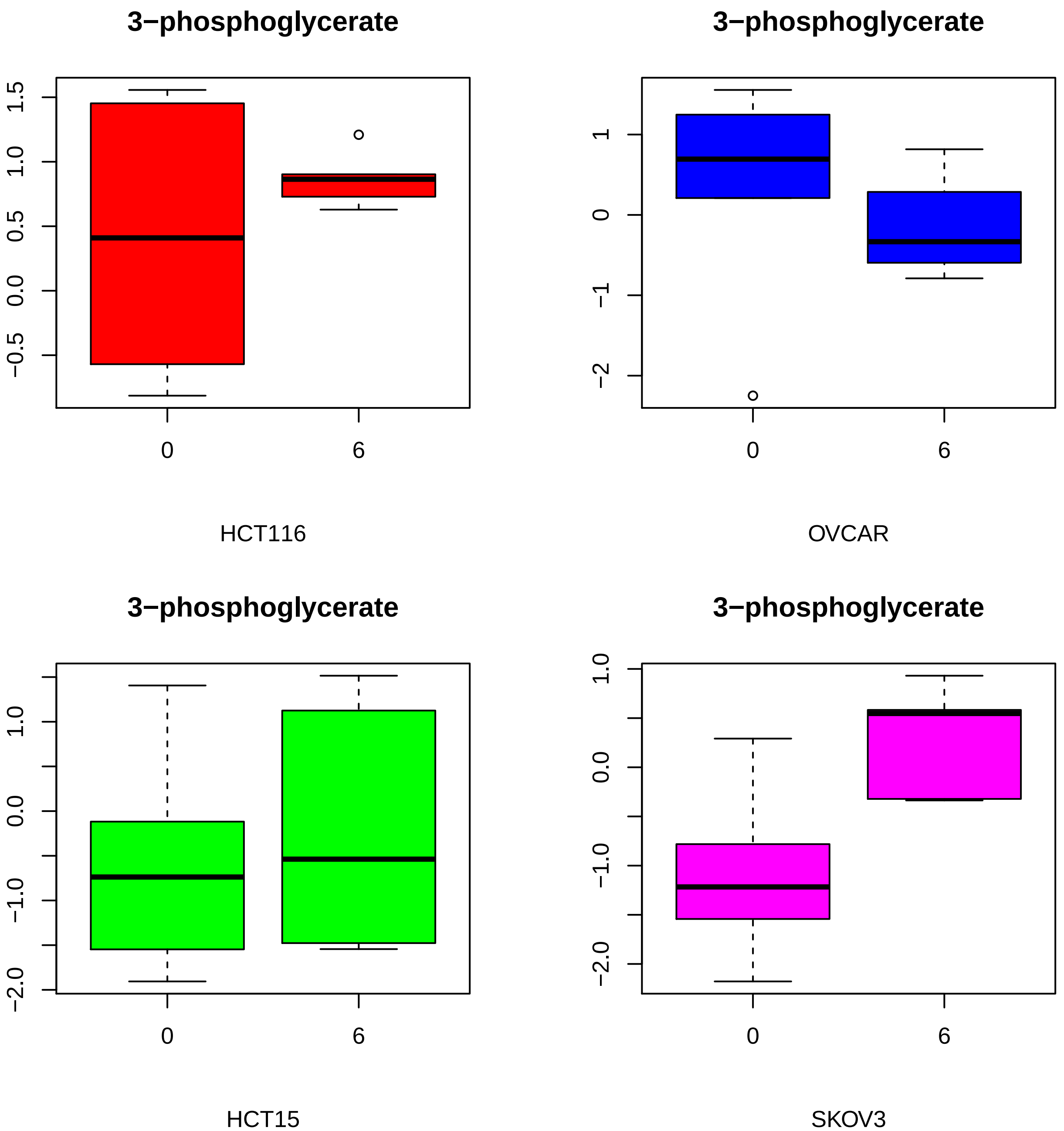
3−phosphoglycerate
1.5
1.0
0.5
0.0
−0.5
0
6
HCT116
3−phosphoglycerate
1
0
−1
−2
0
6
OVCAR
3−phosphoglycerate
1.0
0.0
−1.0
−2.0
0
6
HCT15
3−phosphoglycerate
1.0
0.0
−1.0
−2.0
0
6
SKOV3

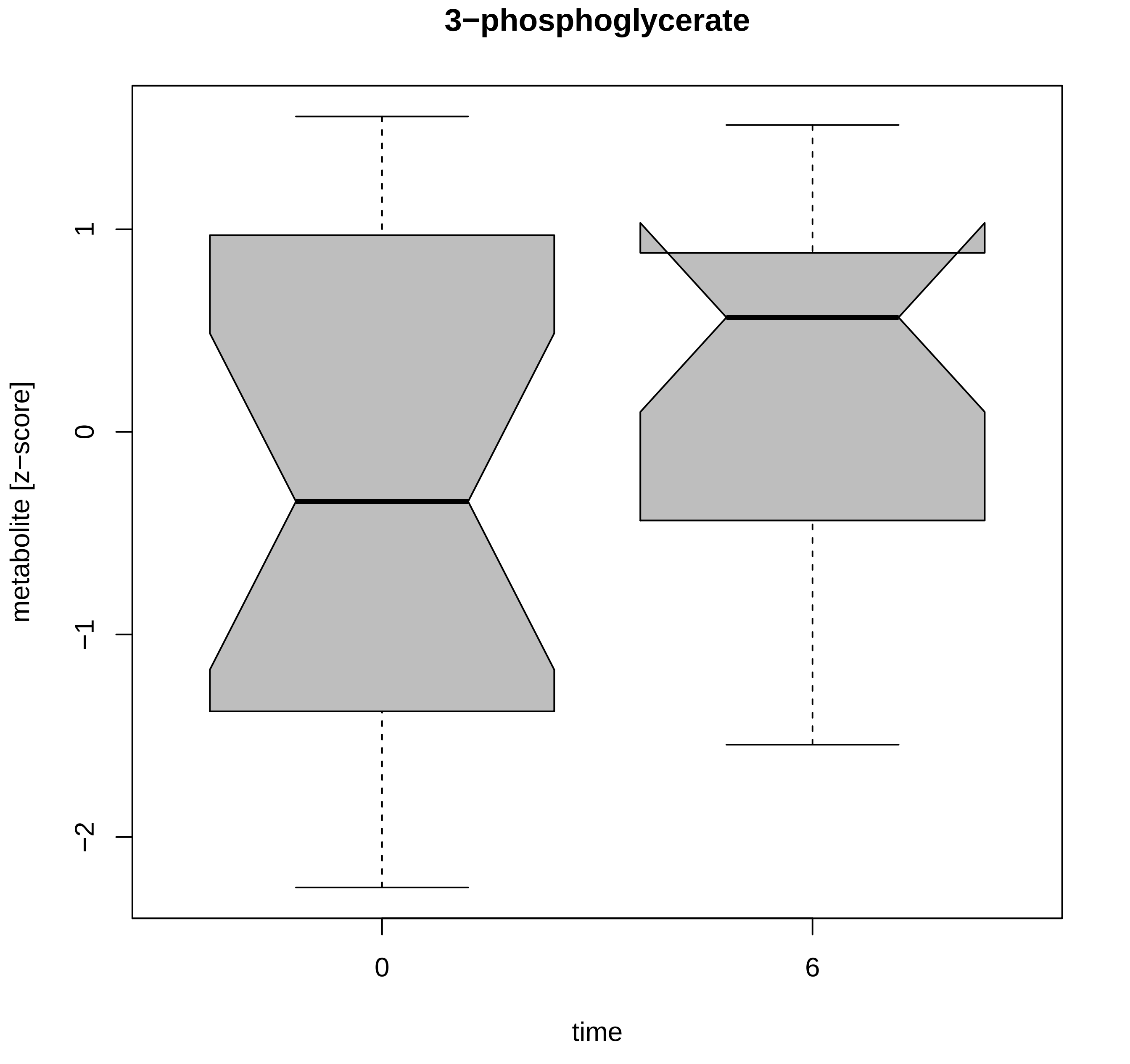

3−phosphoglycerate
metabolite [z−score]
1
0
−1
−2
0
6
time

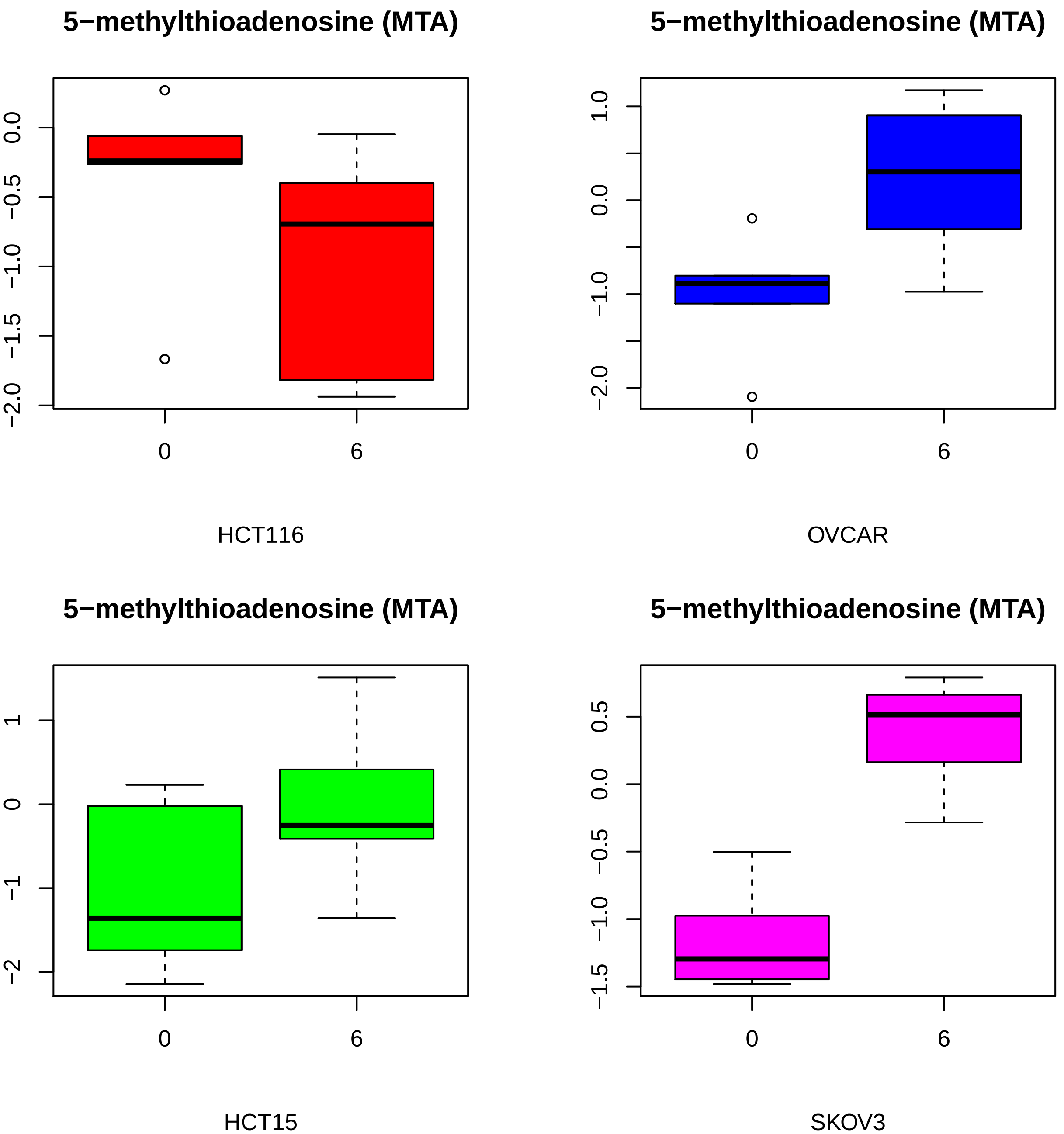
5−methylthioadenosine (MTA)
0.0
−0.5
−1.0
−1.5
−2.0
0
6
HCT116
5−methylthioadenosine (MTA)
1.0
0.0
−1.0
−2.0
0
6
OVCAR
5−methylthioadenosine (MTA)
1
0
−1
−2
0
6
HCT15
5−methylthioadenosine (MTA)
0.5
0.0
−0.5
−1.0
−1.5
0
6
SKOV3

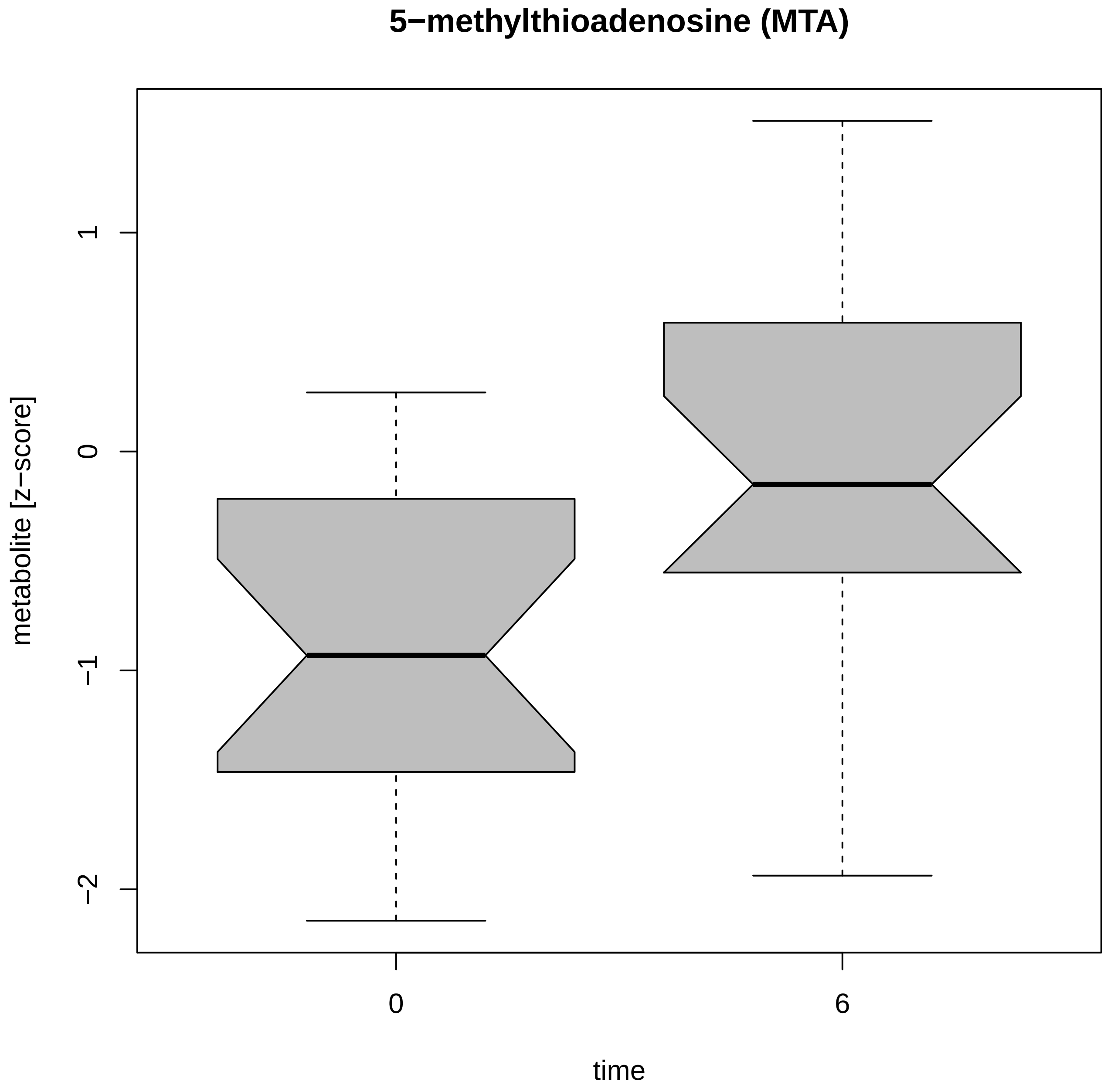
5−methylthioadenosine (MTA)
metabolite [z−score]
1
0
−1
−2
0
6
time

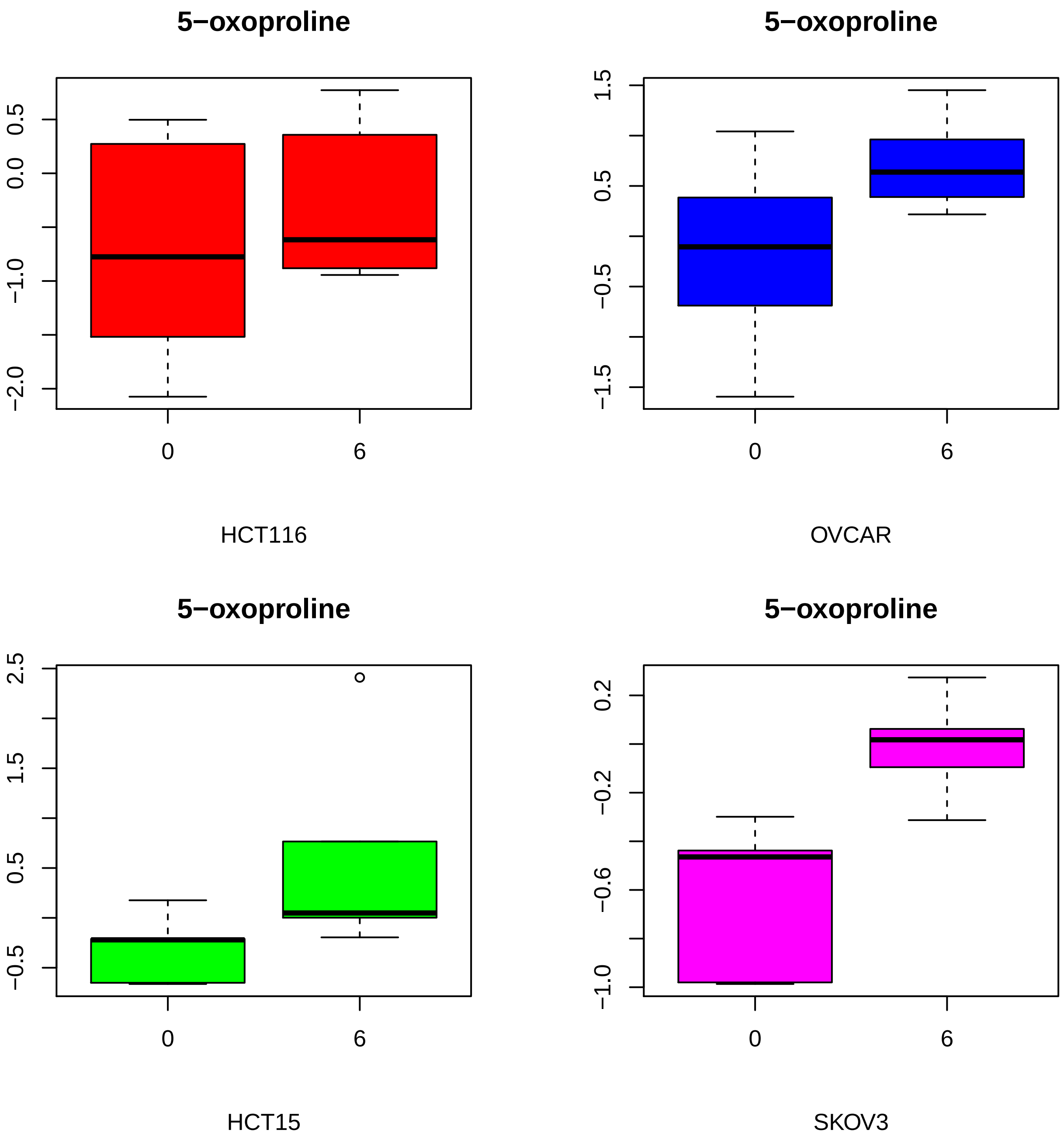
5−oxoproline
0.5
0.0
−1.0
−2.0
0
6
HCT116
5−oxoproline
1.5
0.5
−0.5
−1.5
0
6
OVCAR
5−oxoproline
2.5
1.5
0.5
−0.5
0
6
HCT15
5−oxoproline
0.2
−0.2
−0.6
−1.0
0
6
SKOV3

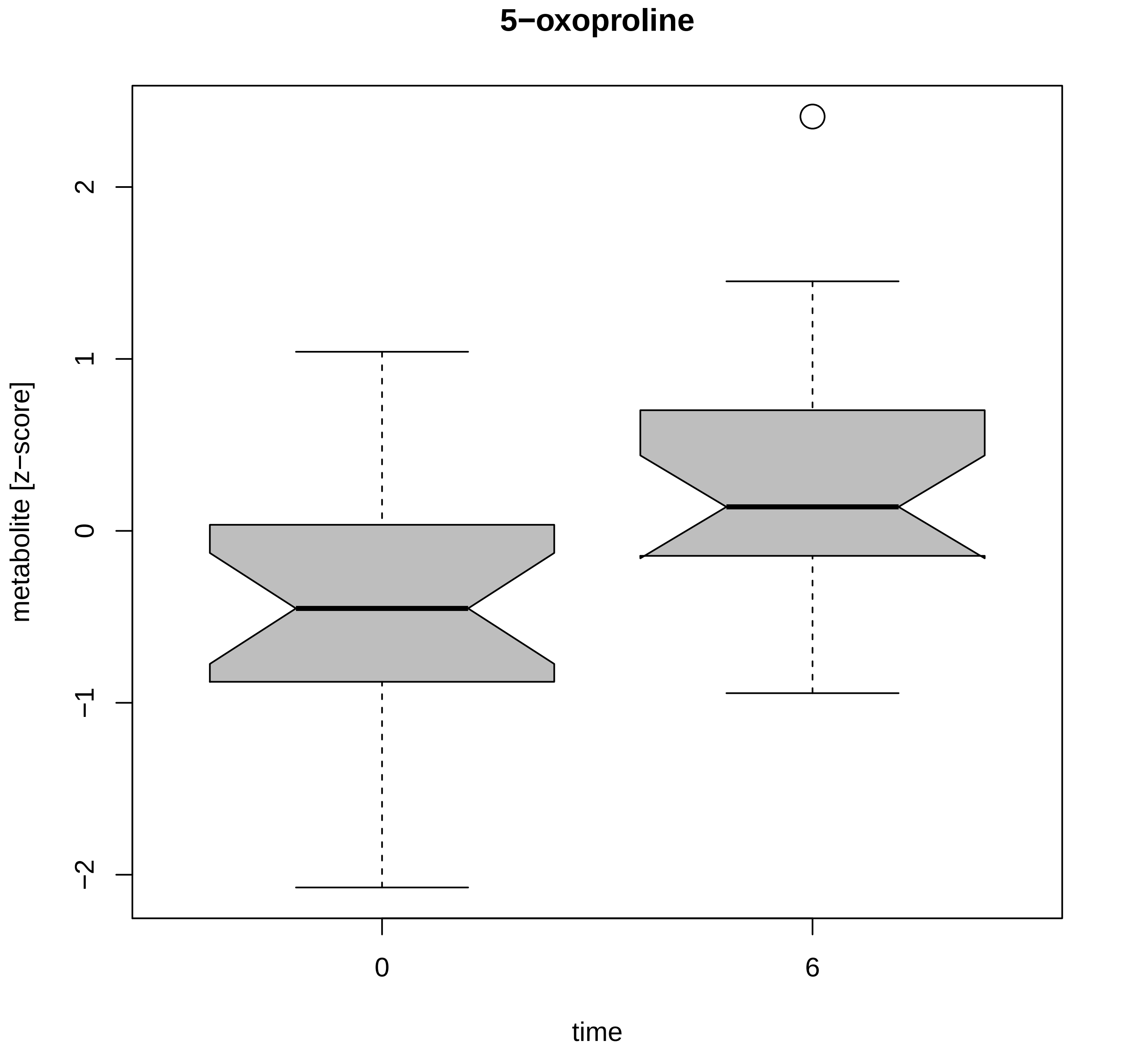
5−oxoproline
metabolite [z−score]
2
1
0
−1
−2
0
6
time

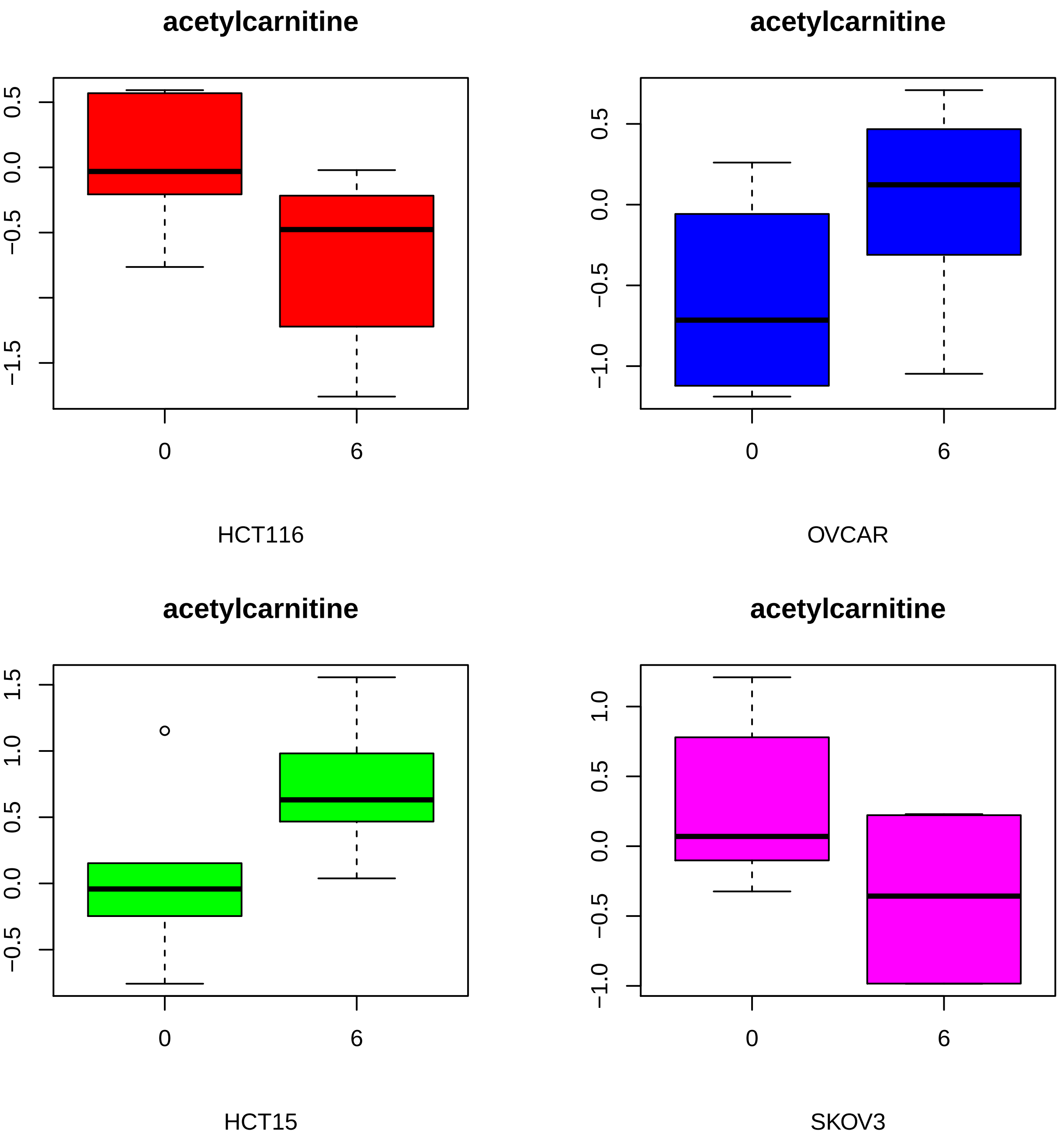
acetylcarnitine
0.5
0.0
−0.5
−1.5
0
6
HCT116
acetylcarnitine
0.5
0.0
−0.5
−1.0
0
6
OVCAR
acetylcarnitine
1.5
1.0
0.5
0.0
−0.5
0
6
HCT15
acetylcarnitine
1.0
0.5
0.0
−0.5
−1.0
0
6
SKOV3

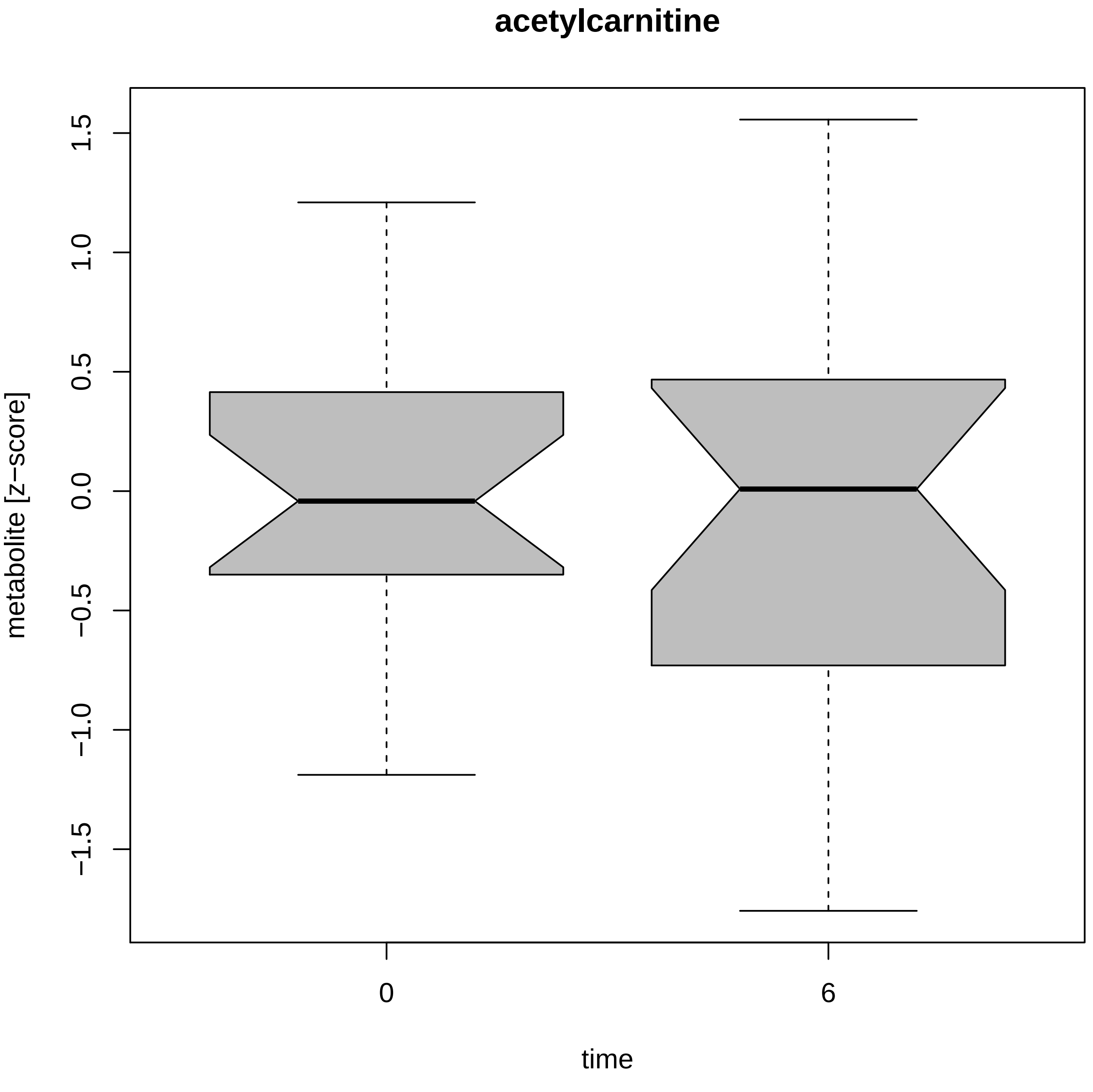
acetylcarnitine
metabolite [z−score]
1.5
1.0
0.5
0.0
−0.5
−1.0
−1.5
0
6
time

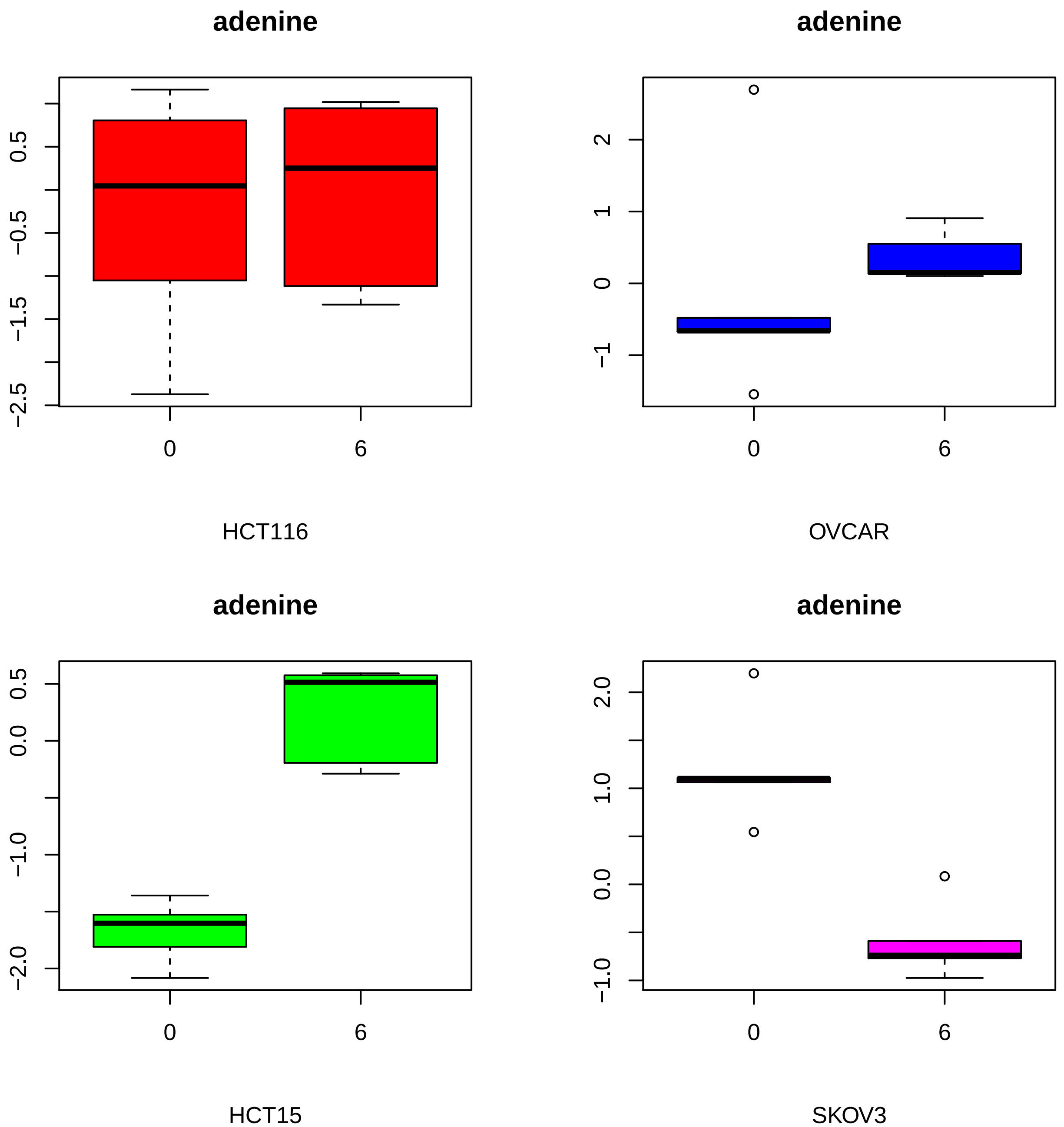

adenine
0.5
−0.5
−1.5
−2.5
0
6
HCT116
adenine
2
1
0
−1
0
6
OVCAR
adenine
0.5
0.0
−1.0
−2.0
0
6
HCT15
adenine
2.0
1.0
0.0
−1.0
0
6
SKOV3

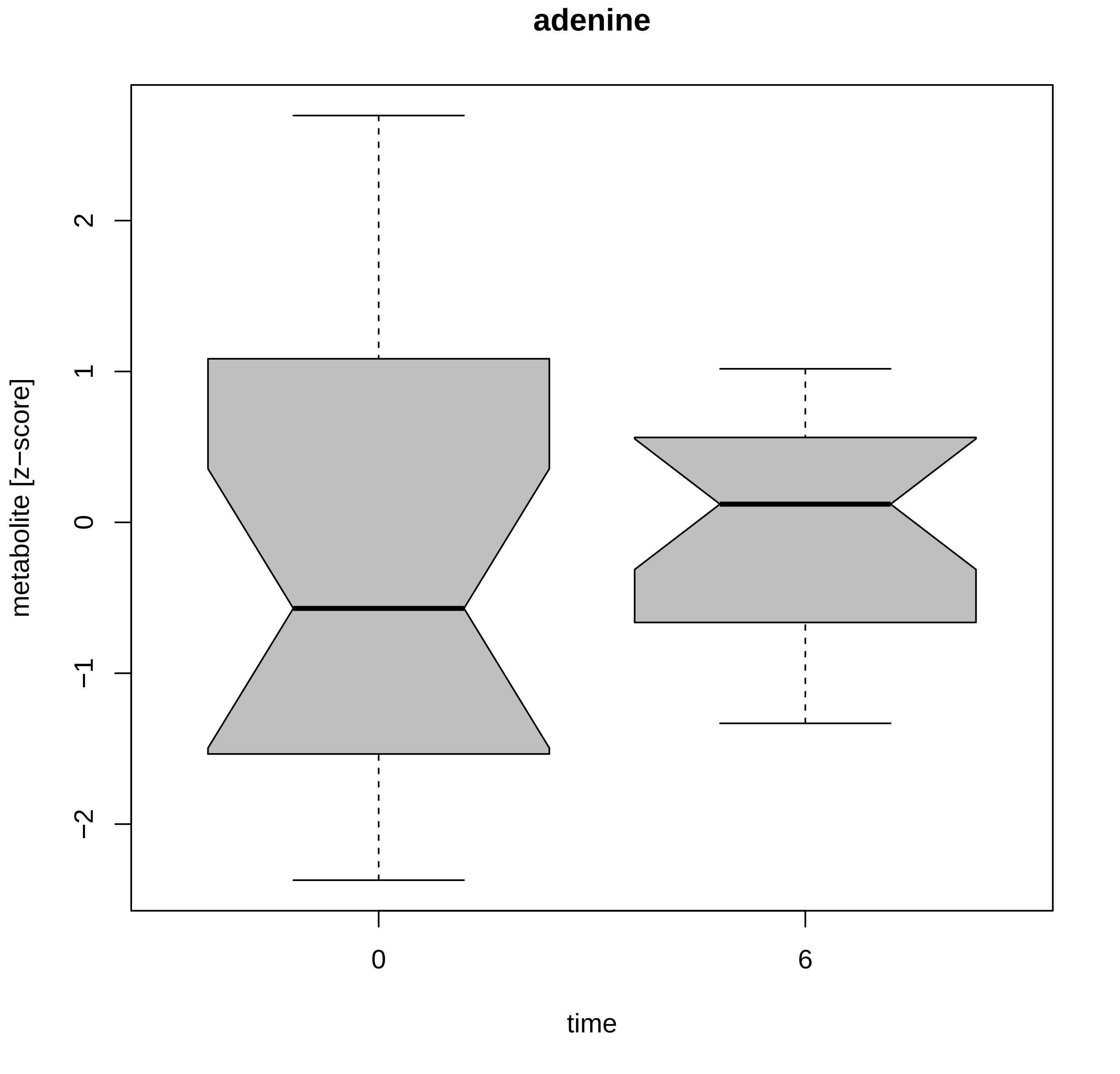
adenine
metabolite [z−score]
−2
−1
0
1
2
0
6
time

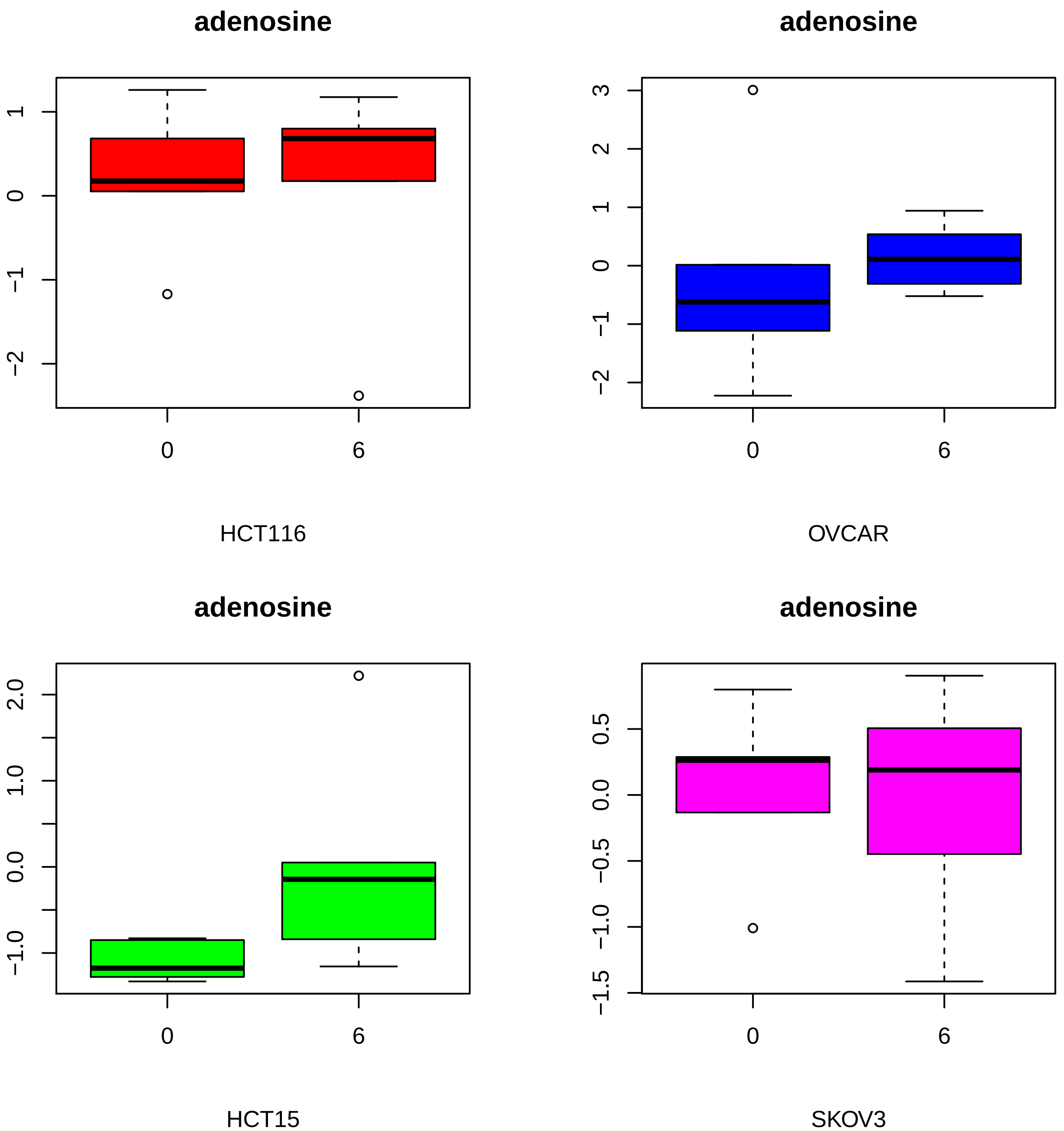
adenosine
1
0
−1
−2
0
6
HCT116
adenosine
3
2
1
0
−1
−2
0
6
OVCAR
adenosine
2.0
1.0
0.0
−1.0
0
6
HCT15
adenosine
0.5
0.0
−0.5
−1.0
−1.5
0
6
SKOV3

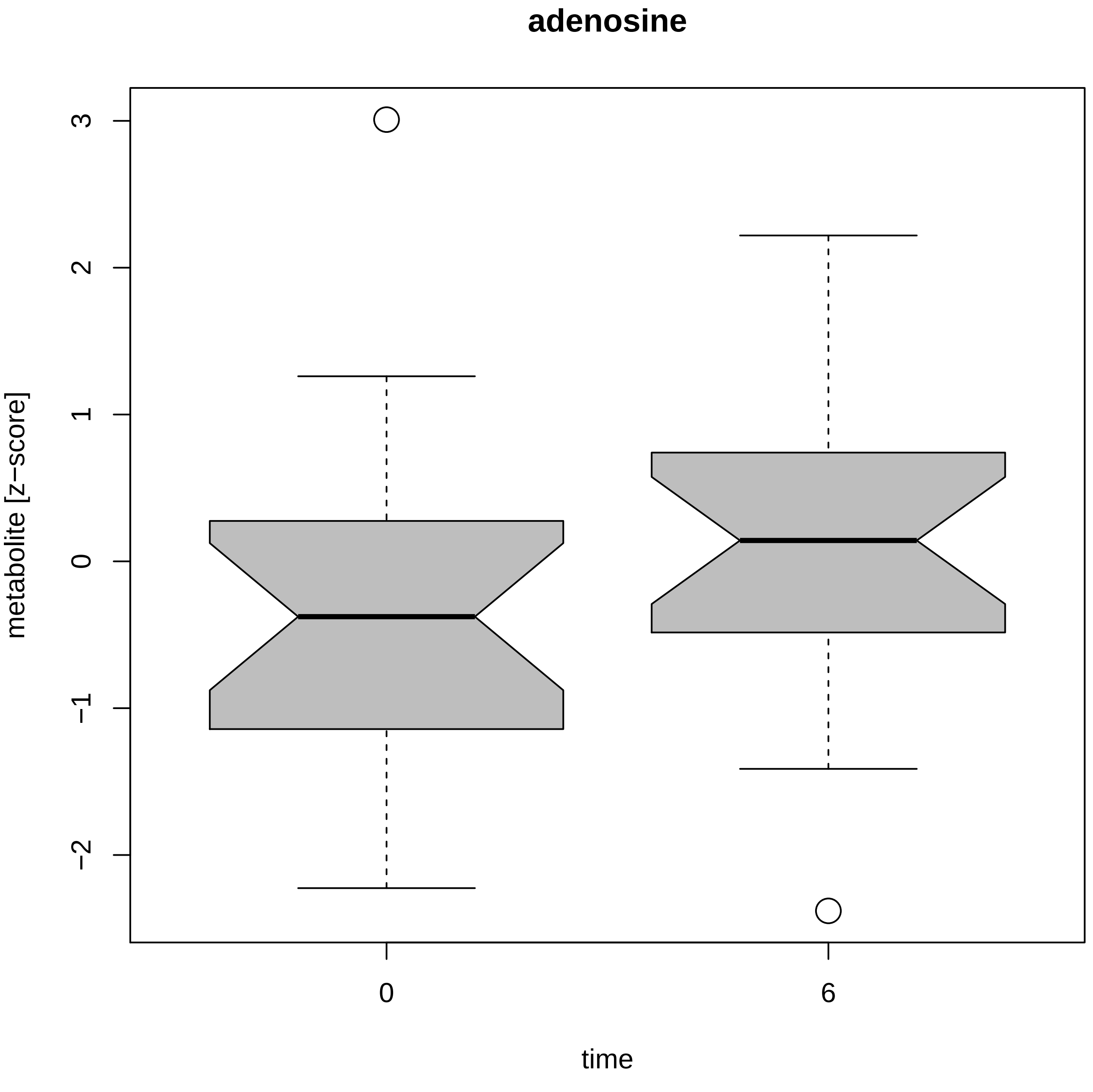
adenosine
metabolite [z−score]
3
2
1
0
−1
−2
0
6
time

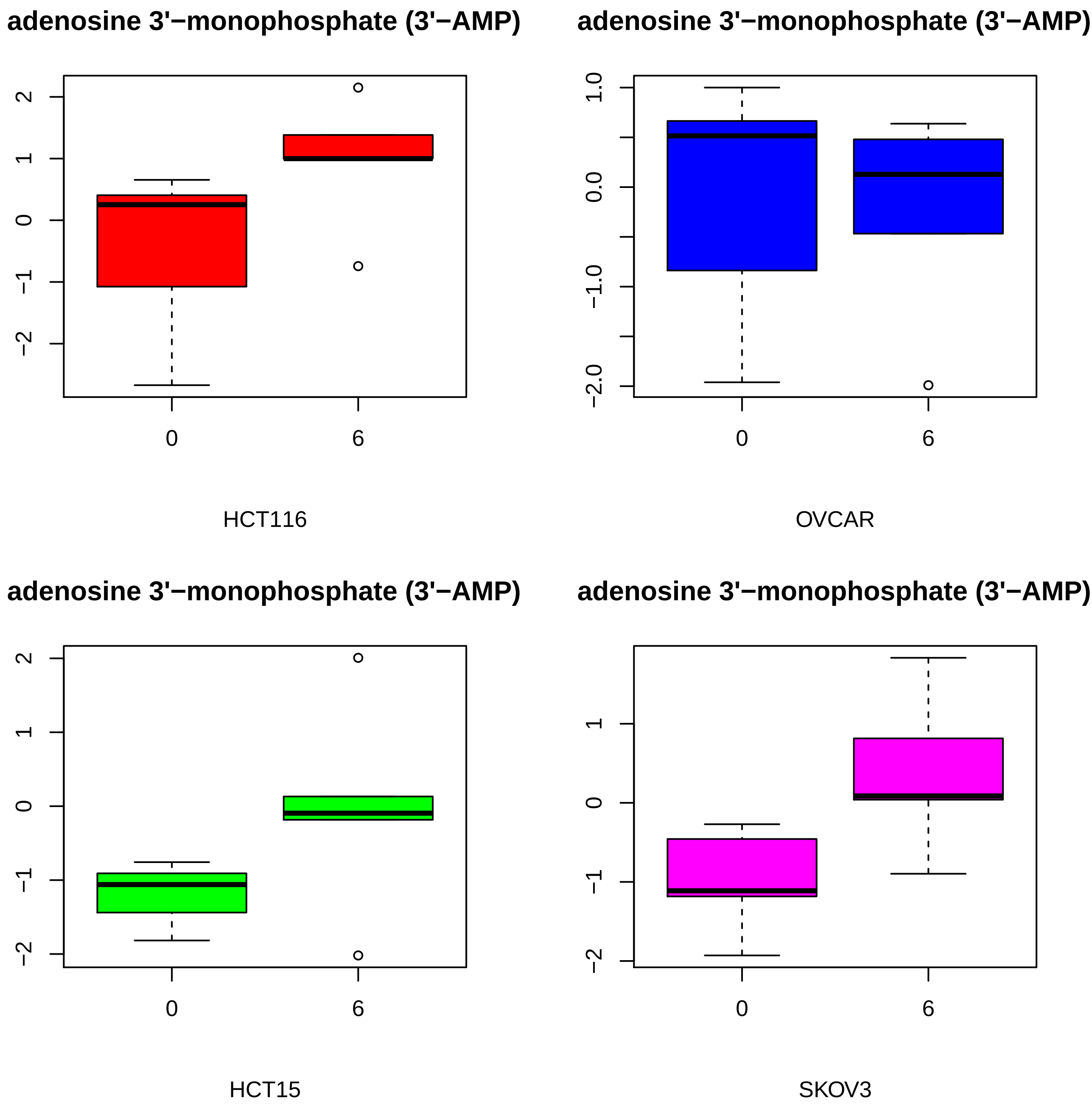

adenosine 3'−monophosphate (3'−AMP)
HCT116
adenosine 3'−monophosphate (3'−AMP)
OVCAR
adenosine 3'−monophosphate (3'−AMP)
HCT15
adenosine 3'−monophosphate (3'−AMP)
SKOV3

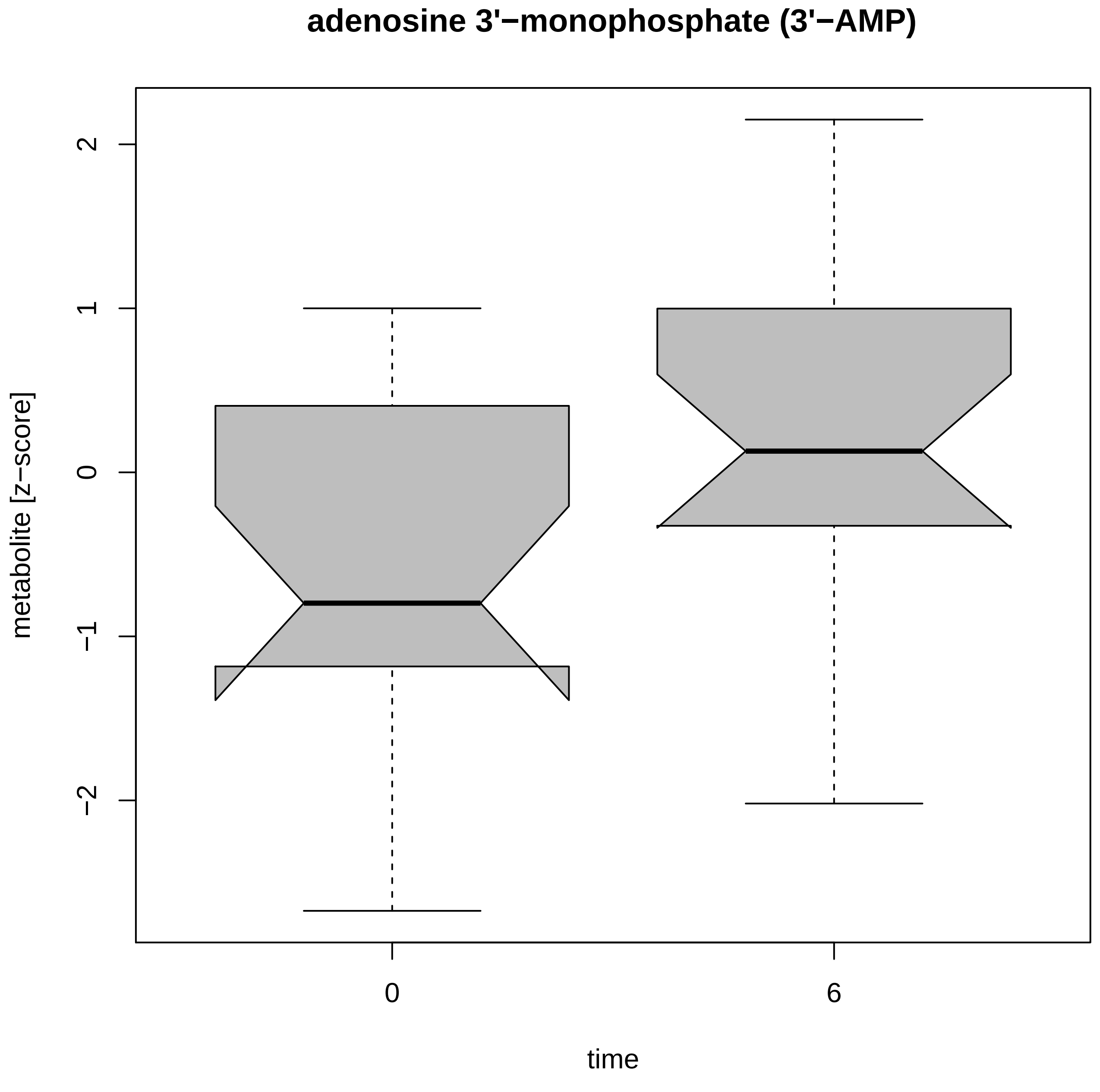
adenosine 3'−monophosphate (3'−AMP)
metabolite [z−score]
2
1
0
−1
−2
0
6
time

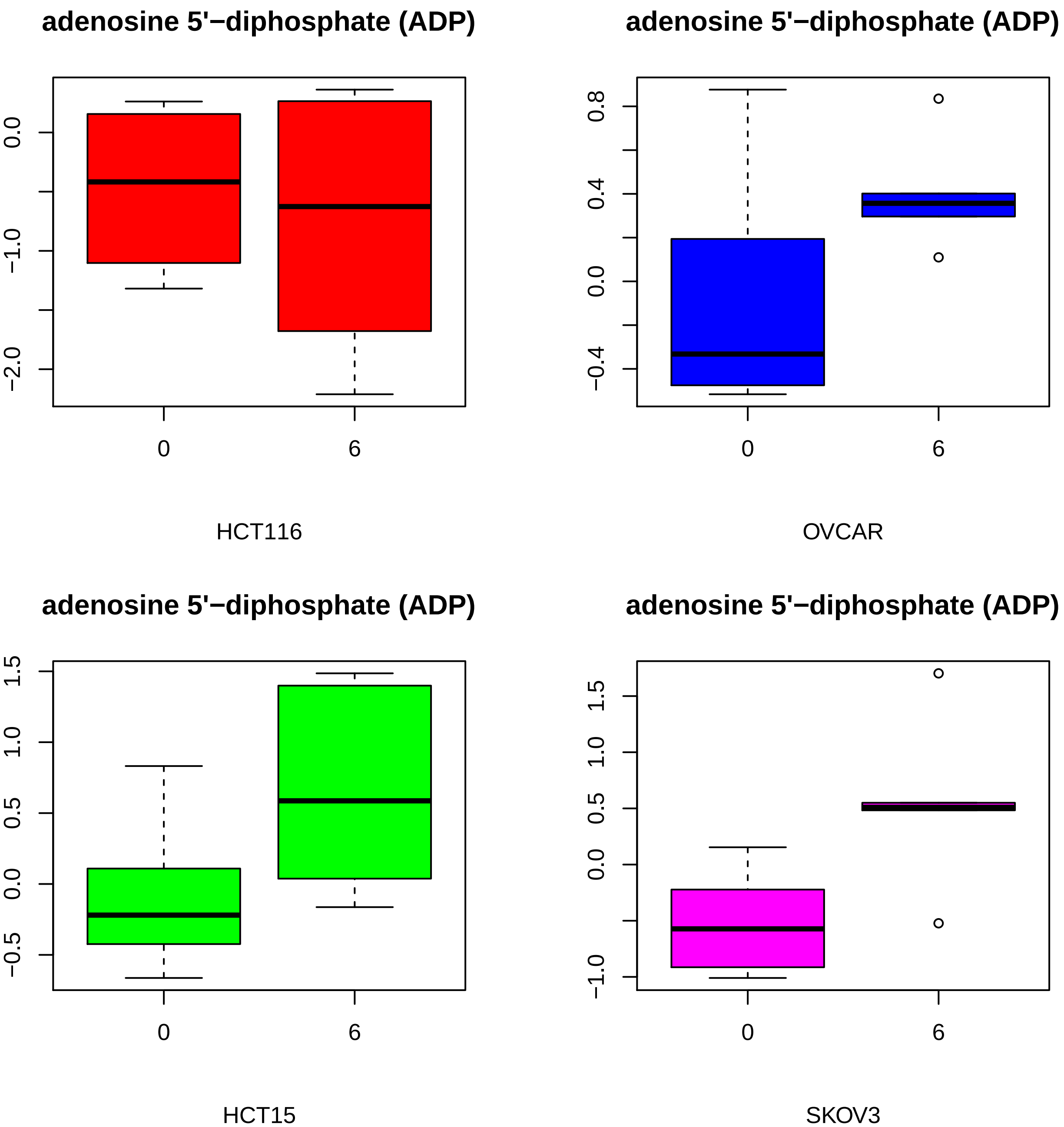

adenosine 5'−diphosphate (ADP)
0.0
−1.0
−2.0
0
6
HCT116
adenosine 5'−diphosphate (ADP)
0.8
0.4
0.0
−0.4
0
6
OVCAR
adenosine 5'−diphosphate (ADP)
1.5
1.0
0.5
0.0
−0.5
0
6
HCT15
adenosine 5'−diphosphate (ADP)
1.5
1.0
0.5
0.0
−1.0
0
6
SKOV3

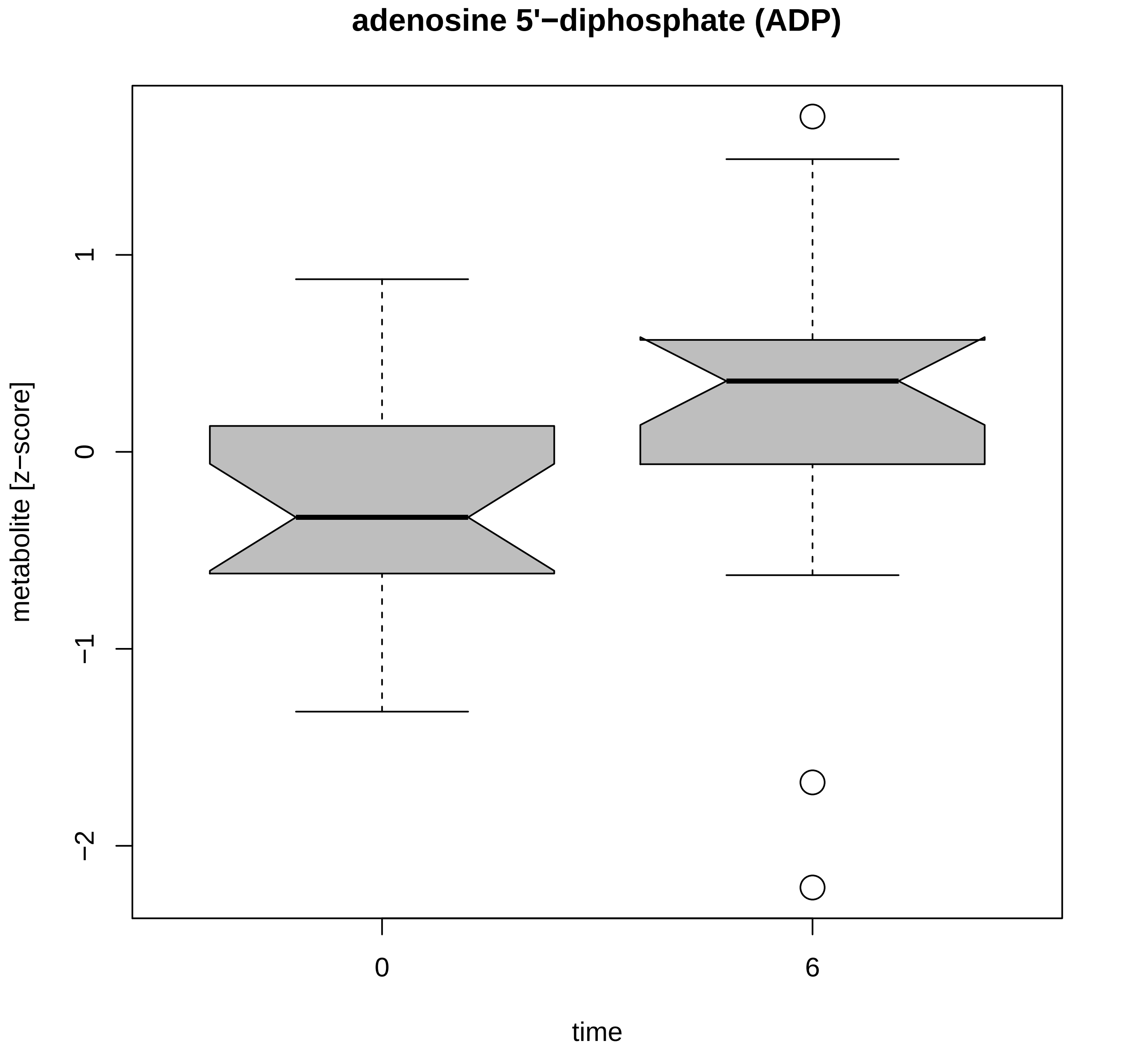

adenosine 5'−diphosphate (ADP)
metabolite [z−score]
1
0
−1
−2
0
6
time

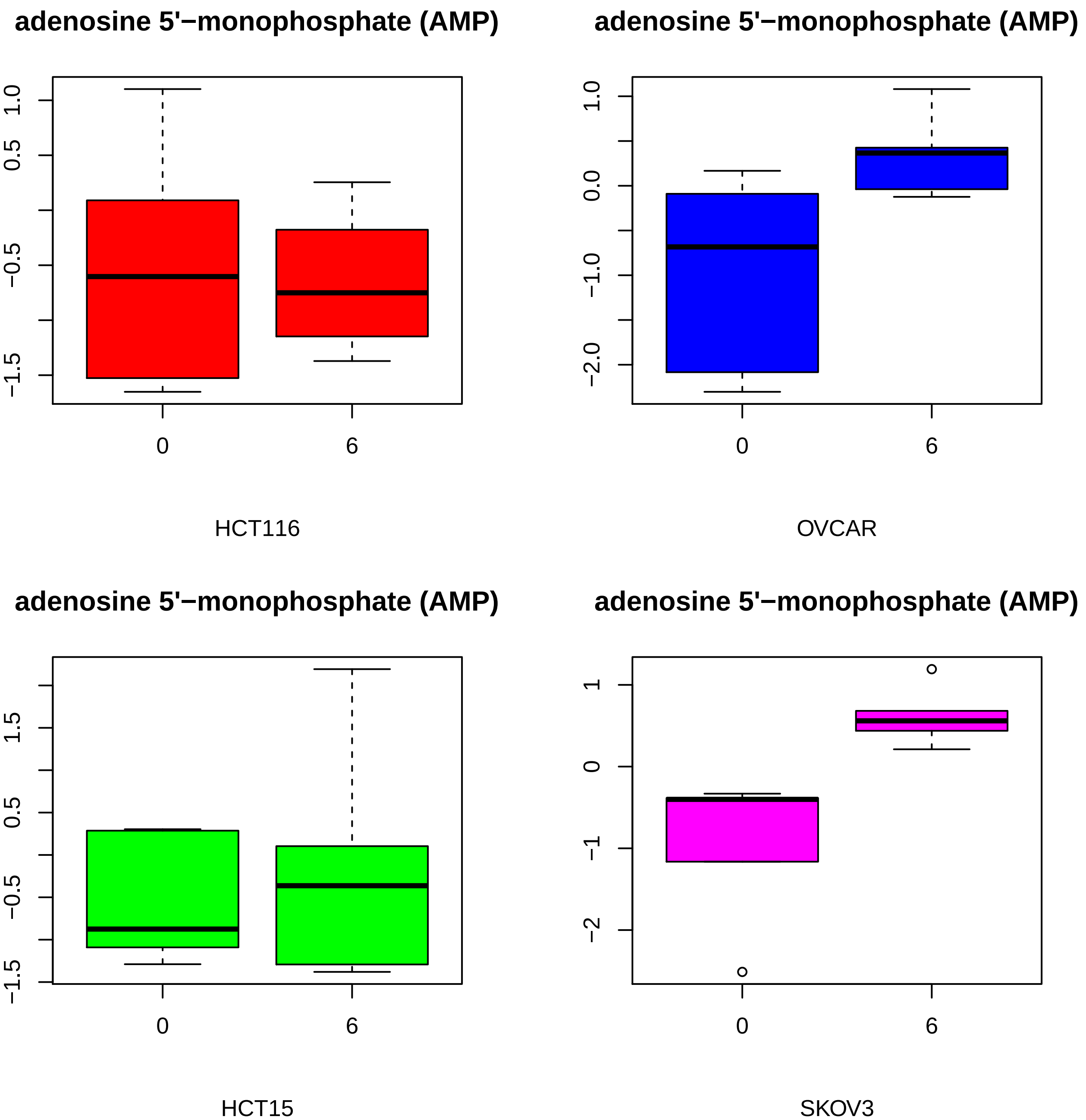
adenosine 5'−monophosphate (AMP)
HCT116
OVCAR
HCT15
SKOV3
0
6
1.0
0.5
−0.5
−1.5
0.0
−1.0
−2.0
1
0
−1
−2

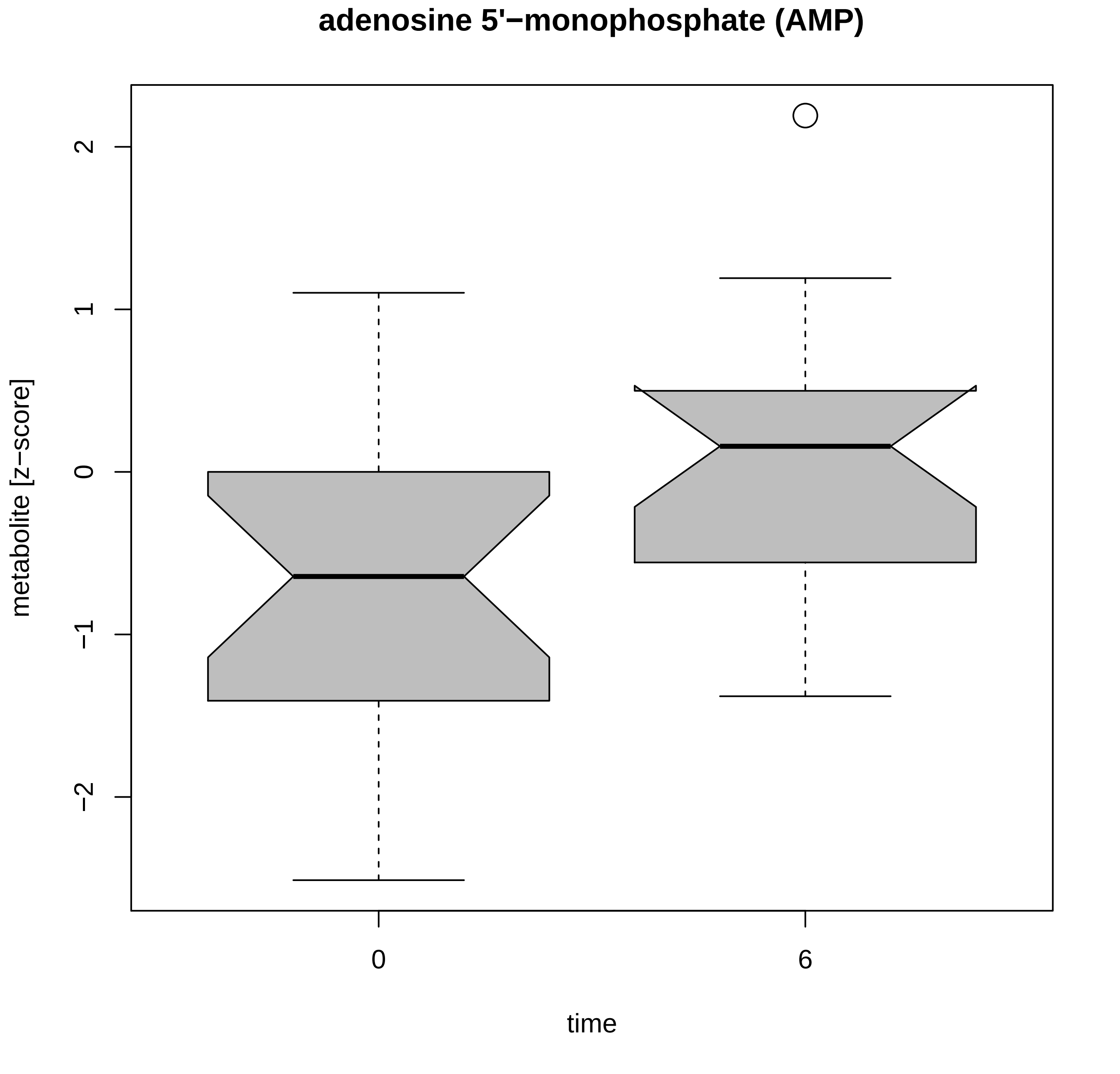
adenosine 5'−monophosphate (AMP)
metabolite [z−score]
2
1
0
−1
−2
0
6
time

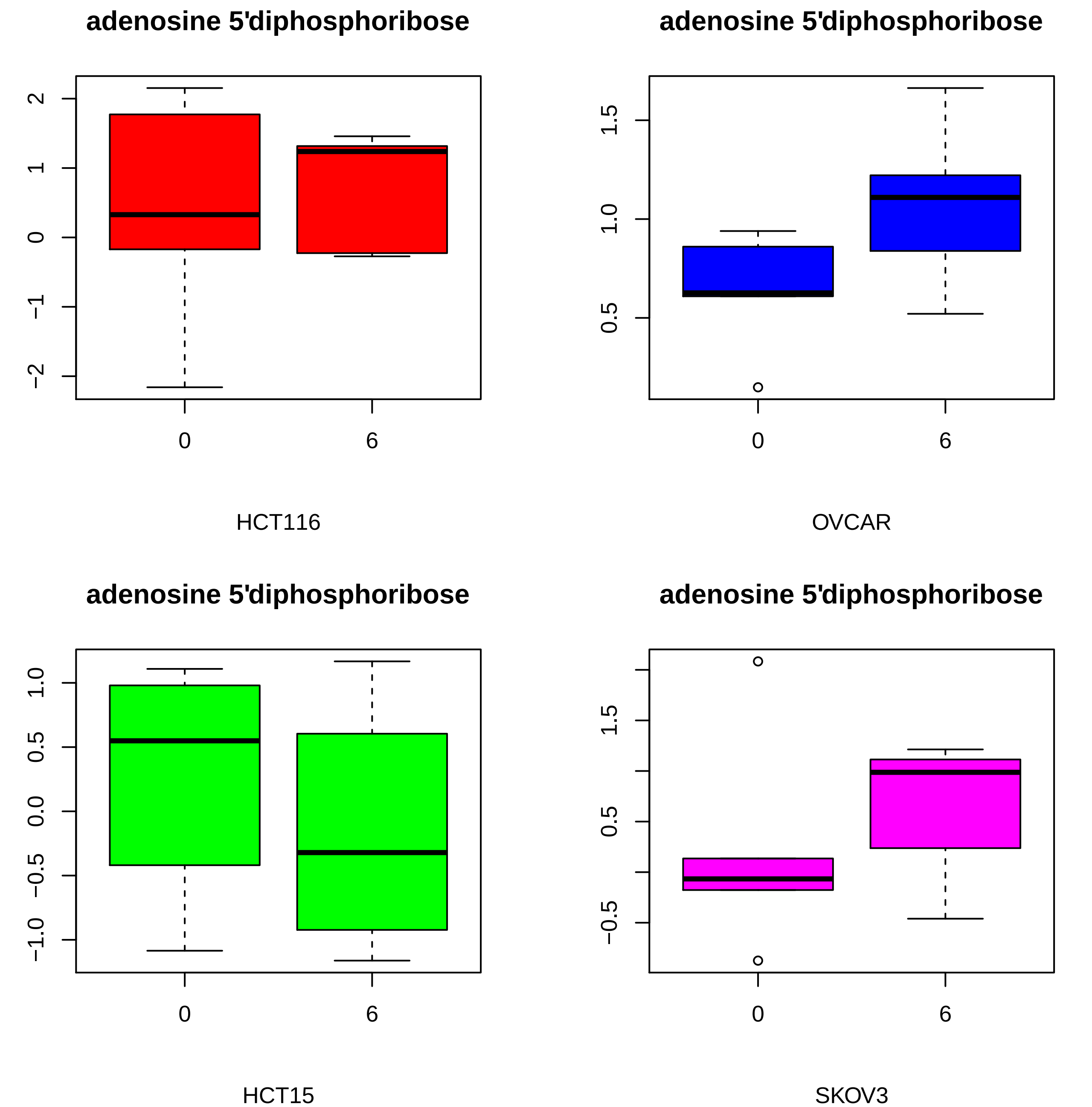
adenosine 5'diphosphoribose
2
1
0
−1
−2
0
6
HCT116
adenosine 5'diphosphoribose
1.5
1.0
0.5
0
6
OVCAR
adenosine 5'diphosphoribose
1.0
0.5
0.0
−0.5
−1.0
0
6
HCT15
adenosine 5'diphosphoribose
1.5
0.5
−0.5
0
6
SKOV3

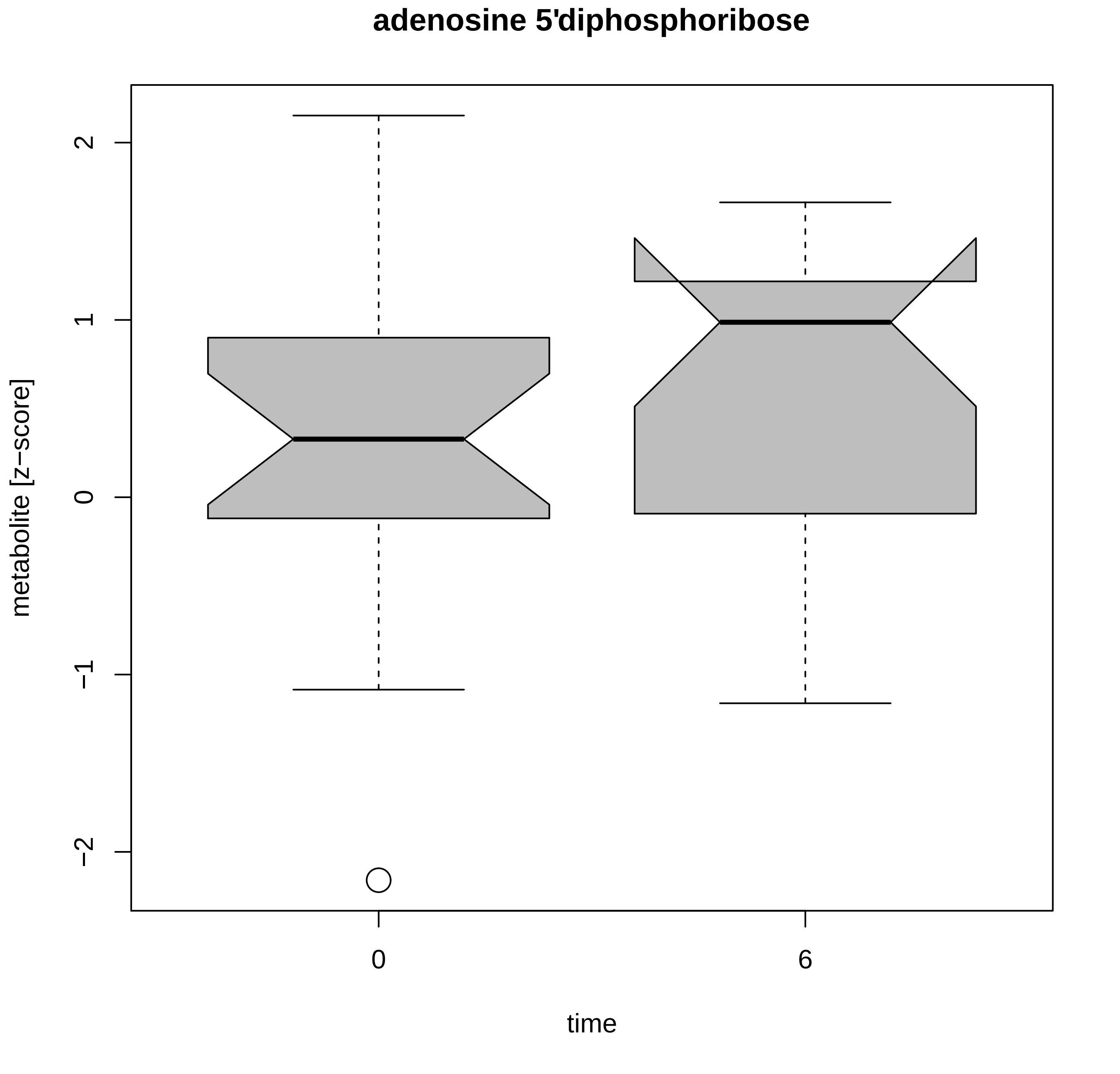
adenosine 5'diphosphoribose
metabolite [z−score]
2
1
0
−1
−2
0
6
time

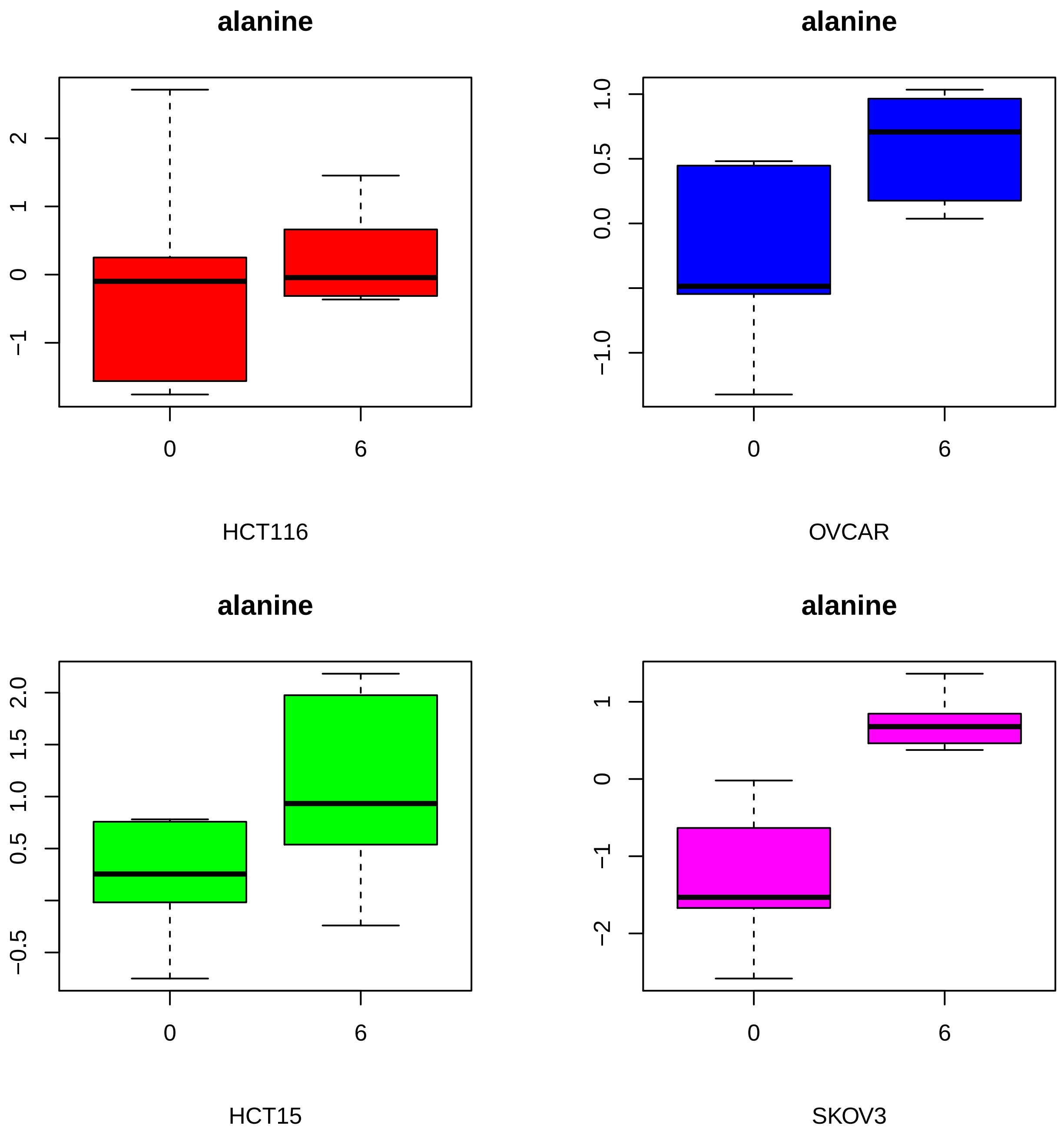

alanine
2
1
0
−1
0
6
HCT116
alanine
1.0
0.5
0.0
−1.0
0
6
OVCAR
alanine
2.0
1.5
1.0
0.5
−0.5
0
6
HCT15
alanine
1
0
−1
−2
0
6
SKOV3

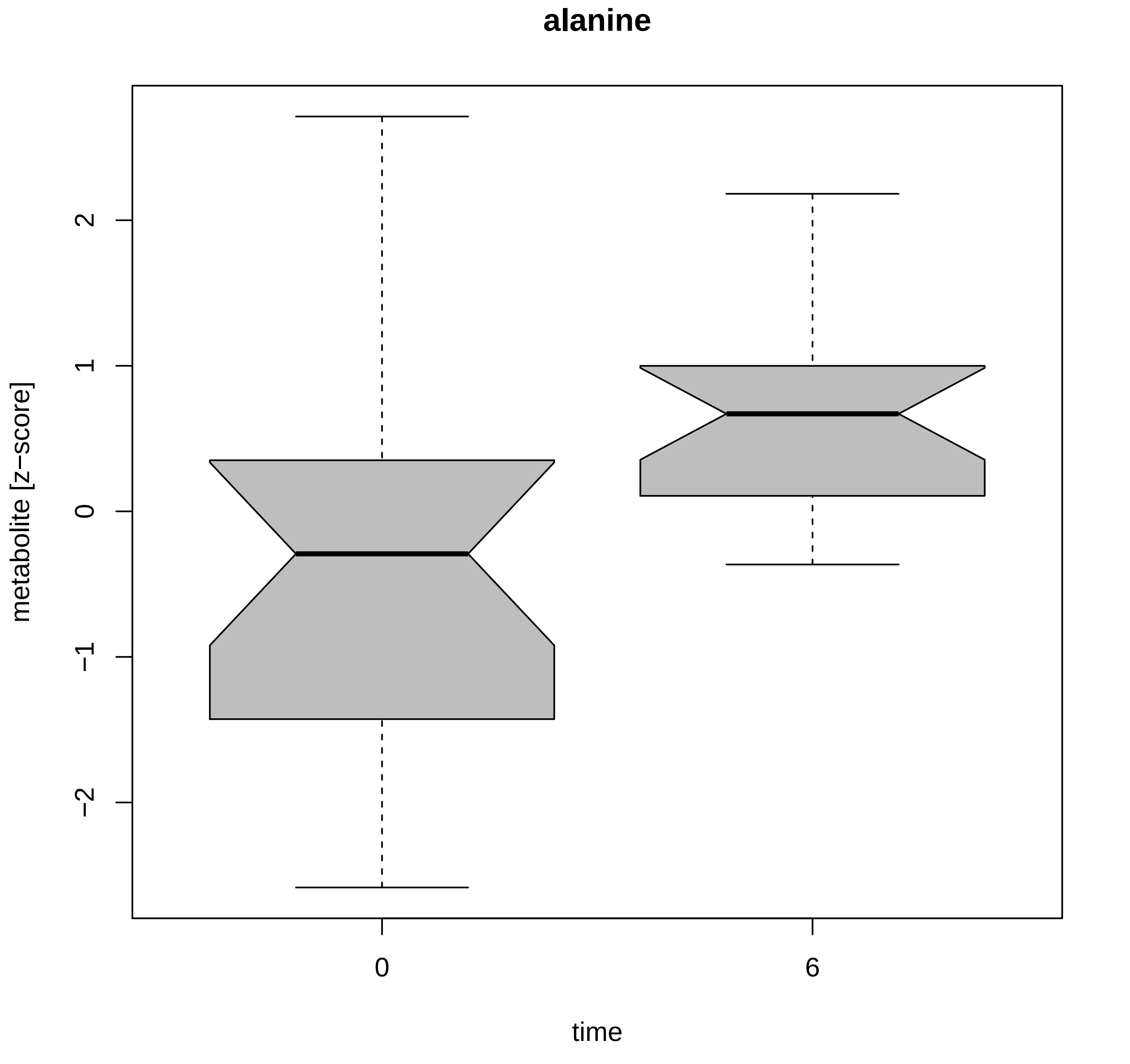
alanine
metabolite [z−score]
−2
−1
0
1
2
0
6
time

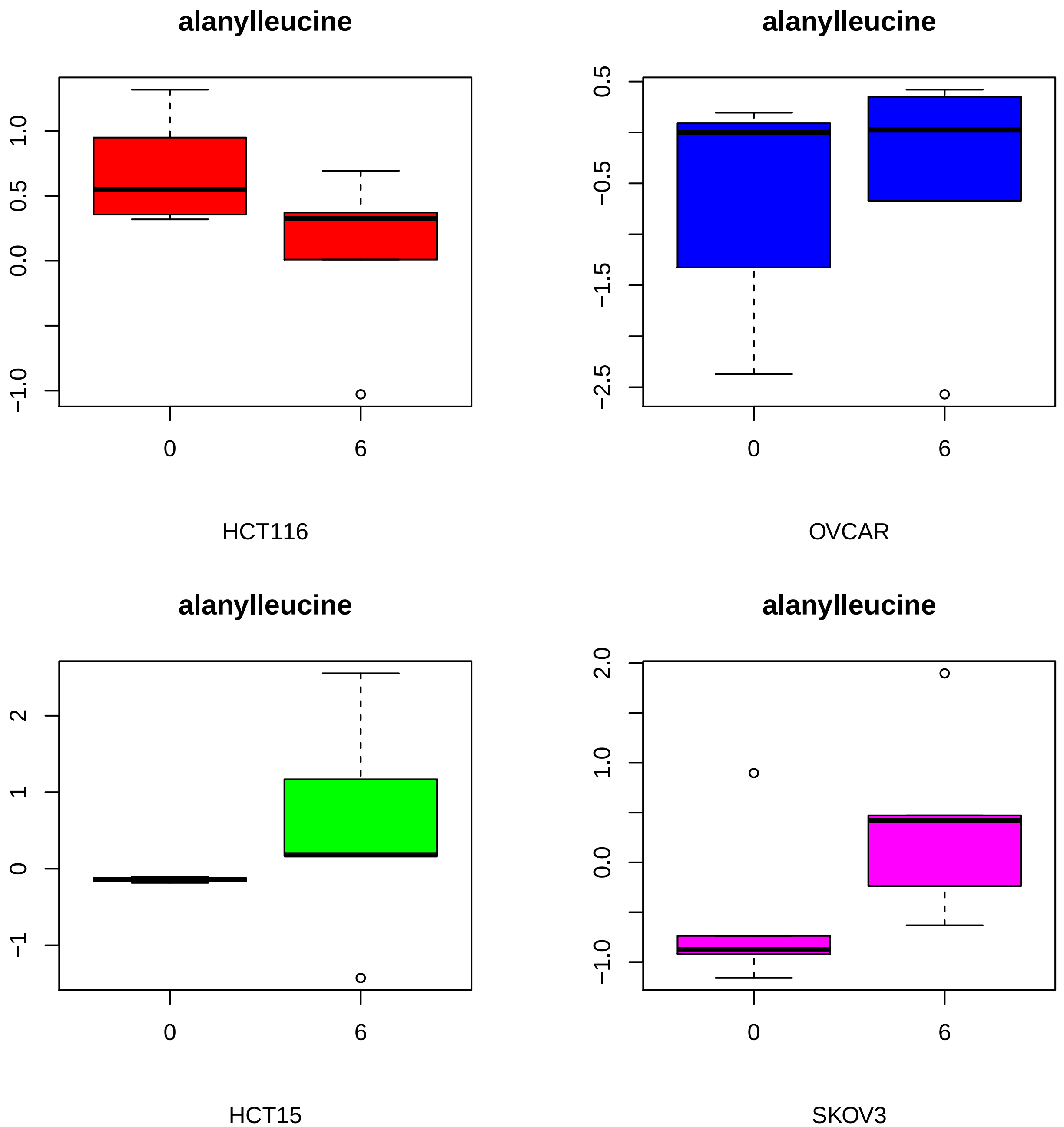
alanylleucine
1.0
0.5
0.0
−1.0
0
6
HCT116
alanylleucine
0.5
−0.5
−1.5
−2.5
0
6
OVCAR
alanylleucine
2
1
0
−1
0
6
HCT15
alanylleucine
2.0
1.0
0.0
−1.0
0
6
SKOV3

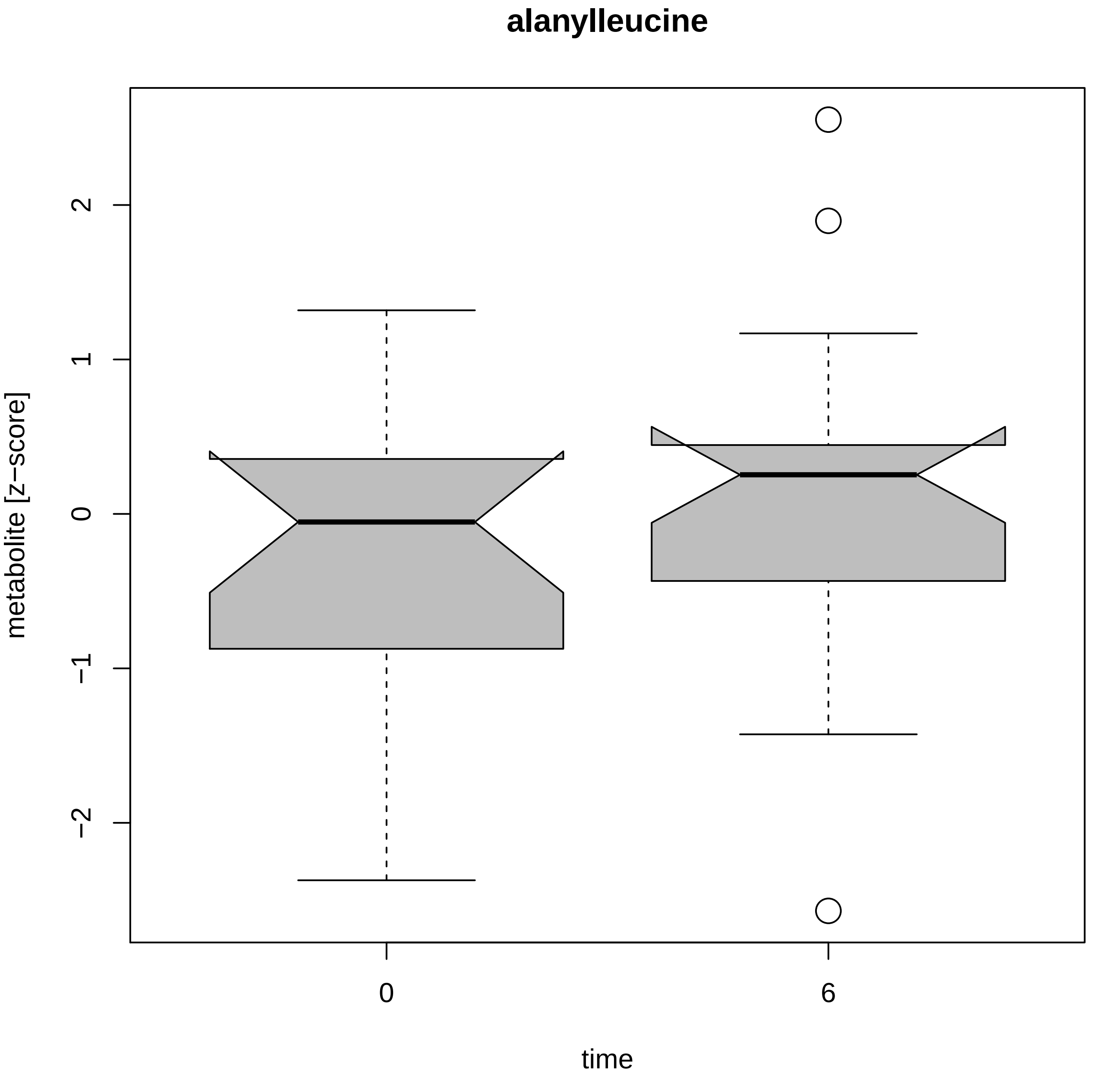
alanylleucine
metabolite [z−score]
2
1
0
−1
−2
0
6
time

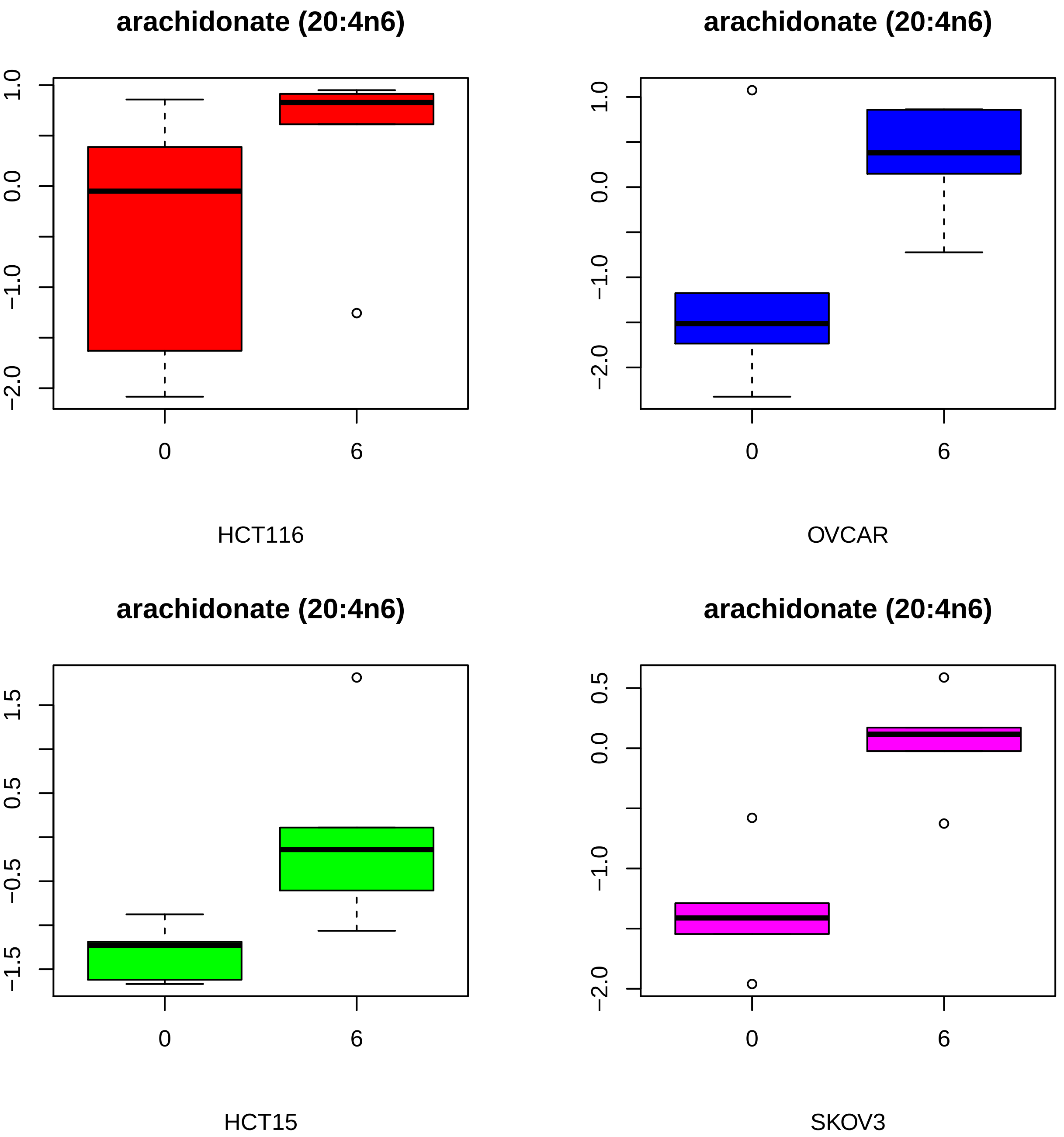
arachidonate (20:4n6)
1.0
0.0
−1.0
−2.0
0
6
HCT116
arachidonate (20:4n6)
1.0
0.0
−1.0
−2.0
0
6
OVCAR
arachidonate (20:4n6)
1.5
0.5
−0.5
−1.5
0
6
HCT15
arachidonate (20:4n6)
0.5
0.0
−1.0
−2.0
0
6
SKOV3

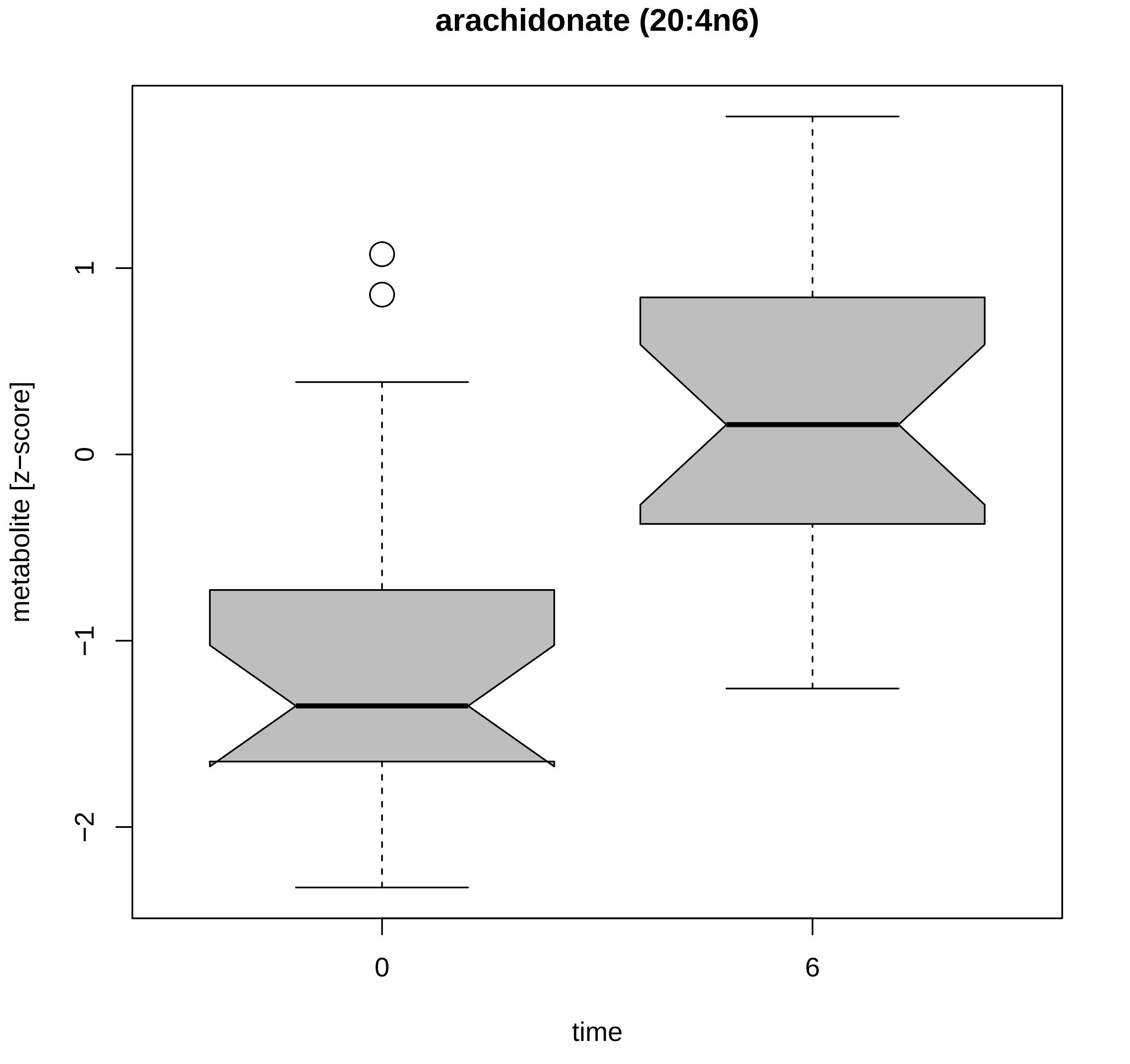
arachidonate (20:4n6)
metabolite [z−score]
1
0
−1
−2
0
6
time

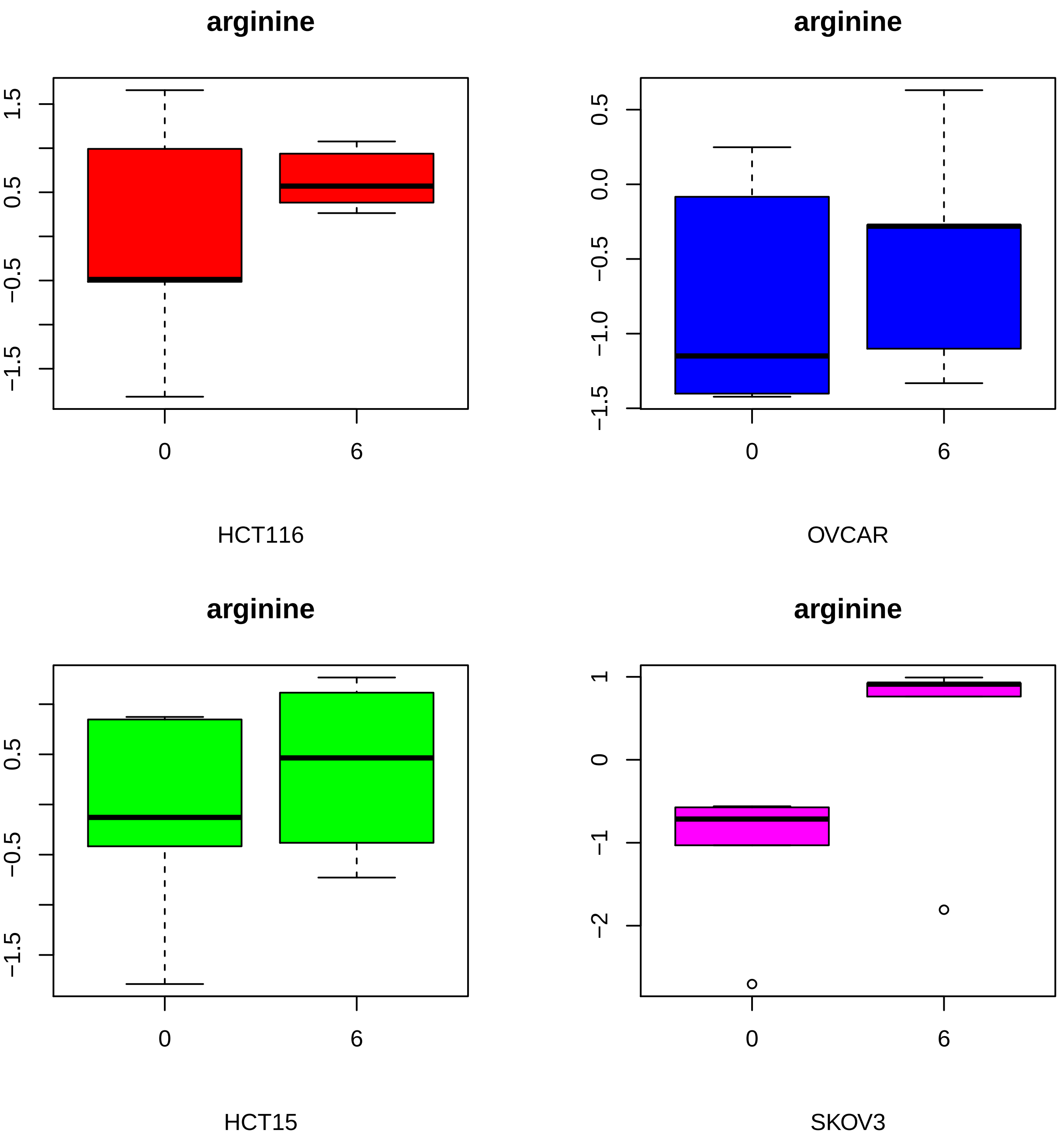
arginine
1.5
0.5
−0.5
−1.5
0
6
HCT116
arginine
0.5
0.0
−0.5
−1.0
−1.5
0
6
OVCAR
arginine
0.5
−0.5
−1.5
0
6
HCT15
arginine
1
0
−1
−2
0
6
SKOV3

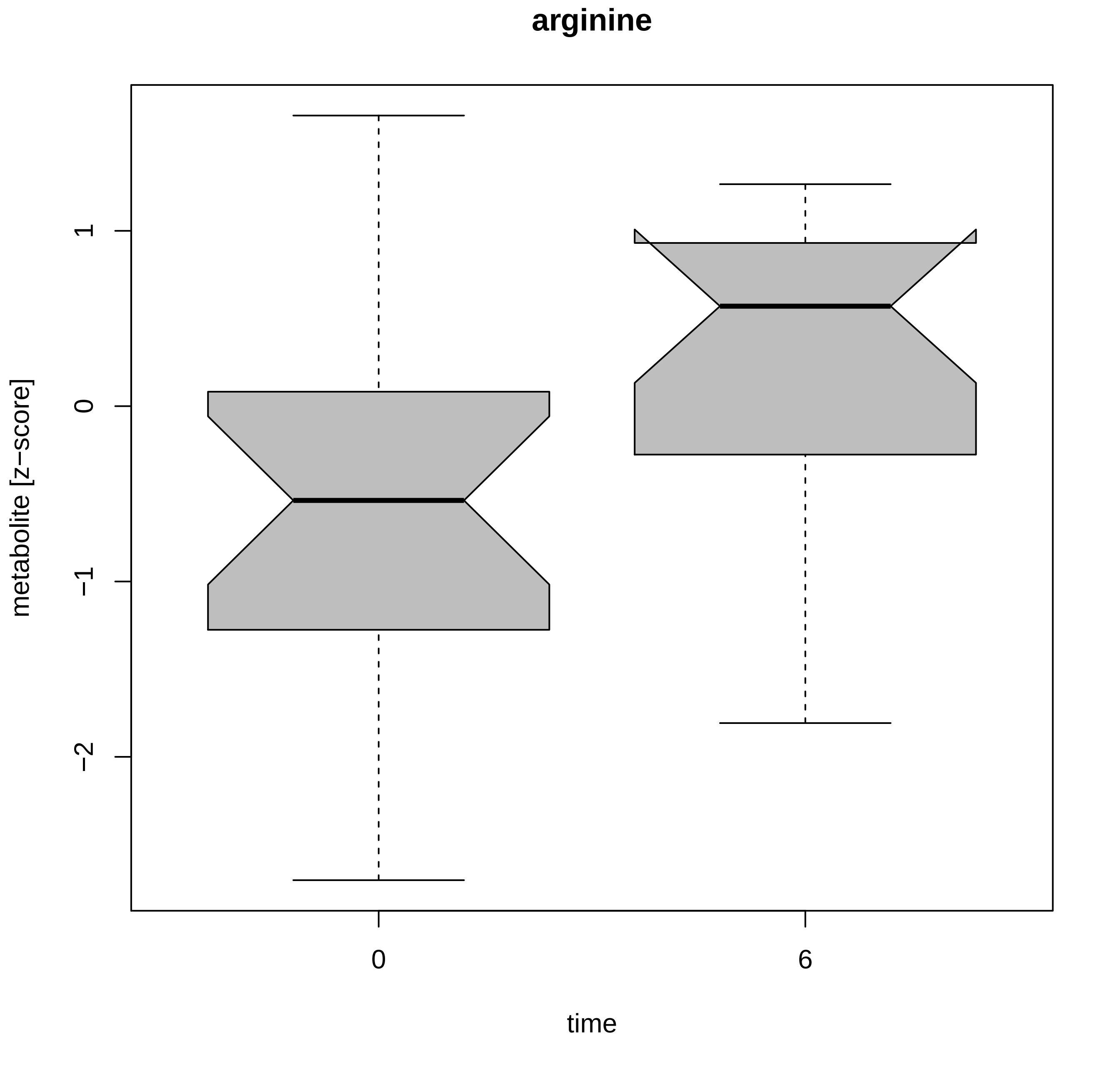
arginine
metabolite [z−score]
1
0
−1
−2
0
6
time

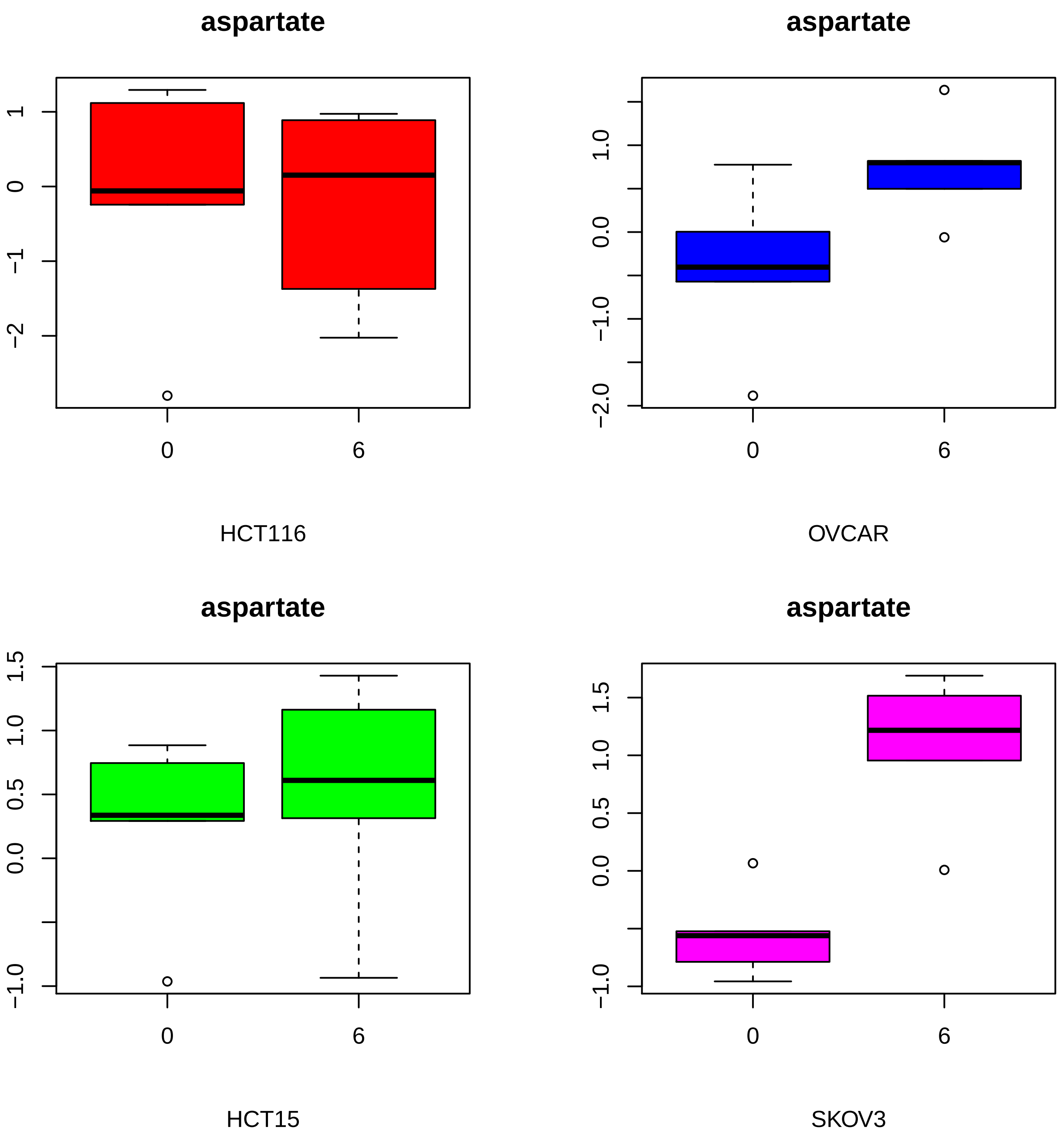
aspartate
1
0
−1
−2
0
6
HCT116
aspartate
1.0
0.0
−1.0
−2.0
0
6
OVCAR
aspartate
1.5
1.0
0.5
0.0
−1.0
0
6
HCT15
aspartate
1.5
1.0
0.5
0.0
−1.0
0
6
SKOV3

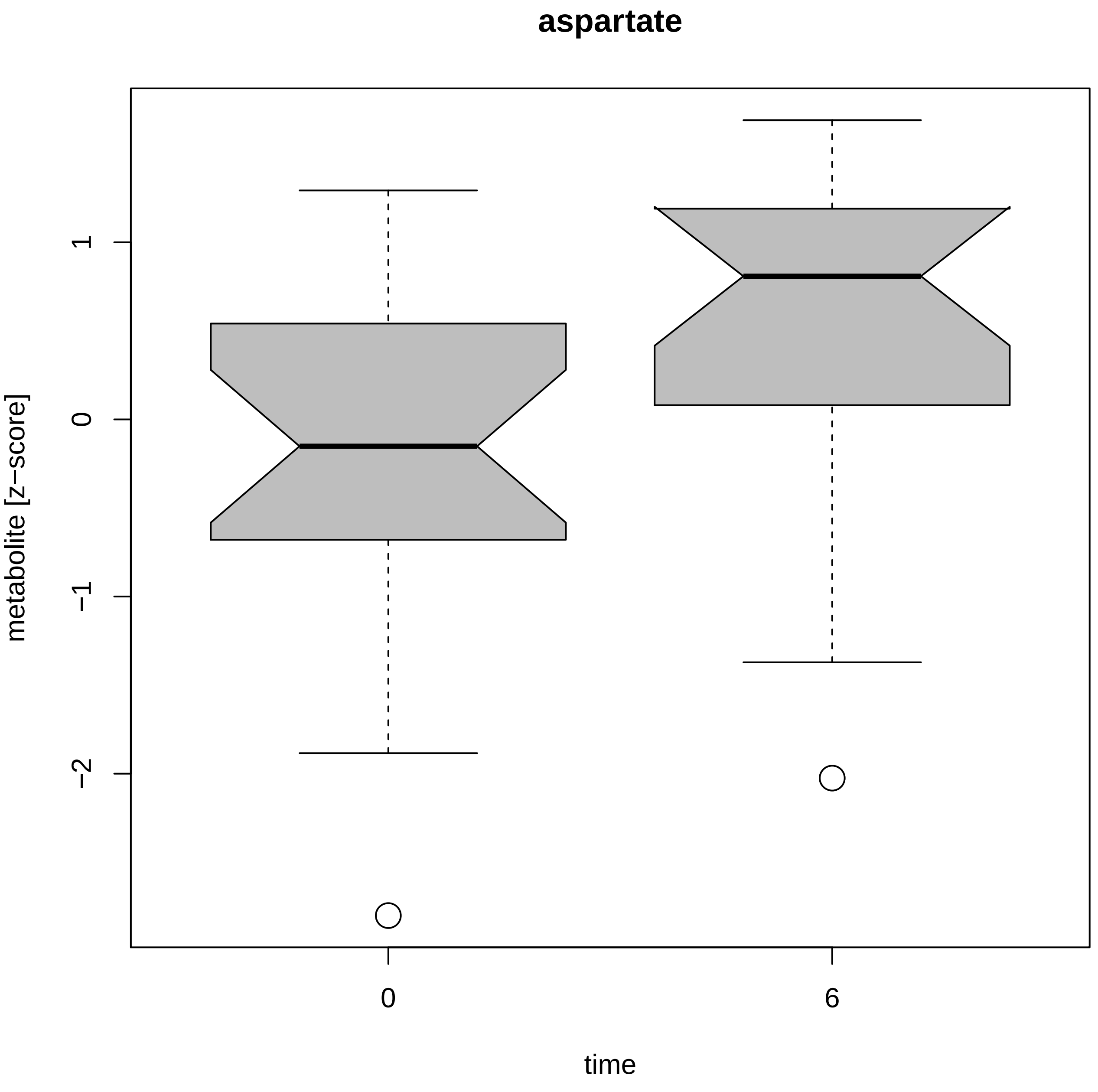
aspartate
metabolite [z−score]
1
0
−1
−2
0
6
time

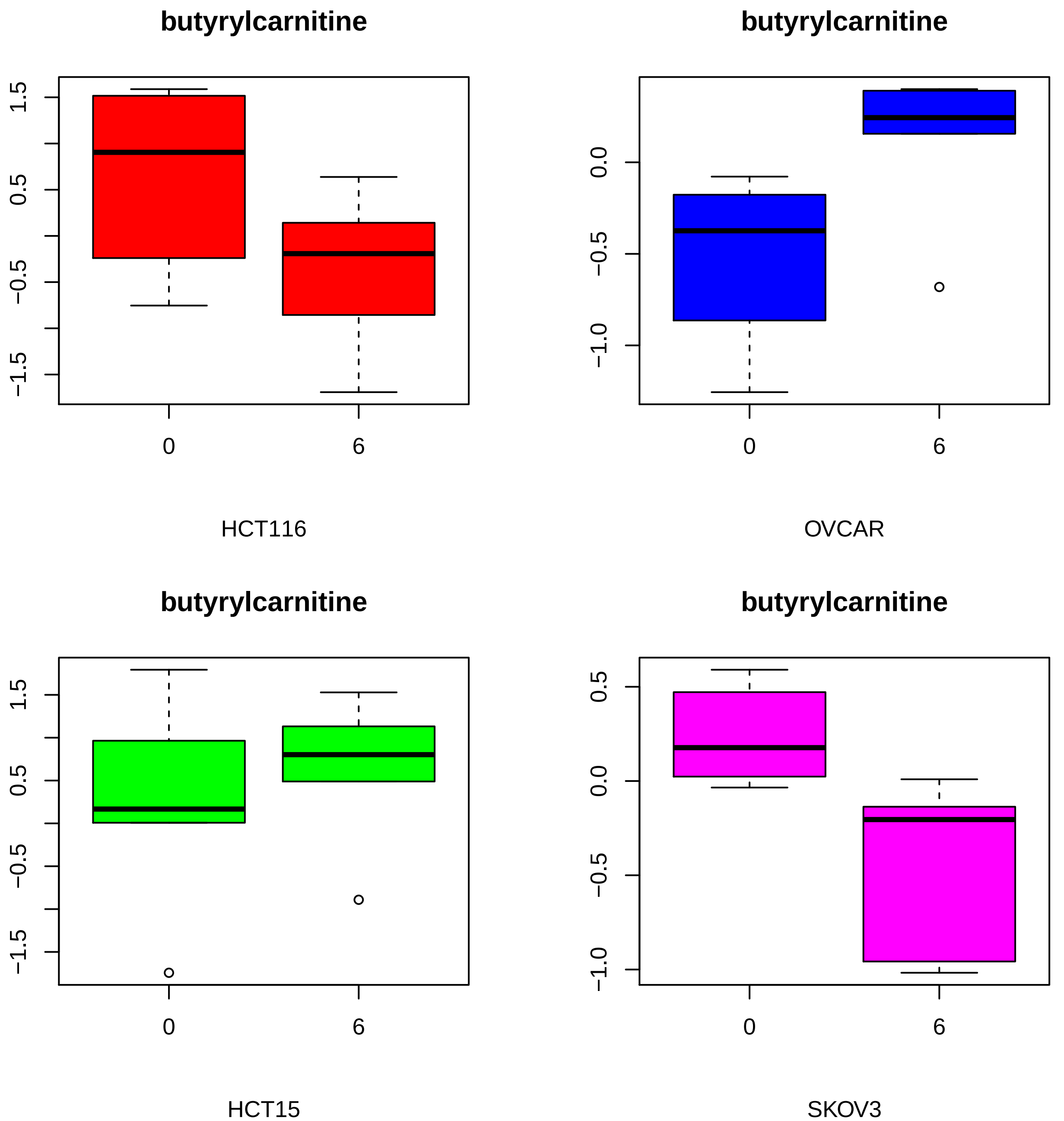
butyrylcarnitine
1.5
0.5
−0.5
−1.5
0
6
HCT116
butyrylcarnitine
0.0
−0.5
−1.0
0
6
OVCAR
butyrylcarnitine
1.5
0.5
−0.5
−1.5
0
6
HCT15
butyrylcarnitine
0.5
0.0
−0.5
−1.0
0
6
SKOV3

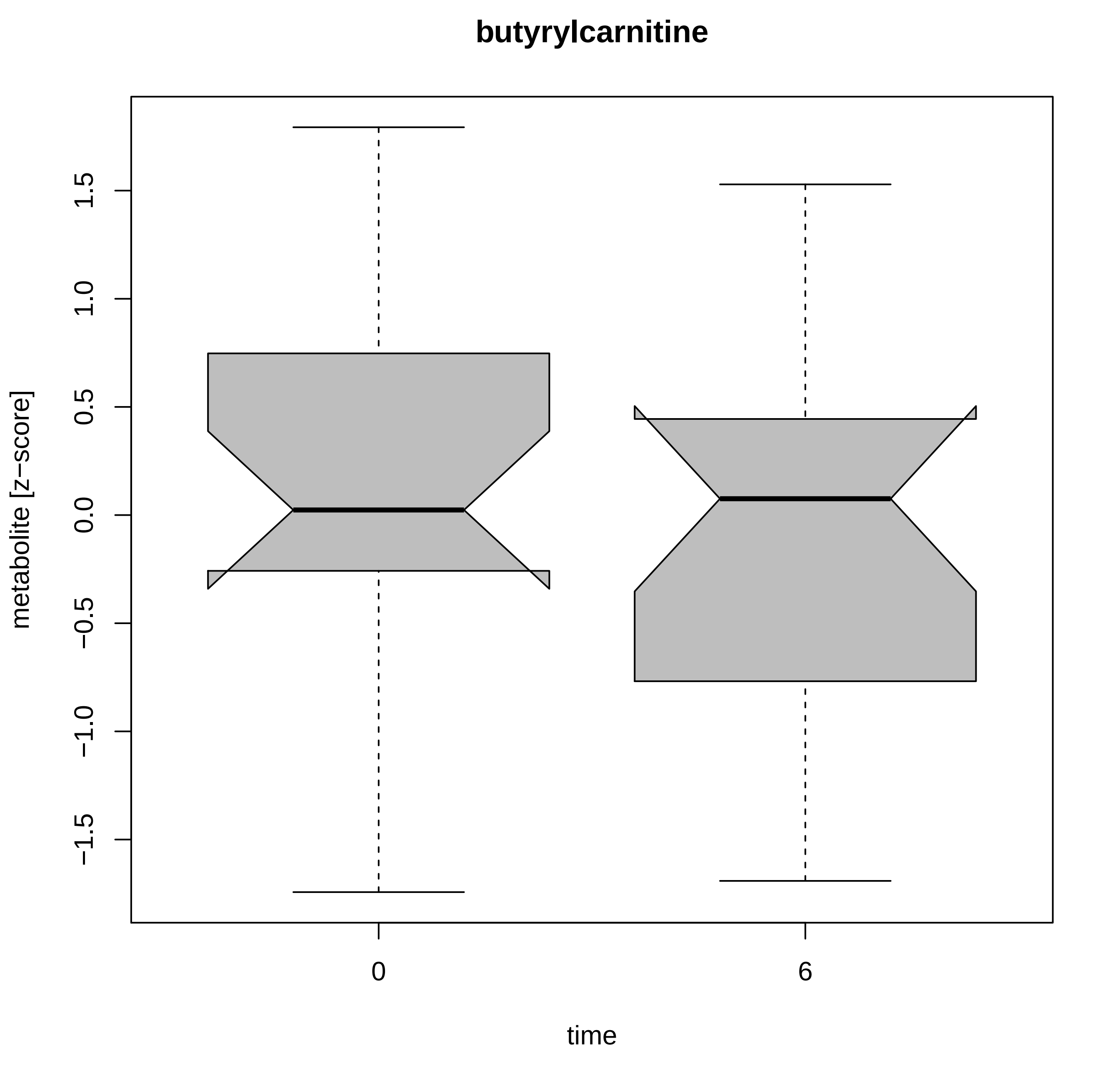
butyrylcarnitine
metabolite [z−score]
1.5
1.0
0.5
0.0
−0.5
−1.0
−1.5
0
6
time

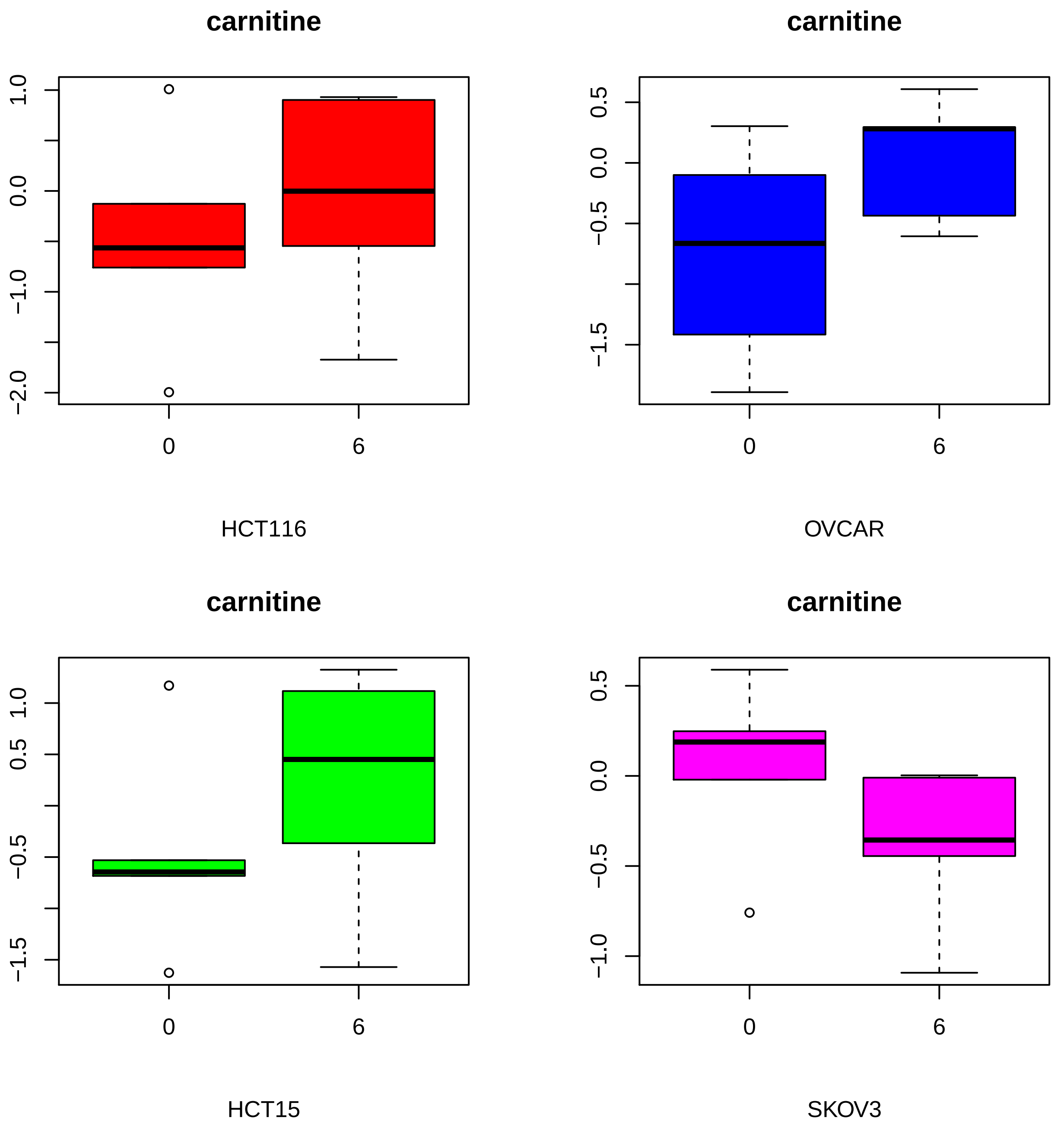
carnitine
1.0
0.0
−1.0
−2.0
0
6
HCT116
carnitine
0.5
0.0
−0.5
−1.5
0
6
OVCAR
carnitine
1.0
0.5
−0.5
−1.5
0
6
HCT15
carnitine
0.5
0.0
−0.5
−1.0
0
6
SKOV3

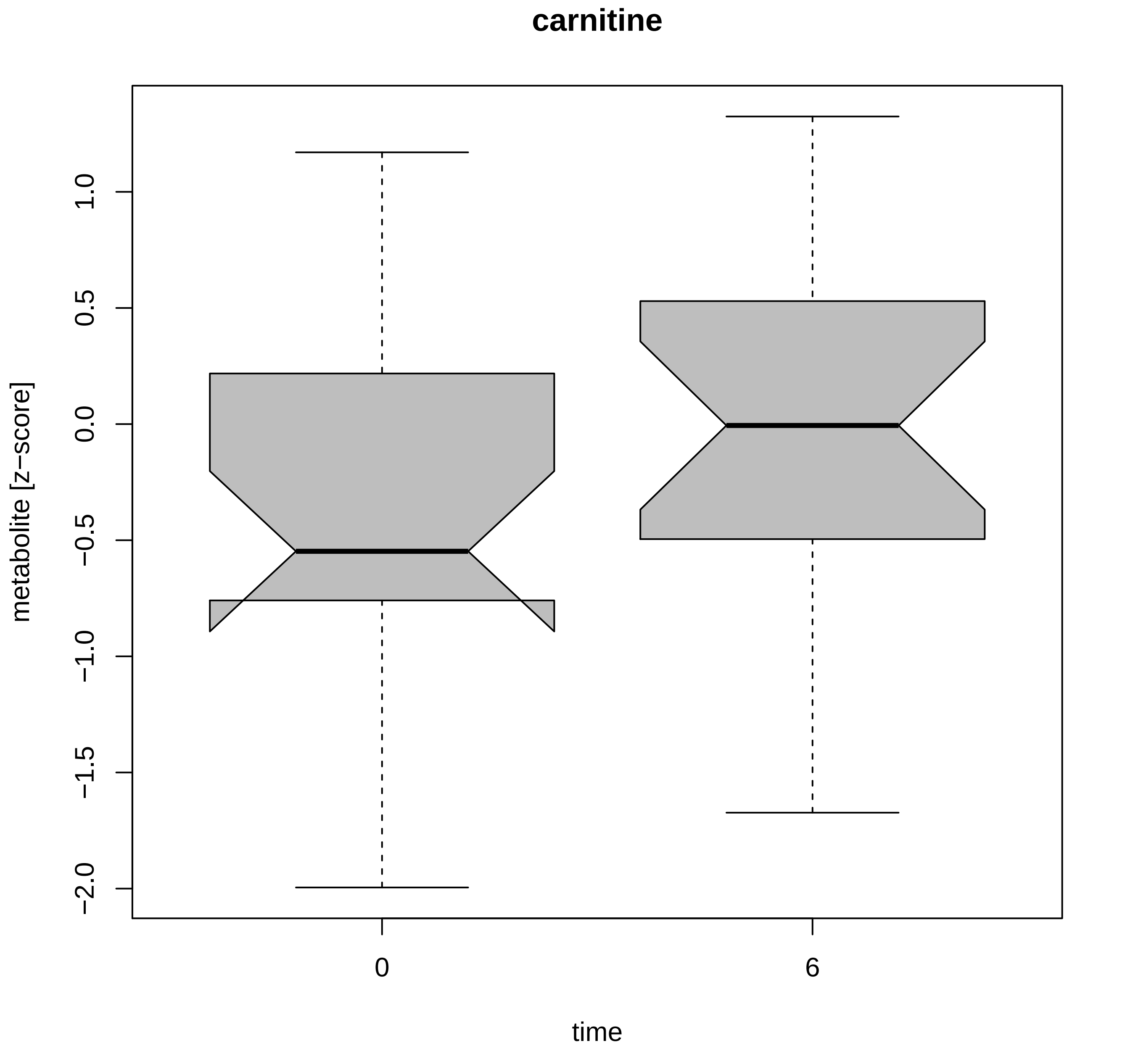

carnitine
metabolite [z−score]
1.0
0.5
0.0
−0.5
−1.0
−1.5
−2.0
0
6
time

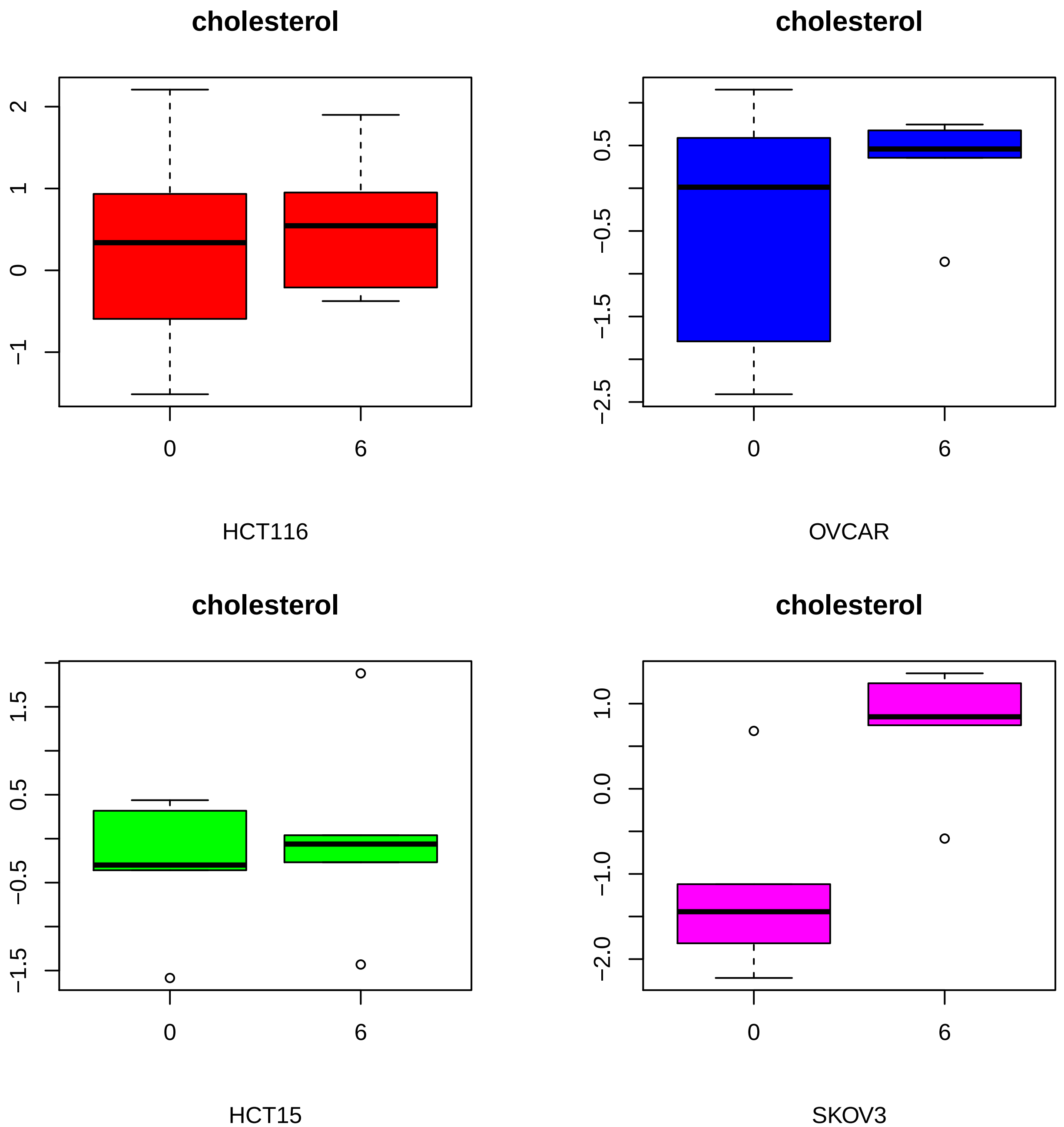
cholesterol
2
1
0
−1
0
6
HCT116
cholesterol
0.5
−0.5
−1.5
−2.5
0
6
OVCAR
cholesterol
1.5
0.5
−0.5
−1.5
0
6
HCT15
cholesterol
1.0
0.0
−1.0
−2.0
0
6
SKOV3

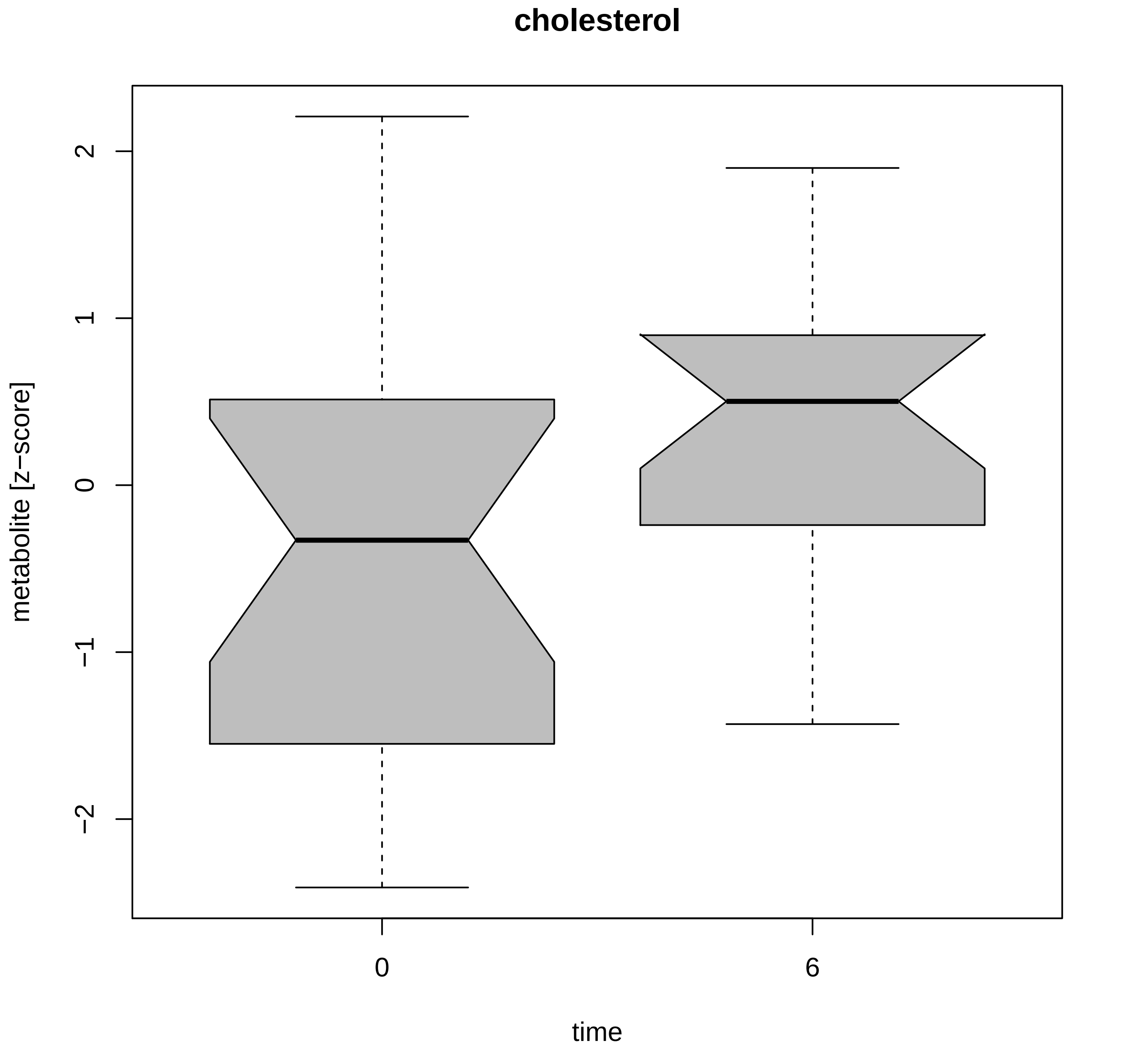
cholesterol
metabolite [z−score]
2
1
0
−1
−2
0
6
time

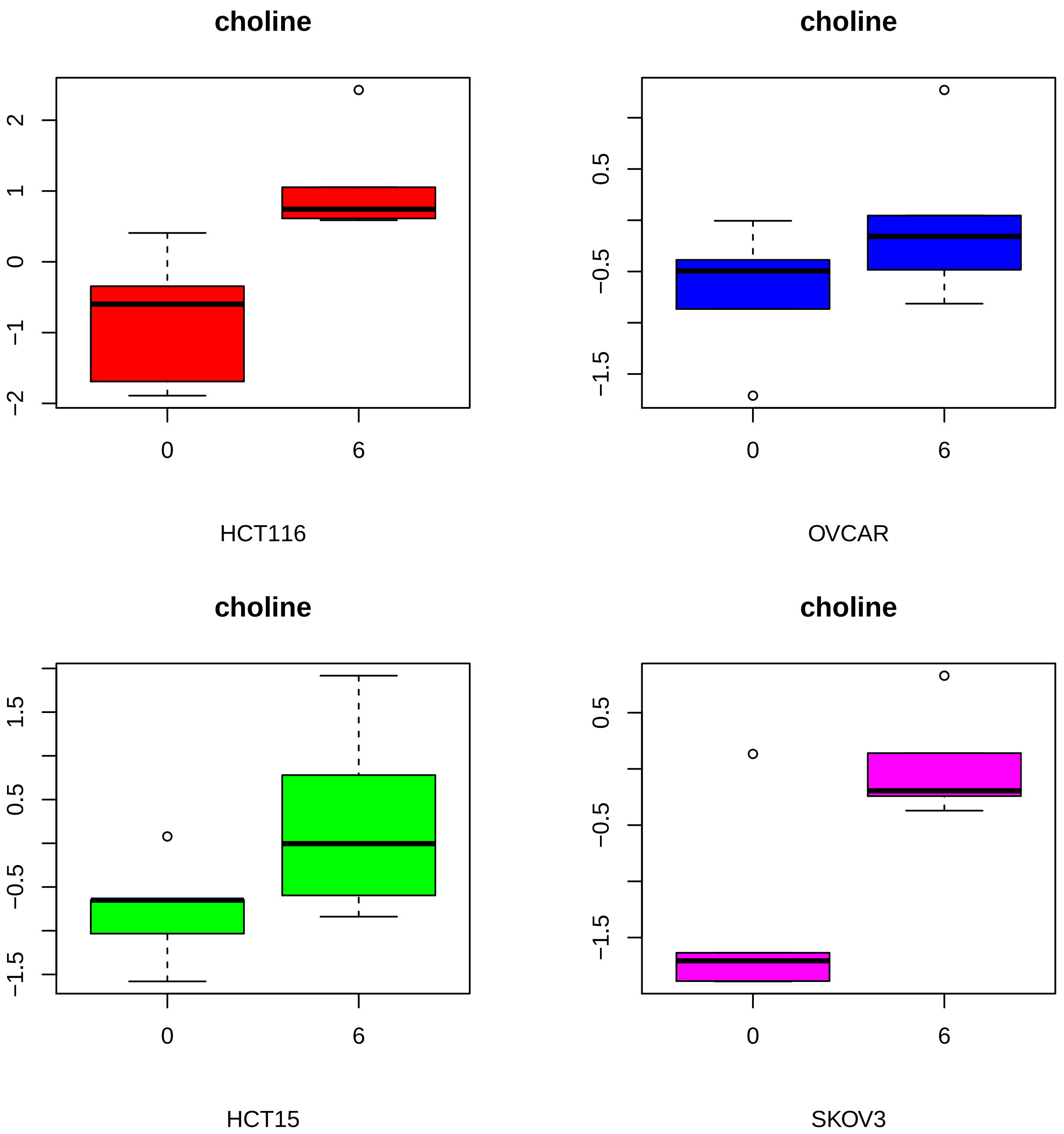
choline
HCT116
choline
OVCAR
choline
HCT15
choline
SKOV3
0
6
2
1
0
−1
−2
0.5
−0.5
−1.5
1.5

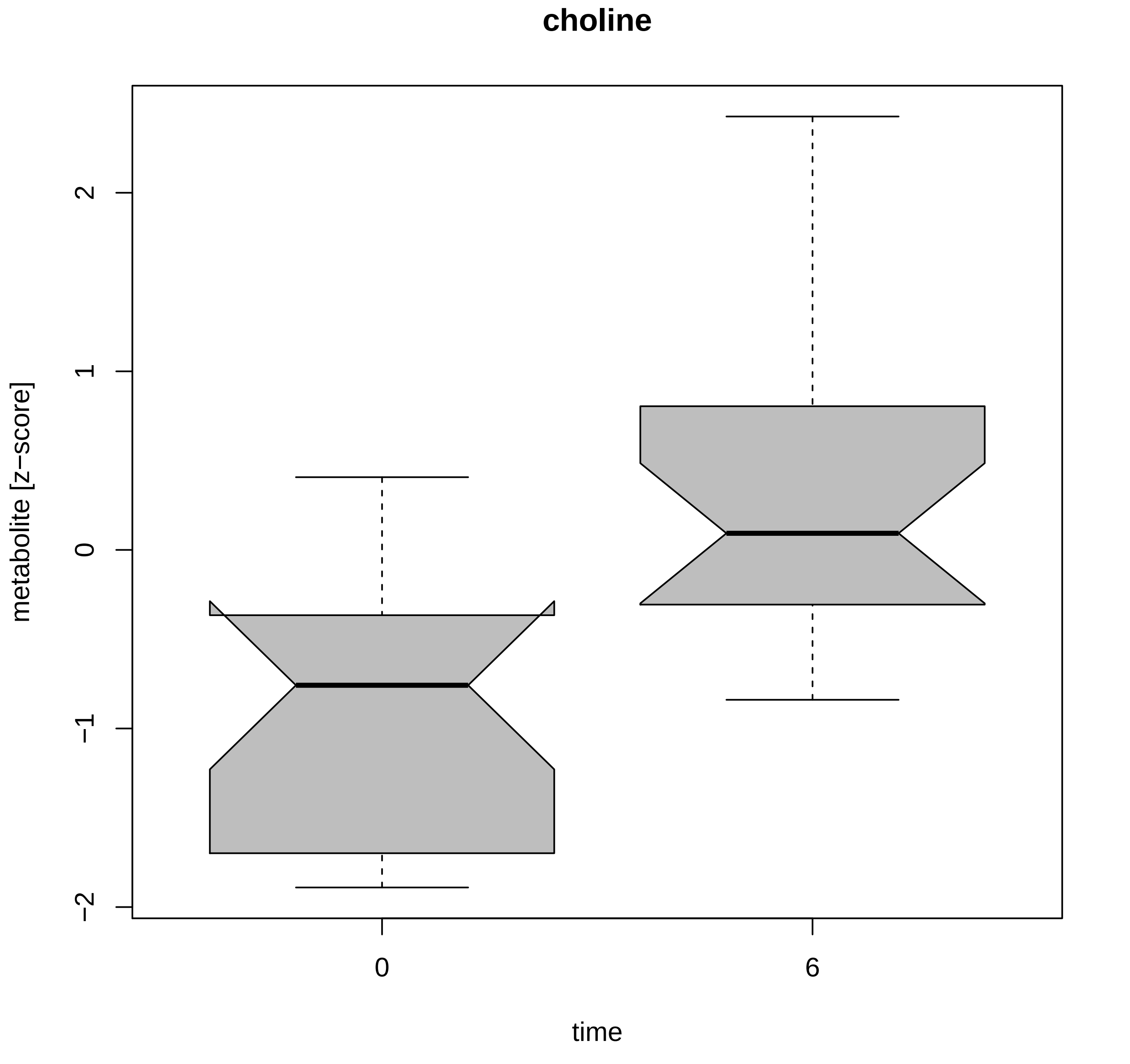
choline
metabolite [z−score]
2
1
0
−1
−2
0
6
time

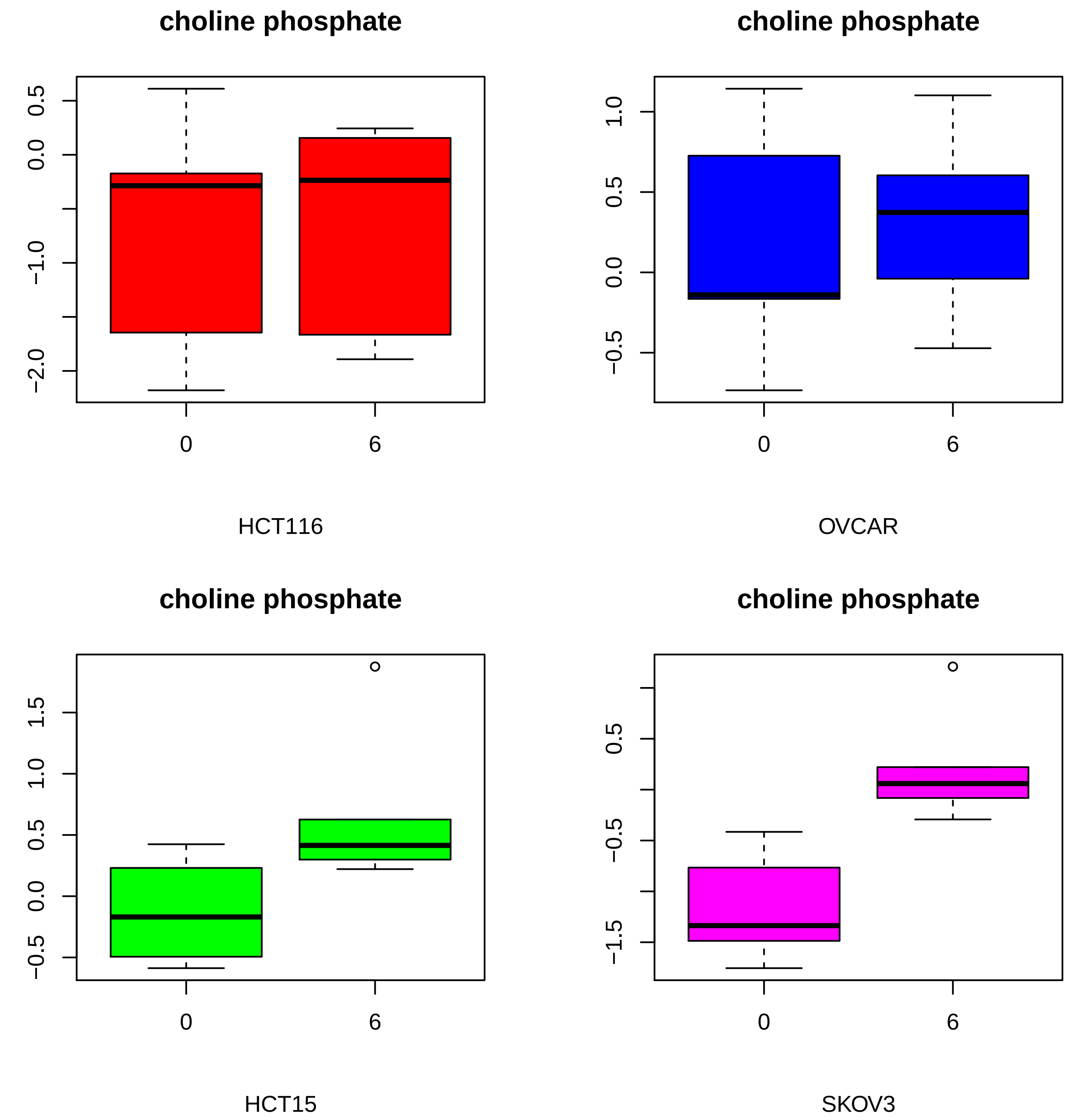
choline phosphate
0.5
0.0
−1.0
−2.0
0
6
HCT116
choline phosphate
1.0
0.5
0.0
−0.5
0
6
OVCAR
choline phosphate
1.5
1.0
0.5
0.0
−0.5
0
6
HCT15
choline phosphate
0.5
−0.5
−1.5
0
6
SKOV3

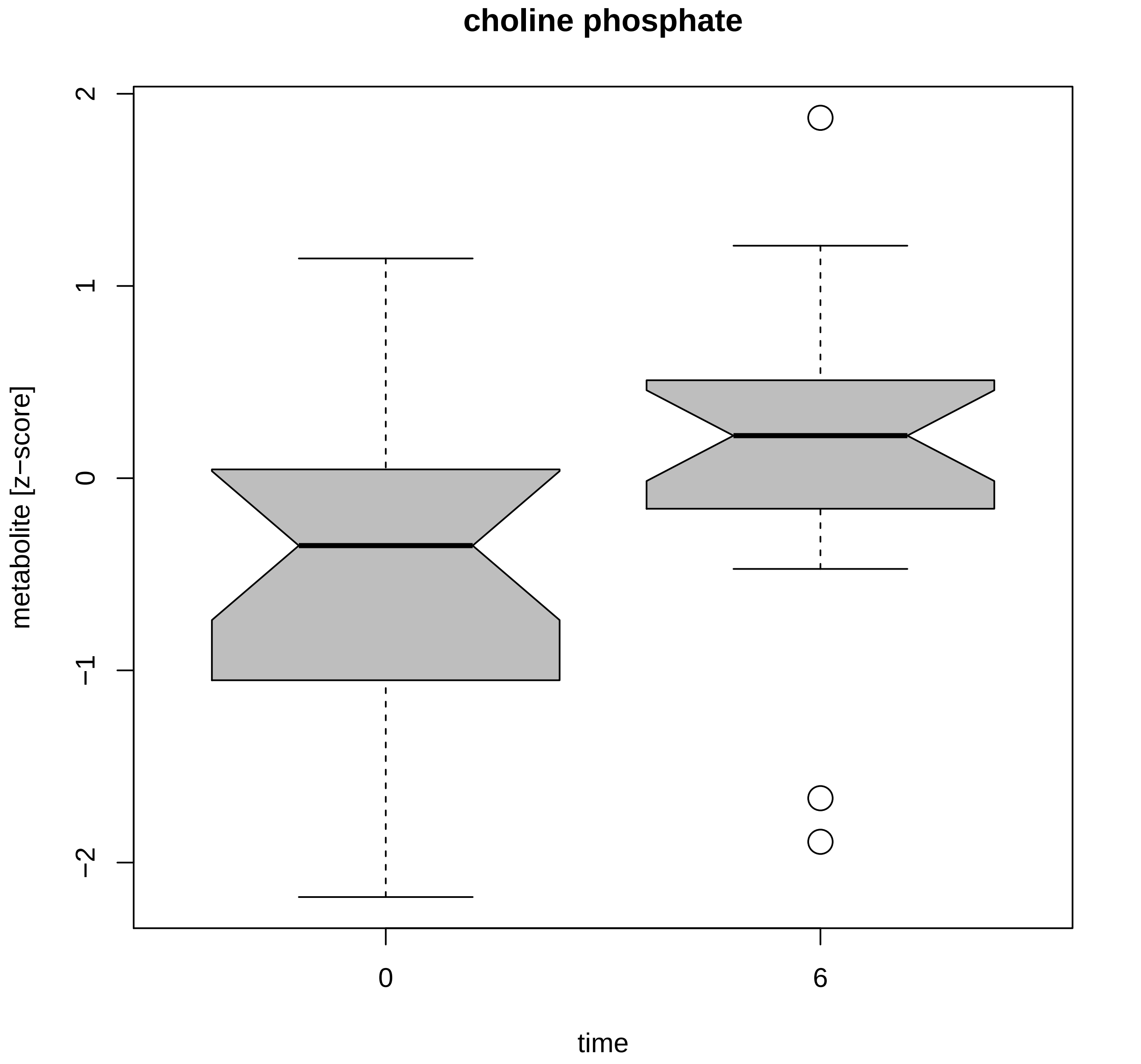

choline phosphate
metabolite [z−score]
2
1
0
−1
−2
0
6
time

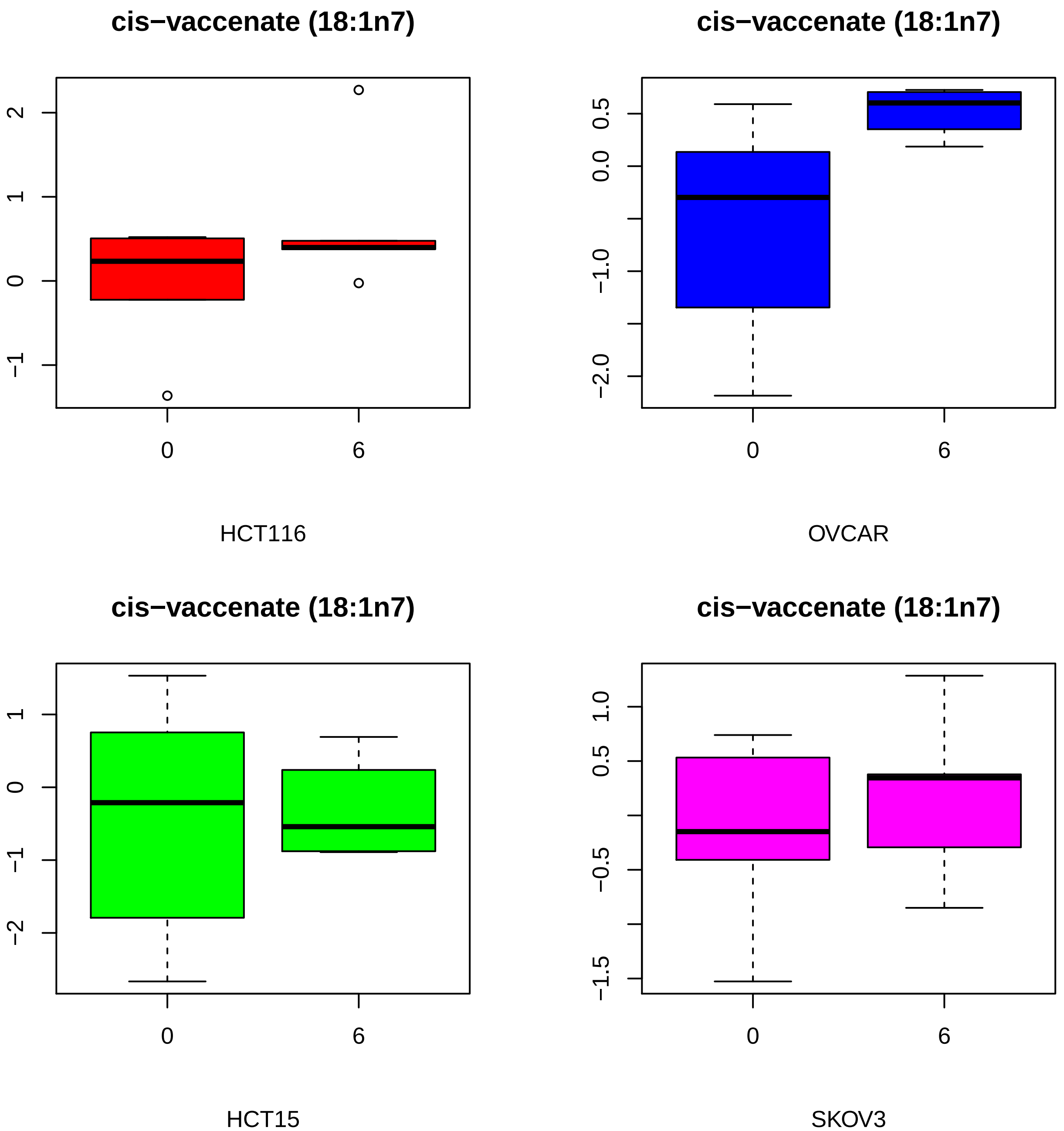

cis−vaccenate (18:1n7)
HCT116
cis−vaccenate (18:1n7)
OVCAR
cis−vaccenate (18:1n7)
HCT15
cis−vaccenate (18:1n7)
SKOV3
0
6

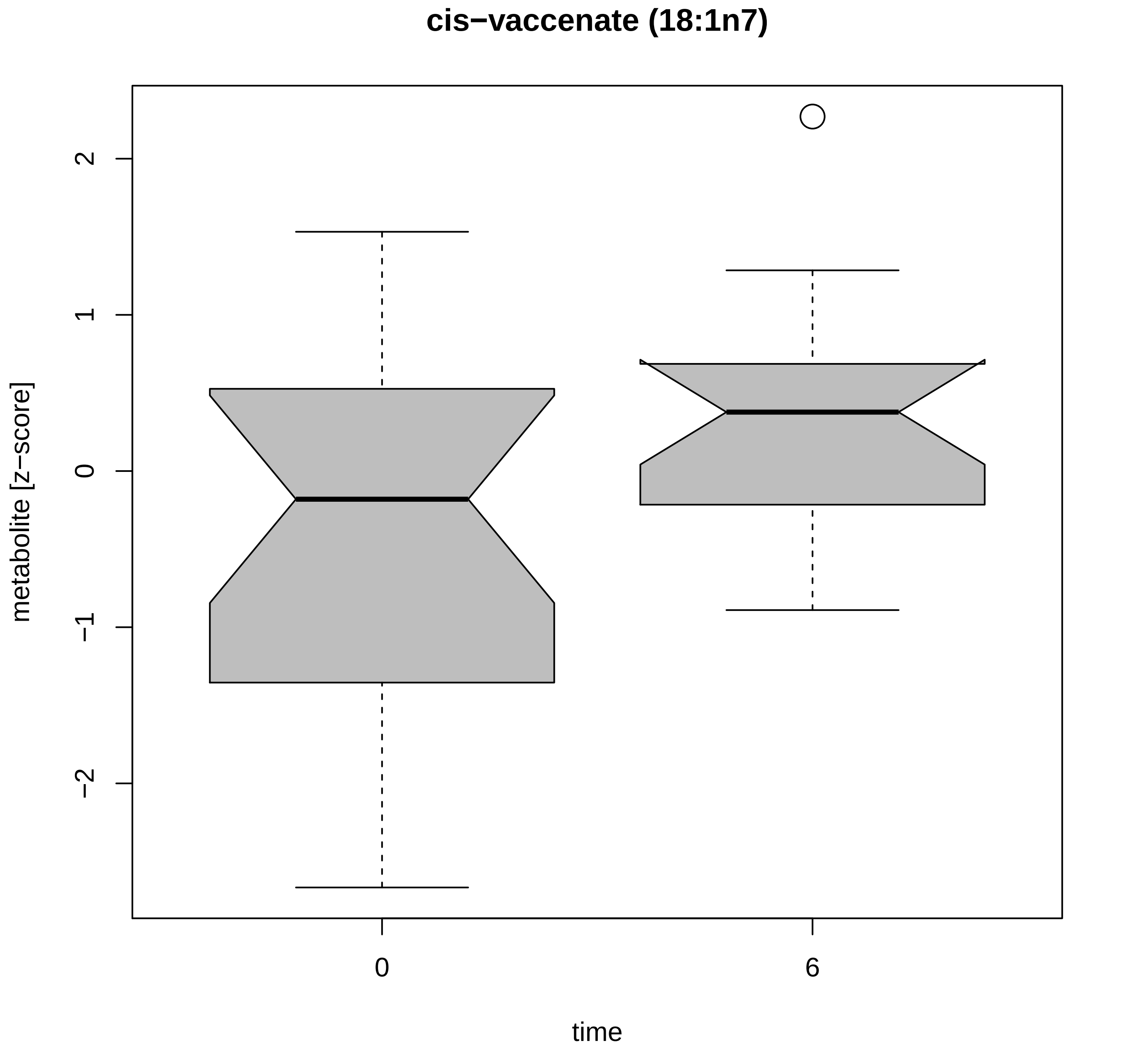

cis−vaccenate (18:1n7)
metabolite [z−score]
2
1
0
−1
−2
0
6
time

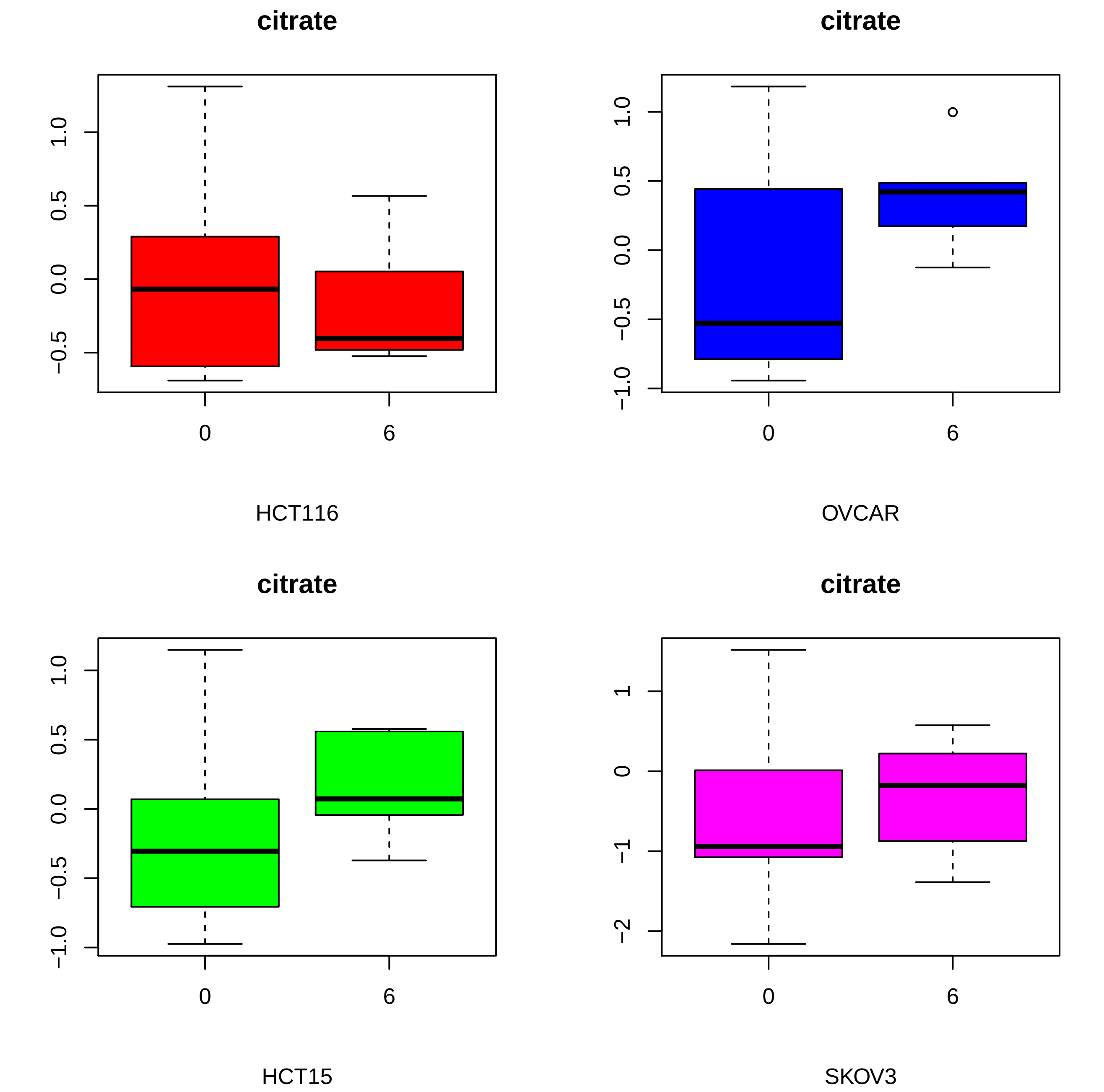
citrate
1.0
0.5
0.0
−0.5
0
6
HCT116
citrate
1.0
0.5
0.0
−0.5
−1.0
0
6
OVCAR
citrate
1.0
0.5
0.0
−0.5
−1.0
0
6
HCT15
citrate
1
0
−1
−2
0
6
SKOV3

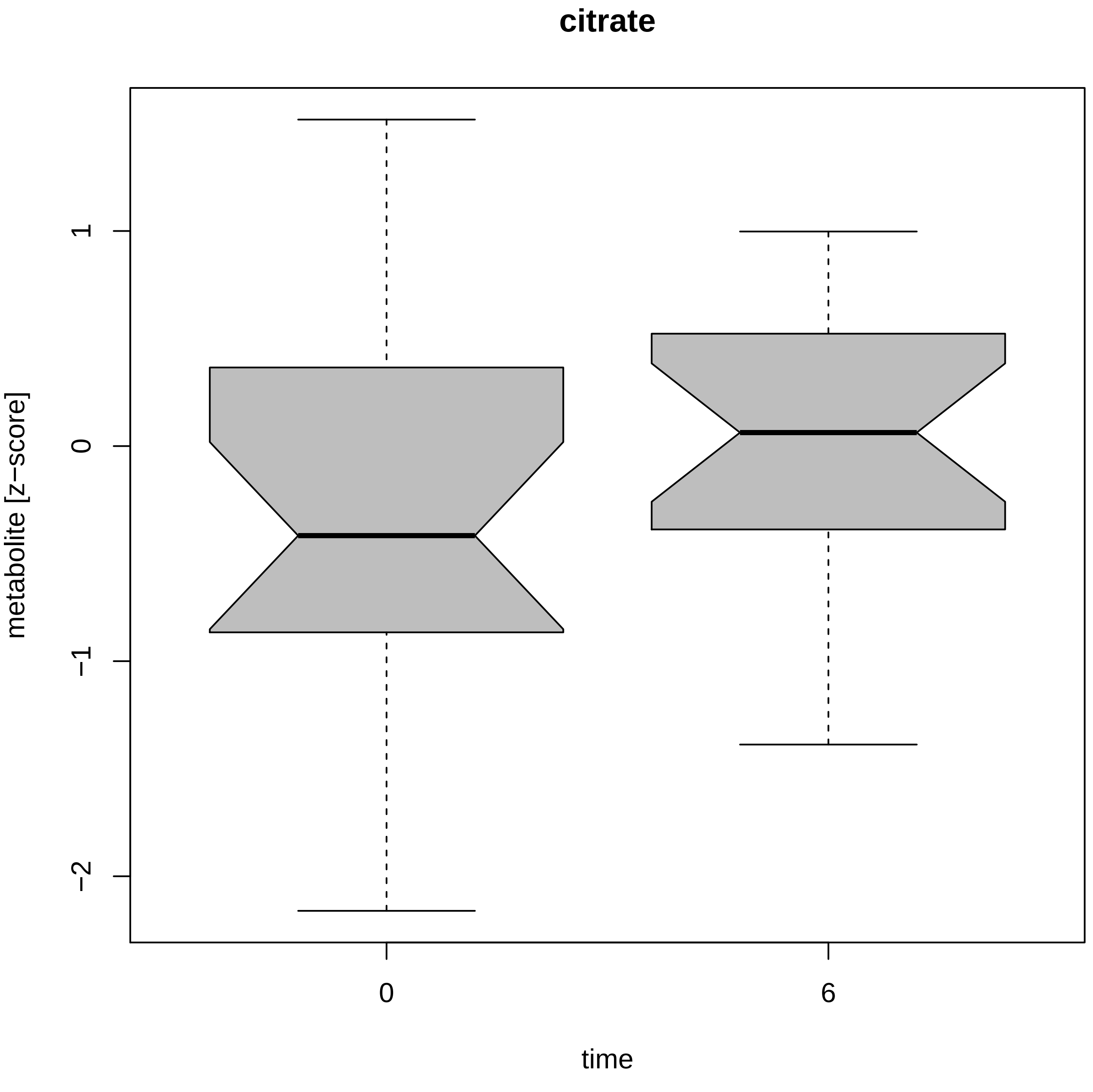

citrate
metabolite [z−score]
1
0
−1
−2
0
6
time

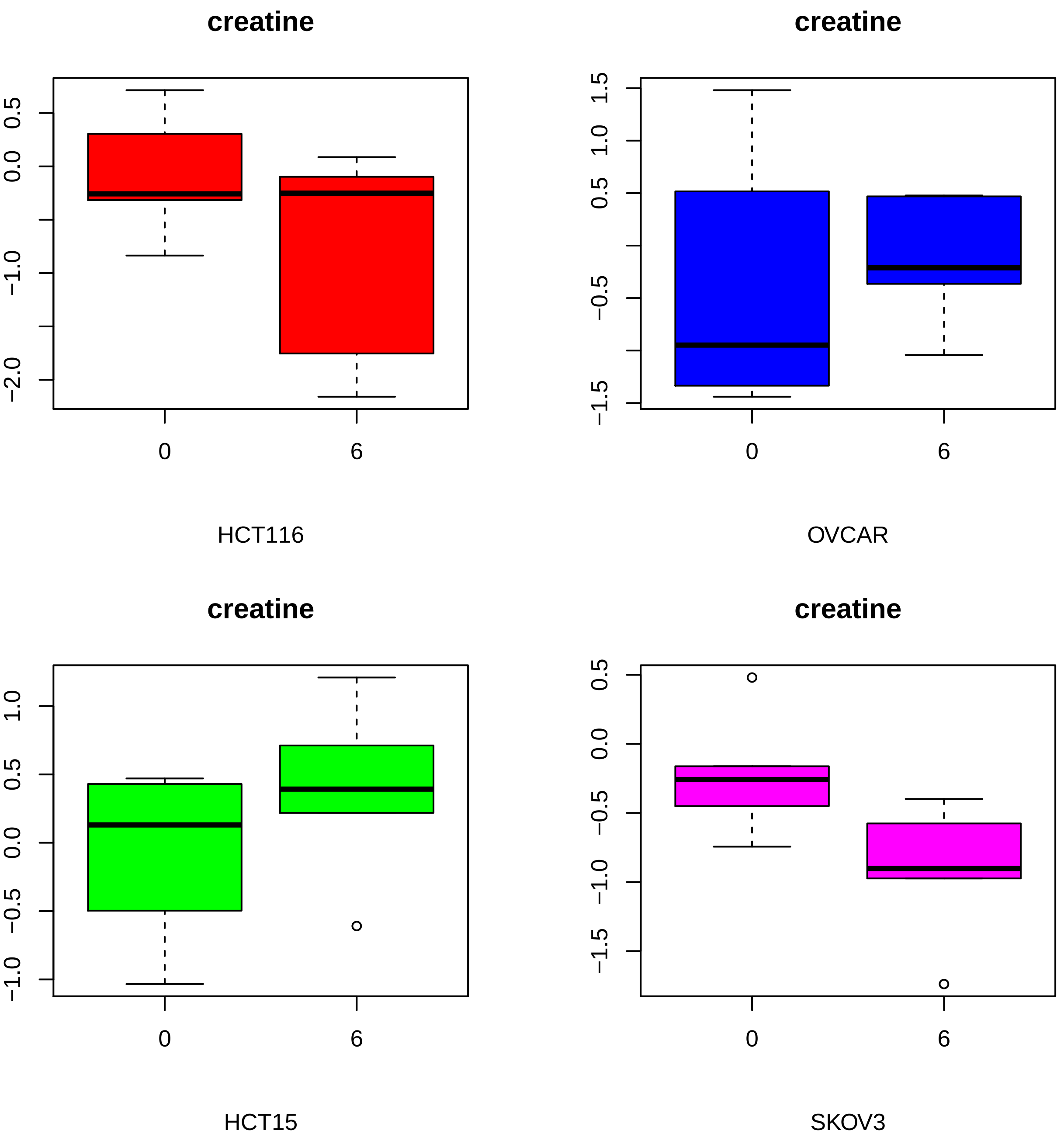
creatine
0.5
0.0
−1.0
−2.0
0
6
HCT116
creatine
1.5
1.0
0.5
−0.5
−1.5
0
6
OVCAR
creatine
1.0
0.5
0.0
−0.5
−1.0
0
6
HCT15
creatine
0.5
0.0
−0.5
−1.0
−1.5
0
6
SKOV3

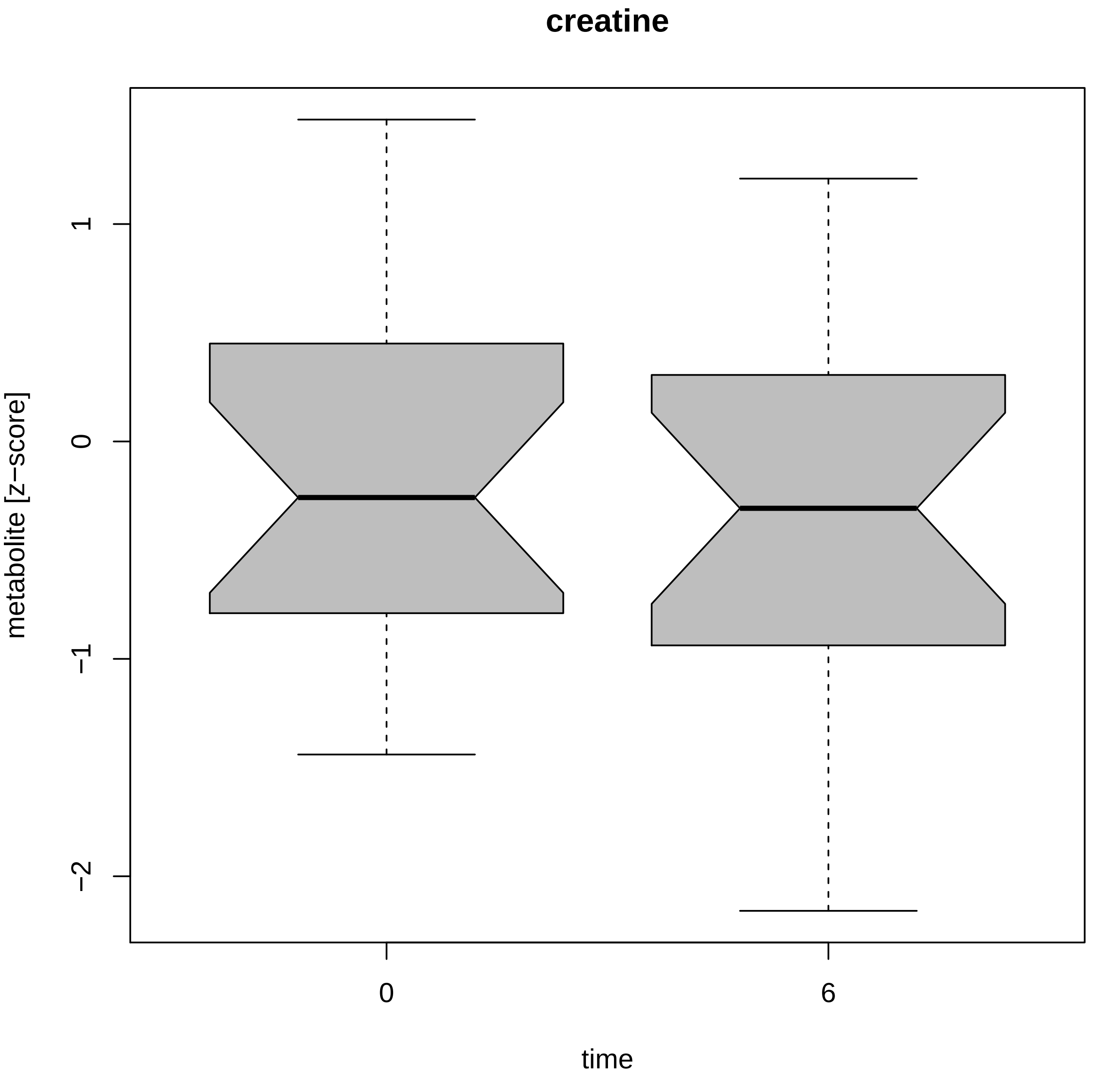

creatine
1
0
−1
−2
metabolite [z−score]
0
6
time

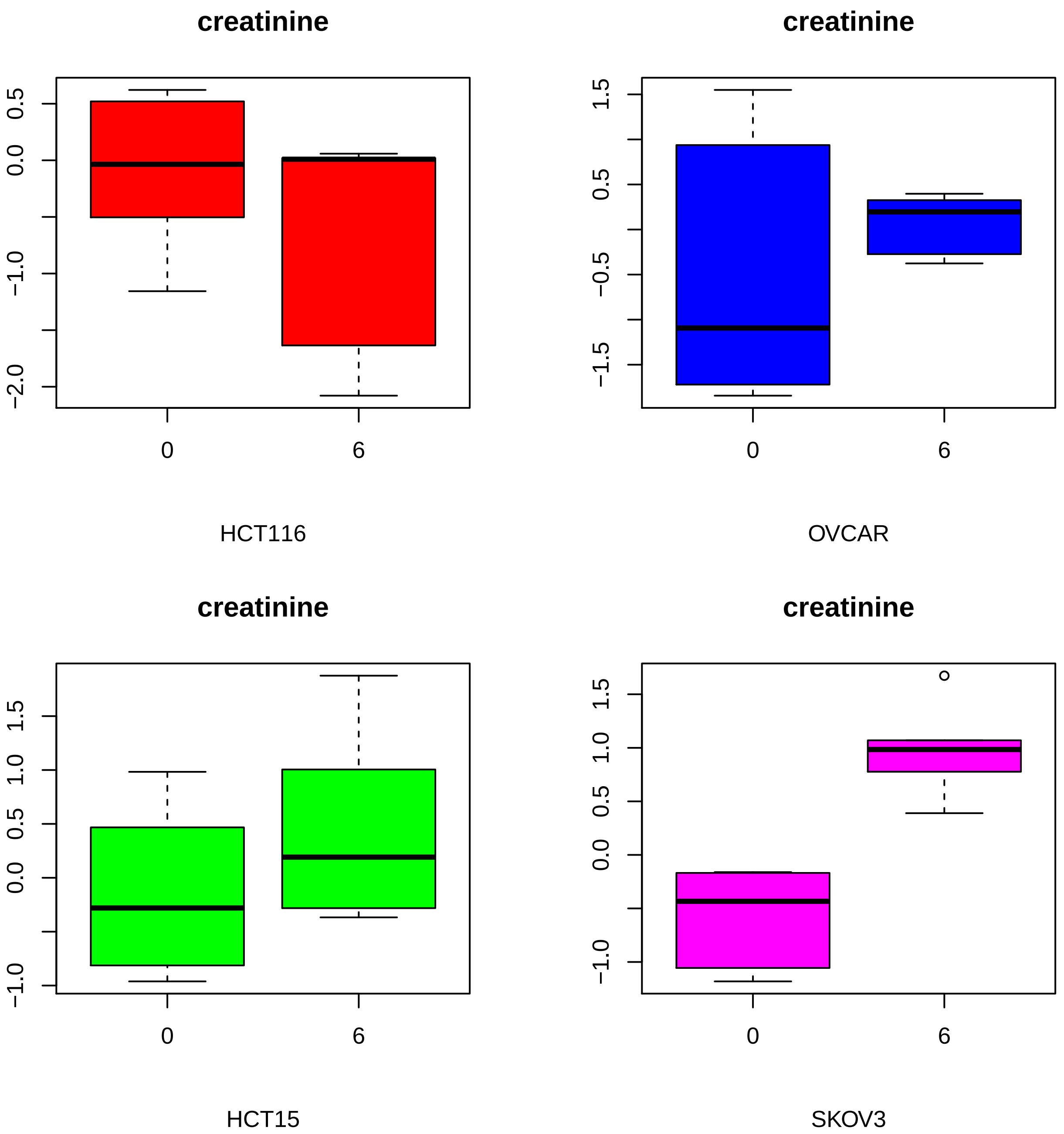
creatinine
0.5
0.0
−1.0
−2.0
0
6
HCT116
creatinine
1.5
0.5
−0.5
−1.5
0
6
OVCAR
creatinine
1.5
1.0
0.5
0.0
−1.0
0
6
HCT15
creatinine
1.5
1.0
0.5
0.0
−1.0
0
6
SKOV3

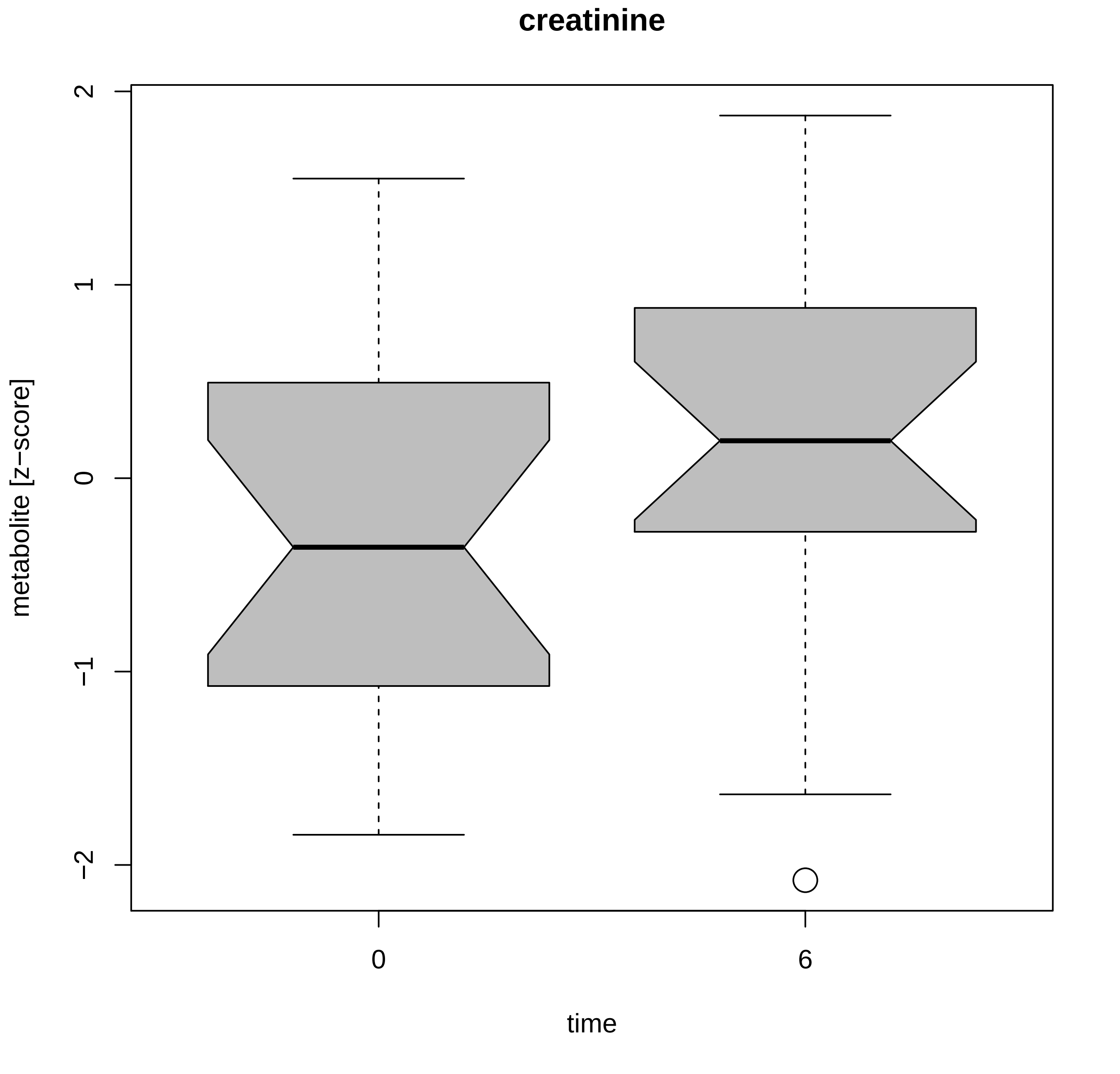
creatinine
metabolite [z−score]
2
1
0
−1
−2
0
6
time

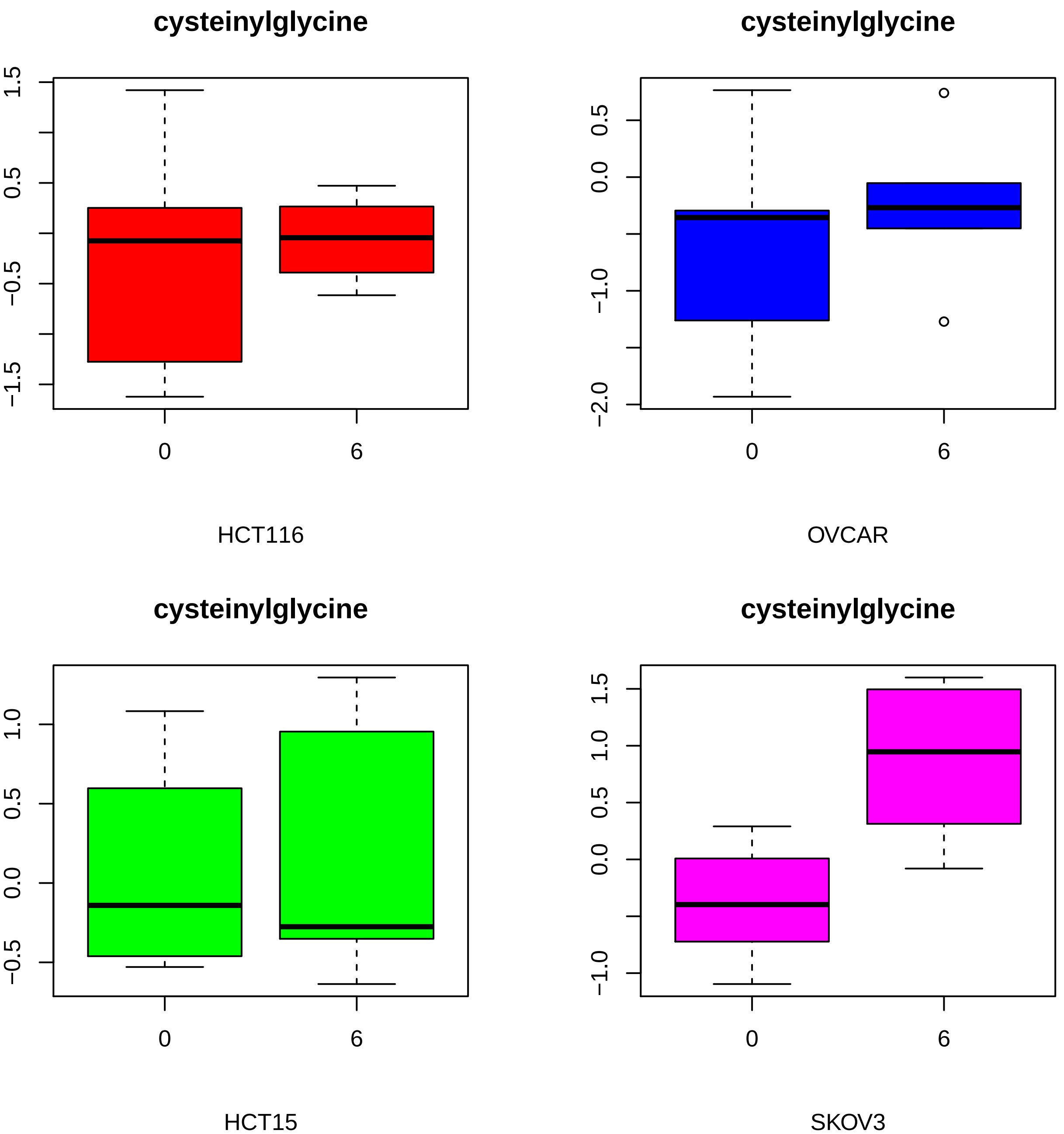

cysteinylglycine
1.5
0.5
−0.5
−1.5
0
6
HCT116
cysteinylglycine
0.5
0.0
−1.0
−2.0
0
6
OVCAR
cysteinylglycine
1.0
0.5
0.0
−0.5
0
6
HCT15
cysteinylglycine
1.5
1.0
0.5
0.0
−1.0
0
6
SKOV3

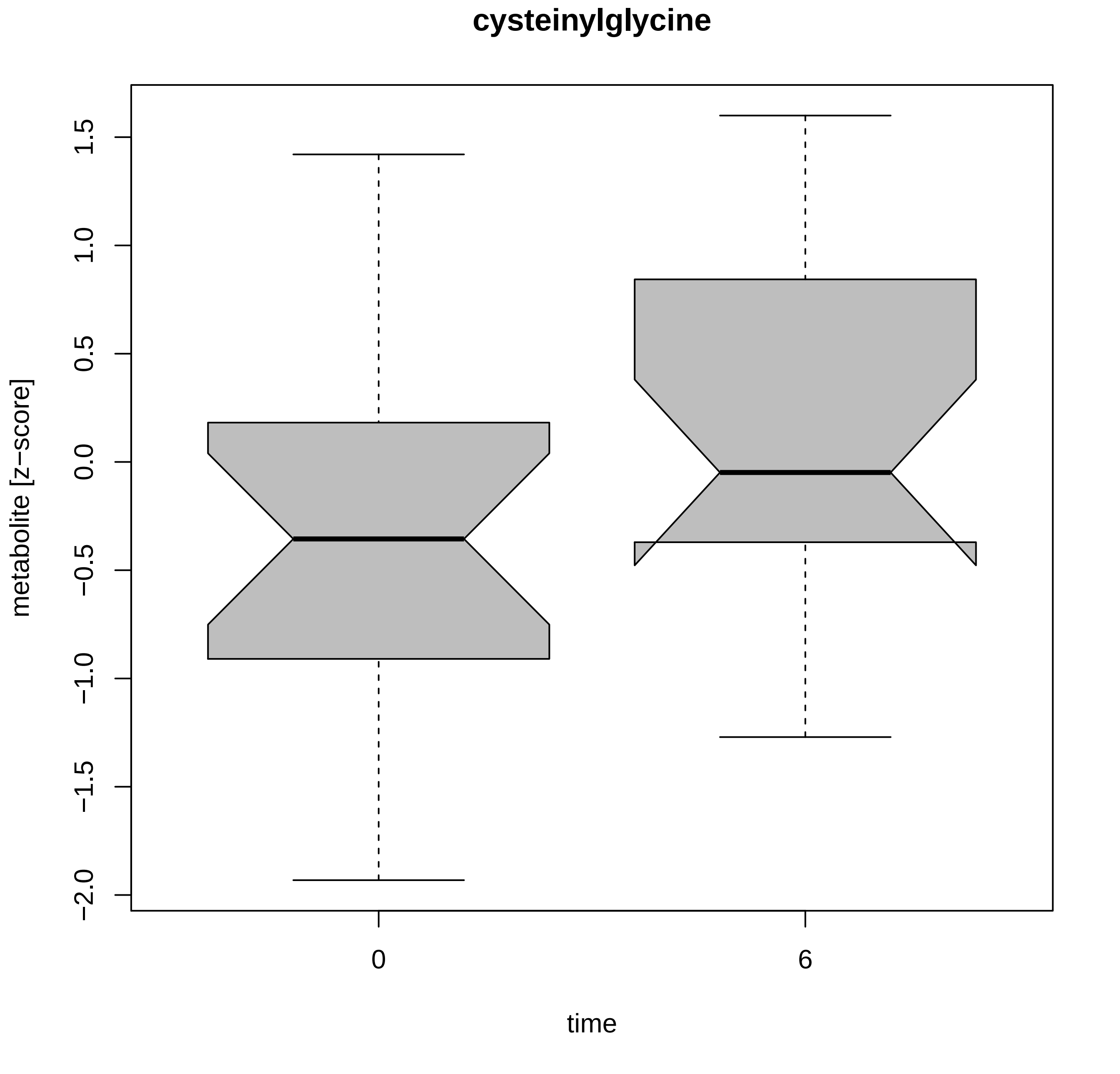
cysteinylglycine
metabolite [z−score]
1.5
1.0
0.5
0.0
−0.5
−1.0
−1.5
−2.0
0
6
time

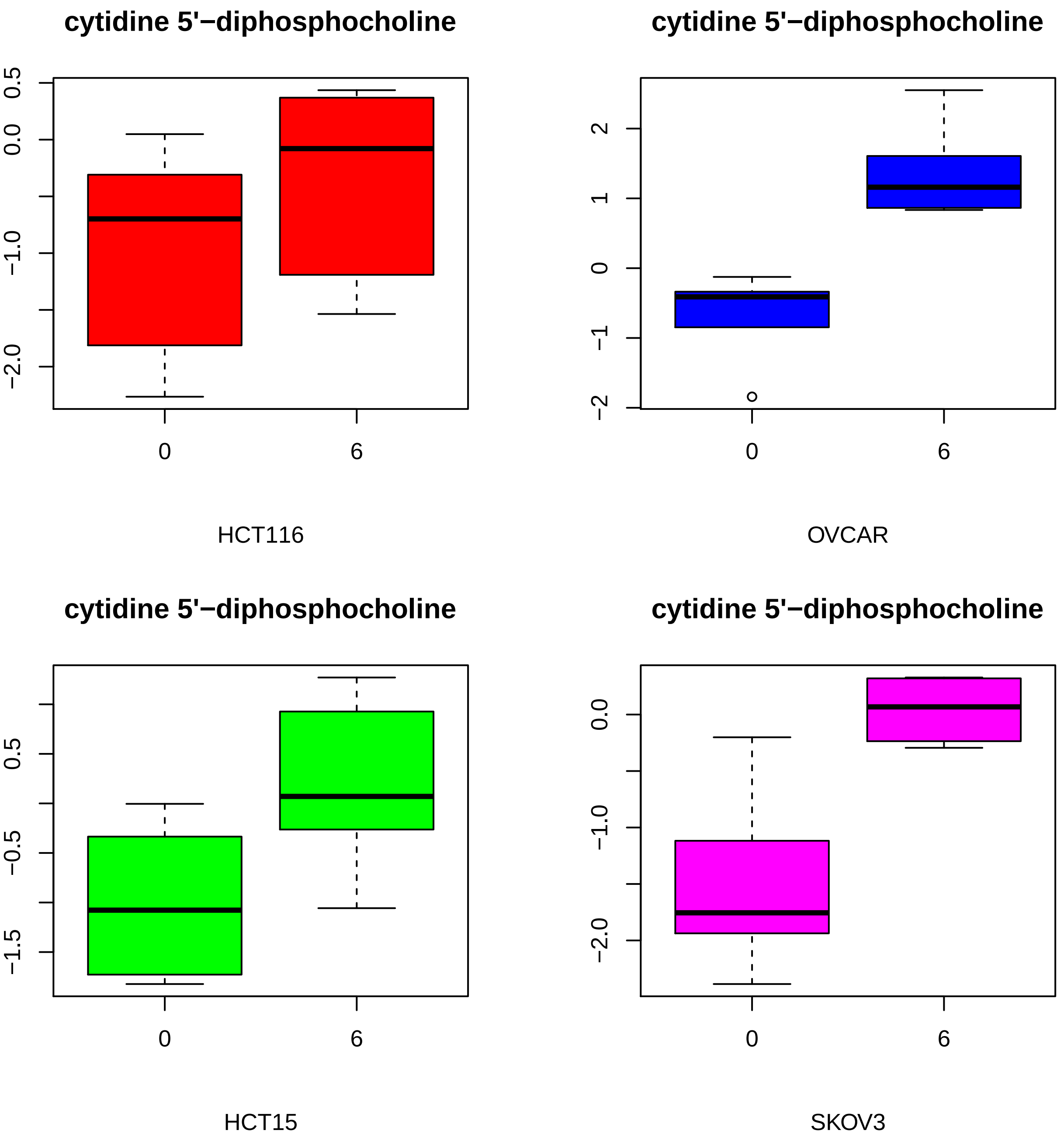
cytidine 5'−diphosphocholine
0.5
0.0
−1.0
−2.0
0
6
HCT116
cytidine 5'−diphosphocholine
2
1
0
−1
−2
0
6
OVCAR
cytidine 5'−diphosphocholine
0.5
−0.5
−1.5
0
6
HCT15
cytidine 5'−diphosphocholine
0.0
−1.0
−2.0
0
6
SKOV3

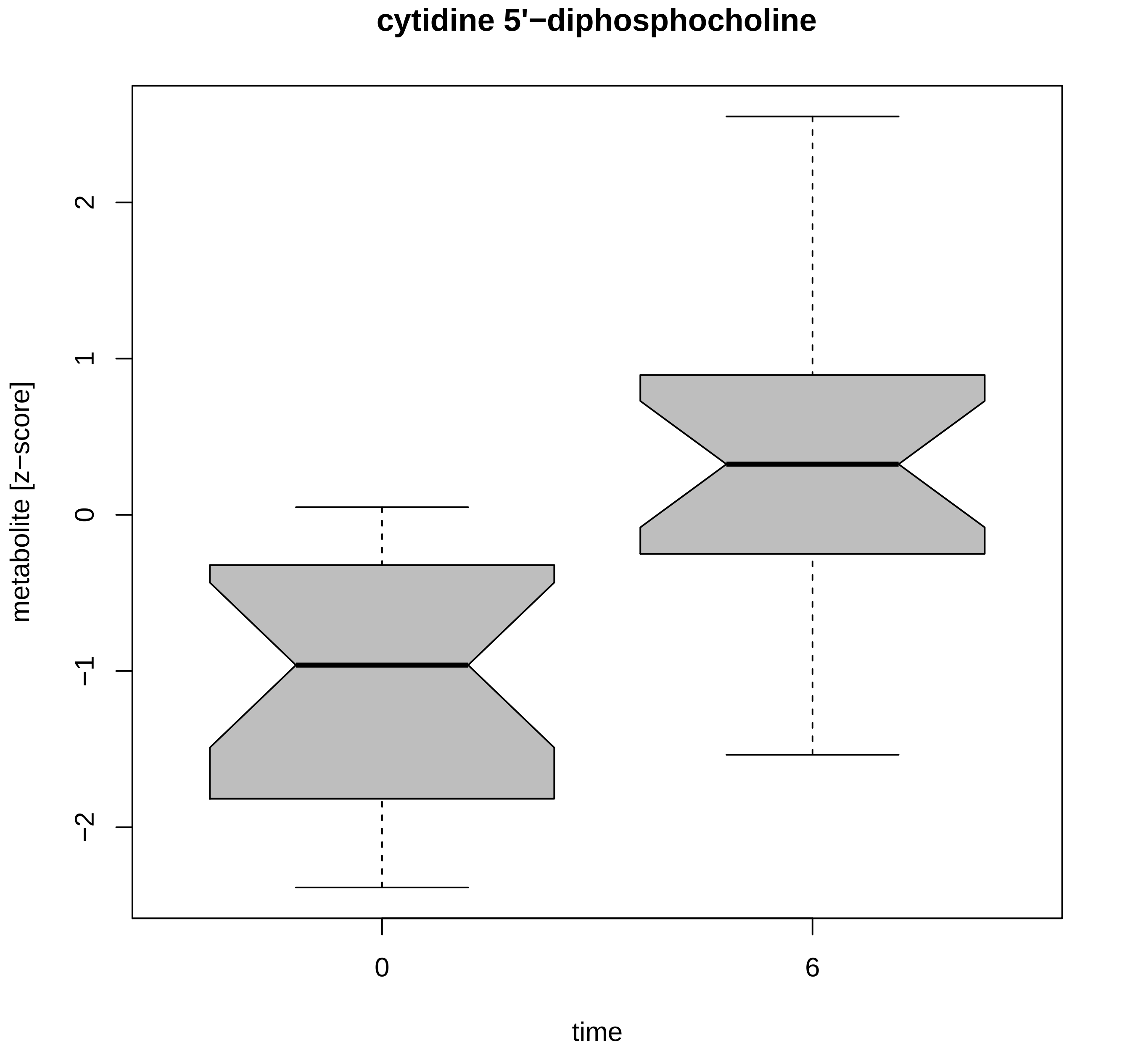
cytidine 5'−diphosphocholine
metabolite [z−score]
2
1
0
−1
−2
0
6
time

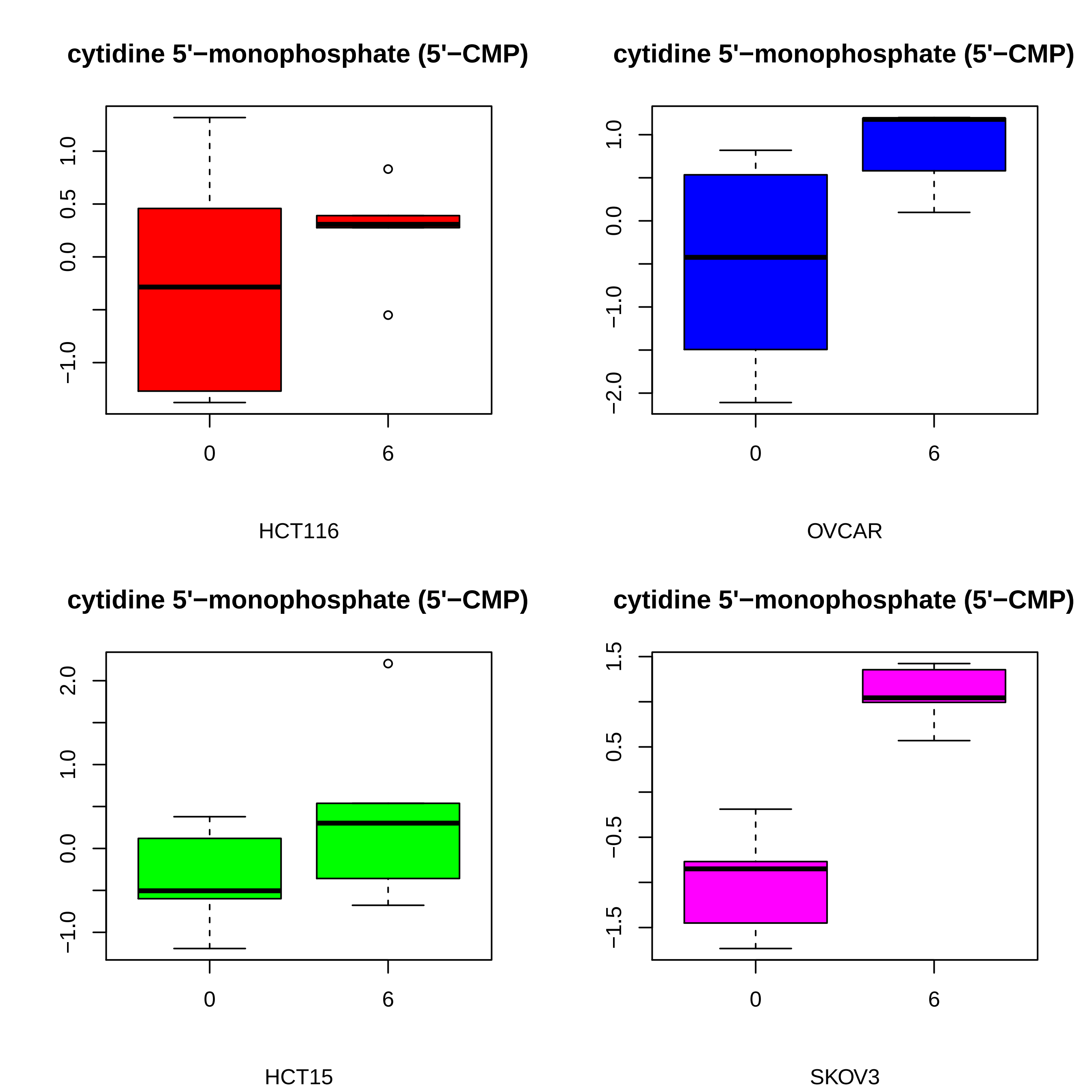

cytidine 5'−monophosphate (5'−CMP)
1.0
0.5
0.0
−1.0
0
6
HCT116
cytidine 5'−monophosphate (5'−CMP)
1.0
0.0
−1.0
−2.0
0
6
OVCAR
cytidine 5'−monophosphate (5'−CMP)
2.0
1.0
0.0
−1.0
0
6
HCT15
cytidine 5'−monophosphate (5'−CMP)
1.5
0.5
−0.5
−1.5
0
6
SKOV3

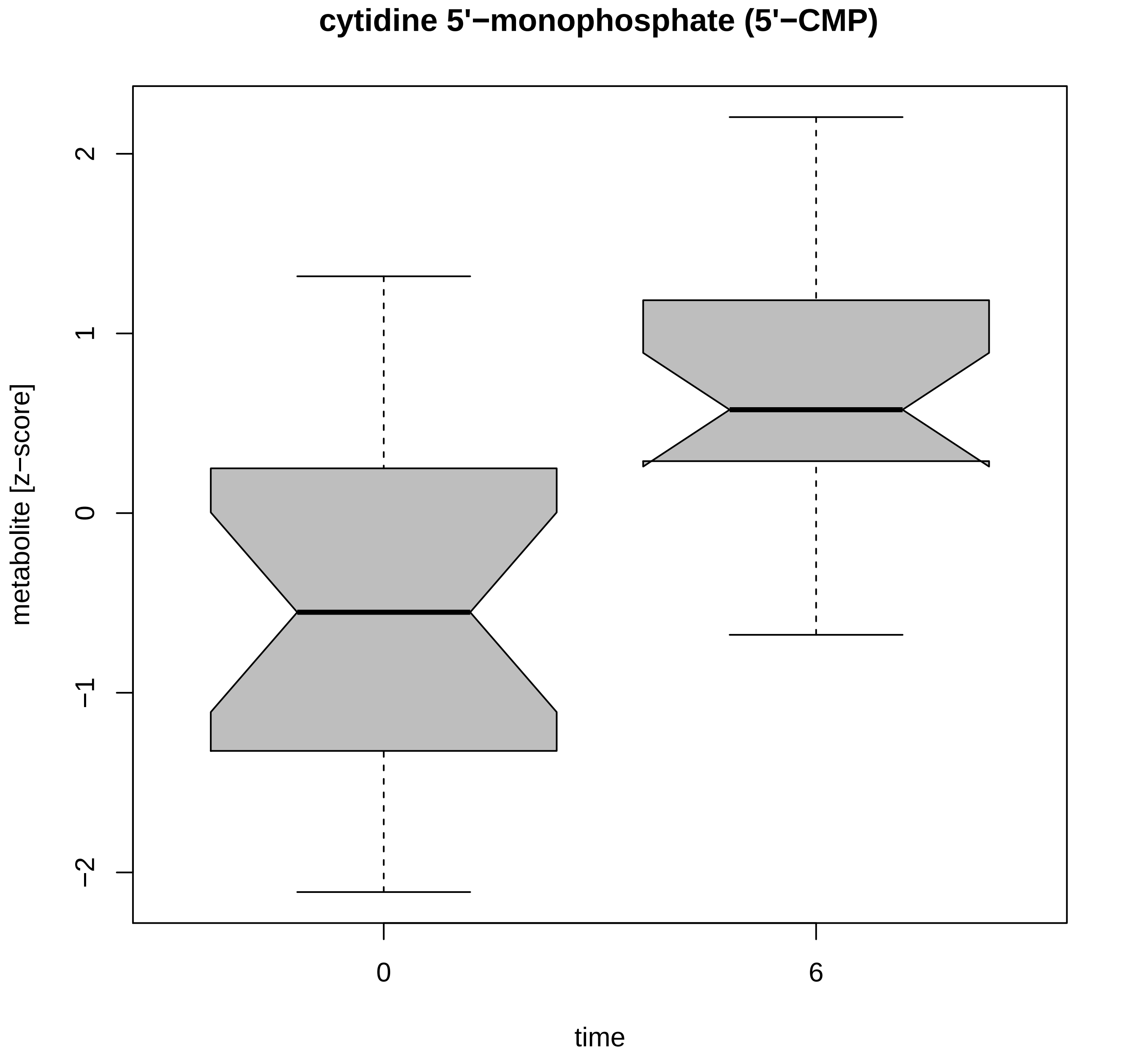

cytidine 5'−monophosphate (5'−CMP)
metabolite [z−score]
2
1
0
−1
−2
0
6
time

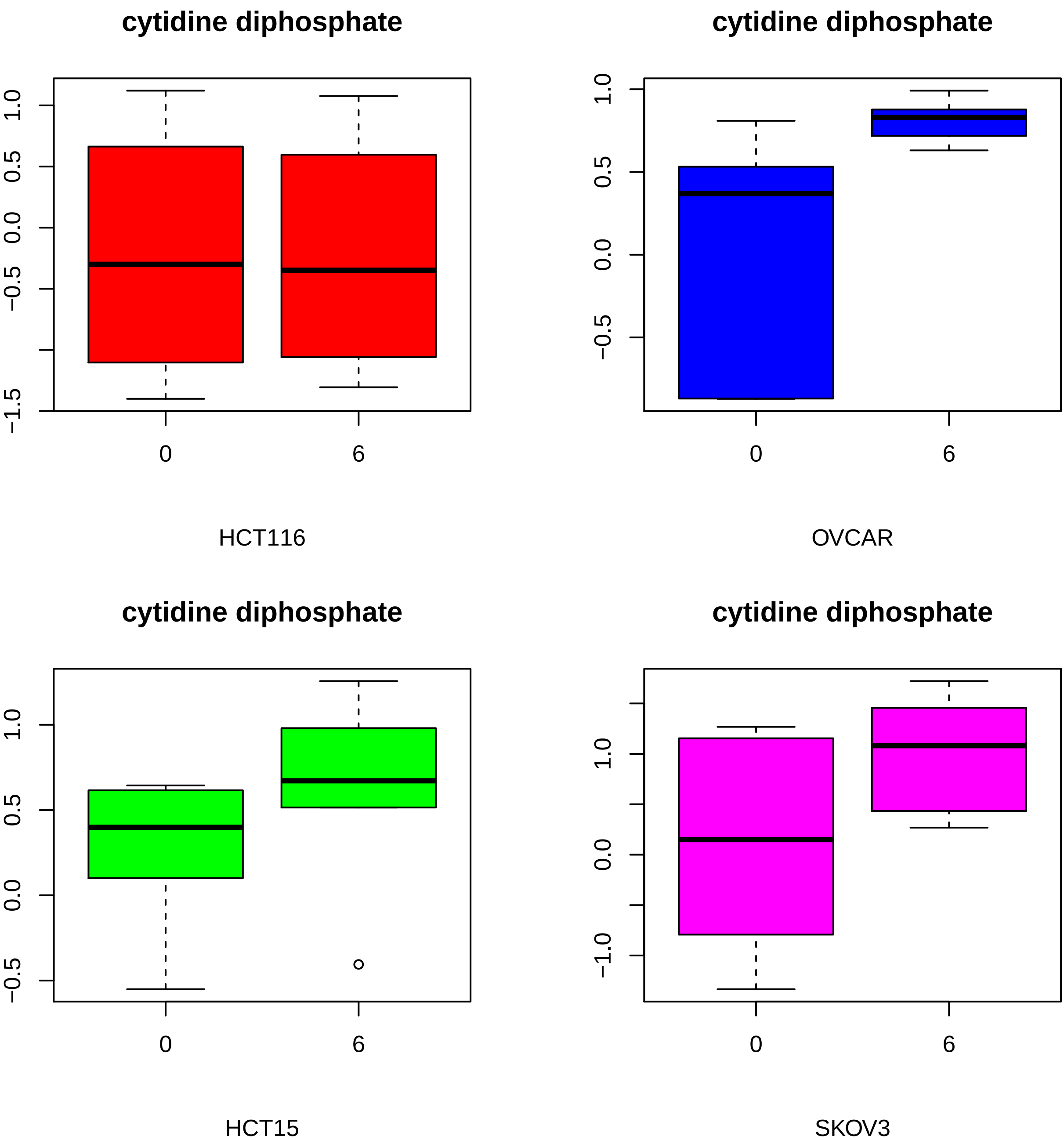
cytidine diphosphate
1.0
0.5
0.0
−0.5
−1.5
0
6
HCT116
cytidine diphosphate
1.0
0.5
0.0
−0.5
0
6
OVCAR
cytidine diphosphate
1.0
0.5
0.0
−0.5
0
6
HCT15
cytidine diphosphate
1.0
0.0
−1.0
0
6
SKOV3

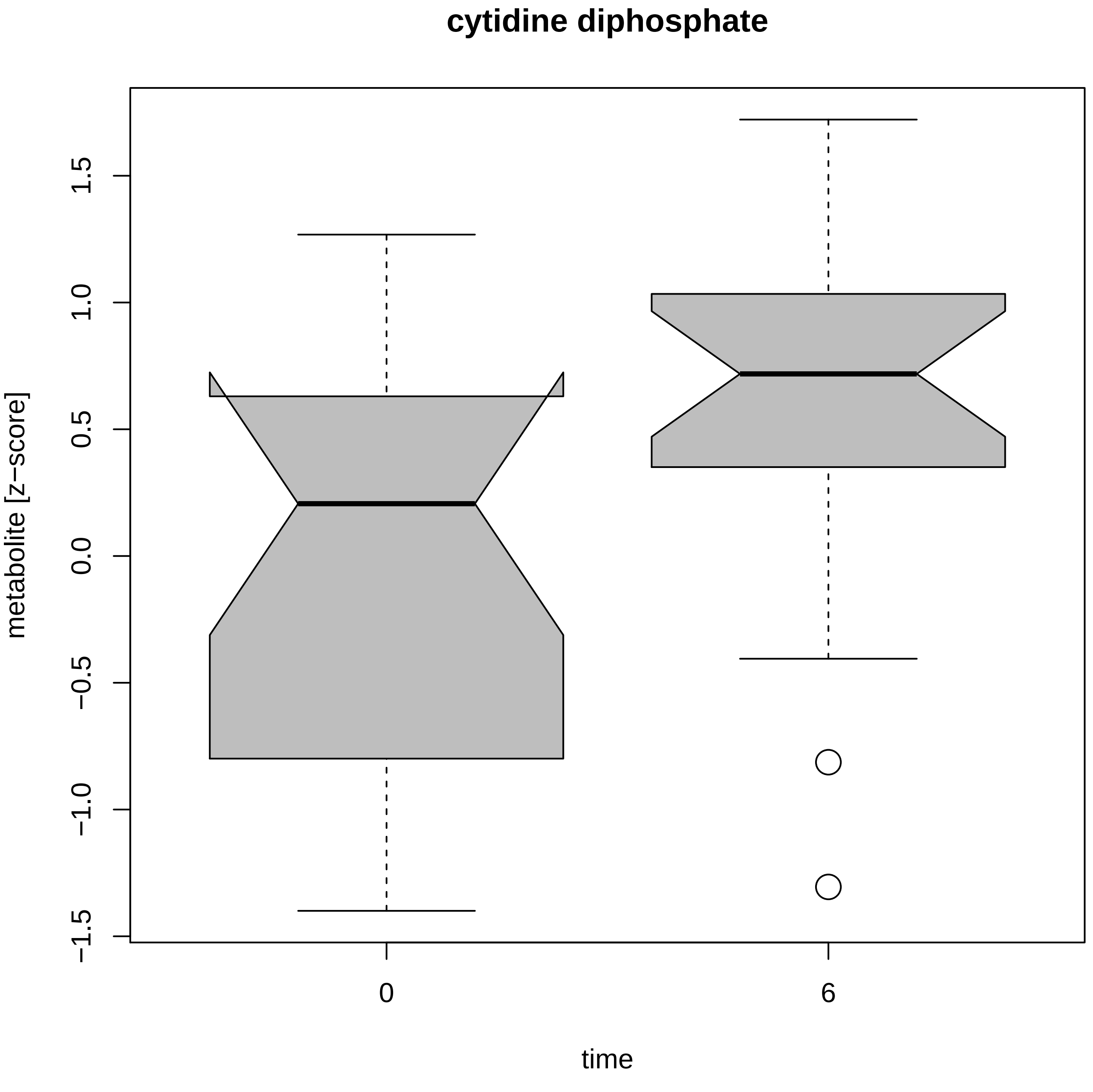

cytidine diphosphate
metabolite [z−score]
1.5
1.0
0.5
0.0
−0.5
−1.0
−1.5
0
6
time

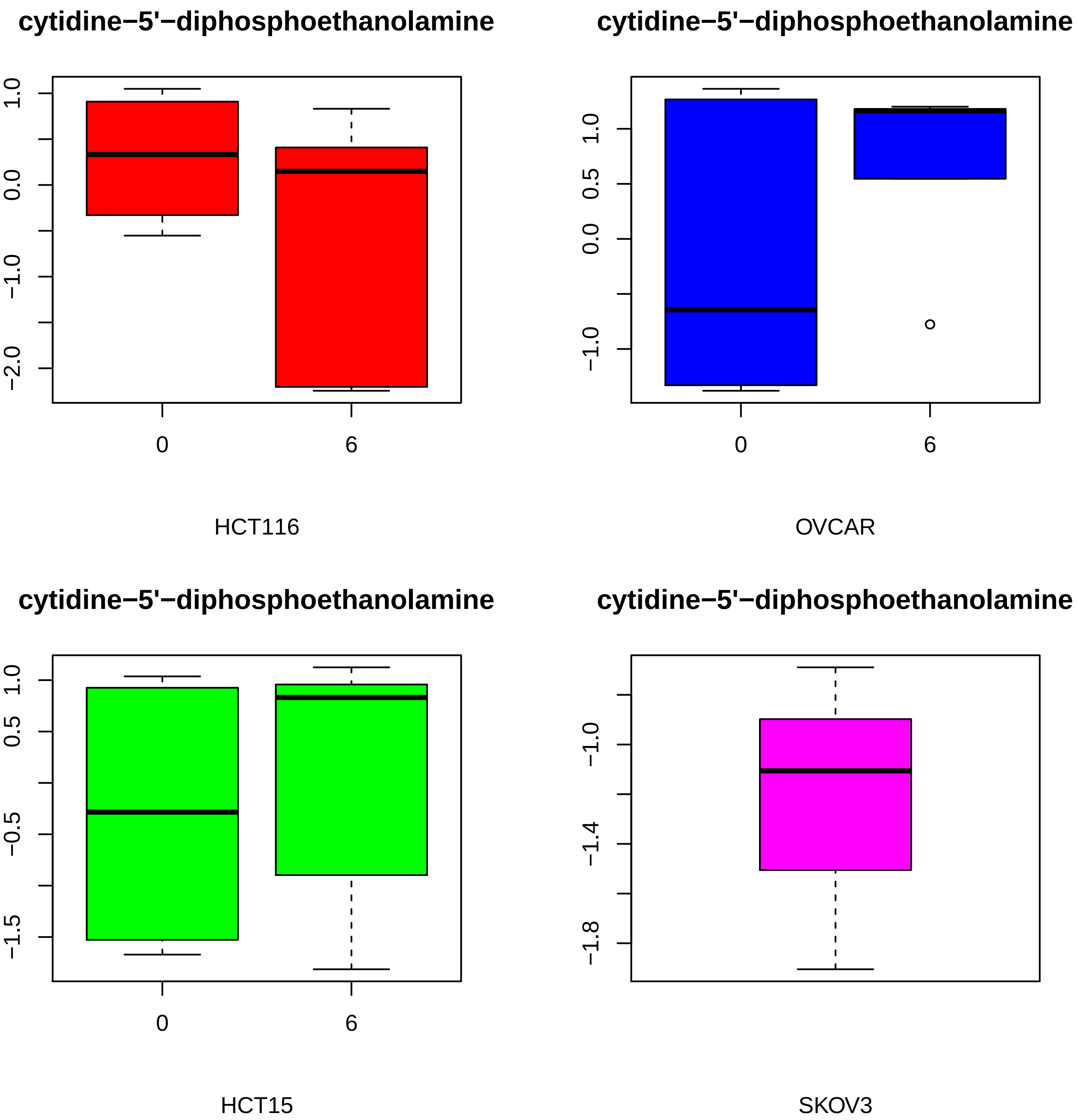
cytidine−5'−diphosphoethanolamine
HCT116
cytidine−5'−diphosphoethanolamine
OVCAR
cytidine−5'−diphosphoethanolamine
HCT15
cytidine−5'−diphosphoethanolamine
SKOV3

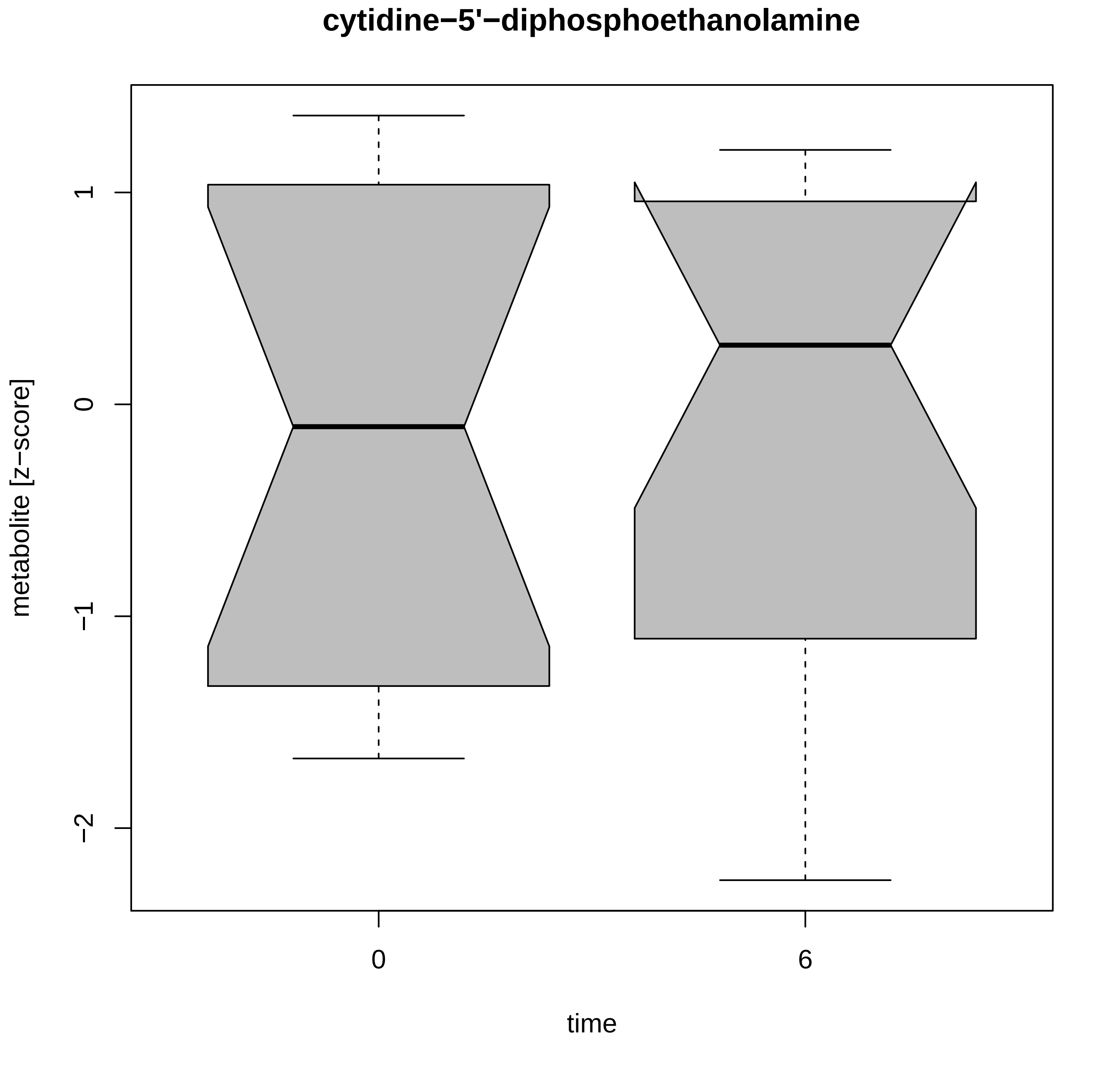

cytidine−5'−diphosphoethanolamine
metabolite [z−score]
1
0
−1
−2
0
6
time

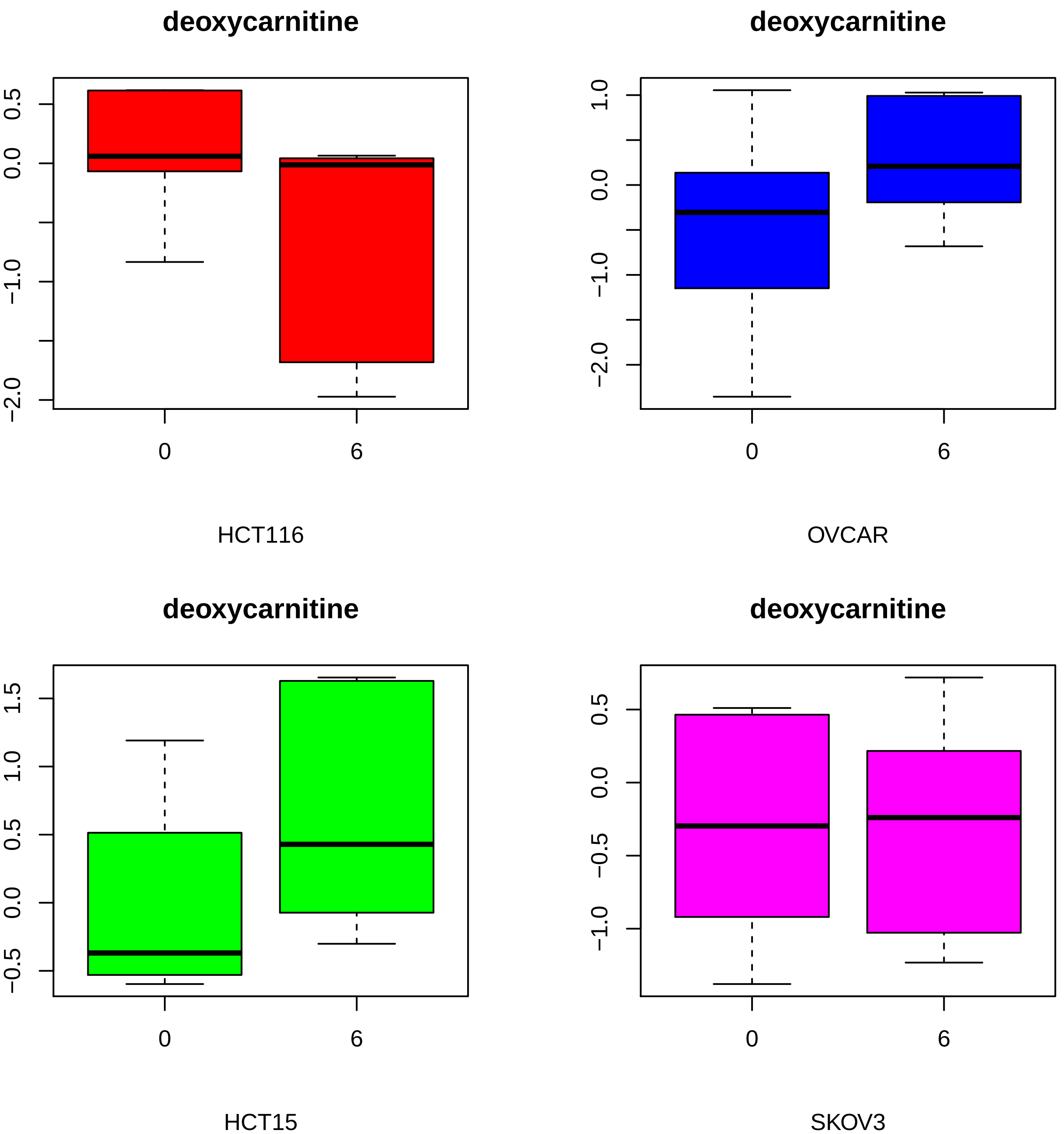
deoxycarnitine
0.5
0.0
−1.0
−2.0
0
6
HCT116
deoxycarnitine
1.0
0.0
−1.0
−2.0
0
6
OVCAR
deoxycarnitine
1.5
1.0
0.5
0.0
−0.5
0
6
HCT15
deoxycarnitine
0.5
0.0
−0.5
−1.0
0
6
SKOV3

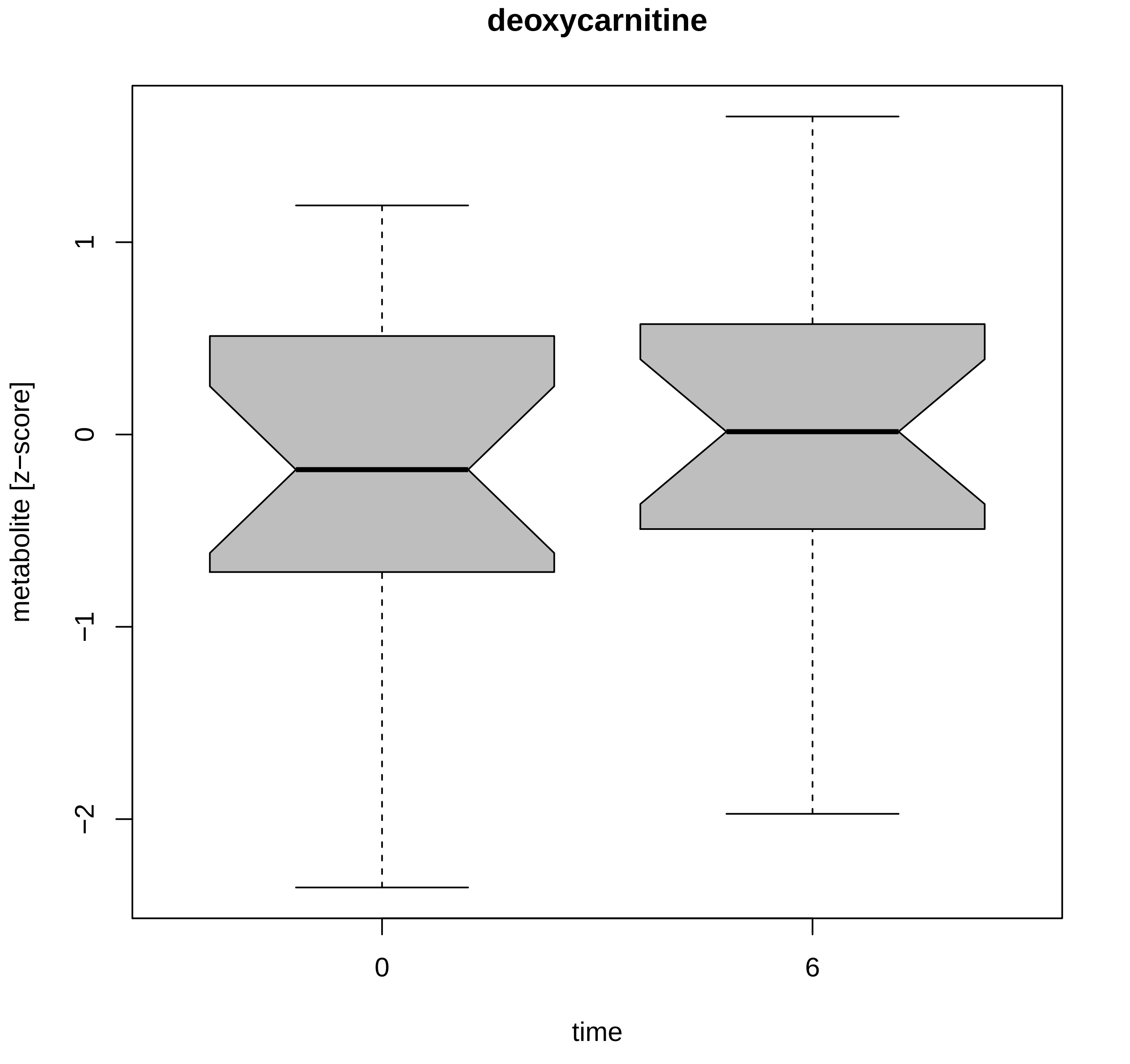

deoxycarnitine
metabolite [z−score]
1
0
−1
−2
0
6
time

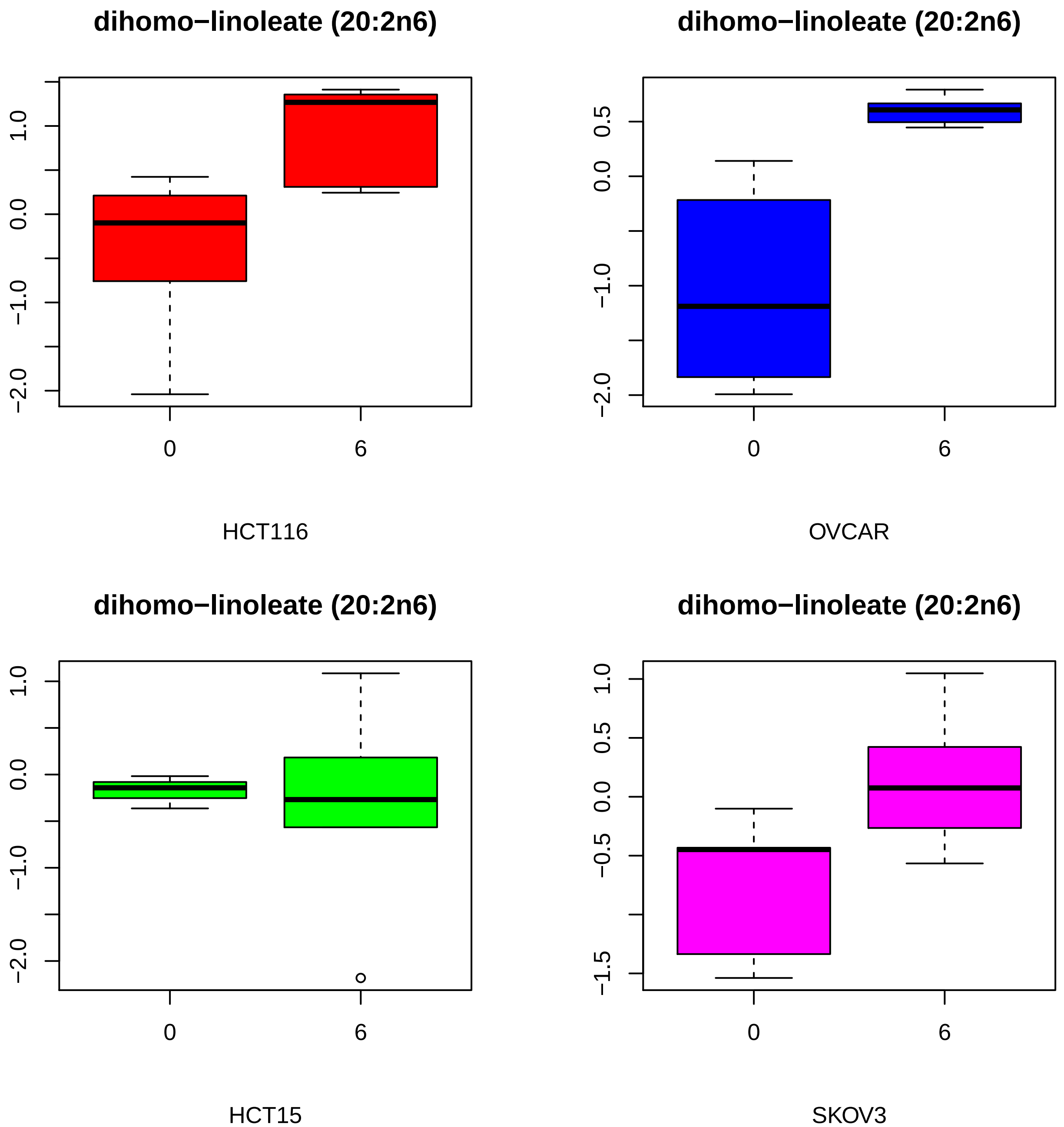
dihomo−linoleate (20:2n6)
HCT116
OVCAR
HCT15
SKOV3
0
6
1.0
0.0
−1.0
−2.0
0.5
−0.5
−1.5

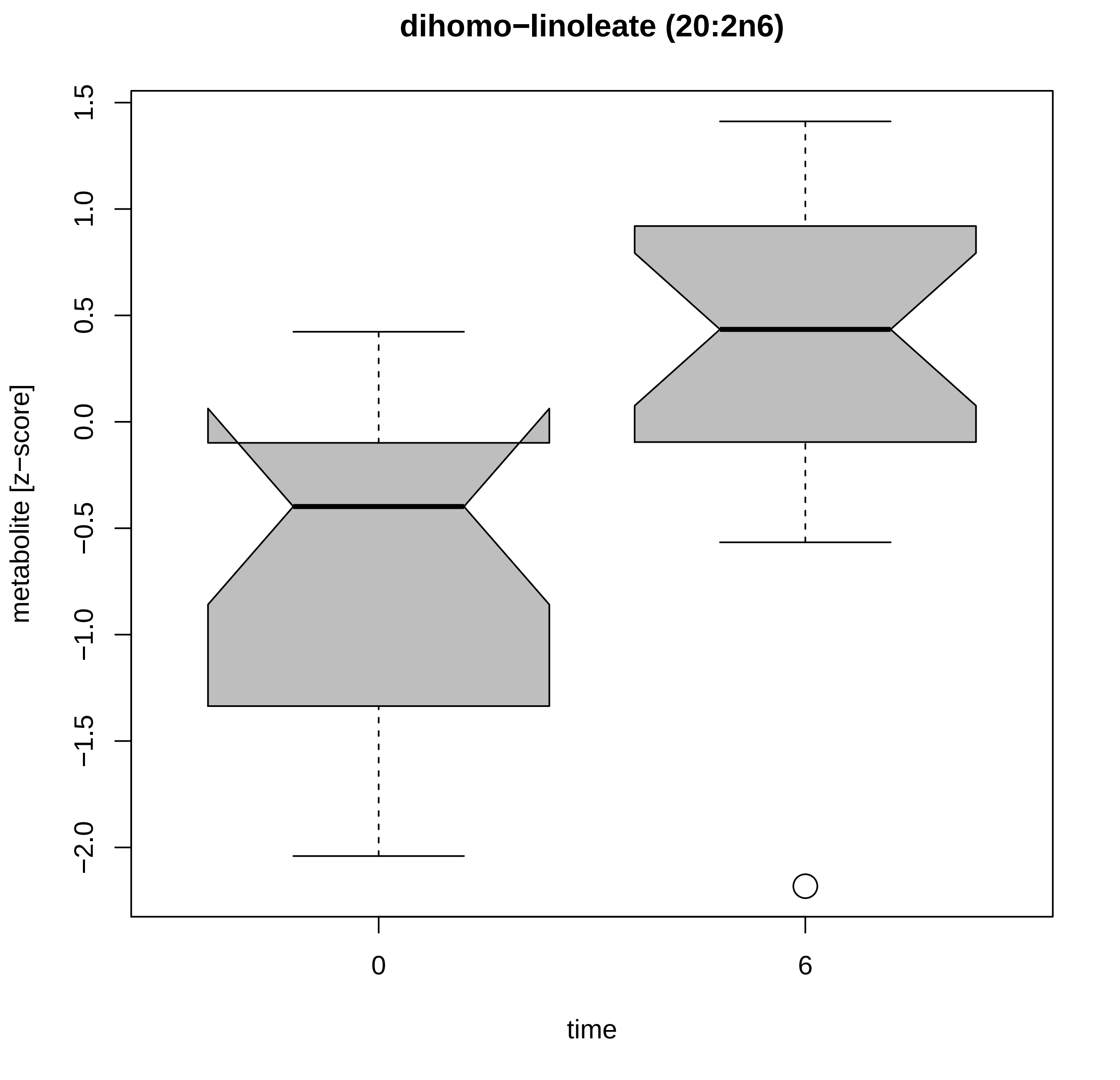
dihomo−linoleate (20:2n6)
metabolite [z−score]
1.5
1.0
0.5
0.0
−0.5
−1.0
−1.5
−2.0
0
6
time

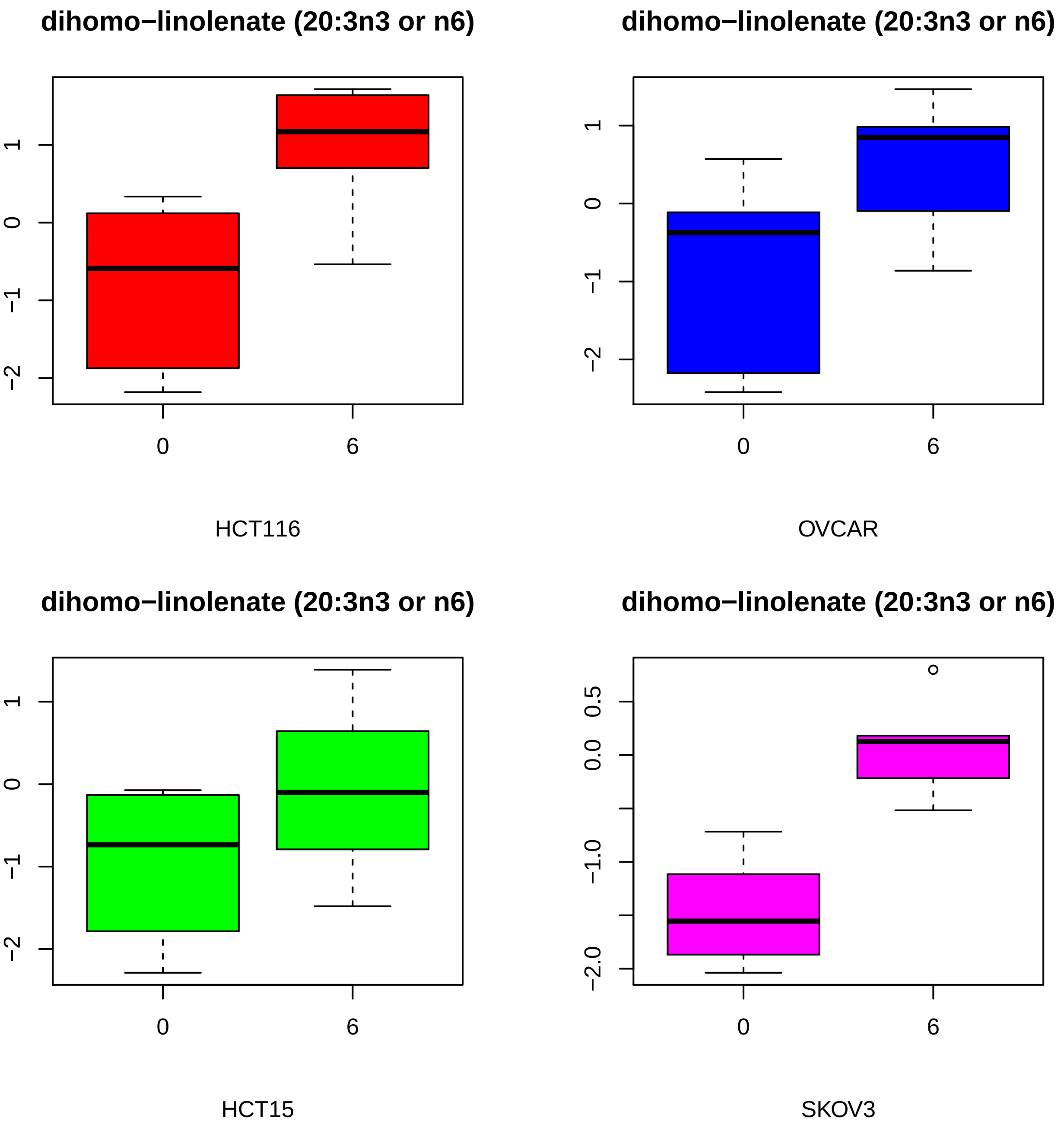
dihomo−linolenate (20:3n3 or n6)
HCT116
dihomo−linolenate (20:3n3 or n6)
OVCAR
dihomo−linolenate (20:3n3 or n6)
HCT15
dihomo−linolenate (20:3n3 or n6)
SKOV3
0
6
−2
−1
0
1
−2.0
−1.0
0.0
0.5

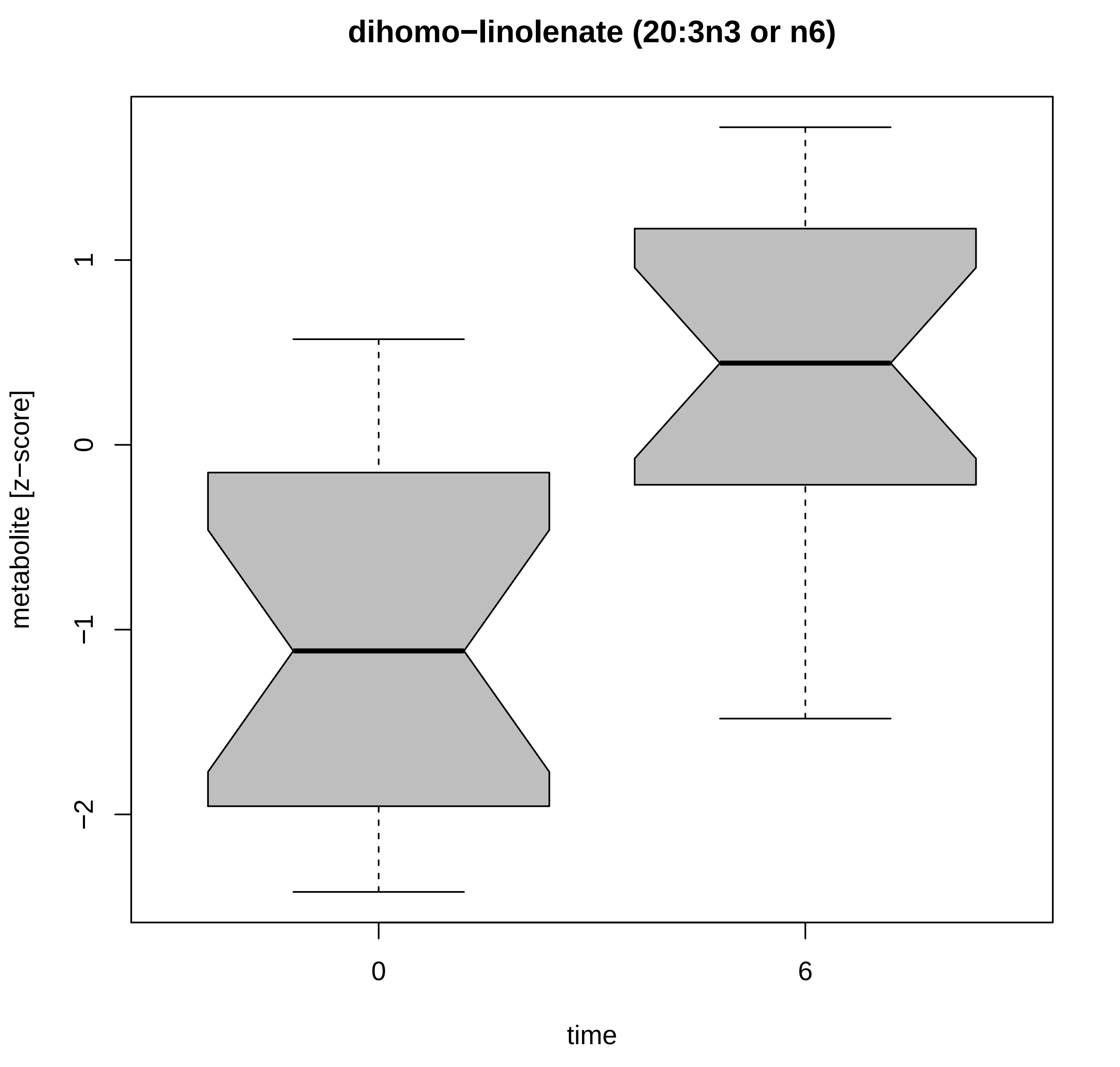

dihomo−linolenate (20:3n3 or n6)
metabolite [z−score]
1
0
−1
−2
0
6
time

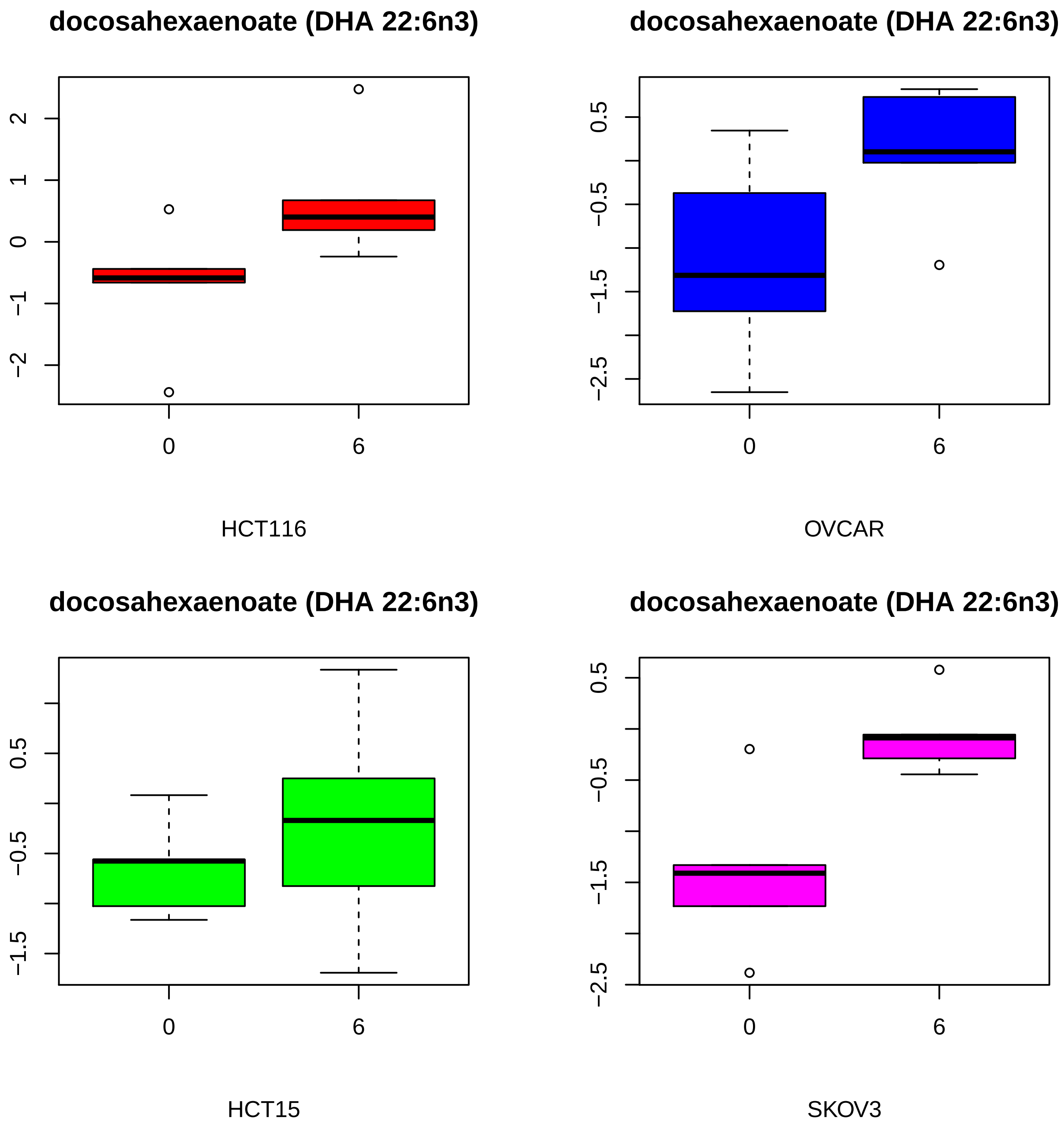

docosahexaenoate (DHA 22:6n3)
2
1
0
−1
−2
0
6
HCT116
docosahexaenoate (DHA 22:6n3)
0.5
−0.5
−1.5
−2.5
0
6
OVCAR
docosahexaenoate (DHA 22:6n3)
0.5
−0.5
−1.5
0
6
HCT15
docosahexaenoate (DHA 22:6n3)
0.5
−0.5
−1.5
−2.5
0
6
SKOV3

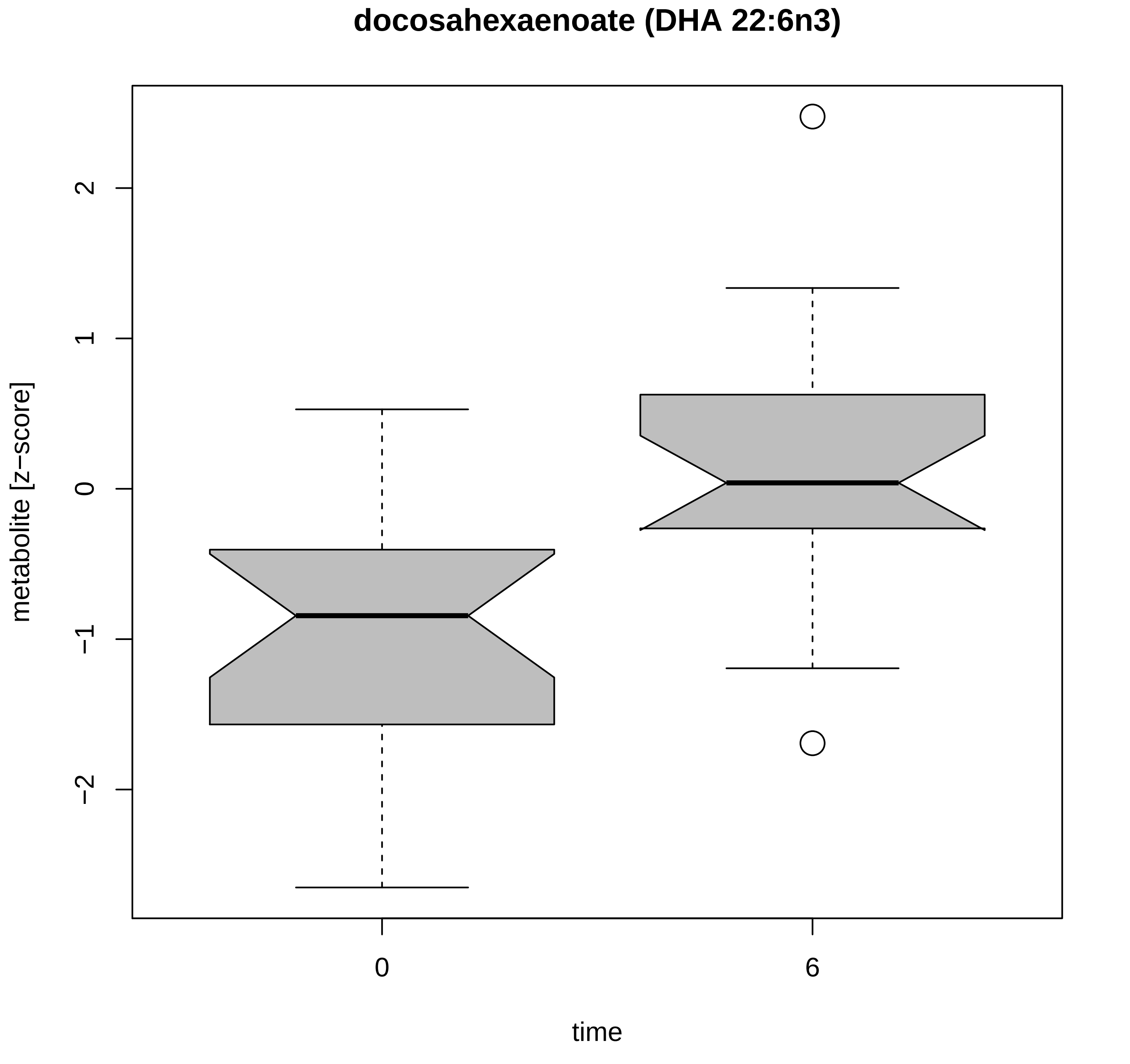

docosahexaenoate (DHA 22:6n3)
metabolite [z−score]
2
1
0
−1
−2
0
6
time

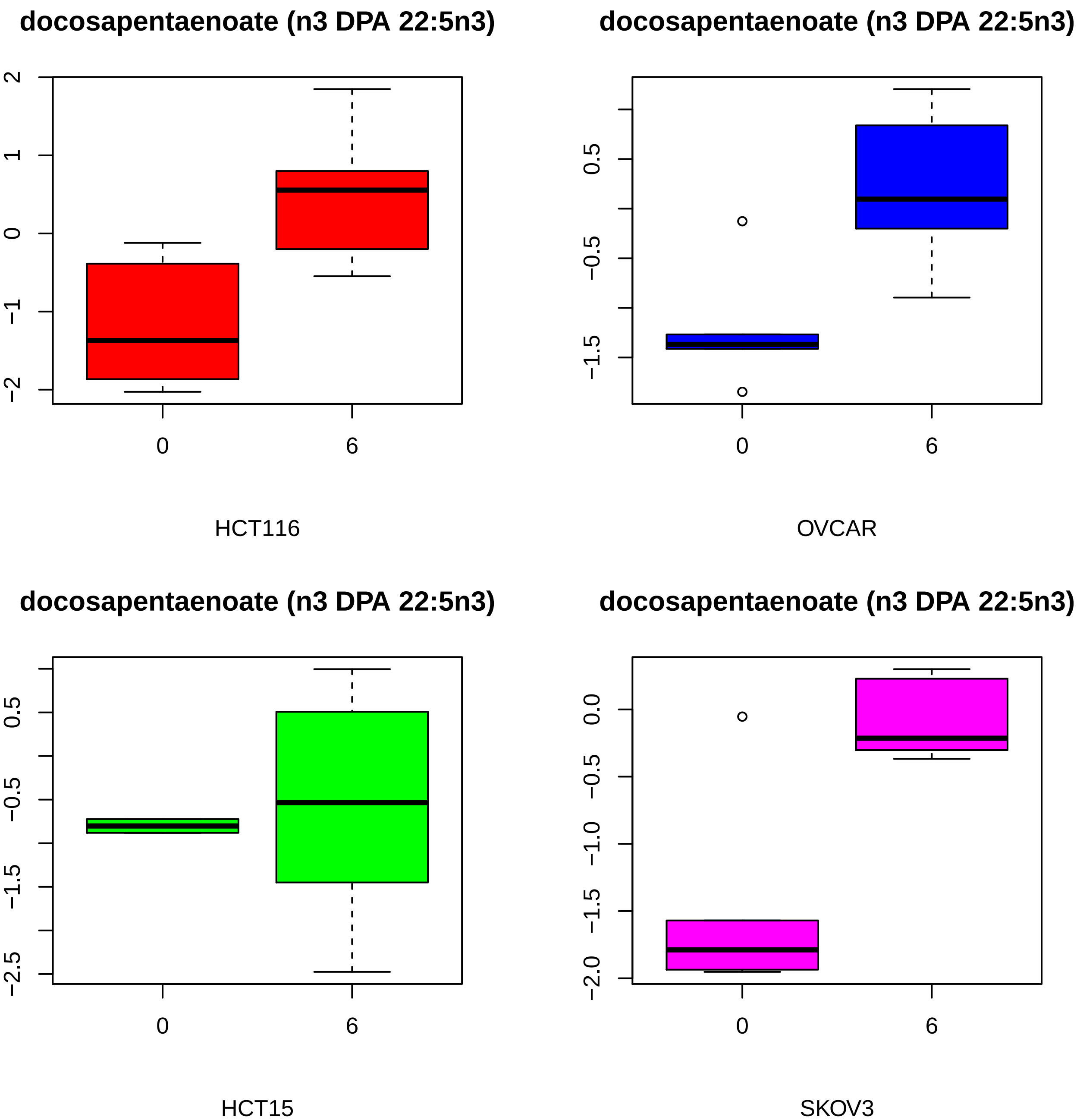
docosapentaenoate (n3 DPA 22:5n3)
HCT116
docosapentaenoate (n3 DPA 22:5n3)
OVCAR
docosapentaenoate (n3 DPA 22:5n3)
HCT15
docosapentaenoate (n3 DPA 22:5n3)
SKOV3

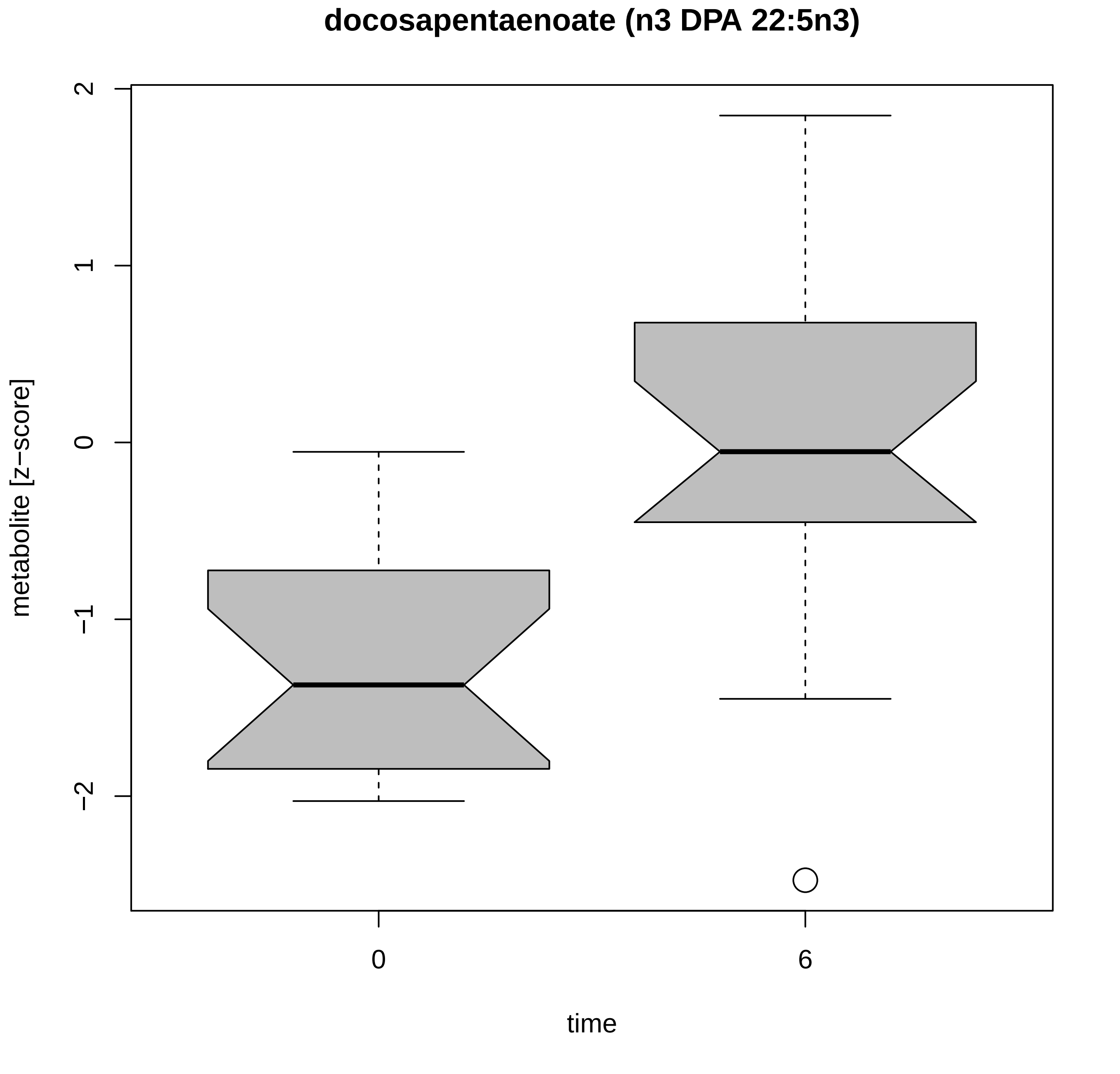
docosapentaenoate (n3 DPA 22:5n3)
metabolite [z−score]
2
1
0
−1
−2
0
6
time

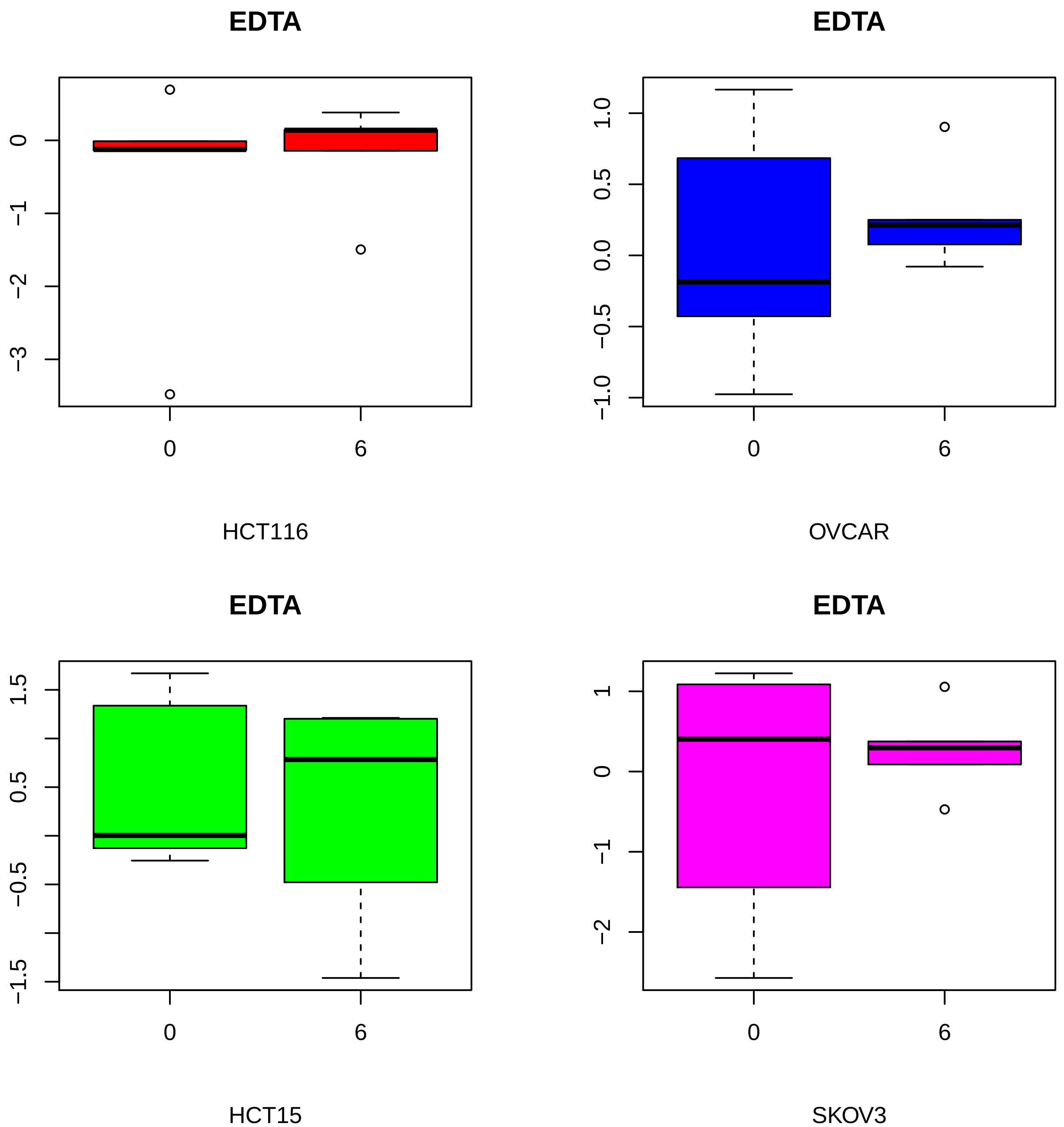

EDTA
HCT116
EDTA
OVCAR
EDTA
HCT15
EDTA
SKOV3

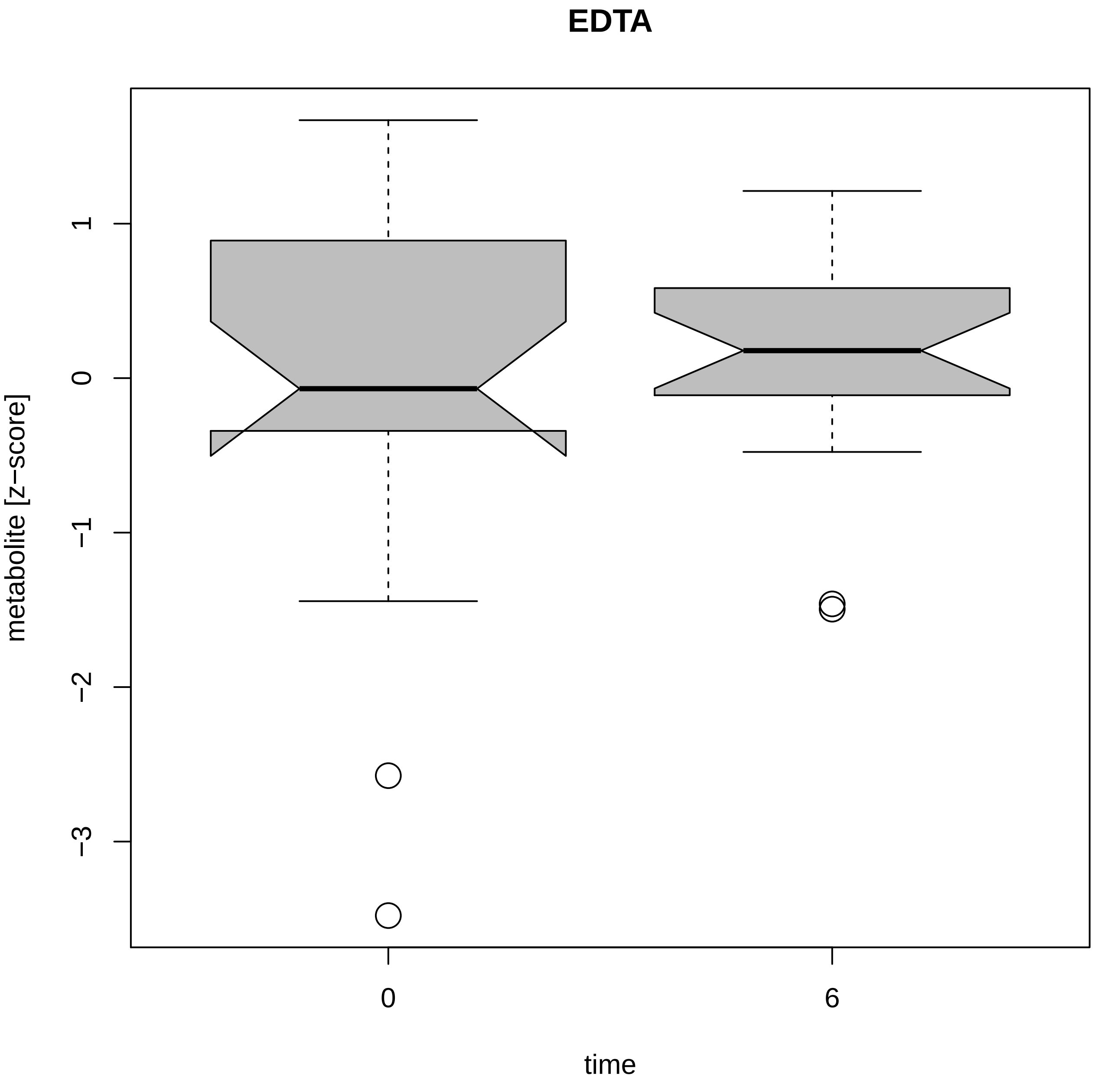
EDTA
metabolite [z−score]
1
0
−1
−2
−3
0
6
time

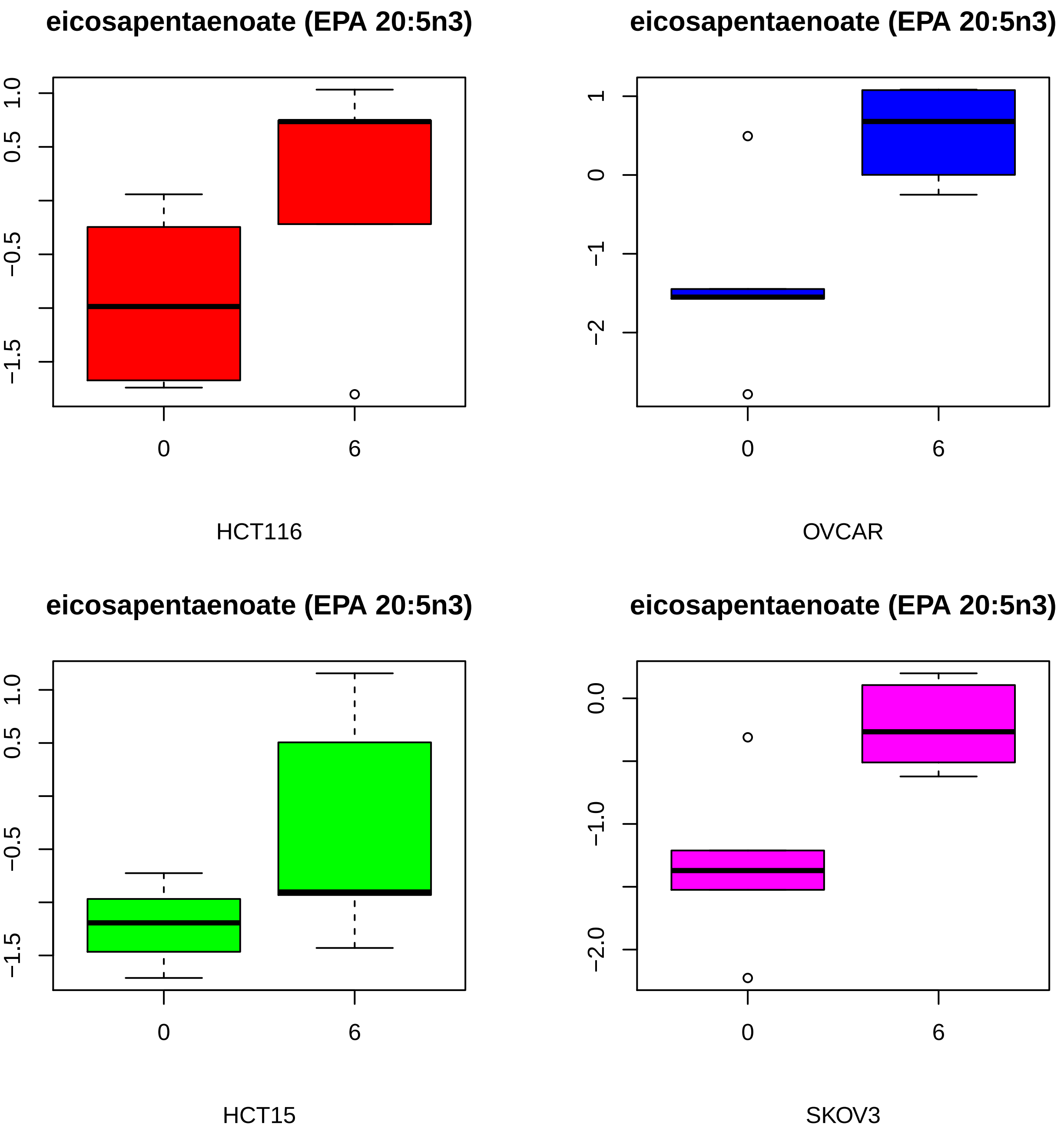
eicosapentaenoate (EPA 20:5n3)
1.0
0.5
−0.5
−1.5
0
6
HCT116
eicosapentaenoate (EPA 20:5n3)
1
0
−1
−2
0
6
OVCAR
eicosapentaenoate (EPA 20:5n3)
1.0
0.5
−0.5
−1.5
0
6
HCT15
eicosapentaenoate (EPA 20:5n3)
0.0
−1.0
−2.0
0
6
SKOV3

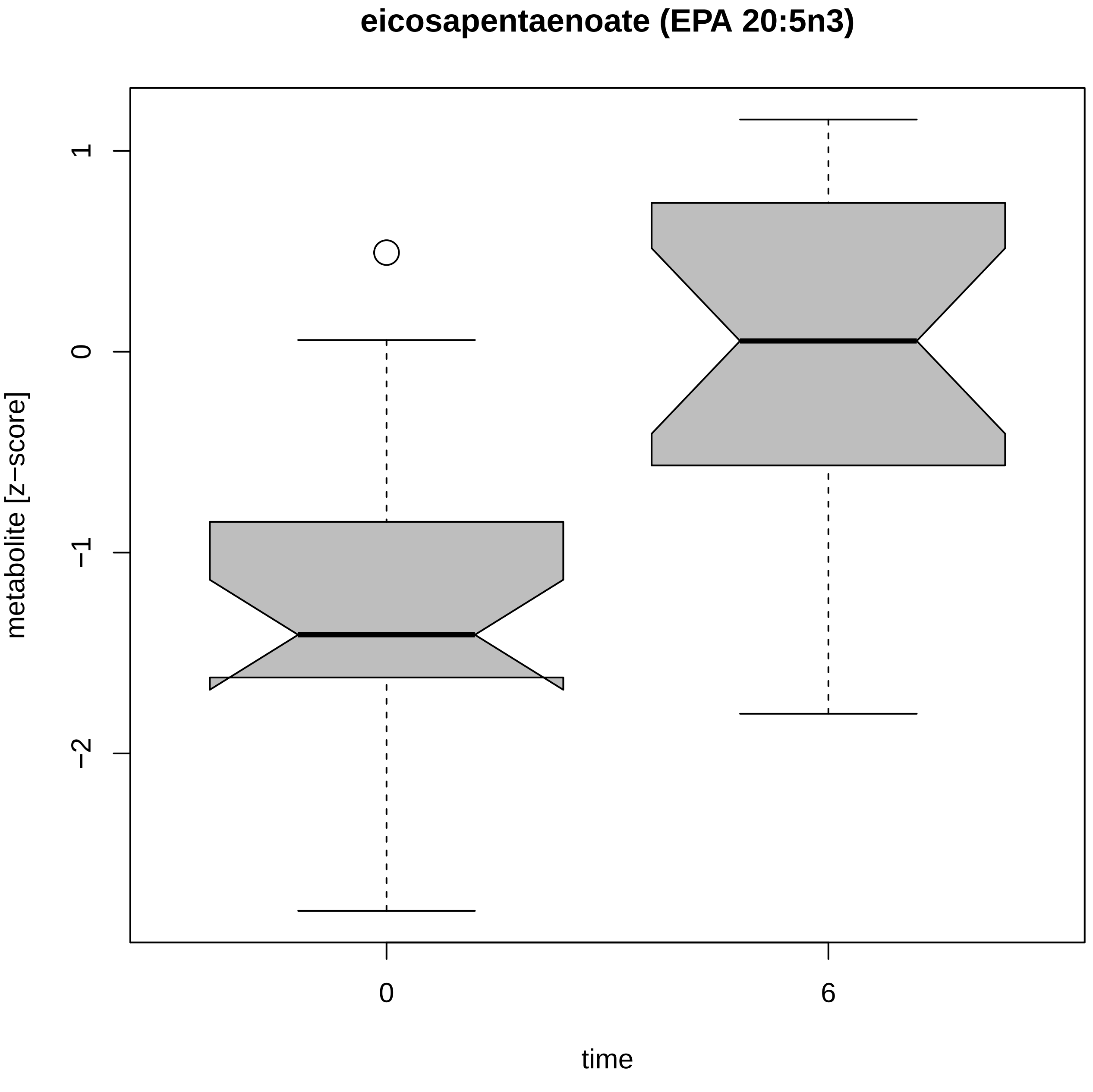
eicosapentaenoate (EPA 20:5n3)
metabolite [z−score]
1
0
−1
−2
0
6
time

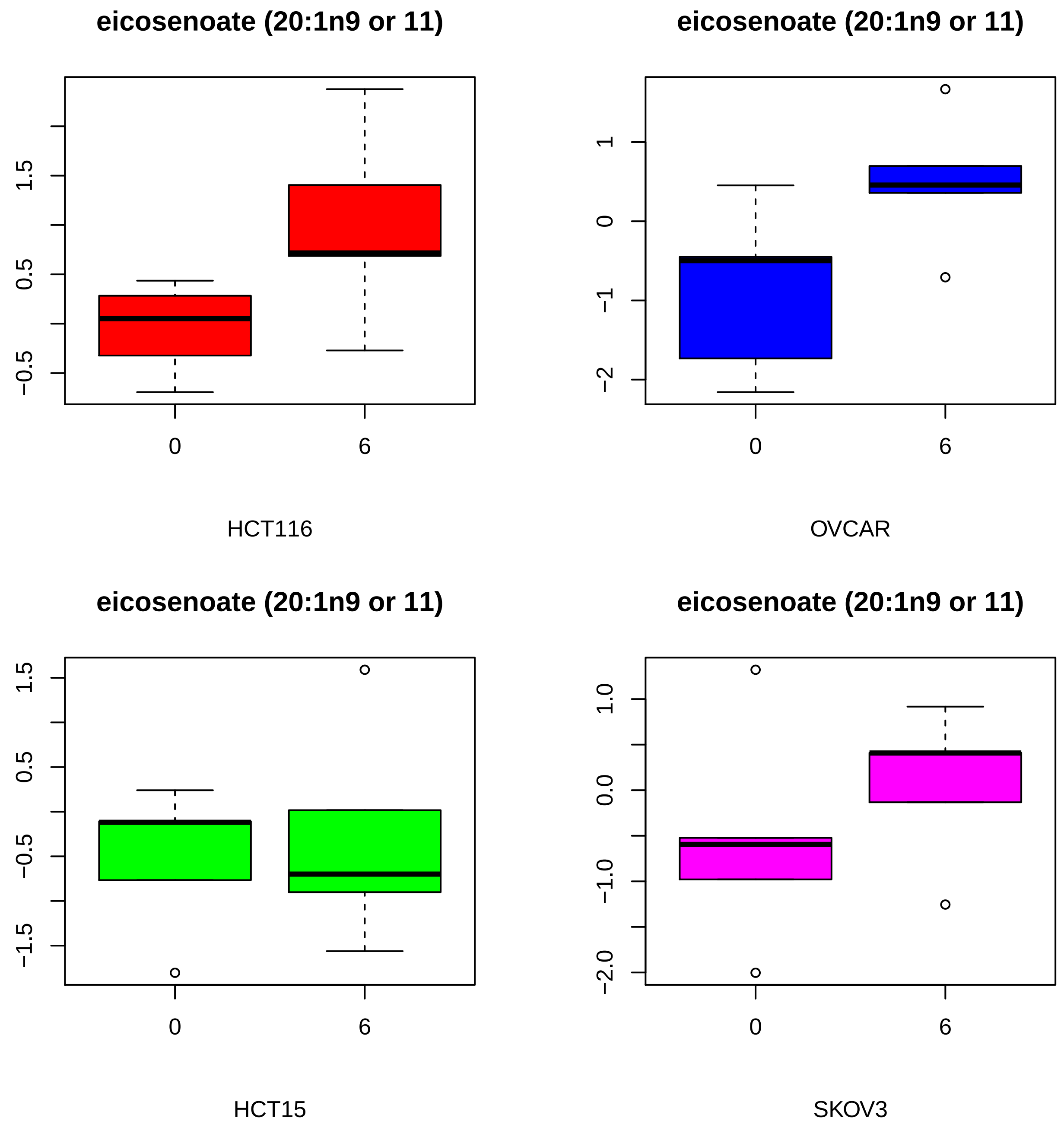
eicosenoate (20:1n9 or 11)
1.5
0.5
−0.5
0
6
HCT116
eicosenoate (20:1n9 or 11)
1
0
−1
−2
0
6
OVCAR
eicosenoate (20:1n9 or 11)
1.5
0.5
−0.5
−1.5
0
6
HCT15
eicosenoate (20:1n9 or 11)
1.0
0.0
−1.0
−2.0
0
6
SKOV3

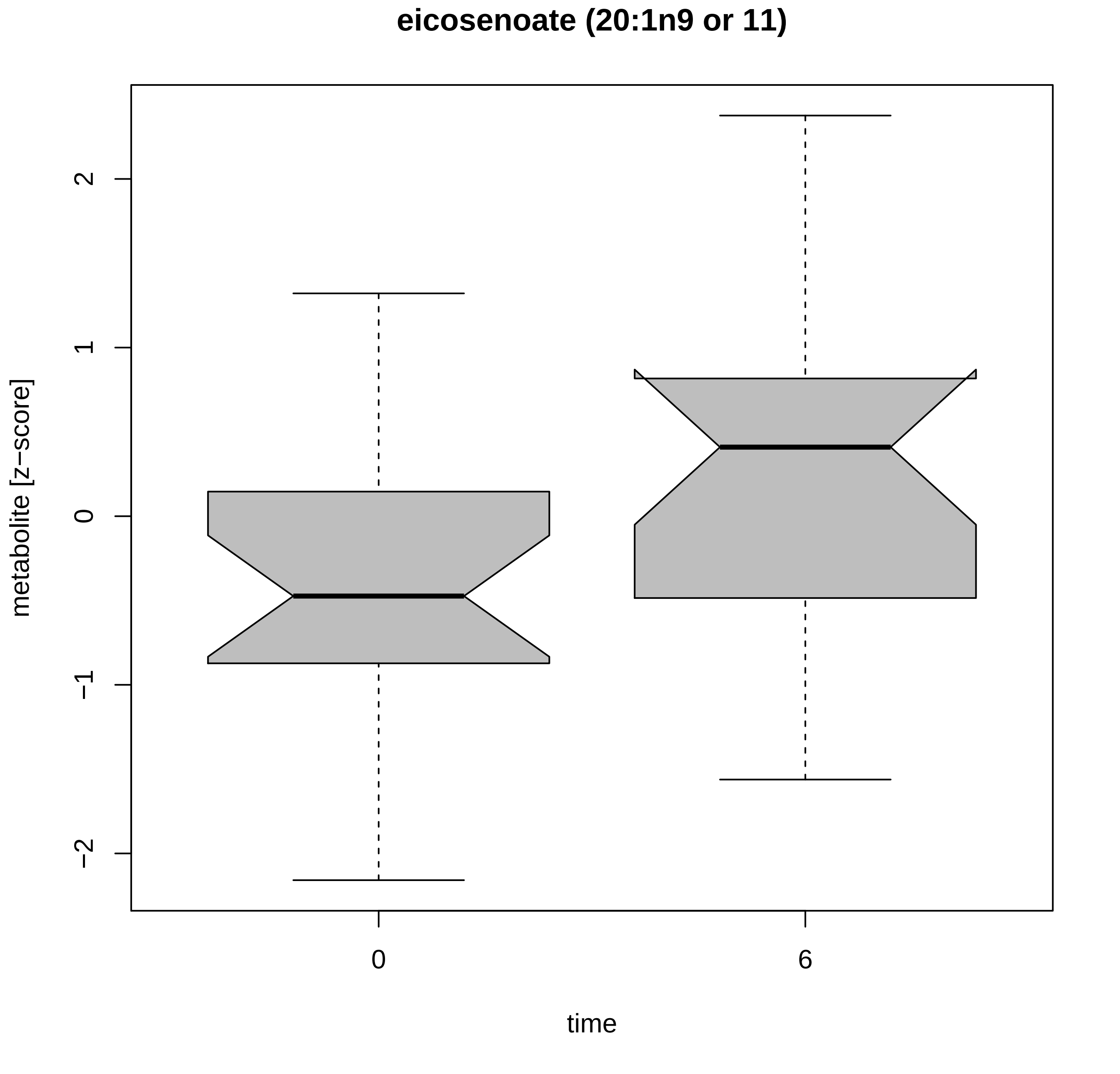
eicosenoate (20:1n9 or 11)
metabolite [z−score]
−2
−1
0
1
2
0
6
time

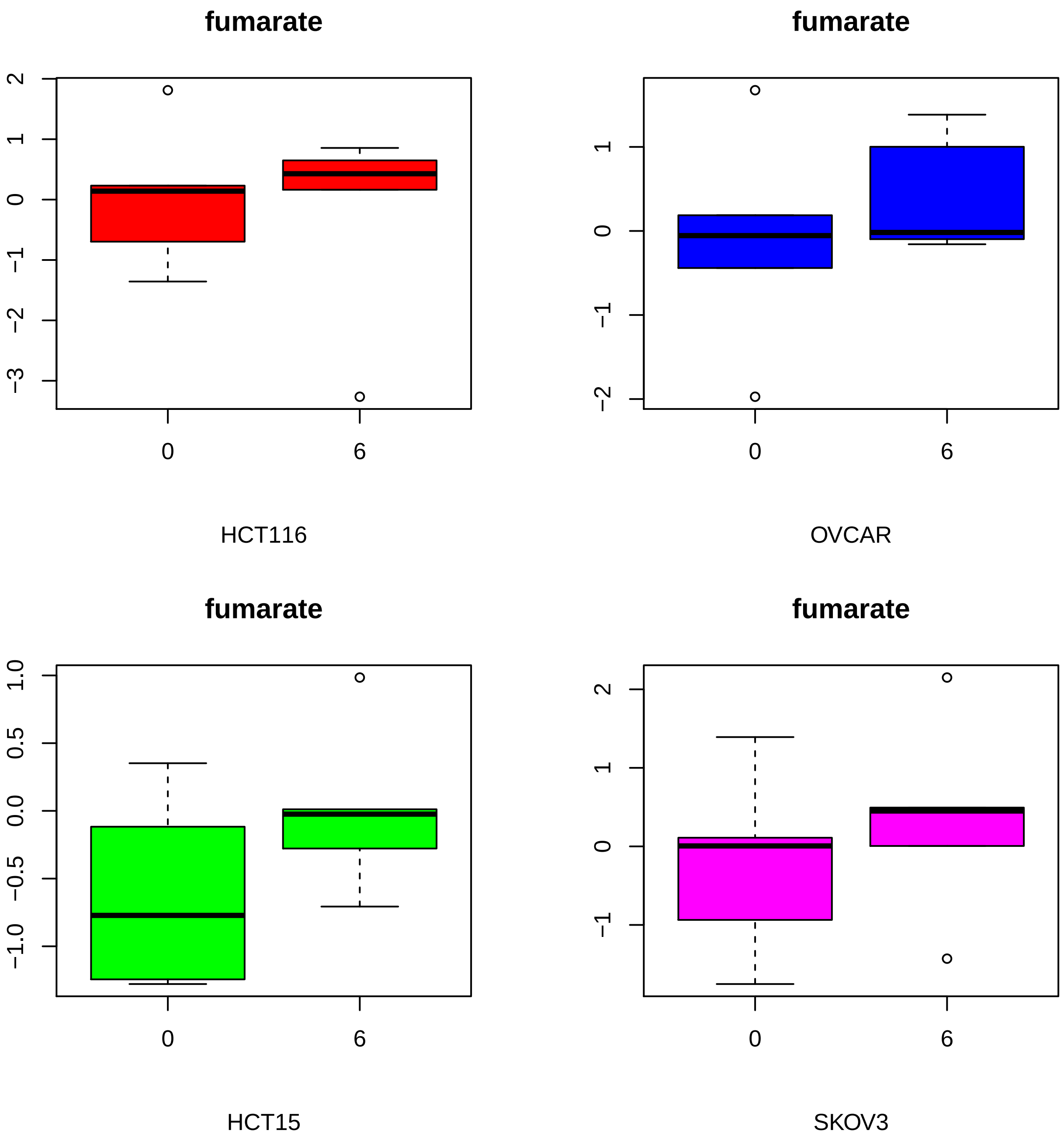
fumarate
HCT116
fumarate
OVCAR
fumarate
HCT15
fumarate
SKOV3
0
6

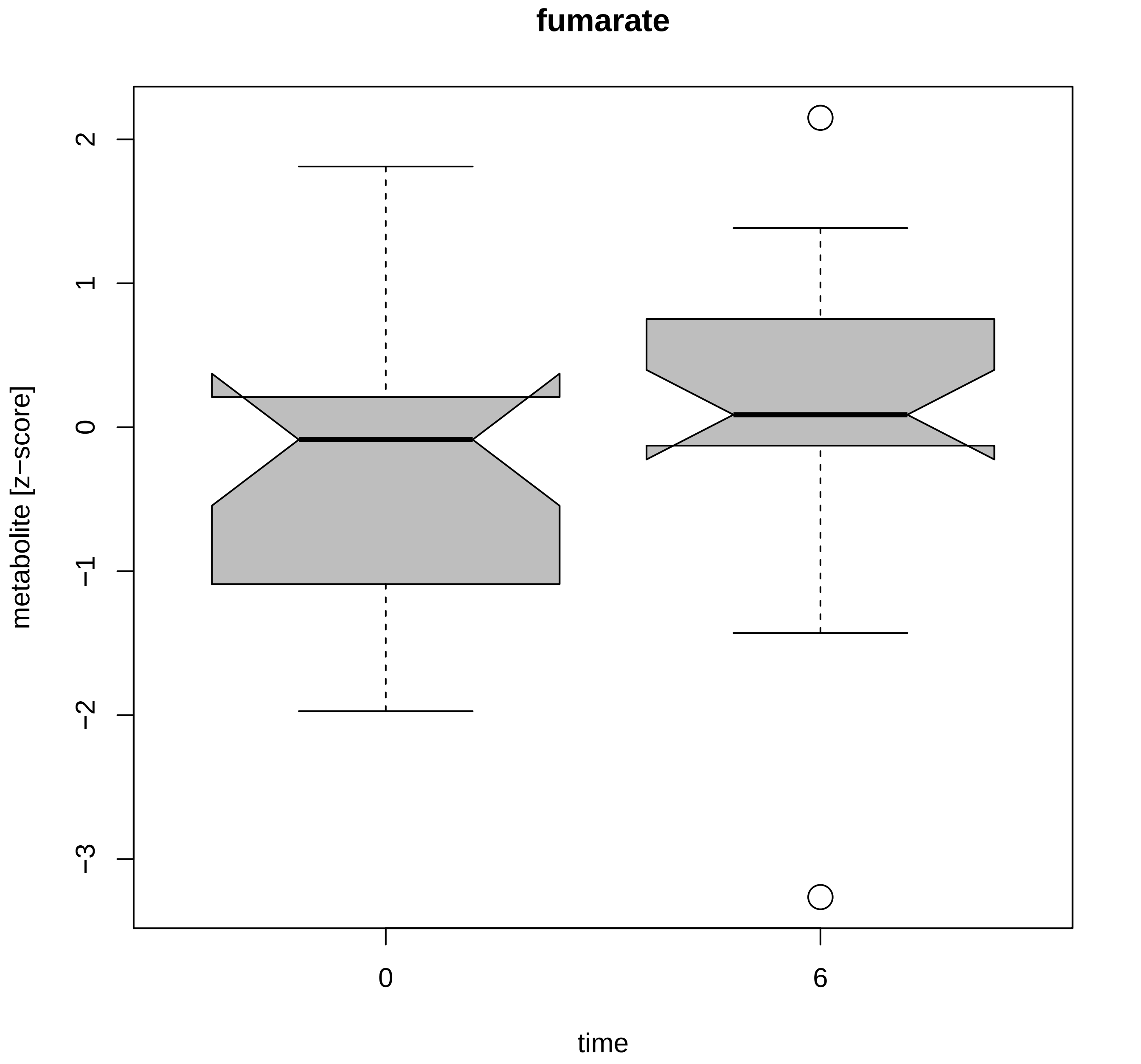

fumarate
metabolite [z−score]
2
1
0
−1
−2
−3
0
6
time

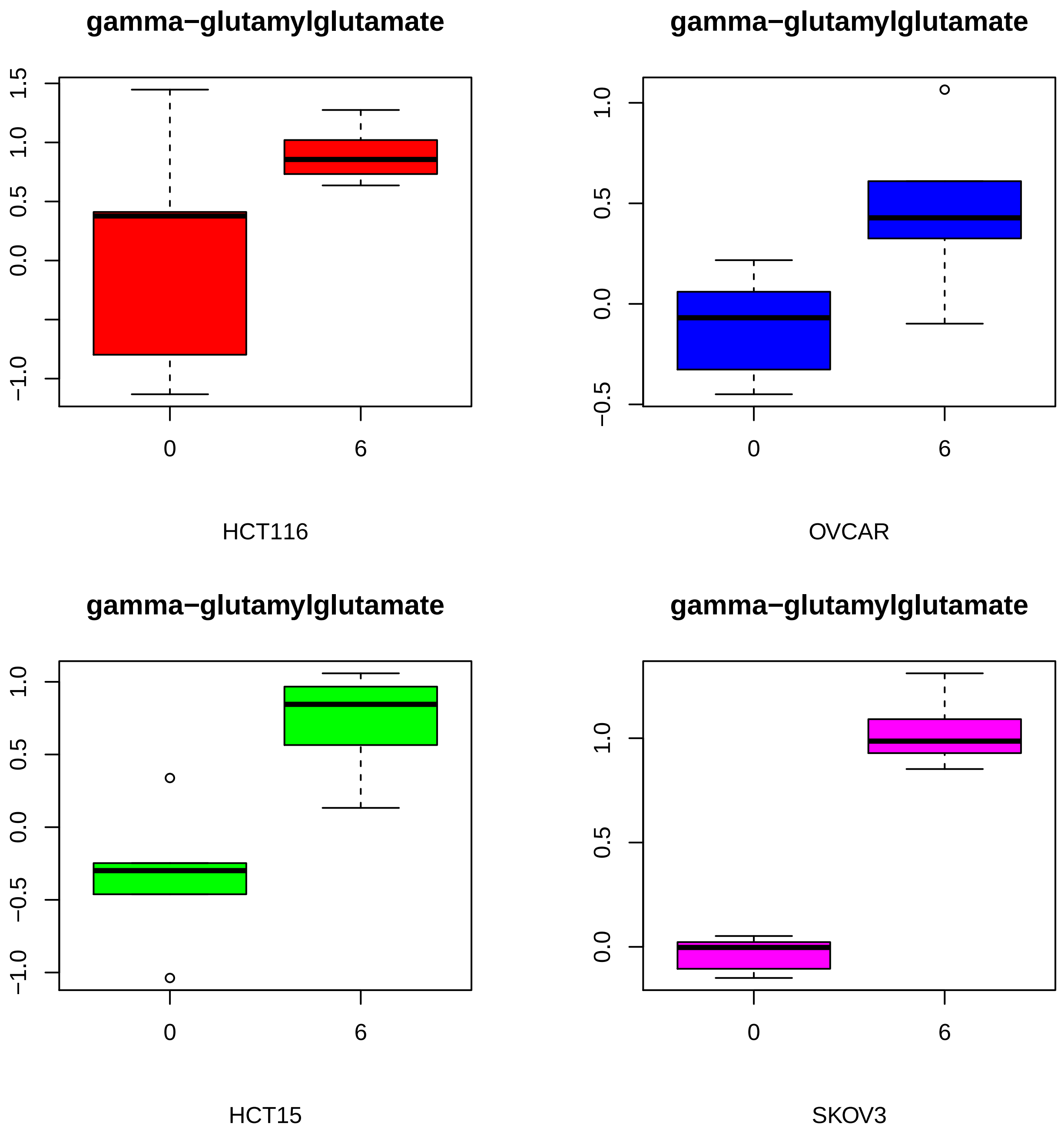
gamma−glutamylglutamate
1.5
1.0
0.5
0.0
−1.0
0
6
HCT116
gamma−glutamylglutamate
1.0
0.5
0.0
−0.5
0
6
OVCAR
gamma−glutamylglutamate
1.0
0.5
0.0
−0.5
−1.0
0
6
HCT15
gamma−glutamylglutamate
1.0
0.5
0.0
0
6
SKOV3

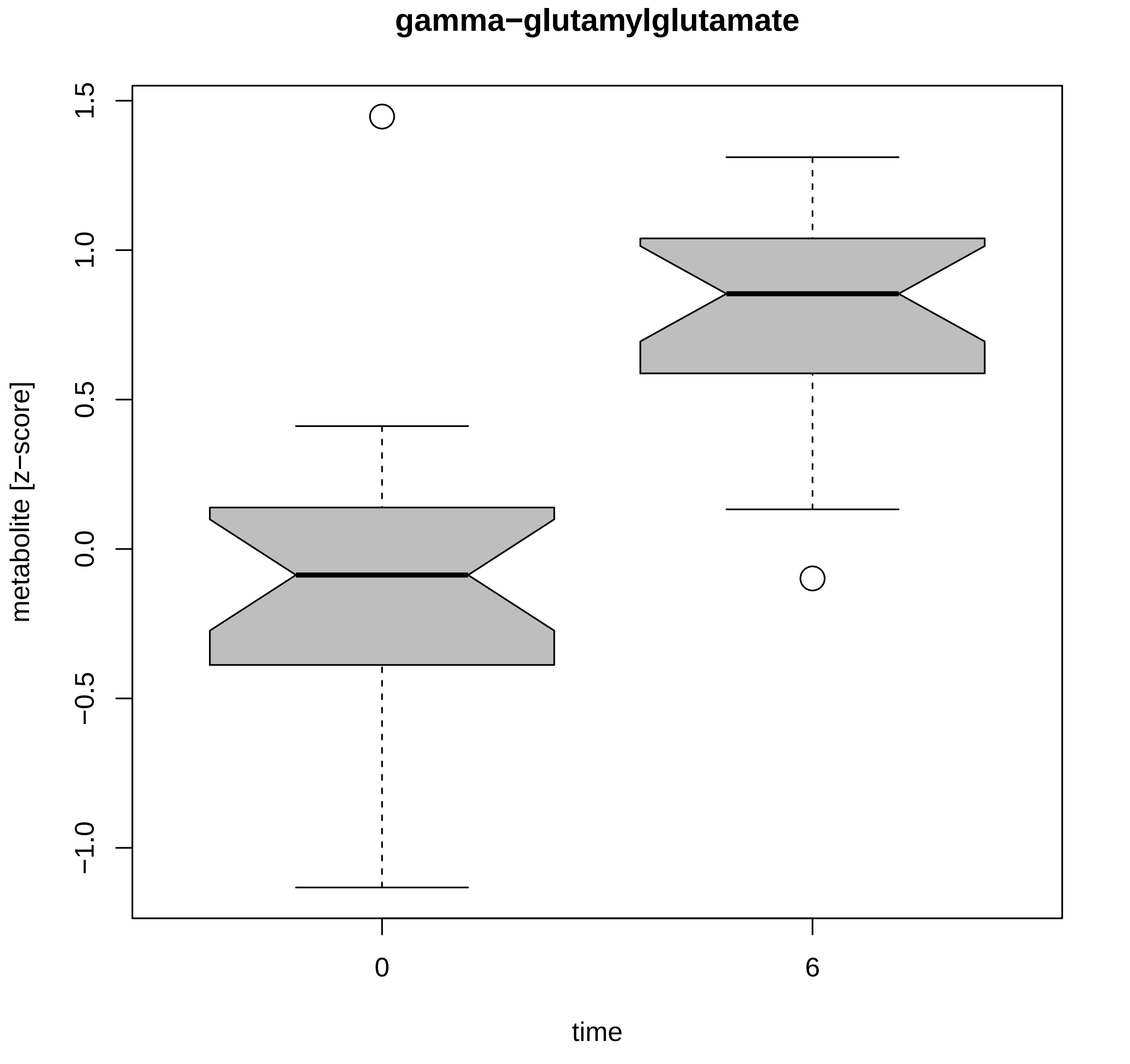
gamma−glutamylglutamate
metabolite [z−score]
1.5
1.0
0.5
0.0
−0.5
−1.0
0
6
time

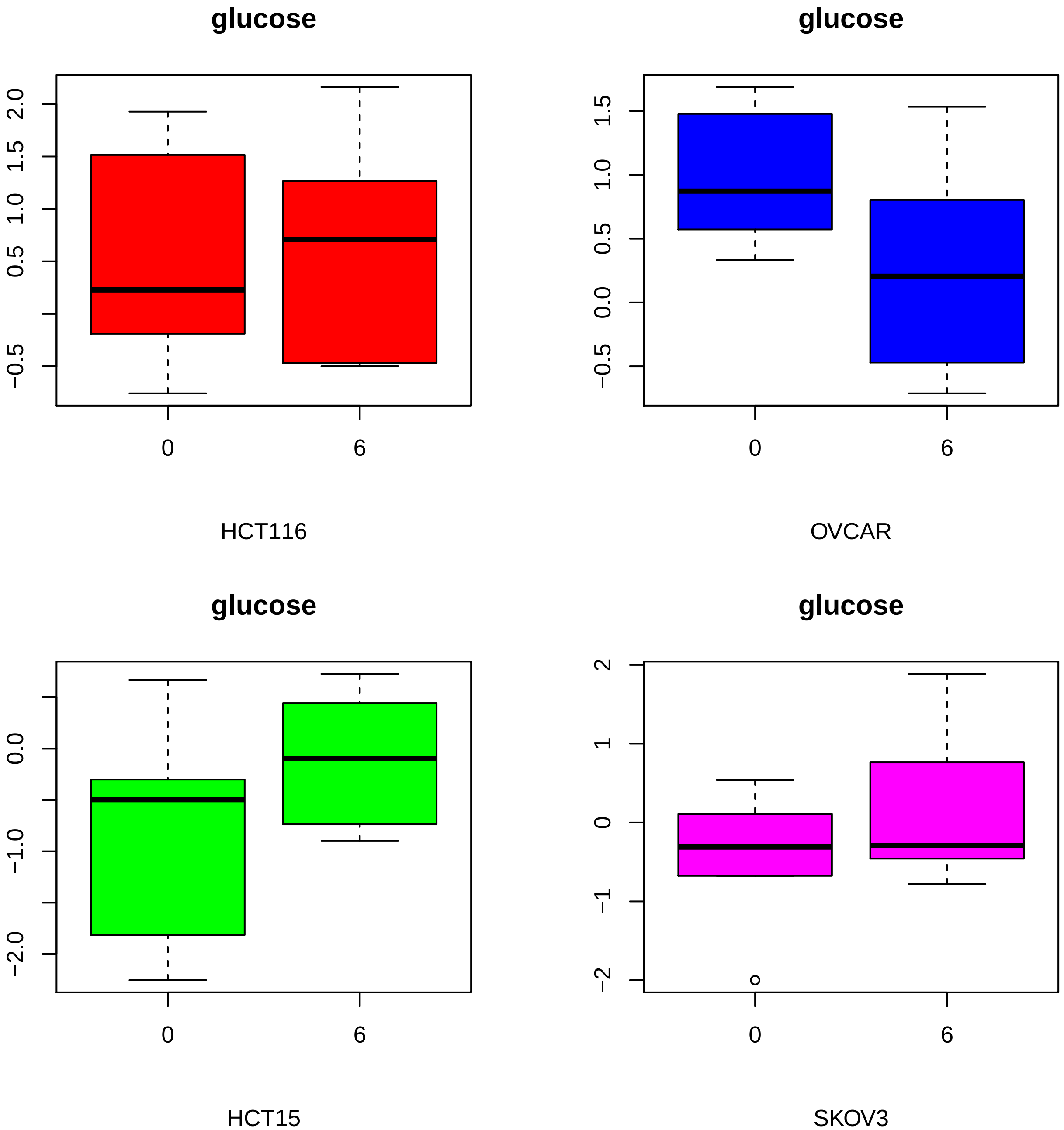

glucose
2.0
1.5
1.0
0.5
−0.5
0
6
HCT116
glucose
1.5
1.0
0.5
0.0
−0.5
0
6
OVCAR
glucose
0.0
−1.0
−2.0
0
6
HCT15
glucose
2
1
0
−1
−2
0
6
SKOV3

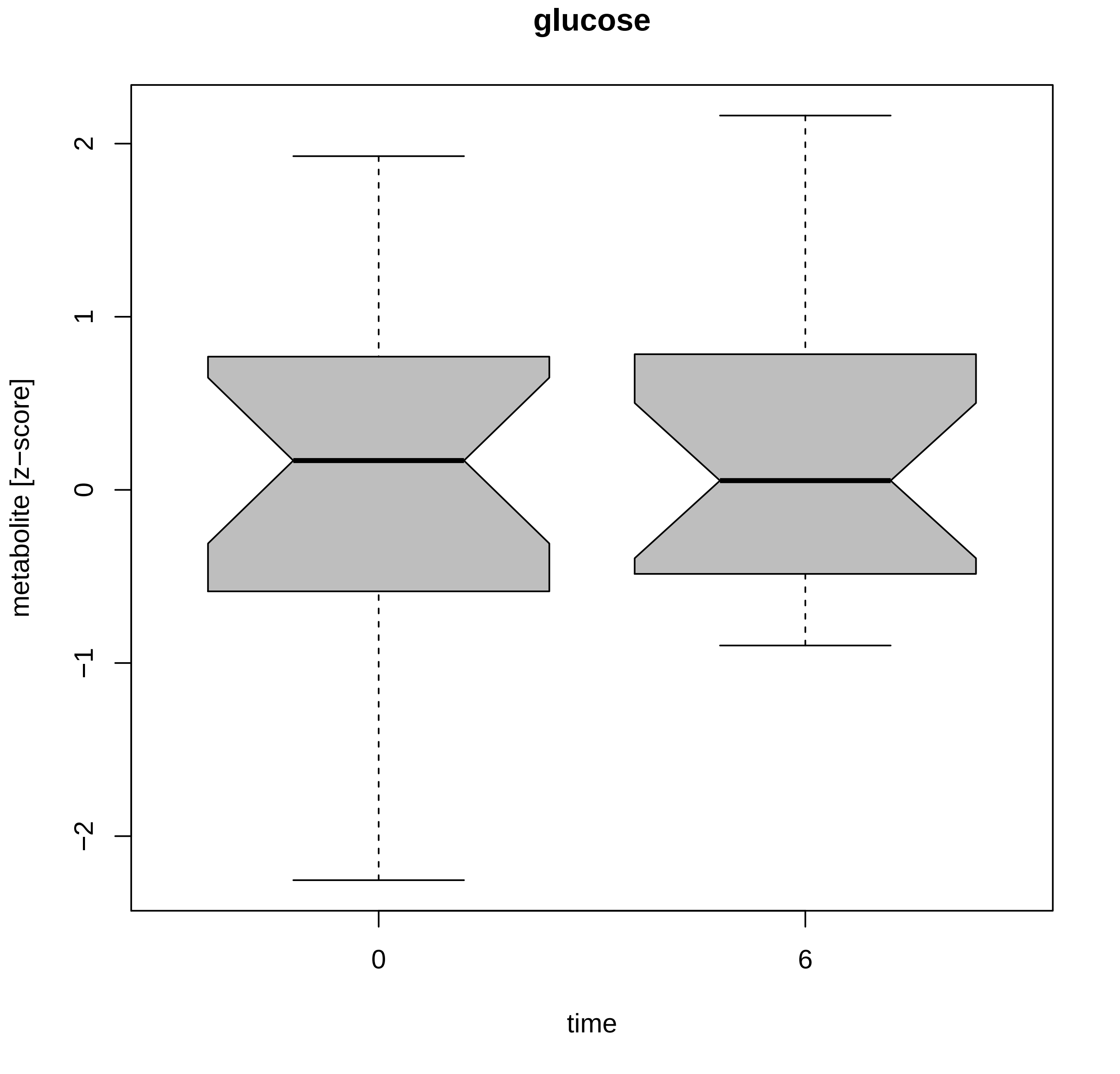
glucose
metabolite [z−score]
2
1
0
−1
−2
0
6
time

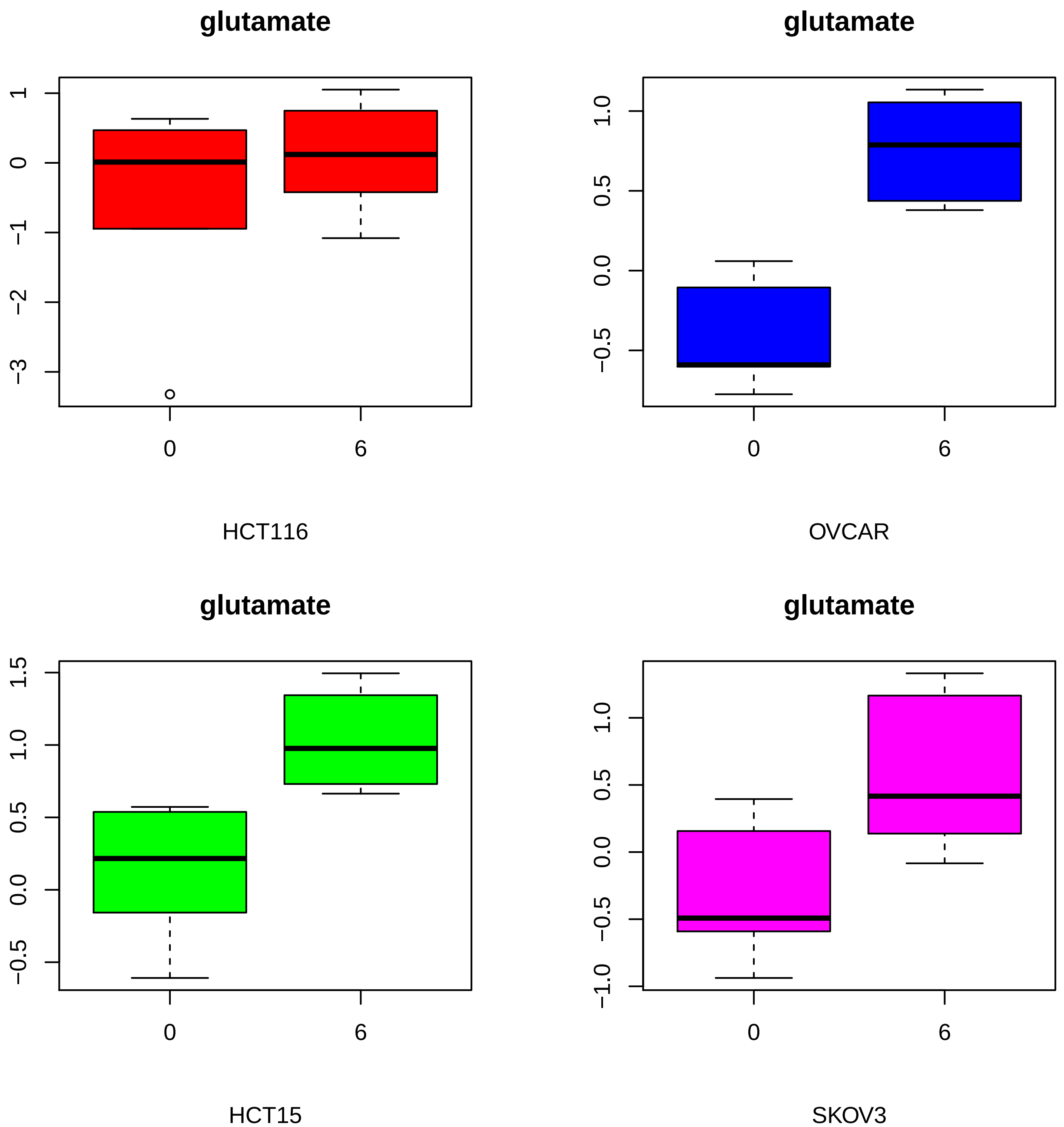
glutamate
1
0
−1
−2
−3
0
6
HCT116
glutamate
1.0
0.5
0.0
−0.5
0
6
OVCAR
glutamate
1.5
1.0
0.5
0.0
−0.5
0
6
HCT15
glutamate
1.0
0.5
0.0
−0.5
−1.0
0
6
SKOV3

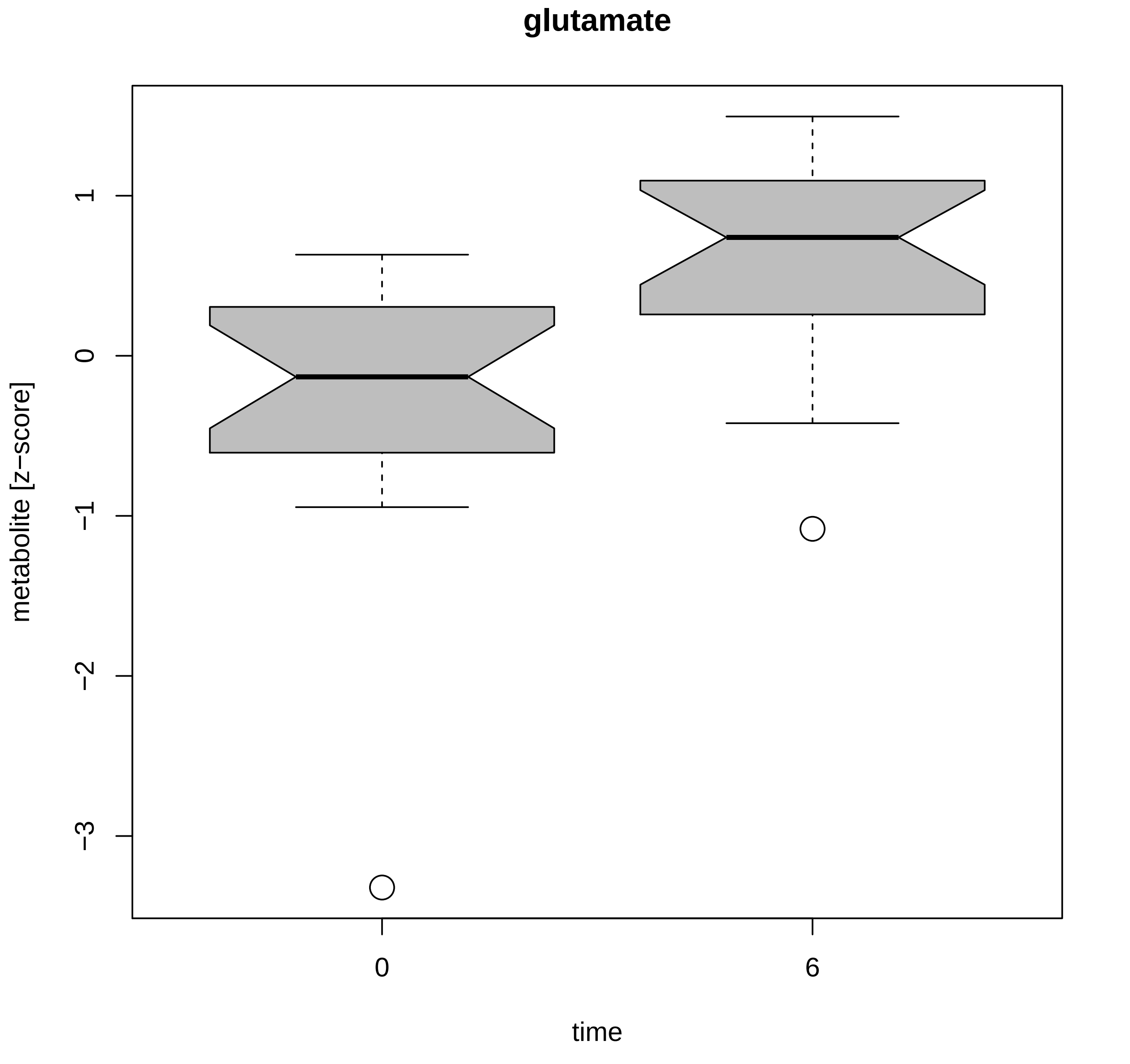
glutamate
metabolite [z−score]
1
0
−1
−2
−3
0
6
time

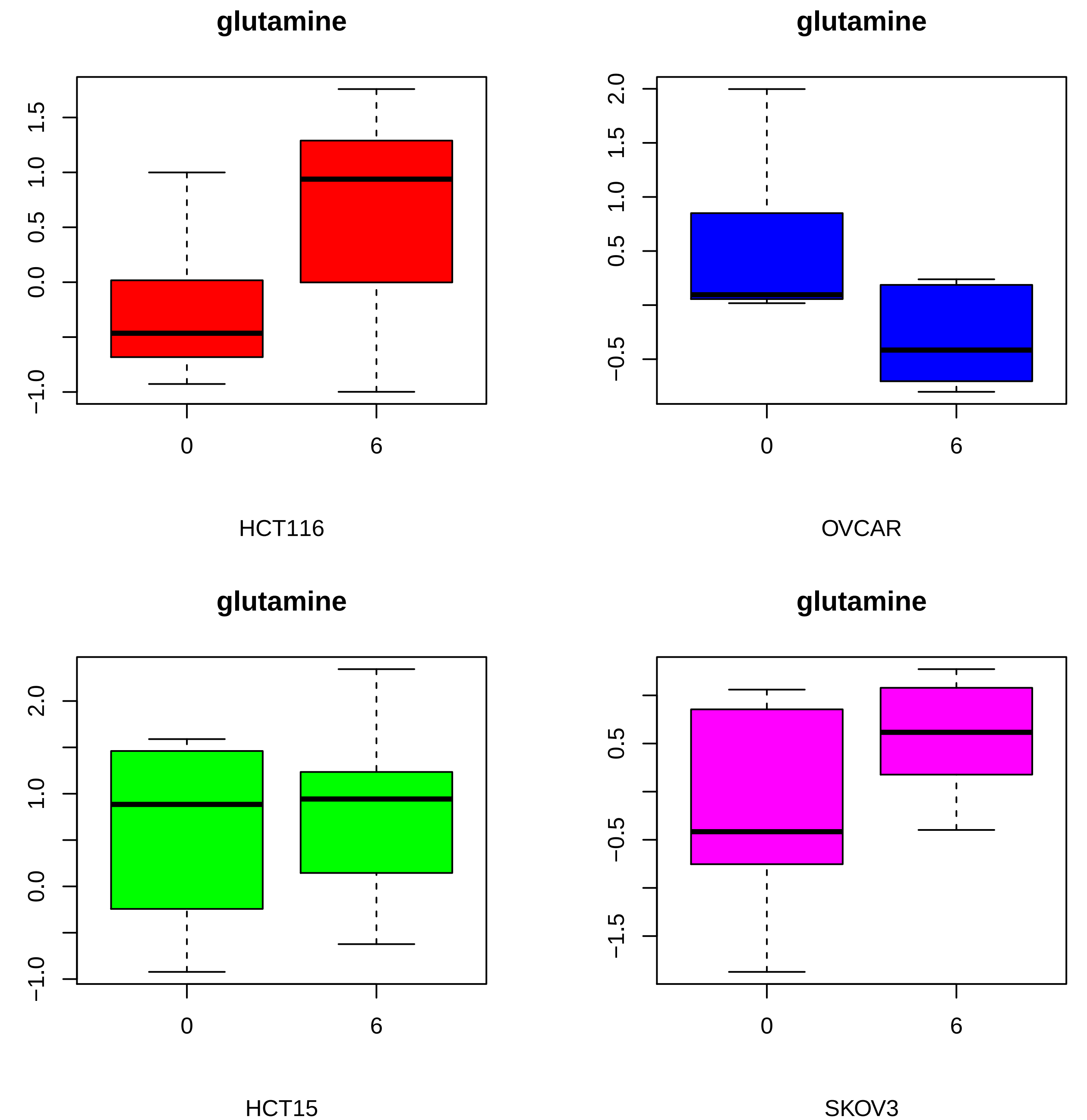
glutamine
1.5
1.0
0.5
0.0
−1.0
0
6
HCT116
glutamine
2.0
1.5
1.0
0.5
−0.5
0
6
OVCAR
glutamine
2.0
1.0
0.0
−1.0
0
6
HCT15
glutamine
0.5
−0.5
−1.5
0
6
SKOV3

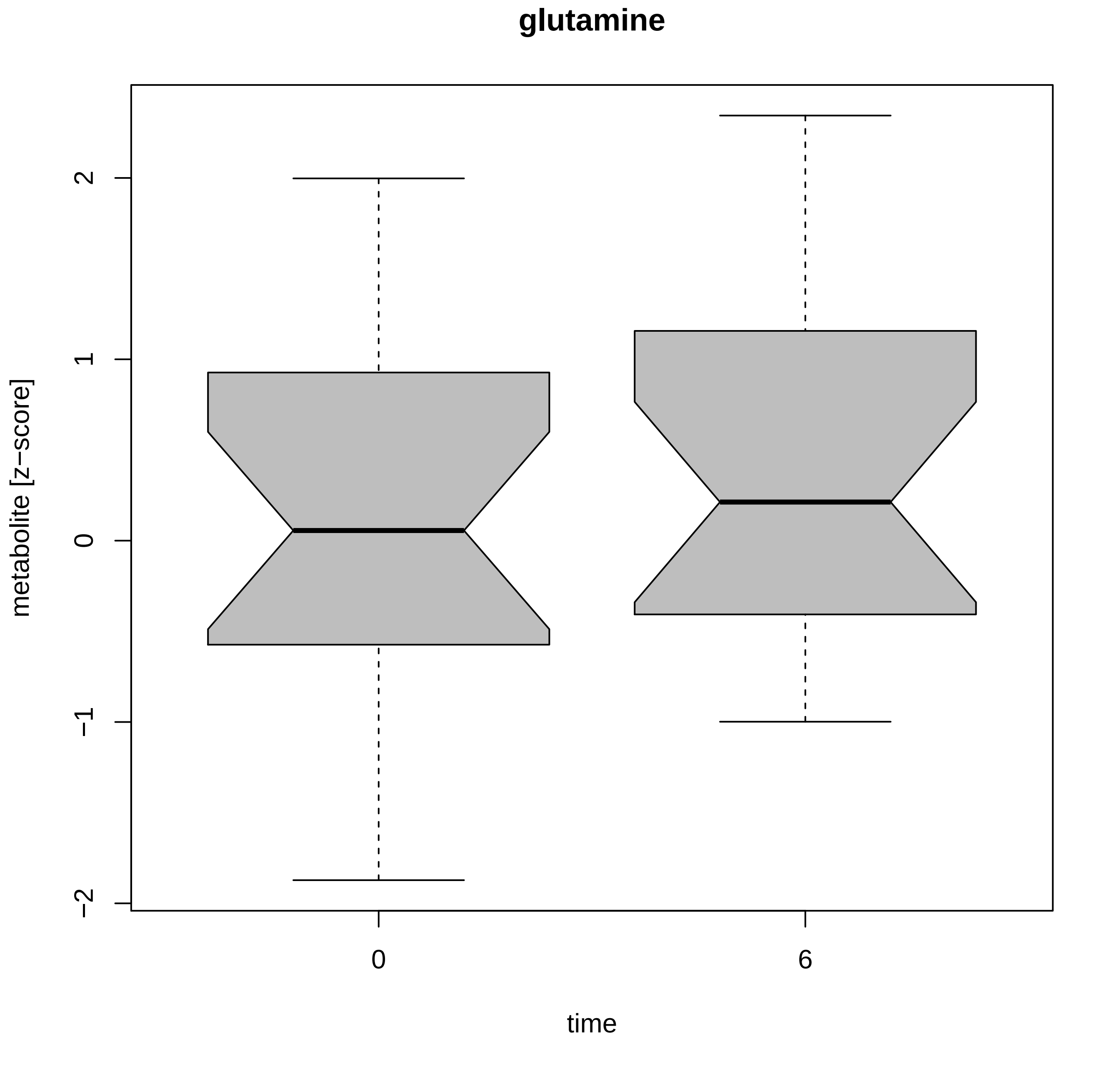
glutamine
metabolite [z−score]
2
1
0
−1
−2
0
6
time

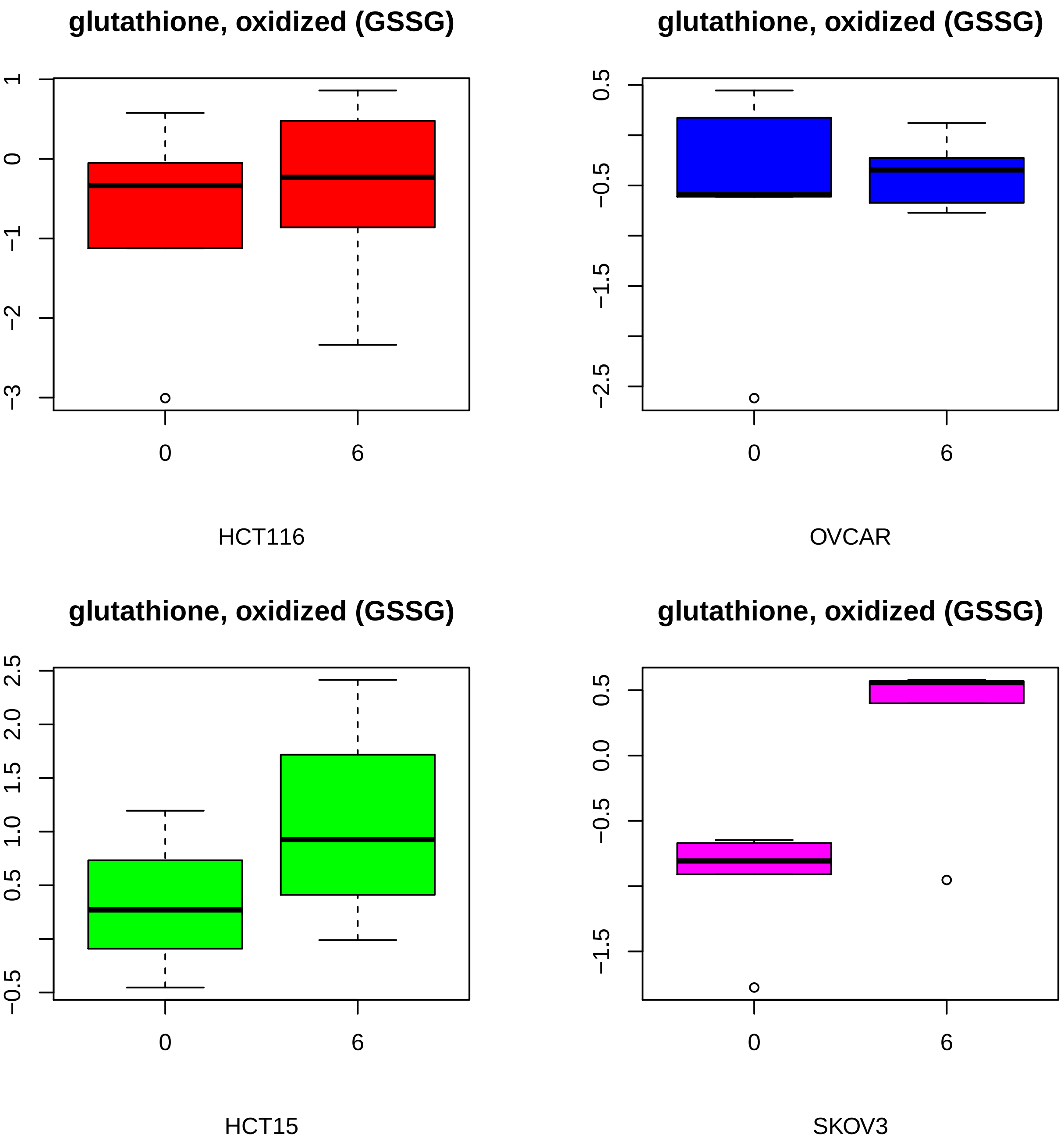

glutathione, oxidized (GSSG)
1
0
−1
−2
−3
0
6
HCT116
glutathione, oxidized (GSSG)
0.5
−0.5
−1.5
−2.5
0
6
OVCAR
glutathione, oxidized (GSSG)
2.5
2.0
1.5
1.0
0.5
−0.5
0
6
HCT15
glutathione, oxidized (GSSG)
0.5
0.0
−0.5
−1.5
0
6
SKOV3

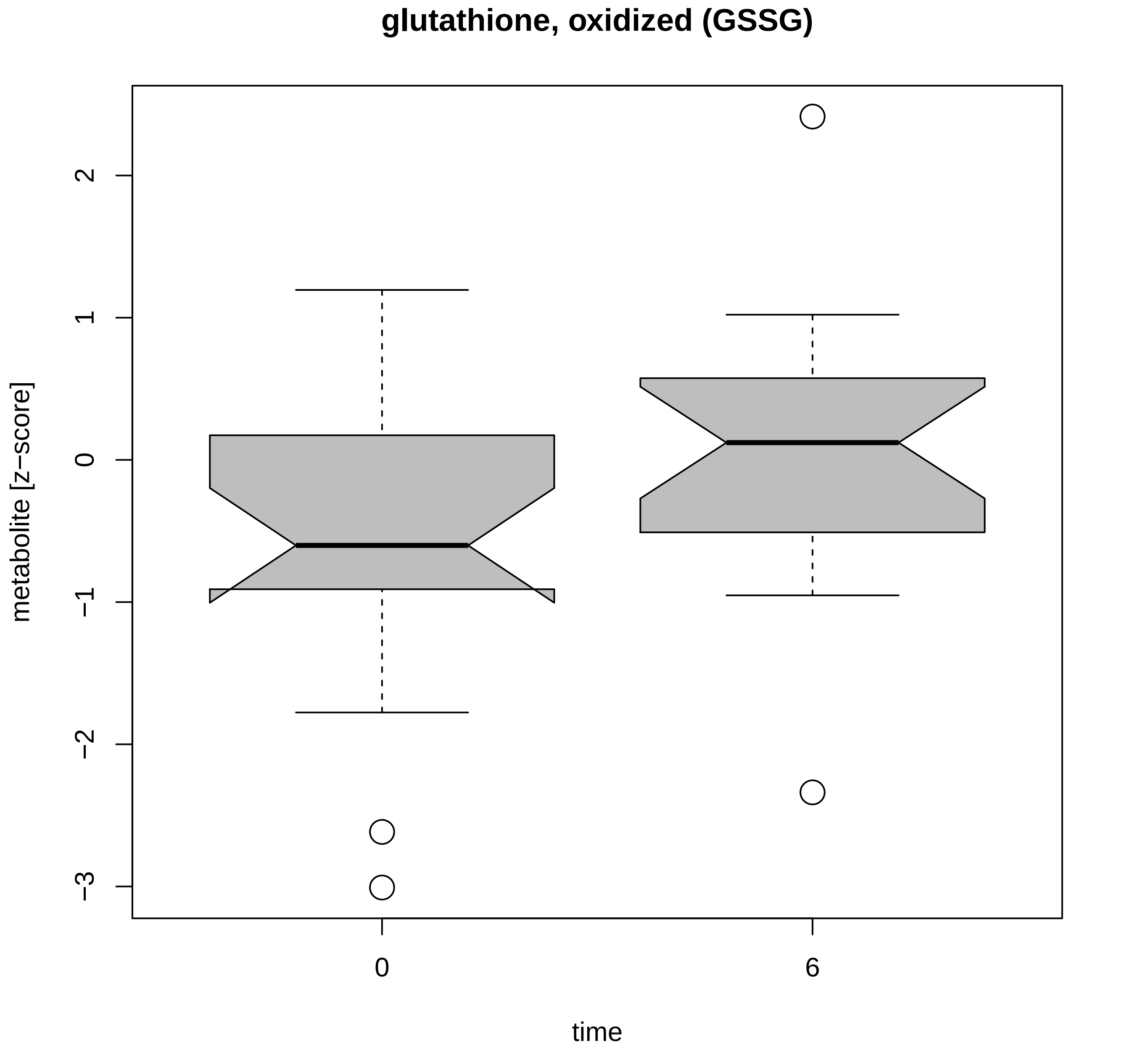
glutathione, oxidized (GSSG)
metabolite [z−score]
2
1
0
−1
−2
−3
0
6
time

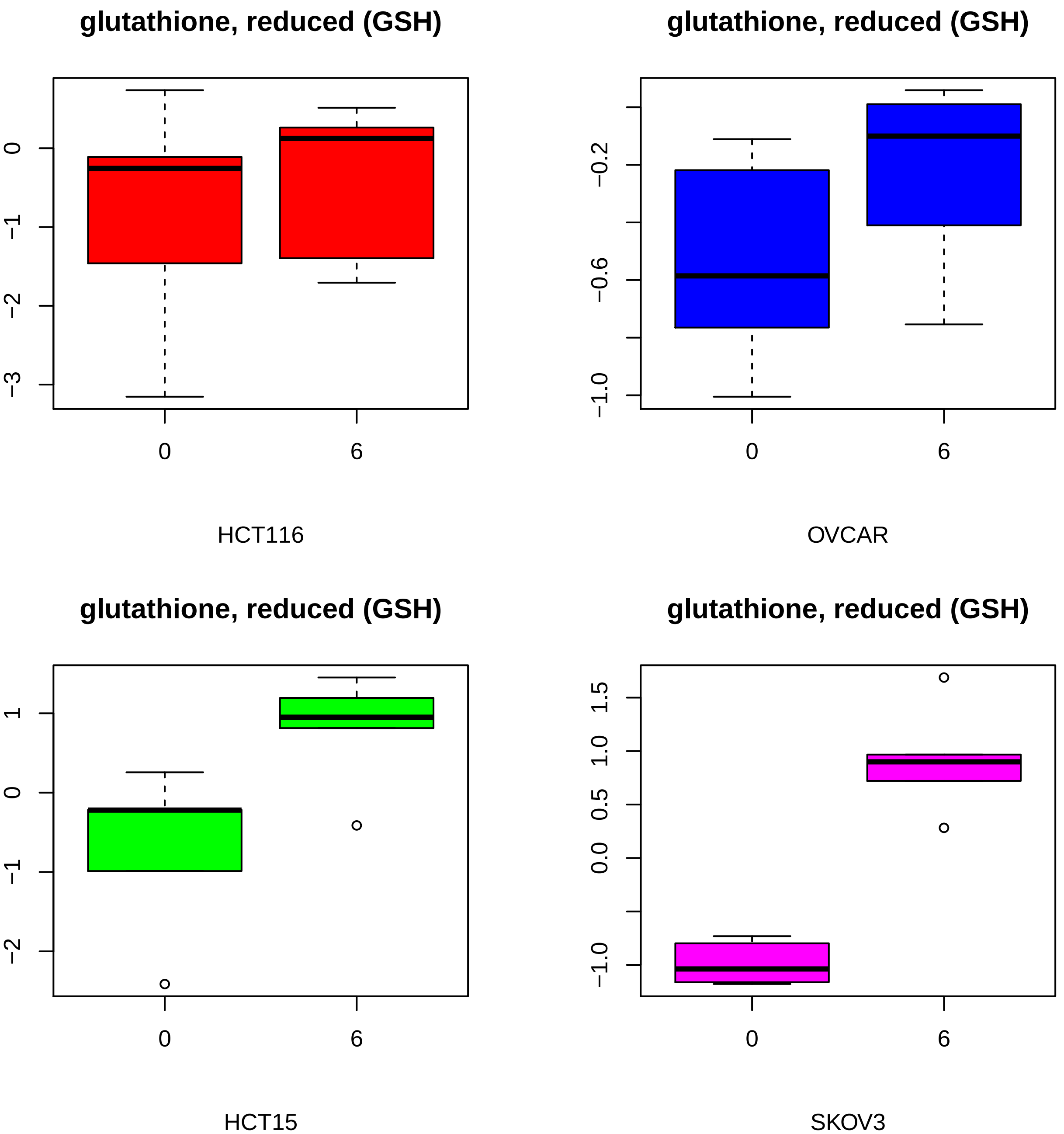

glutathione, reduced (GSH)
0
−1
−2
−3
0
6
HCT116
glutathione, reduced (GSH)
−0.2
−0.6
−1.0
0
6
OVCAR
glutathione, reduced (GSH)
1
0
−1
−2
0
6
HCT15
glutathione, reduced (GSH)
1.5
1.0
0.5
0.0
−1.0
0
6
SKOV3

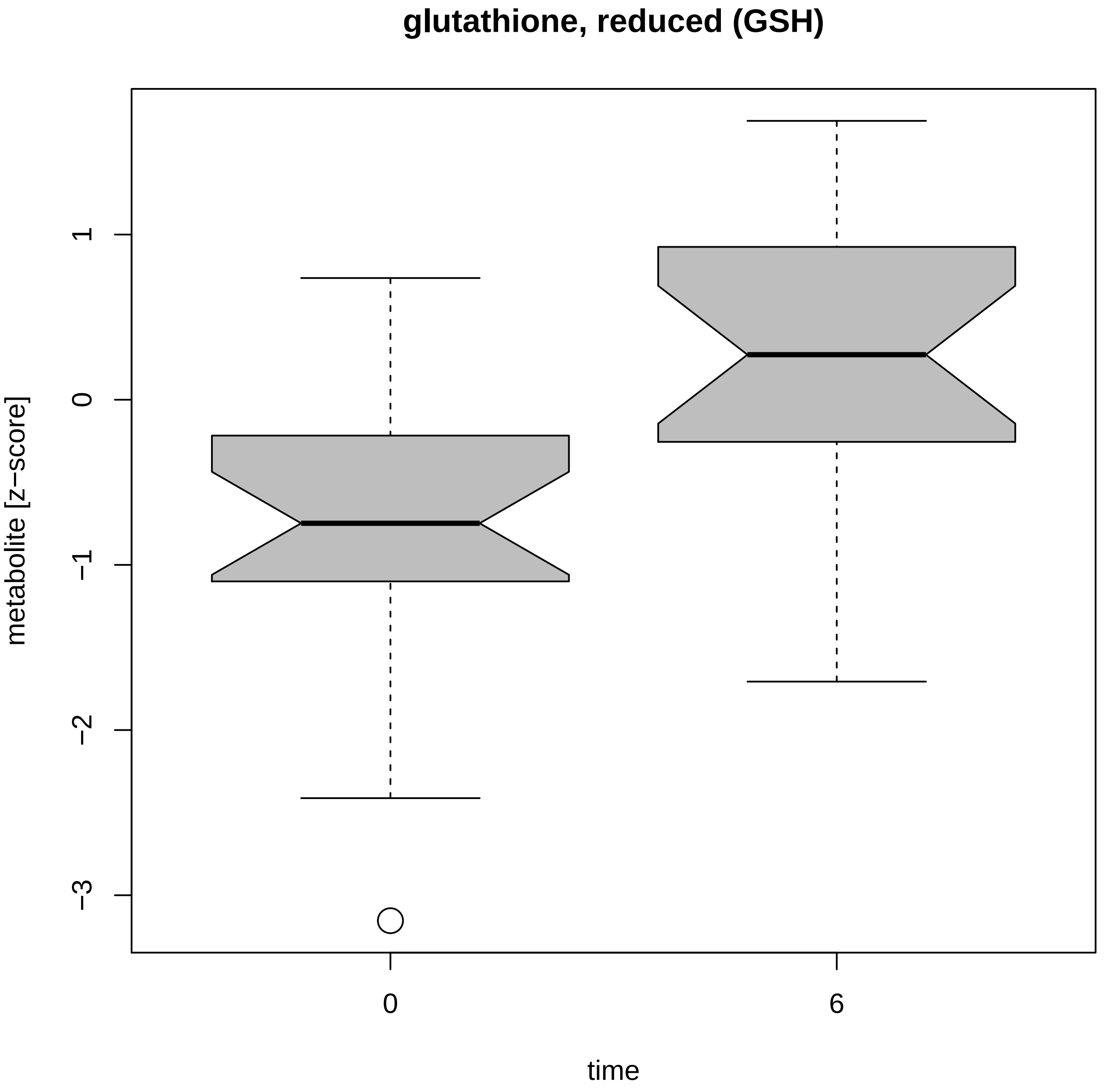

glutathione, reduced (GSH)
metabolite [z−score]
1
0
−1
−2
−3
0
6
time

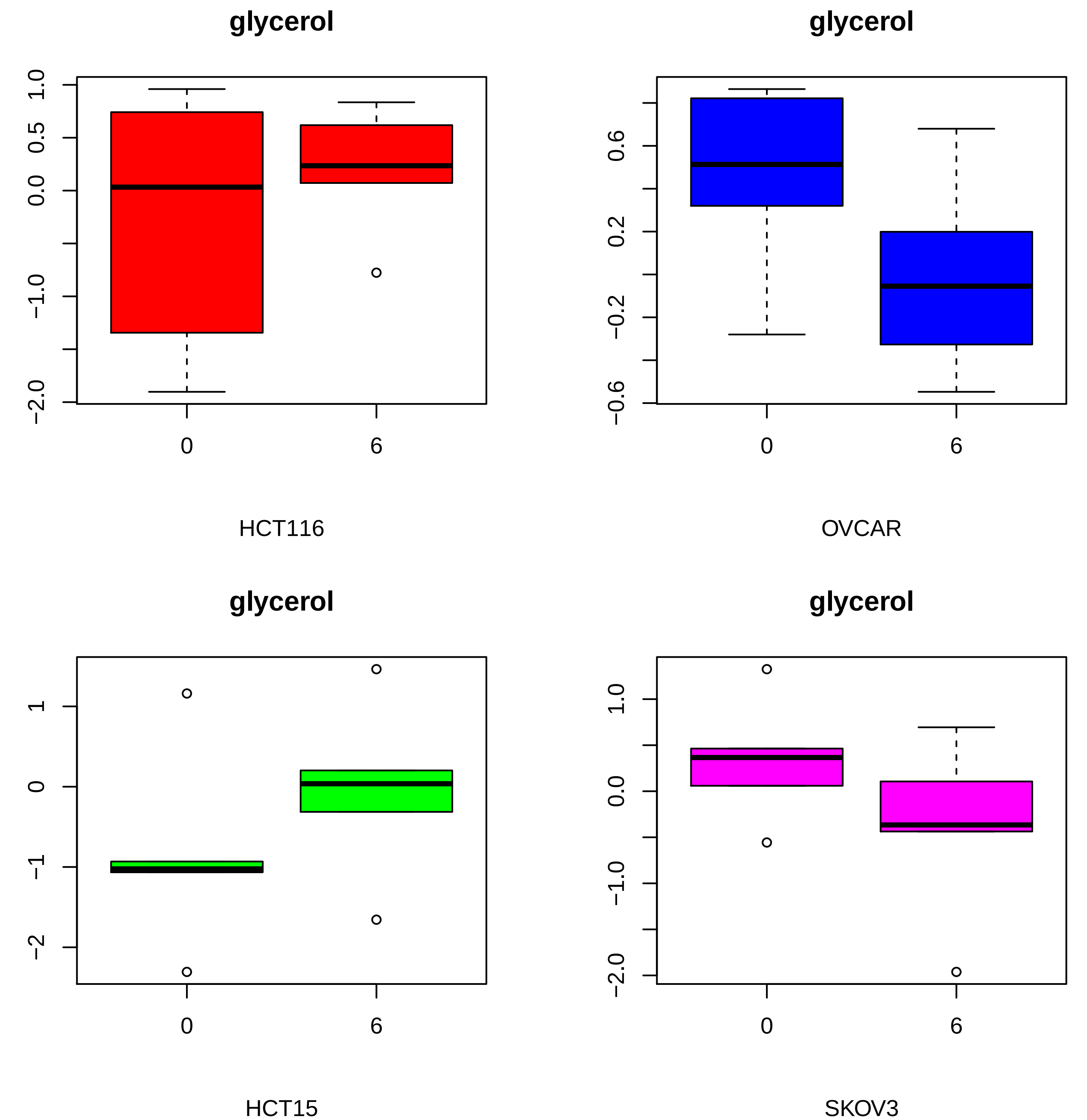

glycerol
1.0
0.5
0.0
−1.0
−2.0
0
6
HCT116
glycerol
0.6
0.2
−0.2
−0.6
0
6
OVCAR
glycerol
1
0
−1
−2
0
6
HCT15
glycerol
1.0
0.0
−1.0
−2.0
0
6
SKOV3

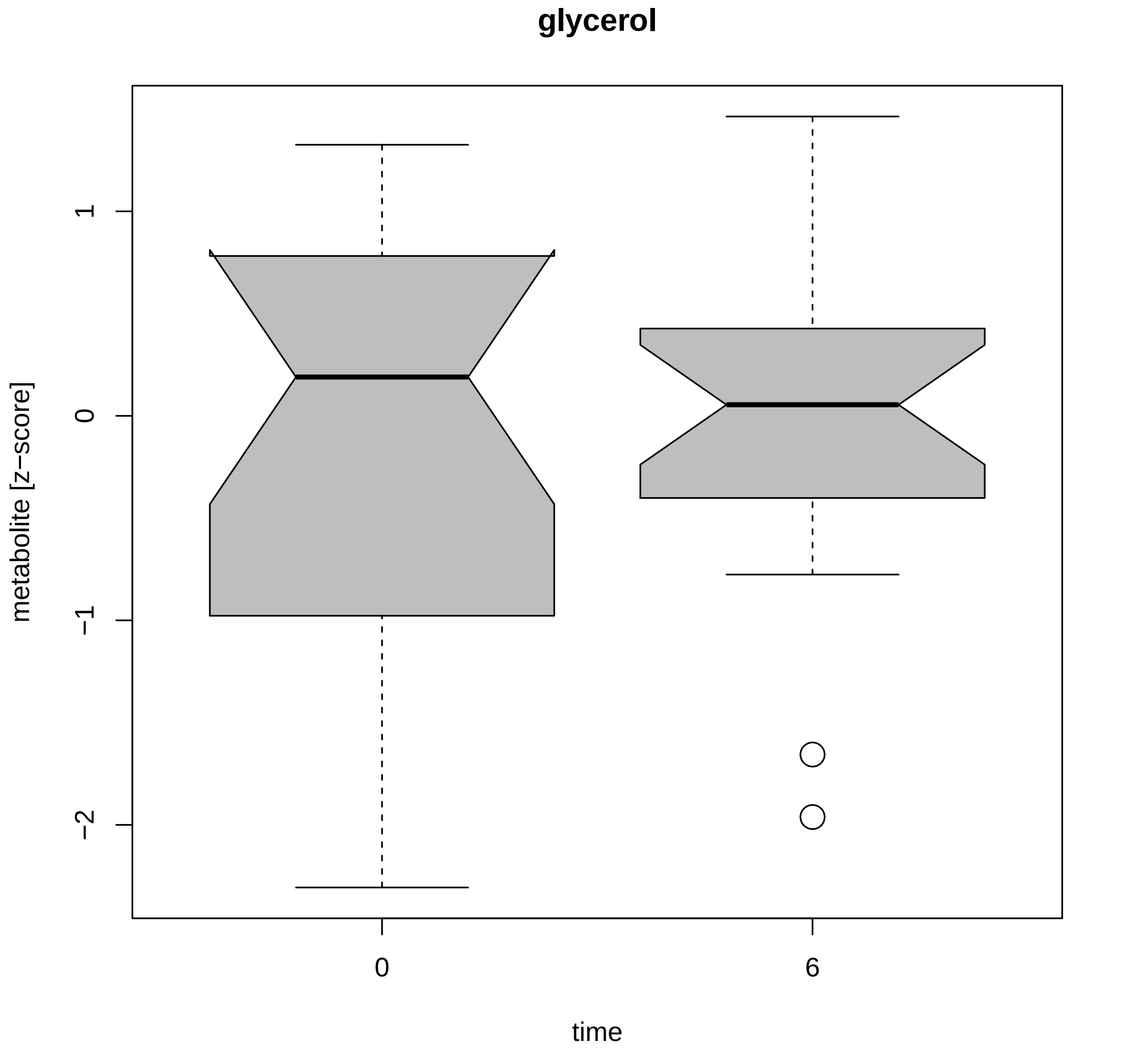
glycerol
metabolite [z−score]
1
0
−1
−2
0
6
time

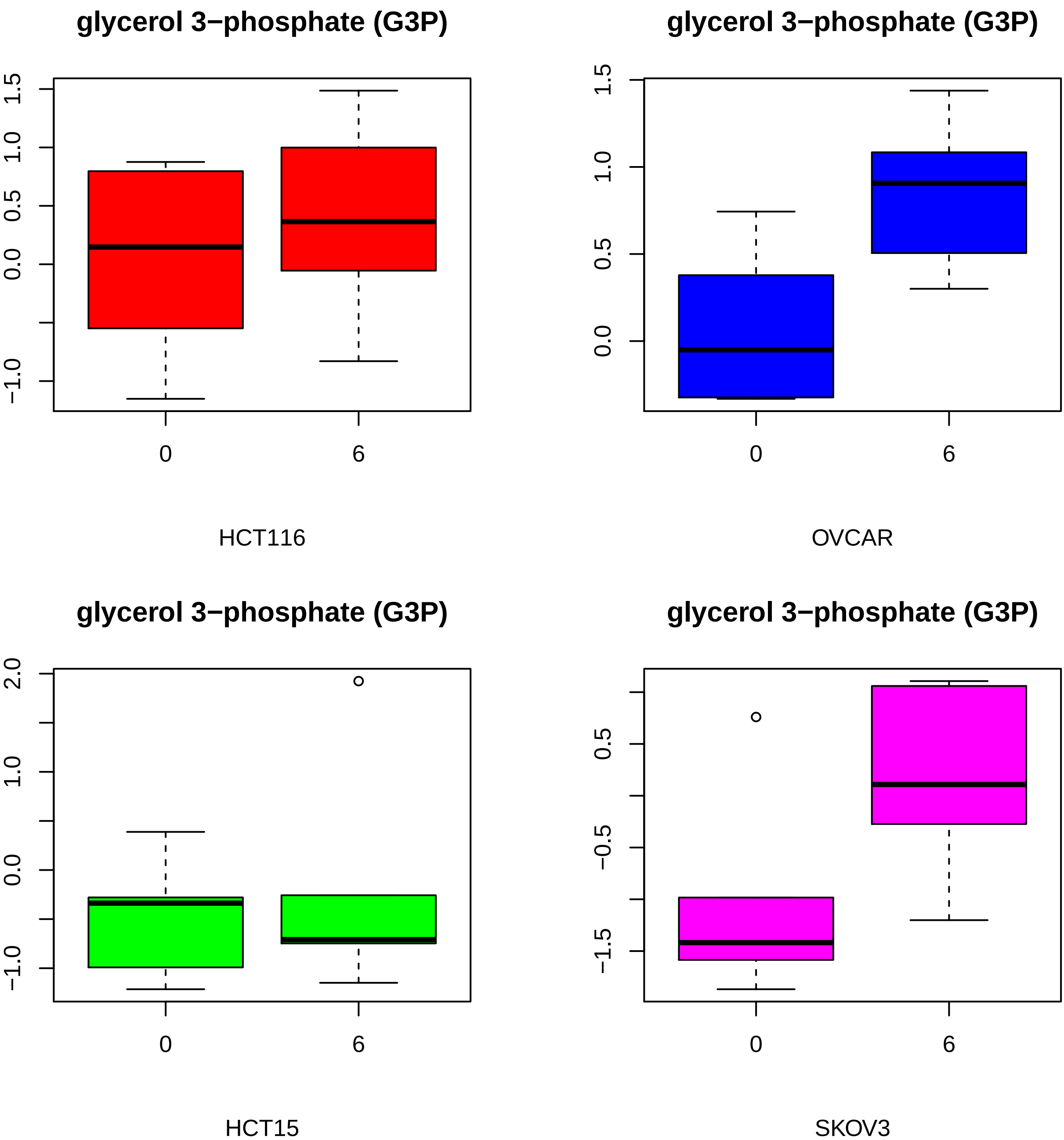
glycerol 3−phosphate (G3P)
1.5
1.0
0.5
0.0
−1.0
0
6
HCT116
glycerol 3−phosphate (G3P)
1.5
1.0
0.5
0.0
0
6
OVCAR
glycerol 3−phosphate (G3P)
2.0
1.0
0.0
−1.0
0
6
HCT15
glycerol 3−phosphate (G3P)
0.5
−0.5
−1.5
0
6
SKOV3

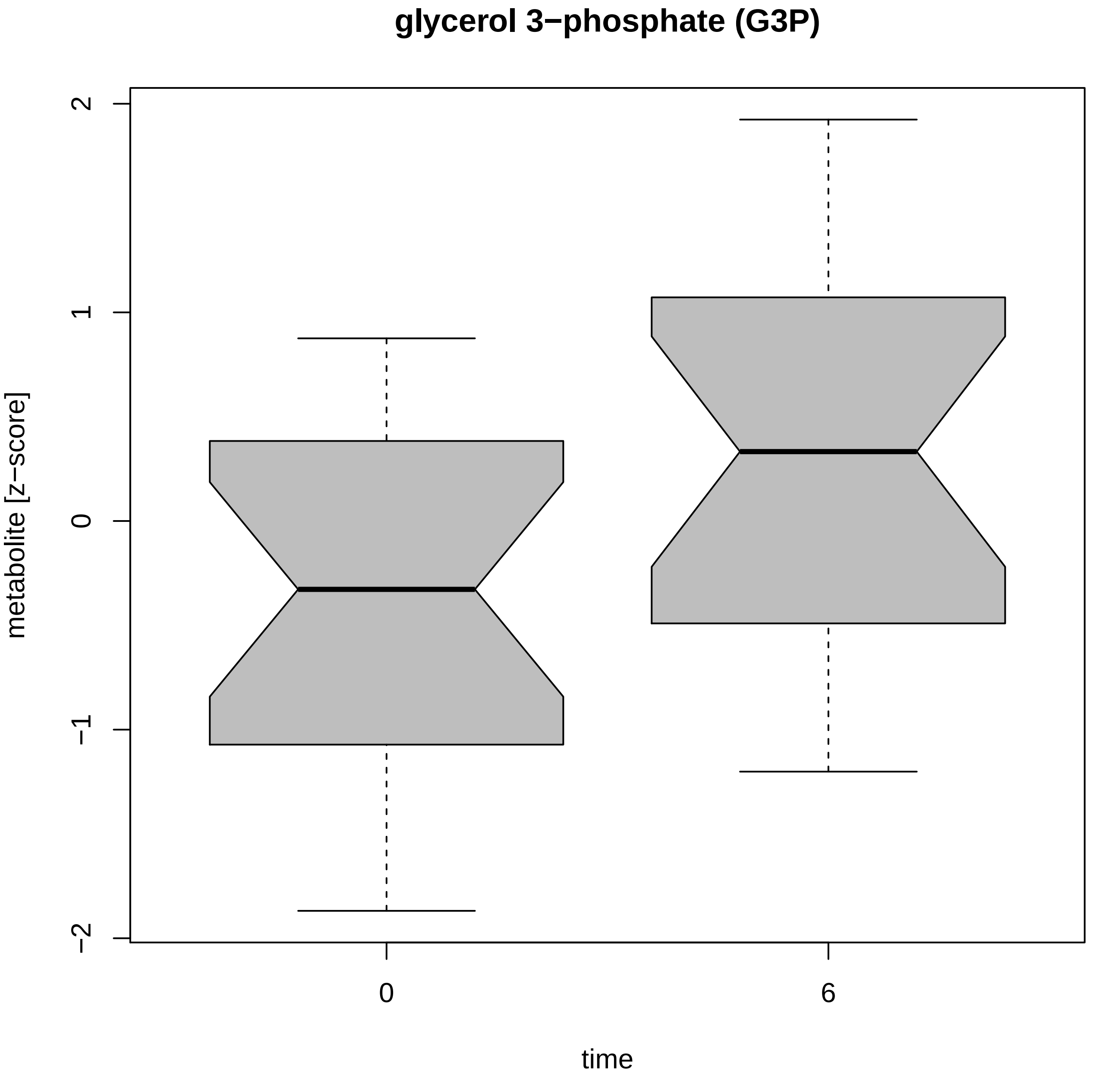

glycerol 3−phosphate (G3P)
metabolite [z−score]
2
1
0
−1
−2
0
6
time

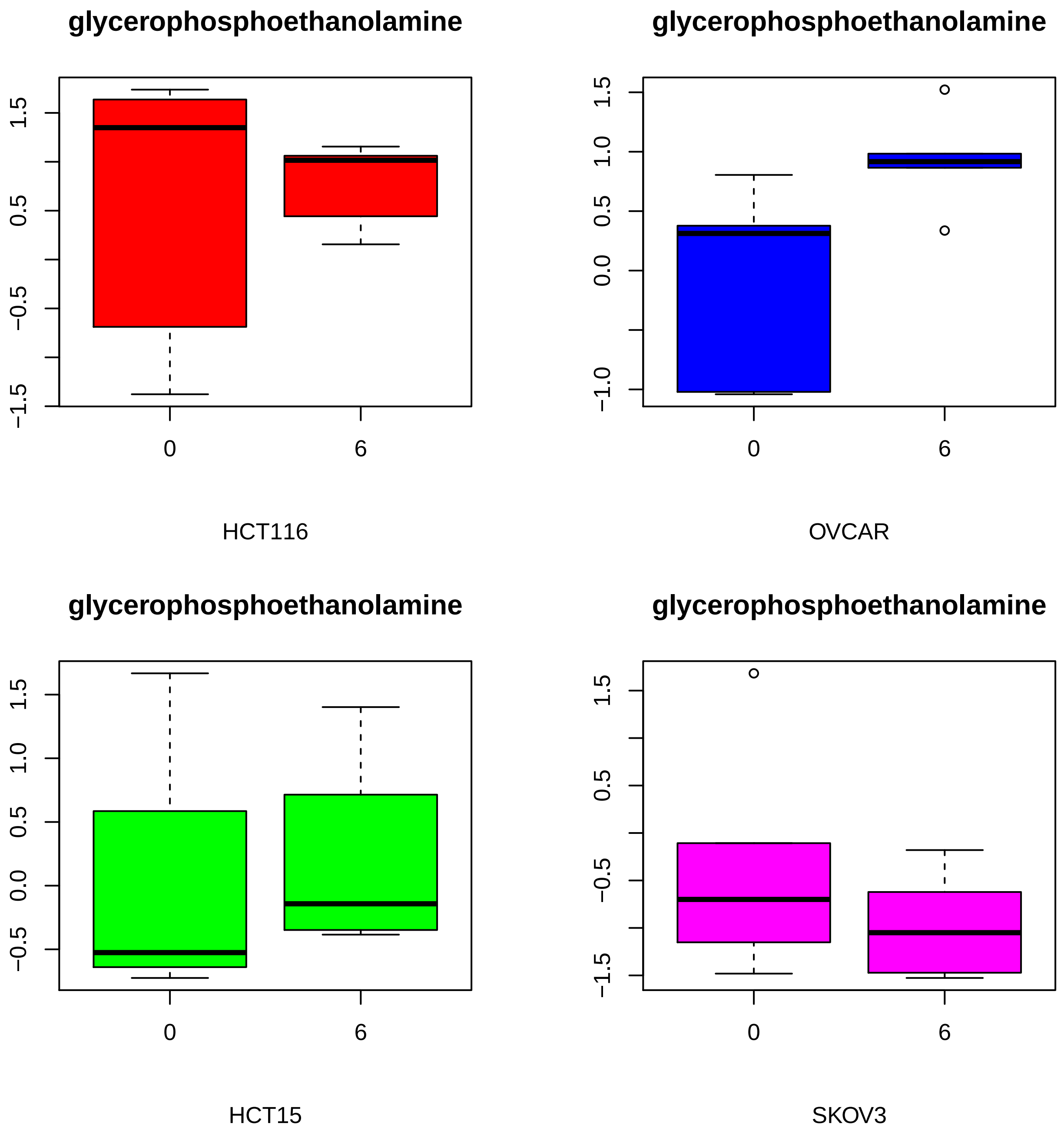
glycerophosphoethanolamine
glycerophosphoethanolamine
glycerophosphoethanolamine
glycerophosphoethanolamine
1.5
0.5
−0.5
−1.5
1.0
0.0
0
6
HCT116
OVCAR
HCT15
SKOV3

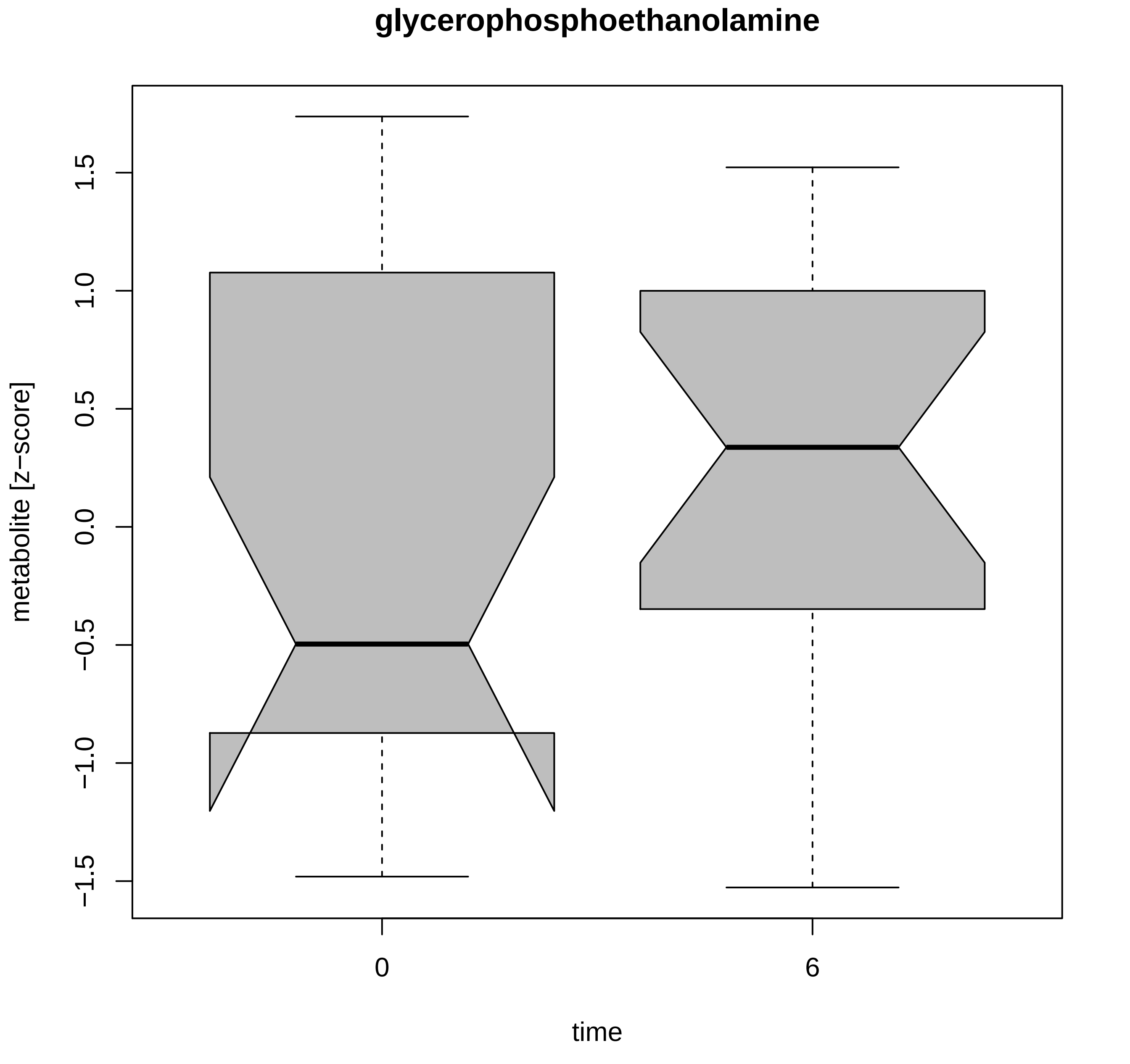

glycerophosphoethanolamine
metabolite [z−score]
1.5
1.0
0.5
0.0
−0.5
−1.0
−1.5
0
6
time

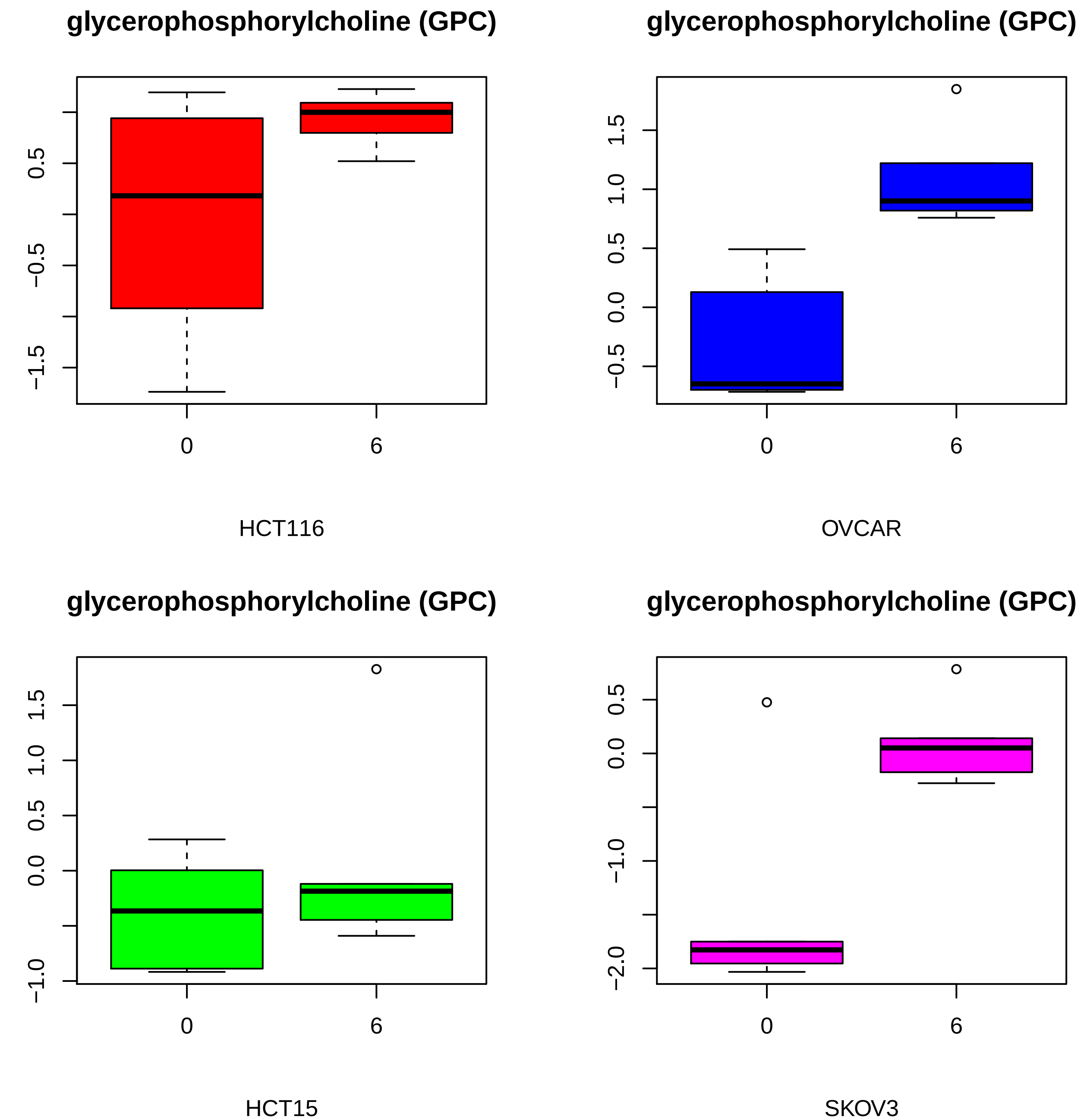

glycerophosphorylcholine (GPC)
0.5
−0.5
−1.5
0
6
HCT116
glycerophosphorylcholine (GPC)
1.5
1.0
0.5
0.0
−0.5
0
6
OVCAR
glycerophosphorylcholine (GPC)
1.5
1.0
0.5
0.0
−1.0
0
6
HCT15
glycerophosphorylcholine (GPC)
0.5
0.0
−1.0
−2.0
0
6
SKOV3

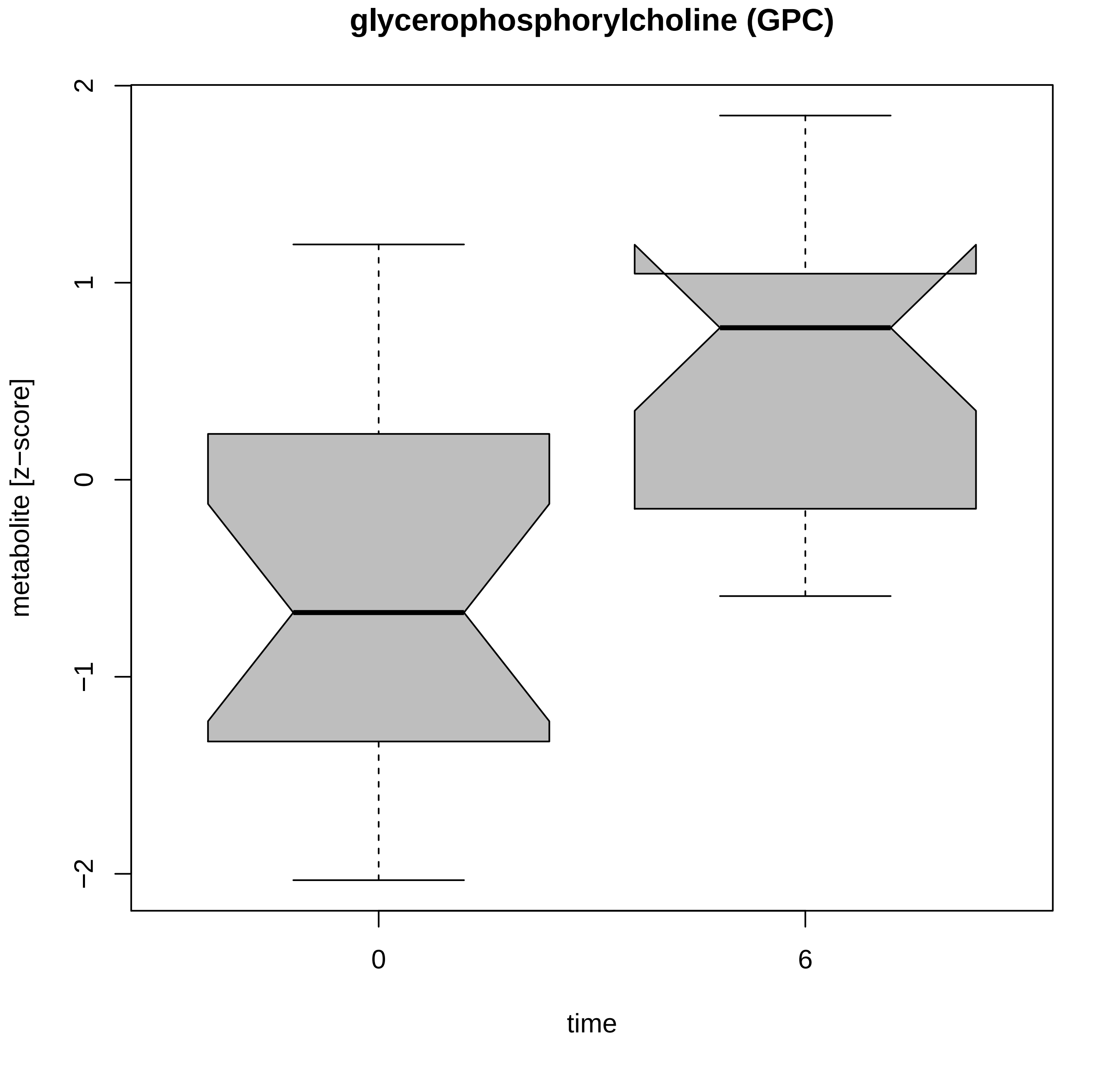
glycerophosphorylcholine (GPC)
metabolite [z−score]
2
1
0
−1
−2
0
6
time

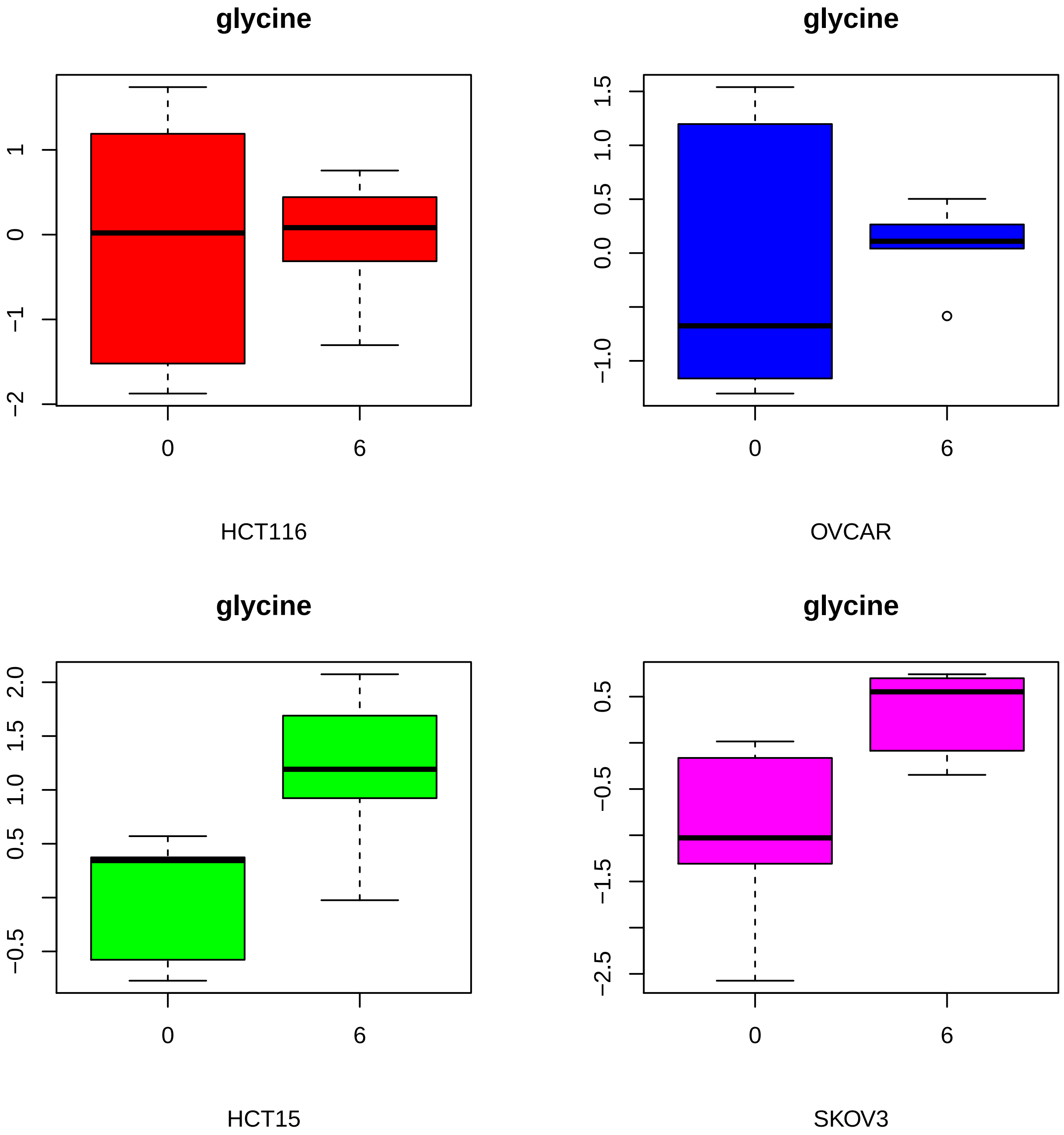
glycine
1
0
−1
−2
0
6
HCT116
glycine
1.5
1.0
0.5
0.0
−1.0
0
6
OVCAR
glycine
2.0
1.5
1.0
0.5
−0.5
0
6
HCT15
glycine
0.5
−0.5
−1.5
−2.5
0
6
SKOV3

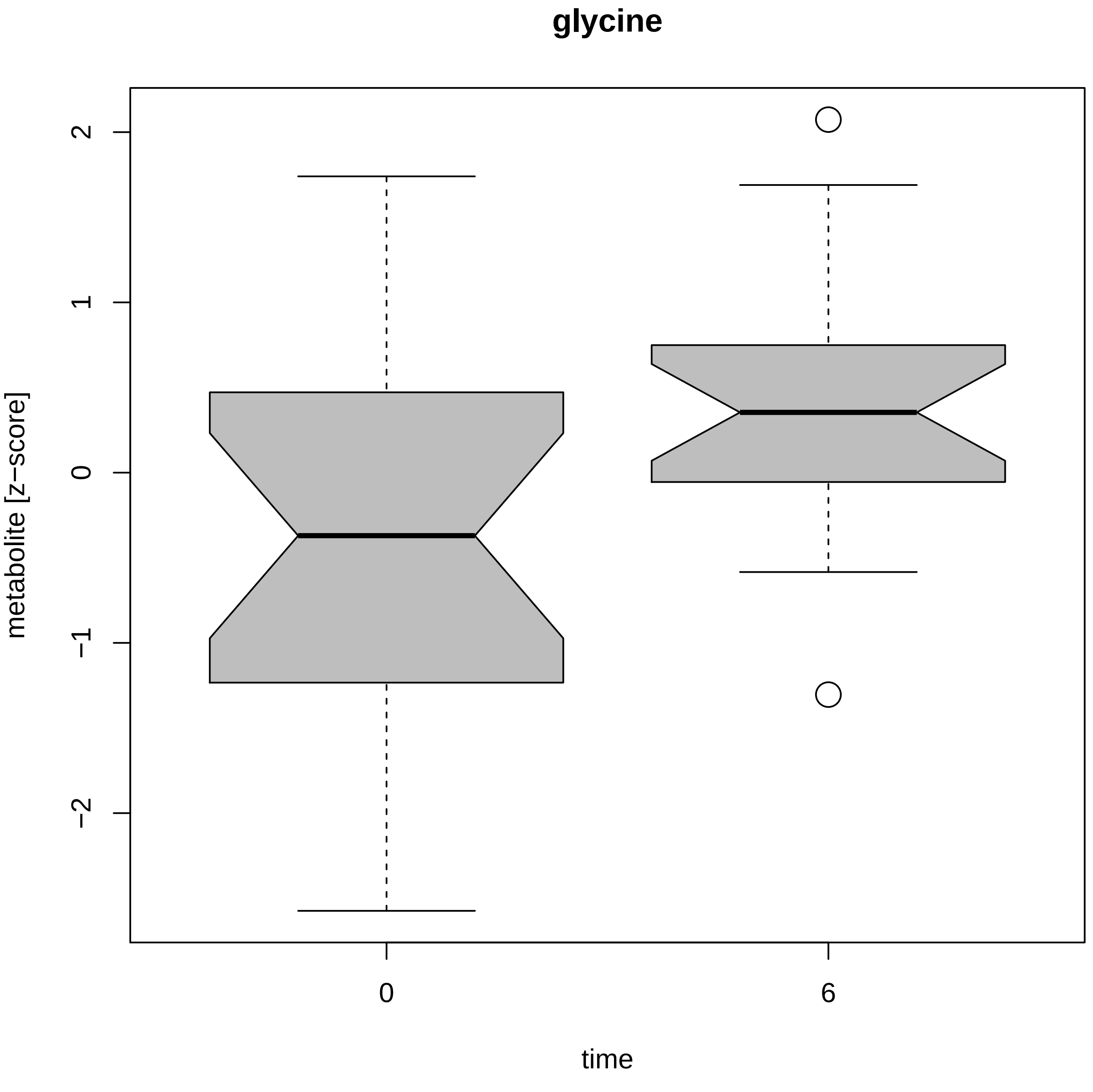
glycine
metabolite [z−score]
2
1
0
−1
−2
0
6
time

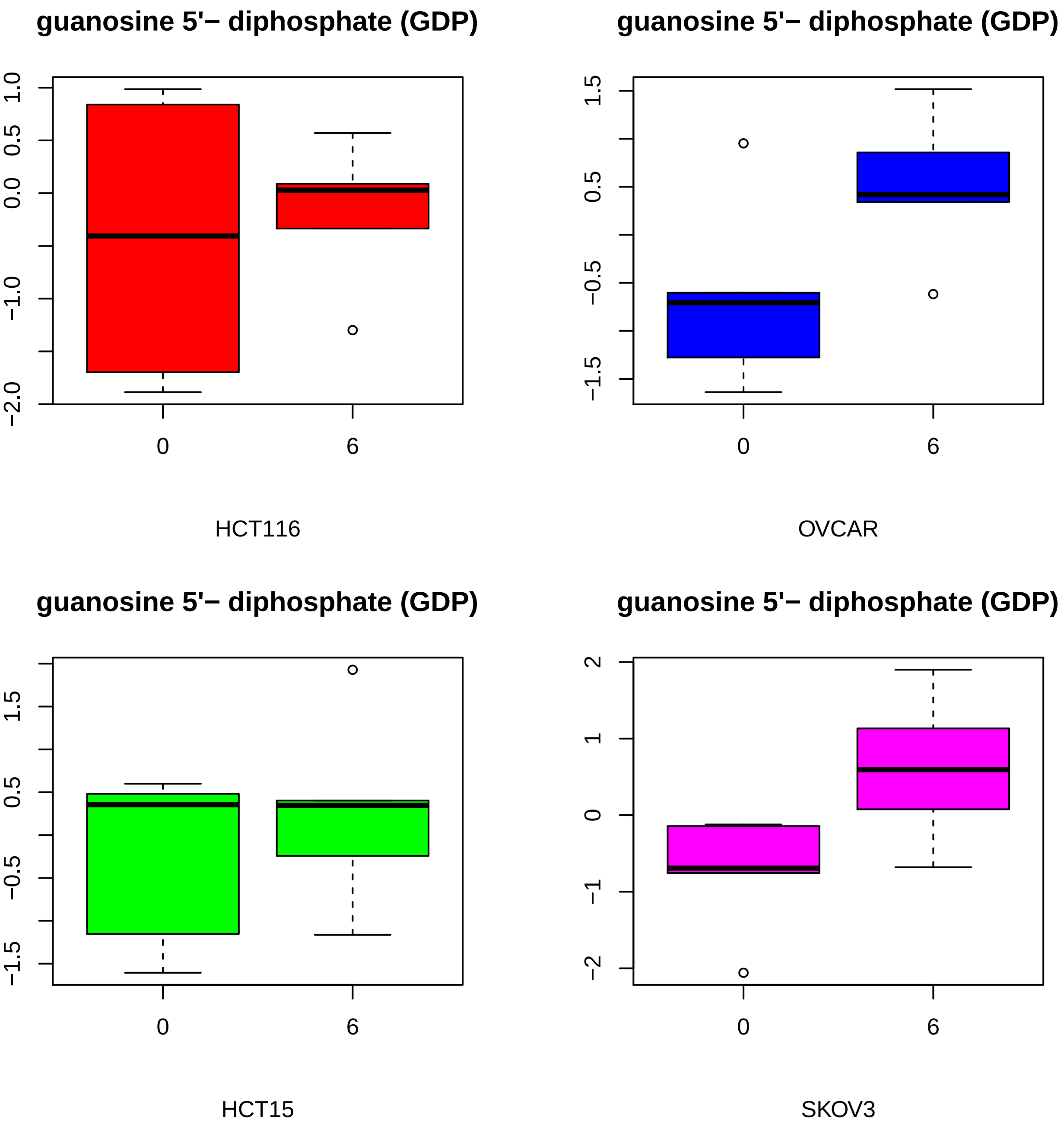
guanosine 5'− diphosphate (GDP)
−2.0
−1.0
0.0
0.5
1.0
0
6
HCT116
guanosine 5'− diphosphate (GDP)
−1.5
−0.5
0.5
1.5
0
6
OVCAR
guanosine 5'− diphosphate (GDP)
−1.5
−0.5
0.5
1.5
0
6
HCT15
guanosine 5'− diphosphate (GDP)
−2
−1
0
1
2
0
6
SKOV3

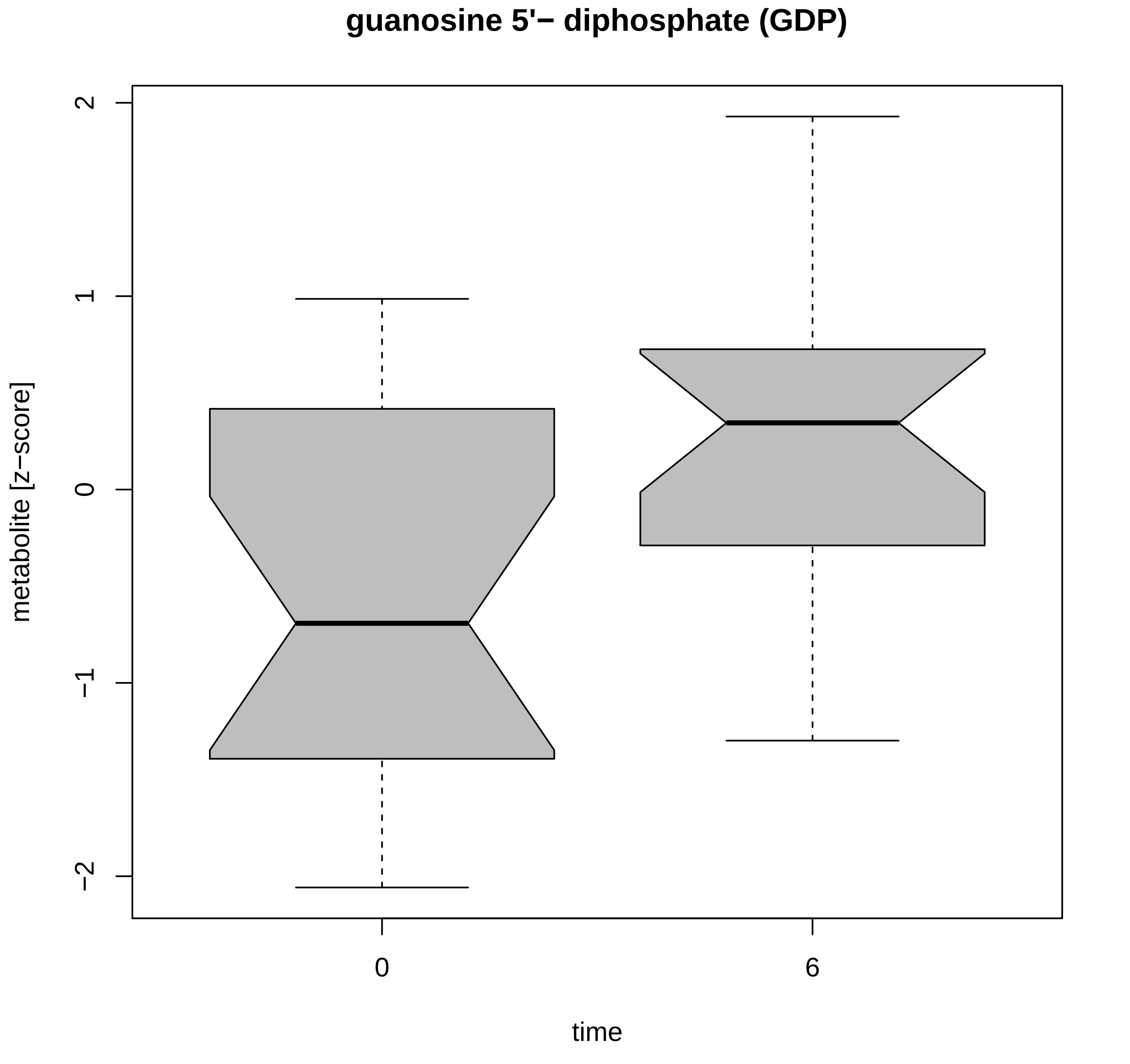

guanosine 5'− diphosphate (GDP)
metabolite [z−score]
time
−2
−1
0
1
2
0
6

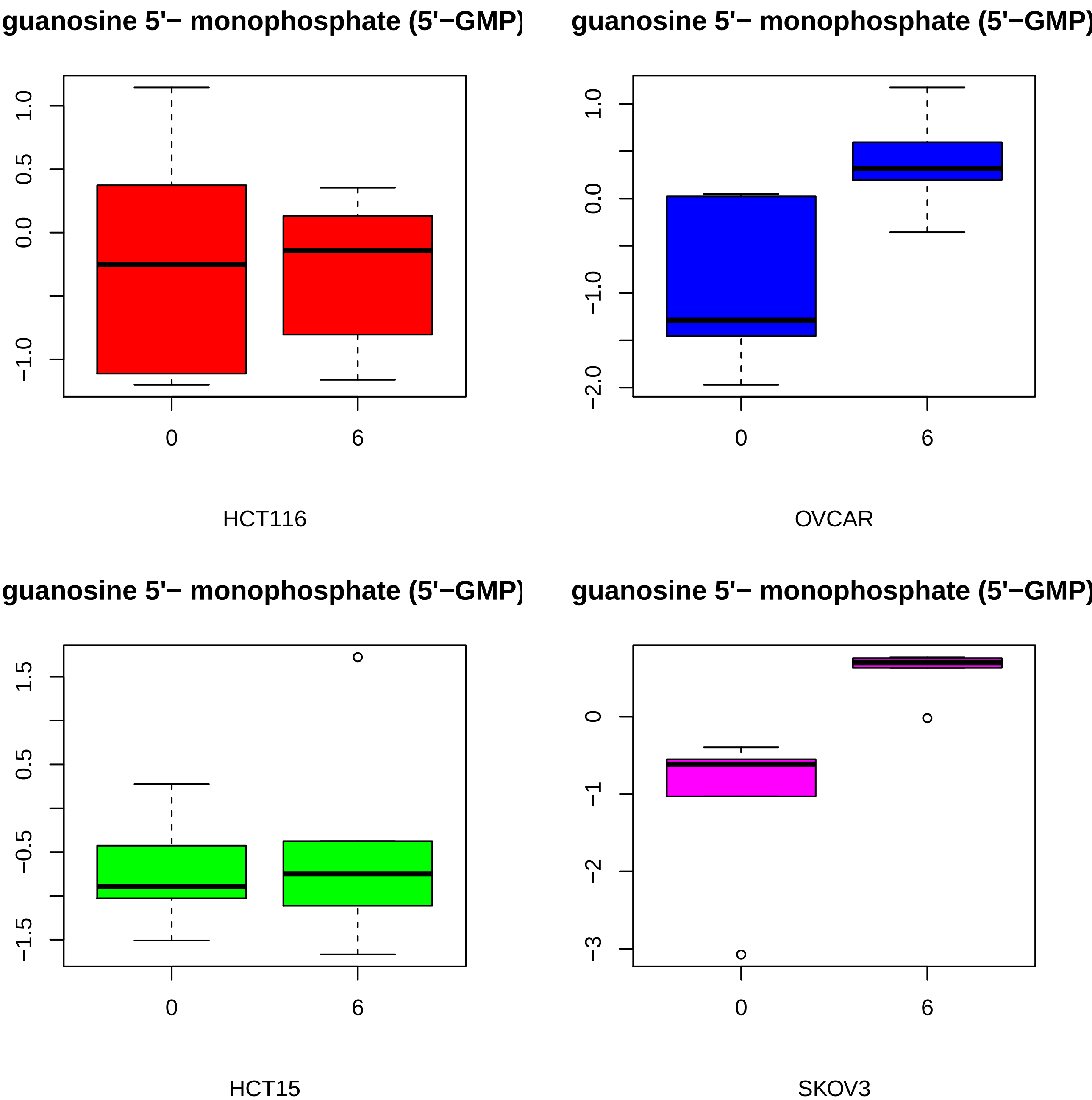

guanosine 5'− monophosphate (5'−GMP)
1.0
0.5
0.0
−1.0
0
6
HCT116
guanosine 5'− monophosphate (5'−GMP)
1.0
0.0
−1.0
−2.0
0
6
OVCAR
guanosine 5'− monophosphate (5'−GMP)
1.5
0.5
−0.5
−1.5
0
6
HCT15
guanosine 5'− monophosphate (5'−GMP)
0
−1
−2
−3
0
6
SKOV3

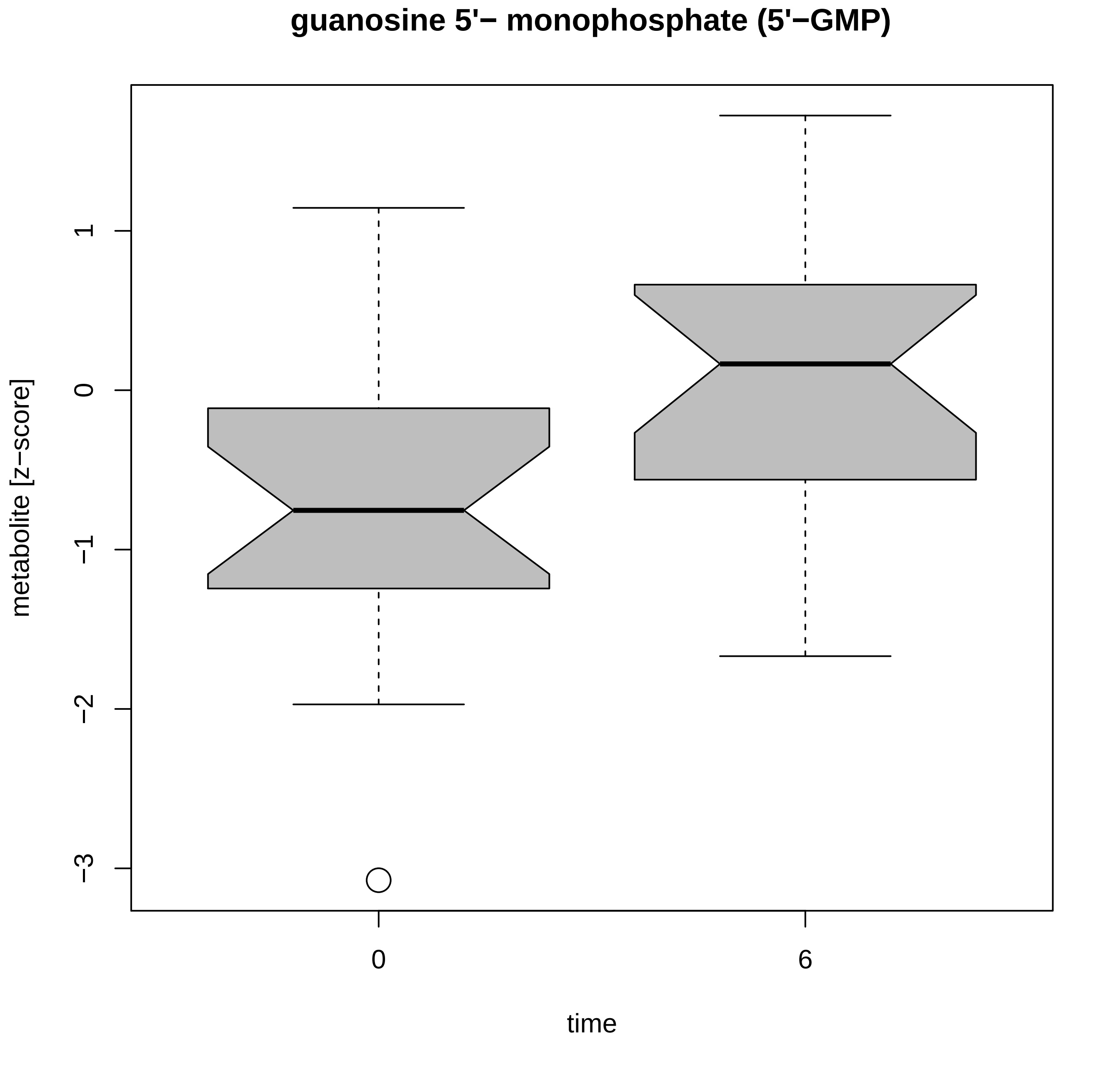
guanosine 5'− monophosphate (5'−GMP)
metabolite [z−score]
1
0
−1
−2
−3
0
6
time

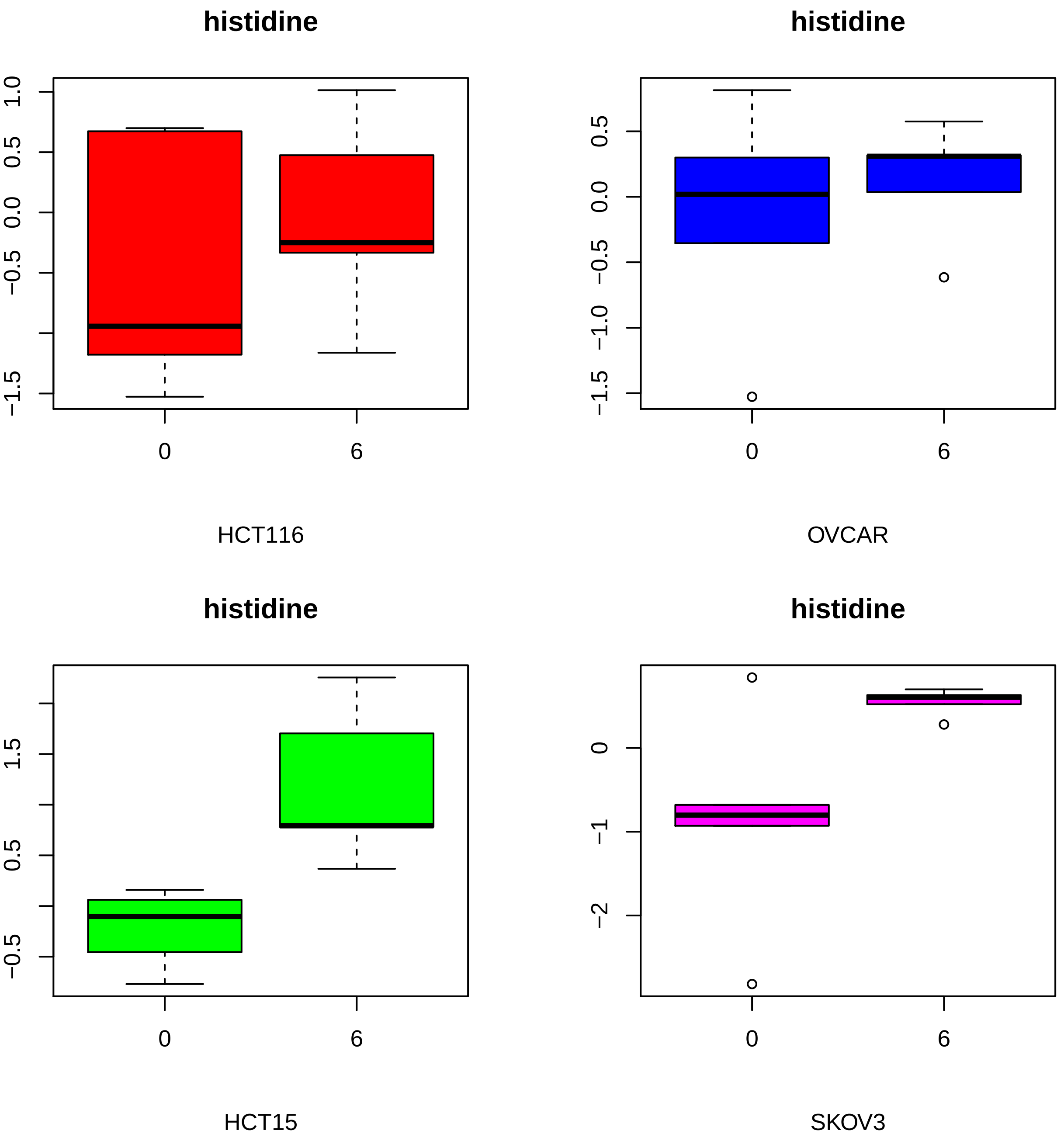
histidine
1.0
0.5
0.0
−0.5
−1.5
0
6
HCT116
histidine
0.5
0.0
−0.5
−1.0
−1.5
0
6
OVCAR
histidine
1.5
0.5
−0.5
0
6
HCT15
histidine
0
−1
−2
0
6
SKOV3

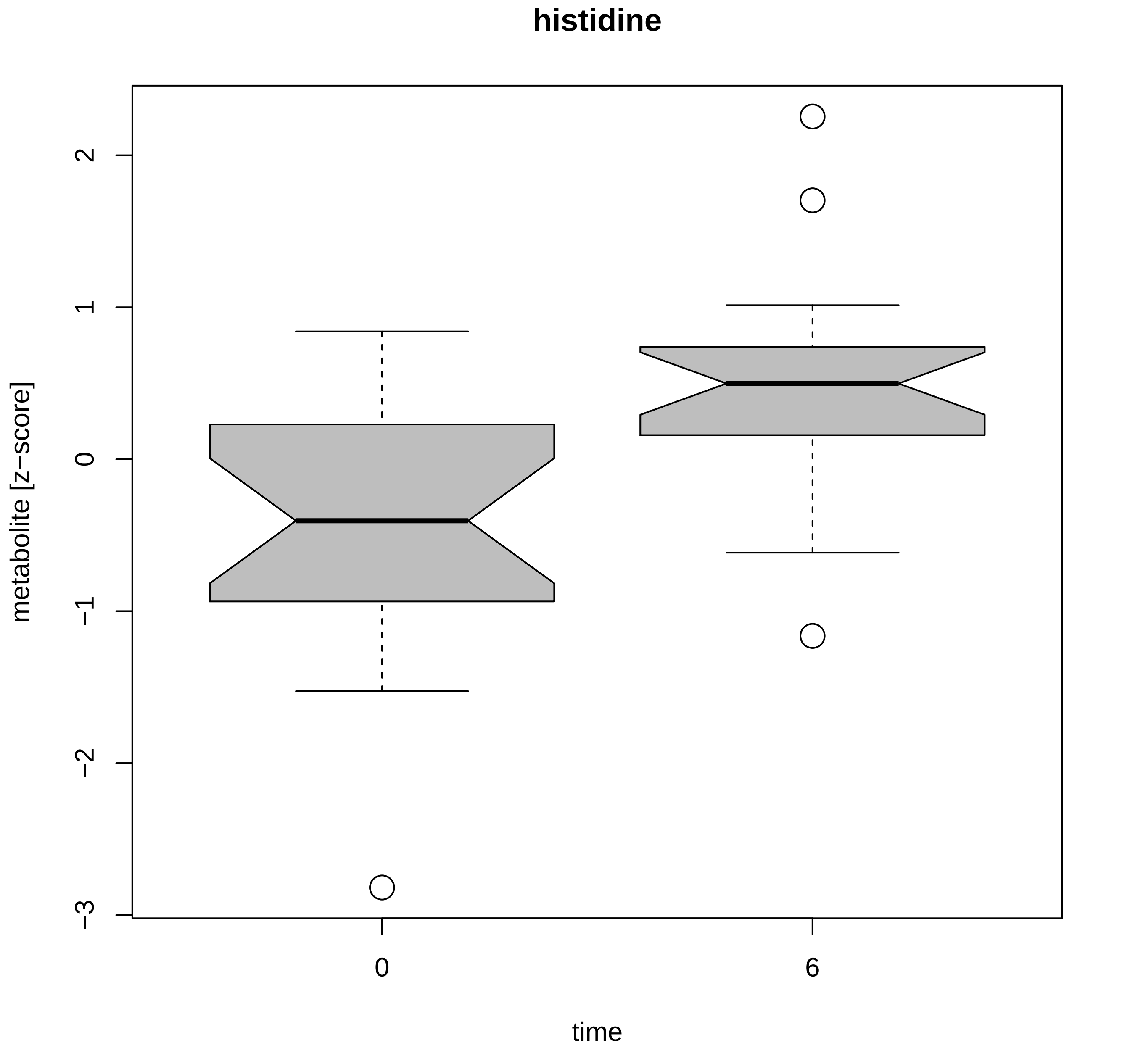
histidine
metabolite [z−score]
2
1
0
−1
−2
−3
0
6
time

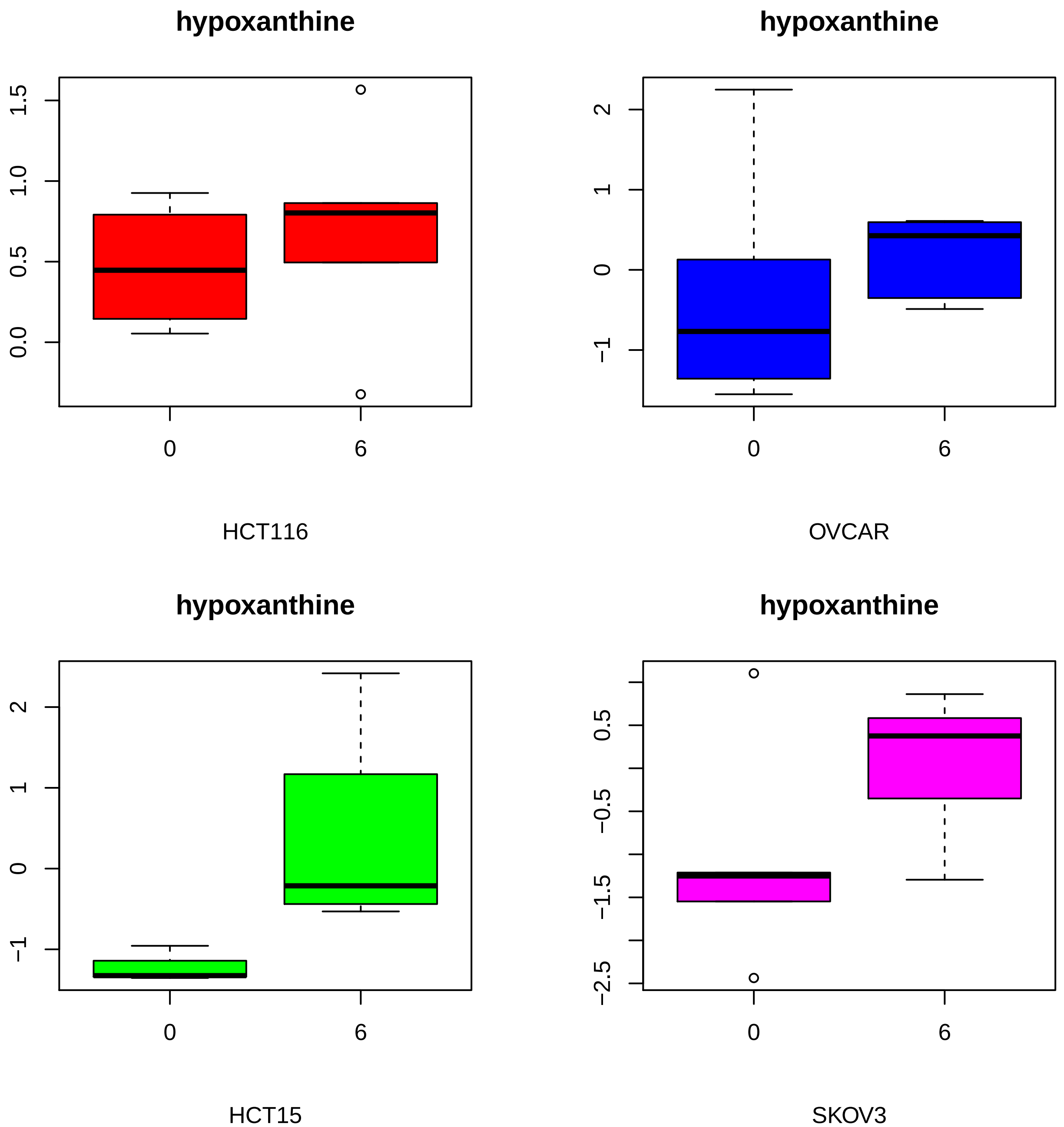
hypoxanthine
1.5
1.0
0.5
0.0
0
6
HCT116
hypoxanthine
2
1
0
−1
0
6
OVCAR
hypoxanthine
2
1
0
−1
0
6
HCT15
hypoxanthine
0.5
−0.5
−1.5
−2.5
0
6
SKOV3

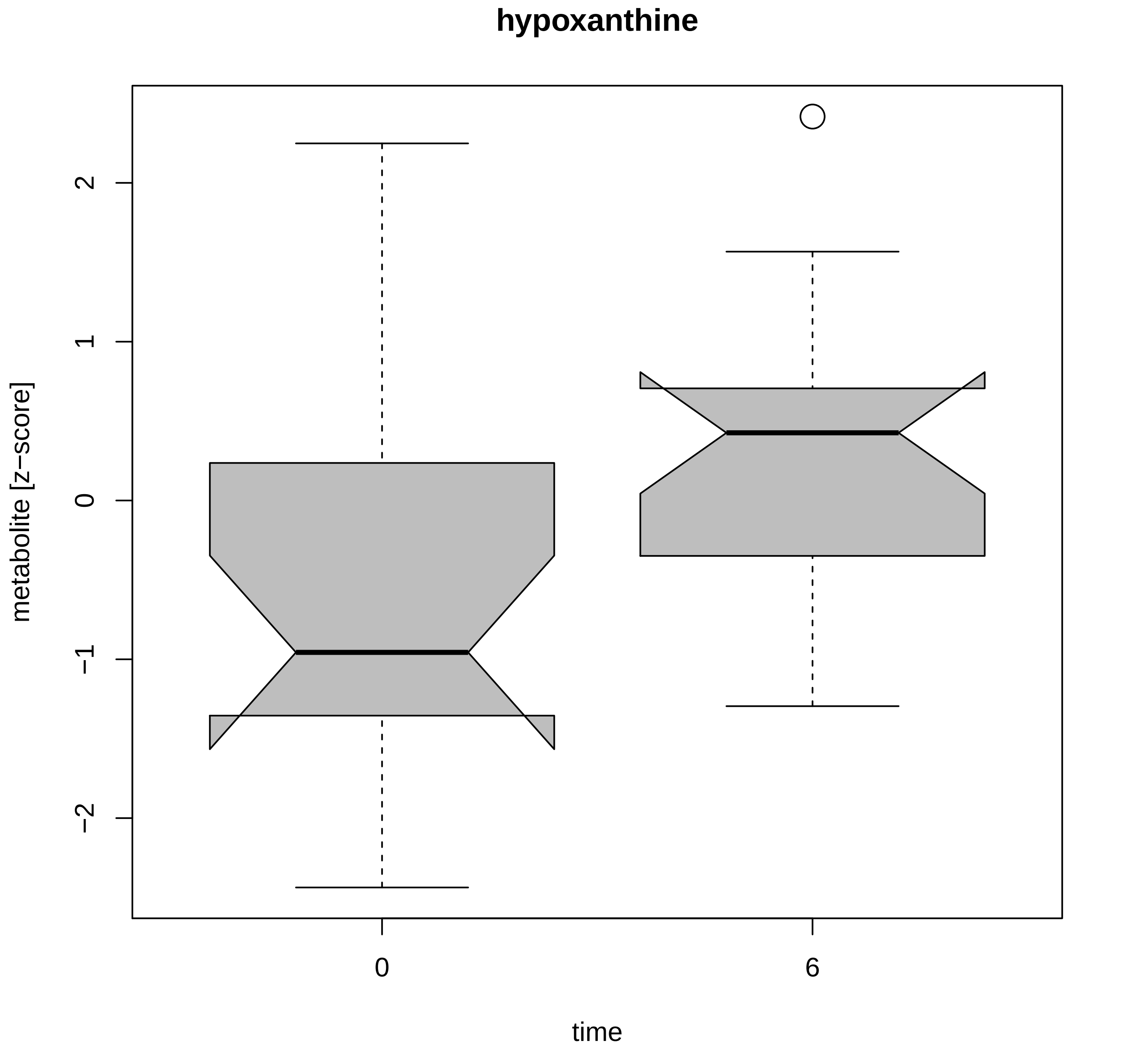

hypoxanthine
metabolite [z−score]
2
1
0
−1
−2
0
6
time

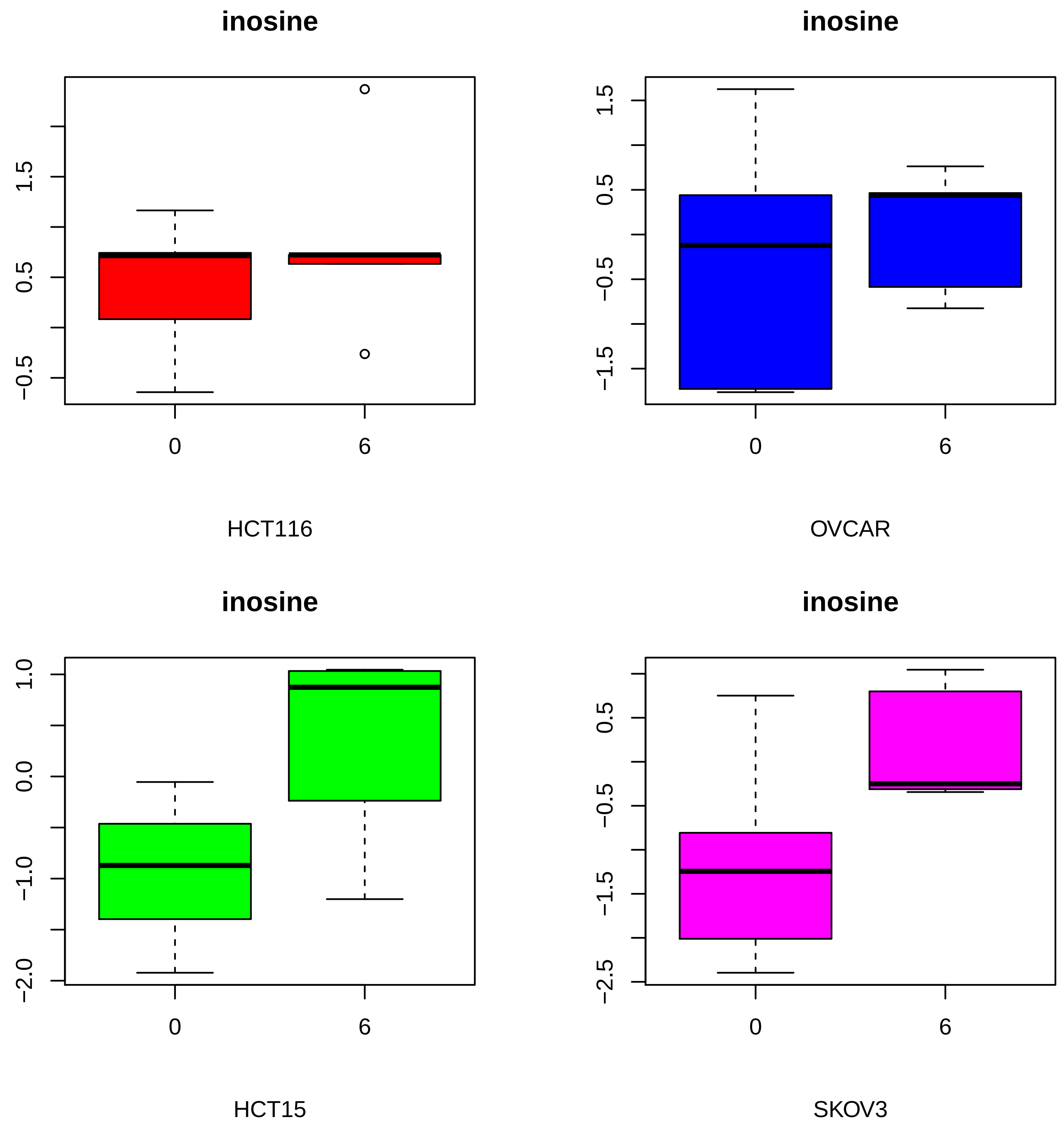
inosine
1.5
0.5
−0.5
0
6
HCT116
inosine
1.5
0.5
−0.5
−1.5
0
6
OVCAR
inosine
1.0
0.0
−1.0
−2.0
0
6
HCT15
inosine
0.5
−0.5
−1.5
−2.5
0
6
SKOV3

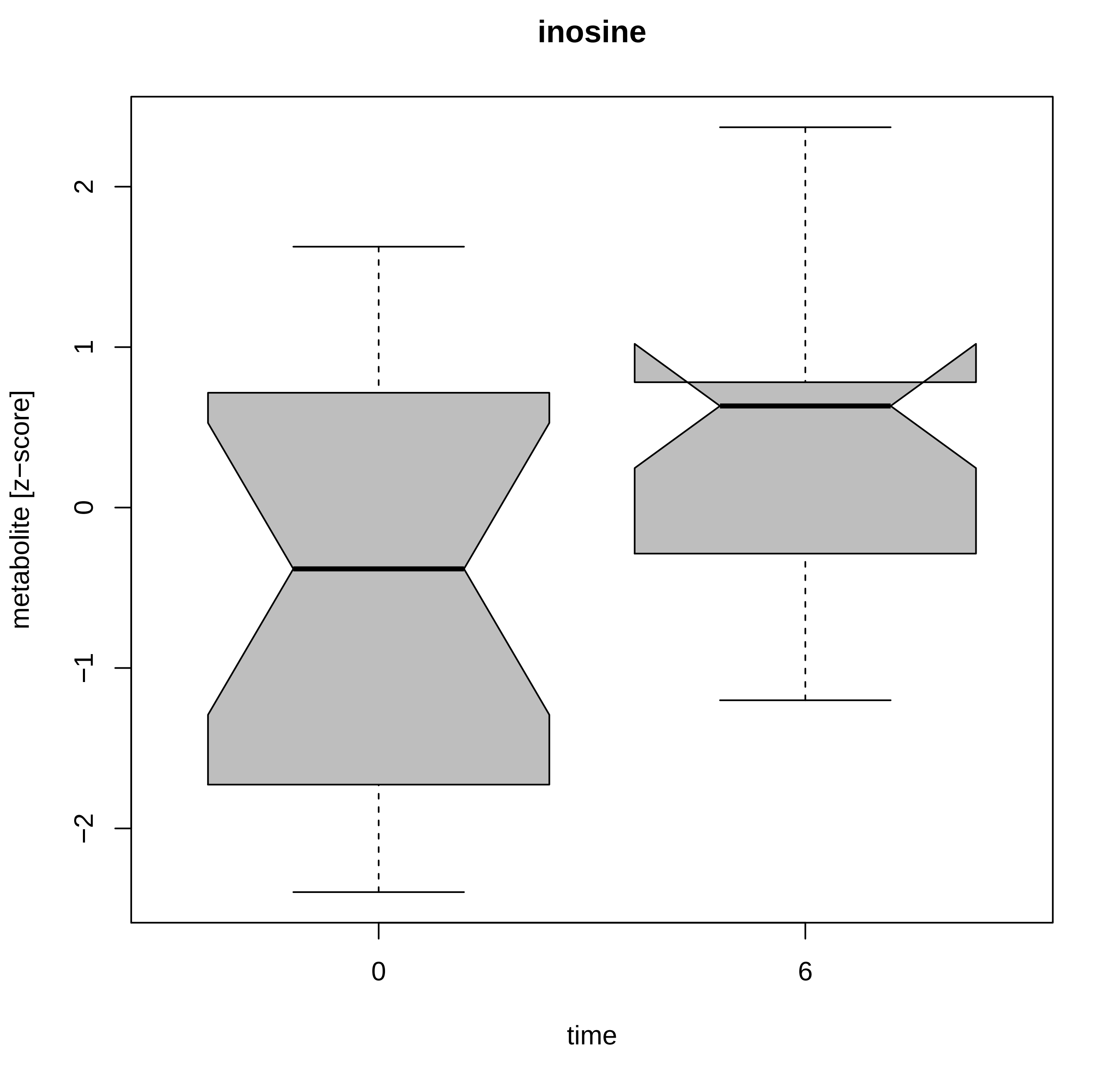
inosine
metabolite [z−score]
2
1
0
−1
−2
0
6
time

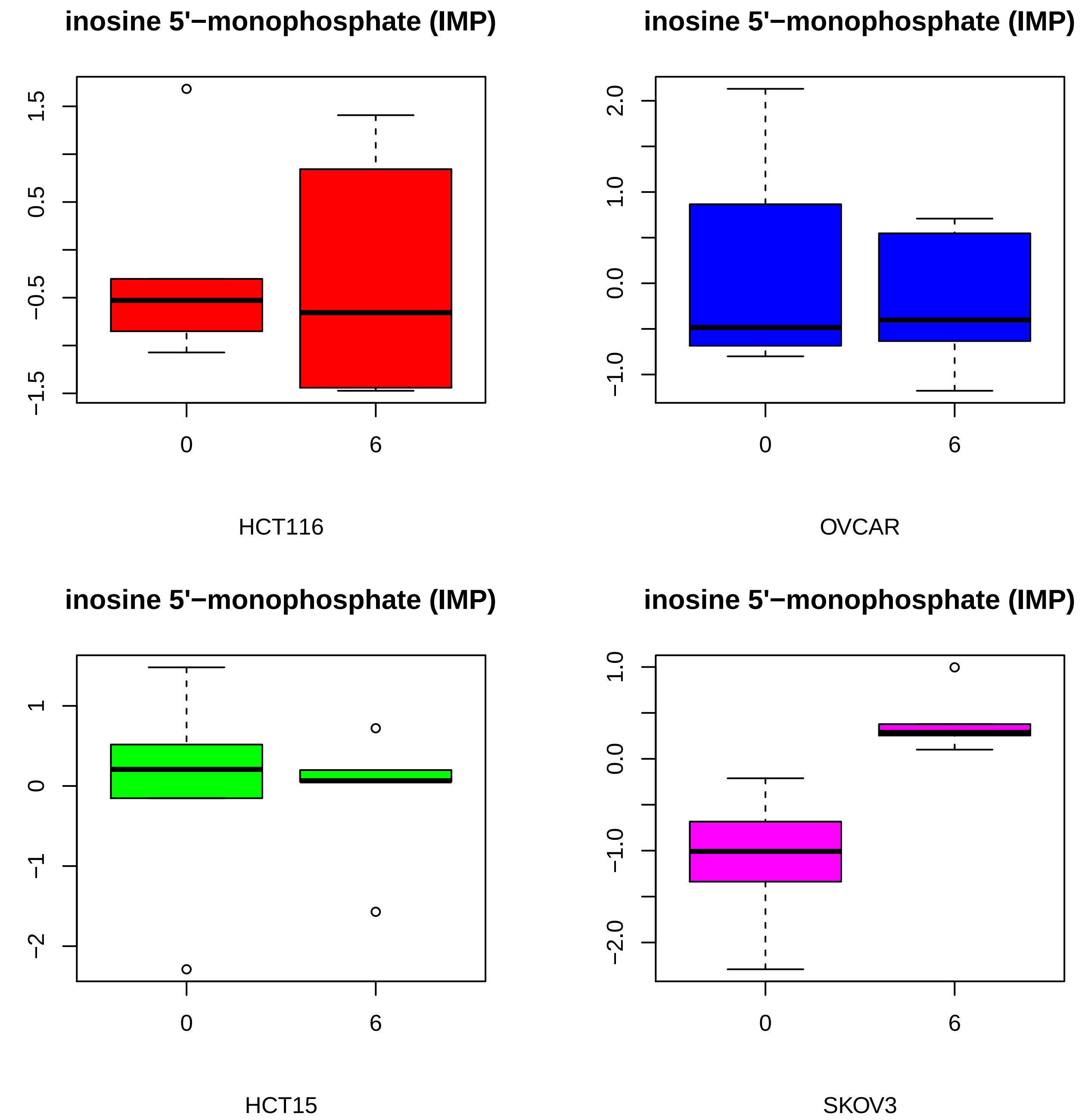
inosine 5'−monophosphate (IMP)
1.5
0.5
−0.5
−1.5
0
6
HCT116
inosine 5'−monophosphate (IMP)
2.0
1.0
0.0
−1.0
0
6
OVCAR
inosine 5'−monophosphate (IMP)
1
0
−1
−2
0
6
HCT15
inosine 5'−monophosphate (IMP)
1.0
0.0
−1.0
−2.0
0
6
SKOV3

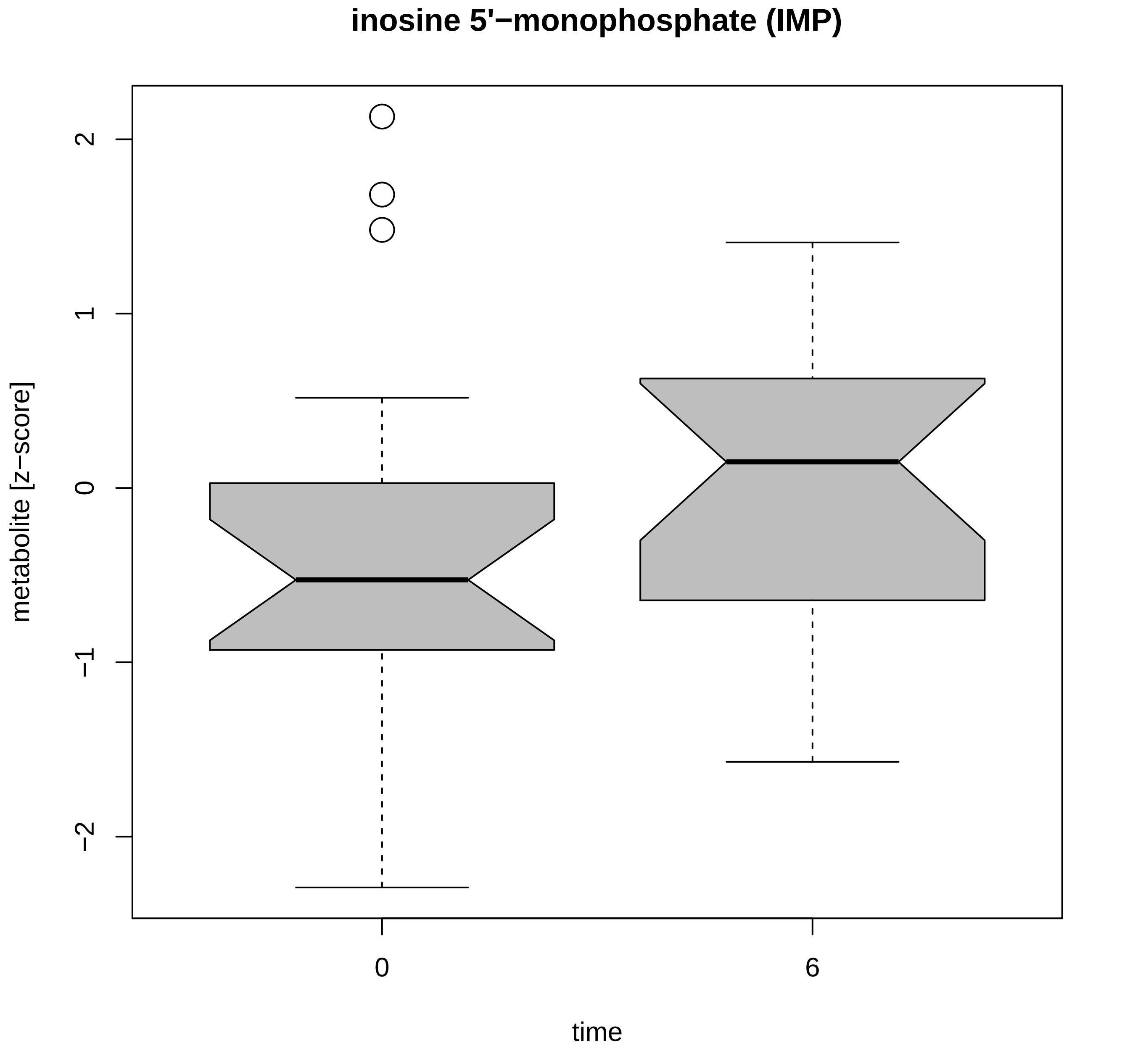

inosine 5'−monophosphate (IMP)
metabolite [z−score]
2
1
0
−1
−2
0
6
time

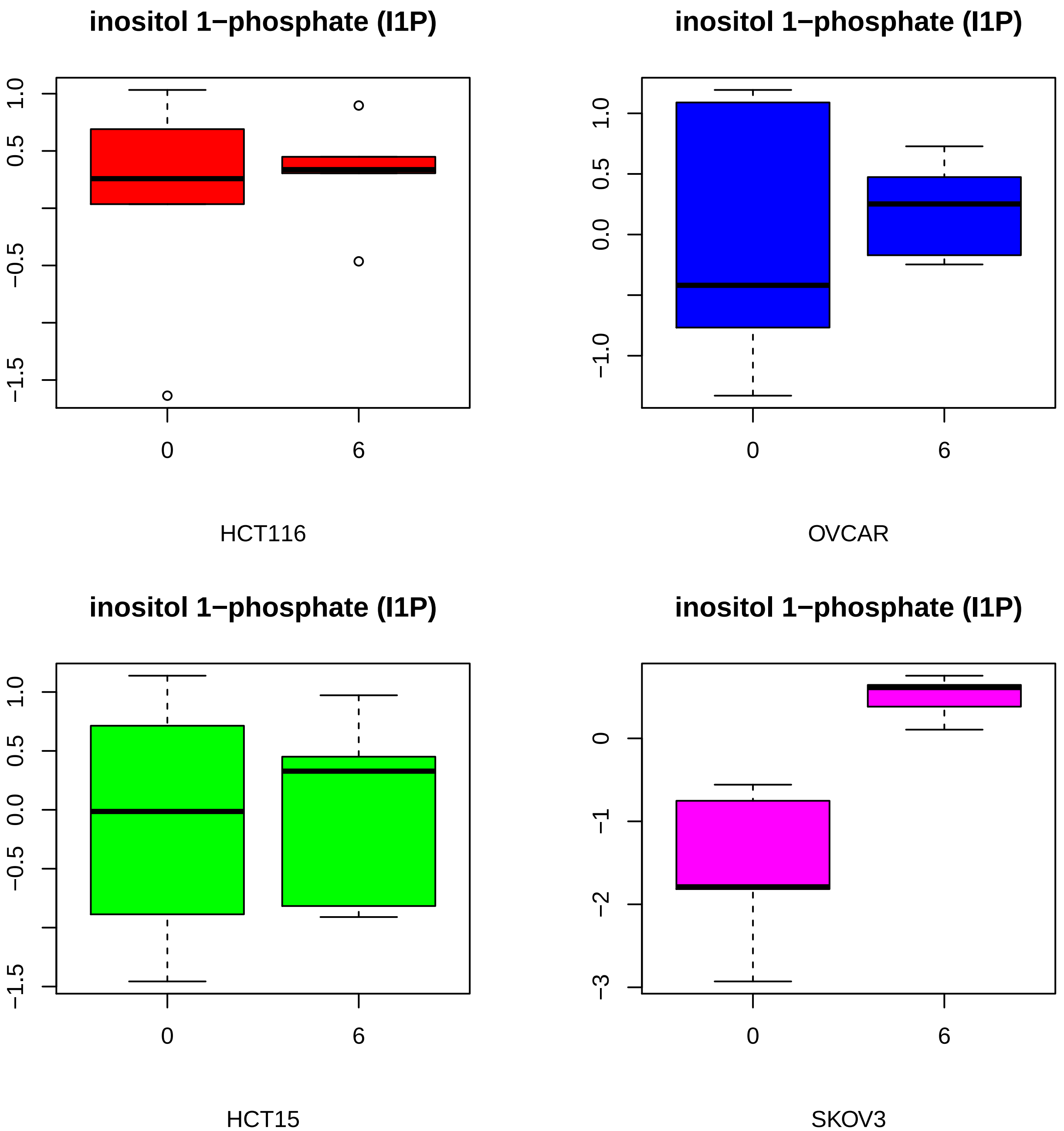
inositol 1−phosphate (I1P)
1.0
0.5
−0.5
−1.5
0
6
HCT116
inositol 1−phosphate (I1P)
1.0
0.5
0.0
−1.0
0
6
OVCAR
inositol 1−phosphate (I1P)
1.0
0.5
0.0
−0.5
−1.5
0
6
HCT15
inositol 1−phosphate (I1P)
0
−1
−2
−3
0
6
SKOV3

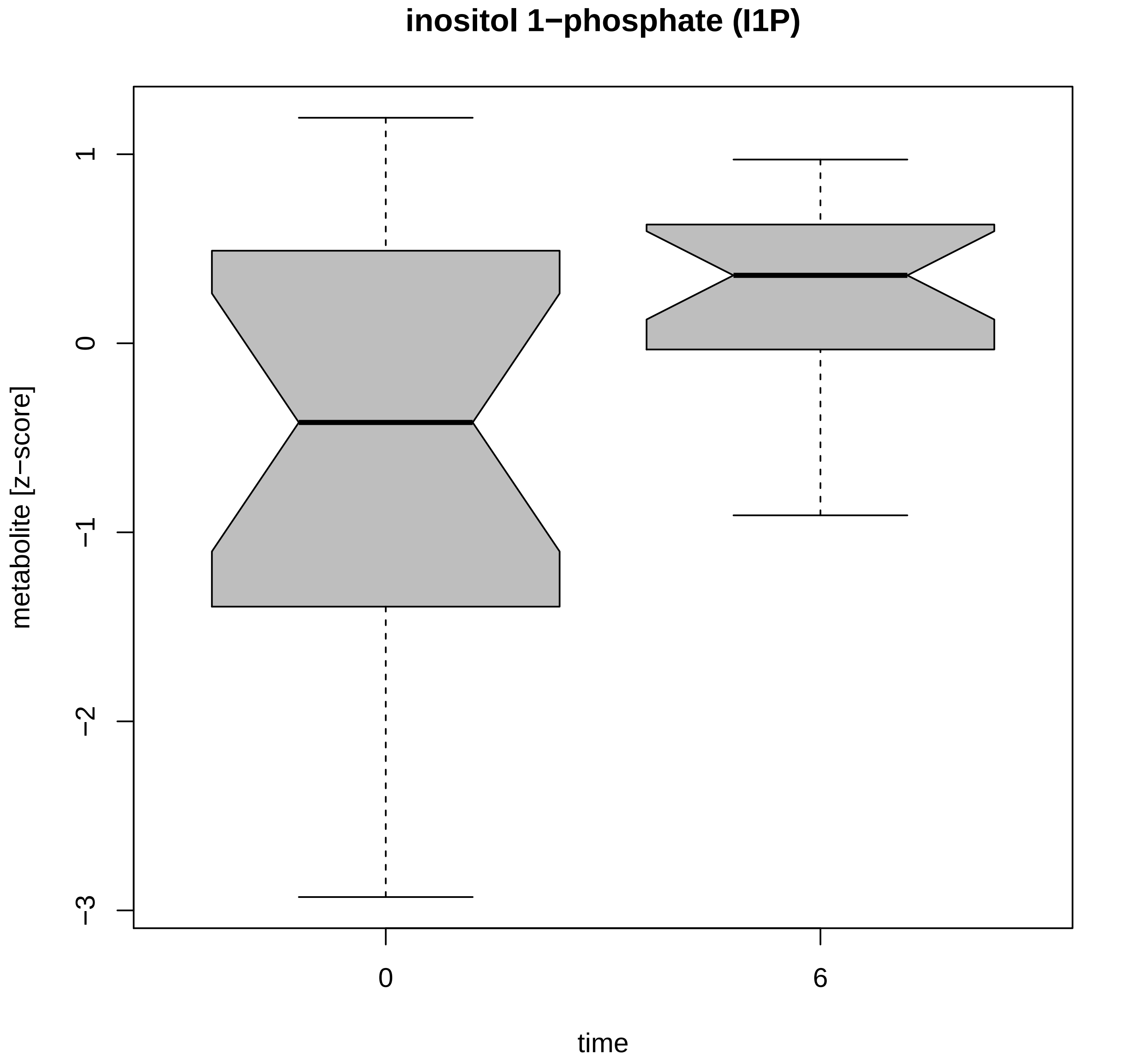
inositol 1−phosphate (I1P)
metabolite [z−score]
1
0
−1
−2
−3
0
6
time

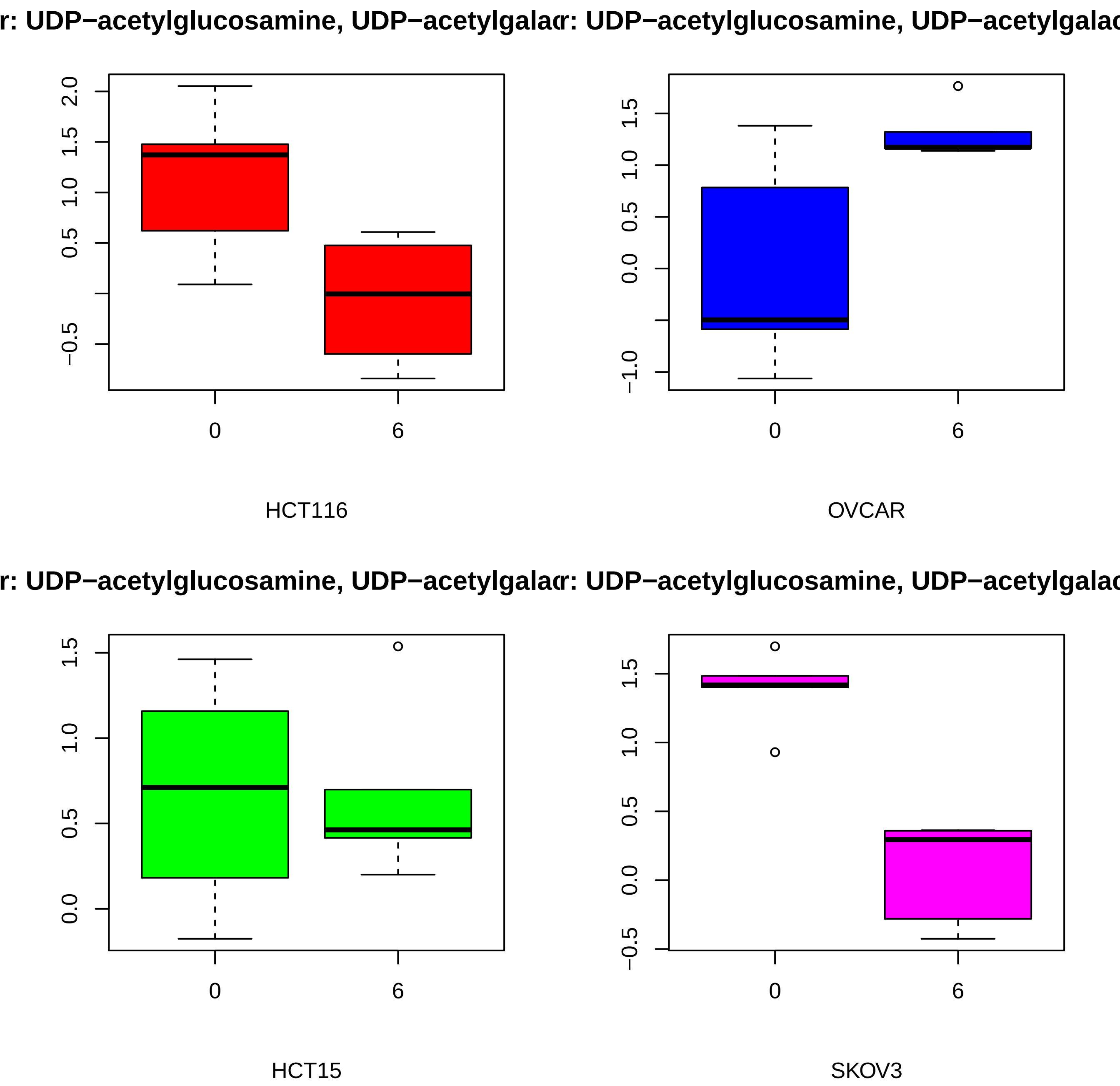
r: UDP−acetylglucosamine, UDP−acetylgalac
r: UDP−acetylglucosamine, UDP−acetylgalac
r: UDP−acetylglucosamine, UDP−acetylgalac
r: UDP−acetylglucosamine, UDP−acetylgalac
−0.5
0.5
1.0
1.5
2.0
0
6
HCT116
−1.0
0.0
0.5
1.0
1.5
0
6
OVCAR
0.0
0.5
1.0
1.5
0
6
HCT15
−0.5
0.0
0.5
1.0
1.5
0
6
SKOV3

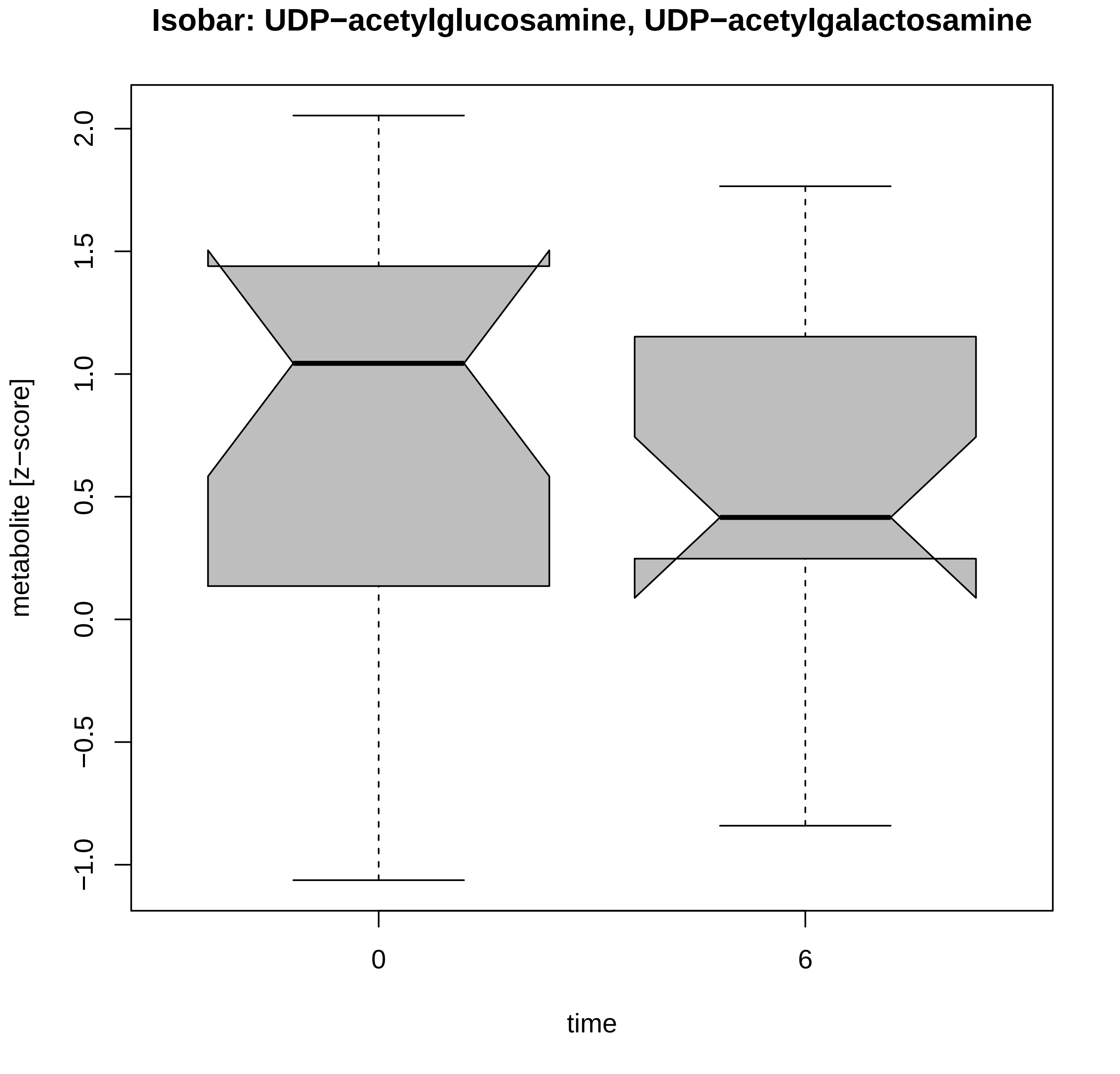
Isobar: UDP−acetylglucosamine, UDP−acetylgalactosamine
metabolite [z−score]
2.0
1.5
1.0
0.5
0.0
−0.5
−1.0
0
6
time

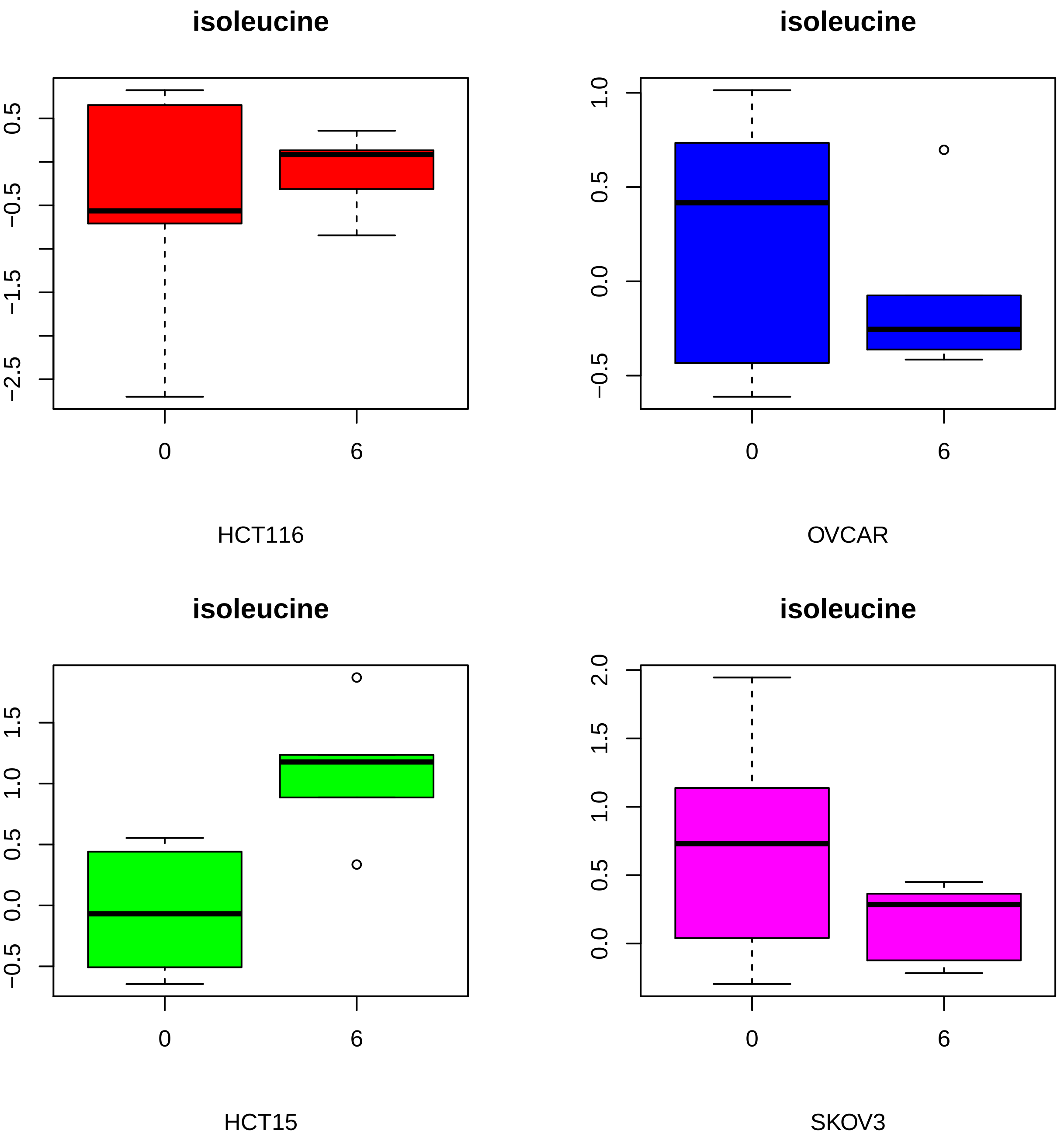
isoleucine
0.5
−0.5
−1.5
−2.5
0
6
HCT116
isoleucine
1.0
0.5
0.0
−0.5
0
6
OVCAR
isoleucine
1.5
1.0
0.5
0.0
−0.5
0
6
HCT15
isoleucine
2.0
1.5
1.0
0.5
0.0
0
6
SKOV3

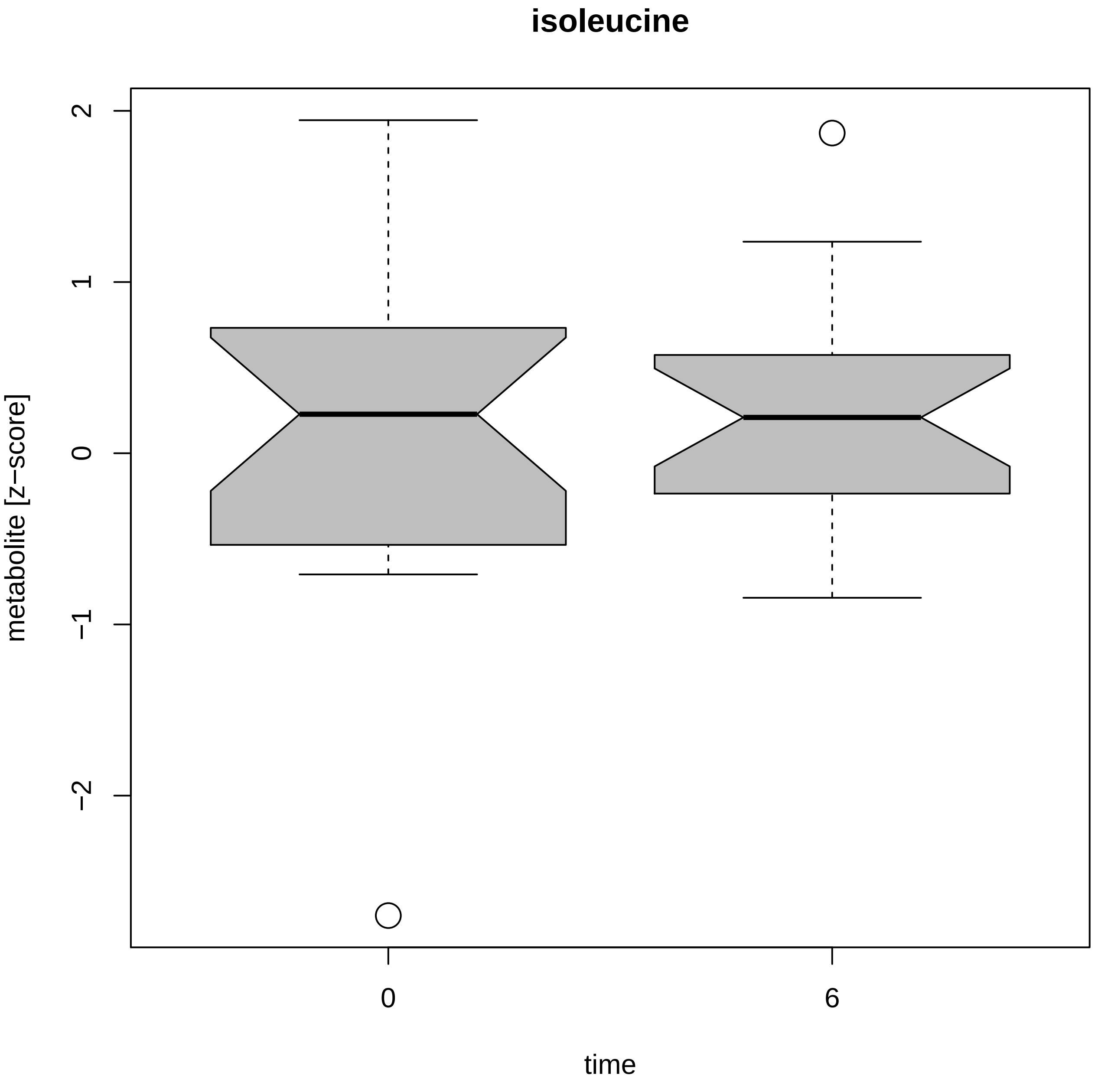
isoleucine
metabolite [z−score]
2
1
0
−1
−2
0
6
time

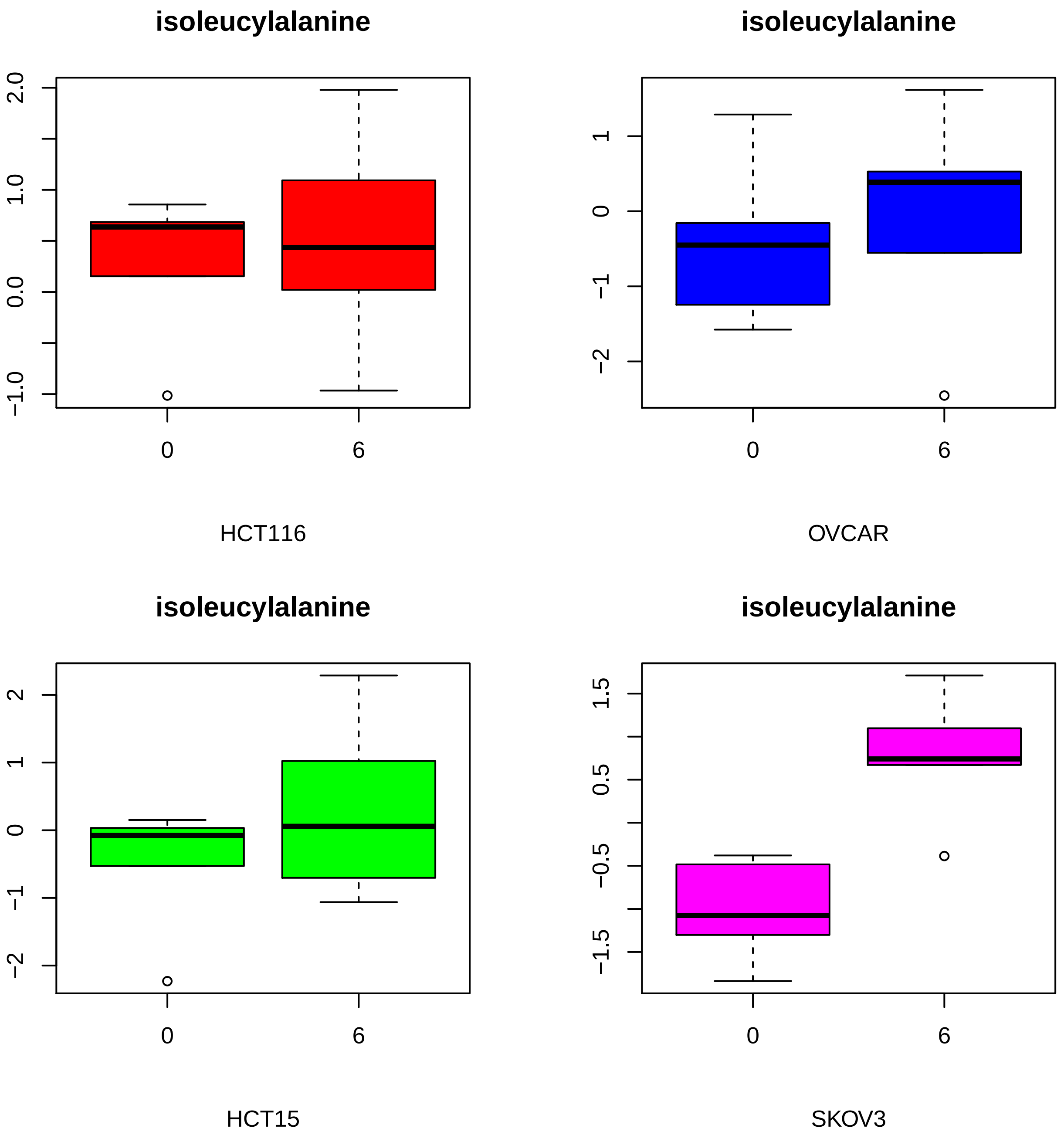
isoleucylalanine
2.0
1.0
0.0
−1.0
0
6
HCT116
isoleucylalanine
1
0
−1
−2
0
6
OVCAR
isoleucylalanine
2
1
0
−1
−2
0
6
HCT15
isoleucylalanine
1.5
0.5
−0.5
−1.5
0
6
SKOV3

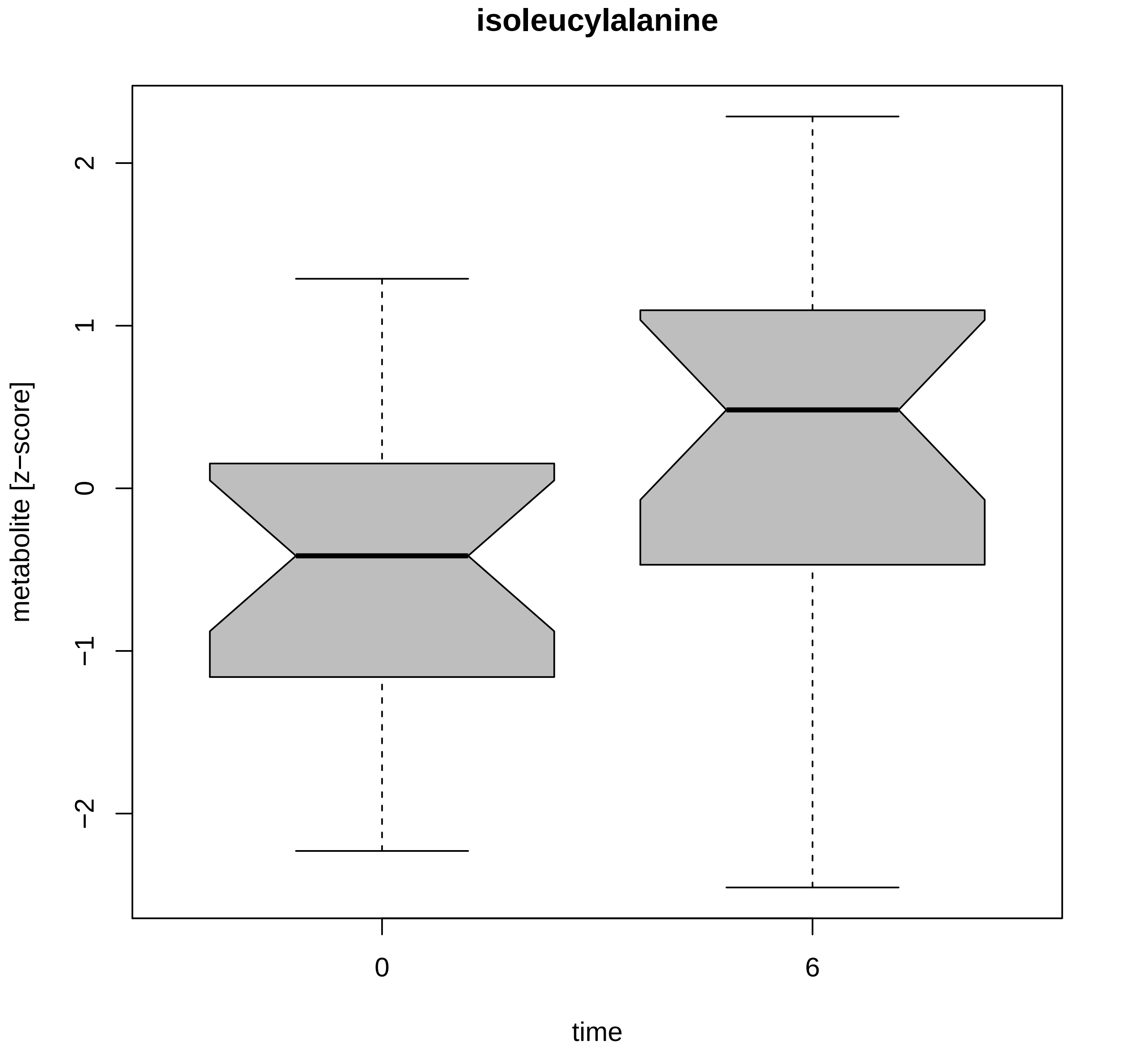
isoleucylalanine
metabolite [z−score]
2
1
0
−1
−2
0
6
time

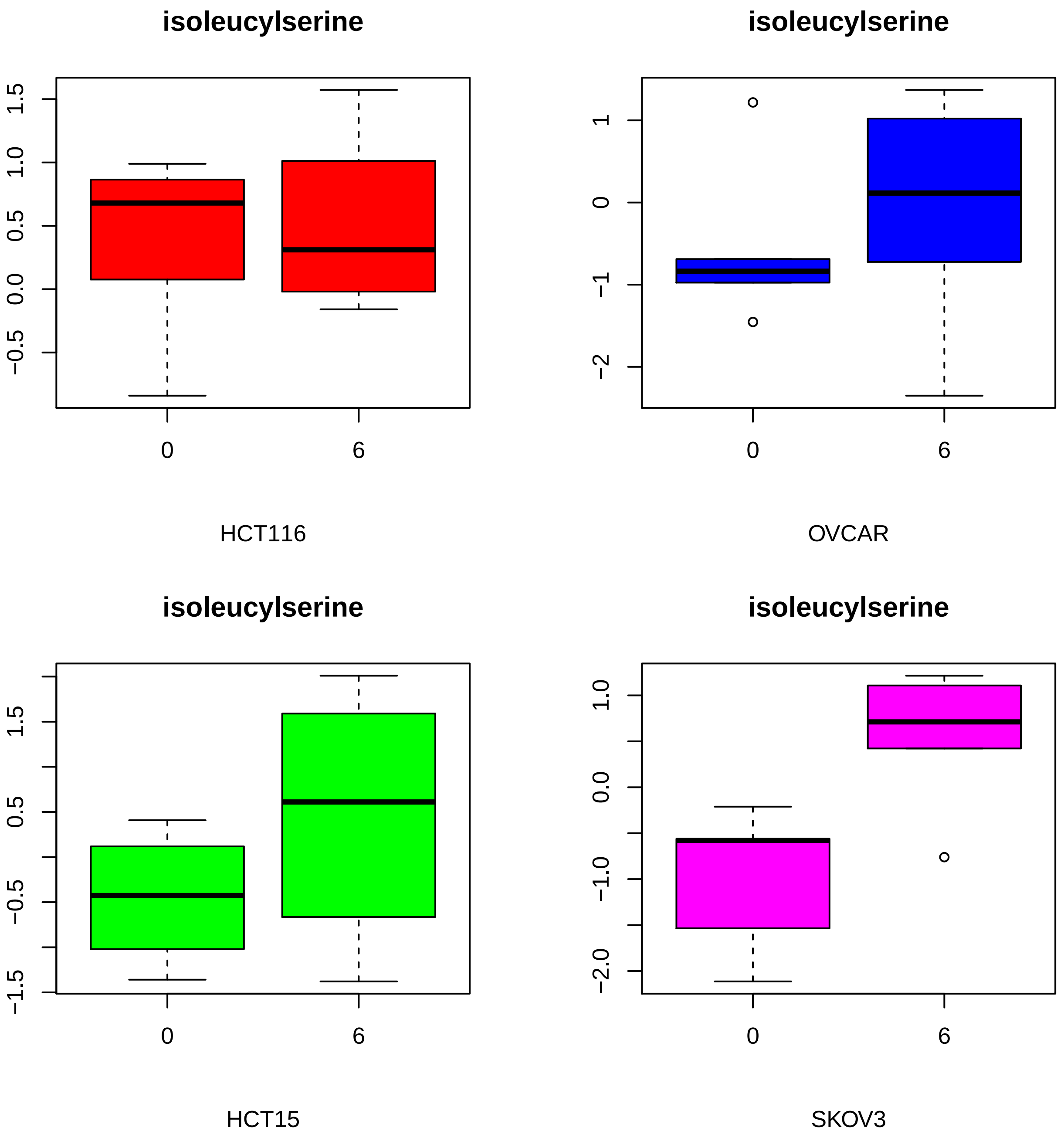
isoleucylserine
1.5
1.0
0.5
0.0
−0.5
0
6
HCT116
isoleucylserine
1
0
−1
−2
0
6
OVCAR
isoleucylserine
1.5
0.5
−0.5
−1.5
0
6
HCT15
isoleucylserine
1.0
0.0
−1.0
−2.0
0
6
SKOV3

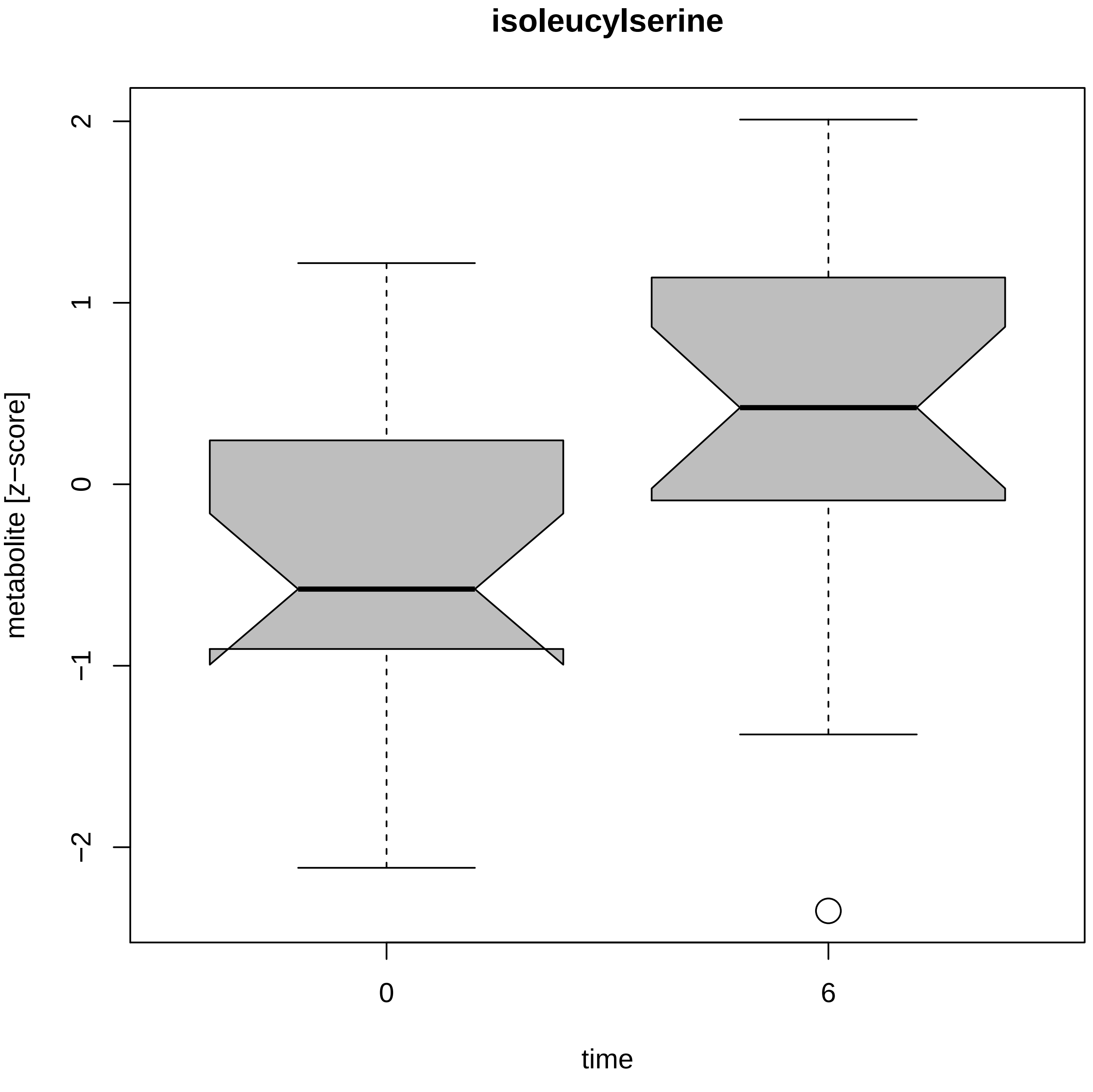

isoleucylserine
metabolite [z−score]
2
1
0
−1
−2
0
6
time

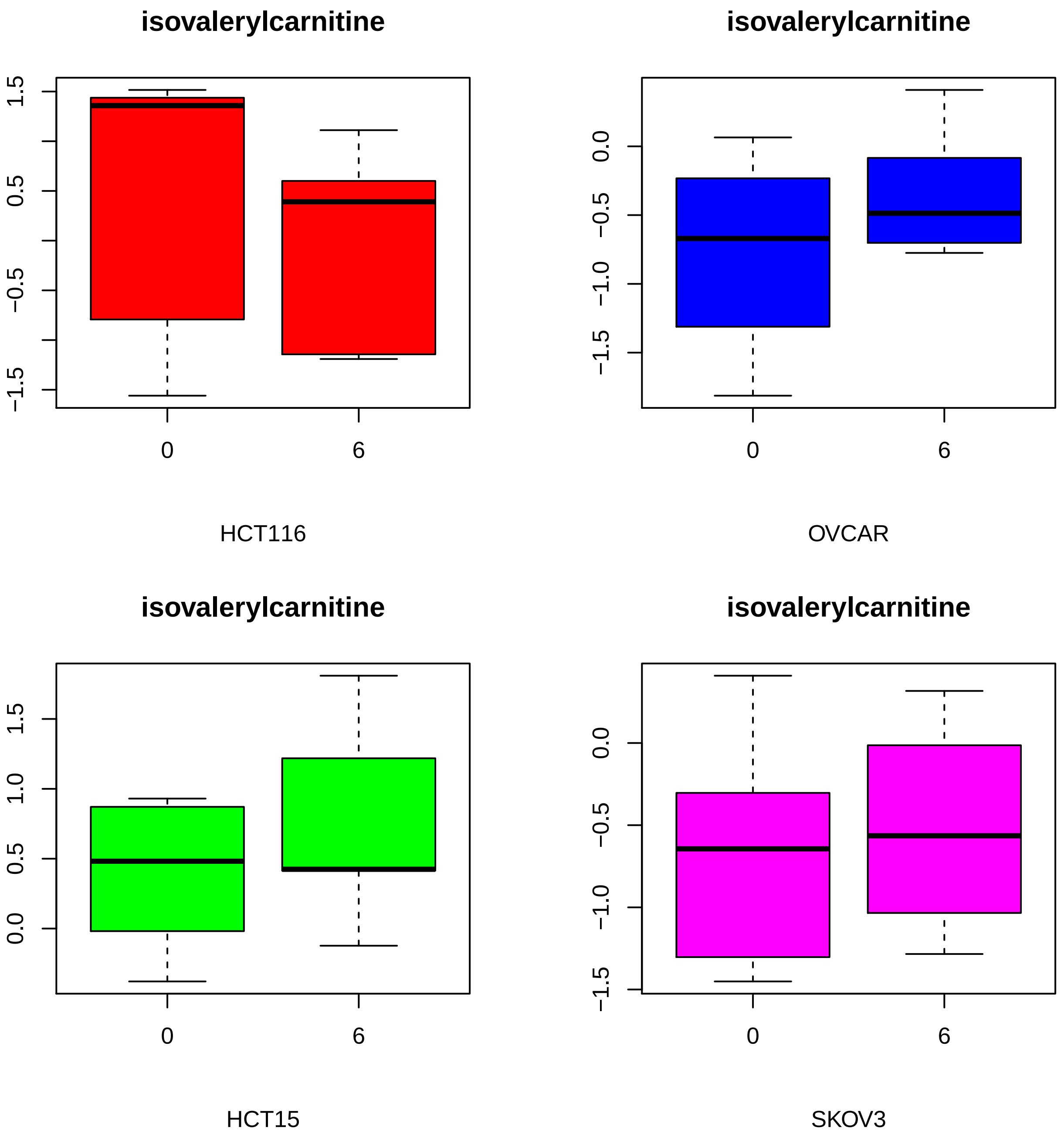
isovalerylcarnitine
1.5
0.5
−0.5
−1.5
0
6
HCT116
isovalerylcarnitine
0.0
−0.5
−1.0
−1.5
0
6
OVCAR
isovalerylcarnitine
1.5
1.0
0.5
0.0
0
6
HCT15
isovalerylcarnitine
0.0
−0.5
−1.0
−1.5
0
6
SKOV3

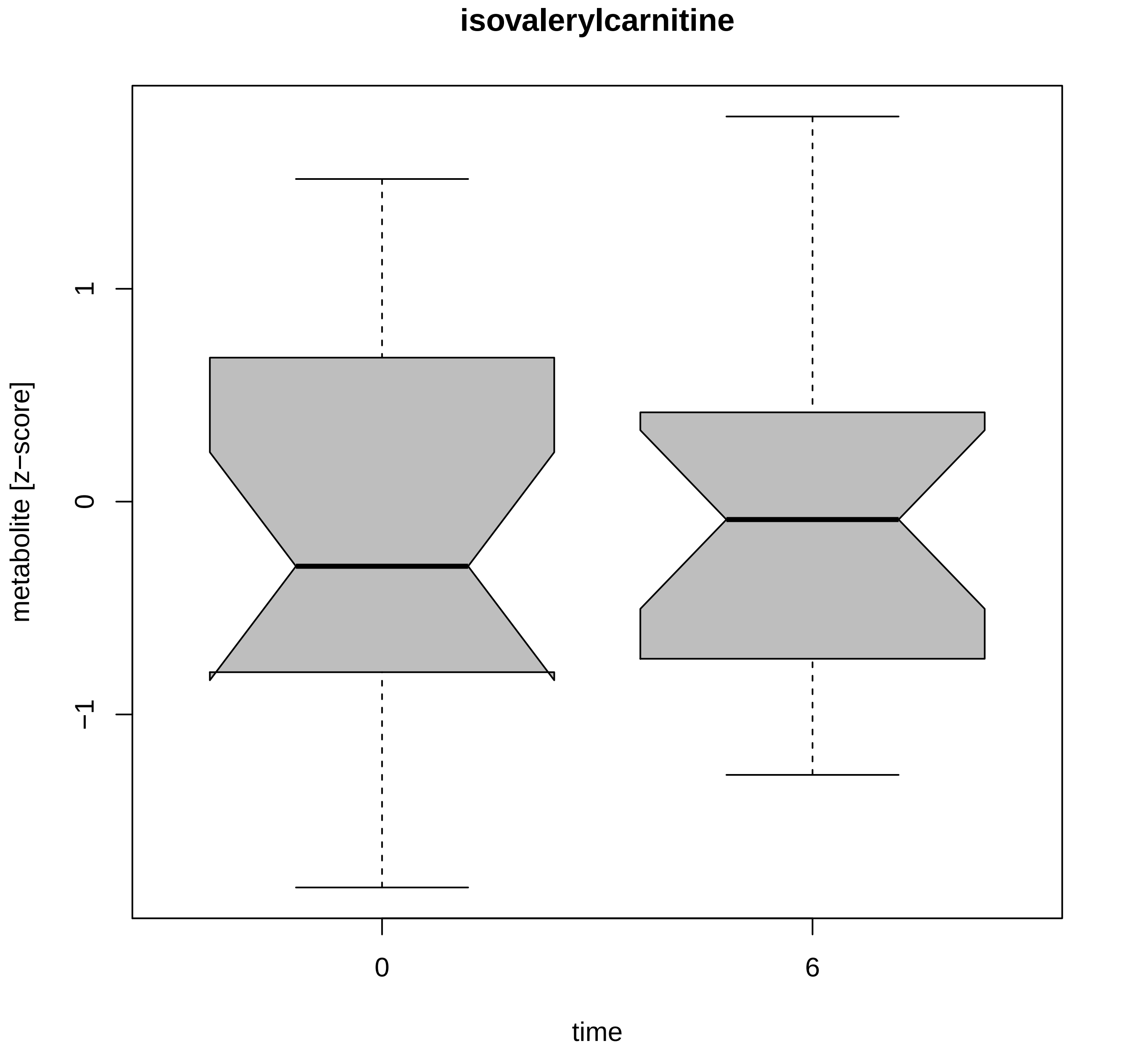
isovalerylcarnitine
metabolite [z−score]
1
0
−1
0
6
time

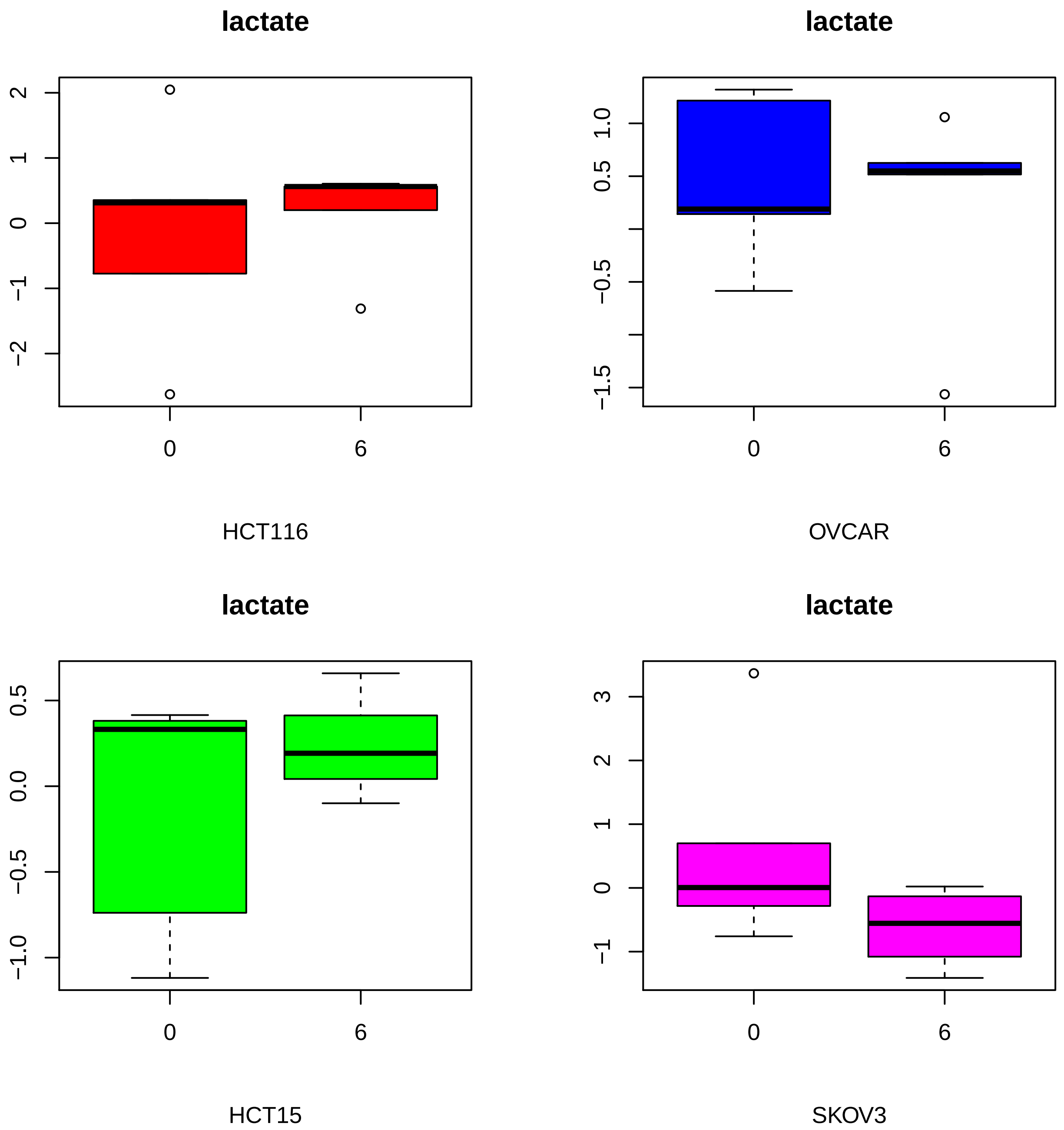
lactate
2
1
0
−1
−2
0
6
HCT116
lactate
1.0
0.5
−0.5
−1.5
0
6
OVCAR
lactate
0.5
0.0
−0.5
−1.0
0
6
HCT15
lactate
3
2
1
0
−1
0
6
SKOV3

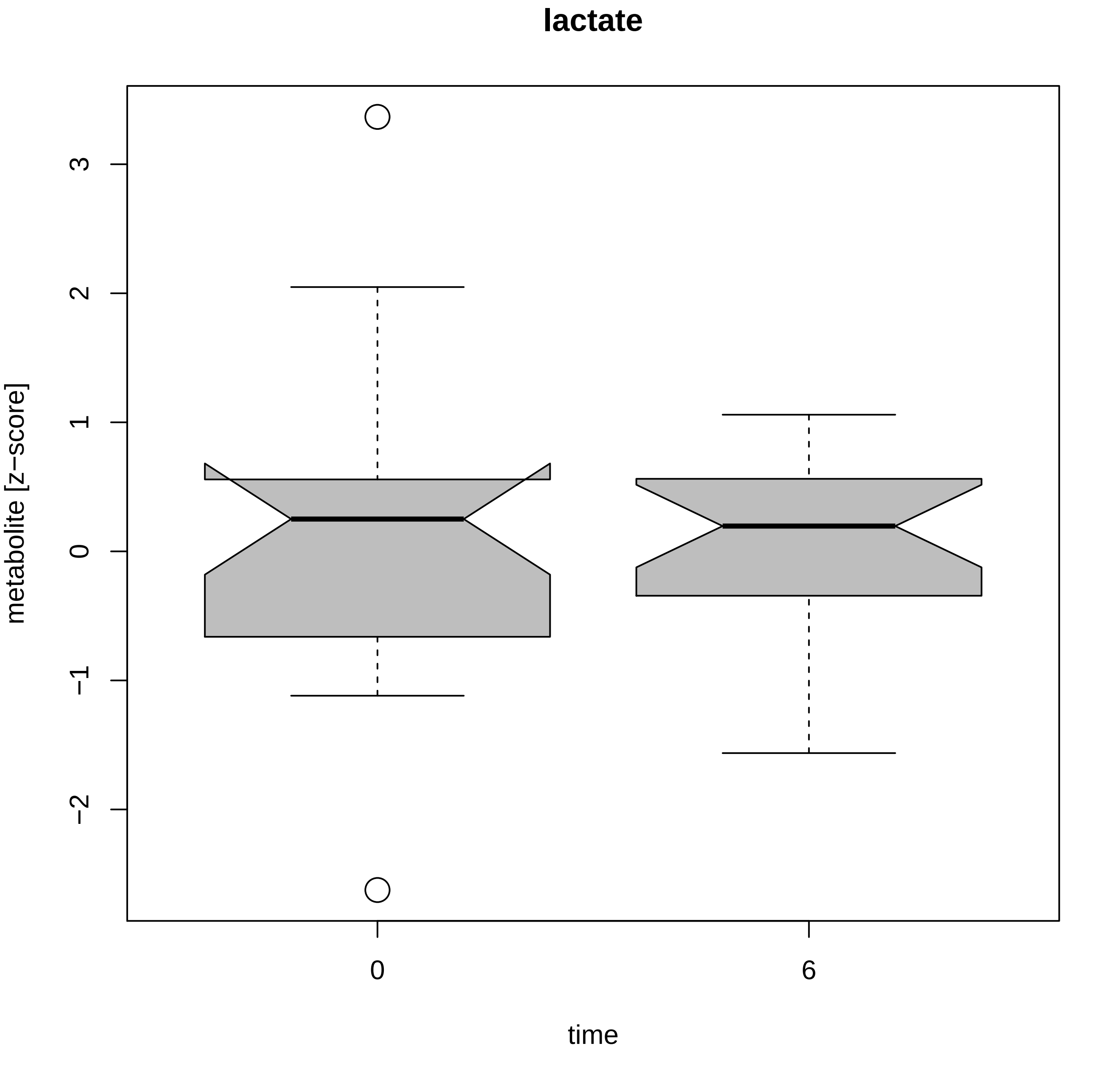
lactate
metabolite [z−score]
3
2
1
0
−1
−2
0
6
time

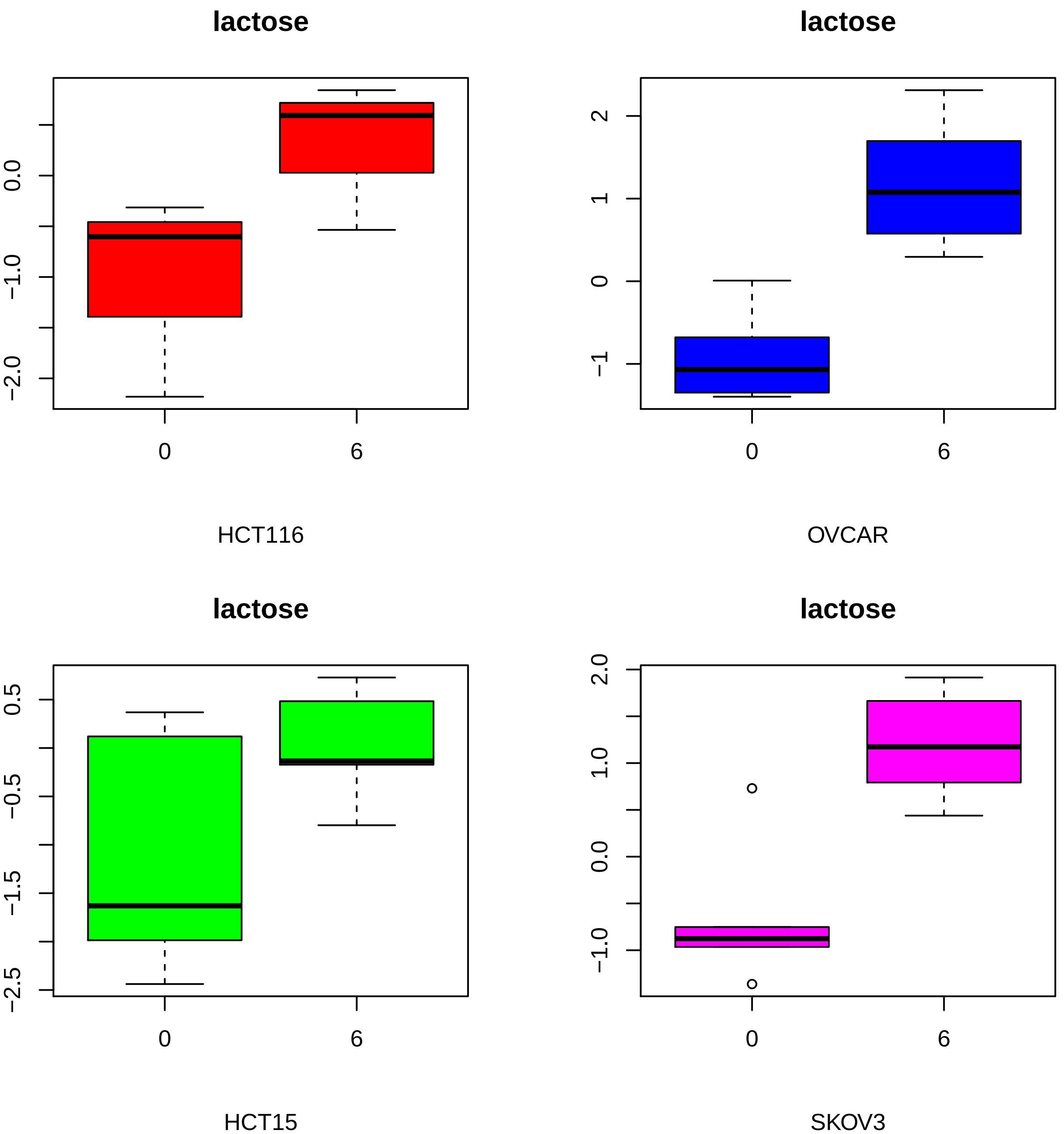

lactose
0.0
−1.0
−2.0
0
6
HCT116
lactose
2
1
0
−1
0
6
OVCAR
lactose
0.5
−0.5
−1.5
−2.5
0
6
HCT15
lactose
2.0
1.0
0.0
−1.0
0
6
SKOV3

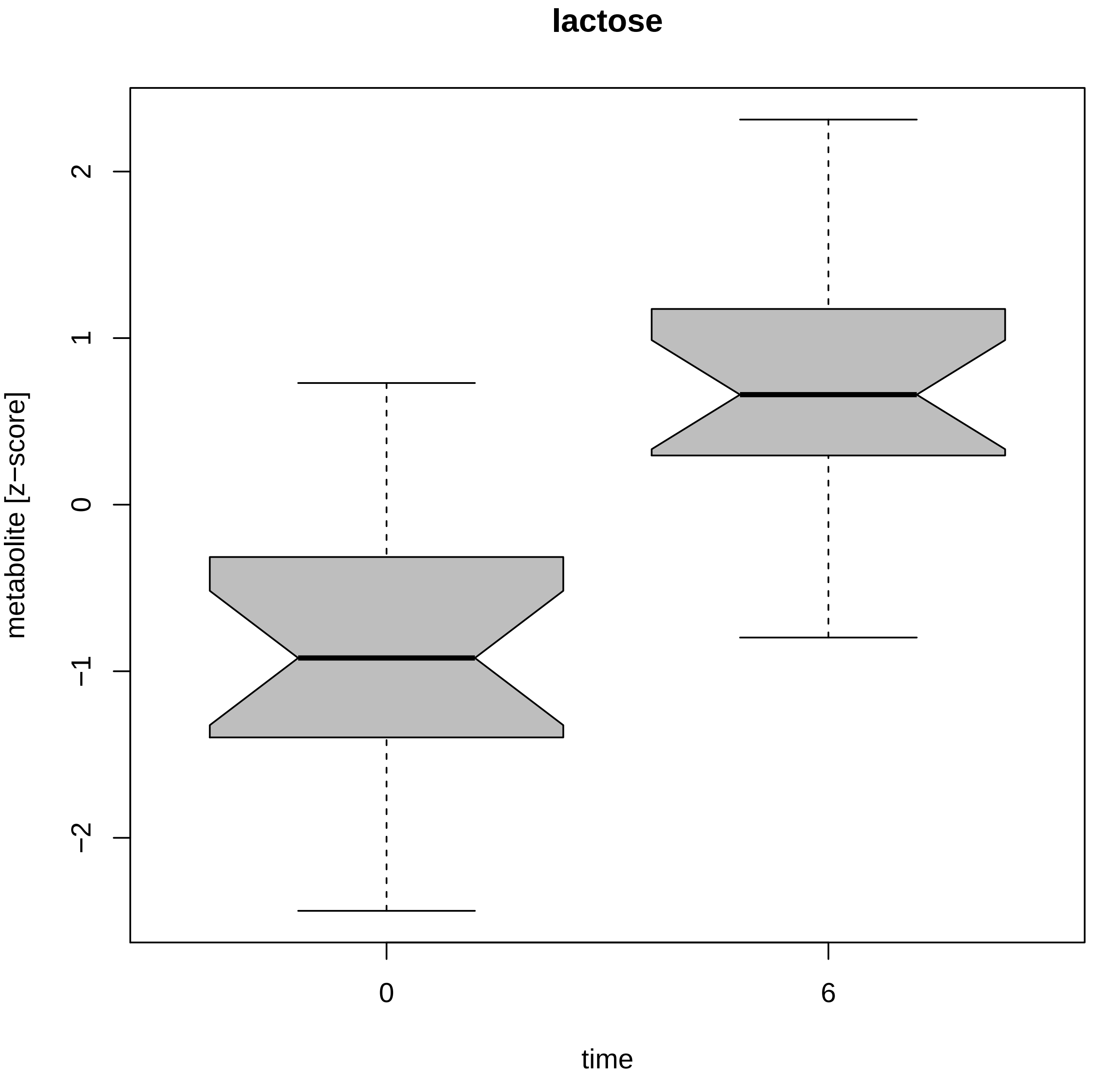
lactose
metabolite [z−score]
2
1
0
−1
−2
0
6
time

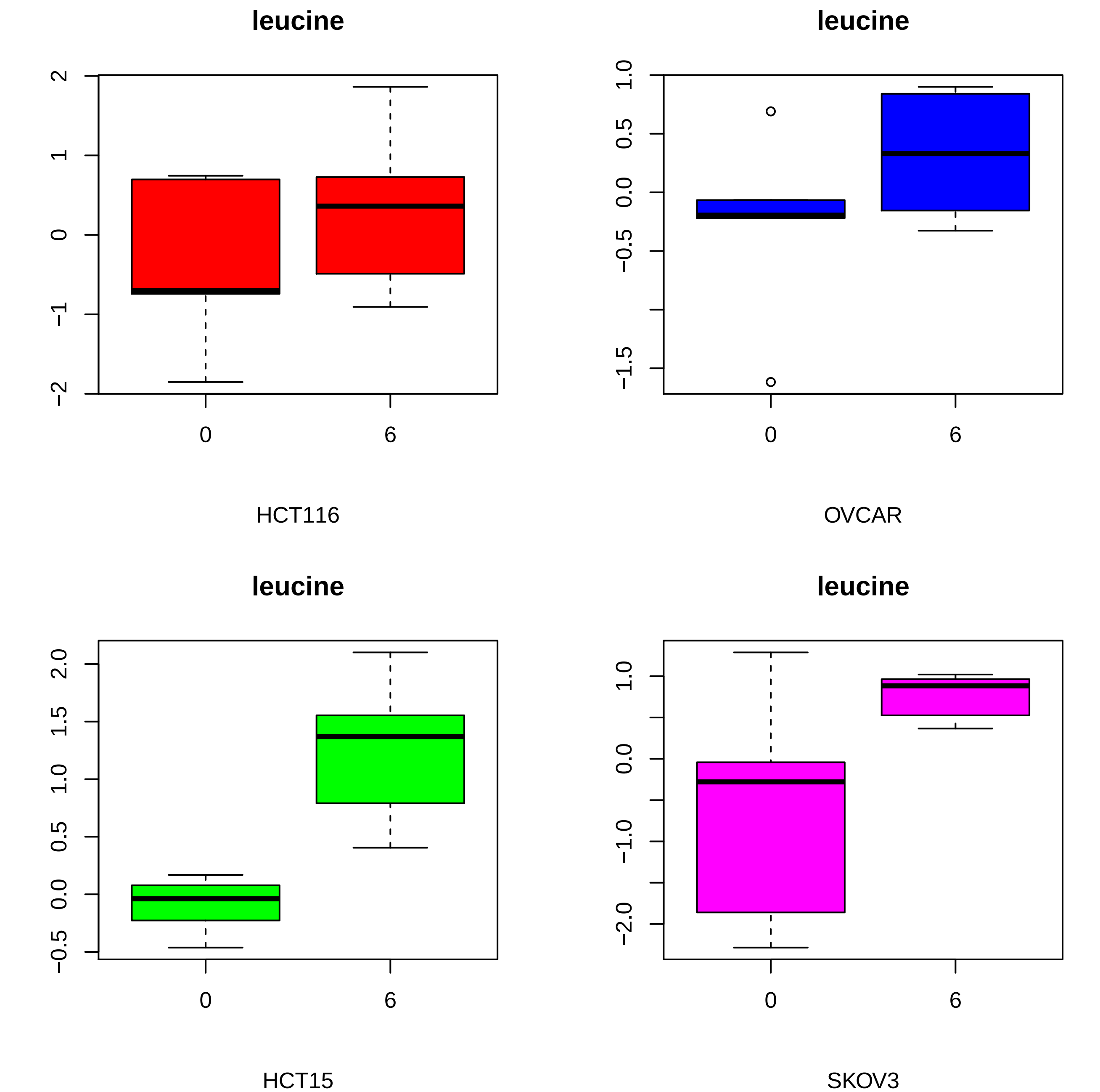
leucine
2
1
0
−1
−2
0
6
HCT116
leucine
1.0
0.5
0.0
−0.5
−1.5
0
6
OVCAR
leucine
2.0
1.5
1.0
0.5
0.0
−0.5
0
6
HCT15
leucine
1.0
0.0
−1.0
−2.0
0
6
SKOV3

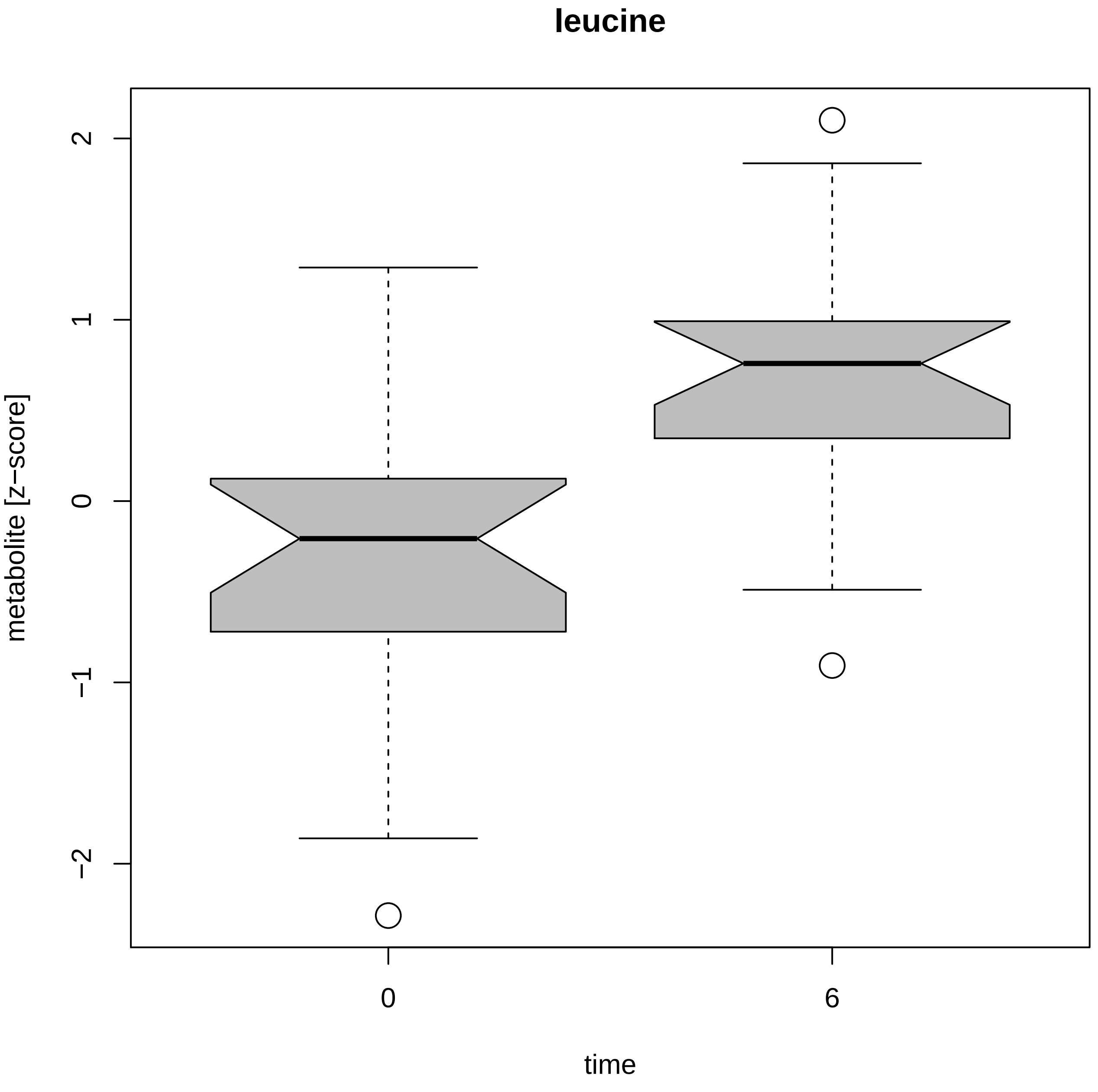

leucine
metabolite [z−score]
2
1
0
−1
−2
0
6
time

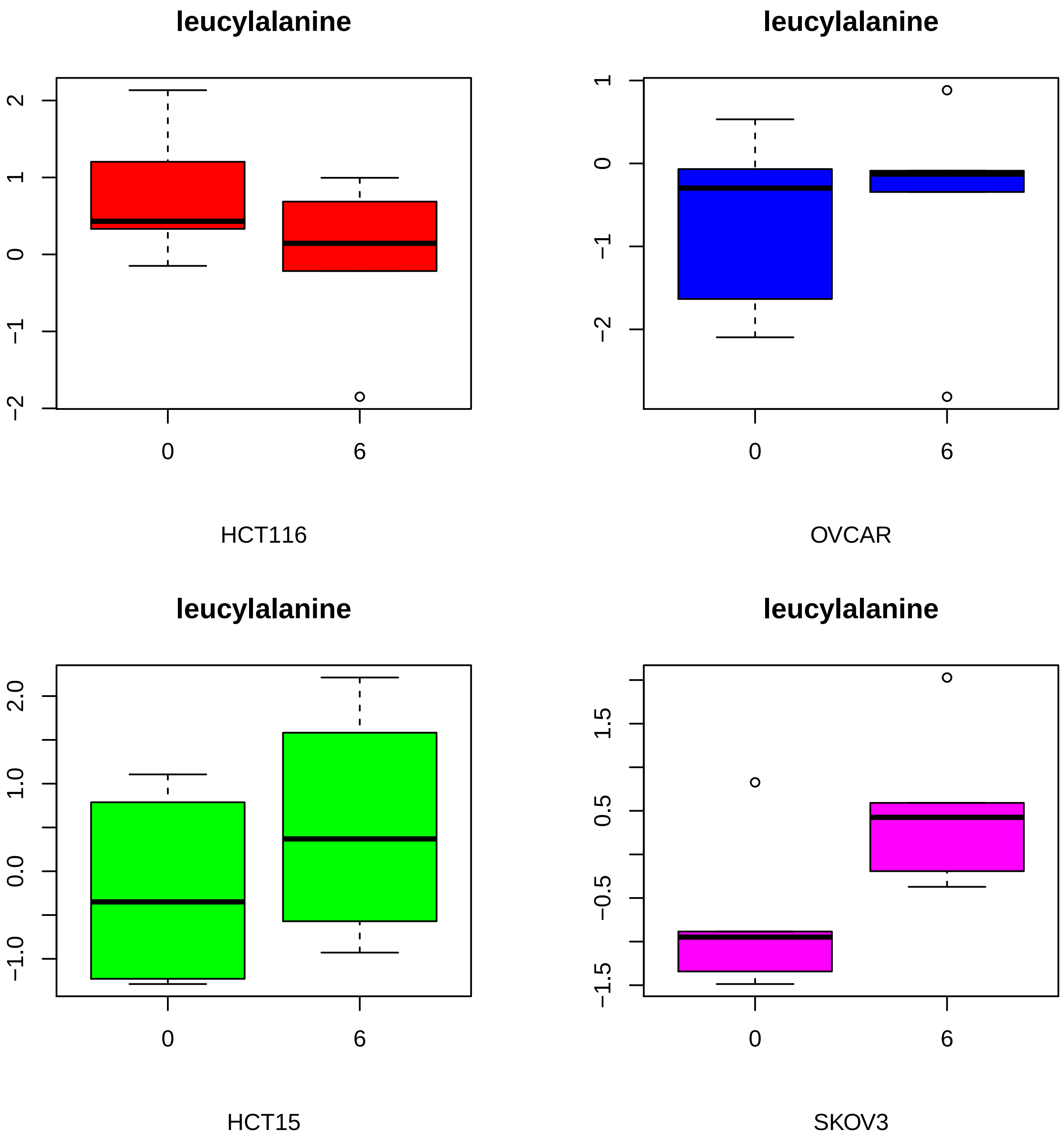
leucylalanine
2
1
0
−1
−2
0
6
HCT116
leucylalanine
1
0
−1
−2
0
6
OVCAR
leucylalanine
2.0
1.0
0.0
−1.0
0
6
HCT15
leucylalanine
1.5
0.5
−0.5
−1.5
0
6
SKOV3

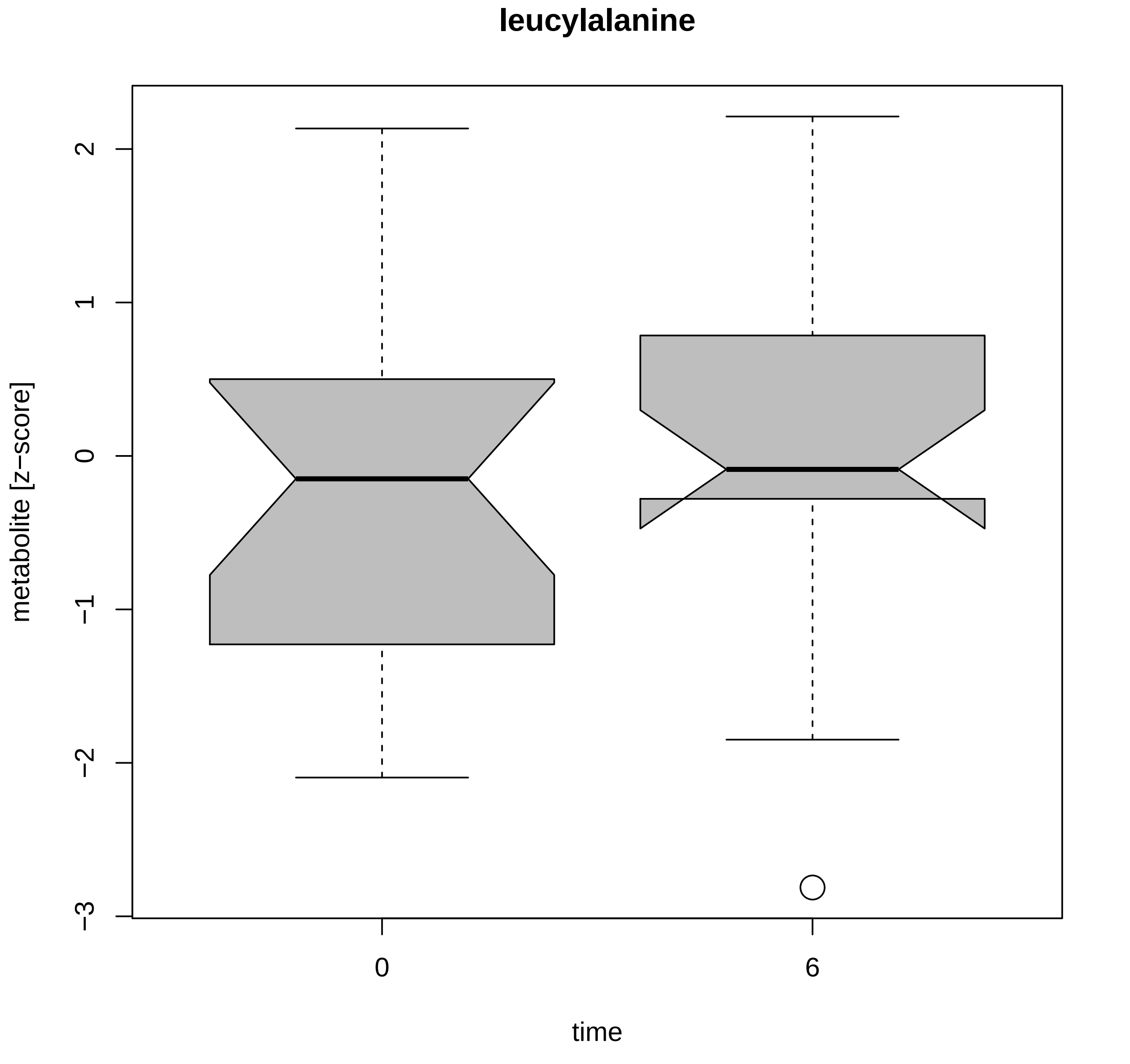

leucylalanine
metabolite [z−score]
2
1
0
−1
−2
−3
0
6
time

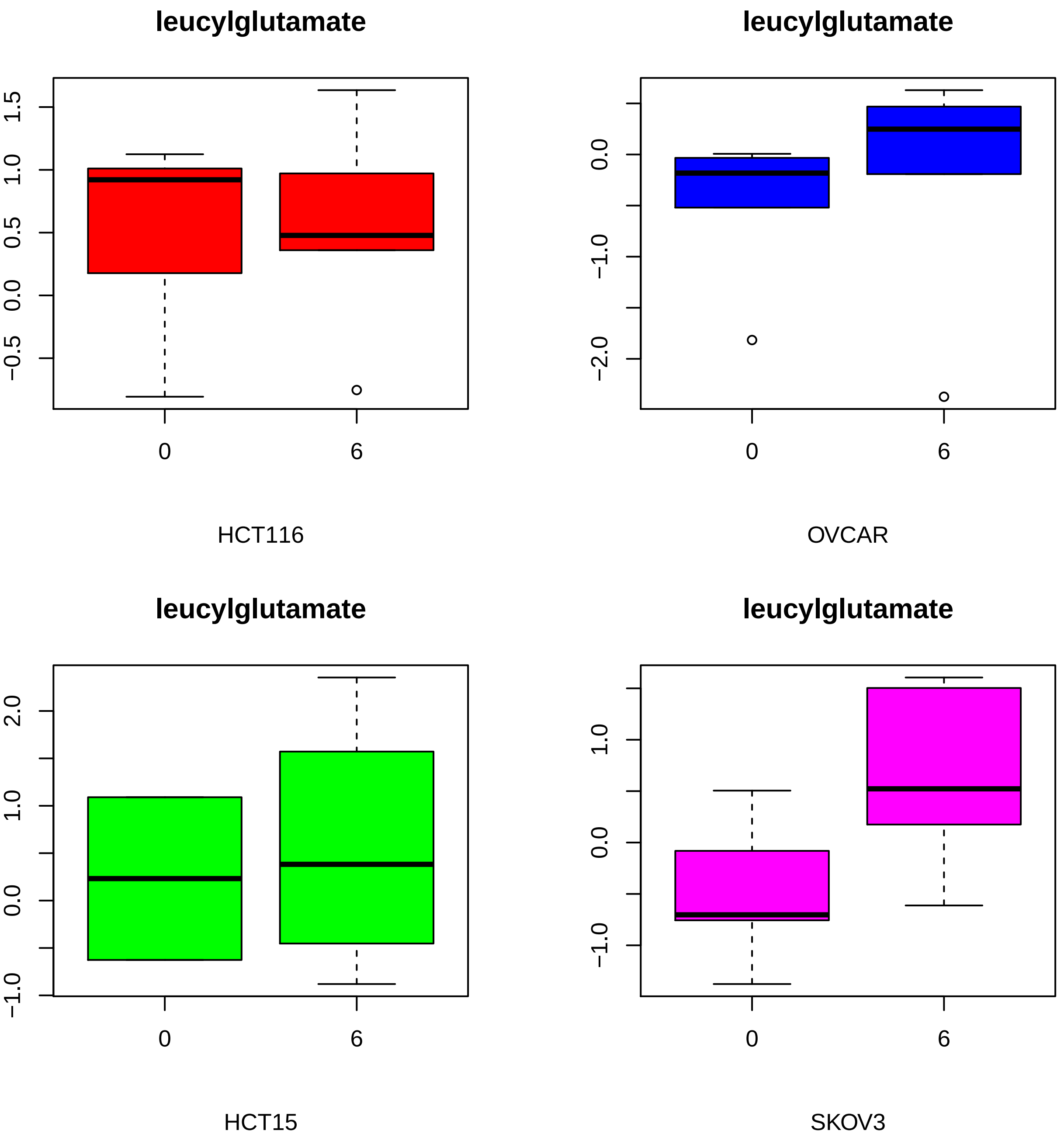
leucylglutamate
HCT116
OVCAR
HCT15
SKOV3
0
6
1.5
1.0
0.5
0.0
−0.5
−1.0
−2.0
2.0

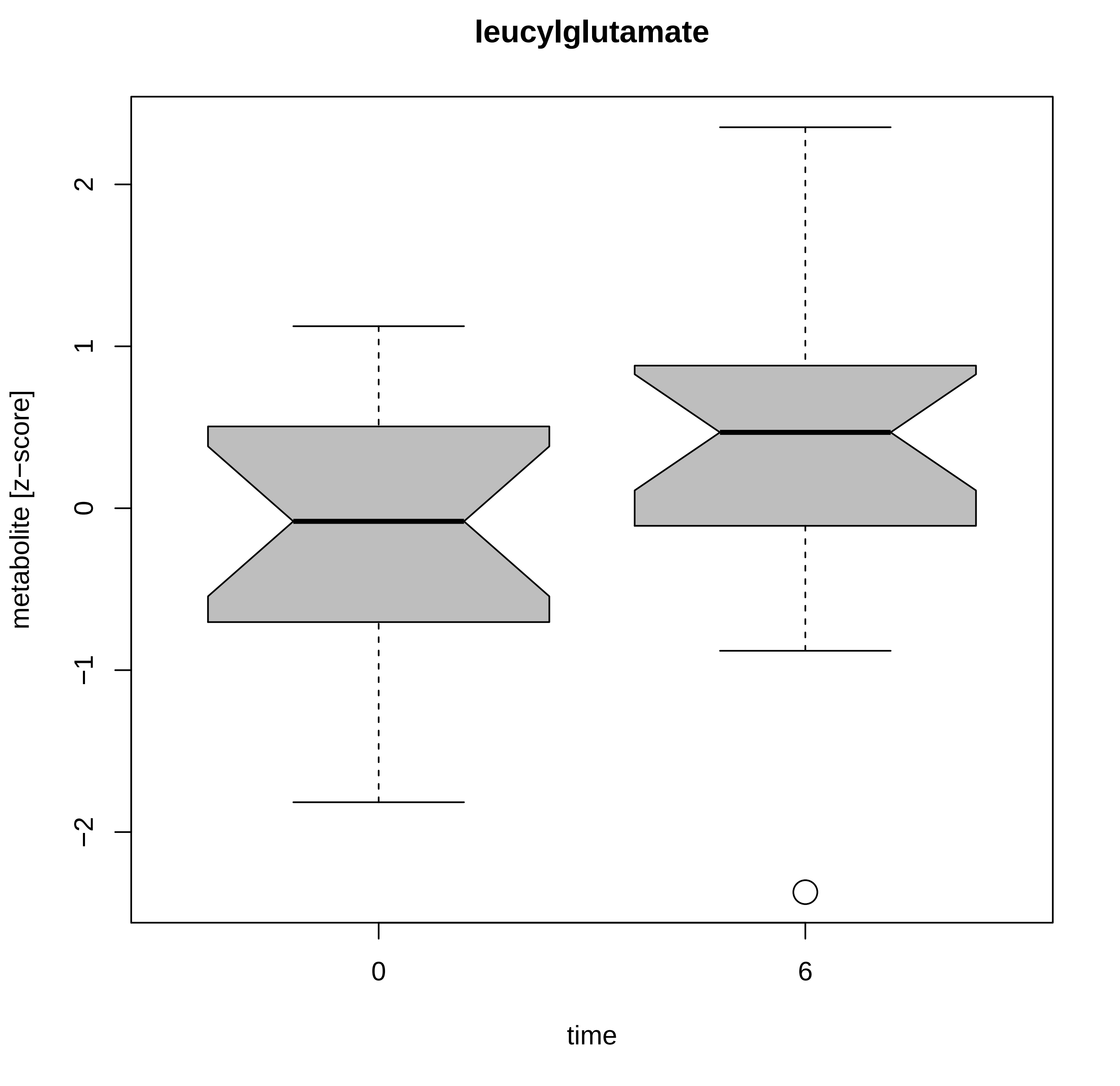
leucylglutamate
metabolite [z−score]
2
1
0
−1
−2
0
6
time

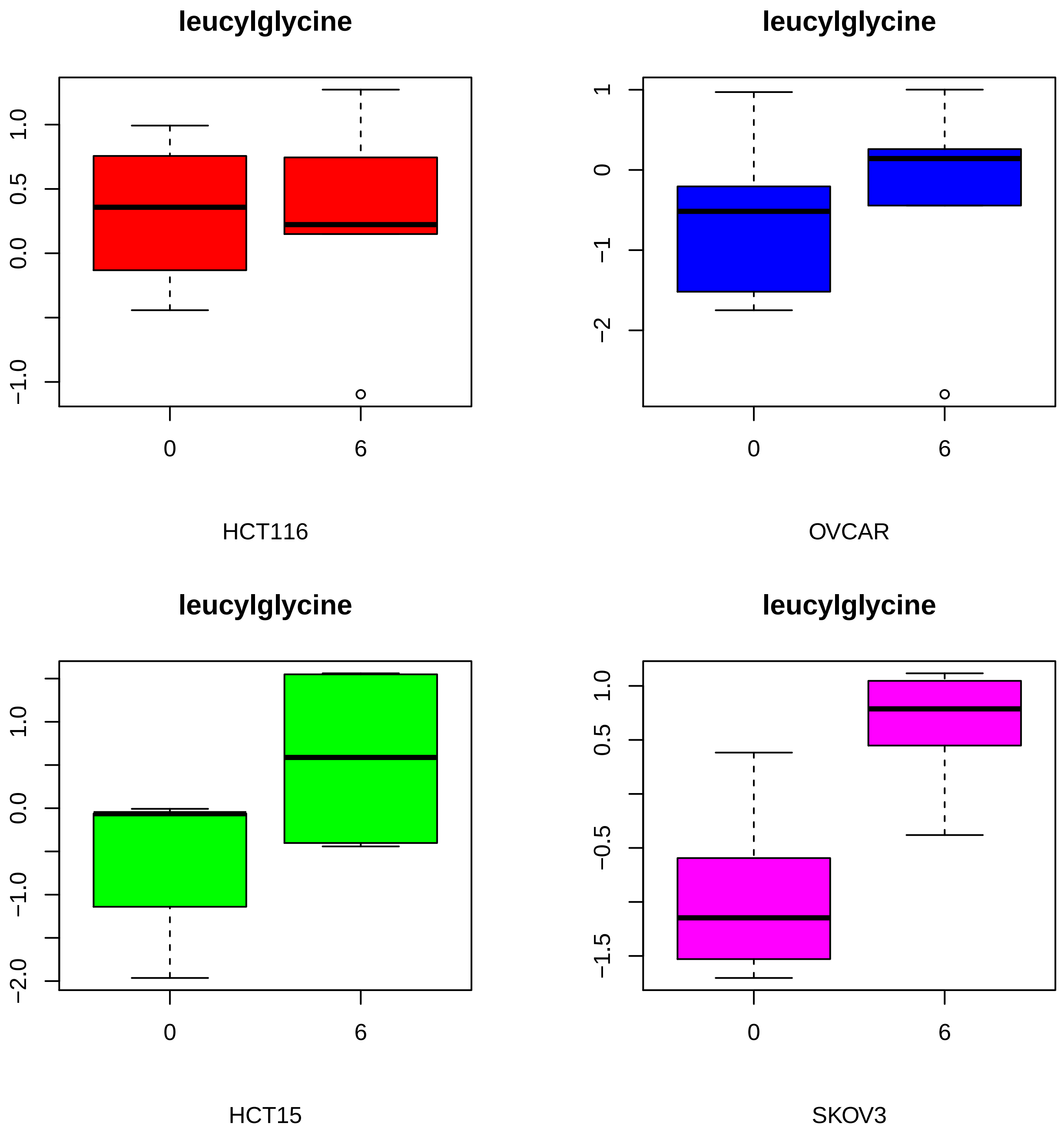
leucylglycine
1.0
0.5
0.0
−1.0
0
6
HCT116
leucylglycine
1
0
−1
−2
0
6
OVCAR
leucylglycine
1.0
0.0
−1.0
−2.0
0
6
HCT15
leucylglycine
1.0
0.5
−0.5
−1.5
0
6
SKOV3

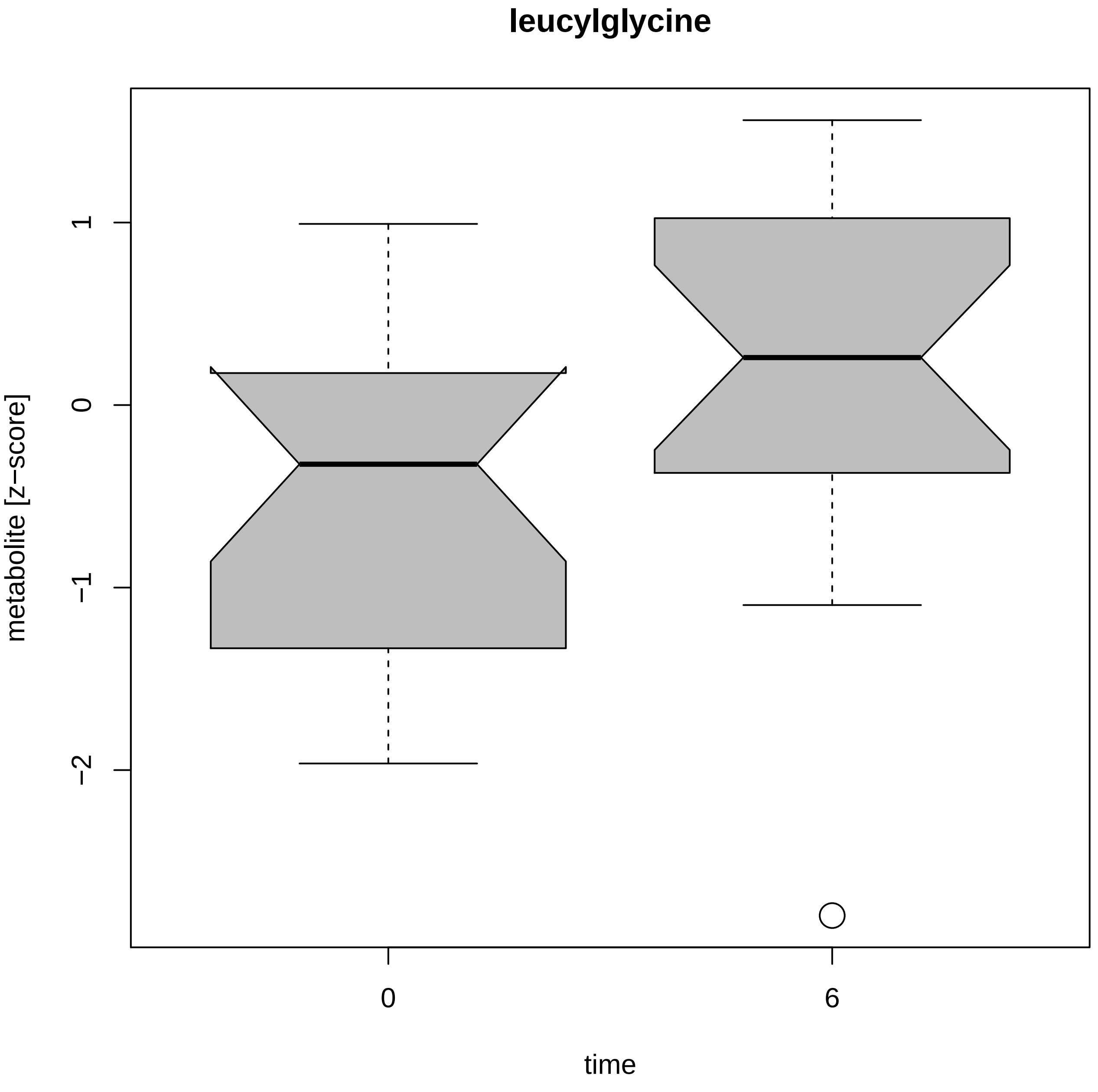
leucylglycine
metabolite [z−score]
1
0
−1
−2
0
6
time

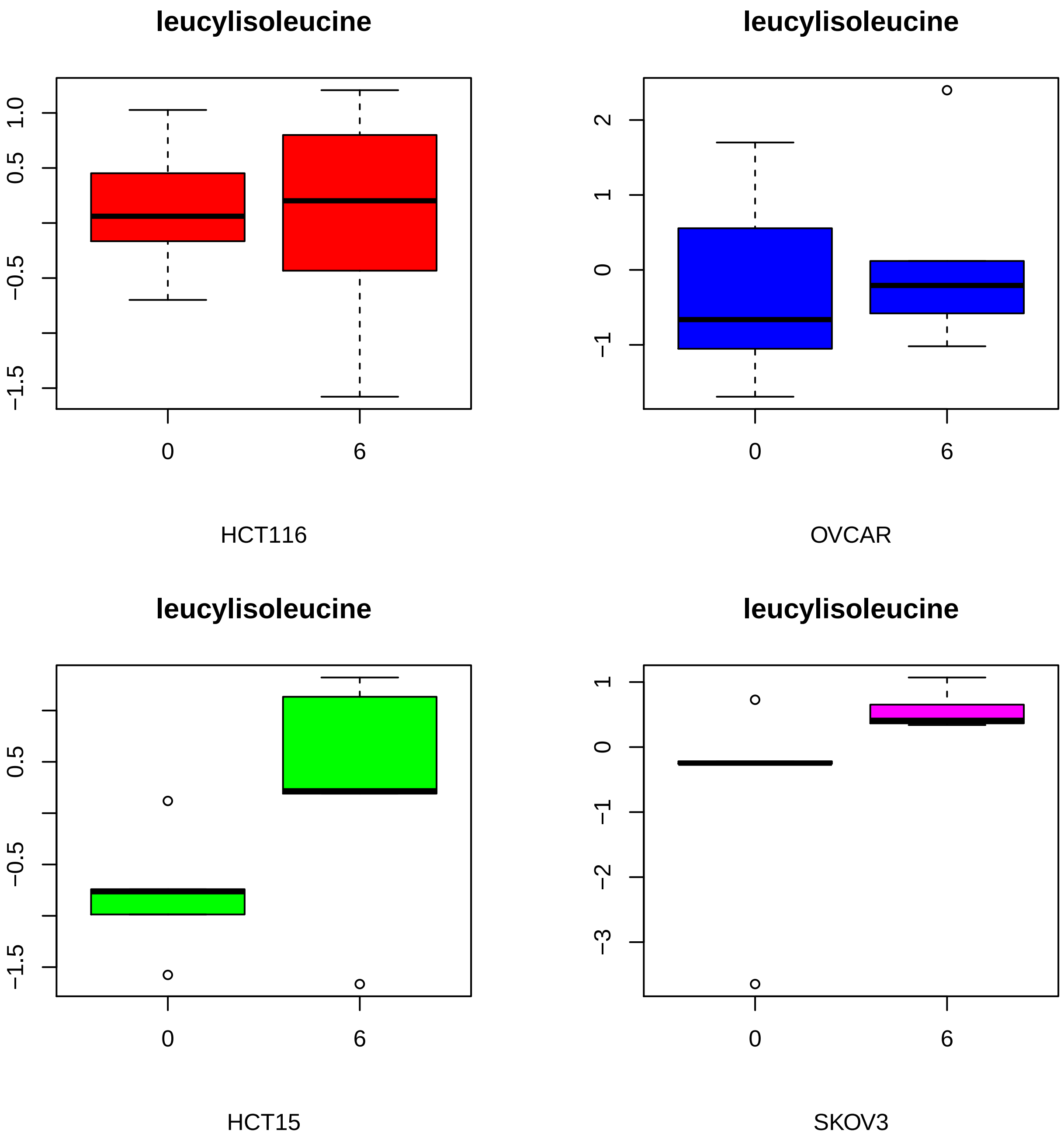
leucylisoleucine
1.0
0.5
−0.5
−1.5
0
6
HCT116
leucylisoleucine
2
1
0
−1
0
6
OVCAR
leucylisoleucine
0.5
−0.5
−1.5
0
6
HCT15
leucylisoleucine
1
0
−1
−2
−3
0
6
SKOV3

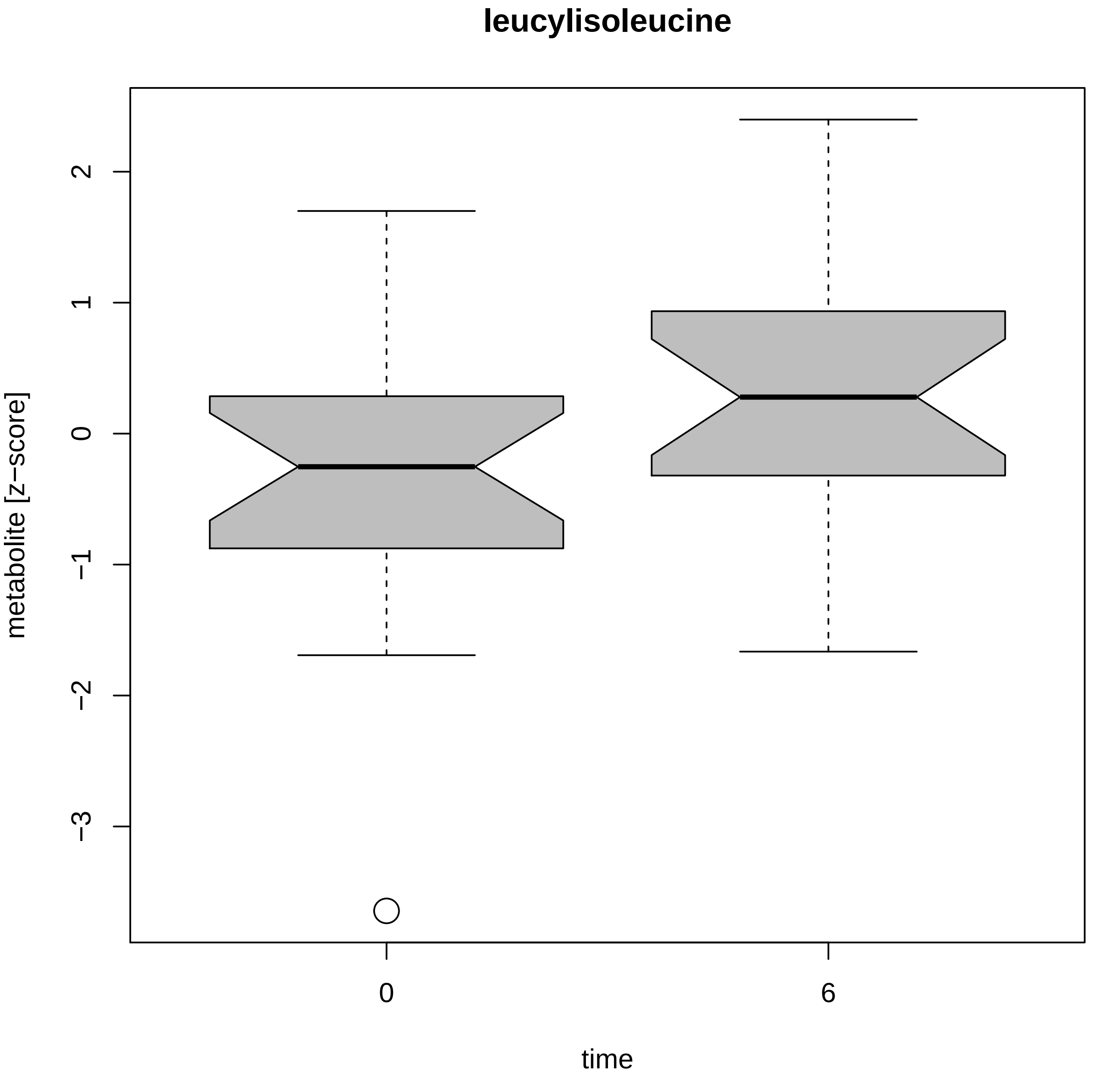
leucylisoleucine
metabolite [z−score]
2
1
0
−1
−2
−3
0
6
time

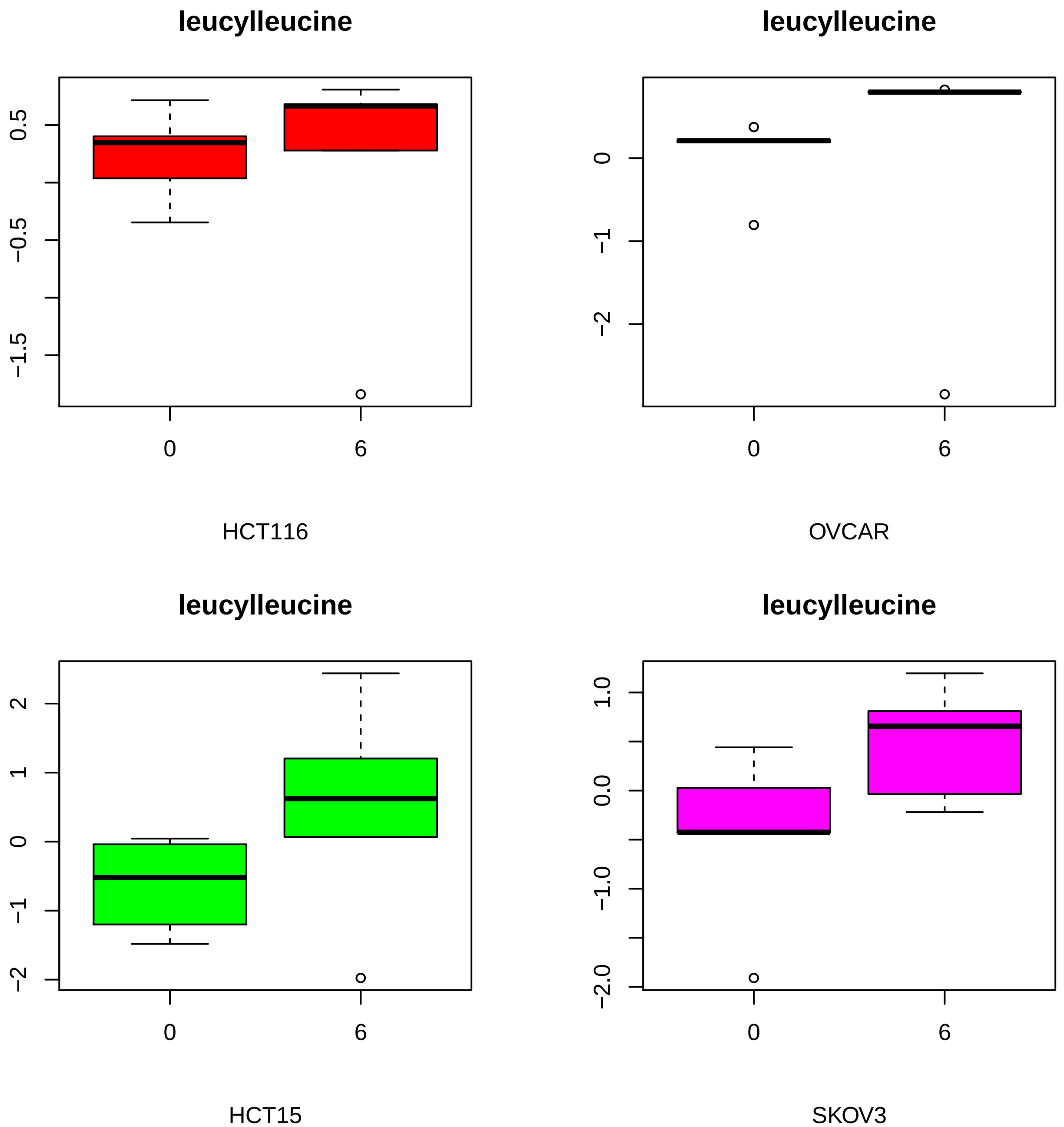
leucylleucine
0.5
−0.5
−1.5
0
6
HCT116
leucylleucine
0
−1
−2
0
6
OVCAR
leucylleucine
2
1
0
−1
−2
0
6
HCT15
leucylleucine
1.0
0.0
−1.0
−2.0
0
6
SKOV3

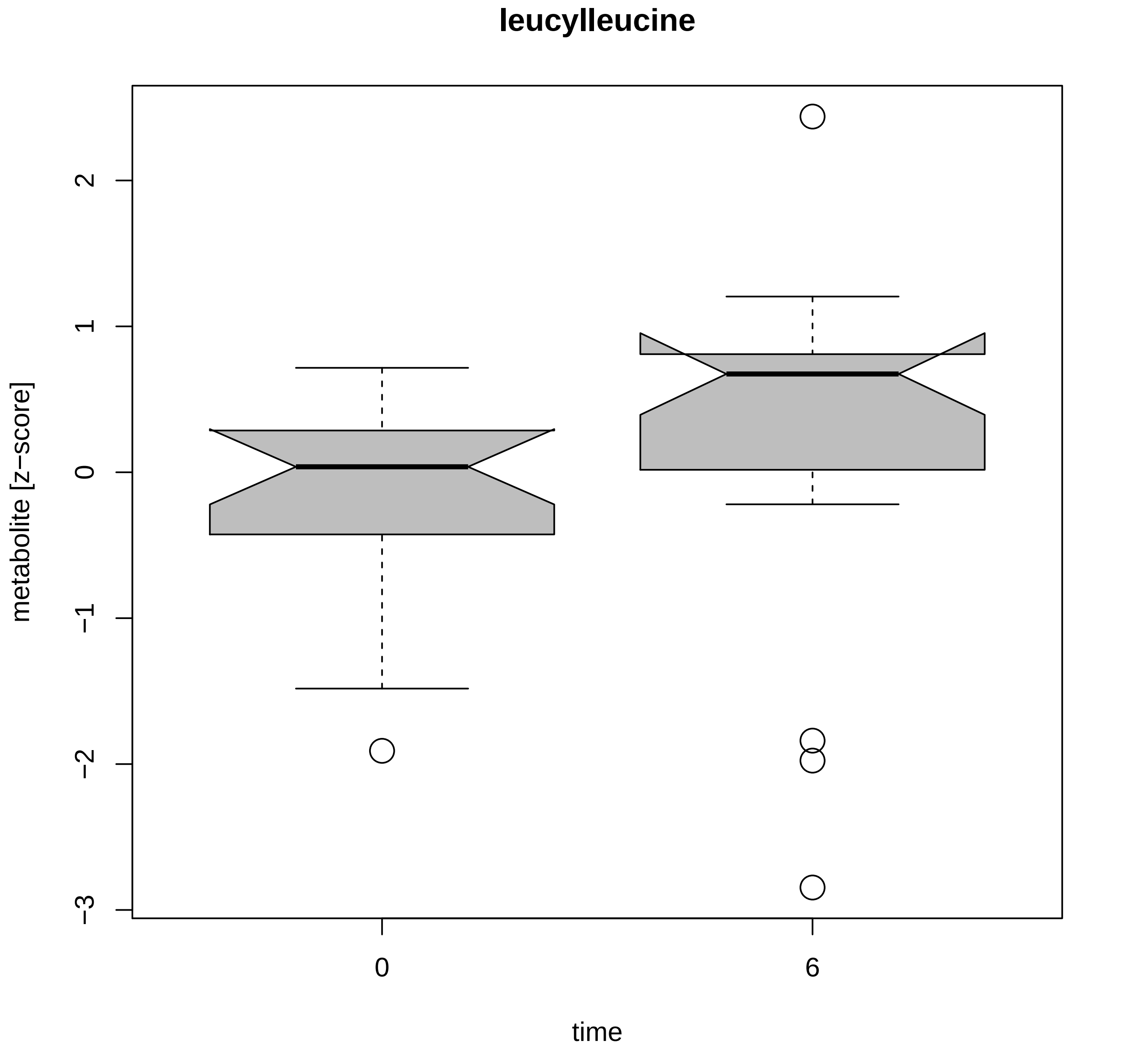

leucylleucine
metabolite [z−score]
2
1
0
−1
−2
−3
0
6
time

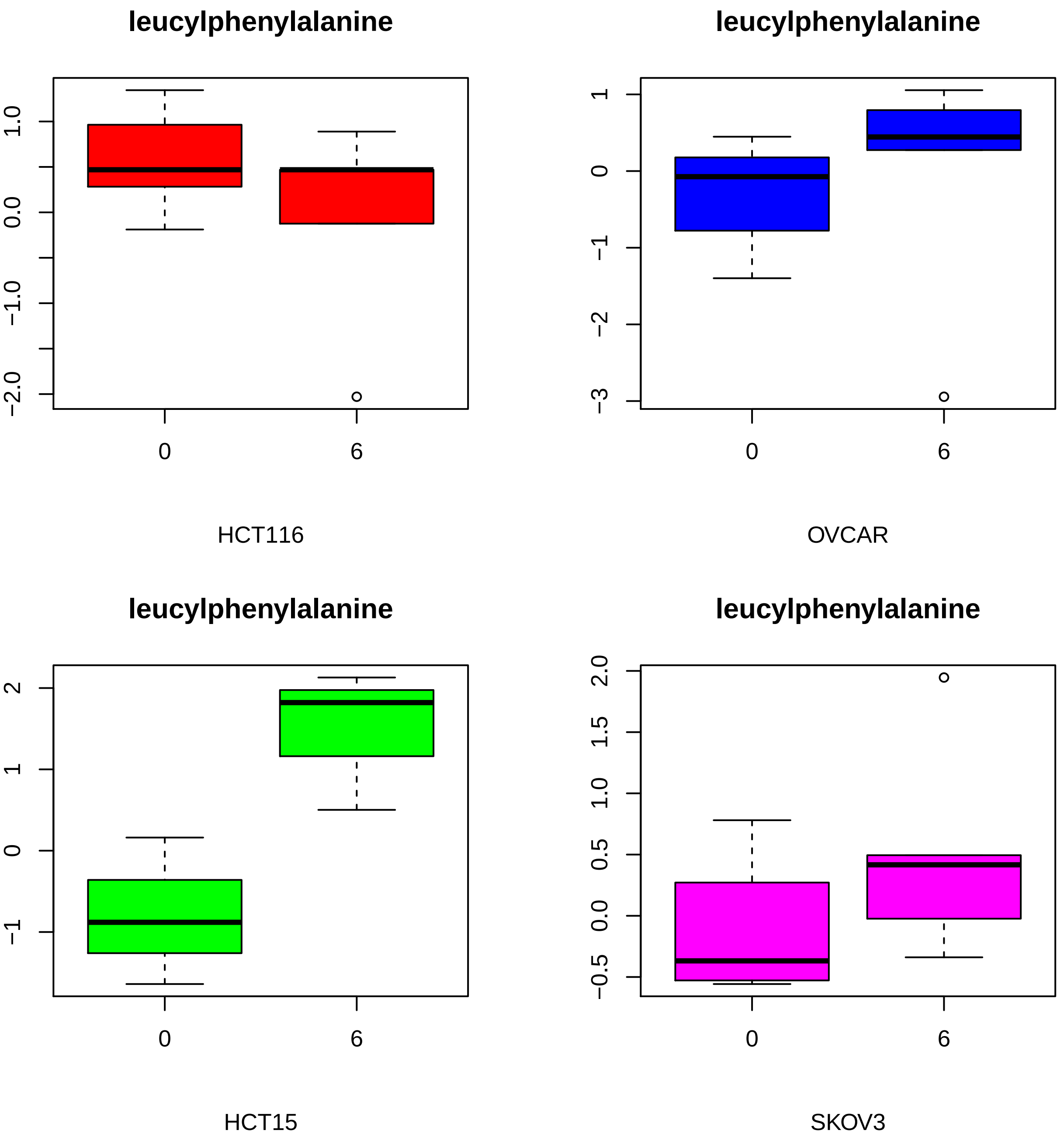

leucylphenylalanine
1.0
0.0
−1.0
−2.0
0
6
HCT116
leucylphenylalanine
1
0
−1
−2
−3
0
6
OVCAR
leucylphenylalanine
2
1
0
−1
0
6
HCT15
leucylphenylalanine
2.0
1.5
1.0
0.5
0.0
−0.5
0
6
SKOV3

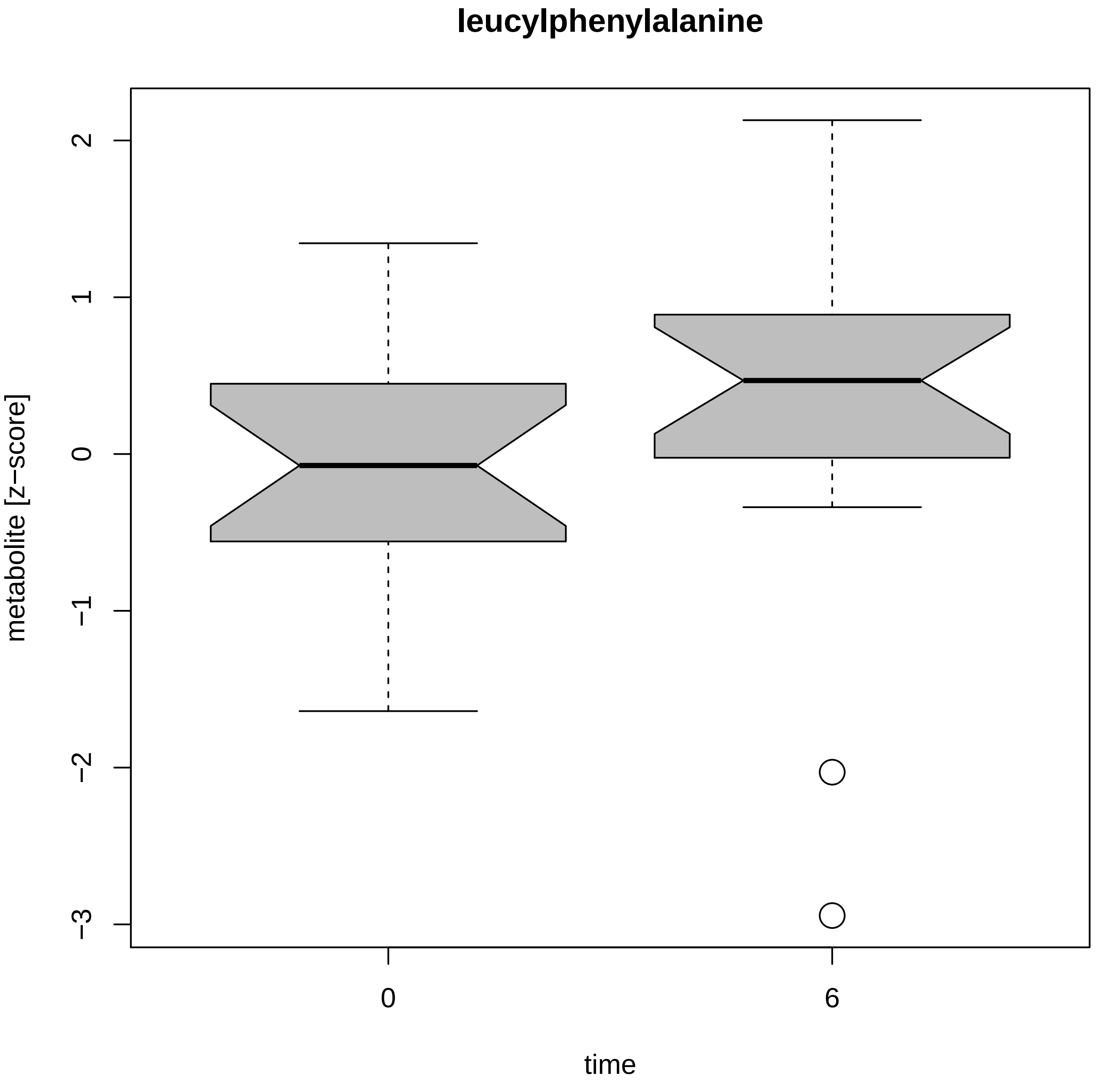
leucylphenylalanine
metabolite [z−score]
−3
−2
−1
0
1
2
0
6
time

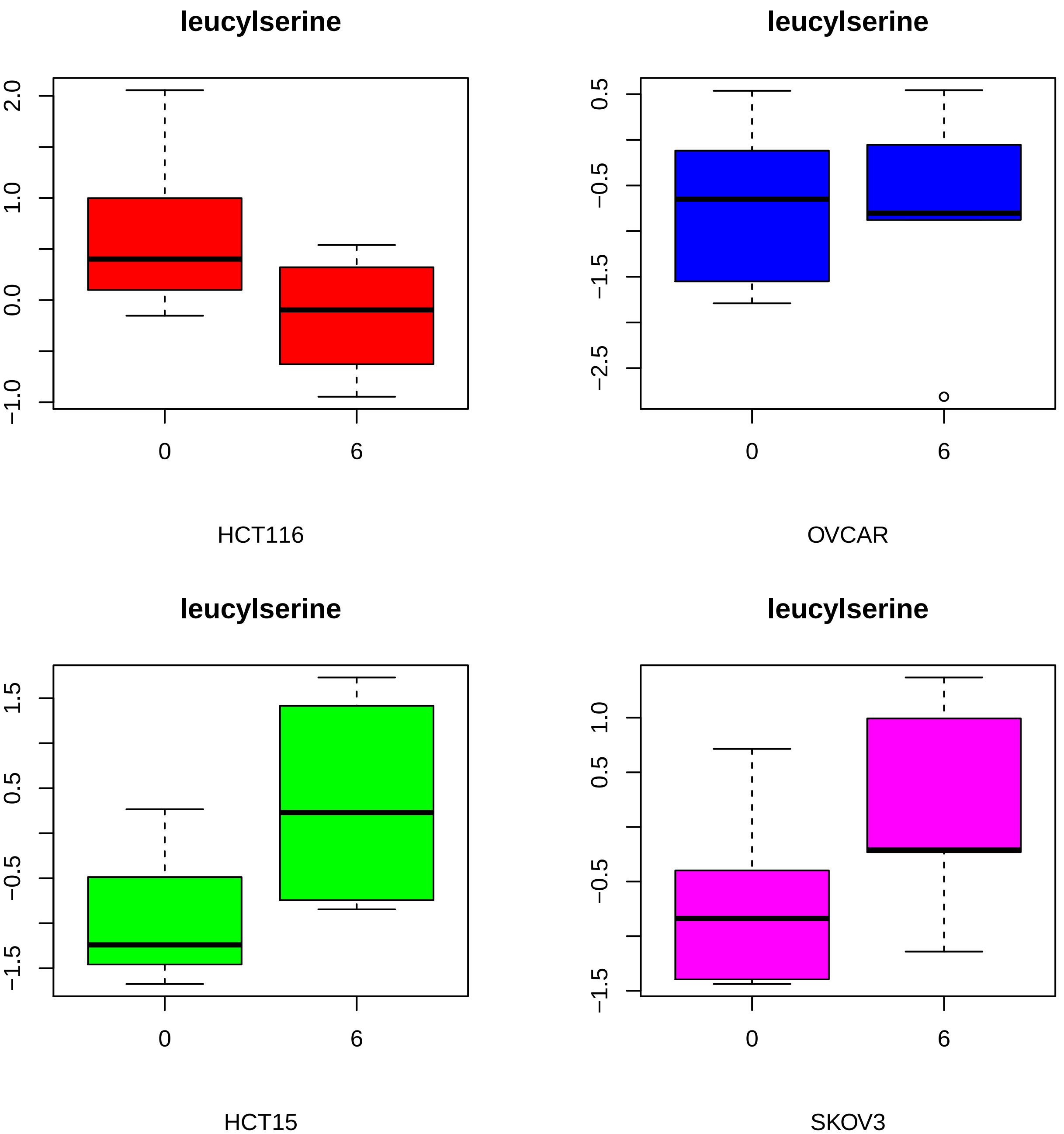
leucylserine
2.0
1.0
0.0
−1.0
0
6
HCT116
leucylserine
0.5
−0.5
−1.5
−2.5
0
6
OVCAR
leucylserine
1.5
0.5
−0.5
−1.5
0
6
HCT15
leucylserine
1.0
0.5
−0.5
−1.5
0
6
SKOV3

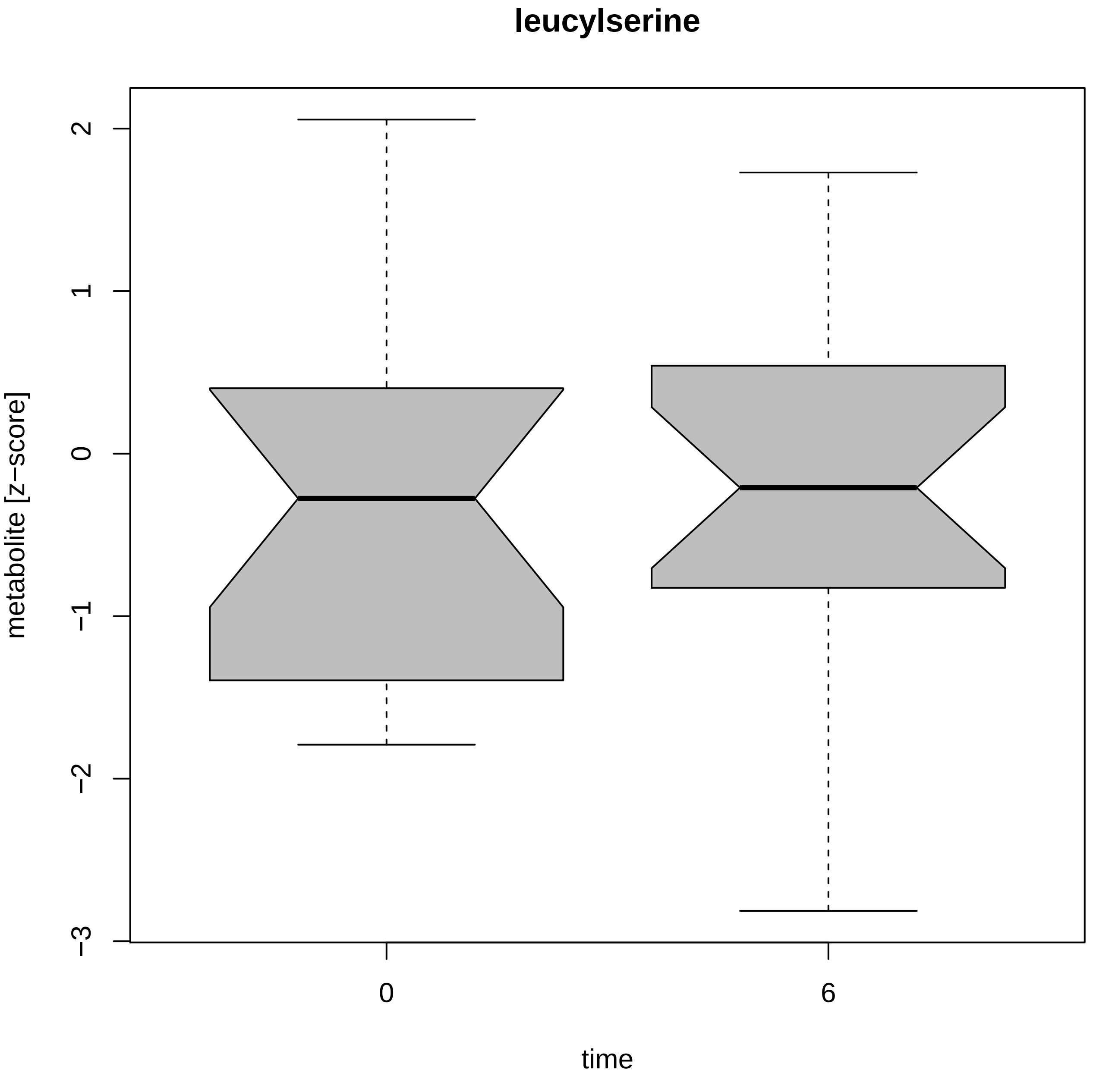

leucylserine
metabolite [z−score]
2
1
0
−1
−2
−3
0
6
time

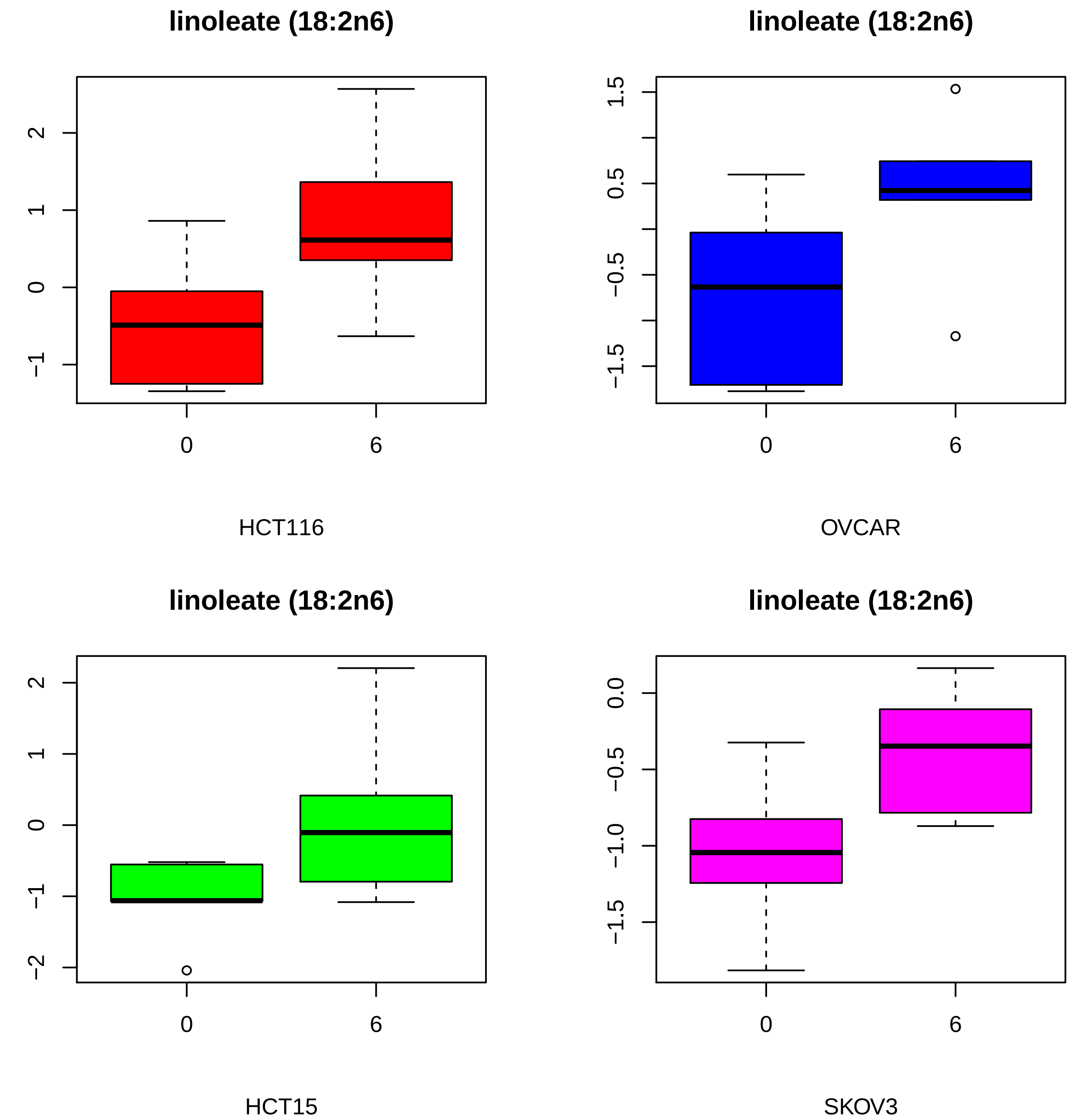
linoleate (18:2n6)
−1
0
1
2
0
6
HCT116
linoleate (18:2n6)
−1.5
−0.5
0.5
1.5
0
6
OVCAR
linoleate (18:2n6)
−2
−1
0
1
2
0
6
HCT15
linoleate (18:2n6)
−1.5
−1.0
−0.5
0.0
0
6
SKOV3

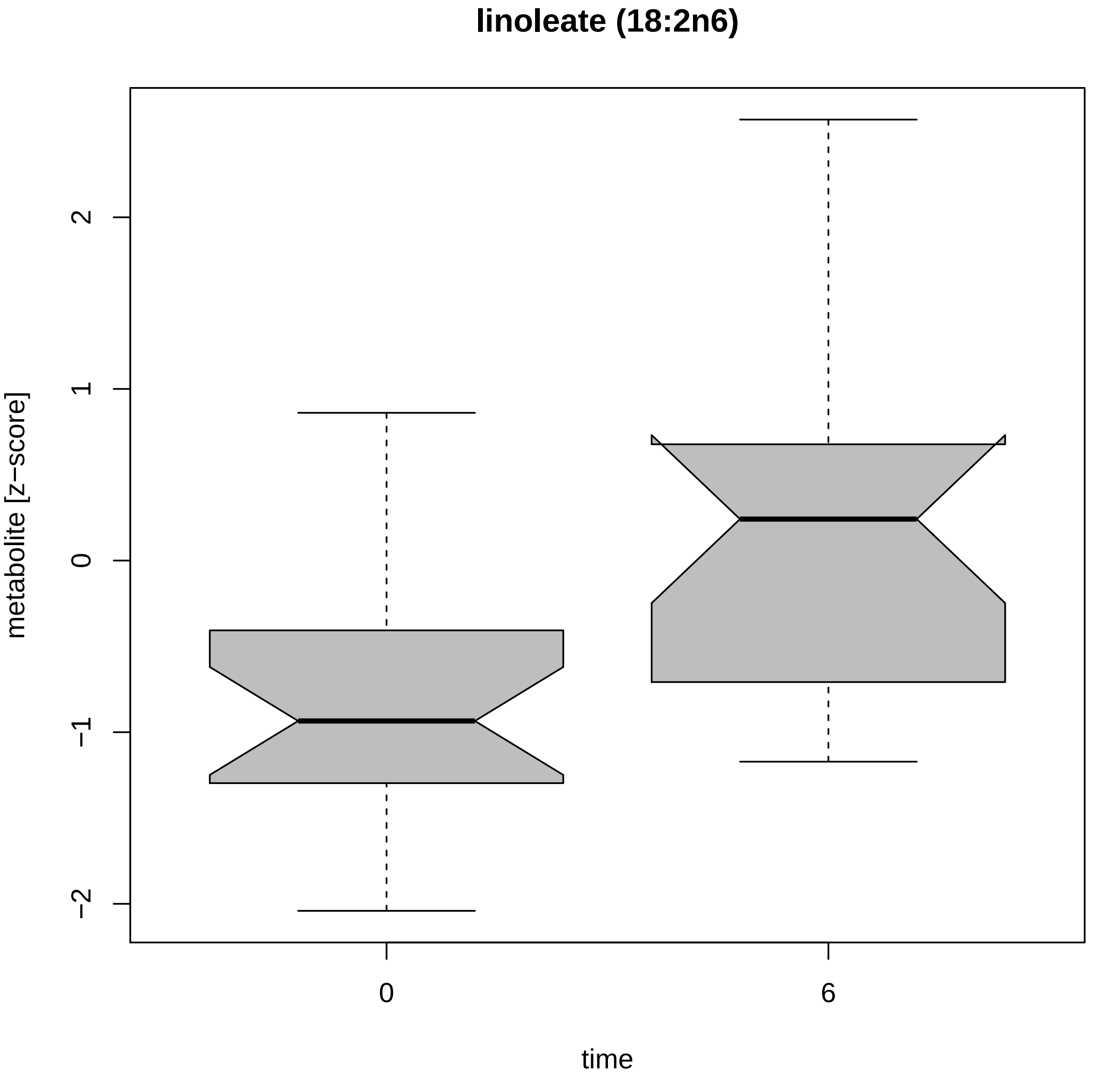
linoleate (18:2n6)
metabolite [z−score]
2
1
0
−1
−2
0
6
time

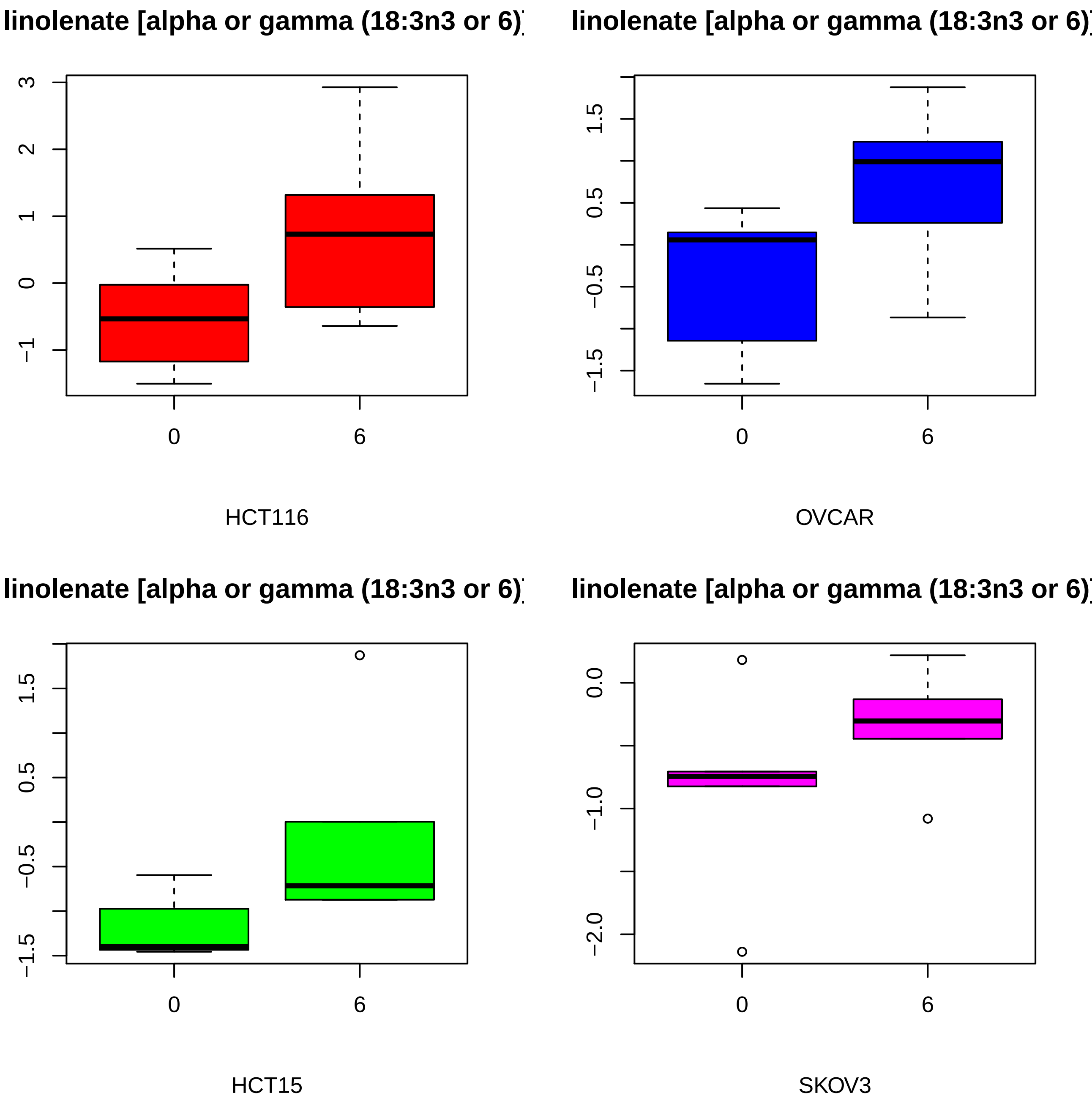
linolenate [alpha or gamma (18:3n3 or 6)]
3
2
1
0
−1
0
6
HCT116
linolenate [alpha or gamma (18:3n3 or 6)]
1.5
0.5
−0.5
−1.5
0
6
OVCAR
linolenate [alpha or gamma (18:3n3 or 6)]
1.5
0.5
−0.5
−1.5
0
6
HCT15
linolenate [alpha or gamma (18:3n3 or 6)]
0.0
−1.0
−2.0
0
6
SKOV3

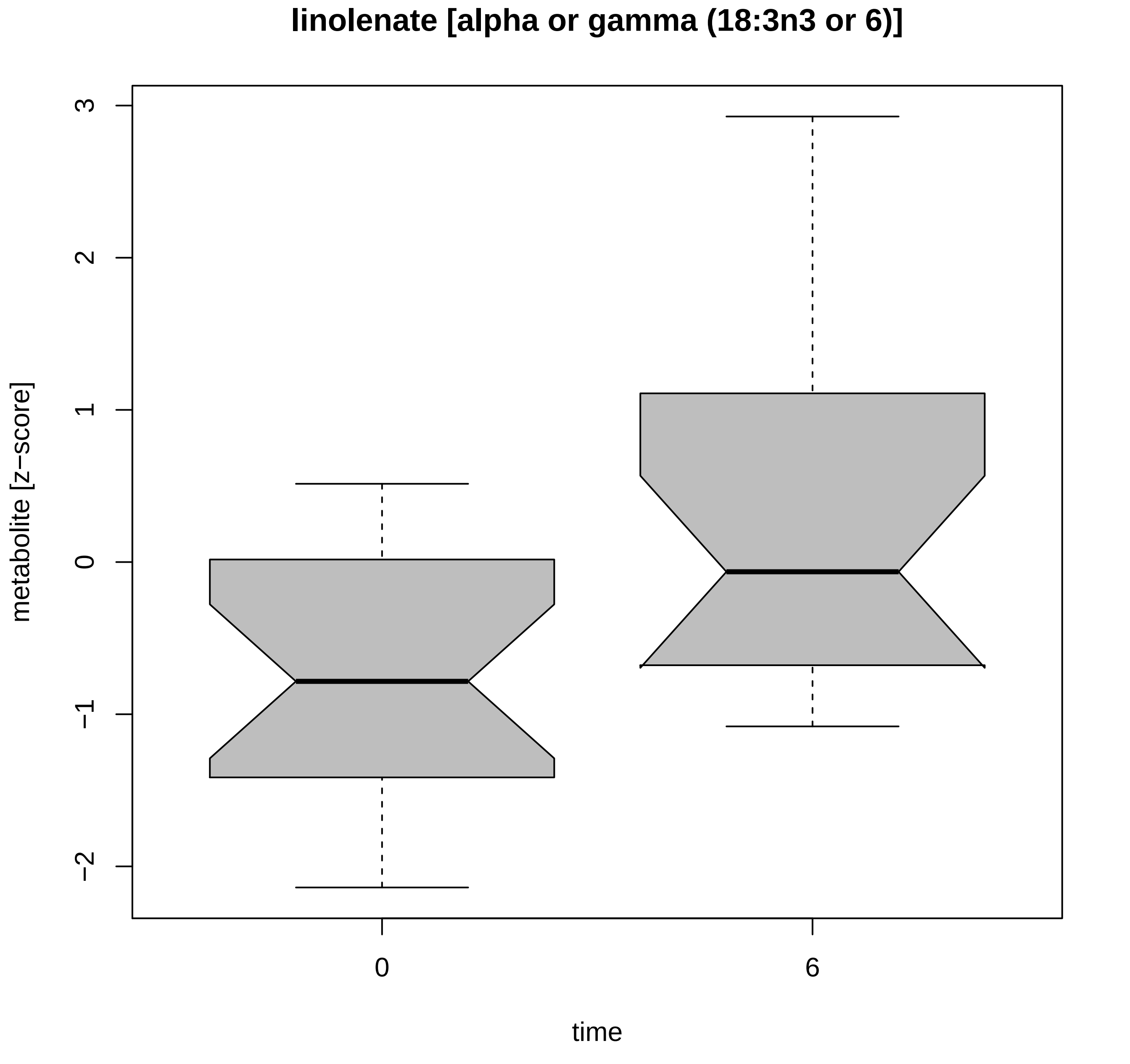

linolenate [alpha or gamma (18:3n3 or 6)]
metabolite [z−score]
3
2
1
0
−1
−2
0
6
time

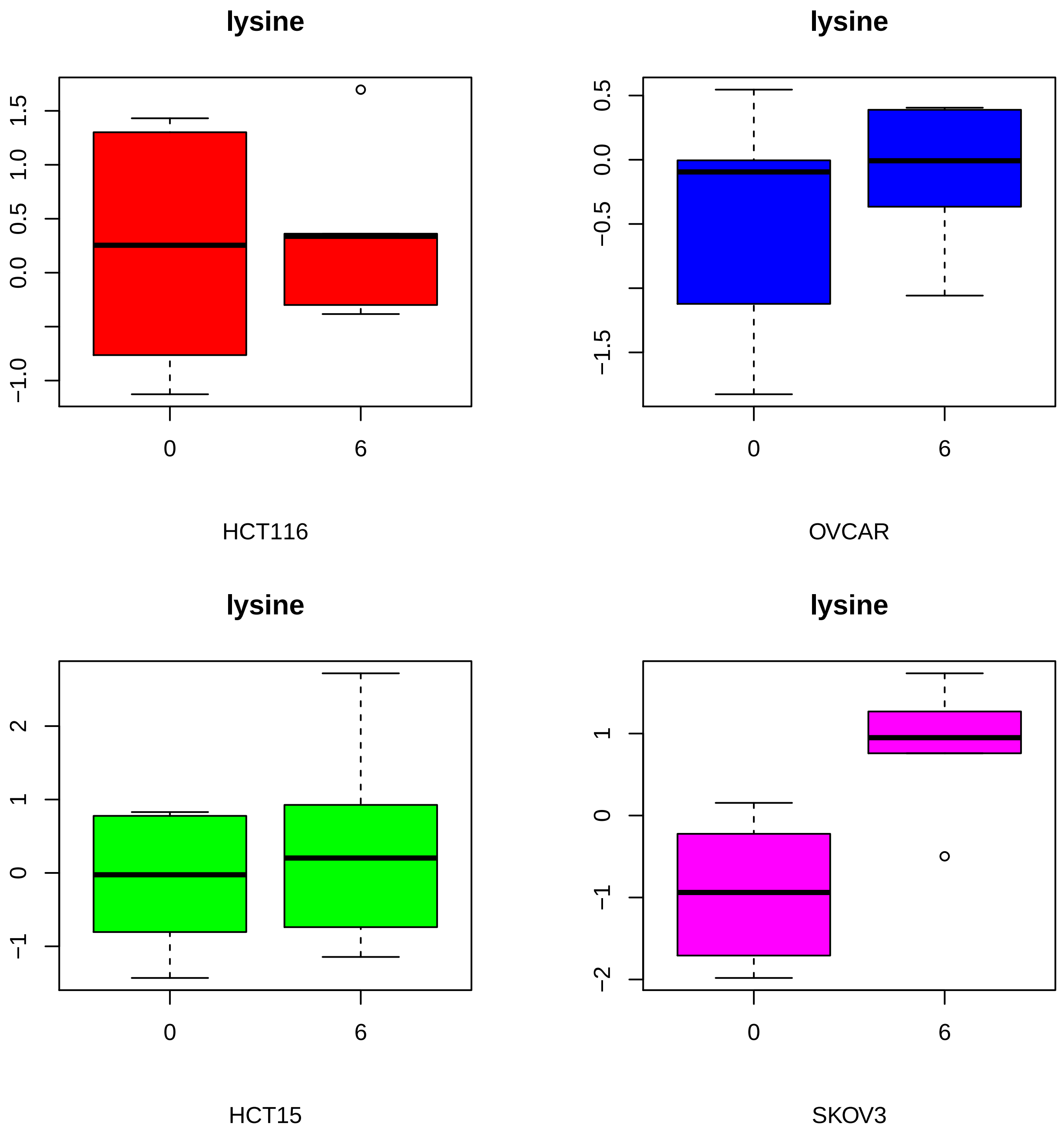
lysine
1.5
1.0
0.5
0.0
−1.0
0
6
HCT116
lysine
0.5
0.0
−0.5
−1.5
0
6
OVCAR
lysine
2
1
0
−1
0
6
HCT15
lysine
1
0
−1
−2
0
6
SKOV3

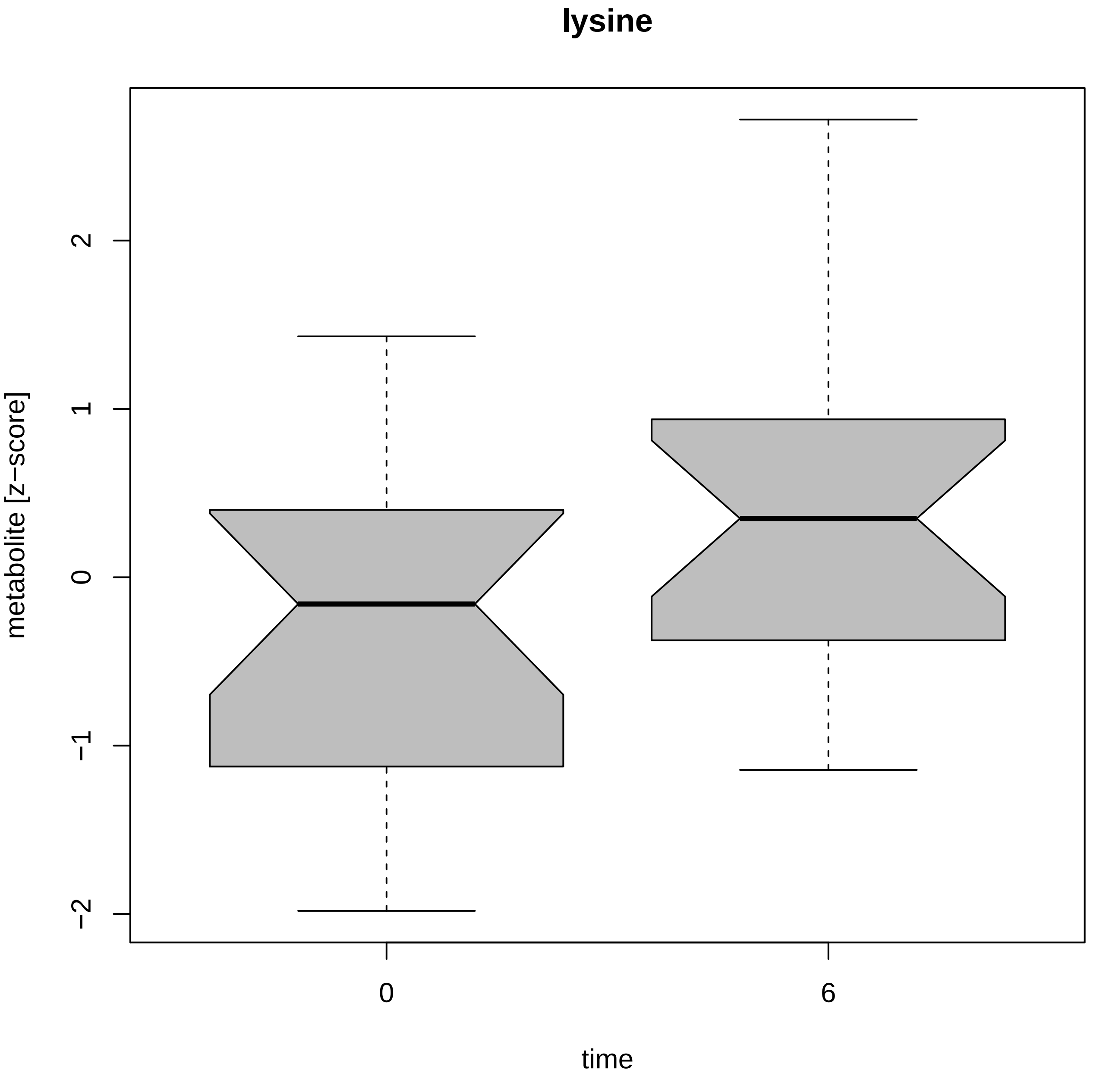

lysine
metabolite [z−score]
2
1
0
−1
−2
0
6
time

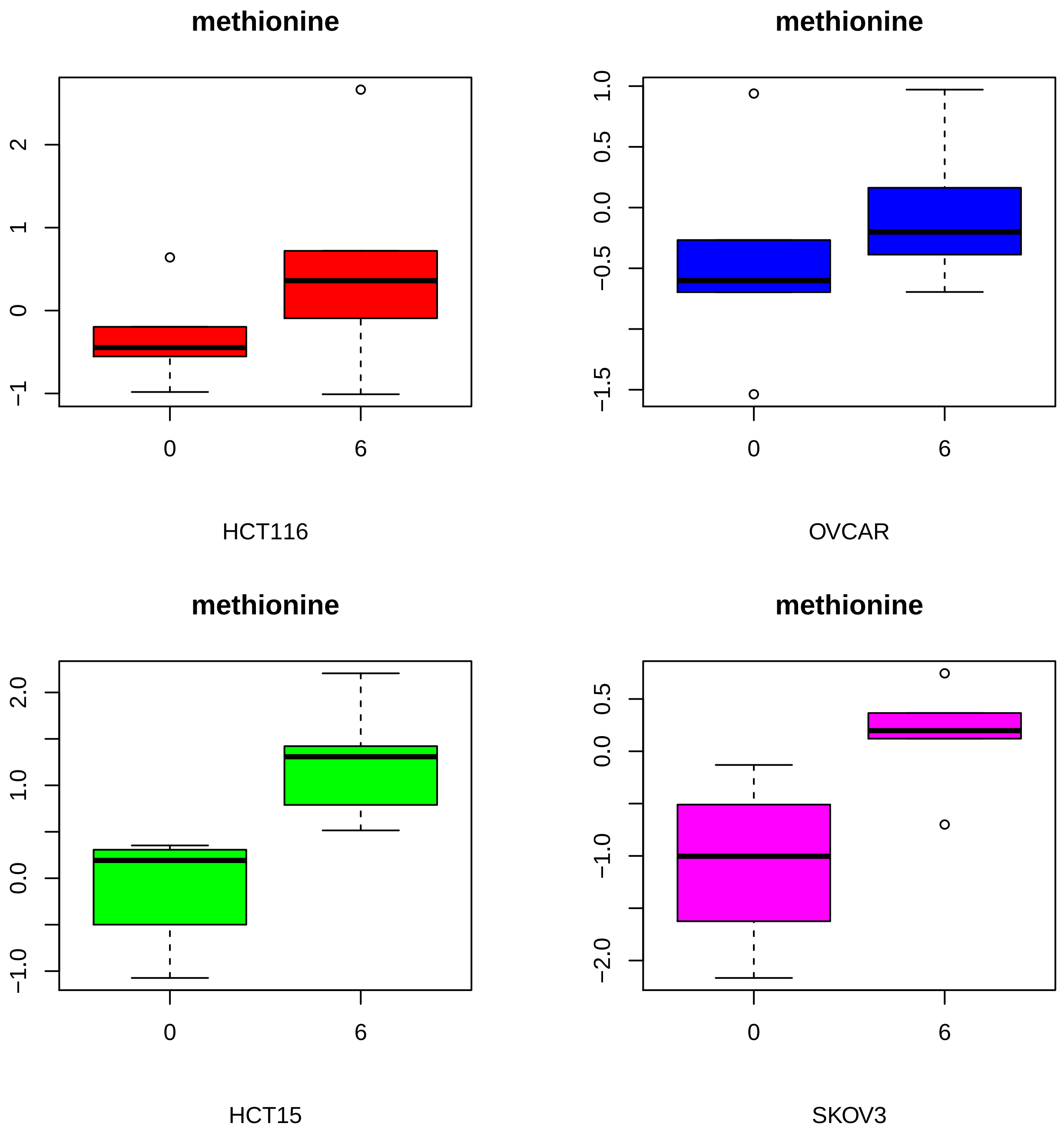
methionine
2
1
0
−1
0
6
HCT116
methionine
1.0
0.5
0.0
−0.5
−1.5
0
6
OVCAR
methionine
2.0
1.0
0.0
−1.0
0
6
HCT15
methionine
0.5
0.0
−1.0
−2.0
0
6
SKOV3

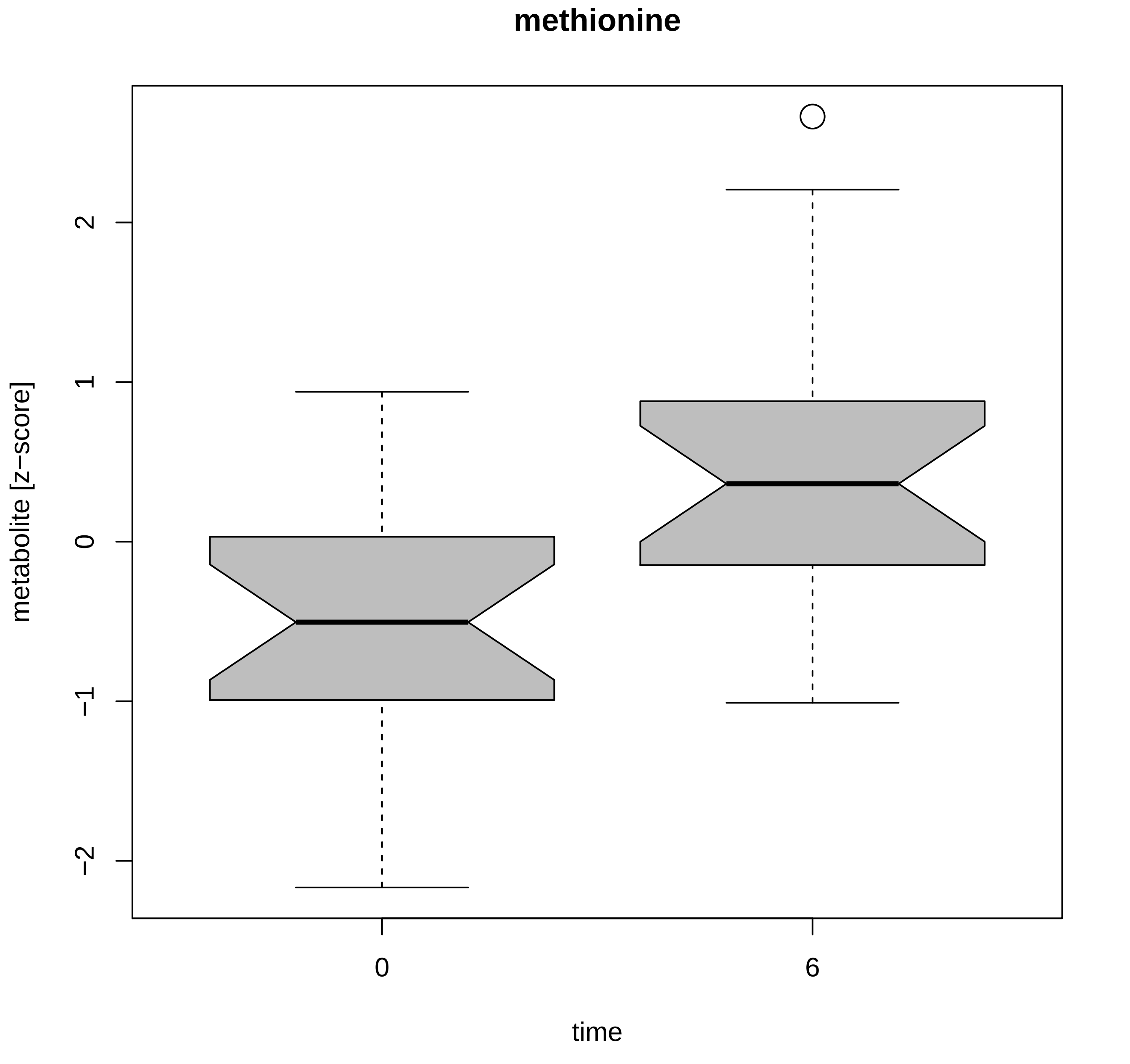
methionine
metabolite [z−score]
2
1
0
−1
−2
0
6
time

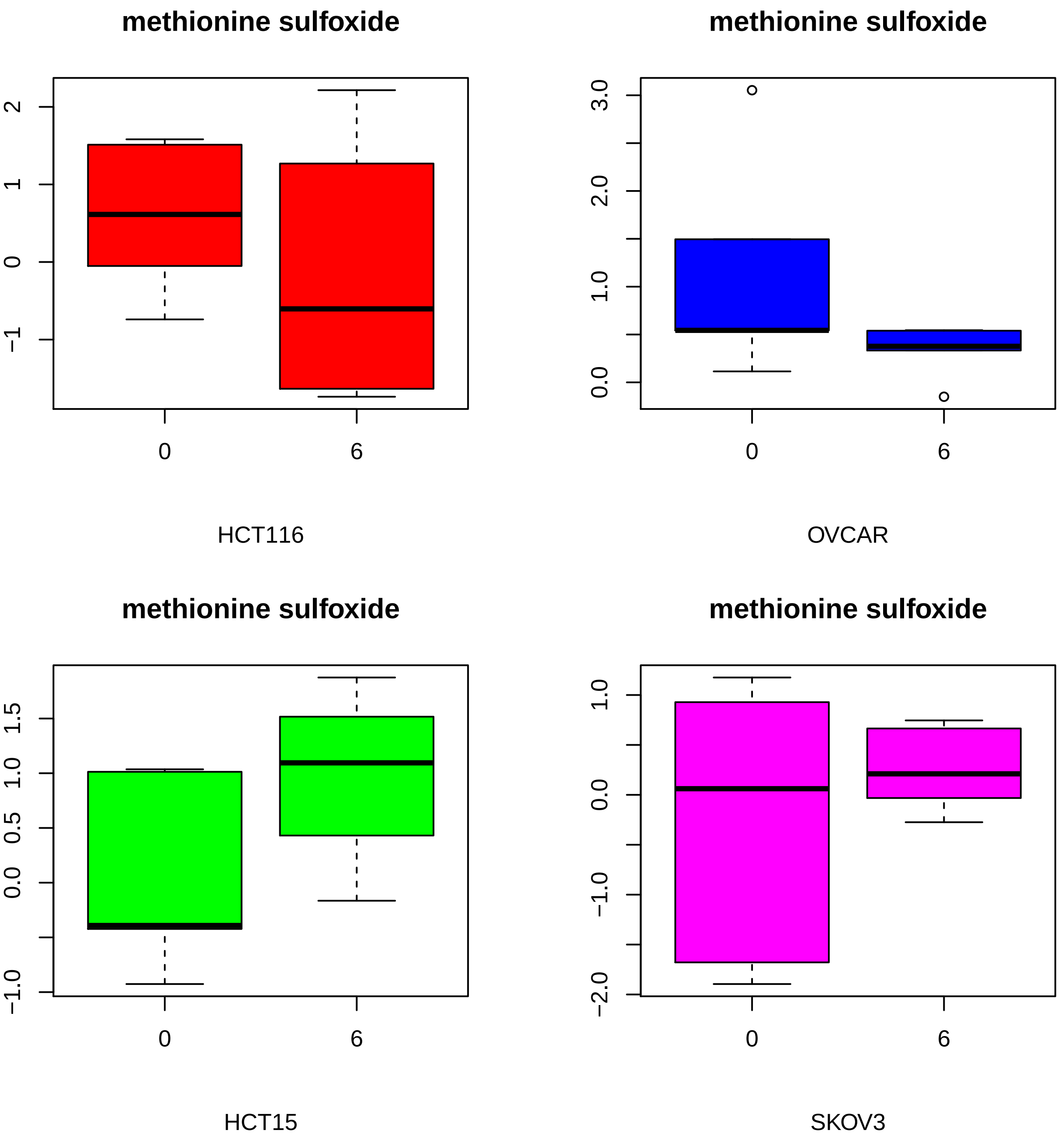
methionine sulfoxide
2
1
0
−1
0
6
HCT116
methionine sulfoxide
3.0
2.0
1.0
0.0
0
6
OVCAR
methionine sulfoxide
1.5
1.0
0.5
0.0
−1.0
0
6
HCT15
methionine sulfoxide
1.0
0.0
−1.0
−2.0
0
6
SKOV3

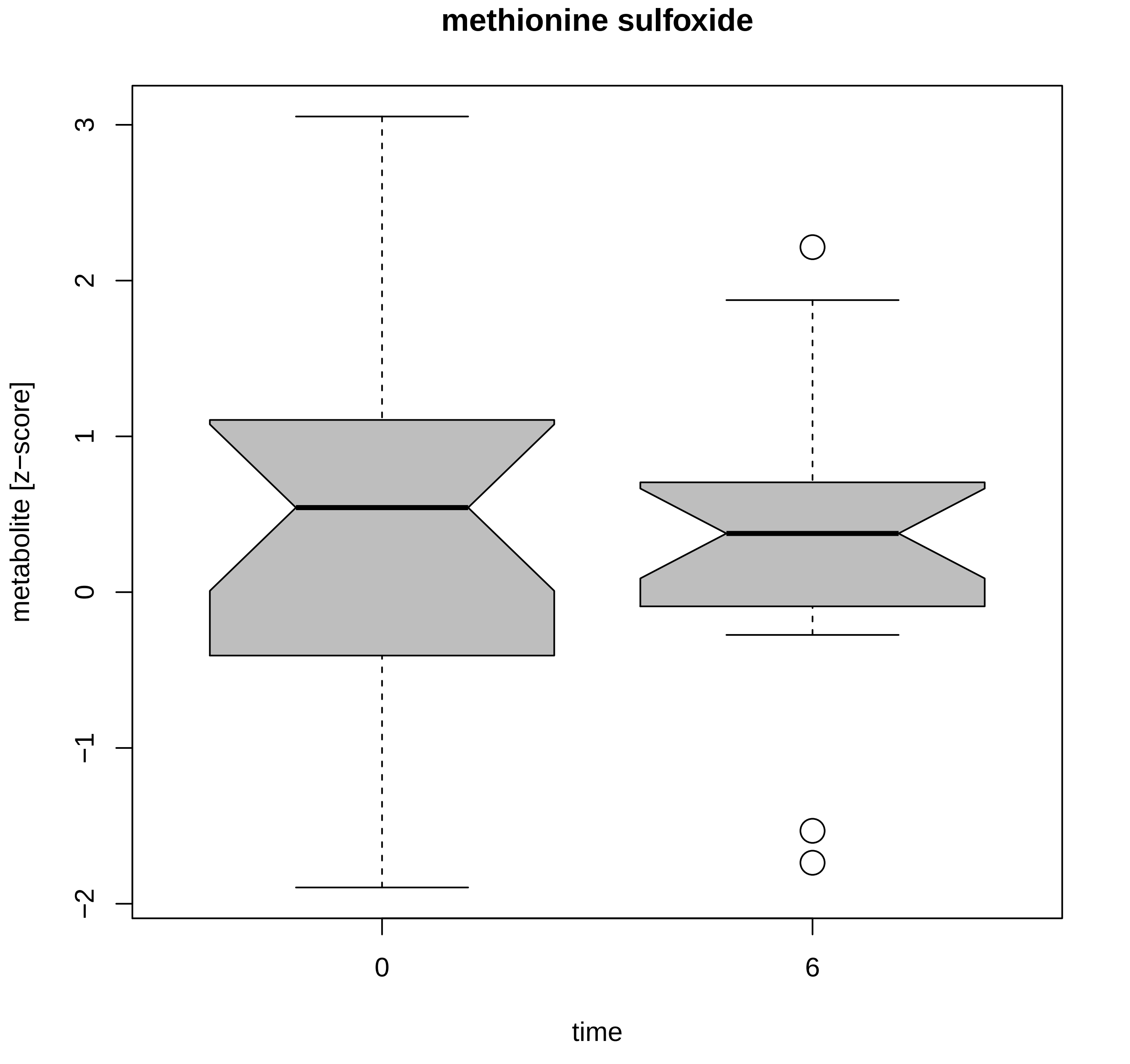
methionine sulfoxide
metabolite [z−score]
3
2
1
0
−1
−2
0
6
time

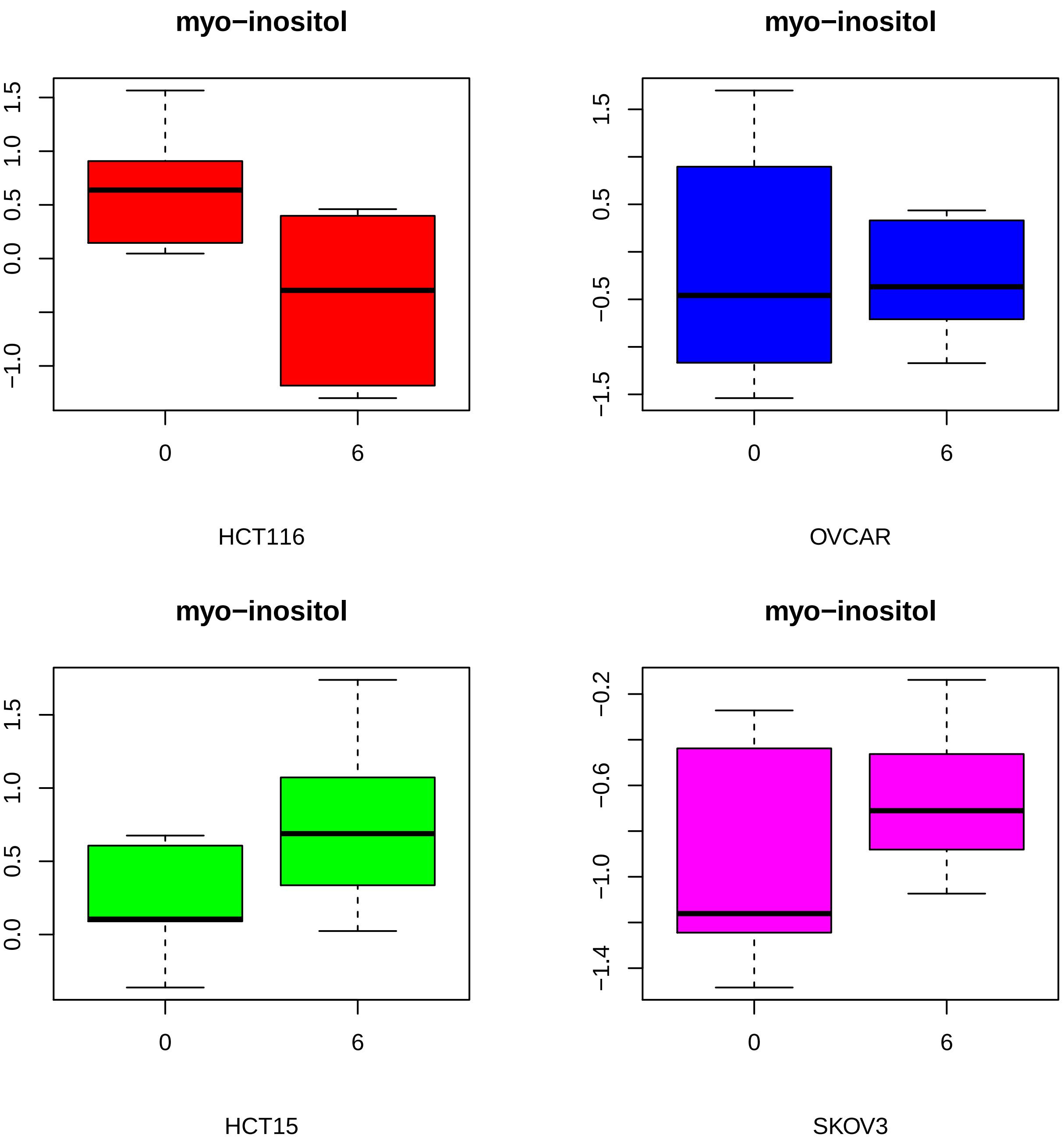
myo−inositol
1.5
1.0
0.5
0.0
−1.0
0
6
HCT116
myo−inositol
1.5
0.5
−0.5
−1.5
0
6
OVCAR
myo−inositol
1.5
1.0
0.5
0.0
0
6
HCT15
myo−inositol
−0.2
−0.6
−1.0
−1.4
0
6
SKOV3

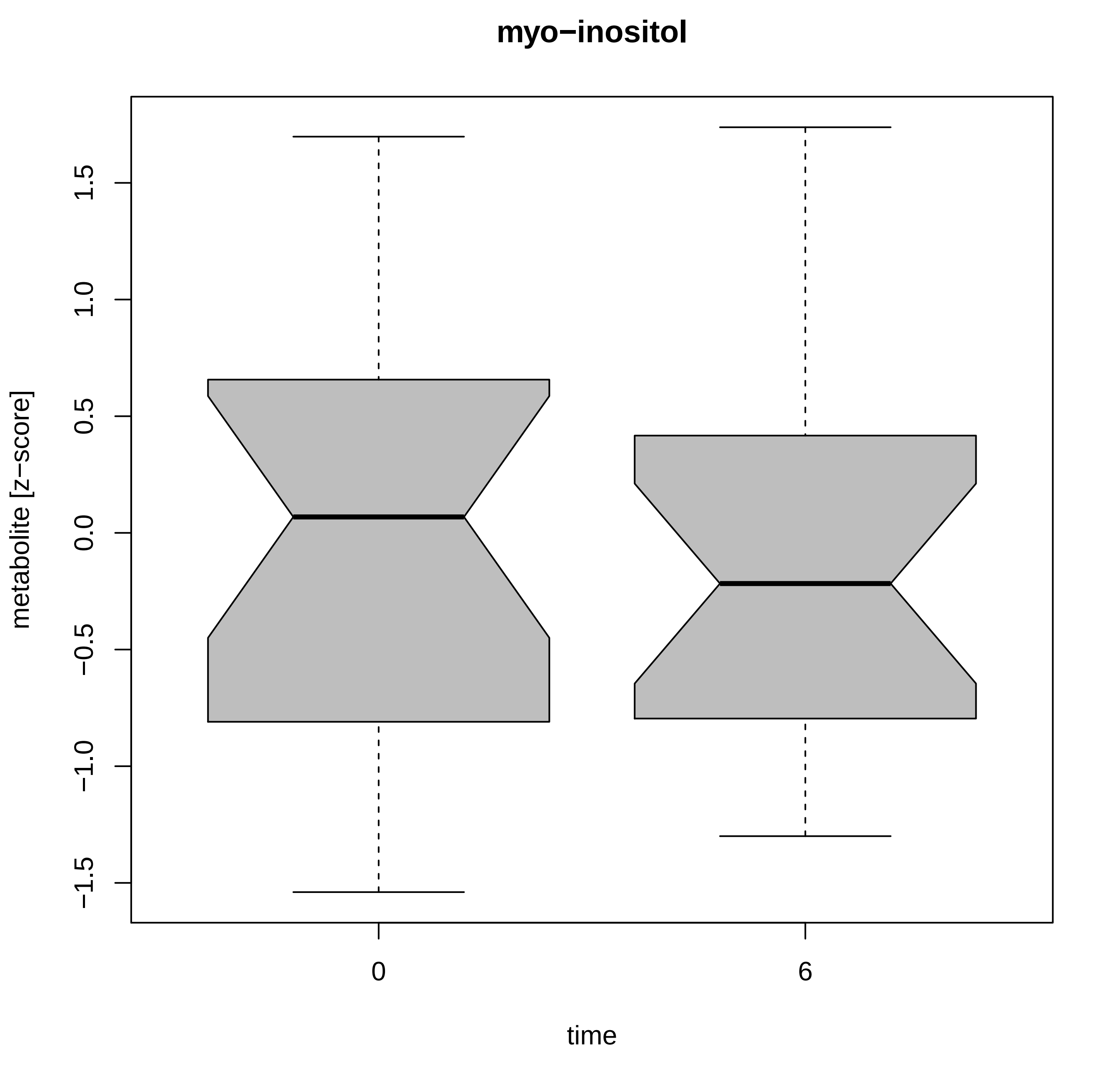

myo−inositol
1.5
1.0
0.5
0.0
−0.5
−1.0
−1.5
metabolite [z−score]
0
6
time

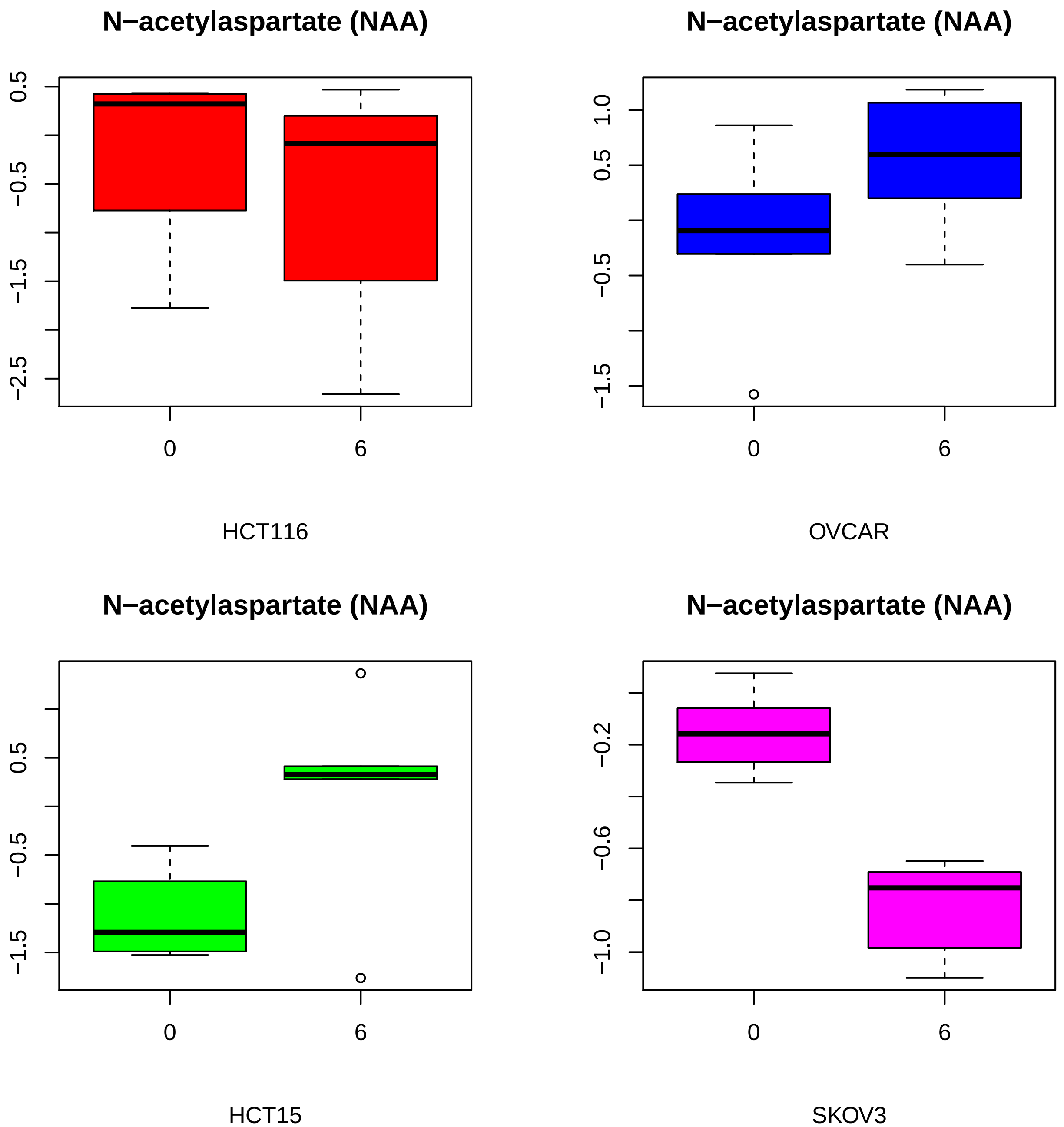
N−acetylaspartate (NAA)
0.5
−0.5
−1.5
−2.5
0
6
HCT116
N−acetylaspartate (NAA)
1.0
0.5
−0.5
−1.5
0
6
OVCAR
N−acetylaspartate (NAA)
0.5
−0.5
−1.5
0
6
HCT15
N−acetylaspartate (NAA)
−0.2
−0.6
−1.0
0
6
SKOV3

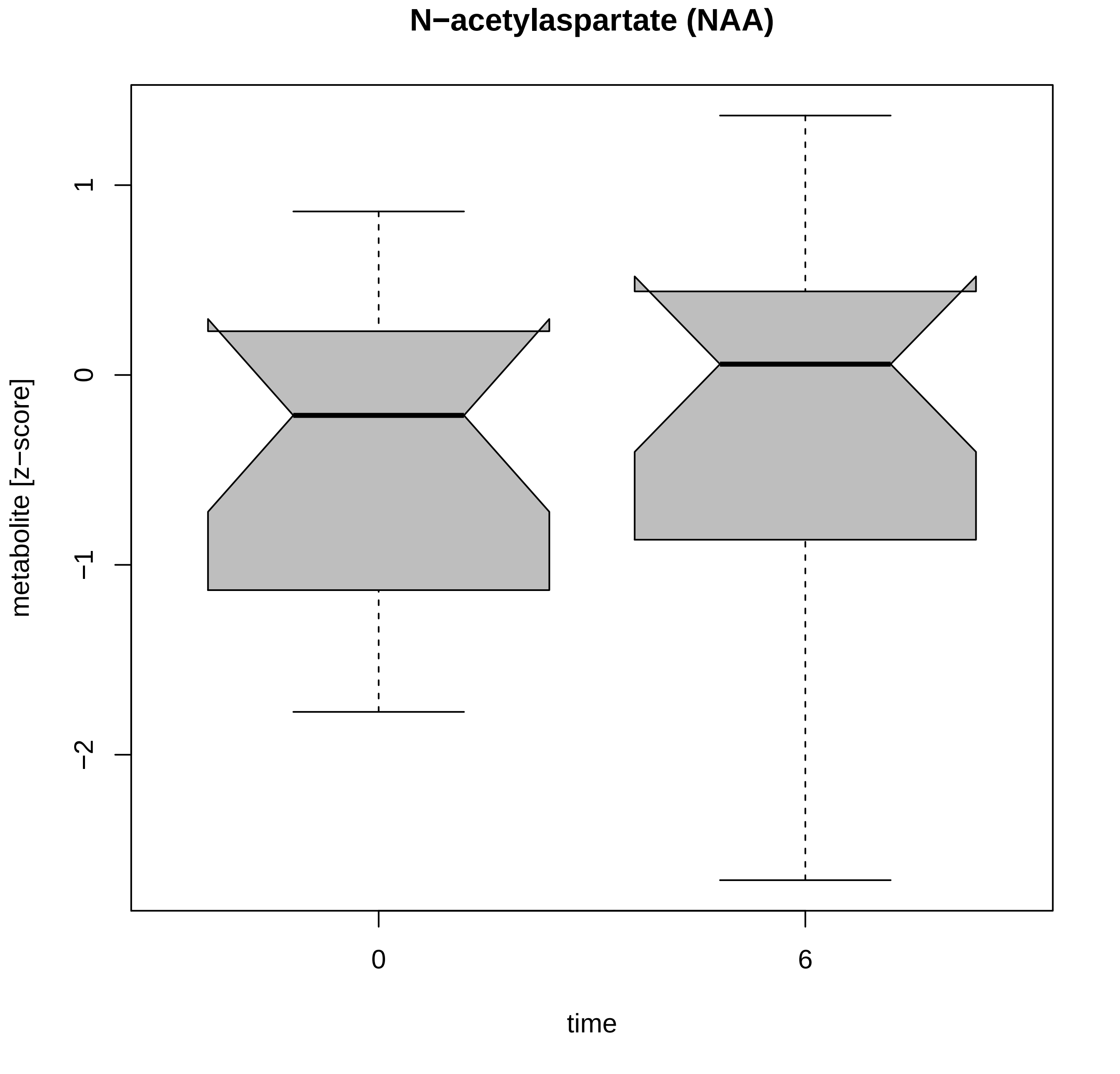
N−acetylaspartate (NAA)
metabolite [z−score]
−2
−1
0
1
0
6
time

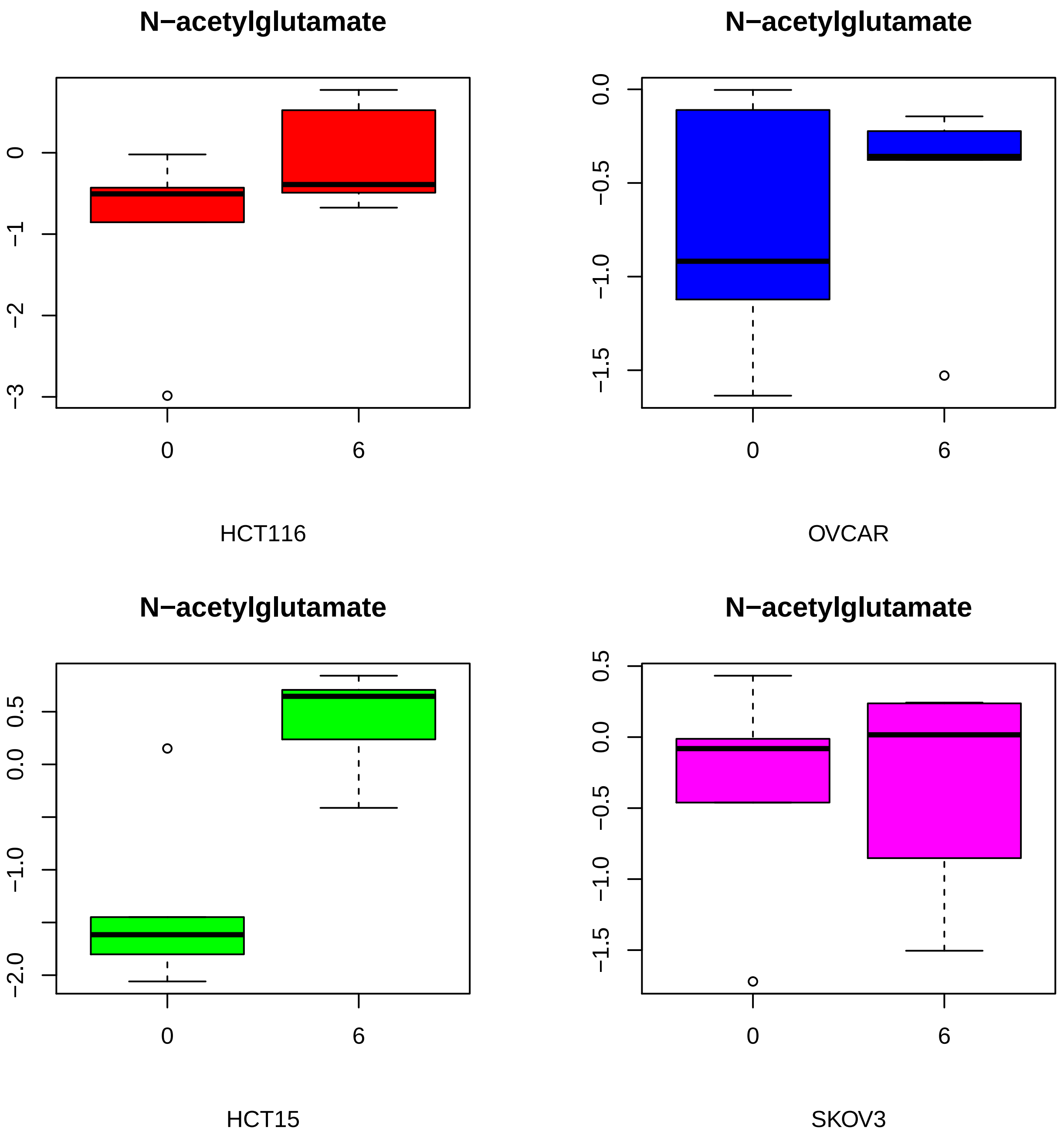
N−acetylglutamate
0
−1
−2
−3
0
6
HCT116
N−acetylglutamate
0.0
−0.5
−1.0
−1.5
0
6
OVCAR
N−acetylglutamate
0.5
0.0
−1.0
−2.0
0
6
HCT15
N−acetylglutamate
0.5
0.0
−0.5
−1.0
−1.5
0
6
SKOV3

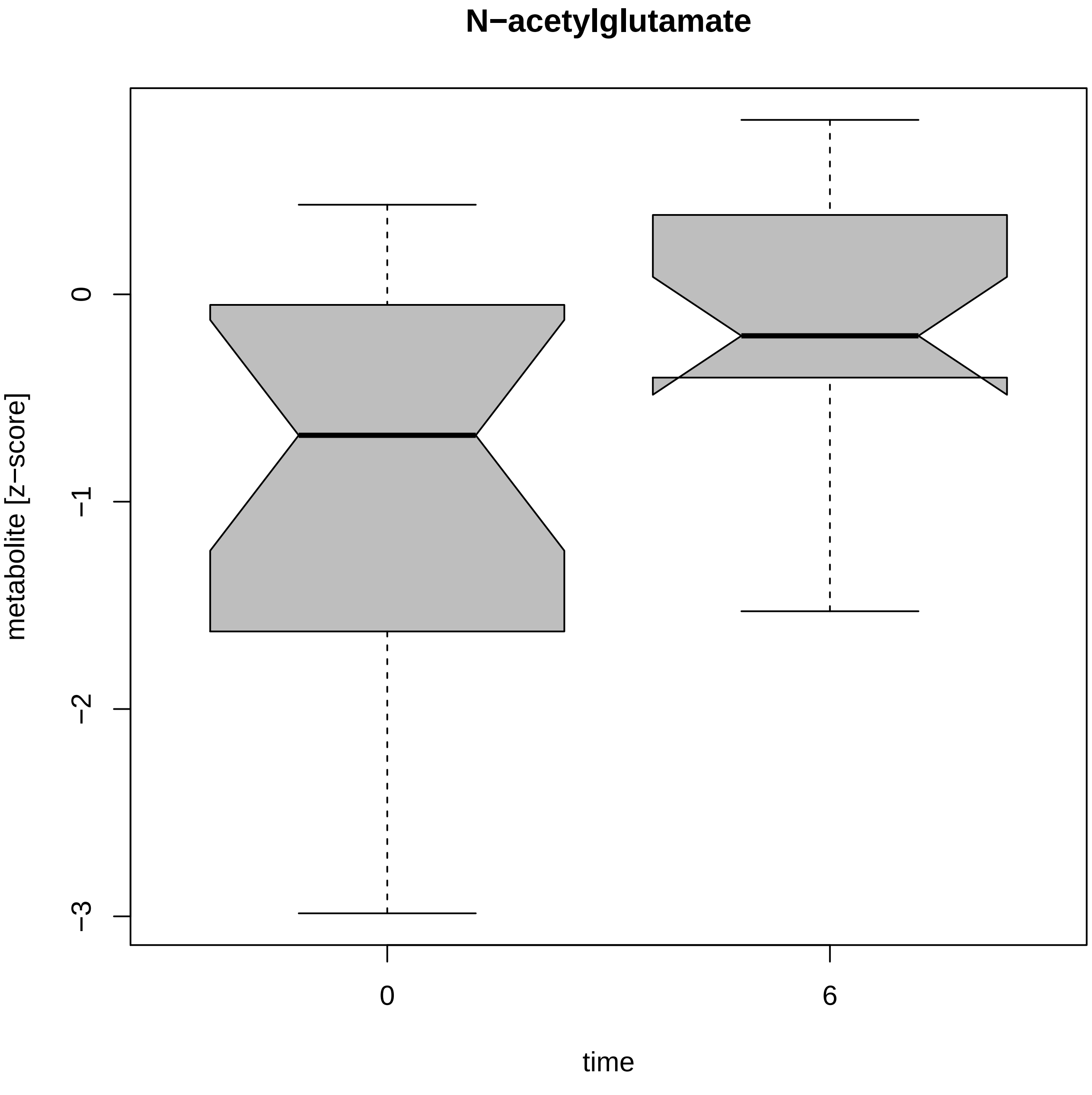
N−acetylglutamate
metabolite [z−score]
0
−1
−2
−3
0
6
time

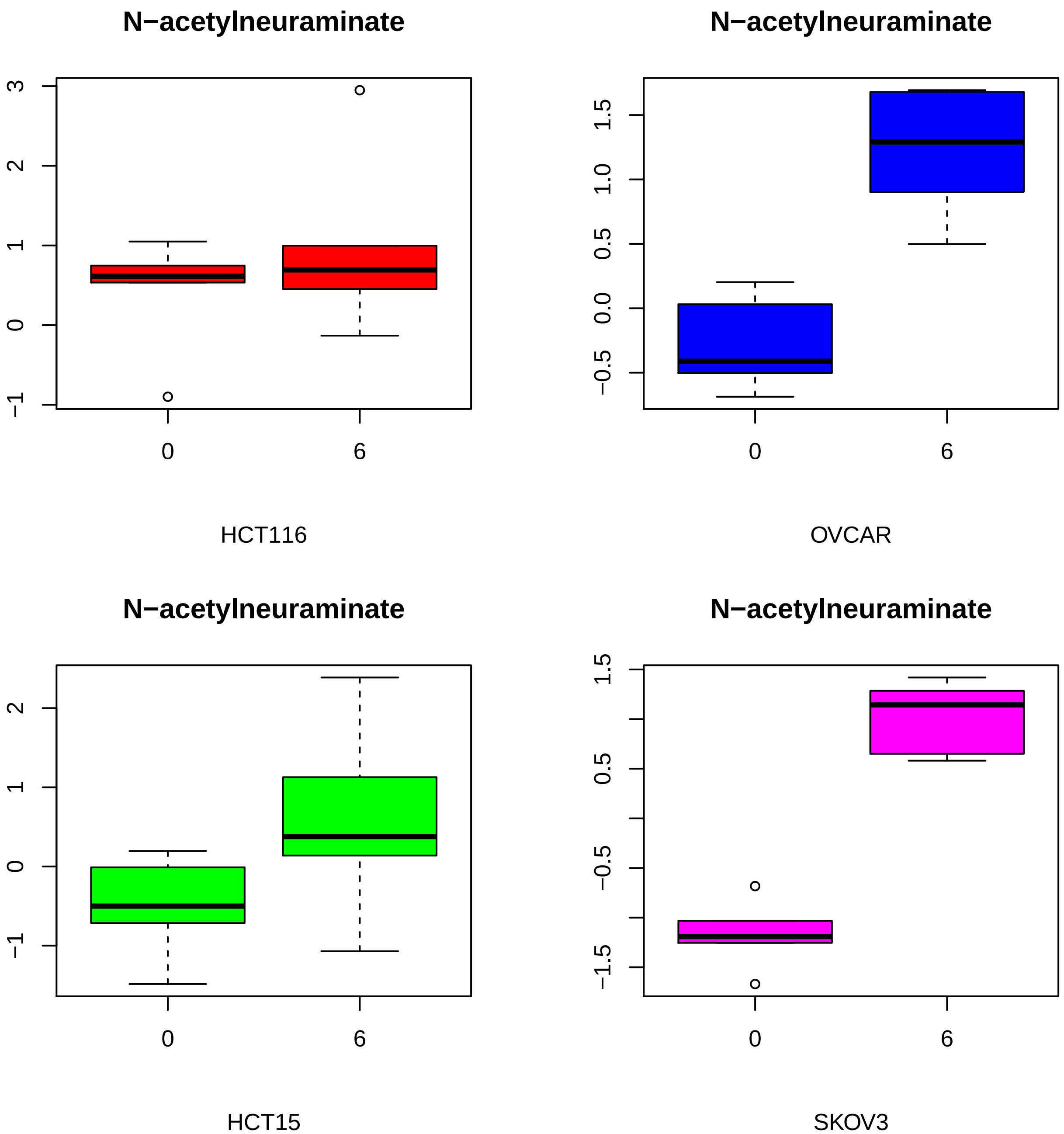
N−acetylneuraminate
3
2
1
0
−1
0
6
HCT116
N−acetylneuraminate
1.5
1.0
0.5
0.0
−0.5
0
6
OVCAR
N−acetylneuraminate
2
1
0
−1
0
6
HCT15
N−acetylneuraminate
1.5
0.5
−0.5
−1.5
0
6
SKOV3

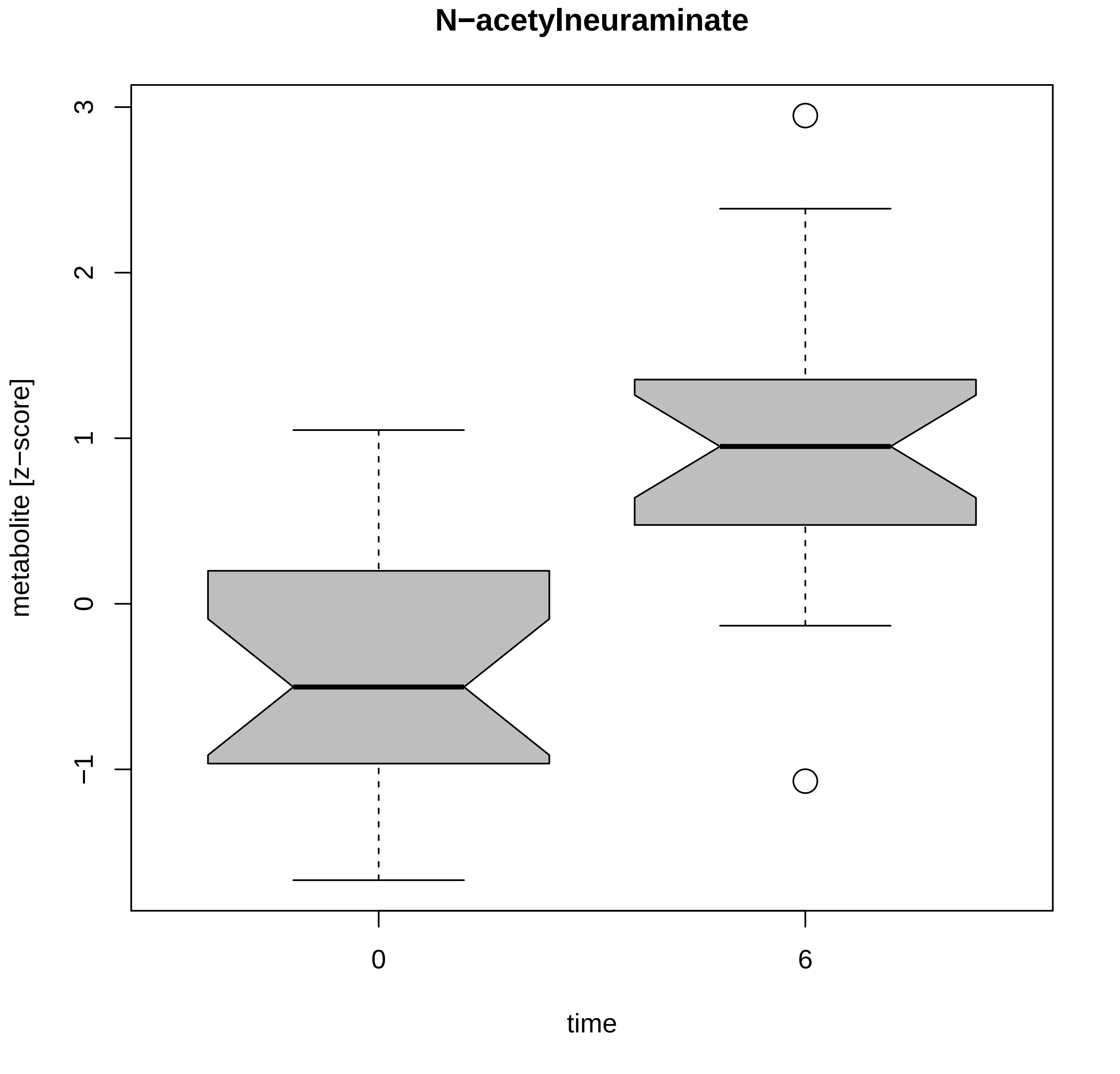

N−acetylneuraminate
metabolite [z−score]
3
2
1
0
−1
0
6
time

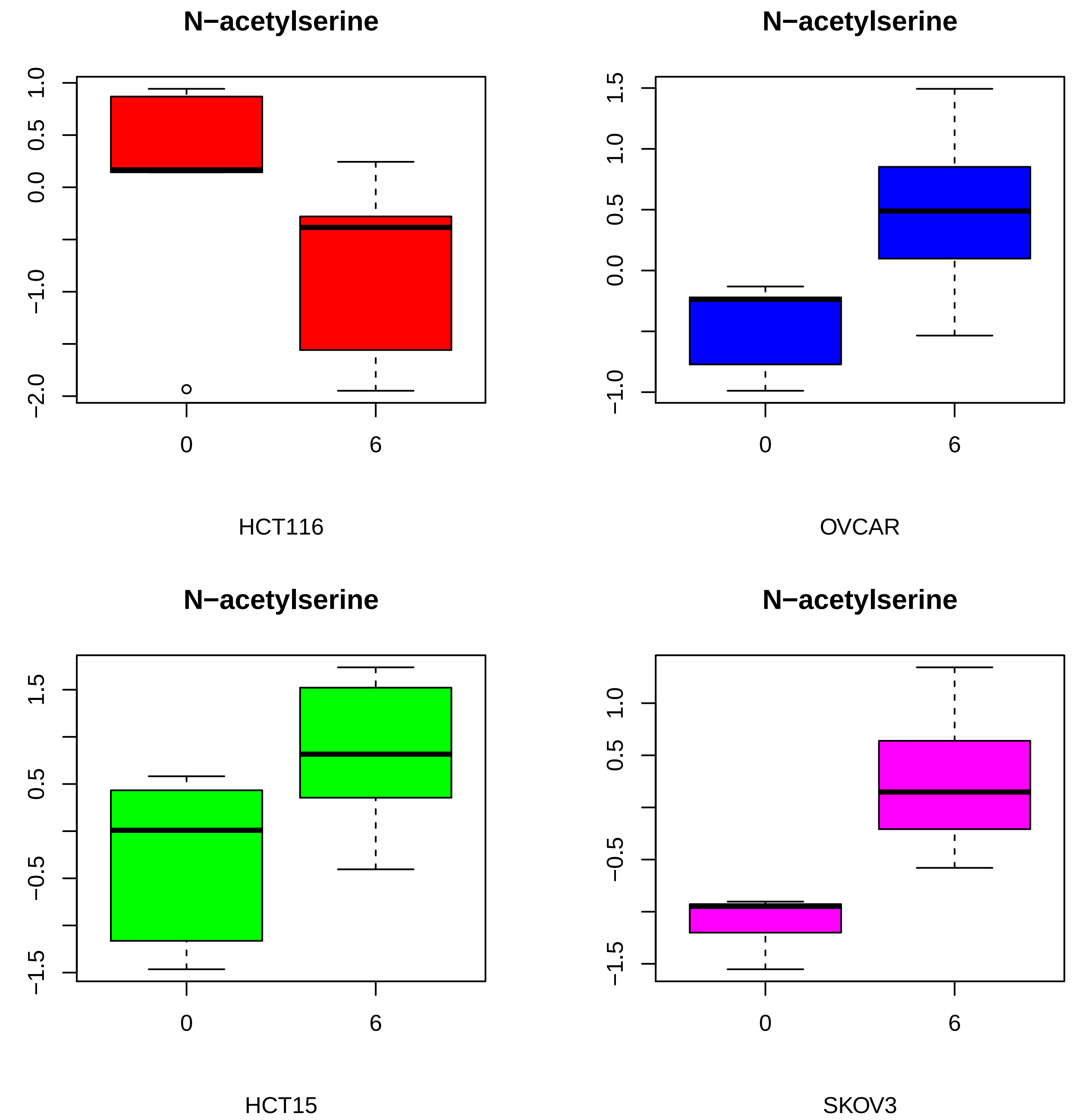
N−acetylserine
1.0
0.5
0.0
−1.0
−2.0
0
6
HCT116
N−acetylserine
1.5
1.0
0.5
0.0
−1.0
0
6
OVCAR
N−acetylserine
1.5
0.5
−0.5
−1.5
0
6
HCT15
N−acetylserine
1.0
0.5
−0.5
−1.5
0
6
SKOV3

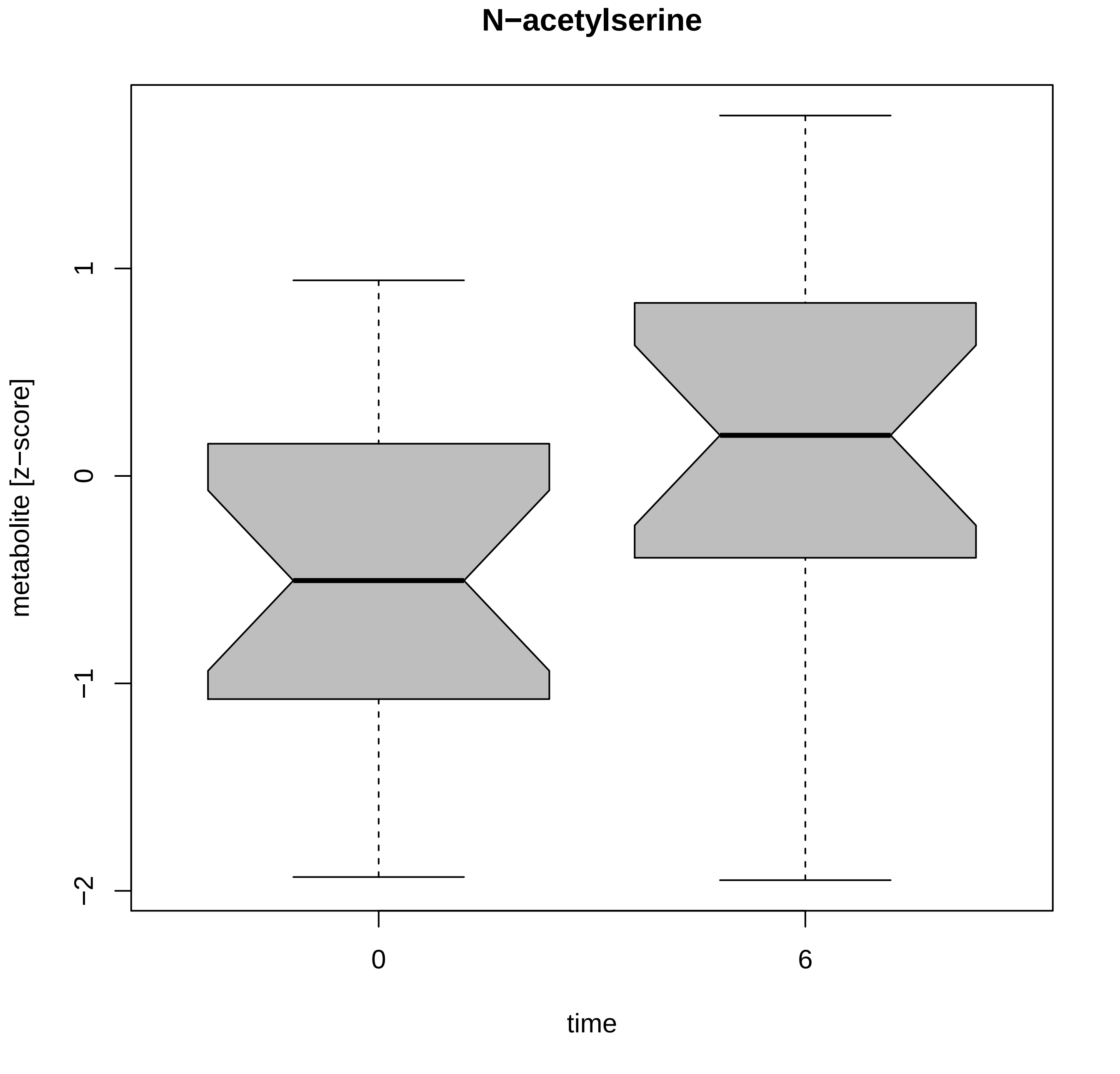
N−acetylserine
metabolite [z−score]
1
0
−1
−2
0
6
time

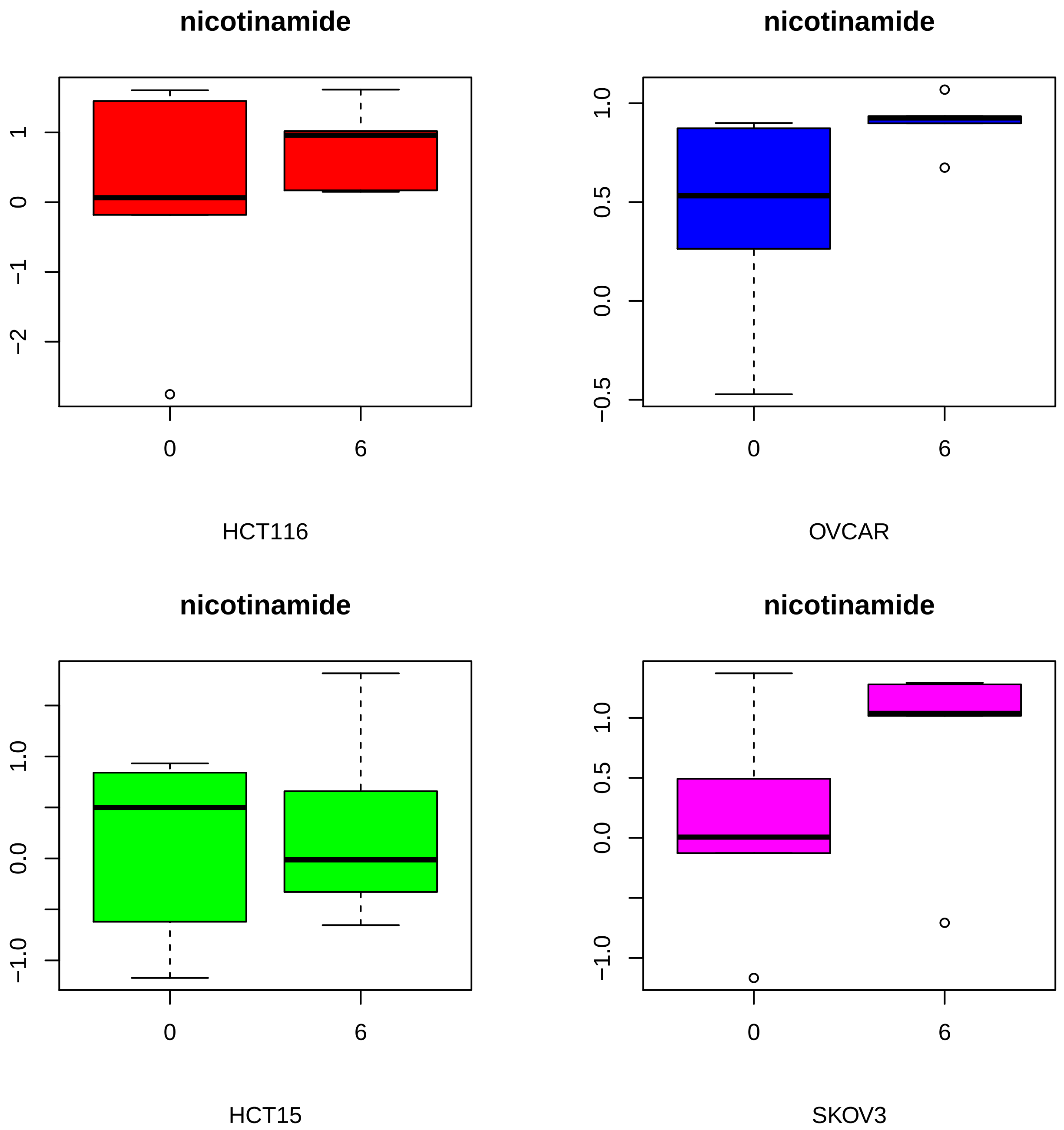
nicotinamide
1
0
−1
−2
0
6
HCT116
nicotinamide
1.0
0.5
0.0
−0.5
0
6
OVCAR
nicotinamide
1.0
0.0
−1.0
0
6
HCT15
nicotinamide
1.0
0.5
0.0
−1.0
0
6
SKOV3

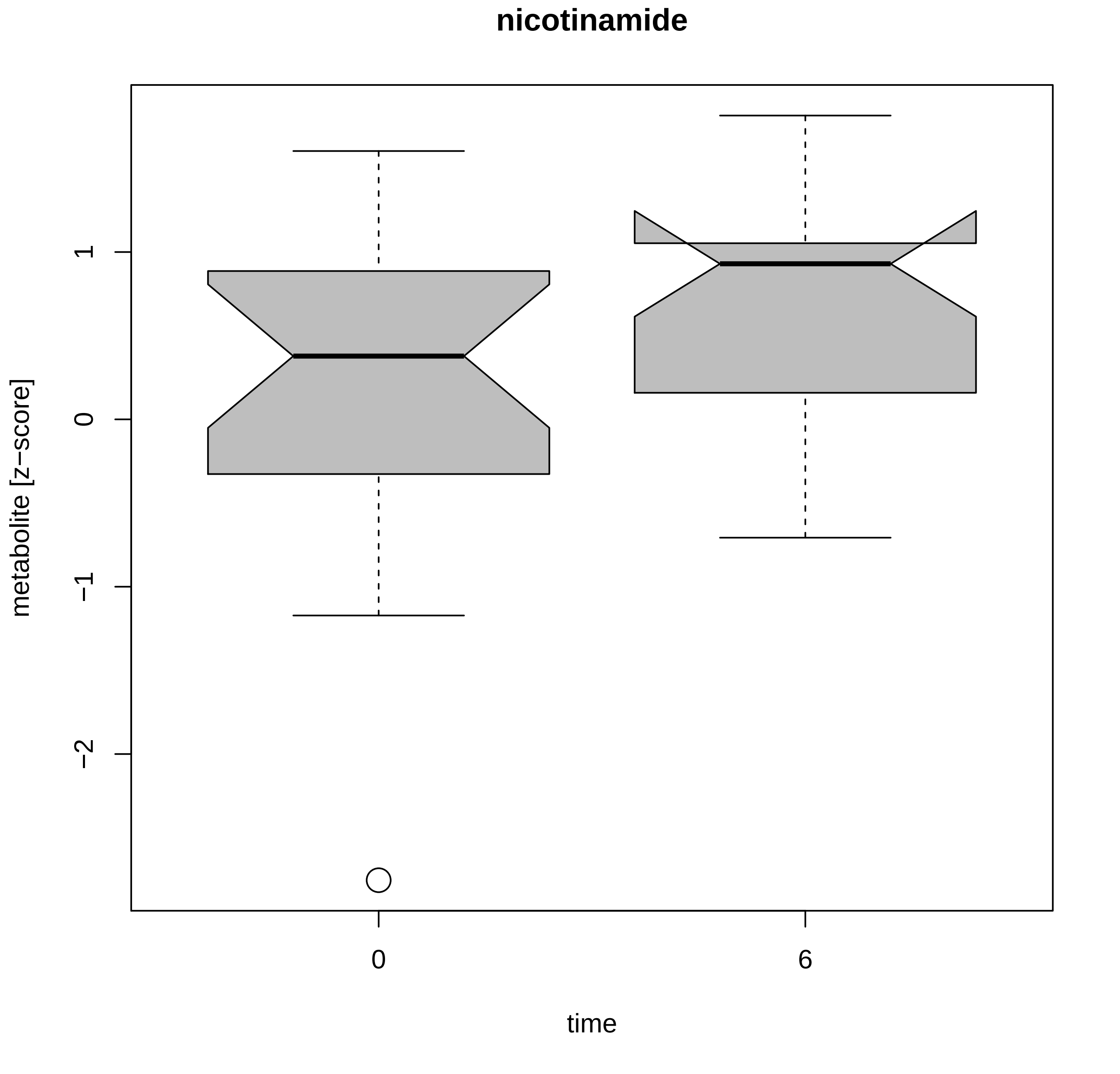
nicotinamide
metabolite [z−score]
1
0
−1
−2
0
6
time

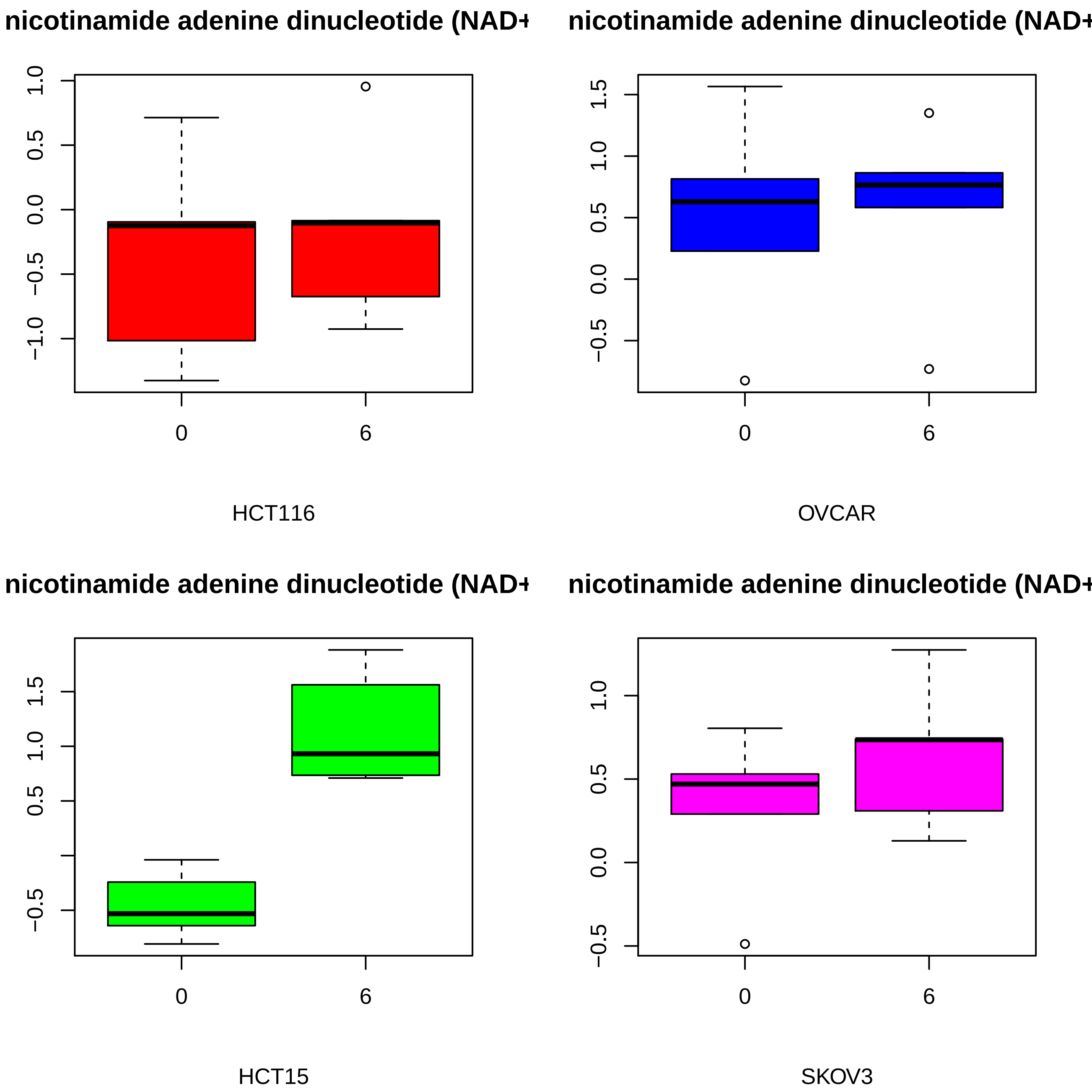
nicotinamide adenine dinucleotide (NAD+
1.0
0.5
0.0
−0.5
−1.0
0
6
HCT116
nicotinamide adenine dinucleotide (NAD+
1.5
1.0
0.5
0.0
−0.5
0
6
OVCAR
nicotinamide adenine dinucleotide (NAD+
1.5
1.0
0.5
−0.5
0
6
HCT15
nicotinamide adenine dinucleotide (NAD+
1.0
0.5
0.0
−0.5
0
6
SKOV3

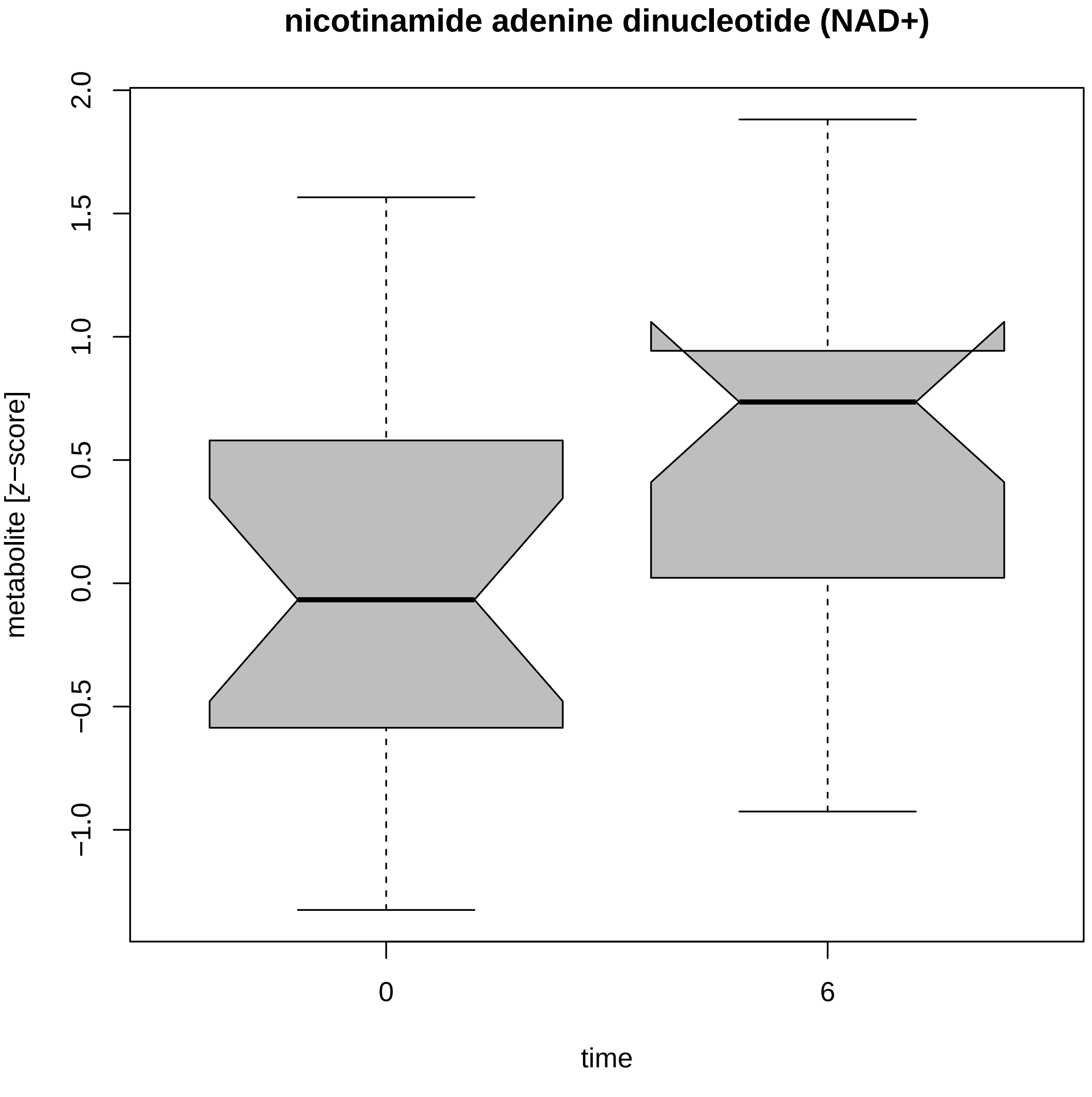
nicotinamide adenine dinucleotide (NAD+)
metabolite [z−score]
2.0
1.5
1.0
0.5
0.0
−0.5
−1.0
0
6
time

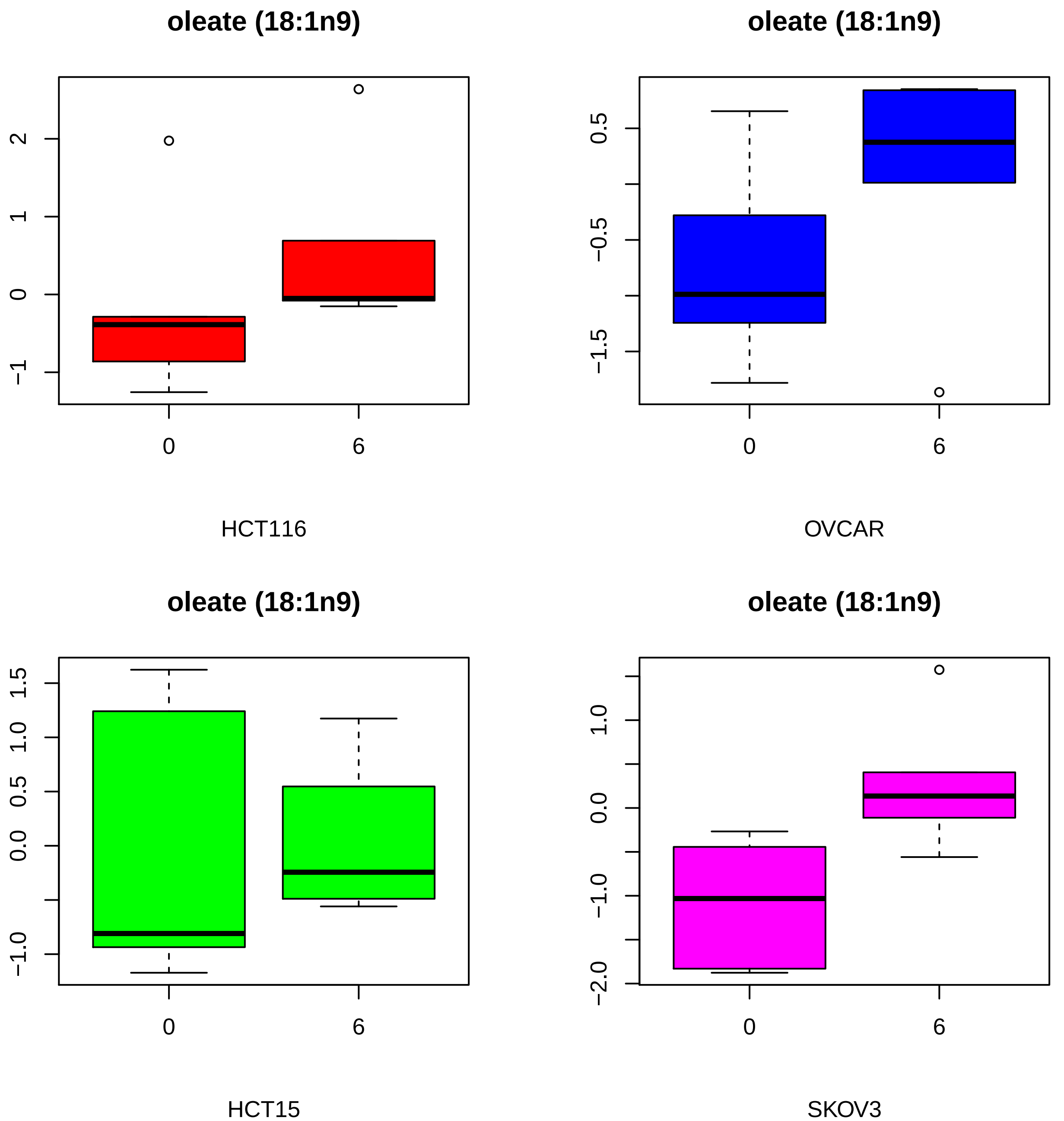
oleate (18:1n9)
HCT116
oleate (18:1n9)
OVCAR
oleate (18:1n9)
HCT15
oleate (18:1n9)
SKOV3

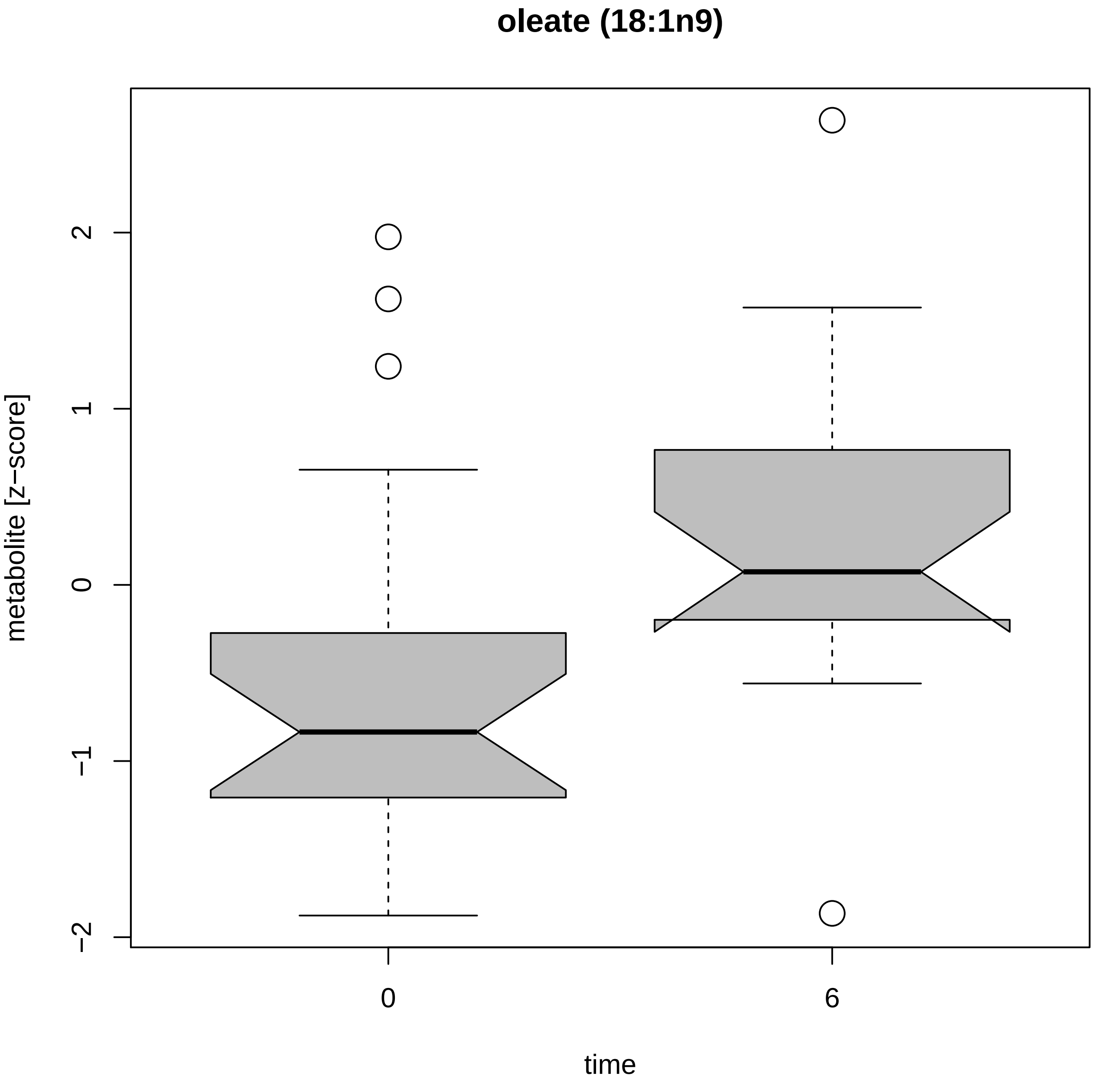

oleate (18:1n9)
metabolite [z−score]
2
1
0
−1
−2
0
6
time

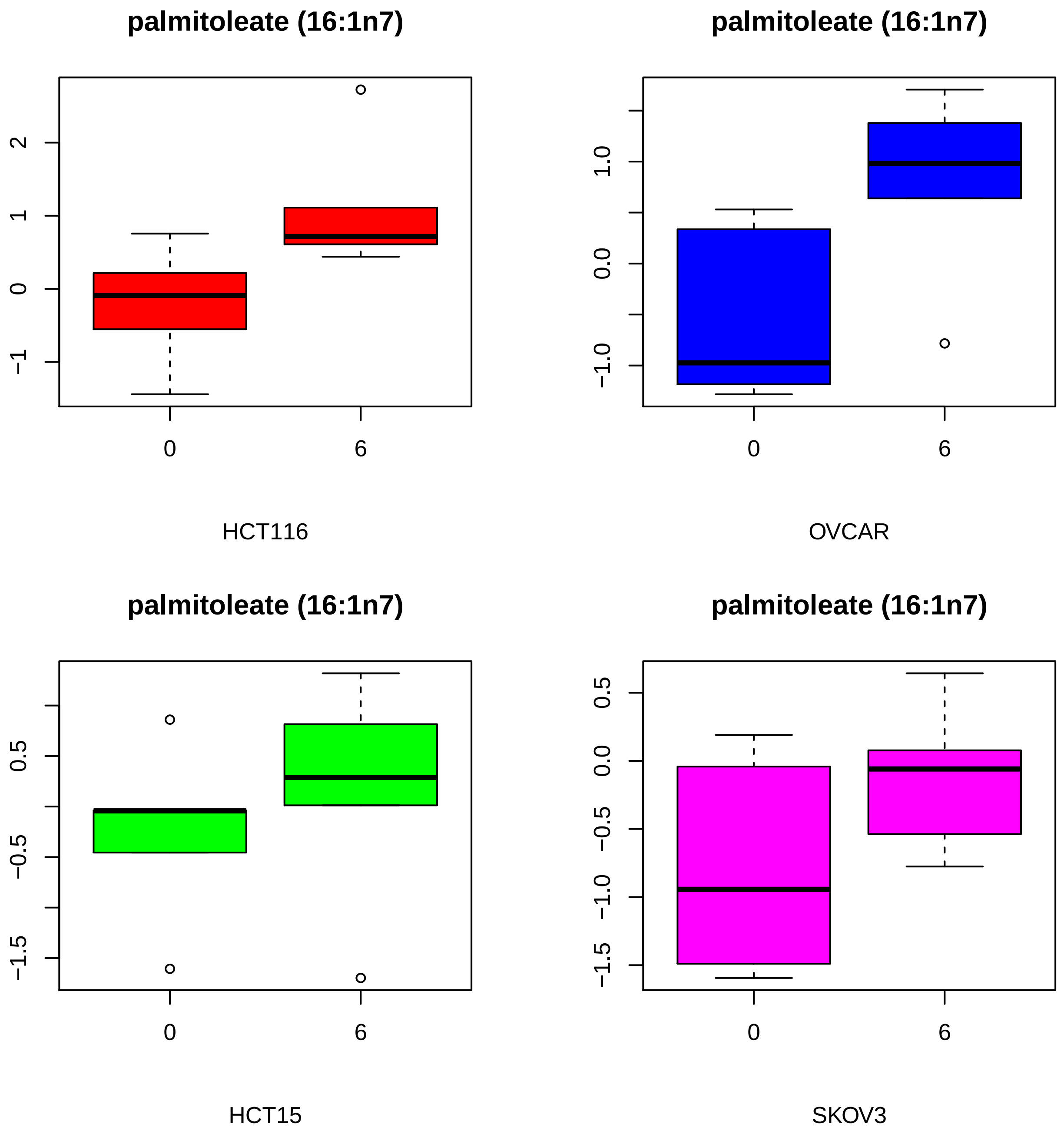
palmitoleate (16:1n7)
2
1
0
−1
0
6
HCT116
palmitoleate (16:1n7)
1.0
0.0
−1.0
0
6
OVCAR
palmitoleate (16:1n7)
0.5
−0.5
−1.5
0
6
HCT15
palmitoleate (16:1n7)
0.5
0.0
−0.5
−1.0
−1.5
0
6
SKOV3

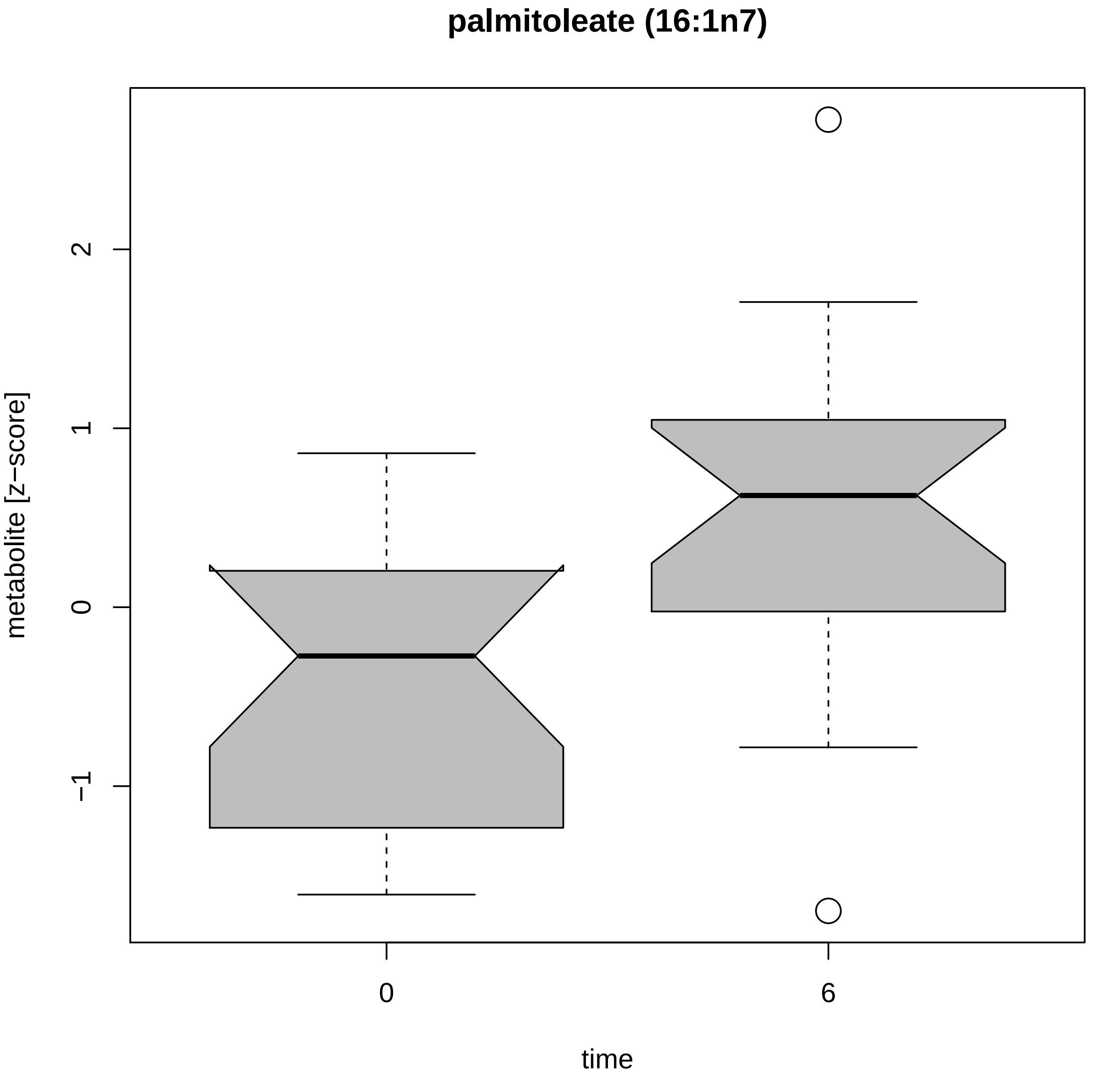
palmitoleate (16:1n7)
metabolite [z−score]
2
1
0
−1
0
6
time

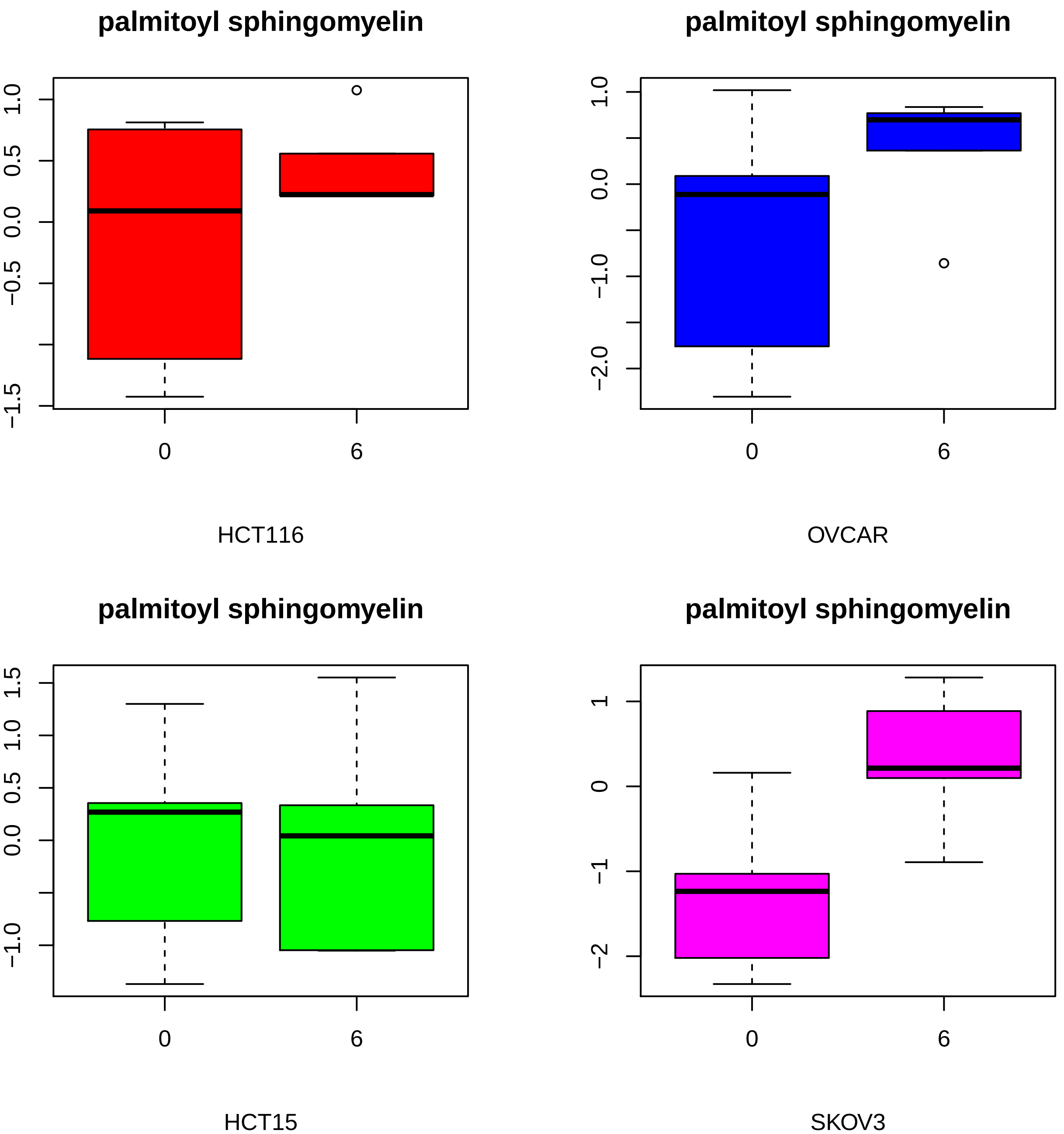
palmitoyl sphingomyelin
1.0
0.5
0.0
−0.5
−1.5
0
6
HCT116
palmitoyl sphingomyelin
1.0
0.0
−1.0
−2.0
0
6
OVCAR
palmitoyl sphingomyelin
1.5
1.0
0.5
0.0
−1.0
0
6
HCT15
palmitoyl sphingomyelin
1
0
−1
−2
0
6
SKOV3

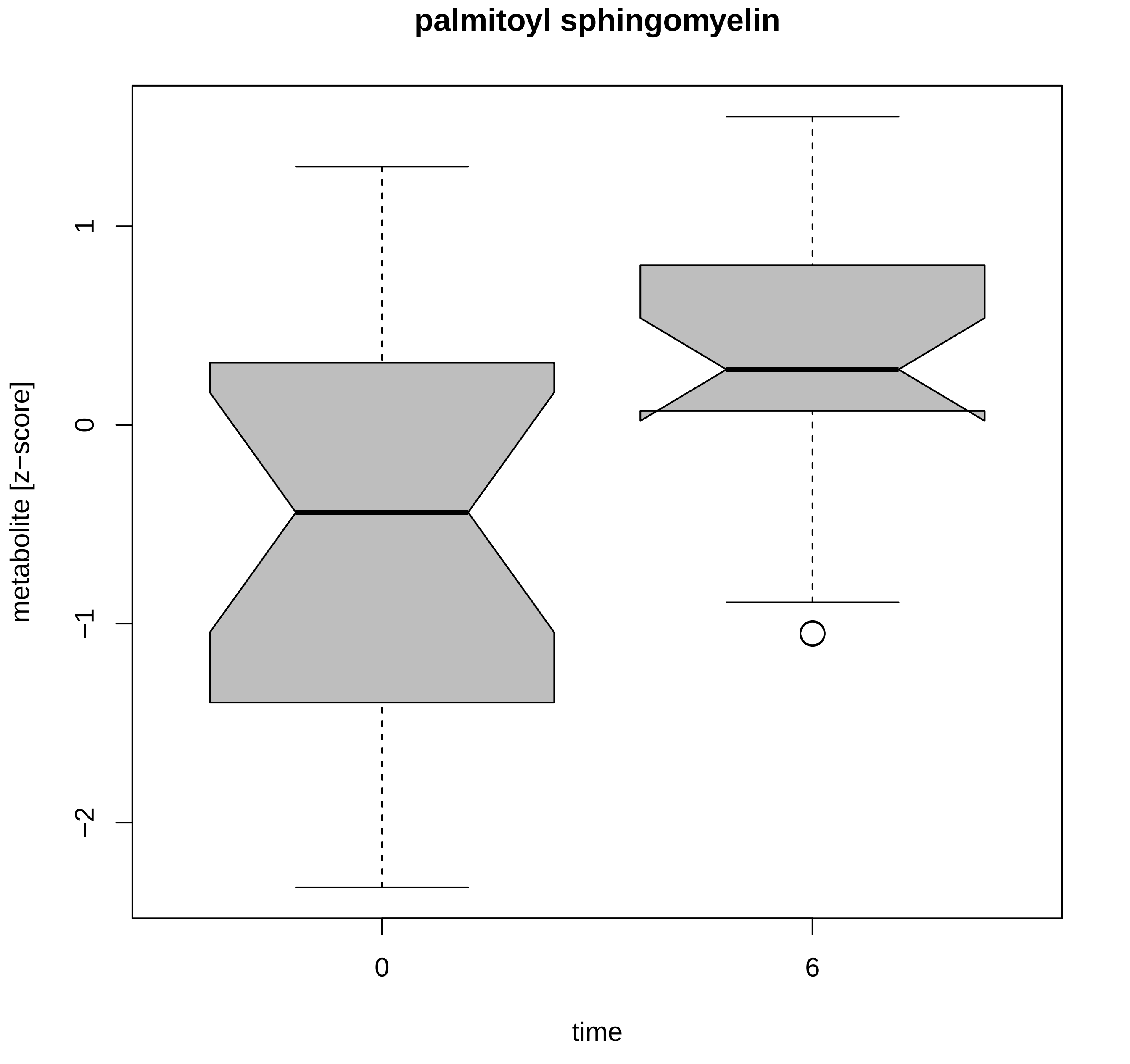
palmitoyl sphingomyelin
metabolite [z−score]
−2
−1
0
1
0
6
time

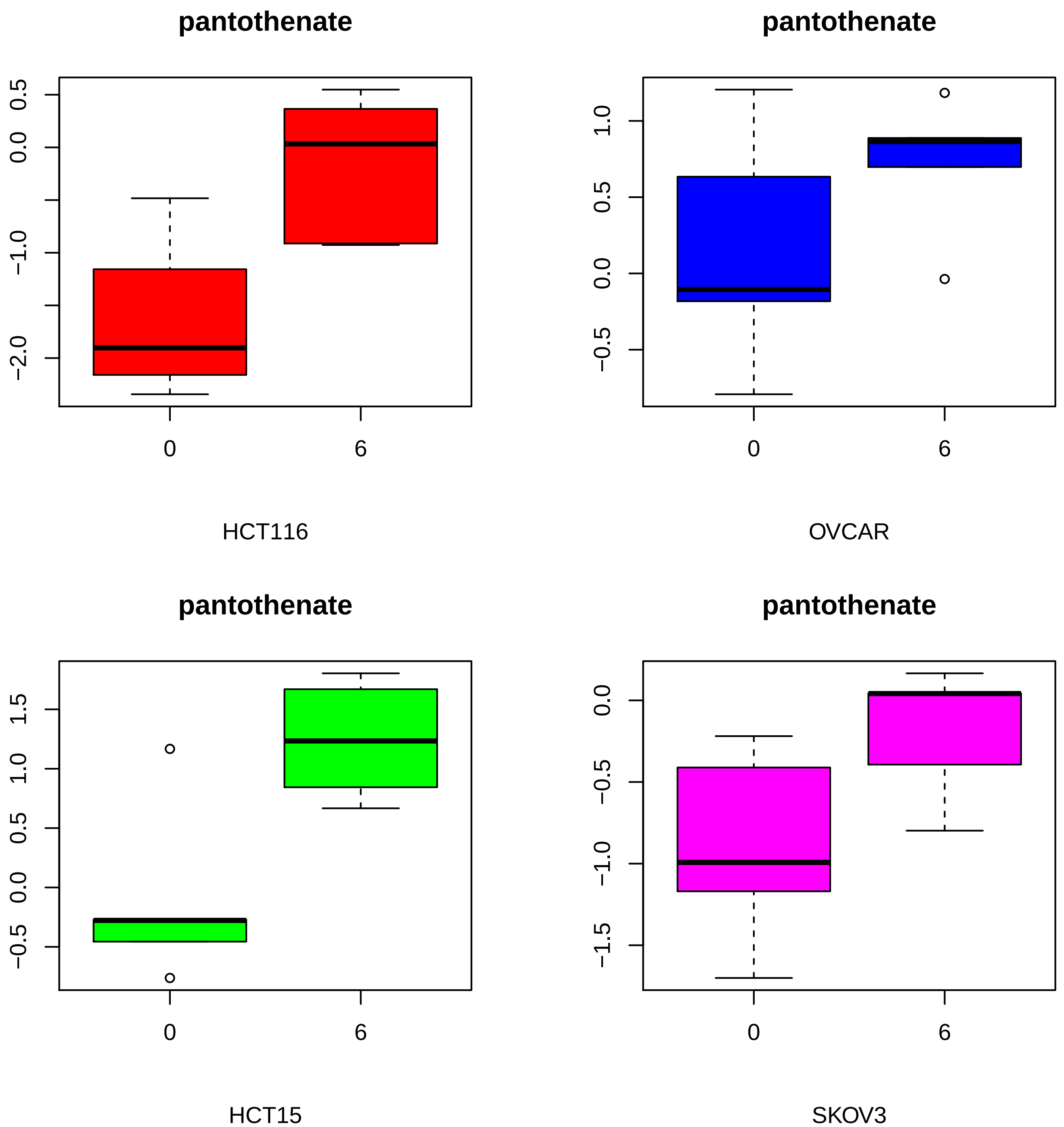
pantothenate
0.5
0.0
−1.0
−2.0
0
6
HCT116
pantothenate
1.0
0.5
0.0
−0.5
0
6
OVCAR
pantothenate
1.5
1.0
0.5
0.0
−0.5
0
6
HCT15
pantothenate
0.0
−0.5
−1.0
−1.5
0
6
SKOV3

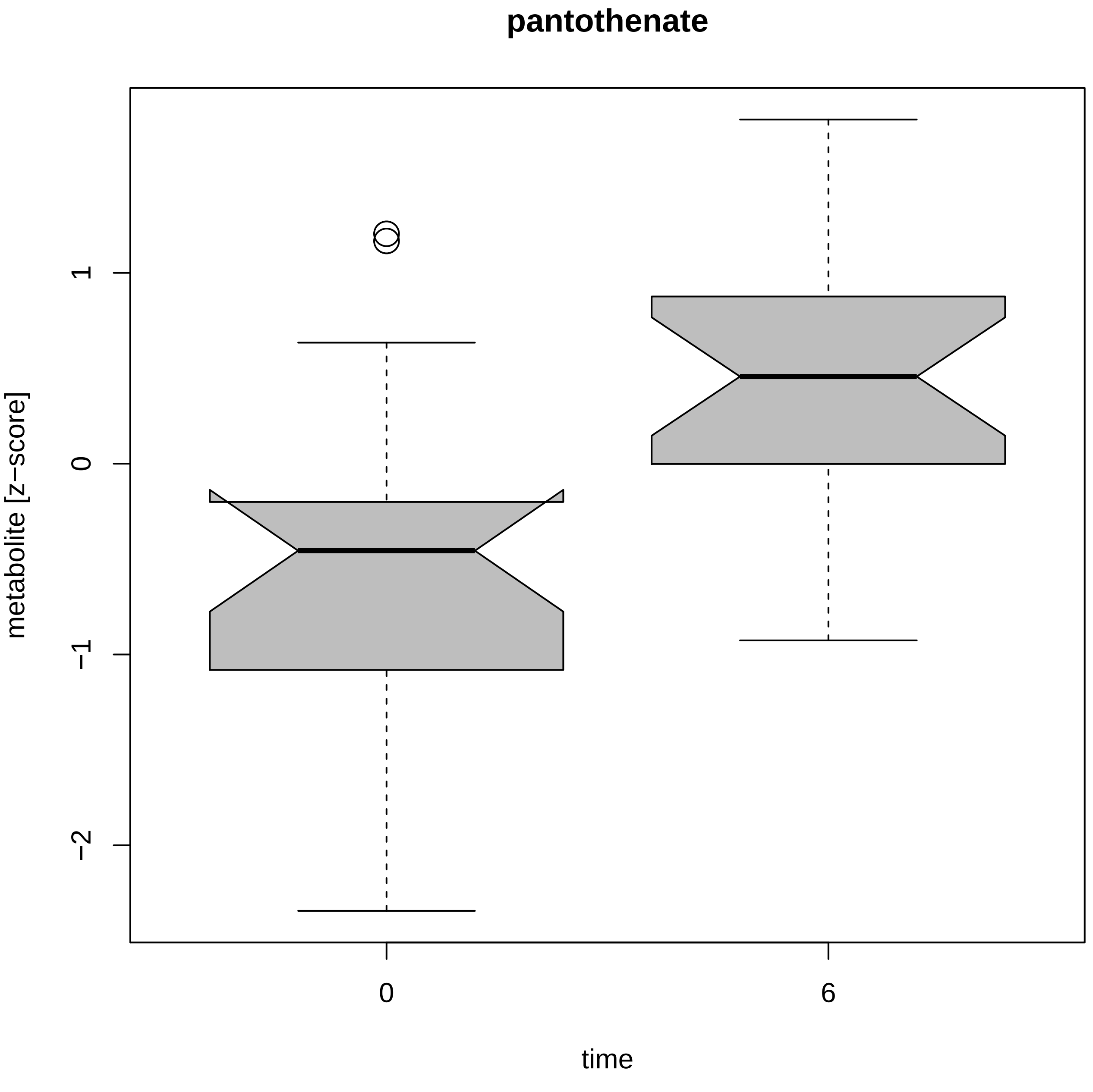
pantothenate
metabolite [z−score]
1
0
−1
−2
0
6
time

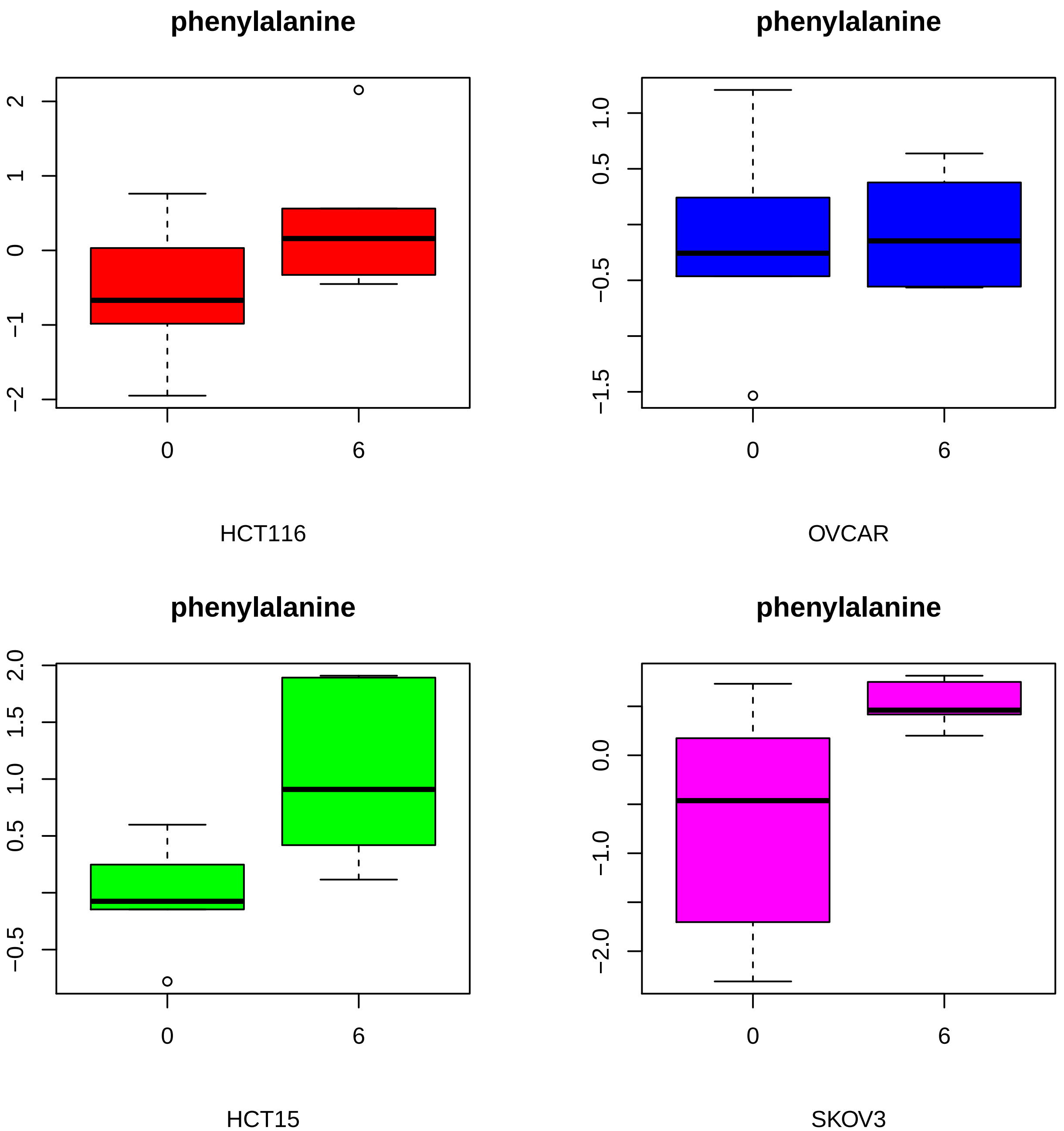
phenylalanine
2
1
0
−1
−2
0
6
HCT116
phenylalanine
1.0
0.5
−0.5
−1.5
0
6
OVCAR
phenylalanine
2.0
1.5
1.0
0.5
−0.5
0
6
HCT15
phenylalanine
0.0
−1.0
−2.0
0
6
SKOV3

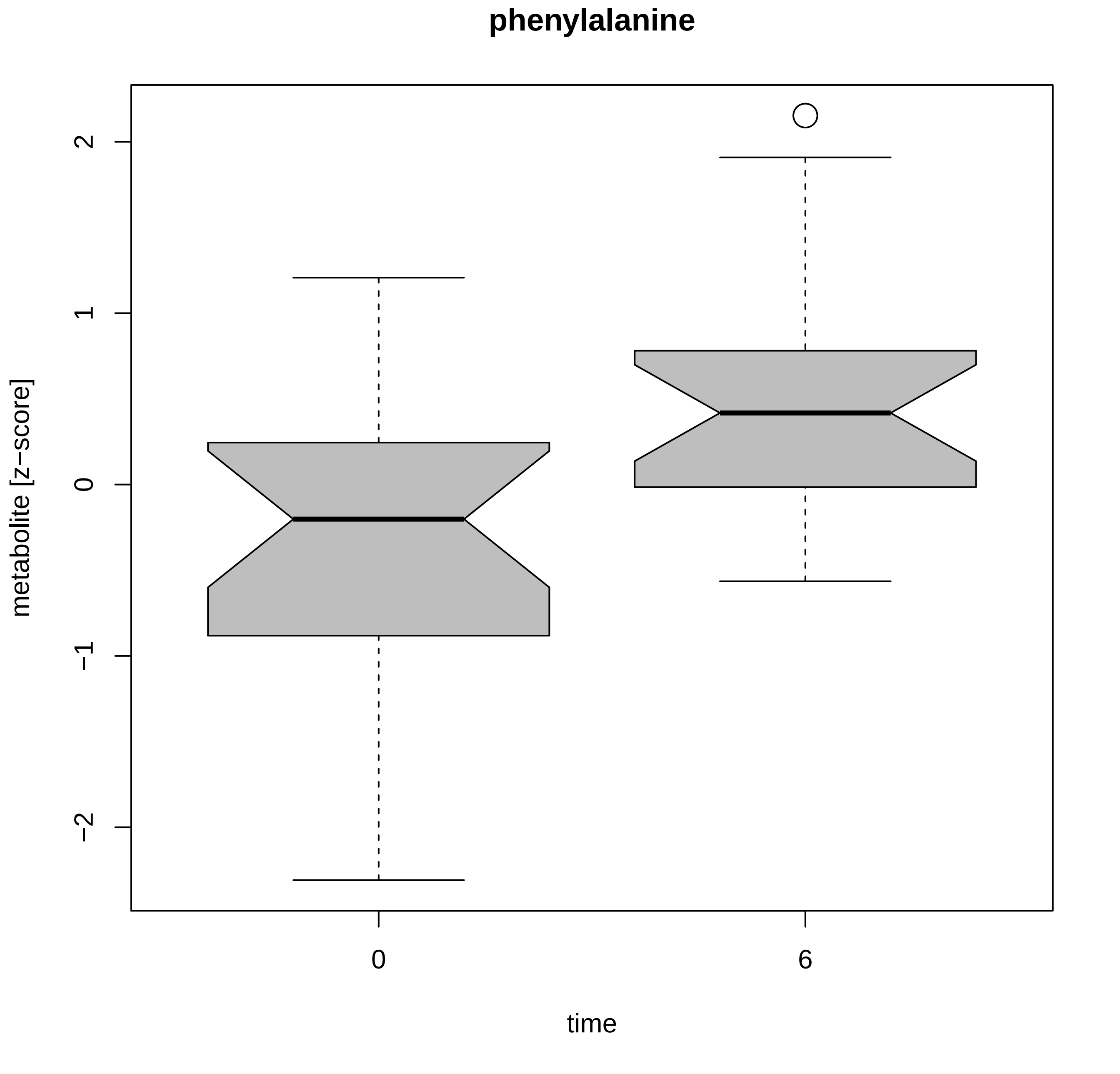
phenylalanine
metabolite [z−score]
2
1
0
−1
−2
0
6
time

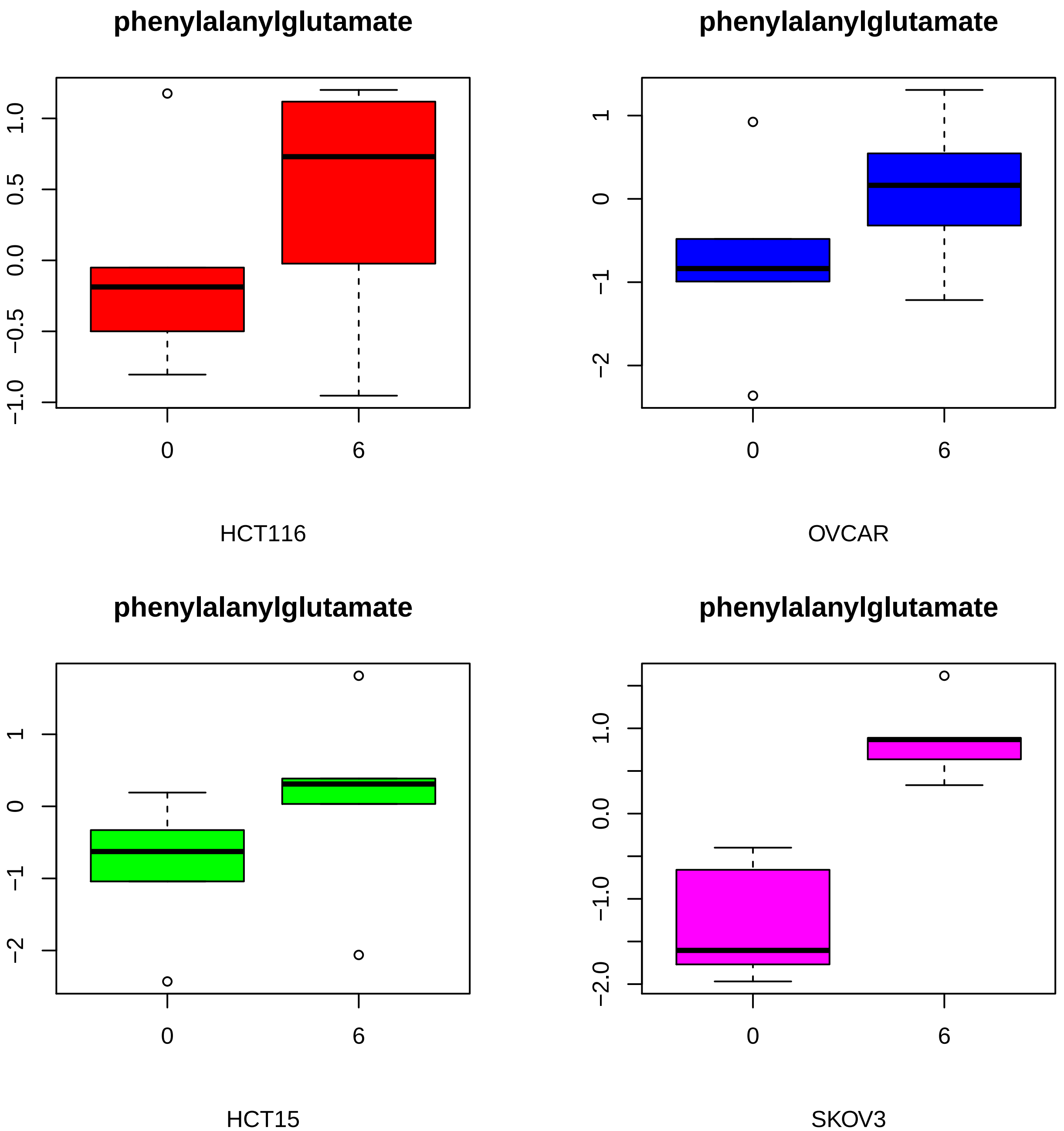
phenylalanylglutamate
1.0
0.5
0.0
−0.5
−1.0
0
6
HCT116
phenylalanylglutamate
1
0
−1
−2
0
6
OVCAR
phenylalanylglutamate
1
0
−1
−2
0
6
HCT15
phenylalanylglutamate
1.0
0.0
−1.0
−2.0
0
6
SKOV3

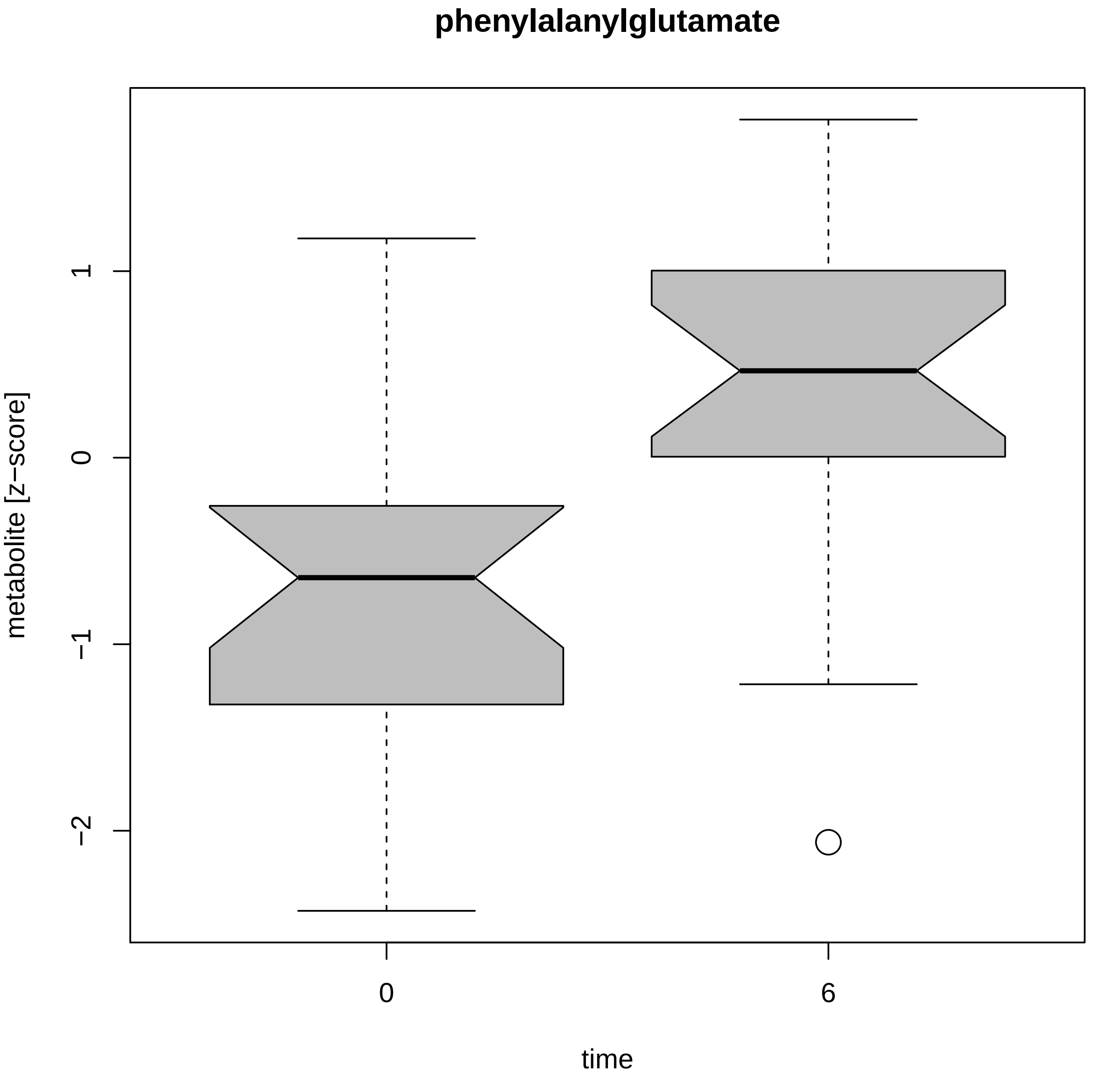
phenylalanylglutamate
metabolite [z−score]
1
0
−1
−2
0
6
time

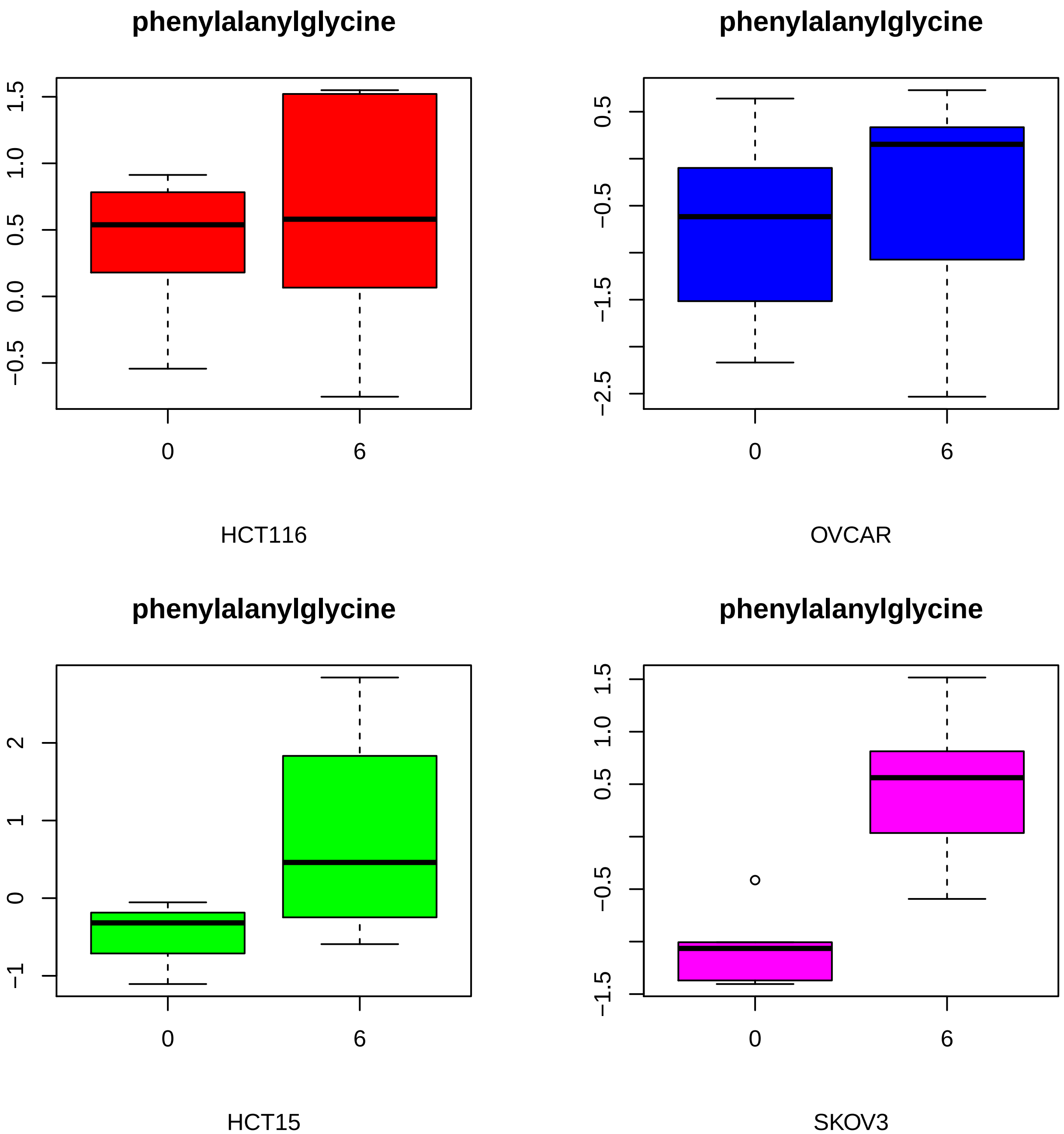
phenylalanylglycine
−0.5
0.0
0.5
1.0
1.5
0
6
HCT116
phenylalanylglycine
−2.5
−1.5
−0.5
0.5
0
6
OVCAR
phenylalanylglycine
−1
0
1
2
0
6
HCT15
phenylalanylglycine
−1.5
−0.5
0.5
1.0
1.5
0
6
SKOV3

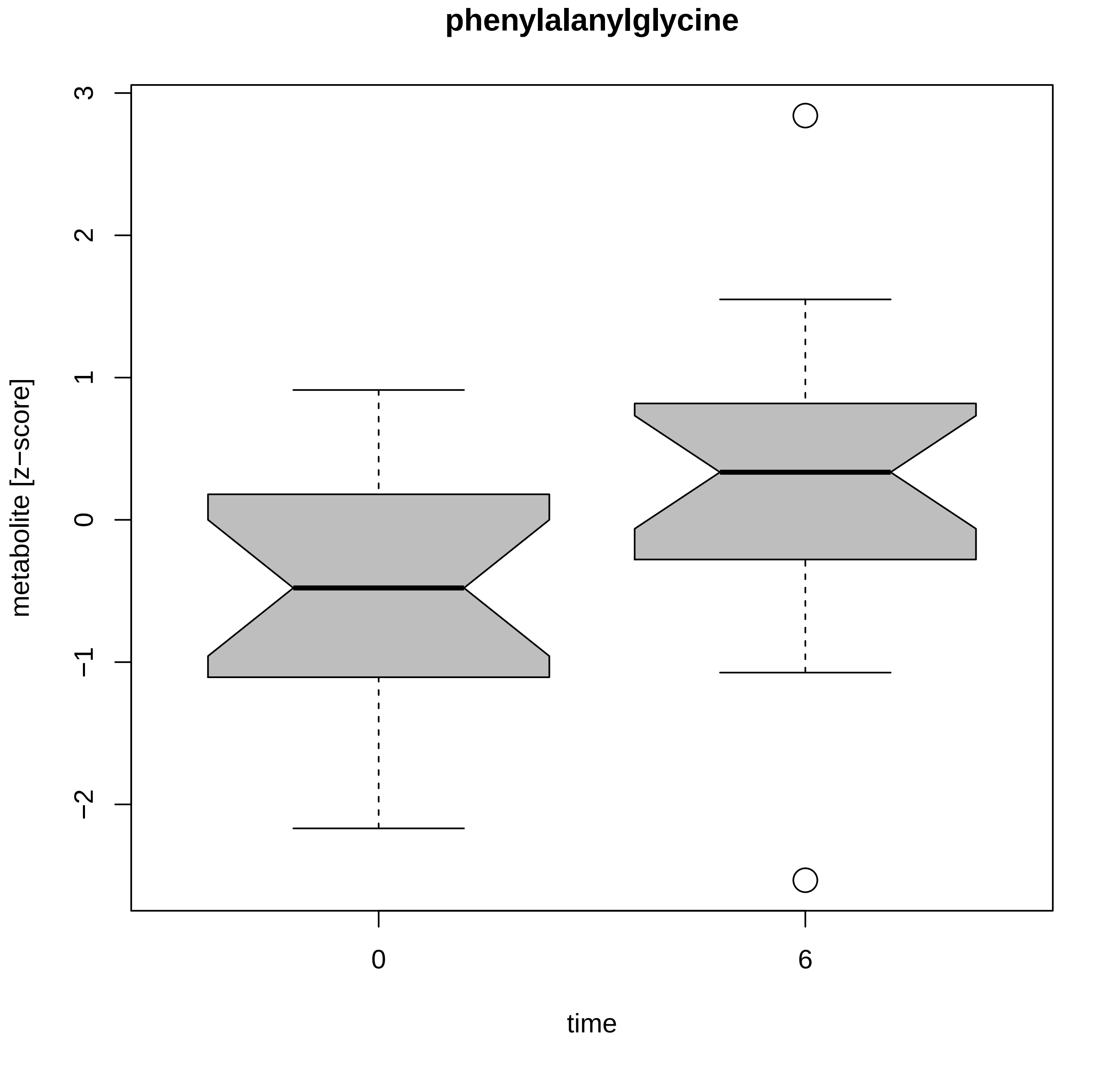
phenylalanylglycine
metabolite [z−score]
3
2
1
0
−1
−2
0
6
time

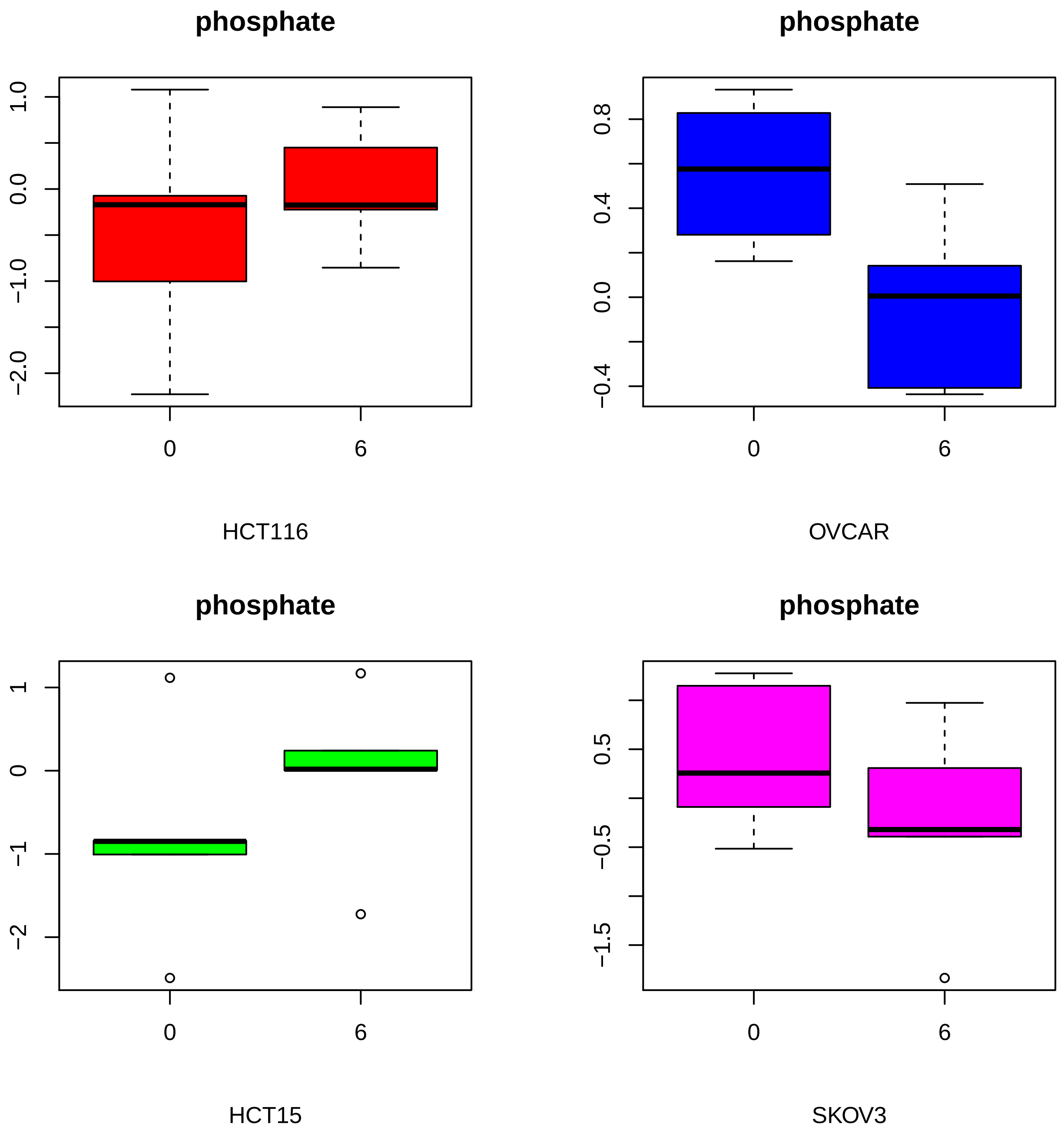
phosphate
1.0
0.0
−1.0
−2.0
0
6
HCT116
phosphate
0.8
0.4
0.0
−0.4
0
6
OVCAR
phosphate
1
0
−1
−2
0
6
HCT15
phosphate
0.5
−0.5
−1.5
0
6
SKOV3

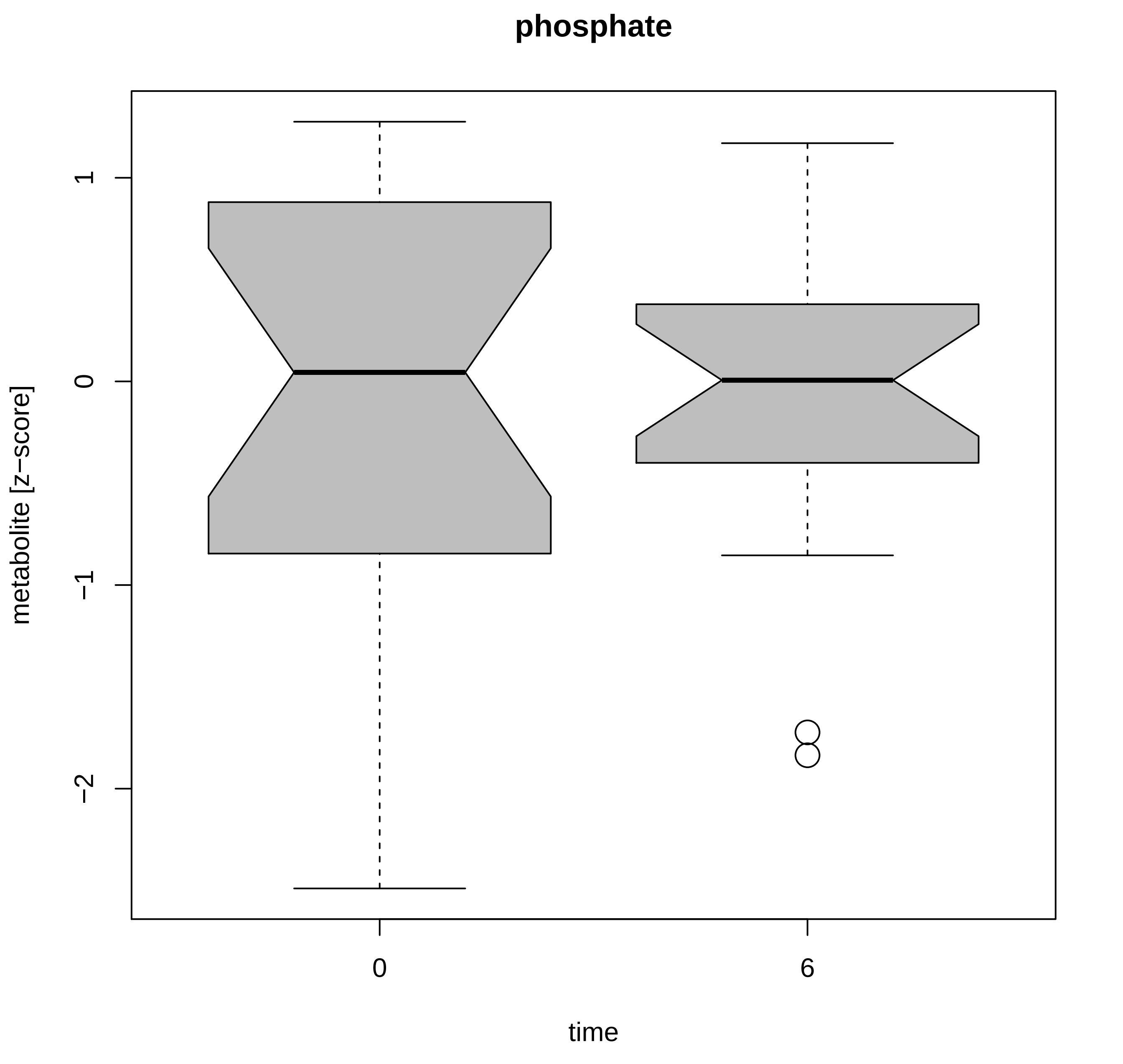

phosphate
metabolite [z−score]
1
0
−1
−2
0
6
time

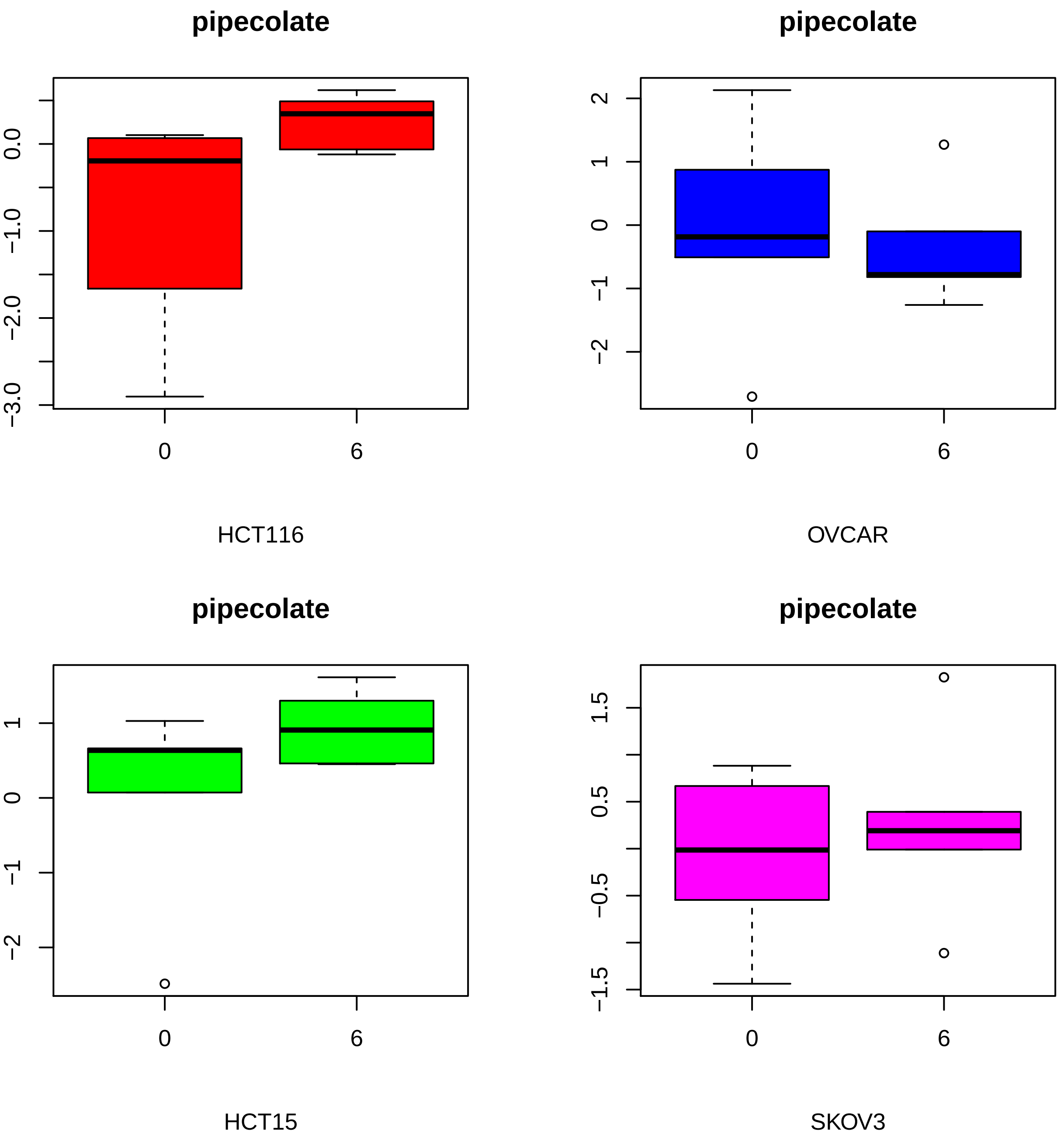
pipecolate
0.0
−1.0
−2.0
−3.0
0
6
HCT116
pipecolate
2
1
0
−1
−2
0
6
OVCAR
pipecolate
1
0
−1
−2
0
6
HCT15
pipecolate
1.5
0.5
−0.5
−1.5
0
6
SKOV3

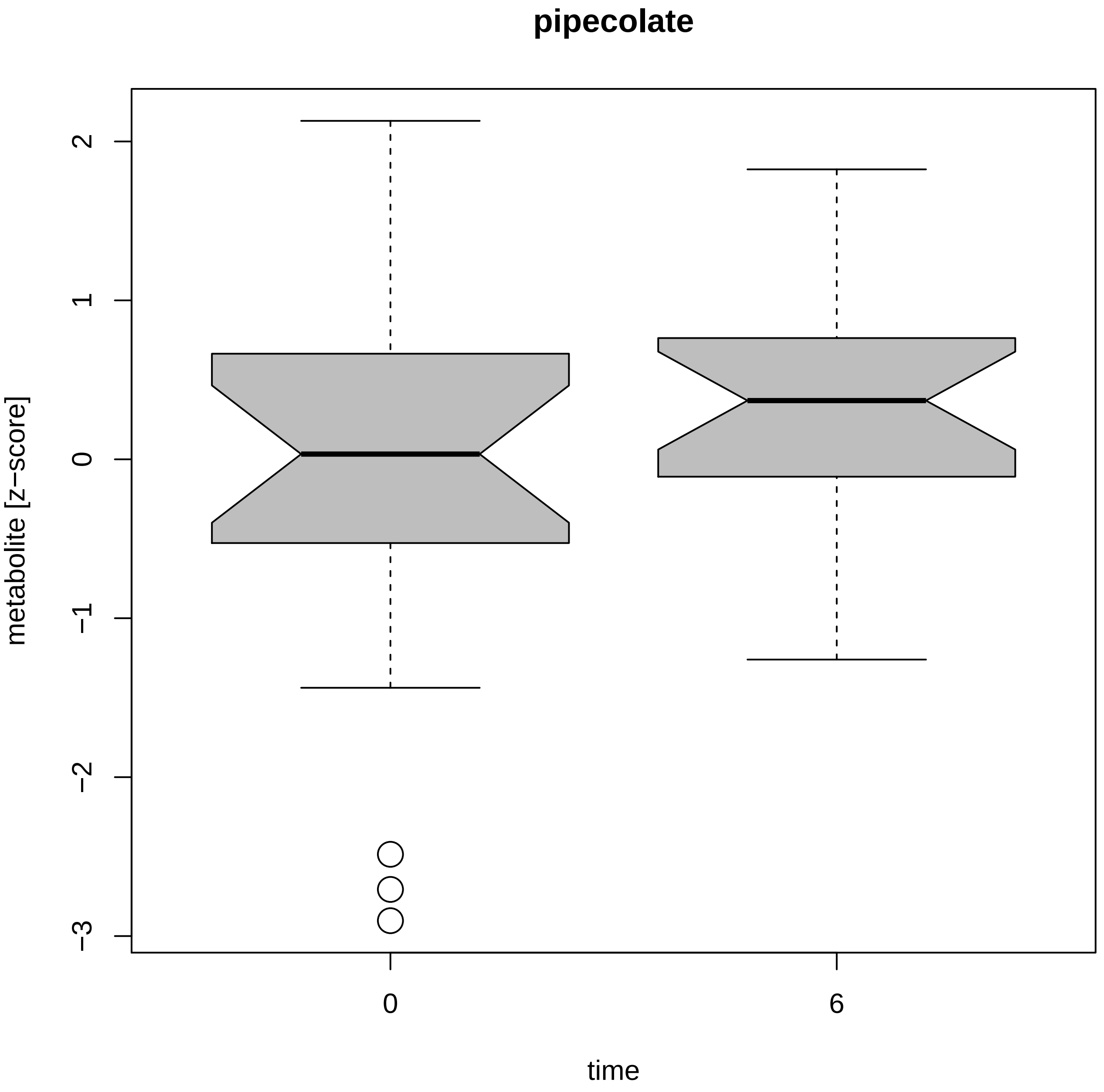
pipecolate
metabolite [z−score]
2
1
0
−1
−2
−3
0
6
time

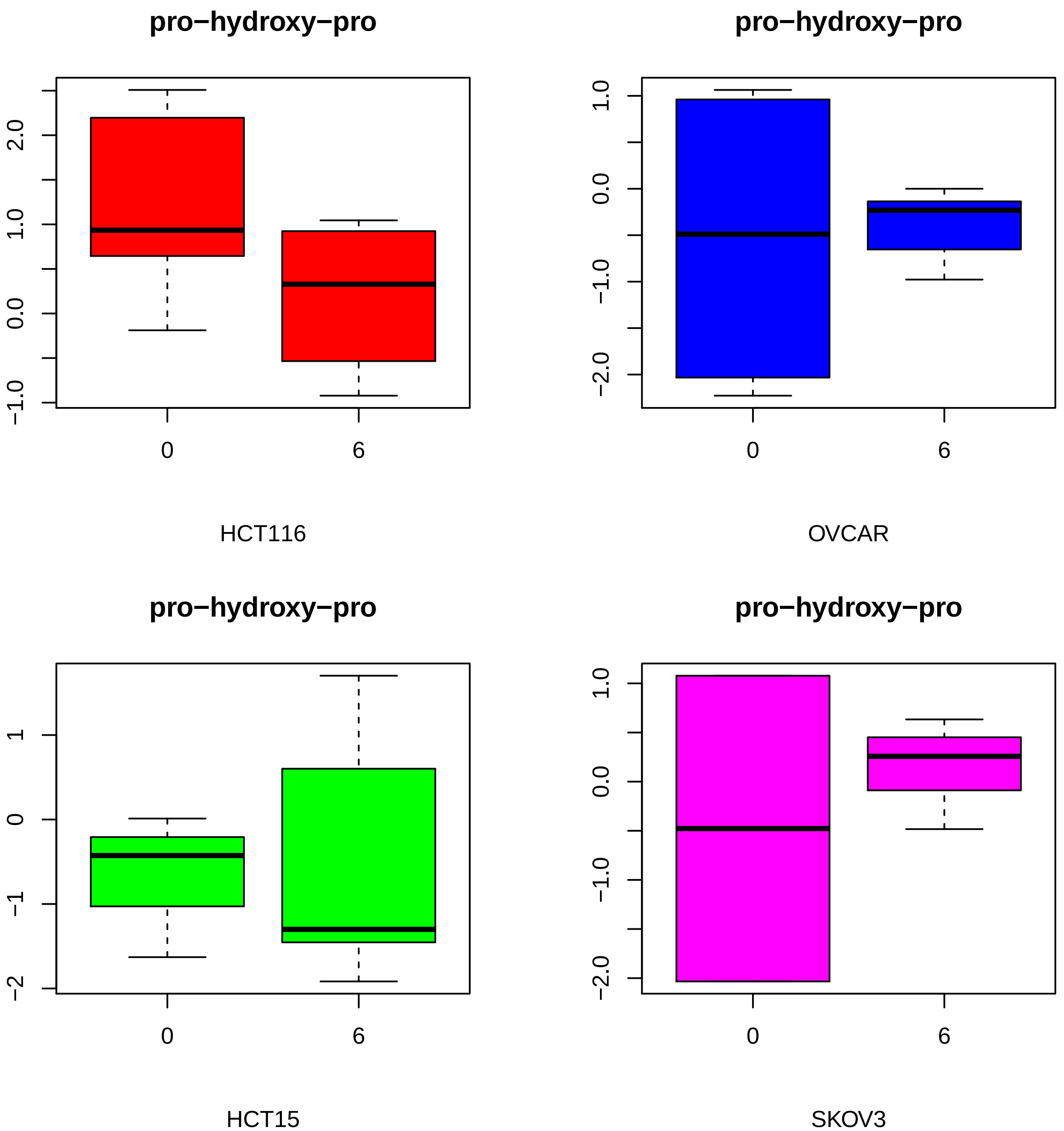
pro−hydroxy−pro
−1.0
0.0
1.0
2.0
0
6
HCT116
pro−hydroxy−pro
−2.0
−1.0
0.0
1.0
0
6
OVCAR
pro−hydroxy−pro
−2
−1
0
1
0
6
HCT15
pro−hydroxy−pro
−2.0
−1.0
0.0
1.0
0
6
SKOV3

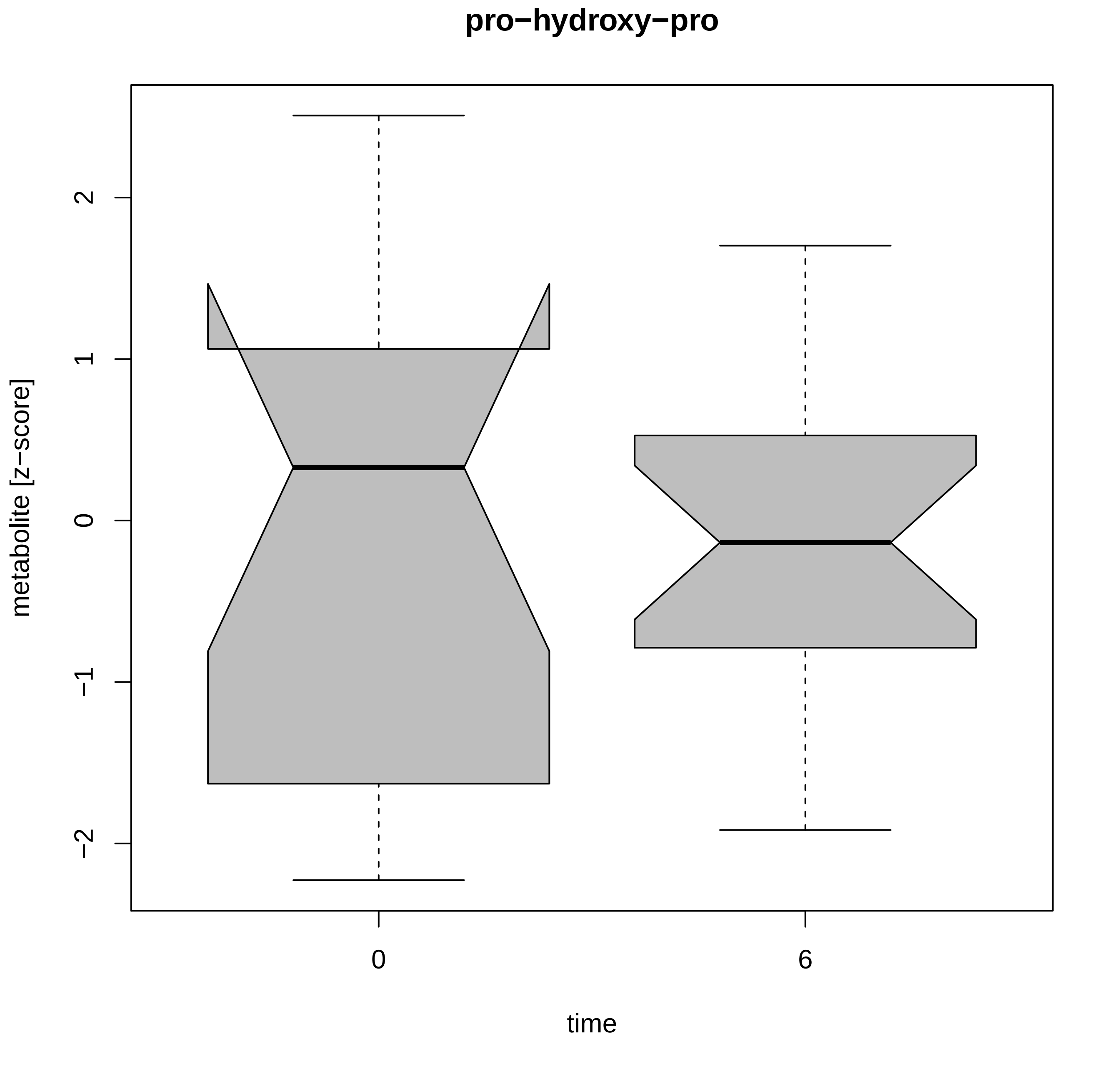

pro−hydroxy−pro
metabolite [z−score]
2
1
0
−1
−2
0
6
time

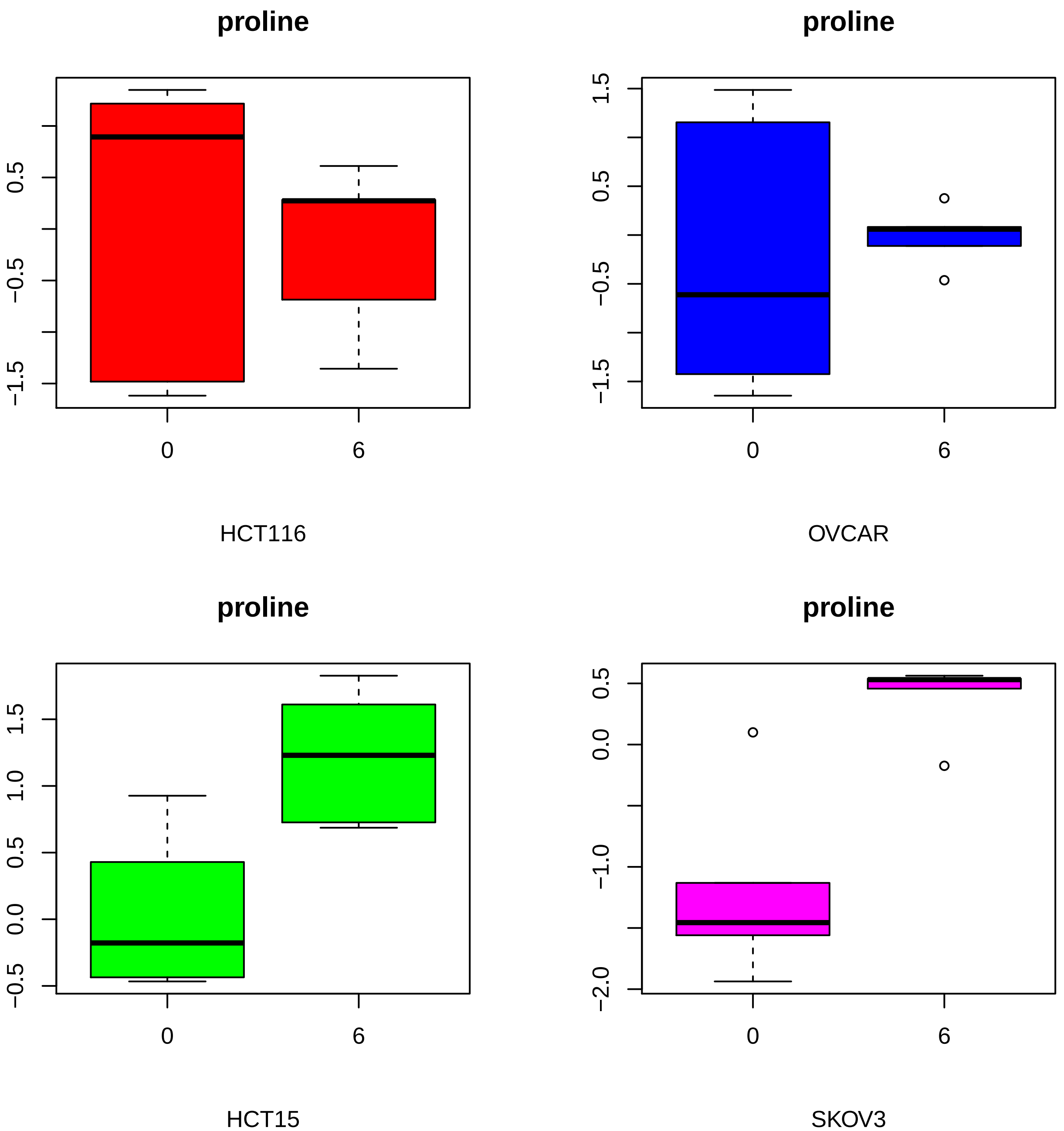
proline
0.5
−0.5
−1.5
0
6
HCT116
proline
1.5
0.5
−0.5
−1.5
0
6
OVCAR
proline
1.5
1.0
0.5
0.0
−0.5
0
6
HCT15
proline
0.5
0.0
−1.0
−2.0
0
6
SKOV3

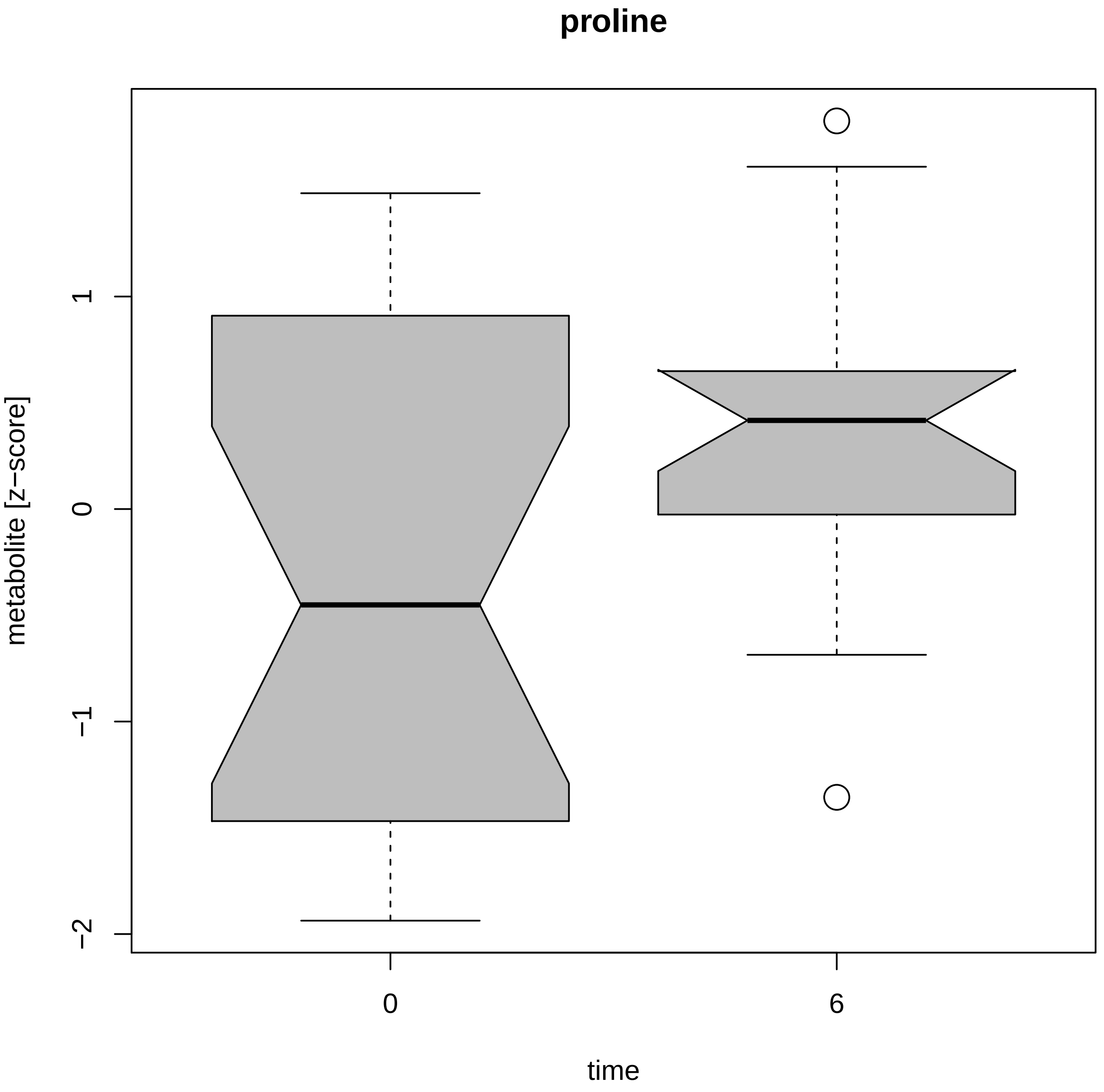
proline
metabolite [z−score]
1
0
−1
−2
0
6
time

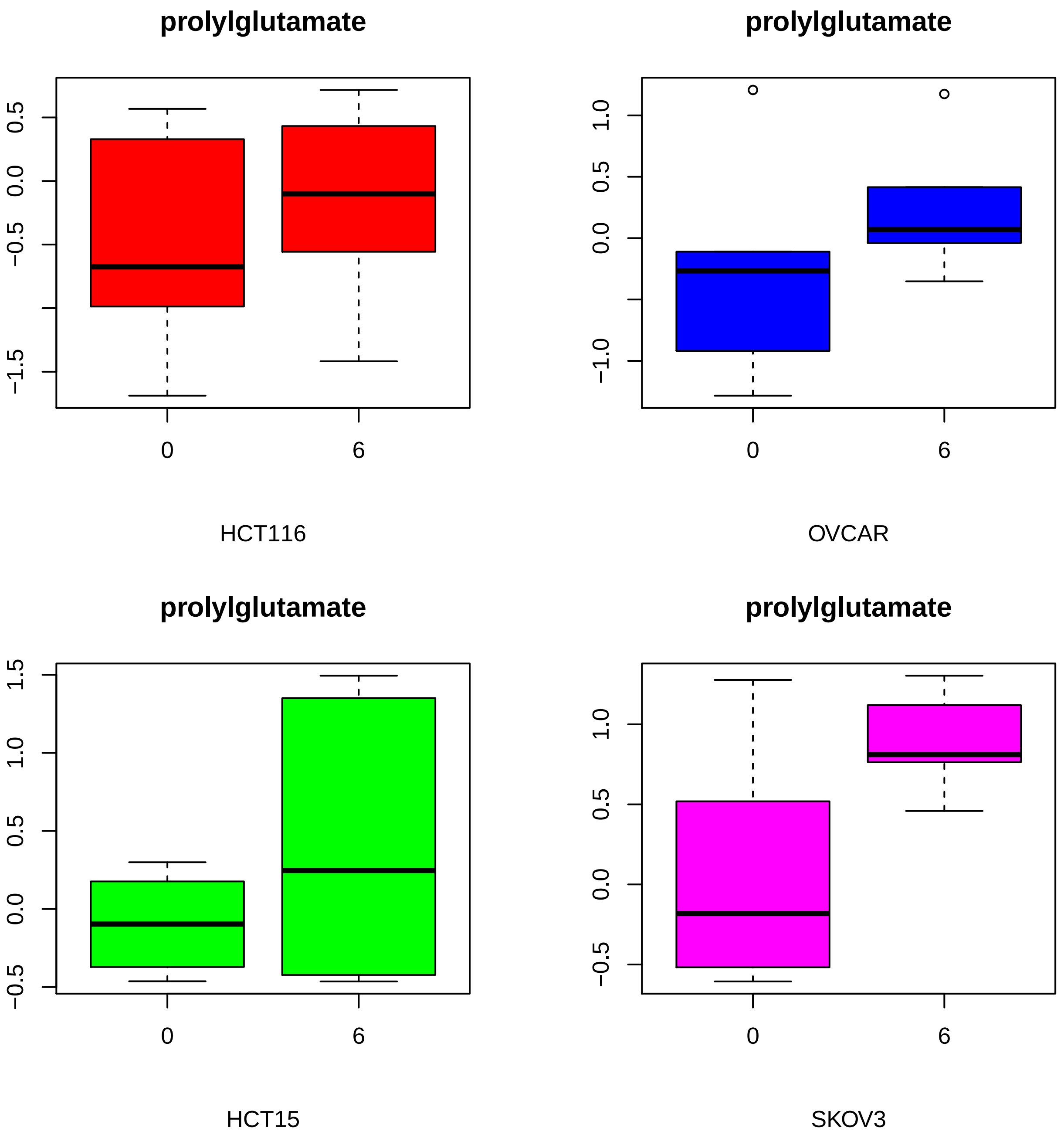

prolylglutamate
0.5
0.0
−0.5
−1.5
0
6
HCT116
prolylglutamate
1.0
0.5
0.0
−1.0
0
6
OVCAR
prolylglutamate
1.5
1.0
0.5
0.0
−0.5
0
6
HCT15
prolylglutamate
1.0
0.5
0.0
−0.5
0
6
SKOV3

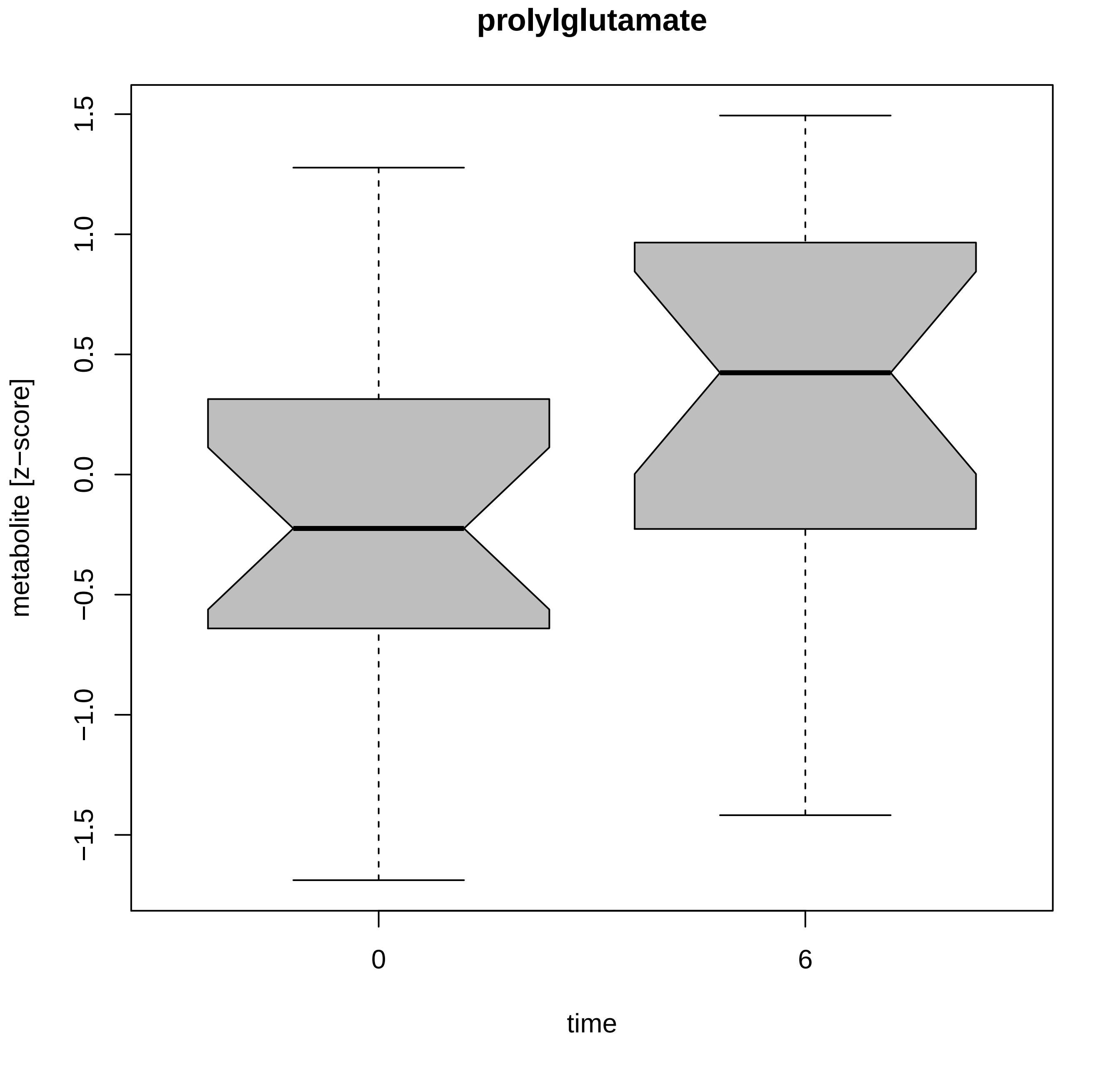

prolylglutamate
metabolite [z−score]
1.5
1.0
0.5
0.0
−0.5
−1.0
−1.5
0
6
time

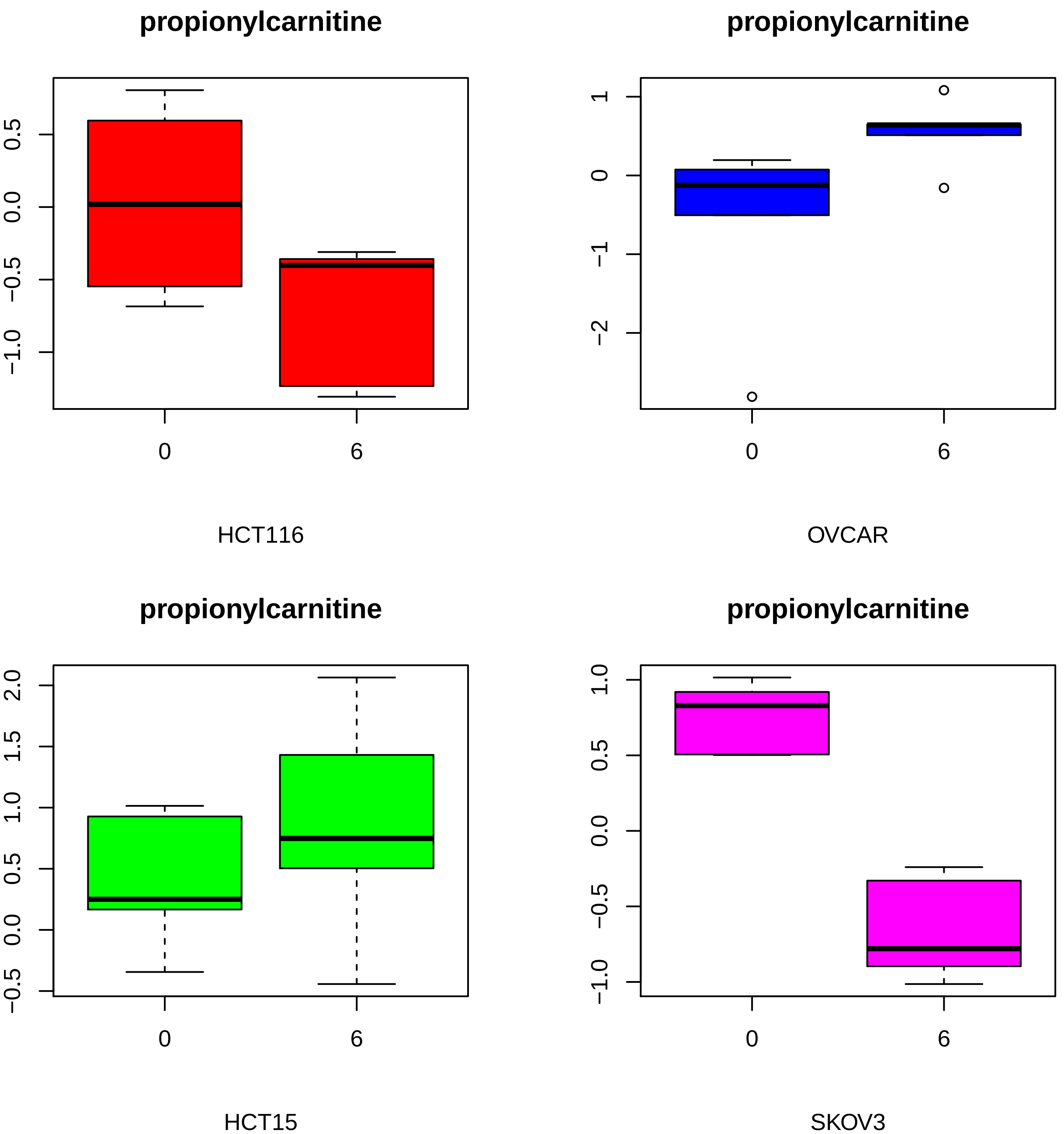
propionylcarnitine
0.5
0.0
−0.5
−1.0
0
6
HCT116
propionylcarnitine
1
0
−1
−2
0
6
OVCAR
propionylcarnitine
2.0
1.5
1.0
0.5
0.0
−0.5
0
6
HCT15
propionylcarnitine
1.0
0.5
0.0
−0.5
−1.0
0
6
SKOV3

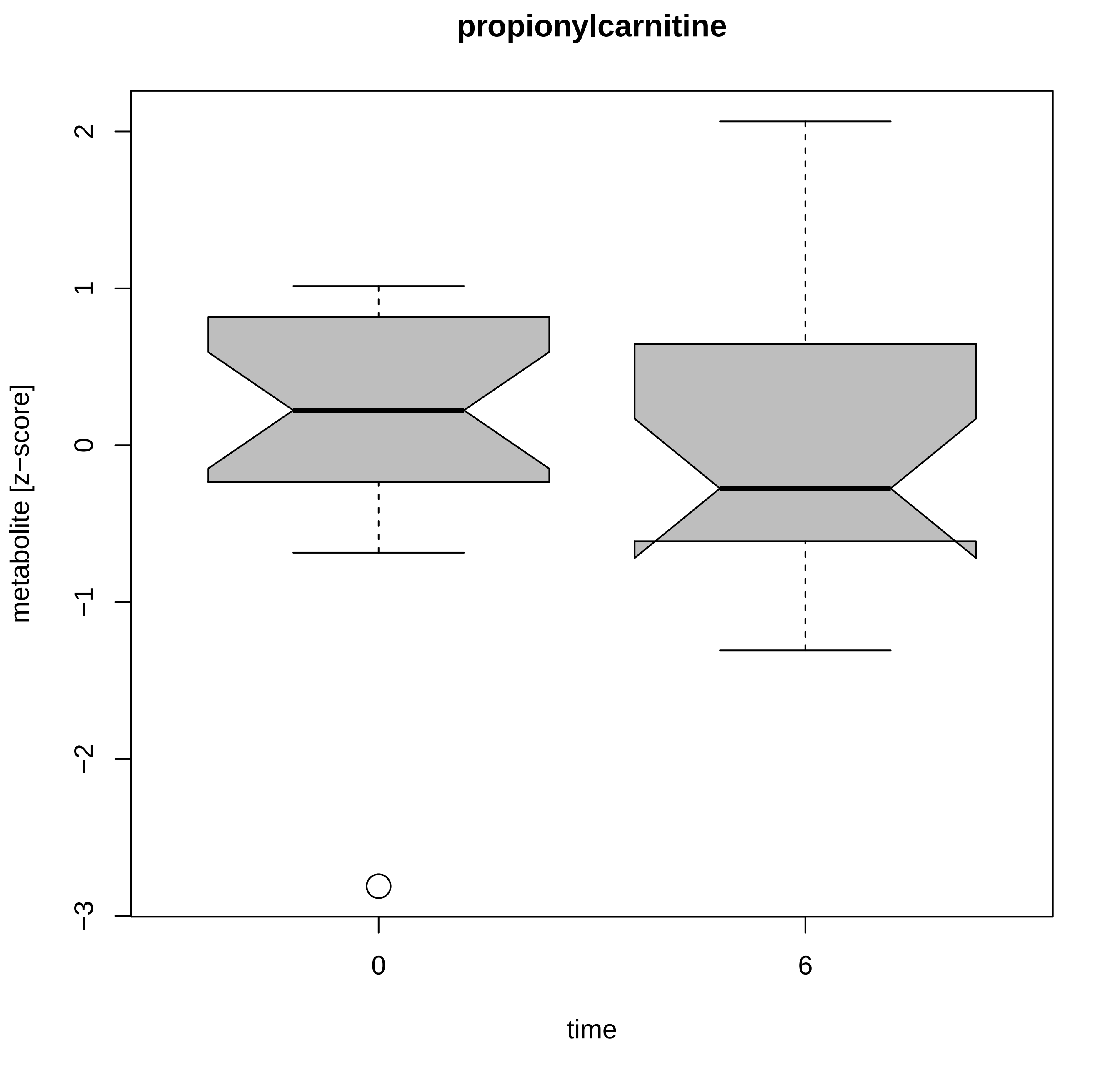
propionylcarnitine
metabolite [z−score]
2
1
0
−1
−2
−3
0
6
time

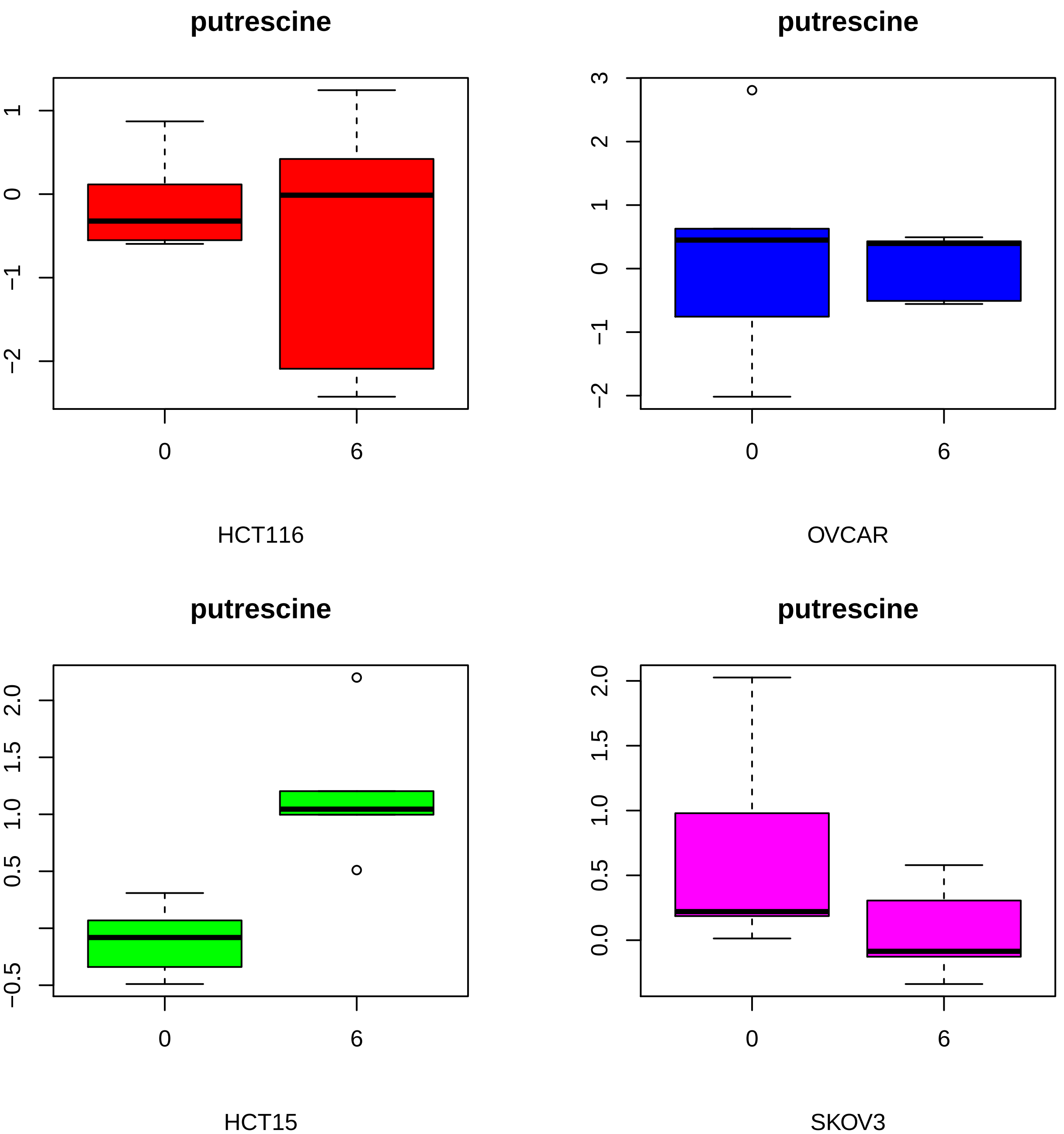
putrescine
1
0
−1
−2
0
6
HCT116
putrescine
3
2
1
0
−1
−2
0
6
OVCAR
putrescine
2.0
1.5
1.0
0.5
−0.5
0
6
HCT15
putrescine
2.0
1.5
1.0
0.5
0.0
0
6
SKOV3

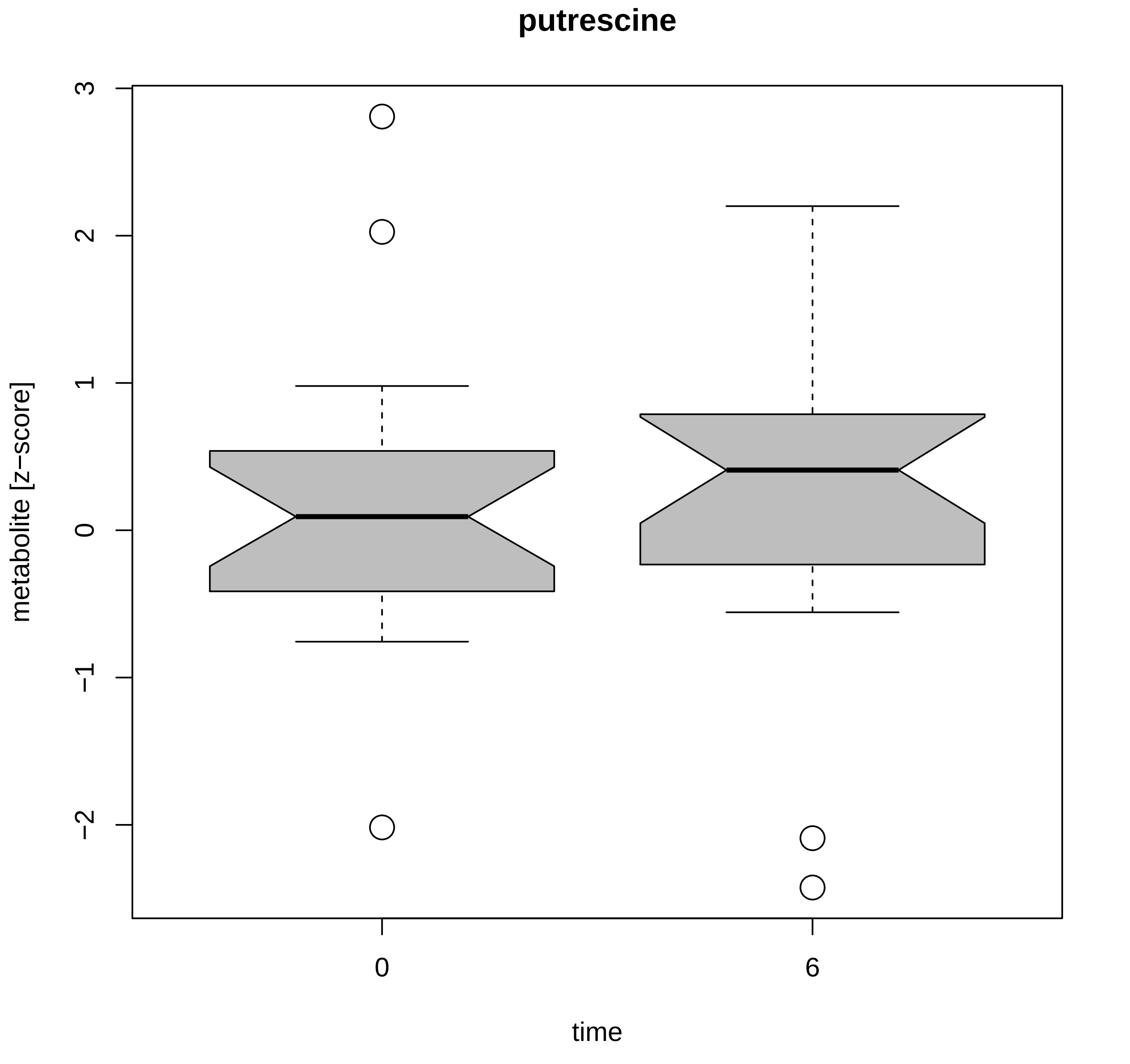
putrescine
metabolite [z−score]
3
2
1
0
−1
−2
0
6
time

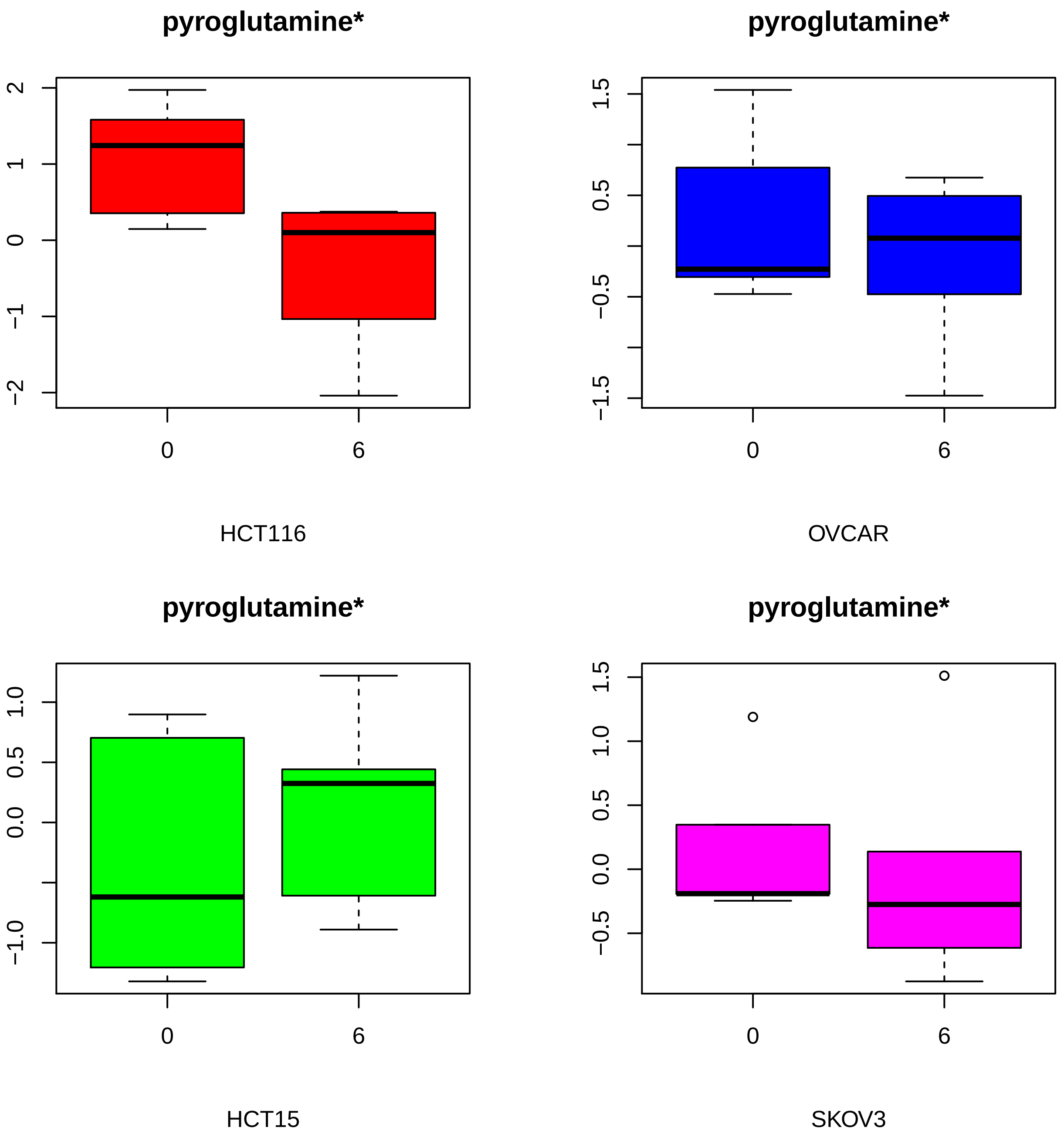
pyroglutamine*
2
1
0
−1
−2
0
6
HCT116
pyroglutamine*
1.5
0.5
−0.5
−1.5
0
6
OVCAR
pyroglutamine*
1.0
0.5
0.0
−1.0
0
6
HCT15
pyroglutamine*
1.5
1.0
0.5
0.0
−0.5
0
6
SKOV3

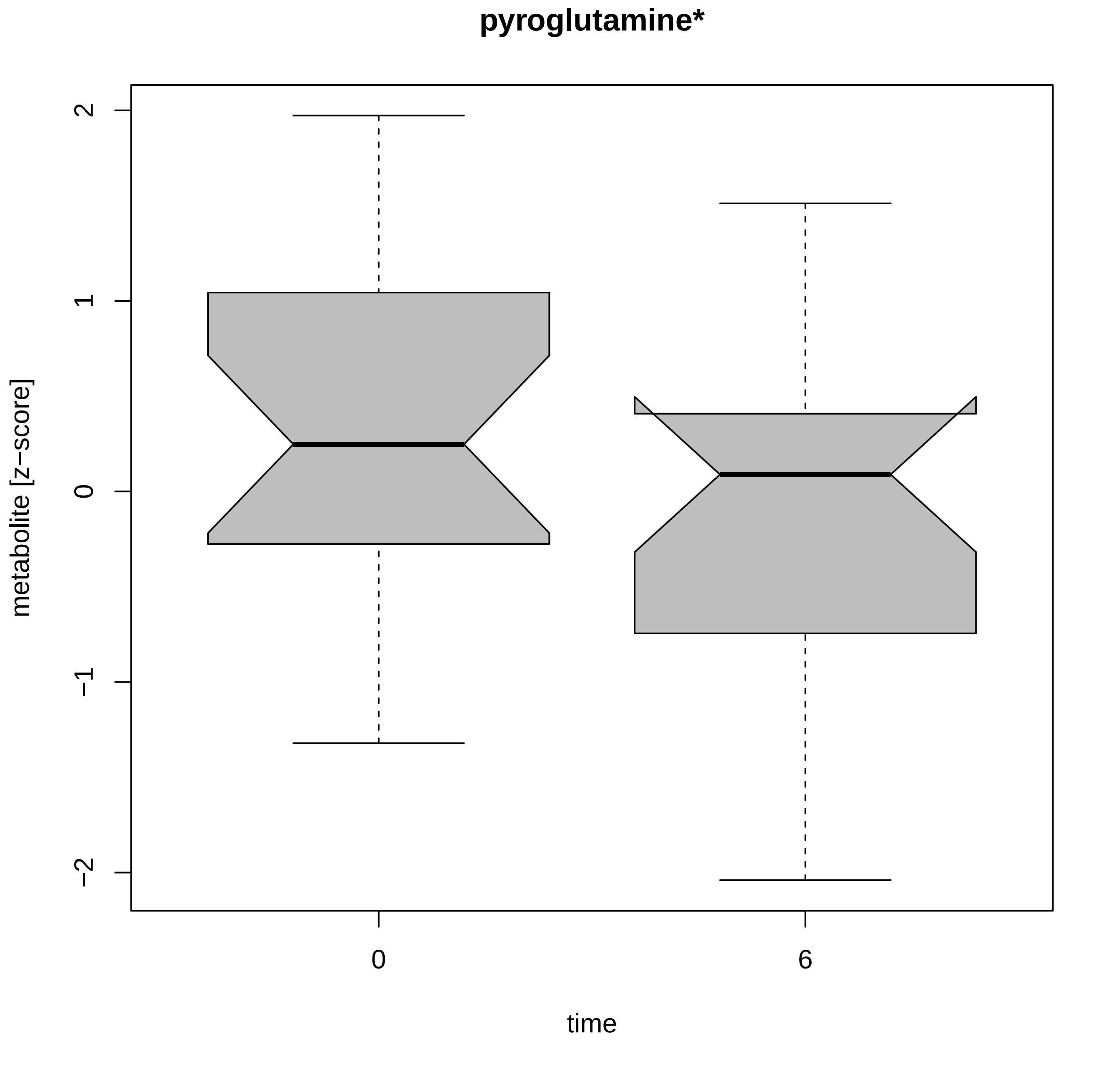
pyroglutamine*
metabolite [z−score]
2
1
0
−1
−2
0
6
time

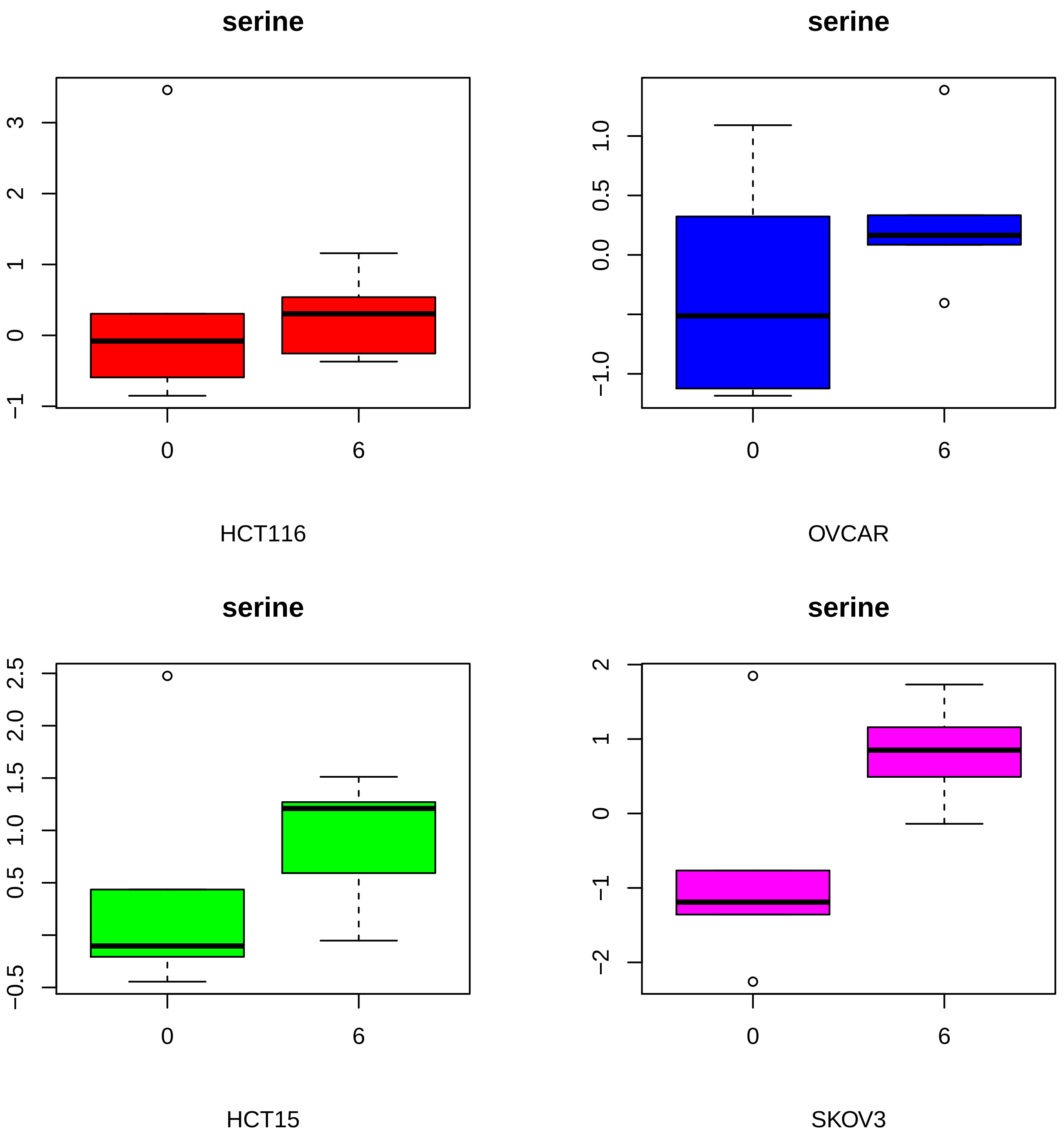
serine
HCT116
OVCAR
HCT15
SKOV3
0
6
−1
0
1
2
3
−1.0
0.0
0.5
1.0
−0.5
0.5
1.0
1.5
2.0
2.5
−2
−1
0
1
2

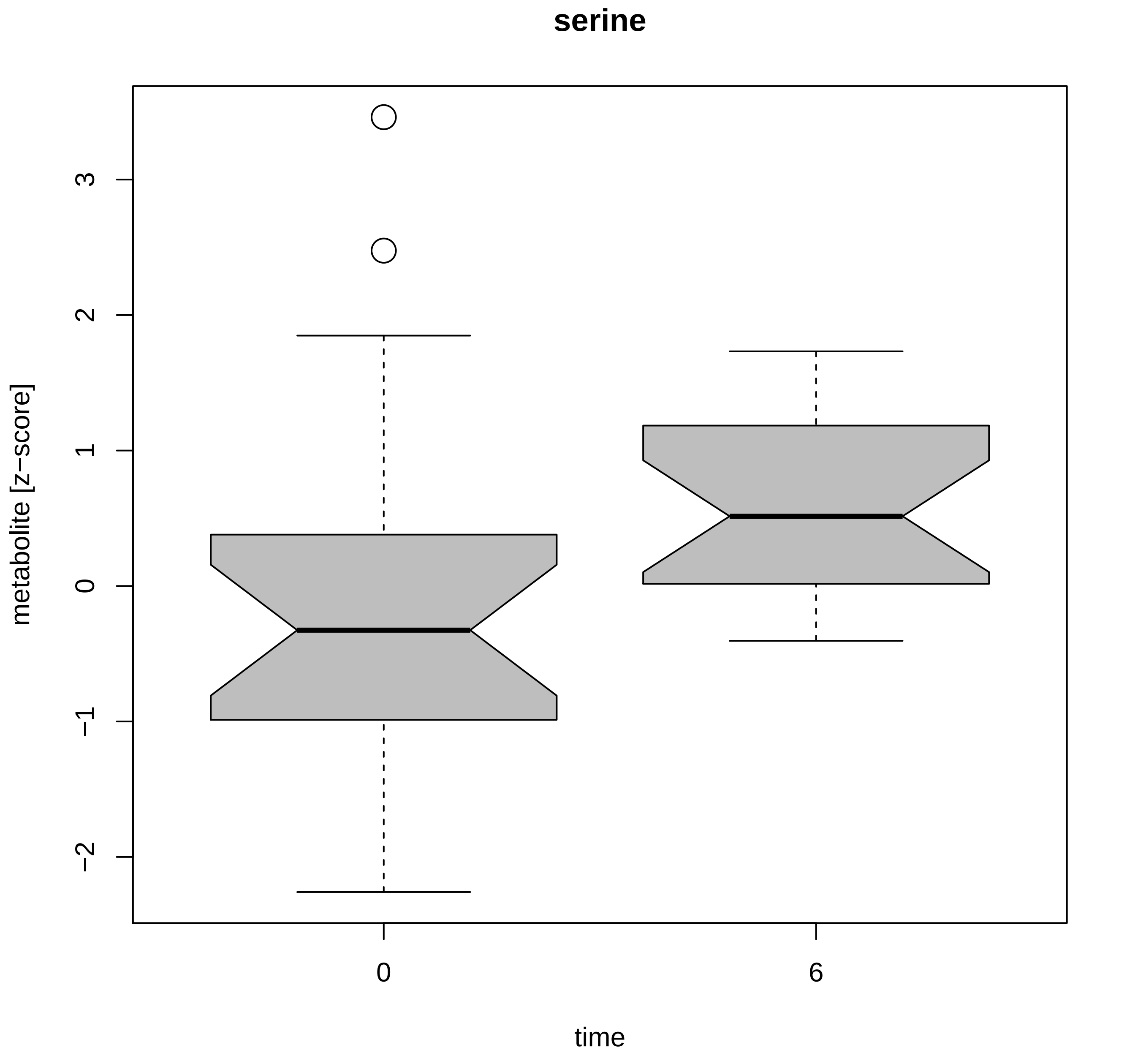
serine
metabolite [z−score]
3
2
1
0
−1
−2
0
6
time

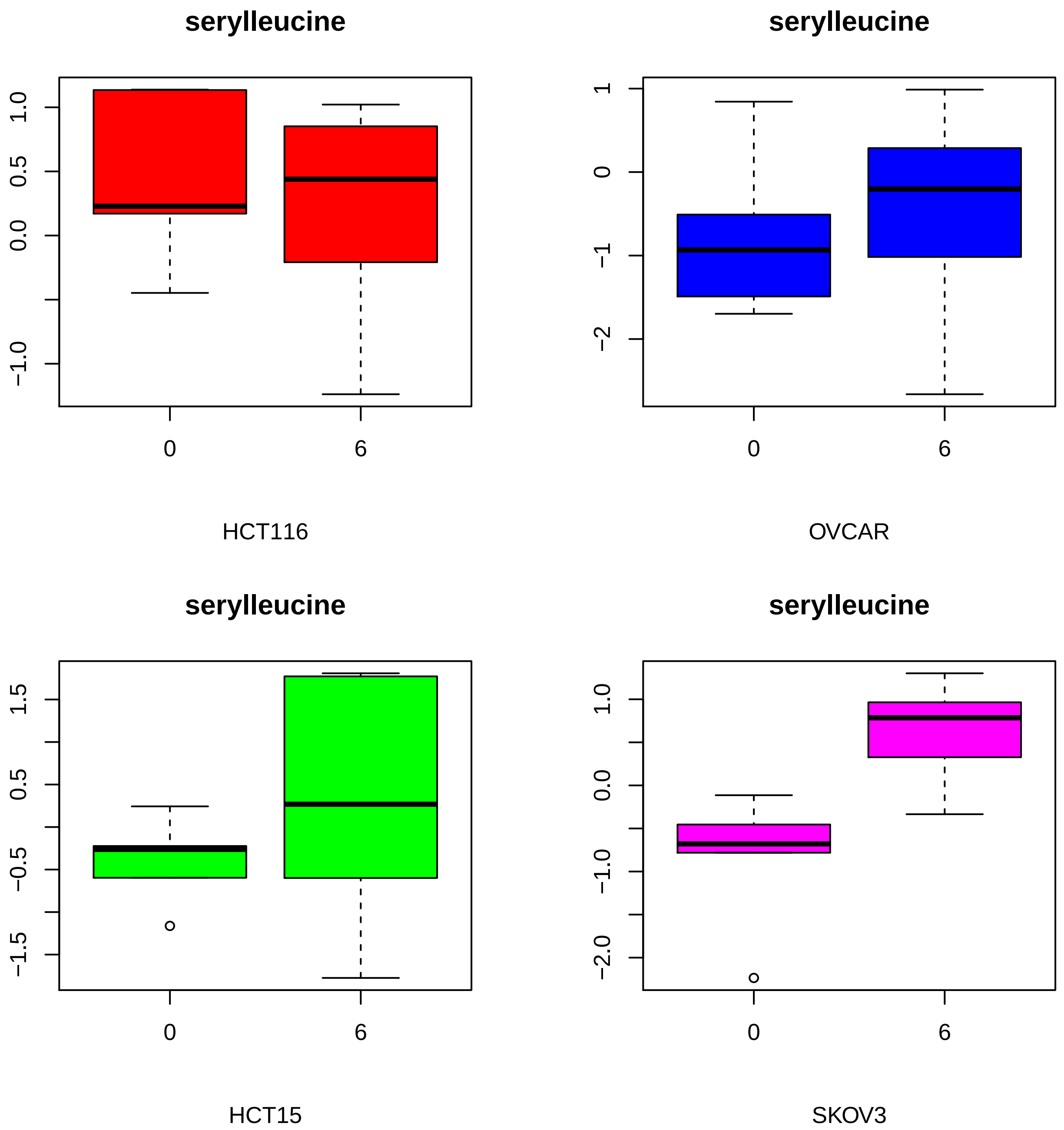

serylleucine
1.0
0.5
0.0
−1.0
0
6
HCT116
serylleucine
1
0
−1
−2
0
6
OVCAR
serylleucine
1.5
0.5
−0.5
−1.5
0
6
HCT15
serylleucine
1.0
0.0
−1.0
−2.0
0
6
SKOV3

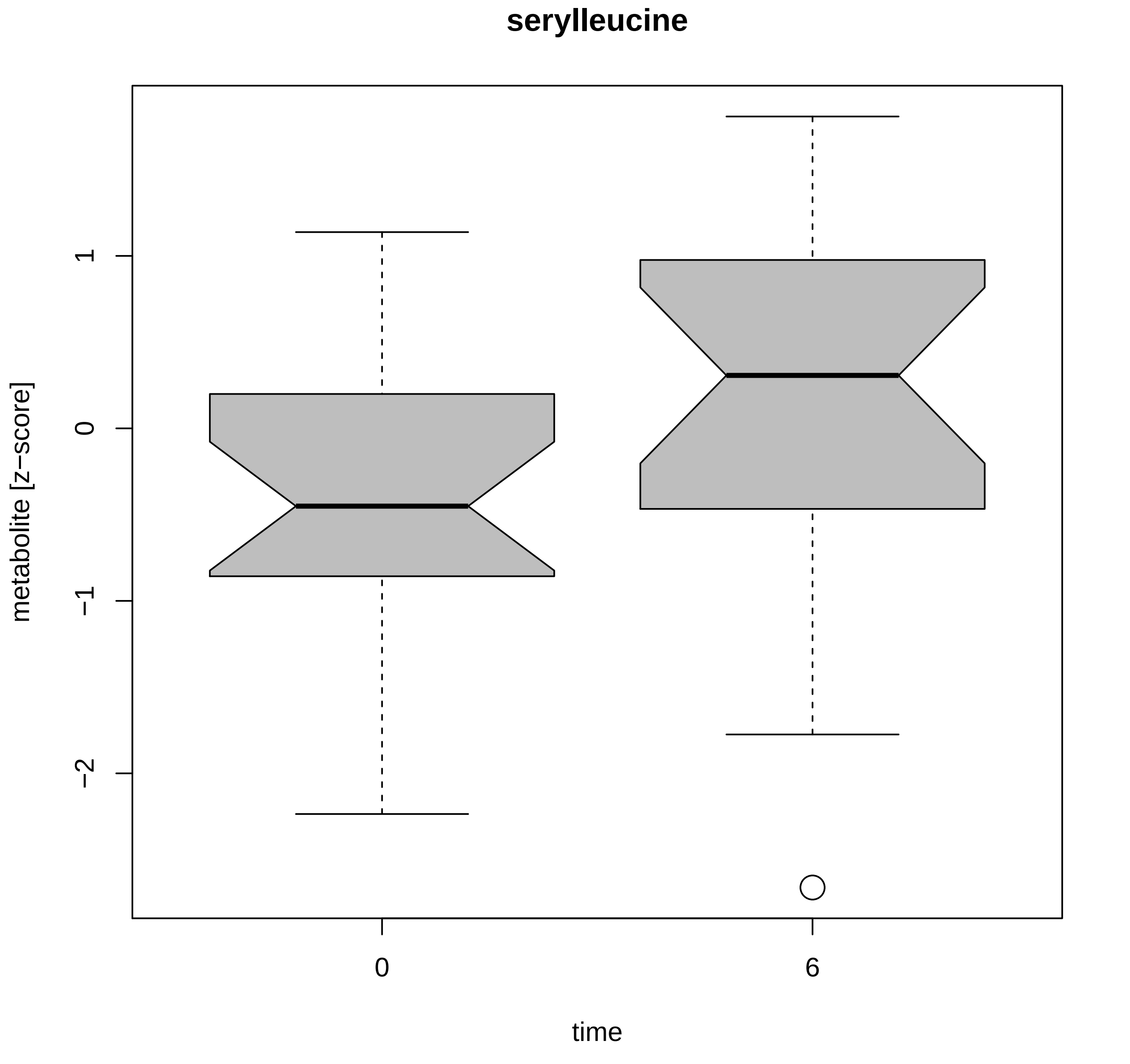
serylleucine
metabolite [z−score]
1
0
−1
−2
0
6
time

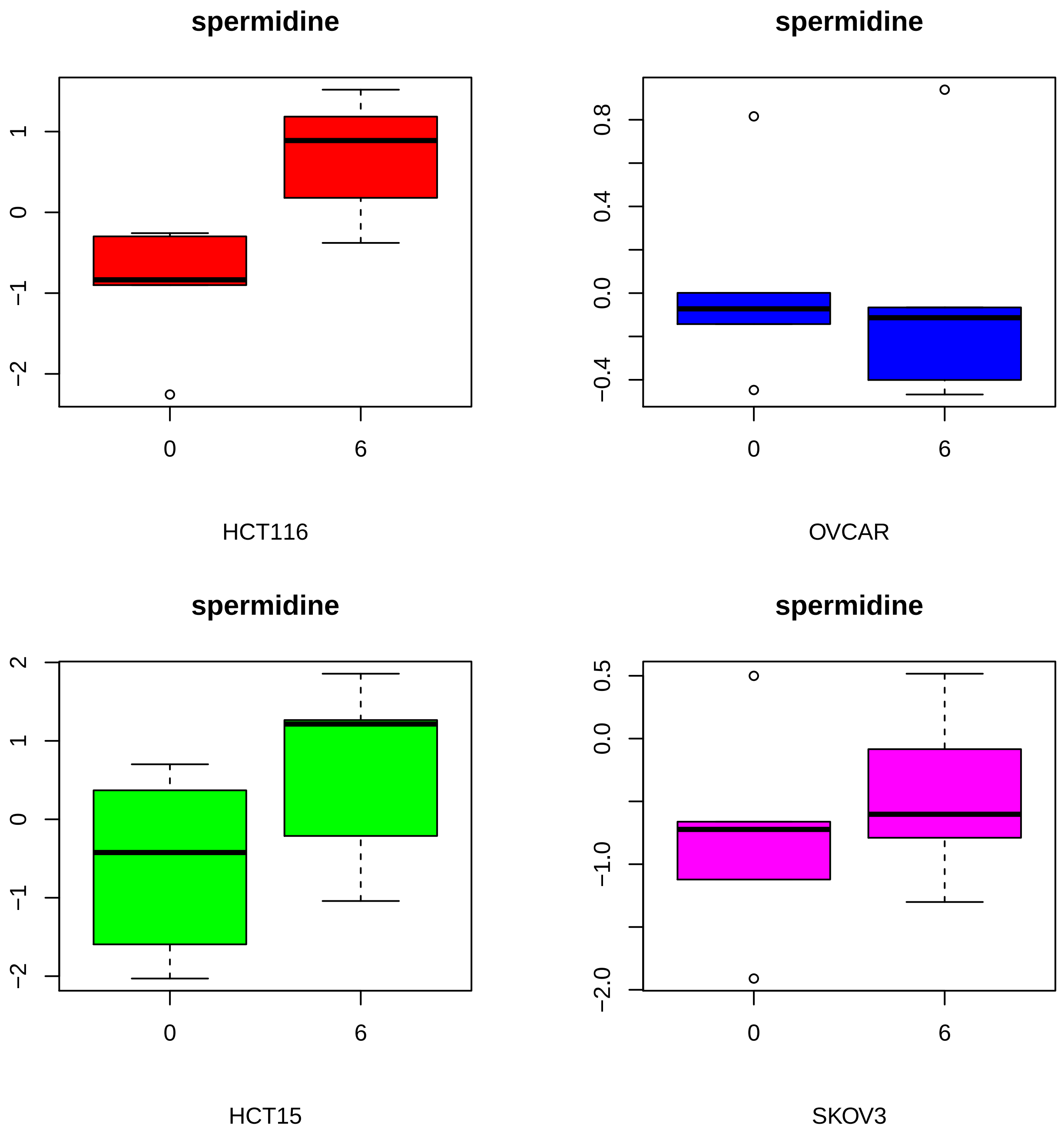
spermidine
1
0
−1
−2
0
6
HCT116
spermidine
0.8
0.4
0.0
−0.4
0
6
OVCAR
spermidine
2
1
0
−1
−2
0
6
HCT15
spermidine
0.5
0.0
−1.0
−2.0
0
6
SKOV3

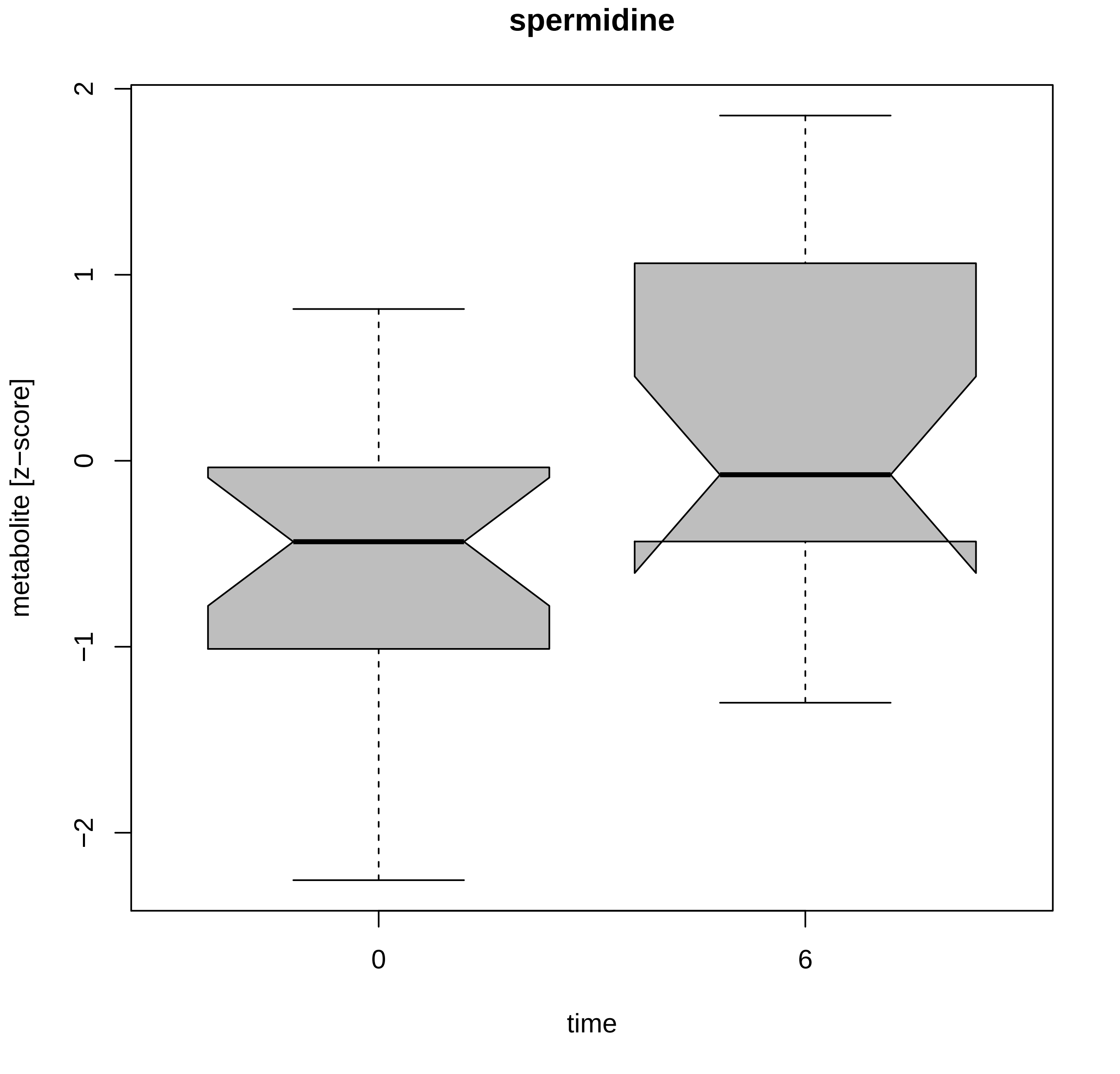

spermidine
metabolite [z−score]
2
1
0
−1
−2
0
6
time

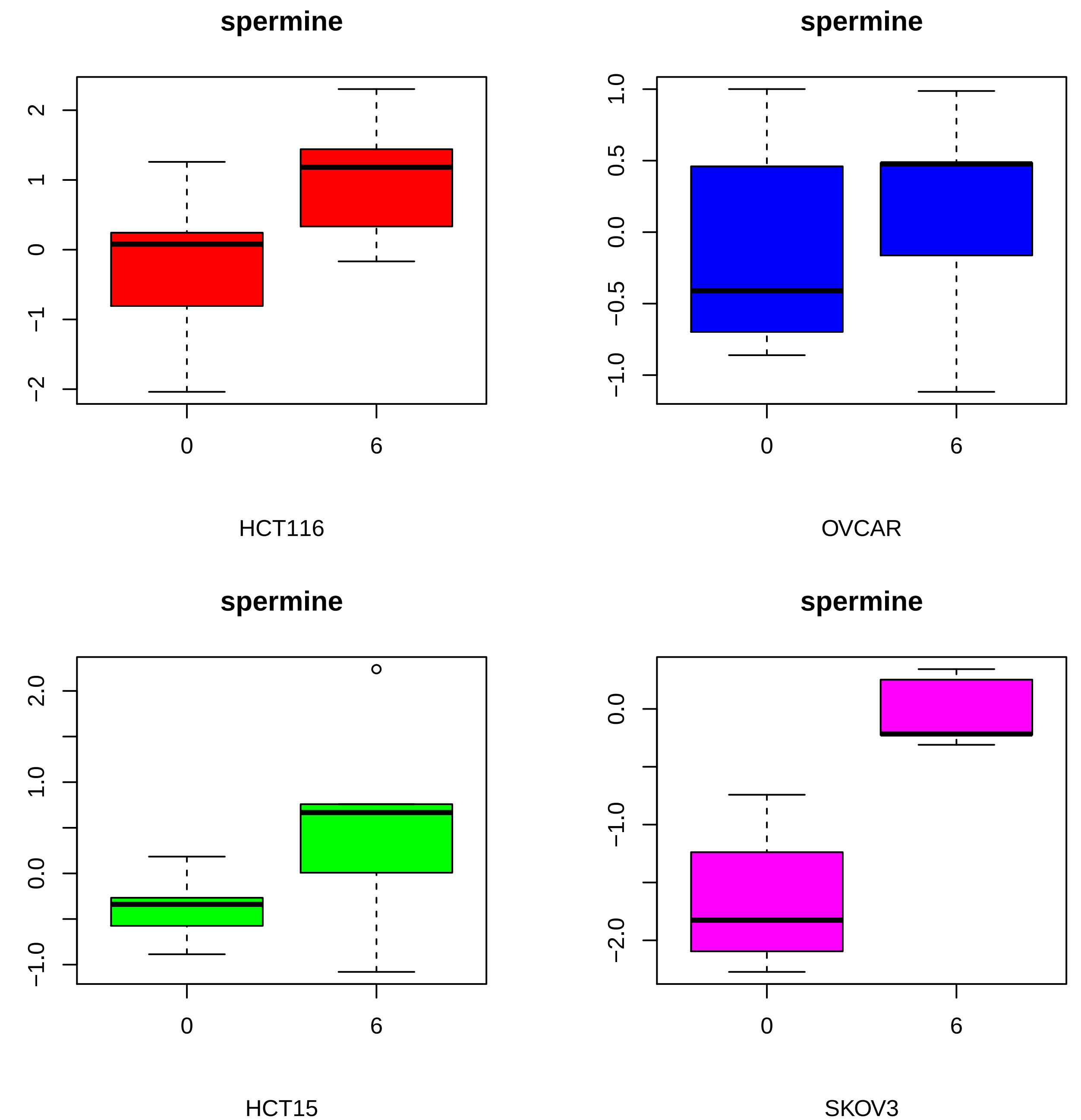
spermine
HCT116
0
6
−2
−1
0
1
2
spermine
OVCAR
0
6
−1.0
−0.5
0.0
0.5
1.0
spermine
HCT15
0
6
−1.0
0.0
1.0
2.0
spermine
SKOV3
0
6
−2.0
−1.0
0.0

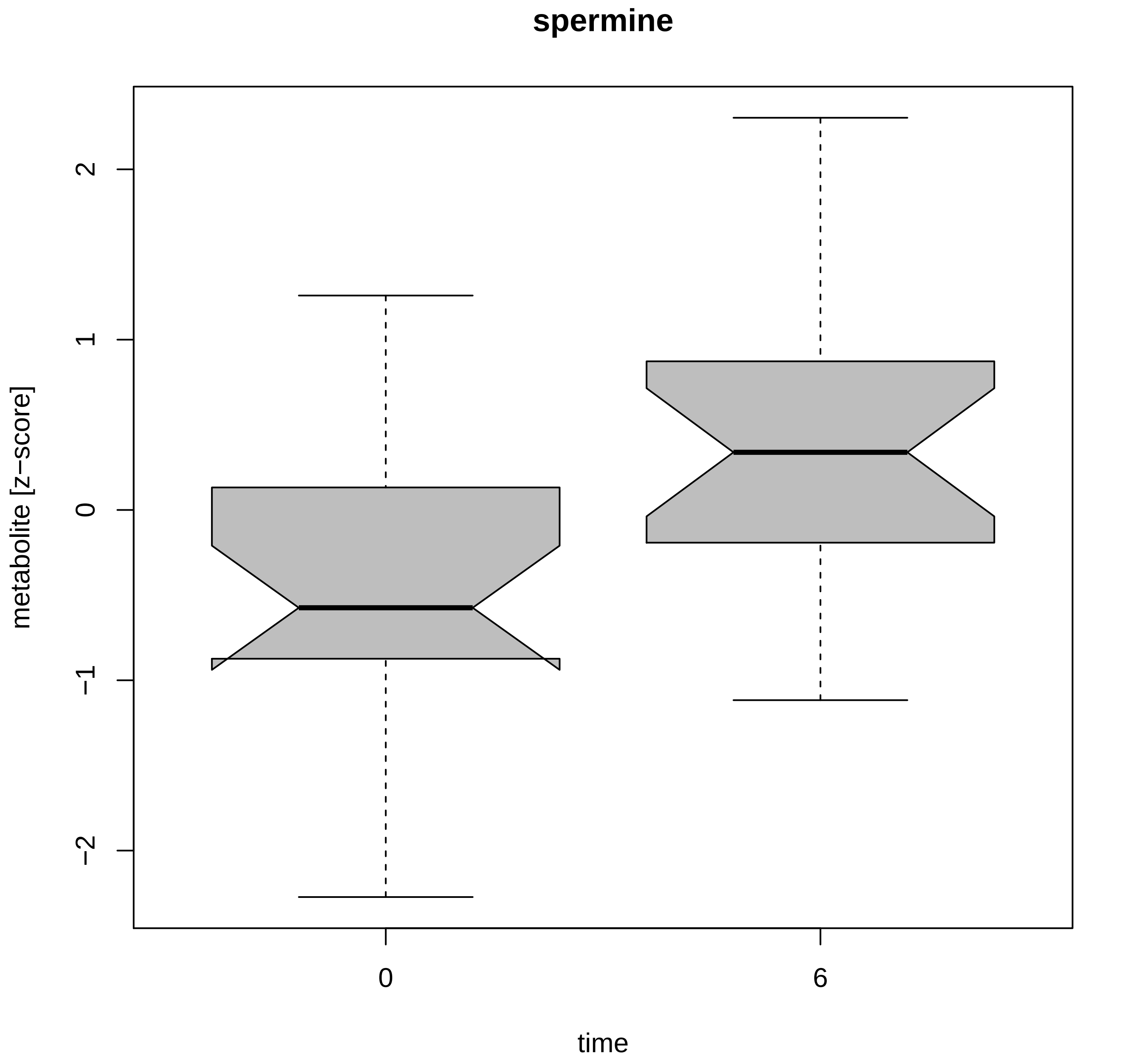
spermine
metabolite [z−score]
−2
−1
0
1
2
0
6
time

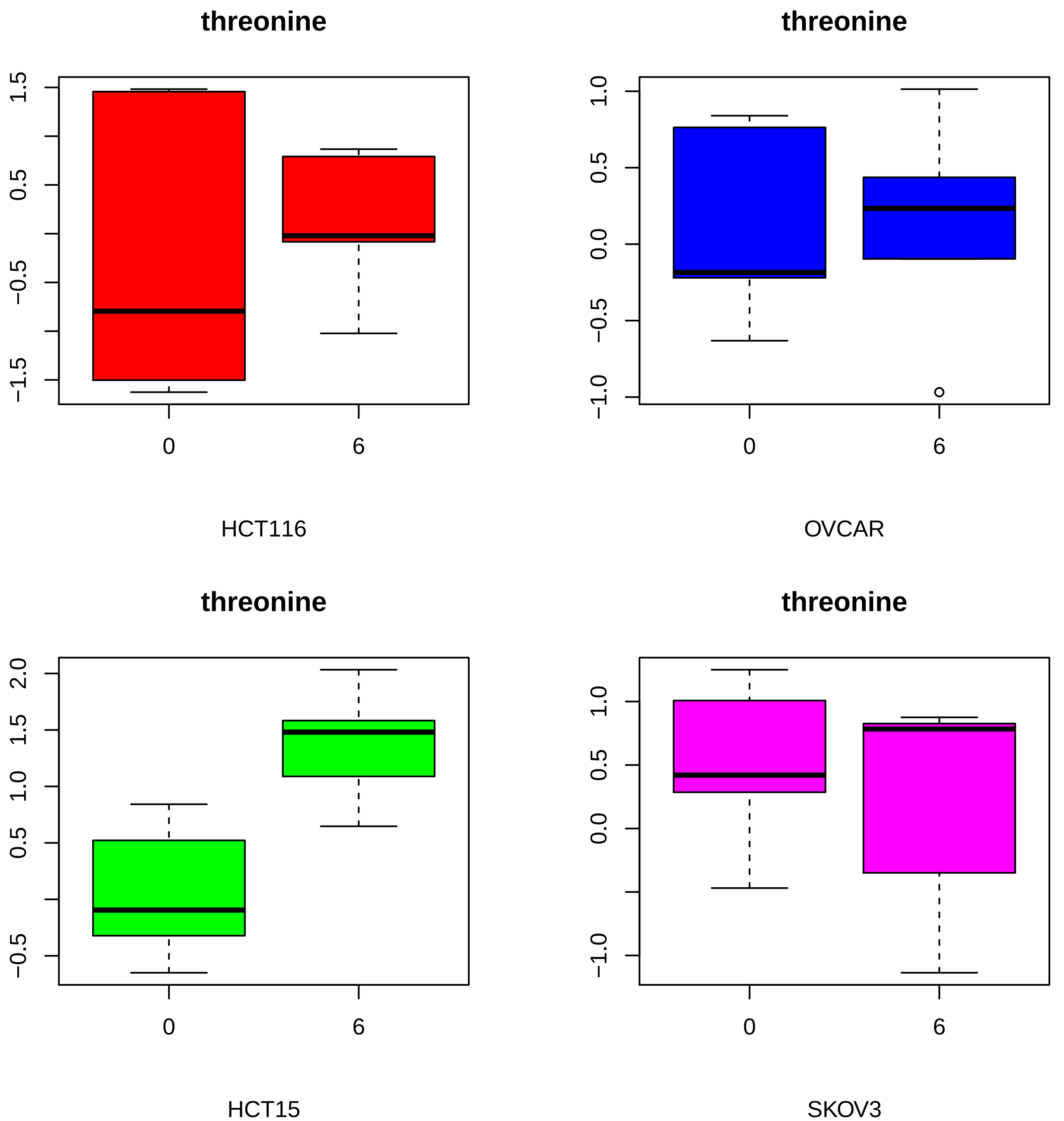
threonine
1.5
0.5
−0.5
−1.5
0
6
HCT116
threonine
1.0
0.5
0.0
−0.5
−1.0
0
6
OVCAR
threonine
2.0
1.5
1.0
0.5
−0.5
0
6
HCT15
threonine
1.0
0.5
0.0
−1.0
0
6
SKOV3

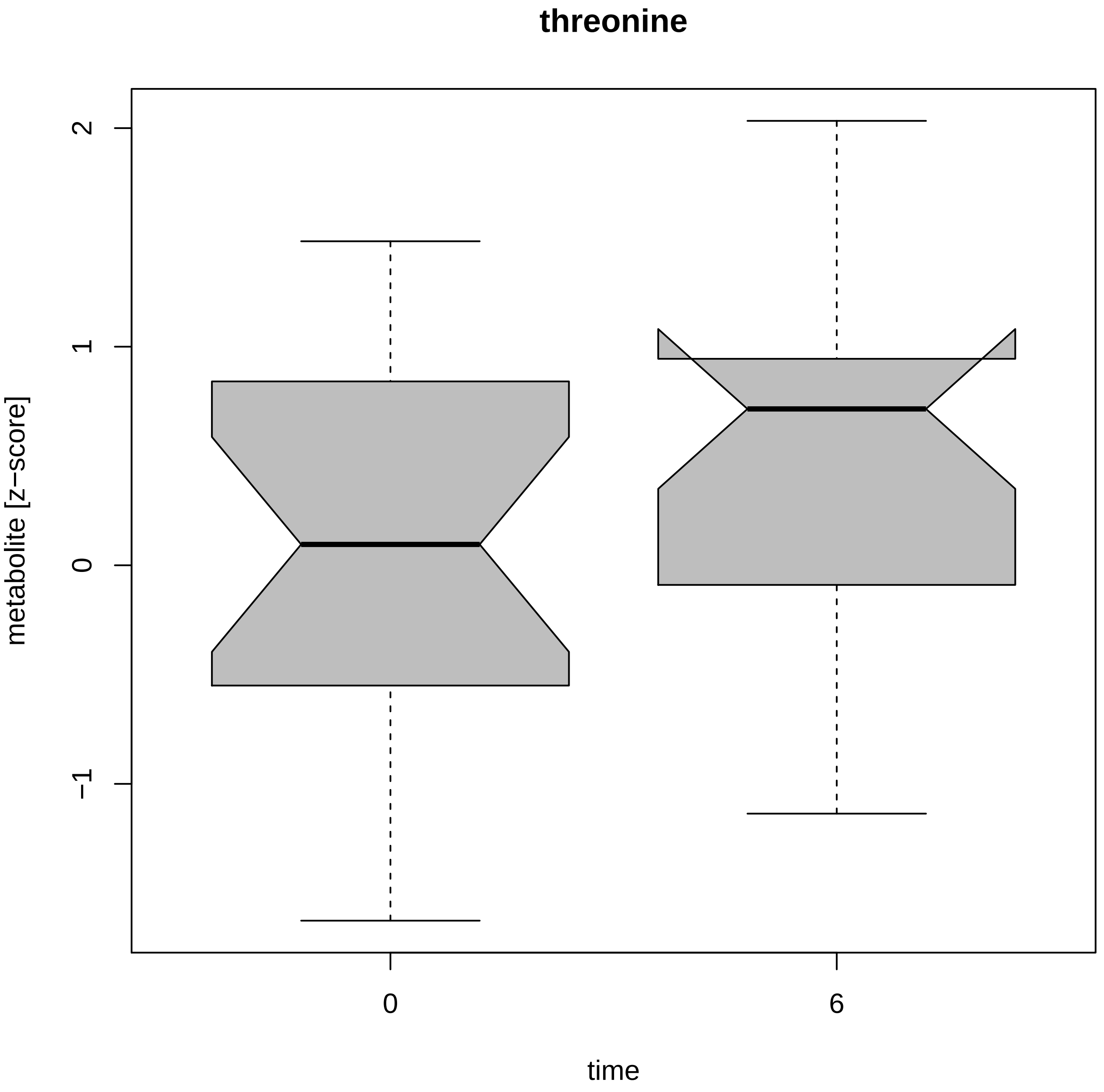
threonine
2
1
0
−1
metabolite [z−score]
0
6
time

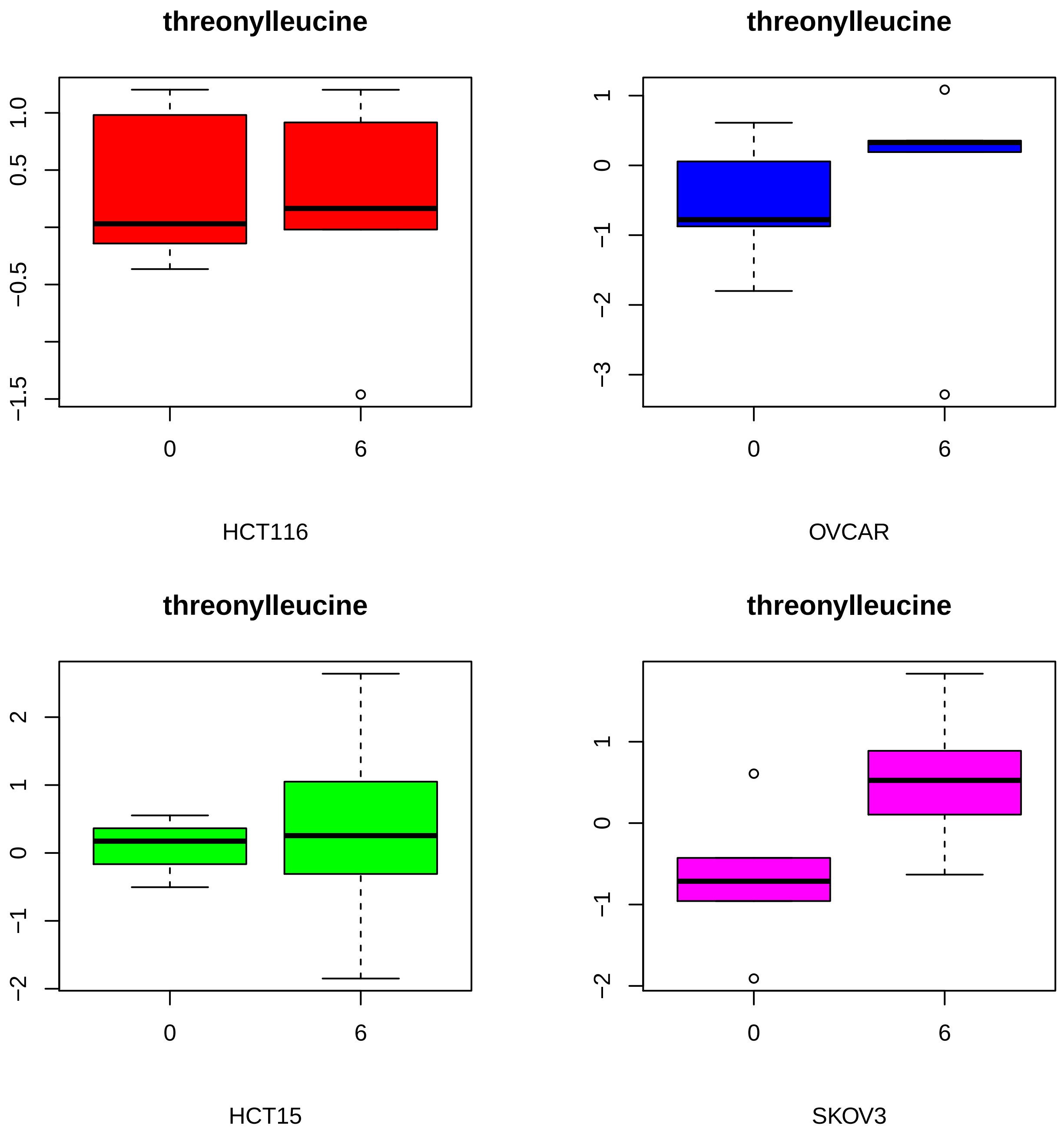
threonylleucine
1.0
0.5
−0.5
−1.5
0
6
HCT116
threonylleucine
1
0
−1
−2
−3
0
6
OVCAR
threonylleucine
2
1
0
−1
−2
0
6
HCT15
threonylleucine
1
0
−1
−2
0
6
SKOV3

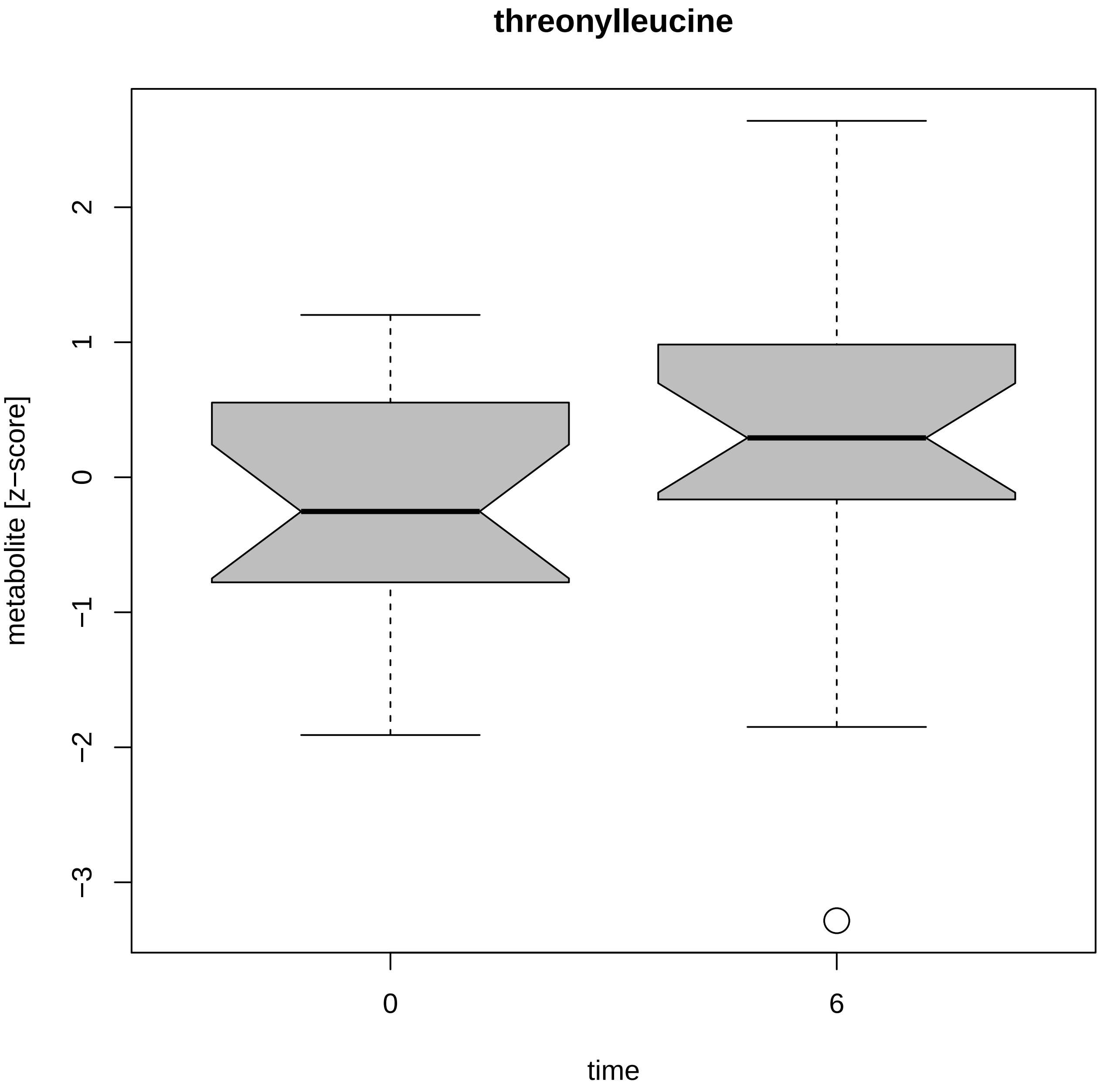
threonylleucine
metabolite [z−score]
2
1
0
−1
−2
−3
0
6
time

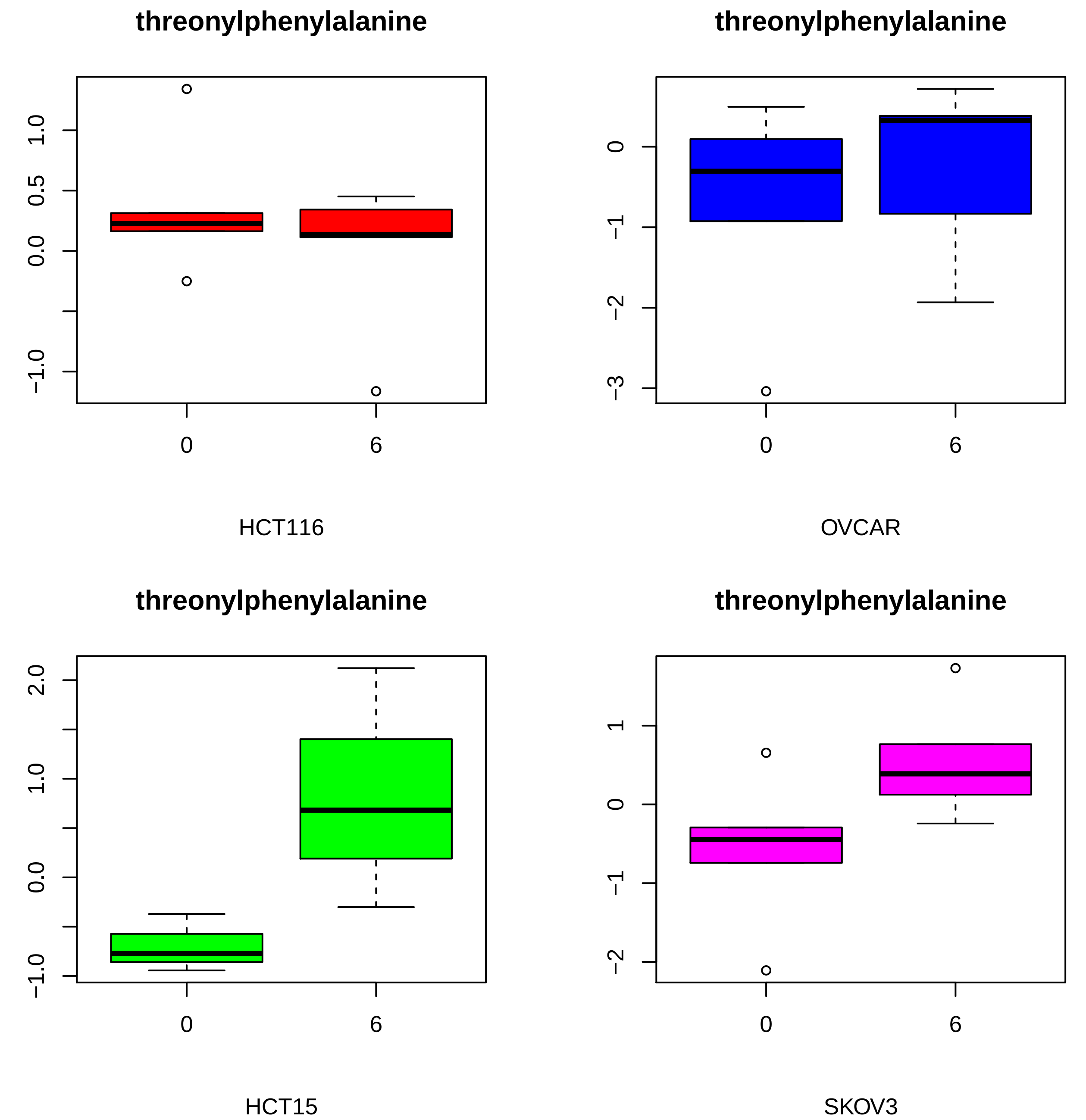

threonylphenylalanine
1.0
0.5
0.0
−1.0
0
6
HCT116
threonylphenylalanine
0
−1
−2
−3
0
6
OVCAR
threonylphenylalanine
2.0
1.0
0.0
−1.0
0
6
HCT15
threonylphenylalanine
1
0
−1
−2
0
6
SKOV3

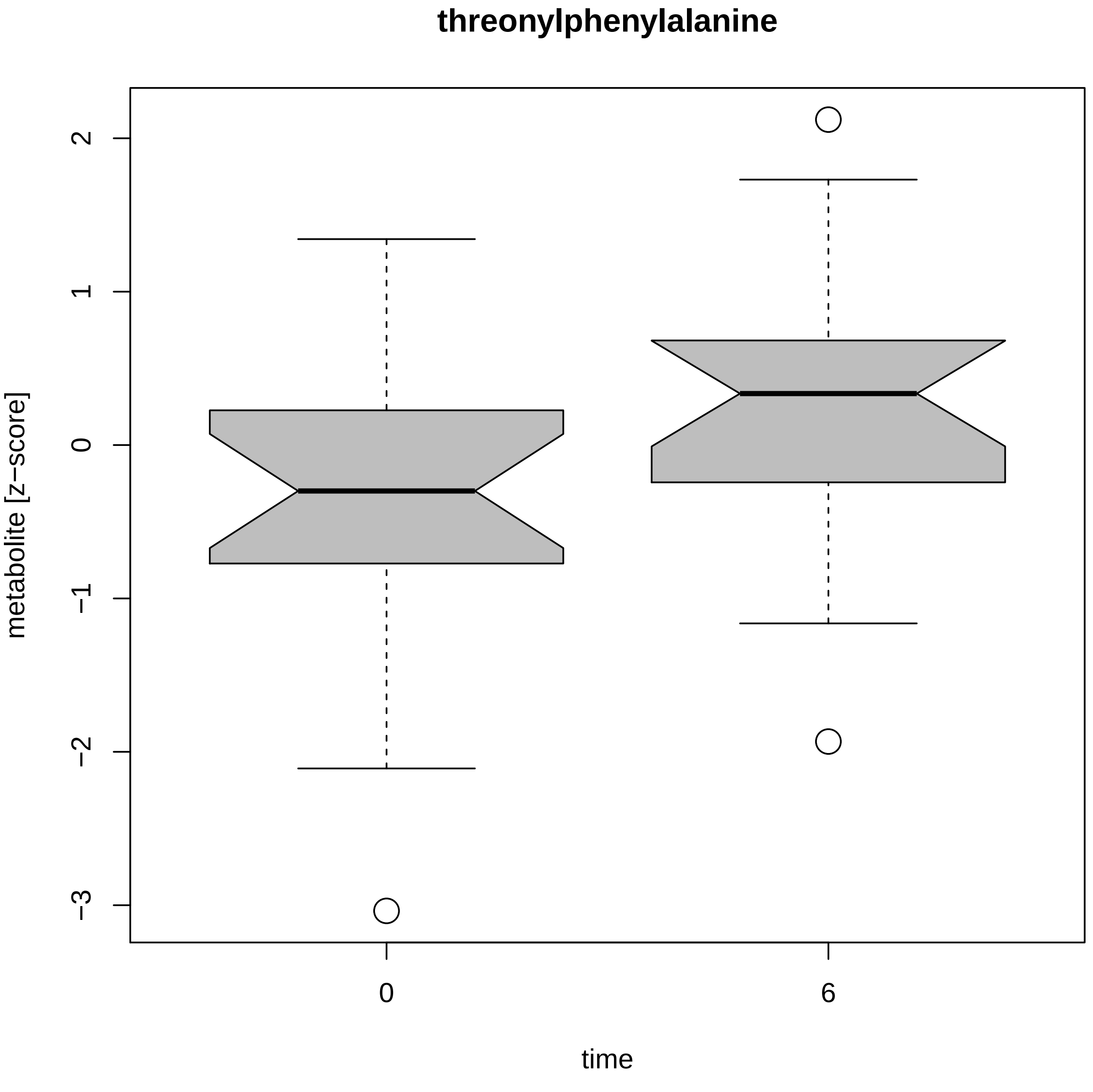
threonylphenylalanine
metabolite [z−score]
2
1
0
−1
−2
−3
0
6
time

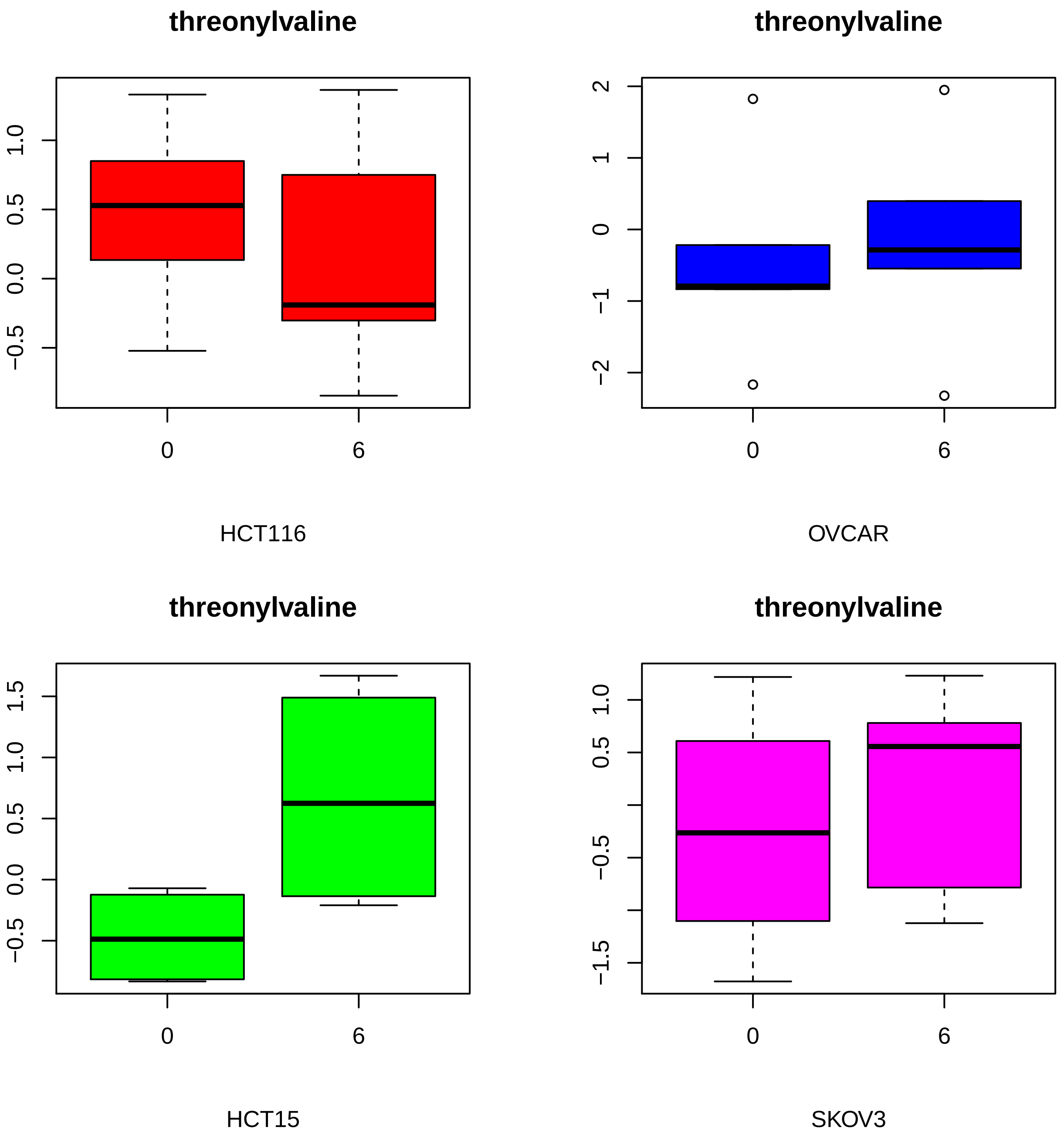
threonylvaline
1.0
0.5
0.0
−0.5
0
6
HCT116
threonylvaline
2
1
0
−1
−2
0
6
OVCAR
threonylvaline
1.5
1.0
0.5
0.0
−0.5
0
6
HCT15
threonylvaline
1.0
0.5
−0.5
−1.5
0
6
SKOV3

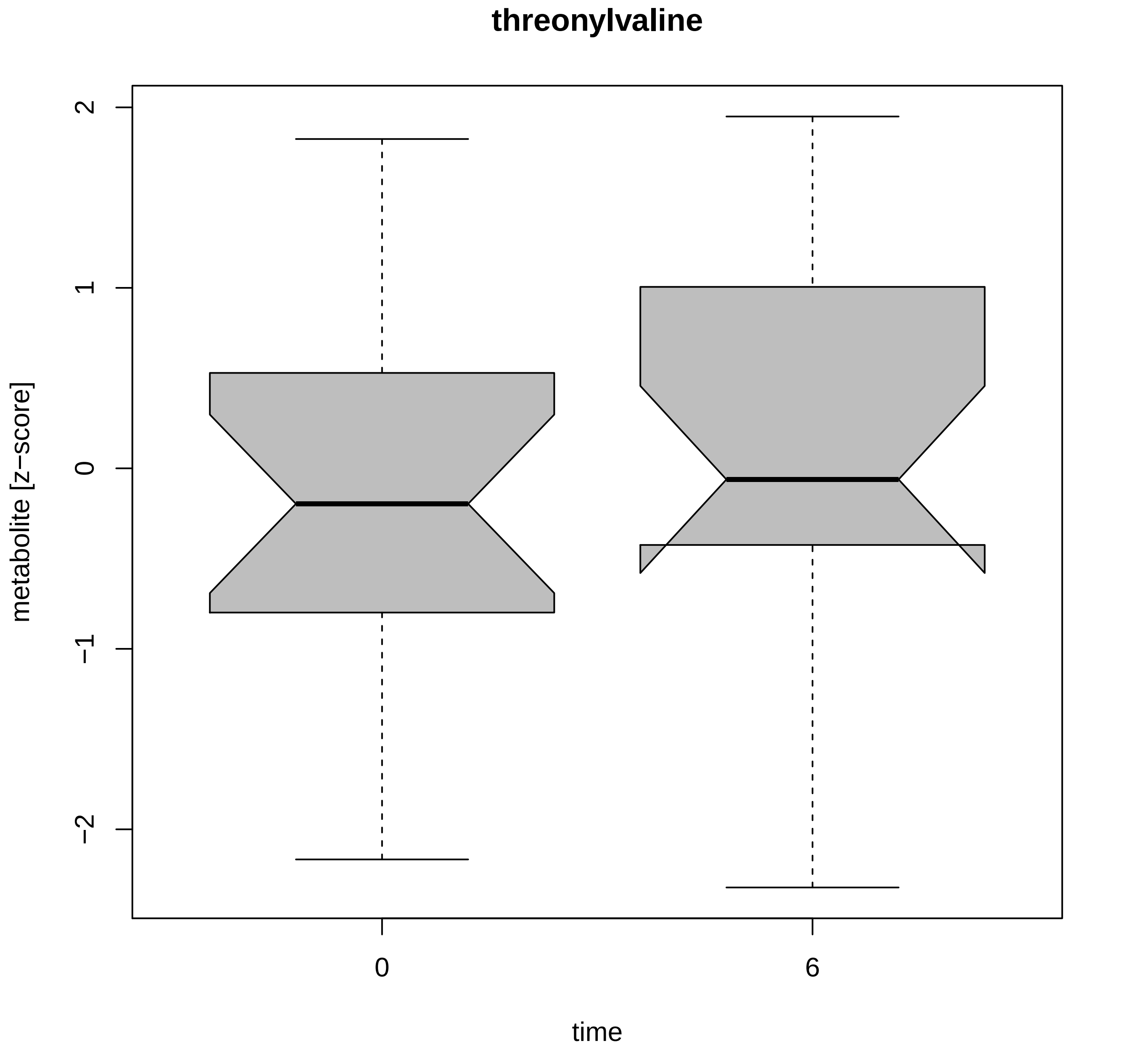
threonylvaline
metabolite [z−score]
2
1
0
−1
−2
0
6
time

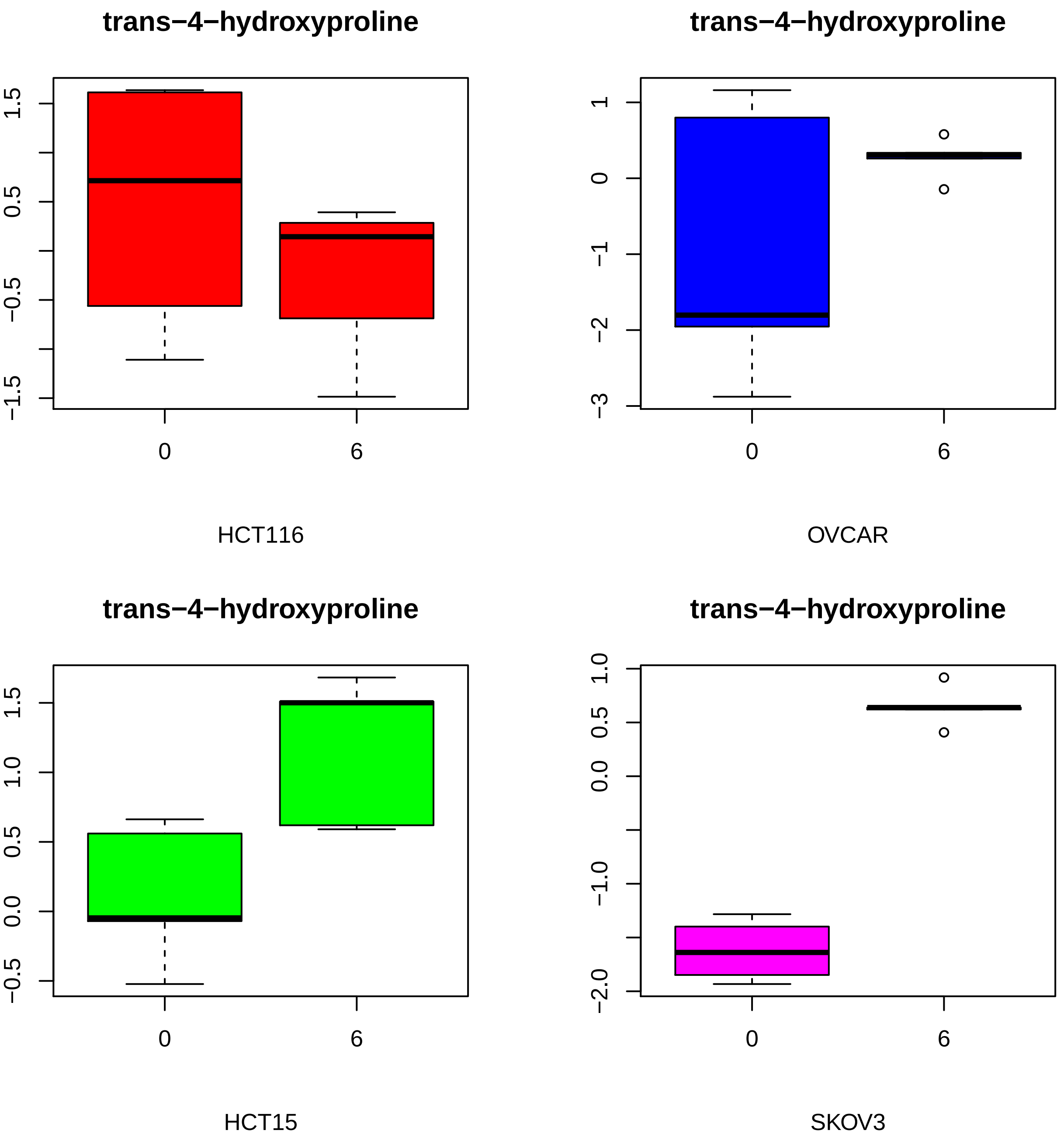
trans−4−hydroxyproline
1.5
0.5
−0.5
−1.5
0
6
HCT116
trans−4−hydroxyproline
1
0
−1
−2
−3
0
6
OVCAR
trans−4−hydroxyproline
1.5
1.0
0.5
0.0
−0.5
0
6
HCT15
trans−4−hydroxyproline
1.0
0.5
0.0
−1.0
−2.0
0
6
SKOV3

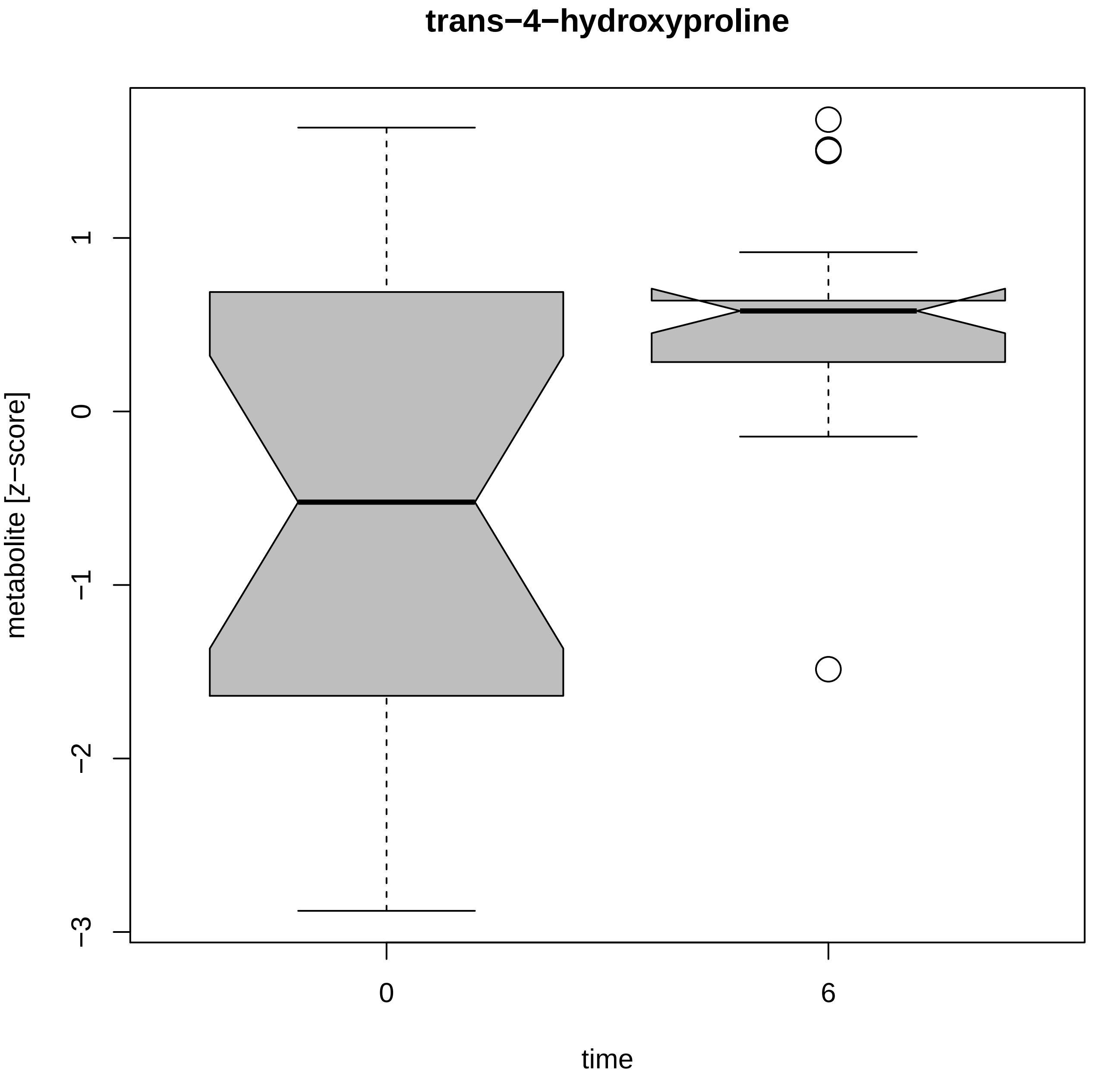
trans−4−hydroxyproline
metabolite [z−score]
time
0
6
1
0
−1
−2
−3

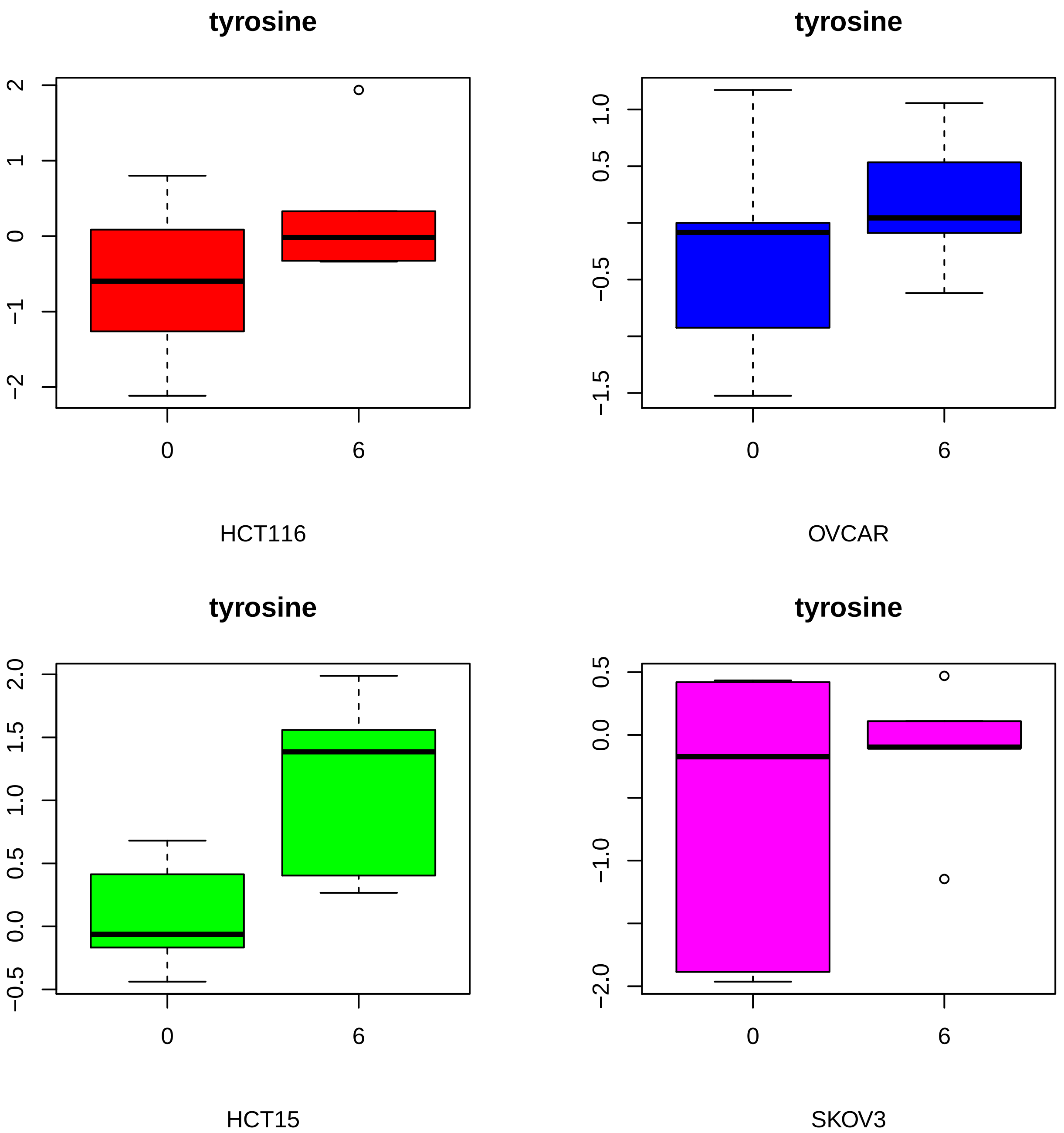
tyrosine
2
1
0
−1
−2
0
6
HCT116
tyrosine
1.0
0.5
−0.5
−1.5
0
6
OVCAR
tyrosine
2.0
1.5
1.0
0.5
0.0
−0.5
0
6
HCT15
tyrosine
0.5
0.0
−1.0
−2.0
0
6
SKOV3

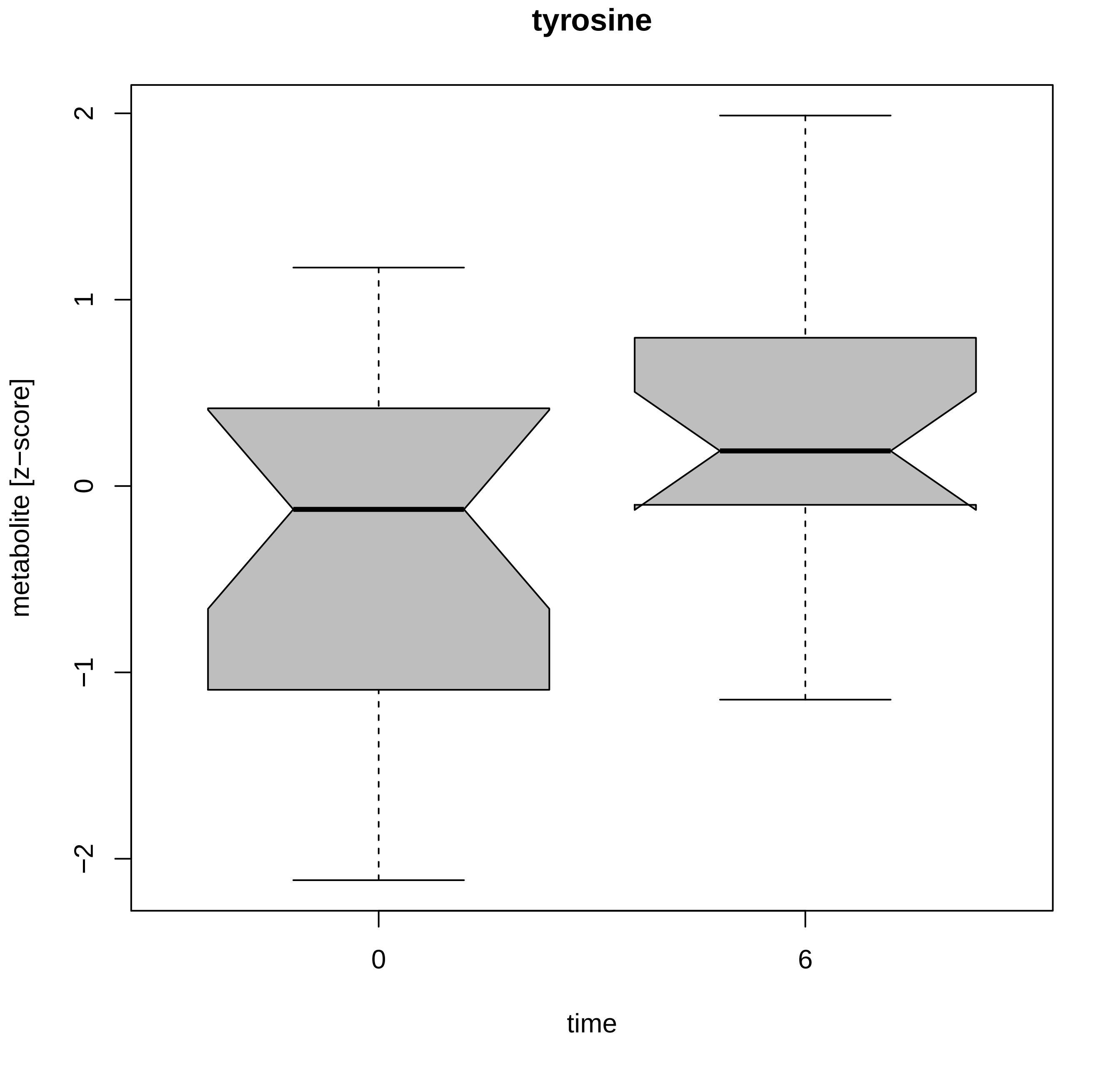
tyrosine
metabolite [z−score]
2
1
0
−1
−2
0
6
time

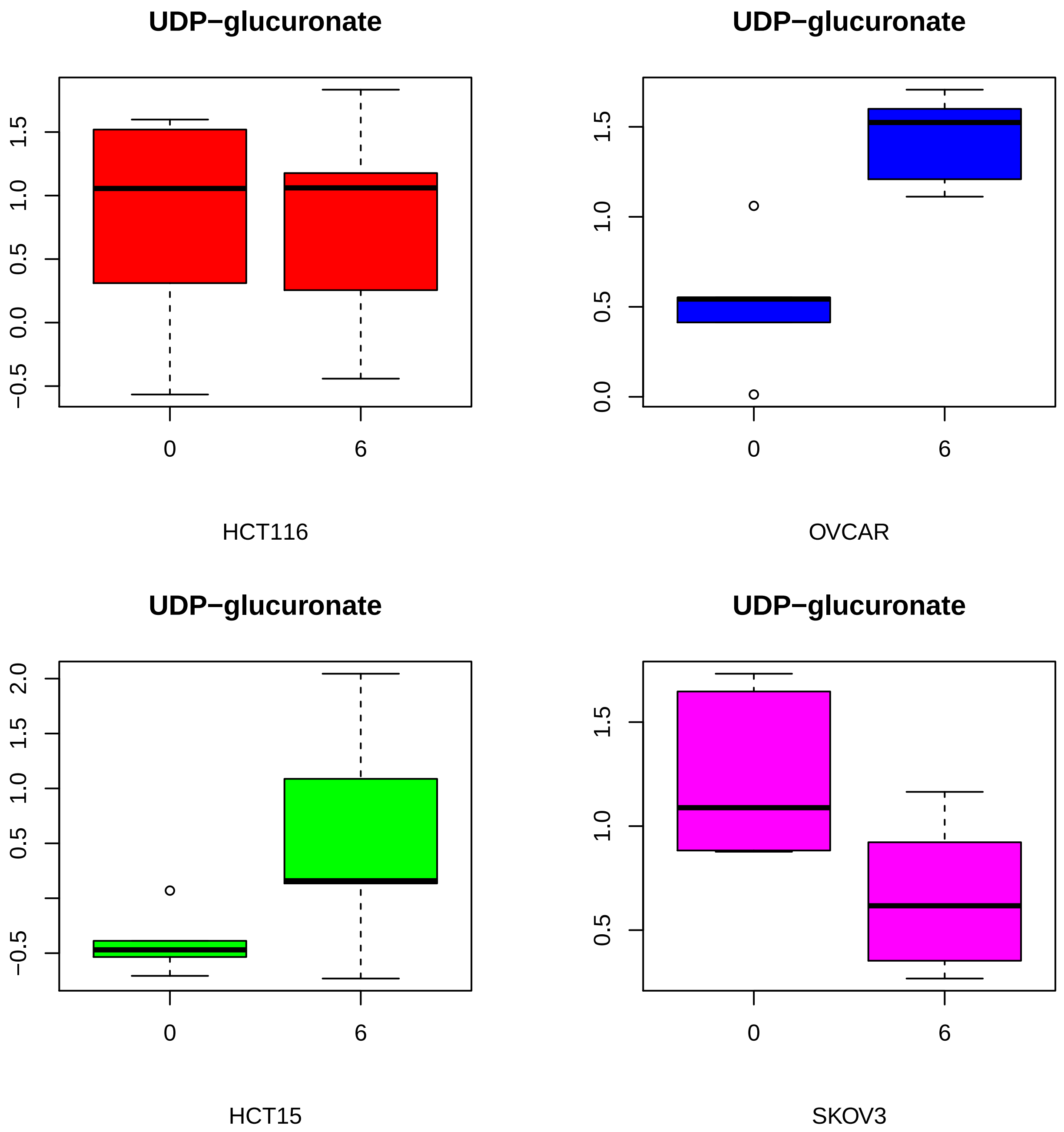
UDP−glucuronate
1.5
1.0
0.5
0.0
−0.5
0
6
HCT116
UDP−glucuronate
1.5
1.0
0.5
0.0
0
6
OVCAR
UDP−glucuronate
2.0
1.5
1.0
0.5
−0.5
0
6
HCT15
UDP−glucuronate
1.5
1.0
0.5
0
6
SKOV3

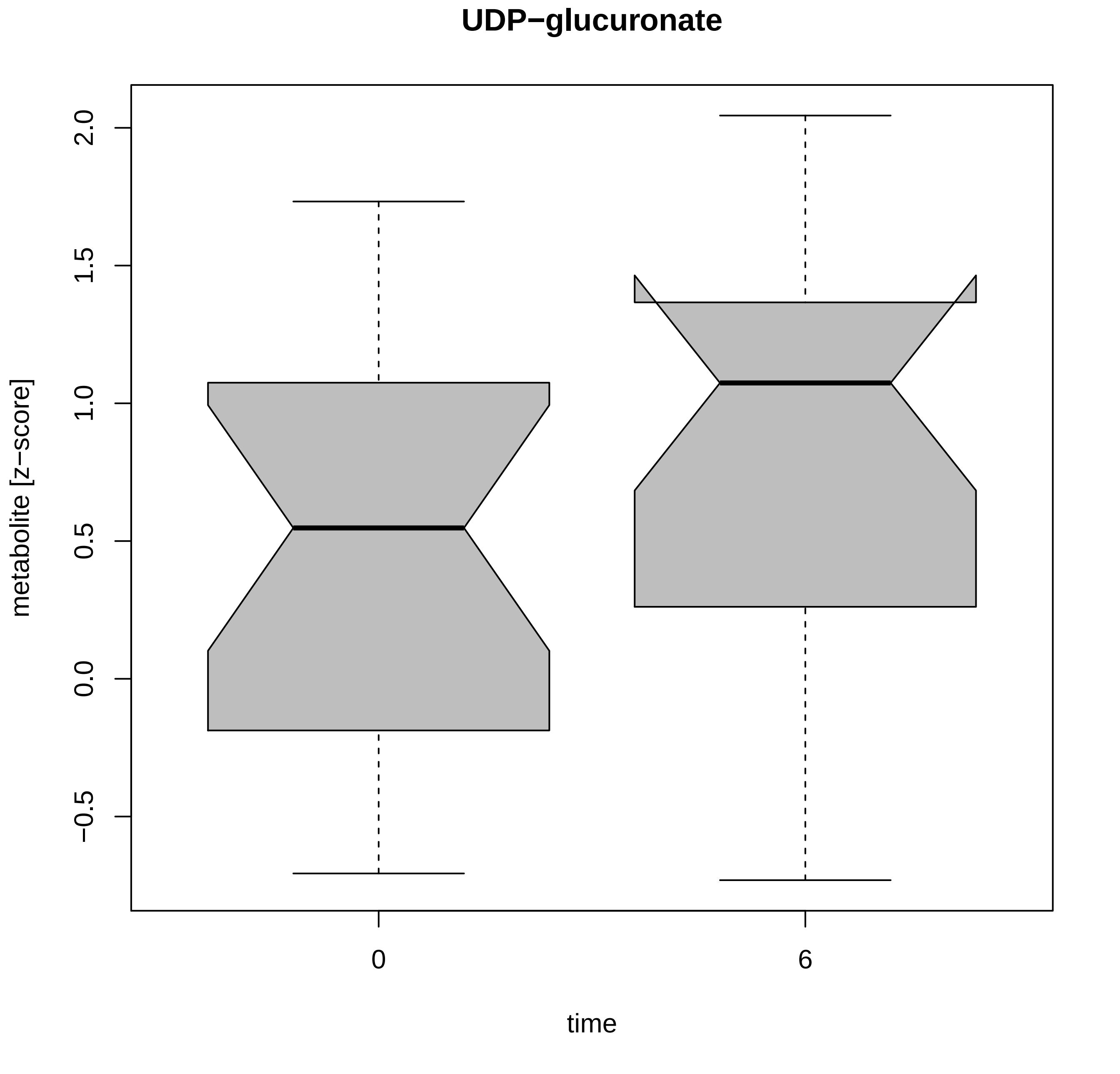

UDP−glucuronate
metabolite [z−score]
2.0
1.5
1.0
0.5
0.0
−0.5
0
6
time

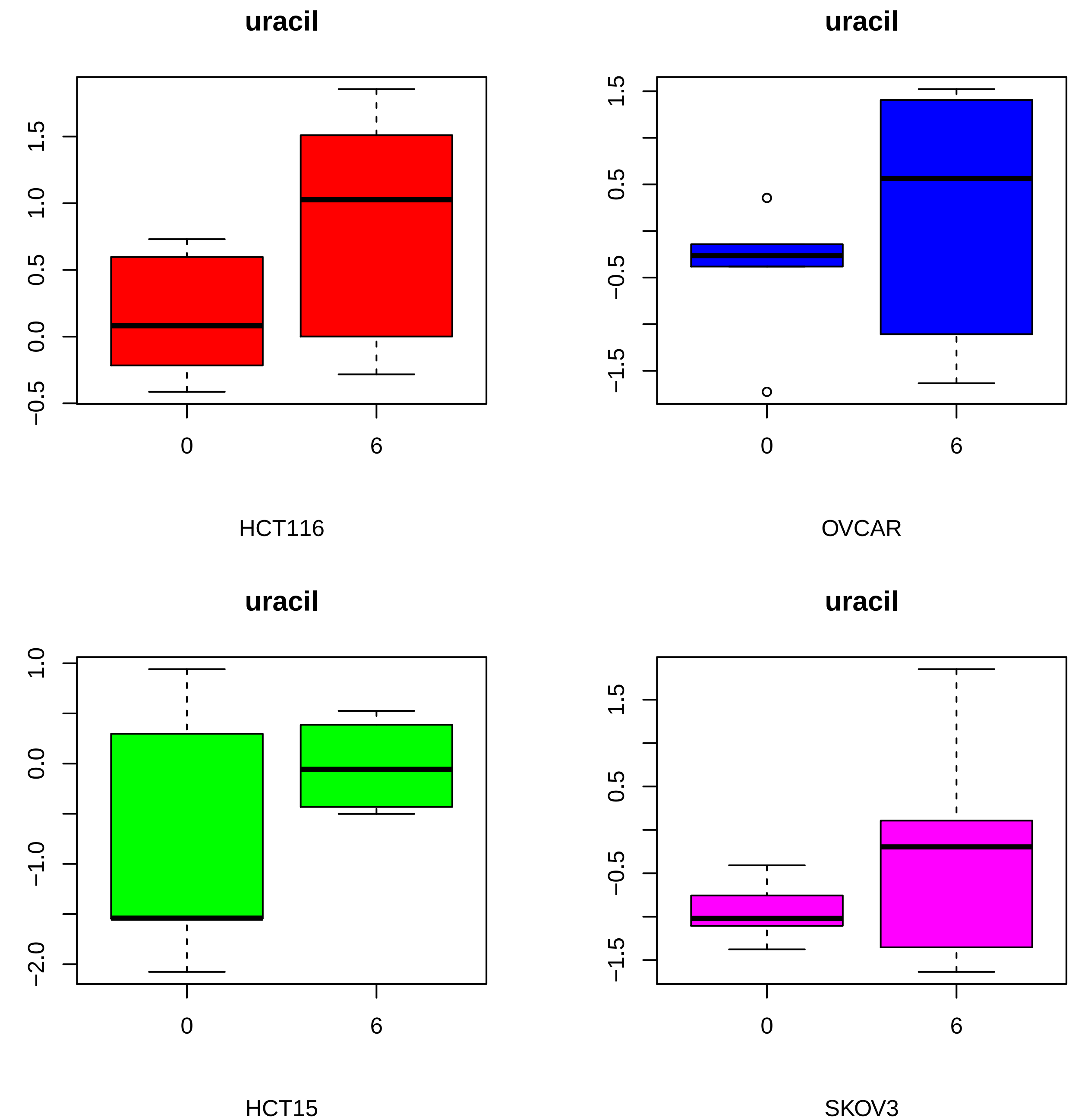
uracil
1.5
1.0
0.5
0.0
−0.5
0
6
HCT116
uracil
1.5
0.5
−0.5
−1.5
0
6
OVCAR
uracil
1.0
0.0
−1.0
−2.0
0
6
HCT15
uracil
1.5
0.5
−0.5
−1.5
0
6
SKOV3

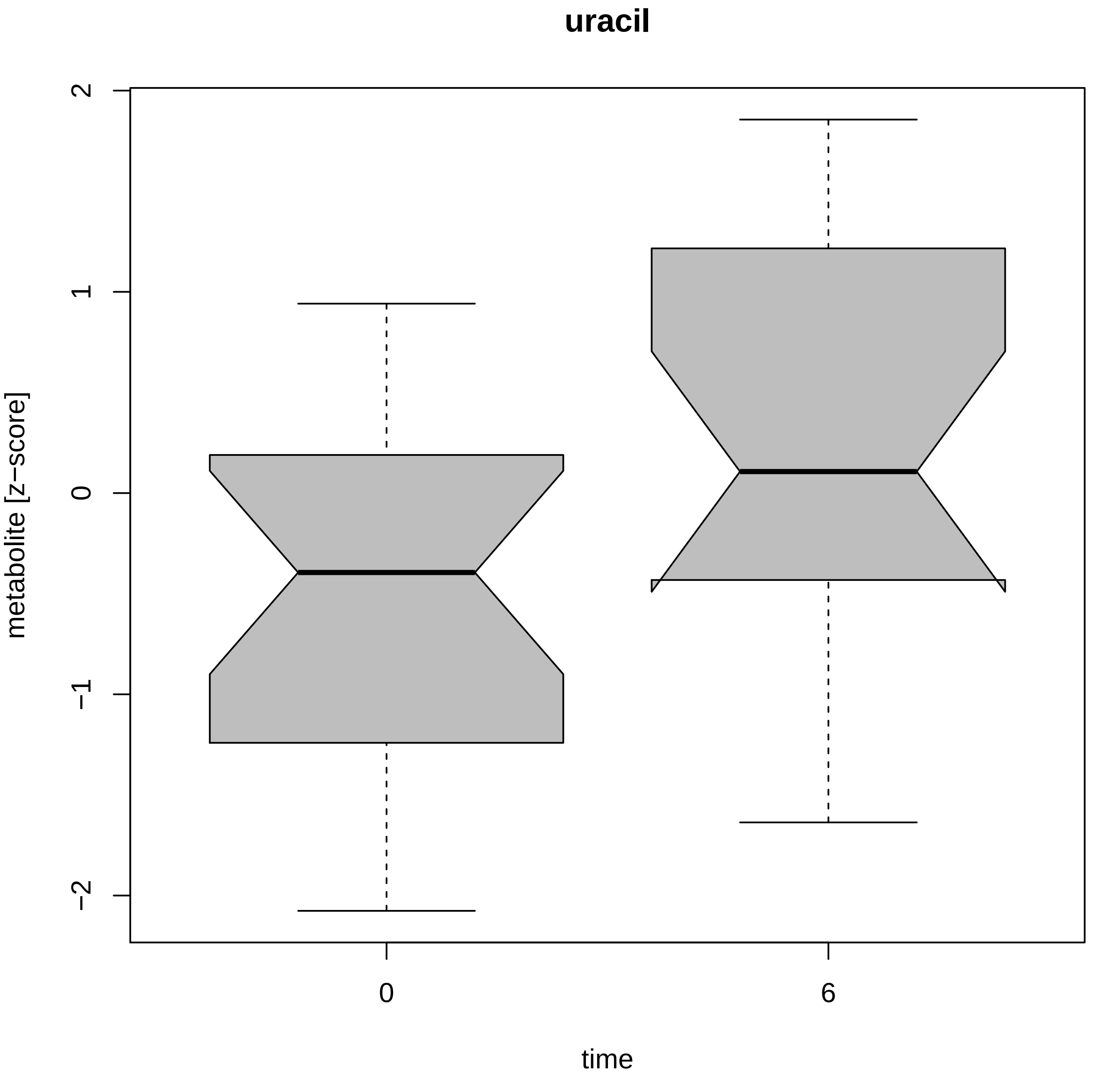

uracil
metabolite [z−score]
2
1
0
−1
−2
0
6
time

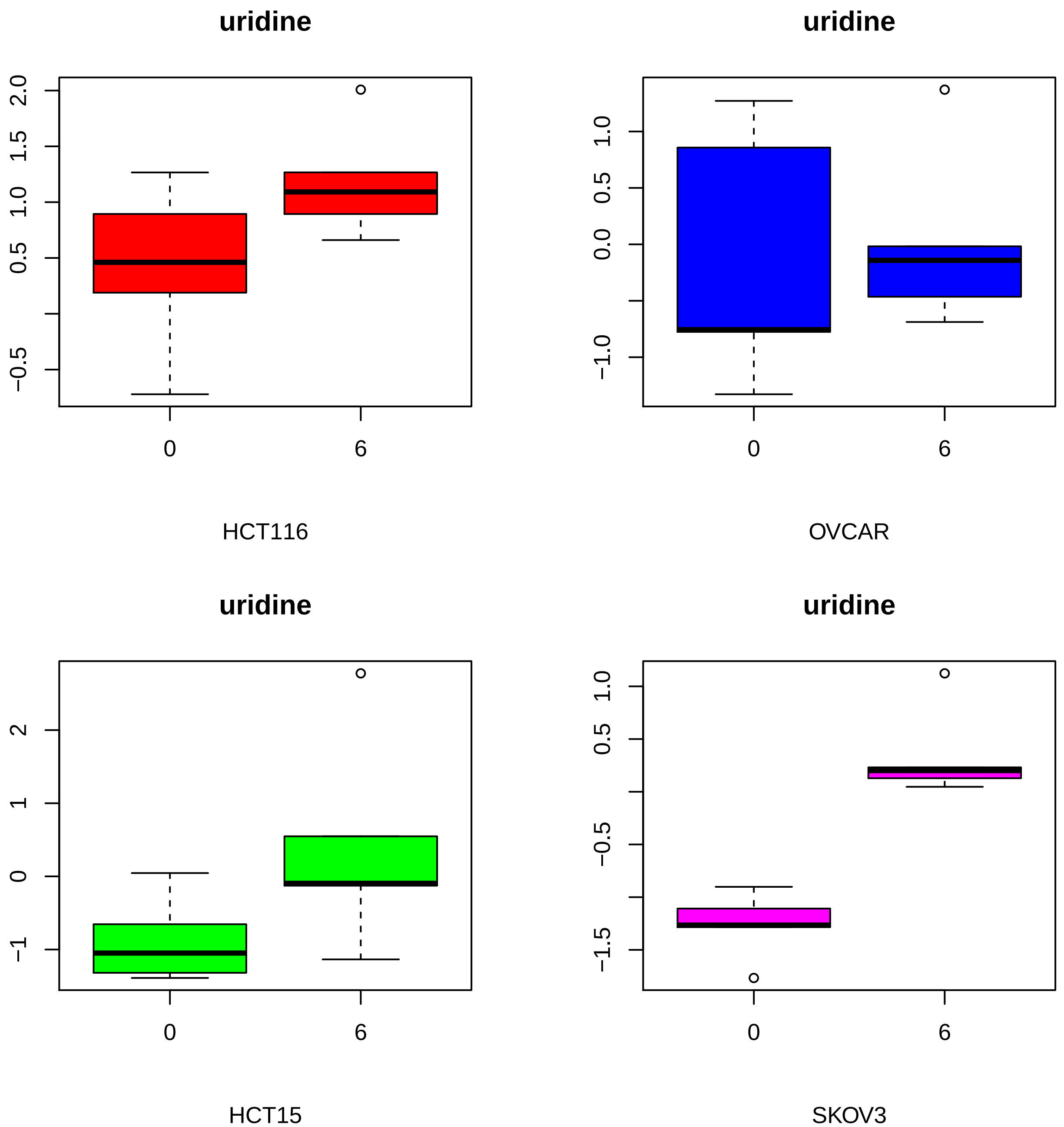
uridine
2.0
1.5
1.0
0.5
−0.5
0
6
HCT116
uridine
1.0
0.5
0.0
−1.0
0
6
OVCAR
uridine
2
1
0
−1
0
6
HCT15
uridine
1.0
0.5
−0.5
−1.5
0
6
SKOV3

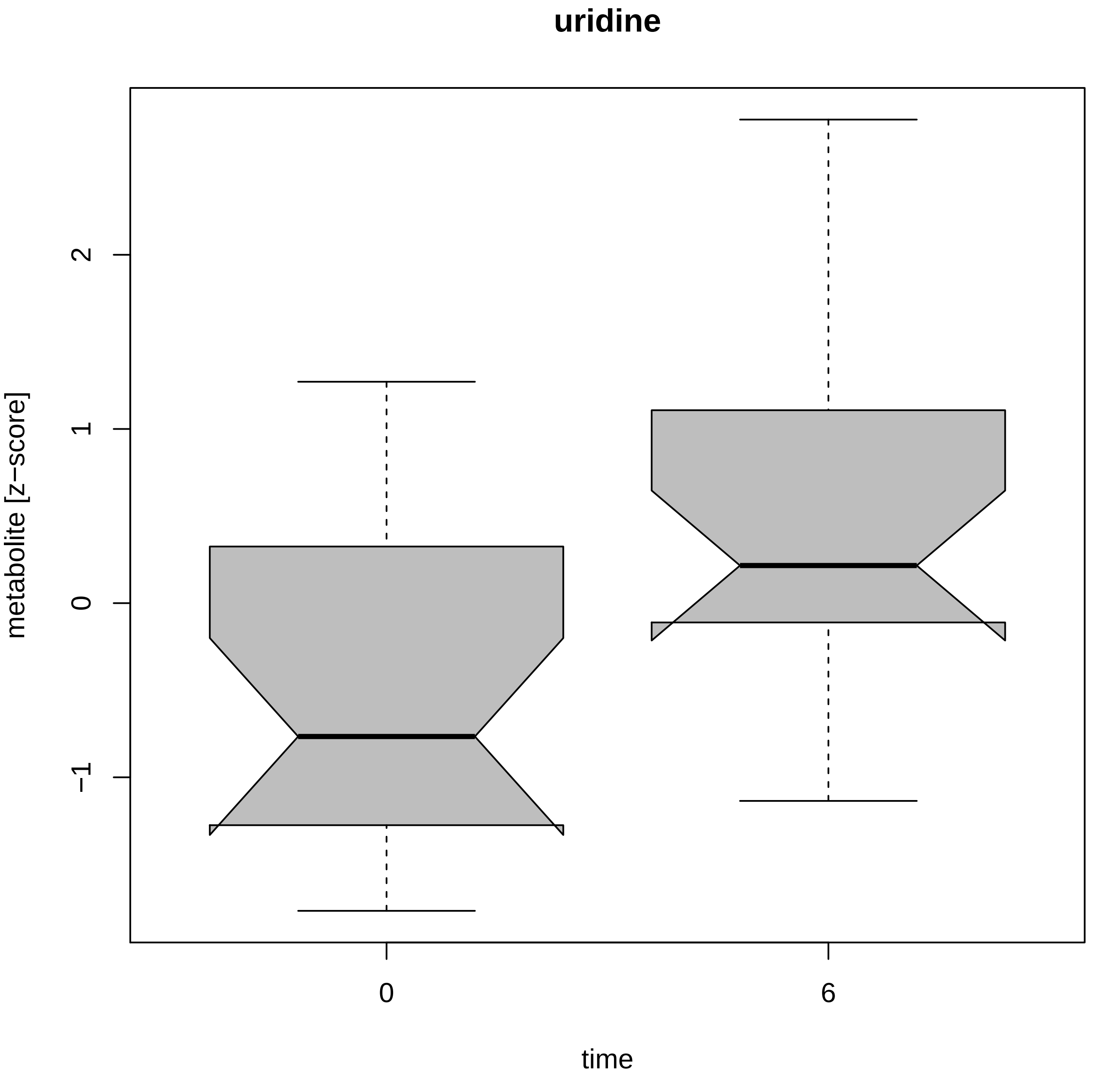

uridine
metabolite [z−score]
2
1
0
−1
0
6
time

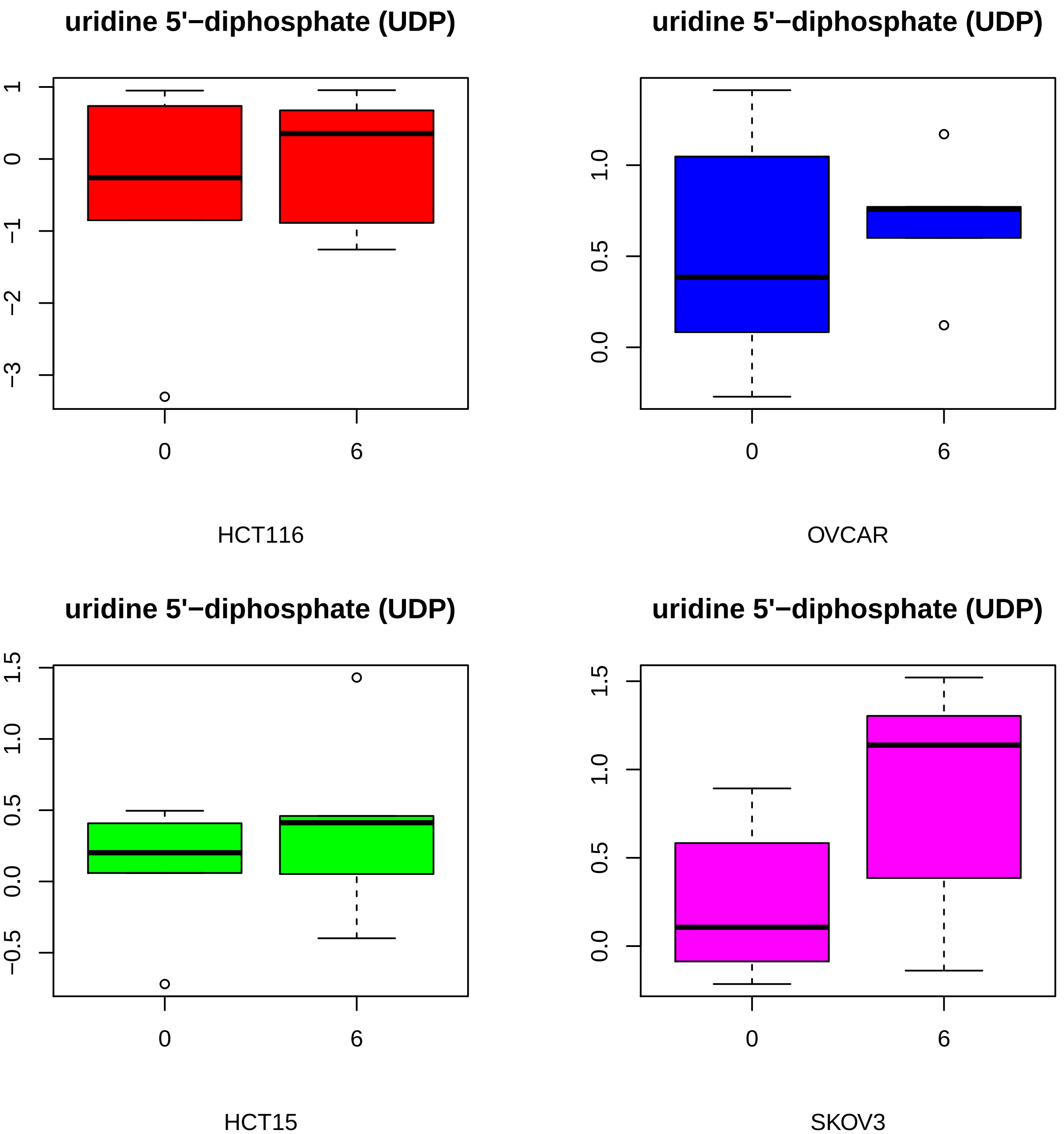
uridine 5'−diphosphate (UDP)
1
0
−1
−2
−3
0
6
HCT116
uridine 5'−diphosphate (UDP)
1.0
0.5
0.0
0
6
OVCAR
uridine 5'−diphosphate (UDP)
1.5
1.0
0.5
0.0
−0.5
0
6
HCT15
uridine 5'−diphosphate (UDP)
1.5
1.0
0.5
0.0
0
6
SKOV3

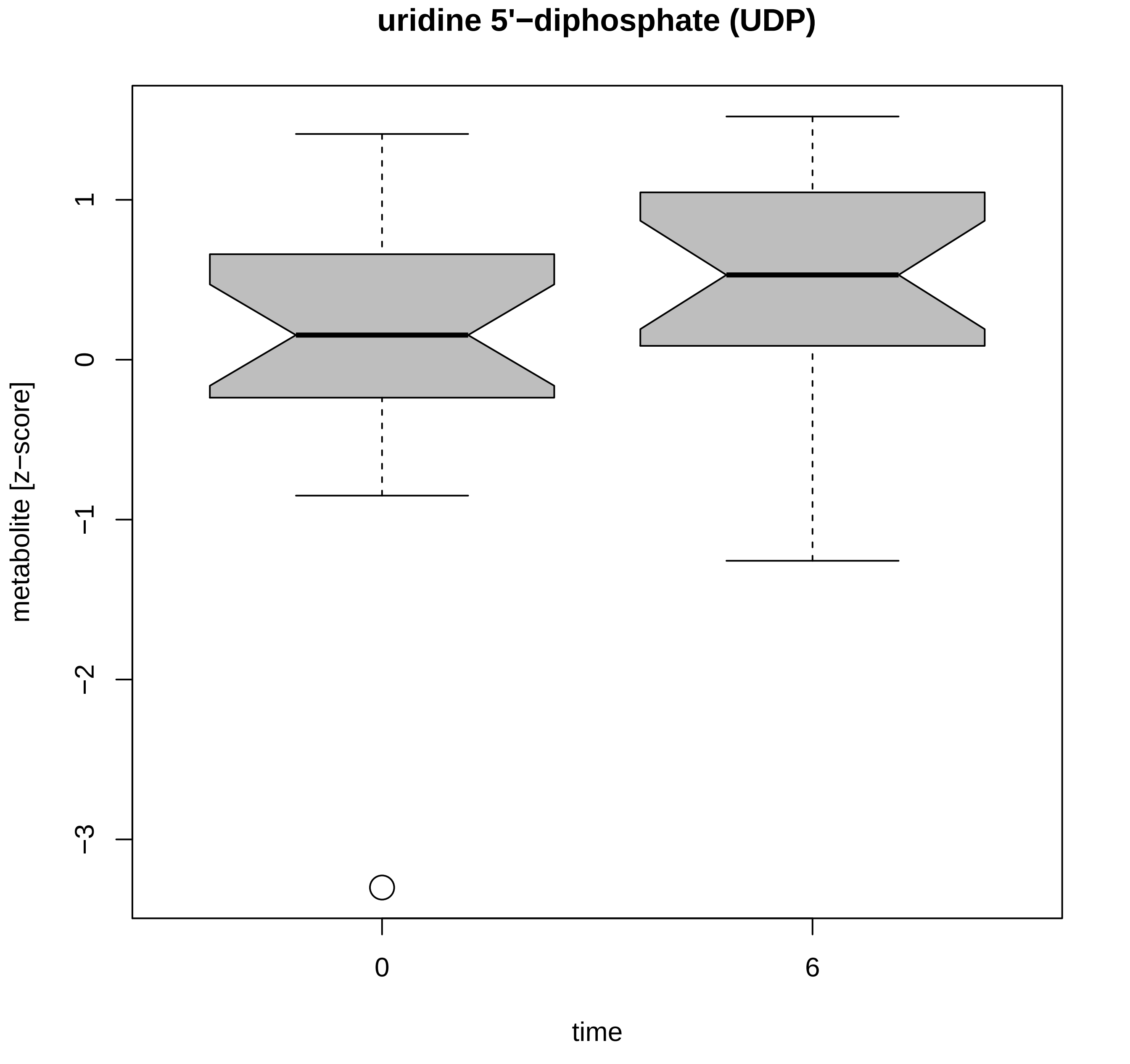

uridine 5'−diphosphate (UDP)
metabolite [z−score]
1
0
−1
−2
−3
0
6
time

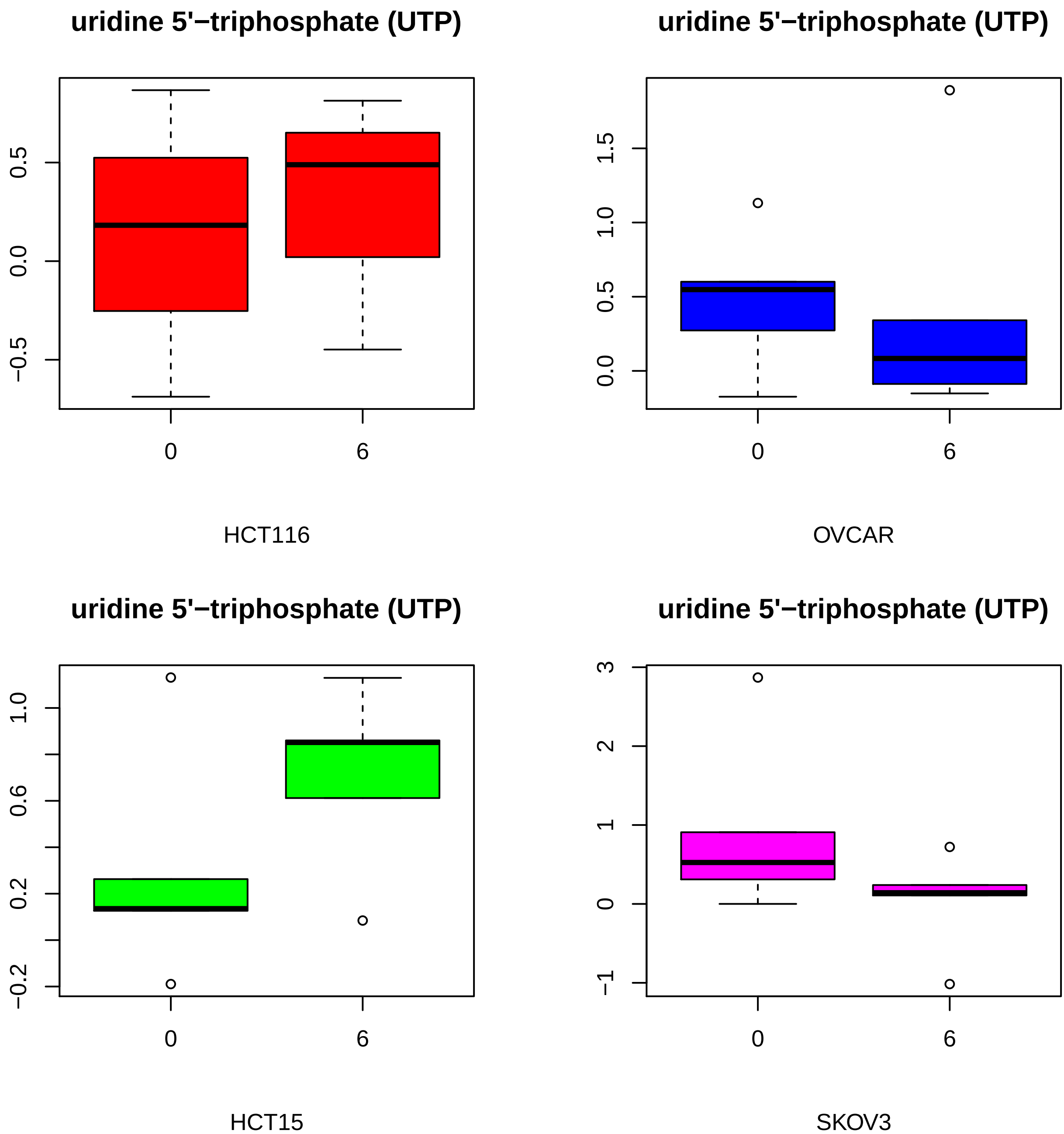
uridine 5'−triphosphate (UTP)
−0.5
0.0
0.5
0
6
HCT116
uridine 5'−triphosphate (UTP)
0.0
0.5
1.0
1.5
0
6
OVCAR
uridine 5'−triphosphate (UTP)
−0.2
0.2
0.6
1.0
0
6
HCT15
uridine 5'−triphosphate (UTP)
−1
0
1
2
3
0
6
SKOV3

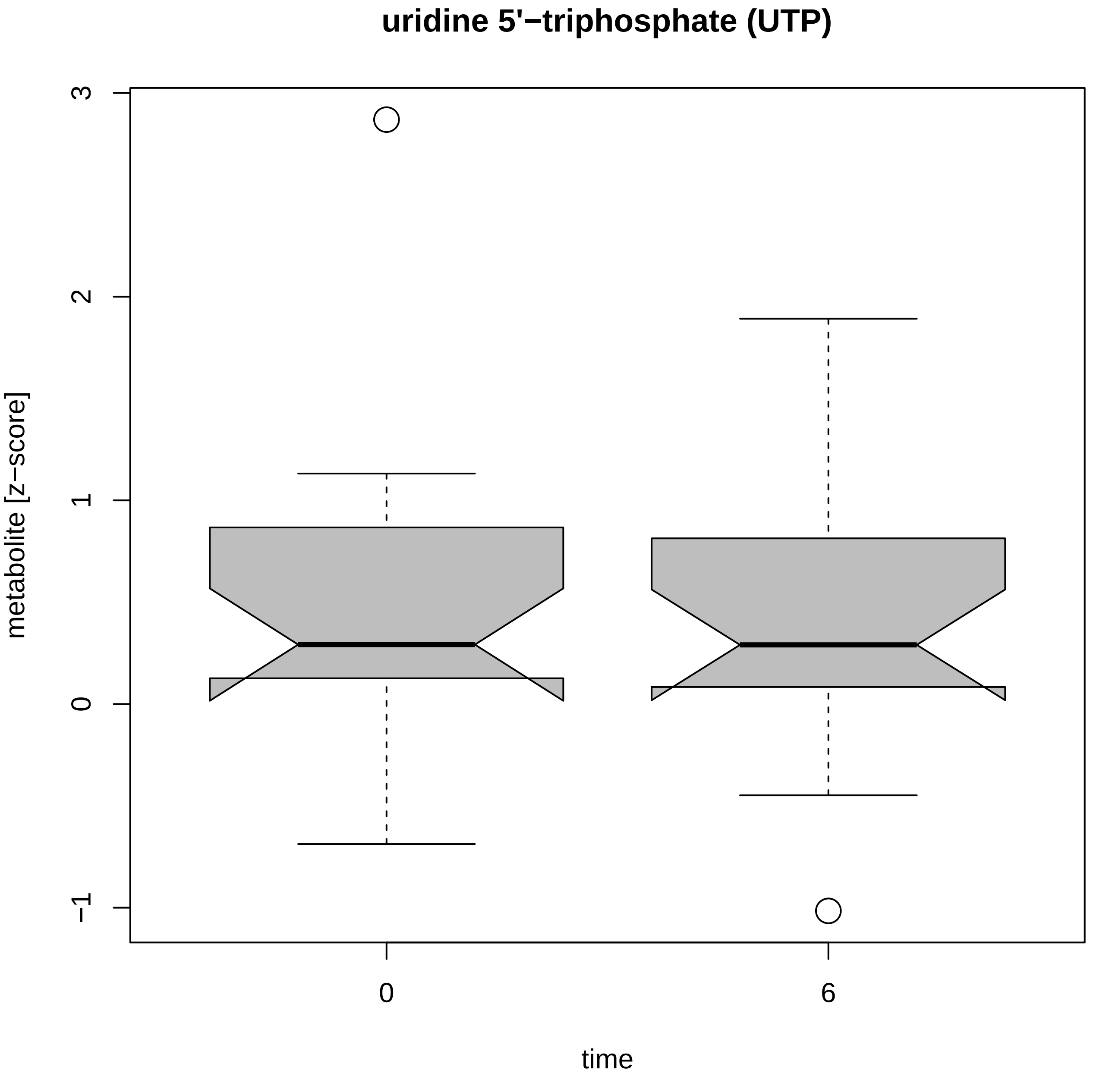

uridine 5'−triphosphate (UTP)
metabolite [z−score]
3
2
1
0
−1
0
6
time

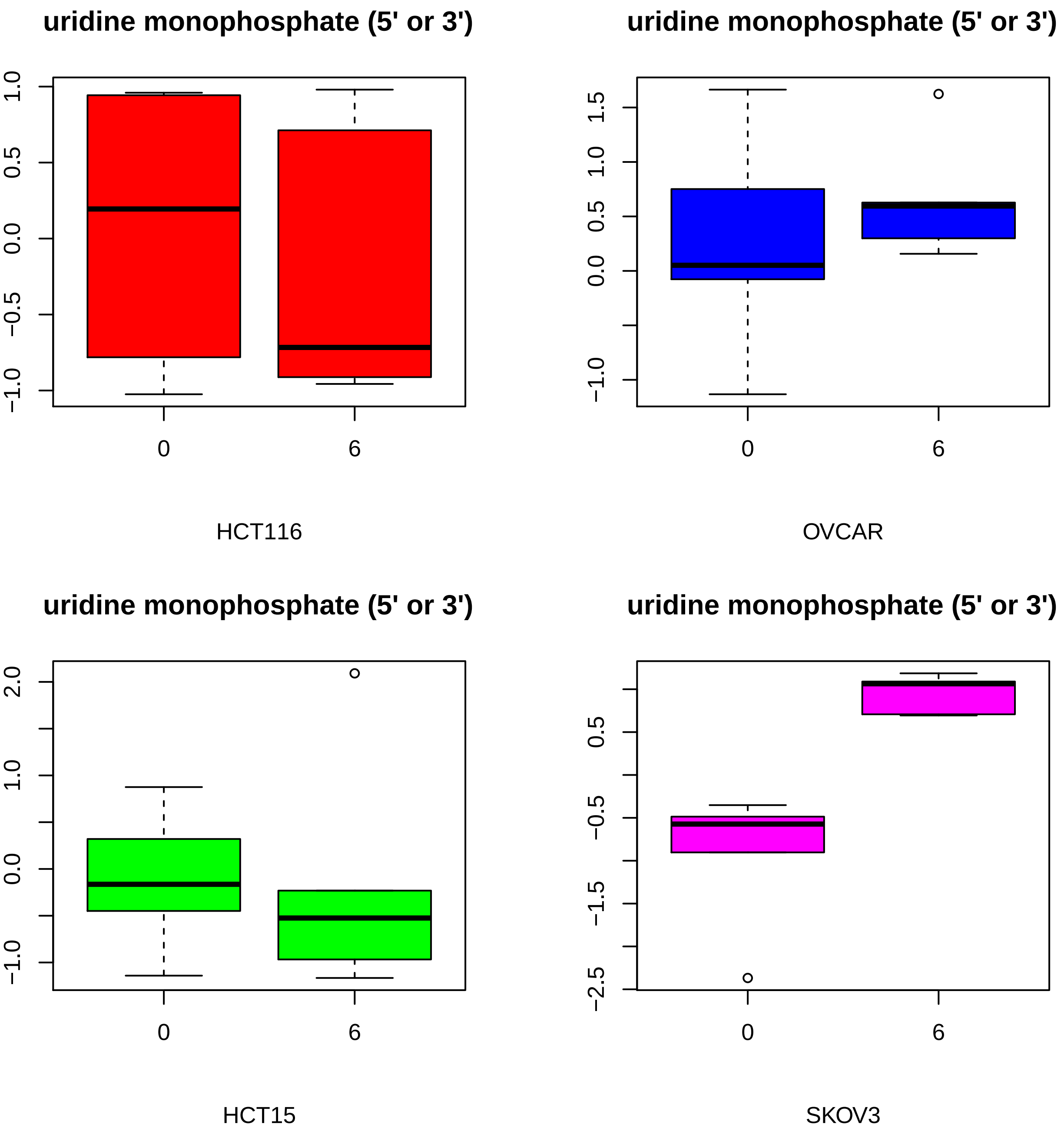
uridine monophosphate (5' or 3')
1.0
0.5
0.0
−0.5
−1.0
0
6
HCT116
uridine monophosphate (5' or 3')
1.5
1.0
0.5
0.0
−1.0
0
6
OVCAR
uridine monophosphate (5' or 3')
2.0
1.0
0.0
−1.0
0
6
HCT15
uridine monophosphate (5' or 3')
0.5
−0.5
−1.5
−2.5
0
6
SKOV3

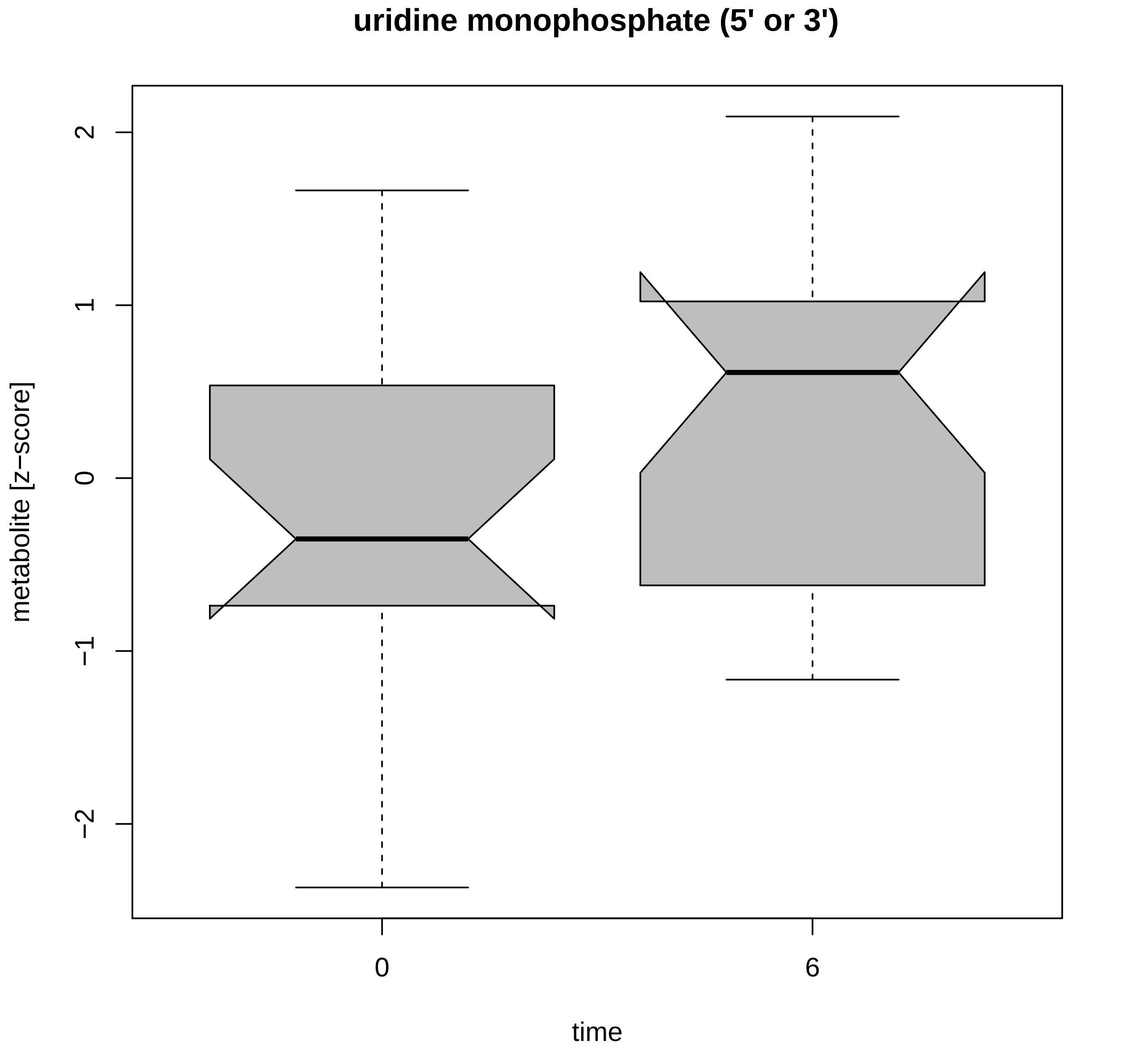
uridine monophosphate (5' or 3')
metabolite [z−score]
2
1
0
−1
−2
0
6
time

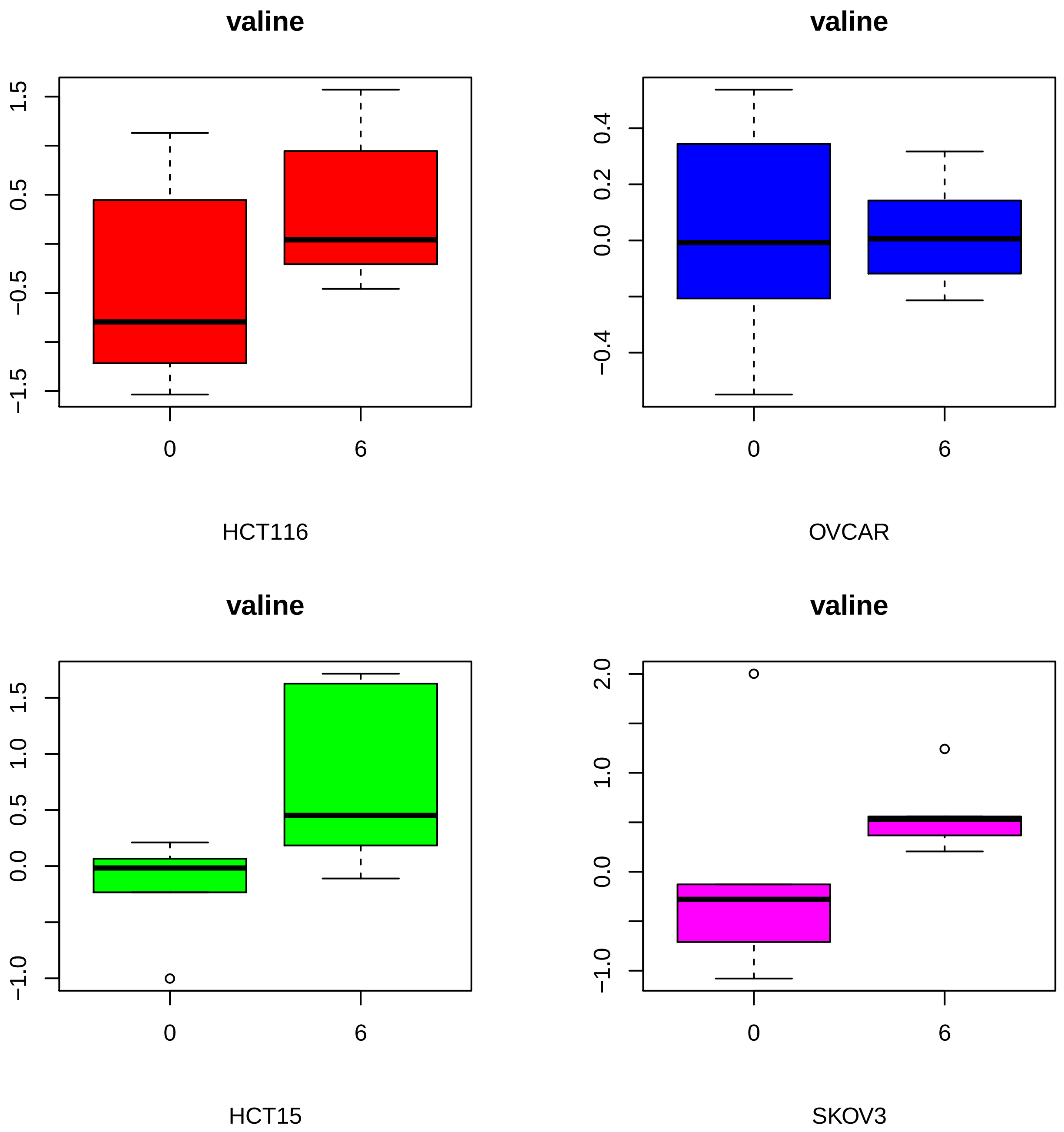
valine
1.5
0.5
−0.5
−1.5
0
6
HCT116
valine
0.4
0.2
0.0
−0.4
0
6
OVCAR
valine
1.5
1.0
0.5
0.0
−1.0
0
6
HCT15
valine
2.0
1.0
0.0
−1.0
0
6
SKOV3

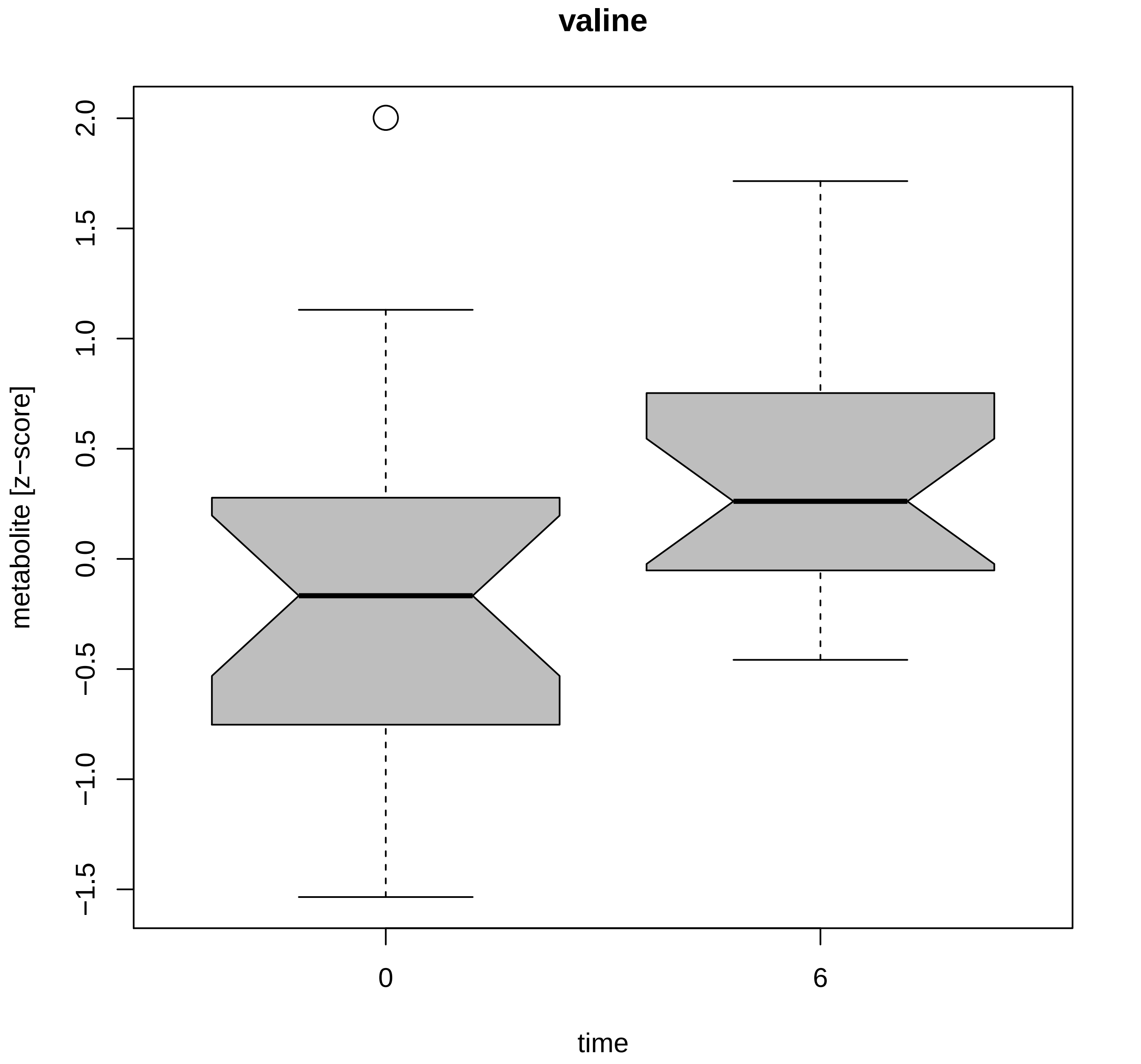
valine
metabolite [z−score]
2.0
1.5
1.0
0.5
0.0
−0.5
−1.0
−1.5
0
6
time

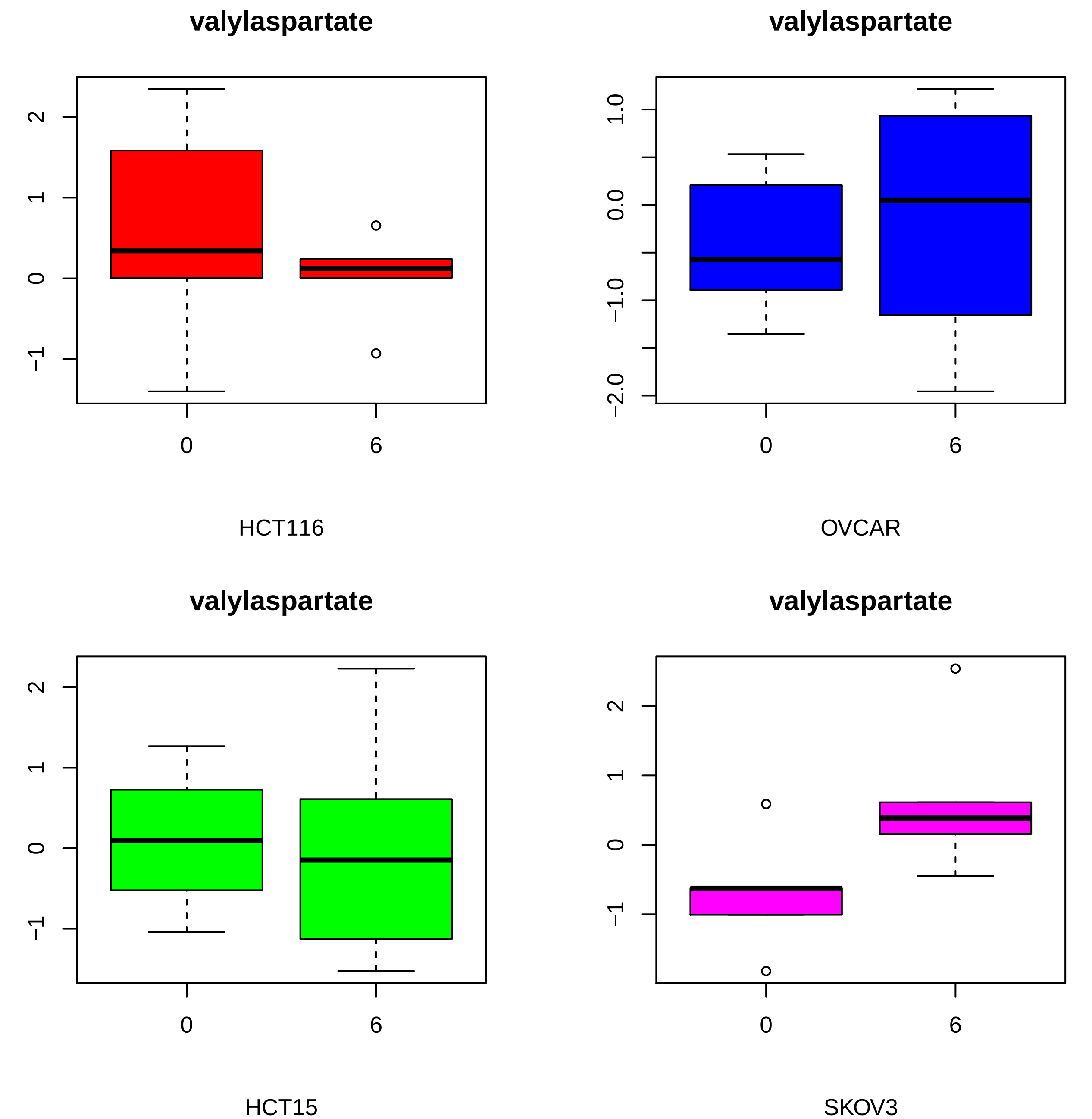
valylaspartate
2
1
0
−1
0
6
HCT116
valylaspartate
1.0
0.0
−1.0
−2.0
0
6
OVCAR
valylaspartate
2
1
0
−1
0
6
HCT15
valylaspartate
2
1
0
−1
0
6
SKOV3

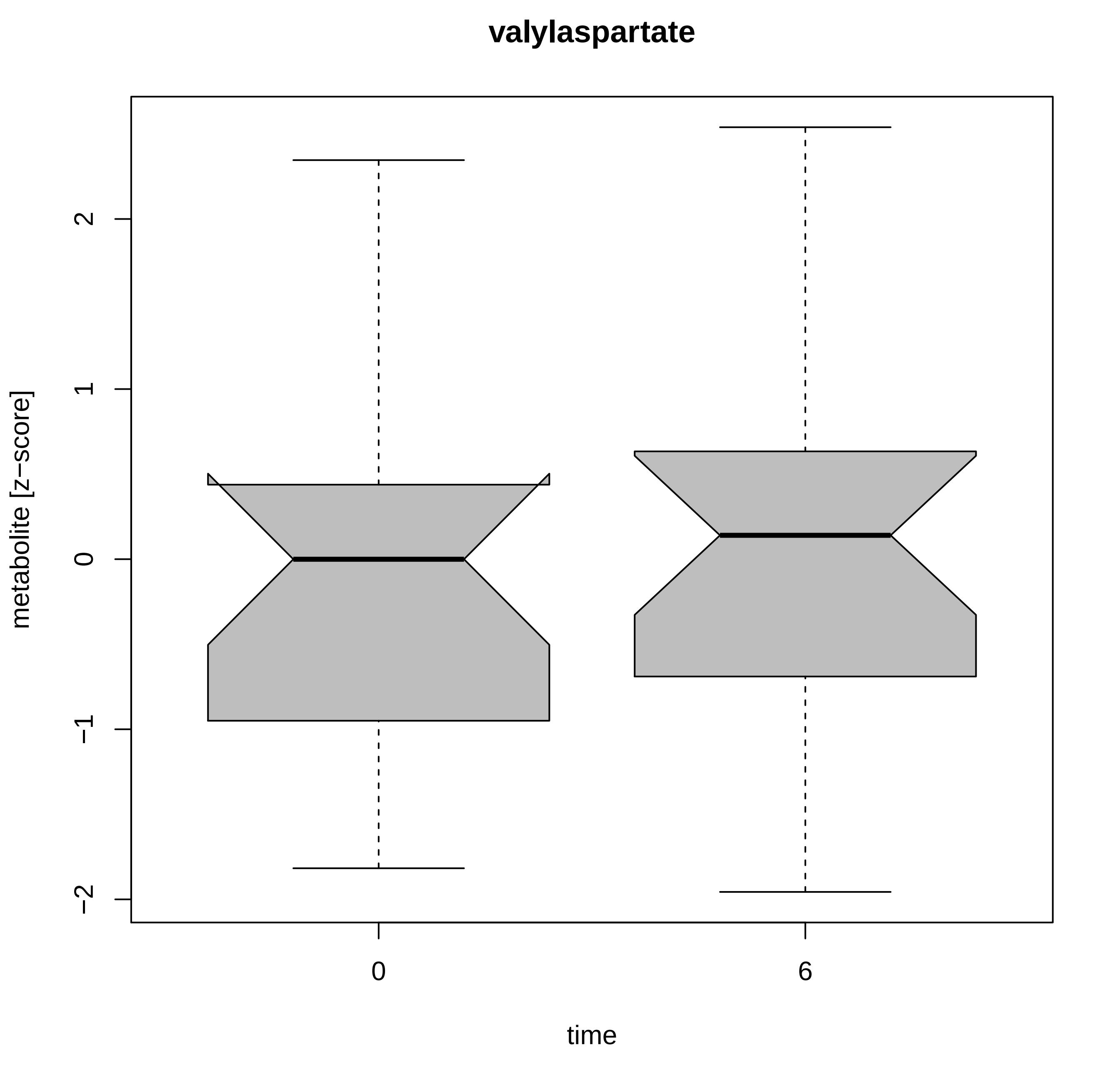
valylaspartate
metabolite [z−score]
2
1
0
−1
−2
0
6
time

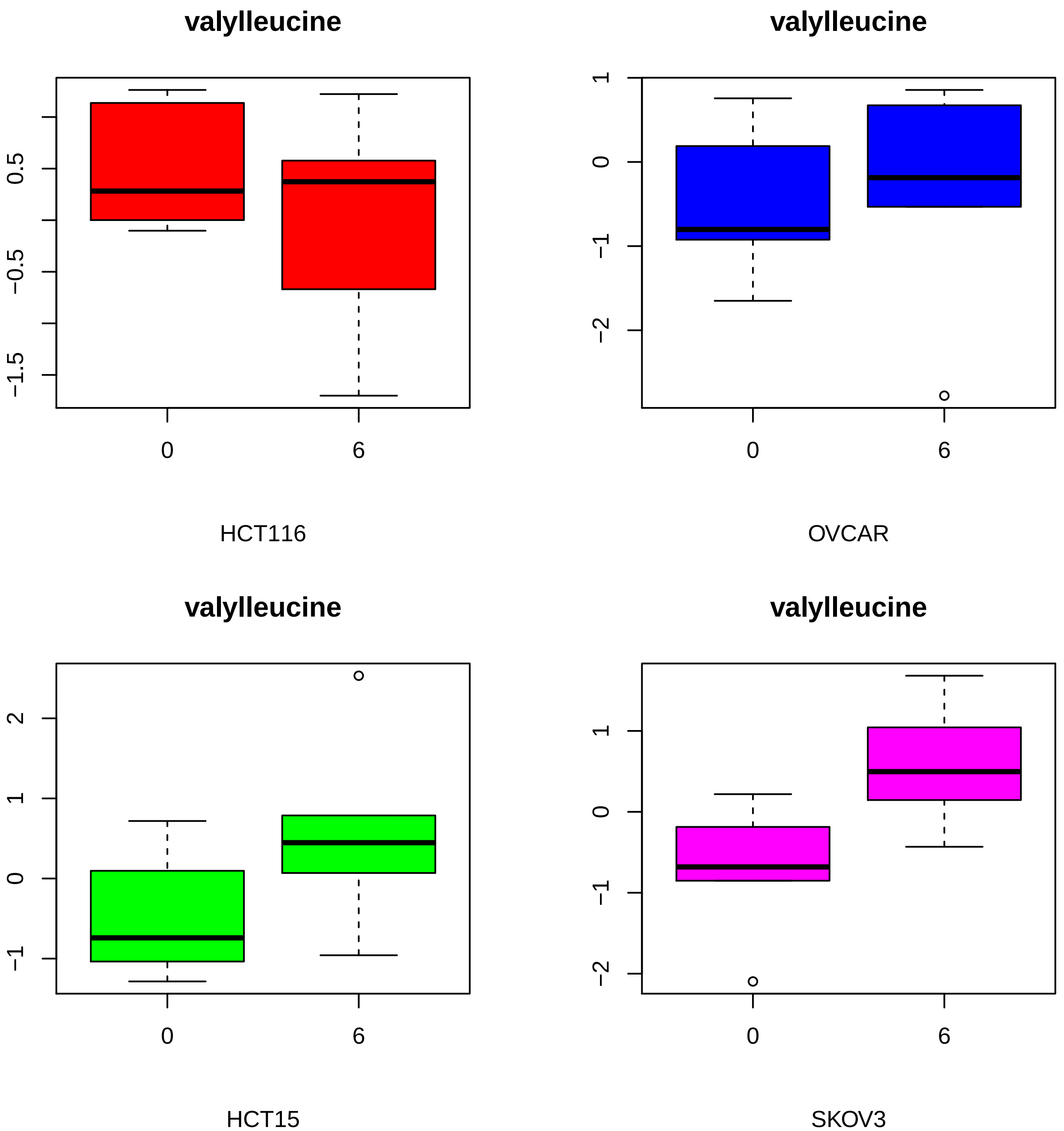
valylleucine
0.5
−0.5
−1.5
0
6
HCT116
valylleucine
1
0
−1
−2
0
6
OVCAR
valylleucine
2
1
0
−1
0
6
HCT15
valylleucine
1
0
−1
−2
0
6
SKOV3

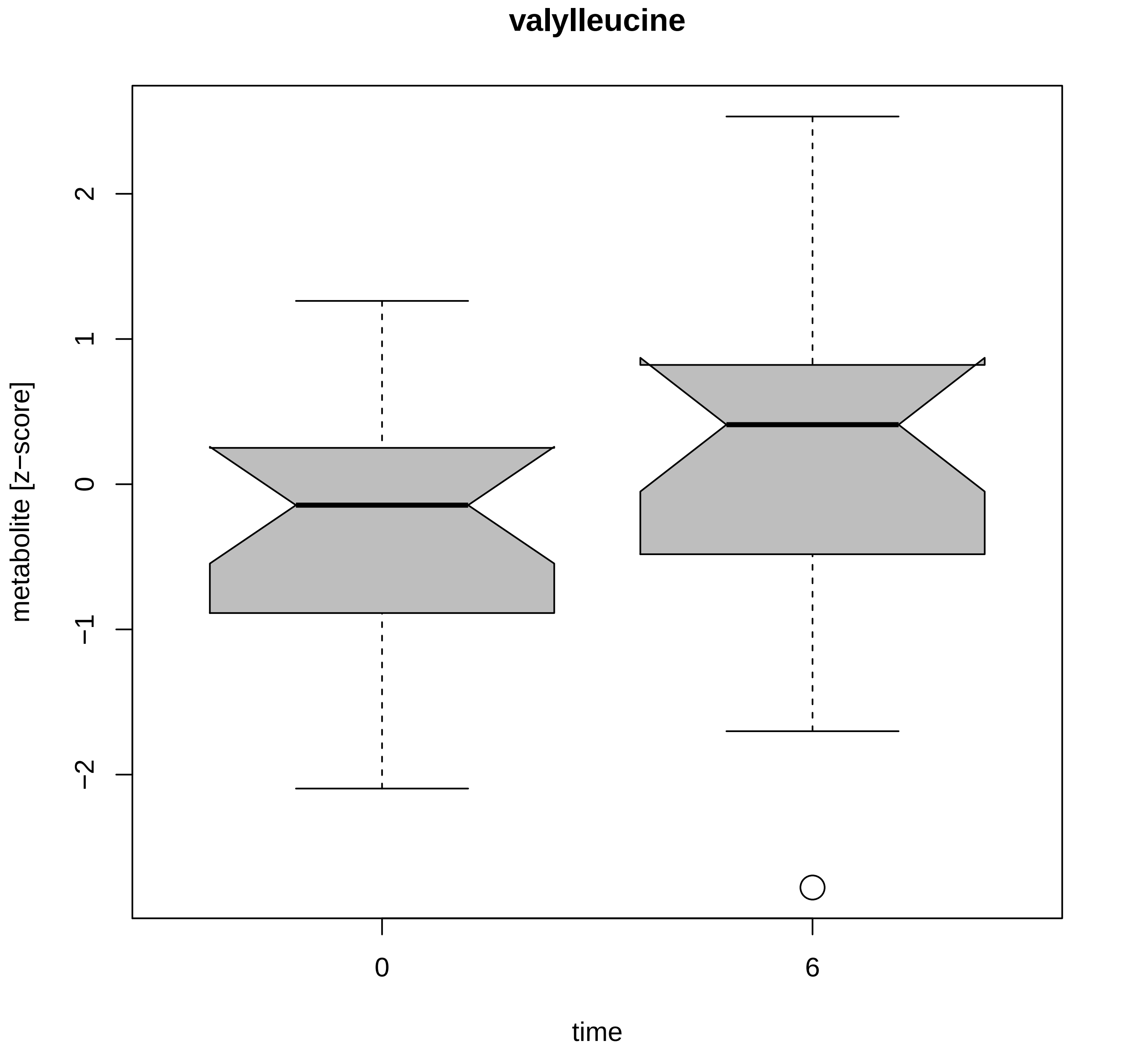

valylleucine
metabolite [z−score]
2
1
0
−1
−2
0
6
time

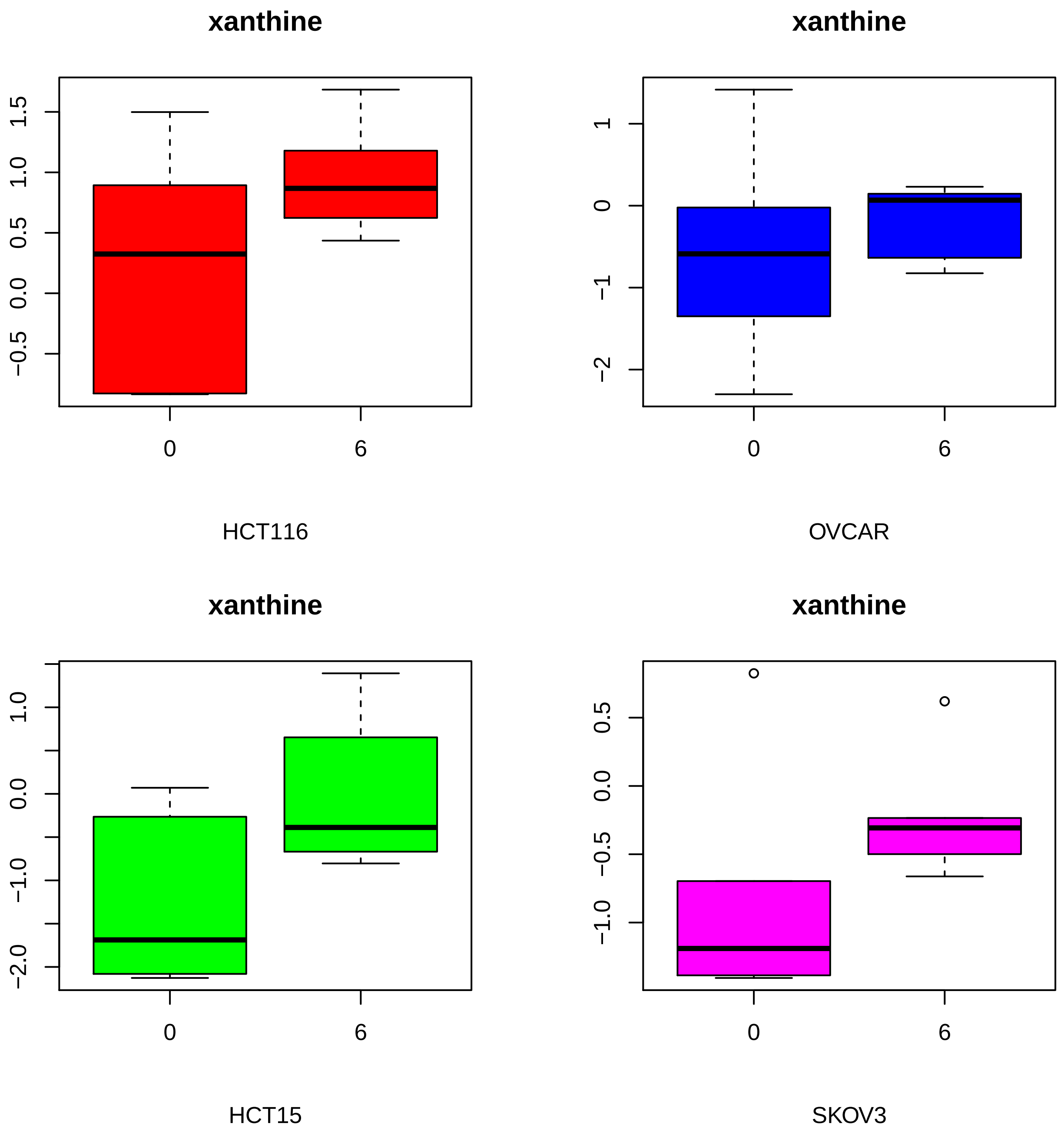
xanthine
1.5
1.0
0.5
0.0
−0.5
0
6
HCT116
xanthine
1
0
−1
−2
0
6
OVCAR
xanthine
1.0
0.0
−1.0
−2.0
0
6
HCT15
xanthine
0.5
0.0
−0.5
−1.0
0
6
SKOV3

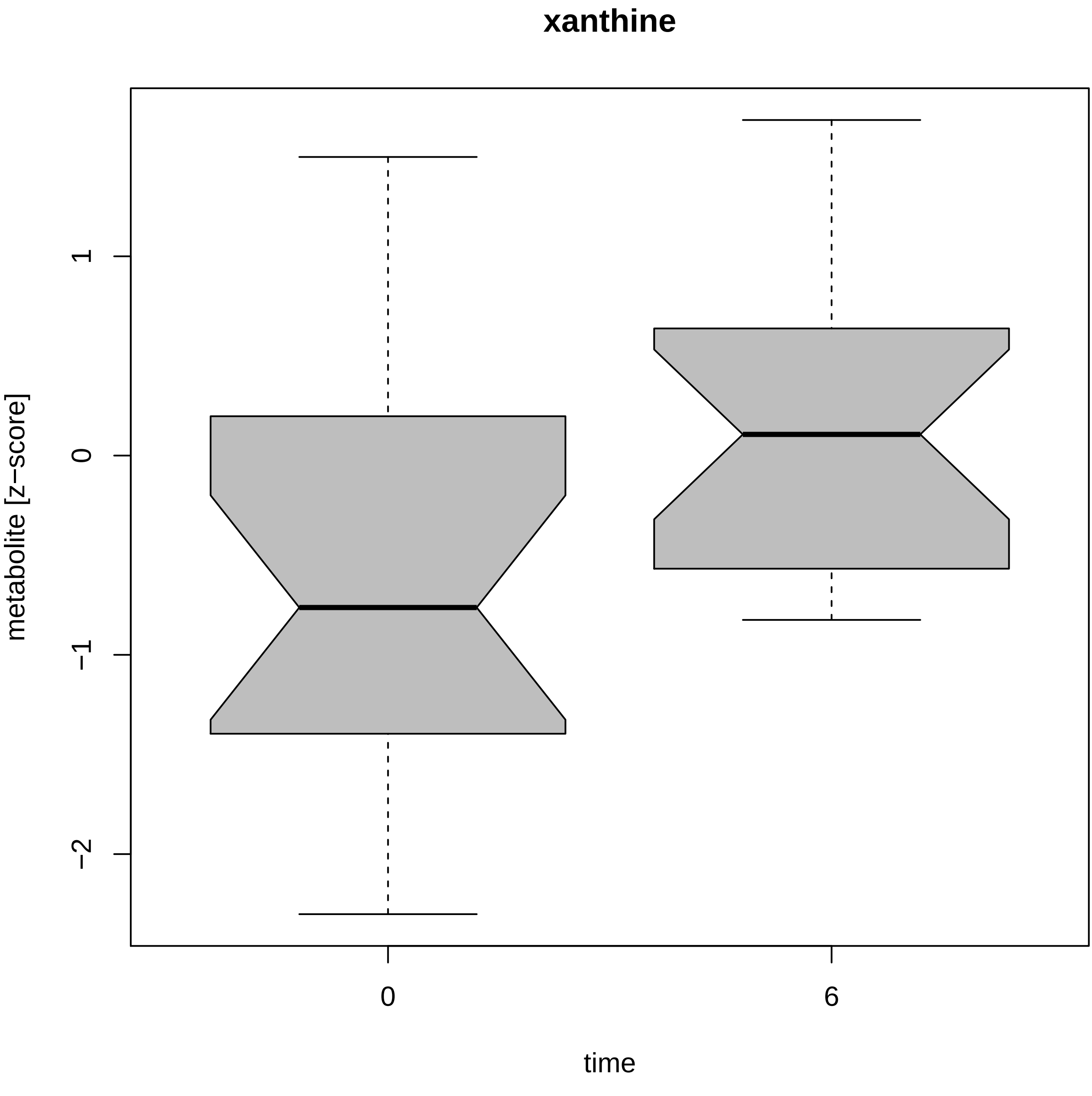
xanthine
metabolite [z−score]
1
0
−1
−2
0
6
time

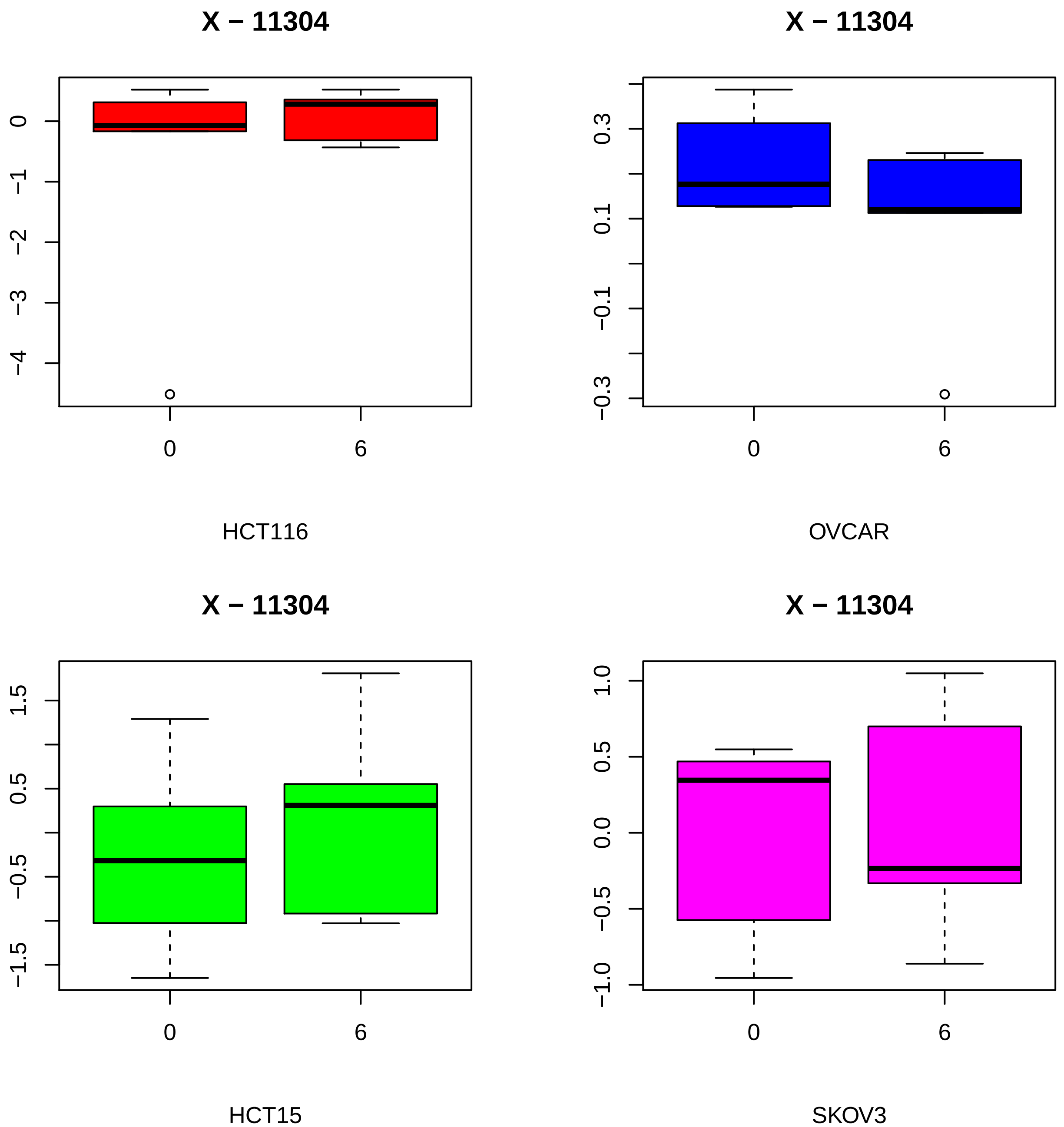
X − 11304
0
−1
−2
−3
−4
0
6
HCT116
X − 11304
0.3
0.1
−0.1
−0.3
0
6
OVCAR
X − 11304
1.5
0.5
−0.5
−1.5
0
6
HCT15
X − 11304
1.0
0.5
0.0
−0.5
−1.0
0
6
SKOV3

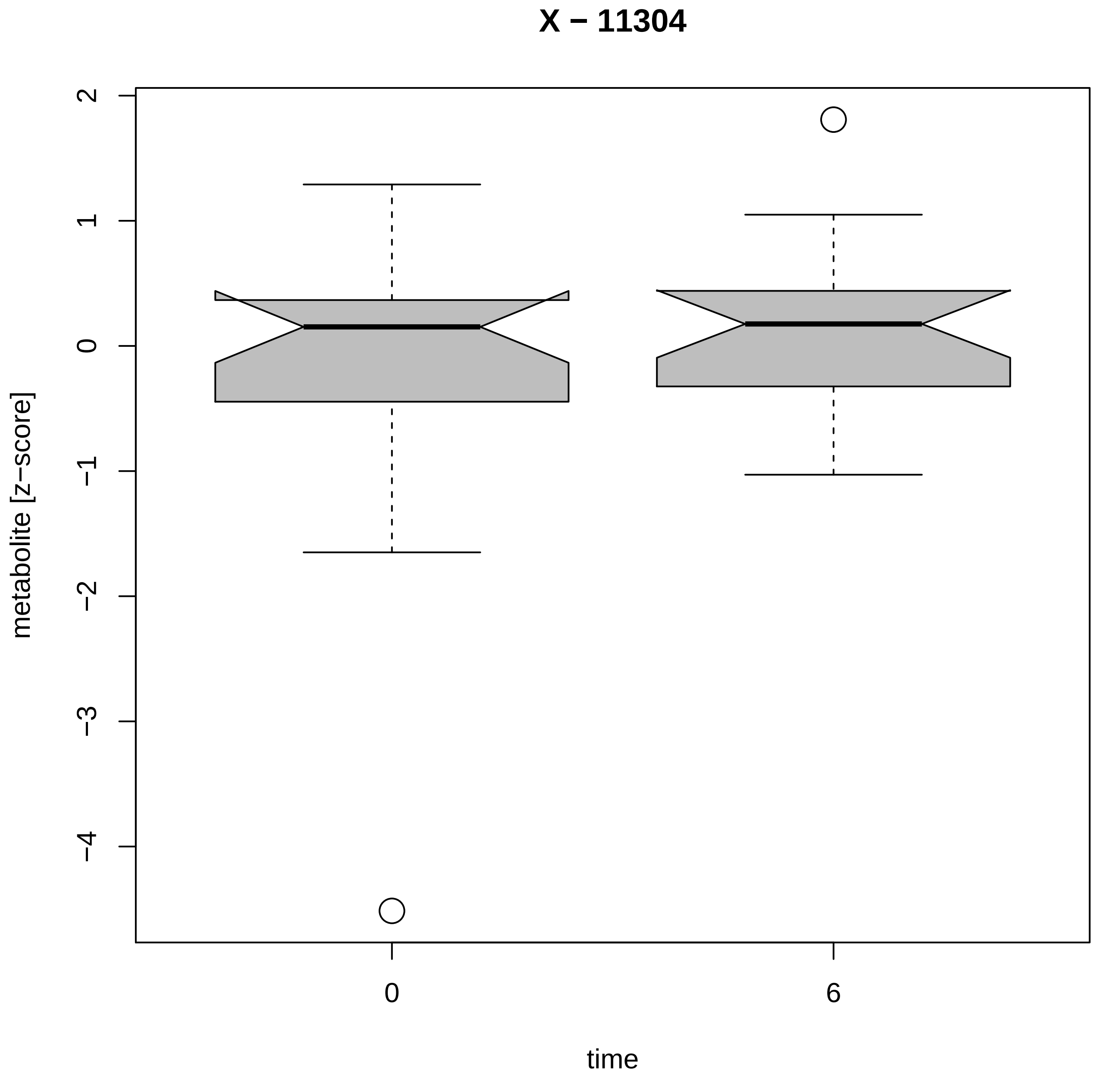
X − 11304
metabolite [z−score]
2
1
0
−1
−2
−3
−4
0
6
time

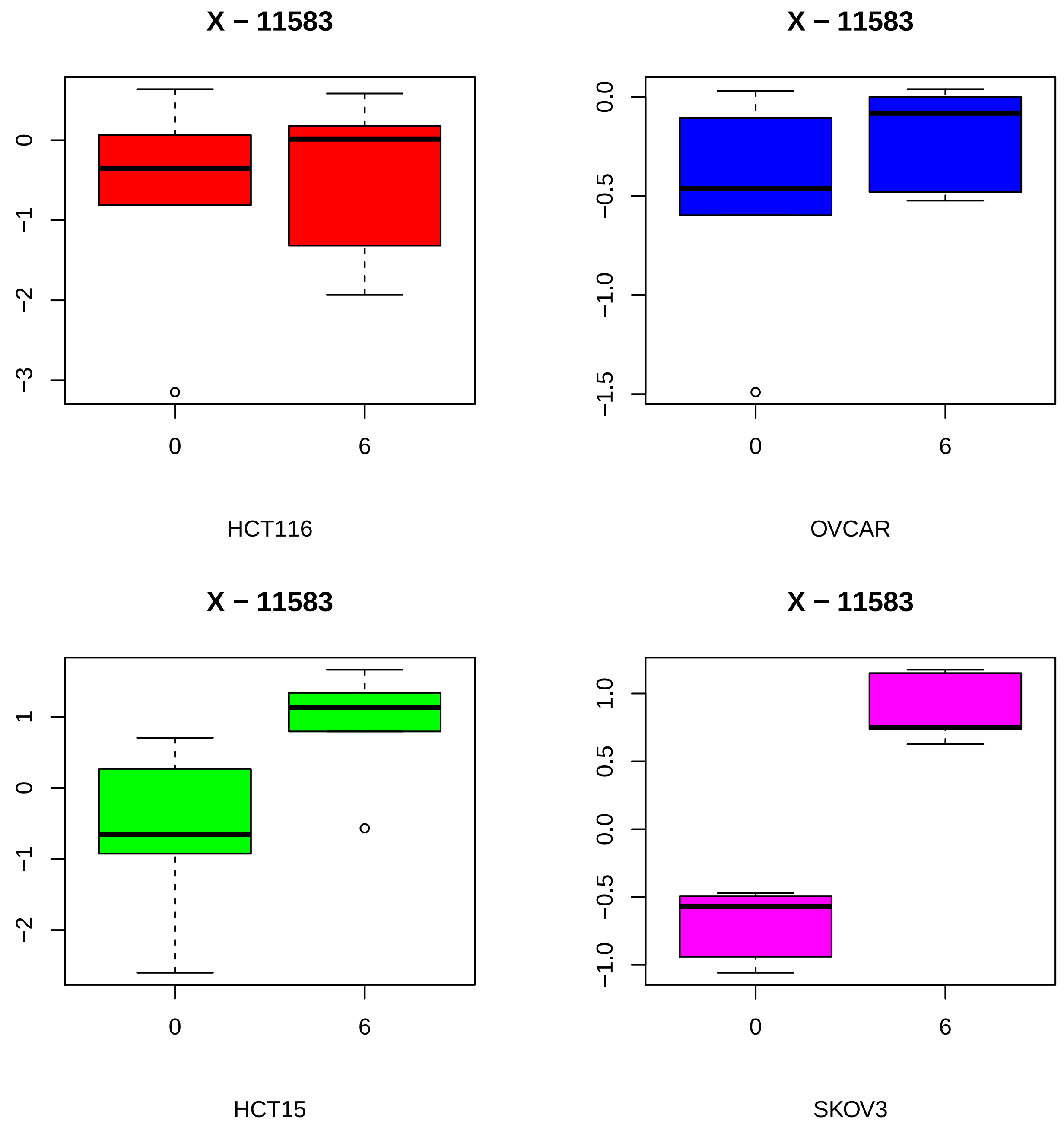
X − 11583
0
−1
−2
−3
0
6
HCT116
X − 11583
0.0
−0.5
−1.0
−1.5
0
6
OVCAR
X − 11583
1
0
−1
−2
0
6
HCT15
X − 11583
1.0
0.5
0.0
−0.5
−1.0
0
6
SKOV3

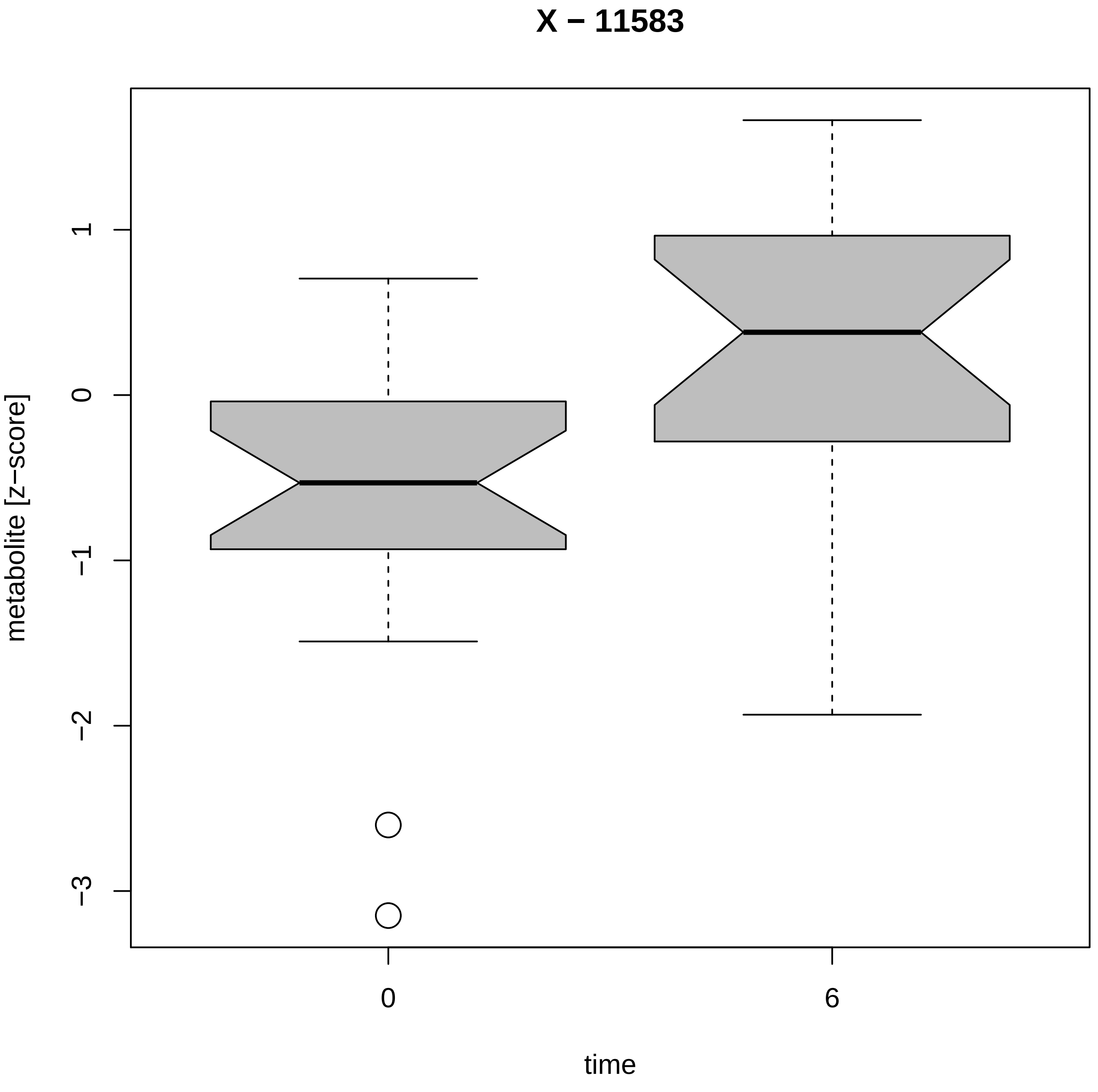

X − 11583
metabolite [z−score]
1
0
−1
−2
−3
0
6
time

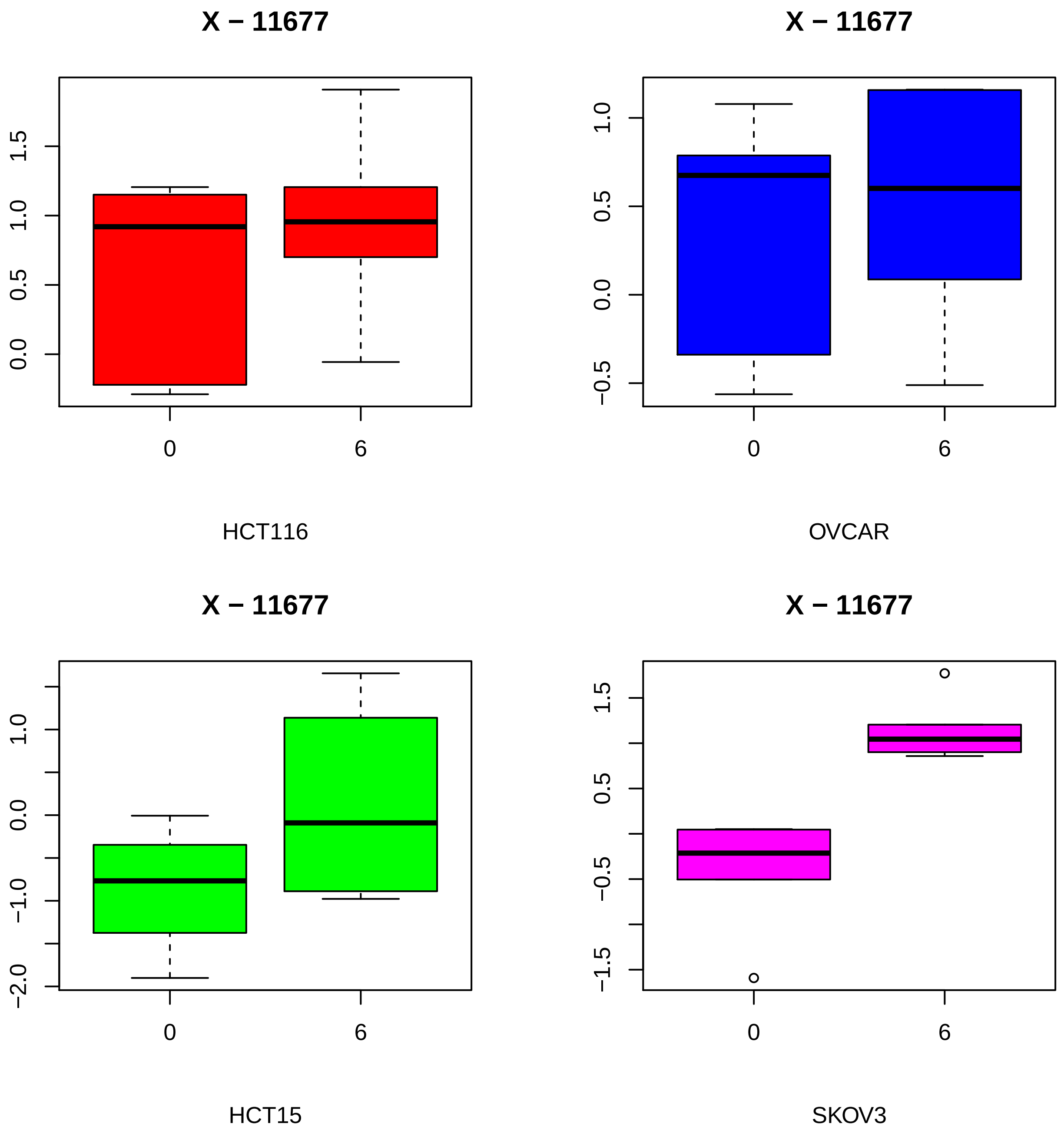

X − 11677
1.5
1.0
0.5
0.0
0
6
HCT116
X − 11677
1.0
0.5
0.0
−0.5
0
6
OVCAR
X − 11677
1.0
0.0
−1.0
−2.0
0
6
HCT15
X − 11677
1.5
0.5
−0.5
−1.5
0
6
SKOV3

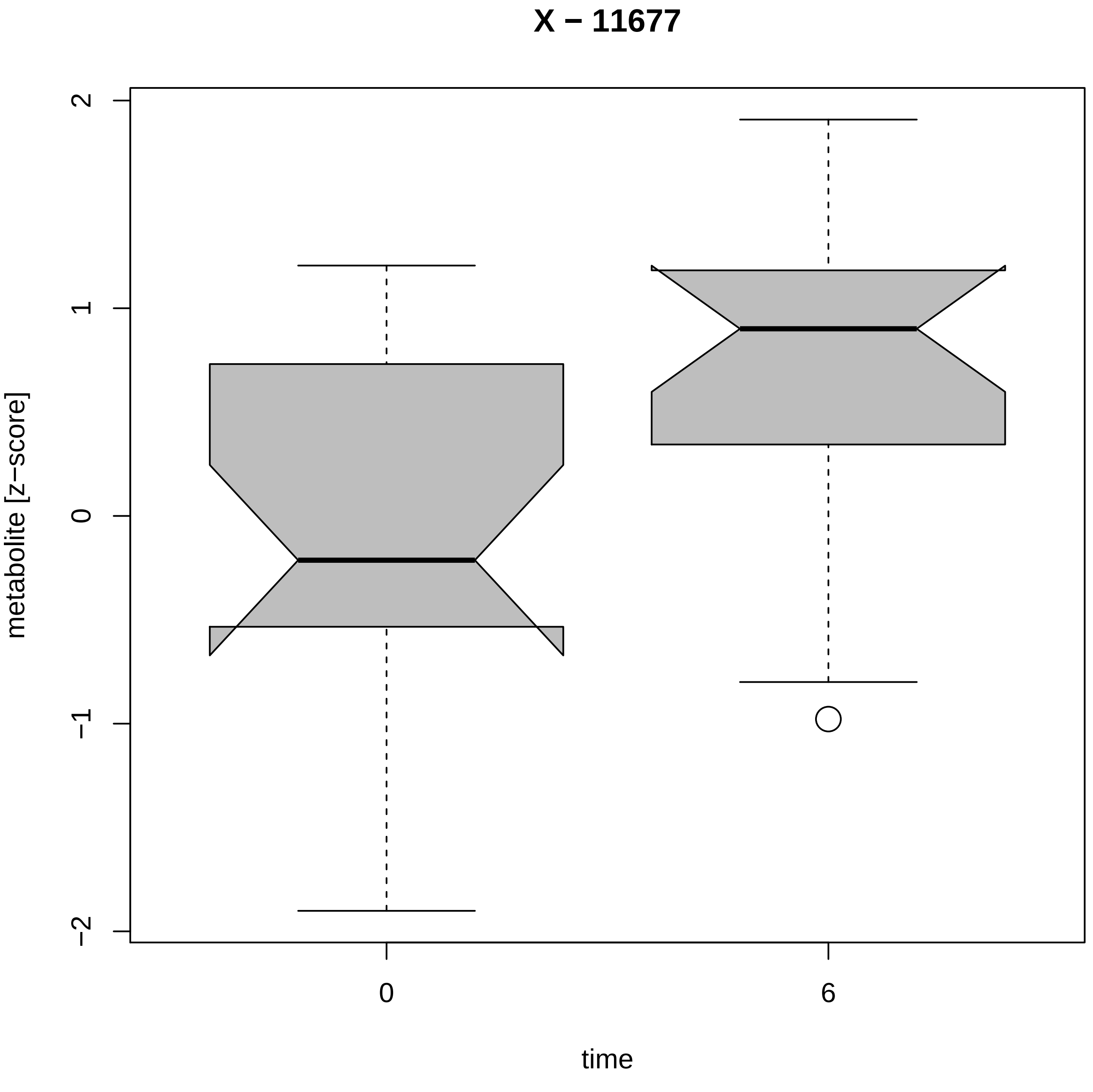
X − 11677
metabolite [z−score]
2
1
0
−1
−2
0
6
time

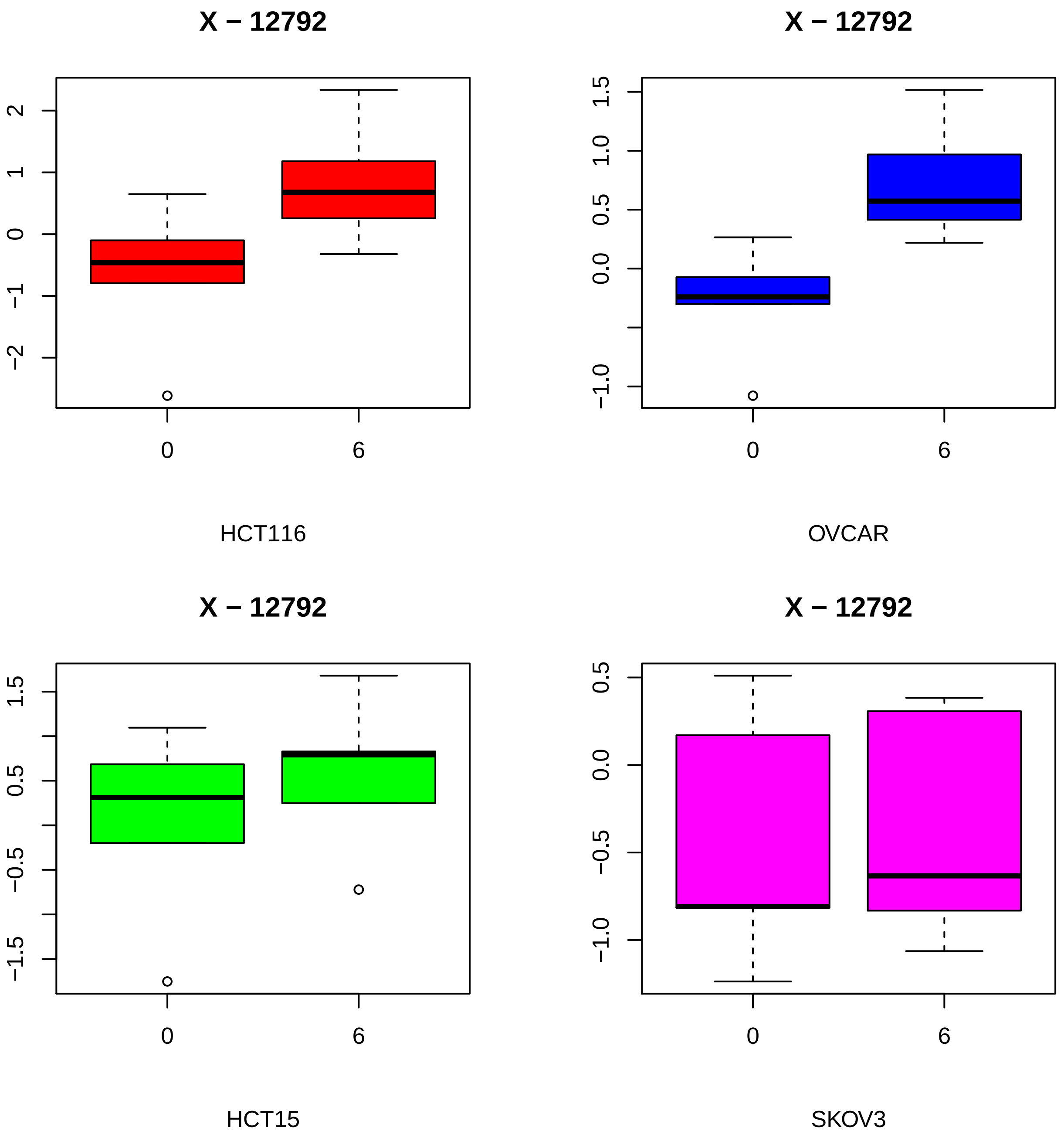
X − 12792
HCT116
X − 12792
OVCAR
X − 12792
HCT15
X − 12792
SKOV3
0
6

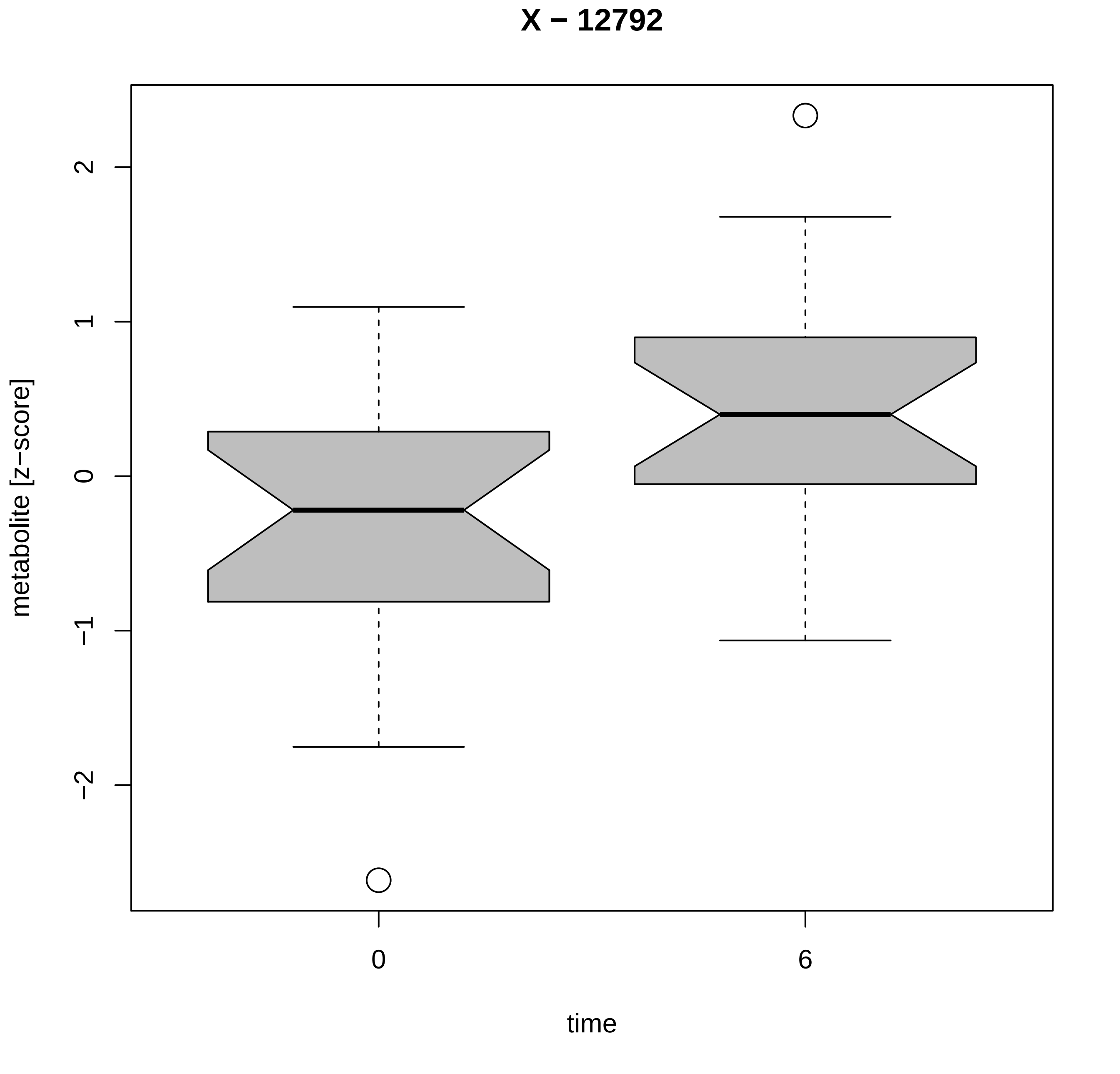

X − 12792
metabolite [z−score]
2
1
0
−1
−2
0
6
time

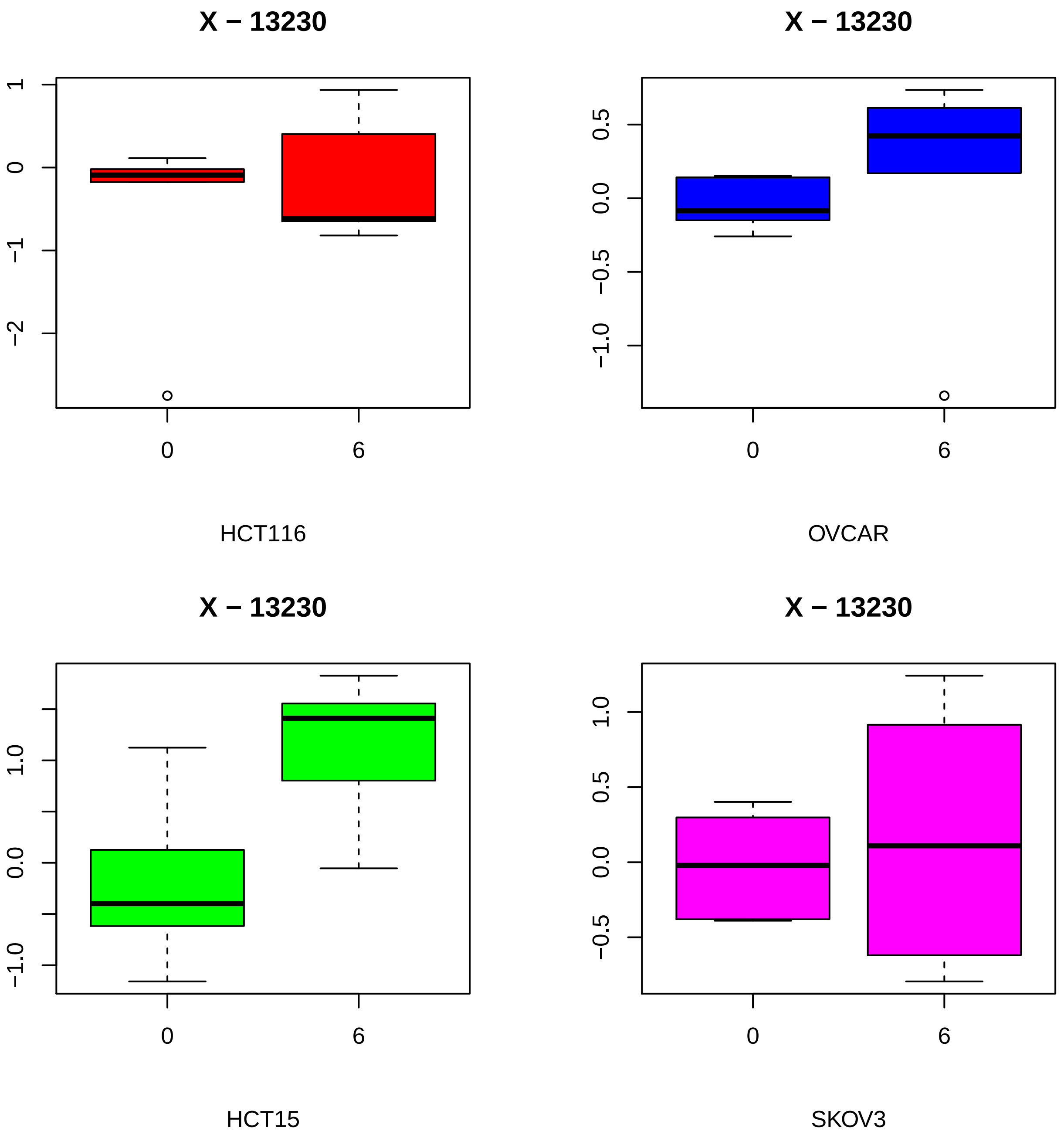
X − 13230
1
0
−1
−2
0
6
HCT116
X − 13230
0.5
0.0
−0.5
−1.0
0
6
OVCAR
X − 13230
1.0
0.0
−1.0
0
6
HCT15
X − 13230
1.0
0.5
0.0
−0.5
0
6
SKOV3

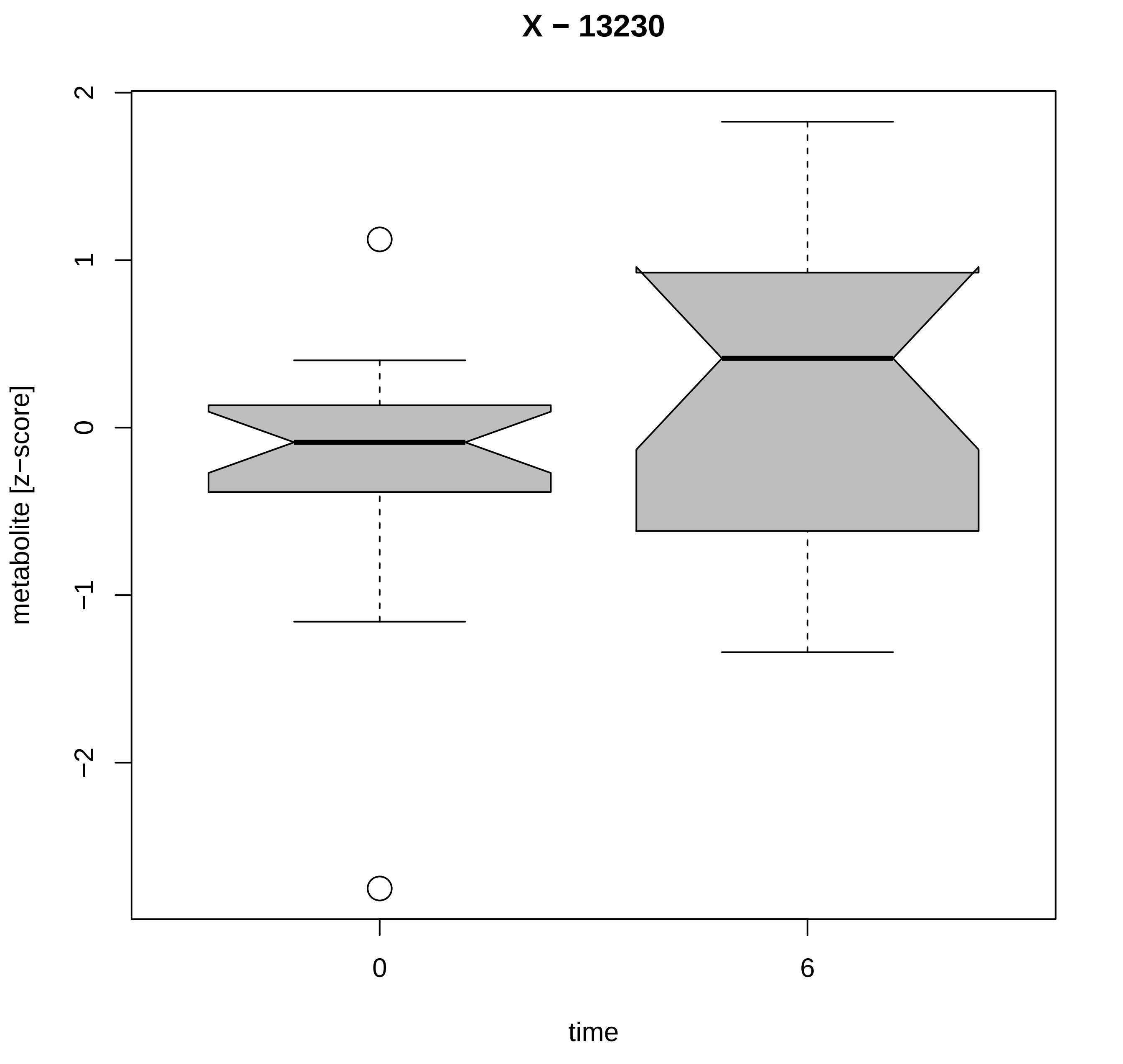
X − 13230
metabolite [z−score]
2
1
0
−1
−2
0
6
time

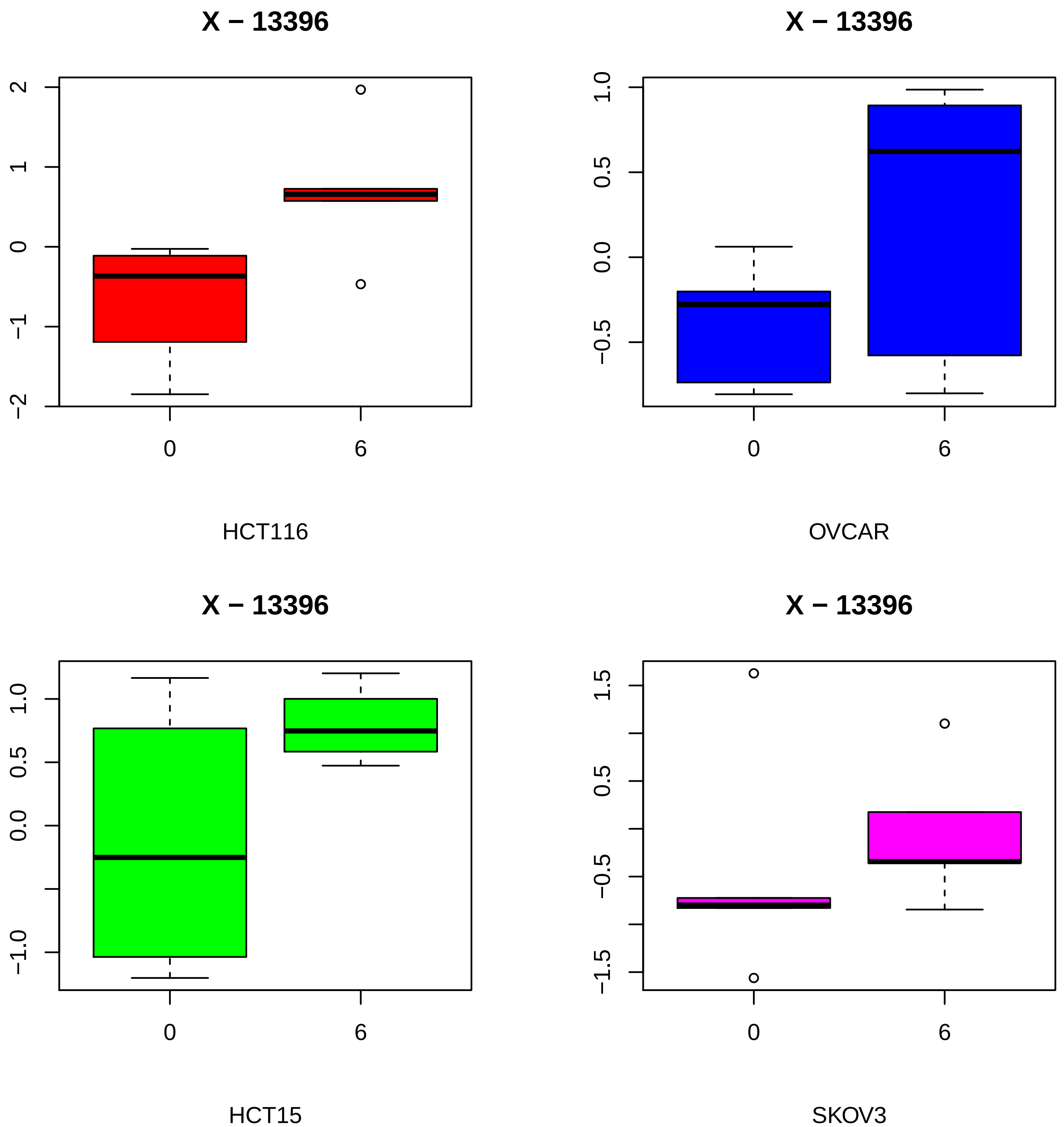
X − 13396
2
1
0
−1
−2
0
6
HCT116
X − 13396
1.0
0.5
0.0
−0.5
0
6
OVCAR
X − 13396
1.0
0.5
0.0
−1.0
0
6
HCT15
X − 13396
1.5
0.5
−0.5
−1.5
0
6
SKOV3

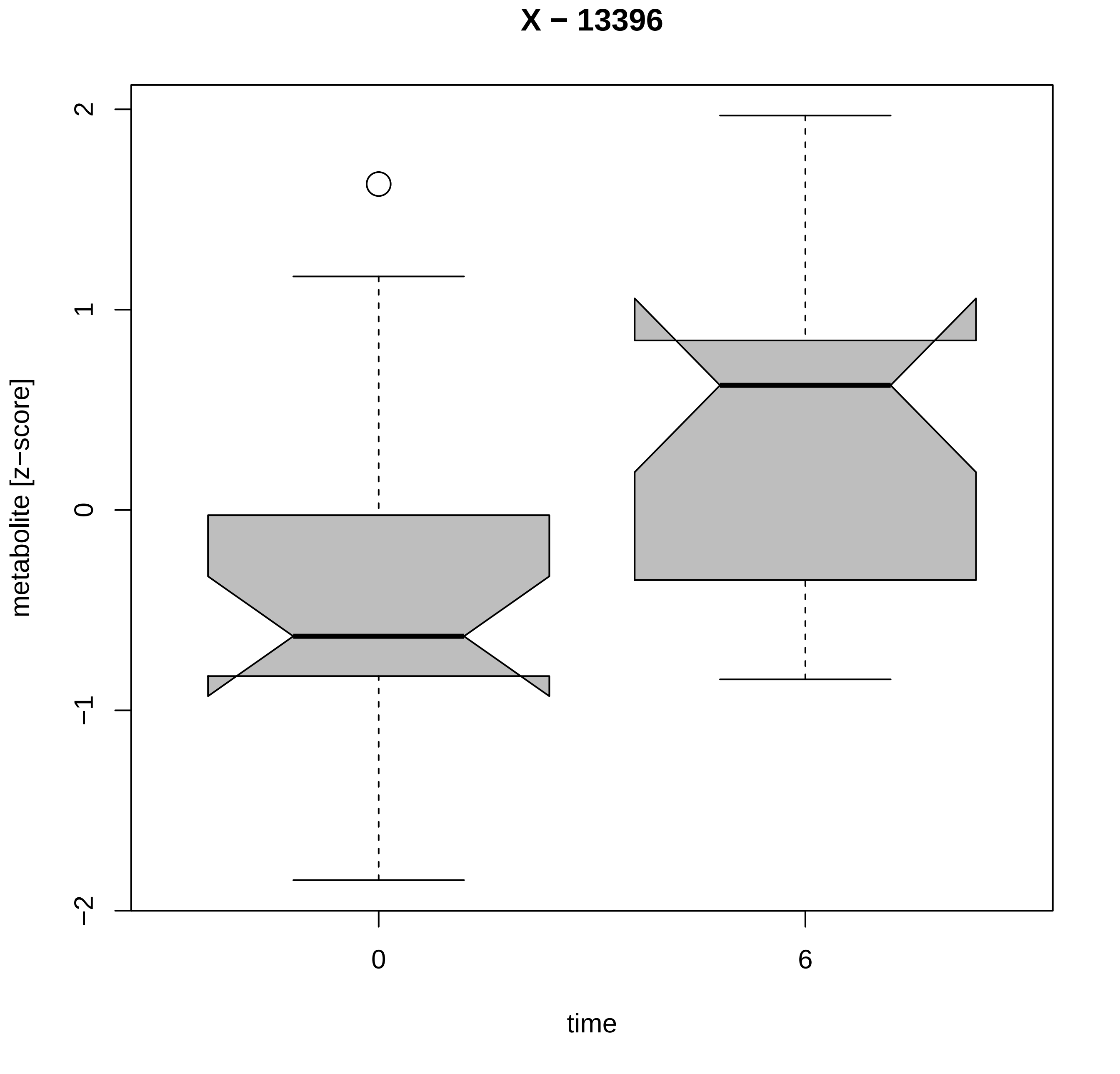
X − 13396
metabolite [z−score]
2
1
0
−1
−2
0
6
time

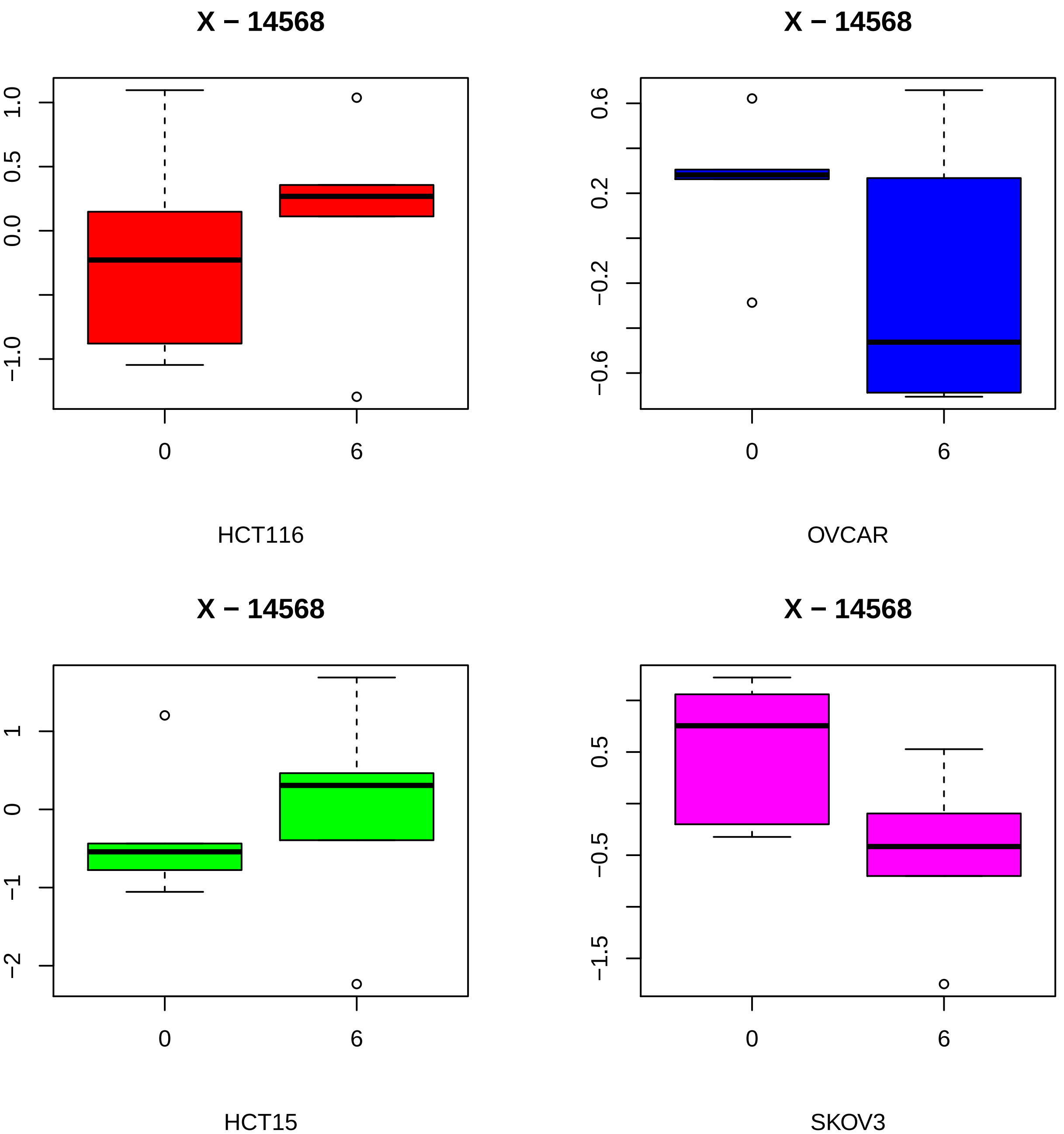
X − 14568
1.0
0.5
0.0
−1.0
0
6
HCT116
X − 14568
0.6
0.2
−0.2
−0.6
0
6
OVCAR
X − 14568
1
0
−1
−2
0
6
HCT15
X − 14568
0.5
−0.5
−1.5
0
6
SKOV3

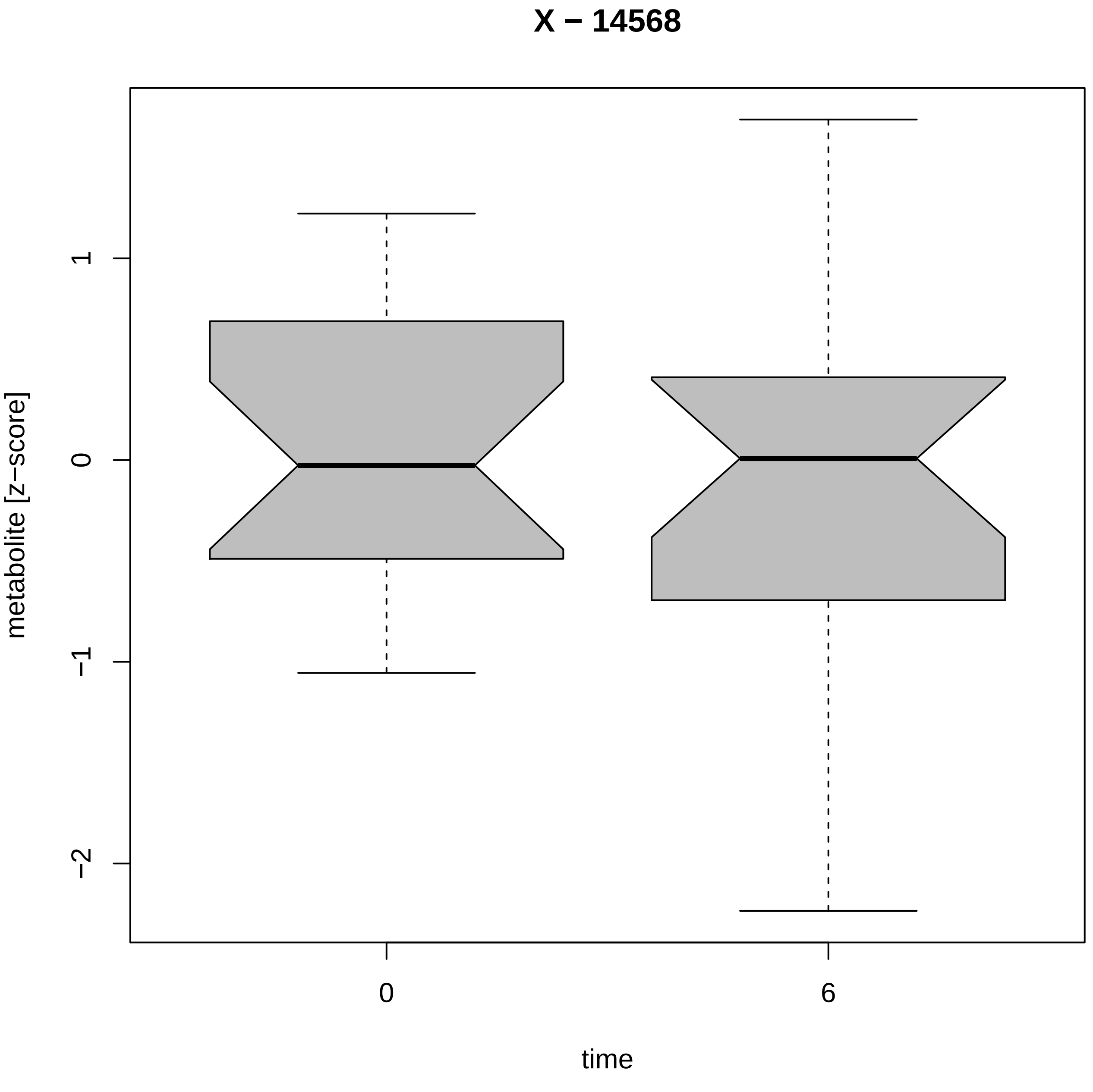

X − 14568
metabolite [z−score]
1
0
−1
−2
0
6
time

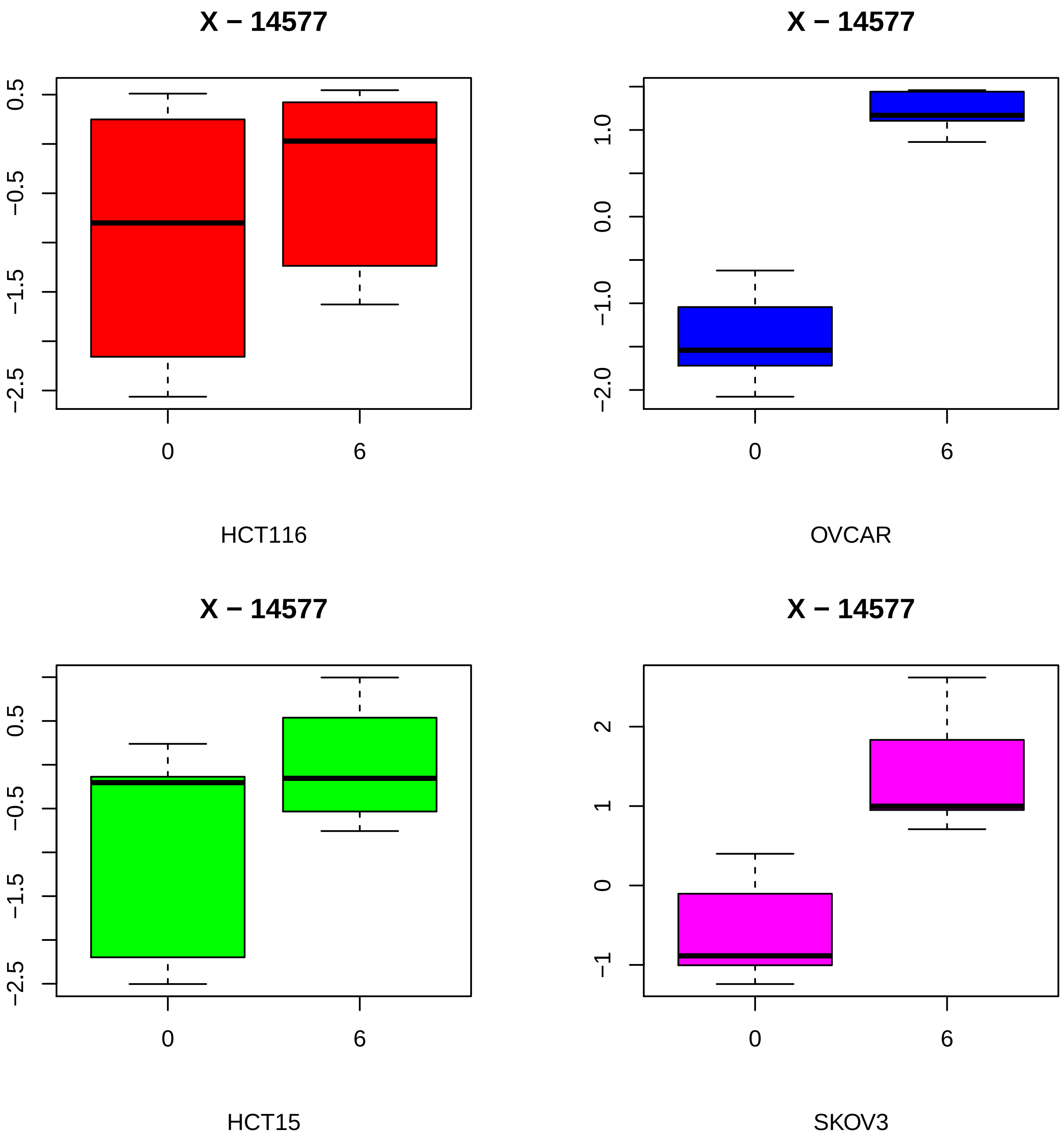
X − 14577
0.5
−0.5
−1.5
−2.5
0
6
HCT116
X − 14577
1.0
0.0
−1.0
−2.0
0
6
OVCAR
X − 14577
0.5
−0.5
−1.5
−2.5
0
6
HCT15
X − 14577
2
1
0
−1
0
6
SKOV3

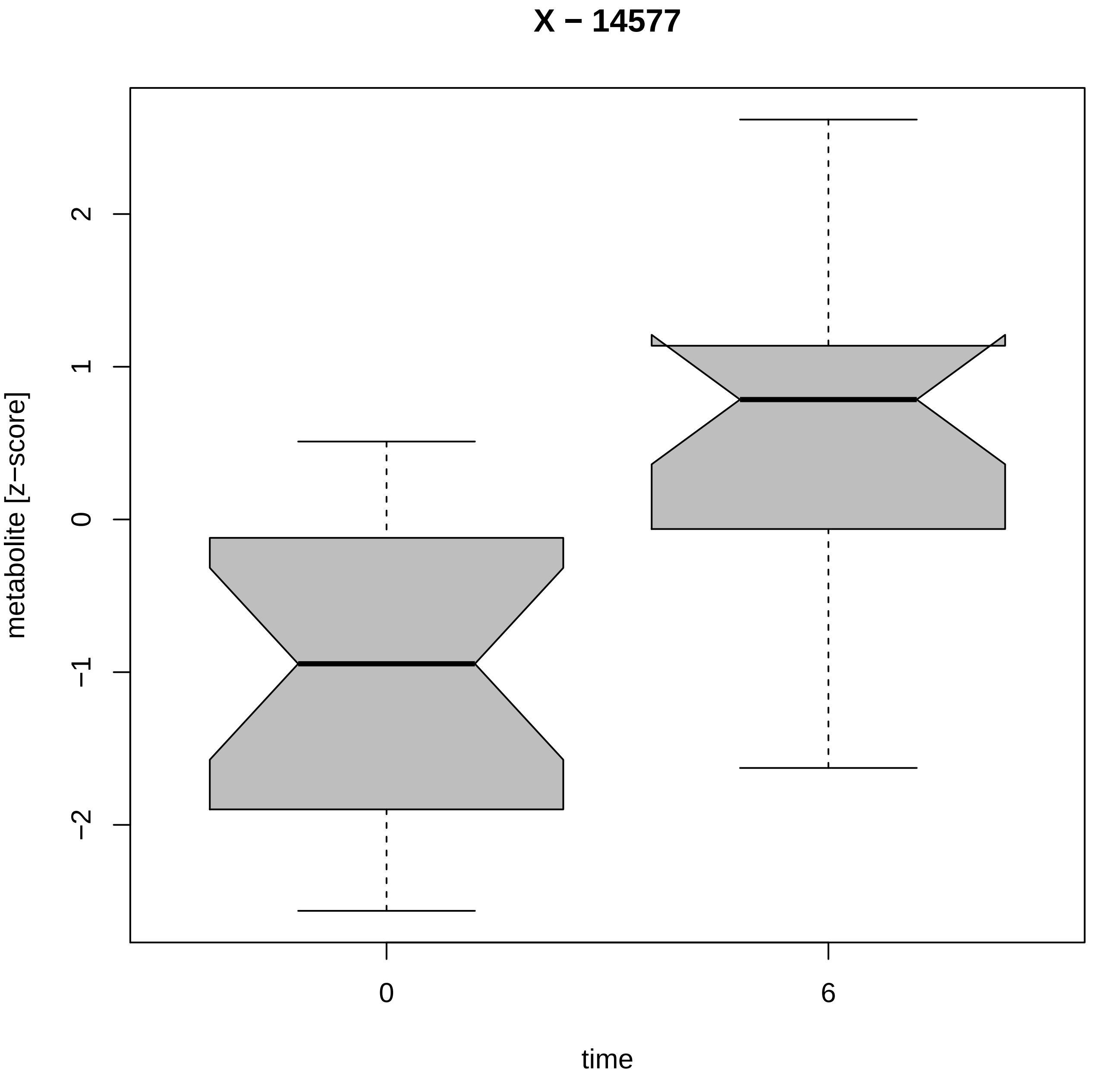
X − 14577
metabolite [z−score]
2
1
0
−1
−2
0
6
time

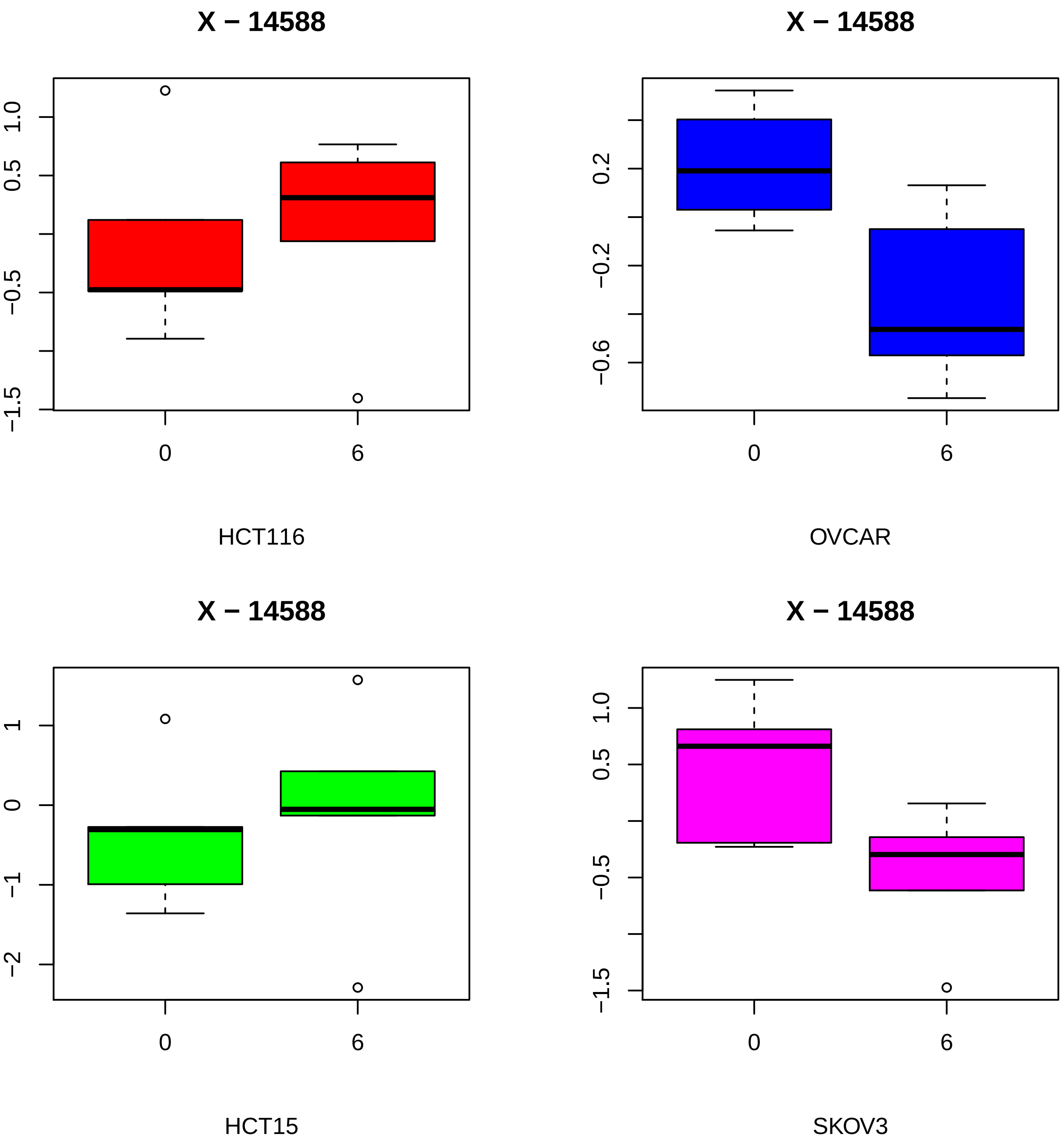
X − 14588
1.0
0.5
−0.5
−1.5
0
6
HCT116
X − 14588
0.2
−0.2
−0.6
0
6
OVCAR
X − 14588
1
0
−1
−2
0
6
HCT15
X − 14588
1.0
0.5
−0.5
−1.5
0
6
SKOV3

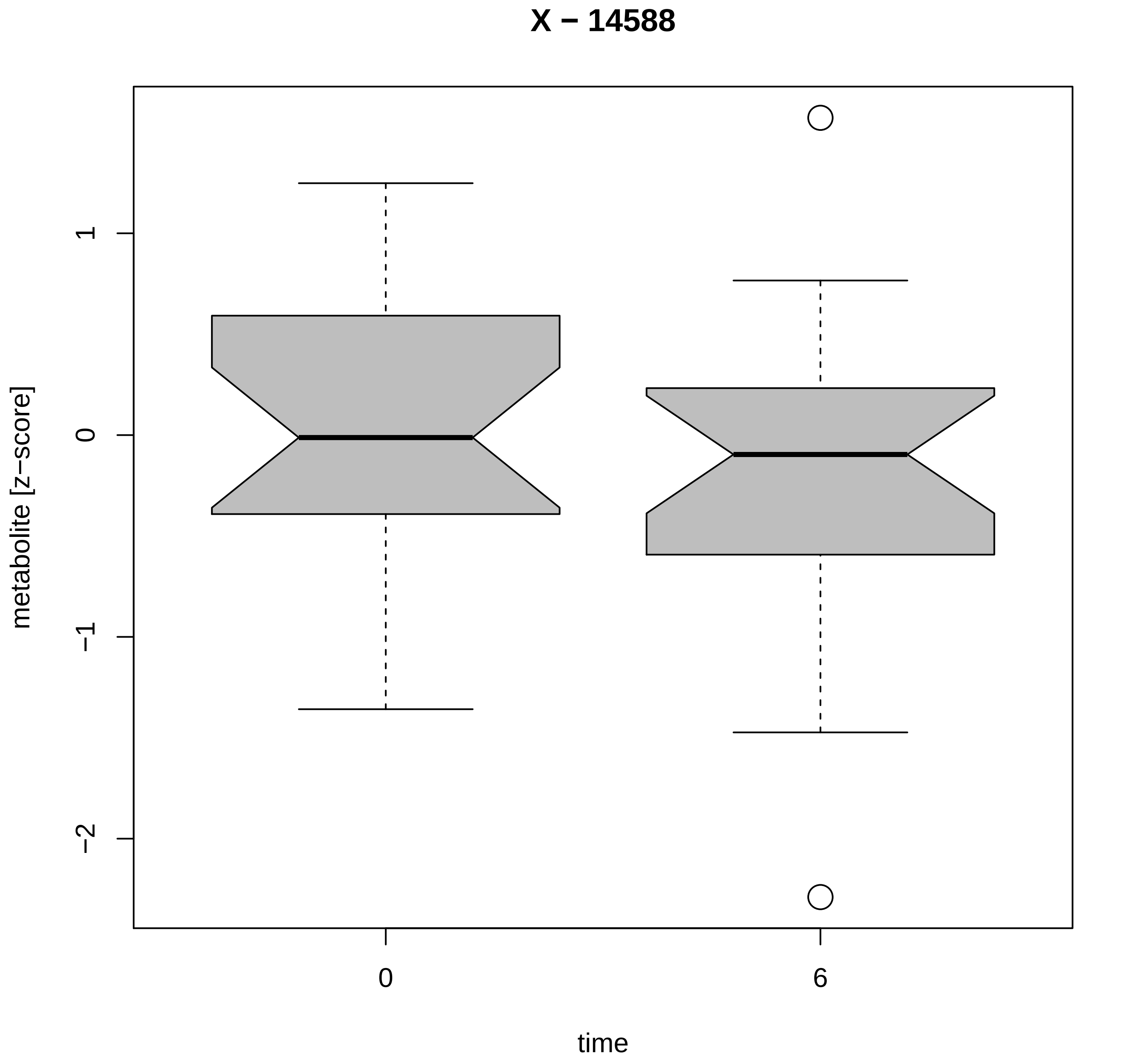
X − 14588
metabolite [z−score]
1
0
−1
−2
0
6
time

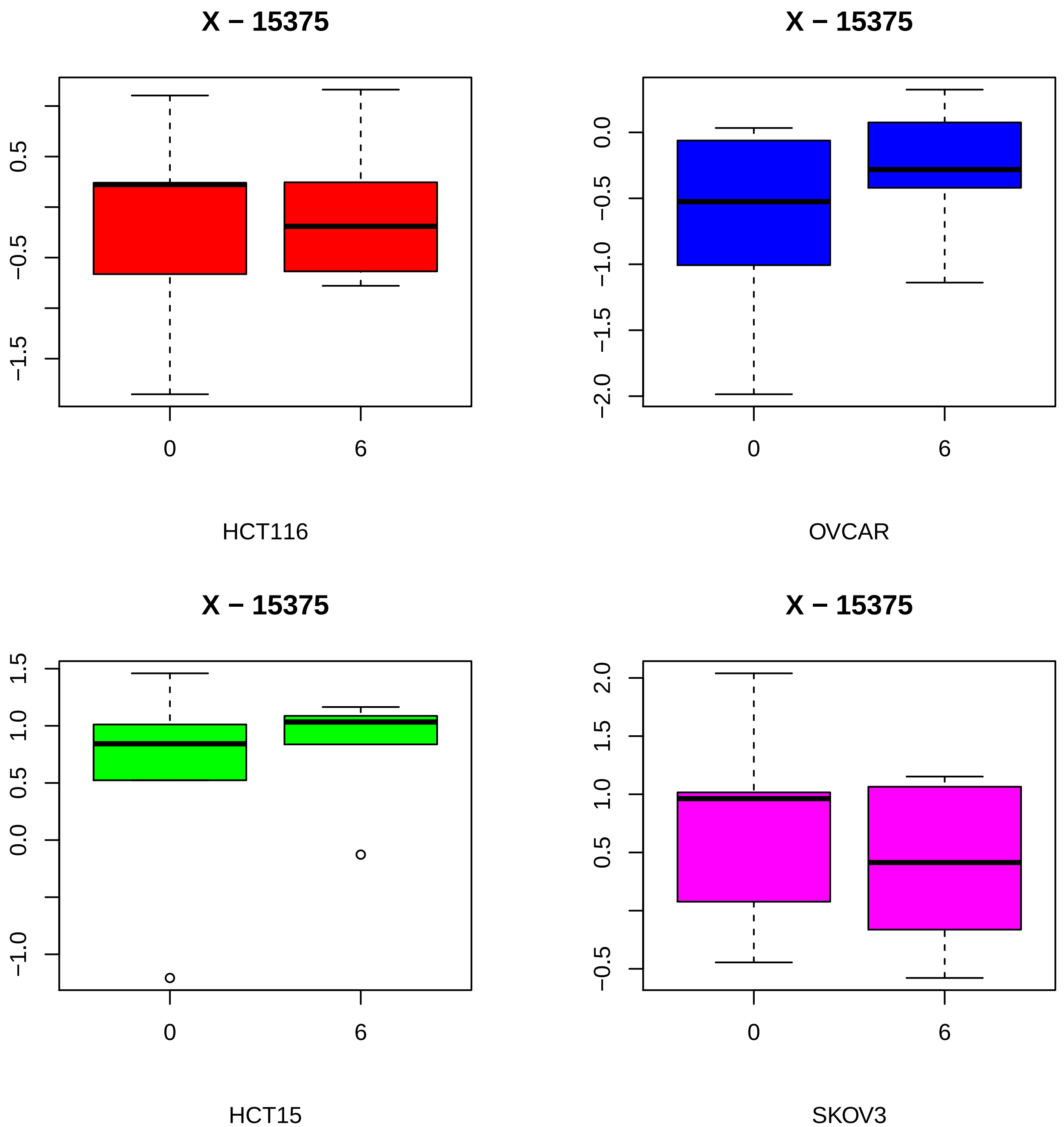

X − 15375
0.5
−0.5
−1.5
0
6
HCT116
X − 15375
0.0
−0.5
−1.0
−1.5
−2.0
0
6
OVCAR
X − 15375
1.5
1.0
0.5
0.0
−1.0
0
6
HCT15
X − 15375
2.0
1.5
1.0
0.5
−0.5
0
6
SKOV3

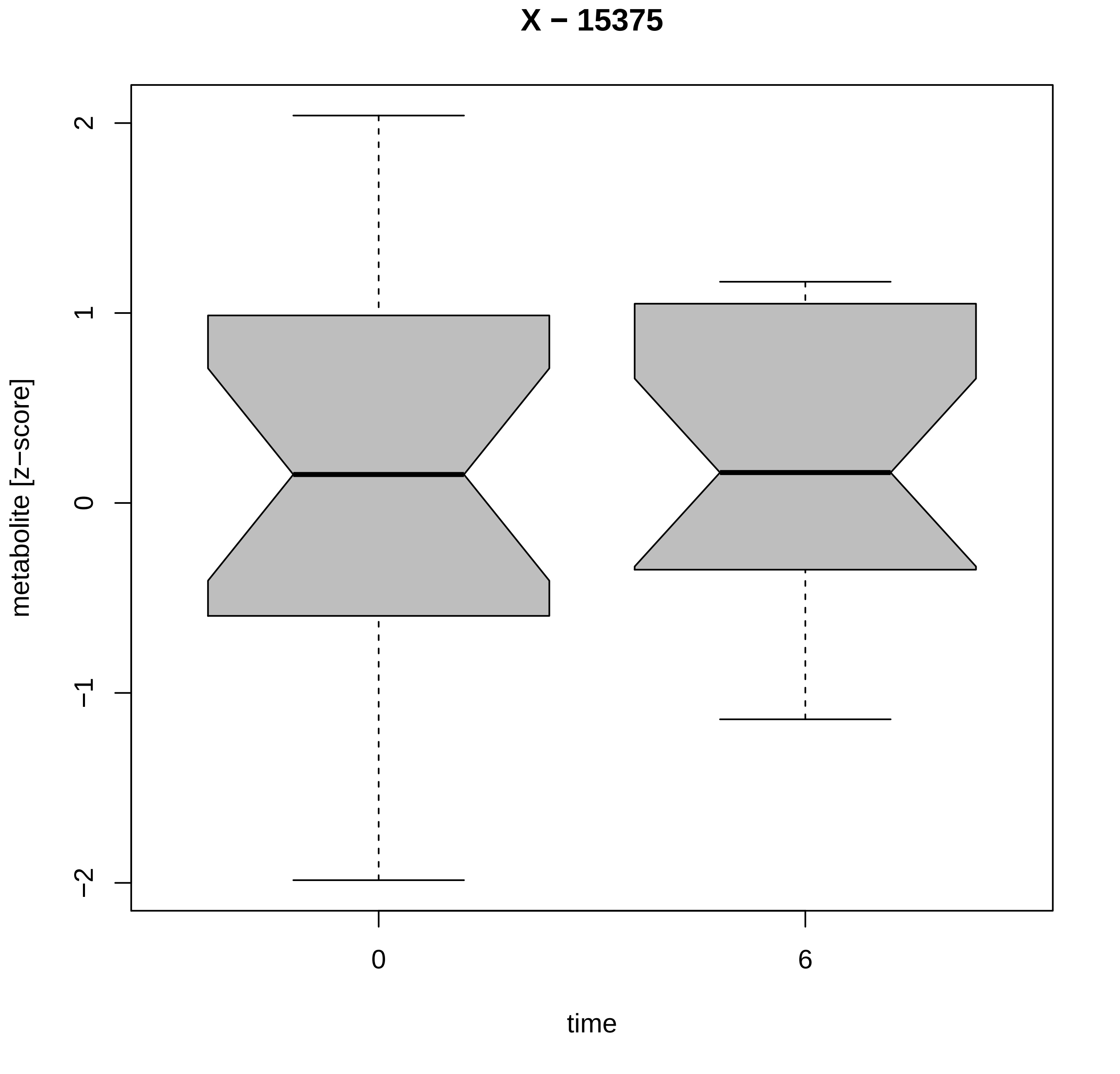
X − 15375
metabolite [z−score]
2
1
0
−1
−2
0
6
time

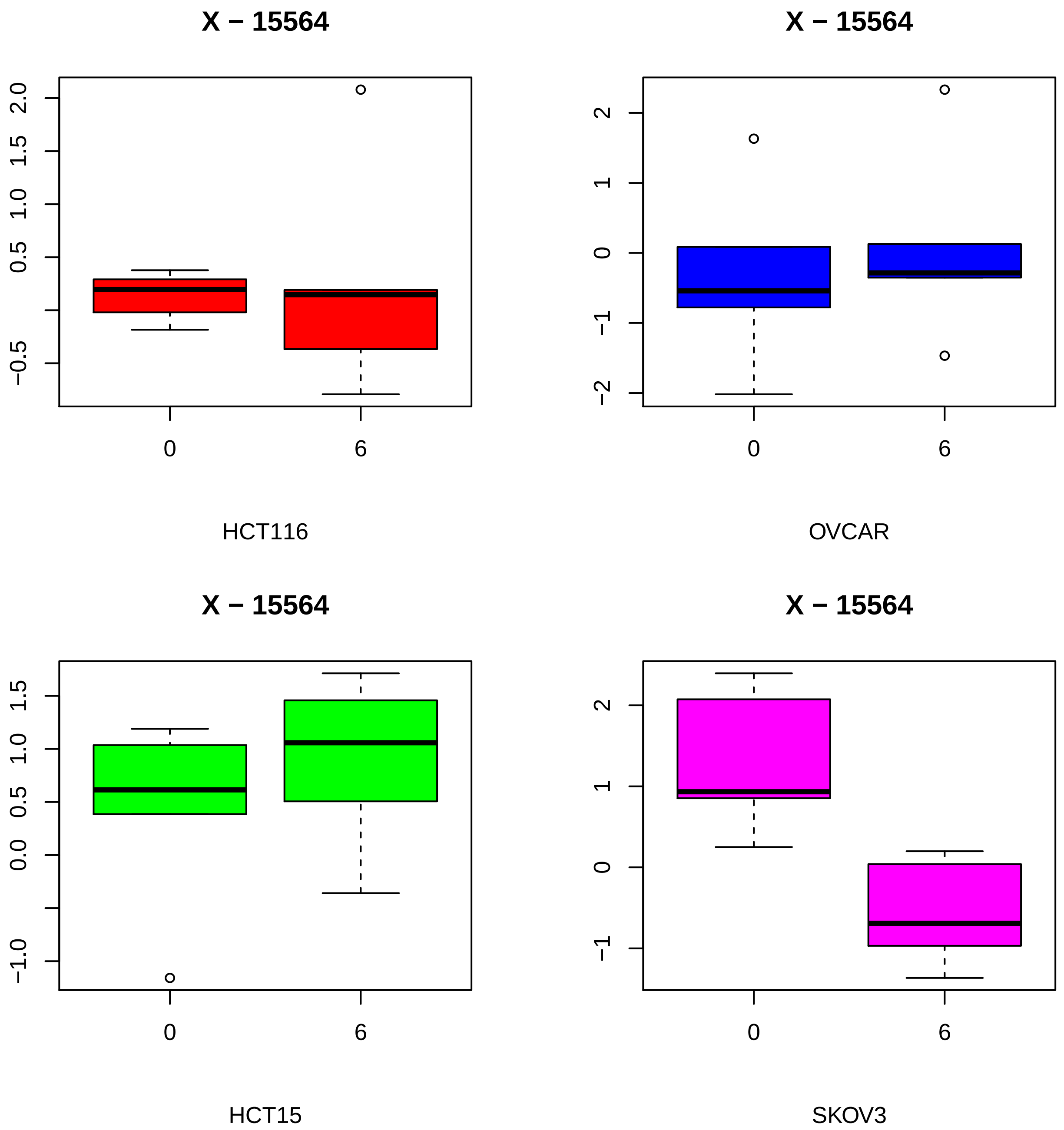
X − 15564
HCT116
X − 15564
OVCAR
X − 15564
HCT15
X − 15564
SKOV3
0
6
2.0
1.5
1.0
0.5
−0.5
2
1
0
−1
−2
0.0
−1.0

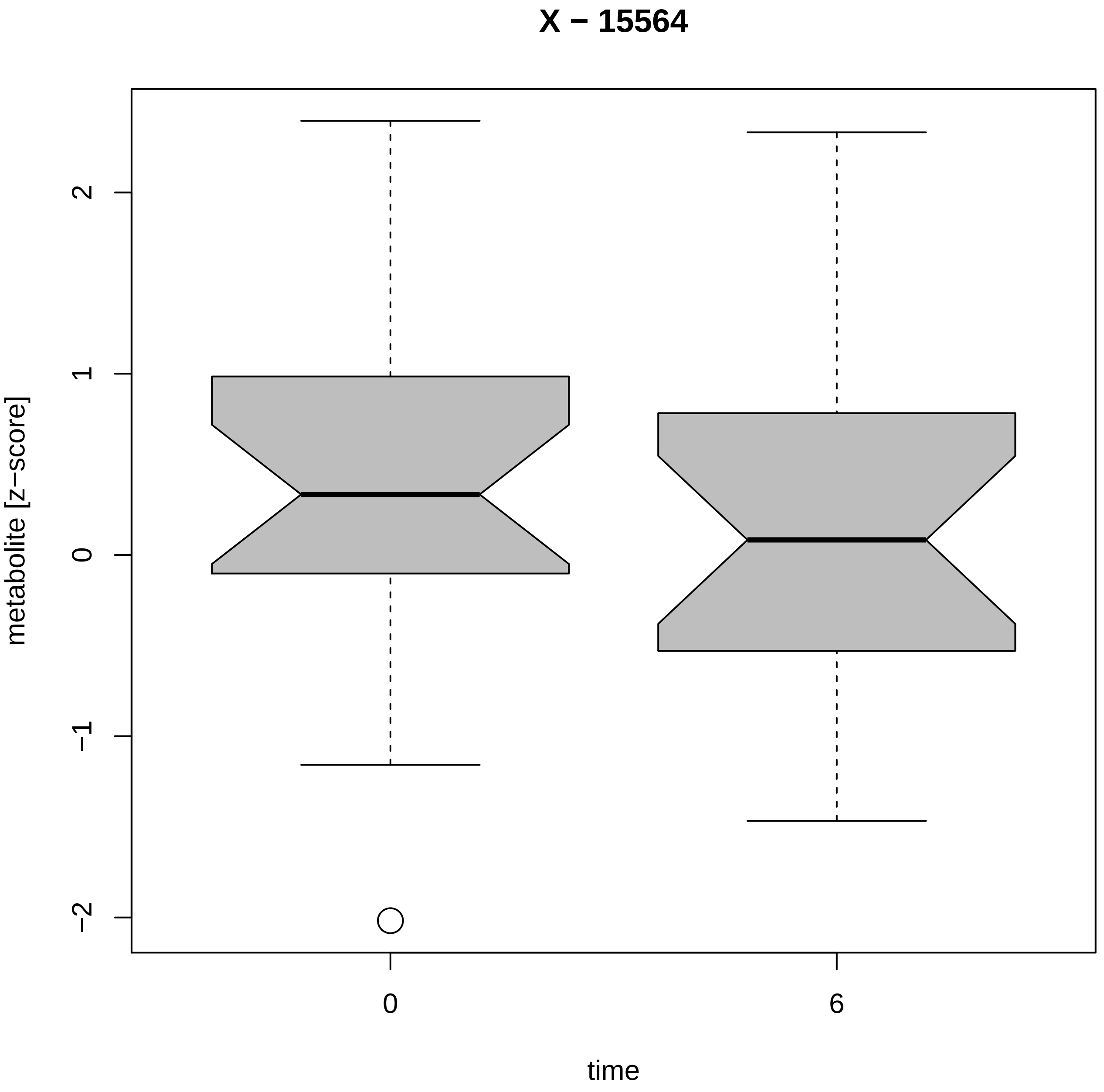
X − 15564
metabolite [z−score]
2
1
0
−1
−2
0
6
time
