# Supplementary material for: Nesting of colon and ovarian cancer cells in the endothelial niche is associated with alterations in glycan and lipid metabolism
Source: Sci Rep. 2017 Jan 4;7:39999. doi: 10.1038/srep39999 (PMC5209689; doi:10.1038/srep39999)
Supplement: Supplementary Figure 5 [file srep39999-s8.pdf]

Nesting of colon and ovarian cancer cells in the endothelial niche is associated with alterations in glycan and lipid metabolism.

Anna Halama[1†], Bella S Guerrouahen[2,3,4†], Jennifer Pasquier[2,3], Noothan J. Satheesh[1†], Karsten Suhre [1,5*] and Arash Rafii [2,3,6*].

†Contributed equally
1. Department of Physiology and Biophysics, Weill Cornell Medicine-Qatar, Qatar-Foundation, P.O. Box 24144, Doha, Qatar.
2. Stem Cell and Microenvironment Laboratory, Weill Cornell Medicine-Qatar, Education City, Qatar Foundation, Doha, Qatar.
3. Department of Genetic Medicine, Weill Cornell Medicine-Qatar, New York, NY 10065, USA.
4. Translational Medicine Division-Research Department, Sidra Medical and Research Center, PO Box 26999, Doha, Qatar.
5. Institute of Bioinformatics and Systems Biology, Helmholtz Zentrum München, German Research Center for Environmental Health, Neuherberg, Germany.
6. Department of Genetic Medicine and Obstetrics and Gynecology, Weill Cornell Medical College, Stem Cell and Microenvironment Laboratory, Weill Cornell Medical College in Qatar, Qatar-Foundation, P.O. Box 24144, Doha, Qatar.

Corresponding authors:
Dr. Karsten Suhre: Department of Physiology and Biophysics, Weill Cornell Medicine-Qatar, Doha, Qatar Weill Cornell Medical College in Qatar, Qatar-Foundation, P.O Box: 24144, Doha, Qatar. Phone: +974 4492 8482. Fax: +974 4492 8422. Email: kas2049@qatar-med.cornell.edu

Dr. Arash Rafii: Department of Genetic Medicine and Obstetrics and Gynecology, Weill Cornell Medicine-Qatar, Stem cell and microenvironment laboratory Weill Cornell Medical College in Qatar, Qatar-Foundation, P.O Box: 24144, Doha, Qatar. Phone: +974 3313 5828. Fax: +974 4492 8422. Email: jat2021@qatar-med.cornell.edu

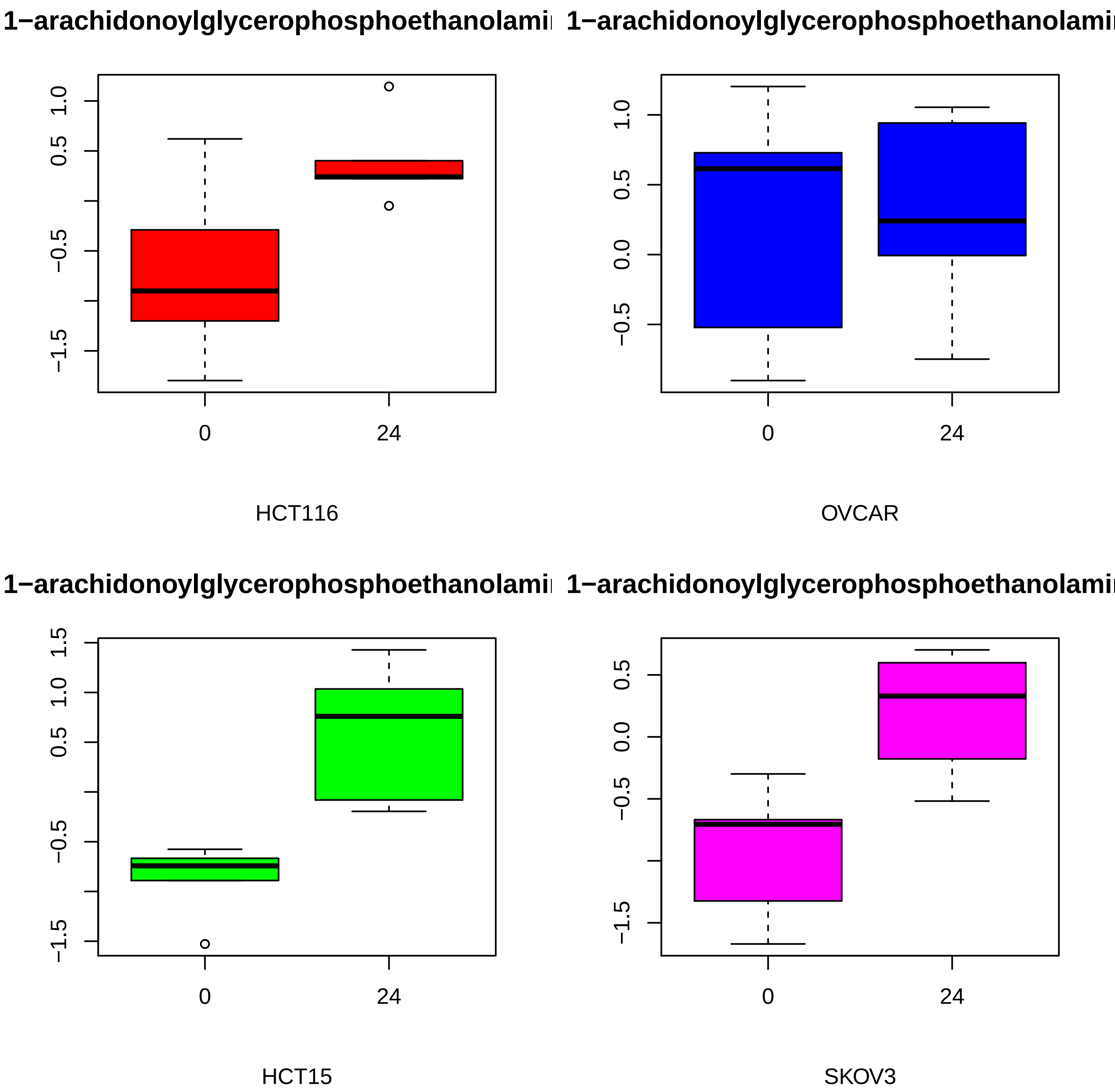
1−arachidonoylglycerophosphoethanolami
1.0
0.5
−0.5
−1.5
0
24
HCT116
1−arachidonoylglycerophosphoethanolami
1.0
0.5
0.0
−0.5
0
24
OVCAR
1−arachidonoylglycerophosphoethanolami
1.5
1.0
0.5
−0.5
−1.5
0
24
HCT15
1−arachidonoylglycerophosphoethanolami
0.5
0.0
−0.5
−1.5
0
24
SKOV3

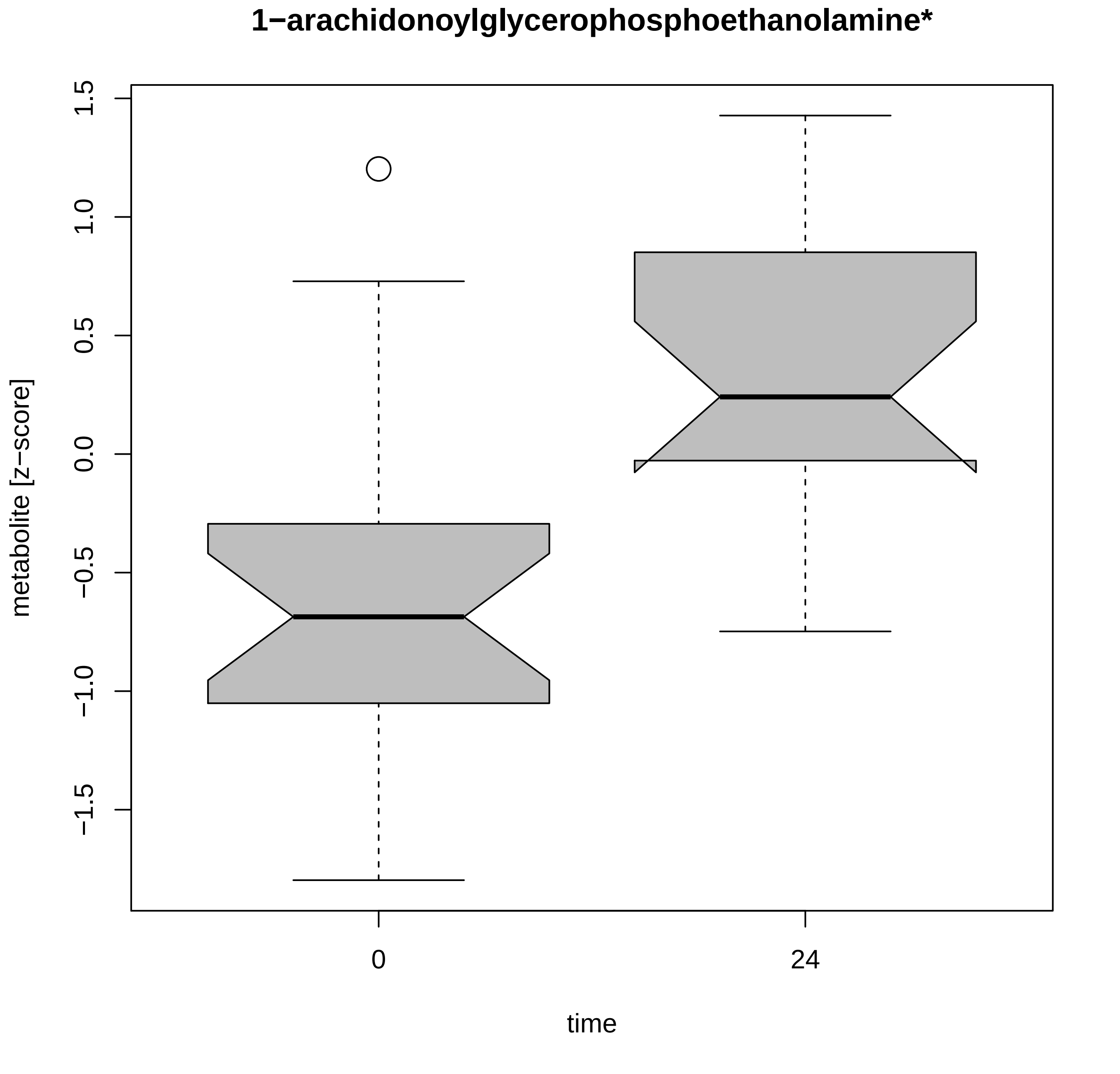

1−arachidonoylglycerophosphoethanolamine*
metabolite [z−score]
1.5
1.0
0.5
0.0
−0.5
−1.0
−1.5
0
24
time

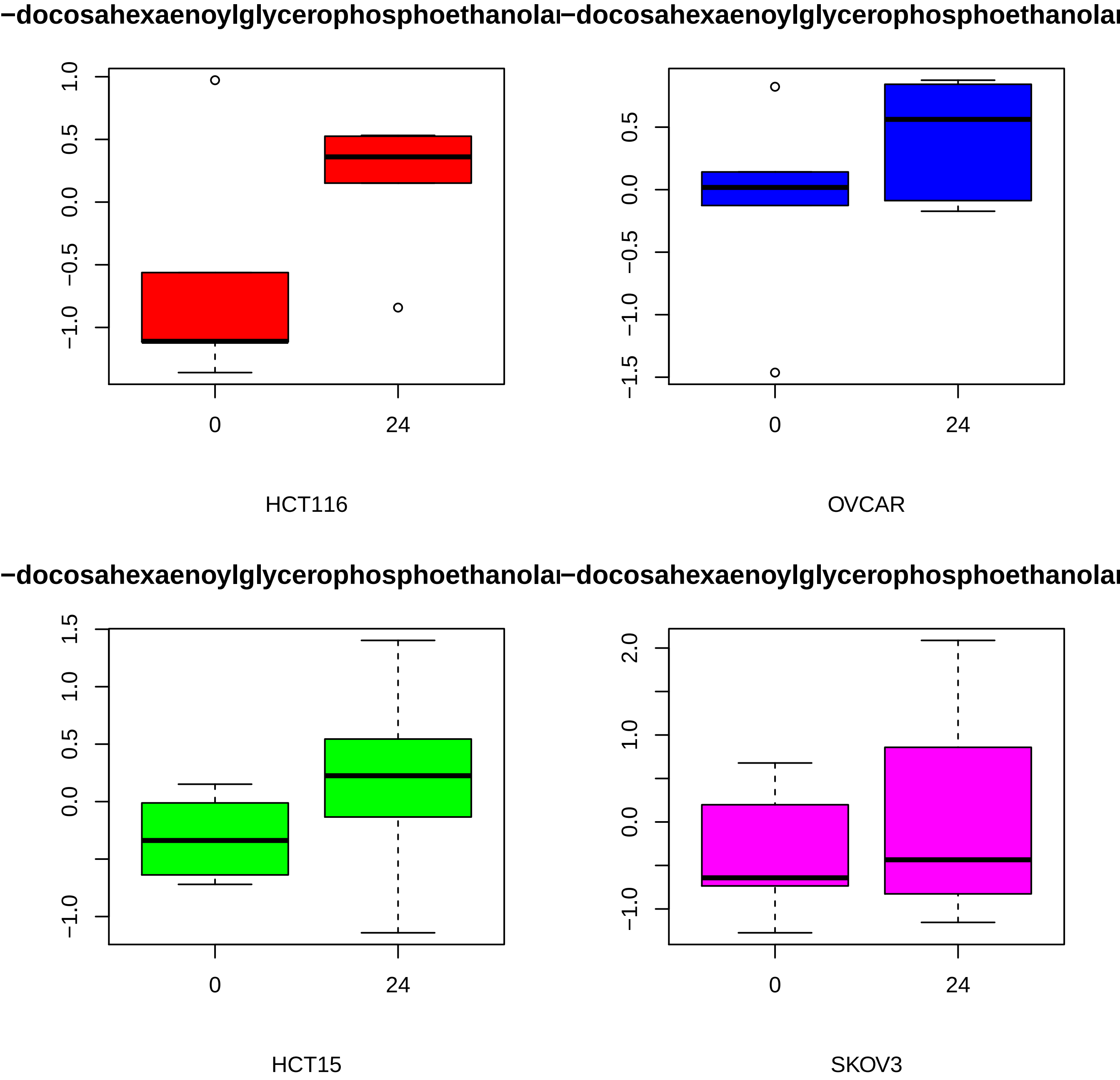
−docosahexaenoylglycerophosphoethanolaı
−docosahexaenoylglycerophosphoethanolar
−docosahexaenoylglycerophosphoethanolaı
−docosahexaenoylglycerophosphoethanolar
1.0
0.5
0.0
−0.5
−1.0
−1.5
1.5
2.0
0
24
HCT116
OVCAR
HCT15
SKOV3

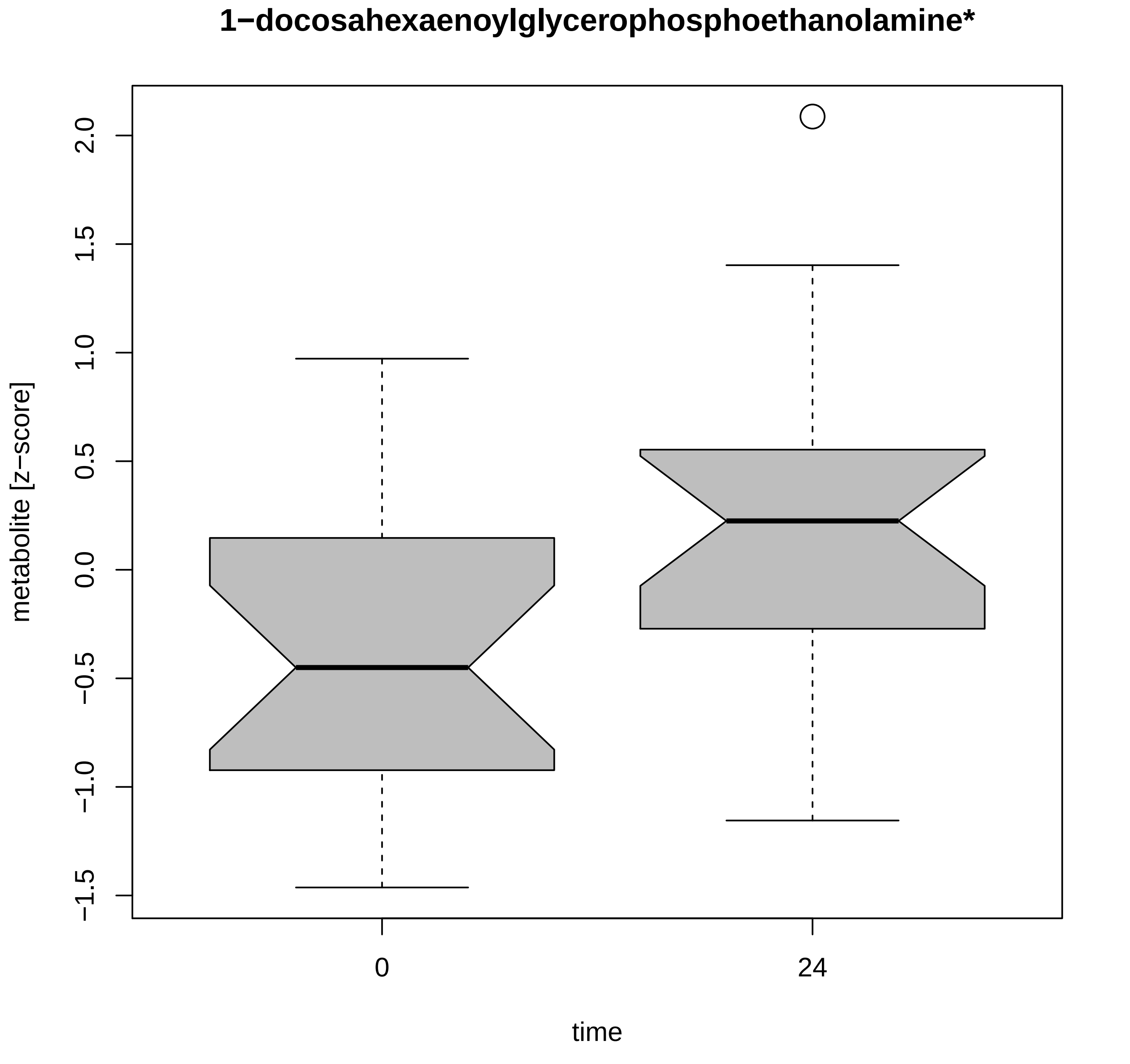

1−docosahexaenoylglycerophosphoethanolamine*
metabolite [z−score]
2.0
1.5
1.0
0.5
0.0
−0.5
−1.0
−1.5
0
24
time

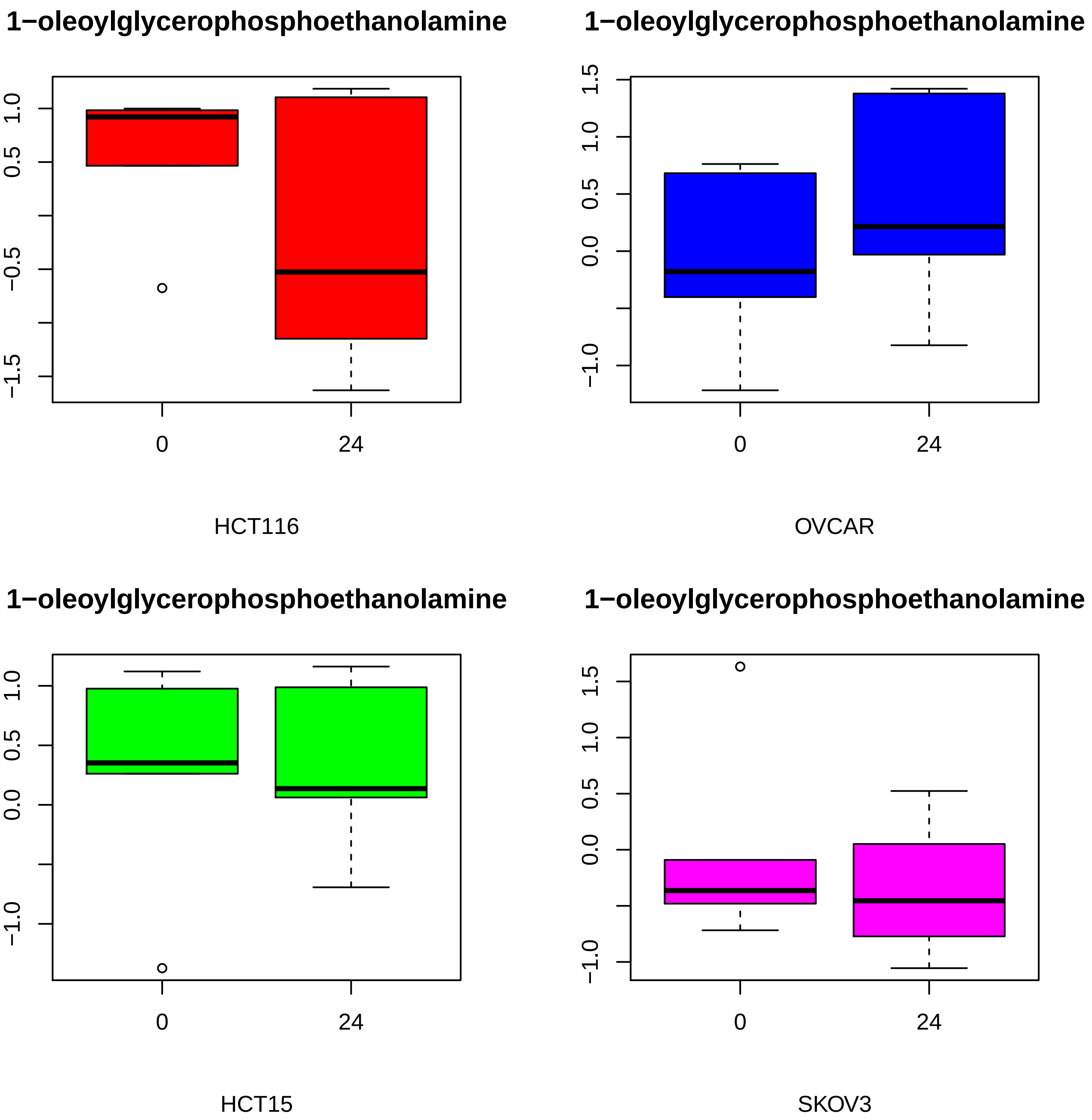
1−oleoylglycerophosphoethanolamine
1.0
0.5
−0.5
−1.5
0
24
HCT116
1−oleoylglycerophosphoethanolamine
1.5
1.0
0.5
0.0
−1.0
0
24
OVCAR
1−oleoylglycerophosphoethanolamine
1.0
0.5
0.0
−1.0
0
24
HCT15
1−oleoylglycerophosphoethanolamine
1.5
1.0
0.5
0.0
−1.0
0
24
SKOV3

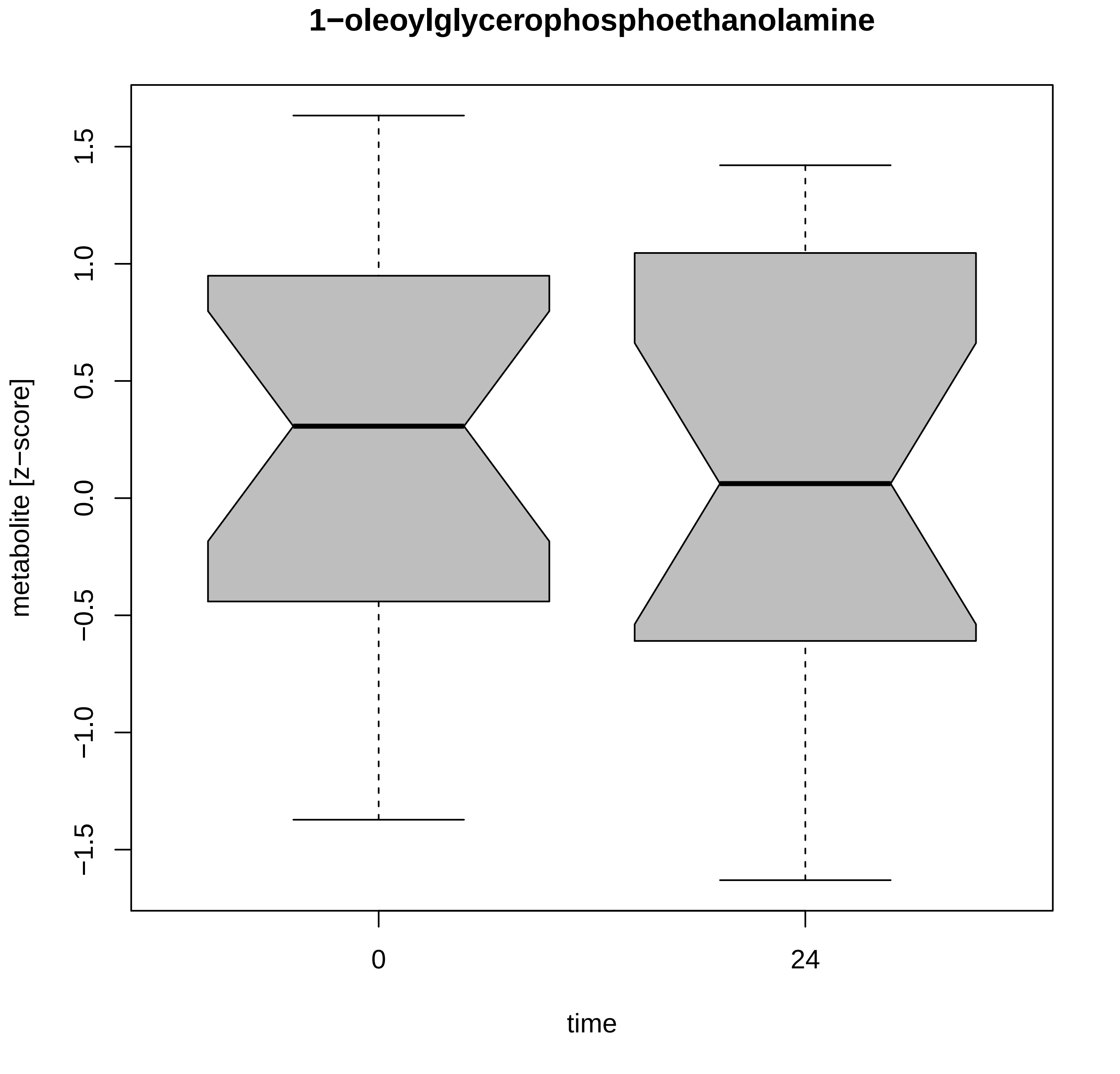
1−oleoylglycerophosphoethanolamine
metabolite [z−score]
1.5
1.0
0.5
0.0
−0.5
−1.0
−1.5
0
24
time

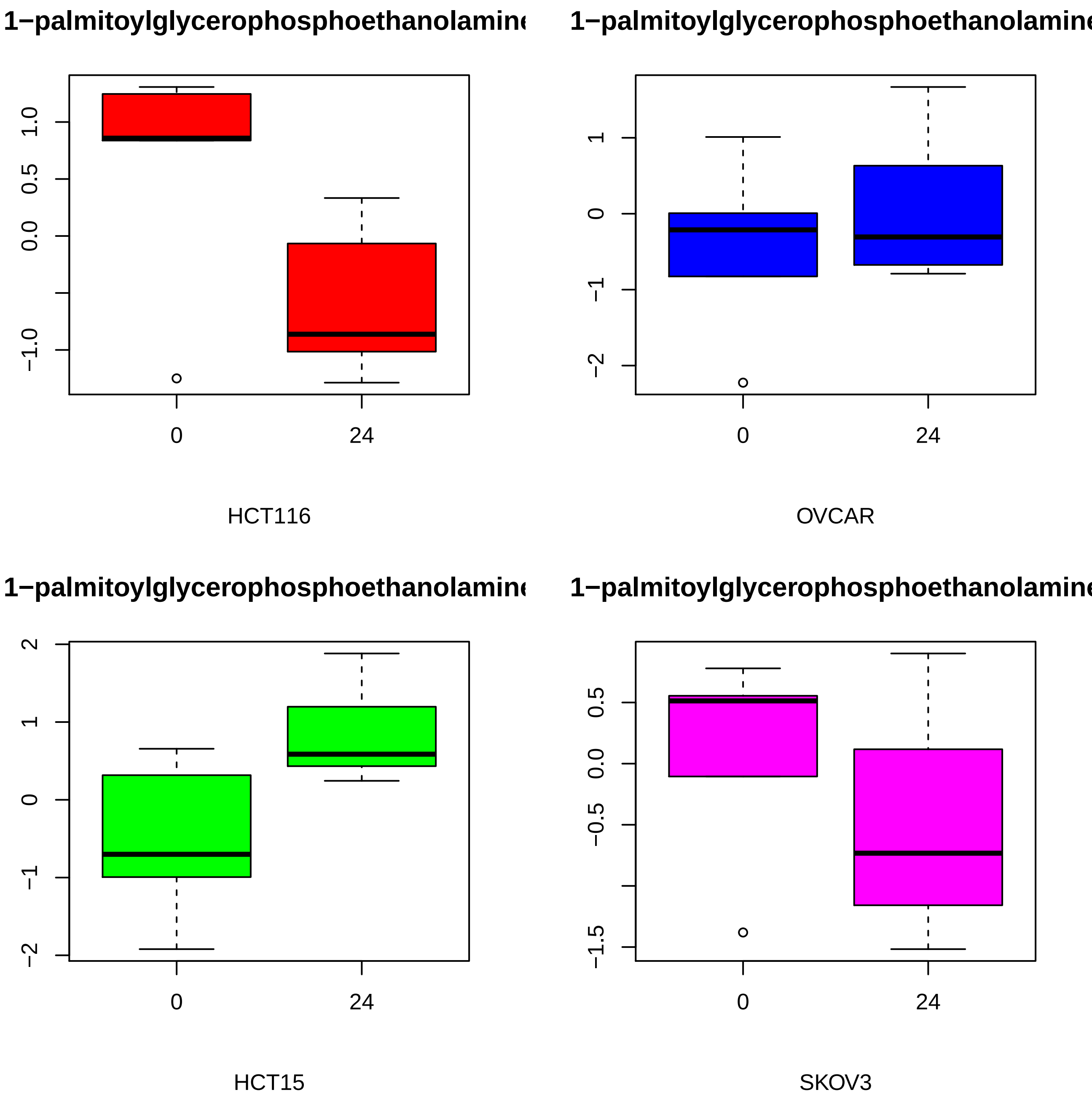
1−palmitoylglycerophosphoethanolamine
1.0
0.5
0.0
−1.0
0
24
HCT116
1−palmitoylglycerophosphoethanolamine
1
0
−1
−2
0
24
OVCAR
1−palmitoylglycerophosphoethanolamine
2
1
0
−1
−2
0
24
HCT15
1−palmitoylglycerophosphoethanolamine
0.5
0.0
−0.5
−1.5
0
24
SKOV3

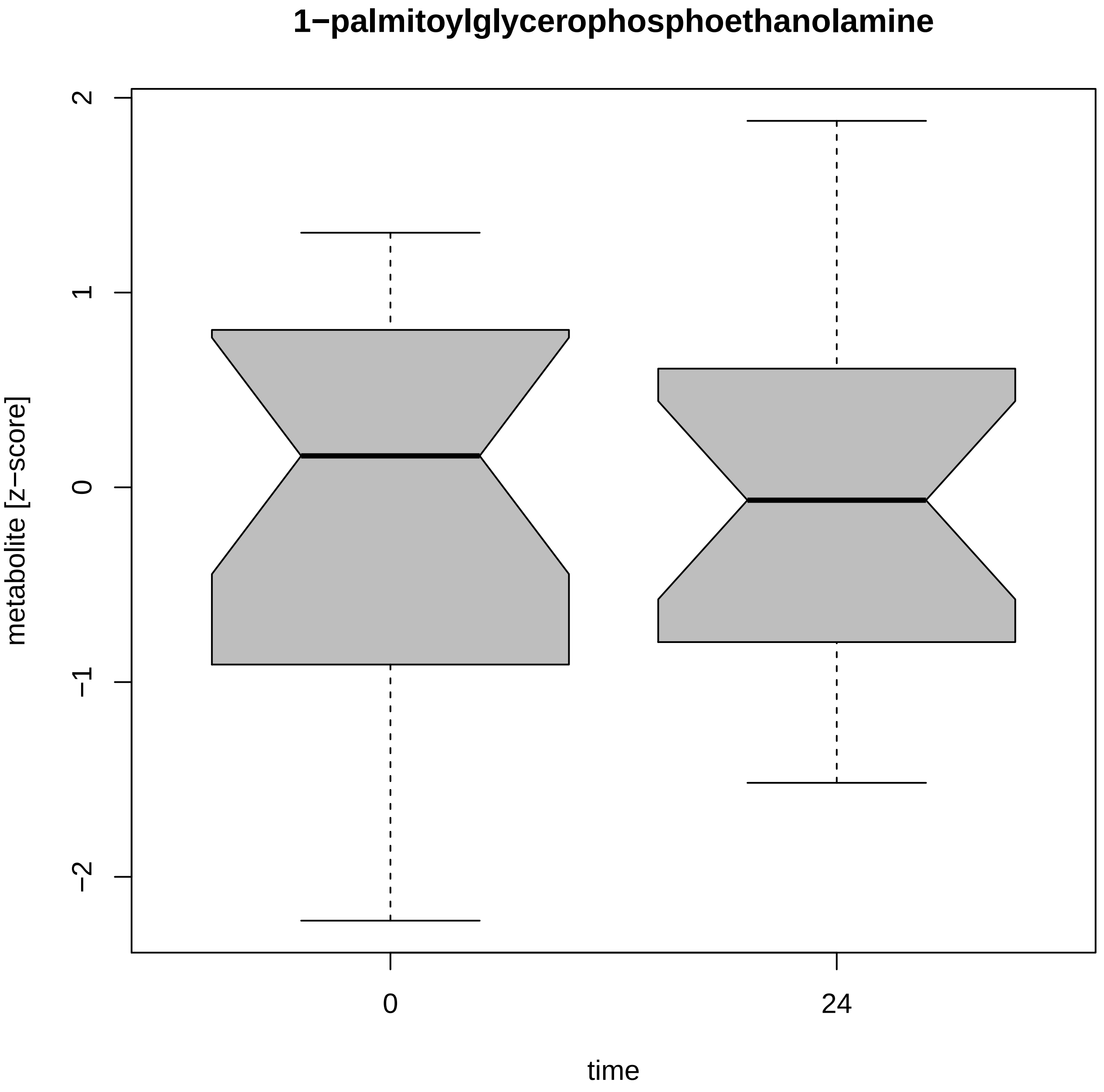
1−palmitoylglycerophosphoethanolamine
metabolite [z−score]
2
1
0
−1
−2
0
24
time

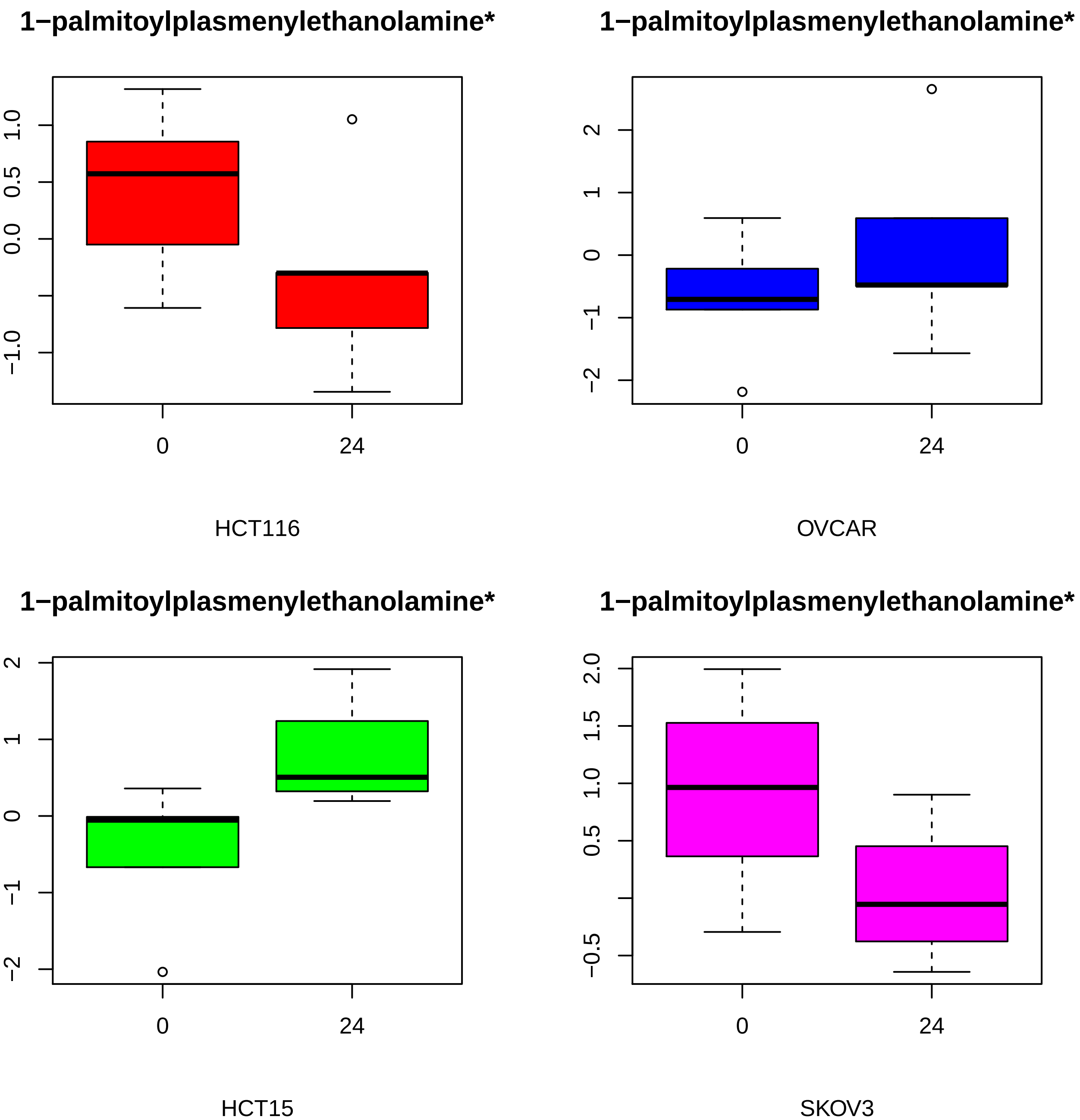
1−palmitoylplasmenylethanolamine*
1.0
0.5
0.0
−1.0
0
24
HCT116
1−palmitoylplasmenylethanolamine*
2
1
0
−1
−2
0
24
OVCAR
1−palmitoylplasmenylethanolamine*
2
1
0
−1
−2
0
24
HCT15
1−palmitoylplasmenylethanolamine*
2.0
1.5
1.0
0.5
−0.5
0
24
SKOV3

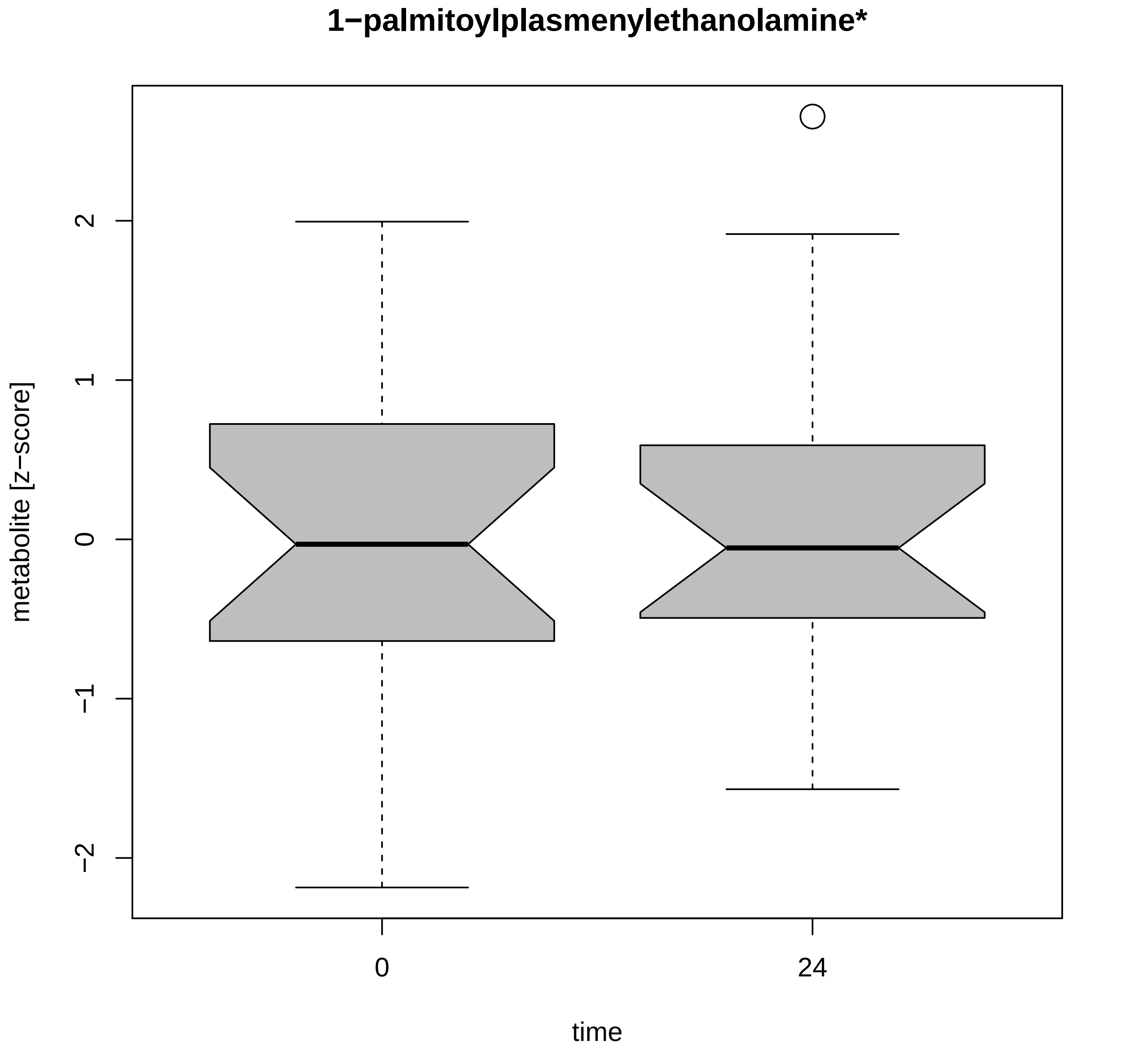
1−palmitoylplasmenylethanolamine*
metabolite [z−score]
2
1
0
−1
−2
0
24
time

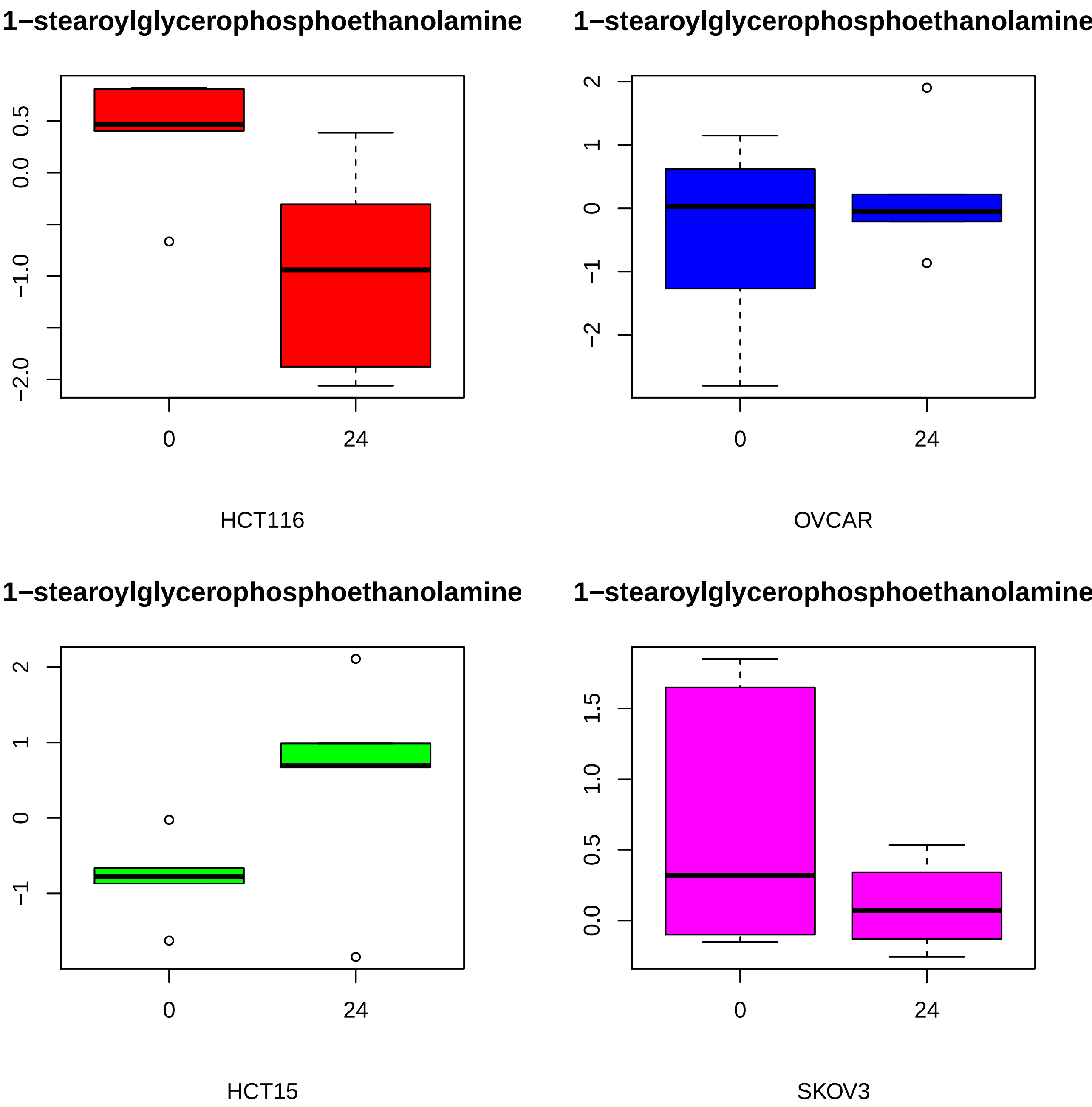
1−stearoylglycerophosphoethanolamine
−2.0
−1.0
0.0
0.5
0
24
HCT116
1−stearoylglycerophosphoethanolamine
−2
−1
0
1
2
0
24
OVCAR
1−stearoylglycerophosphoethanolamine
−1
0
1
2
0
24
HCT15
1−stearoylglycerophosphoethanolamine
0.0
0.5
1.0
1.5
0
24
SKOV3

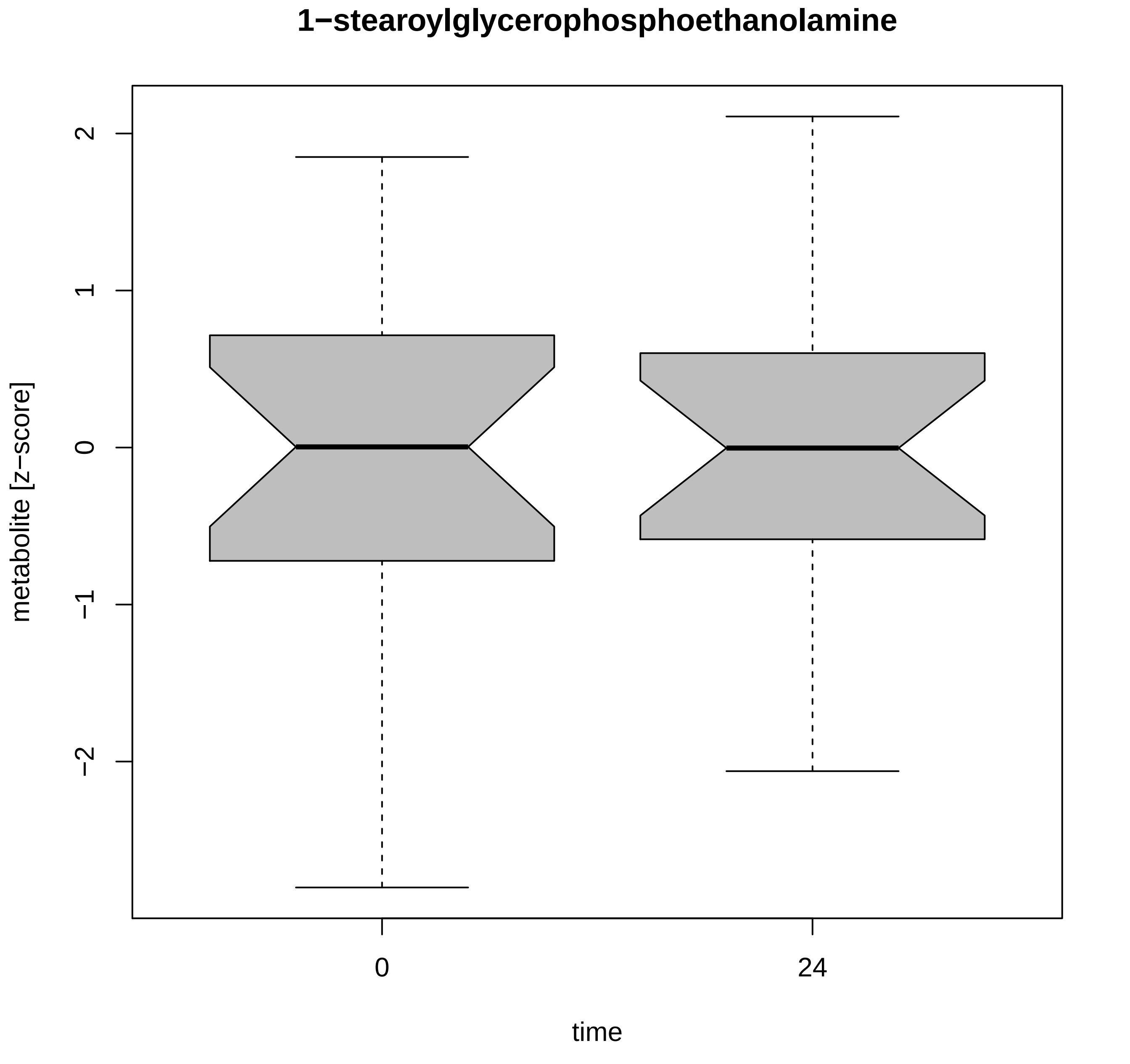
1−stearoylglycerophosphoethanolamine
metabolite [z−score]
2
1
0
−1
−2
0
24
time

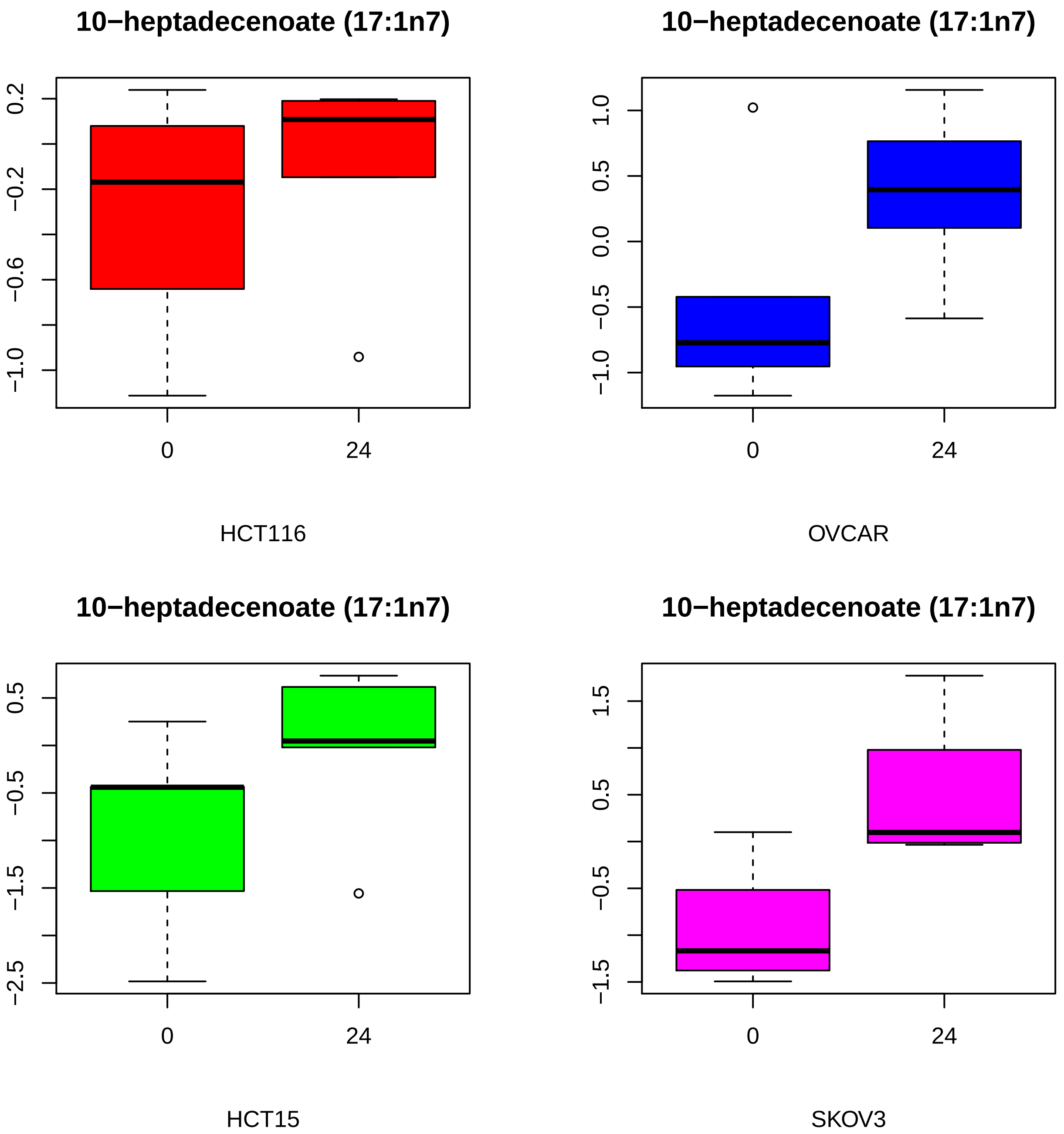
10−heptadecenoate (17:1n7)
0.2
−0.2
−0.6
−1.0
0
24
HCT116
10−heptadecenoate (17:1n7)
1.0
0.5
0.0
−0.5
−1.0
0
24
OVCAR
10−heptadecenoate (17:1n7)
0.5
−0.5
−1.5
−2.5
0
24
HCT15
10−heptadecenoate (17:1n7)
1.5
0.5
−0.5
−1.5
0
24
SKOV3

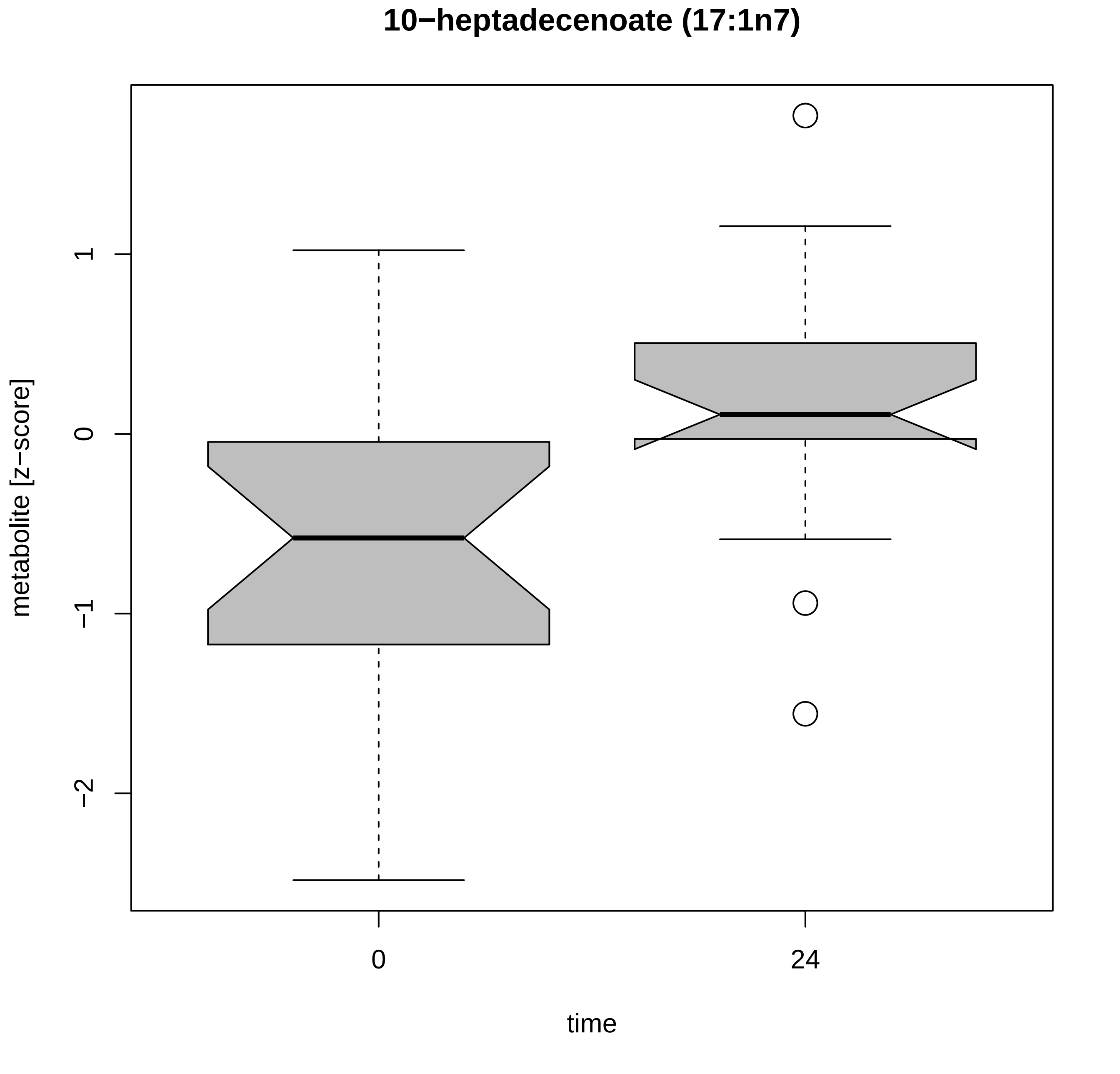
10−heptadecenoate (17:1n7)
metabolite [z−score]
−2
−1
0
1
0
24
time

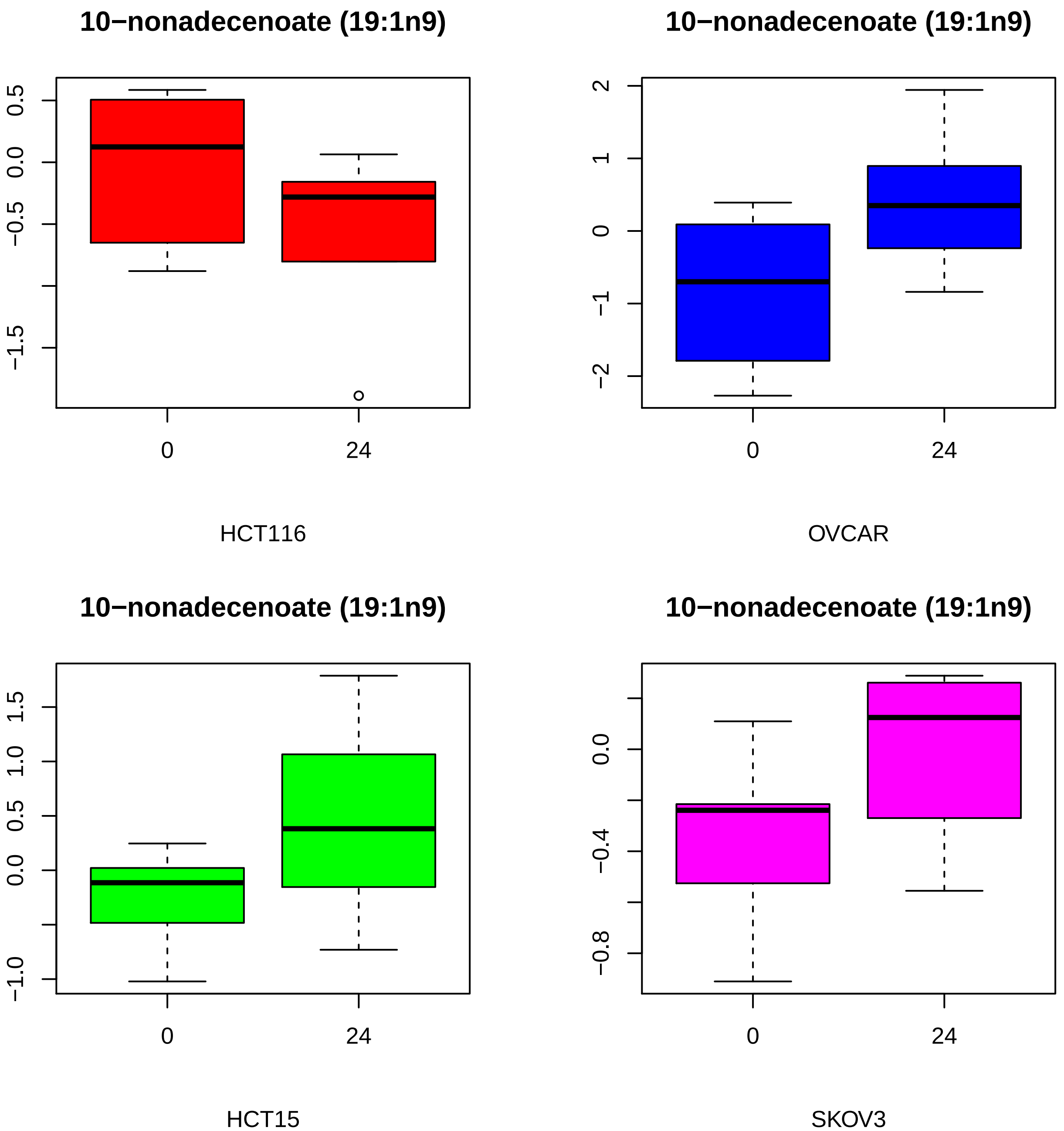

10−nonadecenoate (19:1n9)
0.5
0.0
−0.5
−1.5
0
24
HCT116
10−nonadecenoate (19:1n9)
2
1
0
−1
−2
0
24
OVCAR
10−nonadecenoate (19:1n9)
1.5
1.0
0.5
0.0
−1.0
0
24
HCT15
10−nonadecenoate (19:1n9)
0.0
−0.4
−0.8
0
24
SKOV3

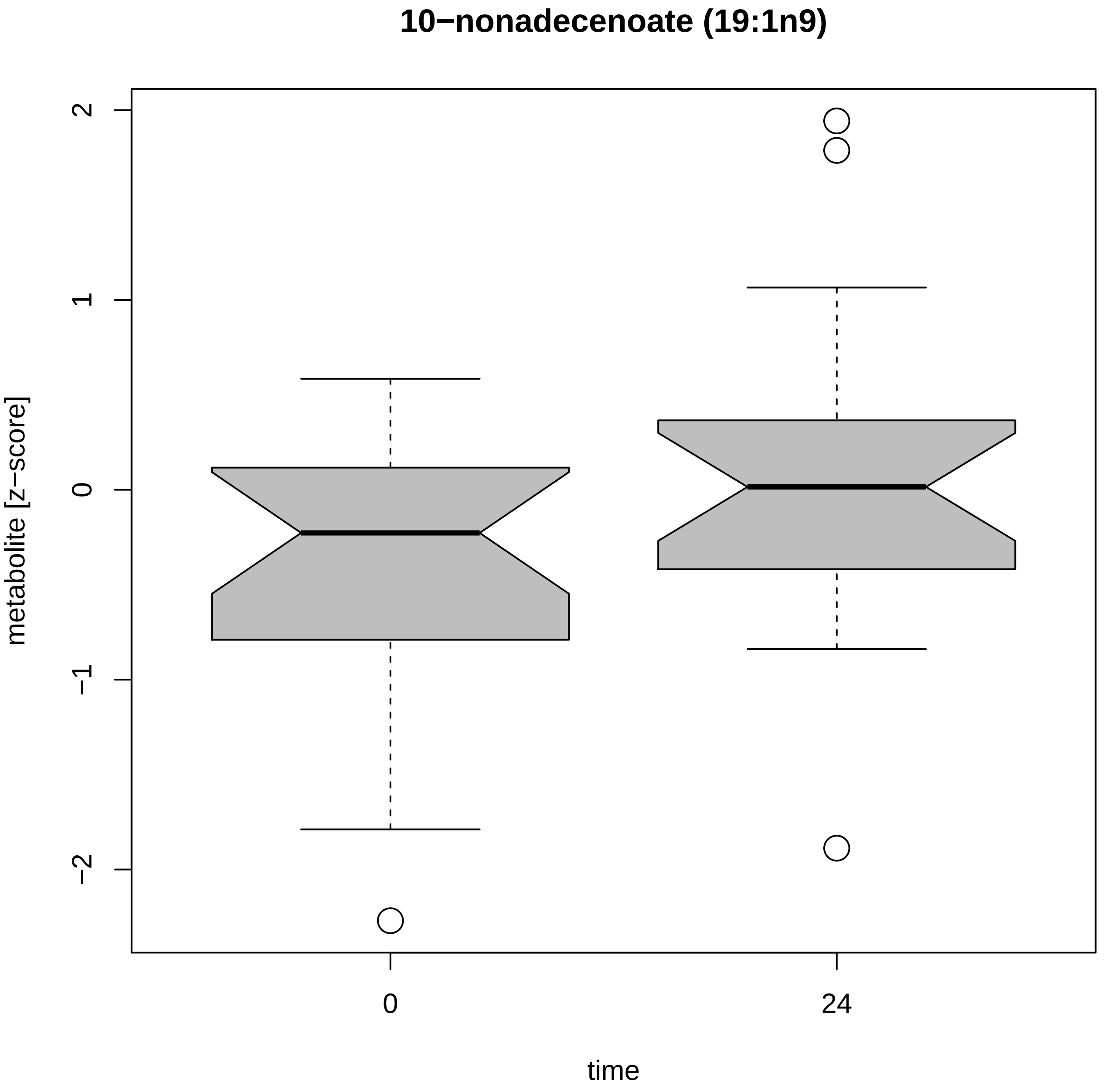

10−nonadecenoate (19:1n9)
metabolite [z−score]
2
1
0
−1
−2
0
24
time

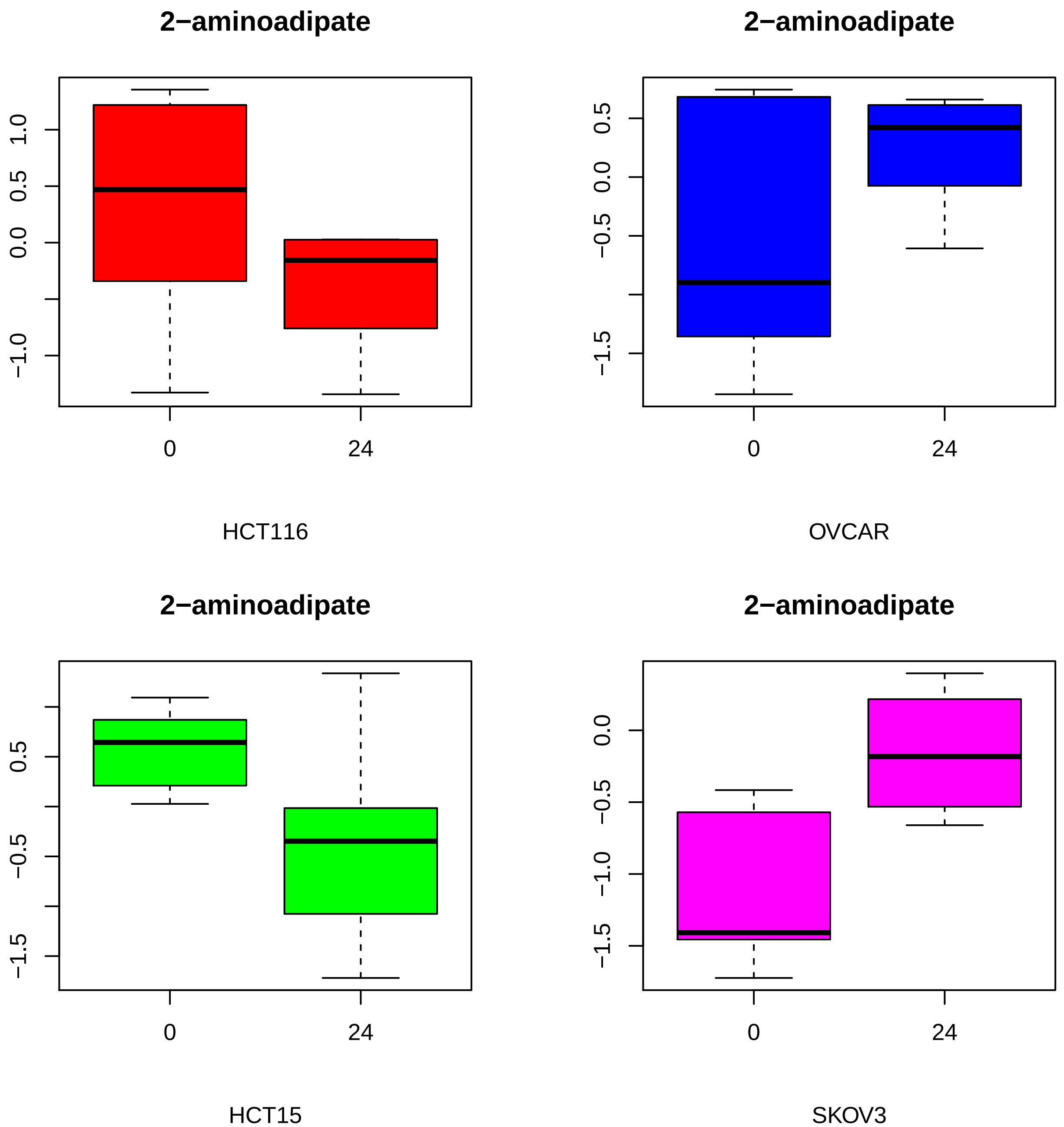
2−aminoadipate
−1.0
0.0
0.5
1.0
0
24
HCT116
2−aminoadipate
−1.5
−0.5
0.0
0.5
0
24
OVCAR
2−aminoadipate
−1.5
−0.5
0.5
0
24
HCT15
2−aminoadipate
−1.5
−1.0
−0.5
0.0
0
24
SKOV3

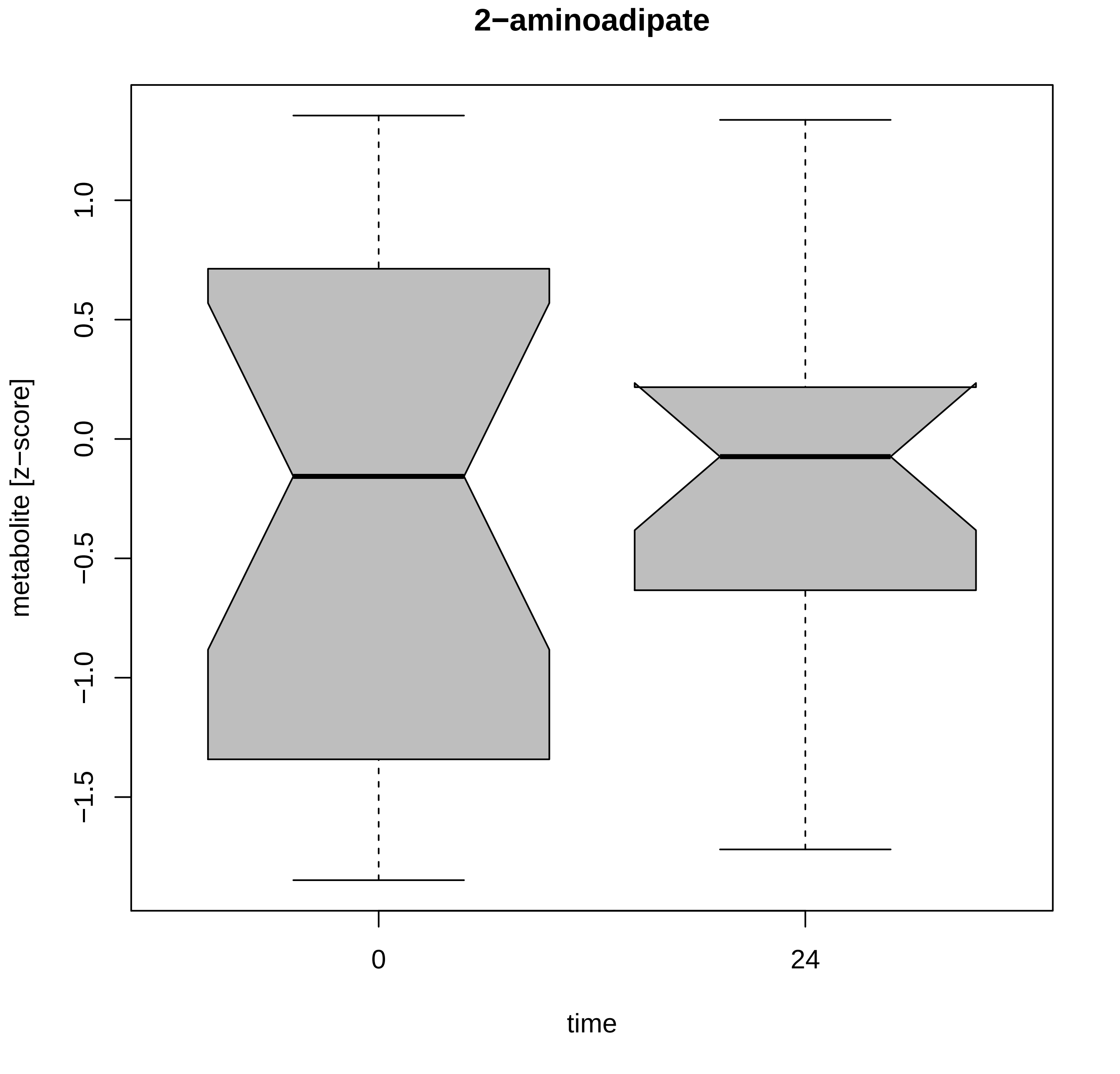

2−aminoadipate
metabolite [z−score]
1.0
0.5
0.0
−0.5
−1.0
−1.5
0
24
time

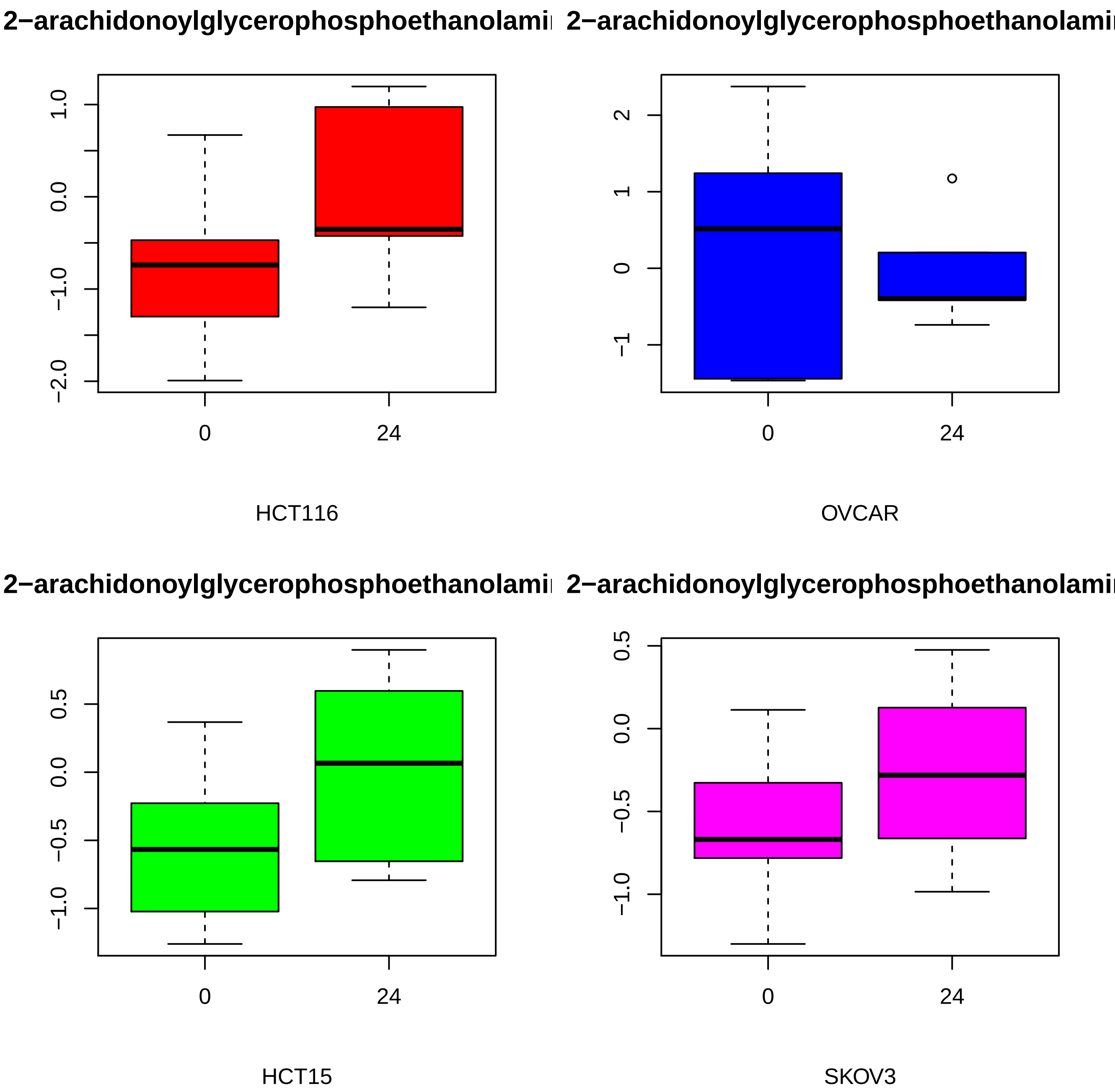

2−arachidonoylglycerophosphoethanolami
2−arachidonoylglycerophosphoethanolami
2−arachidonoylglycerophosphoethanolami
2−arachidonoylglycerophosphoethanolami
1.0
0.0
−1.0
−2.0
2
1
0
−1
0.5
0.0
−0.5
−1.0
0.5
0.0
−0.5
−1.0
0
24
HCT116
OVCAR
HCT15
SKOV3

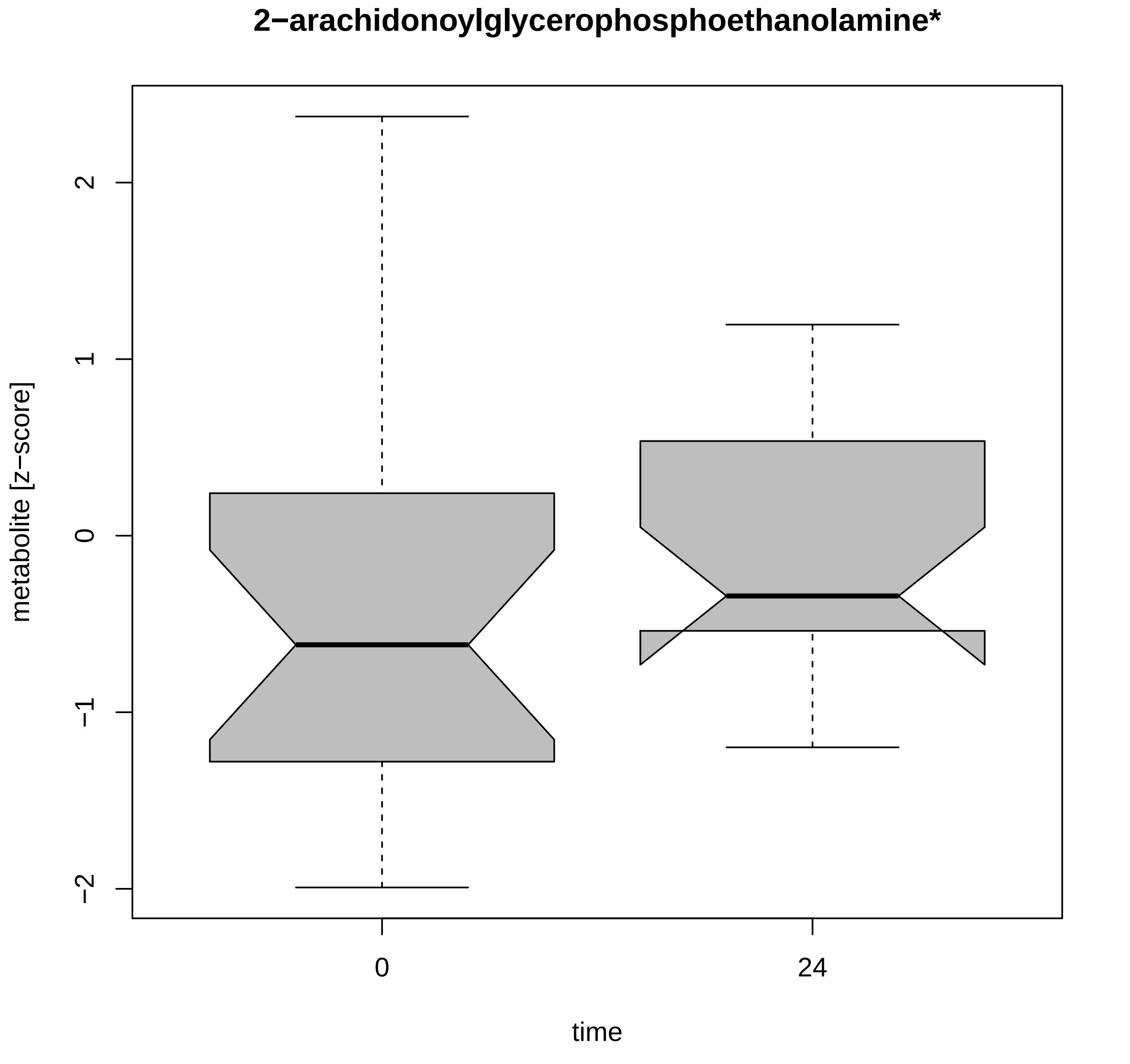

2−arachidonoylglycerophosphoethanolamine*
metabolite [z−score]
2
1
0
−1
−2
0
24
time

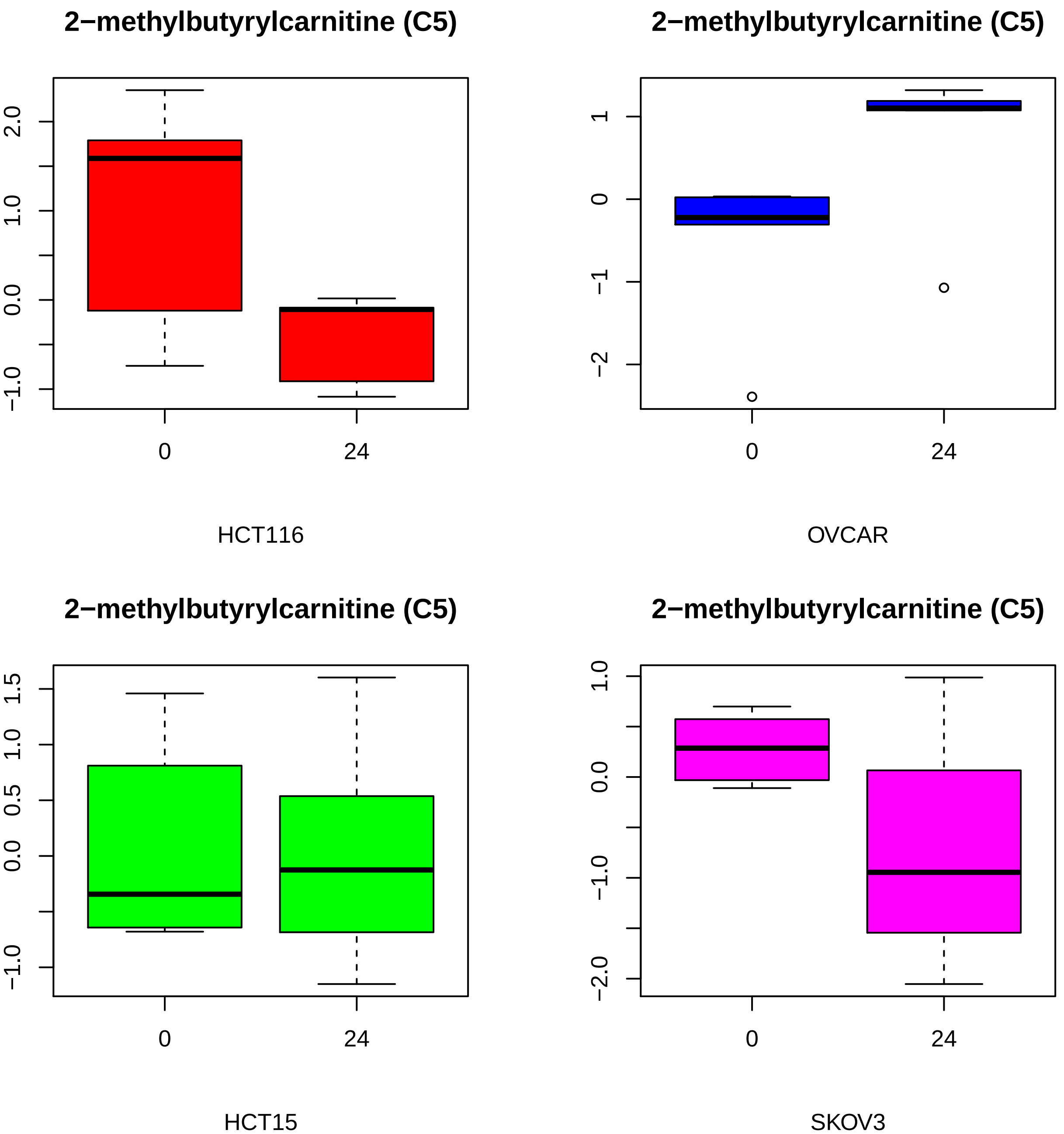
2−methylbutyrylcarnitine (C5)
−1.0
0.0
1.0
2.0
0
24
HCT116
2−methylbutyrylcarnitine (C5)
−2
−1
0
1
0
24
OVCAR
2−methylbutyrylcarnitine (C5)
−1.0
0.0
0.5
1.0
1.5
0
24
HCT15
2−methylbutyrylcarnitine (C5)
−2.0
−1.0
0.0
1.0
0
24
SKOV3

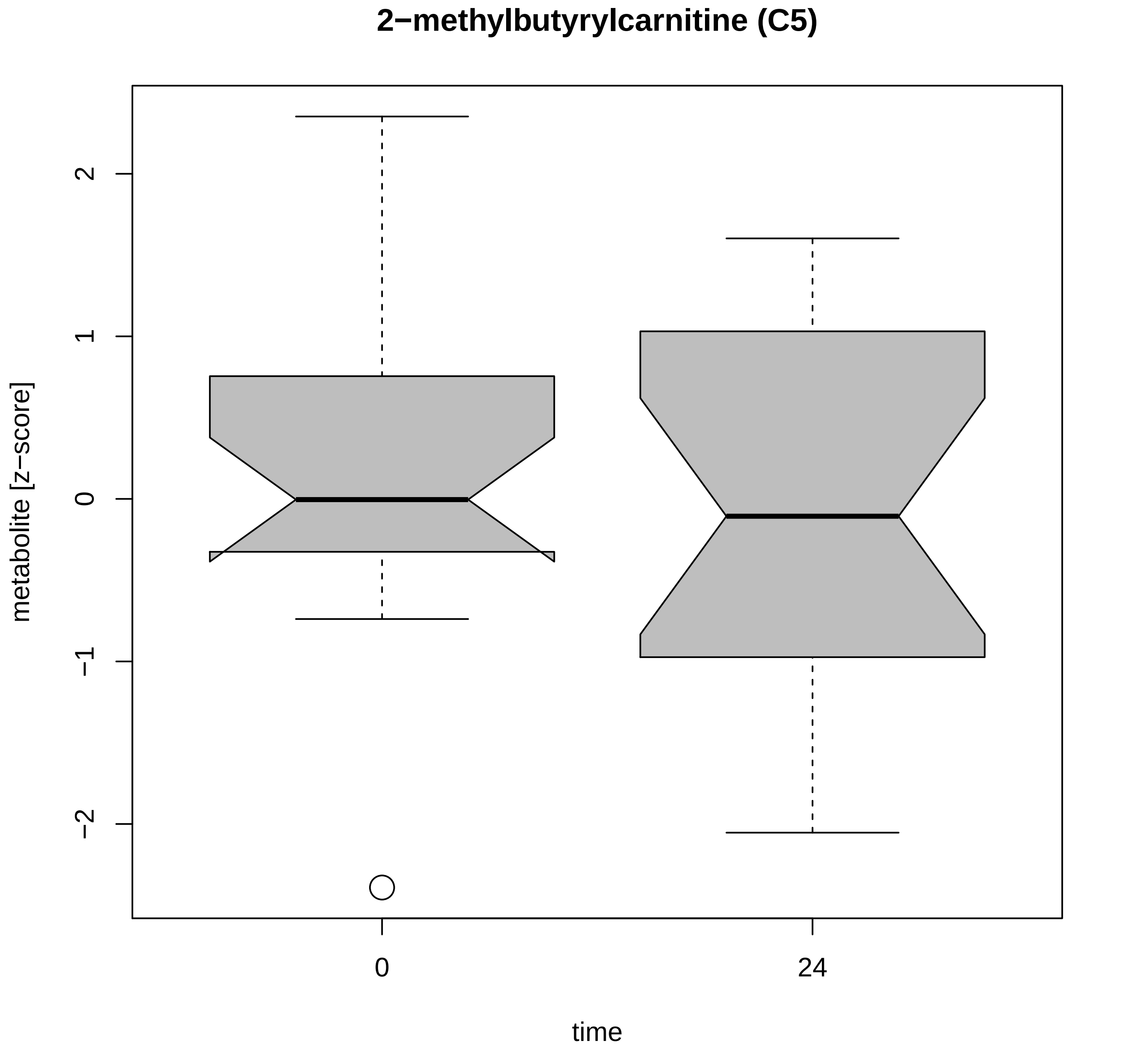

2−methylbutyrylcarnitine (C5)
metabolite [z−score]
2
1
0
−1
−2
0
24
time

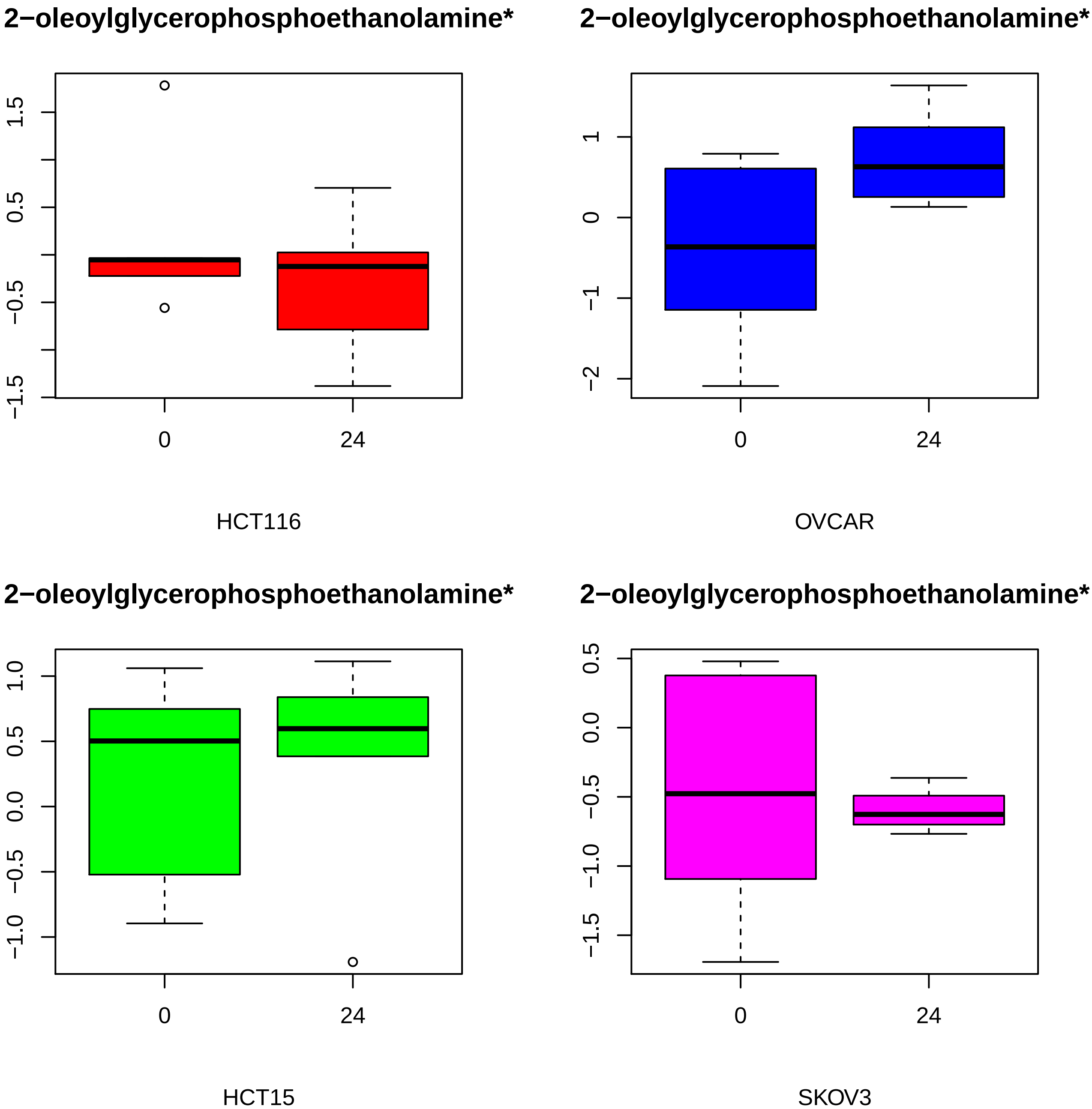
2−oleoylglycerophosphoethanolamine*
−1.5
−0.5
0.5
1.5
0
24
HCT116
2−oleoylglycerophosphoethanolamine*
−2
−1
0
1
0
24
OVCAR
2−oleoylglycerophosphoethanolamine*
−1.0
−0.5
0.0
0.5
1.0
0
24
HCT15
2−oleoylglycerophosphoethanolamine*
−1.5
−1.0
−0.5
0.0
0.5
0
24
SKOV3

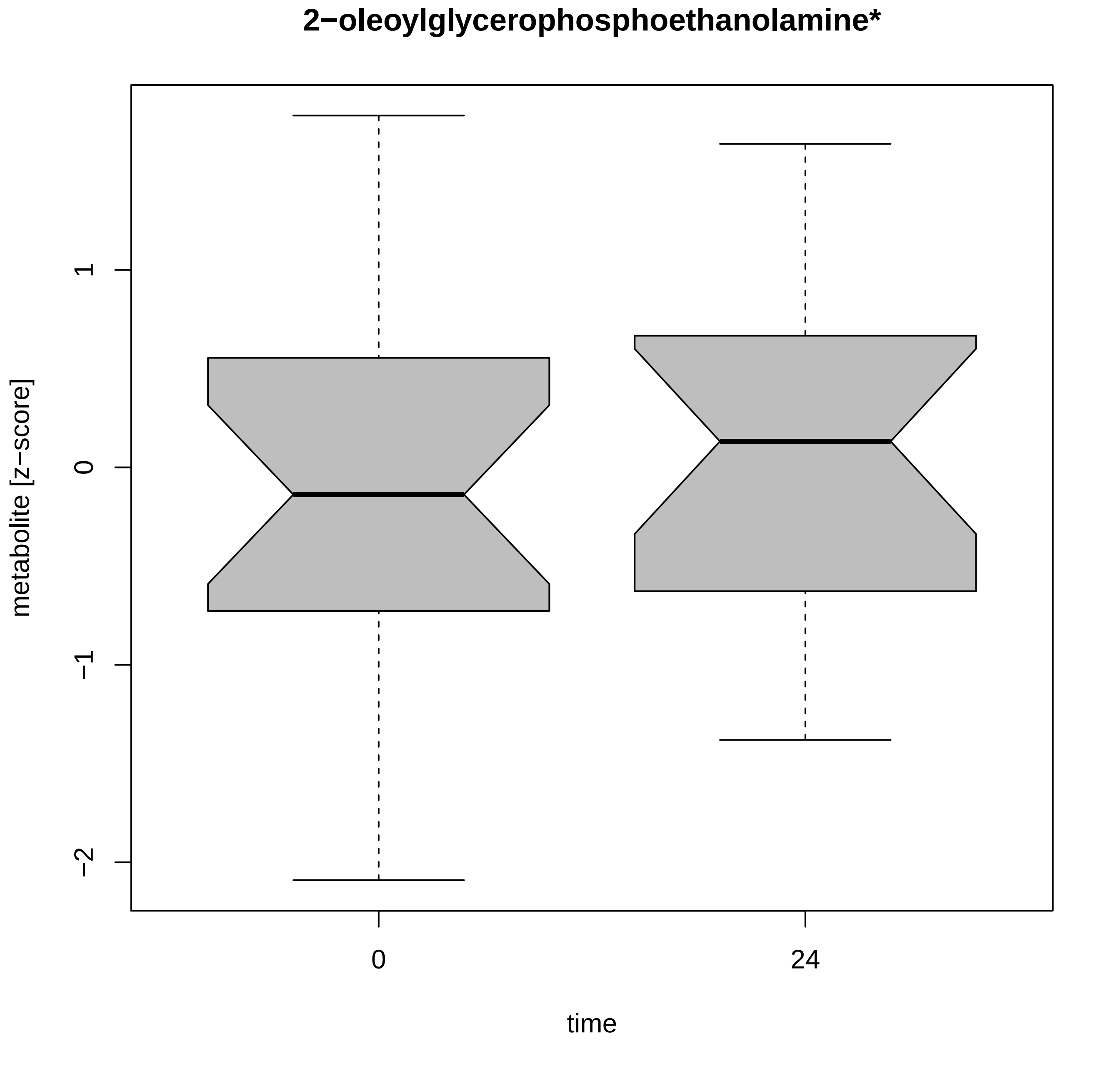

2−oleoylglycerophosphoethanolamine*
metabolite [z−score]
1
0
−1
−2
0
24
time

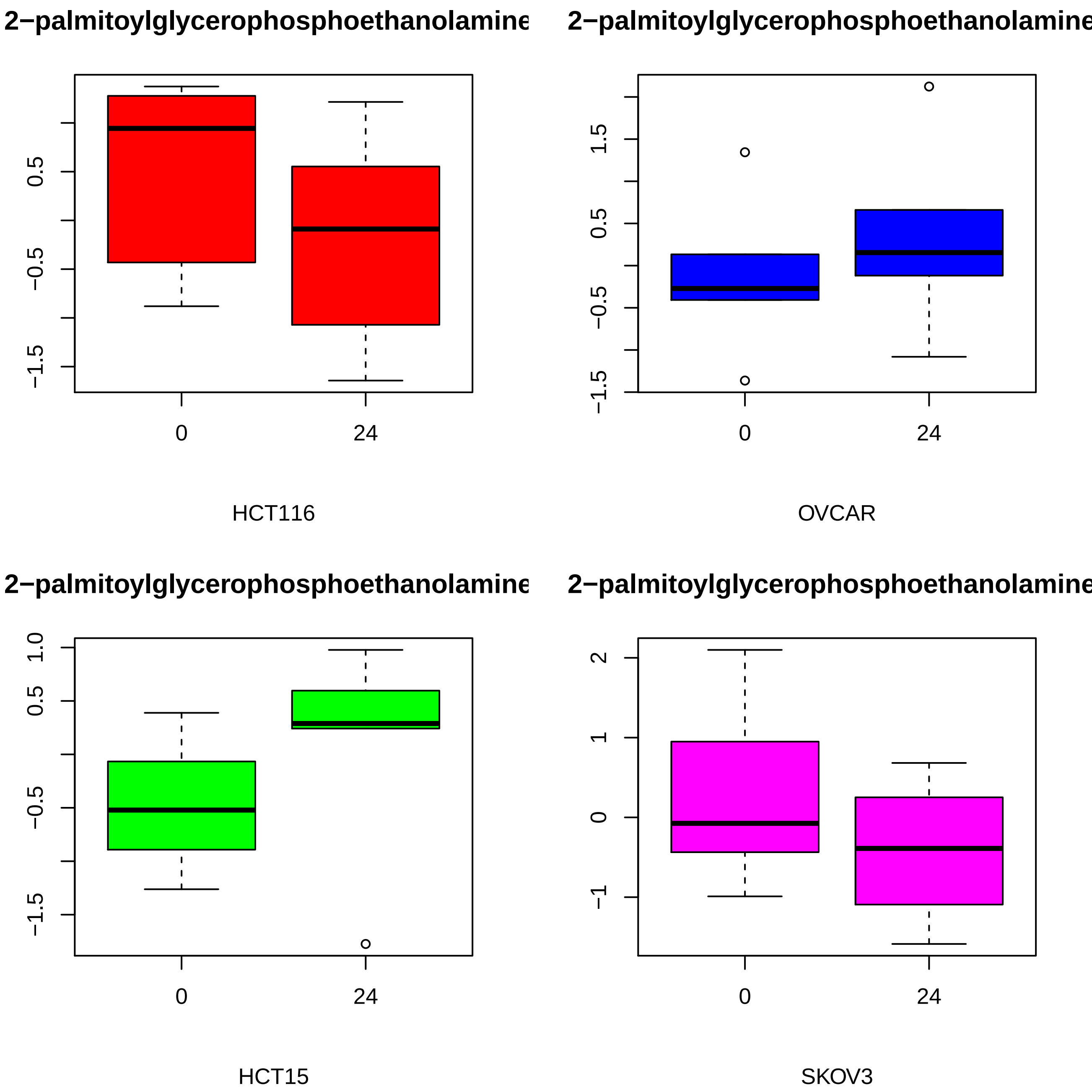

2−palmitoylglycerophosphoethanolamine
0.5
−0.5
−1.5
0
24
HCT116
2−palmitoylglycerophosphoethanolamine
1.5
0.5
−0.5
−1.5
0
24
OVCAR
2−palmitoylglycerophosphoethanolamine
1.0
0.5
−0.5
−1.5
0
24
HCT15
2−palmitoylglycerophosphoethanolamine
2
1
0
−1
0
24
SKOV3

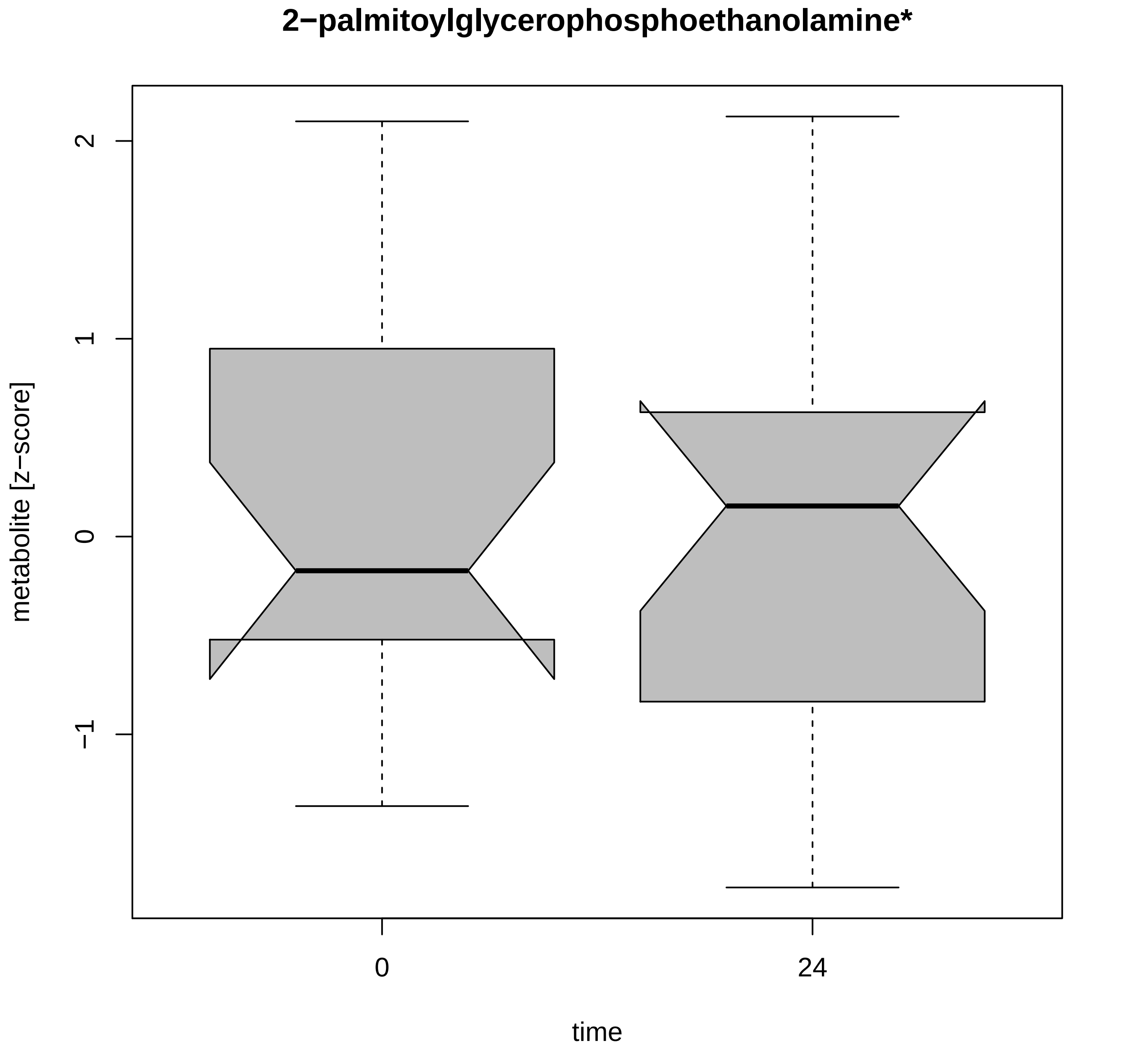

2−palmitoylglycerophosphoethanolamine*
metabolite [z−score]
2
1
0
−1
0
24
time

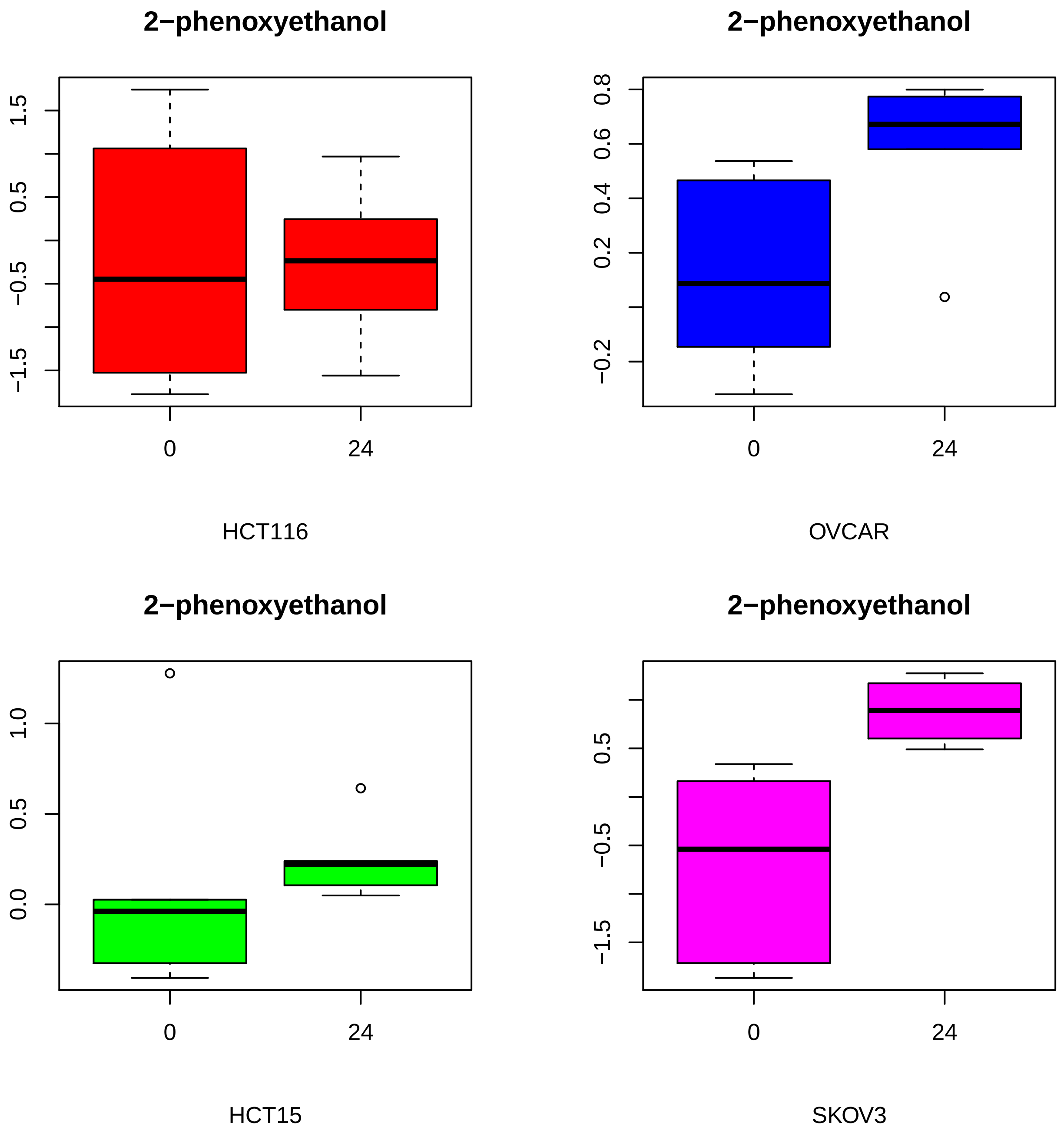
2−phenoxyethanol
1.5
0.5
−0.5
−1.5
0
24
HCT116
2−phenoxyethanol
0.8
0.6
0.4
0.2
−0.2
0
24
OVCAR
2−phenoxyethanol
1.0
0.5
0.0
0
24
HCT15
2−phenoxyethanol
0.5
−0.5
−1.5
0
24
SKOV3

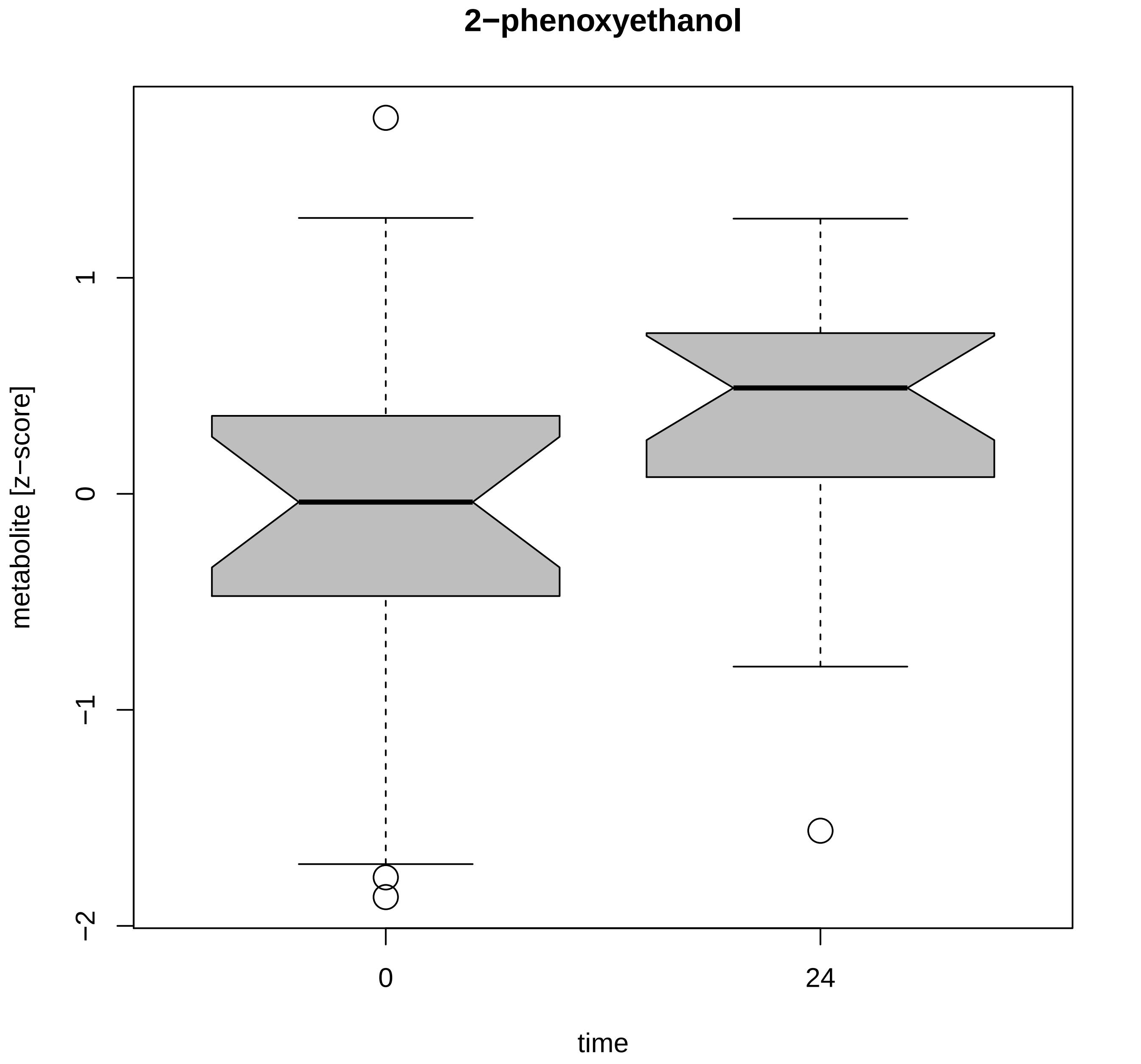
2−phenoxyethanol
metabolite [z−score]
1
0
−1
−2
0
24
time

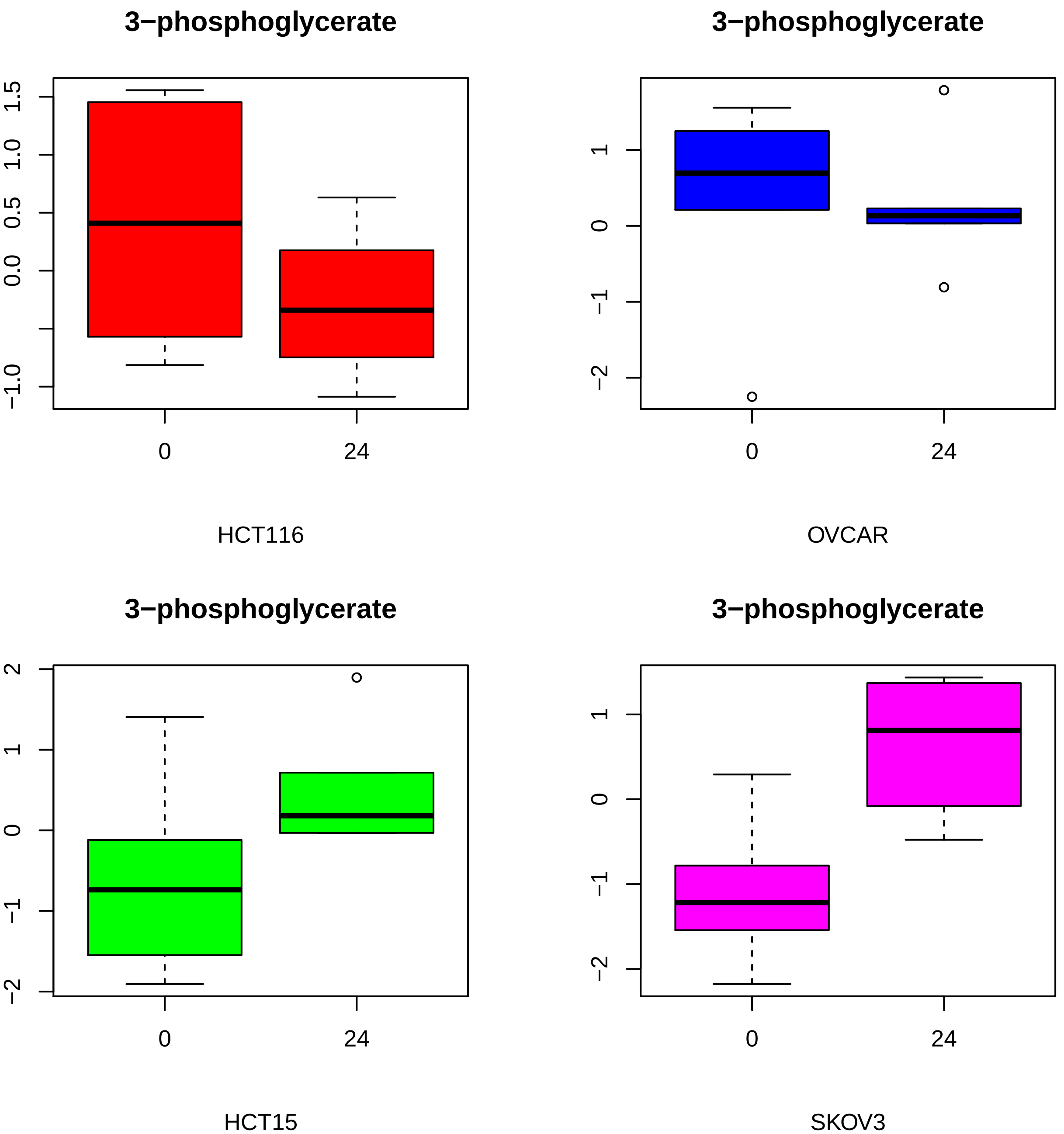
3−phosphoglycerate
1.5
1.0
0.5
0.0
−1.0
0
24
HCT116
3−phosphoglycerate
1
0
−1
−2
0
24
OVCAR
3−phosphoglycerate
2
1
0
−1
−2
0
24
HCT15
3−phosphoglycerate
1
0
−1
−2
0
24
SKOV3

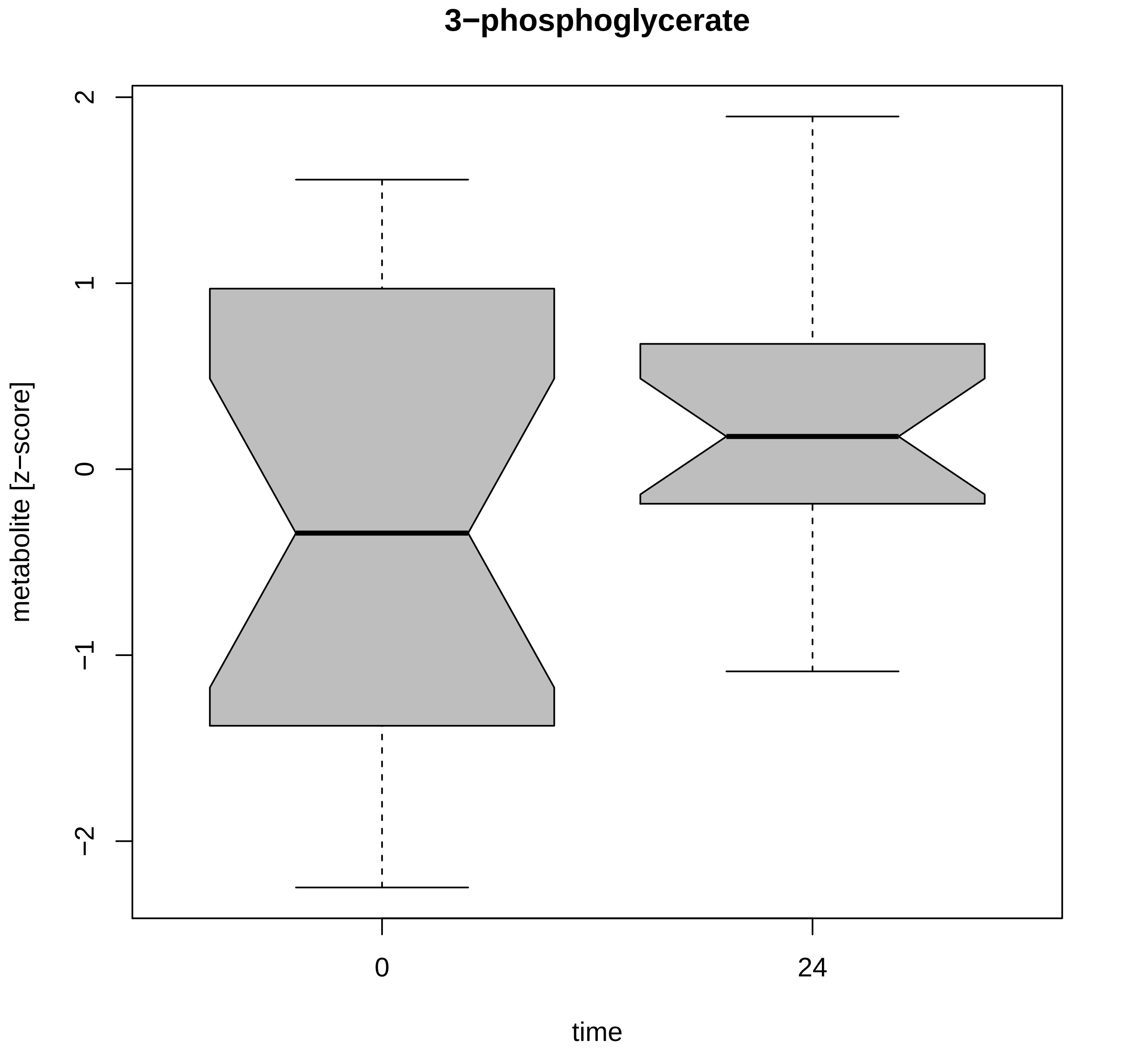
3−phosphoglycerate
metabolite [z−score]
2
1
0
−1
−2
0
24
time

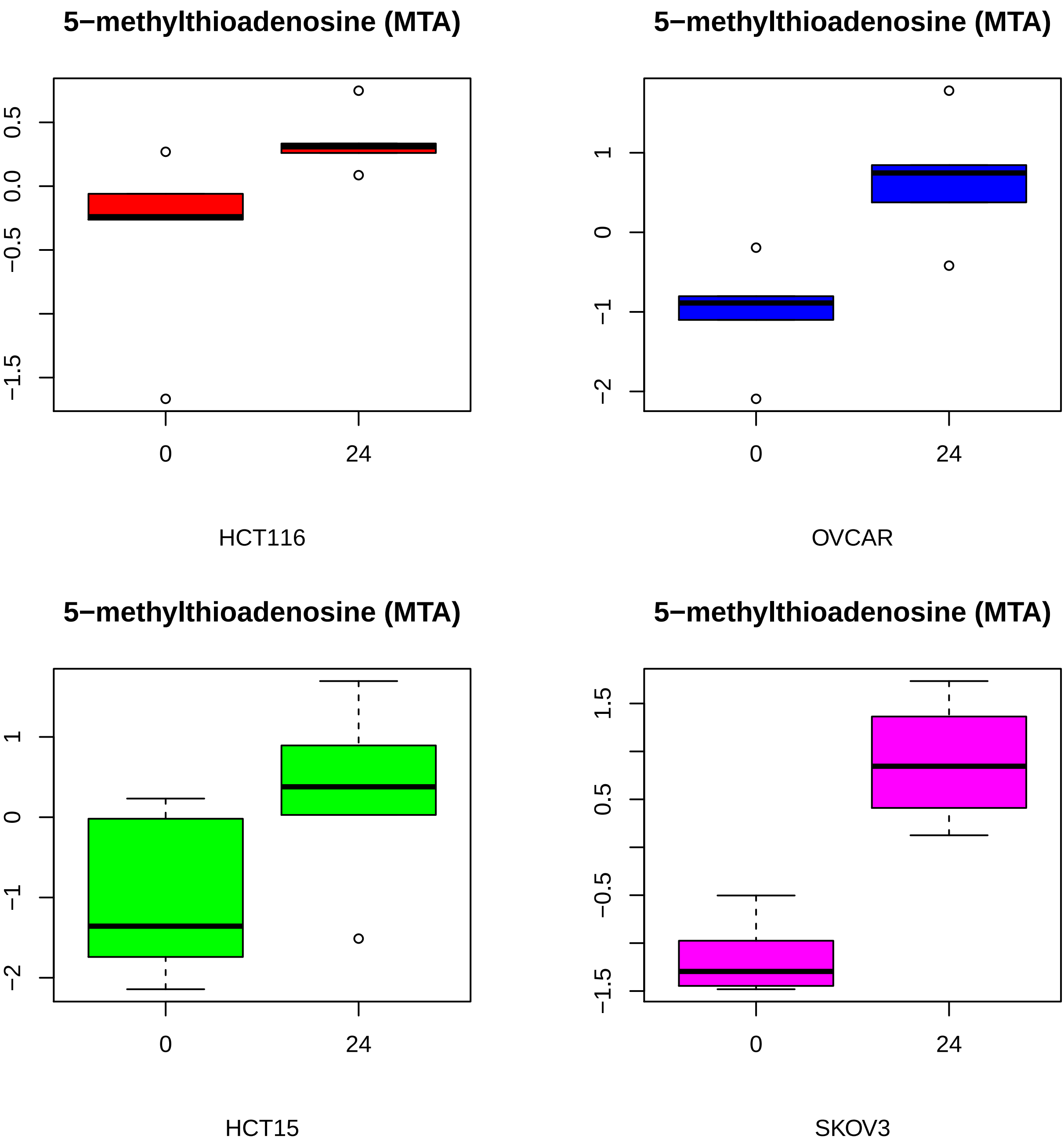
5−methylthioadenosine (MTA)
0.5
0.0
−0.5
−1.5
0
24
HCT116
5−methylthioadenosine (MTA)
1
0
−1
−2
0
24
OVCAR
5−methylthioadenosine (MTA)
1
0
−1
−2
0
24
HCT15
5−methylthioadenosine (MTA)
1.5
0.5
−0.5
−1.5
0
24
SKOV3

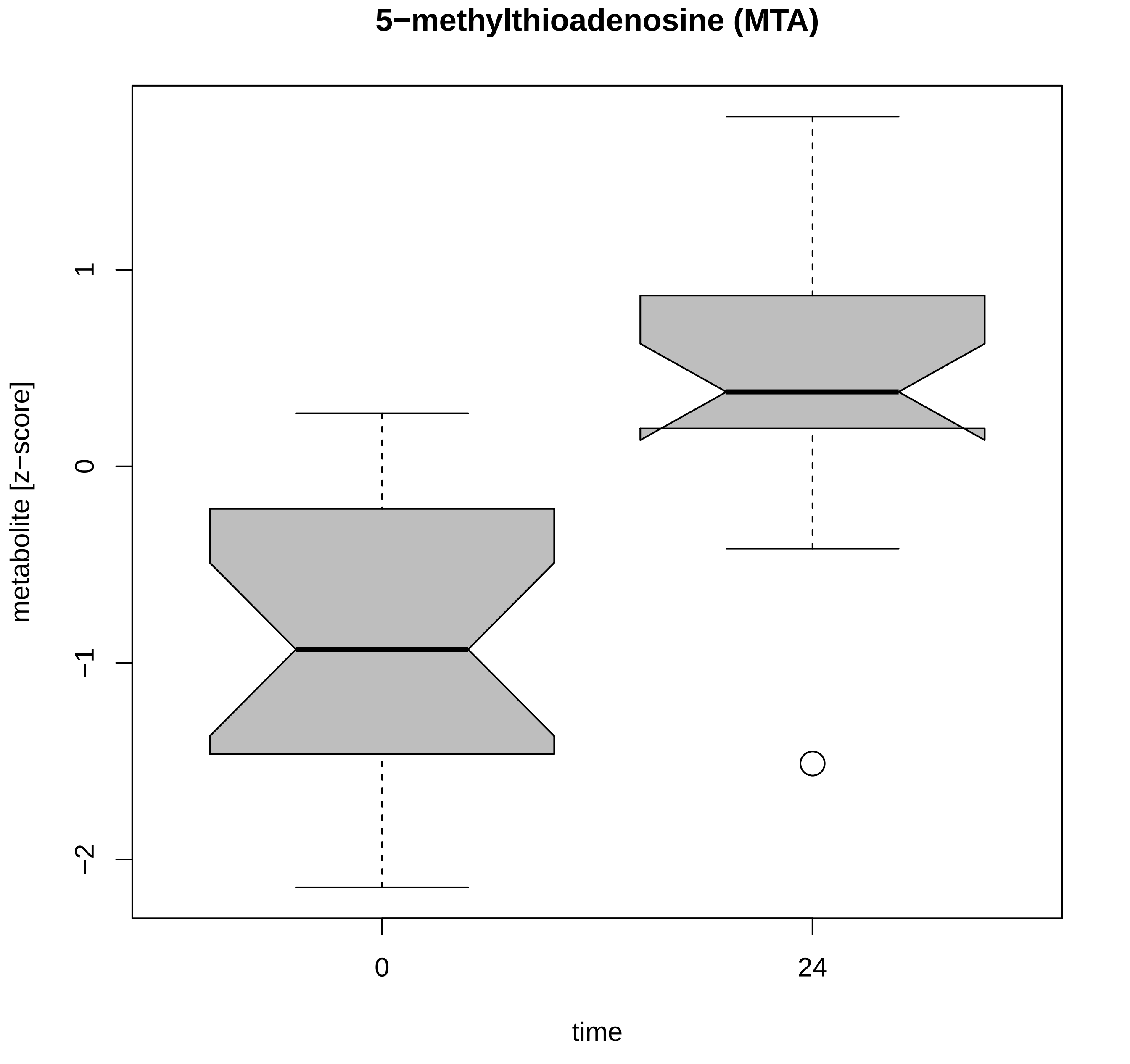

5−methylthioadenosine (MTA)
metabolite [z−score]
−2
−1
0
1
0
24
time

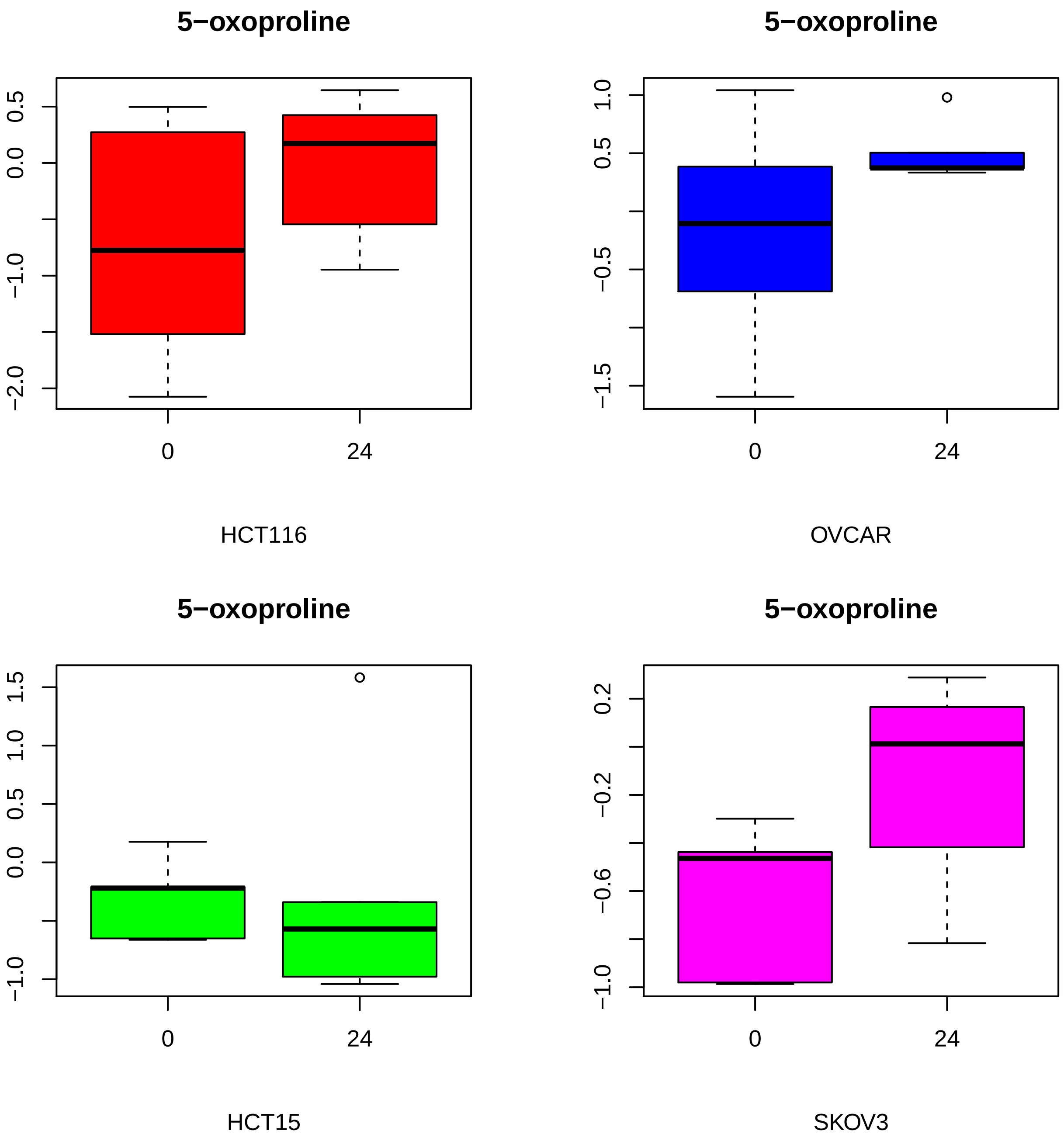

5−oxoproline
0.5
0.0
−1.0
−2.0
0
24
HCT116
5−oxoproline
1.0
0.5
−0.5
−1.5
0
24
OVCAR
5−oxoproline
1.5
1.0
0.5
0.0
−1.0
0
24
HCT15
5−oxoproline
0.2
−0.2
−0.6
−1.0
0
24
SKOV3

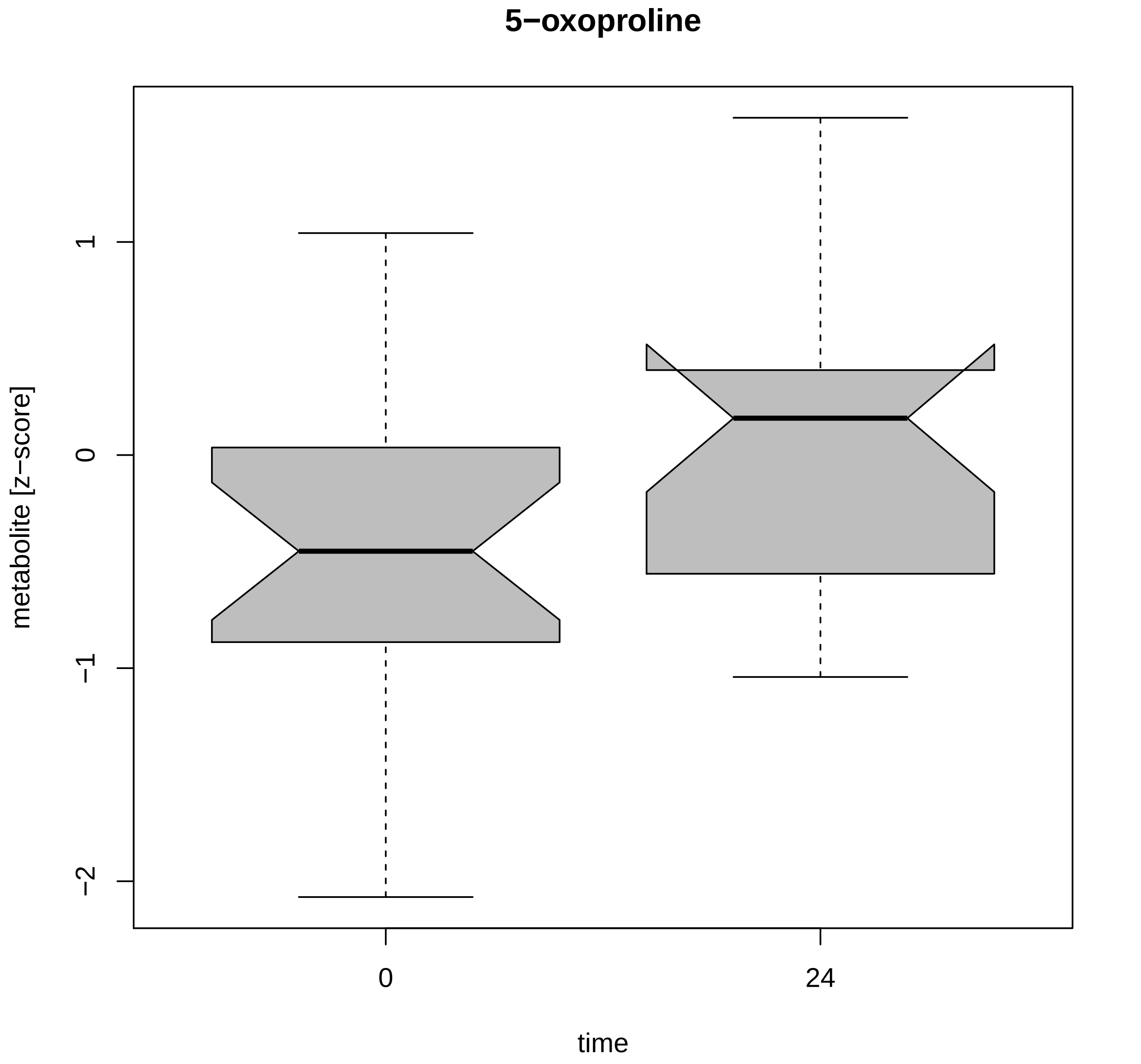
5−oxoproline
metabolite [z−score]
1
0
−1
−2
0
24
time

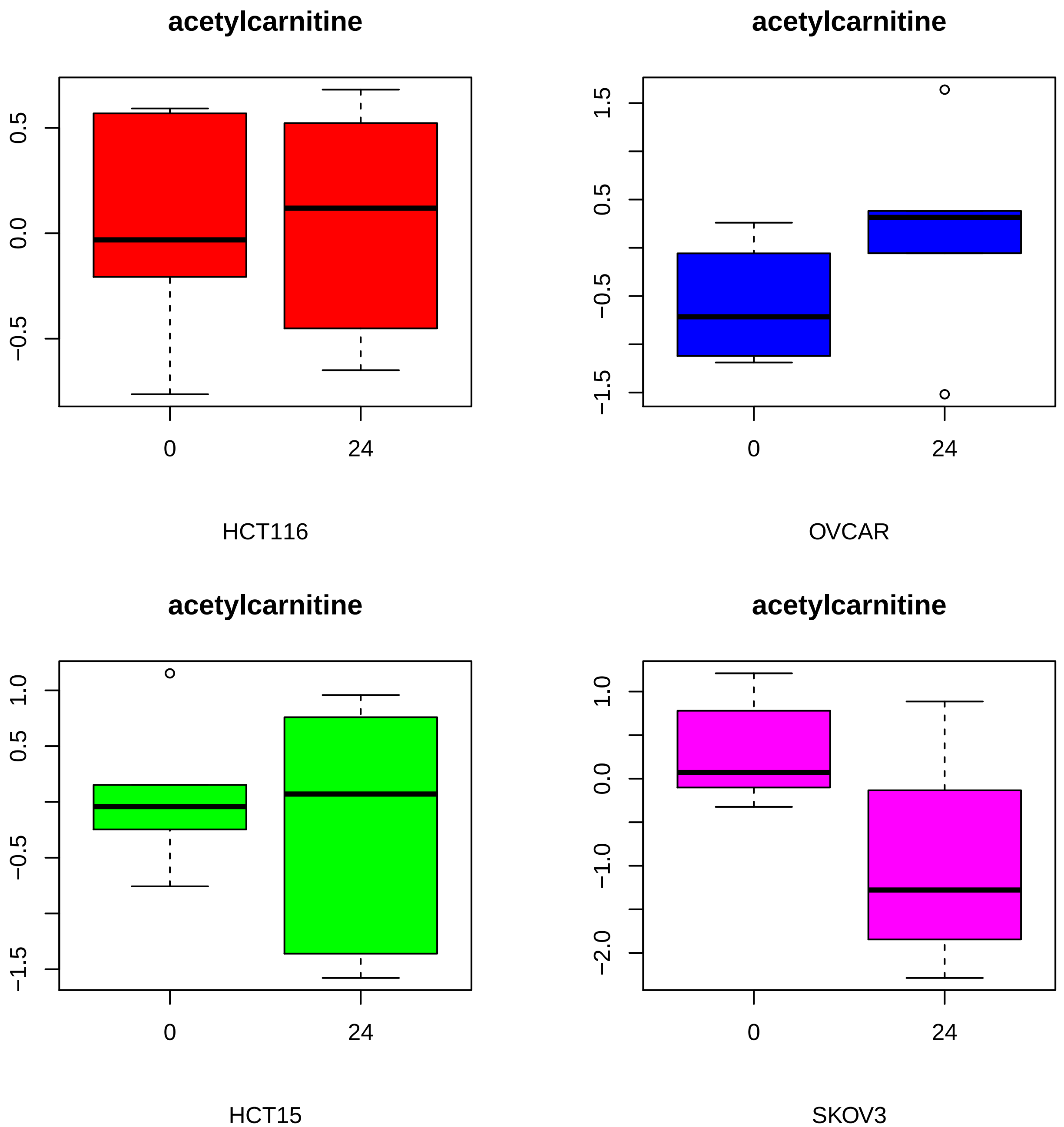
acetylcarnitine
0.5
0.0
−0.5
0
24
HCT116
acetylcarnitine
1.5
0.5
−0.5
−1.5
0
24
OVCAR
acetylcarnitine
1.0
0.5
−0.5
−1.5
0
24
HCT15
acetylcarnitine
1.0
0.0
−1.0
−2.0
0
24
SKOV3

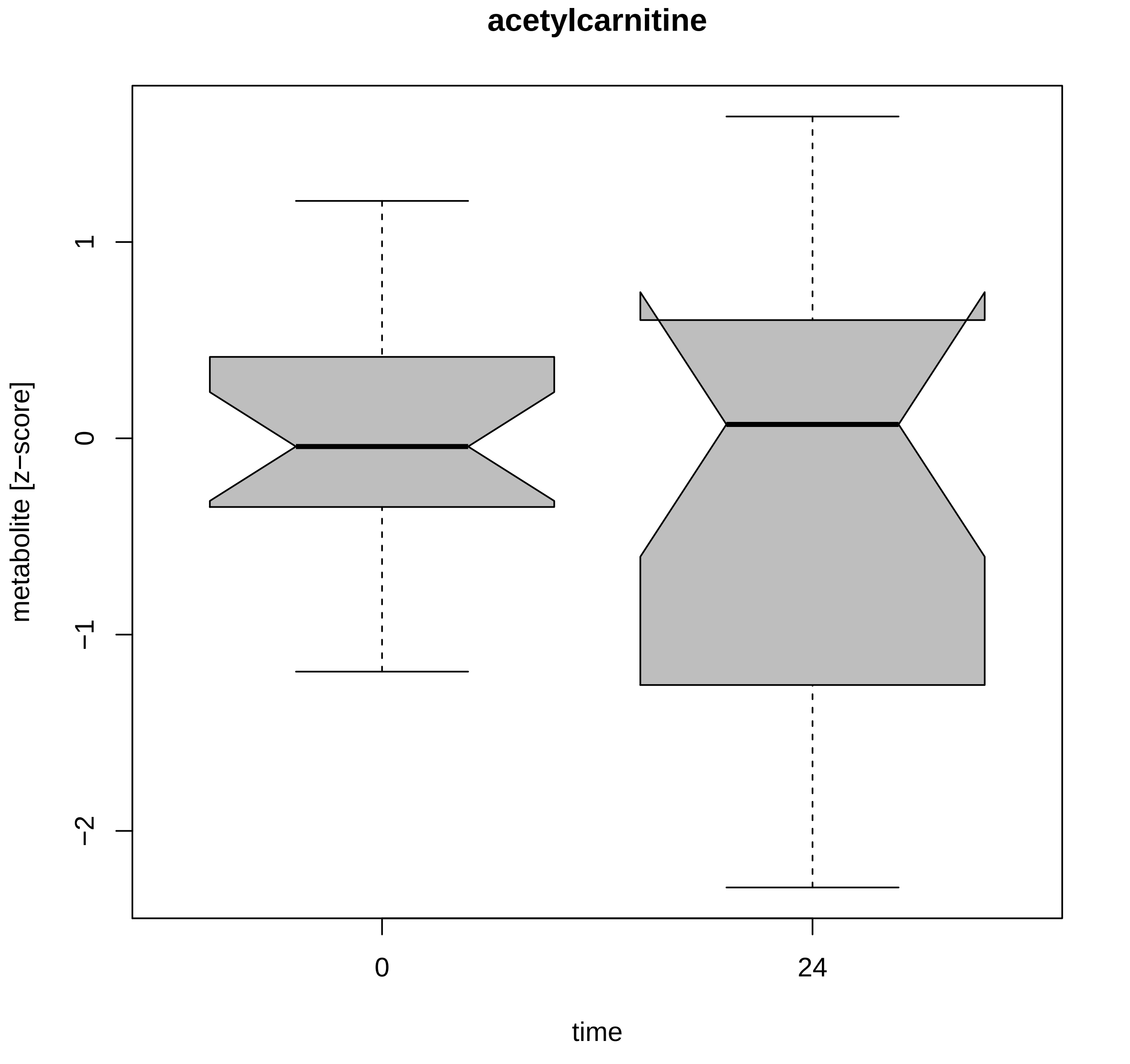
acetylcarnitine
metabolite [z−score]
1
0
−1
−2
0
24
time

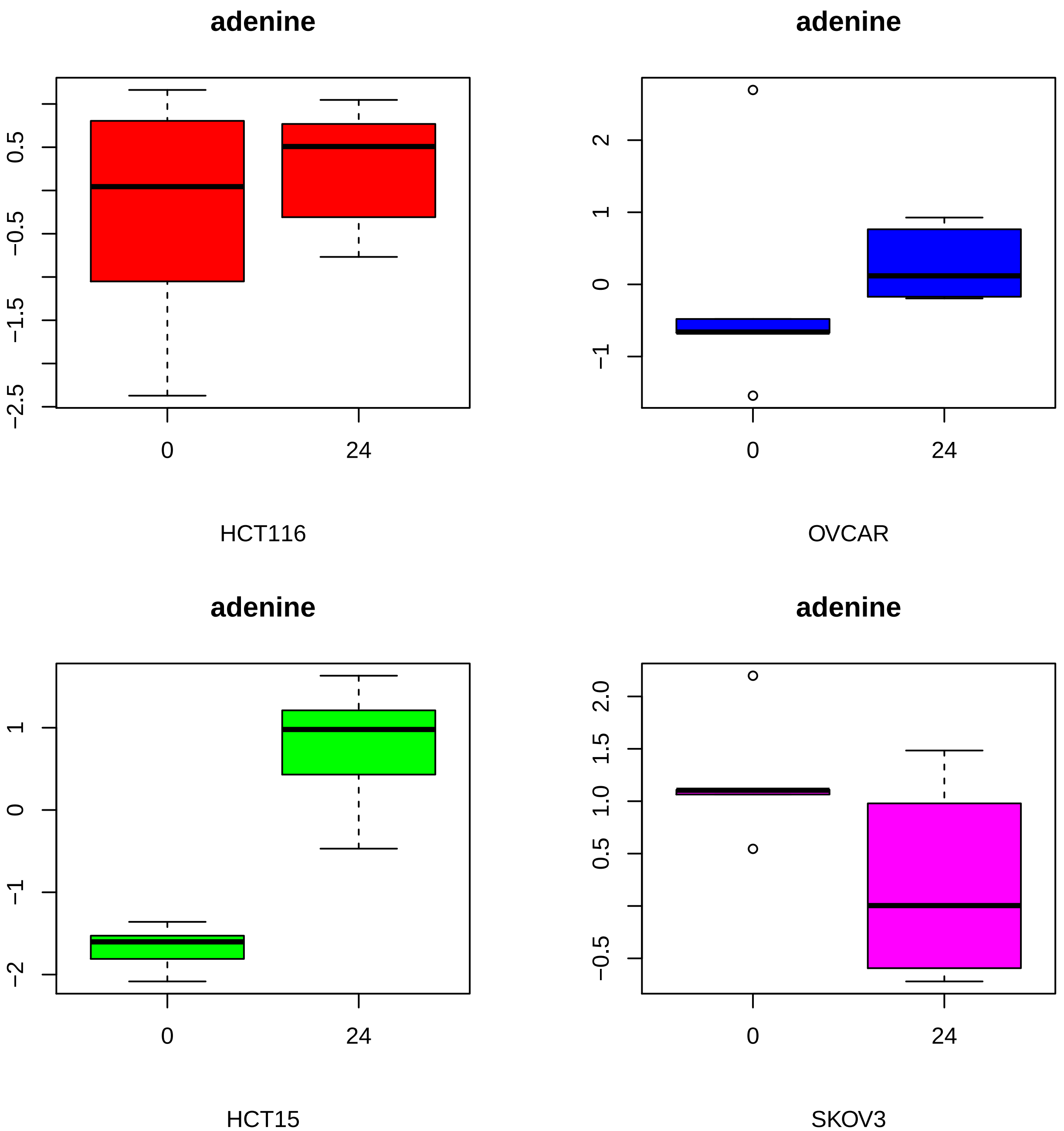
adenine
adenine
adenine
adenine
0
24
HCT116
OVCAR
HCT15
SKOV3
−2.5
−1.5
−0.5
0.5
−1
0
1
2
−2
−0.5
0.5
1.0
1.5
2.0

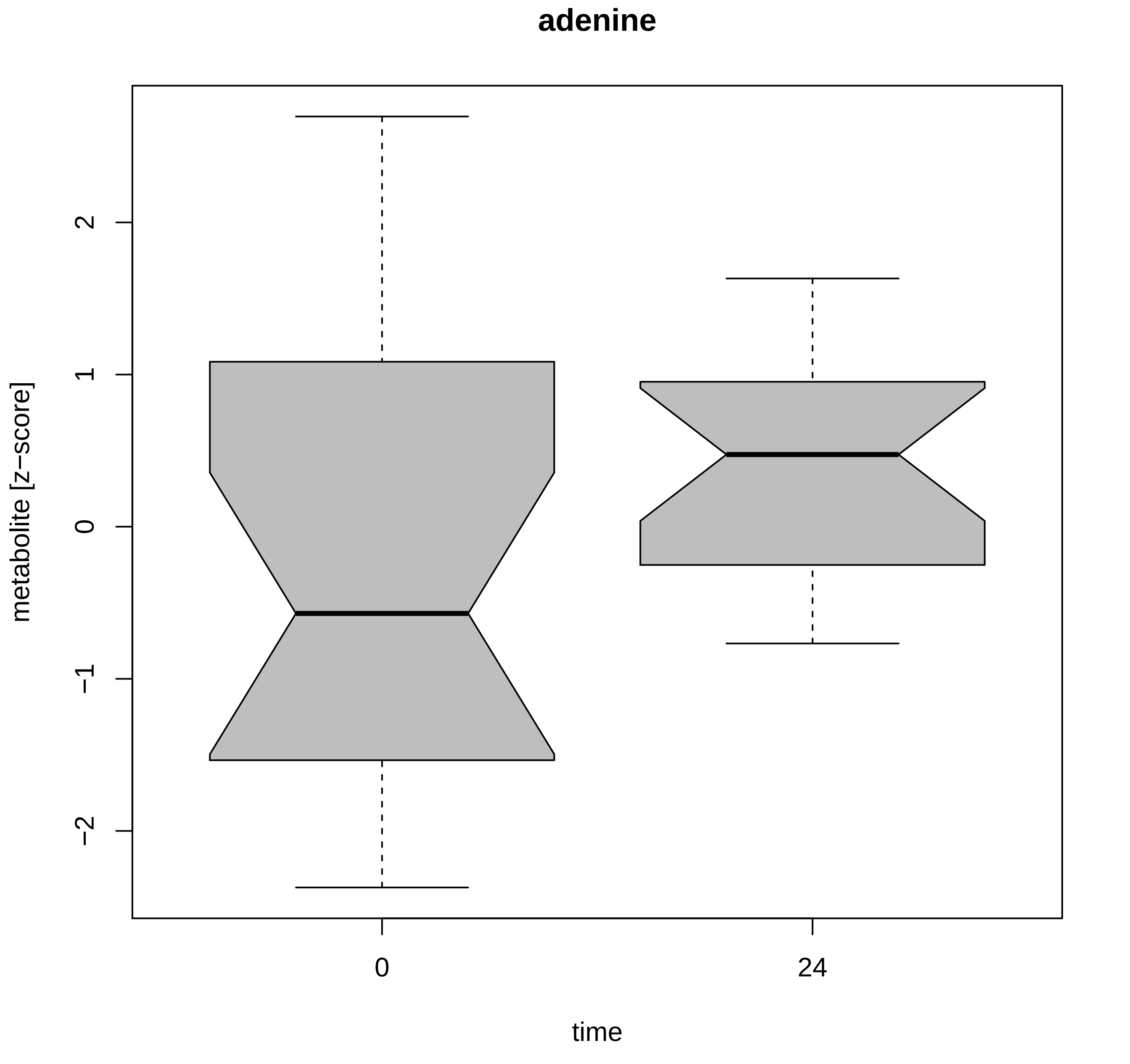
adenine
metabolite [z−score]
2
1
0
−1
−2
0
24
time

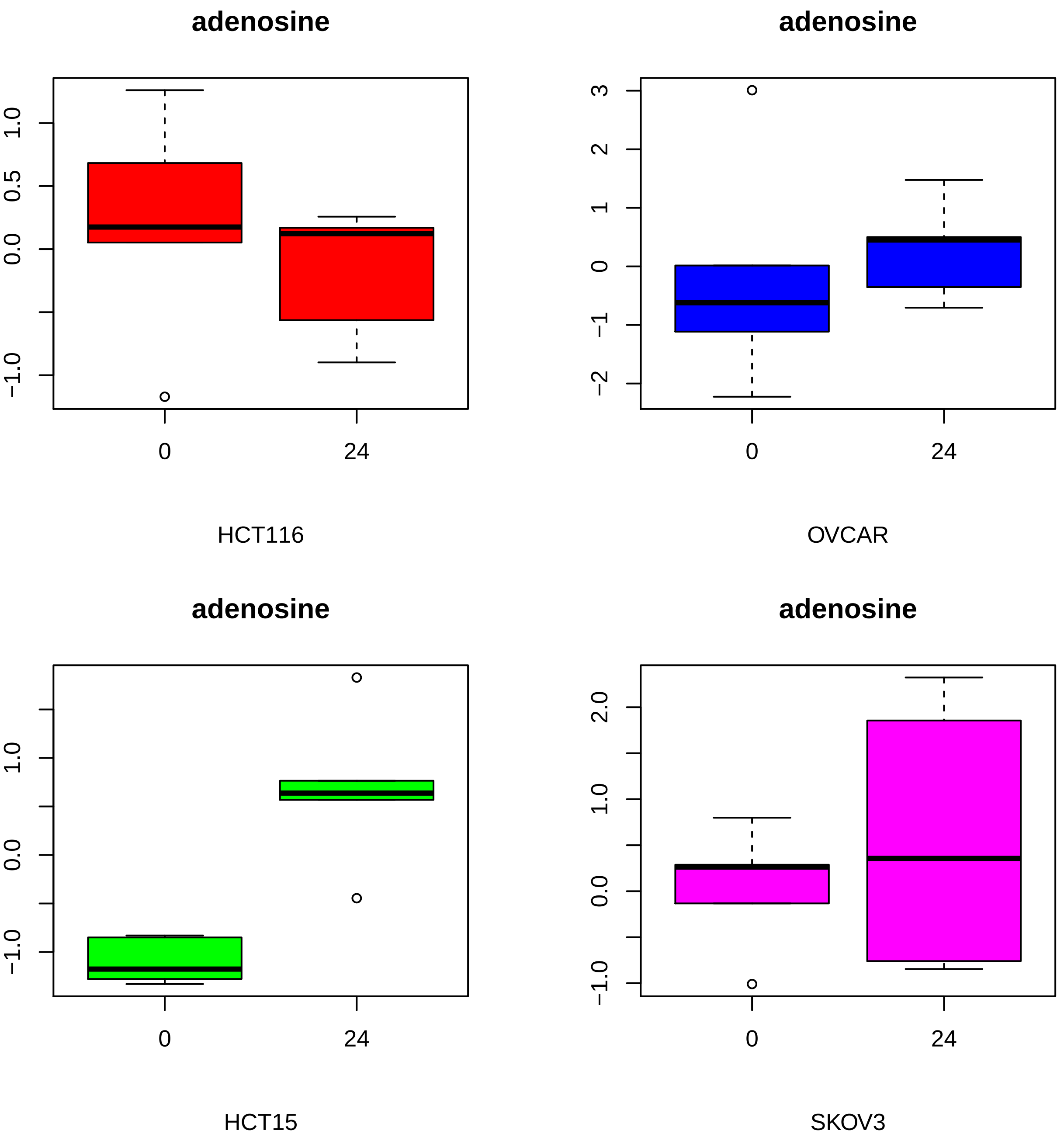

adenosine
1.0
0.5
0.0
−1.0
0
24
HCT116
adenosine
3
2
1
0
−1
−2
0
24
OVCAR
adenosine
1.0
0.0
−1.0
0
24
HCT15
adenosine
2.0
1.0
0.0
−1.0
0
24
SKOV3

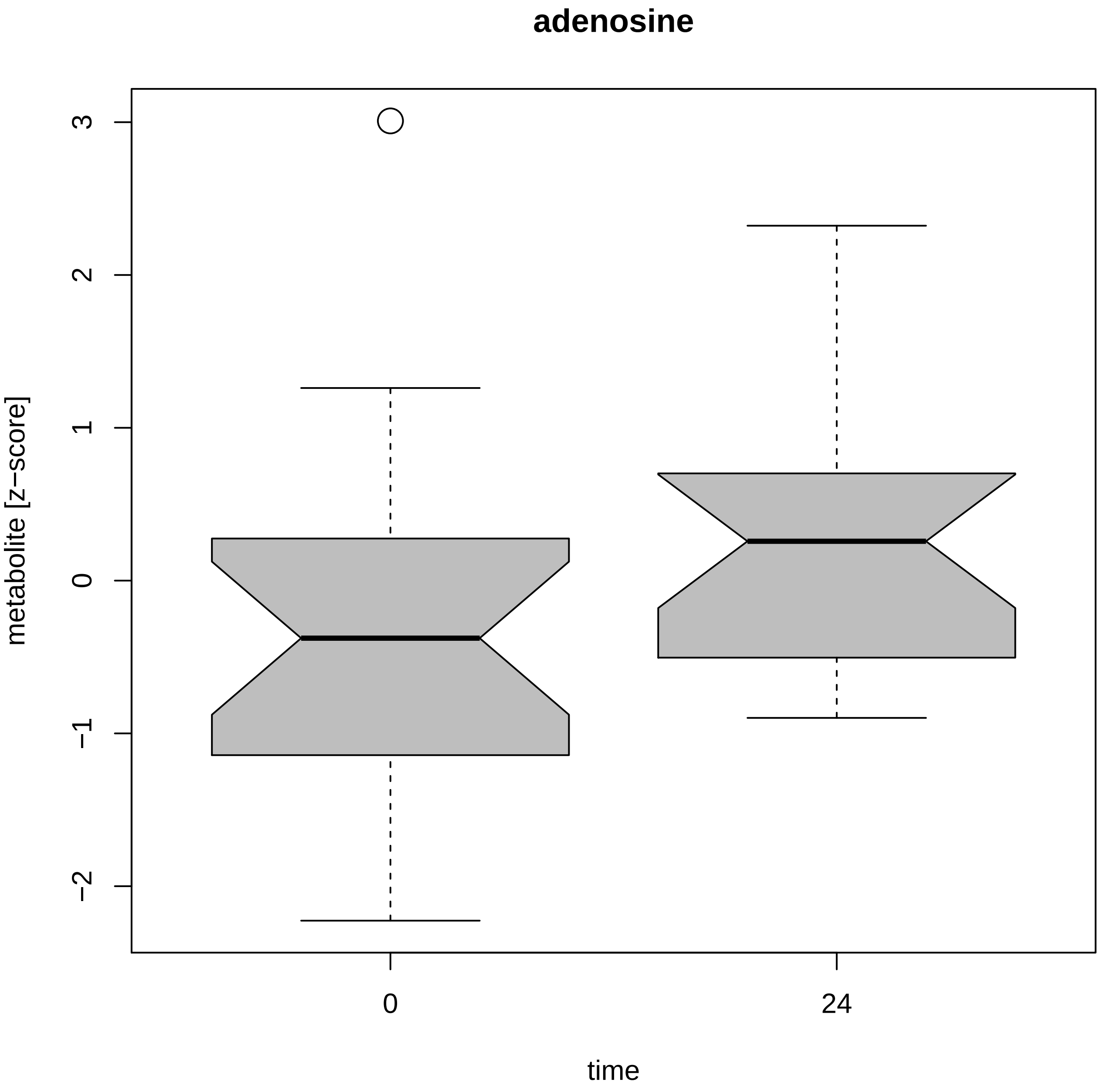
adenosine
metabolite [z−score]
3
2
1
0
−1
−2
0
24
time

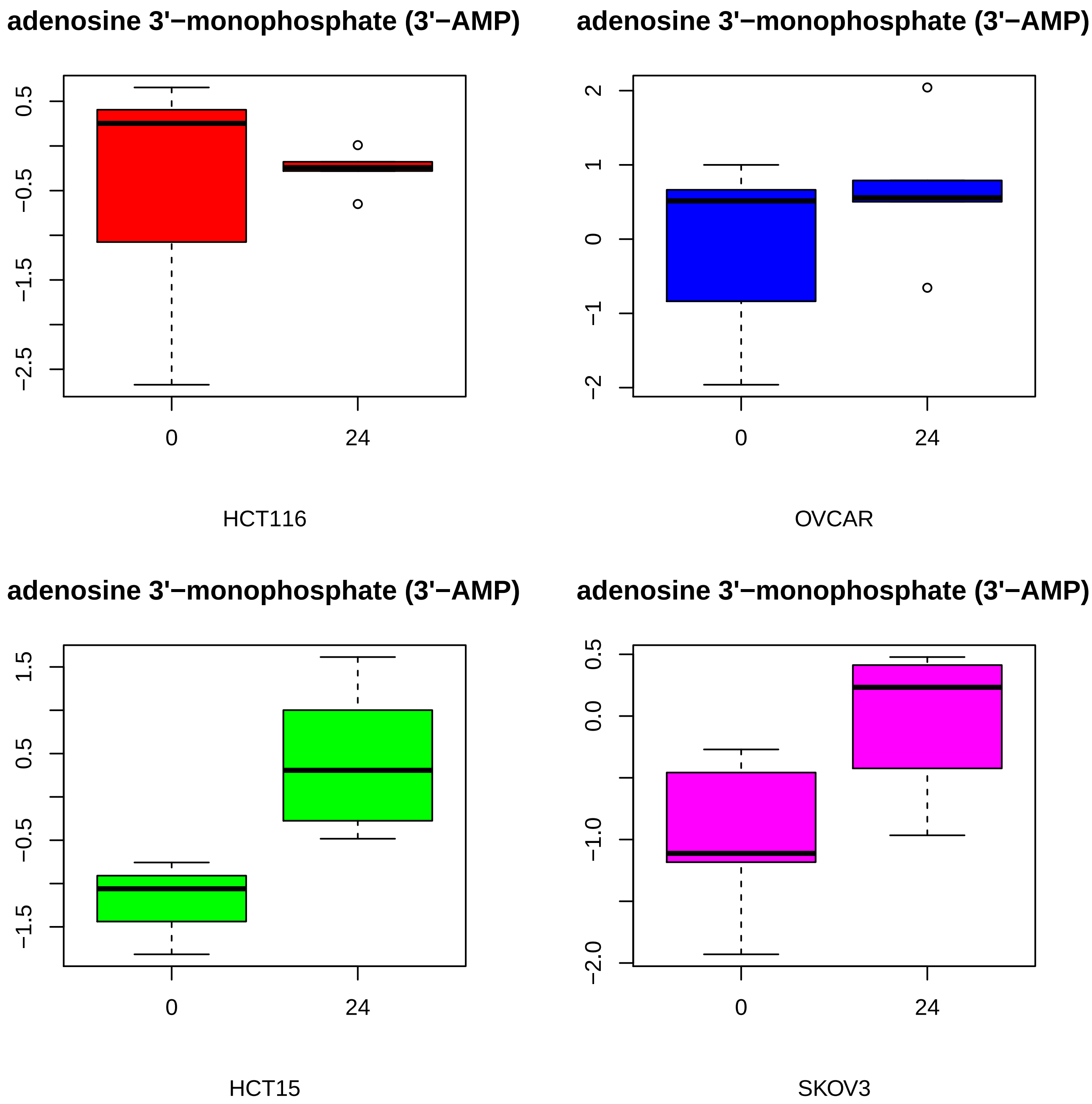

adenosine 3'−monophosphate (3'−AMP)
0.5
−0.5
−1.5
−2.5
0
24
HCT116
adenosine 3'−monophosphate (3'−AMP)
2
1
0
−1
−2
0
24
OVCAR
adenosine 3'−monophosphate (3'−AMP)
1.5
0.5
−0.5
−1.5
0
24
HCT15
adenosine 3'−monophosphate (3'−AMP)
0.5
0.0
−1.0
−2.0
0
24
SKOV3

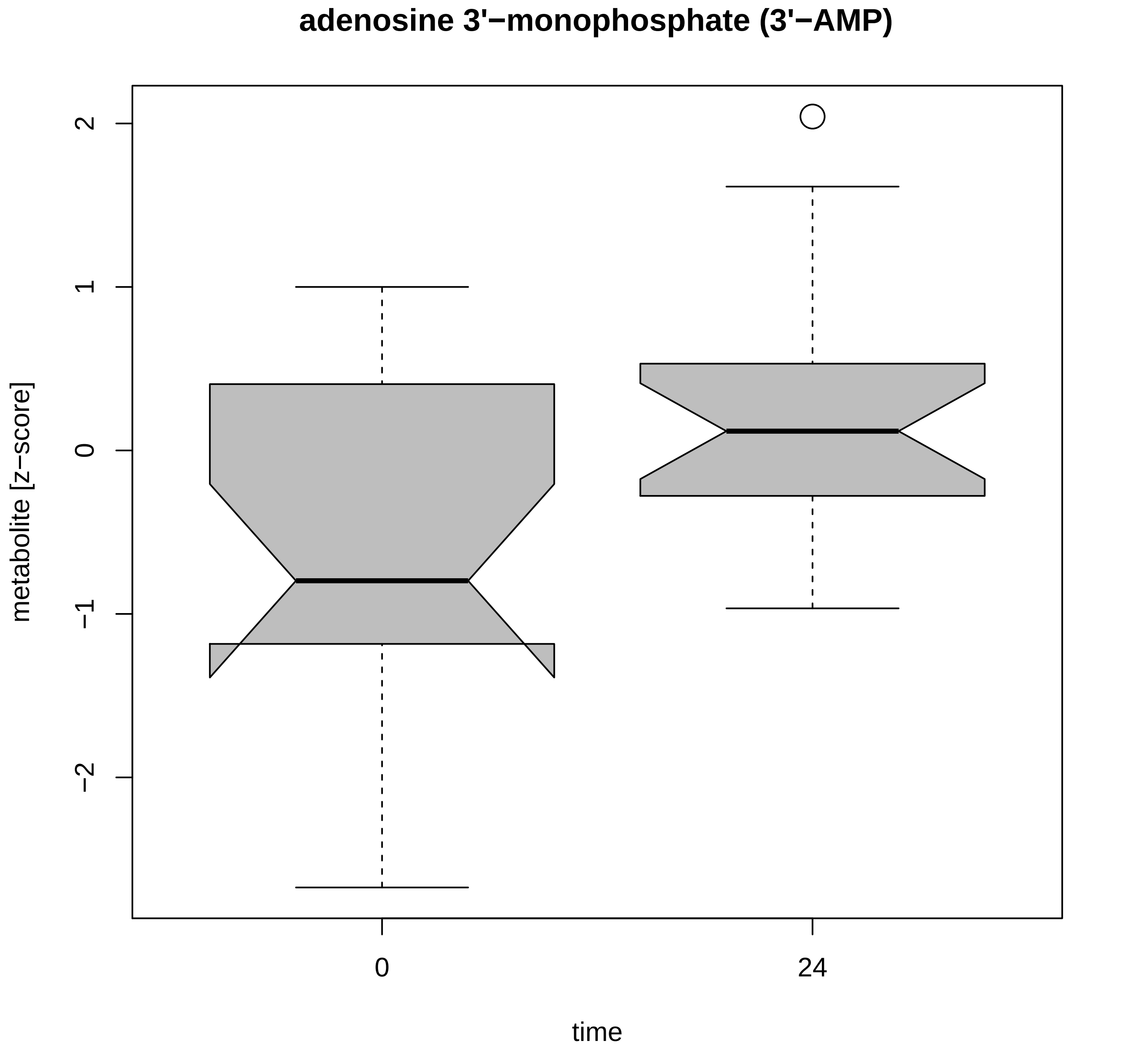

adenosine 3'−monophosphate (3'−AMP)
metabolite [z−score]
2
1
0
−1
−2
0
24
time

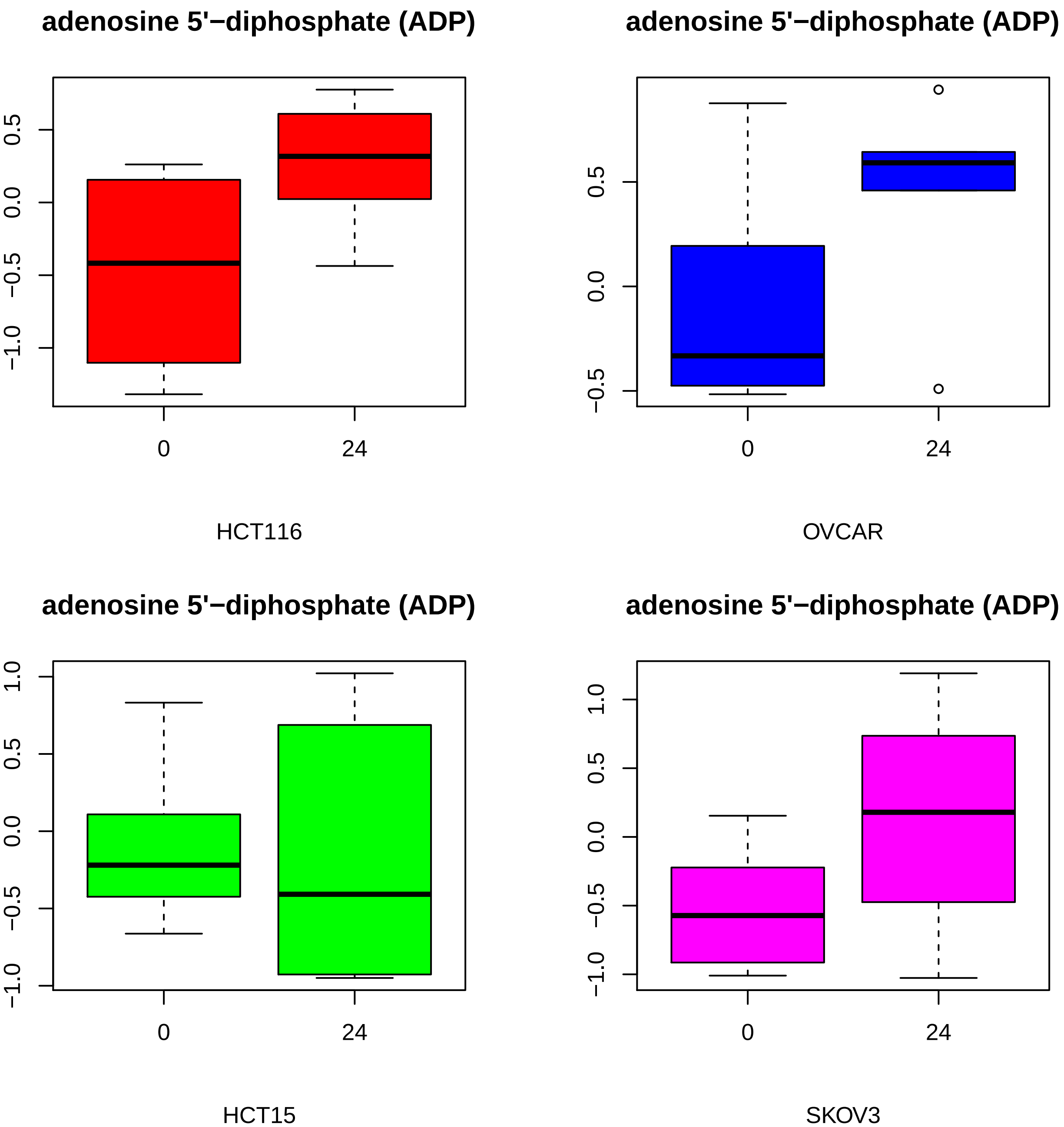
adenosine 5'−diphosphate (ADP)
0.5
0.0
−0.5
−1.0
0
24
HCT116
adenosine 5'−diphosphate (ADP)
0.5
0.0
−0.5
0
24
OVCAR
adenosine 5'−diphosphate (ADP)
1.0
0.5
0.0
−0.5
−1.0
0
24
HCT15
adenosine 5'−diphosphate (ADP)
1.0
0.5
0.0
−0.5
−1.0
0
24
SKOV3

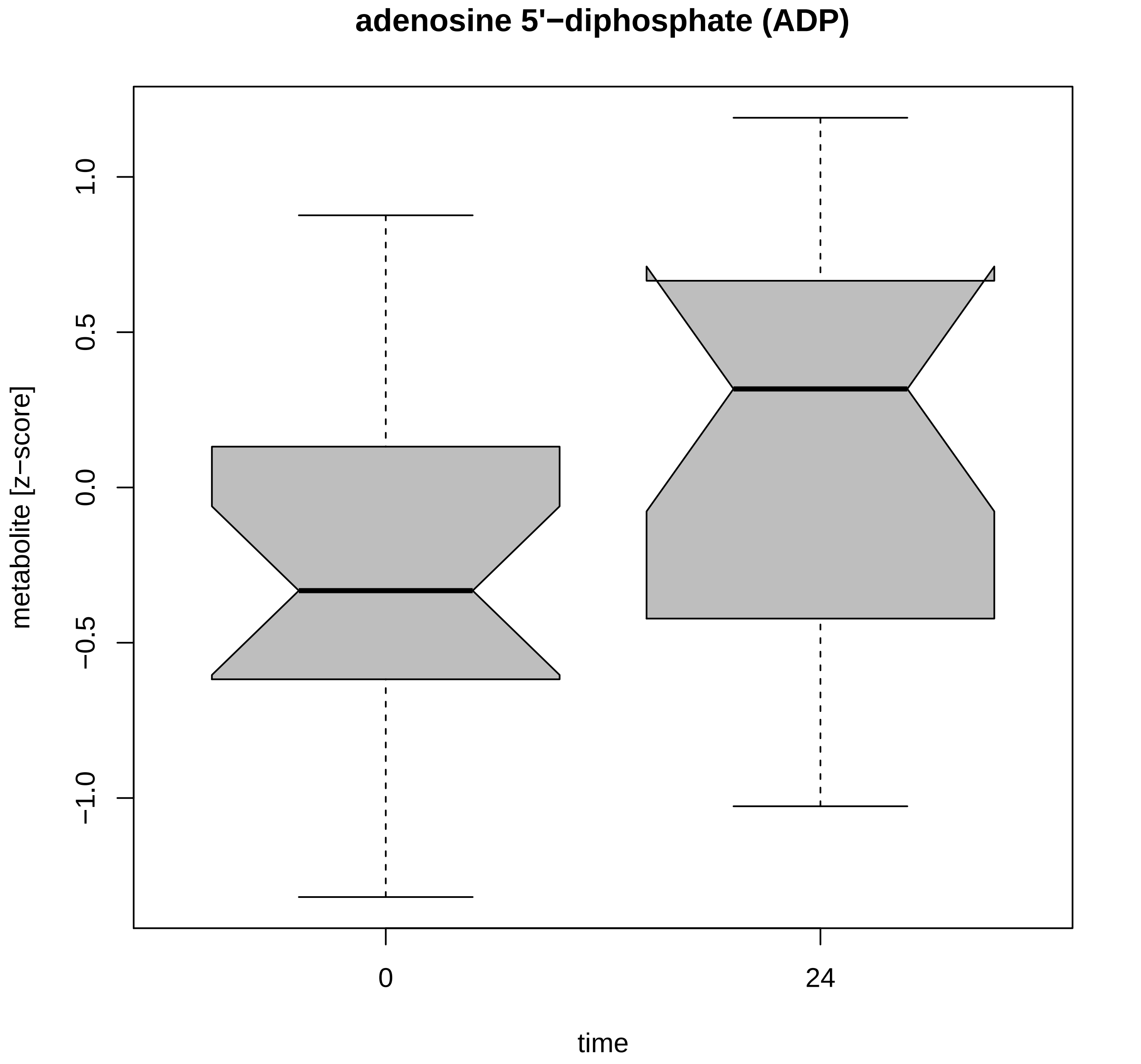

adenosine 5'−diphosphate (ADP)
metabolite [z−score]
1.0
0.5
0.0
−0.5
−1.0
0
24
time

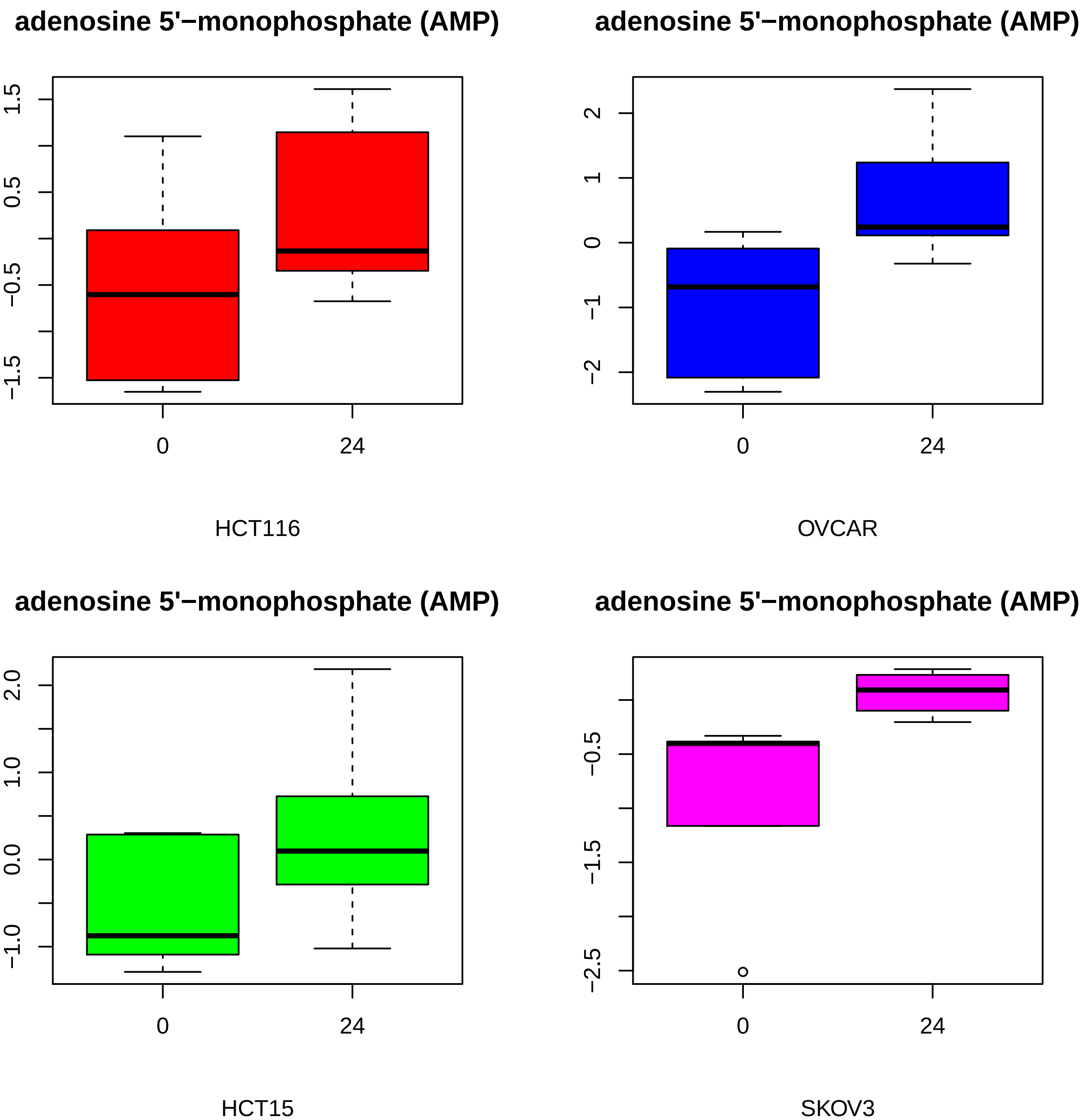
adenosine 5'−monophosphate (AMP)
1.5
0.5
−0.5
−1.5
0
24
HCT116
adenosine 5'−monophosphate (AMP)
2
1
0
−1
−2
0
24
OVCAR
adenosine 5'−monophosphate (AMP)
2.0
1.0
0.0
−1.0
0
24
HCT15
adenosine 5'−monophosphate (AMP)
−0.5
−1.5
−2.5
0
24
SKOV3

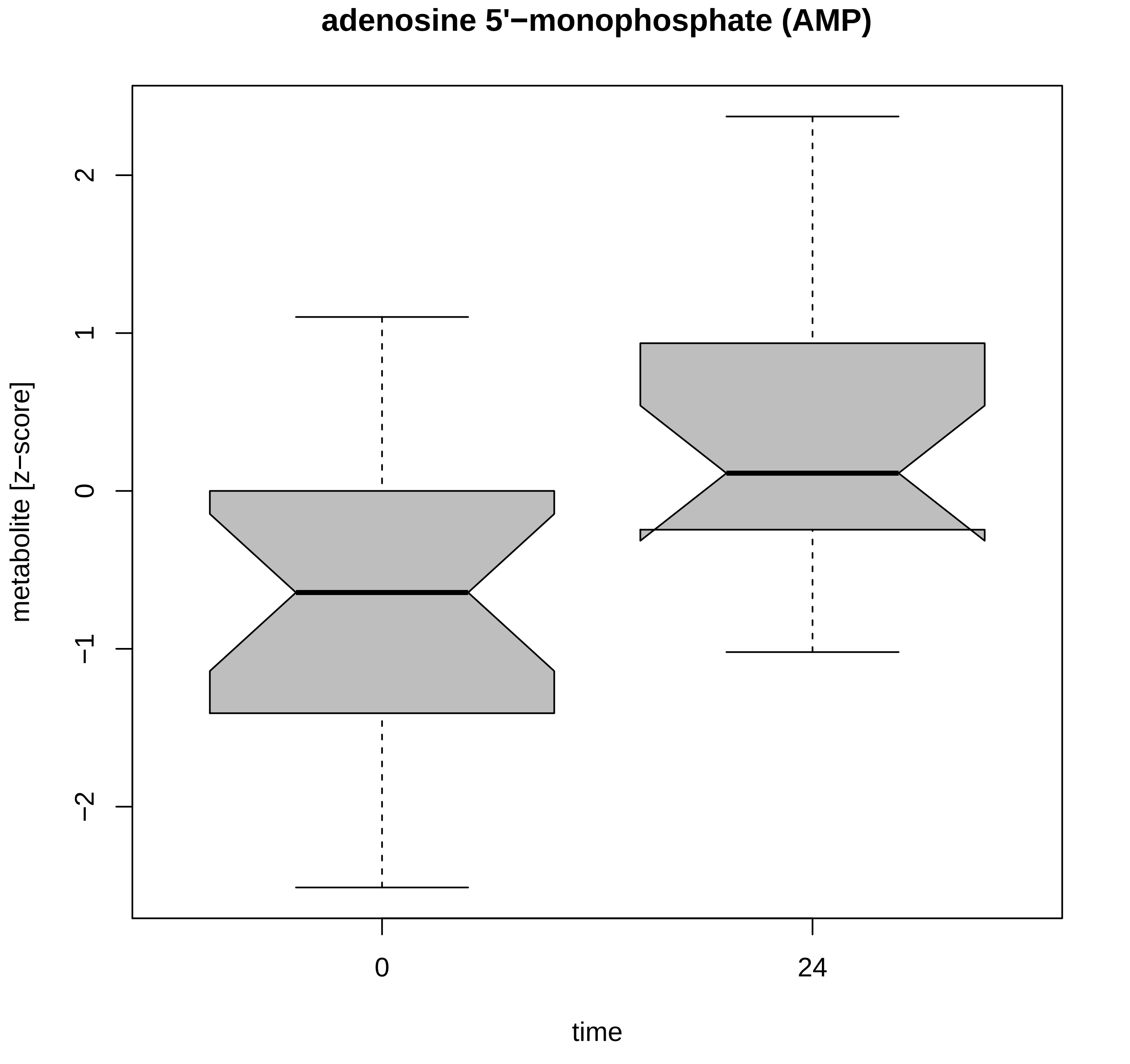
adenosine 5'−monophosphate (AMP)
metabolite [z−score]
2
1
0
−1
−2
0
24
time

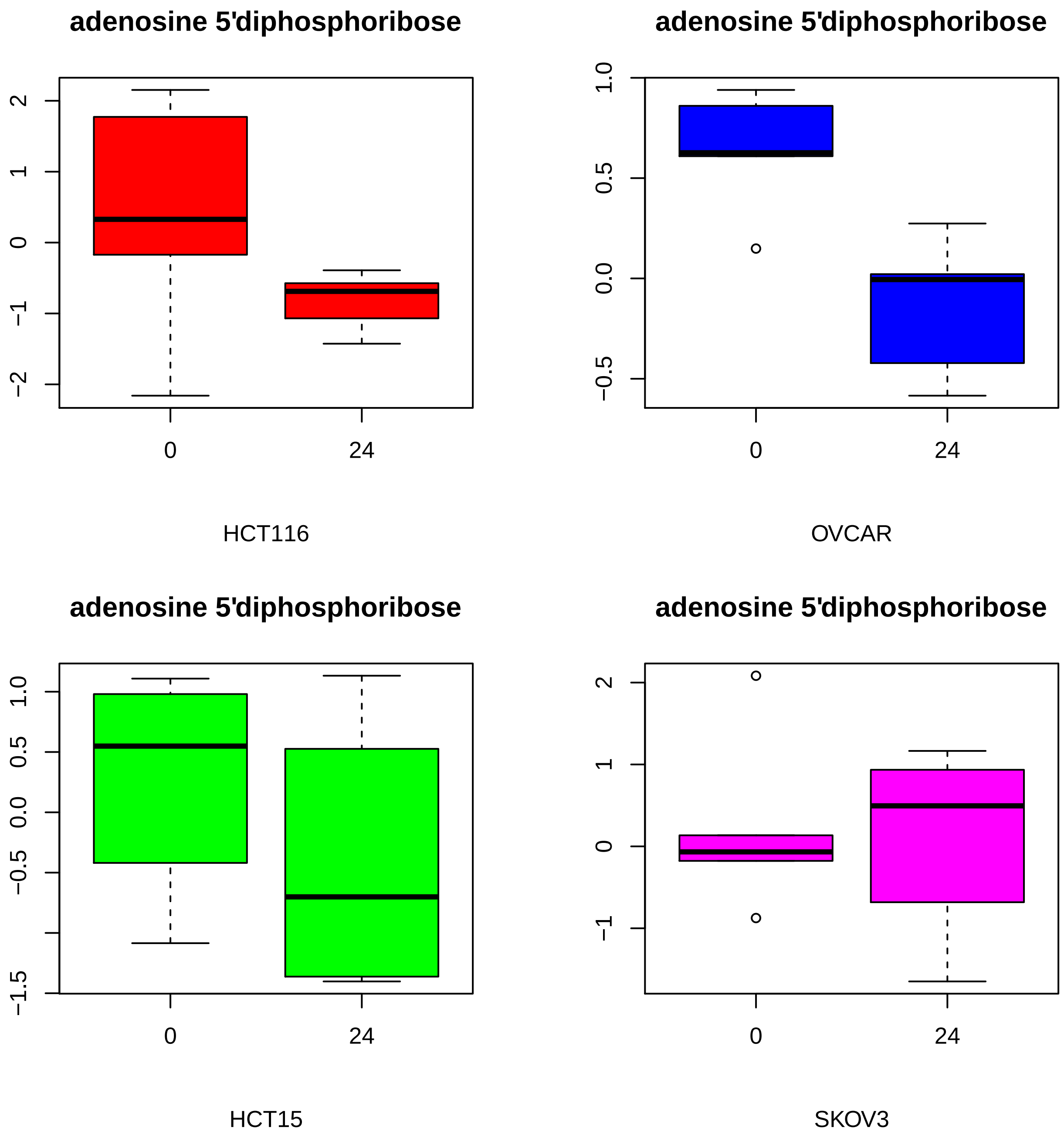
adenosine 5'diphosphoribose
2
1
0
−1
−2
0
24
HCT116
adenosine 5'diphosphoribose
1.0
0.5
0.0
−0.5
0
24
OVCAR
adenosine 5'diphosphoribose
1.0
0.5
0.0
−0.5
−1.5
0
24
HCT15
adenosine 5'diphosphoribose
2
1
0
−1
0
24
SKOV3

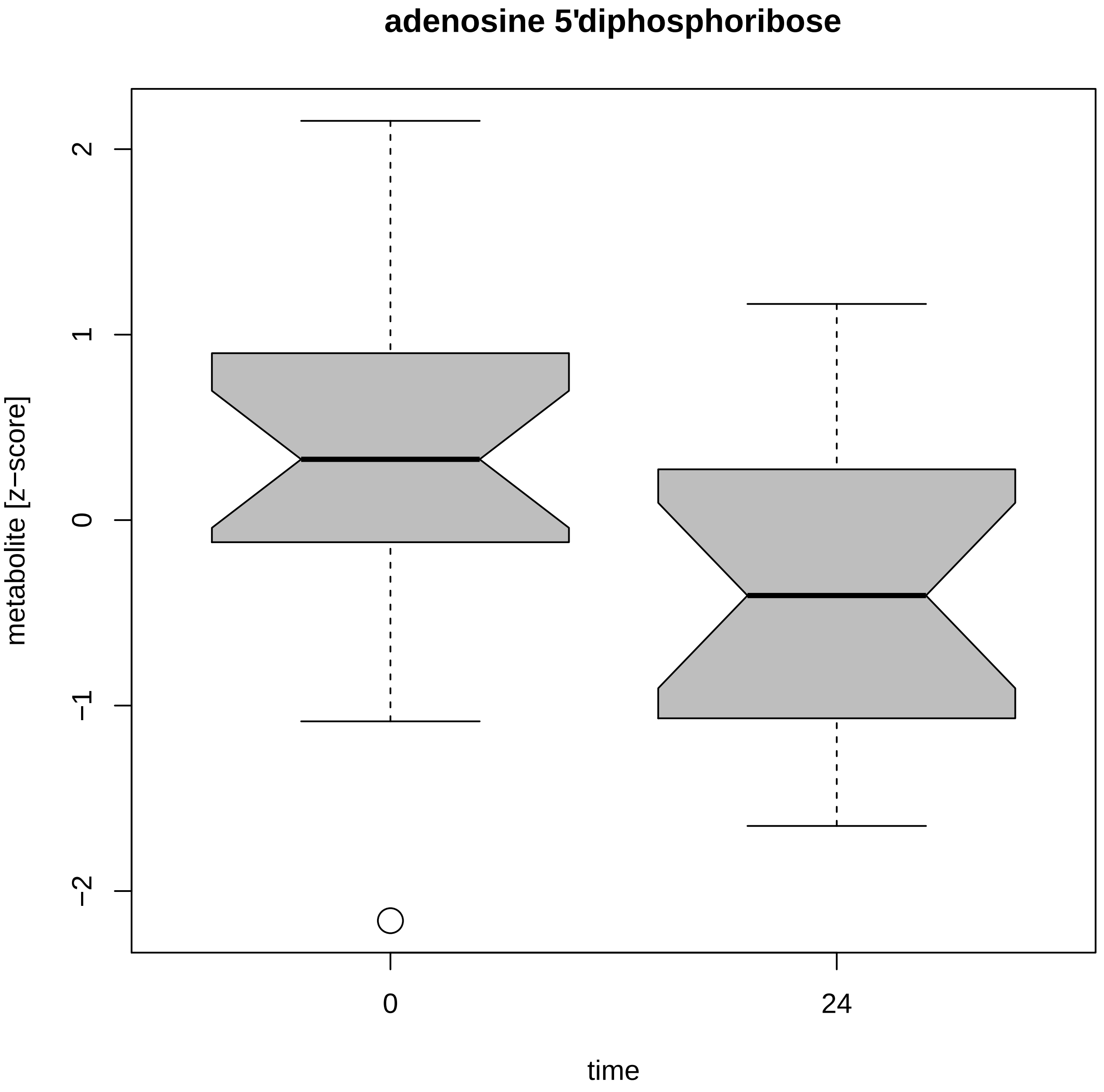
adenosine 5'diphosphoribose
metabolite [z−score]
2
1
0
−1
−2
0
24
time

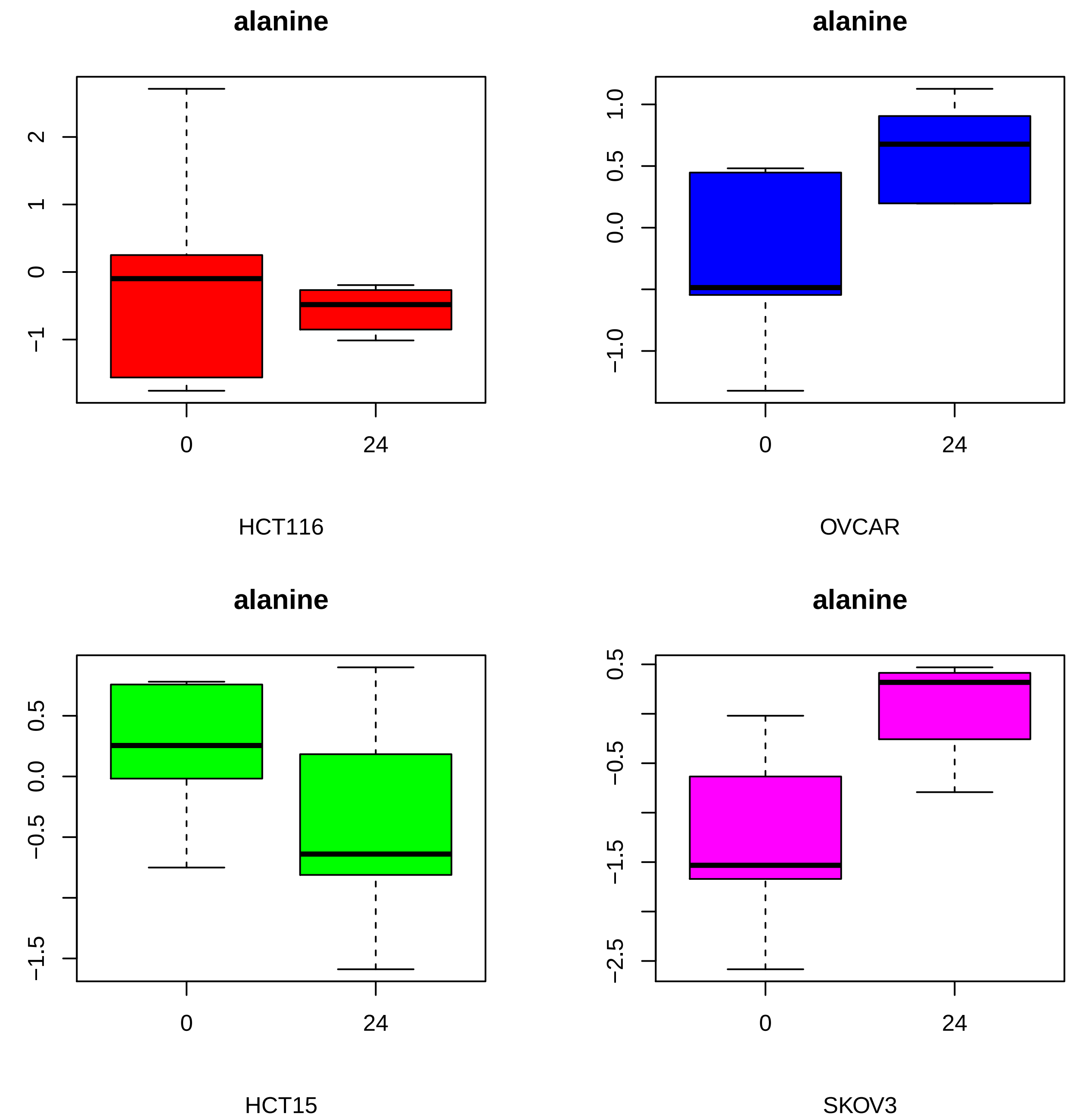
alanine
2
1
0
−1
0
24
HCT116
alanine
1.0
0.5
0.0
−1.0
0
24
OVCAR
alanine
0.5
0.0
−0.5
−1.5
0
24
HCT15
alanine
0.5
−0.5
−1.5
−2.5
0
24
SKOV3

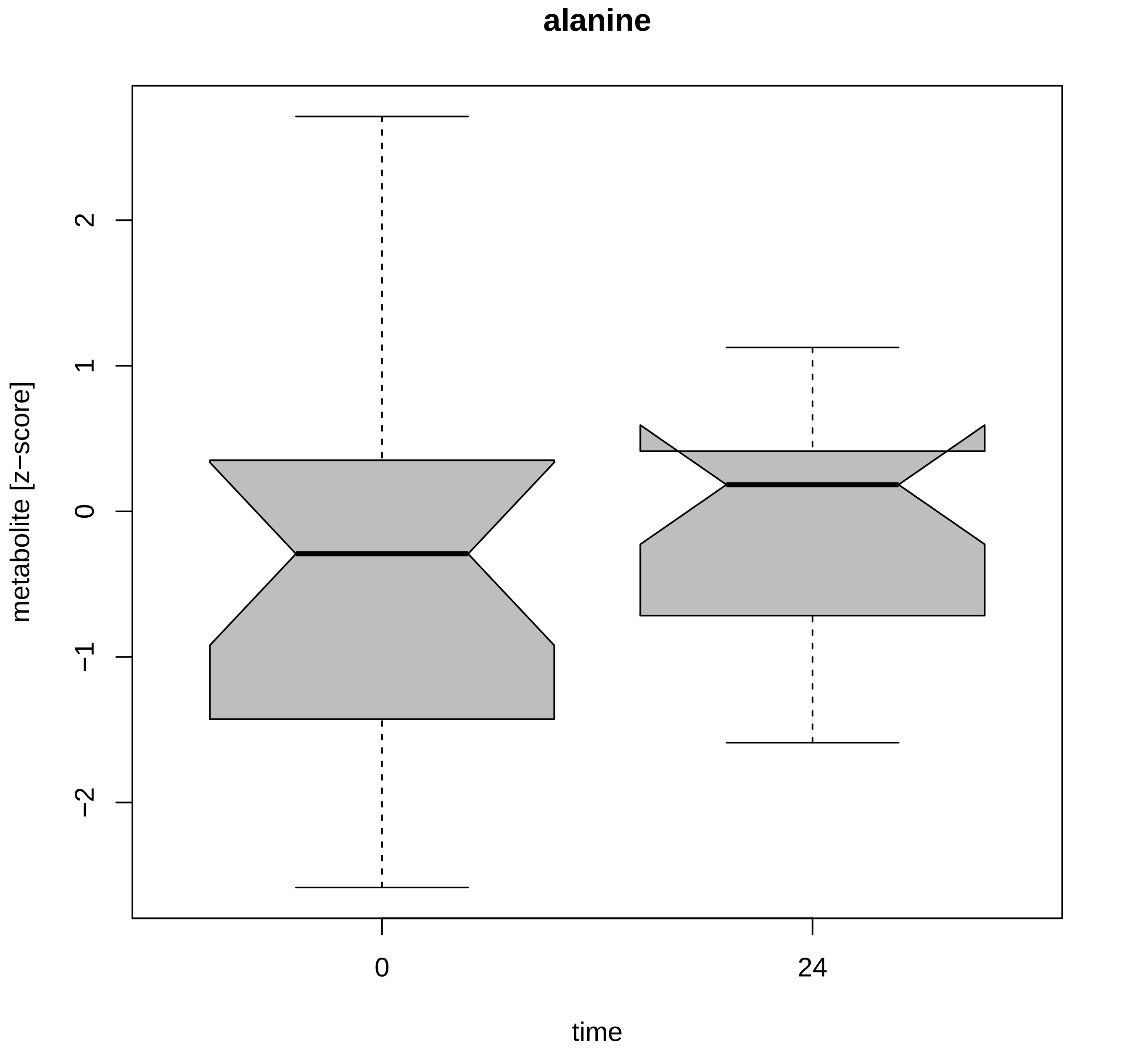

alanine
metabolite [z−score]
2
1
0
−1
−2
0
24
time

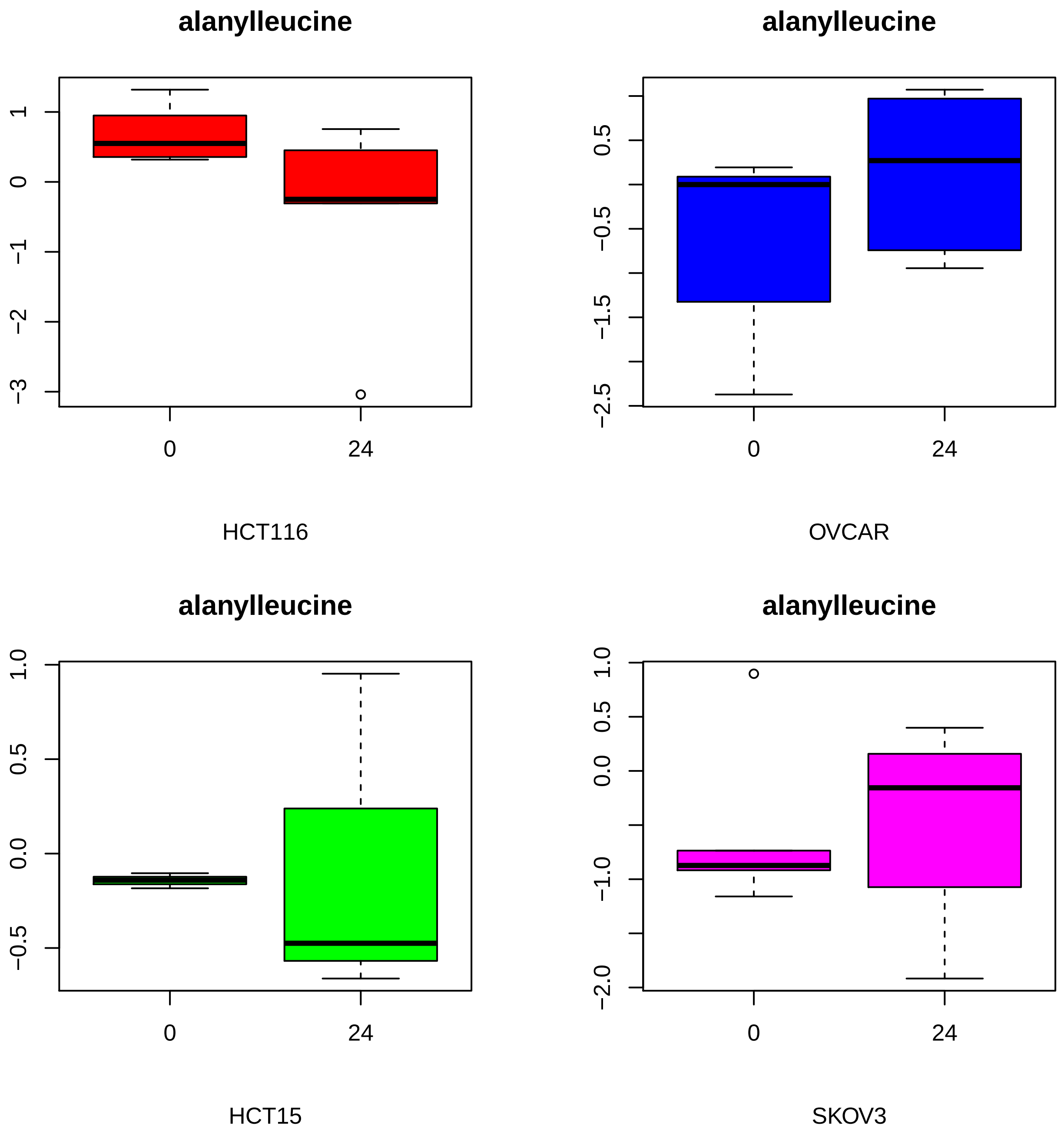
alanylleucine
1
0
−1
−2
−3
0
24
HCT116
alanylleucine
0.5
−0.5
−1.5
−2.5
0
24
OVCAR
alanylleucine
1.0
0.5
0.0
−0.5
0
24
HCT15
alanylleucine
1.0
0.5
0.0
−1.0
−2.0
0
24
SKOV3

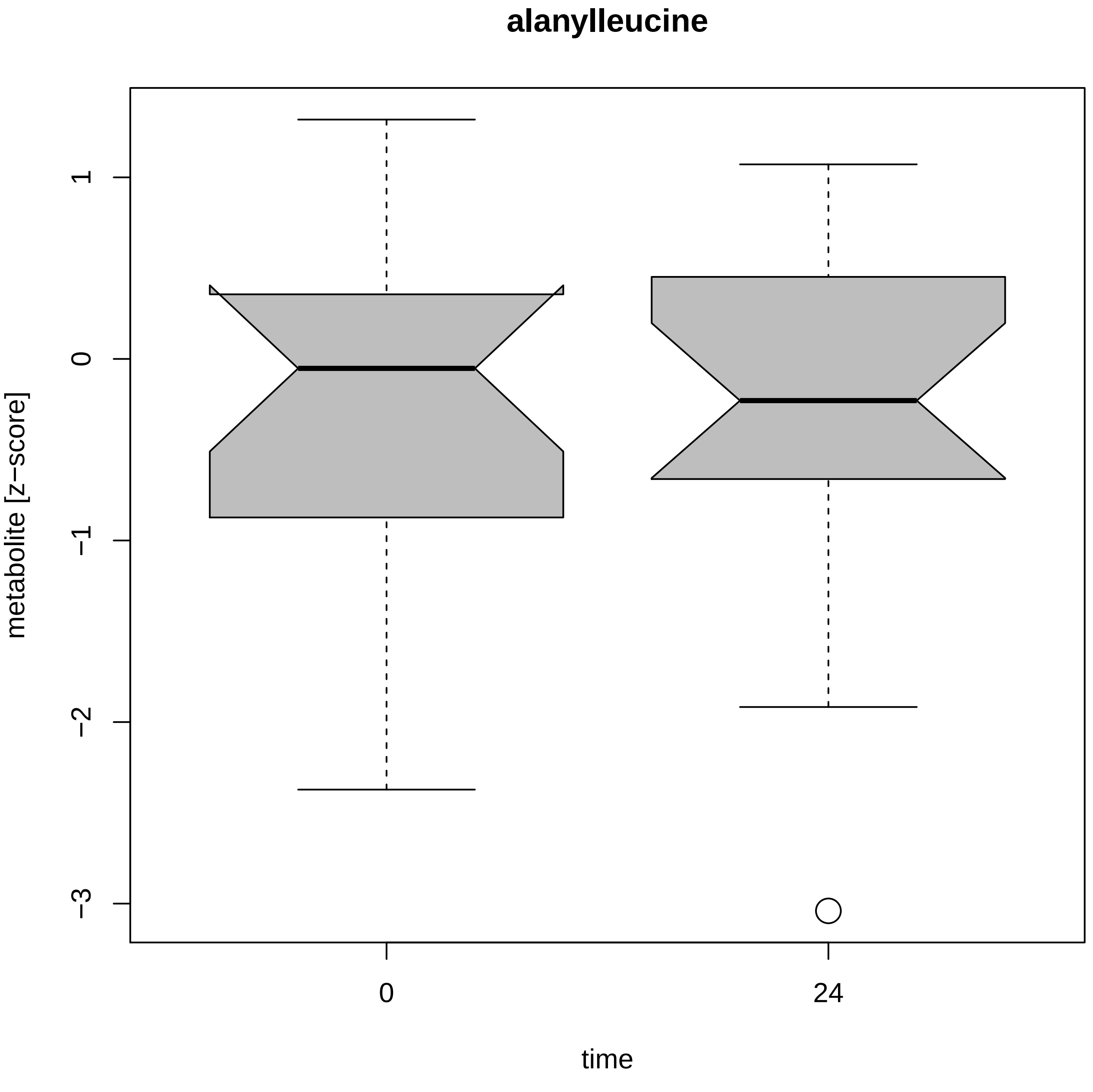
alanylleucine
metabolite [z−score]
1
0
−1
−2
−3
0
24
time

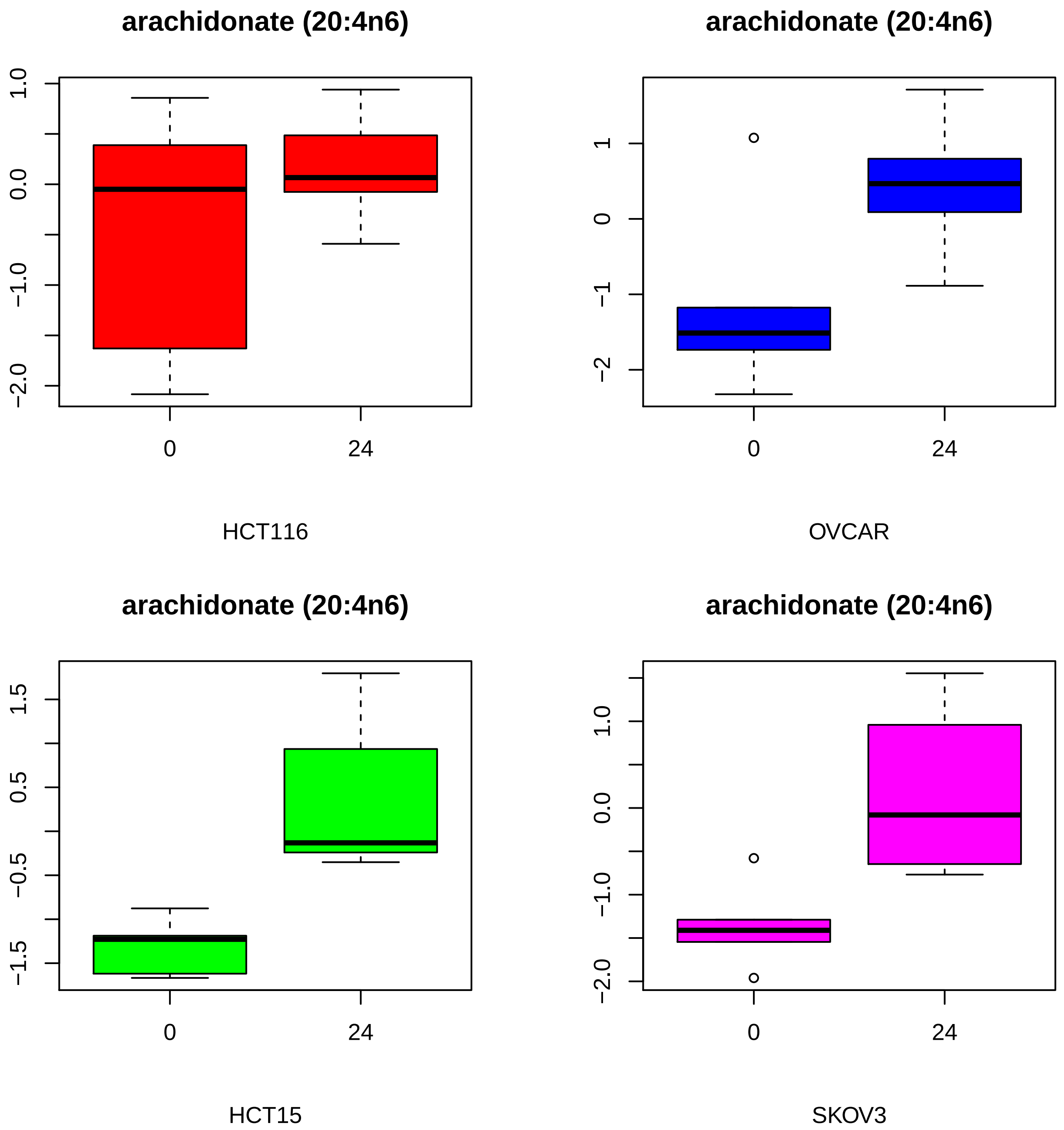
arachidonate (20:4n6)
1.0
0.0
−1.0
−2.0
0
24
HCT116
arachidonate (20:4n6)
1
0
−1
−2
0
24
OVCAR
arachidonate (20:4n6)
1.5
0.5
−0.5
−1.5
0
24
HCT15
arachidonate (20:4n6)
1.0
0.0
−1.0
−2.0
0
24
SKOV3

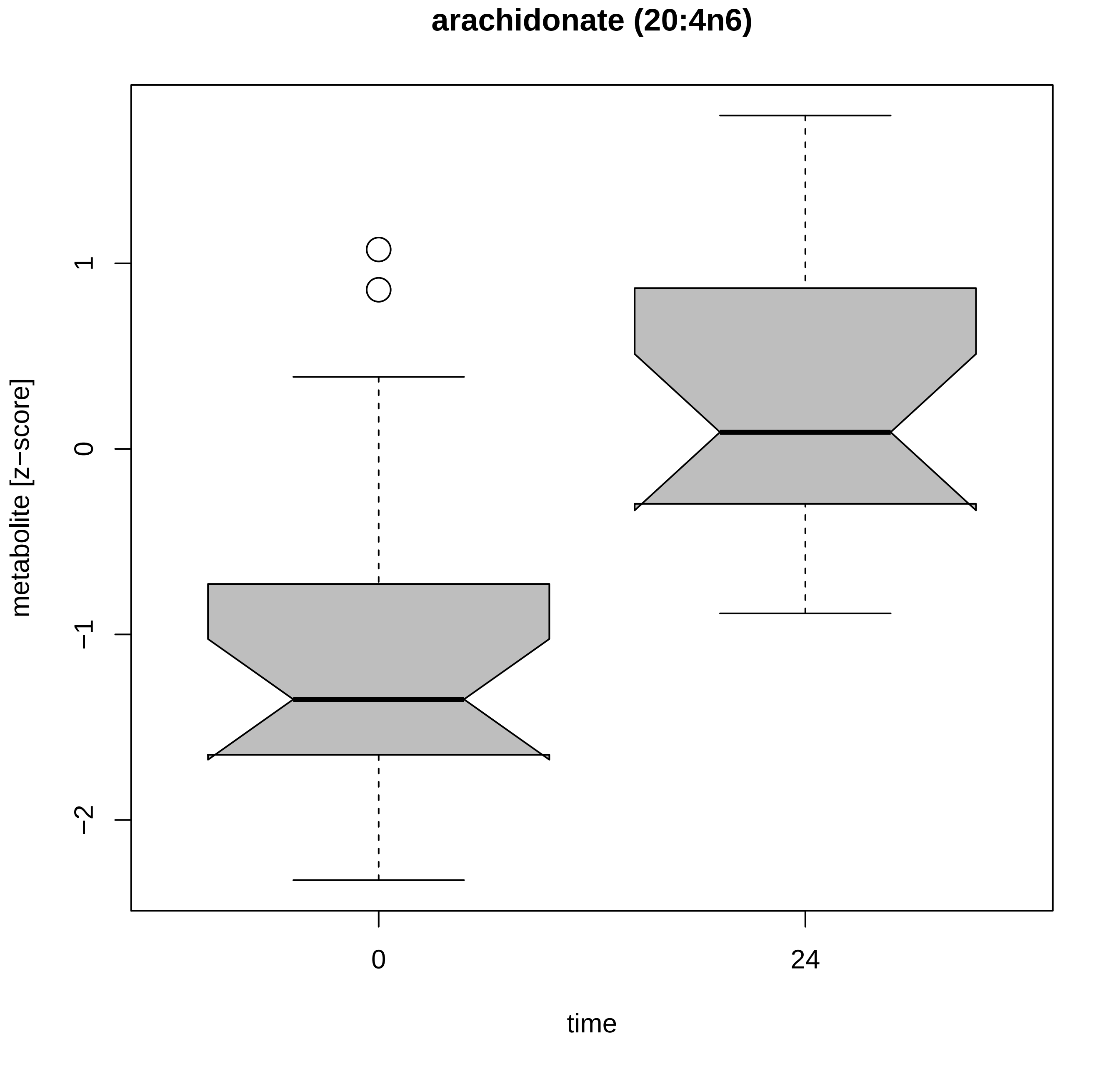

arachidonate (20:4n6)
metabolite [z−score]
1
0
−1
−2
0
24
time

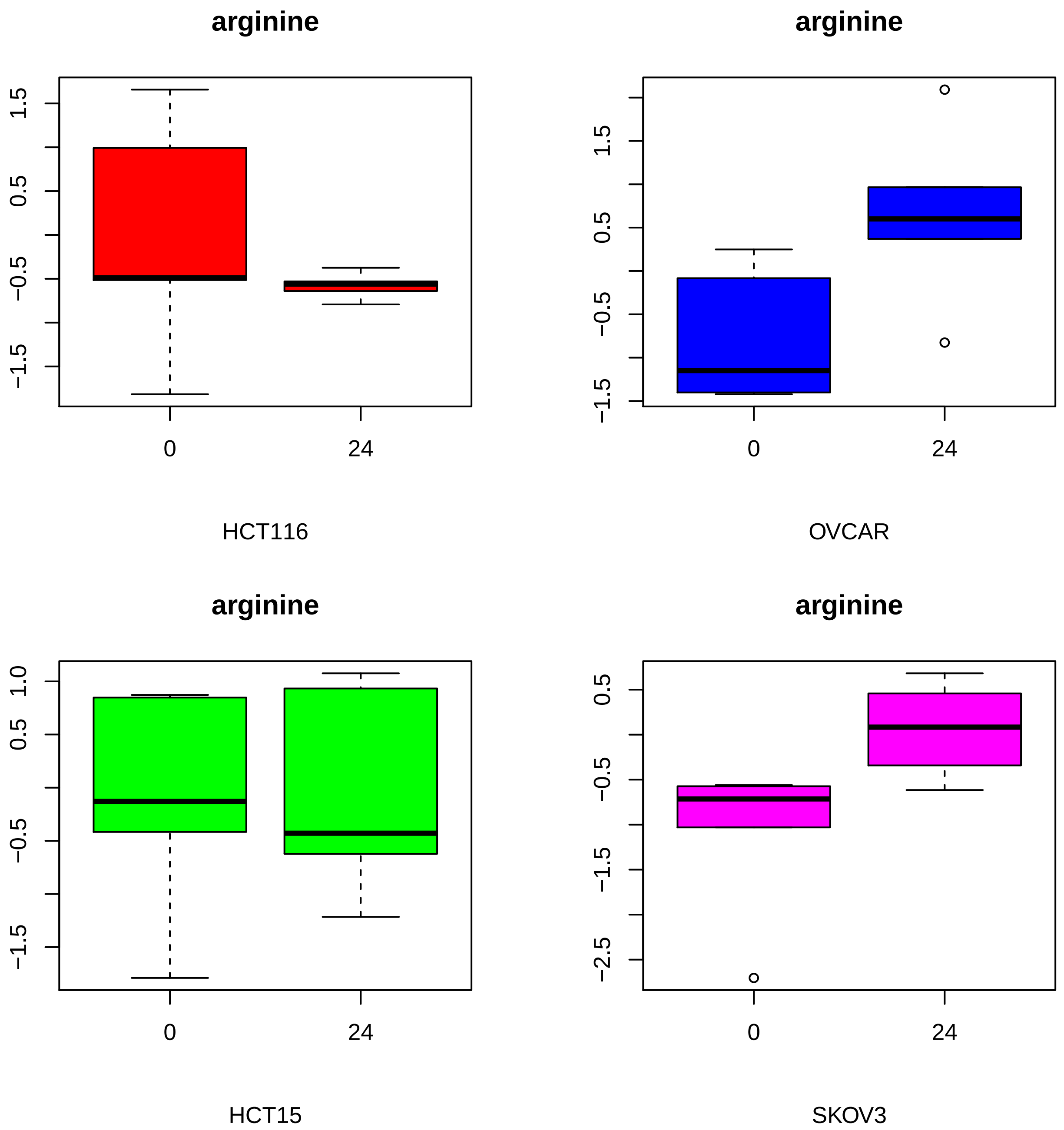
arginine
1.5
0.5
−0.5
−1.5
0
24
HCT116
arginine
1.5
0.5
−0.5
−1.5
0
24
OVCAR
arginine
1.0
0.5
−0.5
−1.5
0
24
HCT15
arginine
0.5
−0.5
−1.5
−2.5
0
24
SKOV3

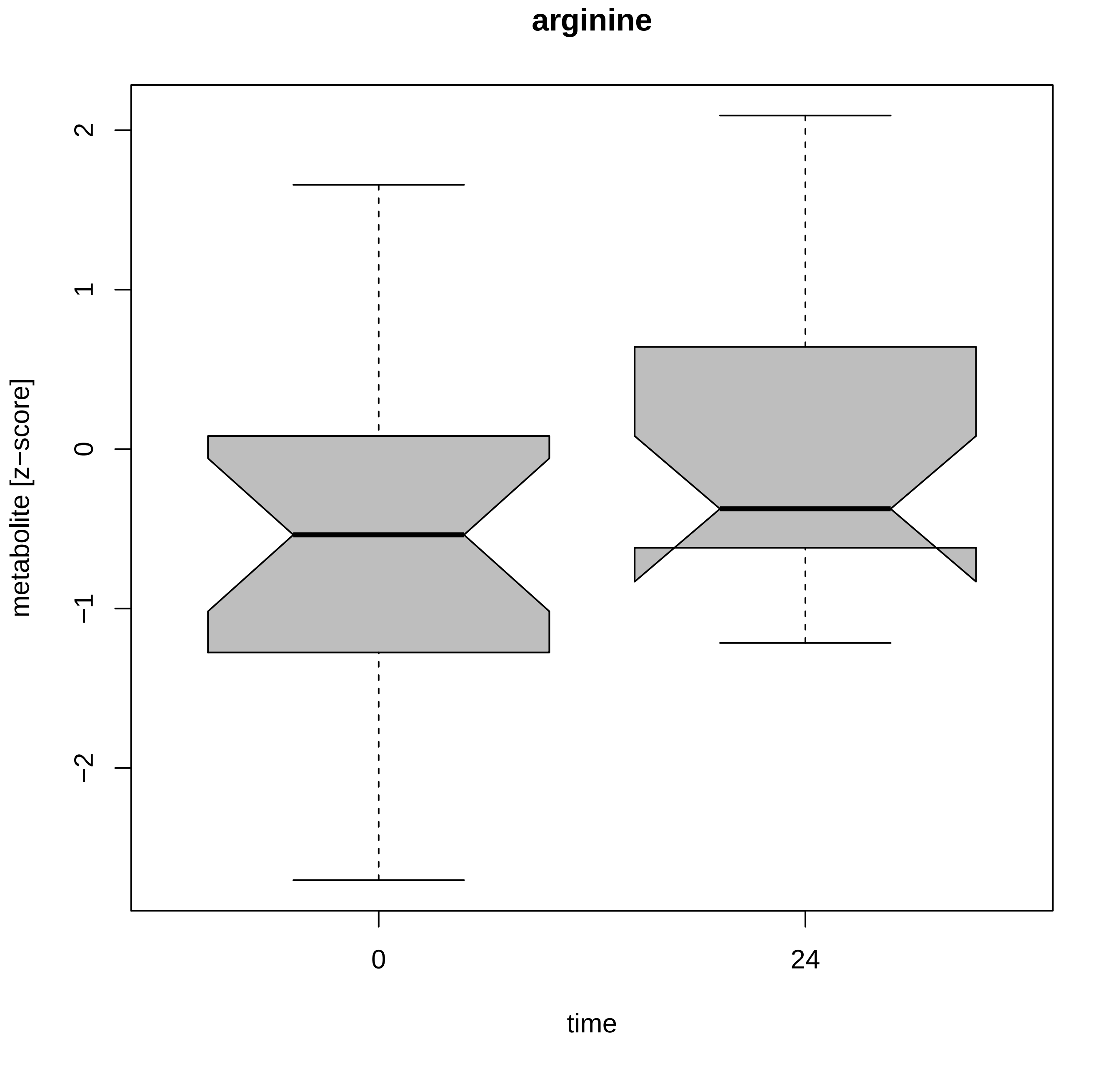

arginine
metabolite [z−score]
2
1
0
−1
−2
0
24
time

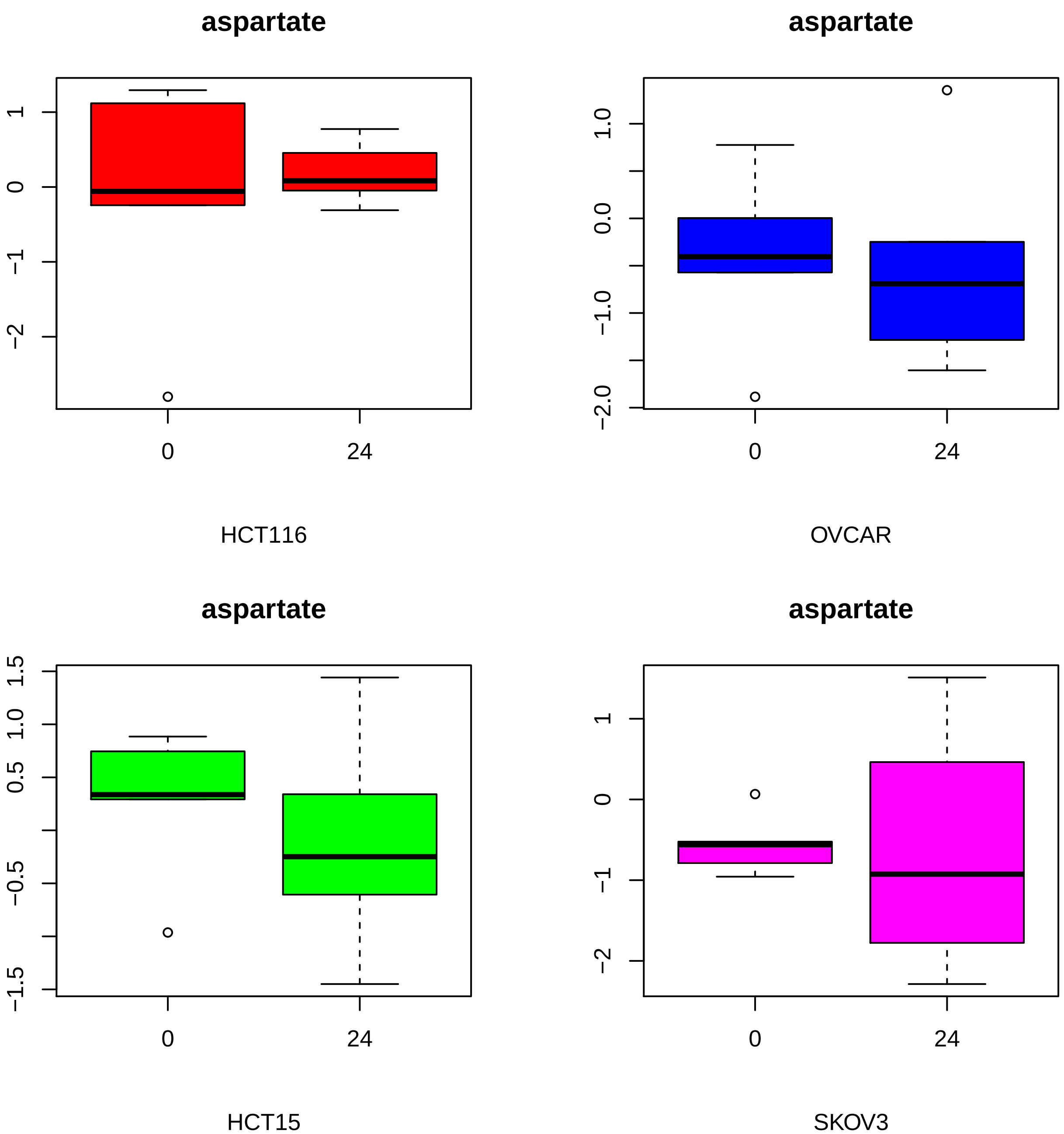
aspartate
1
0
−1
−2
0
24
HCT116
aspartate
1.0
0.0
−1.0
−2.0
0
24
OVCAR
aspartate
1.5
1.0
0.5
−0.5
−1.5
0
24
HCT15
aspartate
1
0
−1
−2
0
24
SKOV3

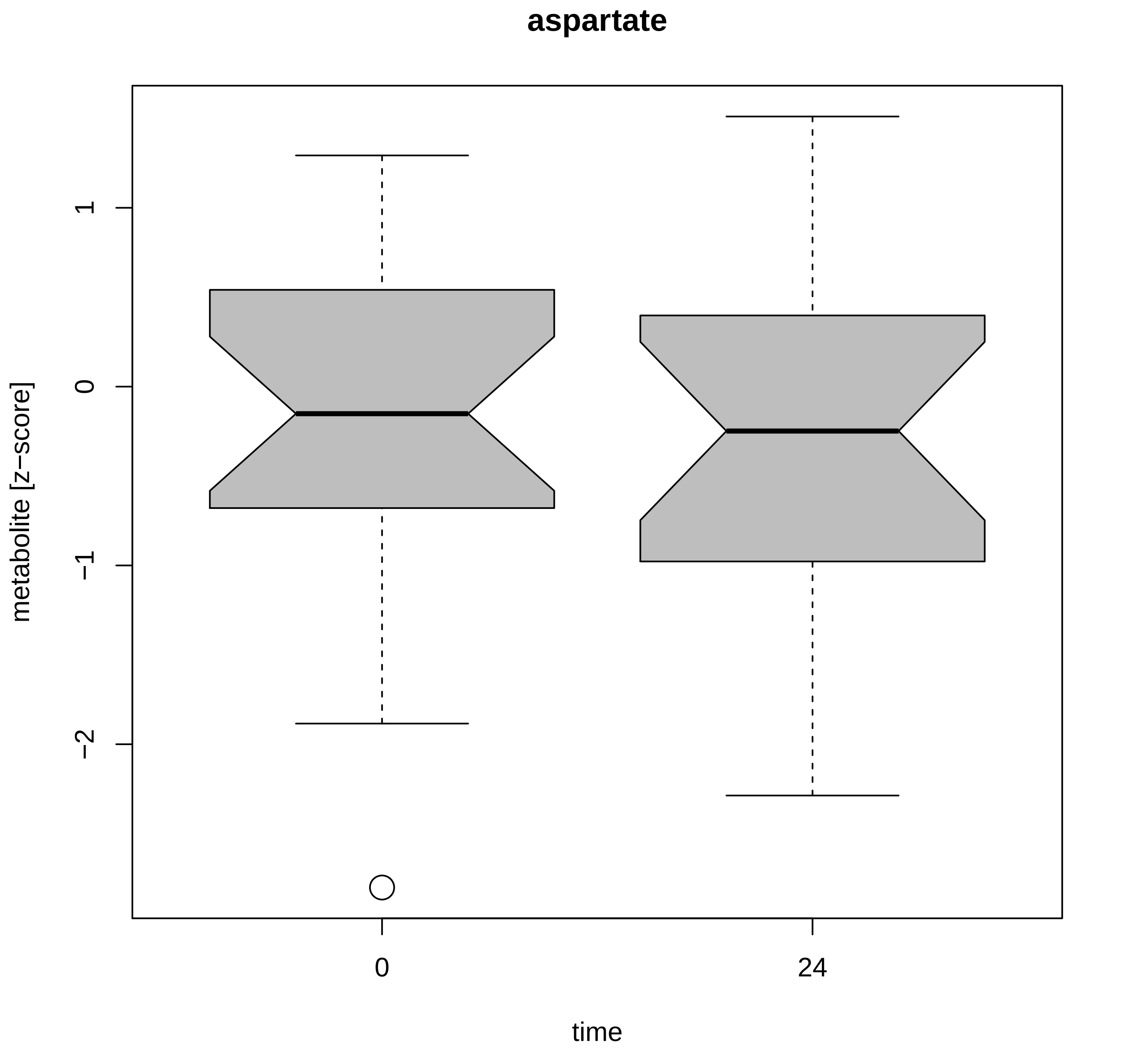
aspartate
metabolite [z−score]
1
0
−1
−2
0
24
time

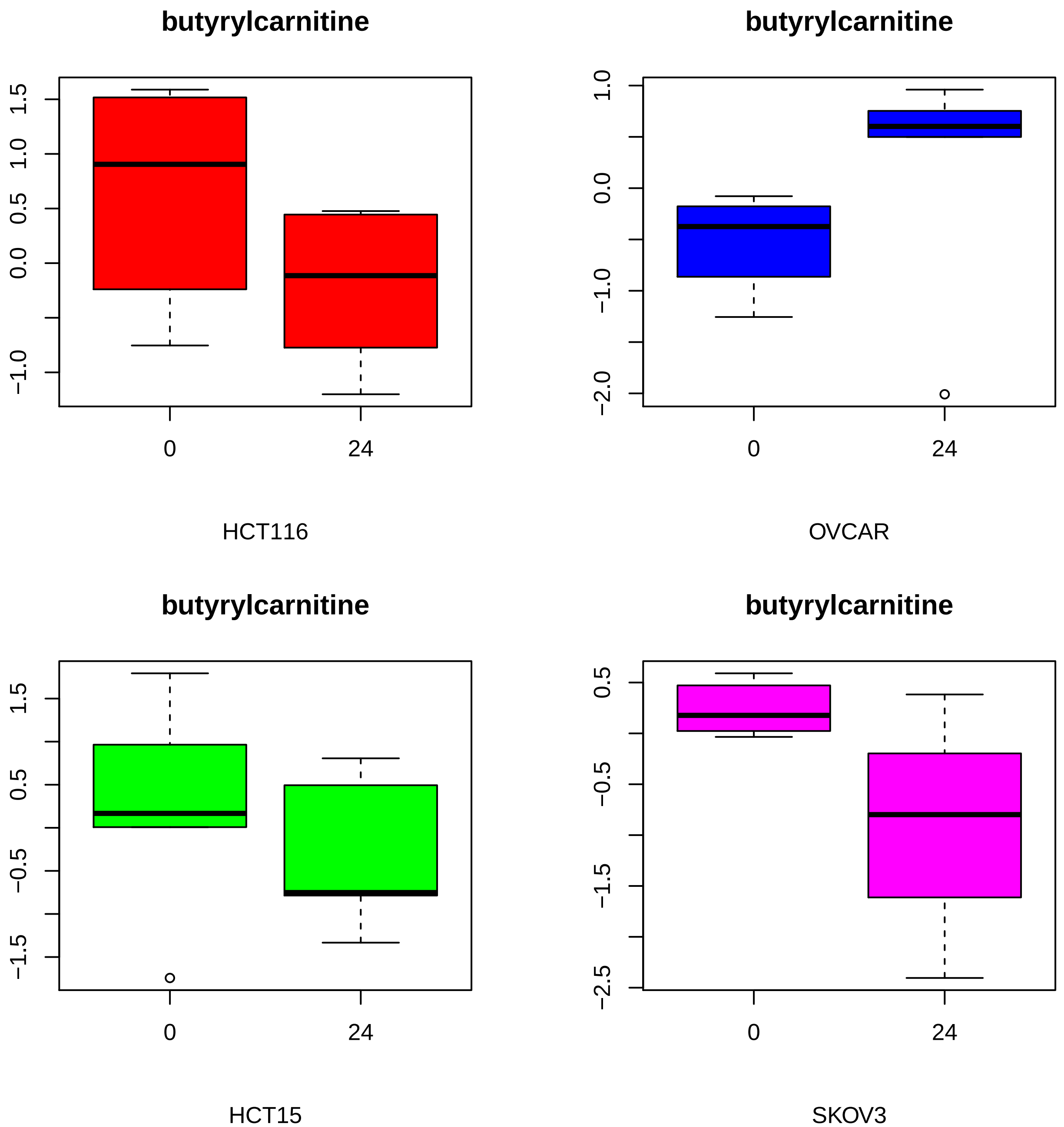
butyrylcarnitine
1.5
1.0
0.5
0.0
−1.0
0
24
HCT116
butyrylcarnitine
1.0
0.0
−1.0
−2.0
0
24
OVCAR
butyrylcarnitine
1.5
0.5
−0.5
−1.5
0
24
HCT15
butyrylcarnitine
0.5
−0.5
−1.5
−2.5
0
24
SKOV3

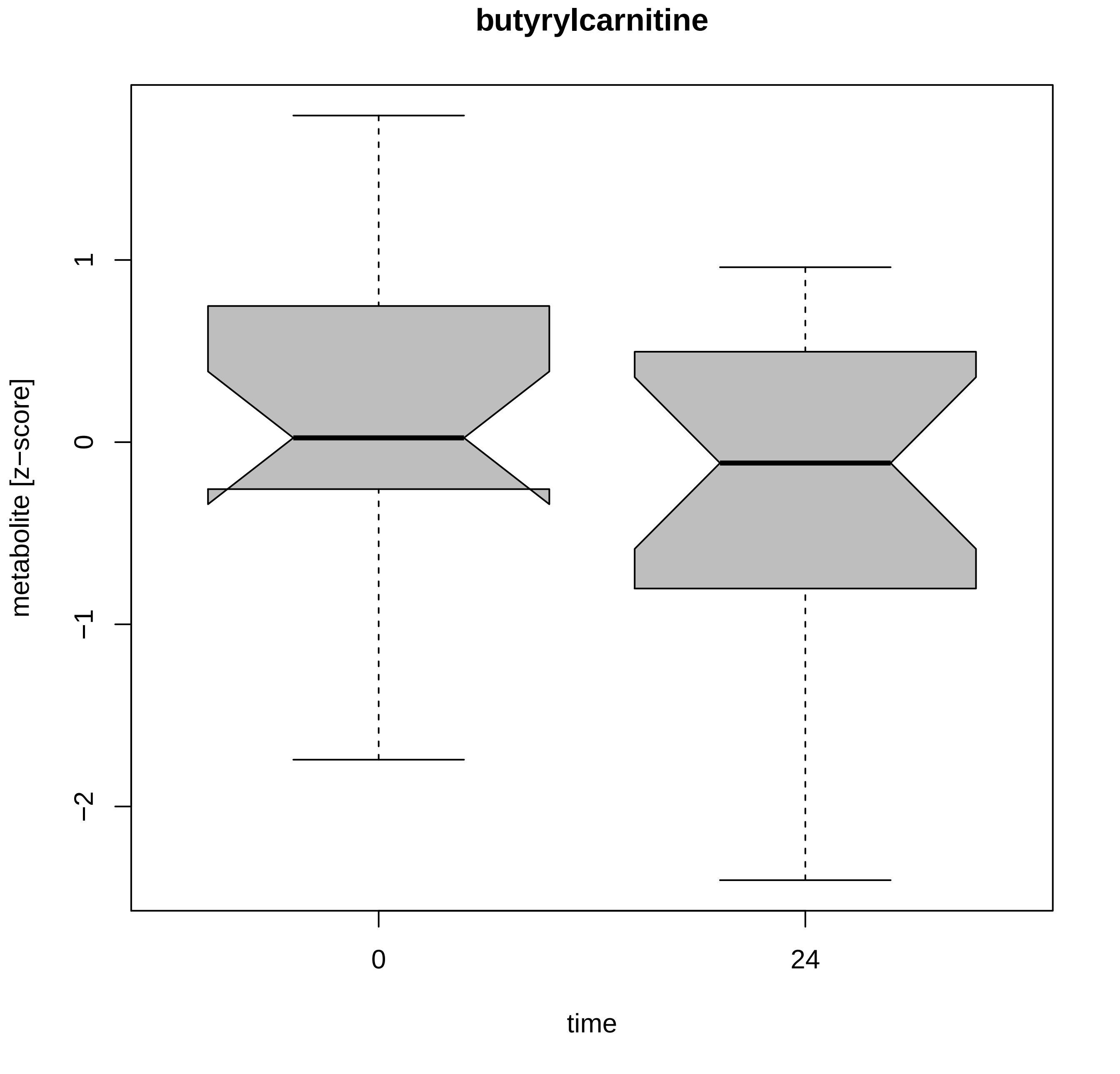
butyrylcarnitine
metabolite [z−score]
1
0
−1
−2
0
24
time

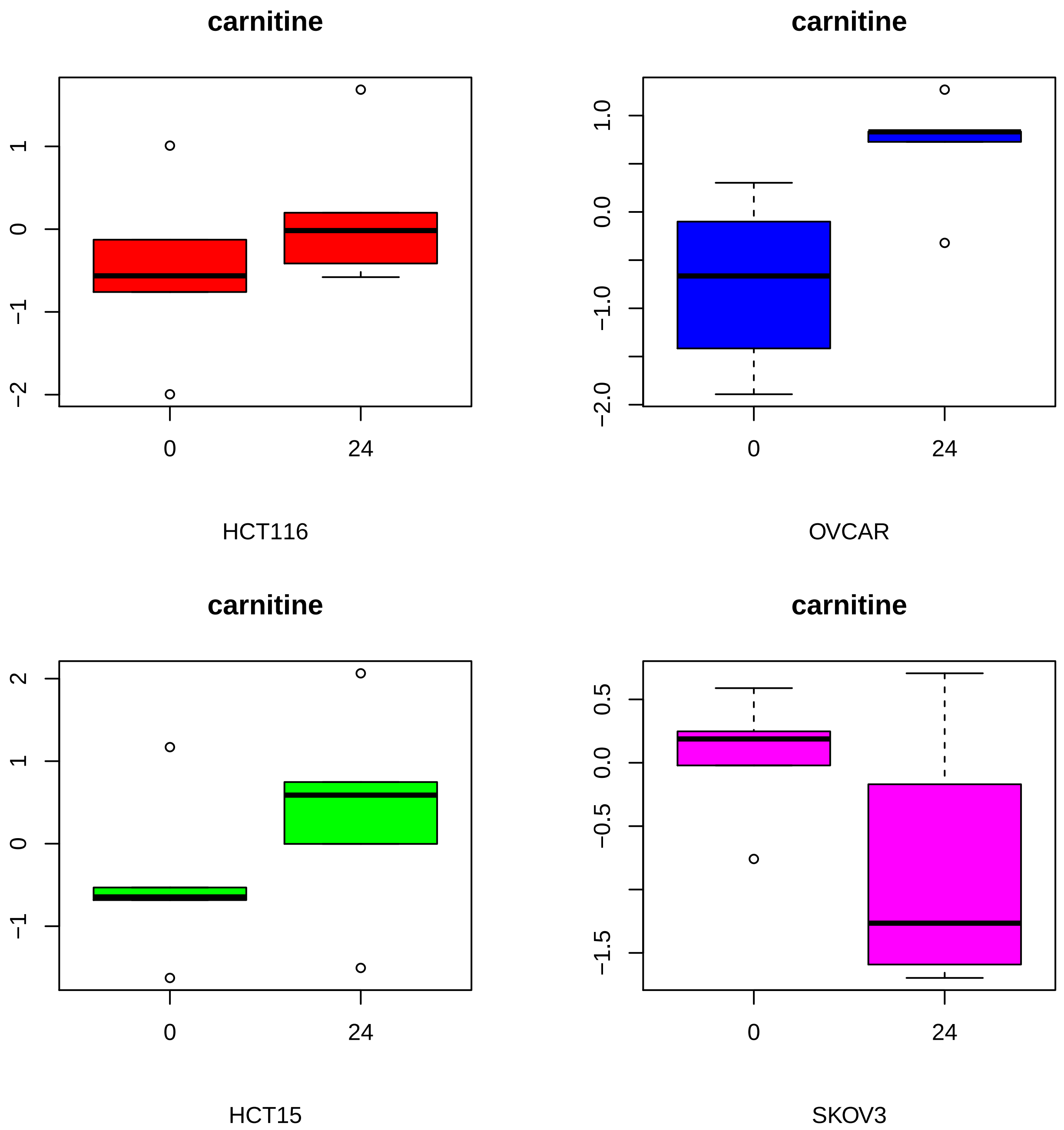
carnitine
1
0
−1
−2
0
24
HCT116
carnitine
1.0
0.0
−1.0
−2.0
0
24
OVCAR
carnitine
2
1
0
−1
0
24
HCT15
carnitine
0.5
0.0
−0.5
−1.5
0
24
SKOV3

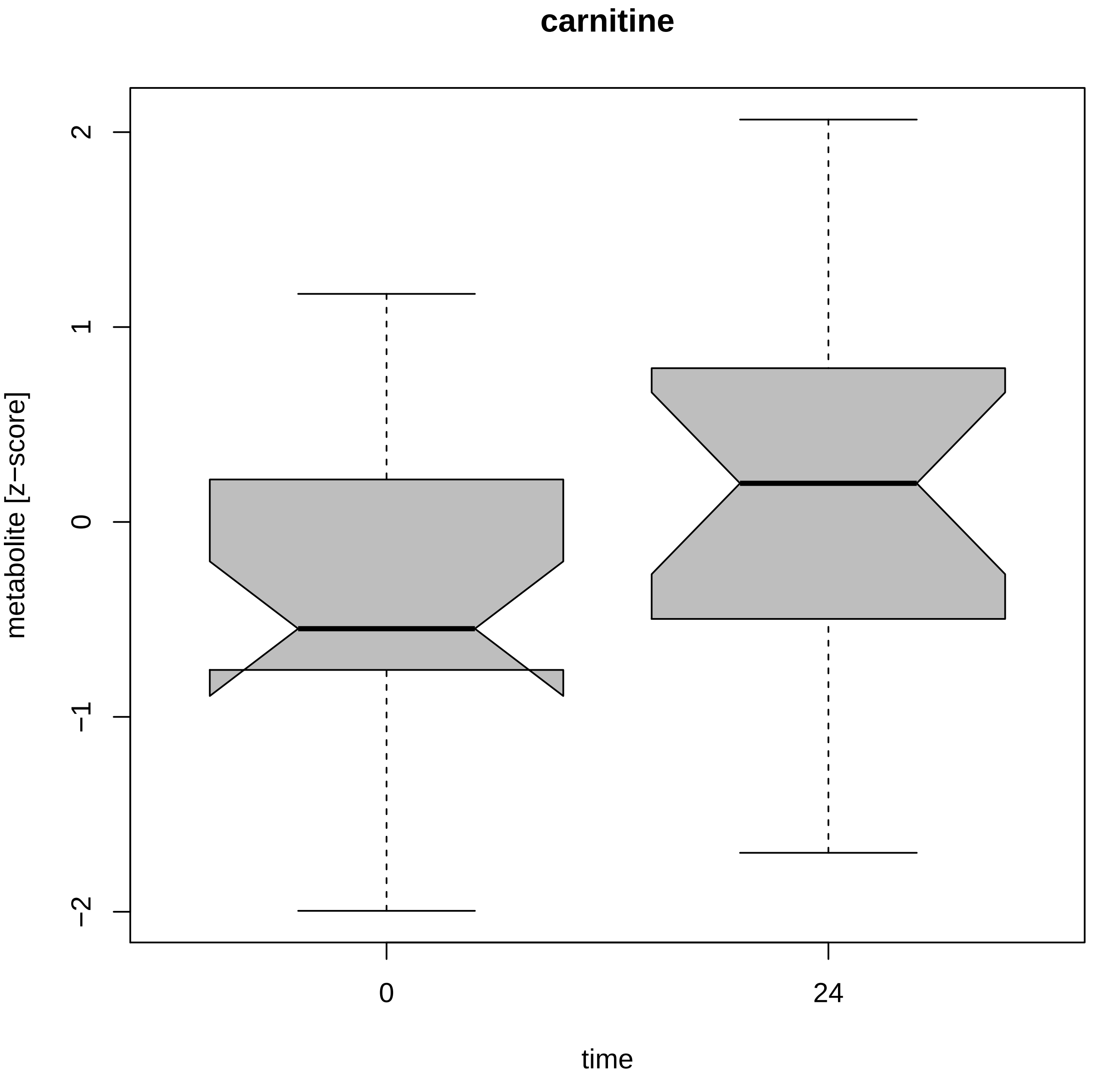
carnitine
metabolite [z−score]
2
1
0
−1
−2
0
24
time

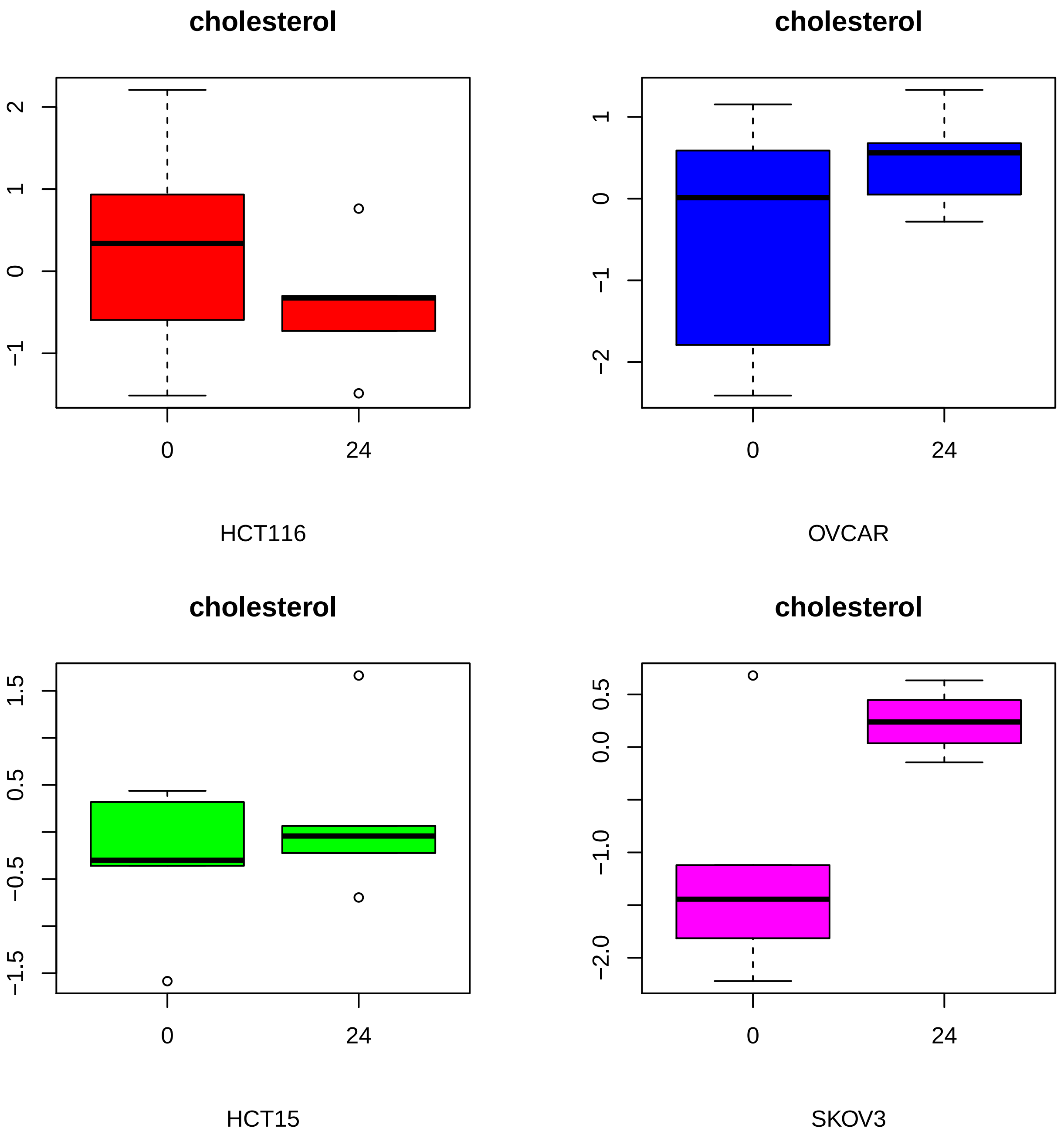
cholesterol
2
1
0
−1
0
24
HCT116
cholesterol
1
0
−1
−2
0
24
OVCAR
cholesterol
1.5
0.5
−0.5
−1.5
0
24
HCT15
cholesterol
0.5
0.0
−1.0
−2.0
0
24
SKOV3

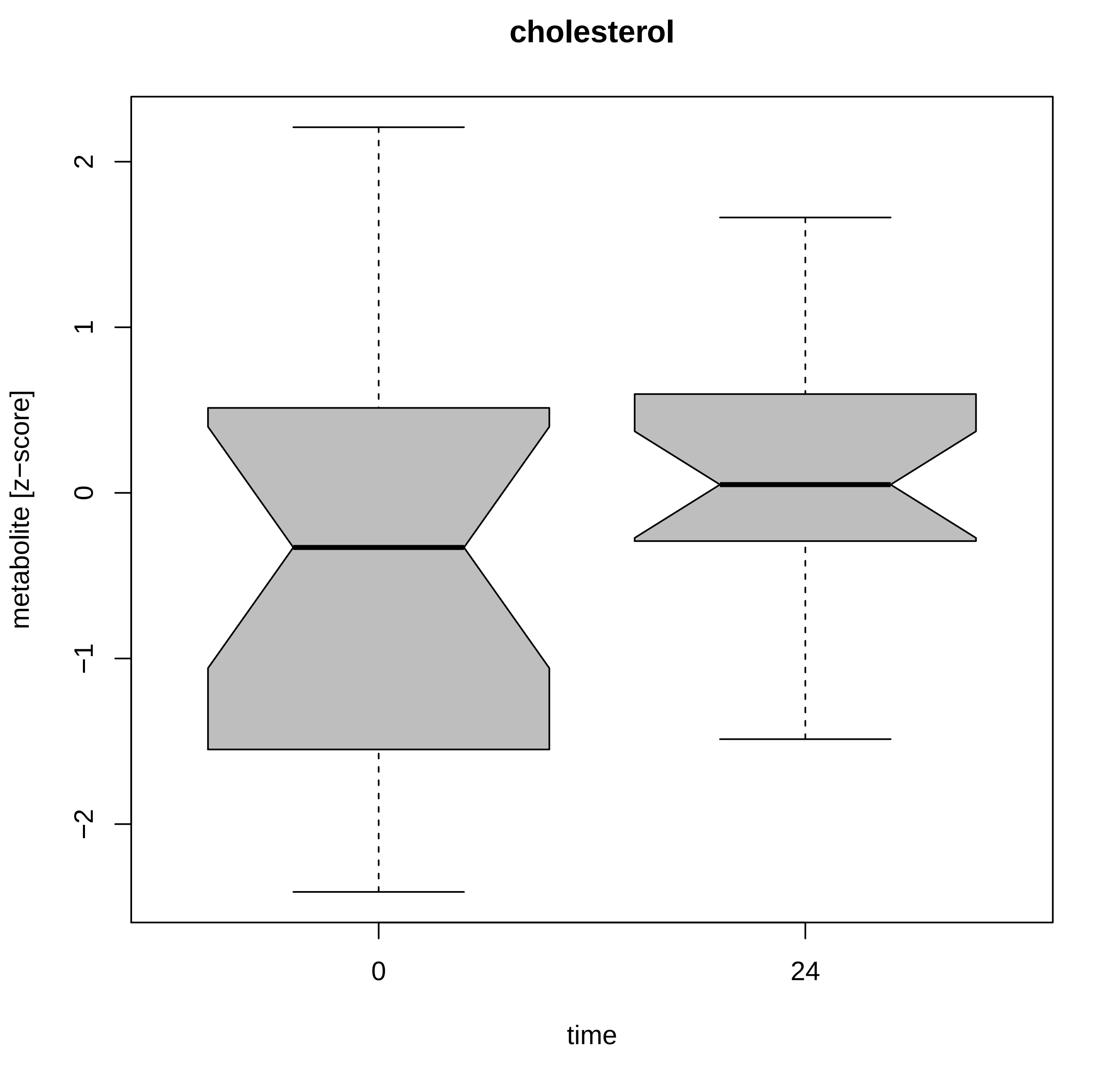
cholesterol
metabolite [z−score]
2
1
0
−1
−2
0
24
time

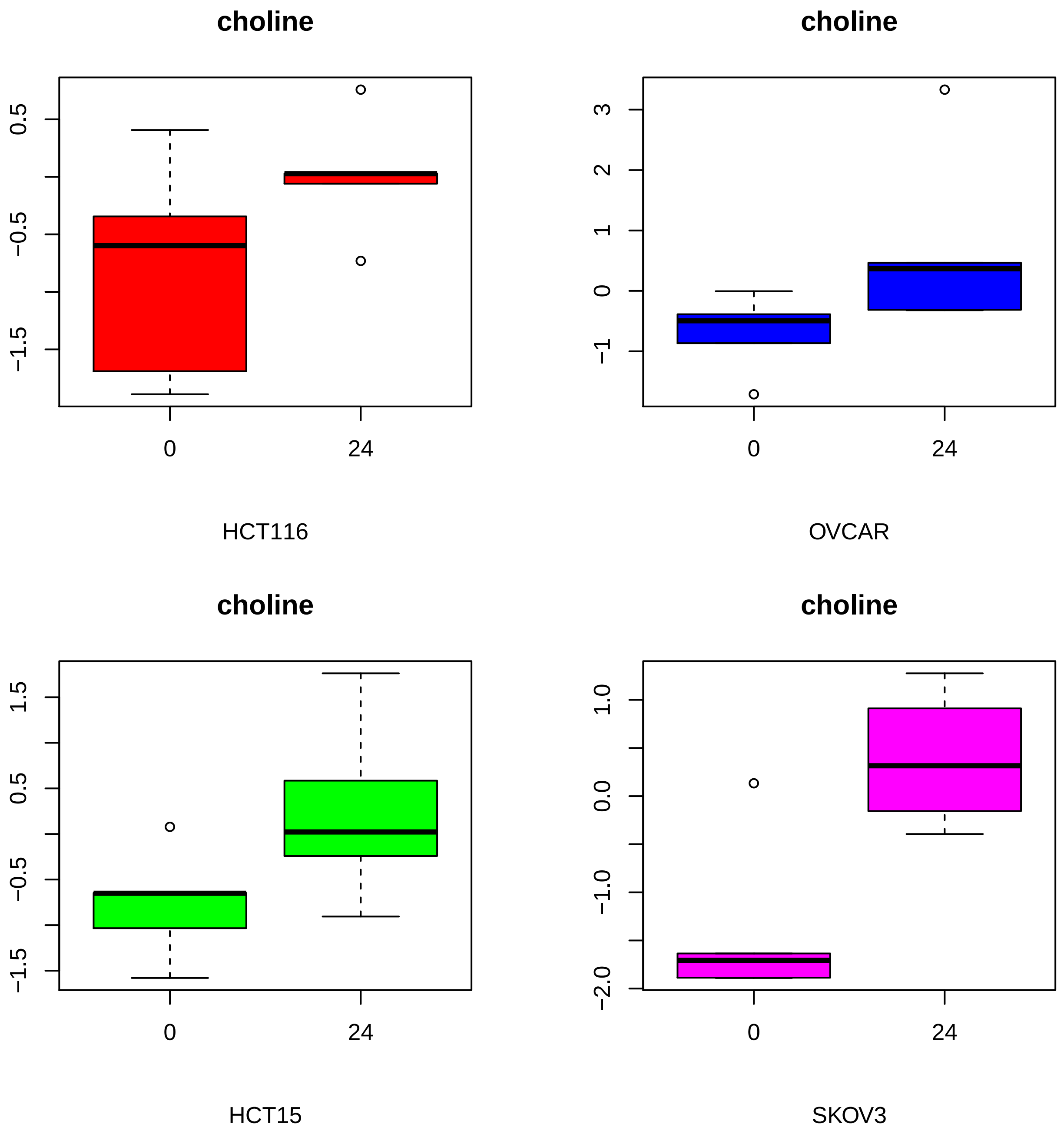
choline
0.5
−0.5
−1.5
0
24
HCT116
choline
3
2
1
0
−1
0
24
OVCAR
choline
1.5
0.5
−0.5
−1.5
0
24
HCT15
choline
1.0
0.0
−1.0
−2.0
0
24
SKOV3

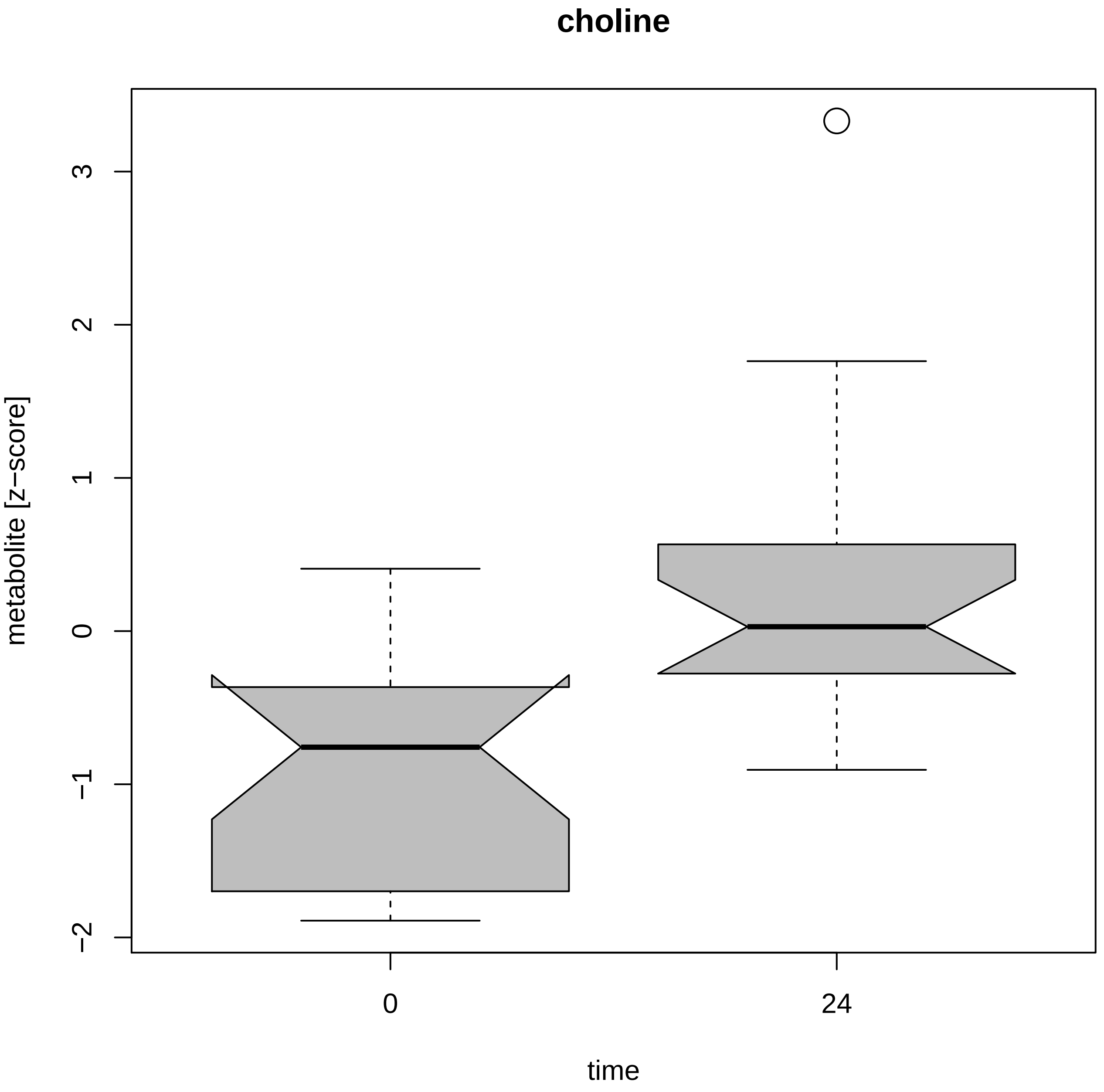

choline
metabolite [z−score]
3
2
1
0
−1
−2
0
24
time

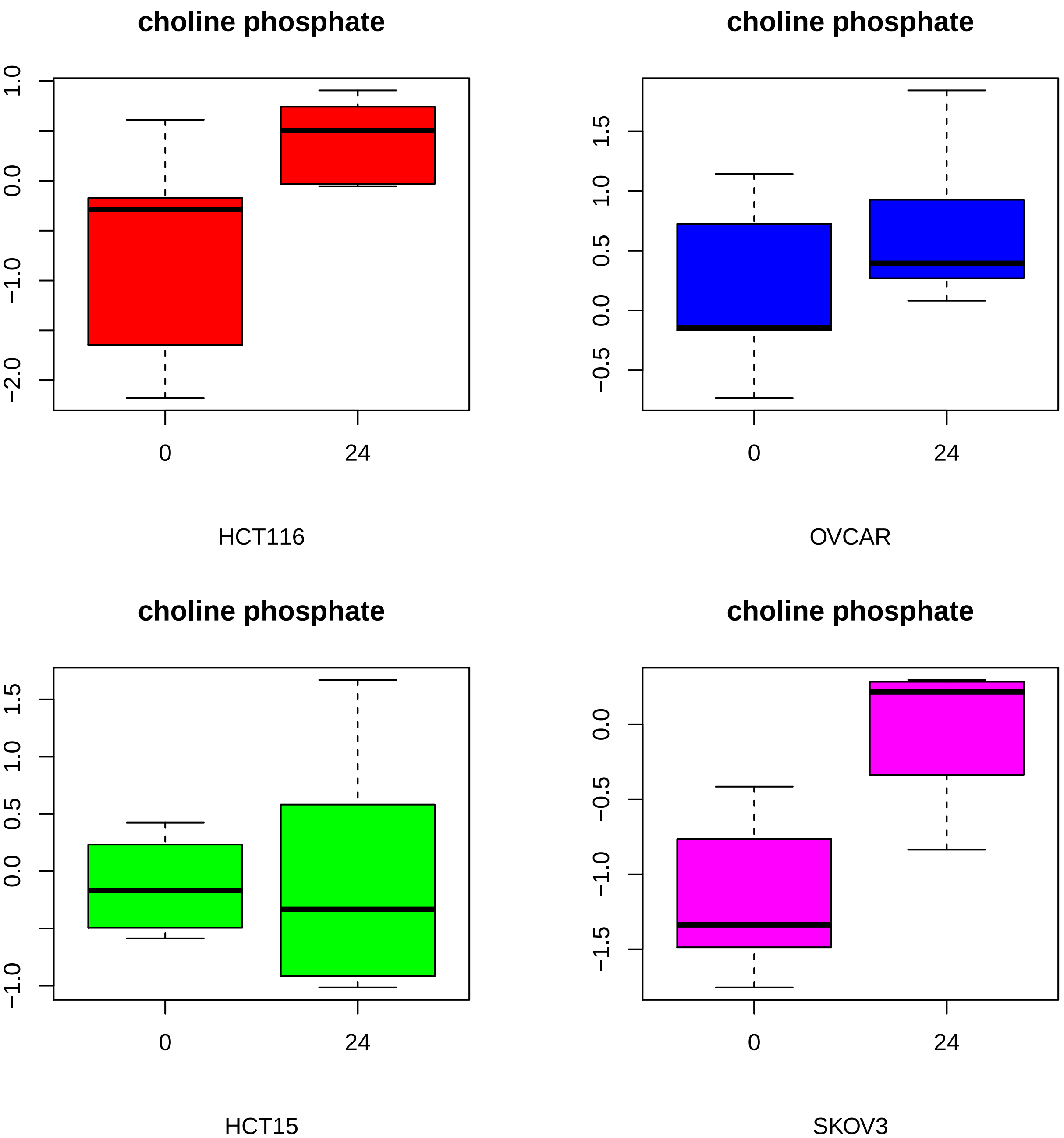

choline phosphate
choline phosphate
choline phosphate
choline phosphate
1.0
0.0
−1.0
−2.0
1.5
1.0
0.5
0.0
−0.5
0
24
HCT116
OVCAR
HCT15
SKOV3

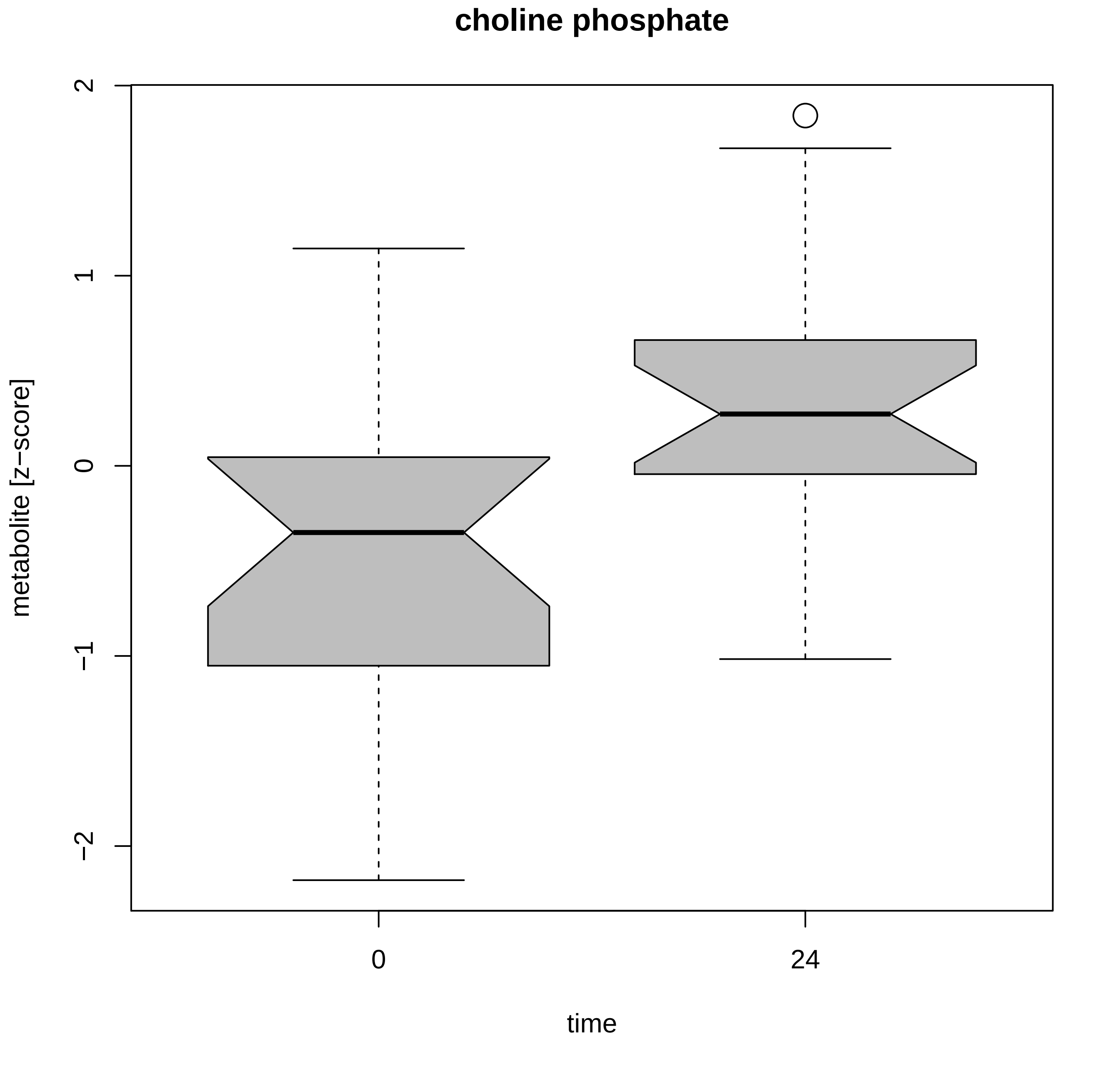
choline phosphate
metabolite [z−score]
2
1
0
−1
−2
0
24
time

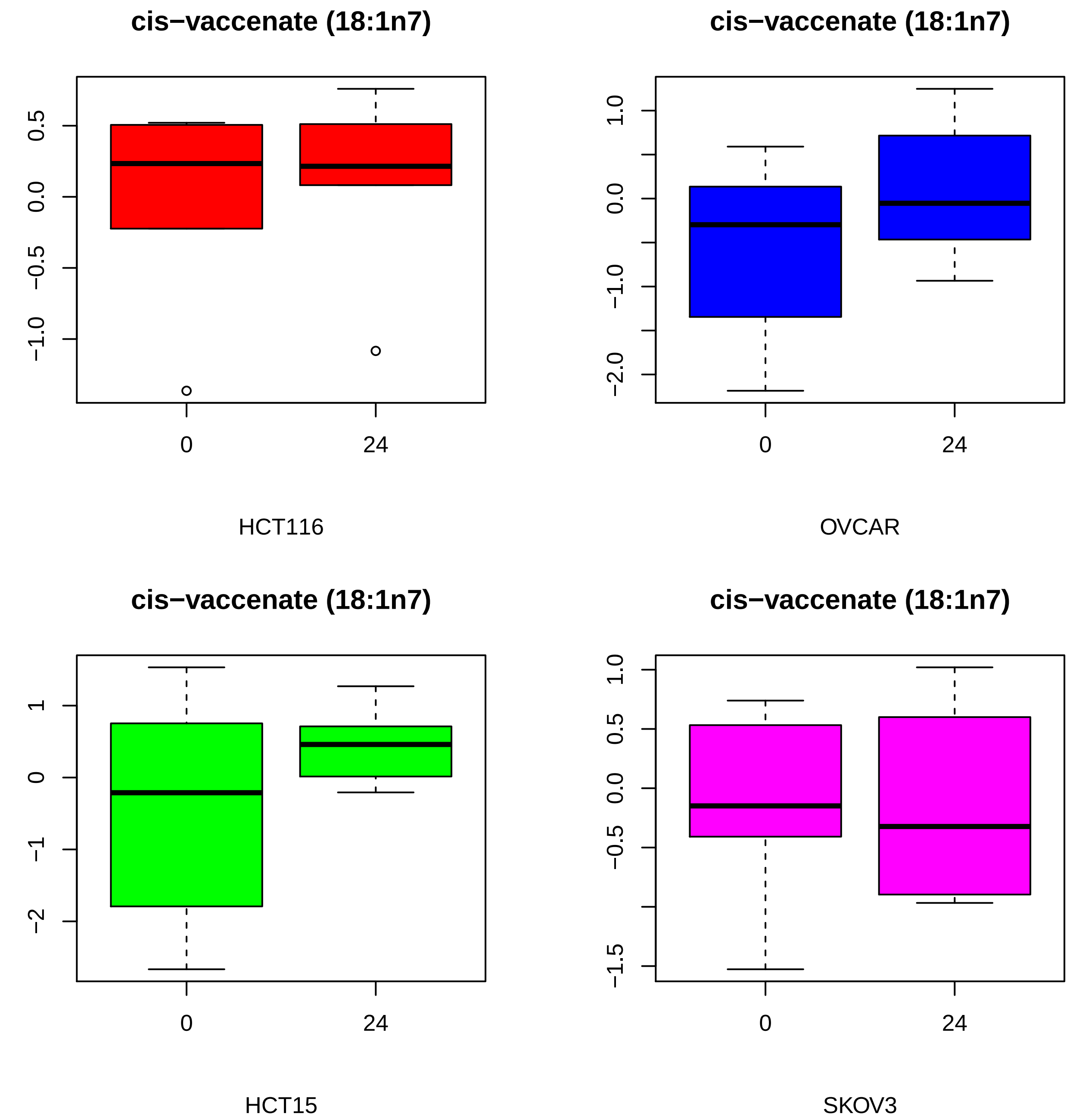
cis−vaccenate (18:1n7)
0.5
0.0
−0.5
−1.0
0
24
HCT116
cis−vaccenate (18:1n7)
1.0
0.0
−1.0
−2.0
0
24
OVCAR
cis−vaccenate (18:1n7)
1
0
−1
−2
0
24
HCT15
cis−vaccenate (18:1n7)
1.0
0.5
0.0
−0.5
−1.5
0
24
SKOV3

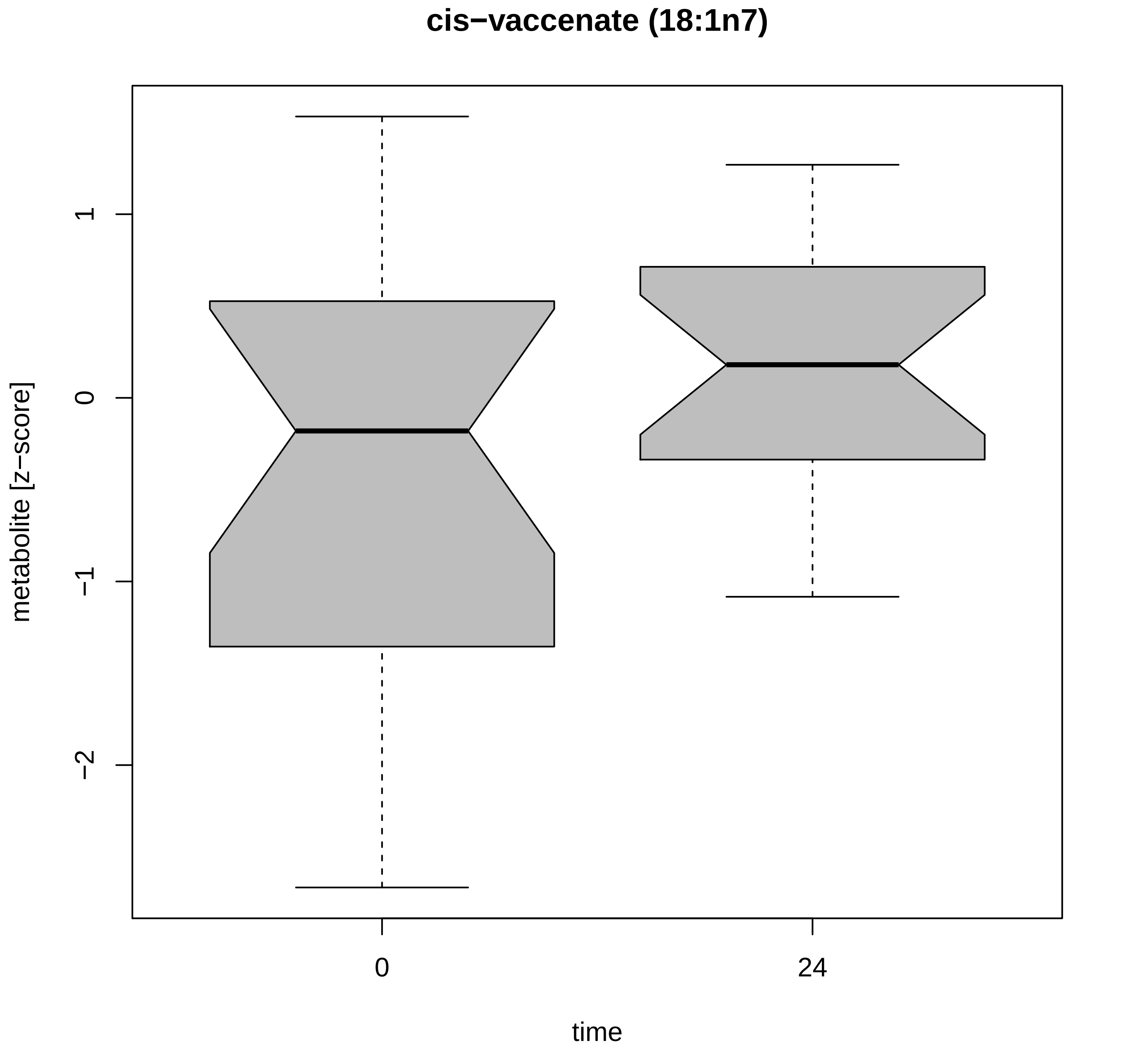
cis−vaccenate (18:1n7)
metabolite [z−score]
1
0
−1
−2
0
24
time

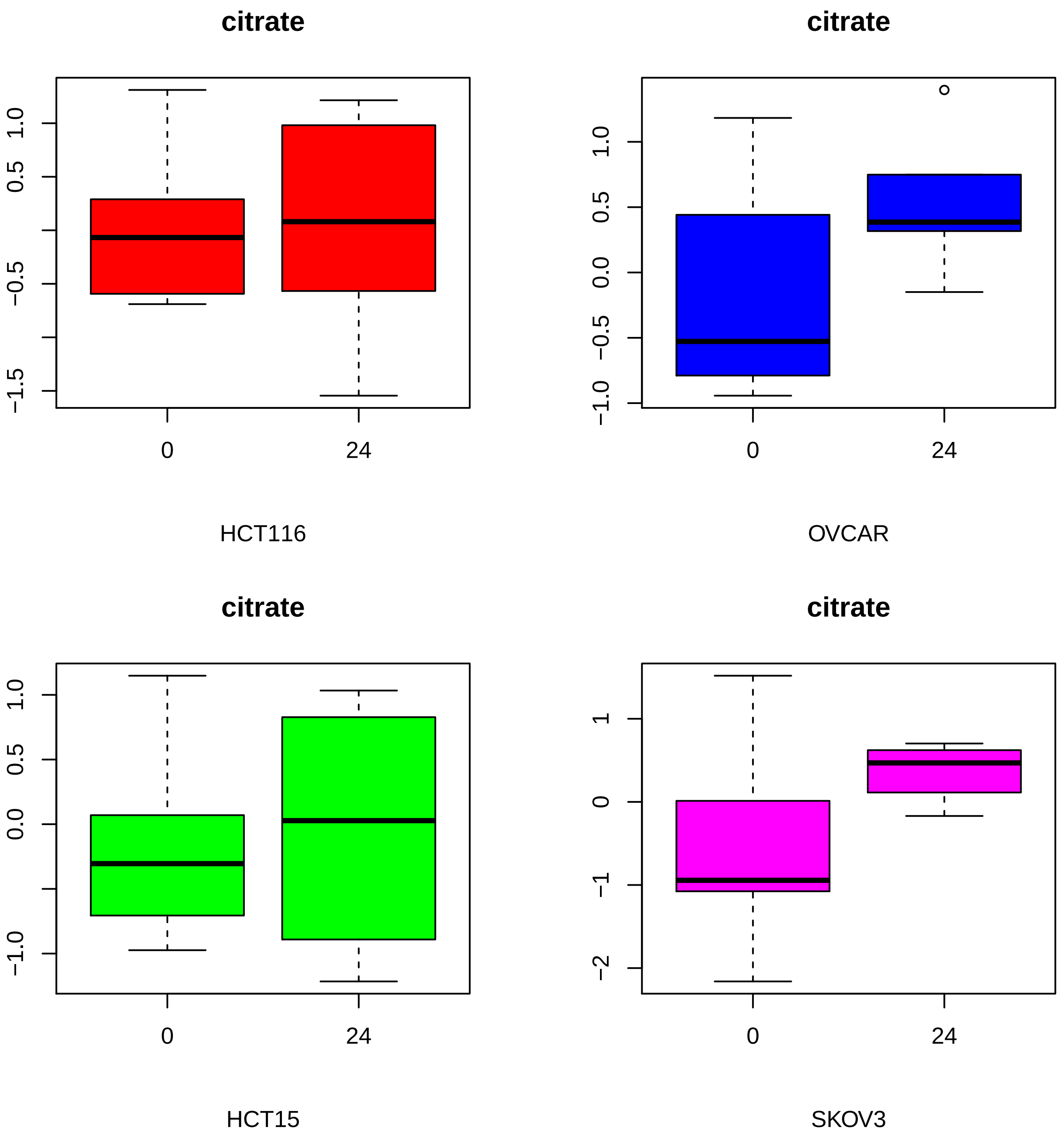
citrate
1.0
0.5
−0.5
−1.5
0
24
HCT116
citrate
1.0
0.5
0.0
−0.5
−1.0
0
24
OVCAR
citrate
1.0
0.5
0.0
−1.0
0
24
HCT15
citrate
1
0
−1
−2
0
24
SKOV3

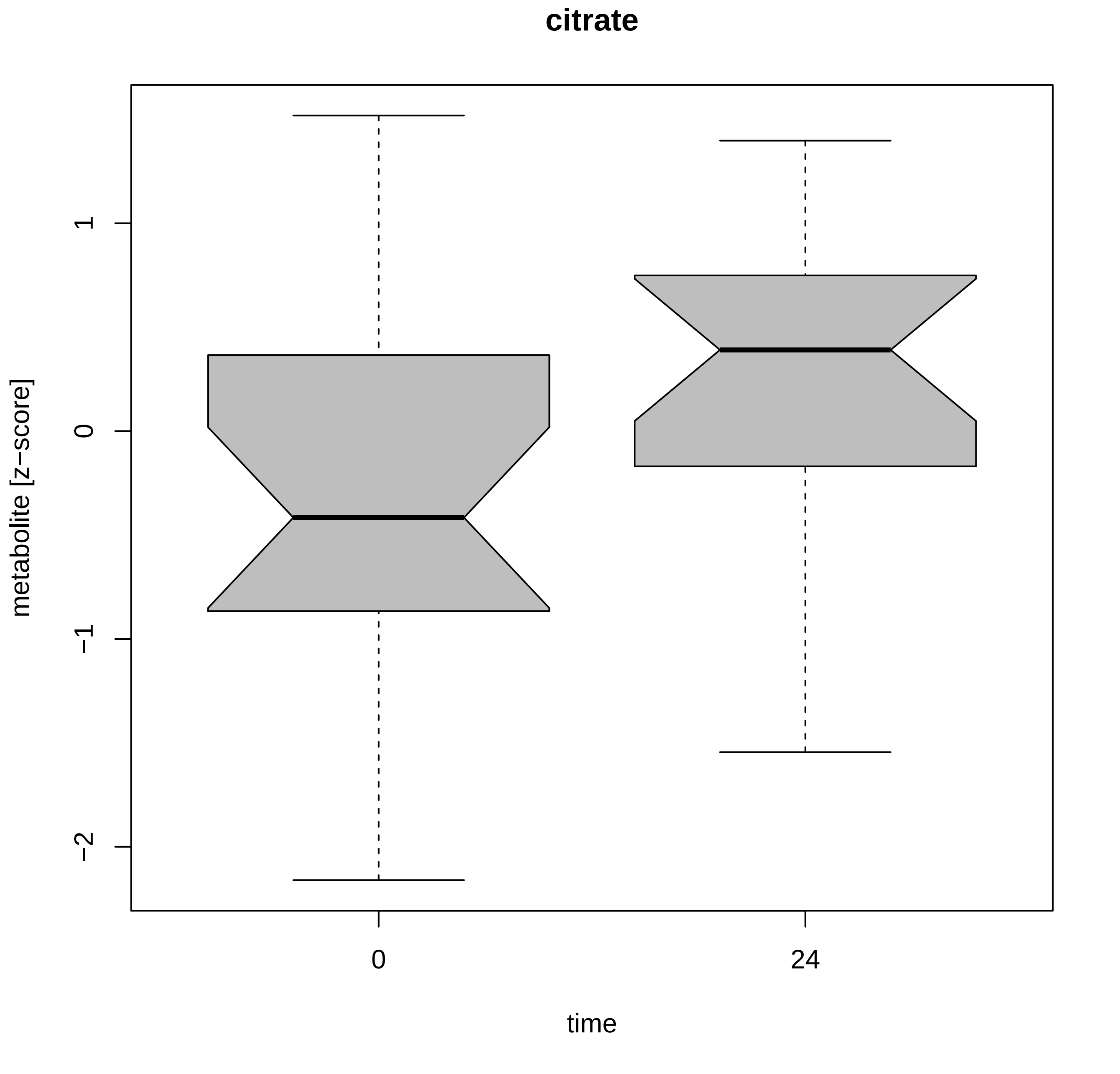

citrate
metabolite [z−score]
1
0
−1
−2
0
24
time

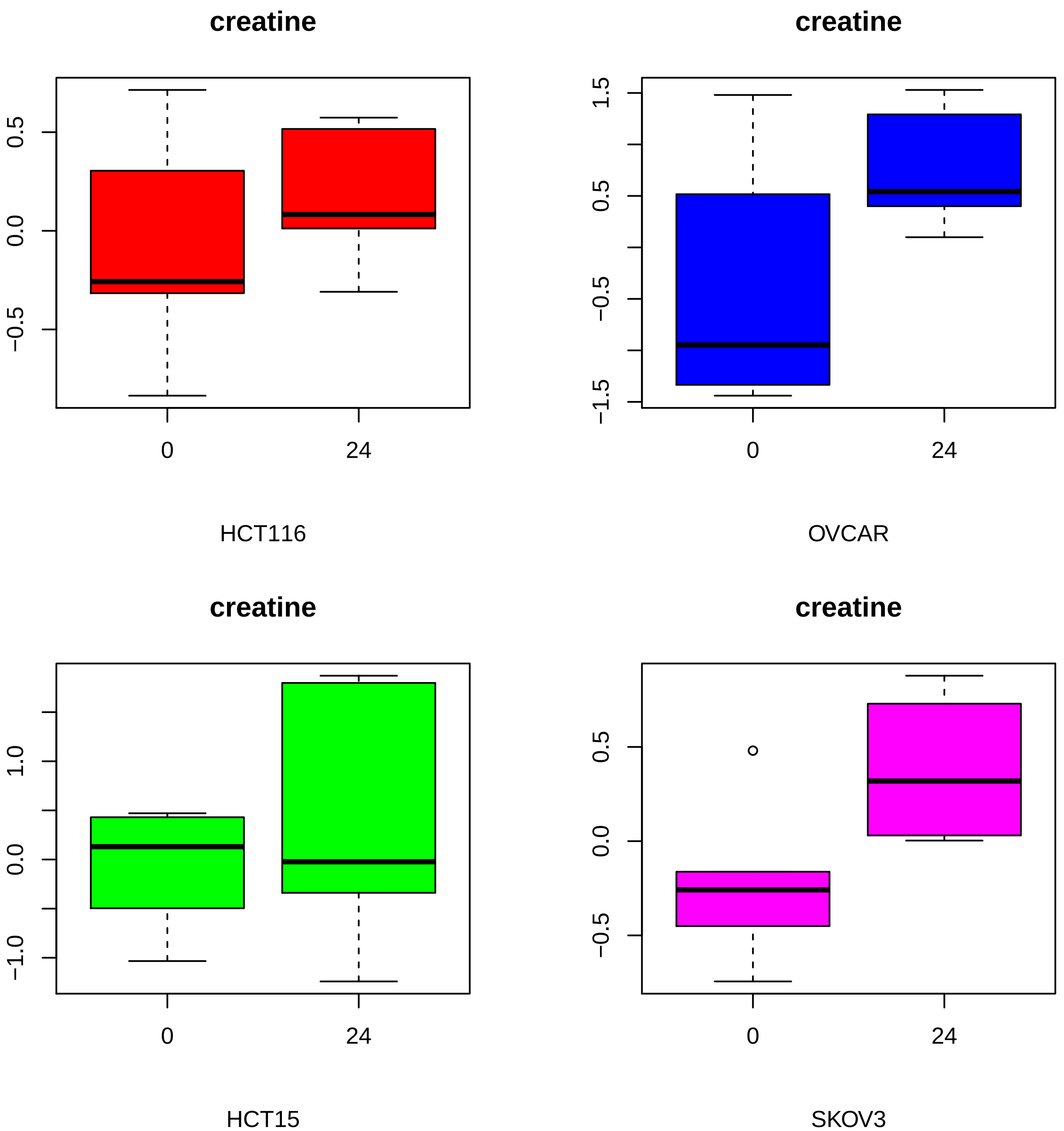
creatine
HCT116
creatine
OVCAR
creatine
HCT15
creatine
SKOV3
0
24
0.5
0.0
−0.5
1.5
−1.5
1.0
−1.0

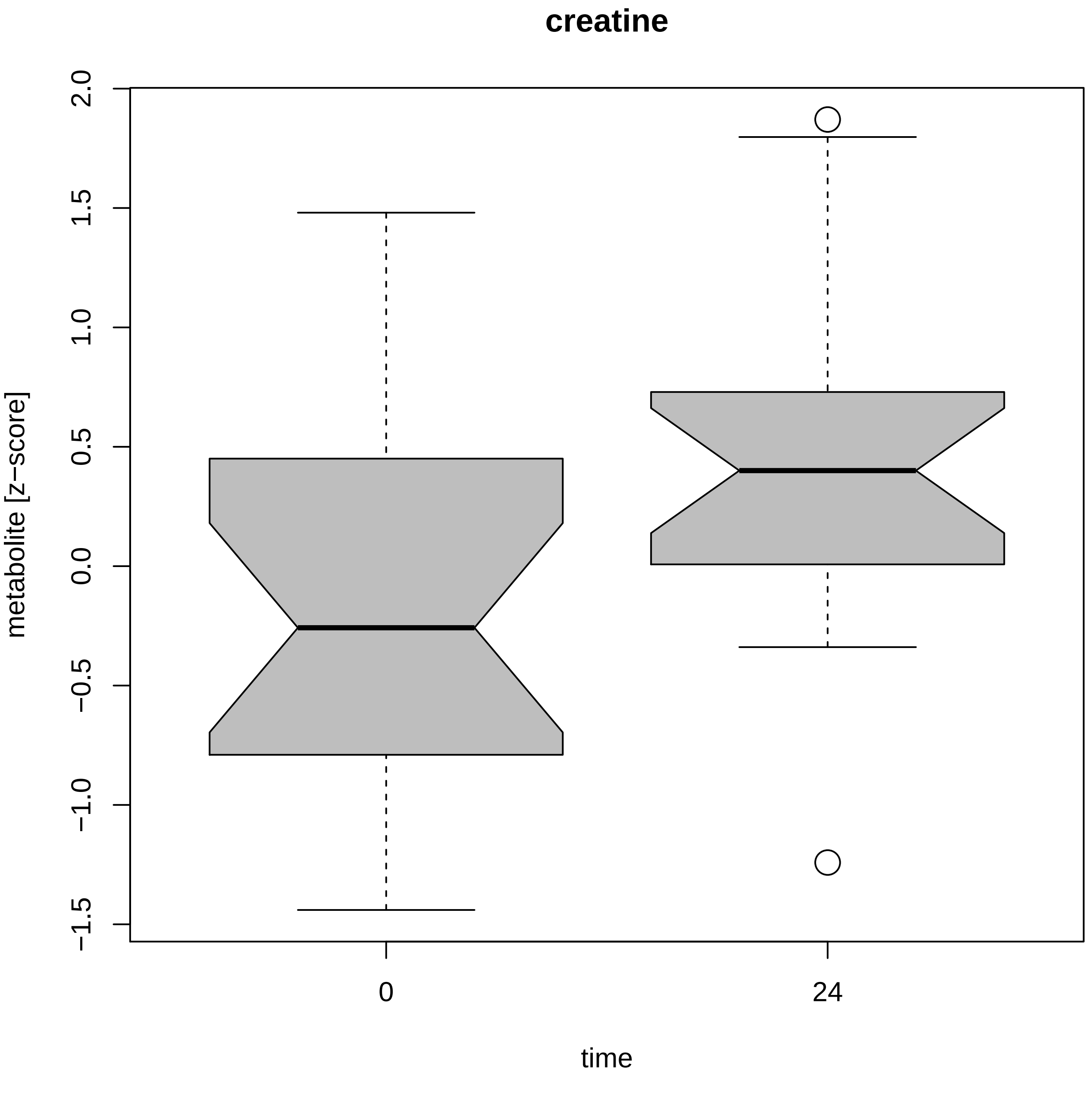
creatine
metabolite [z−score]
2.0
1.5
1.0
0.5
0.0
−0.5
−1.0
−1.5
0
24
time

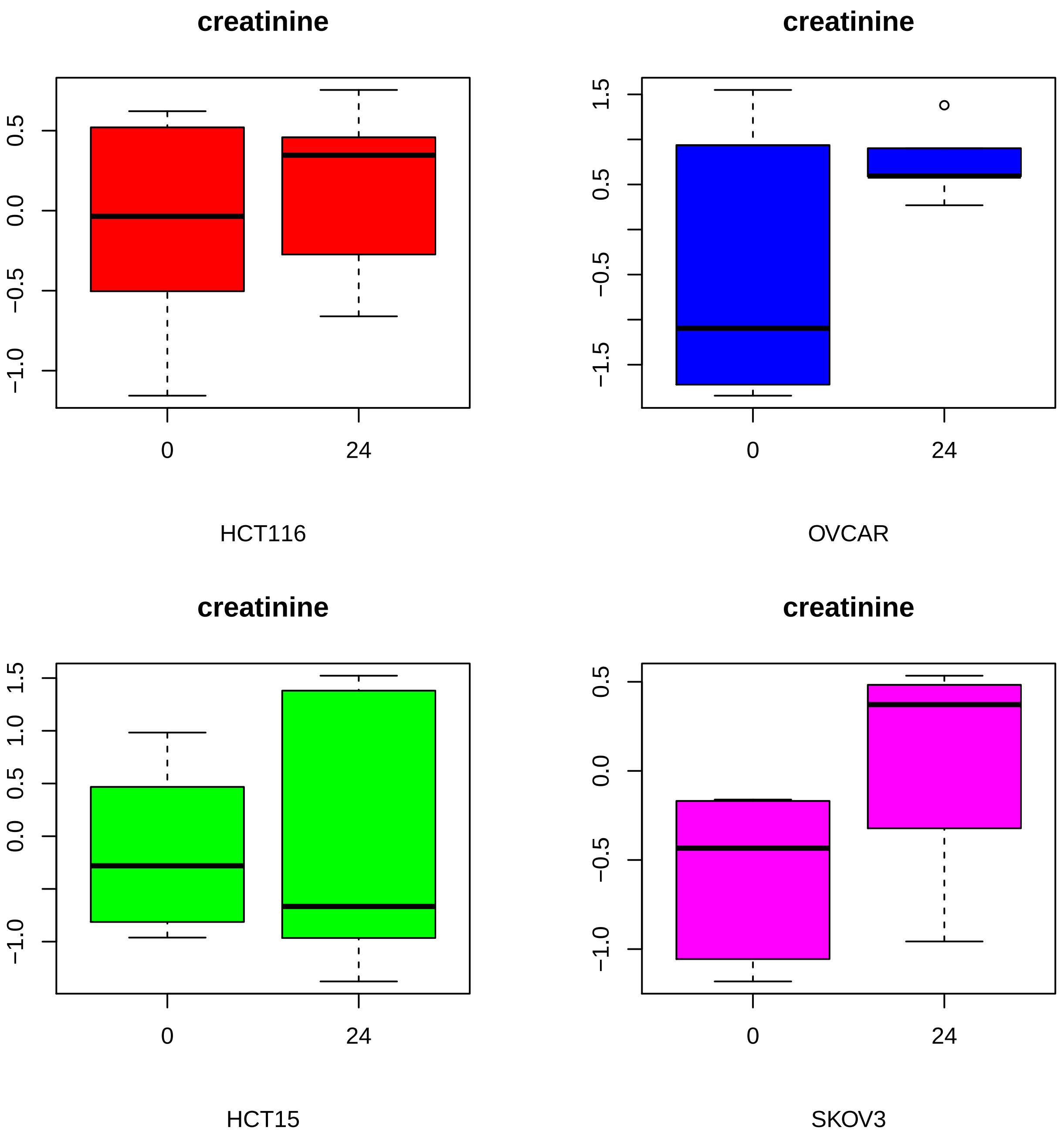
creatinine
0.5
0.0
−0.5
−1.0
0
24
HCT116
creatinine
1.5
0.5
−0.5
−1.5
0
24
OVCAR
creatinine
1.5
1.0
0.5
0.0
−1.0
0
24
HCT15
creatinine
0.5
0.0
−0.5
−1.0
0
24
SKOV3

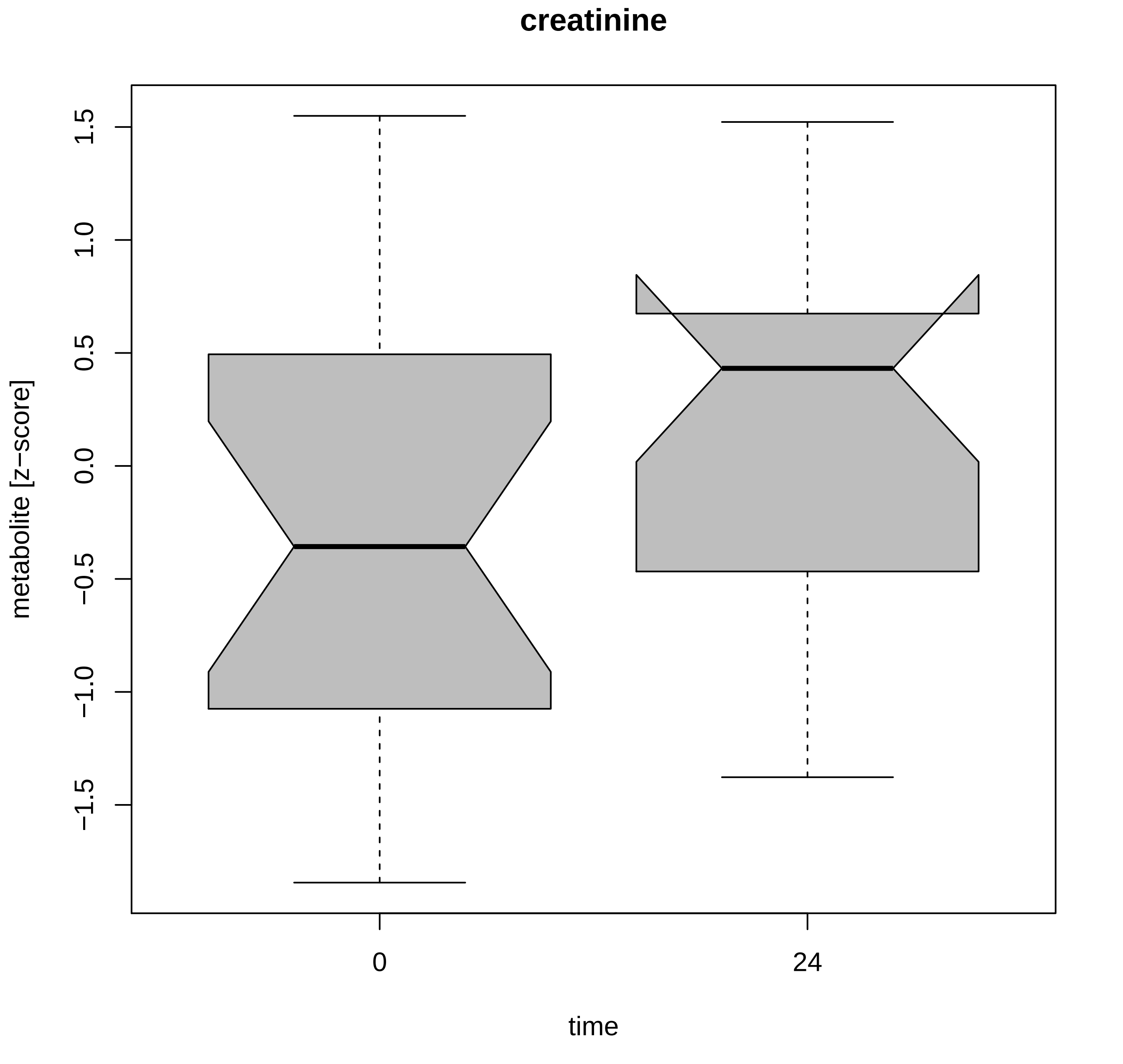
creatinine
metabolite [z−score]
1.5
1.0
0.5
0.0
−0.5
−1.0
−1.5
0
24
time

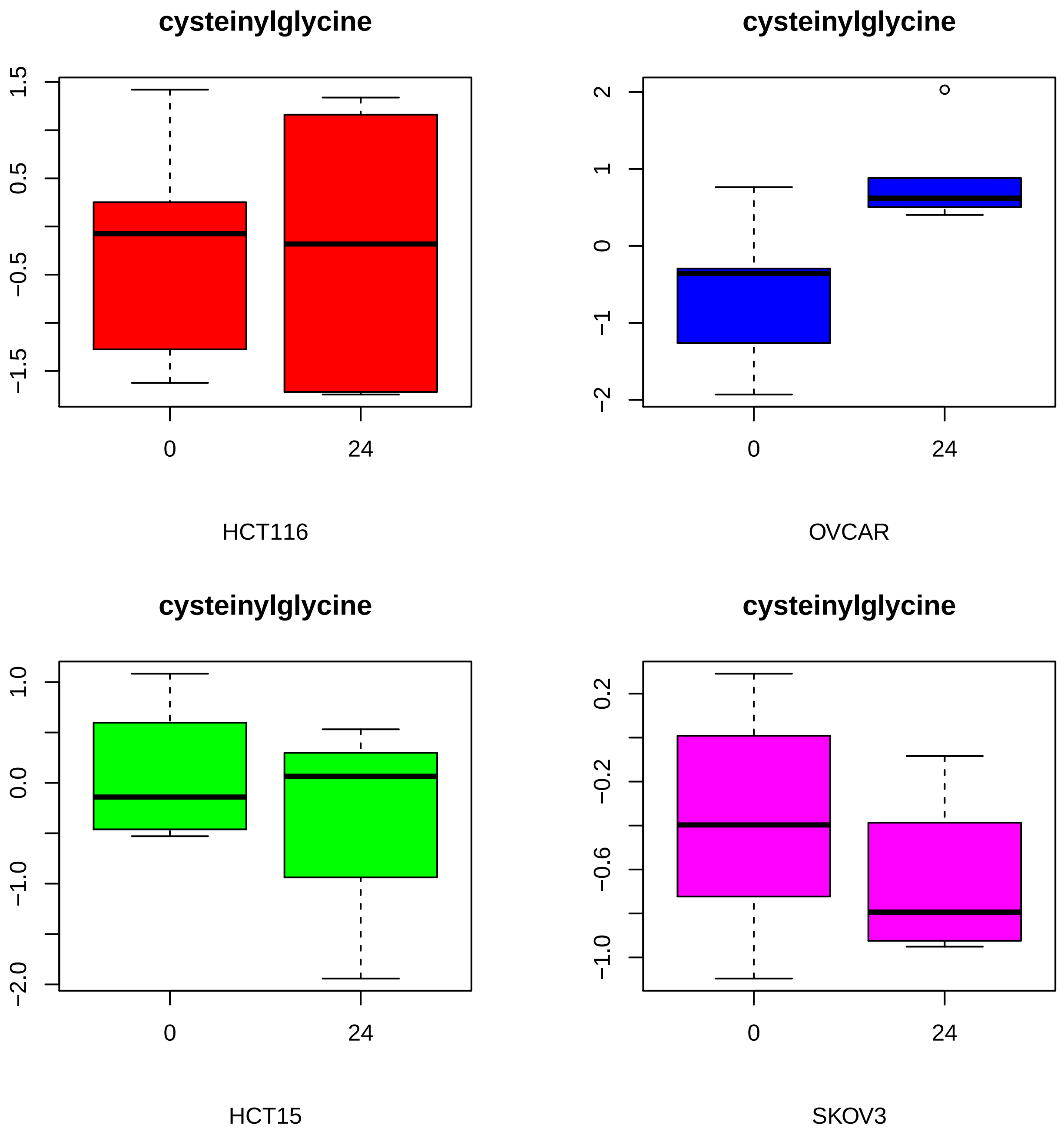
cysteinylglycine
1.5
0.5
−0.5
−1.5
0
24
HCT116
cysteinylglycine
2
1
0
−1
−2
0
24
OVCAR
cysteinylglycine
1.0
0.0
−1.0
−2.0
0
24
HCT15
cysteinylglycine
0.2
−0.2
−0.6
−1.0
0
24
SKOV3

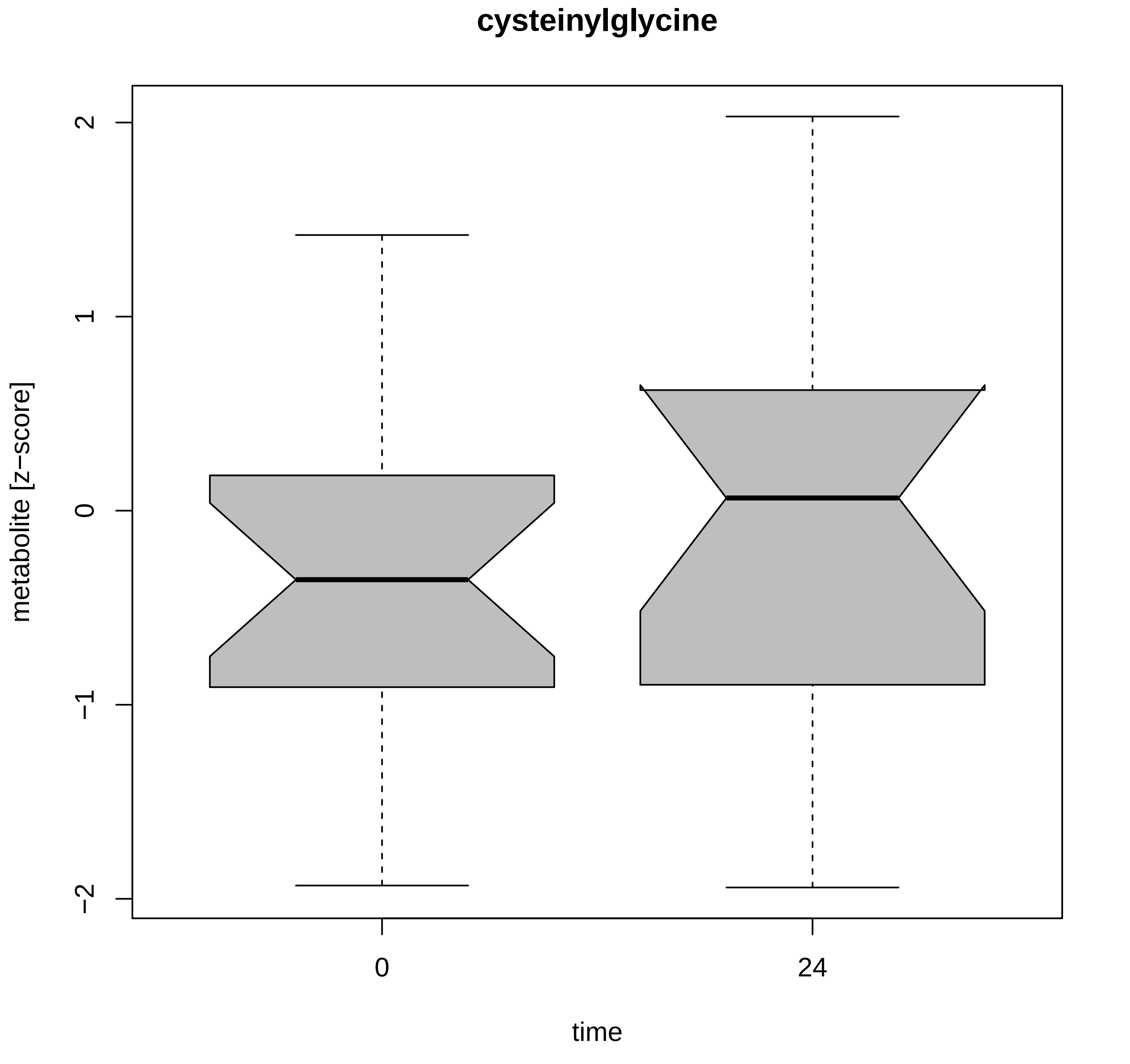
cysteinylglycine
metabolite [z−score]
2
1
0
−1
−2
0
24
time

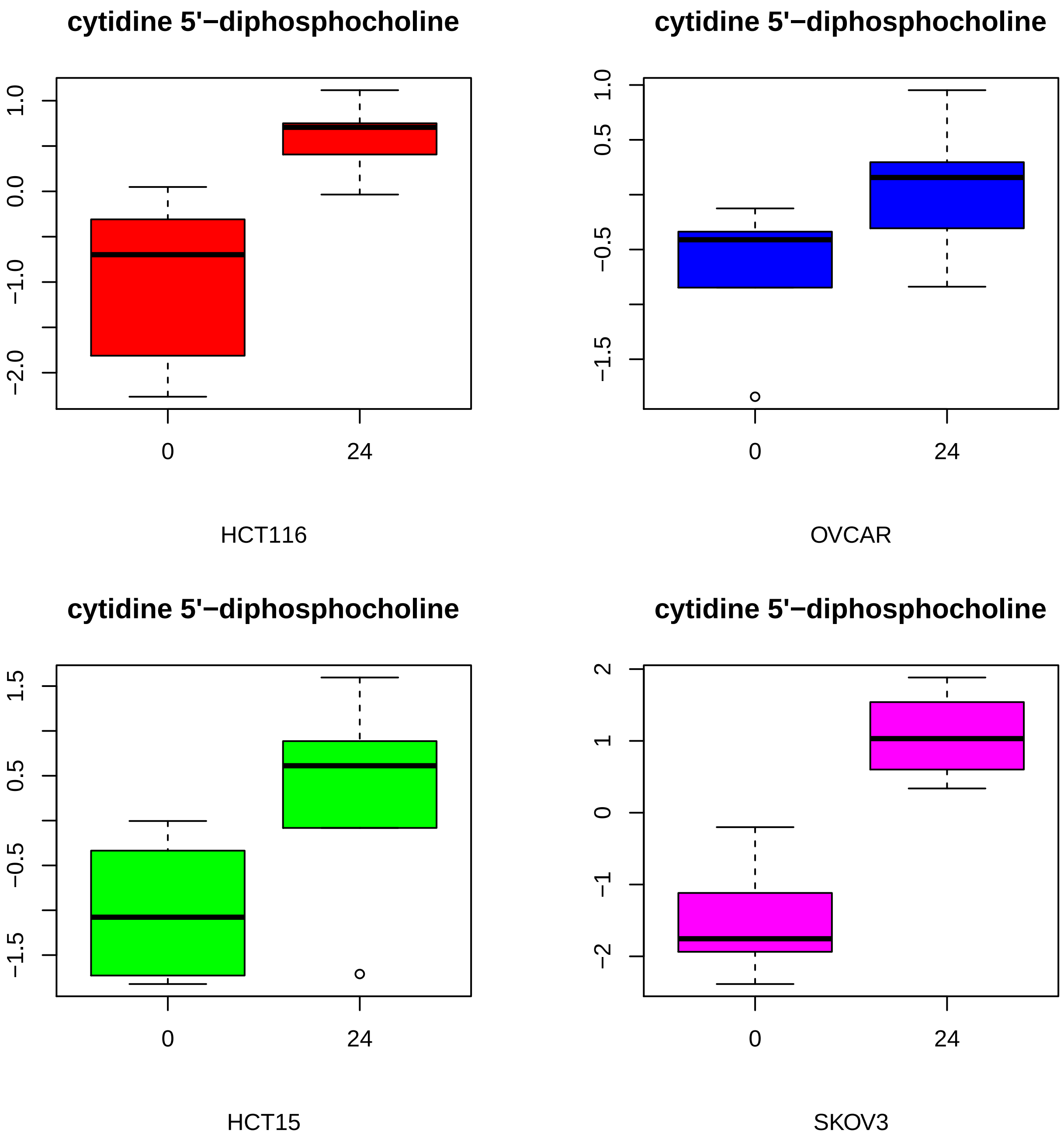
cytidine 5'−diphosphocholine
1.0
0.0
−1.0
−2.0
0
24
HCT116
cytidine 5'−diphosphocholine
1.0
0.5
−0.5
−1.5
0
24
OVCAR
cytidine 5'−diphosphocholine
1.5
0.5
−0.5
−1.5
0
24
HCT15
cytidine 5'−diphosphocholine
2
1
0
−1
−2
0
24
SKOV3

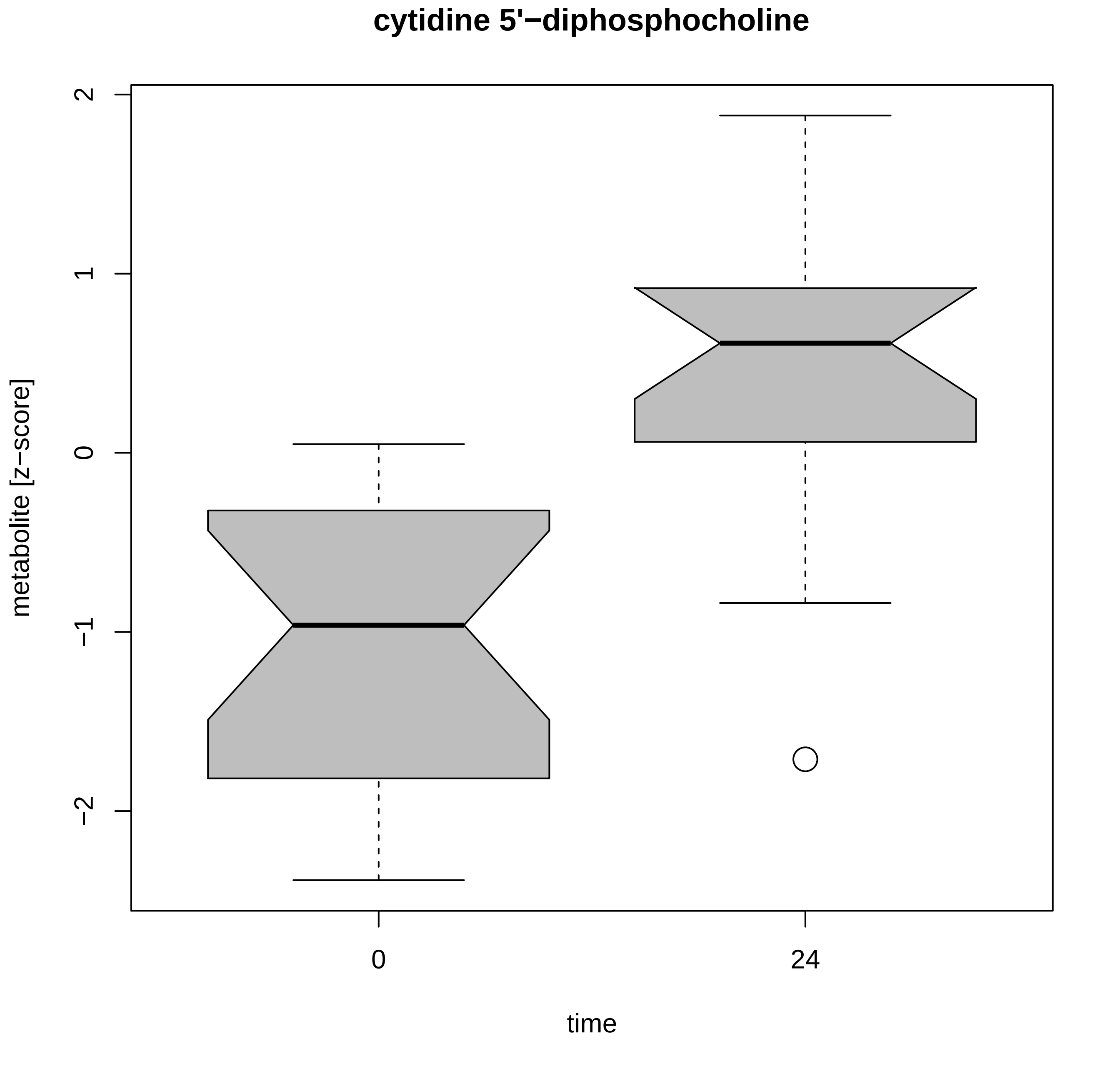

cytidine 5'−diphosphocholine
metabolite [z−score]
2
1
0
−1
−2
0
24
time

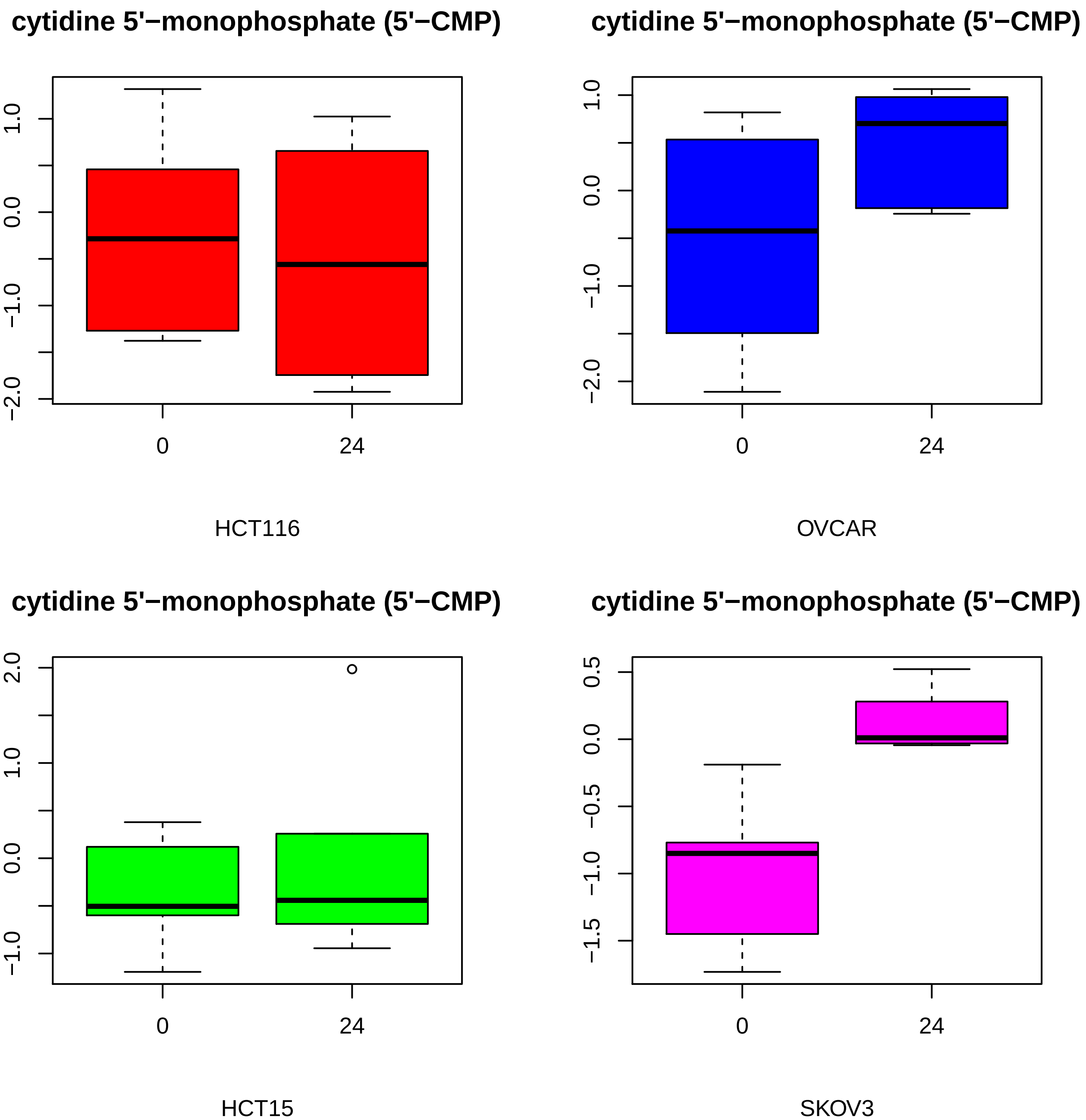
cytidine 5'−monophosphate (5'−CMP)
HCT116
0
24
1.0
0.0
−1.0
−2.0
cytidine 5'−monophosphate (5'−CMP)
OVCAR
0
24
1.0
0.0
−1.0
−2.0
cytidine 5'−monophosphate (5'−CMP)
HCT15
0
24
2.0
1.0
0.0
−1.0
cytidine 5'−monophosphate (5'−CMP)
SKOV3
0
24
0.5
0.0
−0.5
−1.0
−1.5

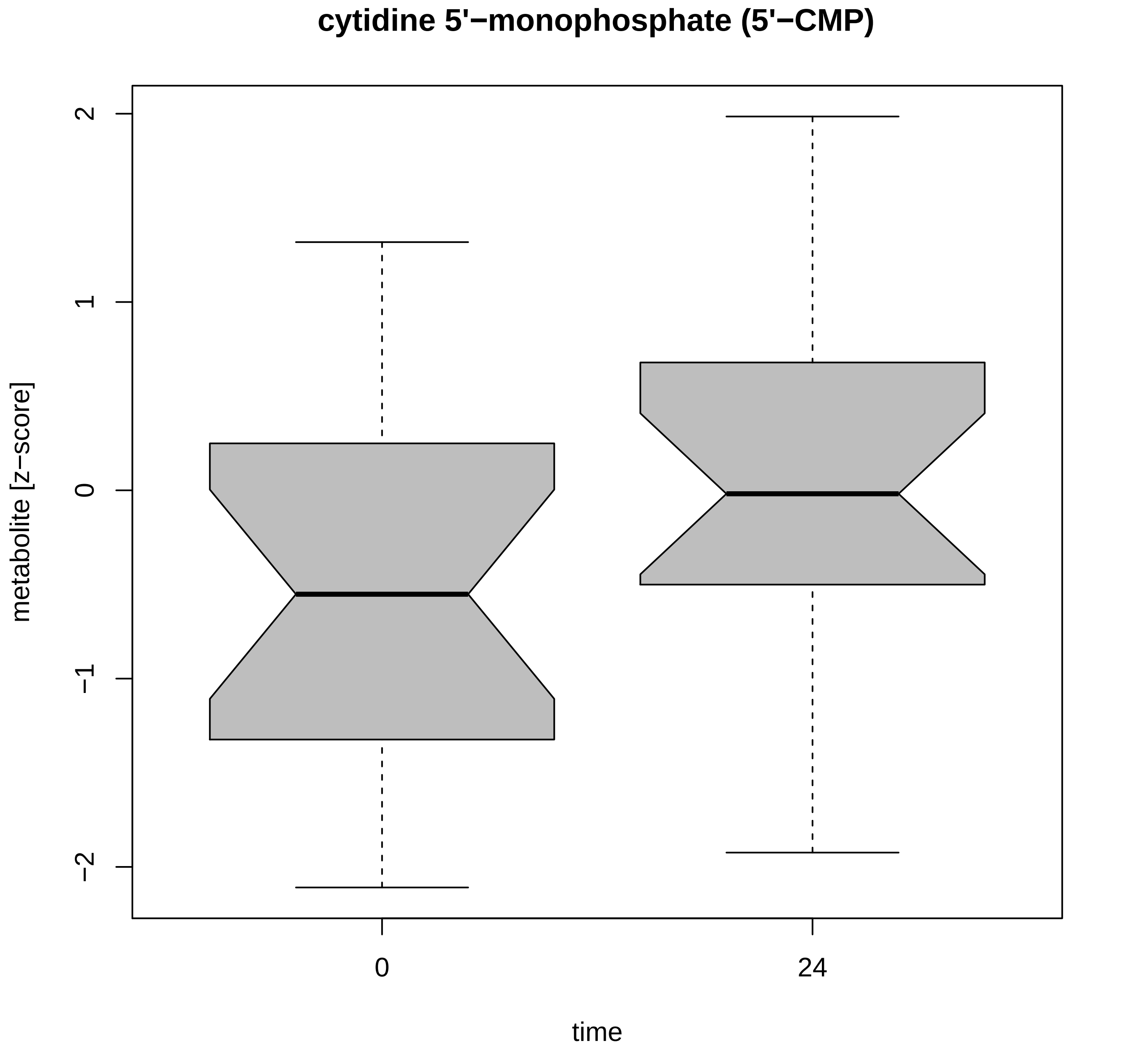
cytidine 5'−monophosphate (5'−CMP)
metabolite [z−score]
2
1
0
−1
−2
0
24
time

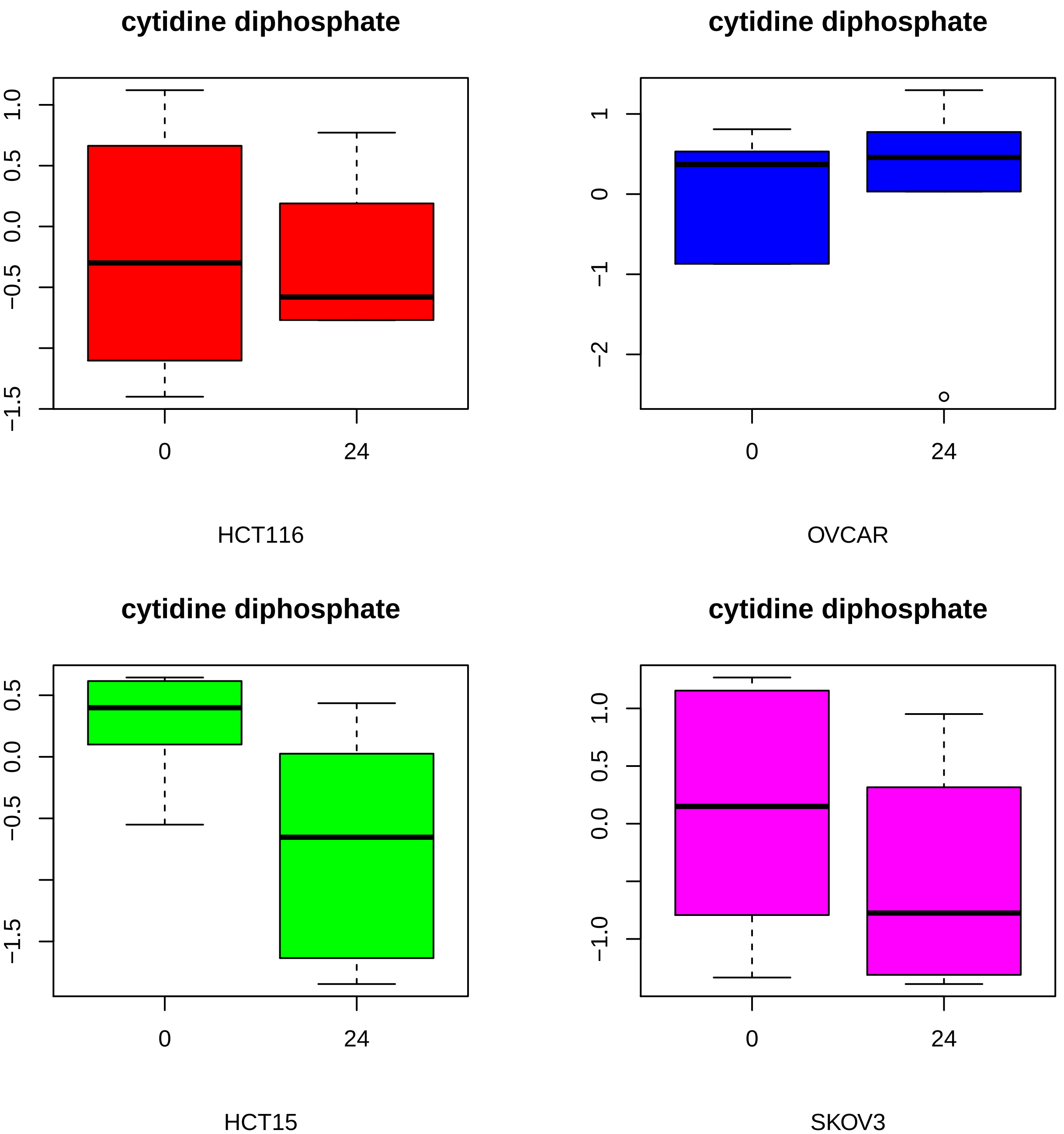
cytidine diphosphate
1.0
0.5
0.0
−0.5
−1.5
0
24
HCT116
cytidine diphosphate
1
0
−1
−2
0
24
OVCAR
cytidine diphosphate
0.5
0.0
−0.5
−1.5
0
24
HCT15
cytidine diphosphate
1.0
0.5
0.0
−1.0
0
24
SKOV3

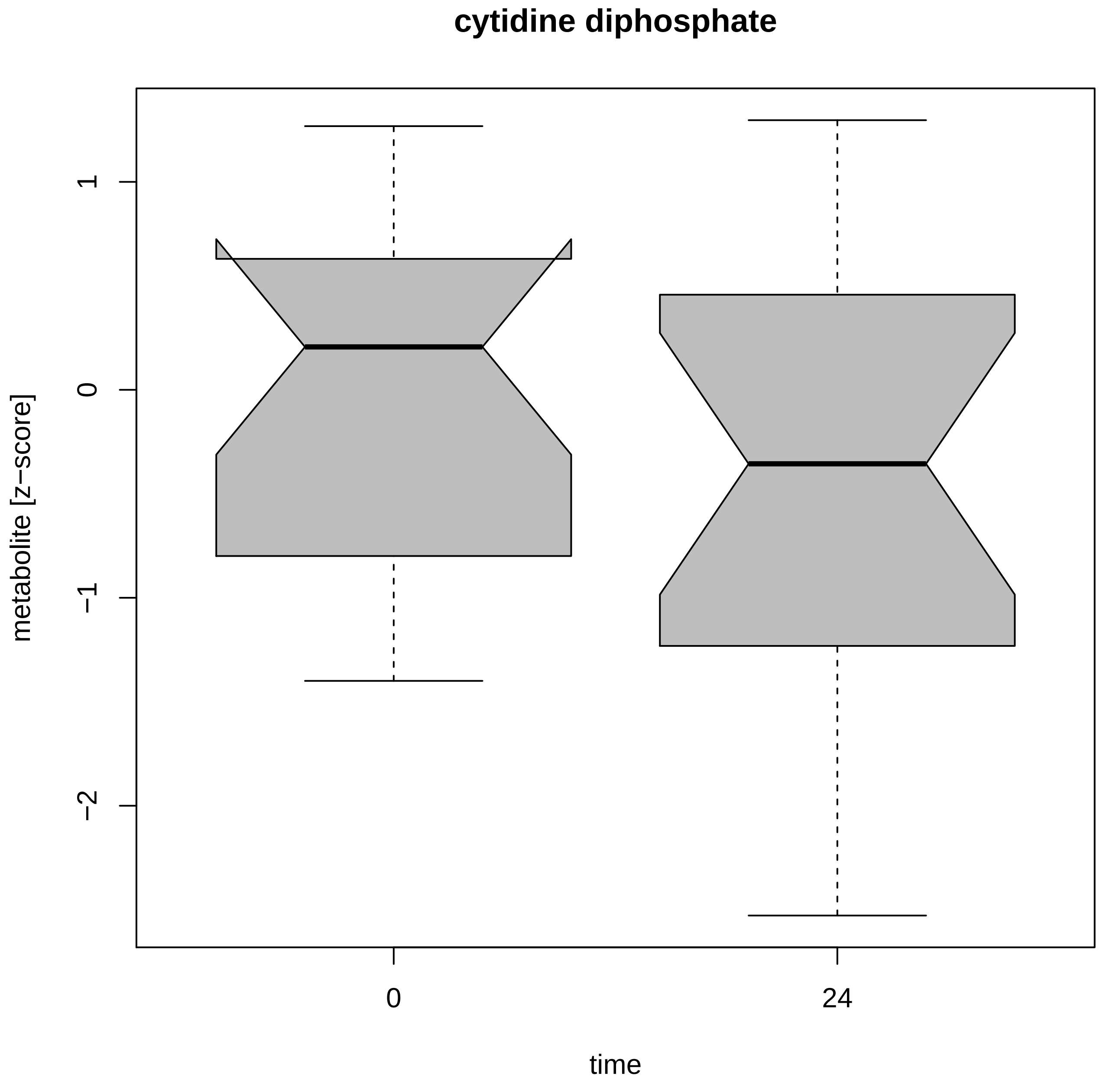

cytidine diphosphate
metabolite [z−score]
1
0
−1
−2
0
24
time

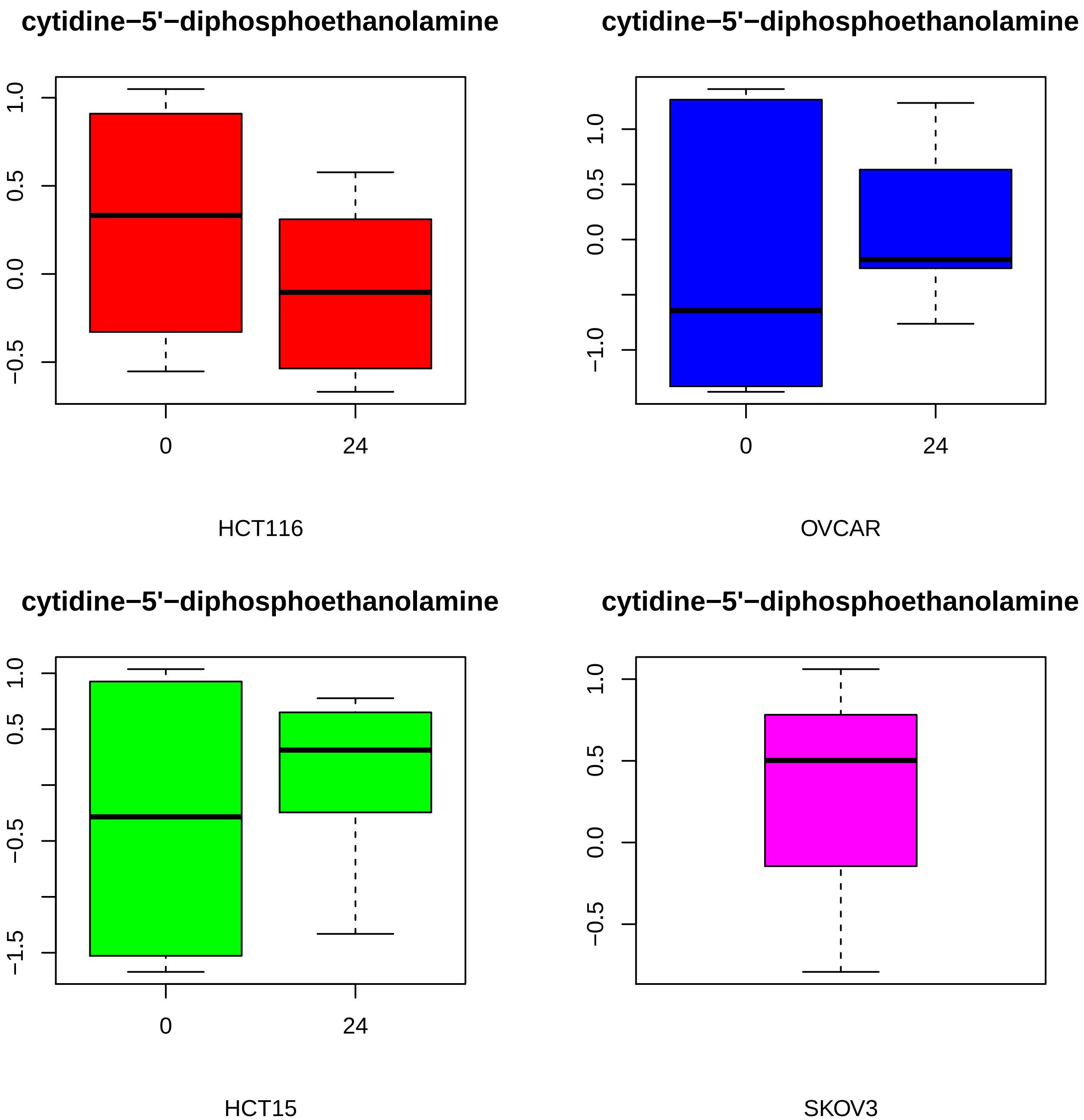
cytidine−5'−diphosphoethanolamine
1.0
0.5
0.0
−0.5
0
24
HCT116
cytidine−5'−diphosphoethanolamine
1.0
0.5
0.0
−1.0
0
24
OVCAR
cytidine−5'−diphosphoethanolamine
1.0
0.5
−0.5
−1.5
0
24
HCT15
cytidine−5'−diphosphoethanolamine
1.0
0.5
0.0
−0.5
SKOV3

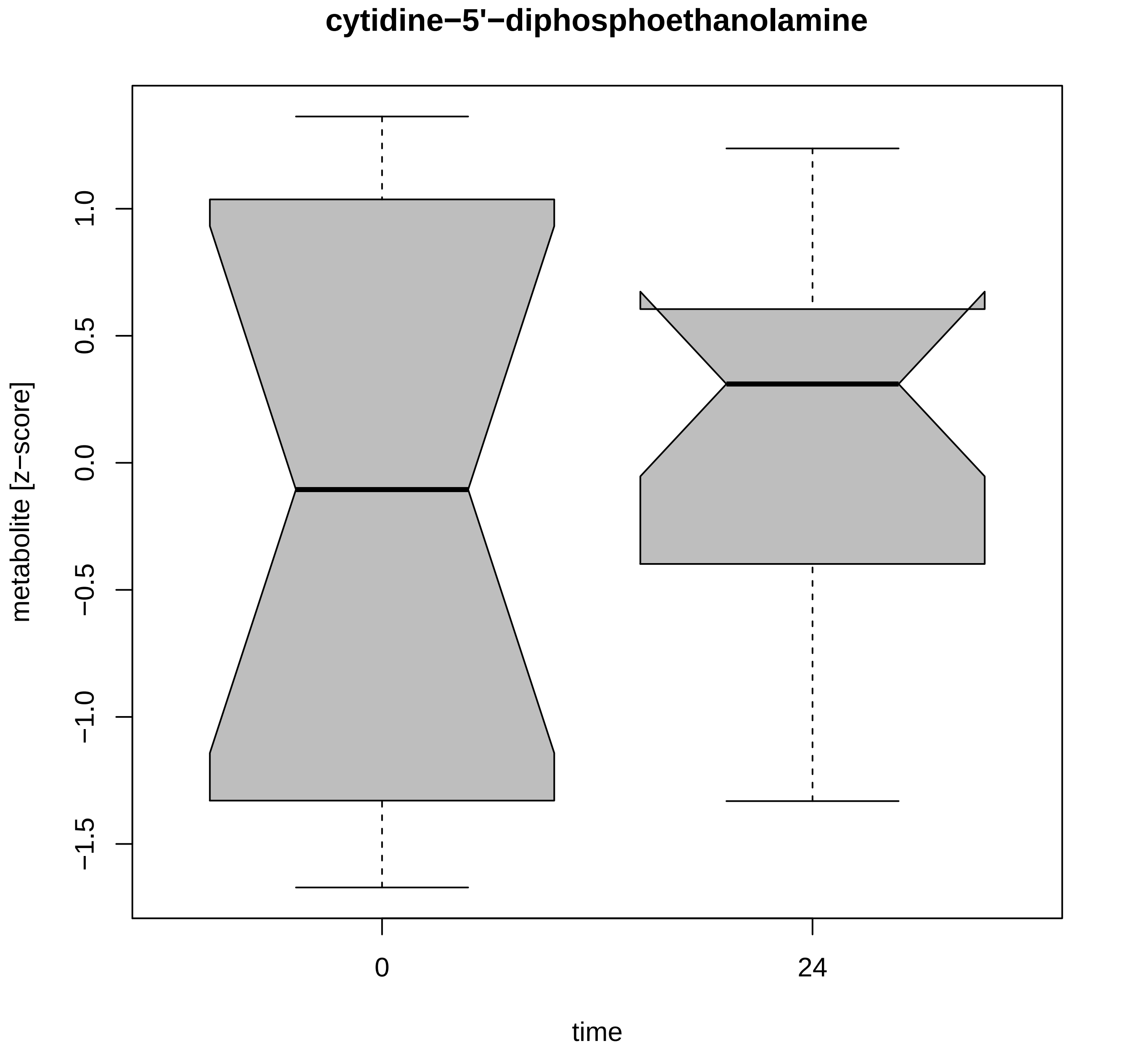

cytidine−5'−diphosphoethanolamine
metabolite [z−score]
1.0
0.5
0.0
−0.5
−1.0
−1.5
0
24
time

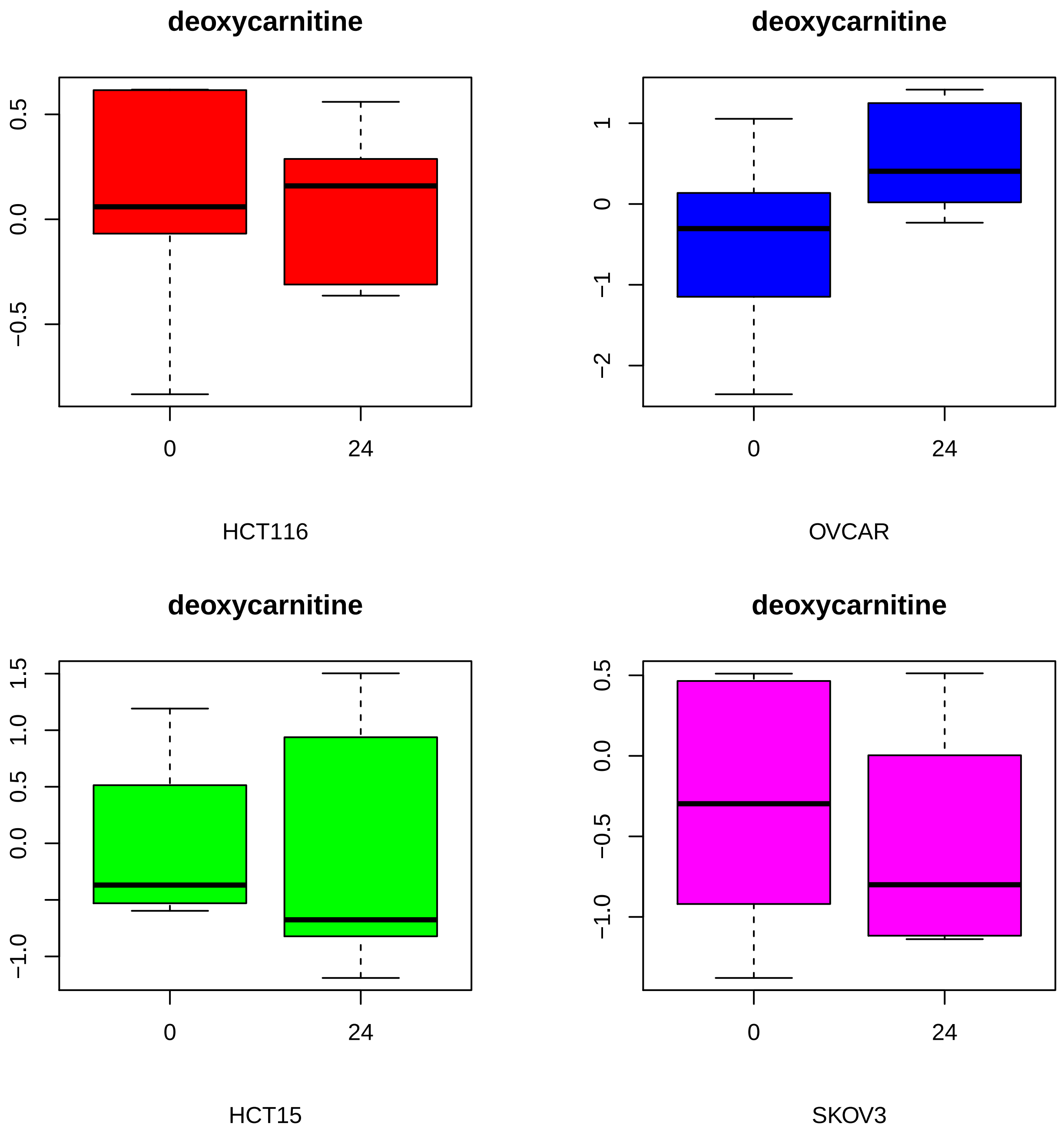
deoxycarnitine
0.5
0.0
−0.5
0
24
HCT116
deoxycarnitine
1
0
−1
−2
0
24
OVCAR
deoxycarnitine
1.5
1.0
0.5
0.0
−1.0
0
24
HCT15
deoxycarnitine
0.5
0.0
−0.5
−1.0
0
24
SKOV3

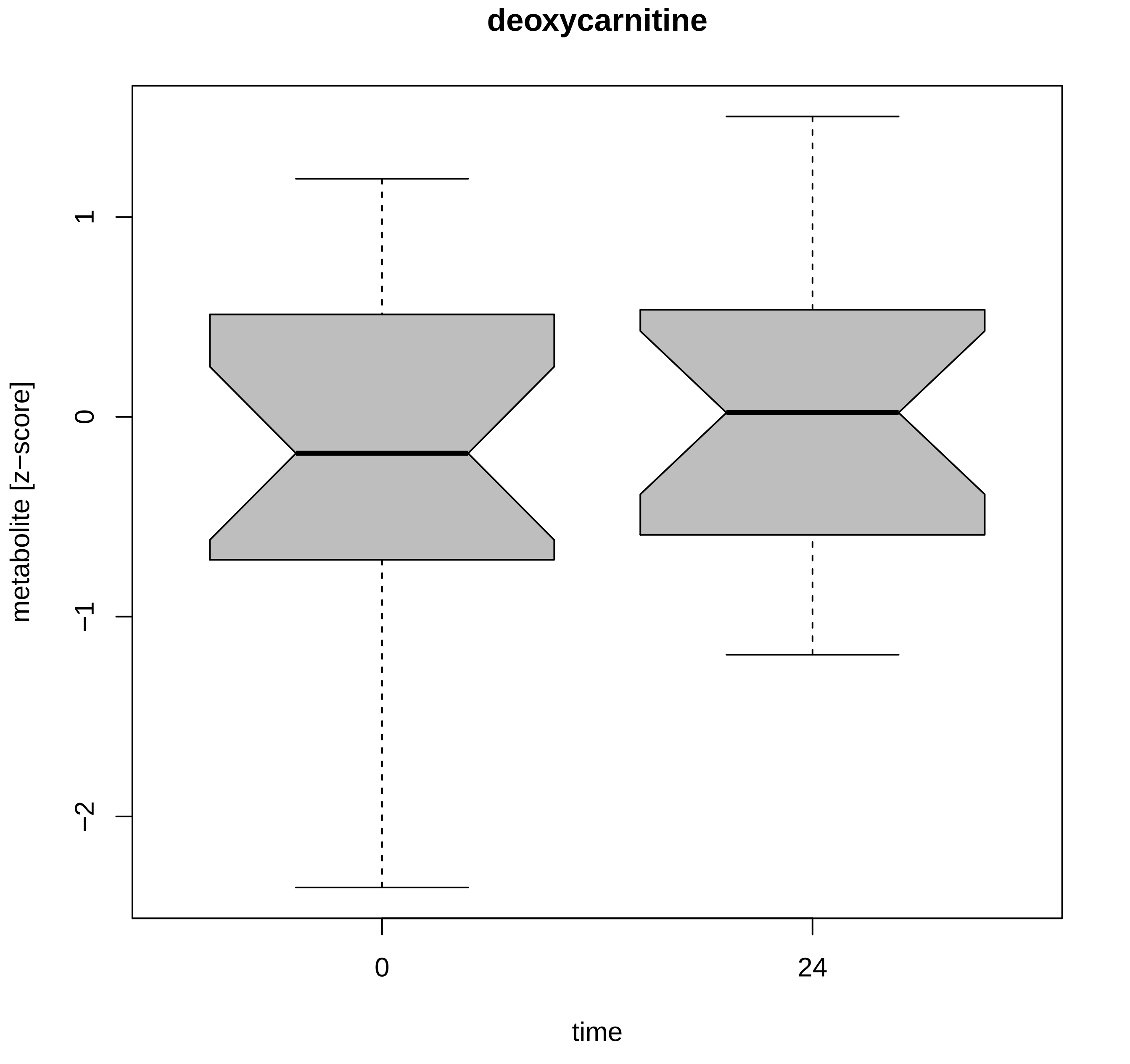
deoxycarnitine
metabolite [z−score]
1
0
−1
−2
0
24
time

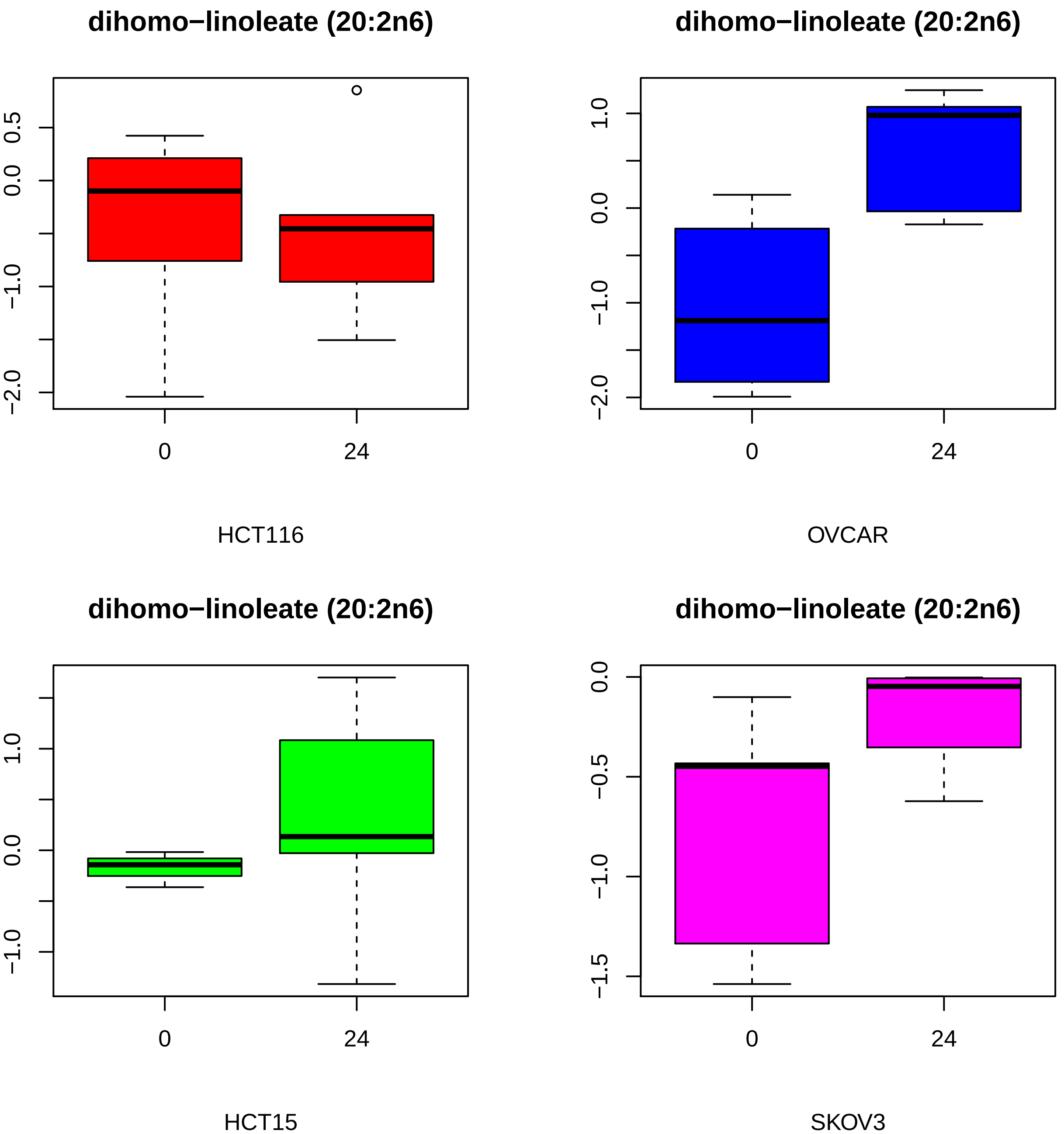
dihomo−linoleate (20:2n6)
0.5
0.0
−1.0
−2.0
0
24
HCT116
dihomo−linoleate (20:2n6)
1.0
0.0
−1.0
−2.0
0
24
OVCAR
dihomo−linoleate (20:2n6)
1.0
0.0
−1.0
0
24
HCT15
dihomo−linoleate (20:2n6)
0.0
−0.5
−1.0
−1.5
0
24
SKOV3

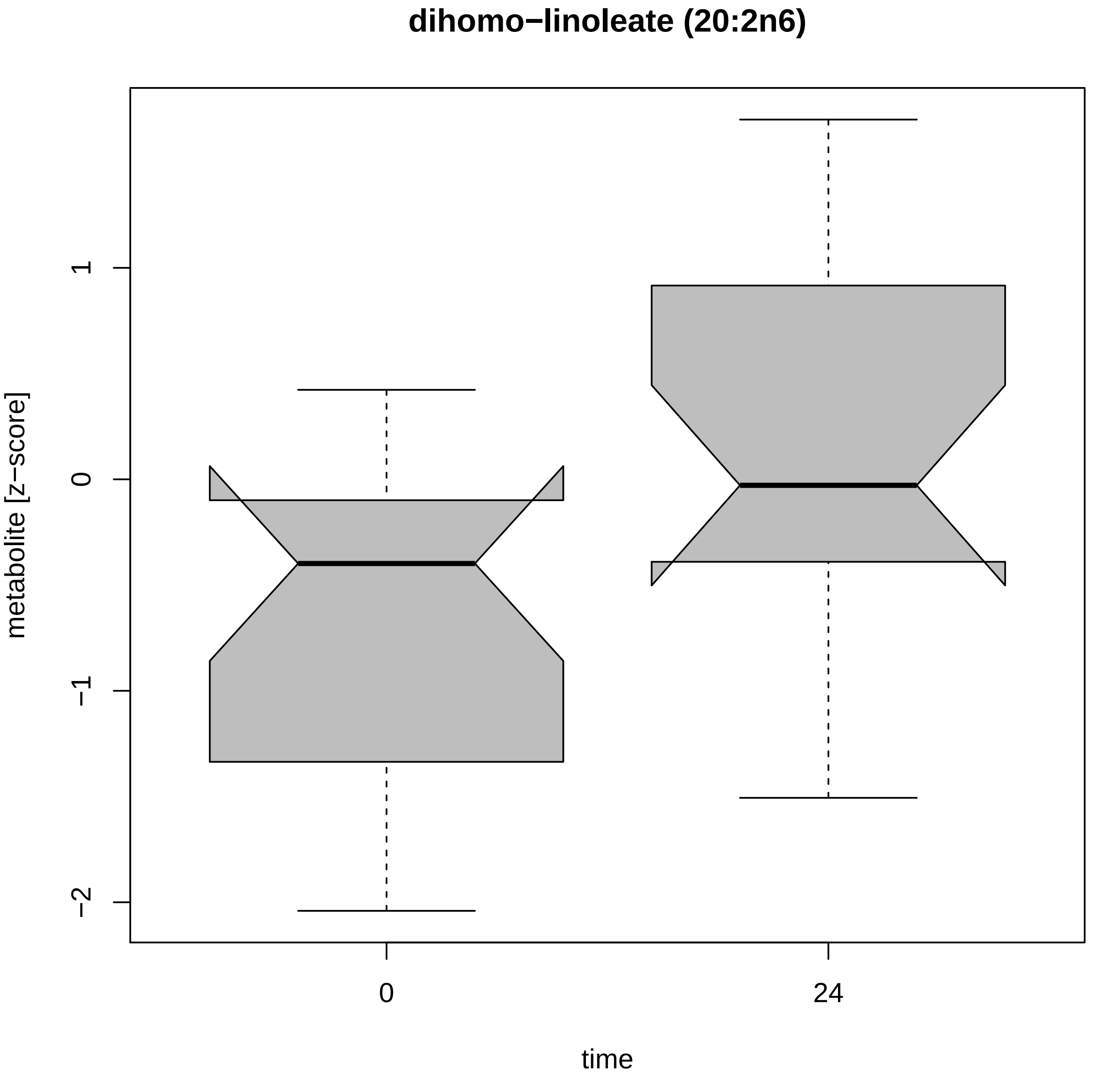
dihomo−linoleate (20:2n6)
metabolite [z−score]
1
0
−1
−2
0
24
time

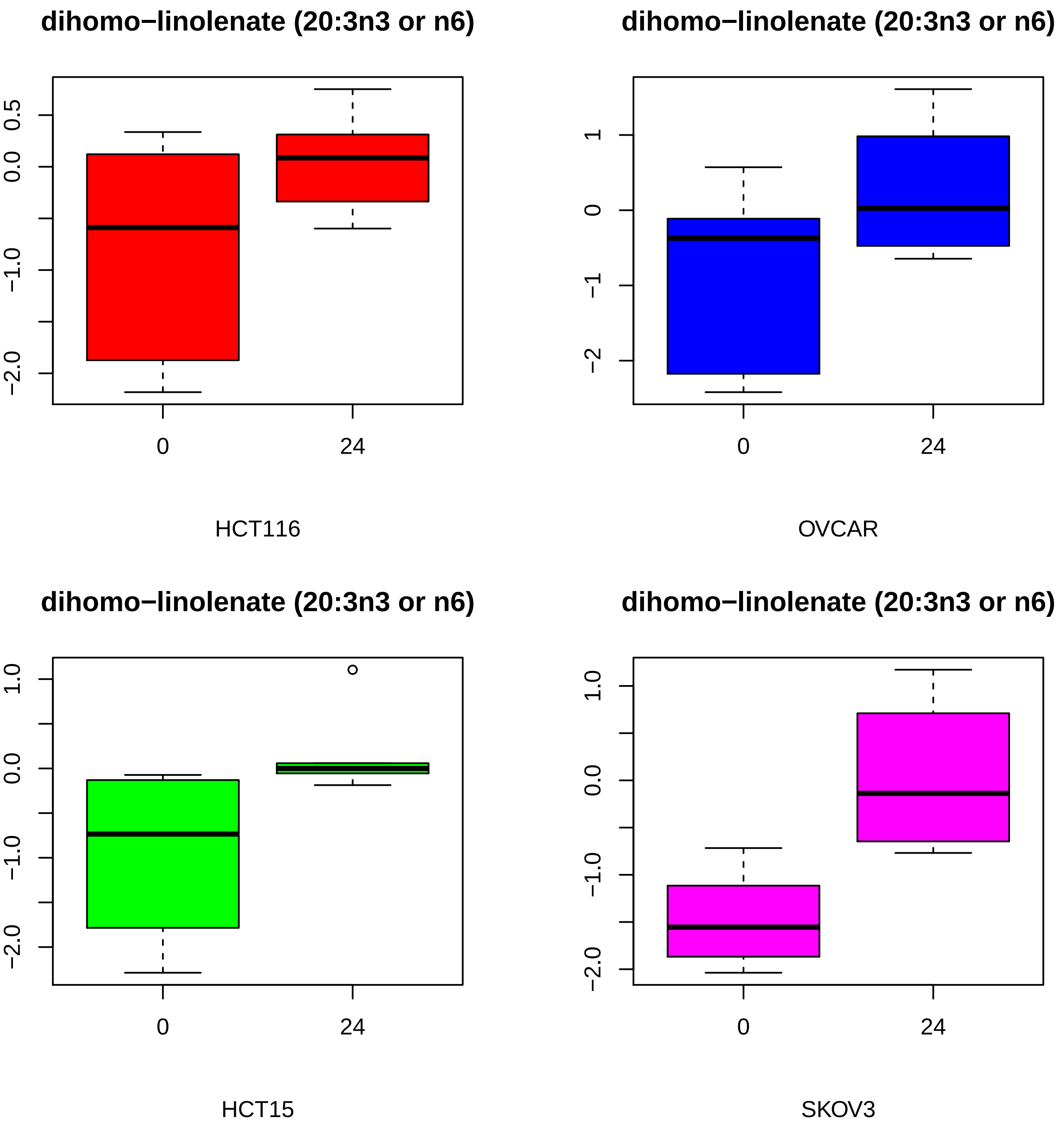
dihomo−linolenate (20:3n3 or n6)
0.5
0.0
−1.0
−2.0
0
24
HCT116
dihomo−linolenate (20:3n3 or n6)
1
0
−1
−2
0
24
OVCAR
dihomo−linolenate (20:3n3 or n6)
1.0
0.0
−1.0
−2.0
0
24
HCT15
dihomo−linolenate (20:3n3 or n6)
1.0
0.0
−1.0
−2.0
0
24
SKOV3

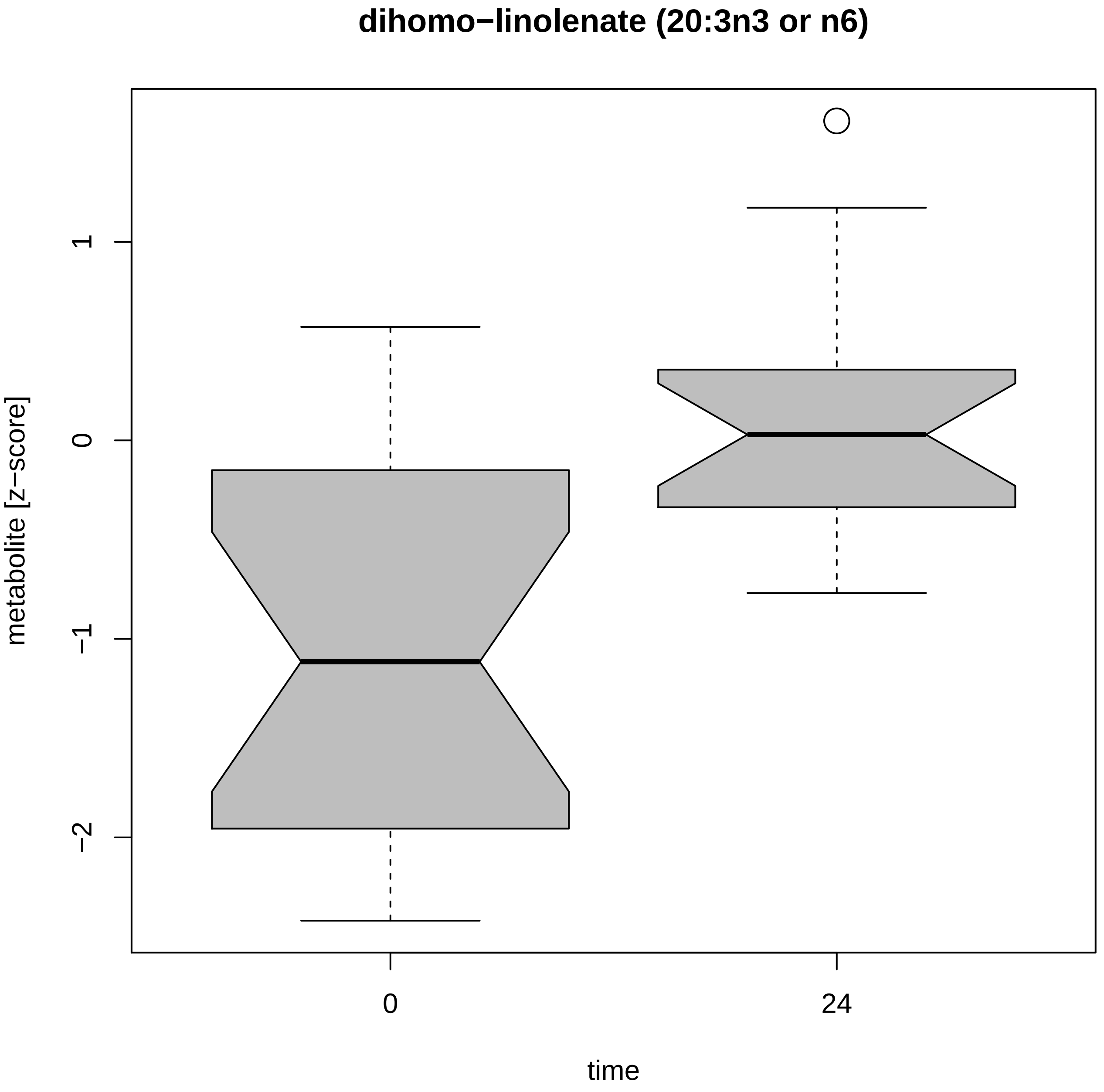

dihomo−linolenate (20:3n3 or n6)
metabolite [z−score]
1
0
−1
−2
0
24
time

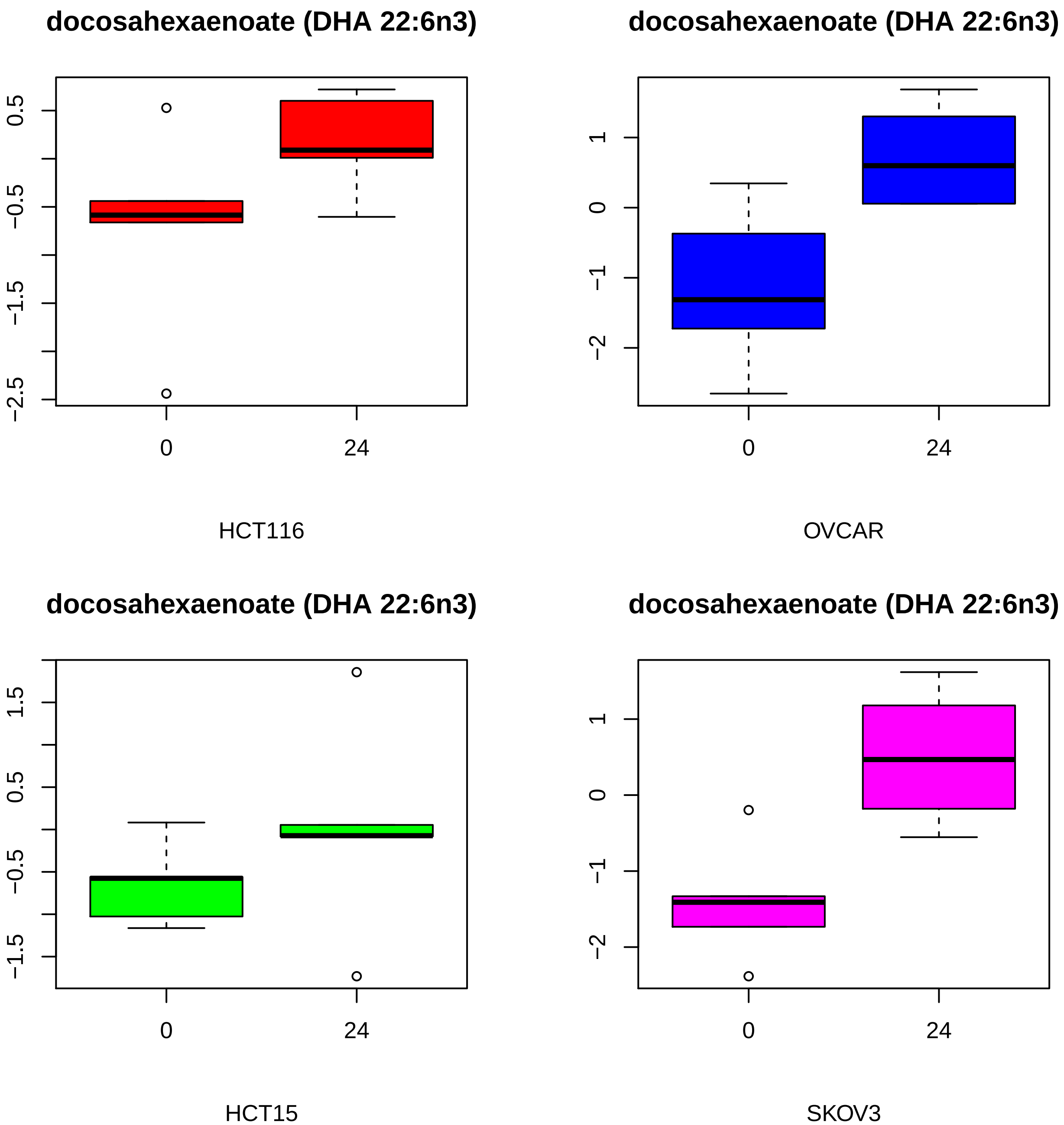
docosahexaenoate (DHA 22:6n3)
0.5
−0.5
−1.5
−2.5
0
24
HCT116
docosahexaenoate (DHA 22:6n3)
1
0
−1
−2
0
24
OVCAR
docosahexaenoate (DHA 22:6n3)
1.5
0.5
−0.5
−1.5
0
24
HCT15
docosahexaenoate (DHA 22:6n3)
1
0
−1
−2
0
24
SKOV3

docosahexaenoate (DHA 22:6n3)

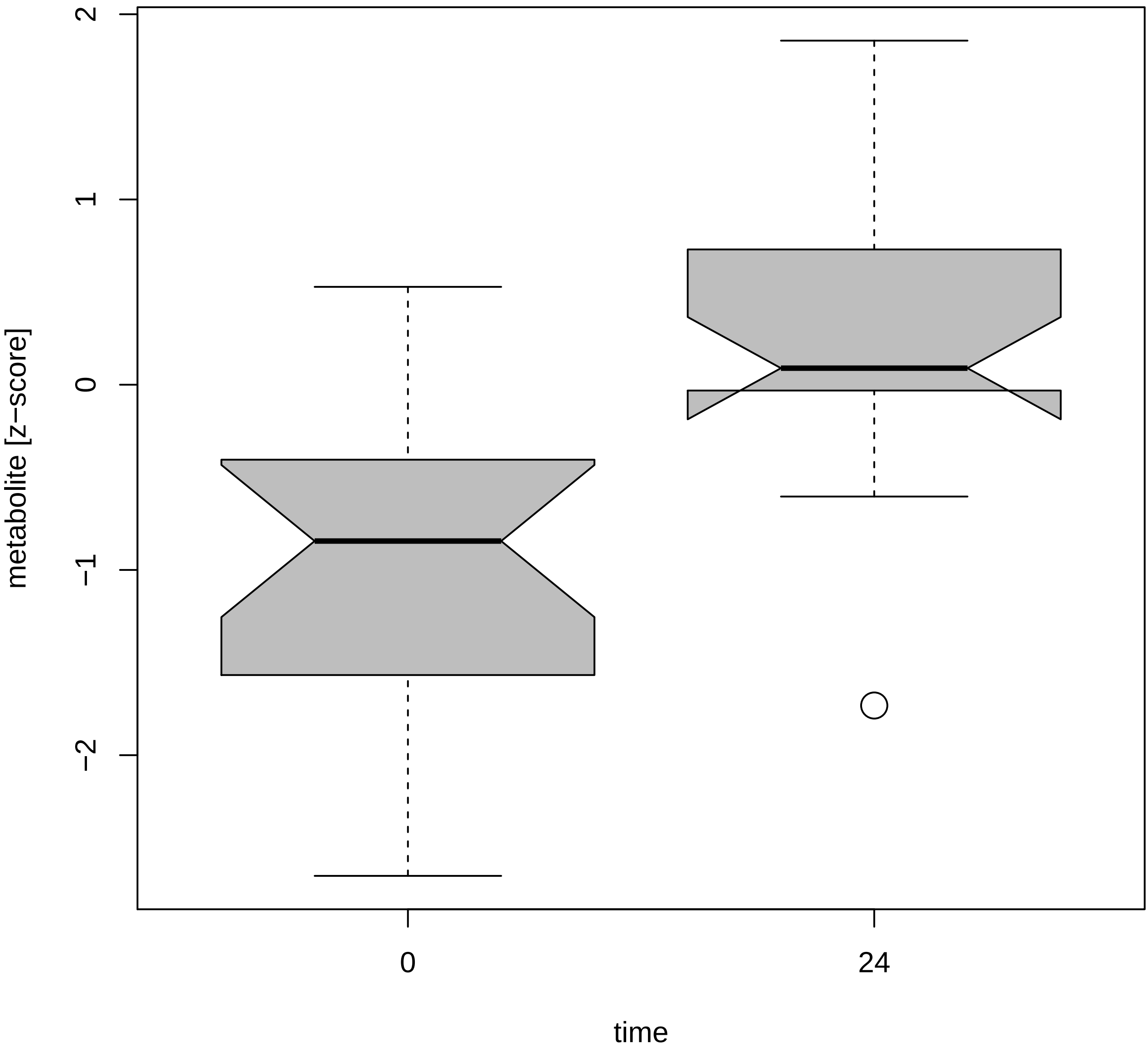

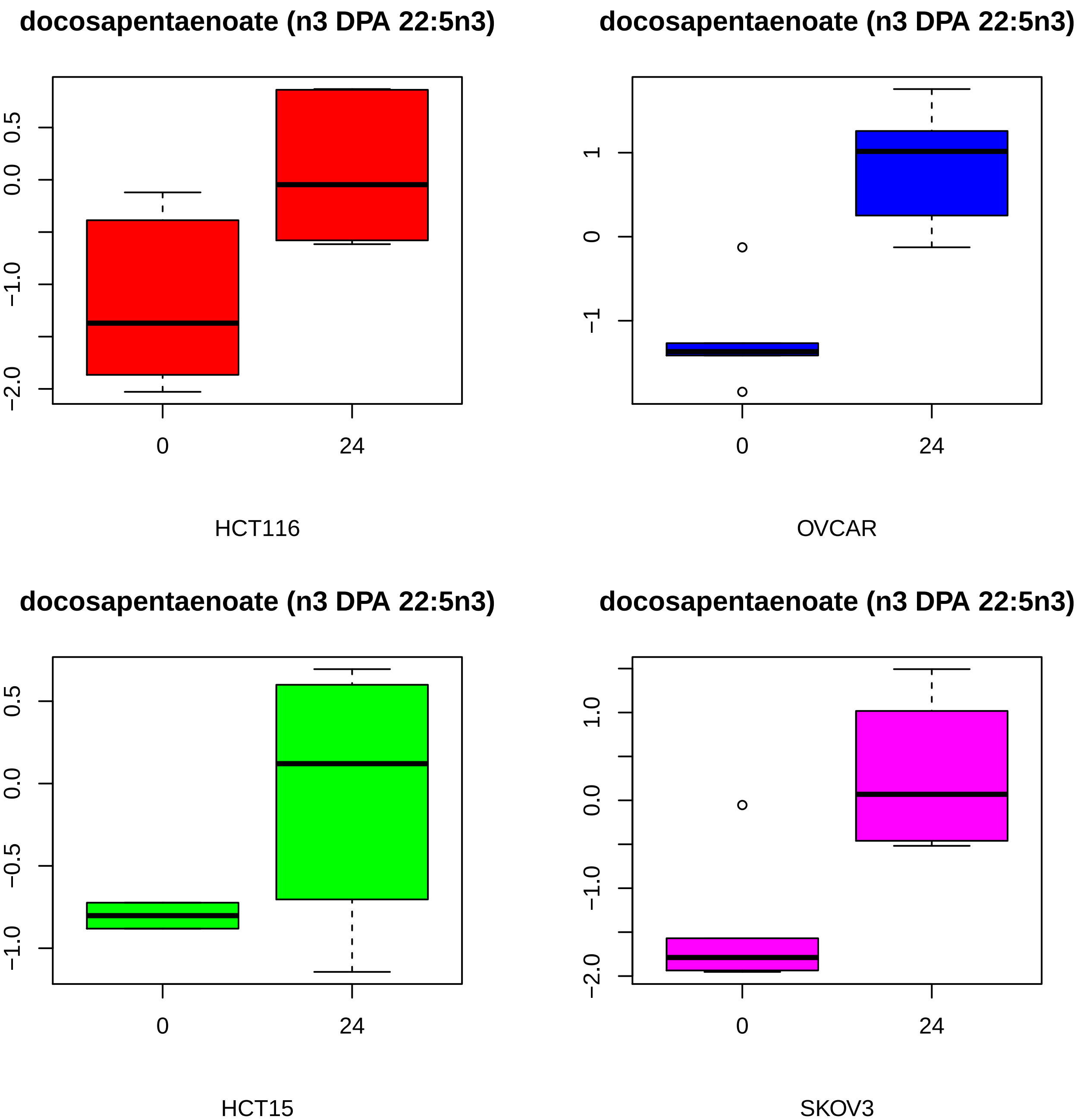
docosapentaenoate (n3 DPA 22:5n3)
0.5
0.0
−1.0
−2.0
0
24
HCT116
docosapentaenoate (n3 DPA 22:5n3)
1
0
−1
0
24
OVCAR
docosapentaenoate (n3 DPA 22:5n3)
0.5
0.0
−0.5
−1.0
0
24
HCT15
docosapentaenoate (n3 DPA 22:5n3)
1.0
0.0
−1.0
−2.0
0
24
SKOV3

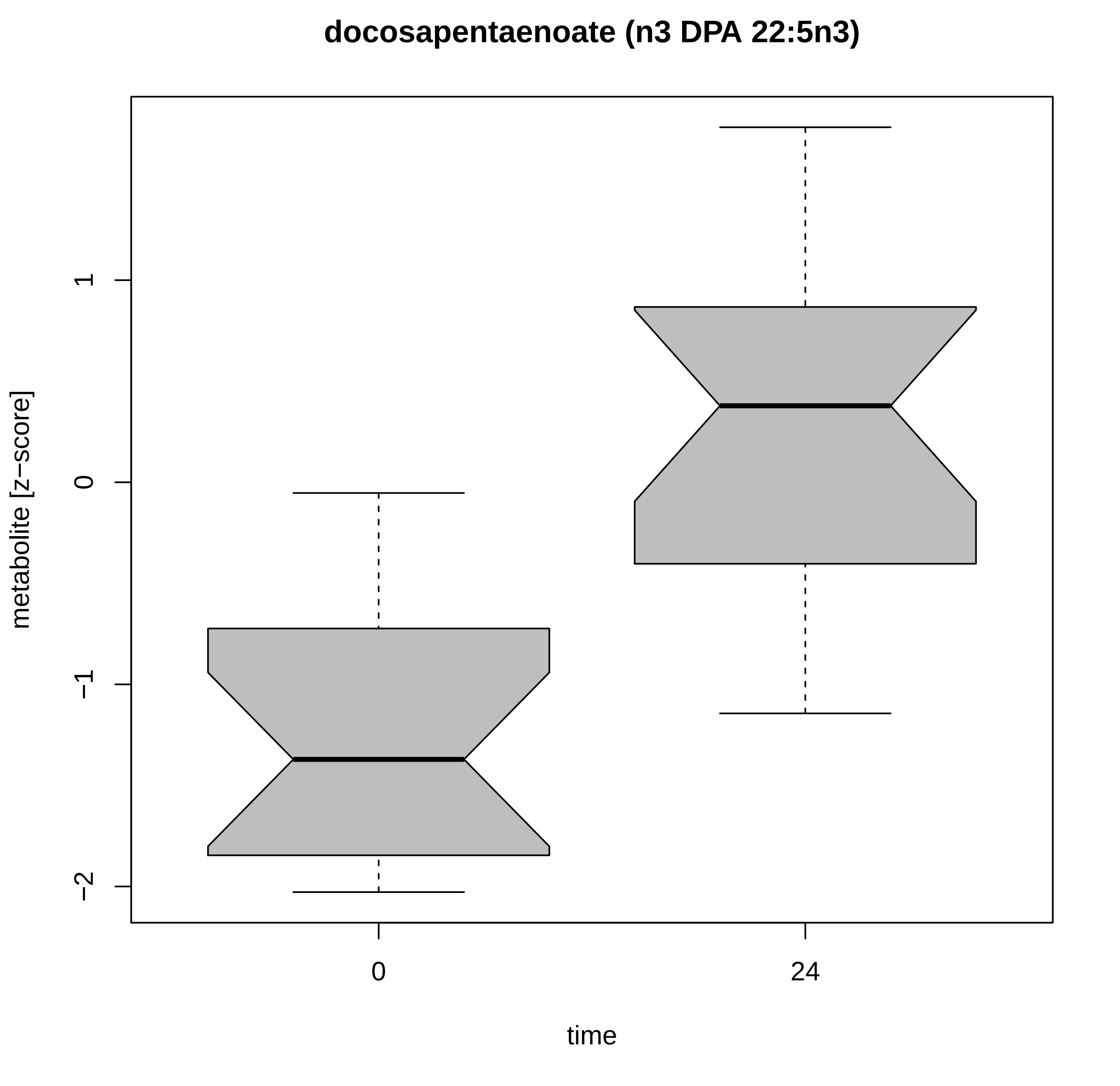

docosapentaenoate (n3 DPA 22:5n3)
metabolite [z−score]
1
0
−1
−2
0
24
time

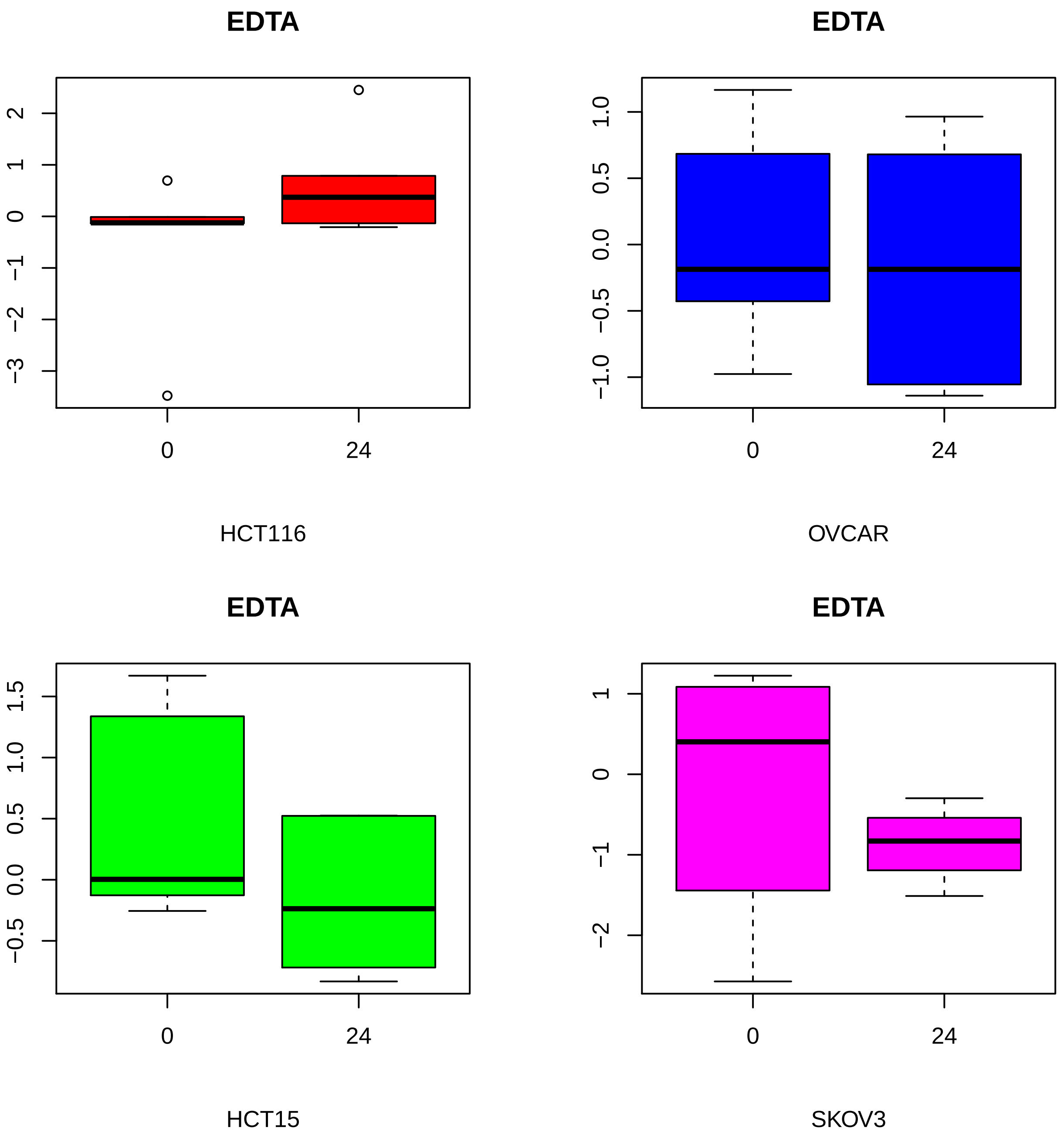
EDTA
HCT116
OVCAR
HCT15
SKOV3
0
24
2
1
0
−1
−2
−3
1.0
0.5
0.0
−0.5
−1.0
1.5

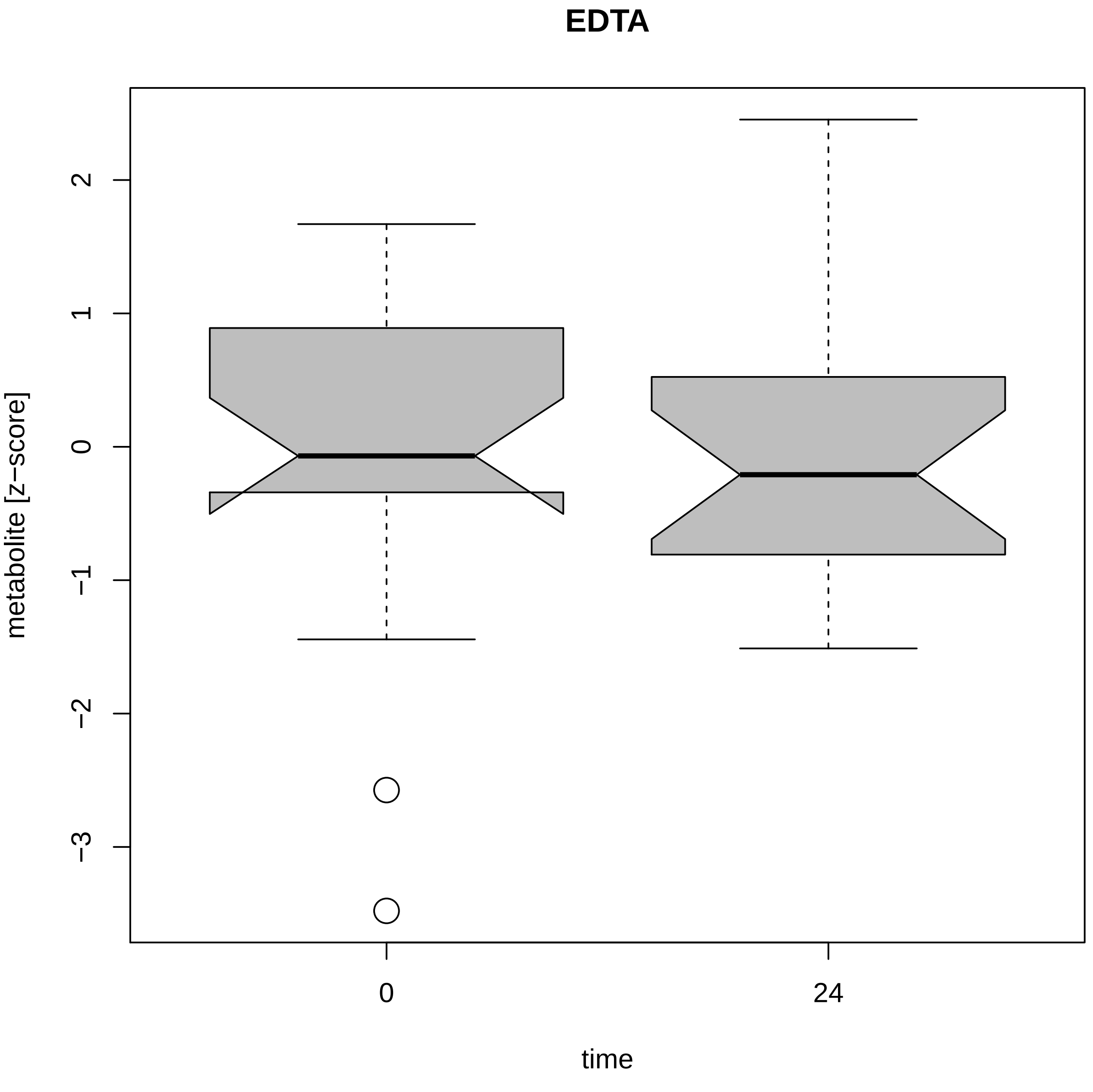
EDTA
metabolite [z−score]
2
1
0
−1
−2
−3
0
24
time

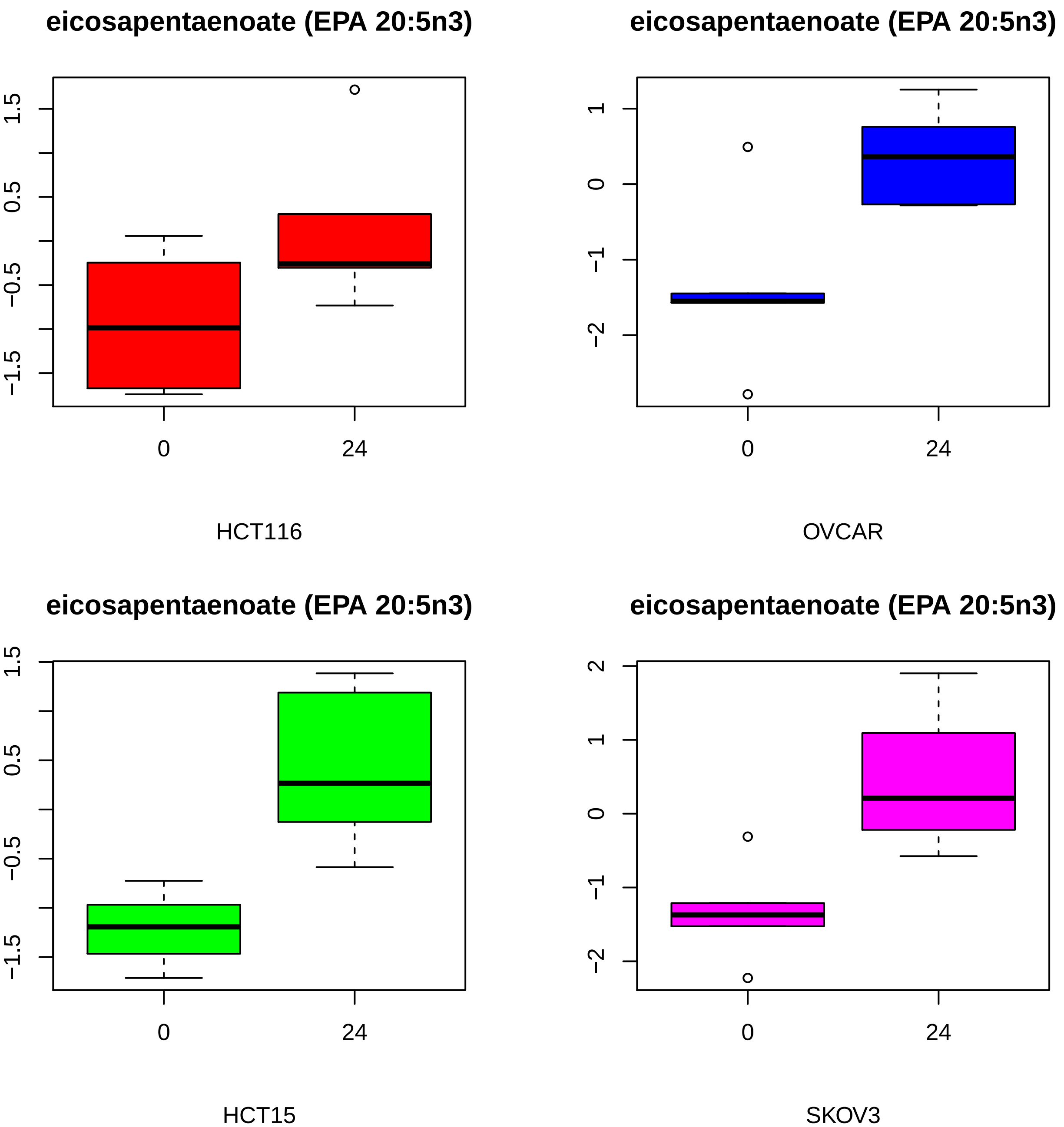
eicosapentaenoate (EPA 20:5n3)
1.5
0.5
−0.5
−1.5
0
24
HCT116
eicosapentaenoate (EPA 20:5n3)
1
0
−1
−2
0
24
OVCAR
eicosapentaenoate (EPA 20:5n3)
1.5
0.5
−0.5
−1.5
0
24
HCT15
eicosapentaenoate (EPA 20:5n3)
2
1
0
−1
−2
0
24
SKOV3

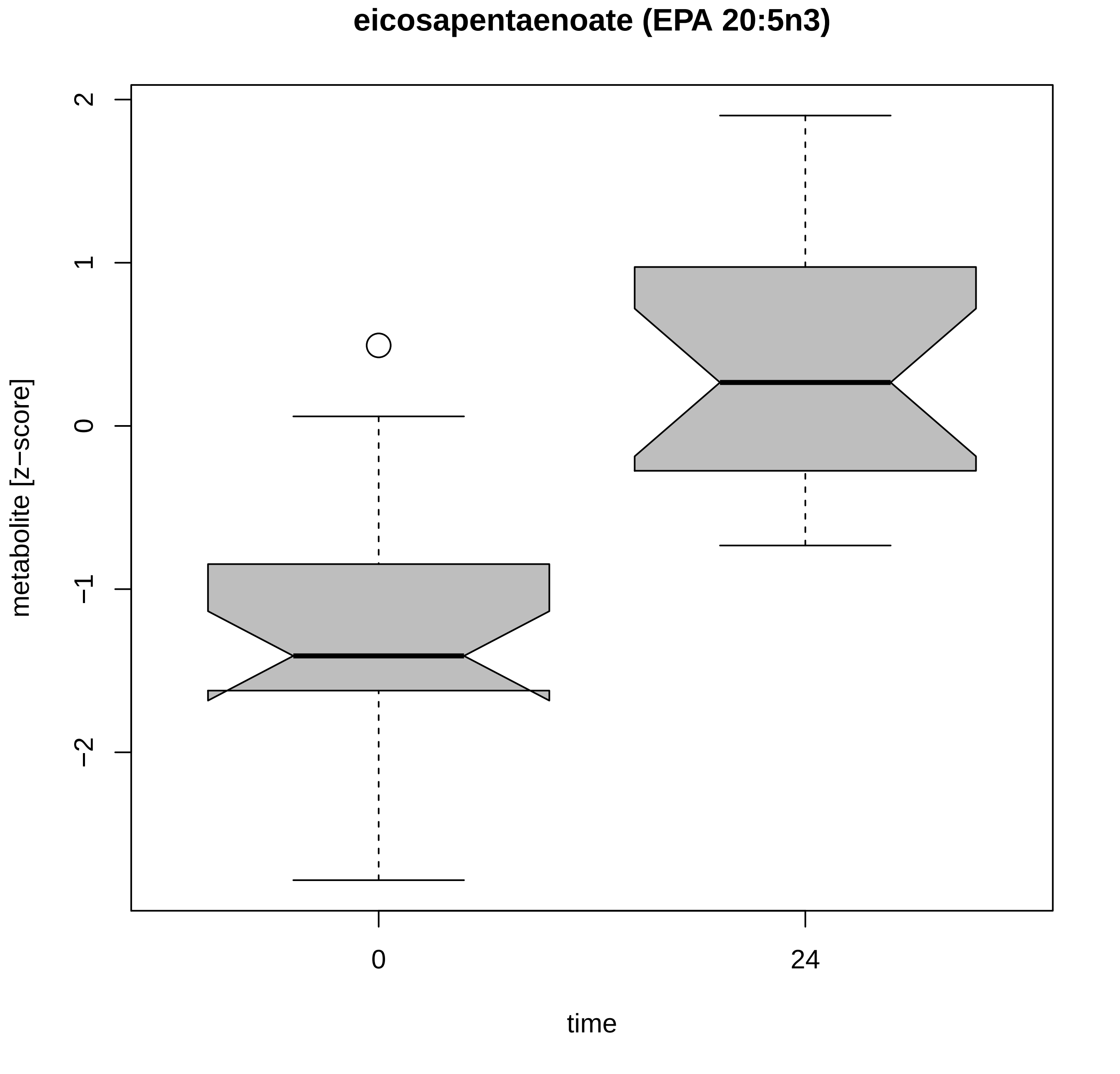

eicosapentaenoate (EPA 20:5n3)
metabolite [z−score]
2
1
0
−1
−2
0
24
time

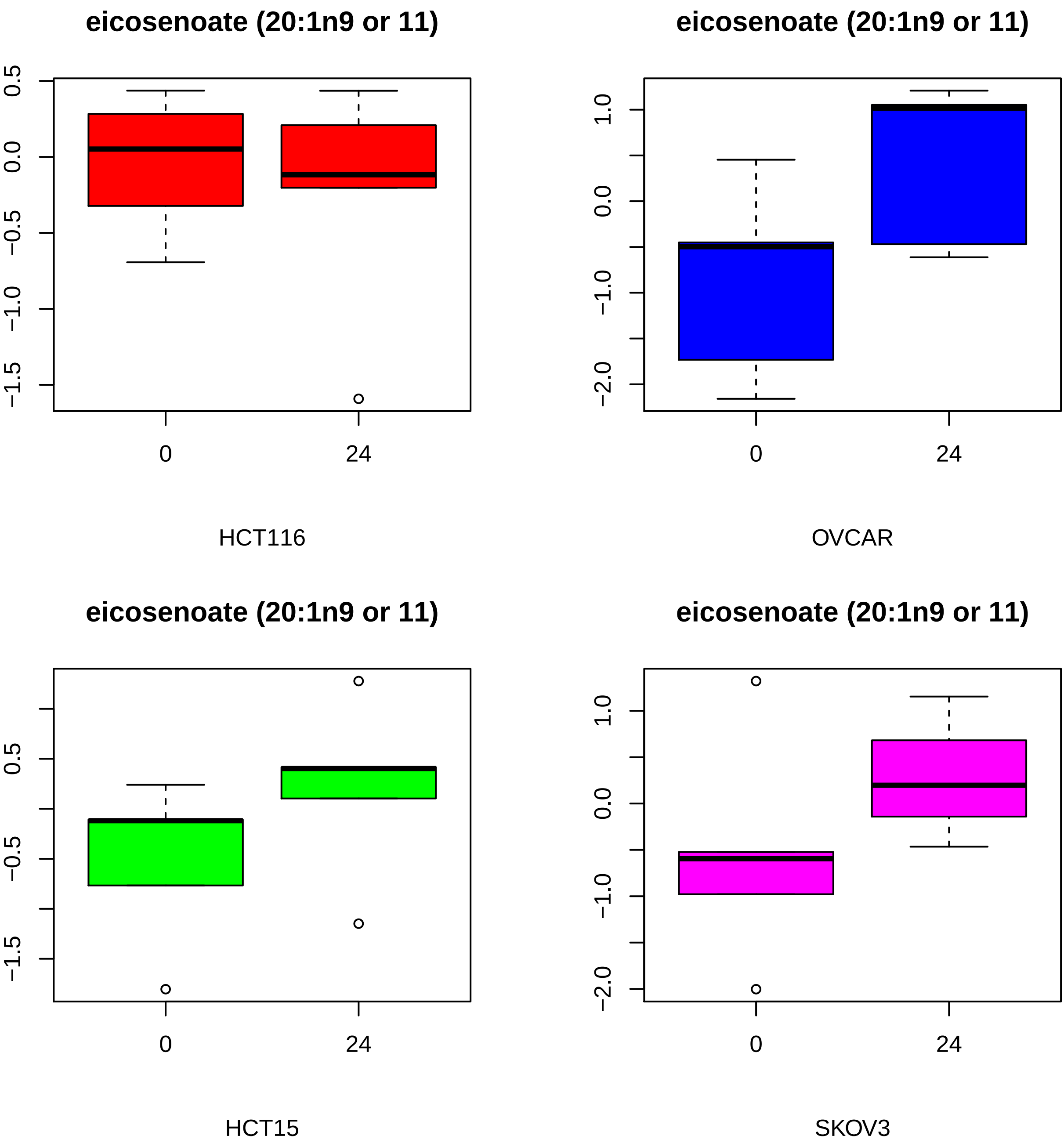
eicosenoate (20:1n9 or 11)
0.5
0.0
−0.5
−1.0
−1.5
0
24
HCT116
eicosenoate (20:1n9 or 11)
1.0
0.0
−1.0
−2.0
0
24
OVCAR
eicosenoate (20:1n9 or 11)
0.5
−0.5
−1.5
0
24
HCT15
eicosenoate (20:1n9 or 11)
1.0
0.0
−1.0
−2.0
0
24
SKOV3

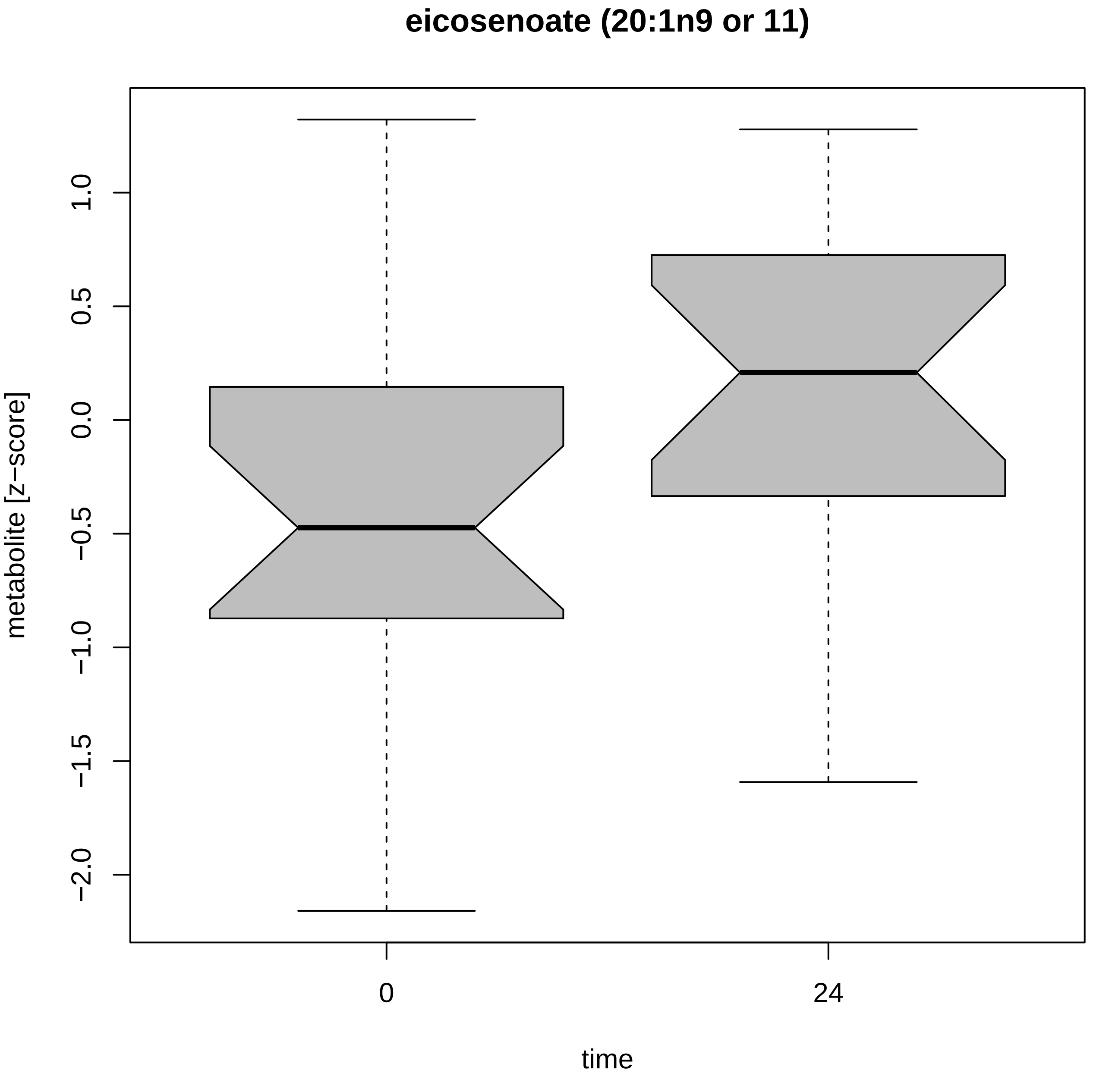
eicosenoate (20:1n9 or 11)
metabolite [z−score]
1.0
0.5
0.0
−0.5
−1.0
−1.5
−2.0
0
24
time

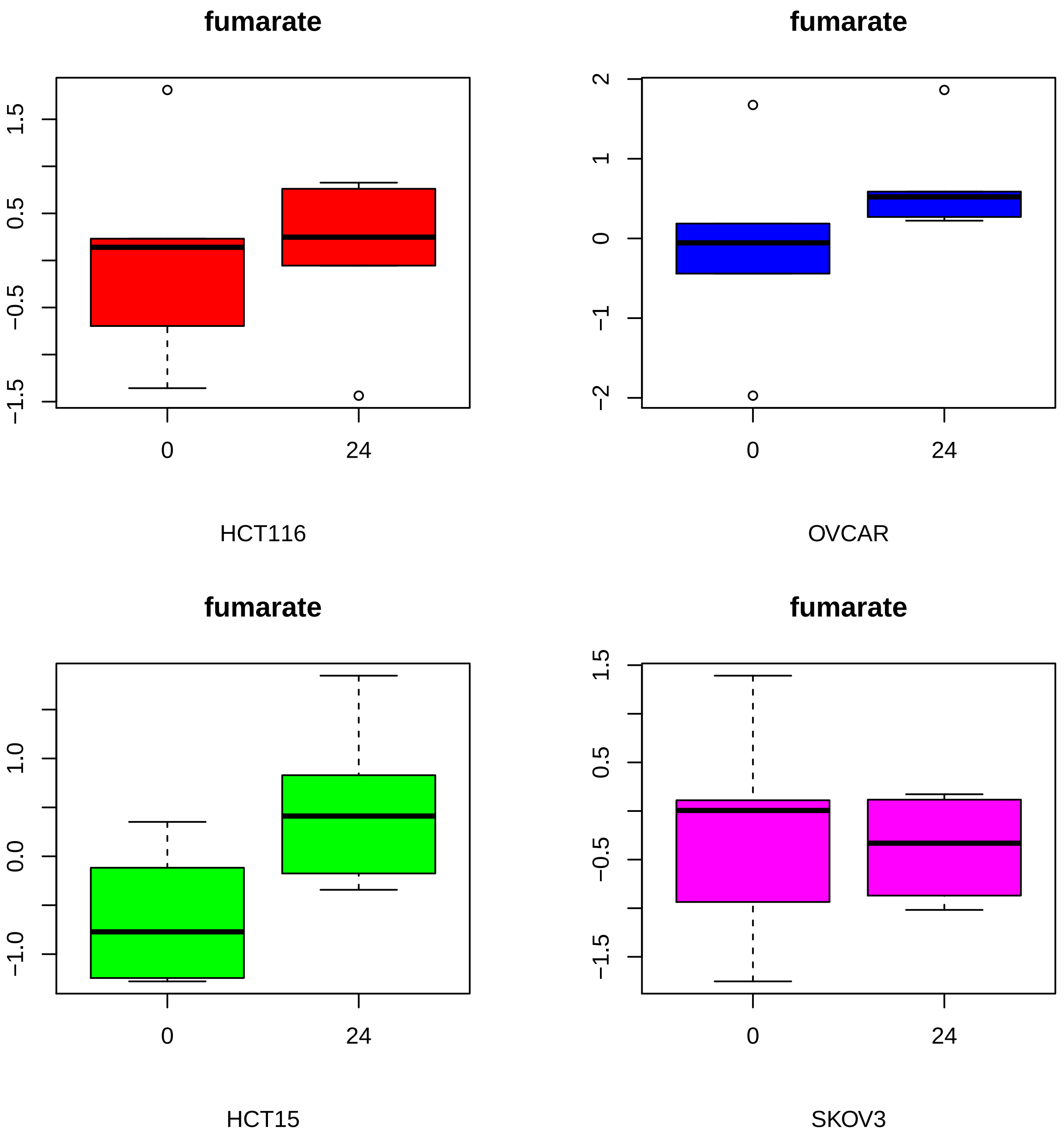
fumarate
1.5
0.5
−0.5
−1.5
0
24
HCT116
fumarate
2
1
0
−1
−2
0
24
OVCAR
fumarate
1.0
0.0
−1.0
0
24
HCT15
fumarate
1.5
0.5
−0.5
−1.5
0
24
SKOV3

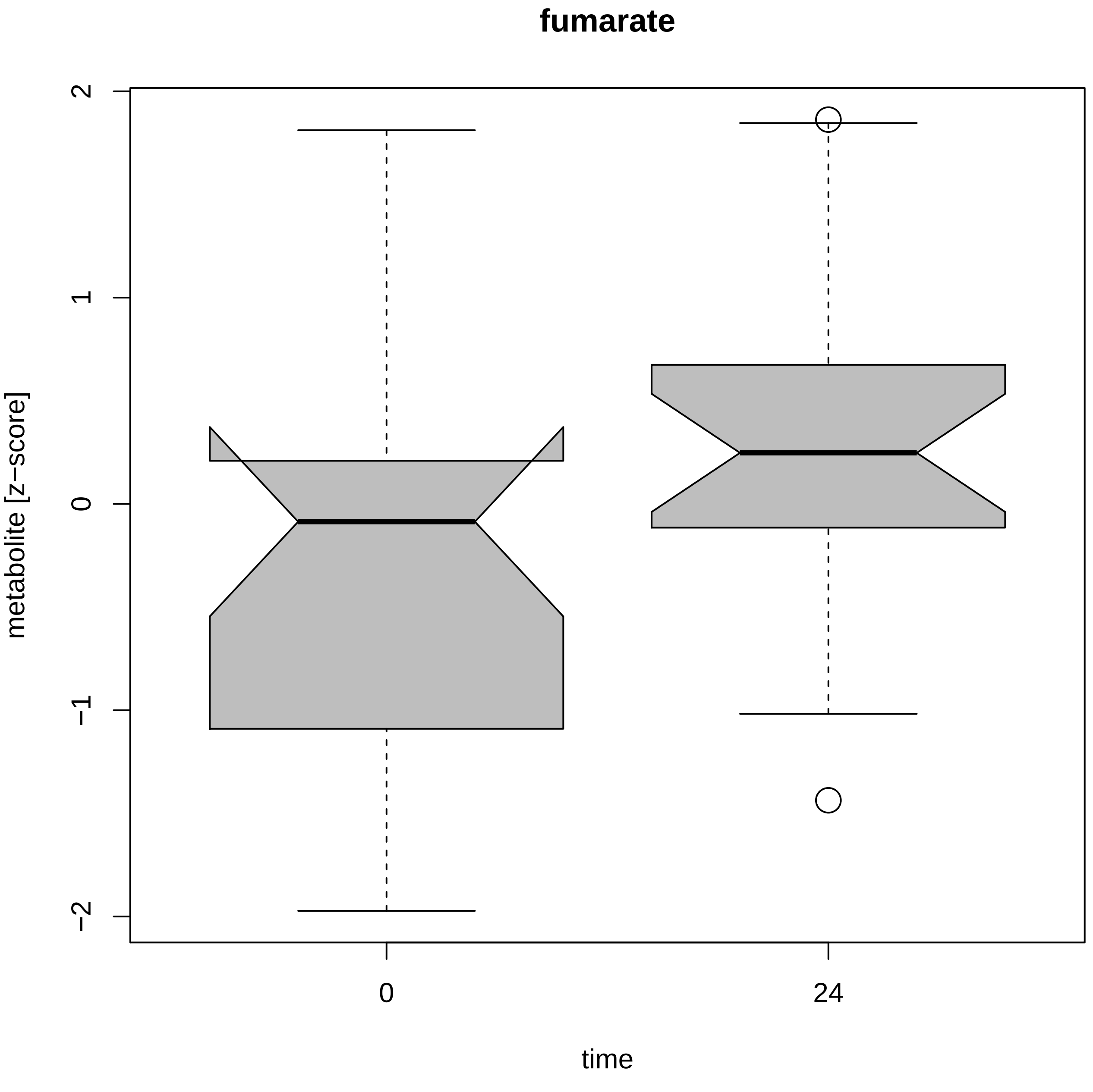
fumarate
metabolite [z−score]
2
1
0
−1
−2
0
24
time

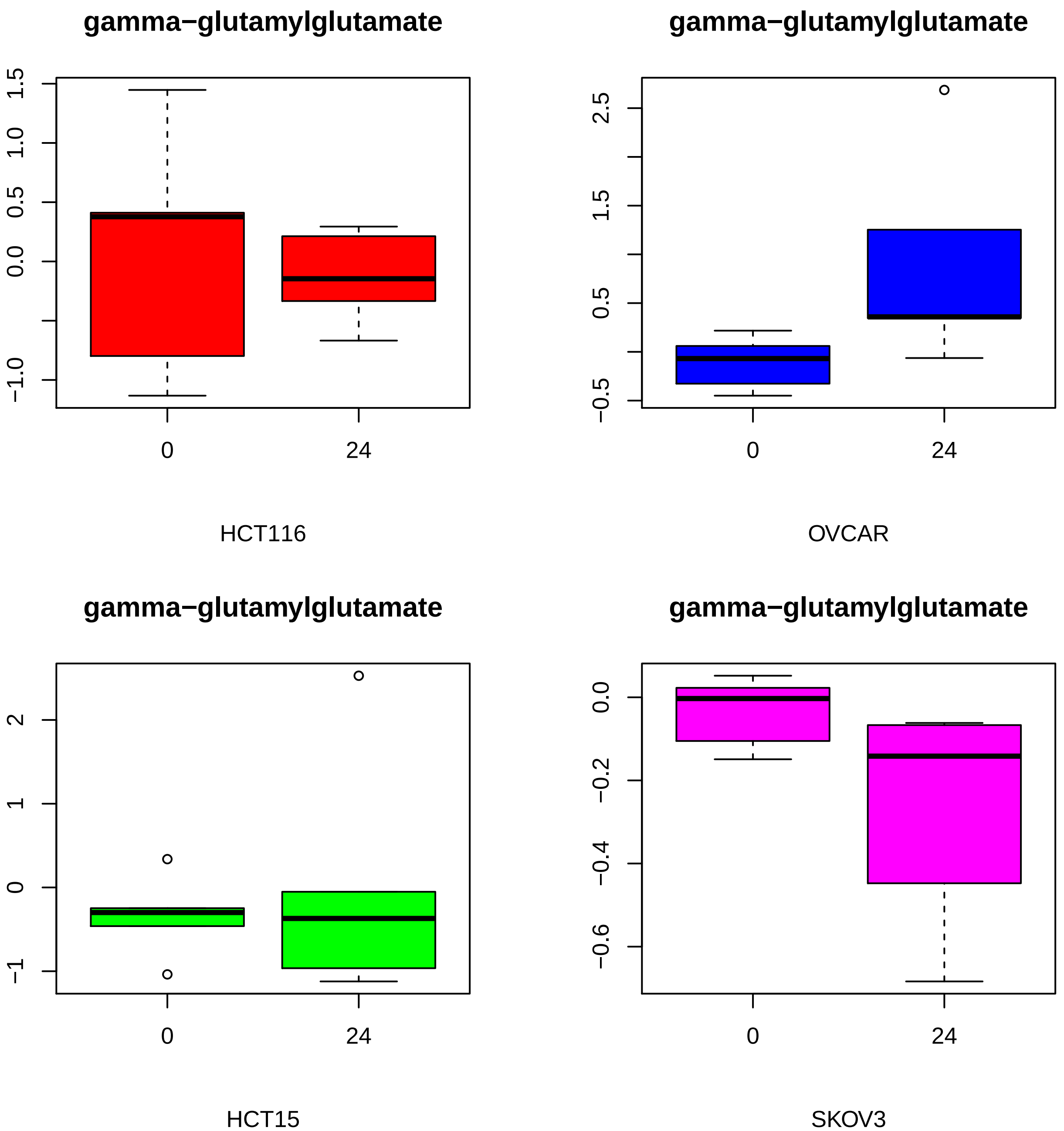
gamma−glutamylglutamate
1.5
1.0
0.5
0.0
−1.0
0
24
HCT116
gamma−glutamylglutamate
2.5
1.5
0.5
−0.5
0
24
OVCAR
gamma−glutamylglutamate
2
1
0
−1
0
24
HCT15
gamma−glutamylglutamate
0.0
−0.2
−0.4
−0.6
0
24
SKOV3

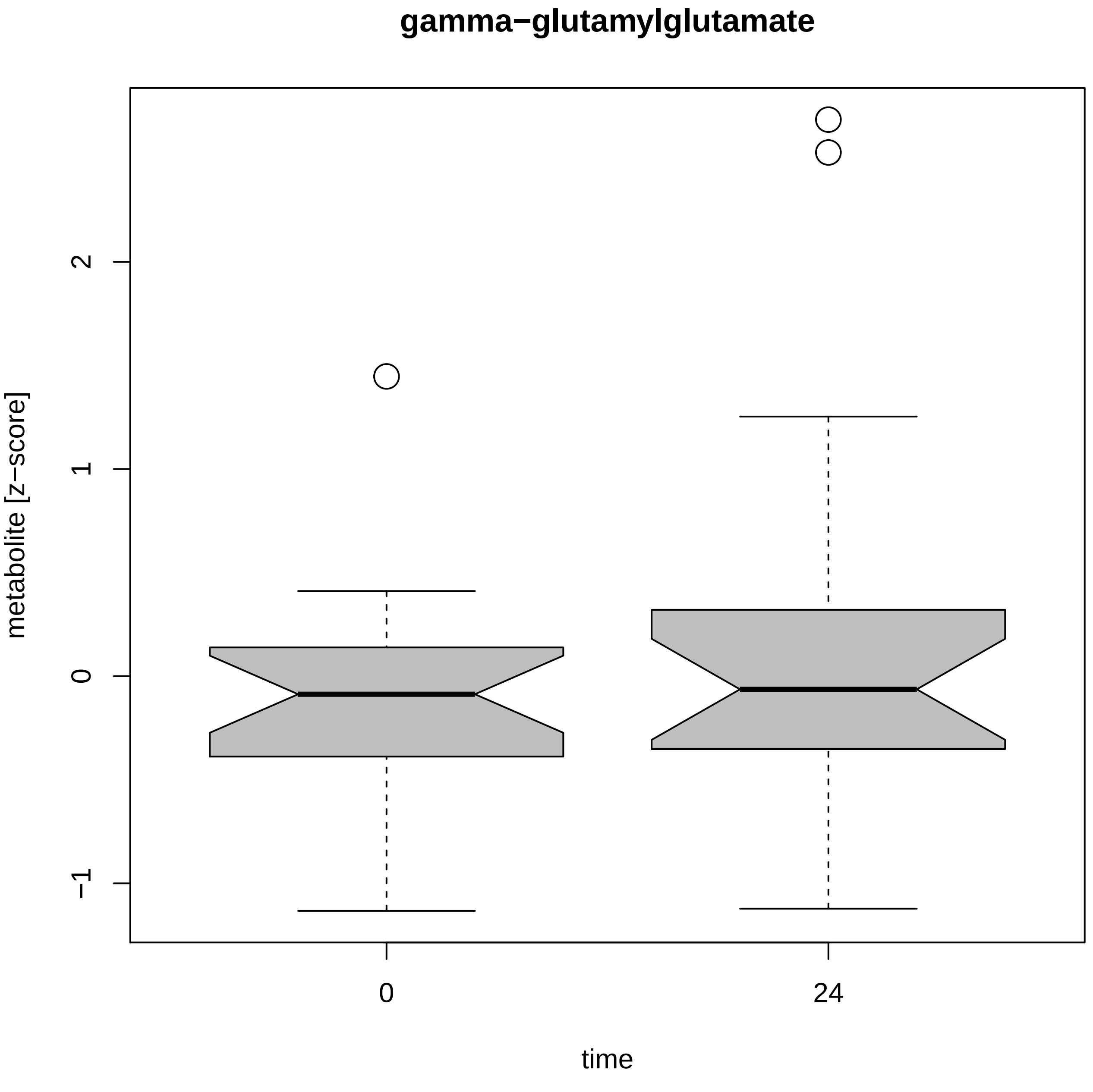

gamma−glutamylglutamate
metabolite [z−score]
−1
0
1
2
0
24
time

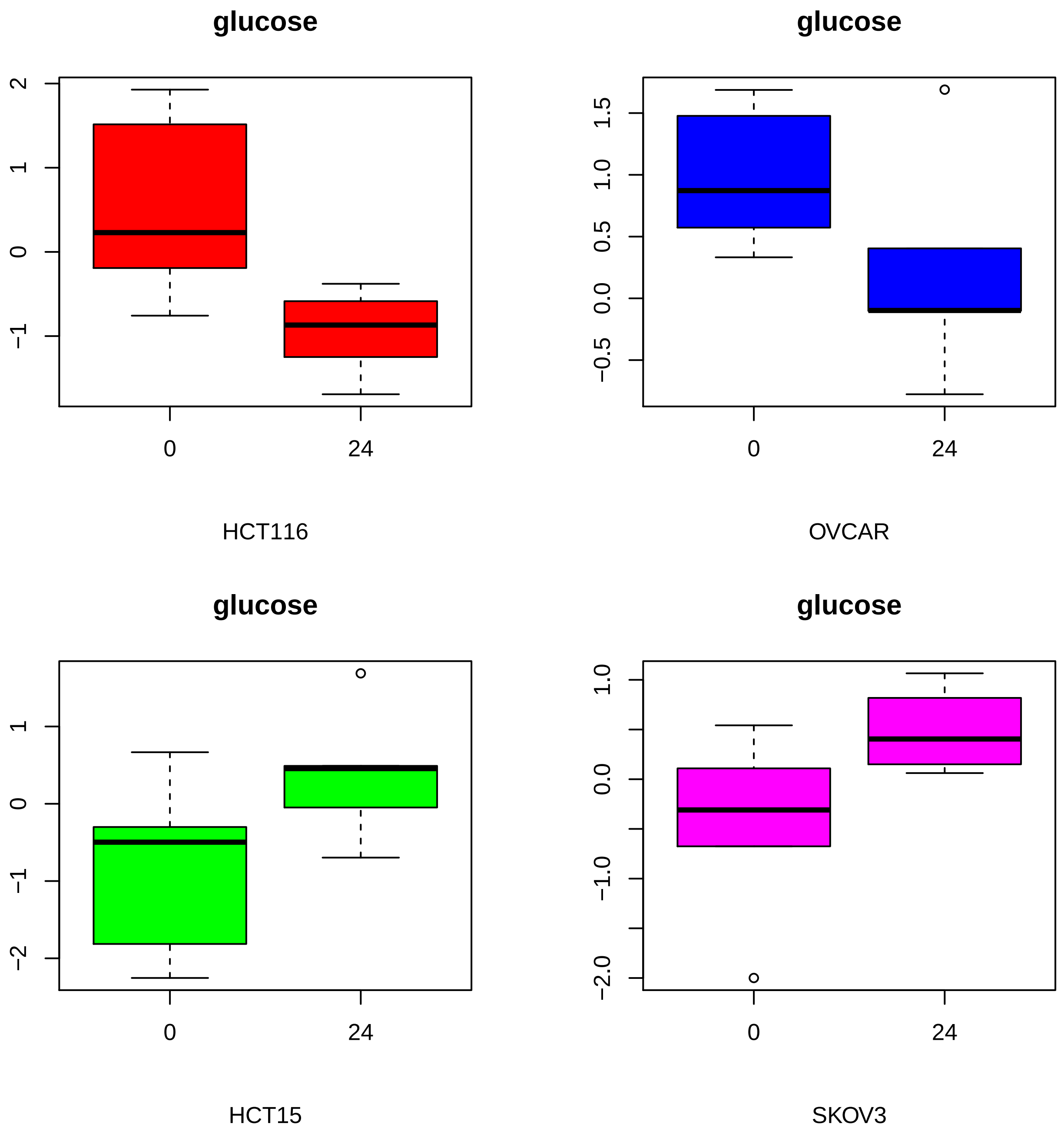
glucose
glucose
glucose
glucose
2
1
0
−1
1.5
1.0
0.5
0.0
−0.5
1
0
−1
−2
1.0
0.0
−1.0
−2.0
0
24
0
24
0
24
0
24
HCT116
OVCAR
HCT15
SKOV3

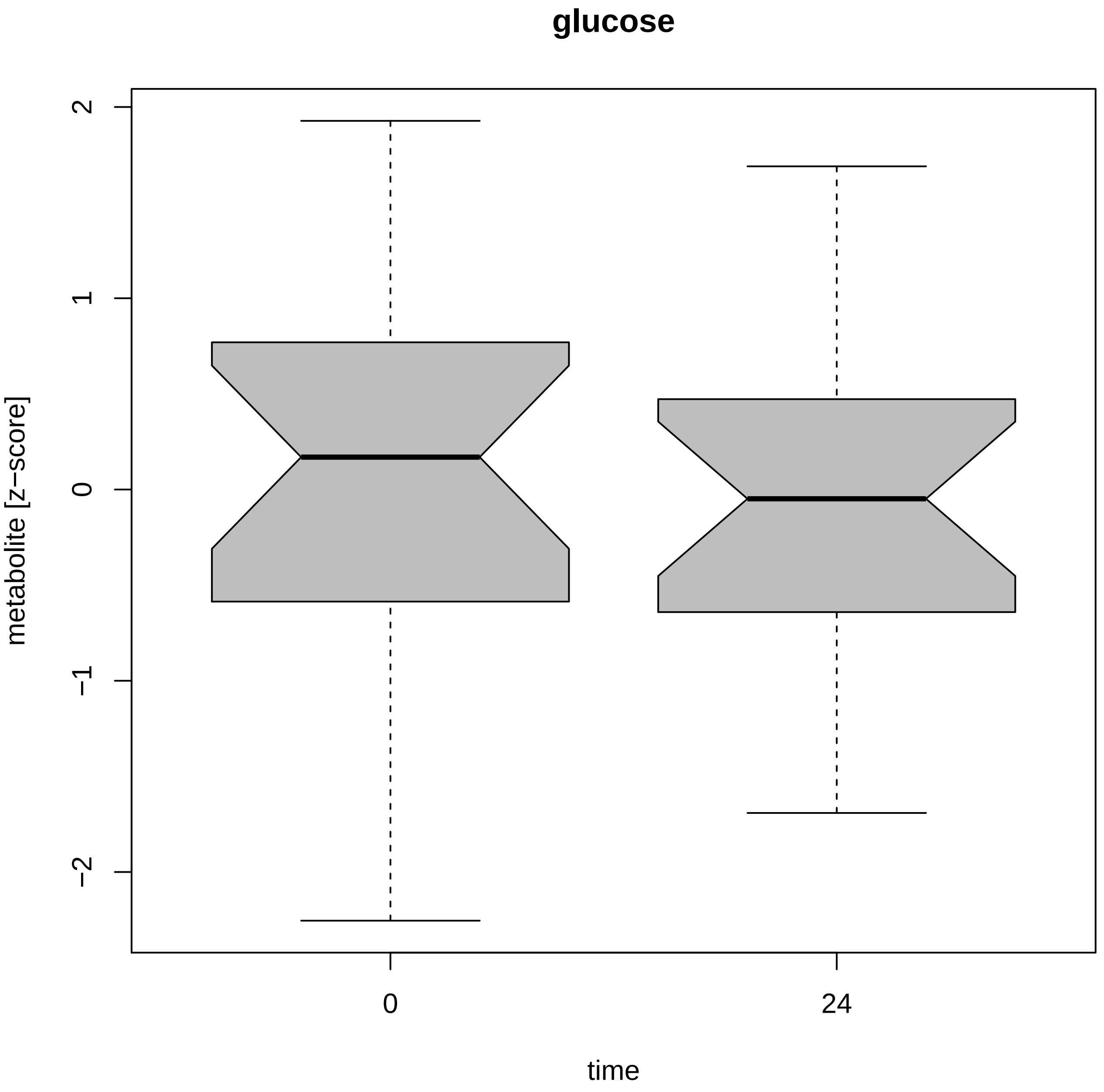
glucose
metabolite [z−score]
2
1
0
−1
−2
0
24
time

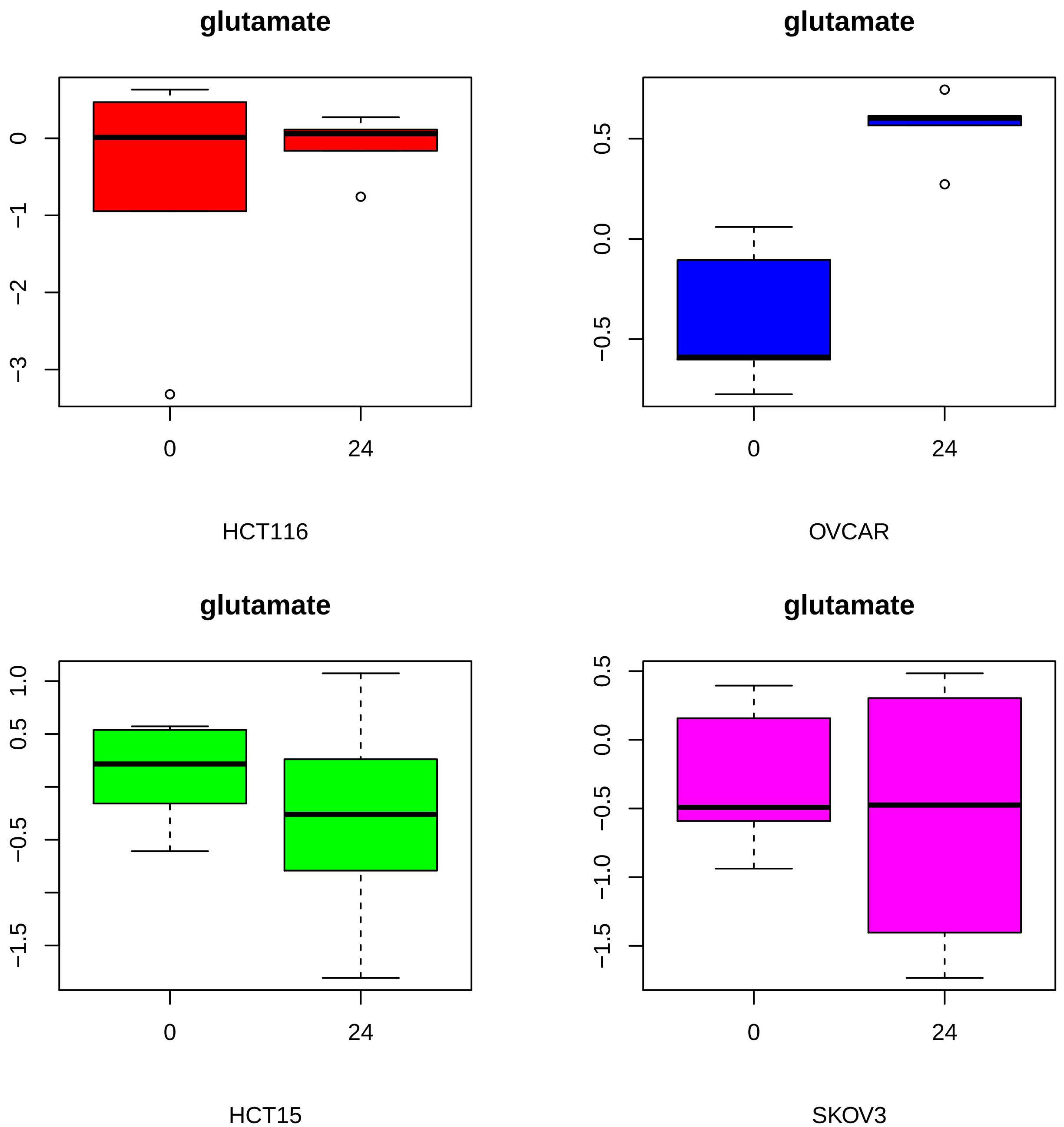
glutamate
0
−1
−2
−3
0
24
HCT116
glutamate
0.5
0.0
−0.5
0
24
OVCAR
glutamate
1.0
0.5
−0.5
−1.5
0
24
HCT15
glutamate
0.5
0.0
−0.5
−1.0
−1.5
0
24
SKOV3

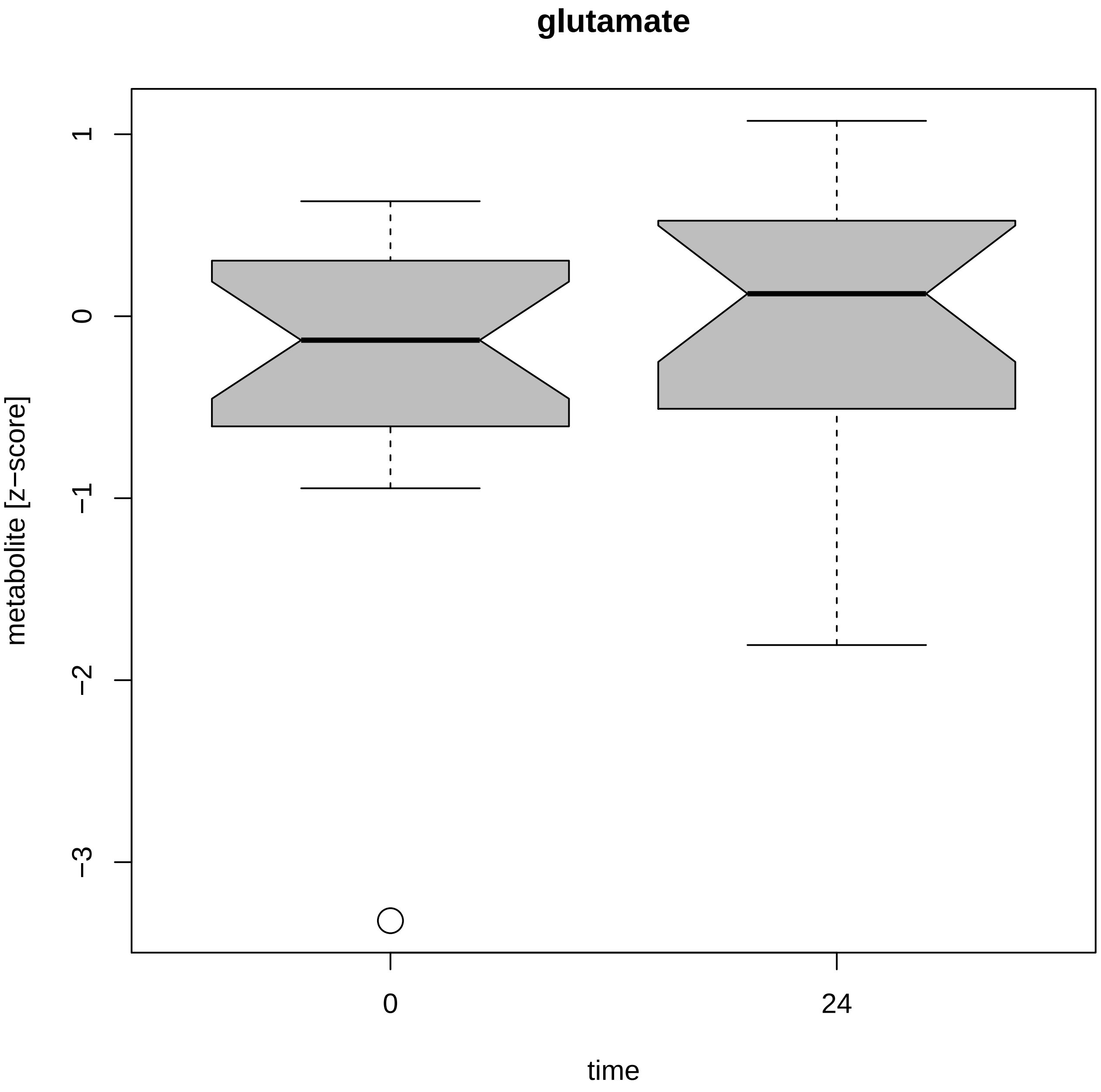
glutamate
metabolite [z−score]
1
0
−1
−2
−3
0
24
time

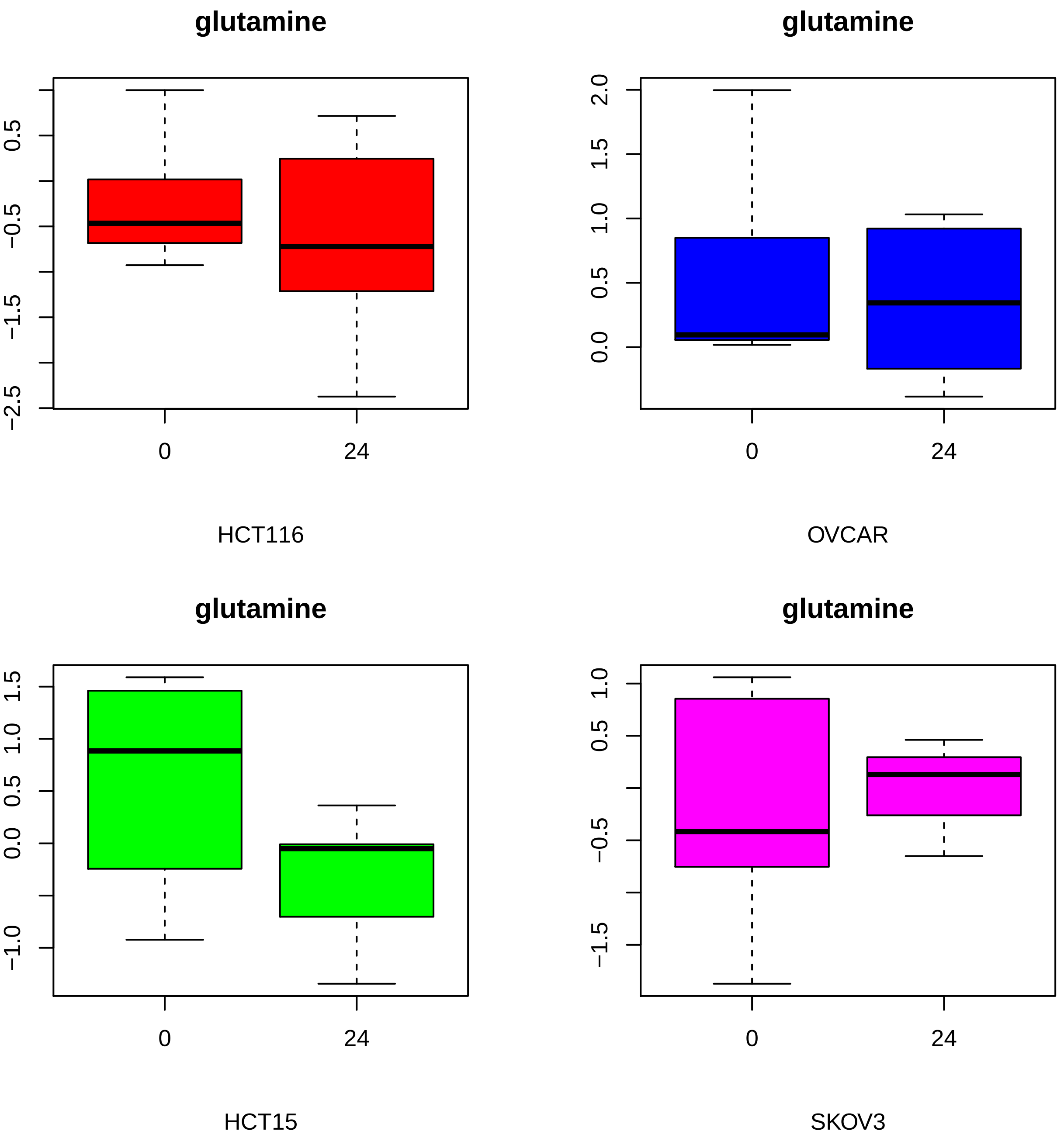
glutamine
0.5
−0.5
−1.5
−2.5
0
24
HCT116
glutamine
2.0
1.5
1.0
0.5
0.0
0
24
OVCAR
glutamine
1.5
1.0
0.5
0.0
−1.0
0
24
HCT15
glutamine
1.0
0.5
−0.5
−1.5
0
24
SKOV3

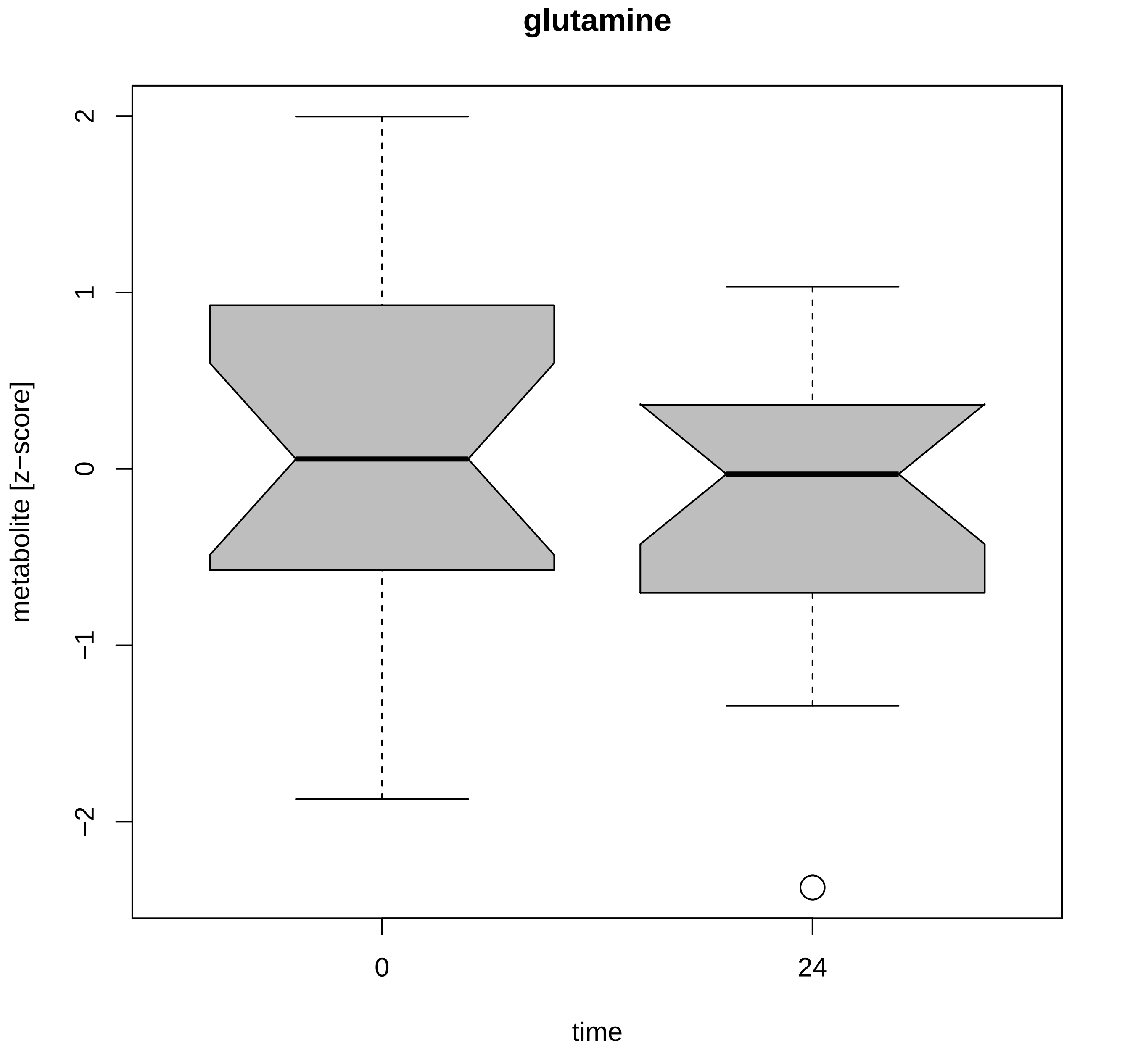
glutamine
metabolite [z−score]
2
1
0
−1
−2
0
24
time

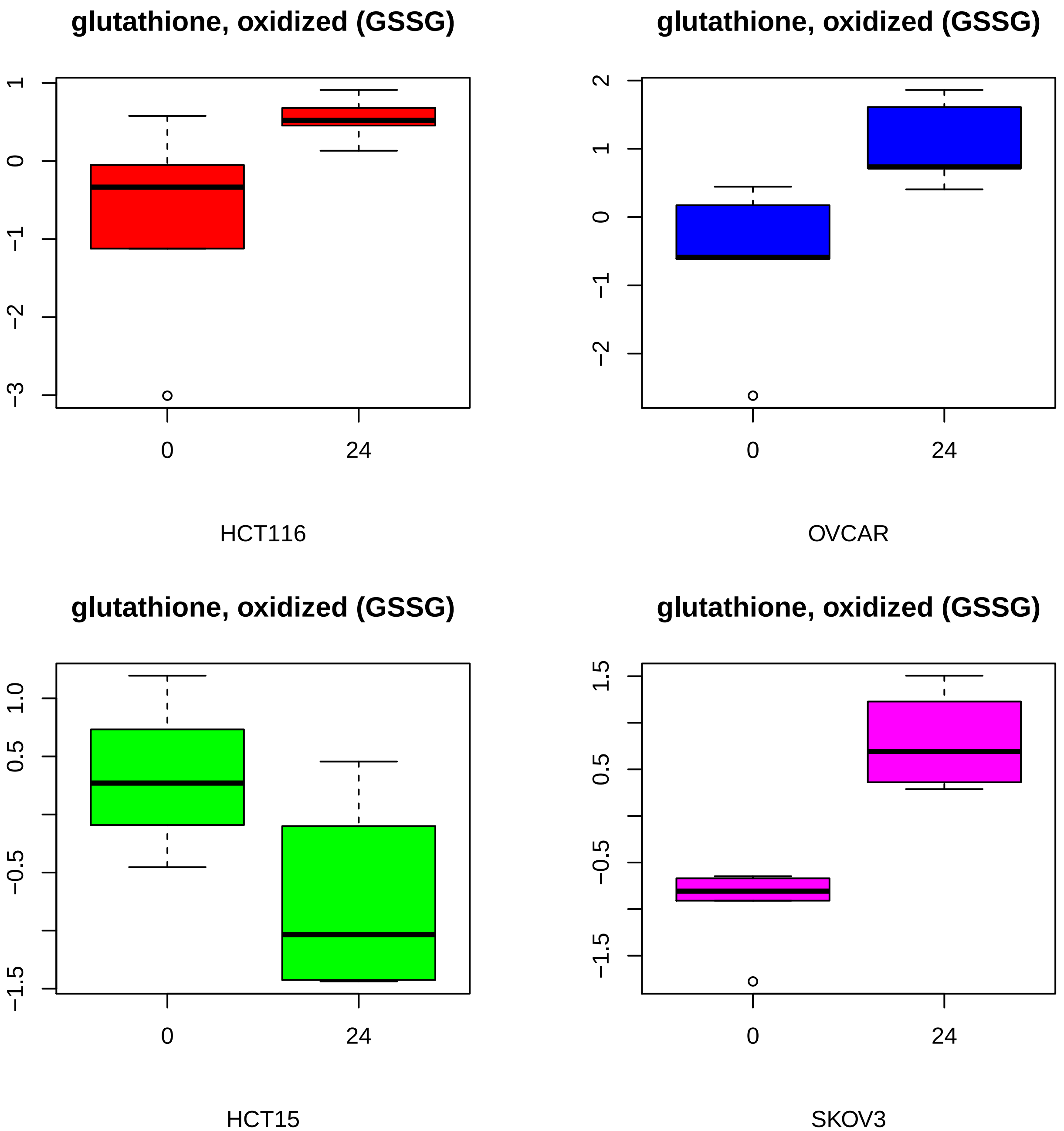
glutathione, oxidized (GSSG)
HCT116
glutathione, oxidized (GSSG)
OVCAR
glutathione, oxidized (GSSG)
HCT15
glutathione, oxidized (GSSG)
SKOV3
0
24

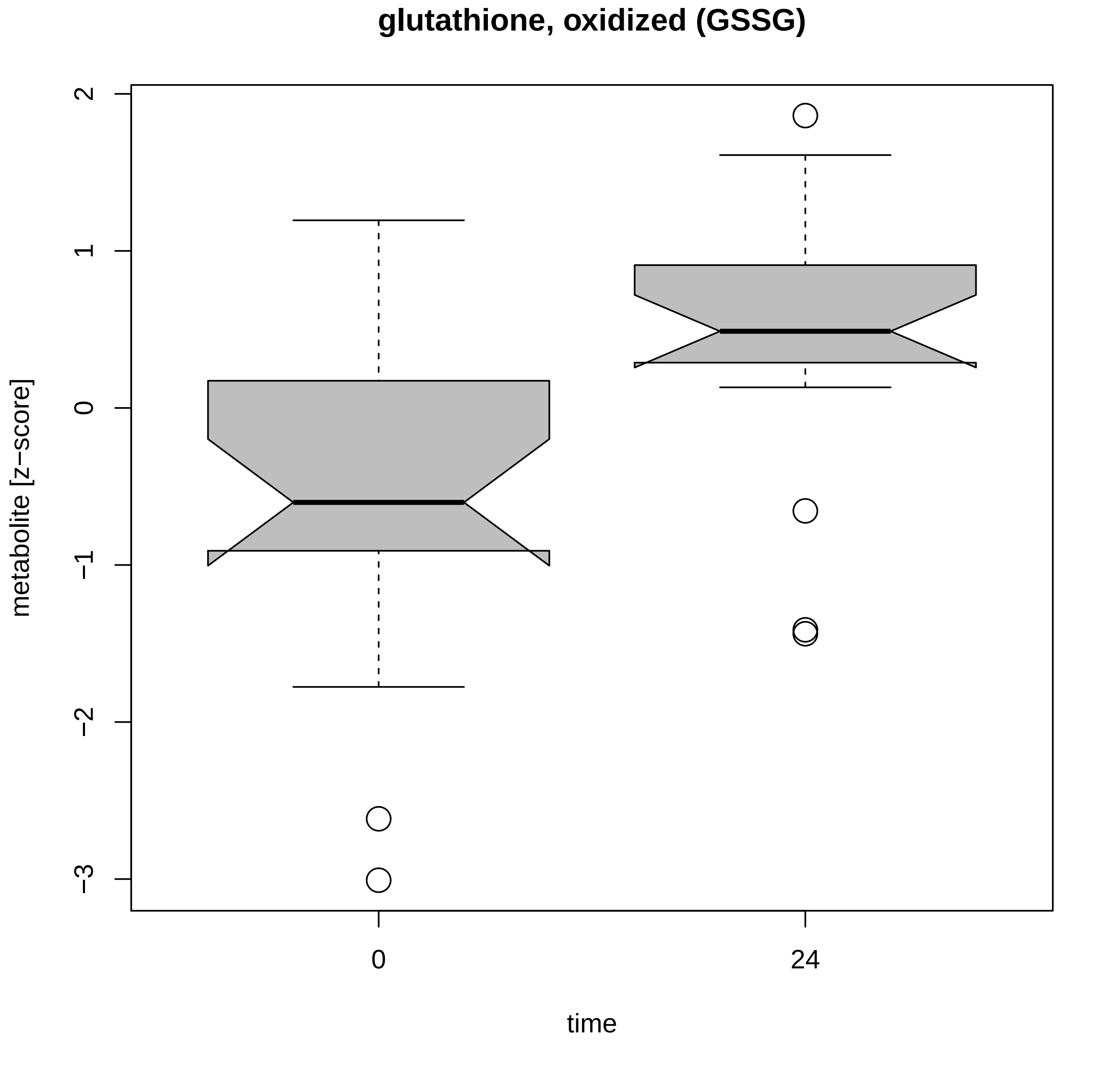

glutathione, oxidized (GSSG)
metabolite [z−score]
2
1
0
−1
−2
−3
0
24
time

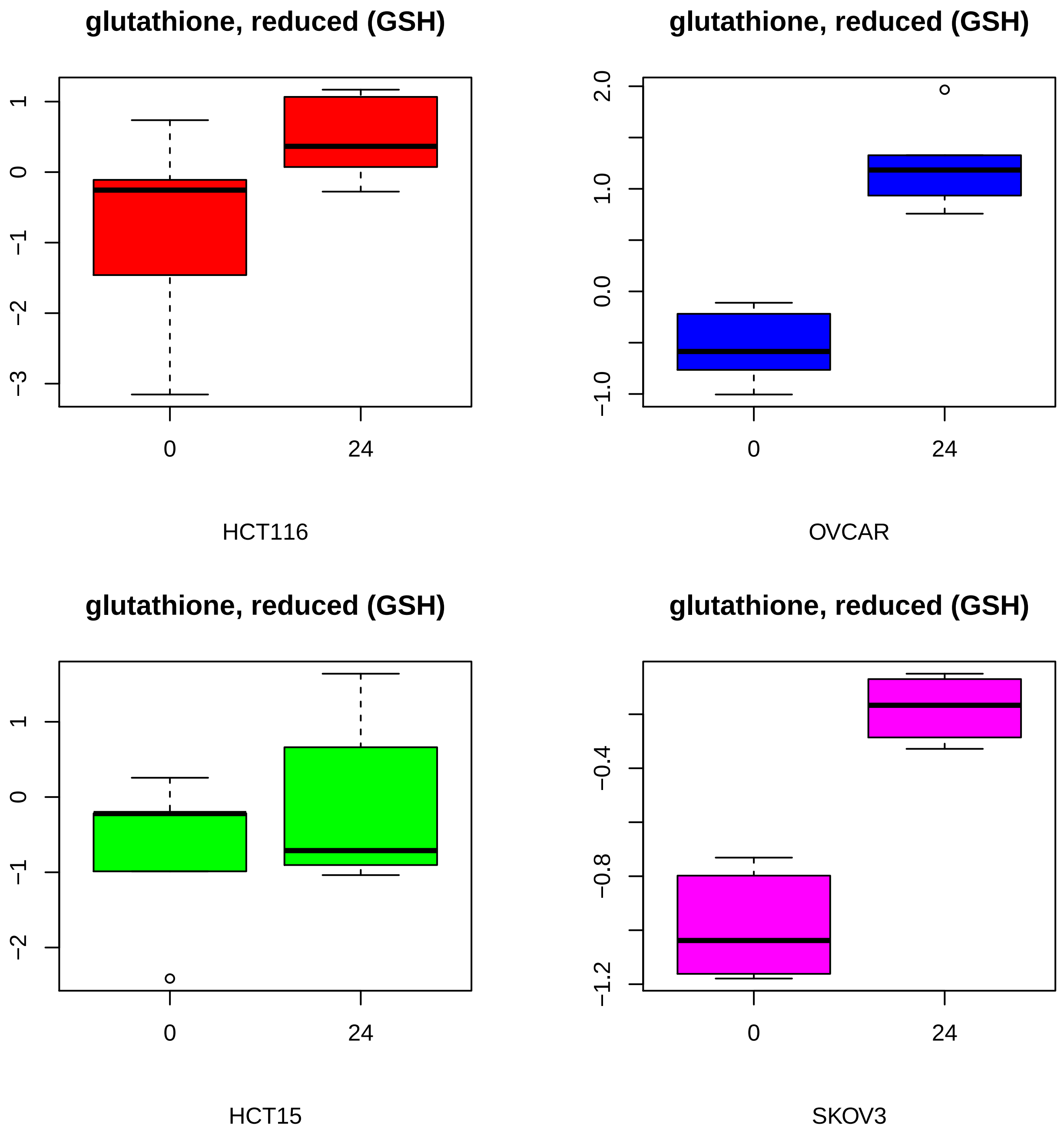
glutathione, reduced (GSH)
1
0
−1
−2
−3
0
24
HCT116
glutathione, reduced (GSH)
2.0
1.0
0.0
−1.0
0
24
OVCAR
glutathione, reduced (GSH)
1
0
−1
−2
0
24
HCT15
glutathione, reduced (GSH)
−0.4
−0.8
−1.2
0
24
SKOV3

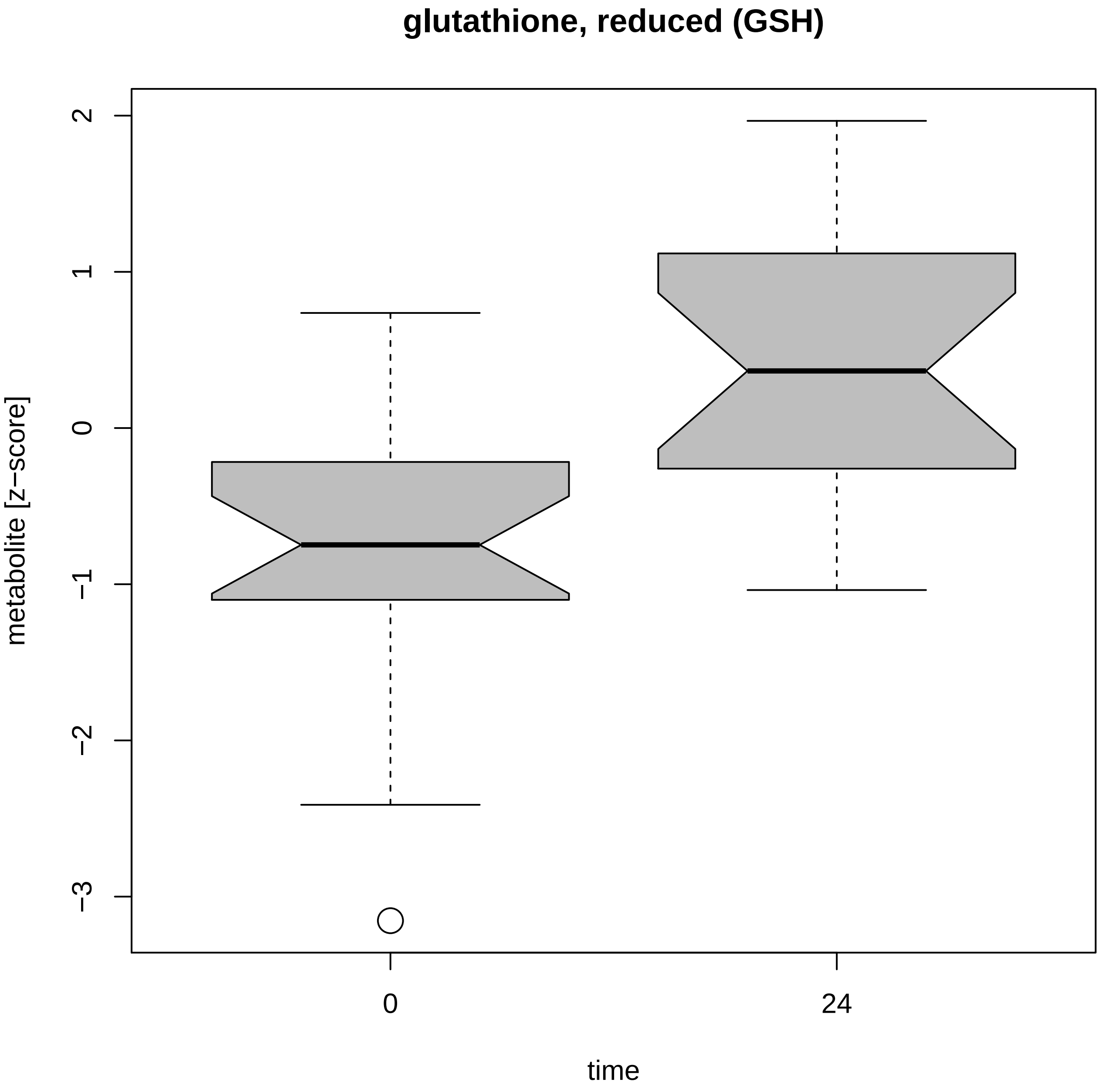
glutathione, reduced (GSH)
metabolite [z−score]
2
1
0
−1
−2
−3
0
24
time

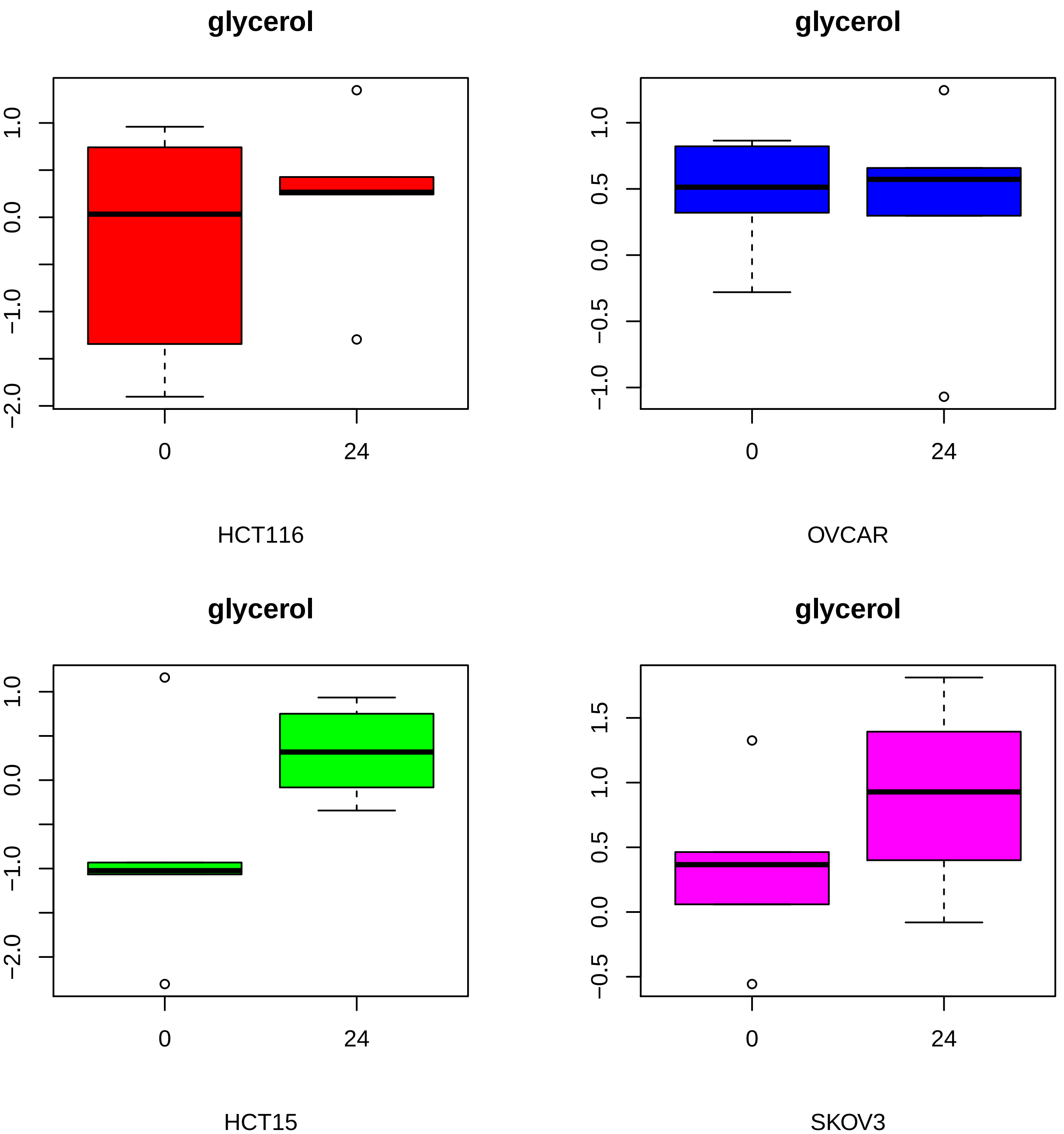

glycerol
1.0
0.0
−1.0
−2.0
0
24
HCT116
glycerol
1.0
0.5
0.0
−0.5
−1.0
0
24
OVCAR
glycerol
1.0
0.0
−1.0
−2.0
0
24
HCT15
glycerol
1.5
1.0
0.5
0.0
−0.5
0
24
SKOV3

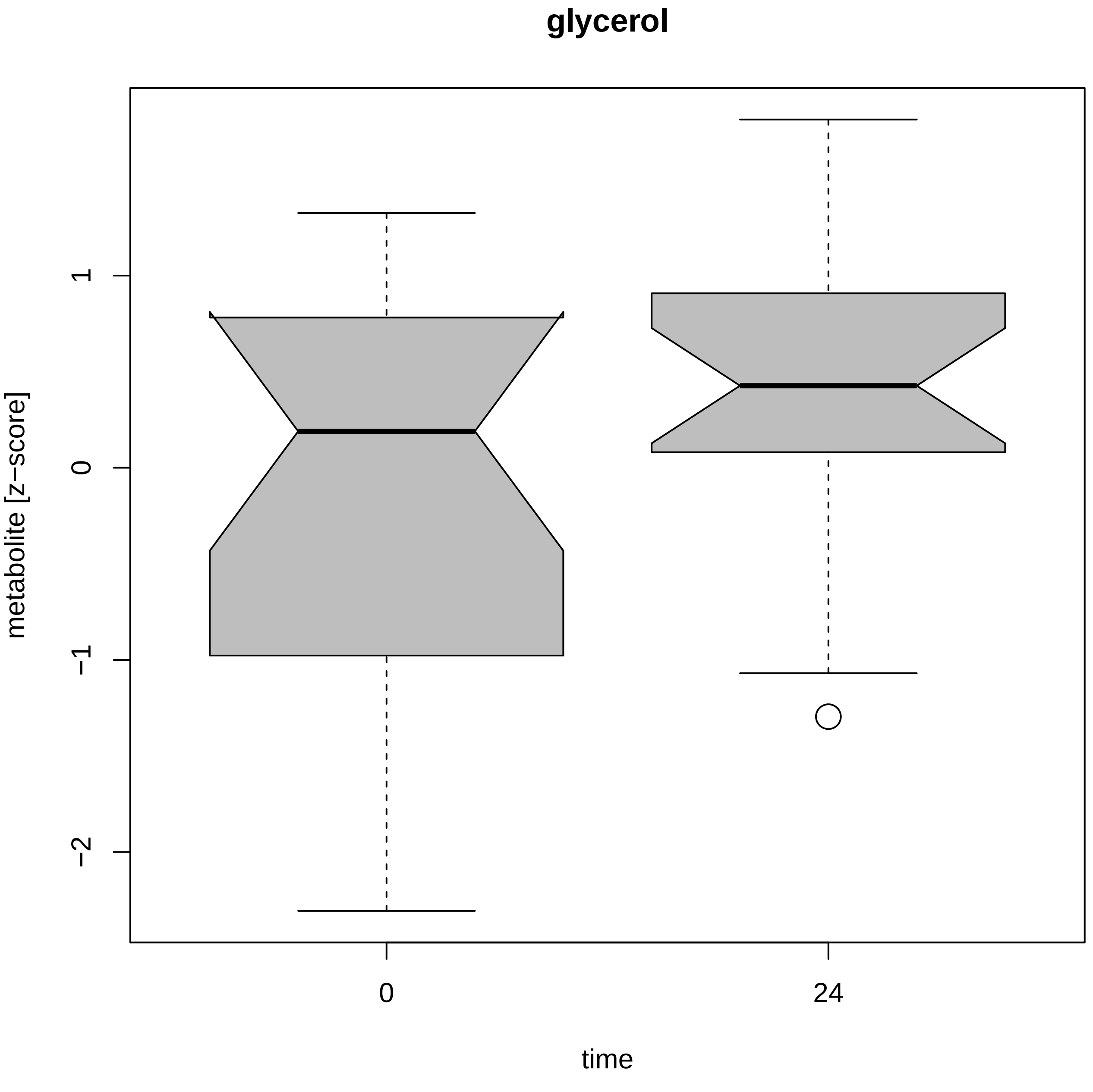
glycerol
metabolite [z−score]
1
0
−1
−2
0
24
time

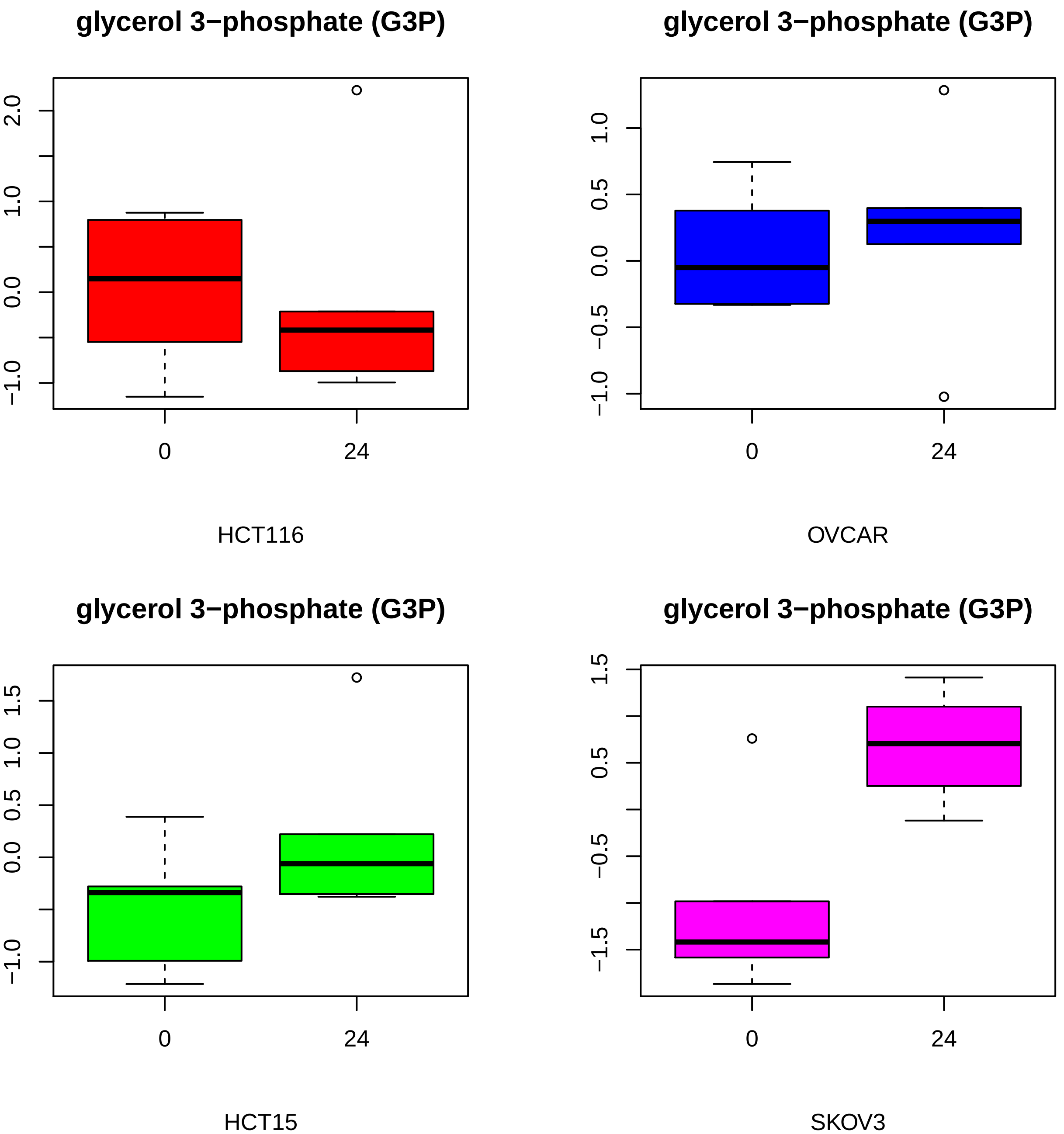
glycerol 3−phosphate (G3P)
2.0
1.0
0.0
−1.0
0
24
HCT116
glycerol 3−phosphate (G3P)
1.0
0.5
0.0
−0.5
−1.0
0
24
OVCAR
glycerol 3−phosphate (G3P)
1.5
1.0
0.5
0.0
−1.0
0
24
HCT15
glycerol 3−phosphate (G3P)
1.5
0.5
−0.5
−1.5
0
24
SKOV3

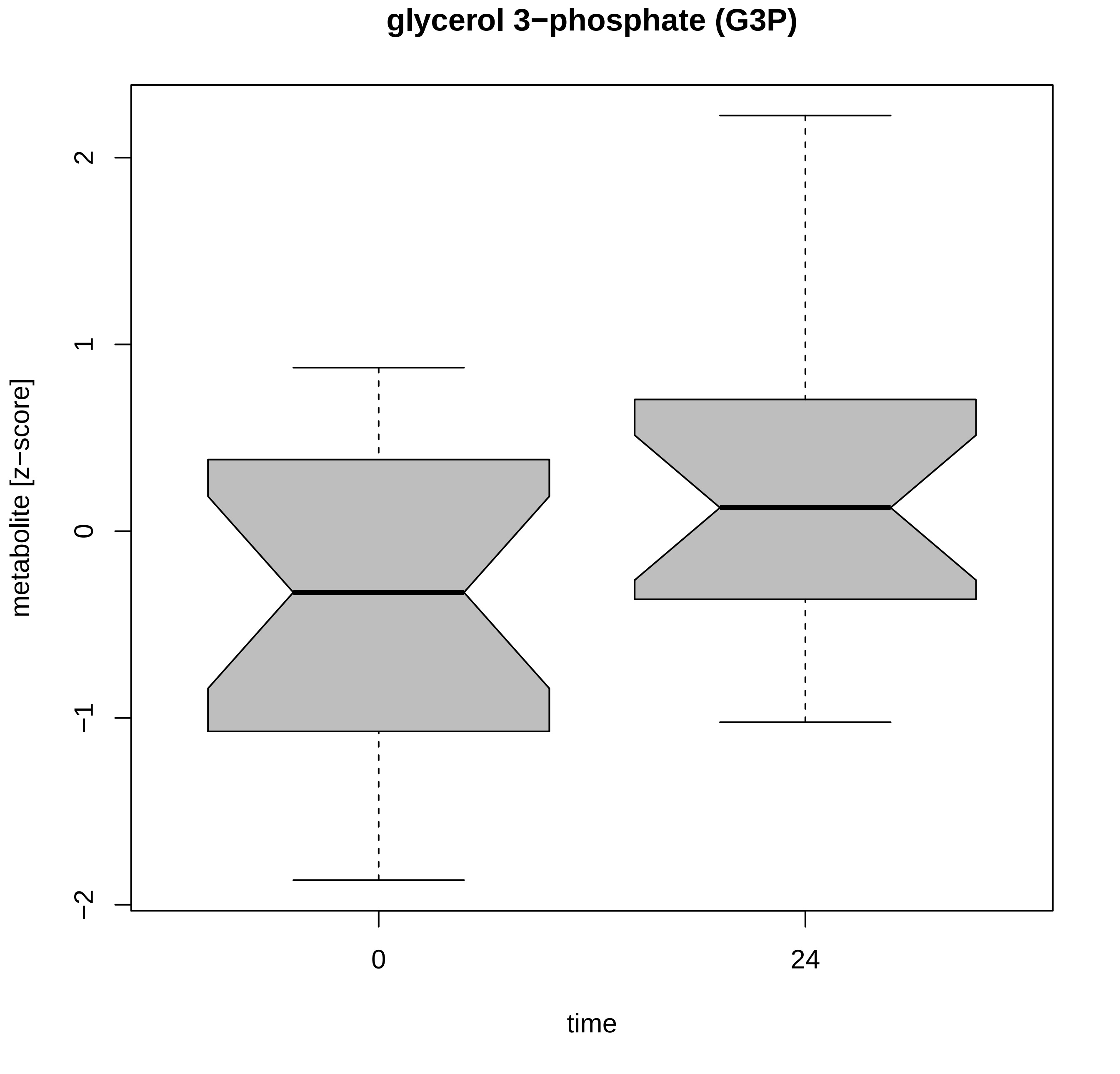
glycerol 3−phosphate (G3P)
metabolite [z−score]
−2
−1
0
1
2
0
24
time

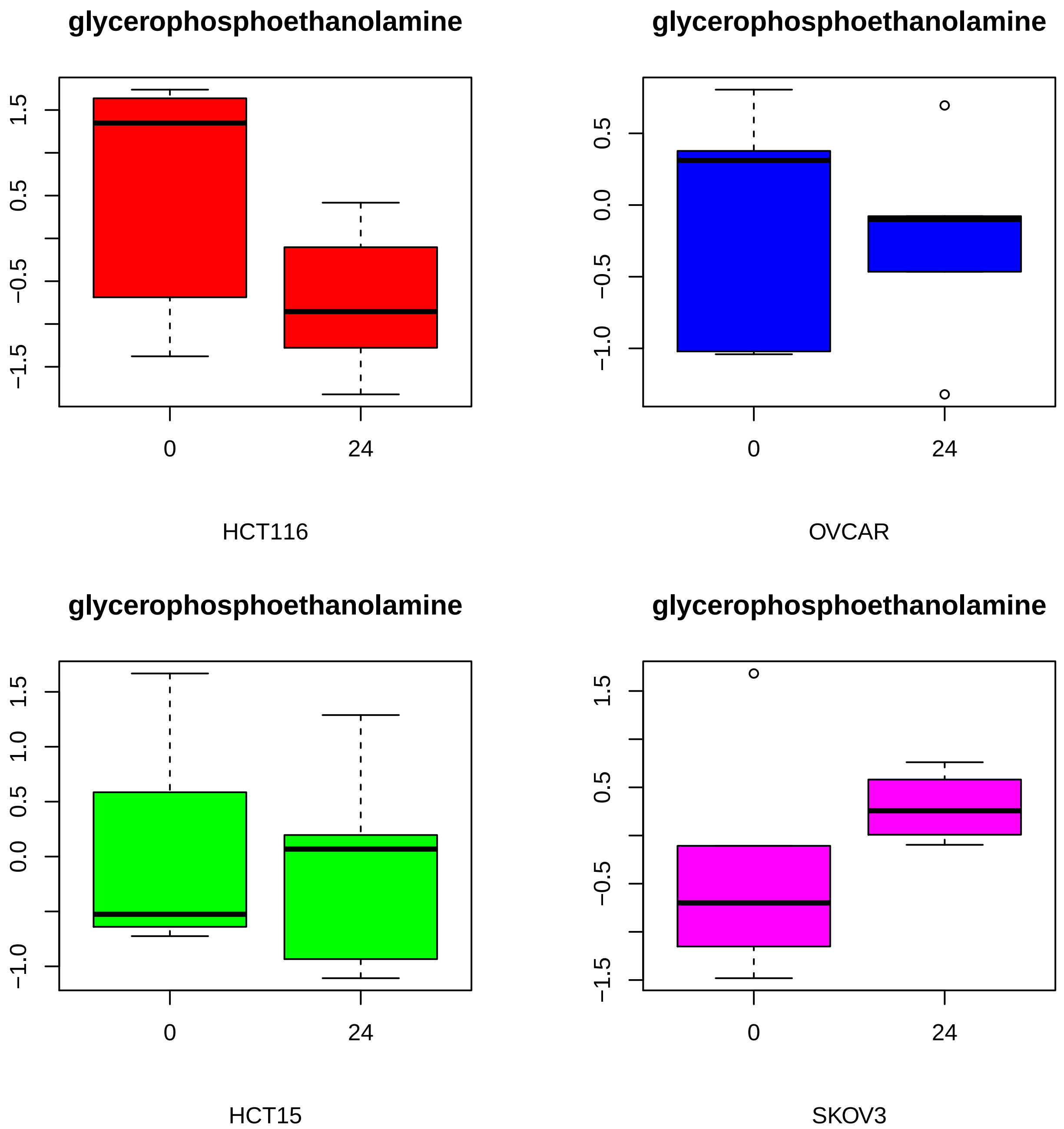
glycerophosphoethanolamine
1.5
0.5
−0.5
−1.5
0
24
HCT116
glycerophosphoethanolamine
0.5
0.0
−0.5
−1.0
0
24
OVCAR
glycerophosphoethanolamine
1.5
1.0
0.5
0.0
−1.0
0
24
HCT15
glycerophosphoethanolamine
1.5
0.5
−0.5
−1.5
0
24
SKOV3

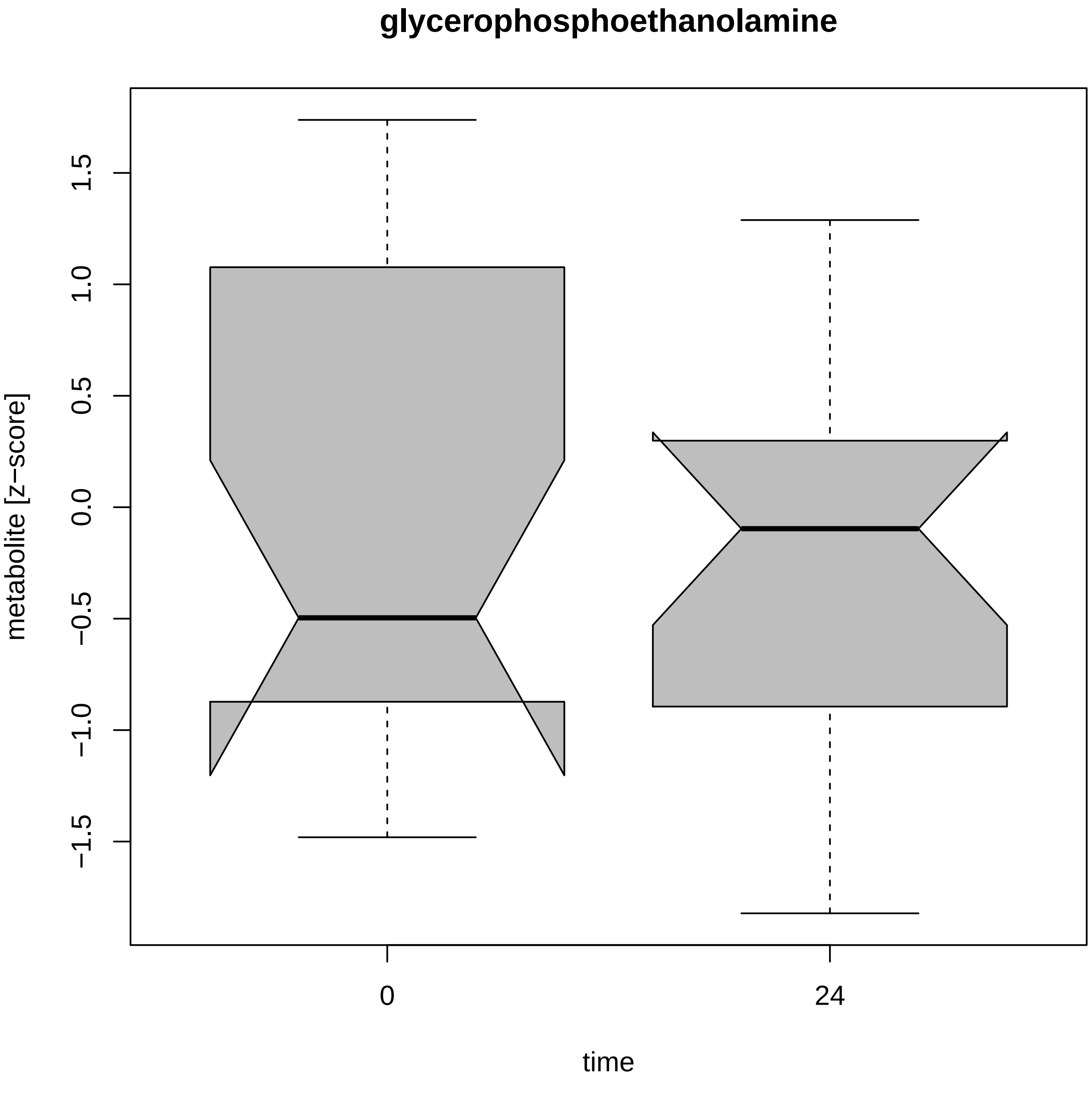
glycerophosphoethanolamine
metabolite [z−score]
1.5
1.0
0.5
0.0
−0.5
−1.0
−1.5
0
24
time

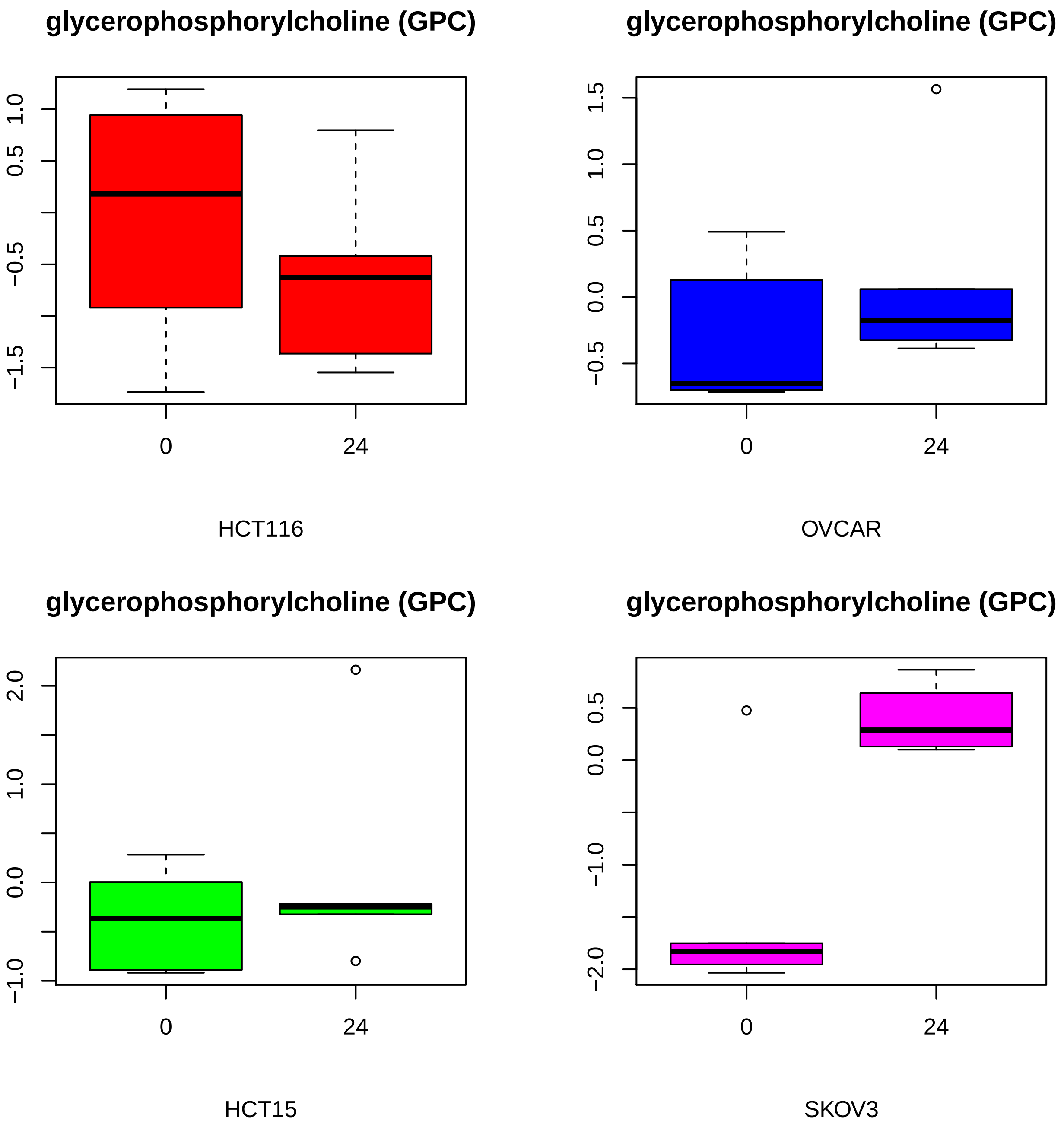
glycerophosphorylcholine (GPC)
1.0
0.5
−0.5
−1.5
0
24
HCT116
glycerophosphorylcholine (GPC)
1.5
1.0
0.5
0.0
−0.5
0
24
OVCAR
glycerophosphorylcholine (GPC)
2.0
1.0
0.0
−1.0
0
24
HCT15
glycerophosphorylcholine (GPC)
0.5
0.0
−1.0
−2.0
0
24
SKOV3

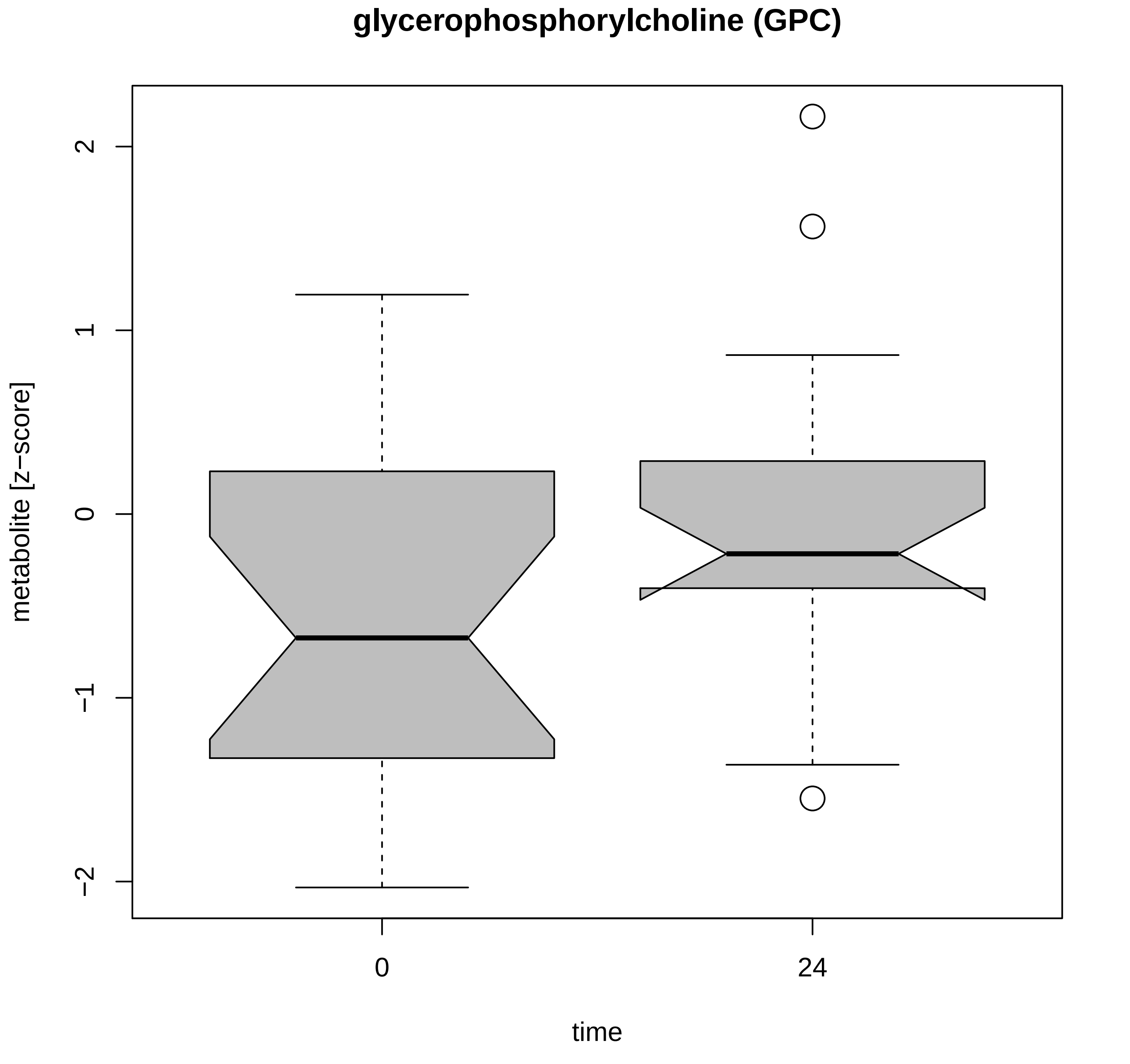

glycerophosphorylcholine (GPC)
metabolite [z−score]
2
1
0
−1
−2
0
24
time

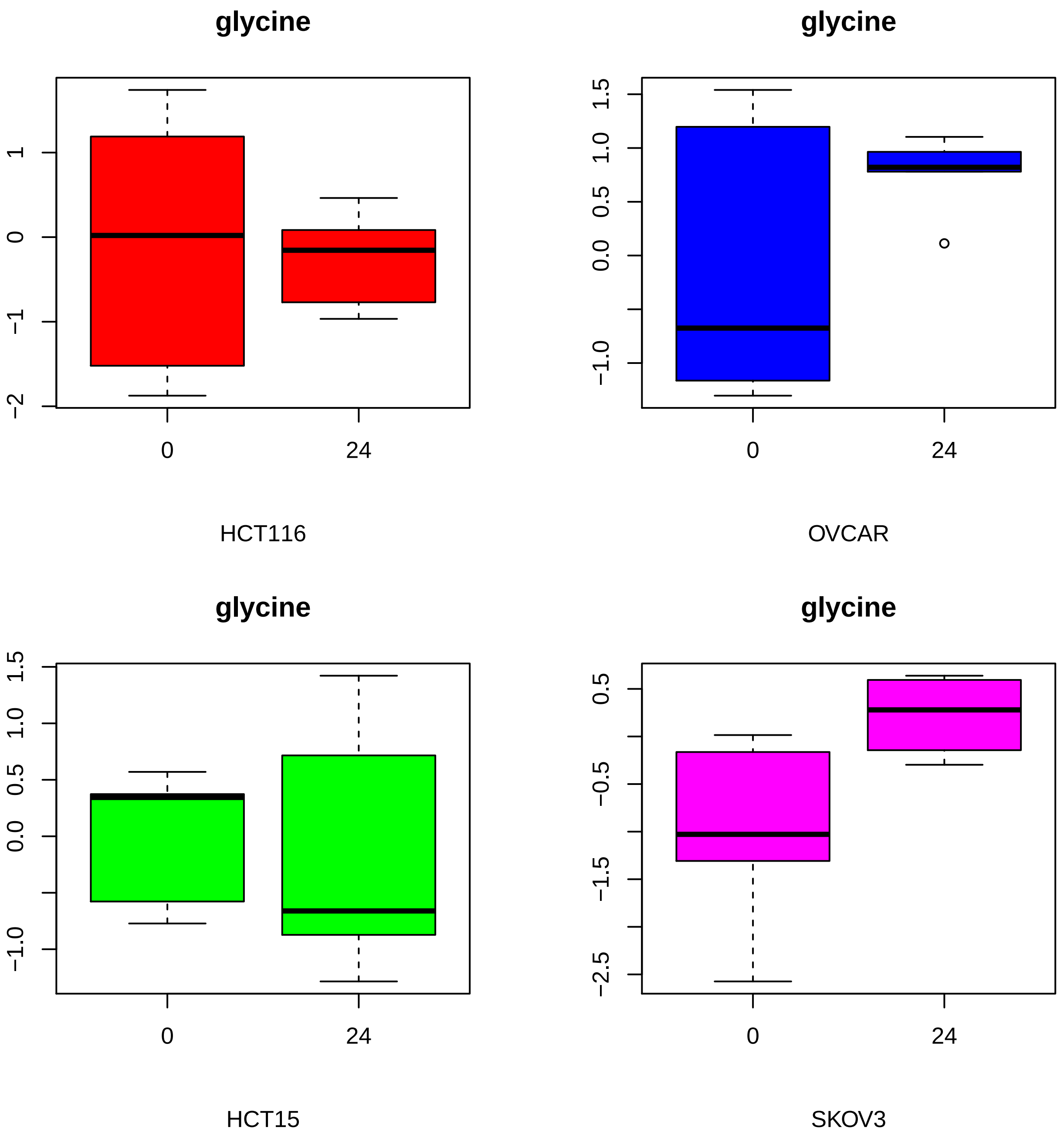
glycine
1
0
−1
−2
0
24
HCT116
glycine
1.5
1.0
0.5
0.0
−1.0
0
24
OVCAR
glycine
1.5
1.0
0.5
0.0
−1.0
0
24
HCT15
glycine
0.5
−0.5
−1.5
−2.5
0
24
SKOV3

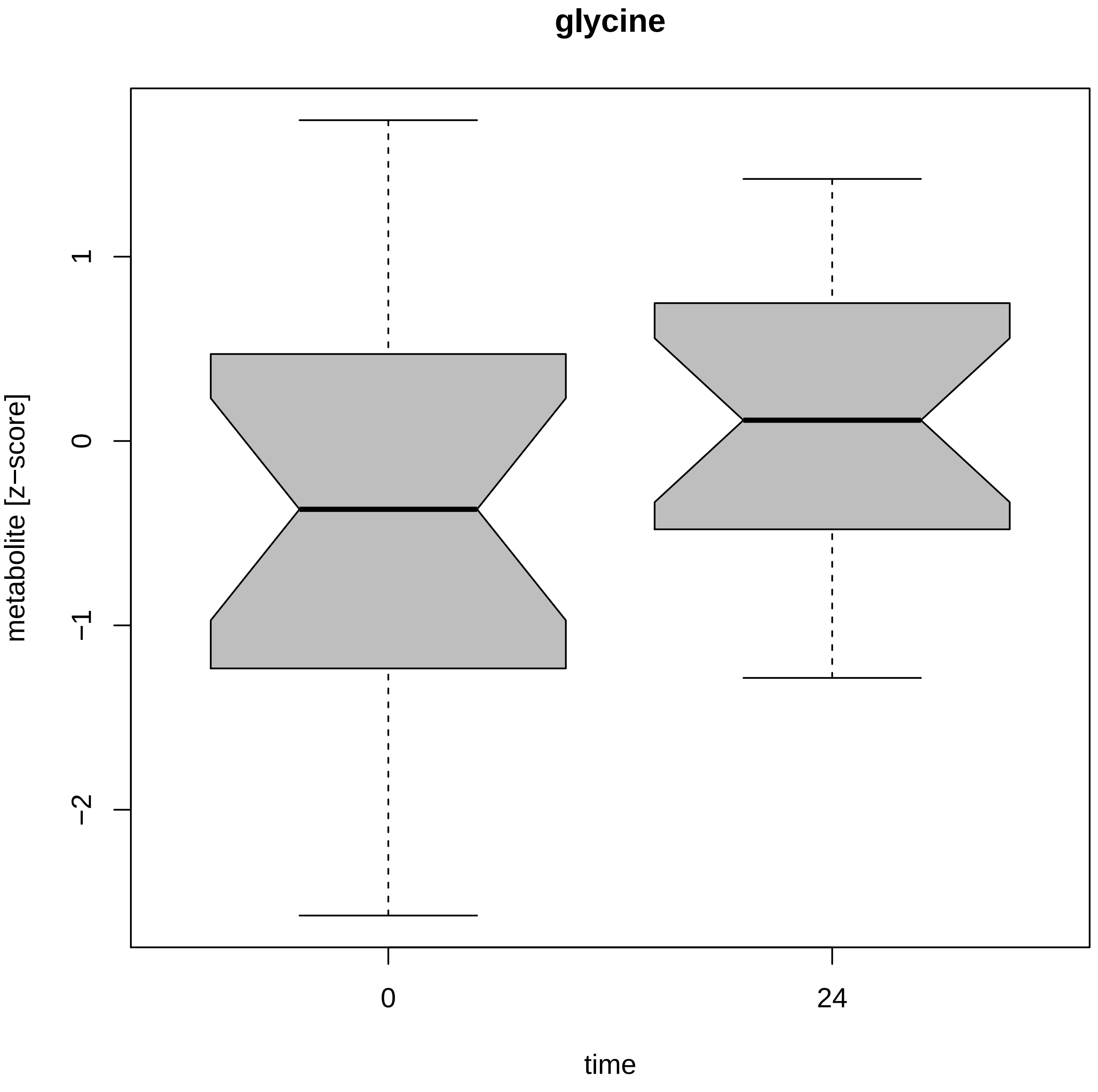
glycine
metabolite [z−score]
1
0
−1
−2
0
24
time

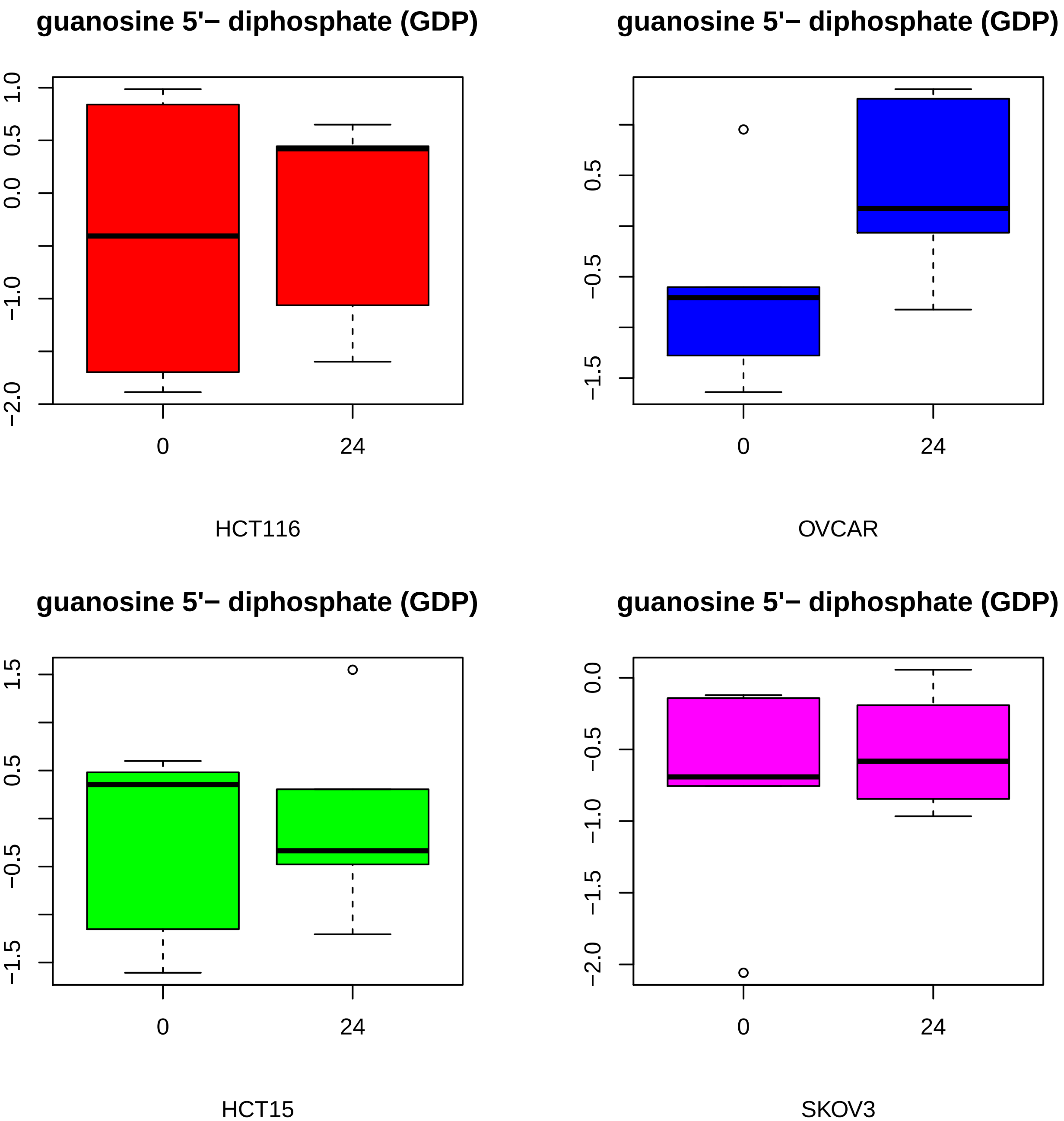
guanosine 5'− diphosphate (GDP)
1.0
0.5
0.0
−1.0
−2.0
0
24
HCT116
guanosine 5'− diphosphate (GDP)
0.5
−0.5
−1.5
0
24
OVCAR
guanosine 5'− diphosphate (GDP)
1.5
0.5
−0.5
−1.5
0
24
HCT15
guanosine 5'− diphosphate (GDP)
0.0
−0.5
−1.0
−1.5
−2.0
0
24
SKOV3

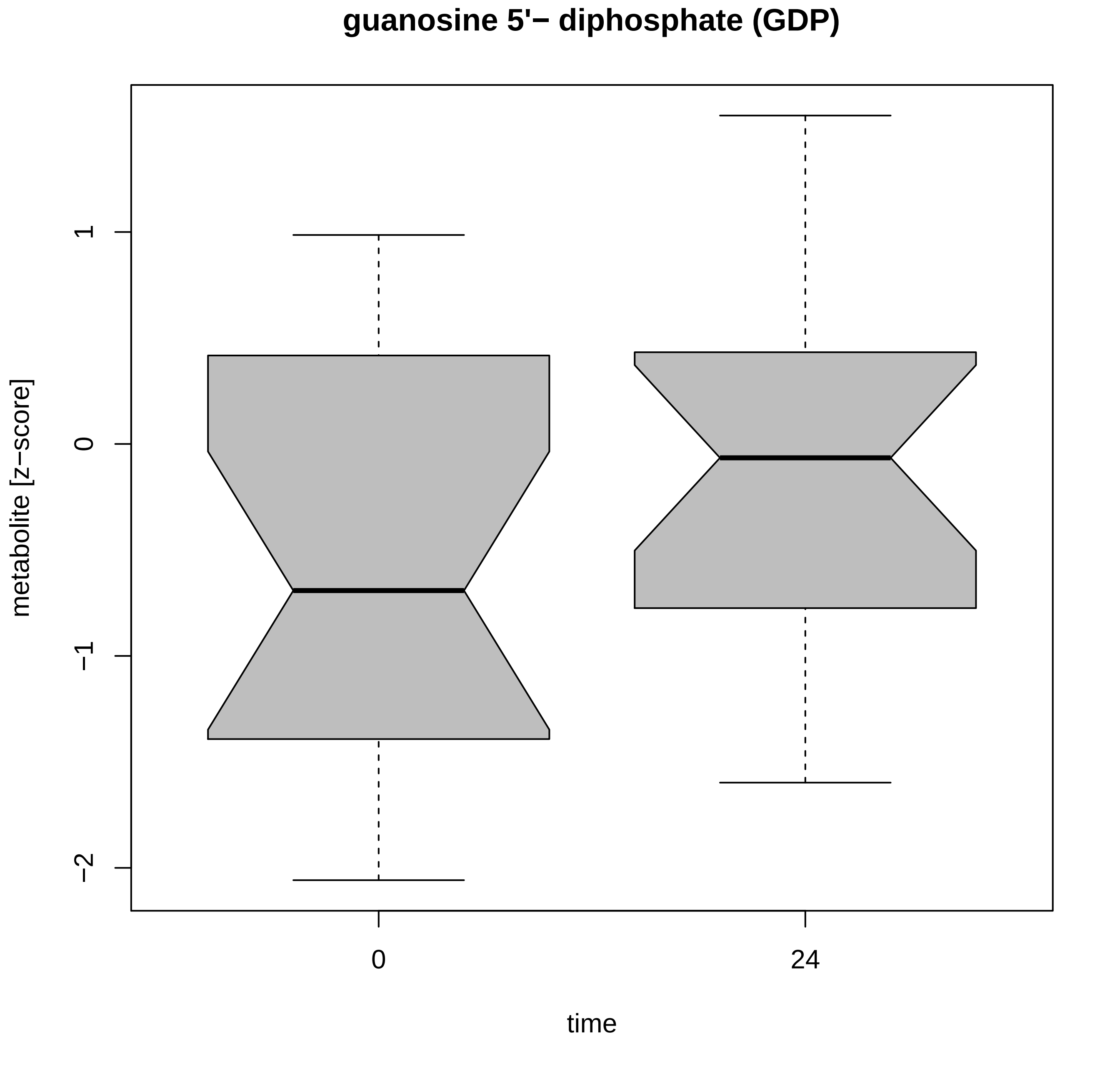
guanosine 5'− diphosphate (GDP)
metabolite [z−score]
1
0
−1
−2
0
24
time

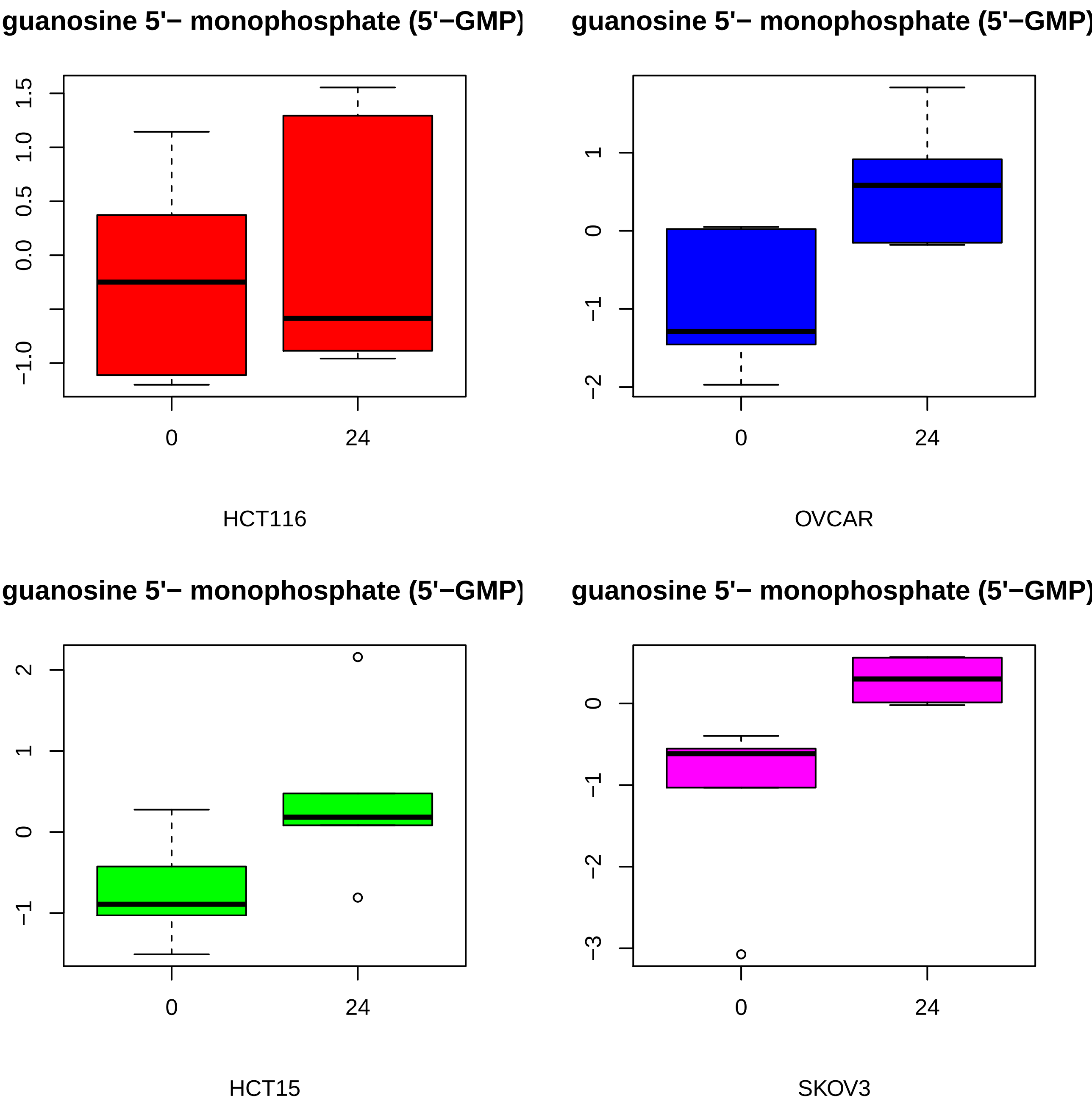
guanosine 5'− monophosphate (5'−GMP)
HCT116
0
24
−1.0
0.0
0.5
1.0
1.5
guanosine 5'− monophosphate (5'−GMP)
OVCAR
0
24
−2
−1
0
1
guanosine 5'− monophosphate (5'−GMP)
HCT15
0
24
−1
0
1
2
guanosine 5'− monophosphate (5'−GMP)
SKOV3
0
24
−3
−2
−1
0

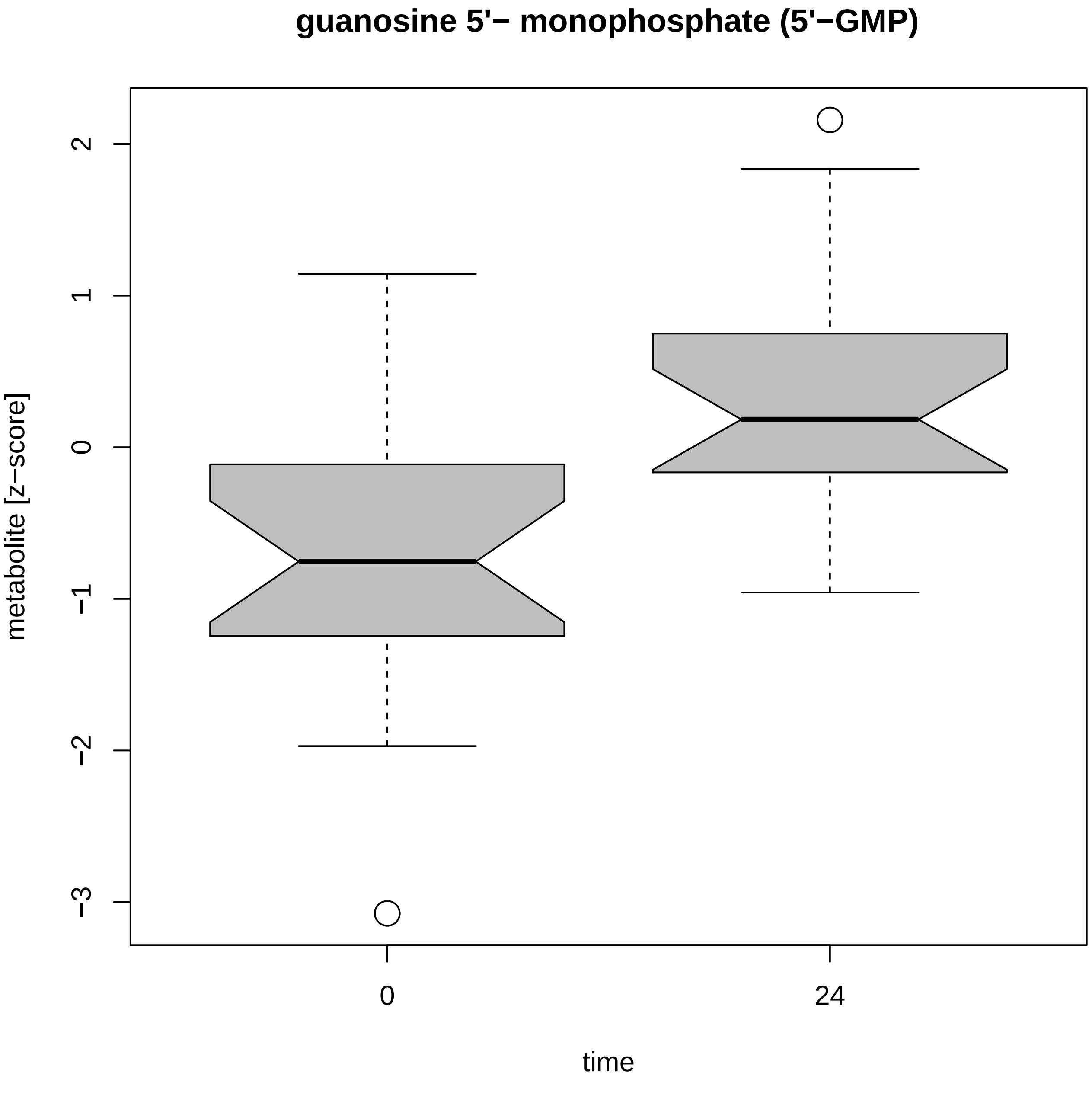

guanosine 5'− monophosphate (5'−GMP)
metabolite [z−score]
2
1
0
−1
−2
−3
0
24
time

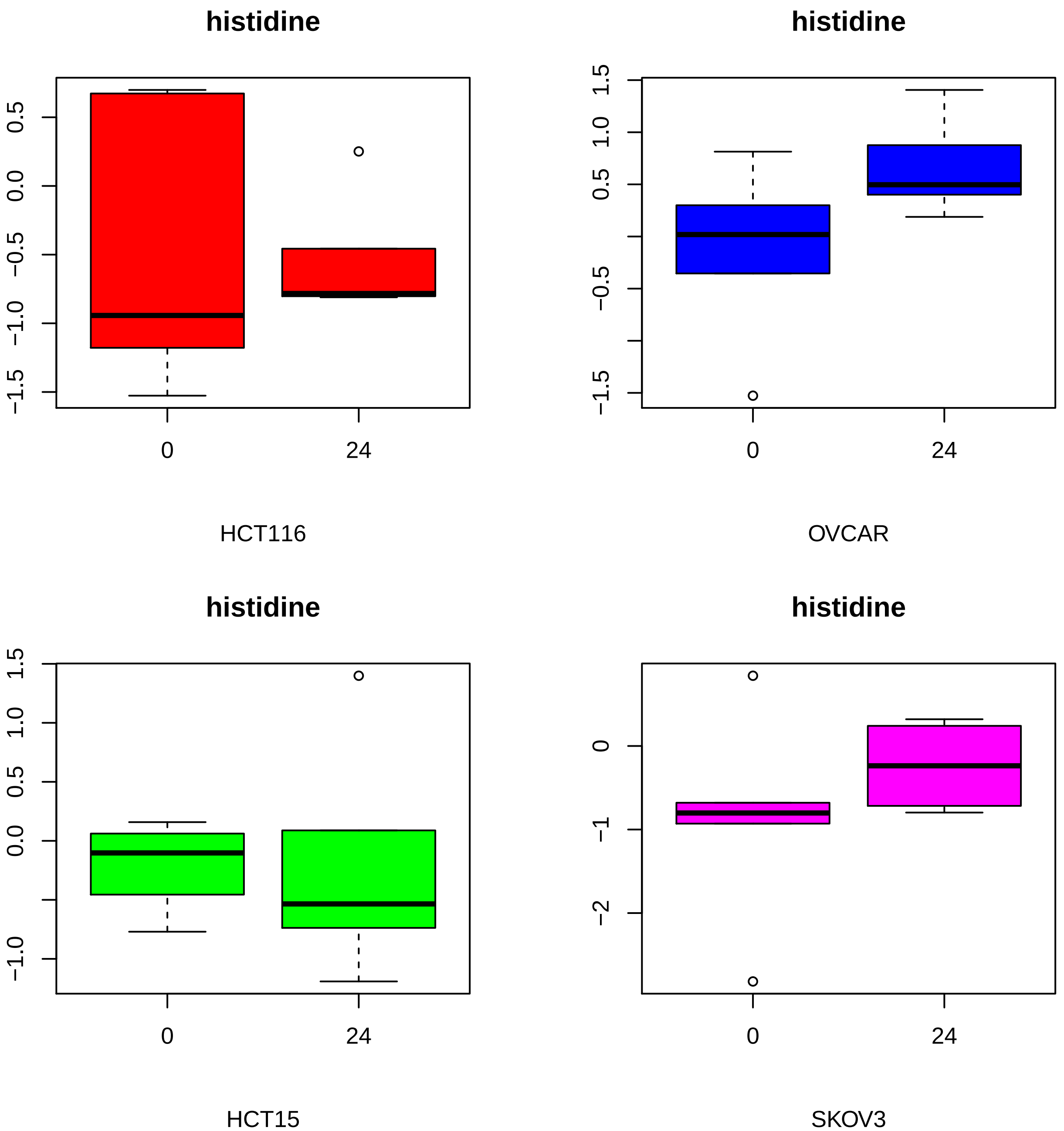
histidine
0.5
0.0
−0.5
−1.0
−1.5
0
24
HCT116
histidine
1.5
1.0
0.5
−0.5
−1.5
0
24
OVCAR
histidine
1.5
1.0
0.5
0.0
−1.0
0
24
HCT15
histidine
0
−1
−2
0
24
SKOV3

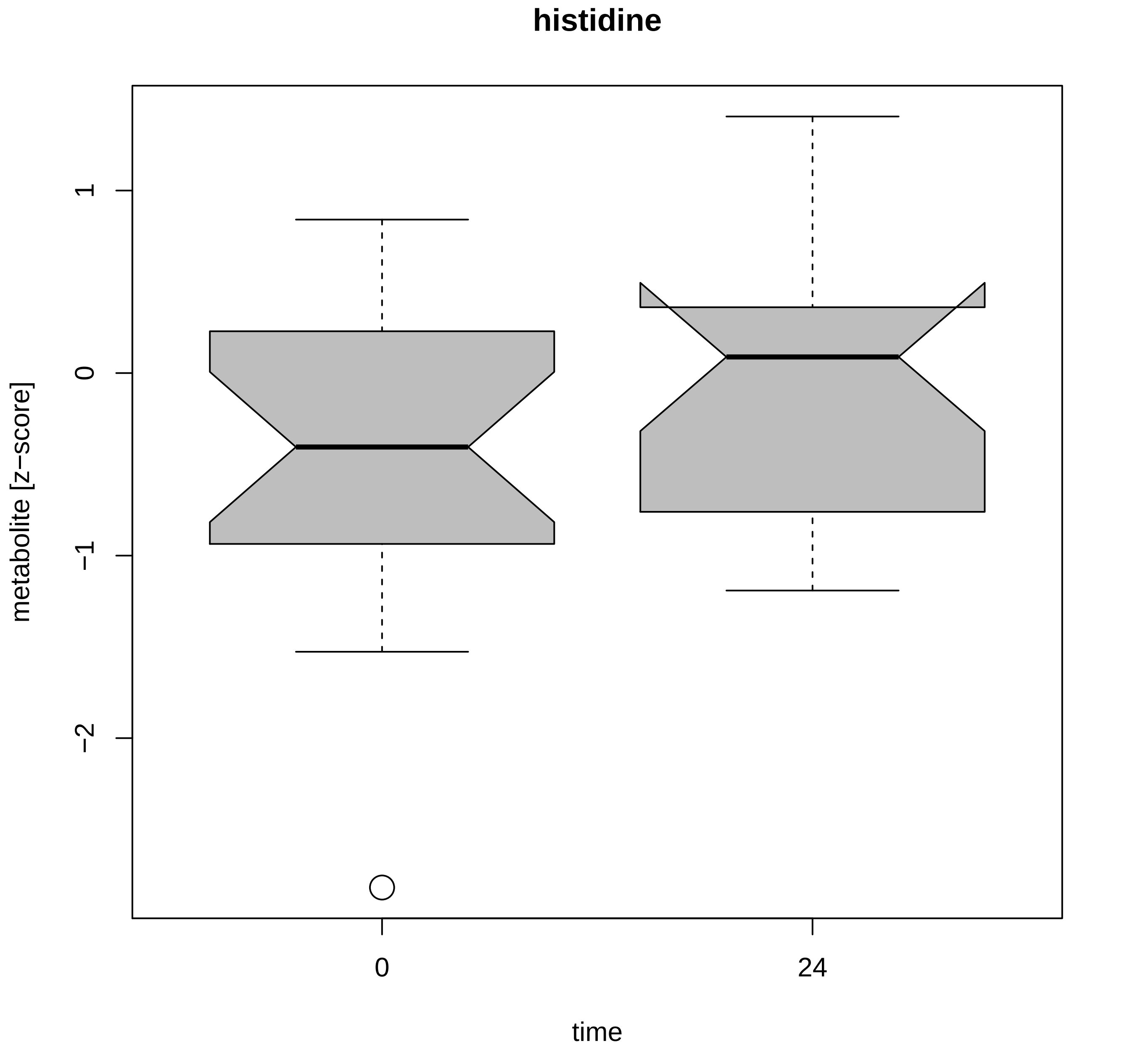
histidine
metabolite [z−score]
1
0
−1
−2
0
24
time

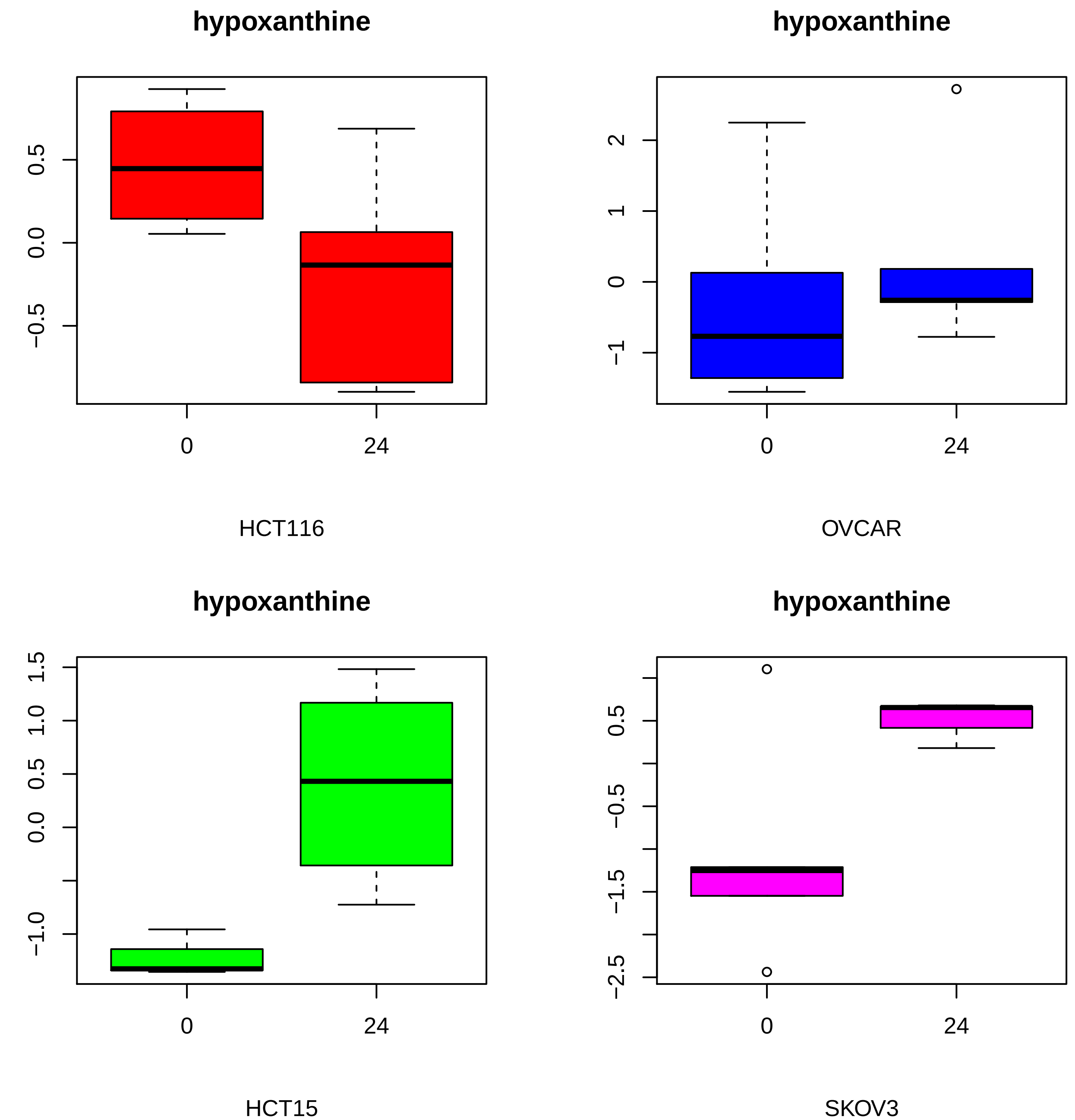
hypoxanthine
0.5
0.0
−0.5
0
24
HCT116
hypoxanthine
2
1
0
−1
0
24
OVCAR
hypoxanthine
1.5
1.0
0.5
0.0
−1.0
0
24
HCT15
hypoxanthine
0.5
−0.5
−1.5
−2.5
0
24
SKOV3

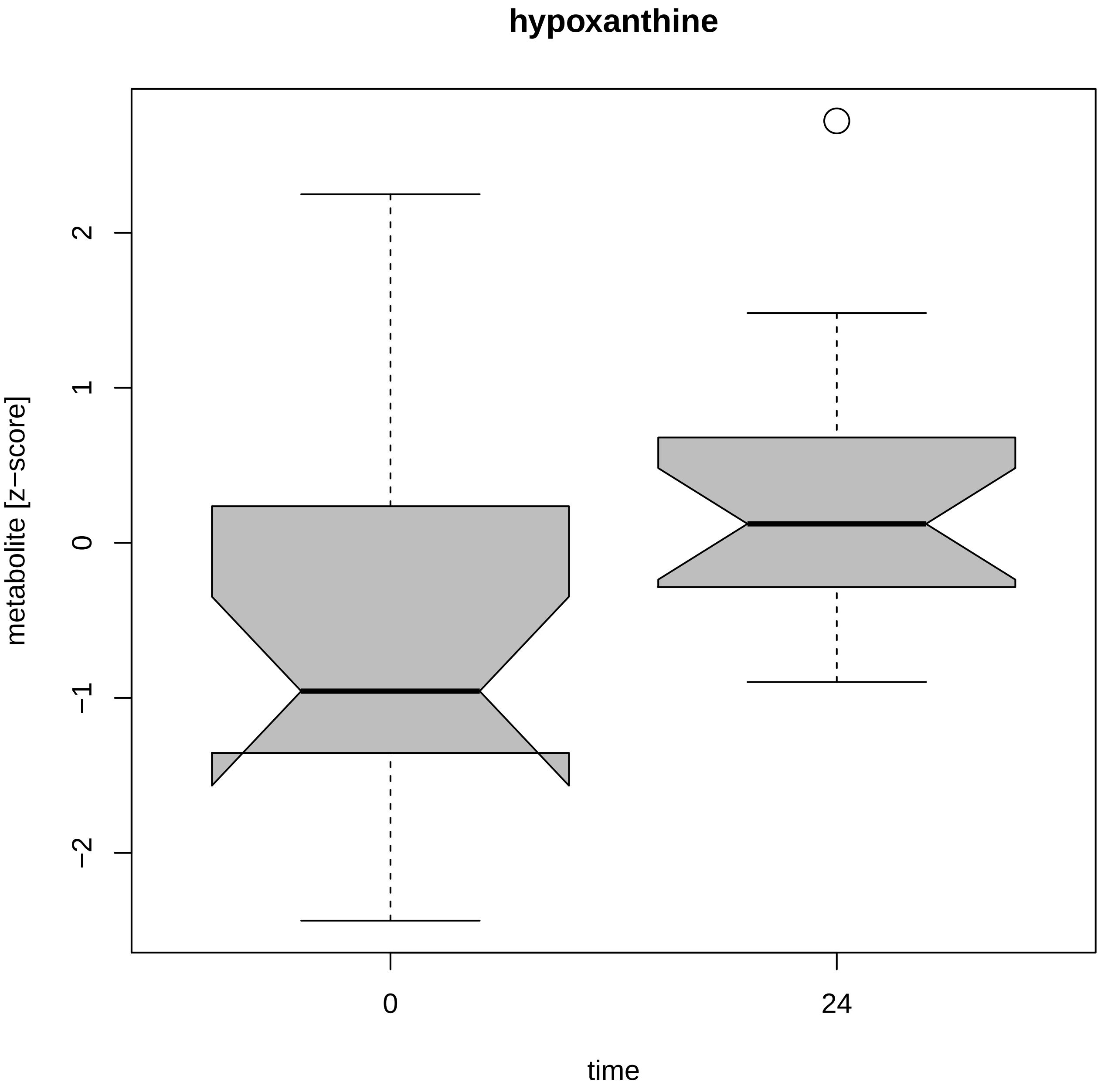
hypoxanthine
metabolite [z−score]
−2
−1
0
1
2
0
24
time

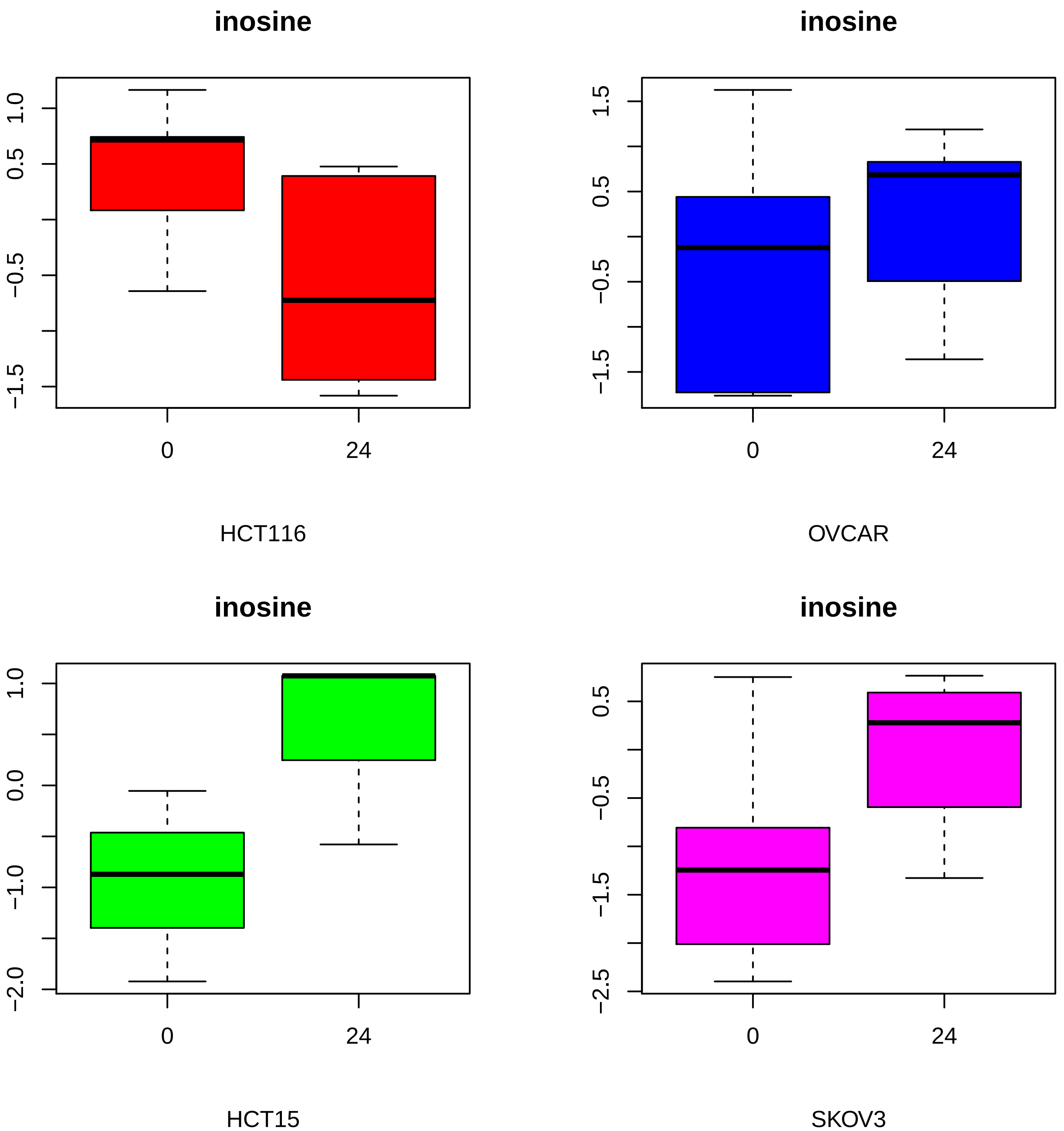
inosine
1.0
0.5
−0.5
−1.5
0
24
HCT116
inosine
1.5
0.5
−0.5
−1.5
0
24
OVCAR
inosine
1.0
0.0
−1.0
−2.0
0
24
HCT15
inosine
0.5
−0.5
−1.5
−2.5
0
24
SKOV3

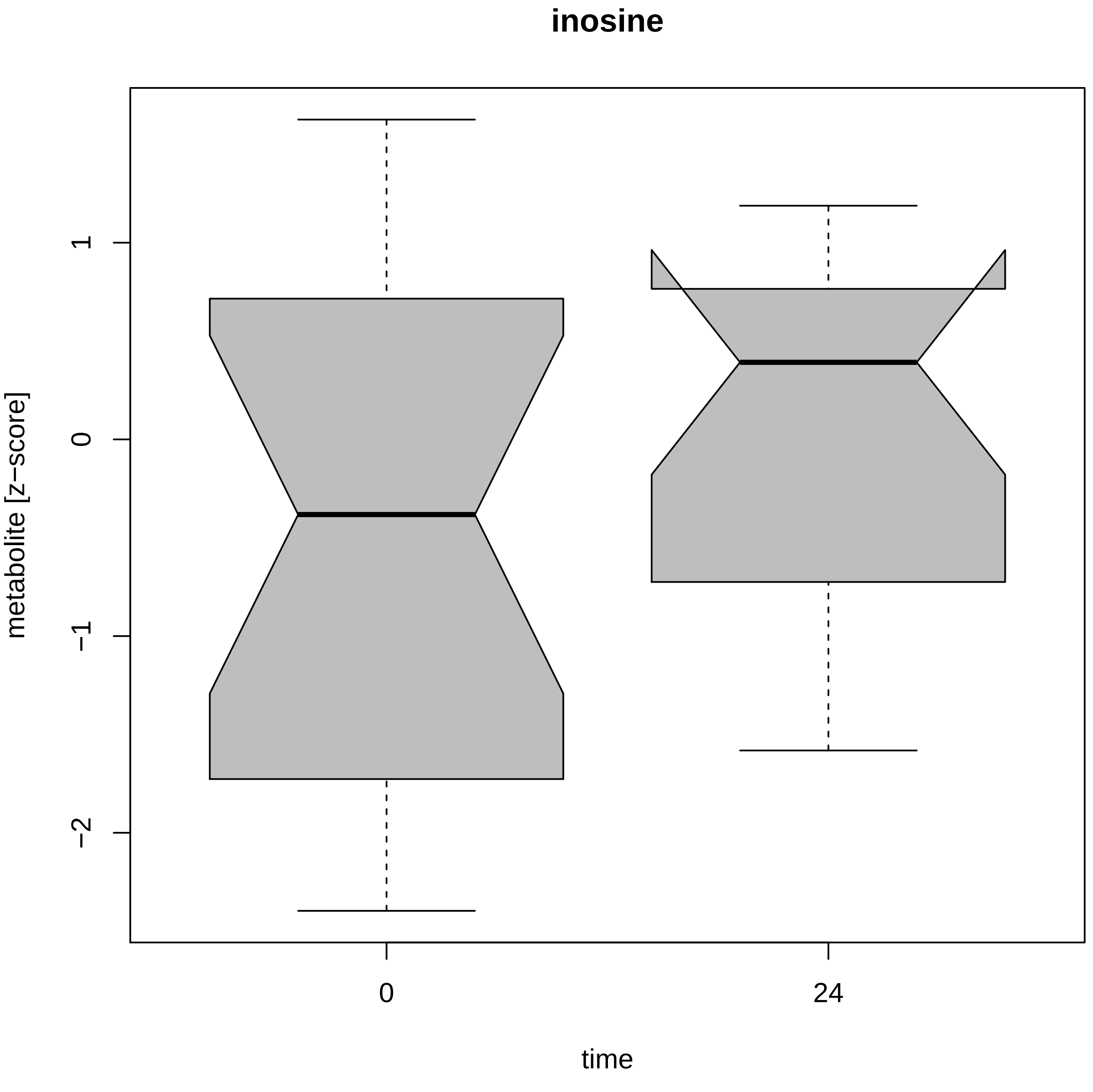
inosine
metabolite [z−score]
1
0
−1
−2
0
24
time

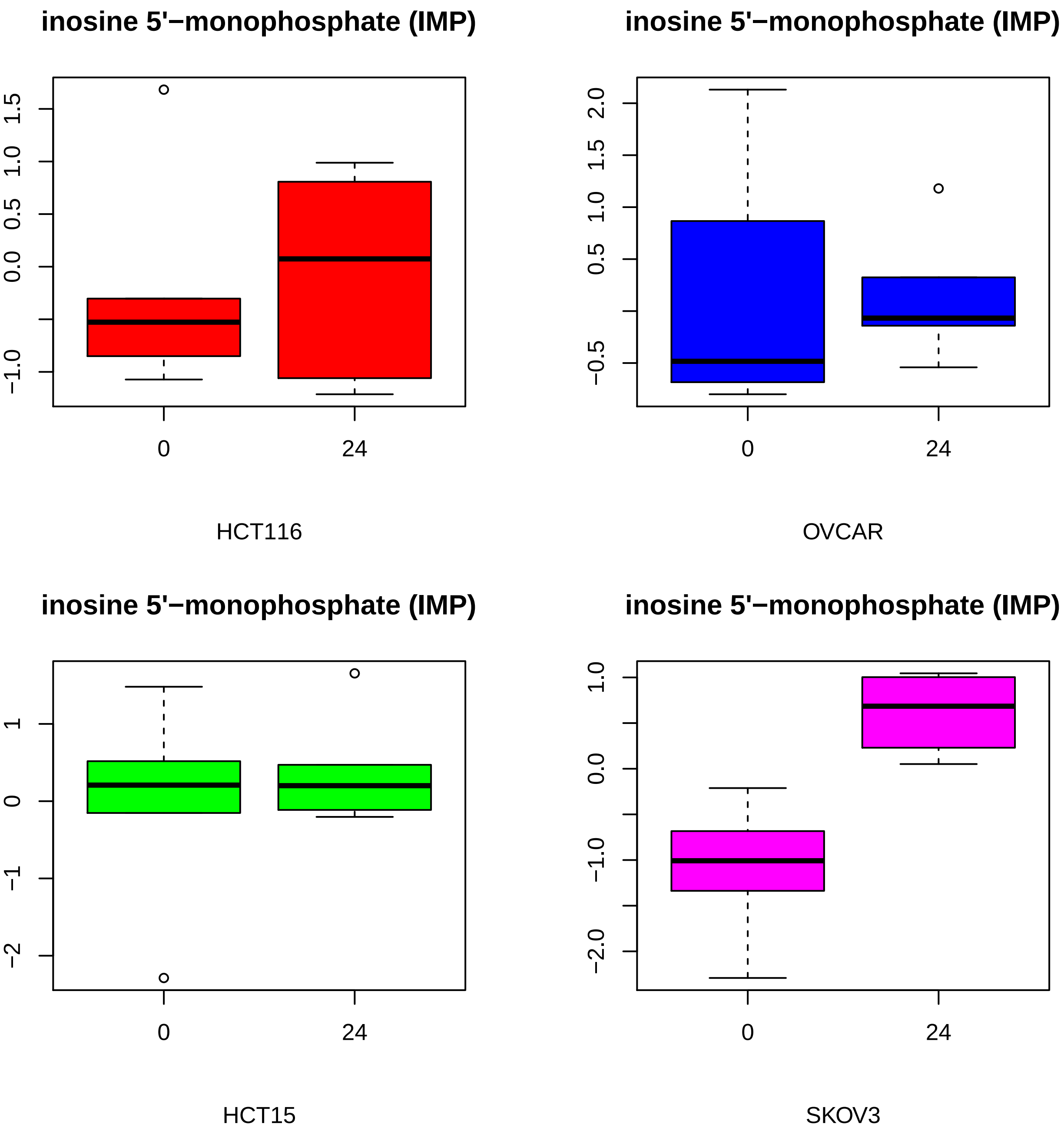
inosine 5'−monophosphate (IMP)
1.5
1.0
0.5
0.0
−1.0
0
24
HCT116
inosine 5'−monophosphate (IMP)
2.0
1.5
1.0
0.5
−0.5
0
24
OVCAR
inosine 5'−monophosphate (IMP)
1
0
−1
−2
0
24
HCT15
inosine 5'−monophosphate (IMP)
1.0
0.0
−1.0
−2.0
0
24
SKOV3

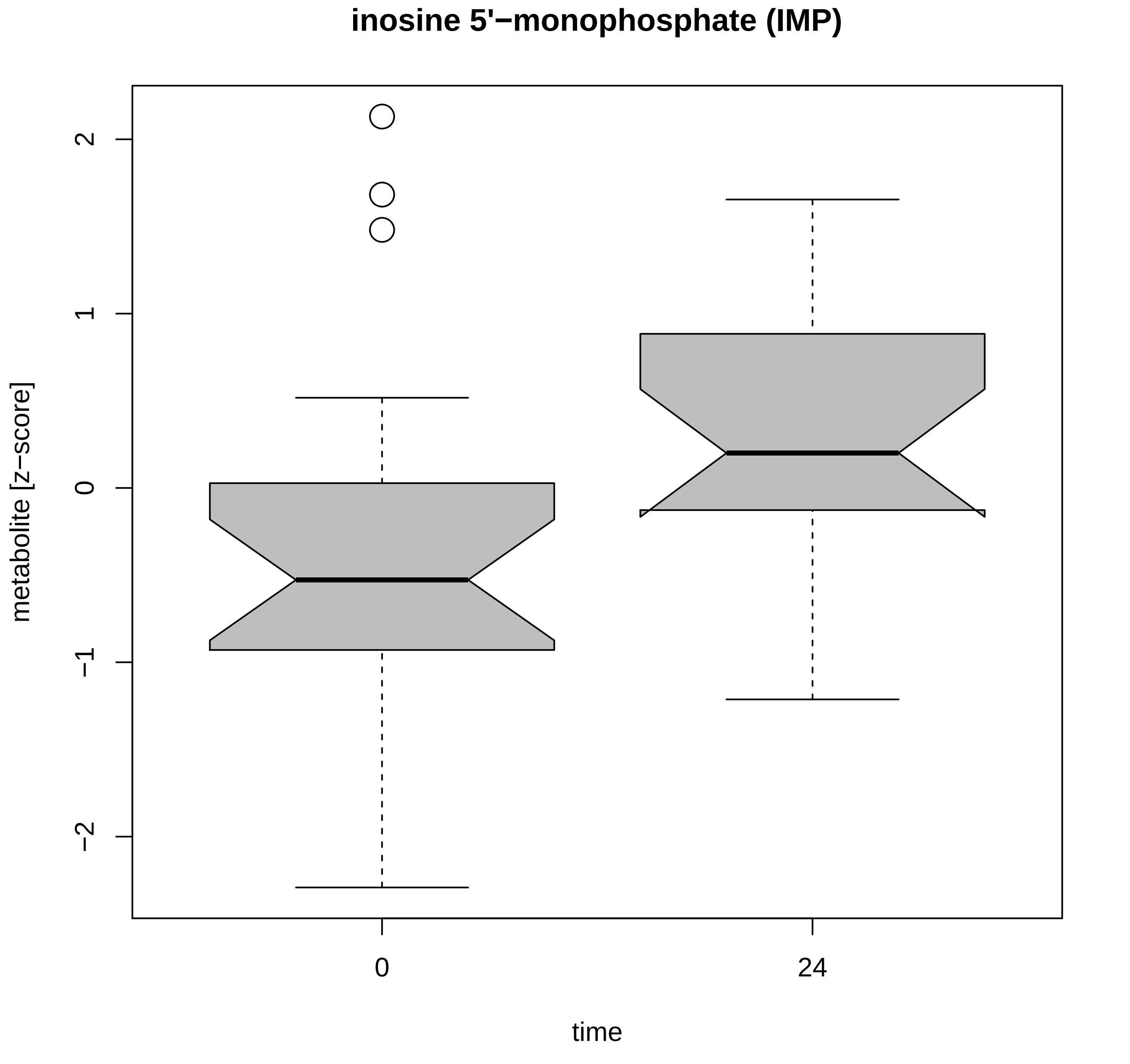
inosine 5'−monophosphate (IMP)
metabolite [z−score]
2
1
0
−1
−2
0
24
time

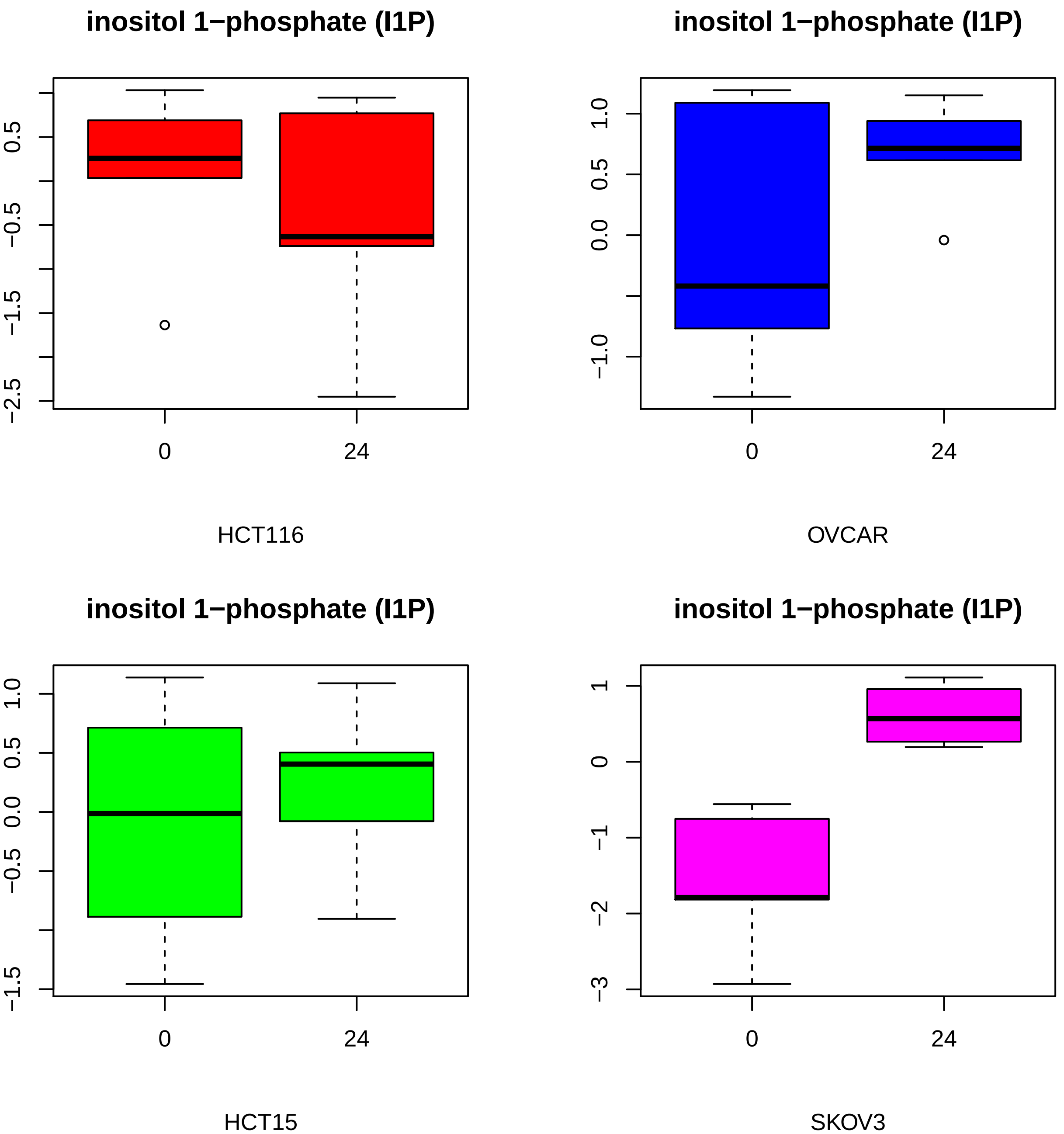

inositol 1−phosphate (I1P)
0.5
−0.5
−1.5
−2.5
0
24
HCT116
inositol 1−phosphate (I1P)
1.0
0.5
0.0
−1.0
0
24
OVCAR
inositol 1−phosphate (I1P)
1.0
0.5
0.0
−0.5
−1.5
0
24
HCT15
inositol 1−phosphate (I1P)
1
0
−1
−2
−3
0
24
SKOV3

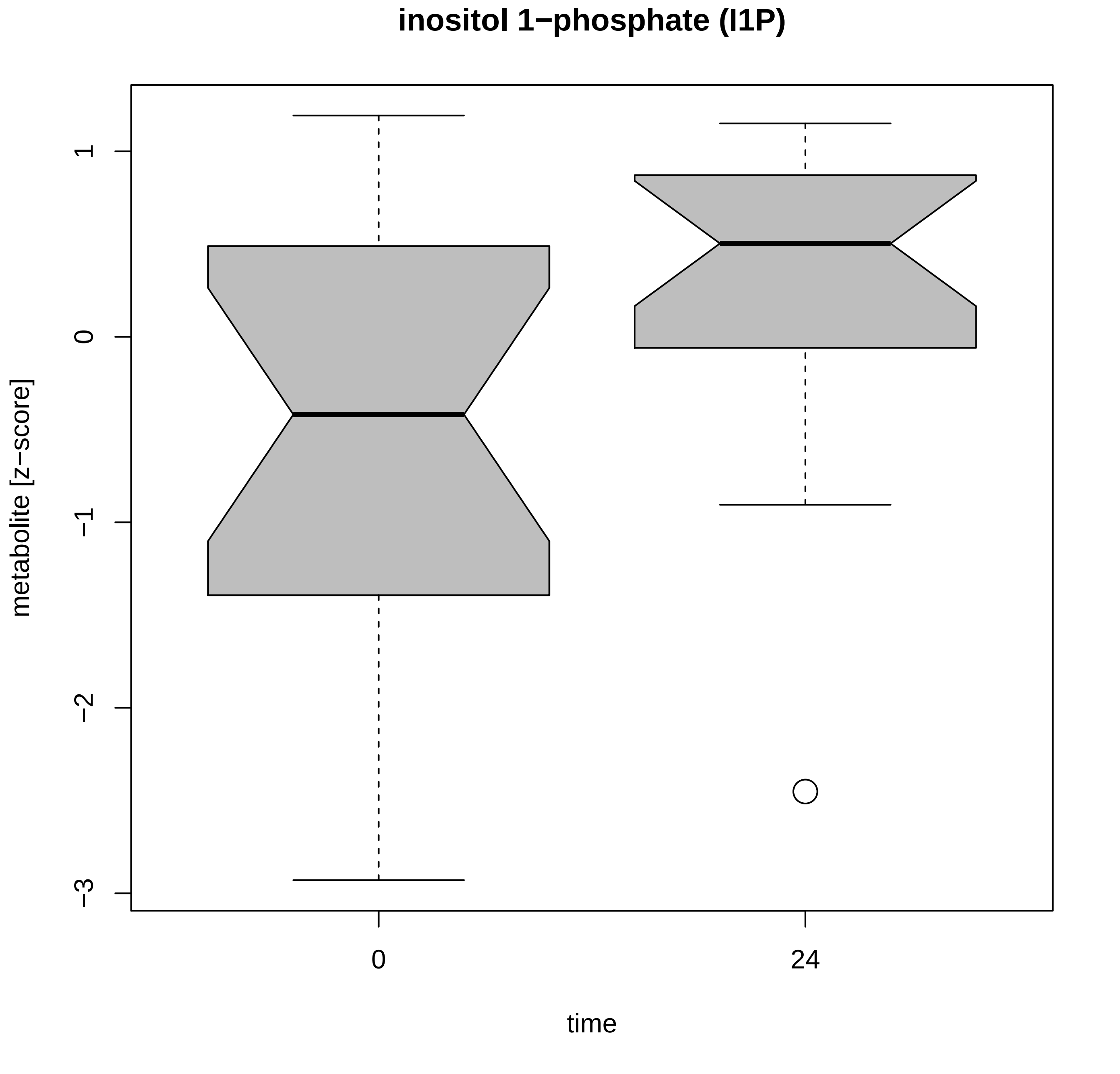

inositol 1−phosphate (I1P)
metabolite [z−score]
1
0
−1
−2
−3
0
24
time

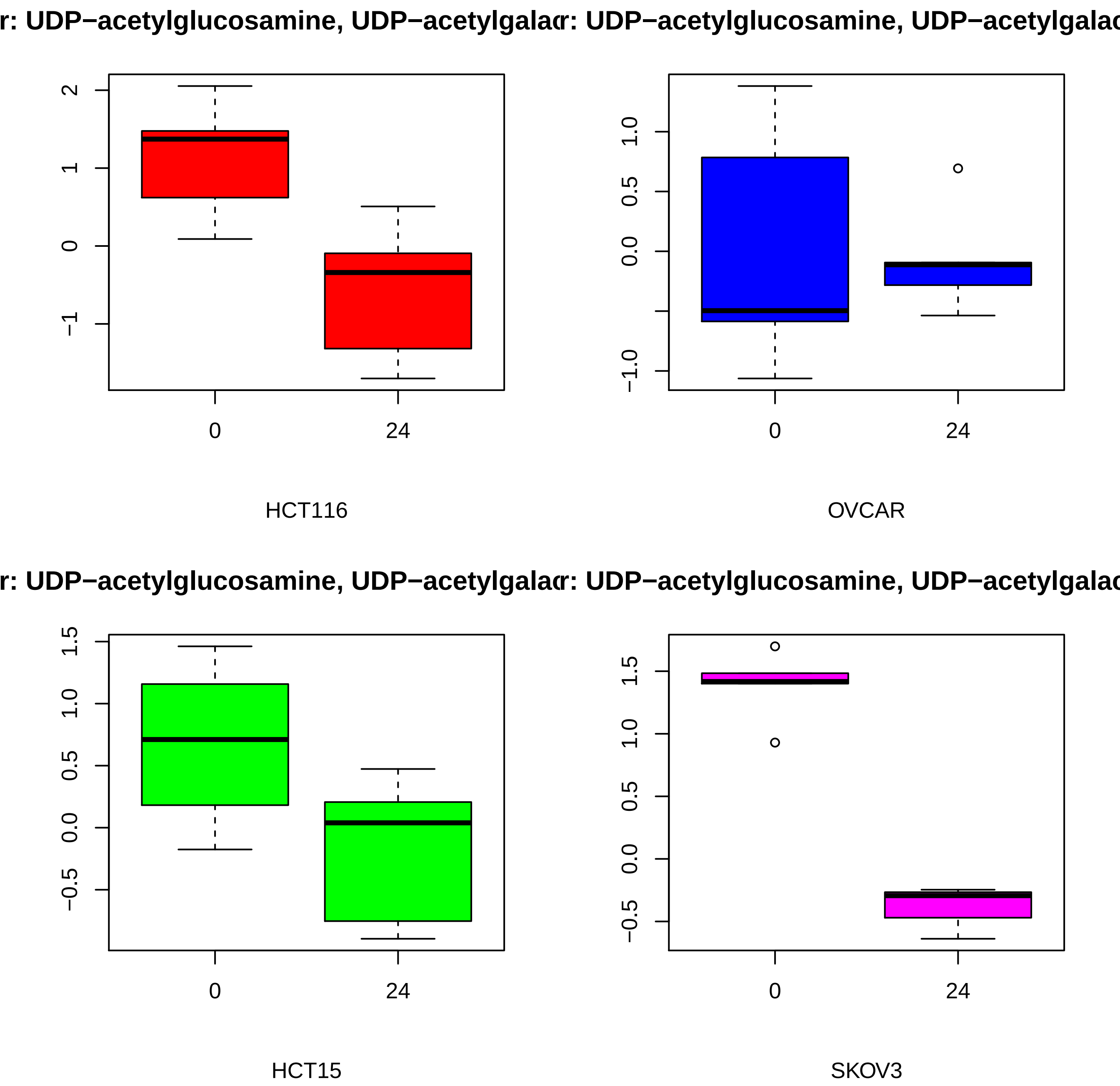
r: UDP−acetylglucosamine, UDP−acetylgala
r: UDP−acetylglucosamine, UDP−acetylgalac
2
1
0
−1
0
24
HCT116
1.0
0.5
0.0
−1.0
0
24
OVCAR
r: UDP−acetylglucosamine, UDP−acetylgala
r: UDP−acetylglucosamine, UDP−acetylgalac
1.5
1.0
0.5
0.0
−0.5
0
24
HCT15
1.5
1.0
0.5
0.0
−0.5
0
24
SKOV3

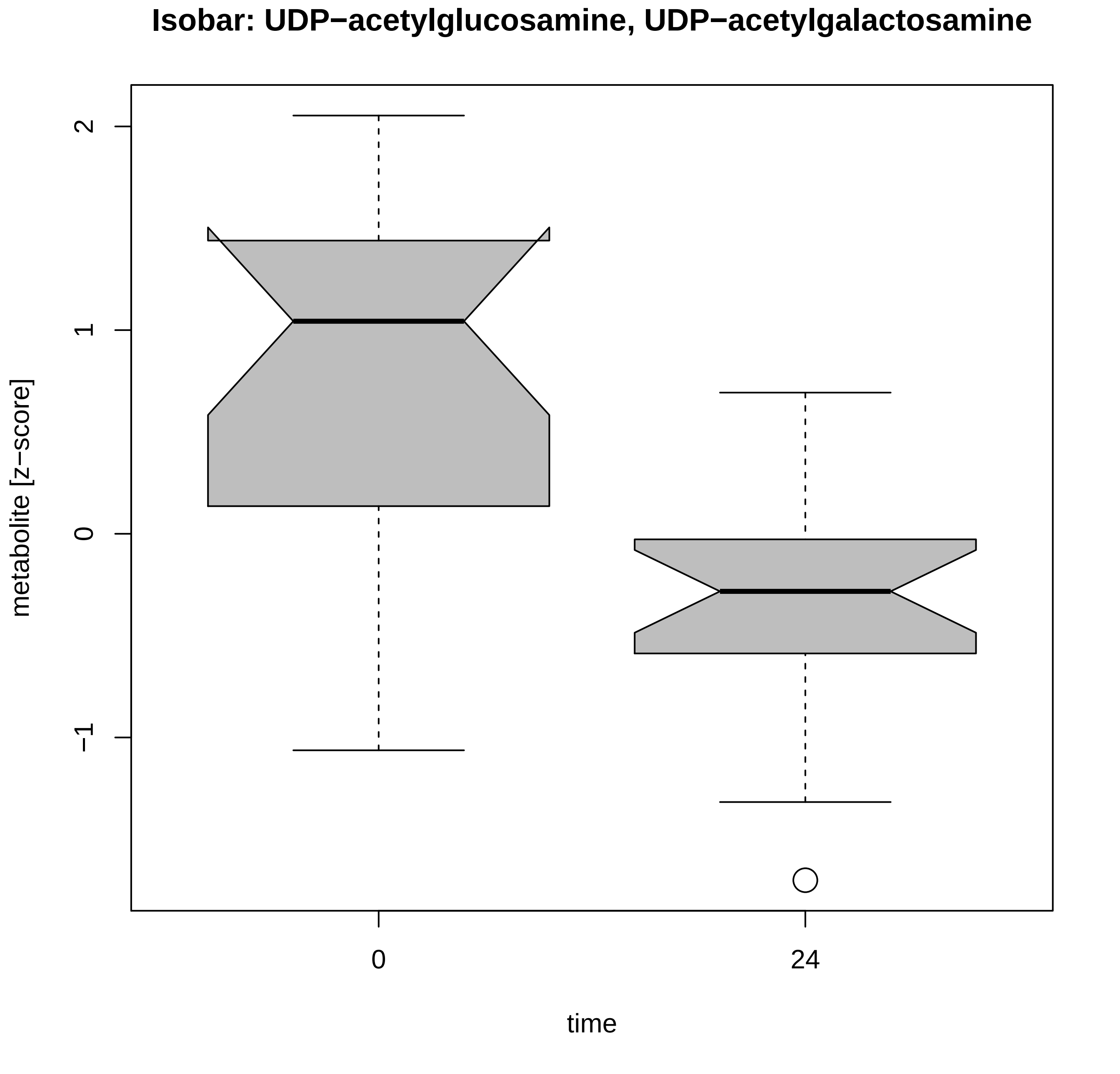

Isobar: UDP−acetylglucosamine, UDP−acetylgalactosamine
metabolite [z−score]
2
1
0
−1
0
24
time

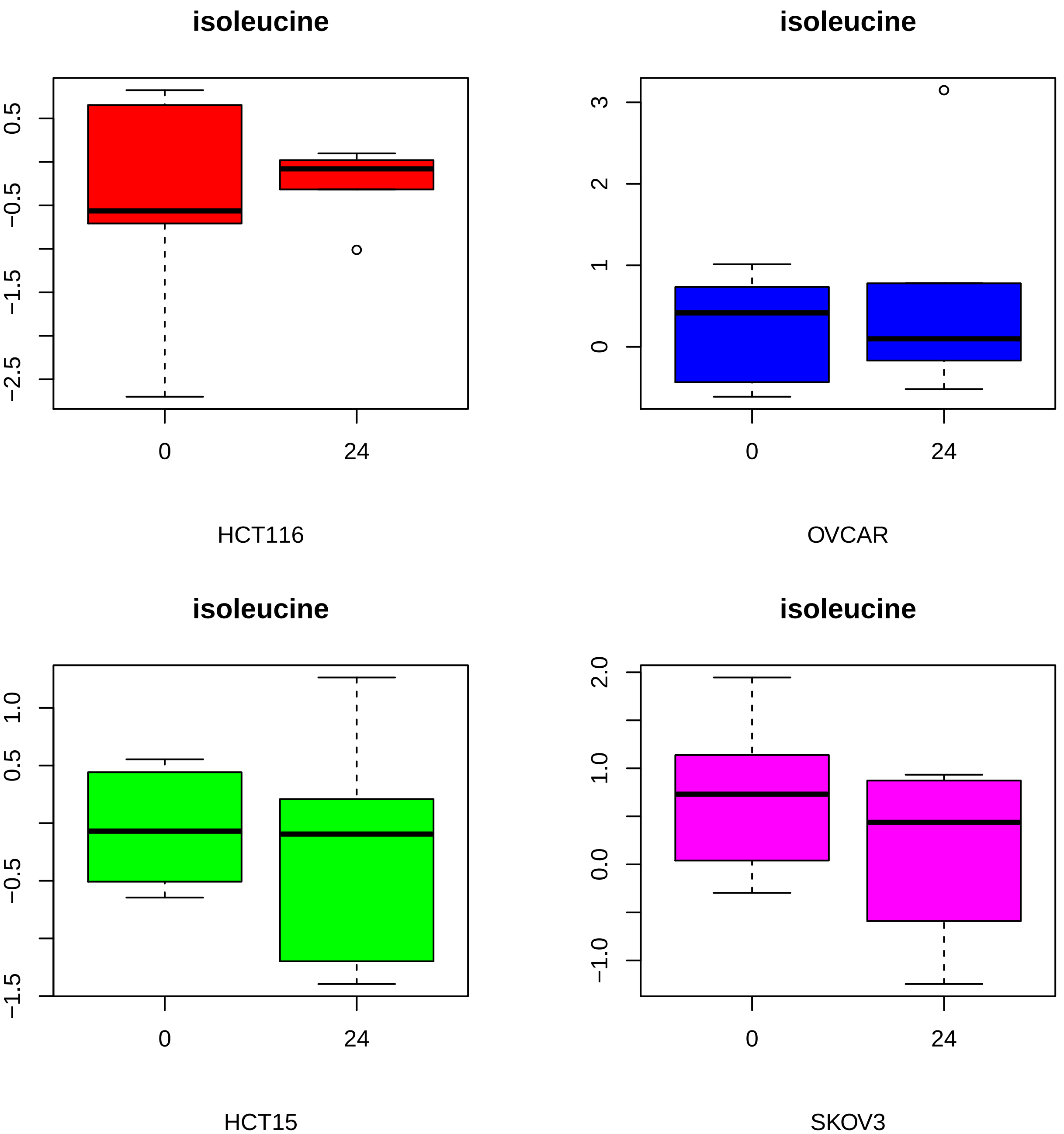
isoleucine
0.5
−0.5
−1.5
−2.5
0
24
HCT116
isoleucine
3
2
1
0
0
24
OVCAR
isoleucine
1.0
0.5
−0.5
−1.5
0
24
HCT15
isoleucine
2.0
1.0
0.0
−1.0
0
24
SKOV3

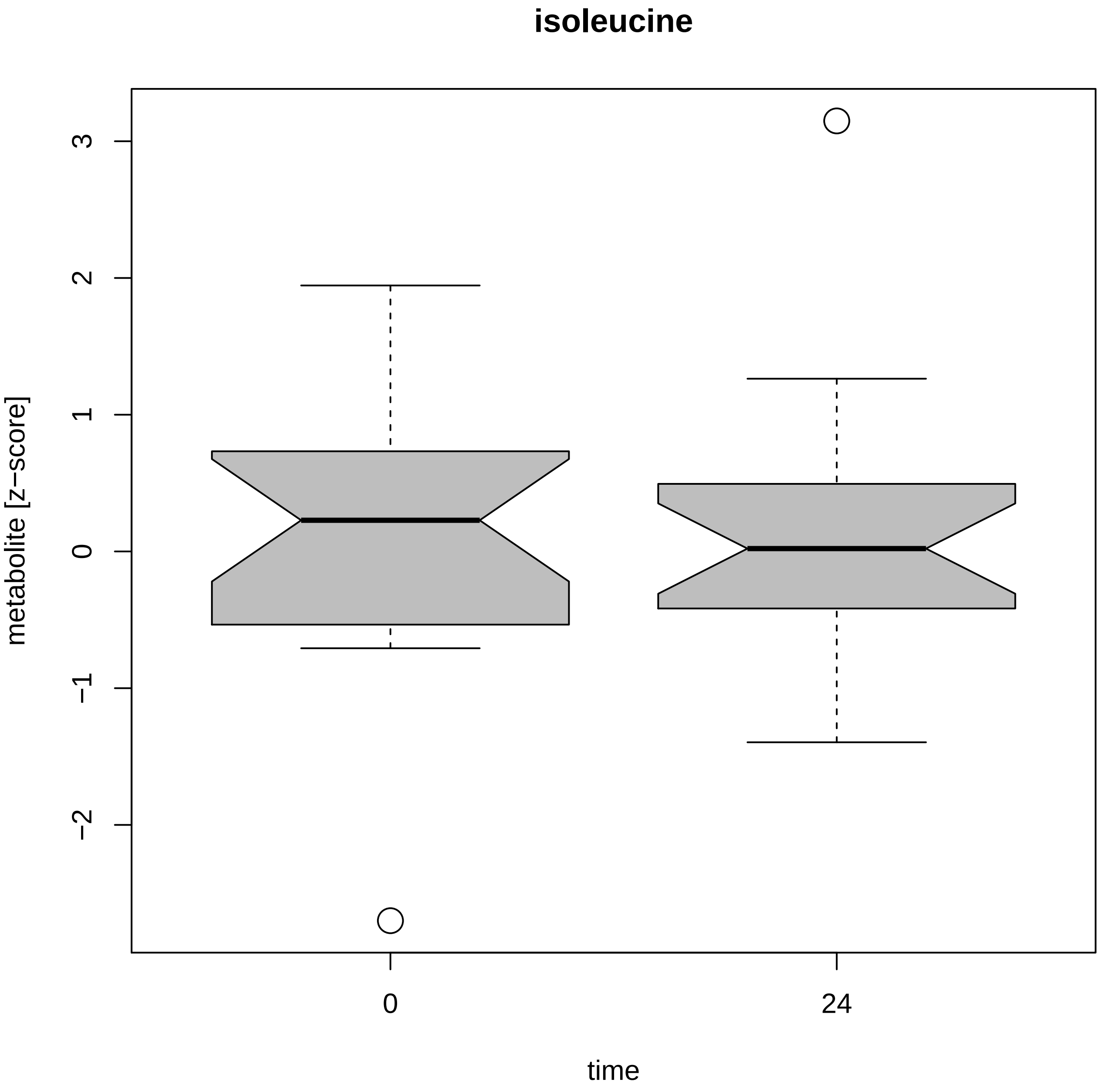
isoleucine
metabolite [z−score]
3
2
1
0
−1
−2
0
24
time

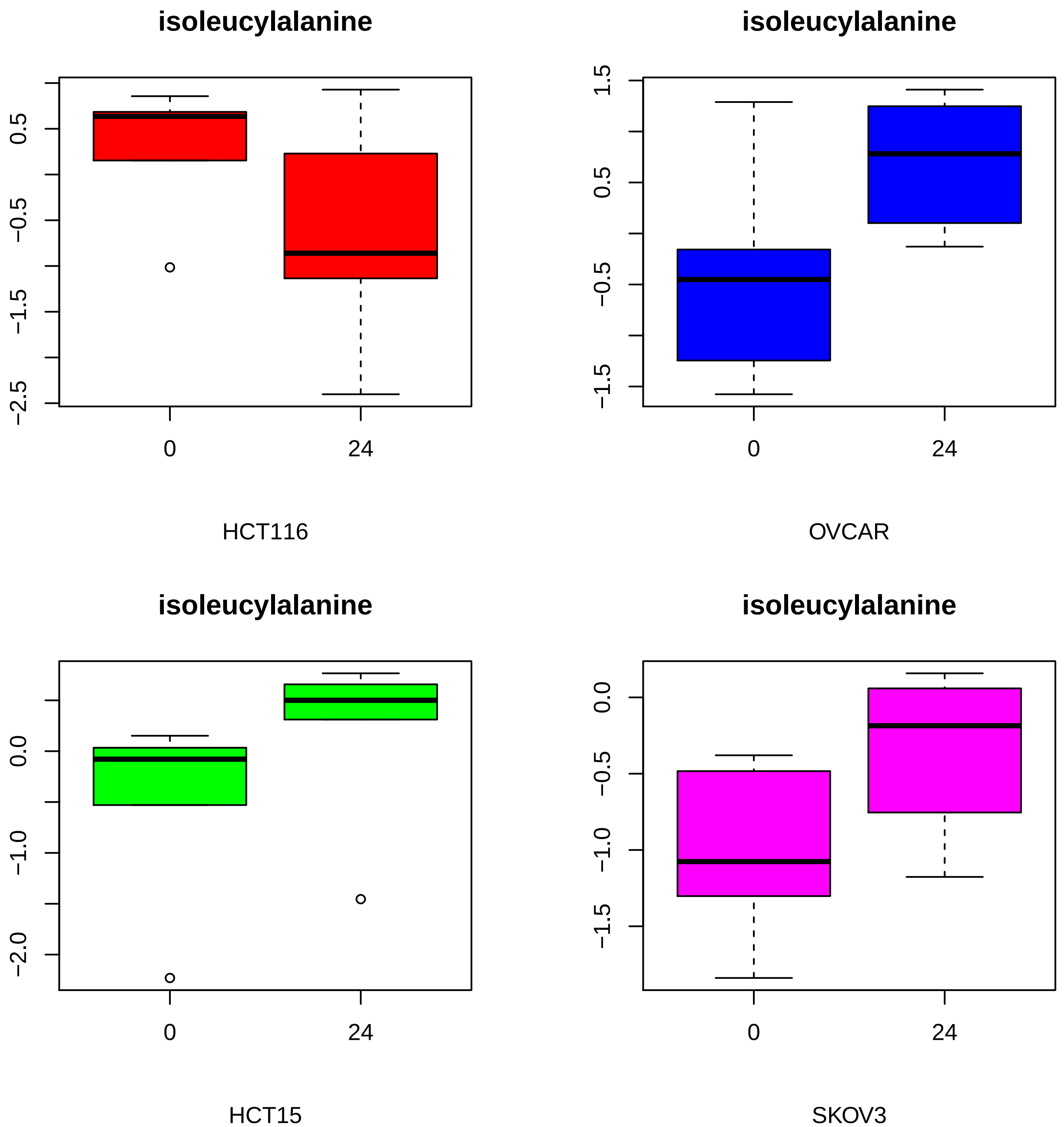
isoleucylalanine
0.5
−0.5
−1.5
−2.5
0
24
HCT116
isoleucylalanine
1.5
0.5
−0.5
−1.5
0
24
OVCAR
isoleucylalanine
0.0
−1.0
−2.0
0
24
HCT15
isoleucylalanine
0.0
−0.5
−1.0
−1.5
0
24
SKOV3

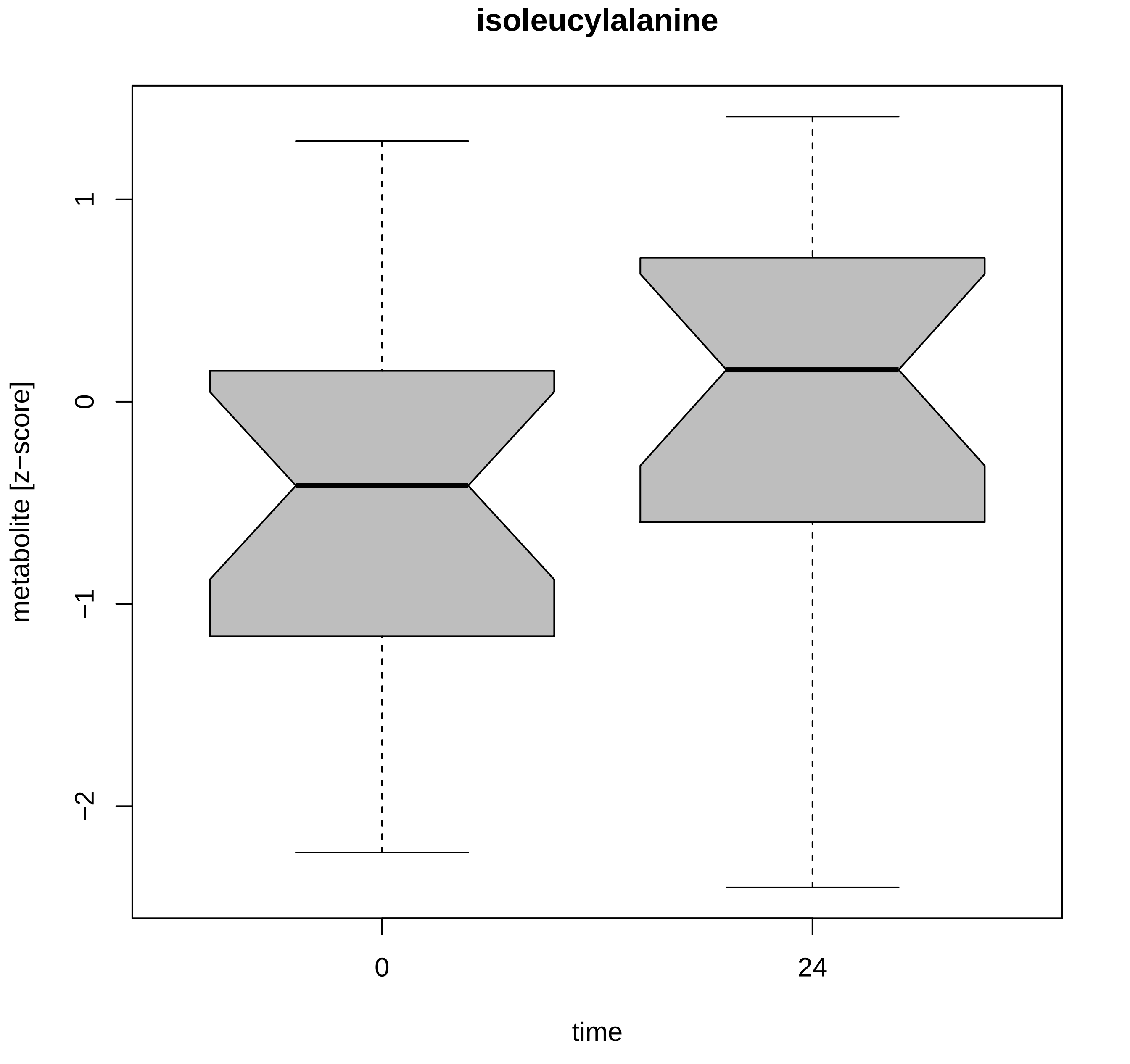

isoleucylalanine
metabolite [z−score]
1
0
−1
−2
0
24
time

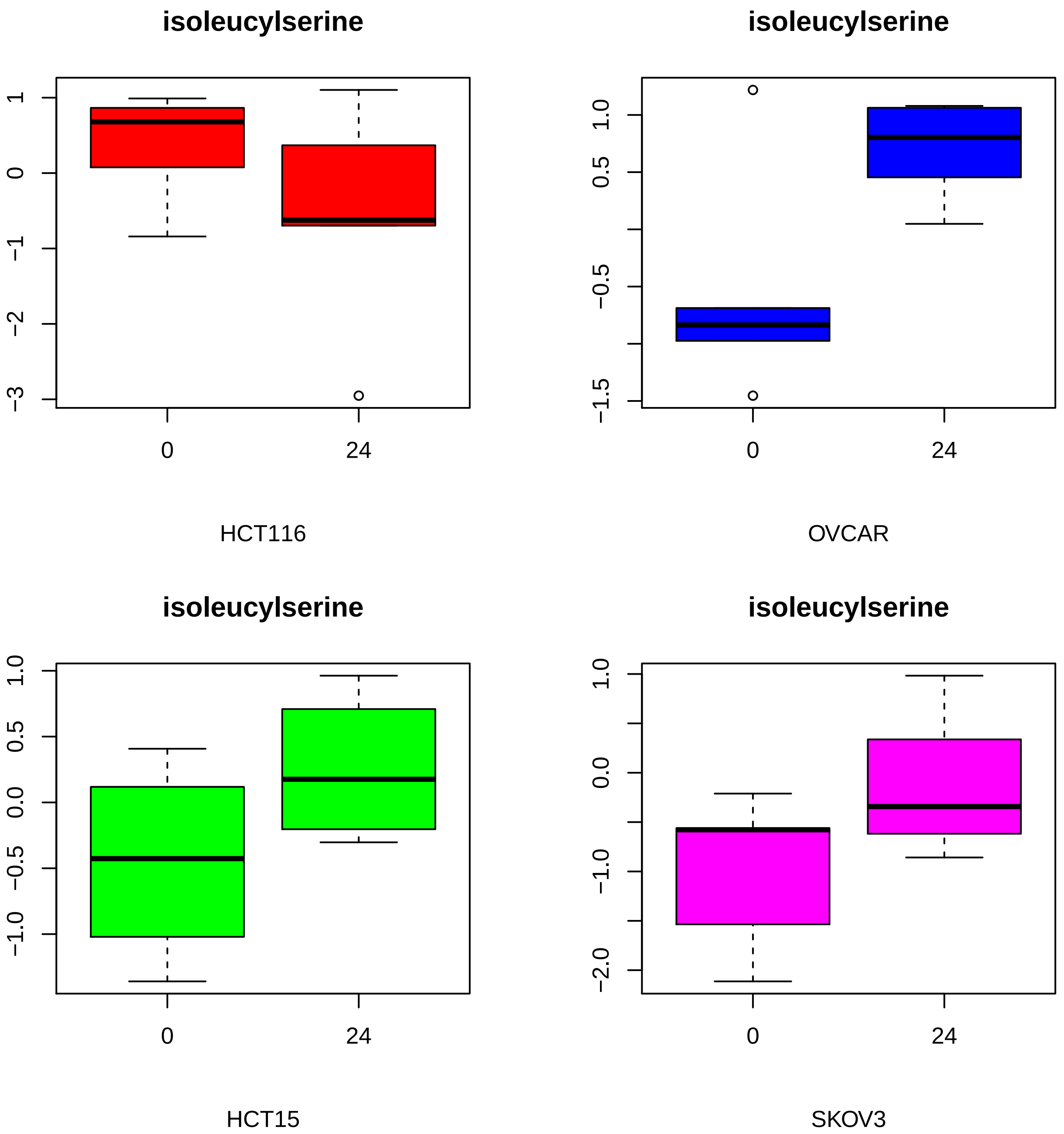
isoleucylserine
1
0
−1
−2
−3
0
24
HCT116
isoleucylserine
1.0
0.5
−0.5
−1.5
0
24
OVCAR
isoleucylserine
1.0
0.5
0.0
−0.5
−1.0
0
24
HCT15
isoleucylserine
1.0
0.0
−1.0
−2.0
0
24
SKOV3

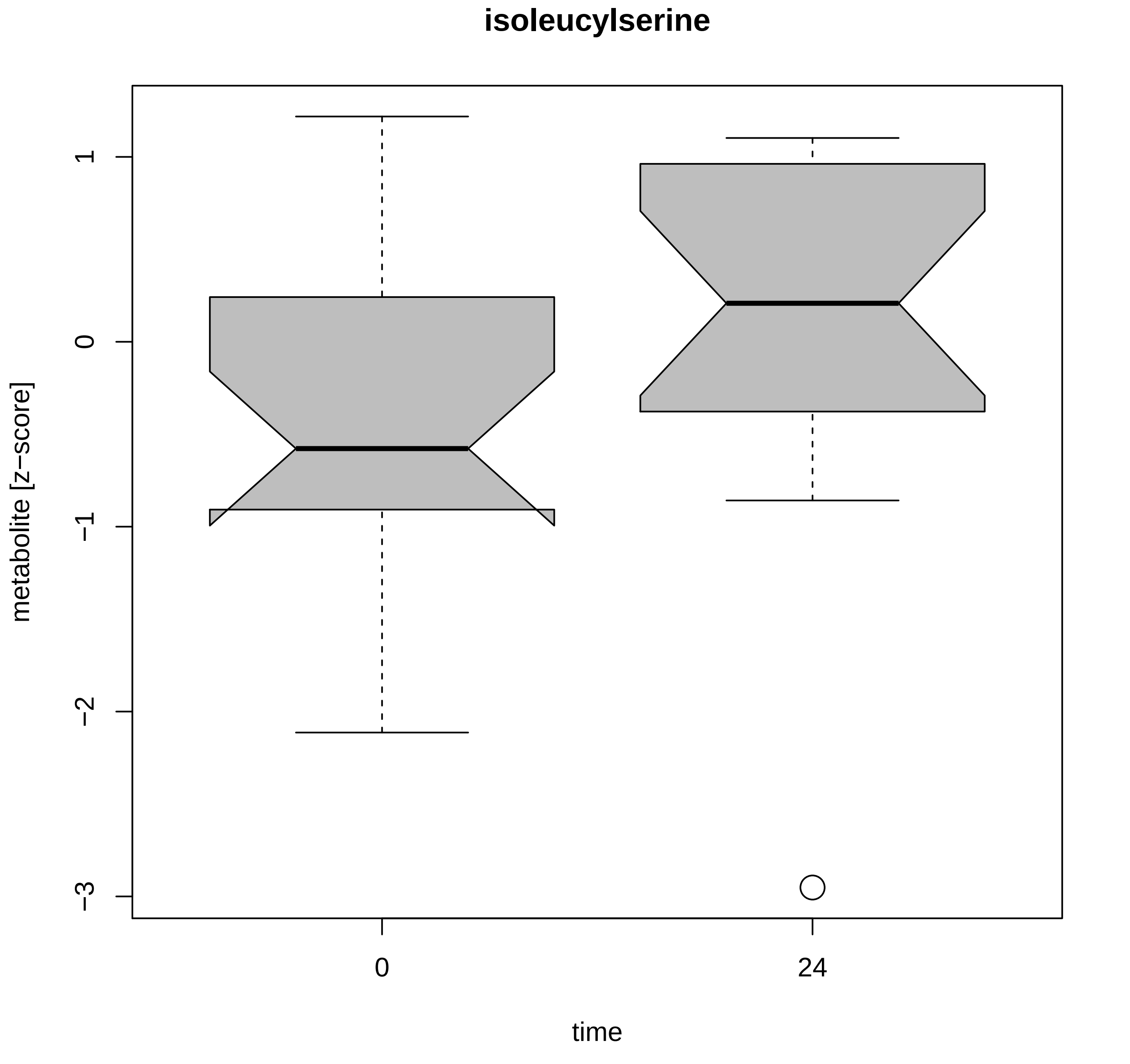
isoleucylserine
metabolite [z−score]
1
0
−1
−2
−3
0
24
time

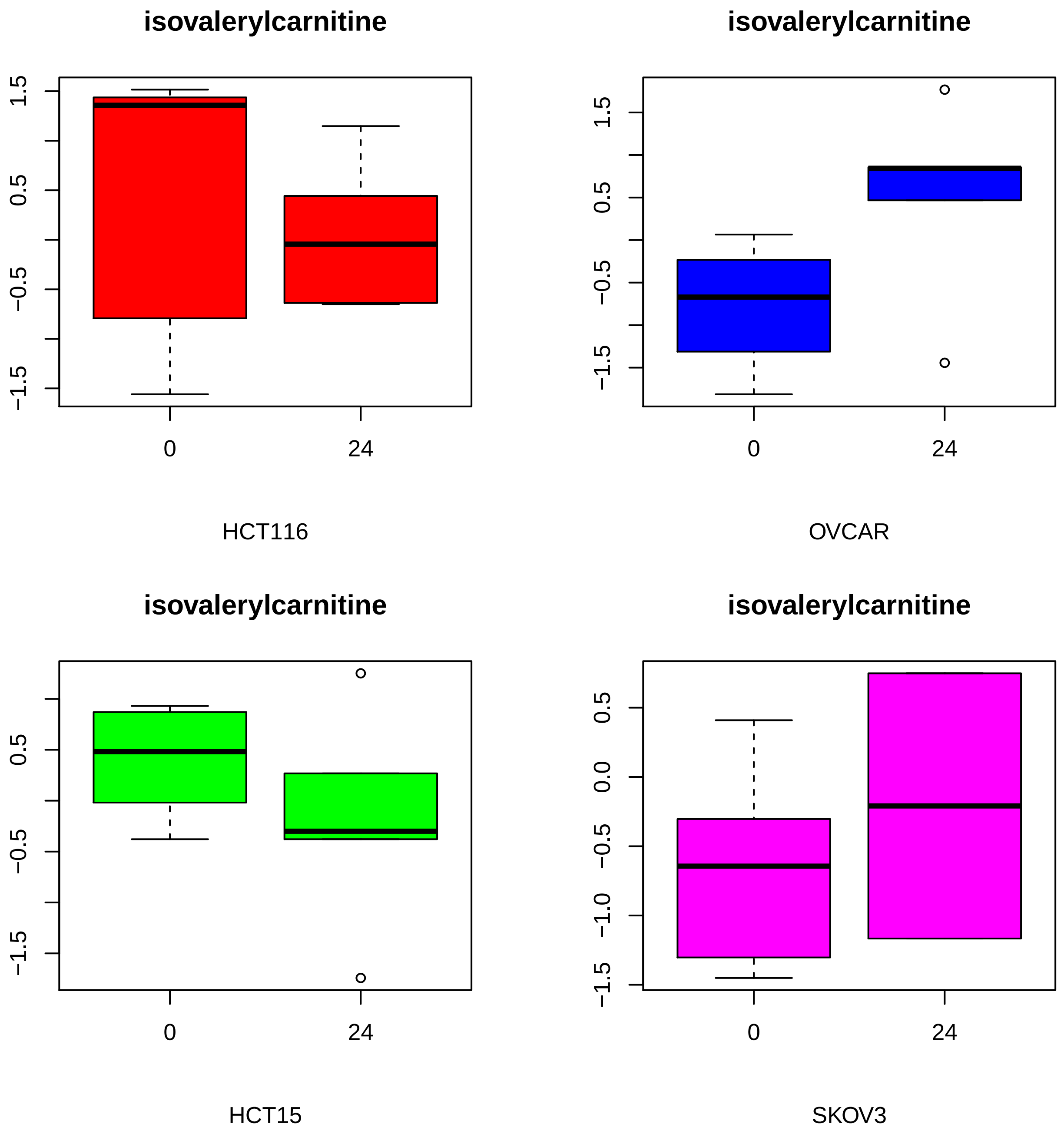
isovalerylcarnitine
1.5
0.5
−0.5
−1.5
0
24
HCT116
isovalerylcarnitine
1.5
0.5
−0.5
−1.5
0
24
OVCAR
isovalerylcarnitine
0.5
−0.5
−1.5
0
24
HCT15
isovalerylcarnitine
0.5
0.0
−0.5
−1.0
−1.5
0
24
SKOV3

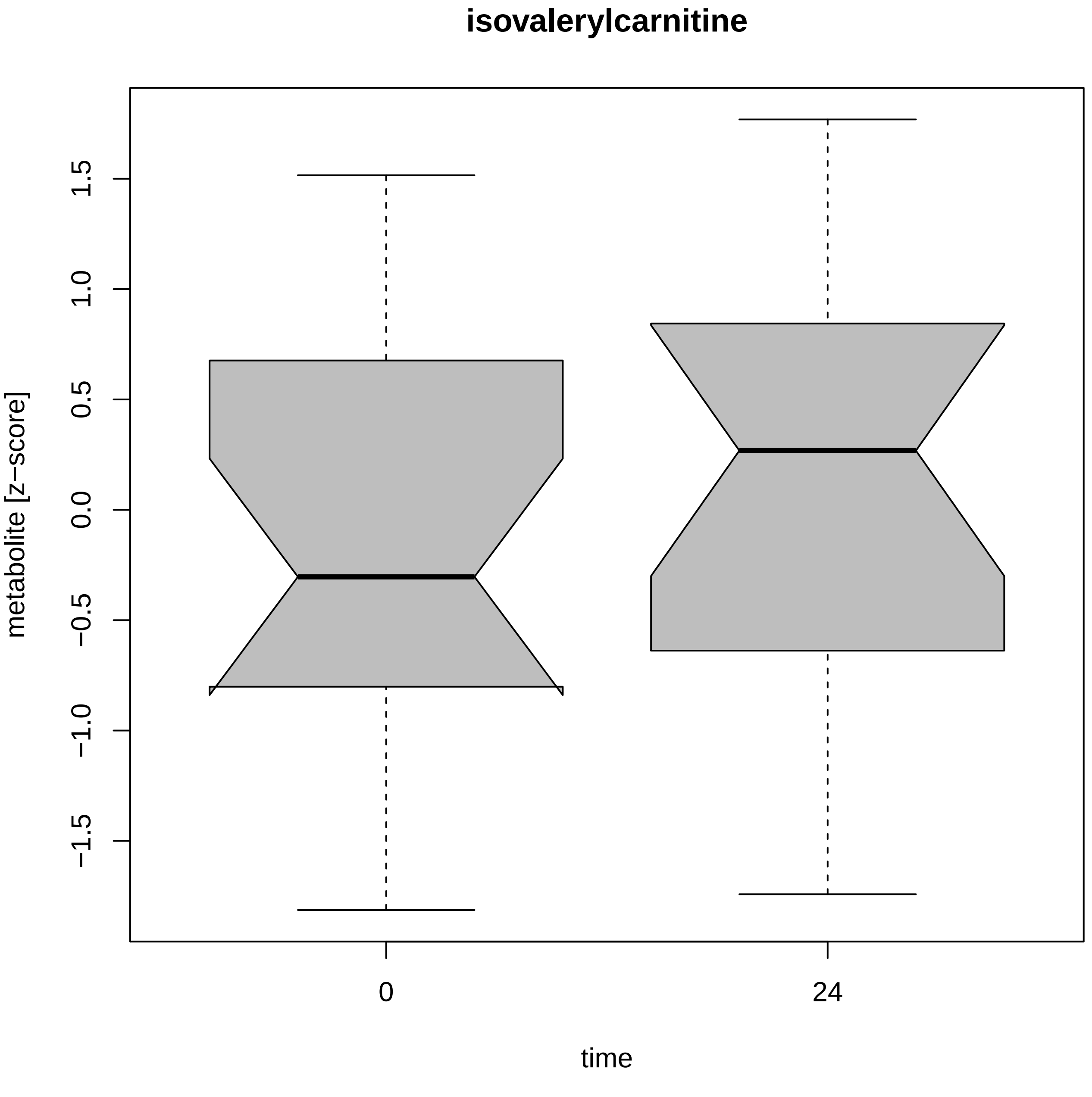
isovalerylcarnitine
metabolite [z−score]
1.5
1.0
0.5
0.0
−0.5
−1.0
−1.5
0
24
time

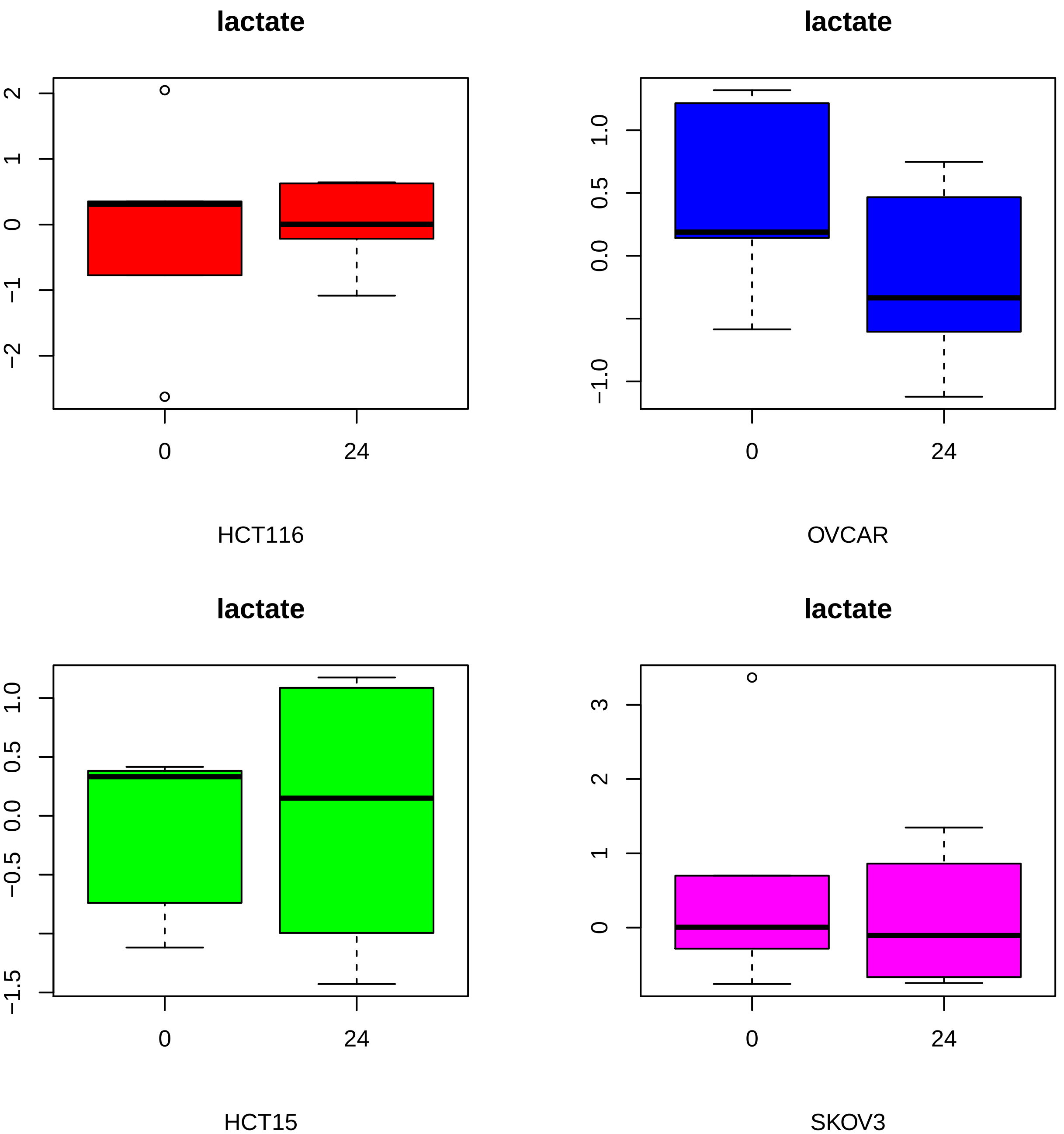
lactate
2
1
0
−1
−2
0
24
HCT116
lactate
1.0
0.5
0.0
−1.0
0
24
OVCAR
lactate
1.0
0.5
0.0
−0.5
−1.5
0
24
HCT15
lactate
3
2
1
0
0
24
SKOV3

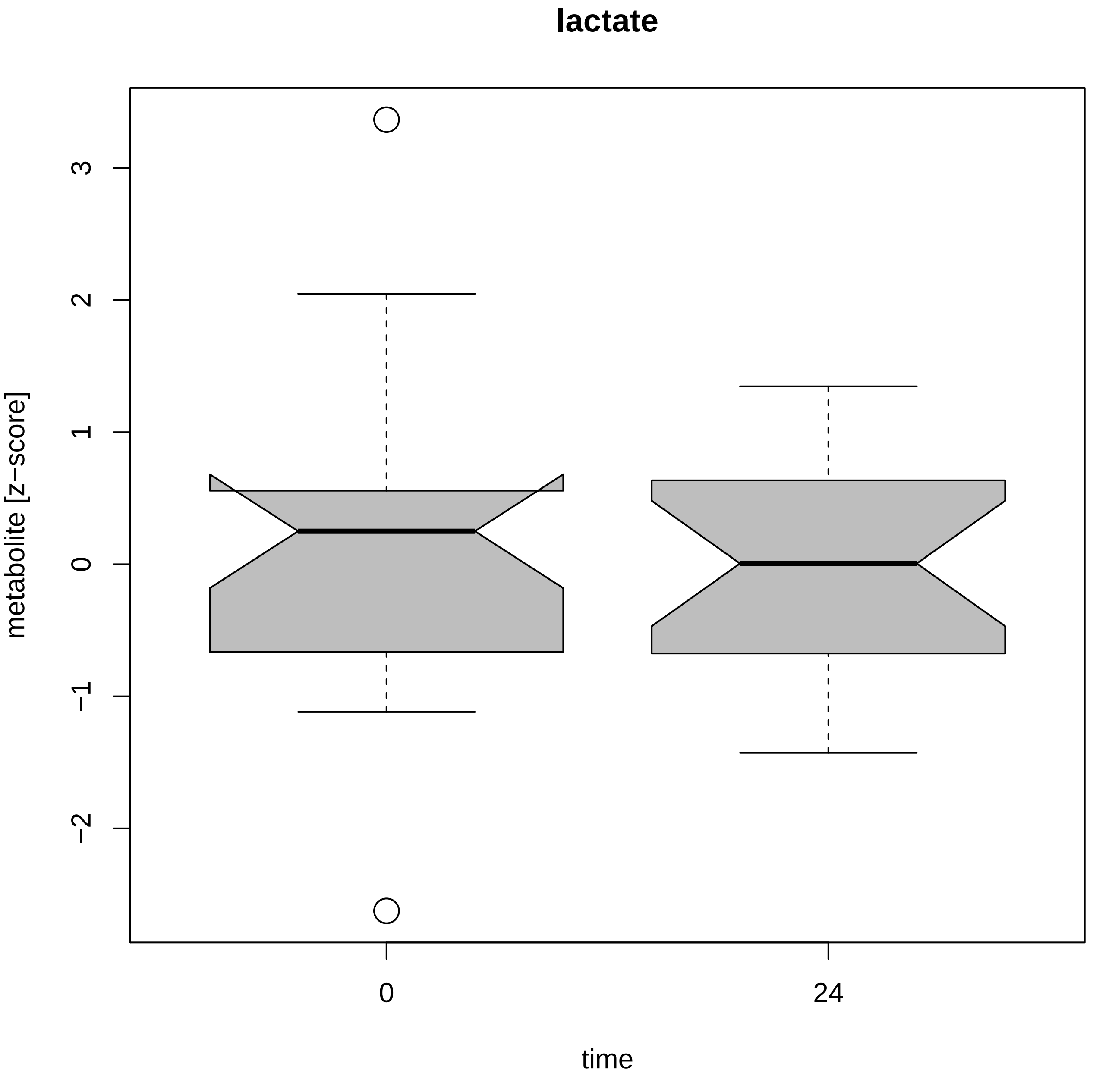
lactate
metabolite [z−score]
3
2
1
0
−1
−2
0
24
time

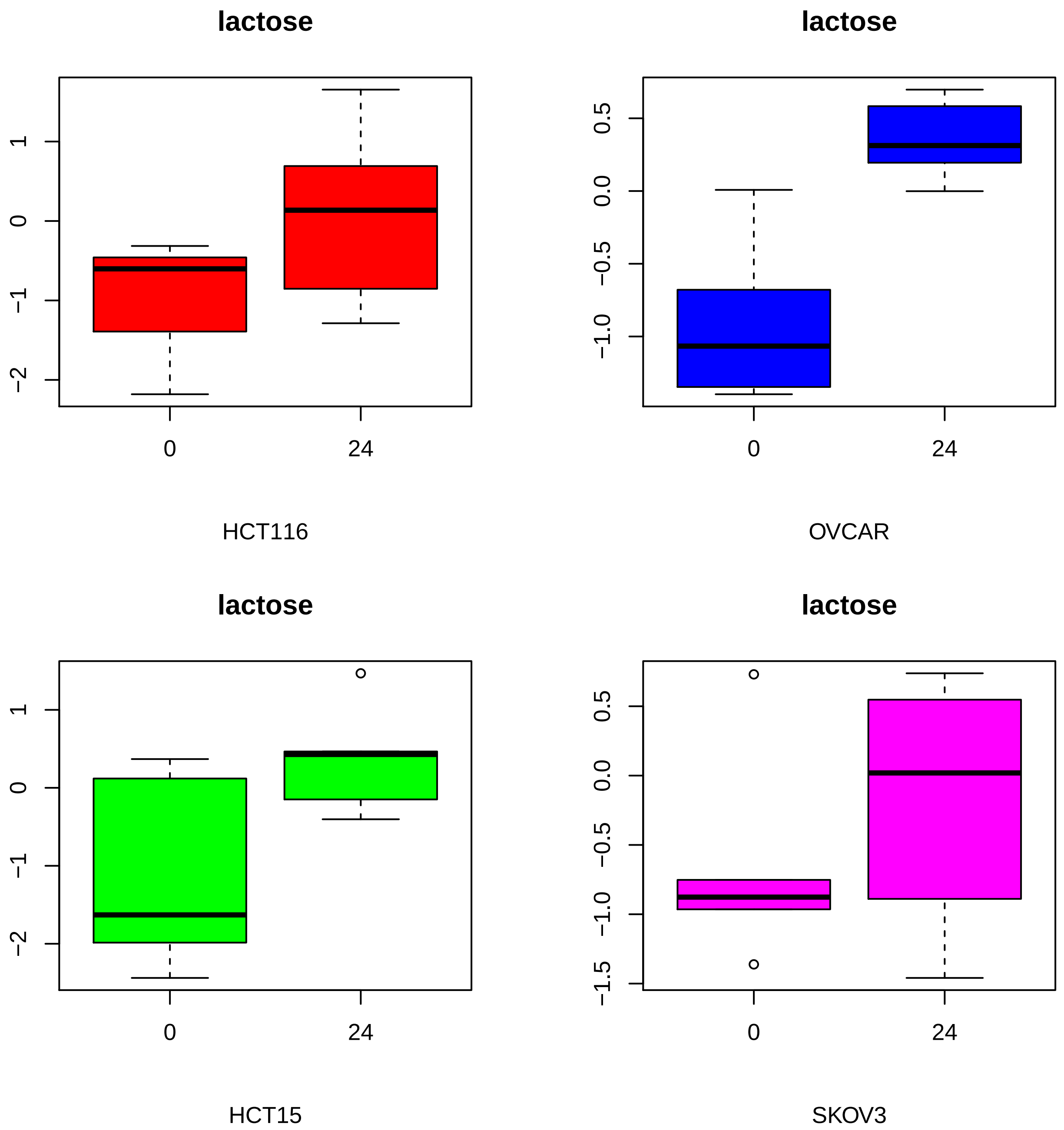
lactose
0
24
HCT116
lactose
0
24
OVCAR
lactose
0
24
HCT15
lactose
0
24
SKOV3

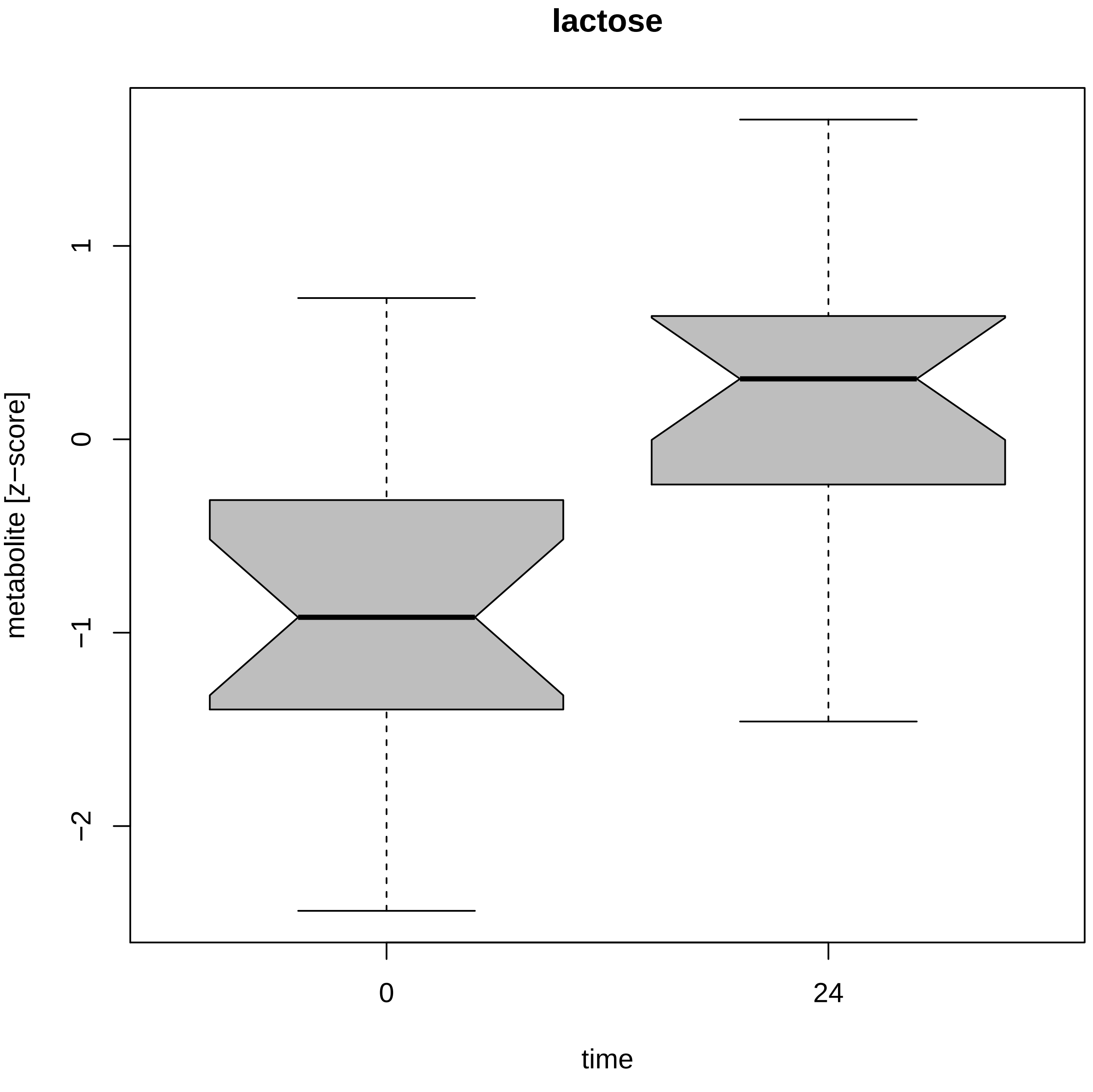
lactose
metabolite [z−score]
1
0
−1
−2
0
24
time

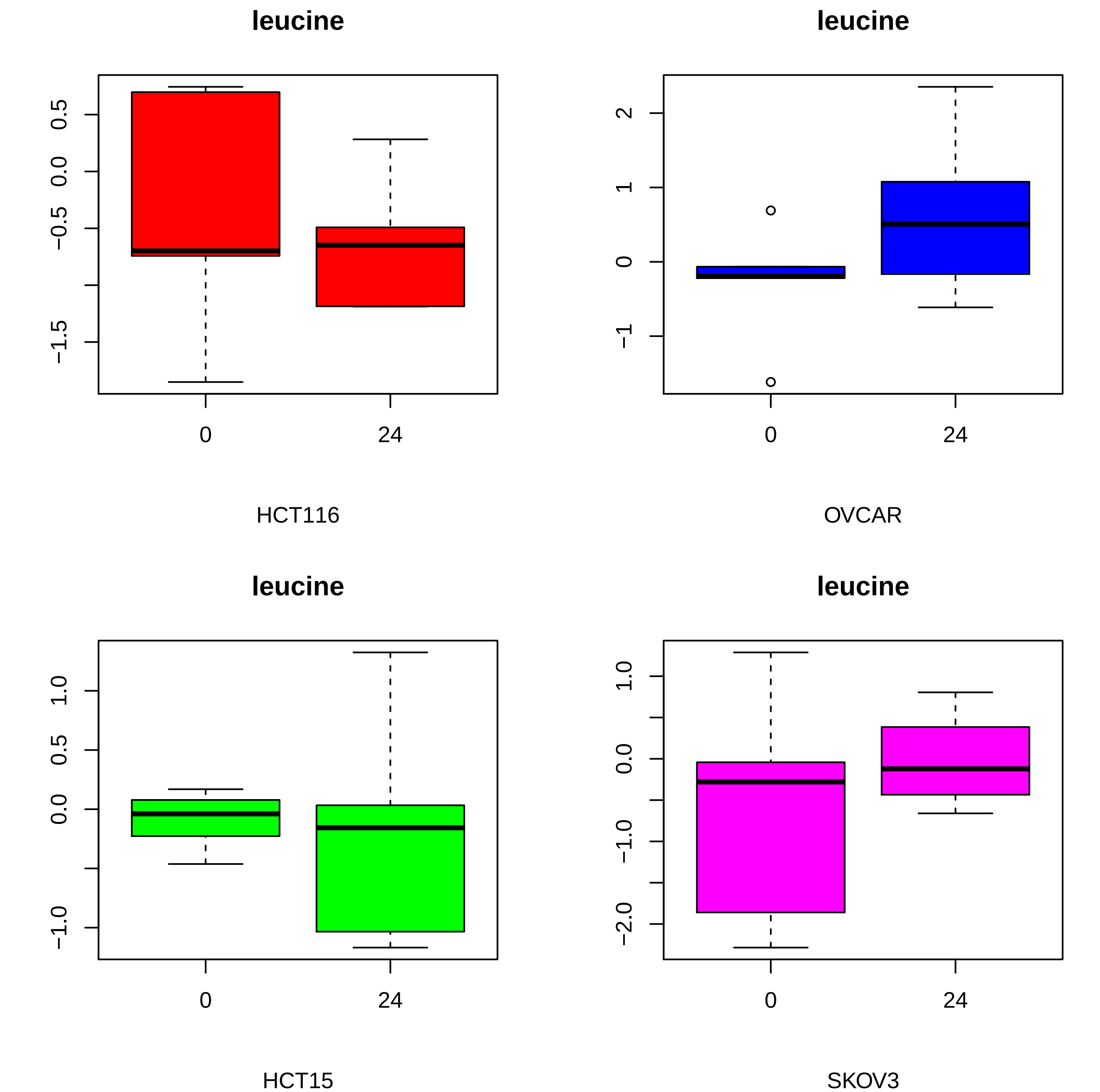
leucine
0.5
0.0
−0.5
−1.5
0
24
HCT116
leucine
2
1
0
−1
0
24
OVCAR
leucine
1.0
0.5
0.0
−1.0
0
24
HCT15
leucine
1.0
0.0
−1.0
−2.0
0
24
SKOV3

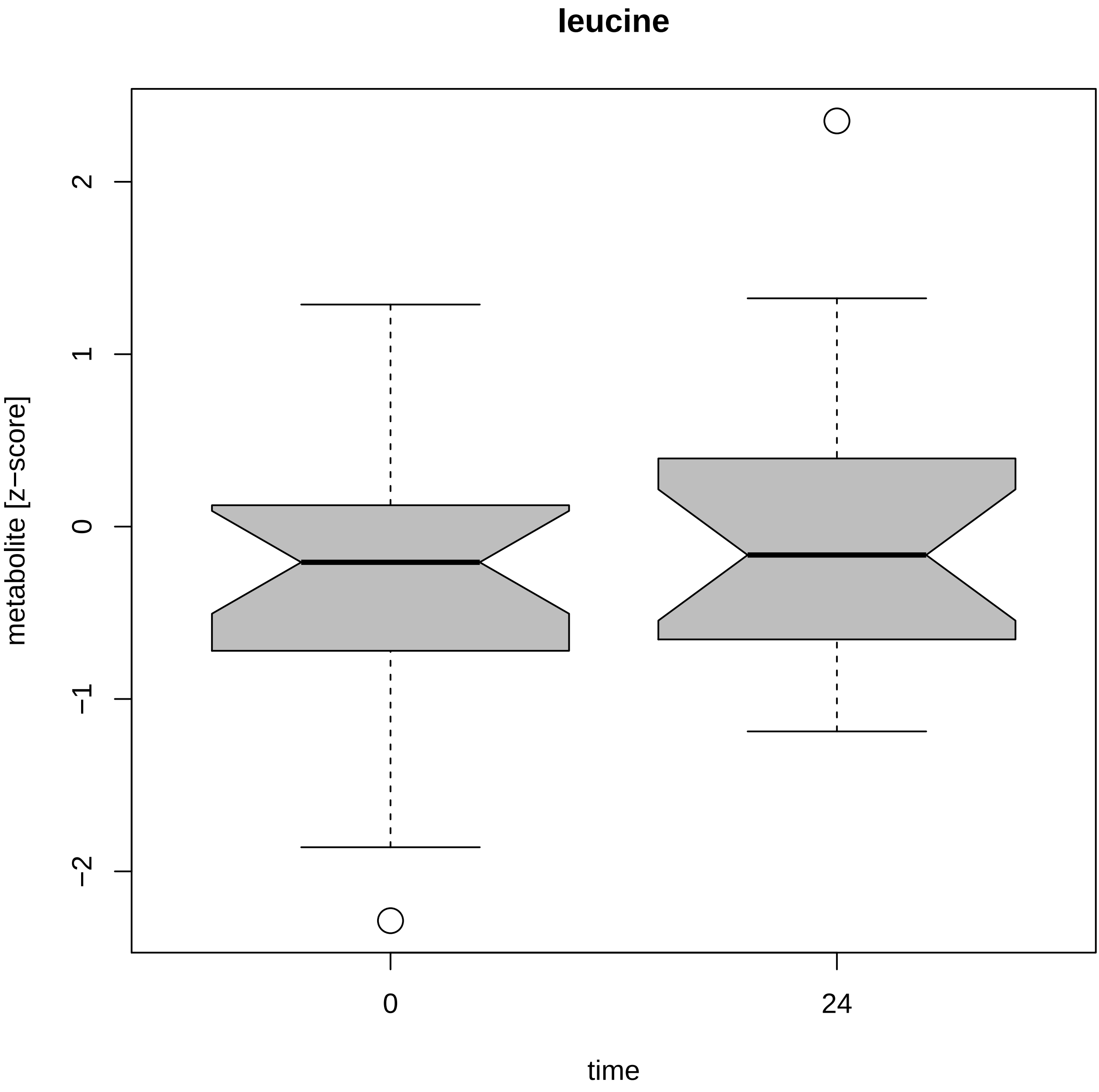
leucine
metabolite [z−score]
2
1
0
−1
−2
0
24
time

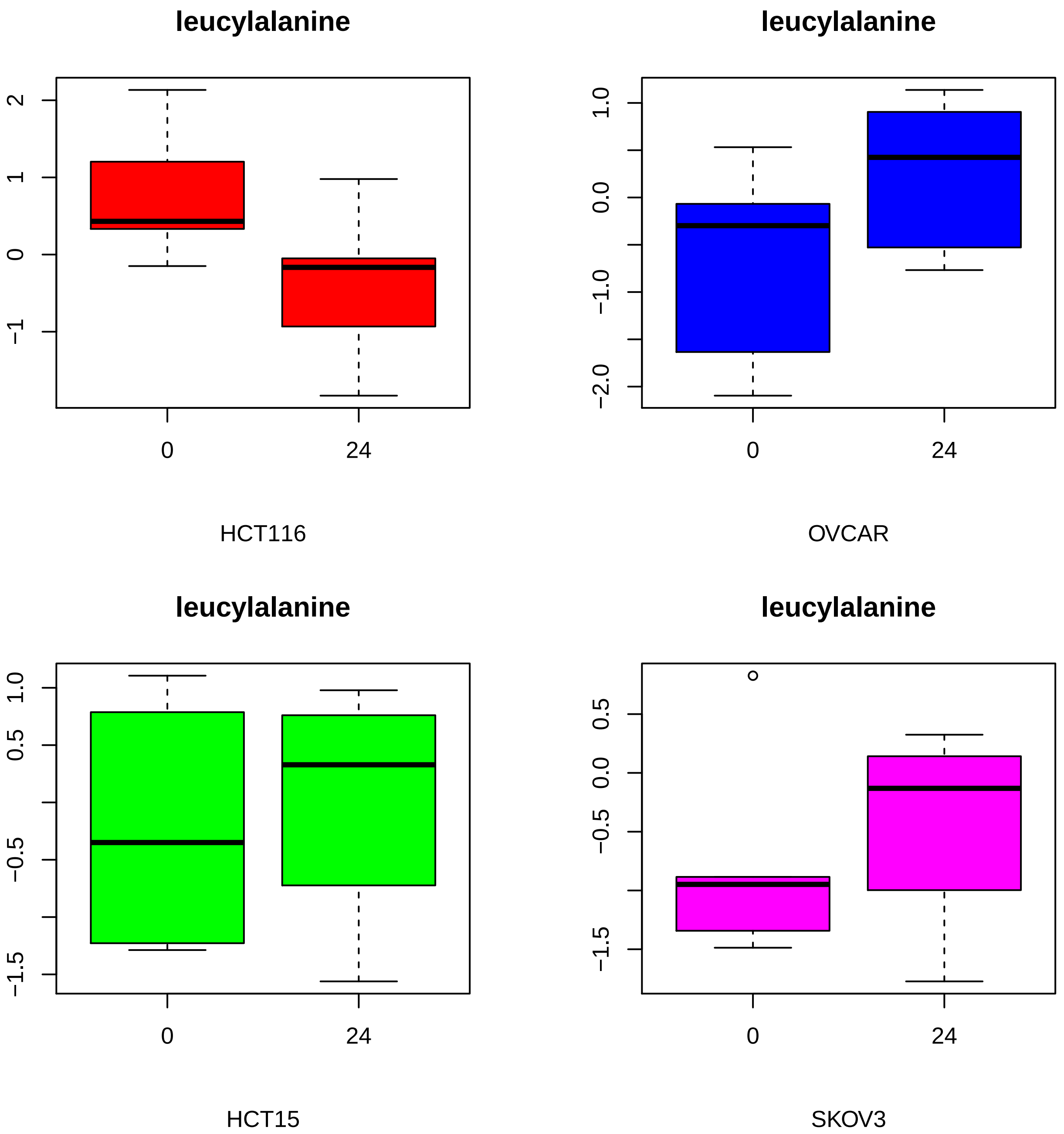
leucylalanine
2
1
0
−1
0
24
HCT116
leucylalanine
1.0
0.0
−1.0
−2.0
0
24
OVCAR
leucylalanine
1.0
0.5
−0.5
−1.5
0
24
HCT15
leucylalanine
0.5
0.0
−0.5
−1.5
0
24
SKOV3

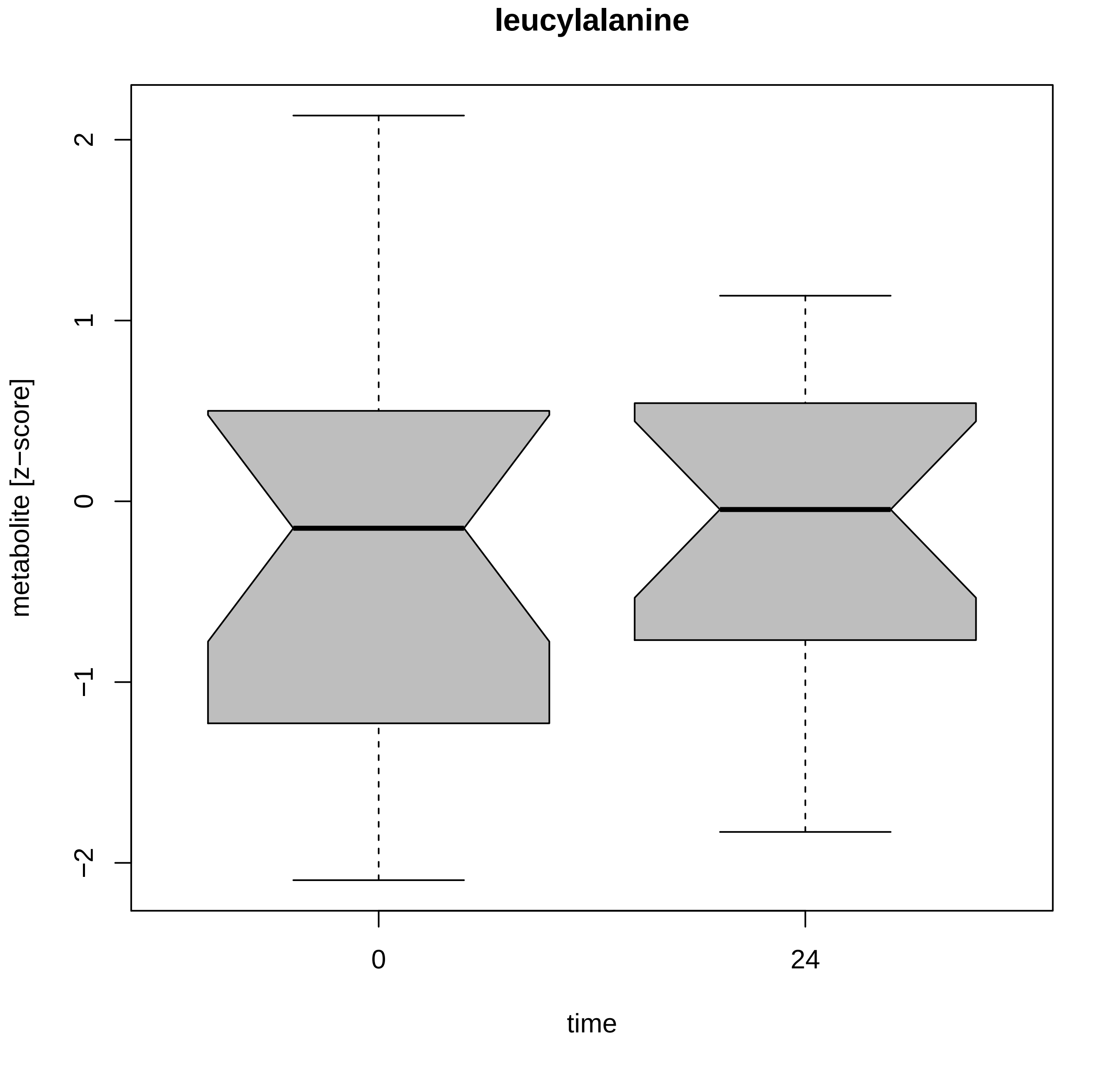
leucylalanine
metabolite [z−score]
2
1
0
−1
−2
0
24
time

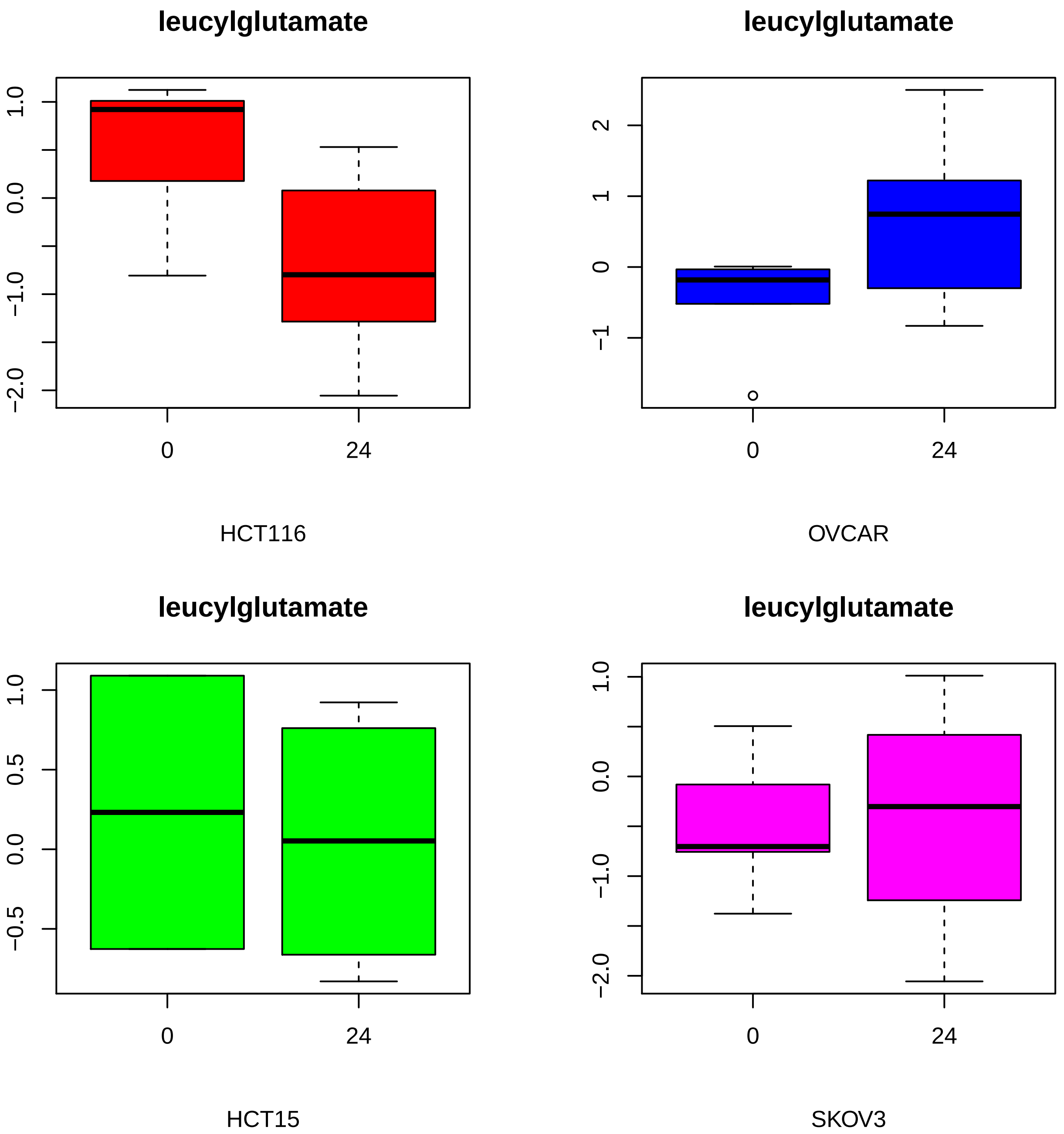
leucylglutamate
1.0
0.0
−1.0
−2.0
0
24
HCT116
leucylglutamate
2
1
0
−1
0
24
OVCAR
leucylglutamate
1.0
0.5
0.0
−0.5
0
24
HCT15
leucylglutamate
1.0
0.0
−1.0
−2.0
0
24
SKOV3

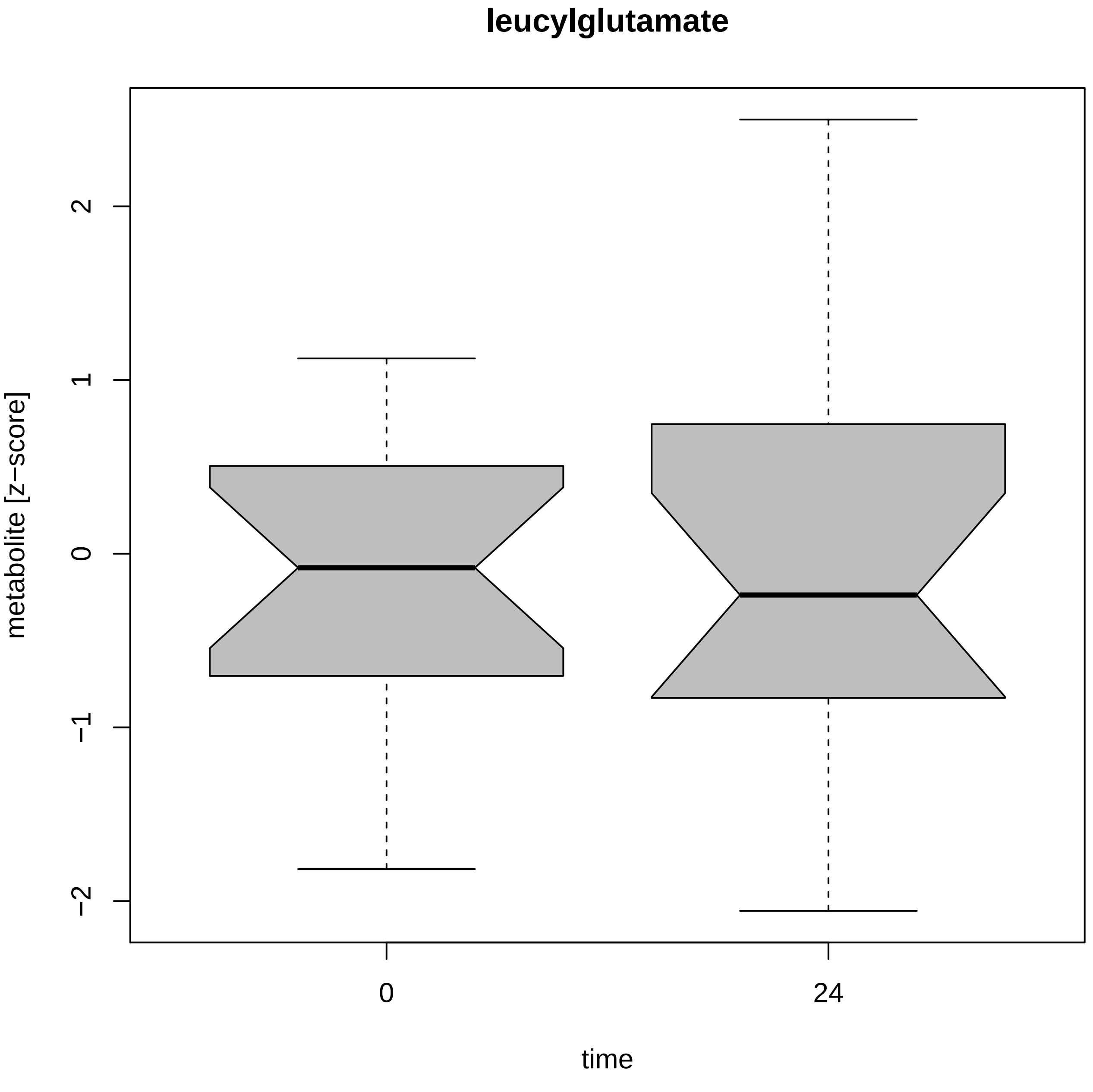
leucylglutamate
metabolite [z−score]
time
0
24
−2
−1
0
1
2

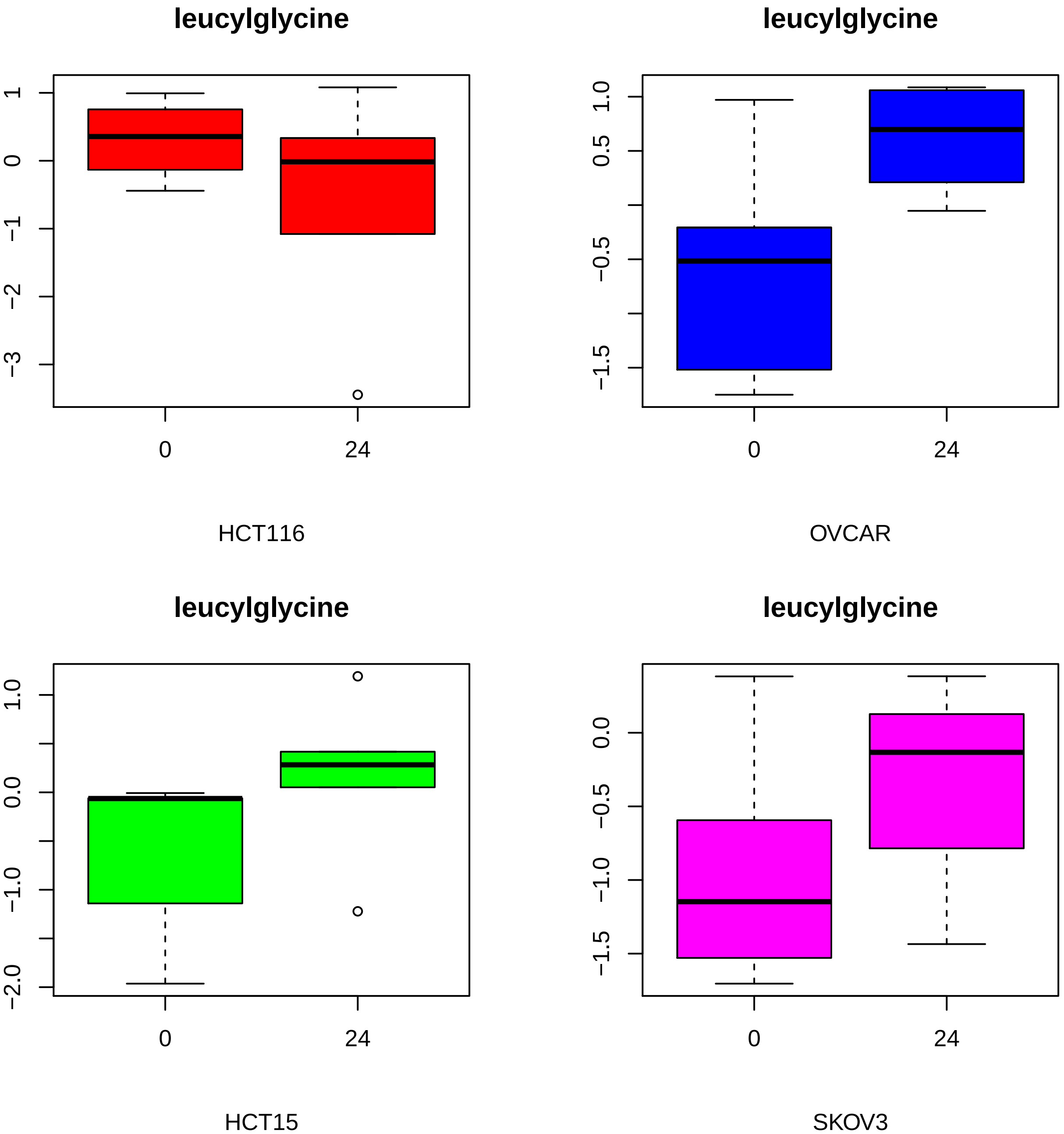
leucylglycine
1
0
−1
−2
−3
0
24
HCT116
leucylglycine
1.0
0.5
−0.5
−1.5
0
24
OVCAR
leucylglycine
1.0
0.0
−1.0
−2.0
0
24
HCT15
leucylglycine
0.0
−0.5
−1.0
−1.5
0
24
SKOV3

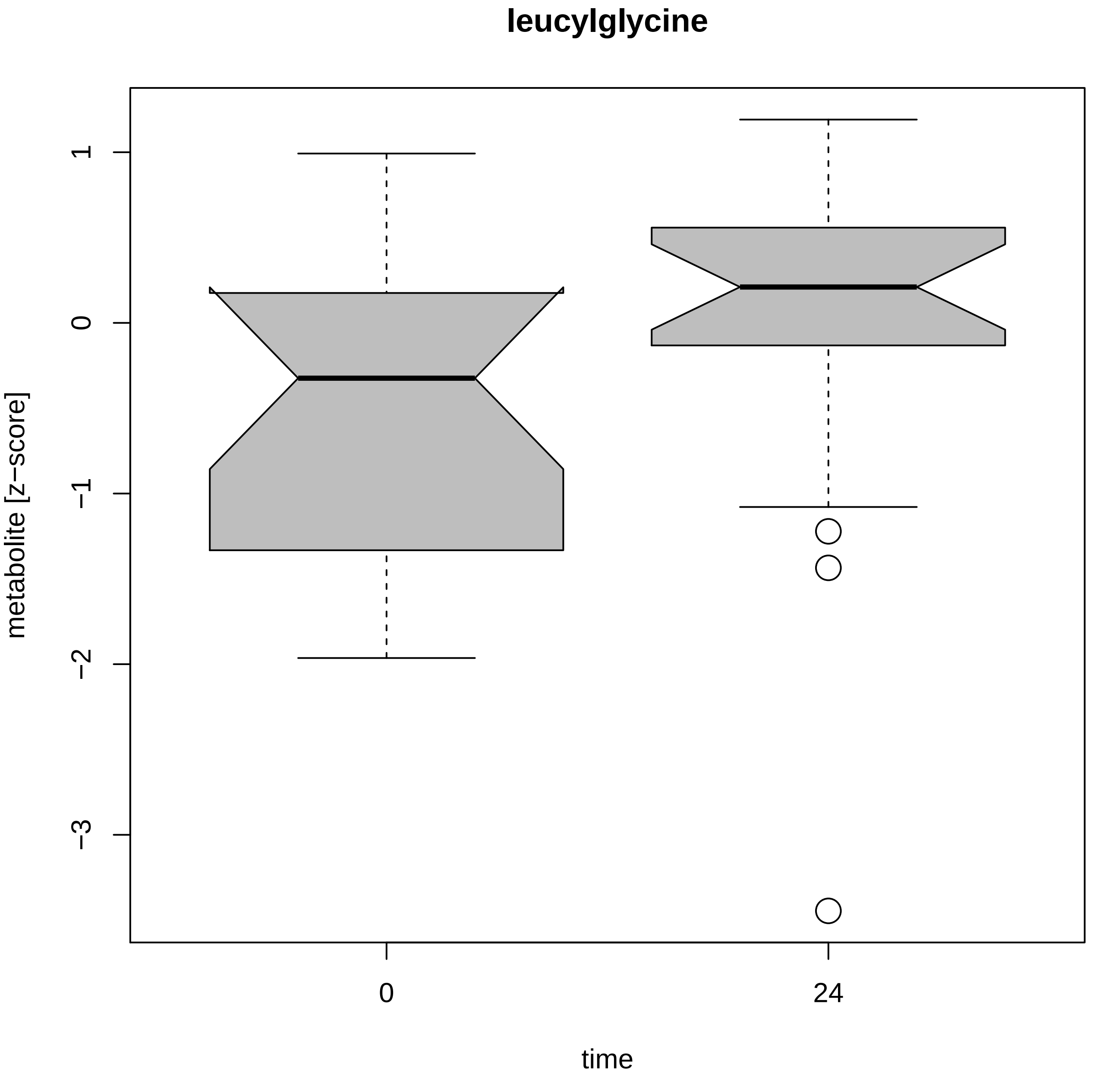
leucylglycine
metabolite [z−score]
1
0
−1
−2
−3
0
24
time

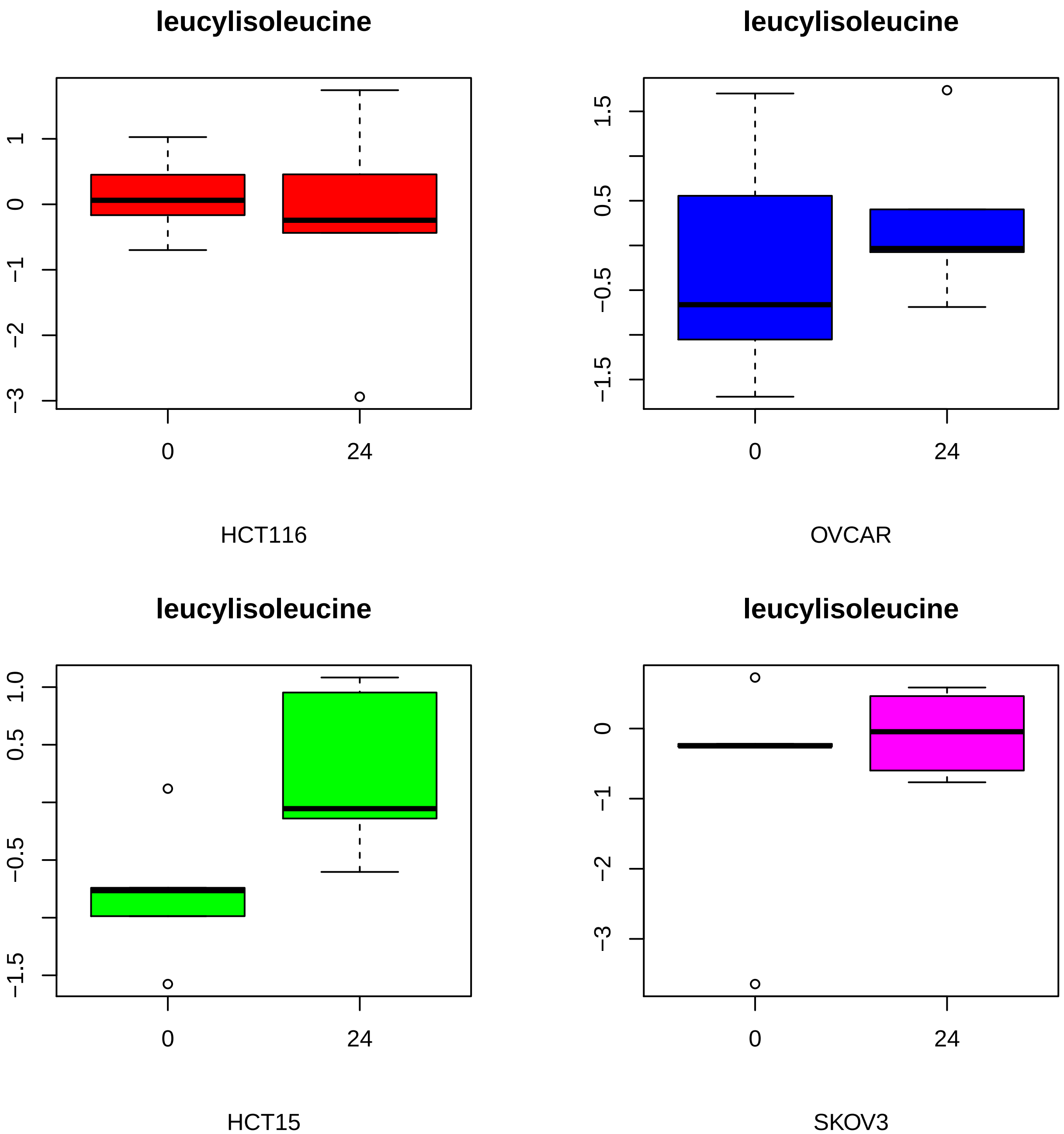
leucylisoleucine
1
0
−1
−2
−3
0
24
HCT116
leucylisoleucine
1.5
0.5
−0.5
−1.5
0
24
OVCAR
leucylisoleucine
1.0
0.5
−0.5
−1.5
0
24
HCT15
leucylisoleucine
0
−1
−2
−3
0
24
SKOV3

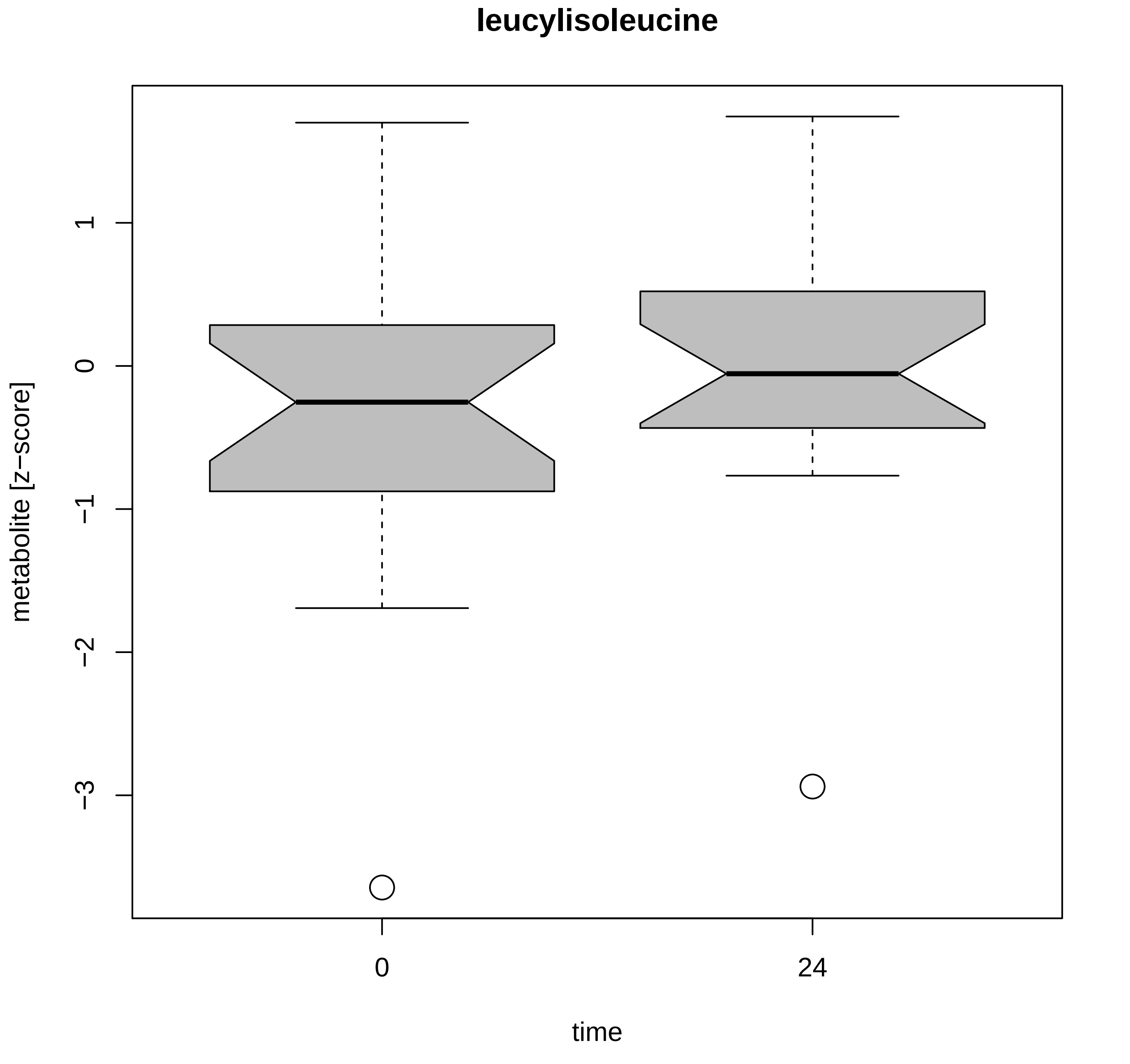
leucylisoleucine
metabolite [z−score]
1
0
−1
−2
−3
0
24
time

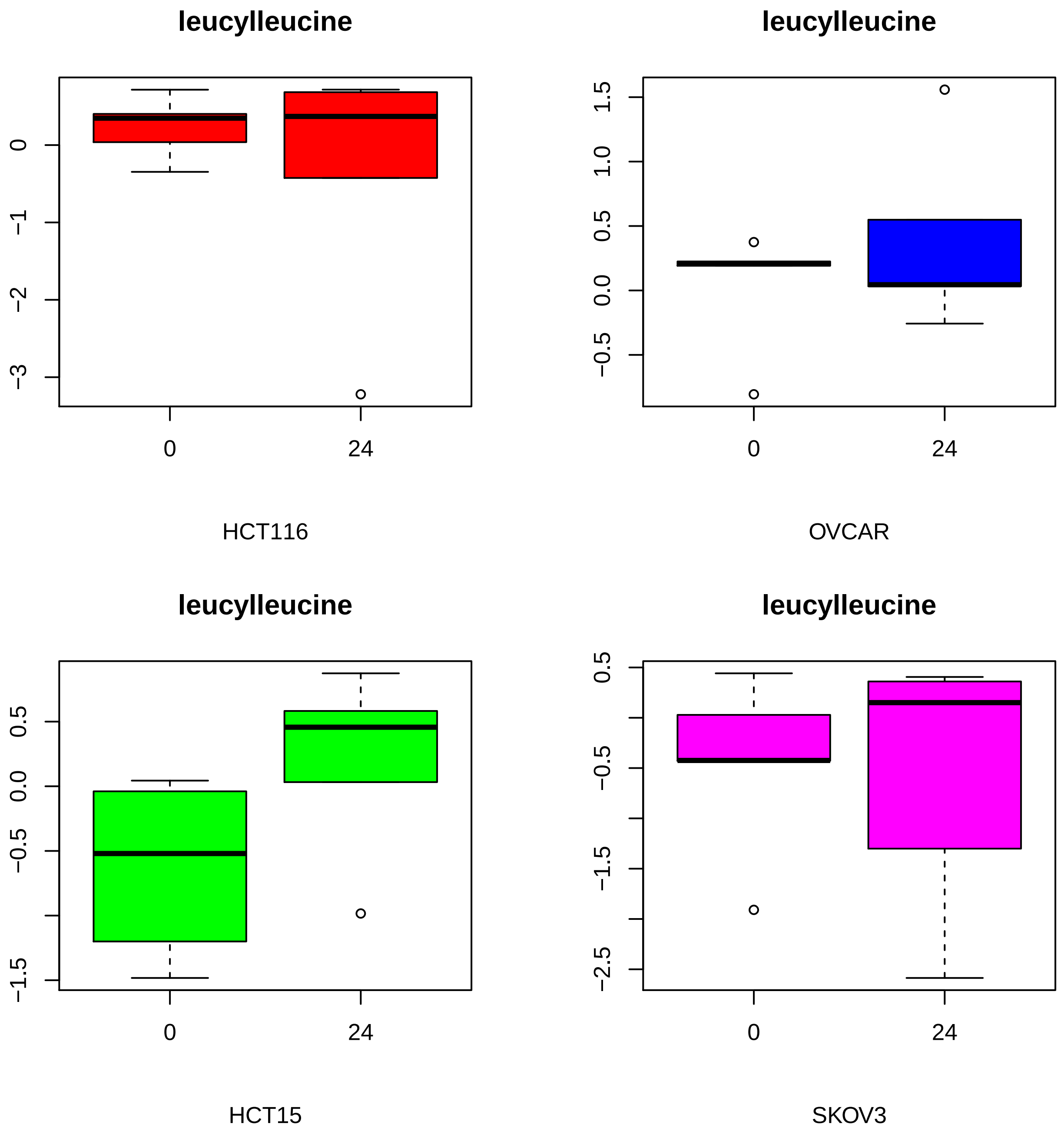
leucylleucine
0
−1
−2
−3
0
24
HCT116
leucylleucine
1.5
1.0
0.5
0.0
−0.5
0
24
OVCAR
leucylleucine
0.5
0.0
−0.5
−1.5
0
24
HCT15
leucylleucine
0.5
−0.5
−1.5
−2.5
0
24
SKOV3

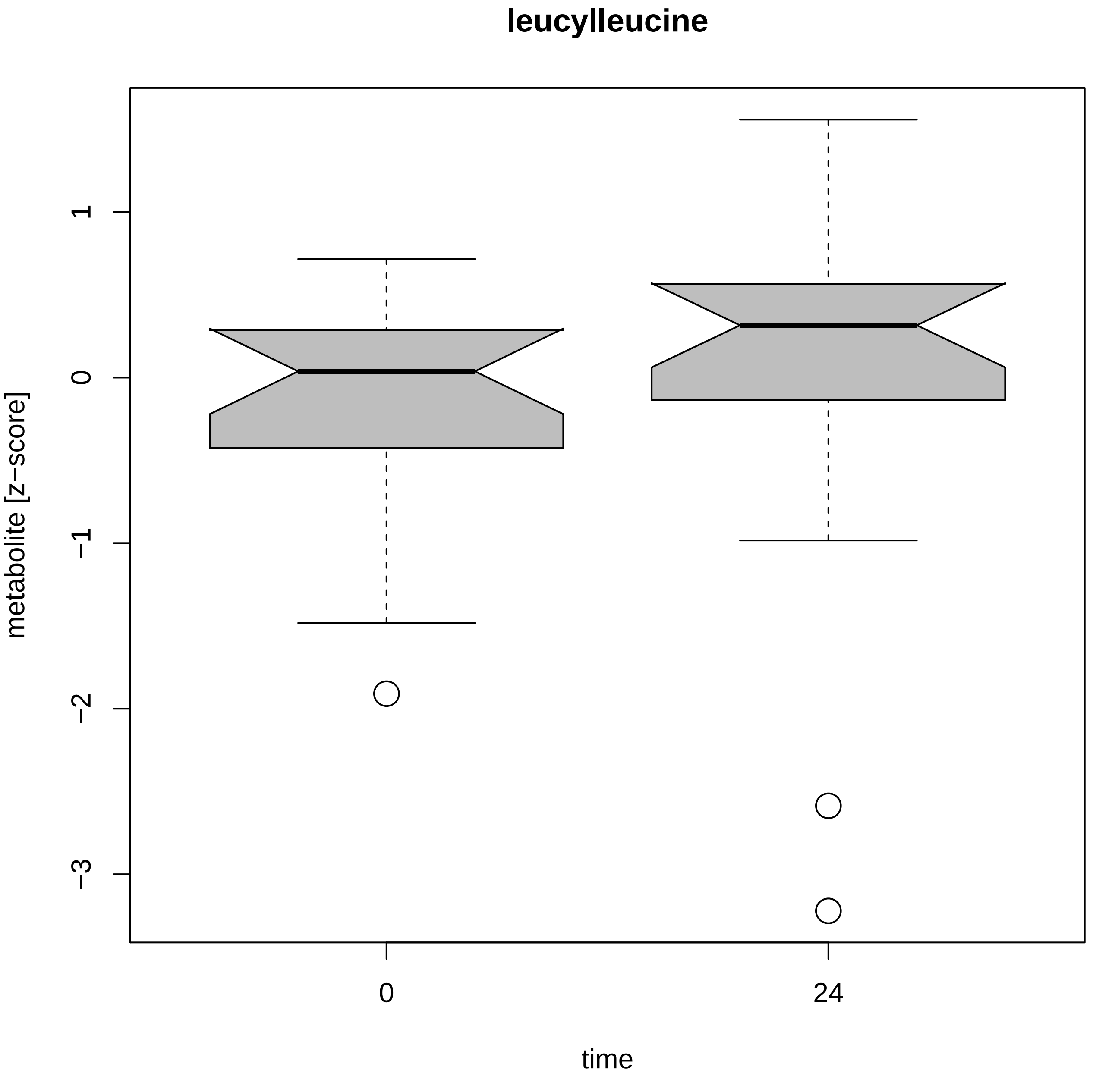

leucylleucine
metabolite [z−score]
1
0
−1
−2
−3
0
24
time

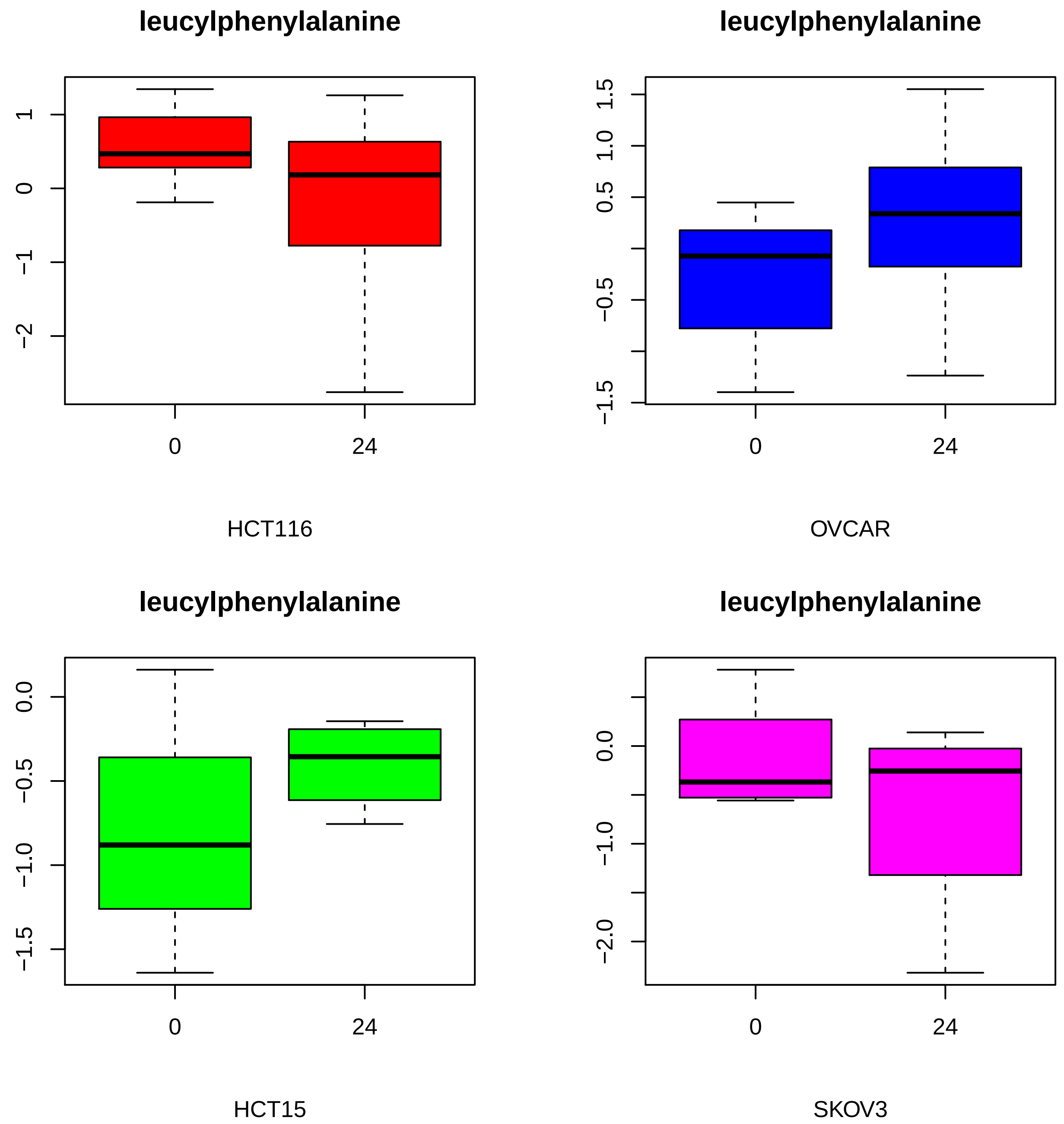
leucylphenylalanine
1
0
−1
−2
0
24
HCT116
leucylphenylalanine
1.5
1.0
0.5
−0.5
−1.5
0
24
OVCAR
leucylphenylalanine
0.0
−0.5
−1.0
−1.5
0
24
HCT15
leucylphenylalanine
0.0
−1.0
−2.0
0
24
SKOV3

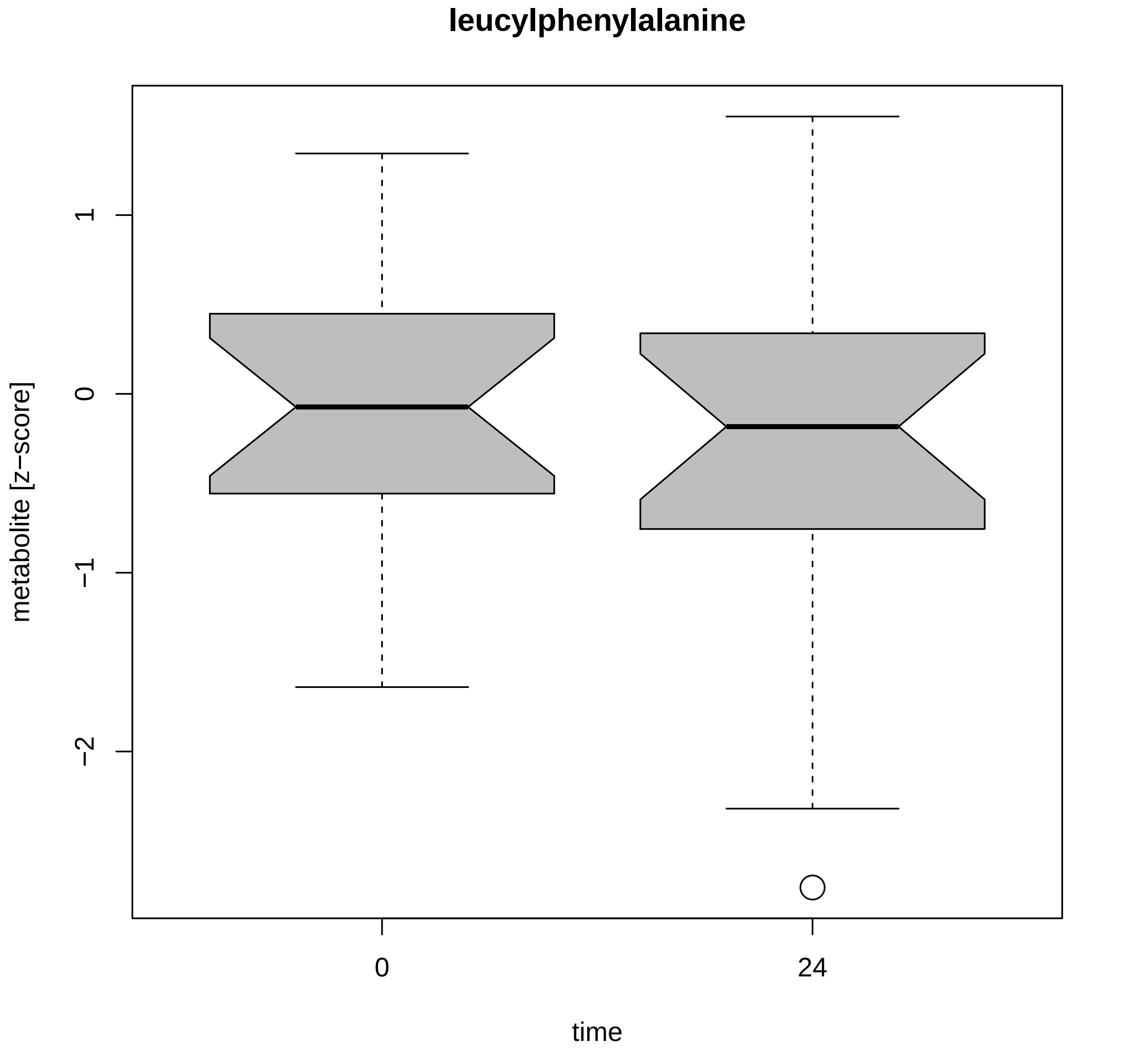
leucylphenylalanine
metabolite [z−score]
1
0
−1
−2
0
24
time

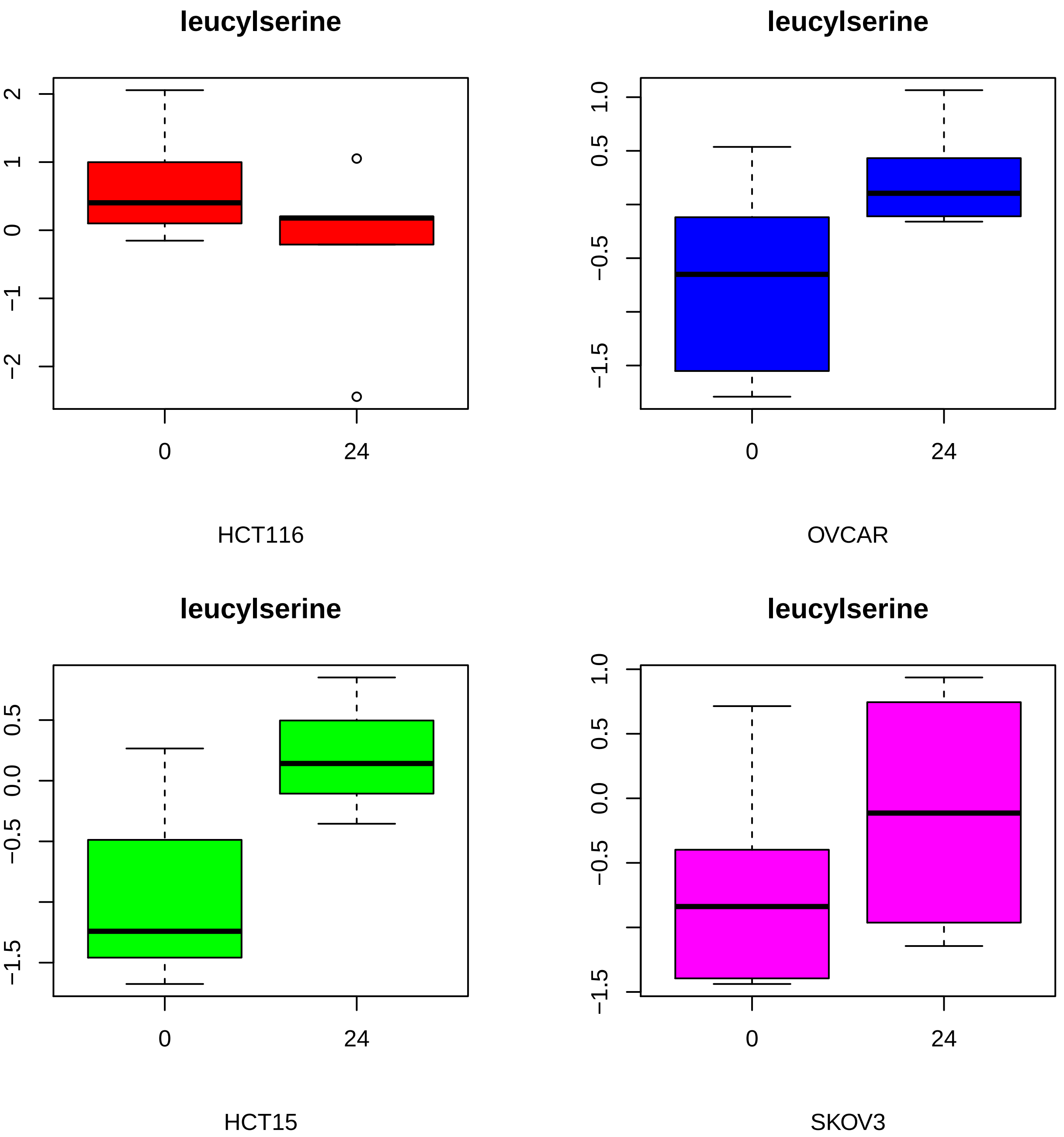
leucylserine
2
1
0
−1
−2
0
24
HCT116
leucylserine
1.0
0.5
−0.5
−1.5
0
24
OVCAR
leucylserine
0.5
0.0
−0.5
−1.5
0
24
HCT15
leucylserine
1.0
0.5
0.0
−0.5
−1.5
0
24
SKOV3

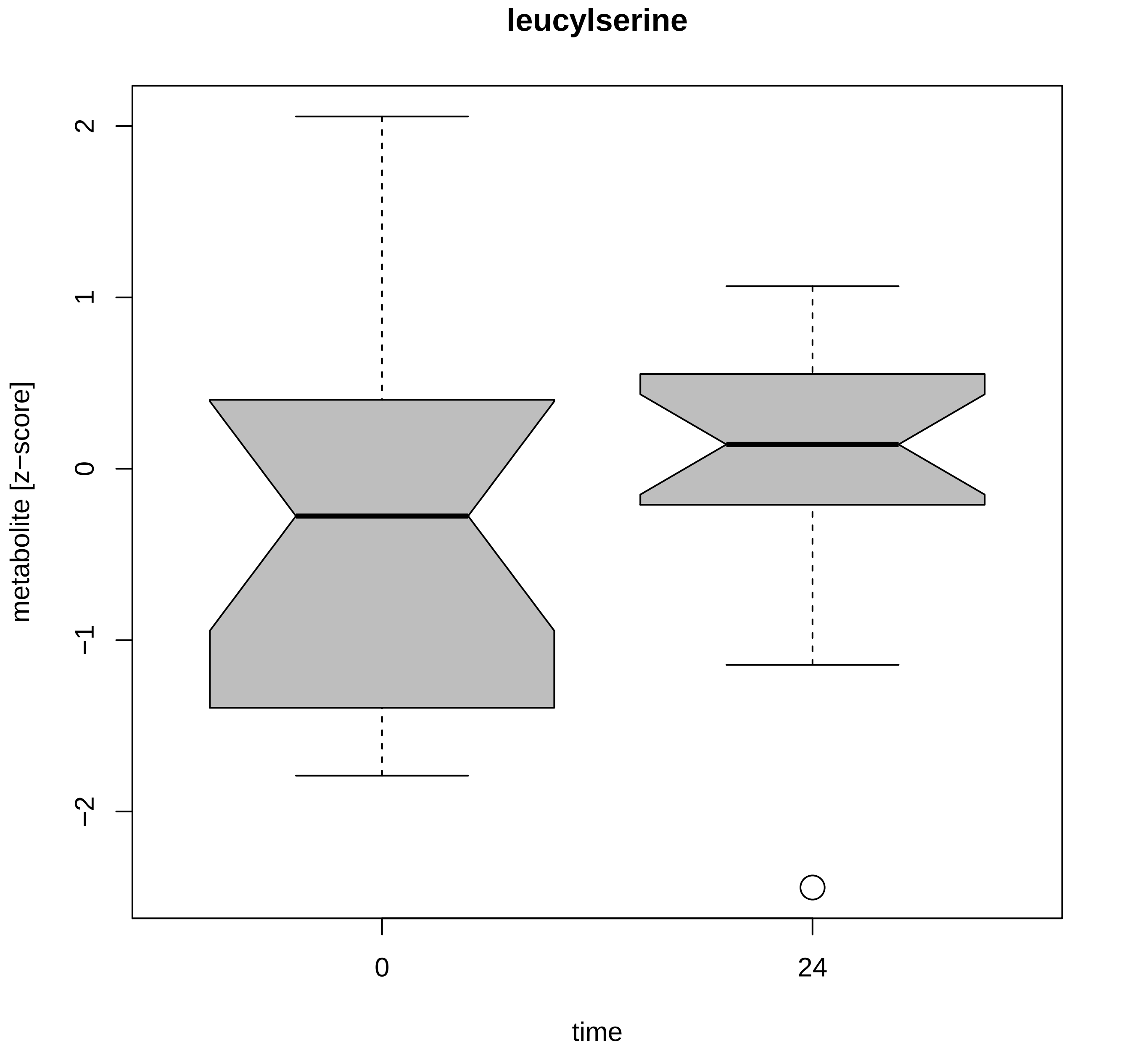

leucylserine
metabolite [z−score]
2
1
0
−1
−2
0
24
time

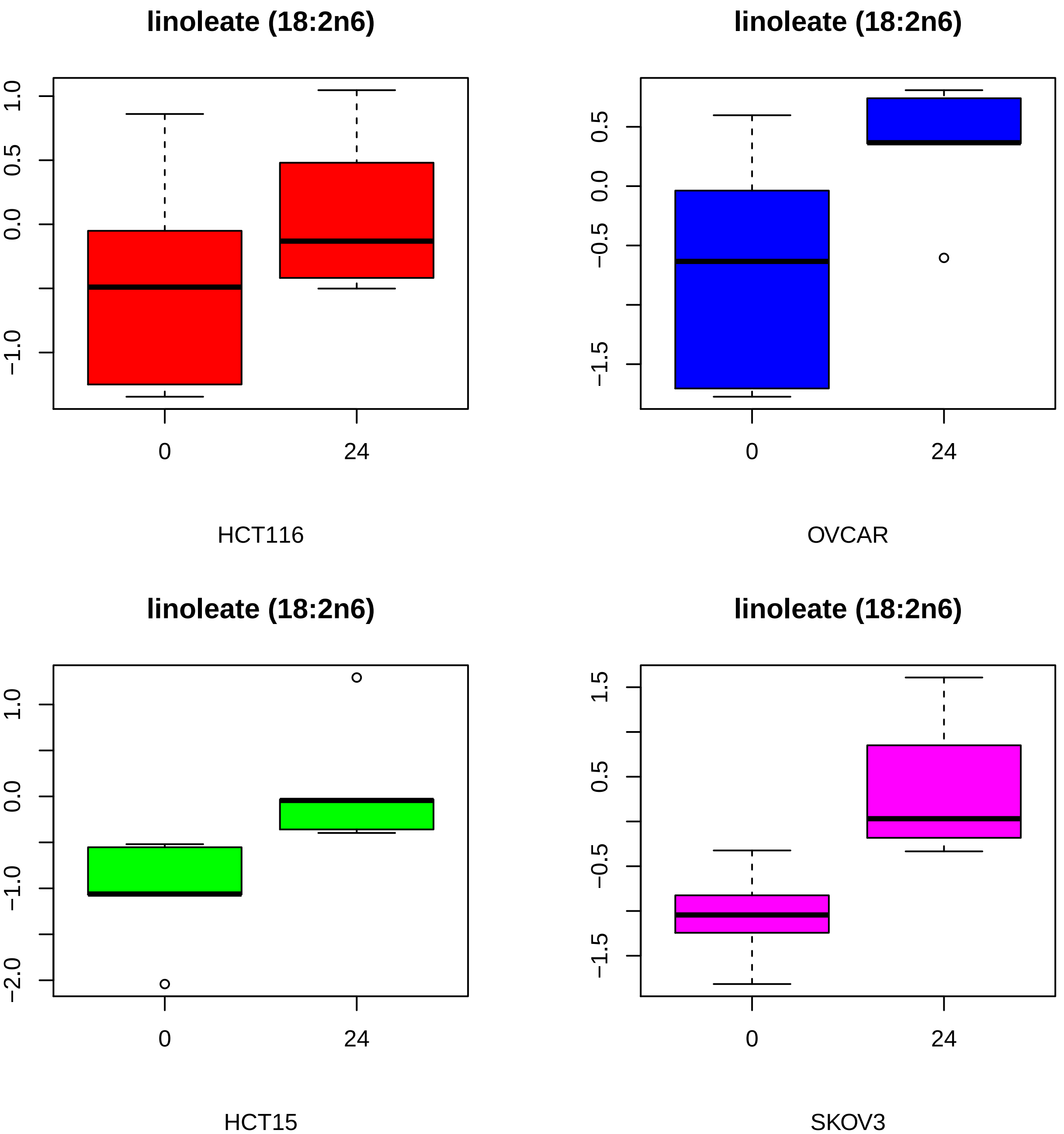
linoleate (18:2n6)
1.0
0.5
0.0
−1.0
0
24
HCT116
linoleate (18:2n6)
0.5
0.0
−0.5
−1.5
0
24
OVCAR
linoleate (18:2n6)
1.0
0.0
−1.0
−2.0
0
24
HCT15
linoleate (18:2n6)
1.5
0.5
−0.5
−1.5
0
24
SKOV3

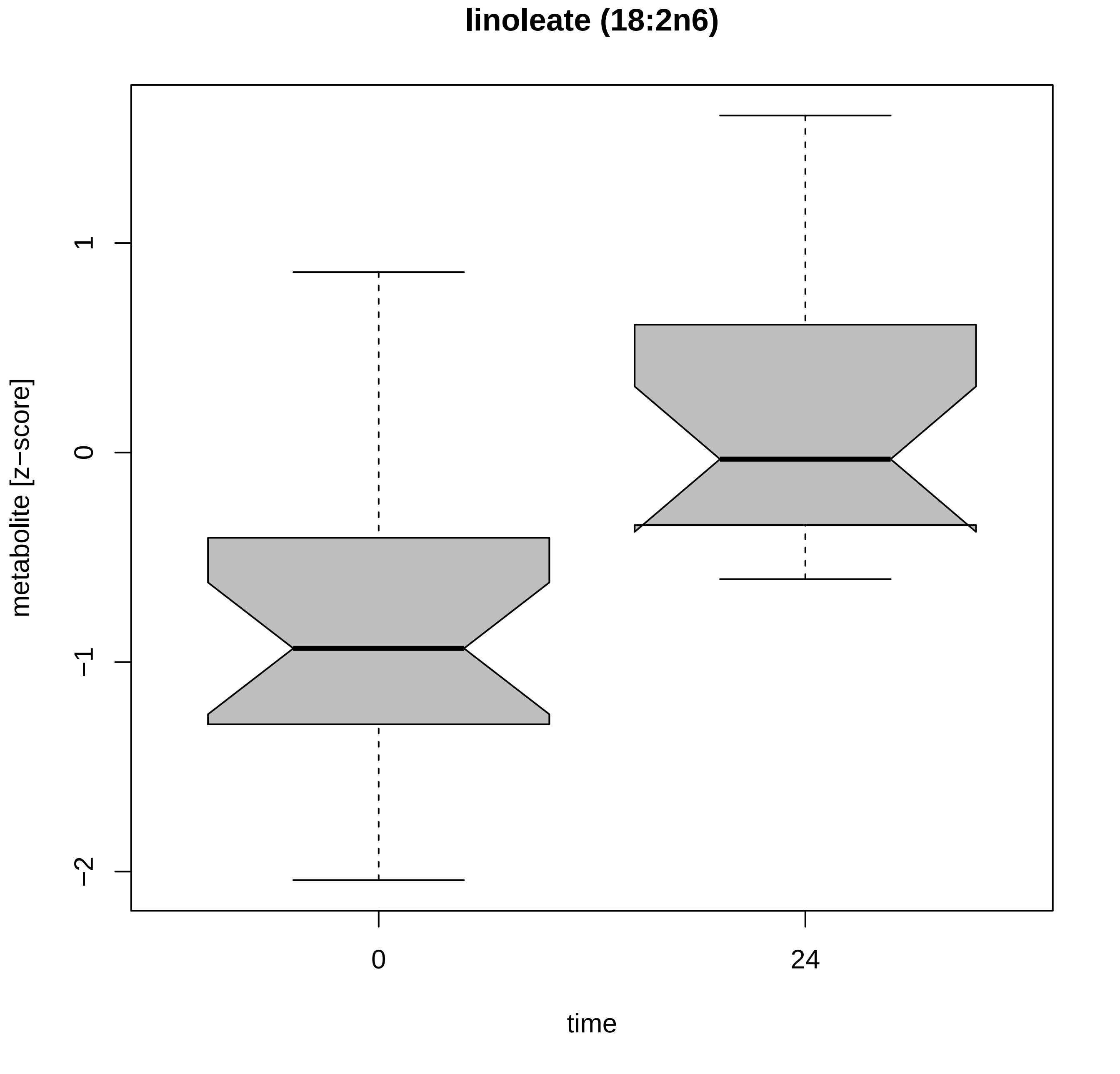

linoleate (18:2n6)
metabolite [z−score]
1
0
−1
−2
0
24
time

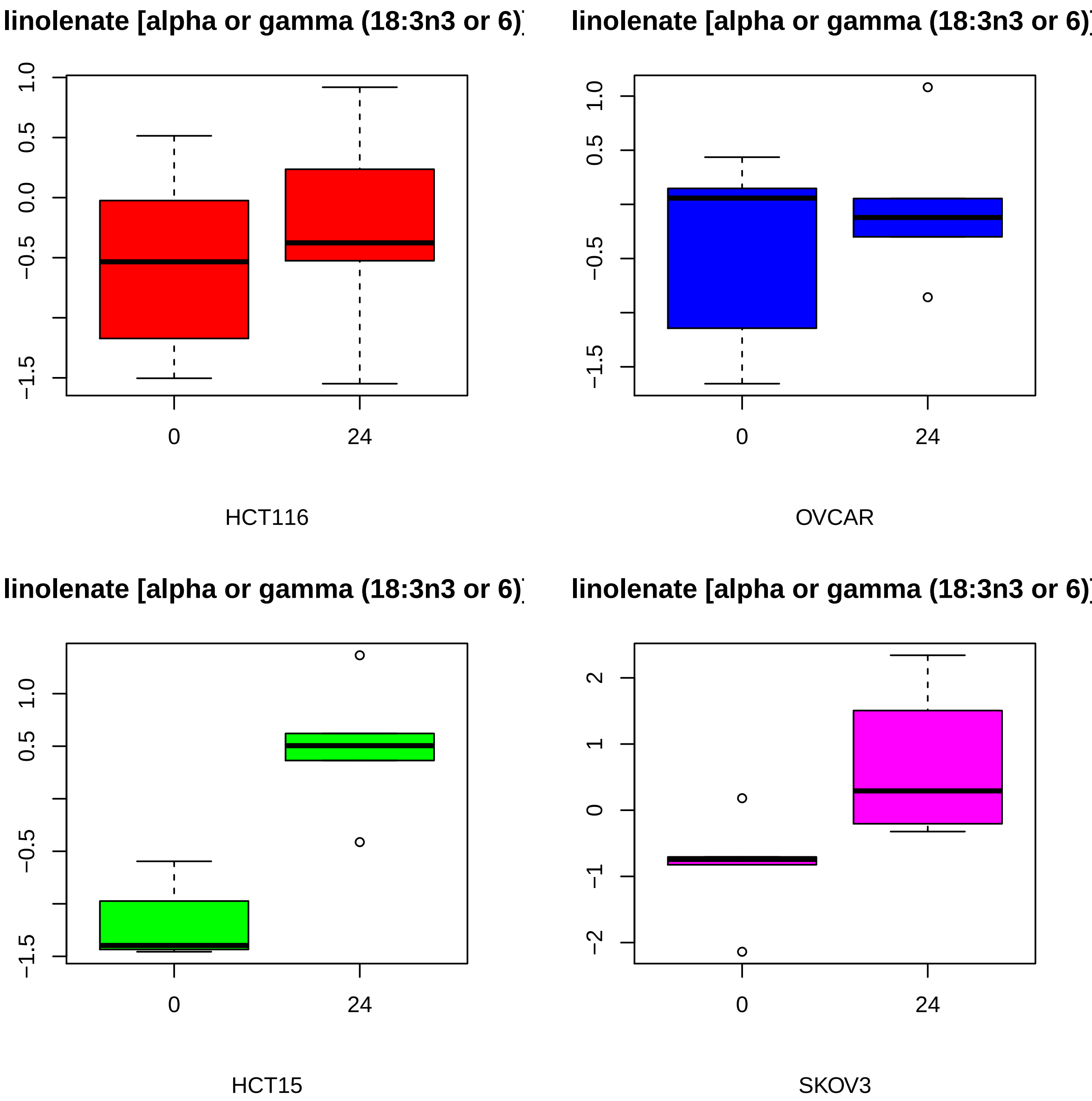
linolenate [alpha or gamma (18:3n3 or 6)]
1.0
0.5
0.0
−0.5
−1.5
0
24
HCT116
linolenate [alpha or gamma (18:3n3 or 6)]
1.0
0.5
−0.5
−1.5
0
24
OVCAR
linolenate [alpha or gamma (18:3n3 or 6)]
1.0
0.5
−0.5
−1.5
0
24
HCT15
linolenate [alpha or gamma (18:3n3 or 6)]
2
1
0
−1
−2
0
24
SKOV3

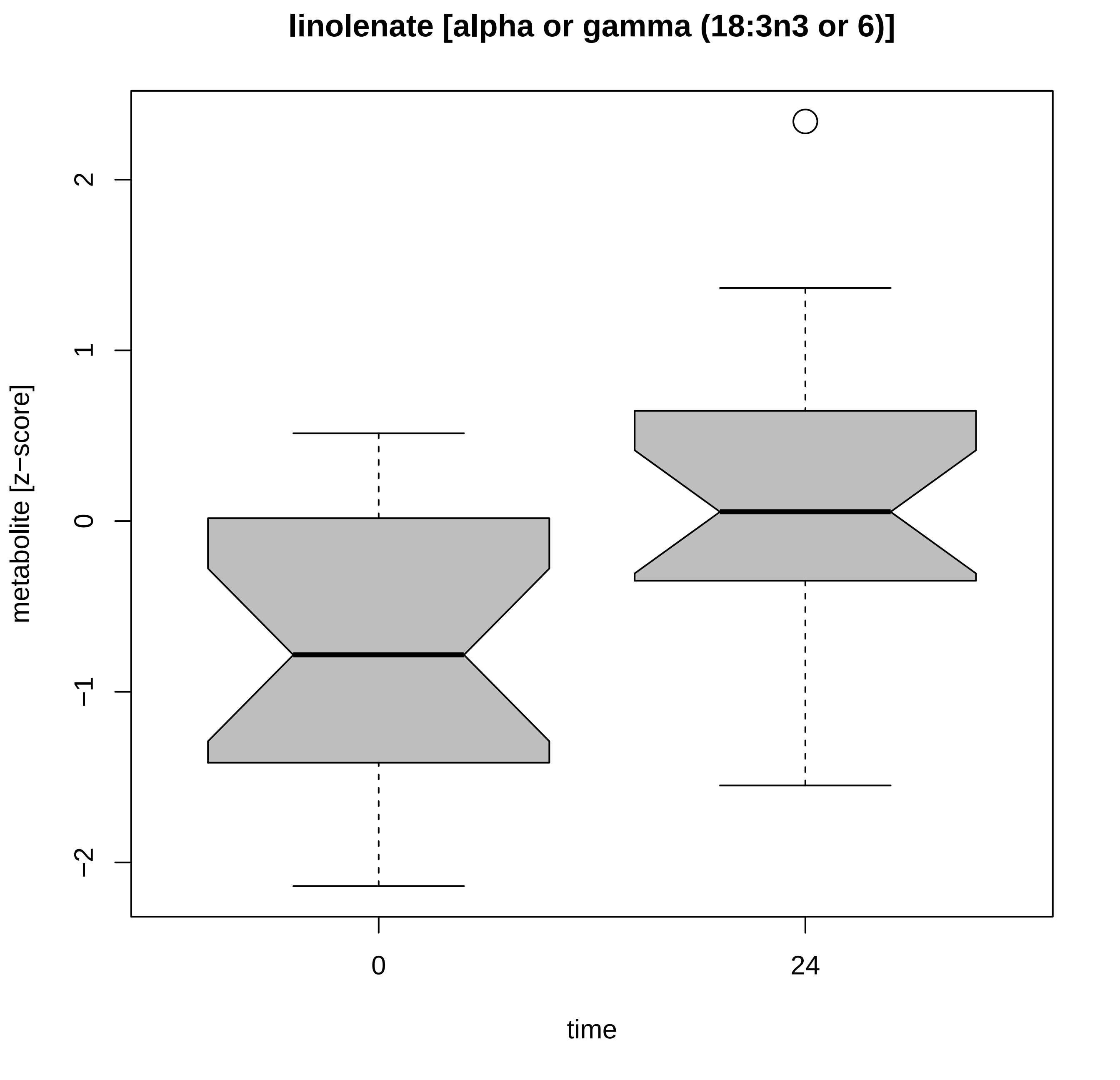

linolenate [alpha or gamma (18:3n3 or 6)]
metabolite [z−score]
2
1
0
−1
−2
0
24
time

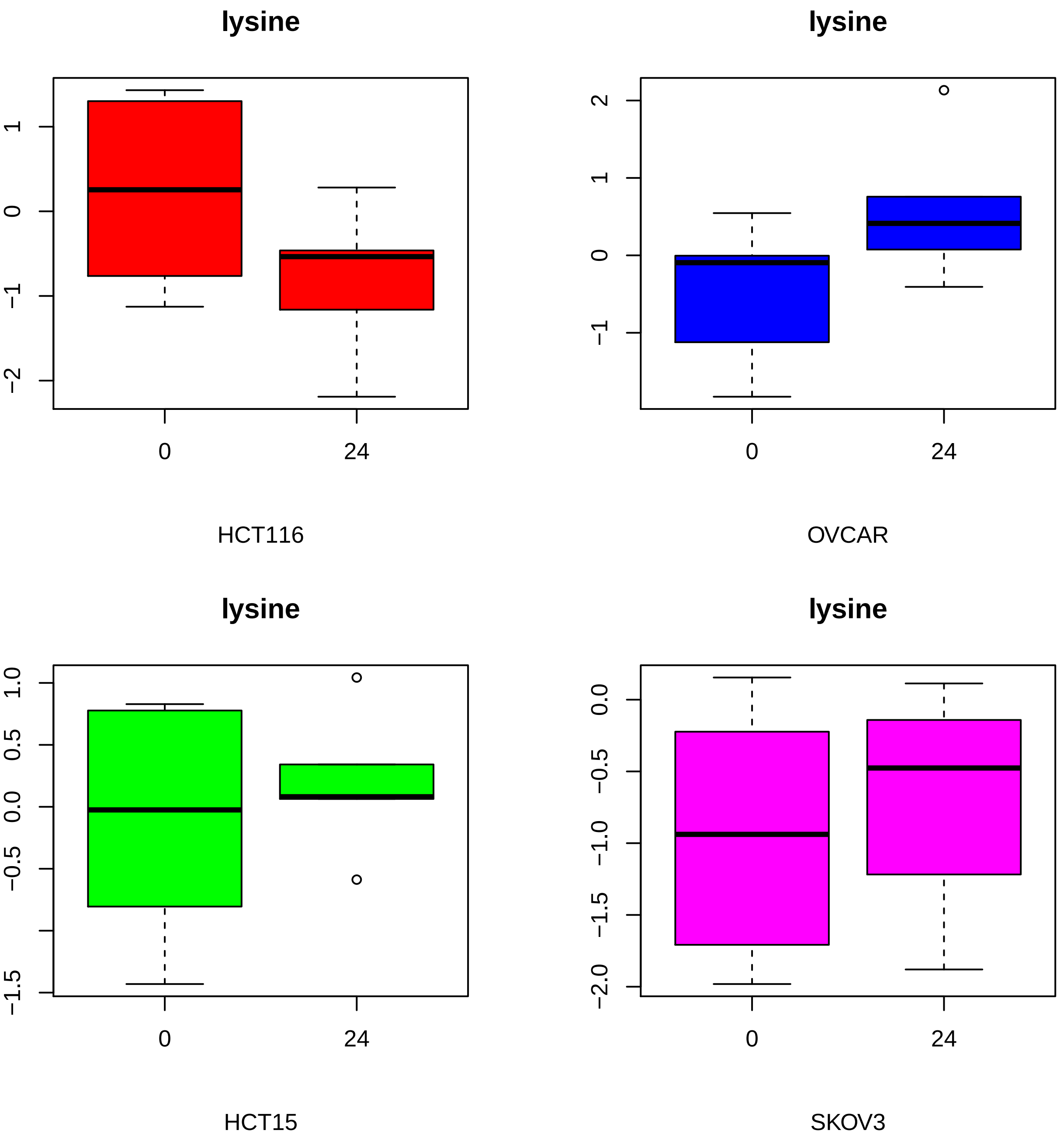
lysine
1
0
−1
−2
0
24
HCT116
lysine
2
1
0
−1
0
24
OVCAR
lysine
1.0
0.5
0.0
−0.5
−1.5
0
24
HCT15
lysine
0.0
−0.5
−1.0
−1.5
−2.0
0
24
SKOV3

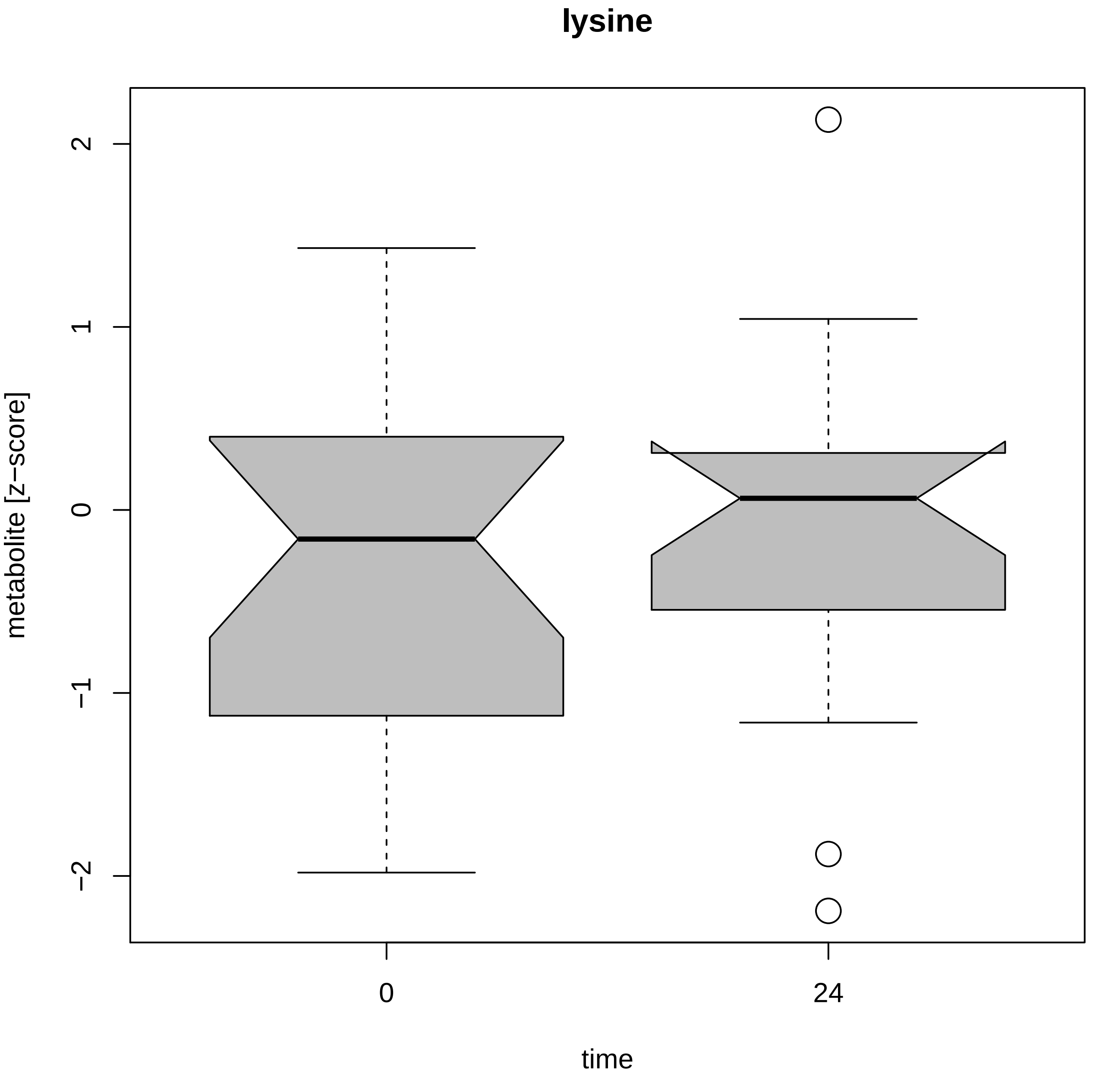
lysine
2
1
0
−1
−2
metabolite [z−score]
0
24
time

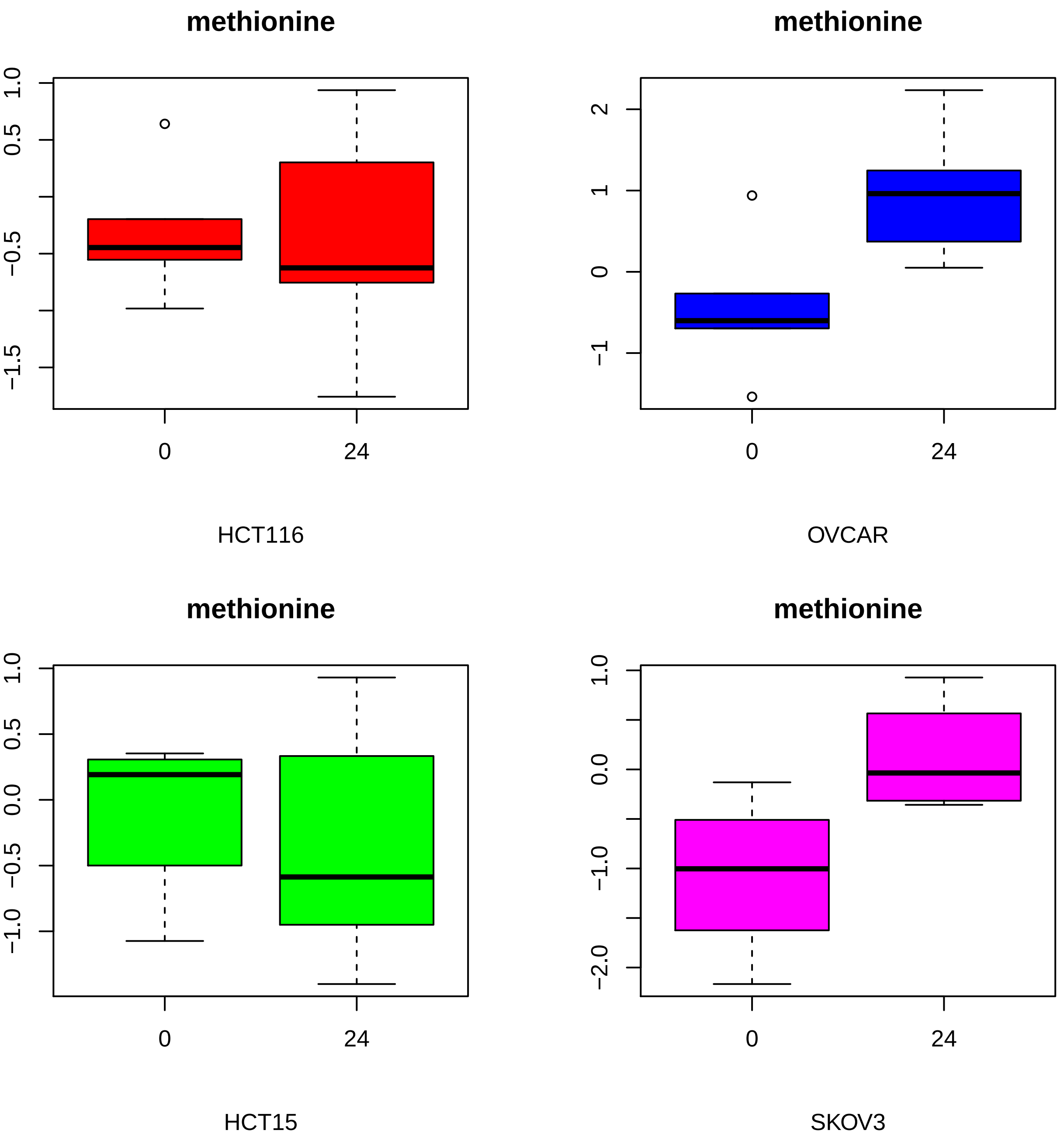

methionine
1.0
0.5
−0.5
−1.5
0
24
HCT116
methionine
2
1
0
−1
0
24
OVCAR
methionine
1.0
0.5
0.0
−0.5
−1.0
0
24
HCT15
methionine
1.0
0.0
−1.0
−2.0
0
24
SKOV3

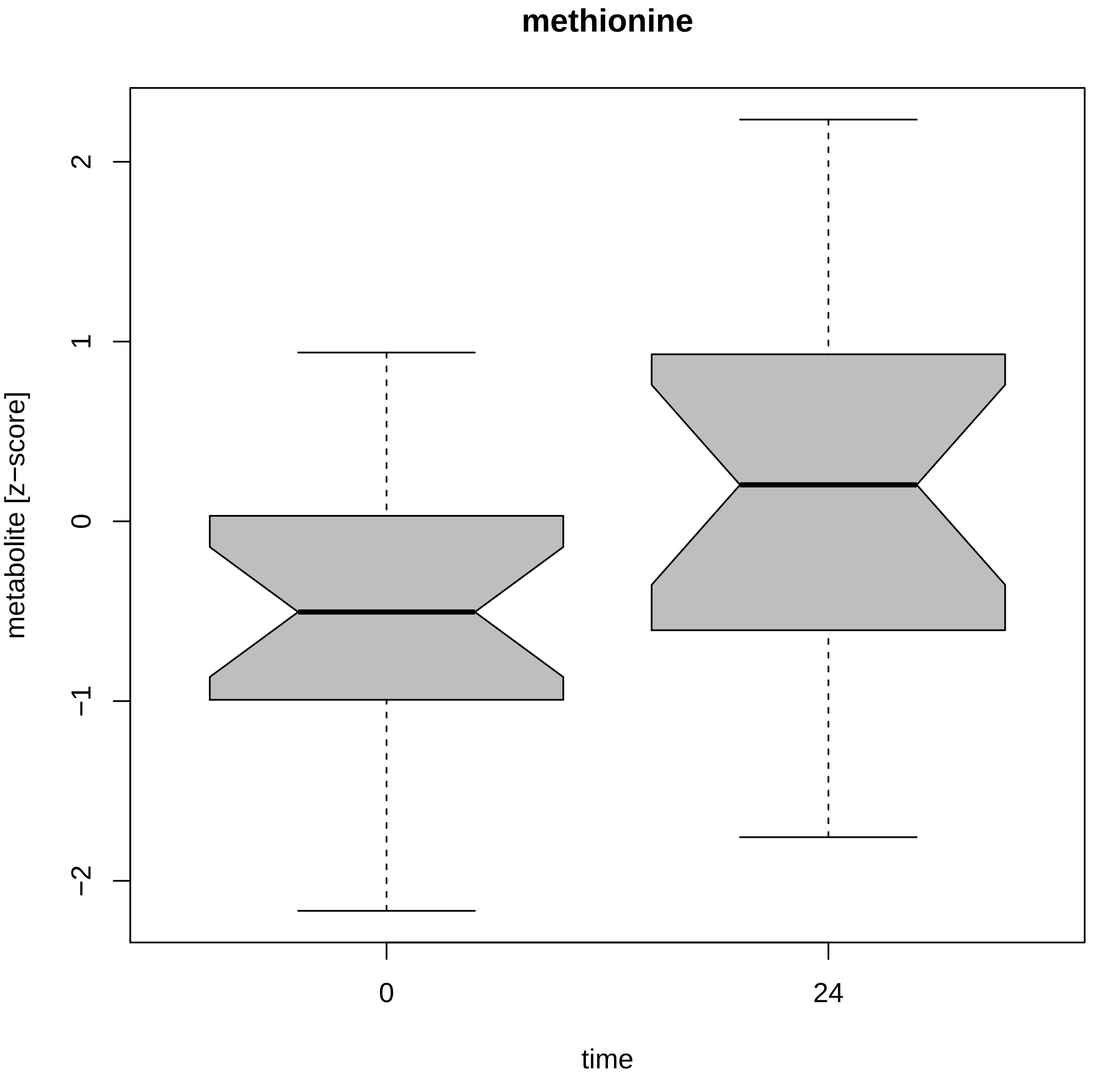
methionine
metabolite [z−score]
2
1
0
−1
−2
0
24
time

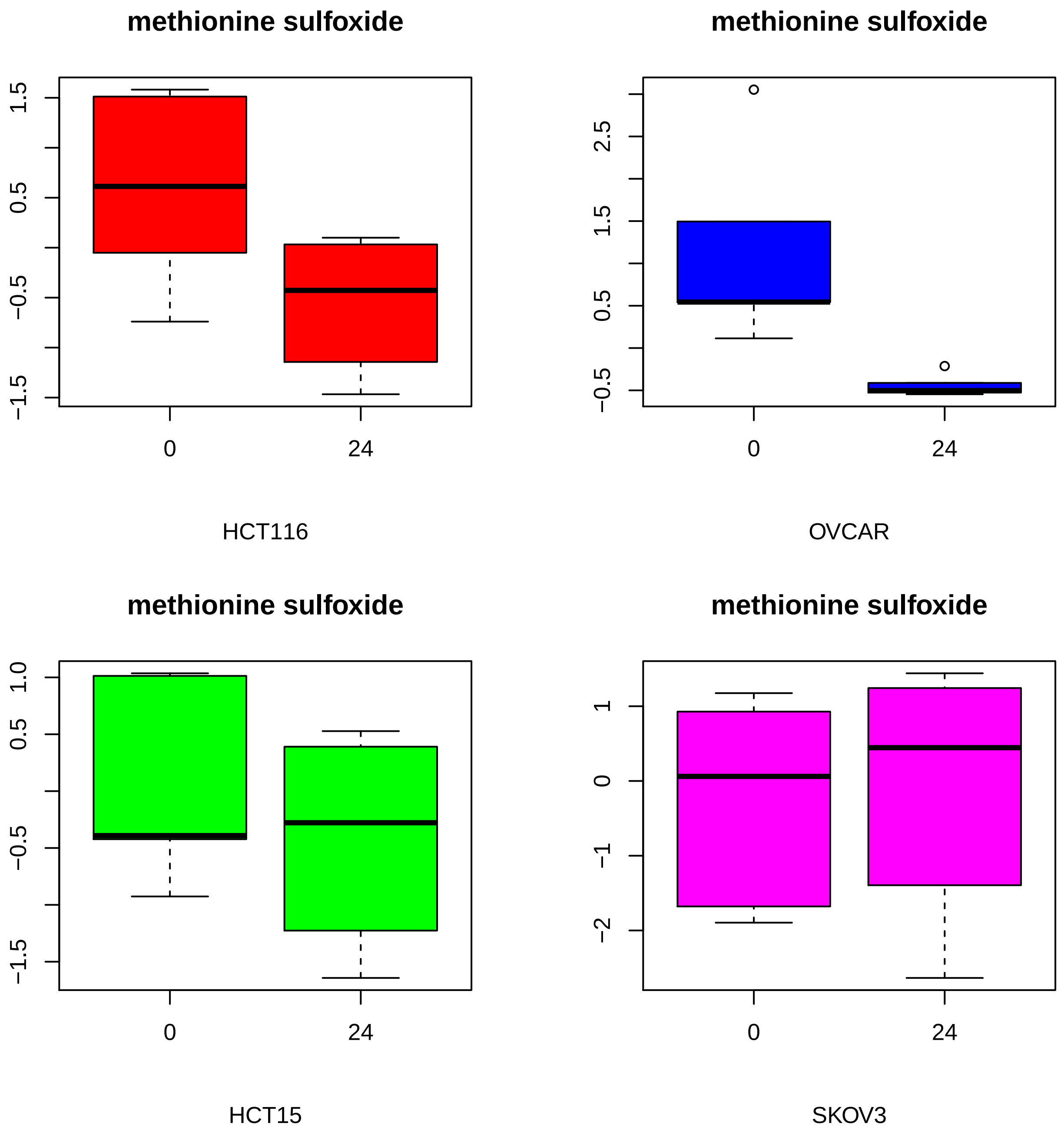
methionine sulfoxide
1.5
0.5
−0.5
−1.5
0
24
HCT116
methionine sulfoxide
2.5
1.5
0.5
−0.5
0
24
OVCAR
methionine sulfoxide
1.0
0.5
−0.5
−1.5
0
24
HCT15
methionine sulfoxide
1
0
−1
−2
0
24
SKOV3

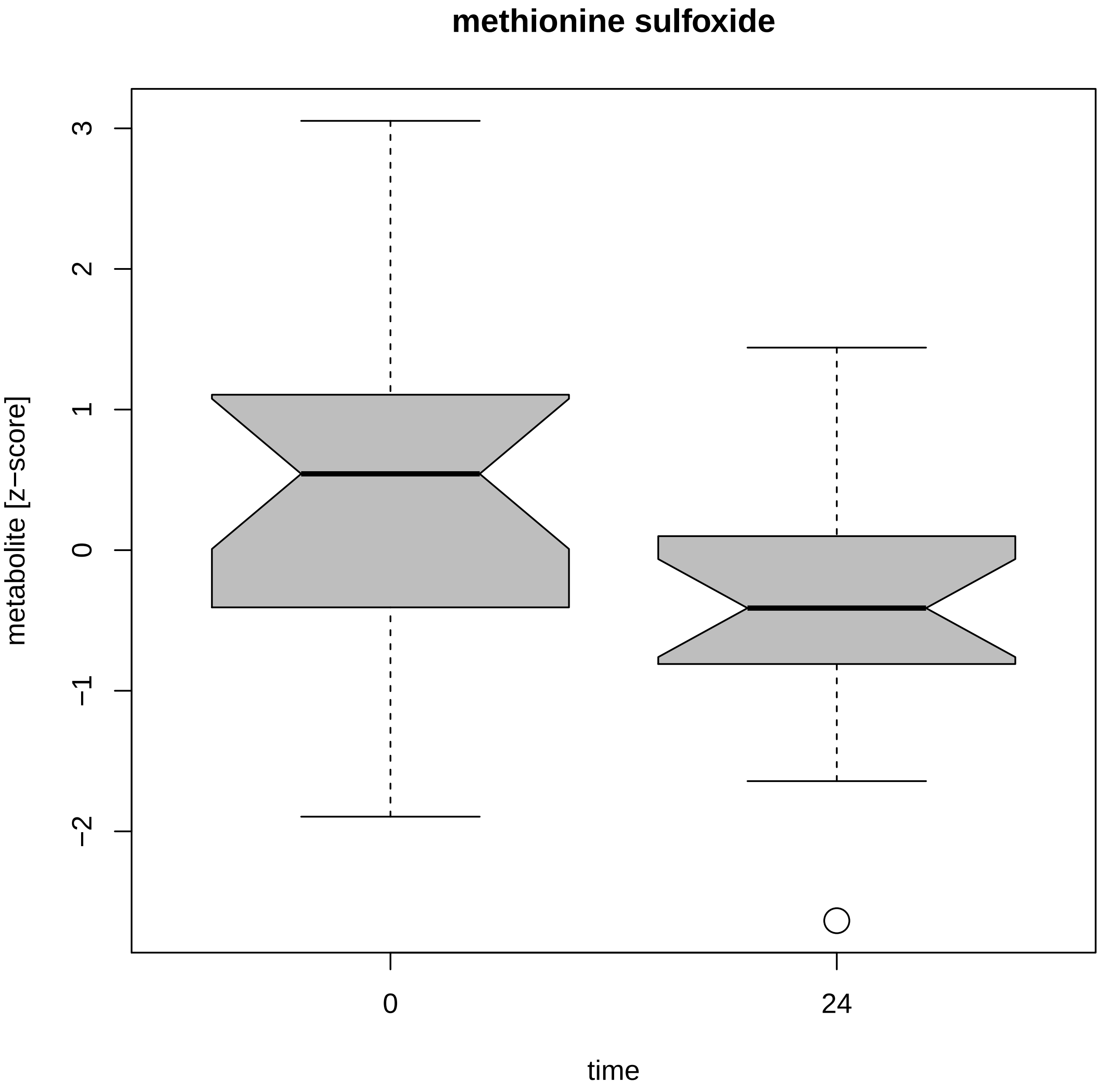
methionine sulfoxide
metabolite [z−score]
3
2
1
0
−1
−2
0
24
time

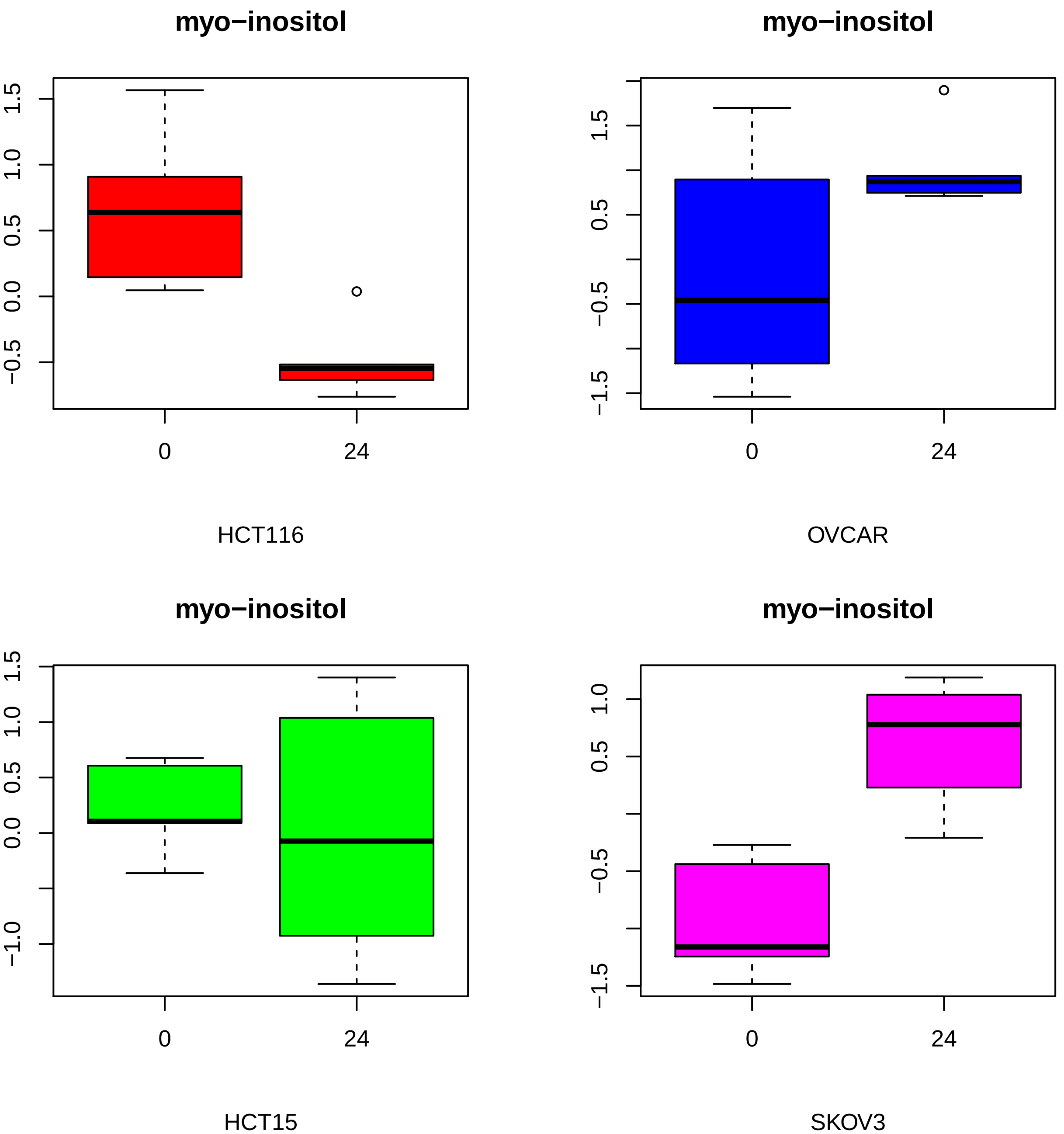
myo−inositol
1.5
1.0
0.5
0.0
−0.5
0
24
HCT116
myo−inositol
1.5
0.5
−0.5
−1.5
0
24
OVCAR
myo−inositol
1.5
1.0
0.5
0.0
−1.0
0
24
HCT15
myo−inositol
1.0
0.5
−0.5
−1.5
0
24
SKOV3

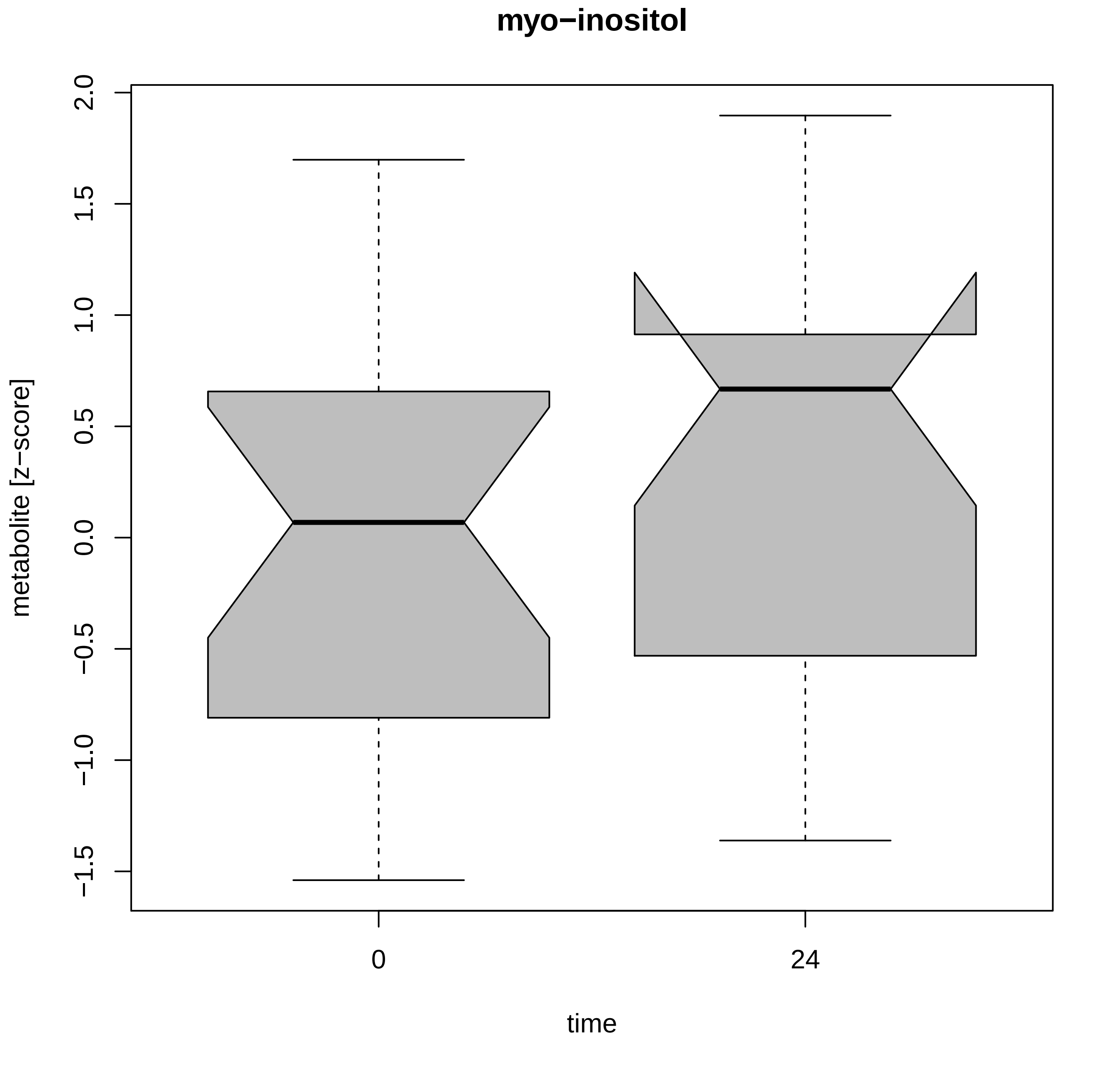

myo−inositol
metabolite [z−score]
2.0
1.5
1.0
0.5
0.0
−0.5
−1.0
−1.5
0
24
time

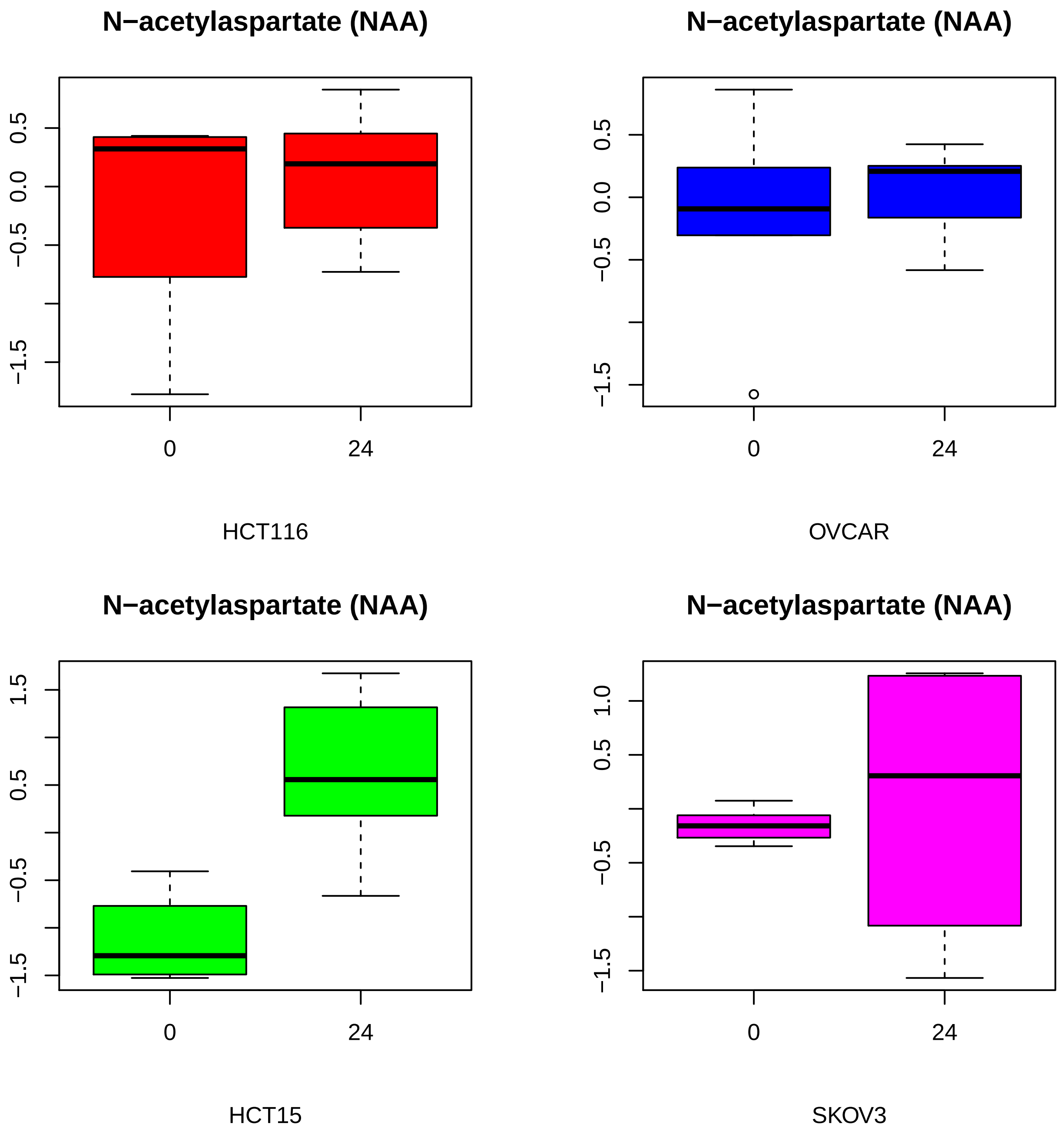
N−acetylaspartate (NAA)
0.5
0.0
−0.5
−1.5
0
24
HCT116
N−acetylaspartate (NAA)
0.5
0.0
−0.5
−1.5
0
24
OVCAR
N−acetylaspartate (NAA)
1.5
0.5
−0.5
−1.5
0
24
HCT15
N−acetylaspartate (NAA)
1.0
0.5
−0.5
−1.5
0
24
SKOV3

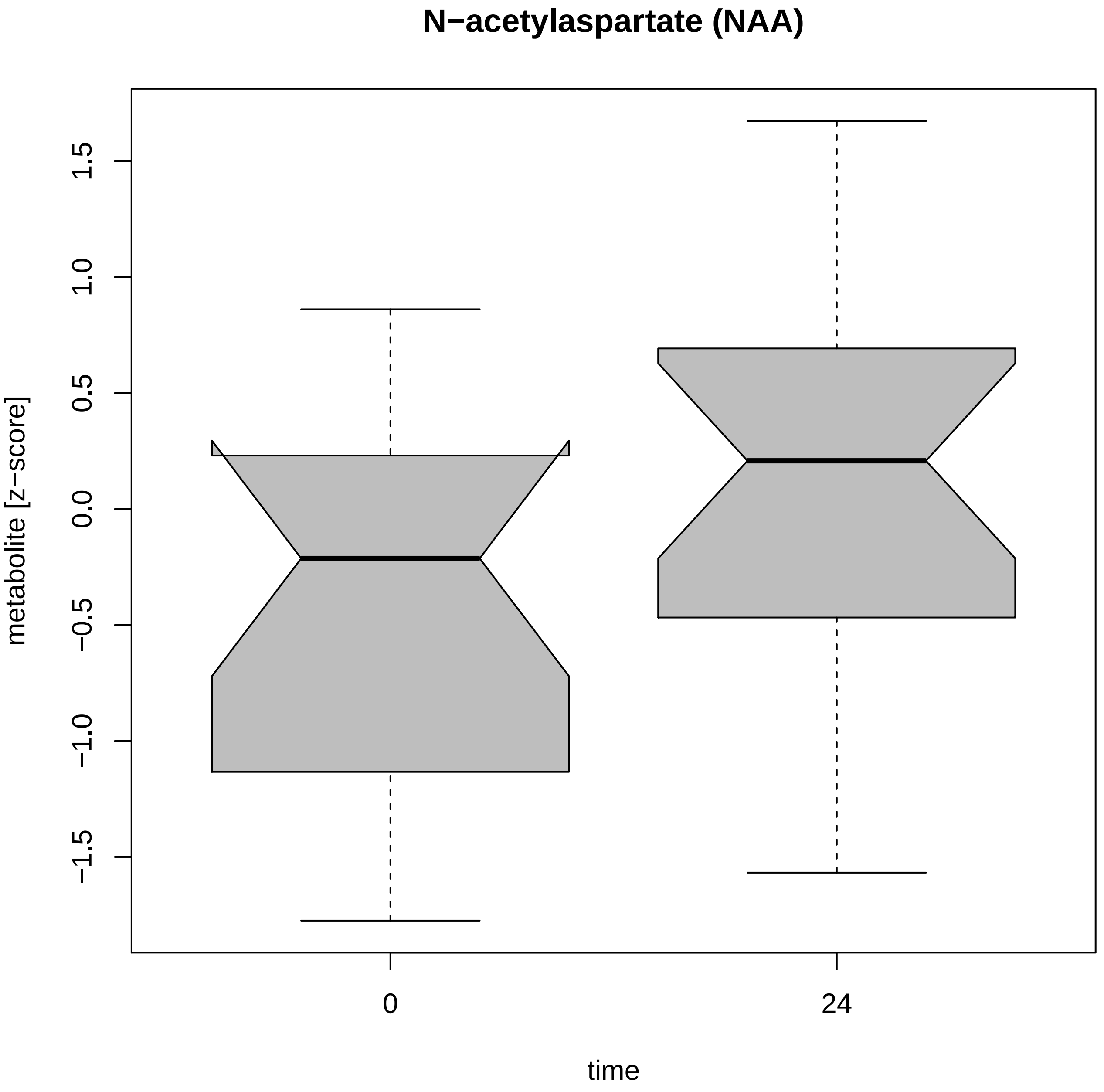
N−acetylaspartate (NAA)
metabolite [z−score]
1.5
1.0
0.5
0.0
−0.5
−1.0
−1.5
0
24
time

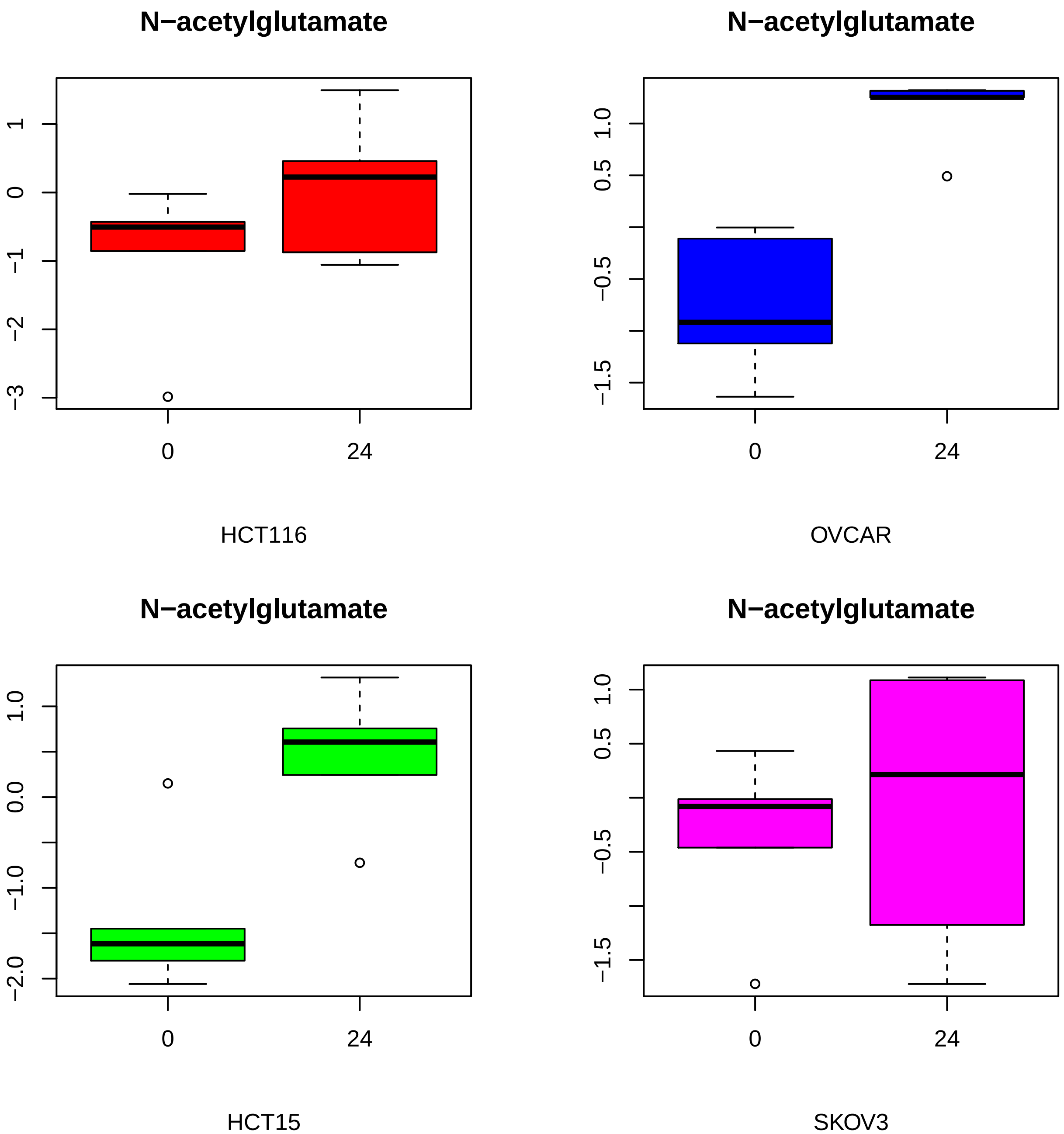
N−acetylglutamate
1
0
−1
−2
−3
0
24
HCT116
N−acetylglutamate
1.0
0.5
−0.5
−1.5
0
24
OVCAR
N−acetylglutamate
1.0
0.0
−1.0
−2.0
0
24
HCT15
N−acetylglutamate
1.0
0.5
−0.5
−1.5
0
24
SKOV3

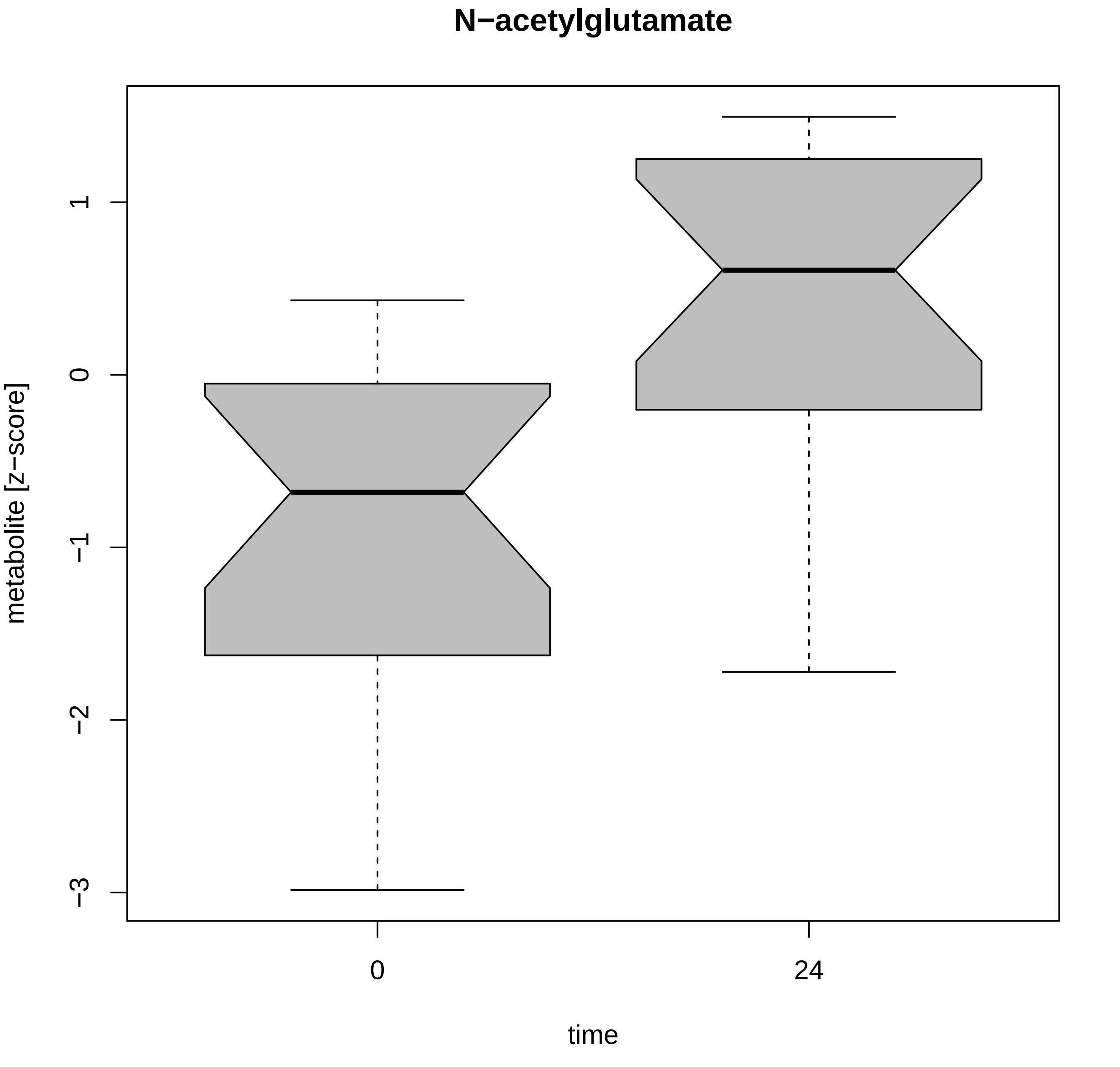

N−acetylglutamate
metabolite [z−score]
1
0
−1
−2
−3
0
24
time

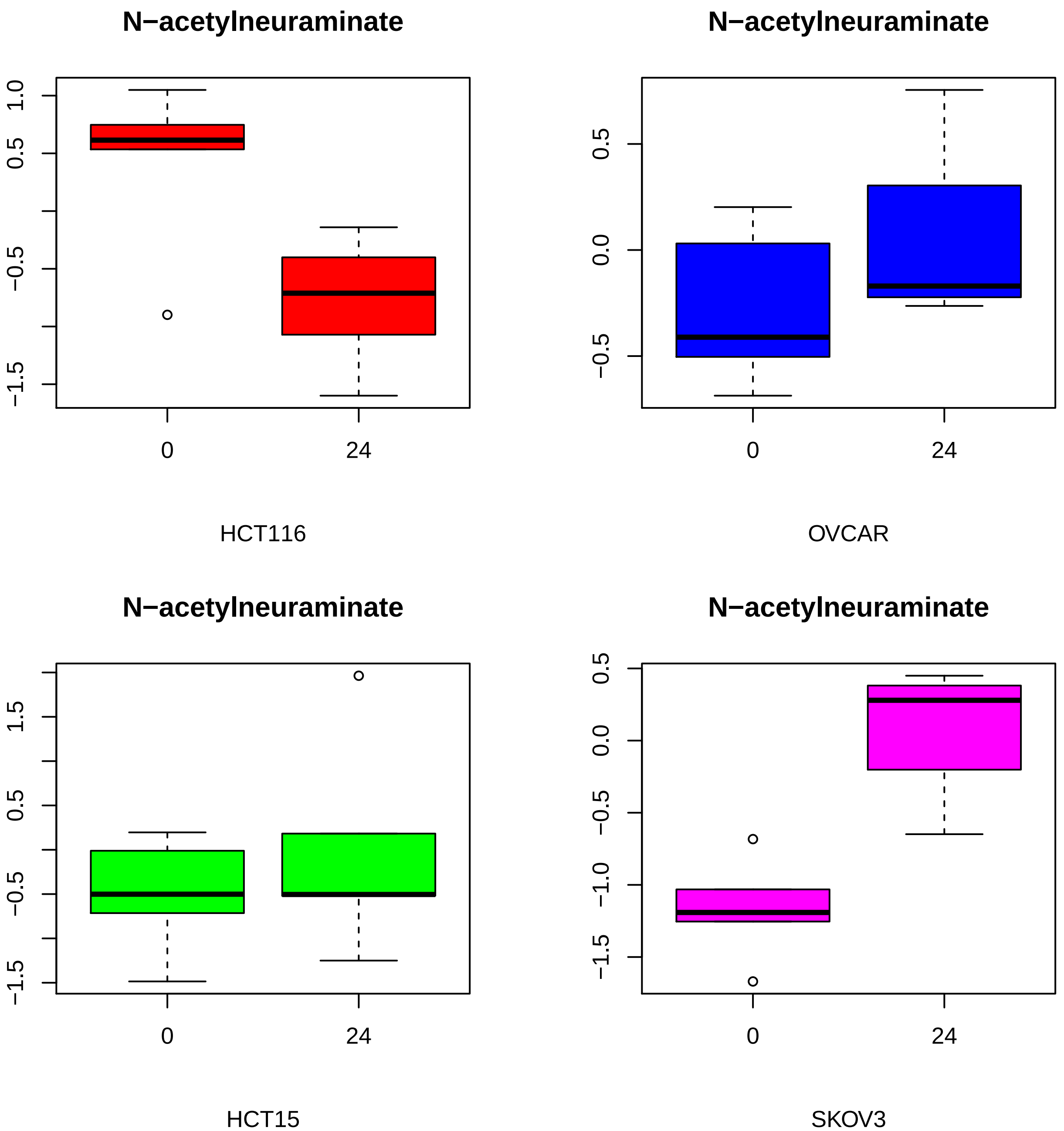
N−acetylneuraminate
1.0
0.5
−0.5
−1.5
0
24
HCT116
N−acetylneuraminate
0.5
0.0
−0.5
0
24
OVCAR
N−acetylneuraminate
1.5
0.5
−0.5
−1.5
0
24
HCT15
N−acetylneuraminate
0.5
0.0
−0.5
−1.0
−1.5
0
24
SKOV3

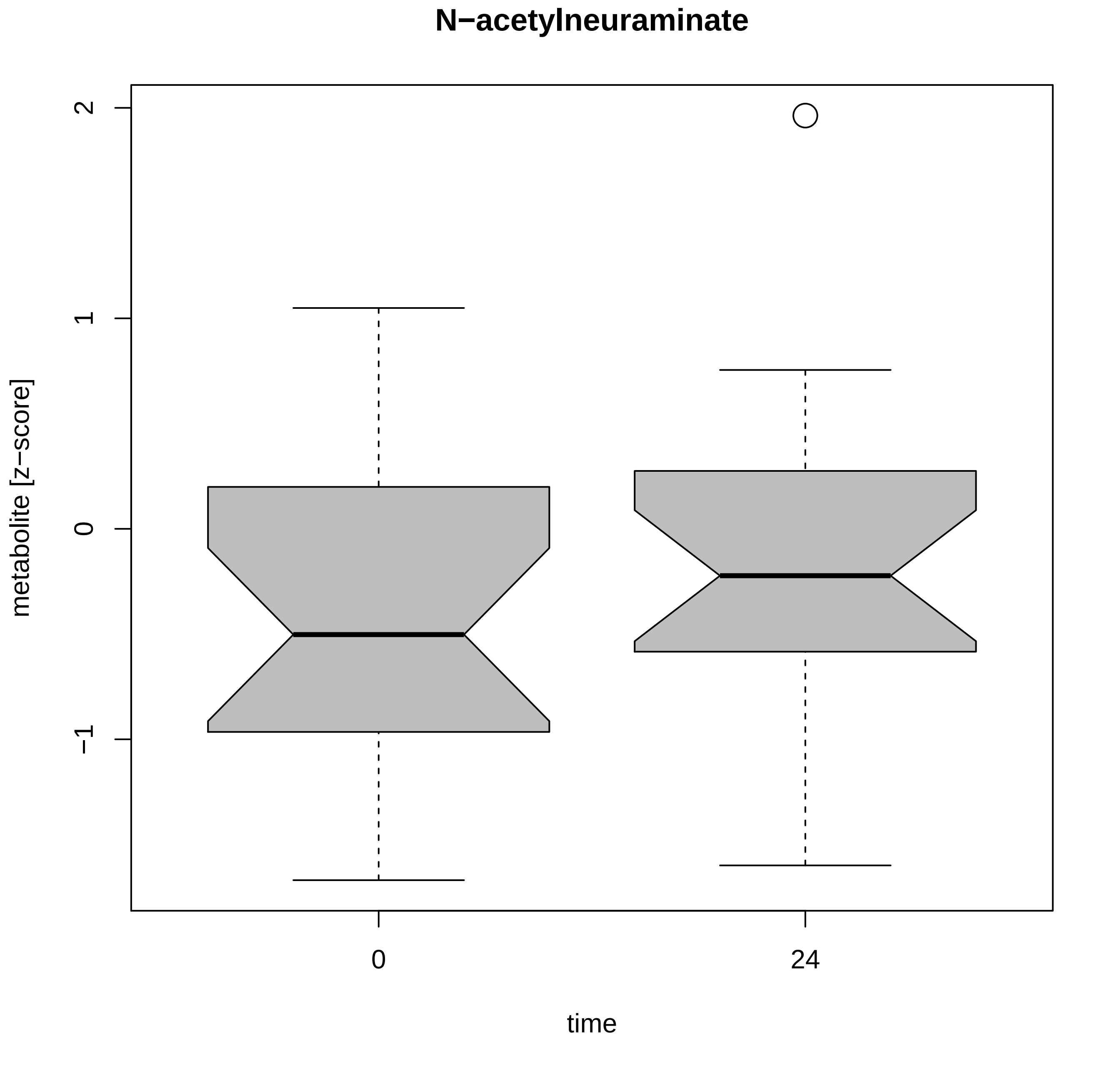

N−acetylneuraminate
metabolite [z−score]
2
1
0
−1
0
24
time

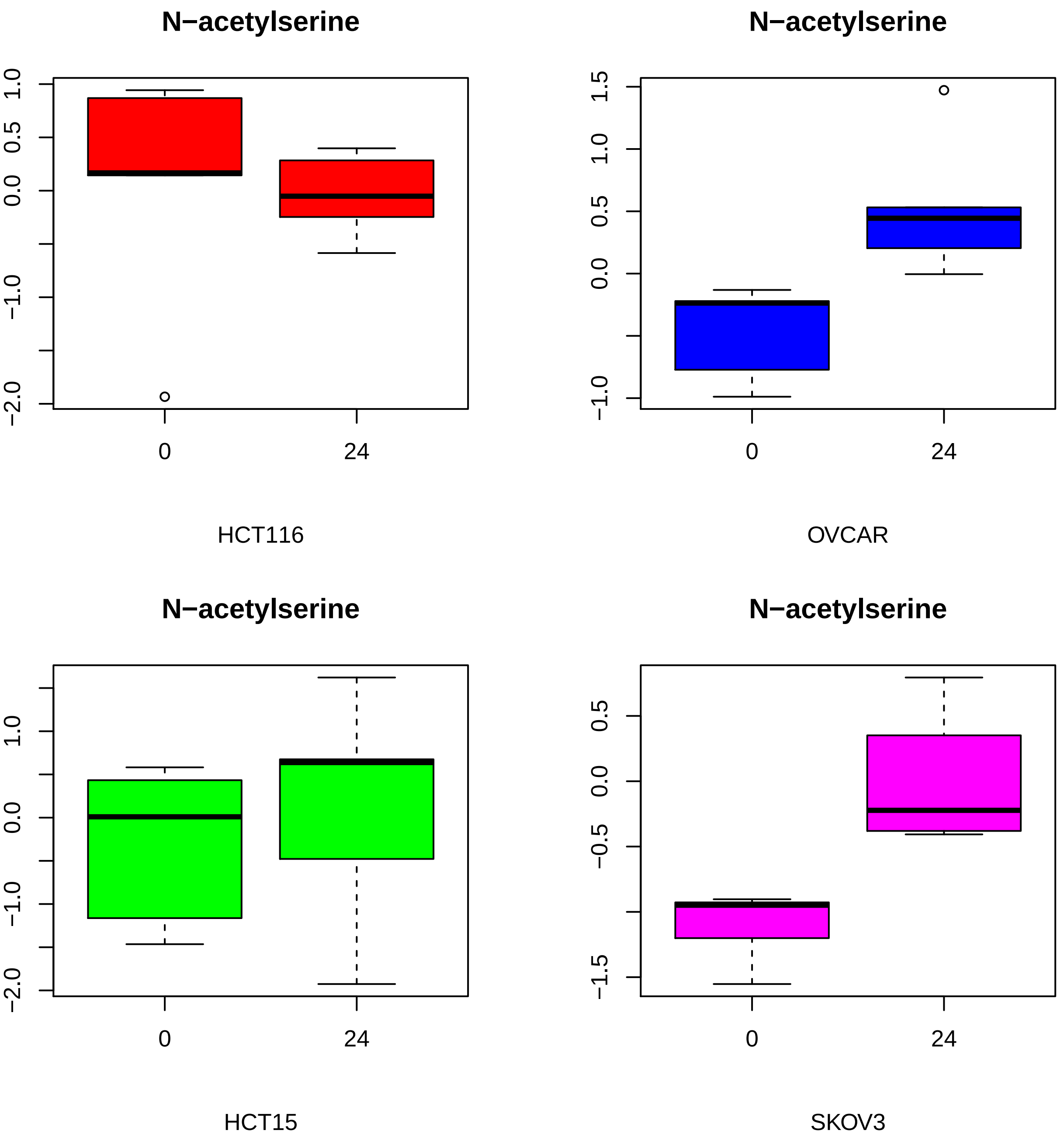
N−acetylserine
1.0
0.5
0.0
−1.0
−2.0
0
24
HCT116
N−acetylserine
1.5
1.0
0.5
0.0
−1.0
0
24
OVCAR
N−acetylserine
1.0
0.0
−1.0
−2.0
0
24
HCT15
N−acetylserine
0.5
0.0
−0.5
−1.5
0
24
SKOV3

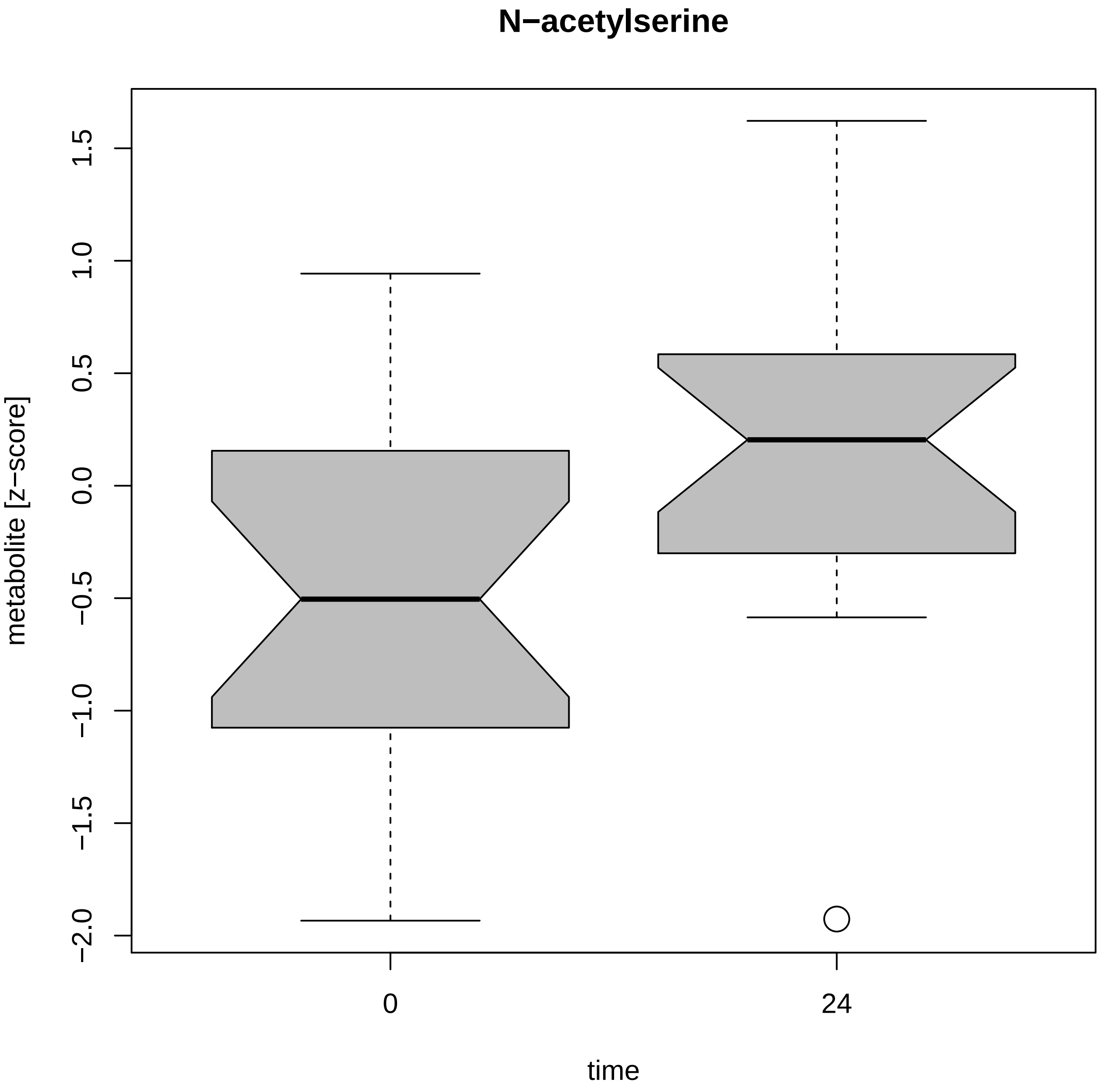

N−acetylserine
metabolite [z−score]
1.5
1.0
0.5
0.0
−0.5
−1.0
−1.5
−2.0
0
24
time

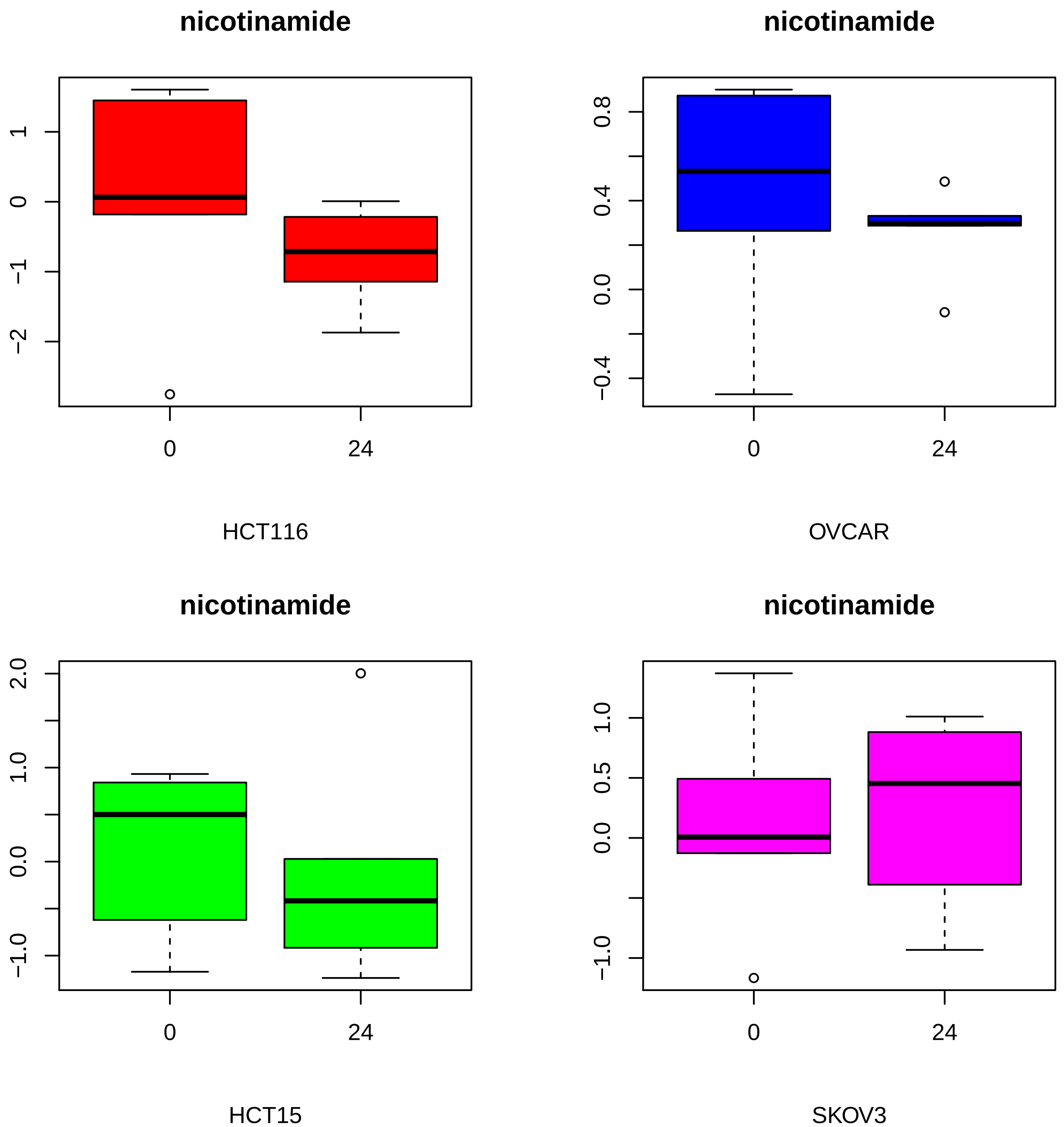

nicotinamide
1
0
−1
−2
0
24
HCT116
nicotinamide
0.8
0.4
0.0
−0.4
0
24
OVCAR
nicotinamide
2.0
1.0
0.0
−1.0
0
24
HCT15
nicotinamide
1.0
0.5
0.0
−1.0
0
24
SKOV3

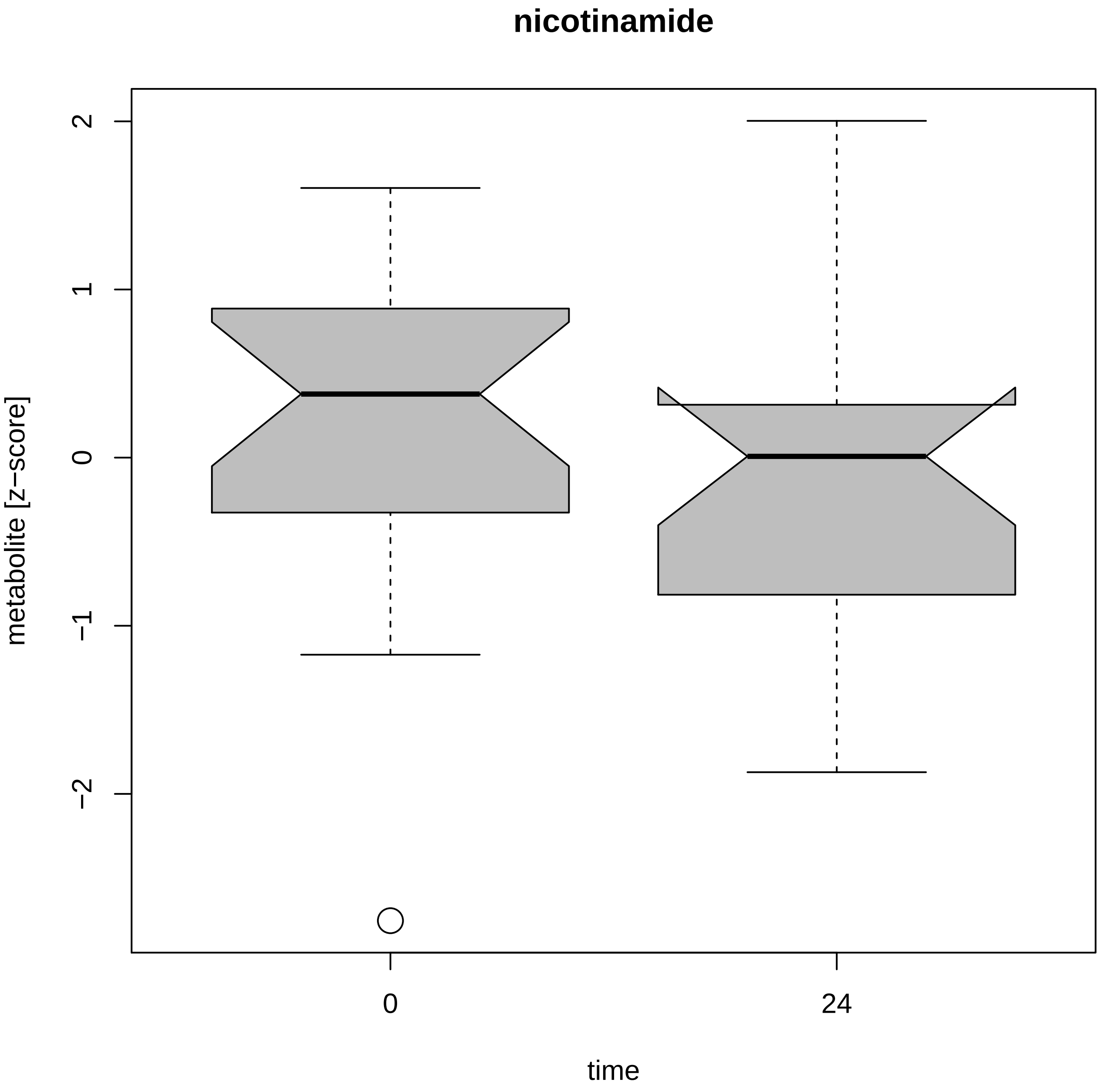

nicotinamide
metabolite [z−score]
2
1
0
−1
−2
0
24
time

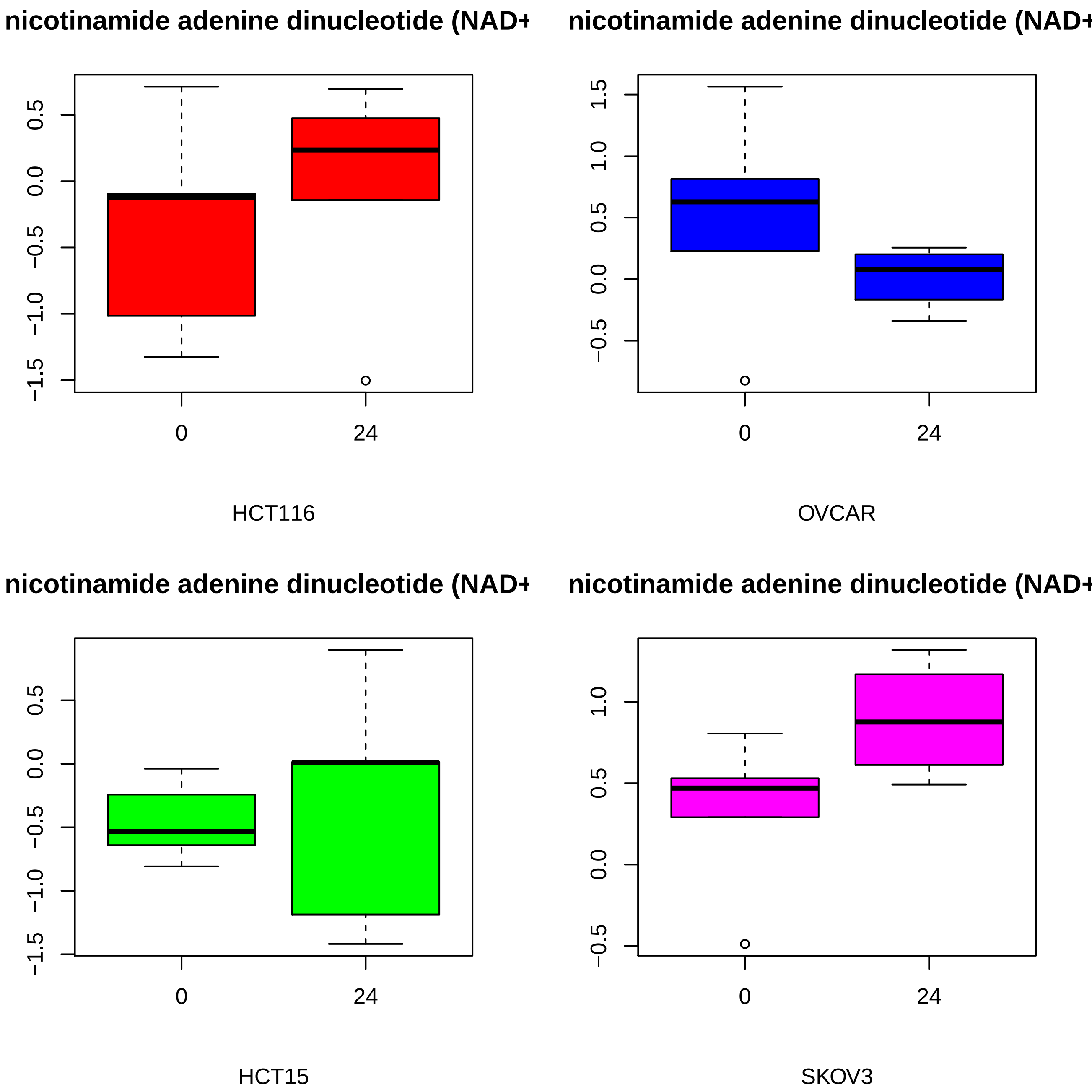

nicotinamide adenine dinucleotide (NAD+
0.5
0.0
−0.5
−1.0
−1.5
0
24
HCT116
nicotinamide adenine dinucleotide (NAD+
1.5
1.0
0.5
0.0
−0.5
0
24
OVCAR
nicotinamide adenine dinucleotide (NAD+
0.5
0.0
−0.5
−1.0
−1.5
0
24
HCT15
nicotinamide adenine dinucleotide (NAD+
1.0
0.5
0.0
−0.5
0
24
SKOV3

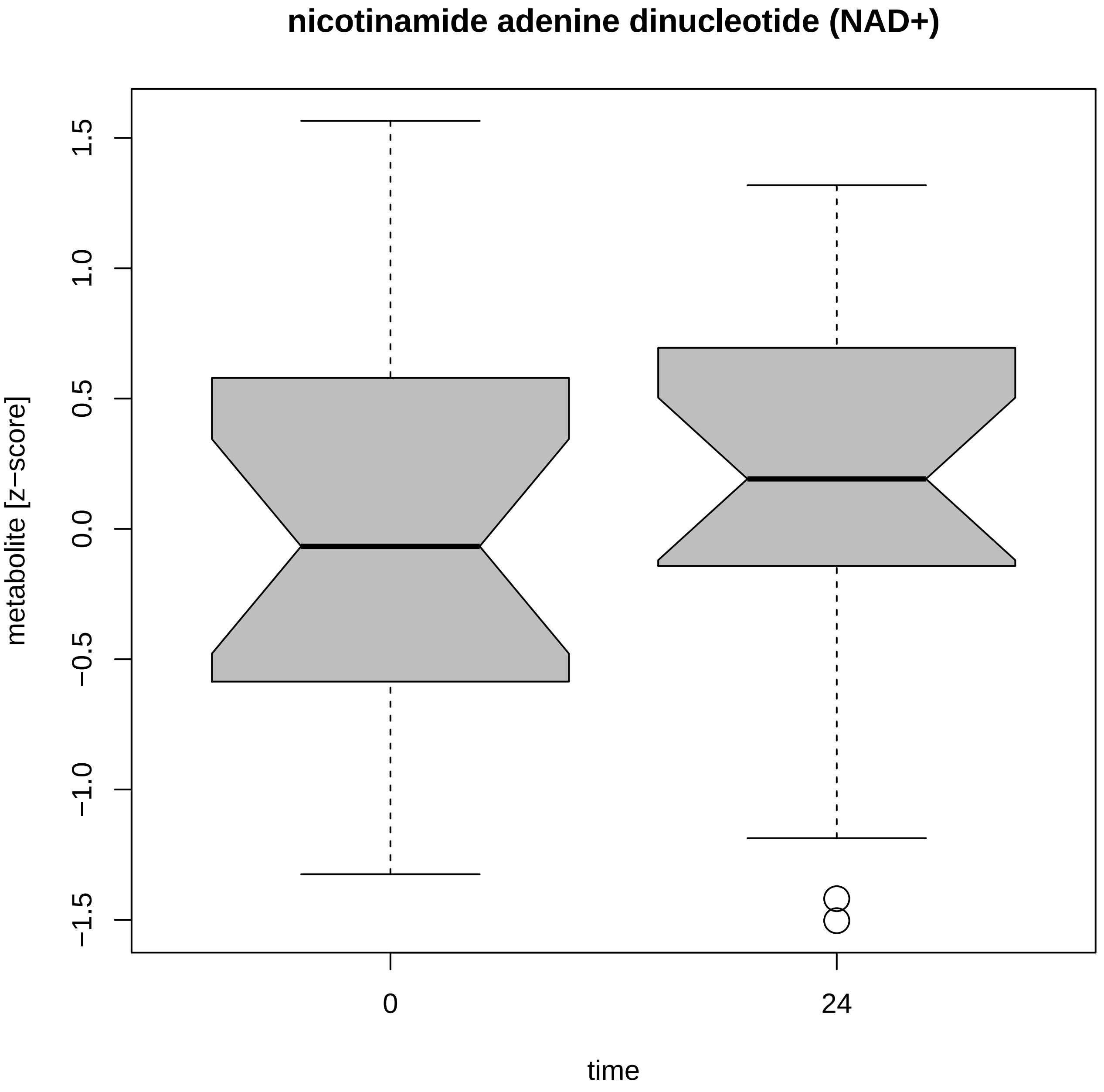
nicotinamide adenine dinucleotide (NAD+)
metabolite [z−score]
1.5
1.0
0.5
0.0
−0.5
−1.0
−1.5
0
24
time

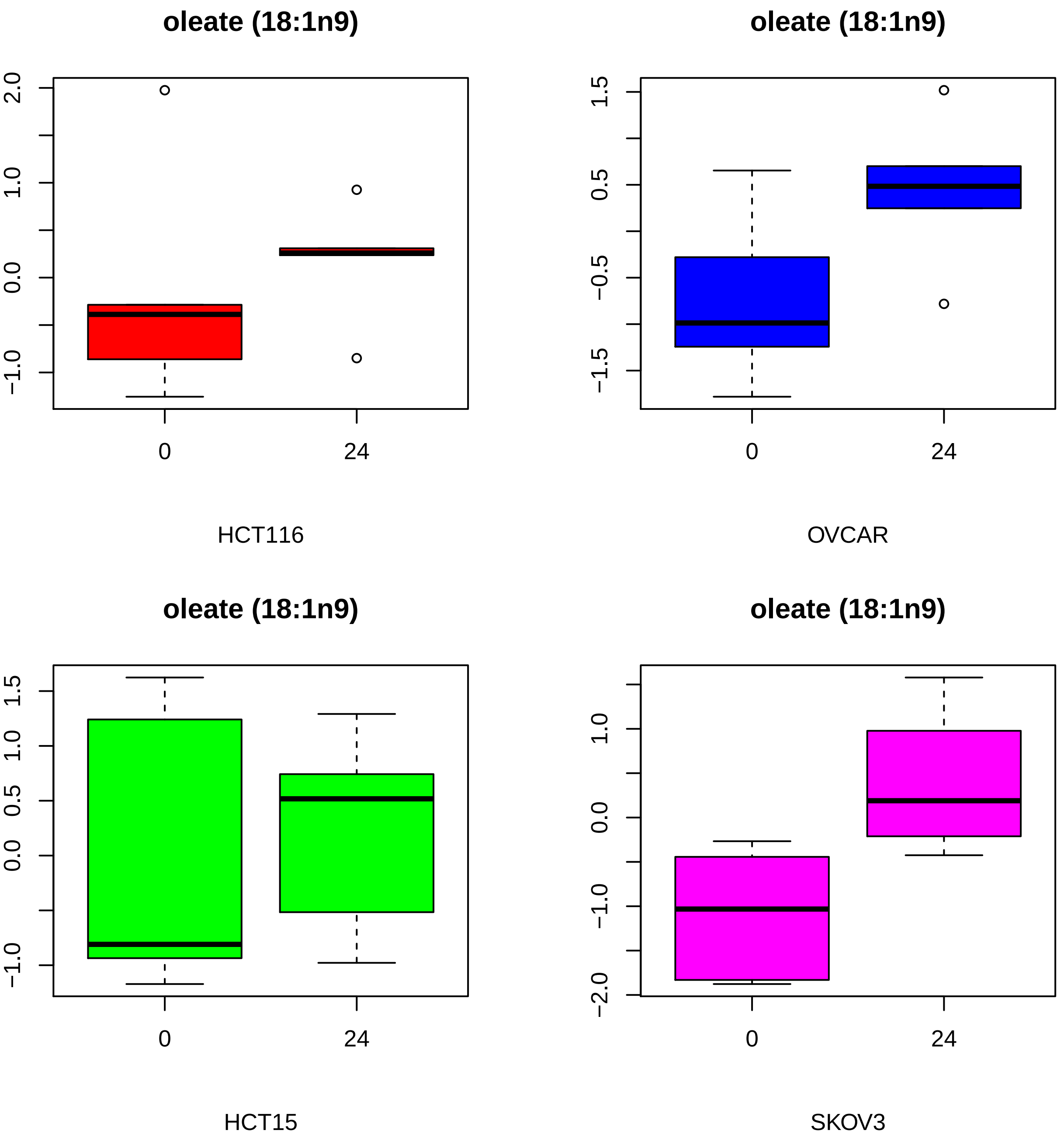
oleate (18:1n9)
2.0
1.0
0.0
−1.0
0
24
HCT116
oleate (18:1n9)
1.5
0.5
−0.5
−1.5
0
24
OVCAR
oleate (18:1n9)
1.5
1.0
0.5
0.0
−1.0
0
24
HCT15
oleate (18:1n9)
1.0
0.0
−1.0
−2.0
0
24
SKOV3

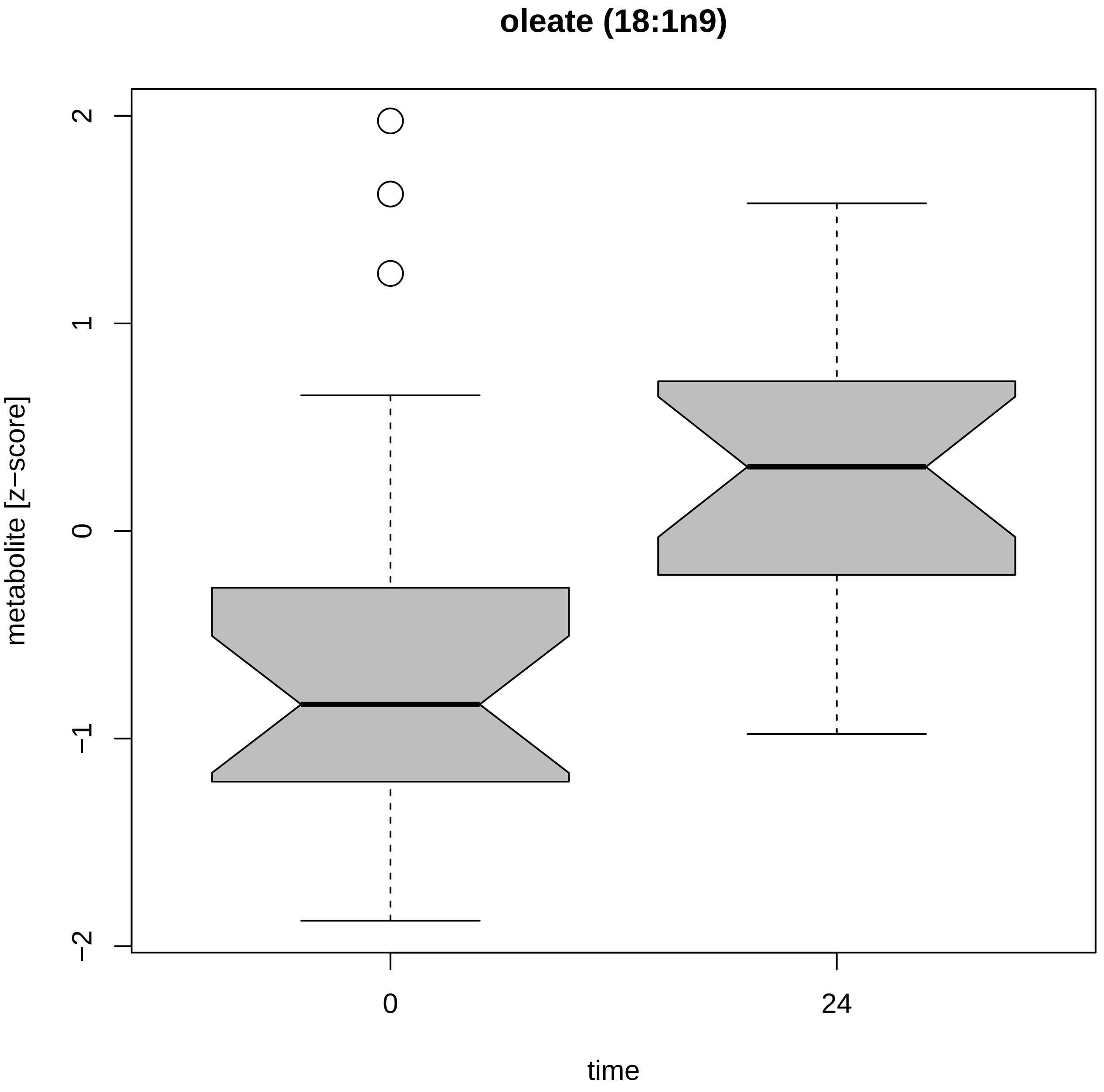

oleate (18:1n9)
metabolite [z−score]
2
1
0
−1
−2
0
24
time

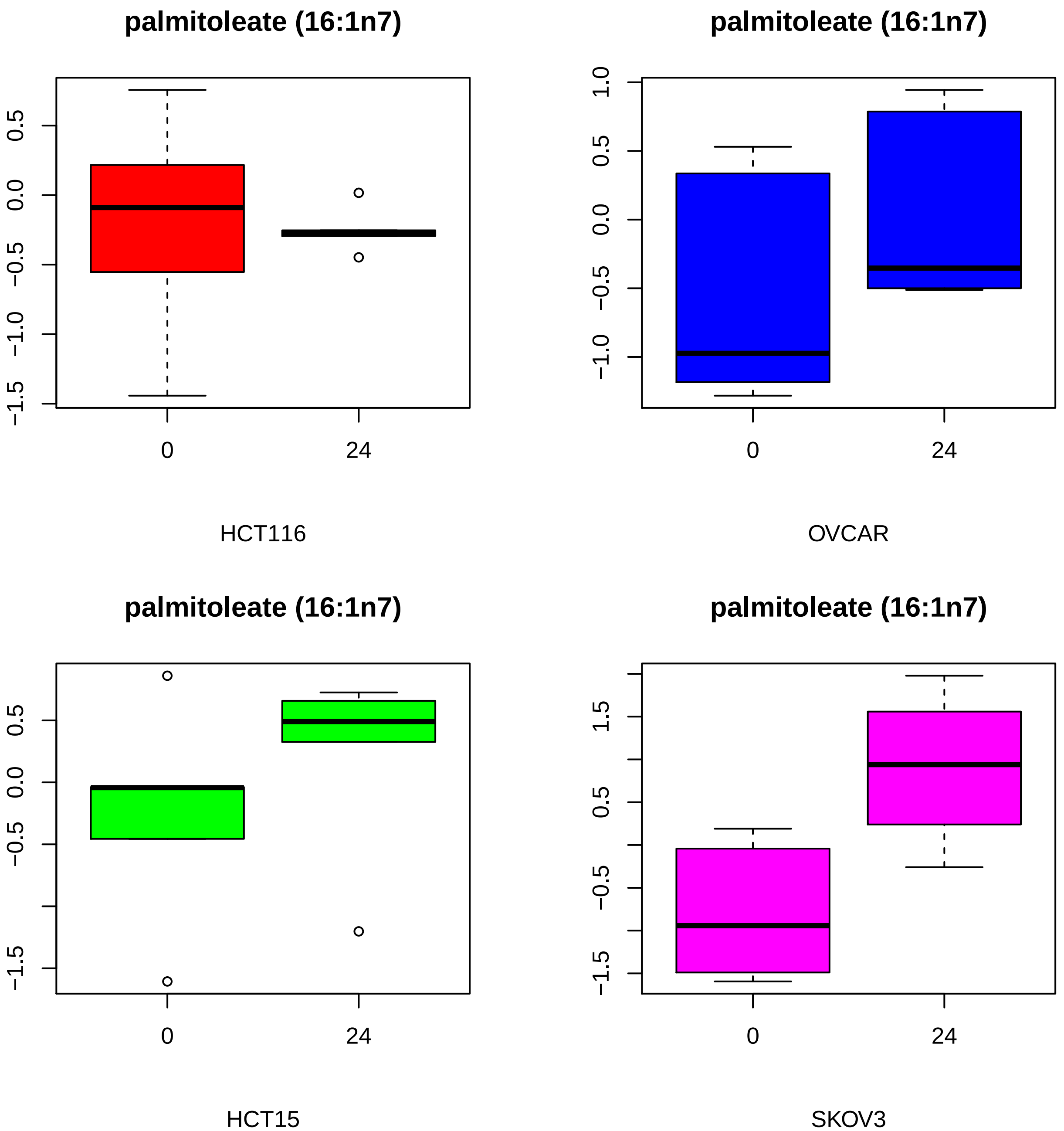

palmitoleate (16:1n7)
0.5
0.0
−0.5
−1.0
−1.5
0
24
HCT116
palmitoleate (16:1n7)
1.0
0.5
0.0
−0.5
−1.0
0
24
OVCAR
palmitoleate (16:1n7)
0.5
0.0
−0.5
−1.5
0
24
HCT15
palmitoleate (16:1n7)
1.5
0.5
−0.5
−1.5
0
24
SKOV3

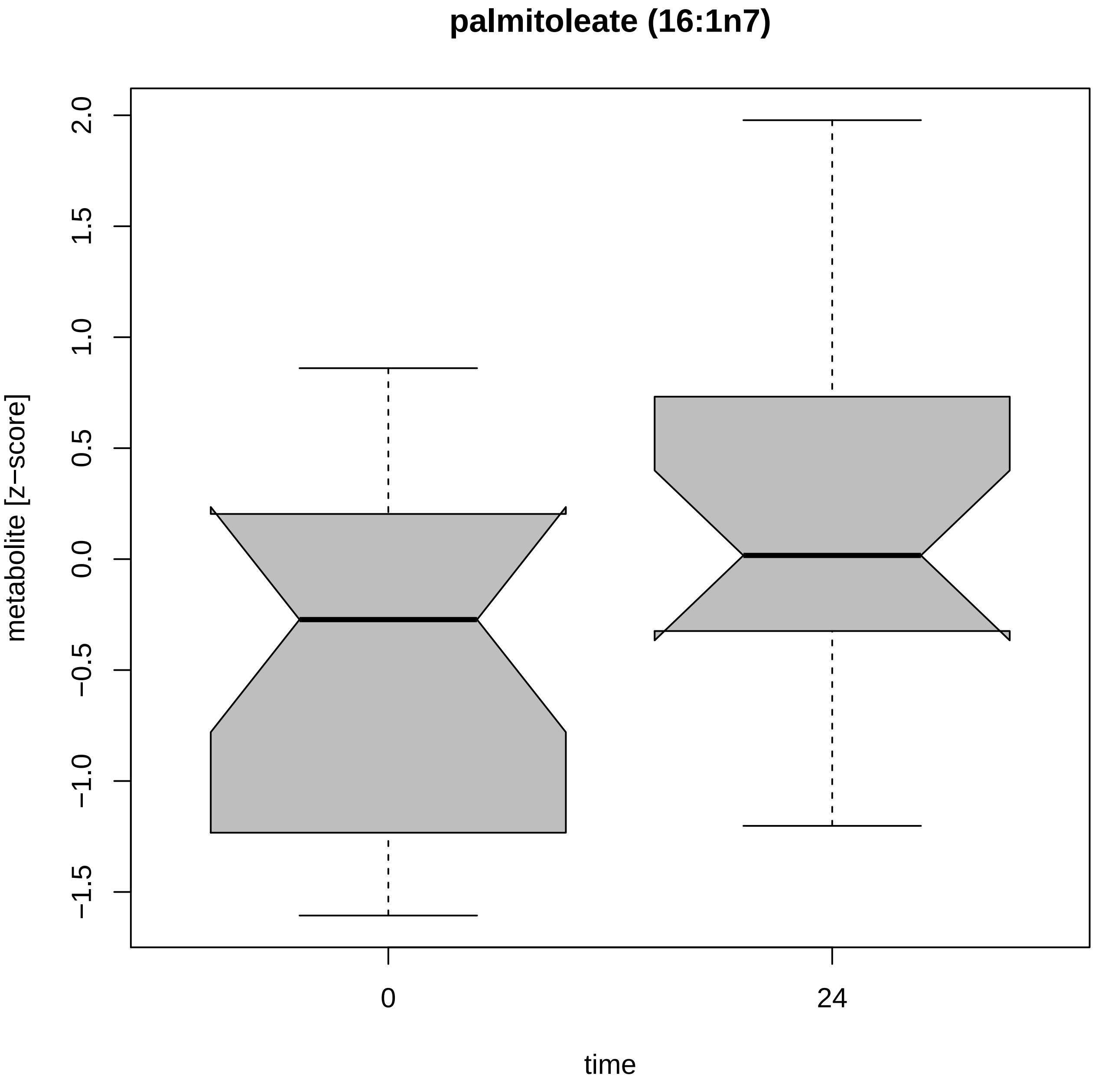
palmitoleate (16:1n7)
metabolite [z−score]
time
0
24
2.0
1.5
1.0
0.5
0.0
−0.5
−1.0
−1.5

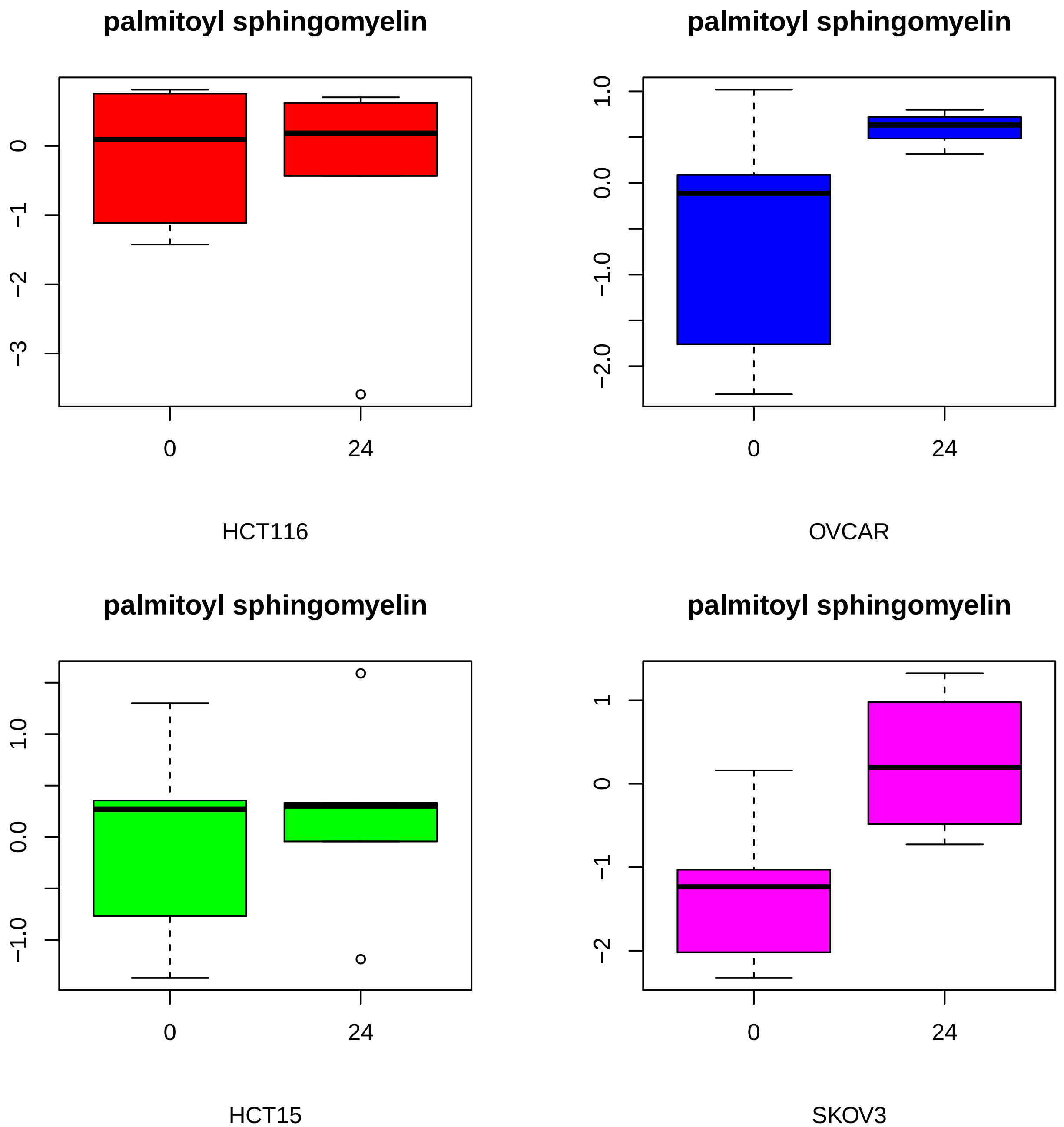
palmitoyl sphingomyelin
0
−1
−2
−3
0
24
HCT116
palmitoyl sphingomyelin
1.0
0.0
−1.0
−2.0
0
24
OVCAR
palmitoyl sphingomyelin
1.0
0.0
−1.0
0
24
HCT15
palmitoyl sphingomyelin
1
0
−1
−2
0
24
SKOV3

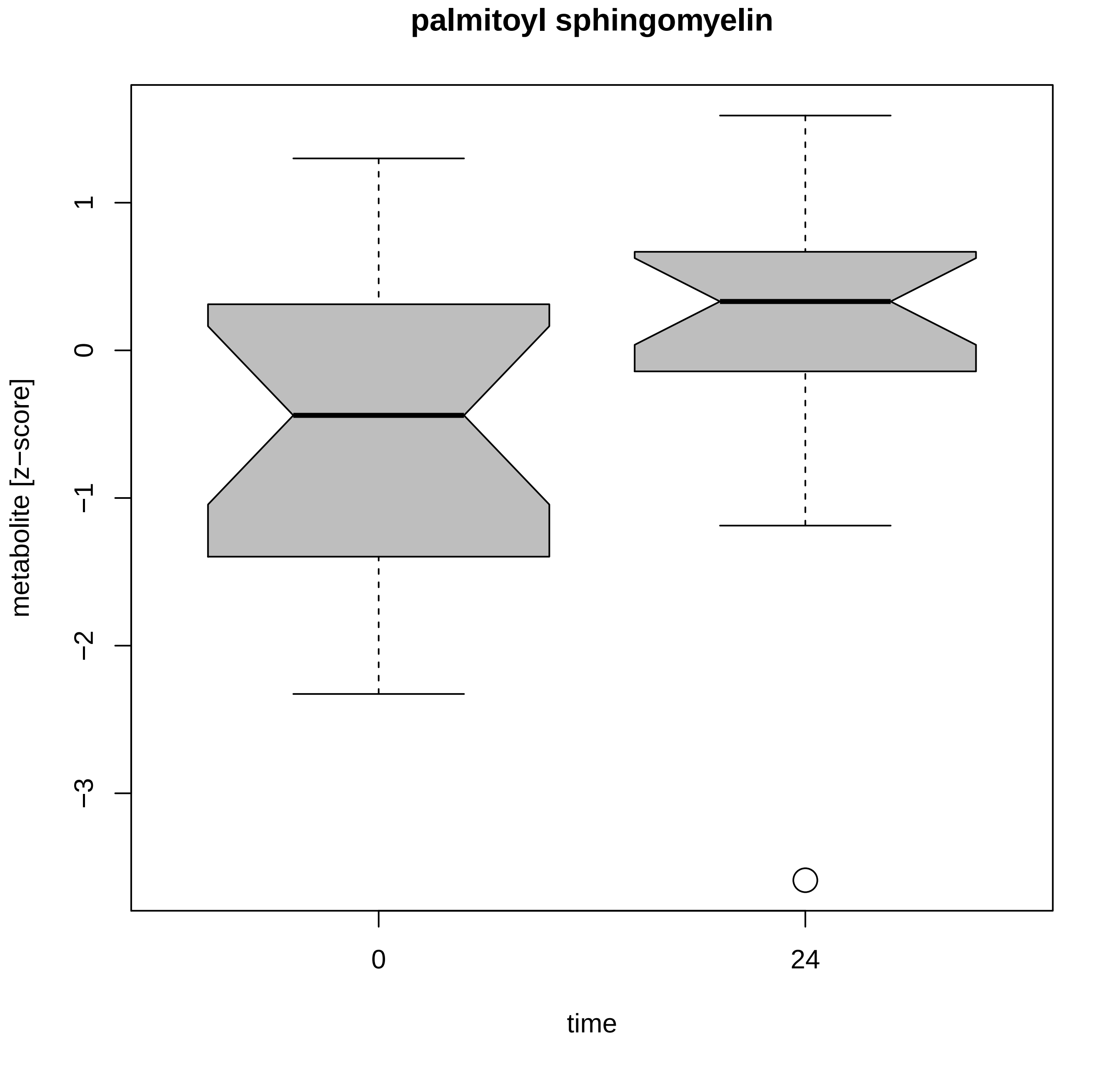

palmitoyl sphingomyelin
1
0
−1
−2
−3
metabolite [z−score]
0
24
time

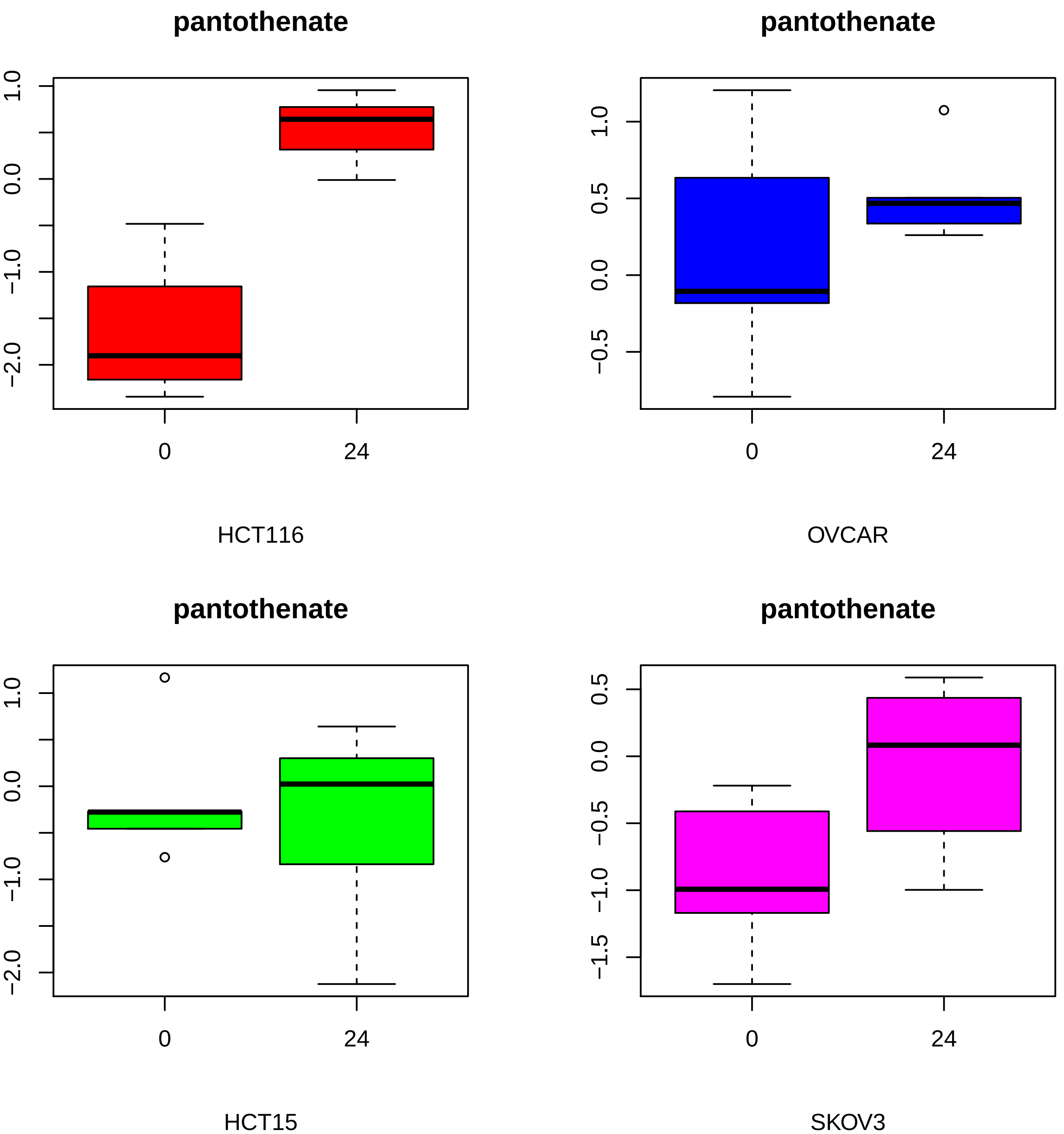
pantothenate
1.0
0.0
−1.0
−2.0
0
24
HCT116
pantothenate
1.0
0.5
0.0
−0.5
0
24
OVCAR
pantothenate
1.0
0.0
−1.0
−2.0
0
24
HCT15
pantothenate
0.5
0.0
−0.5
−1.0
−1.5
0
24
SKOV3

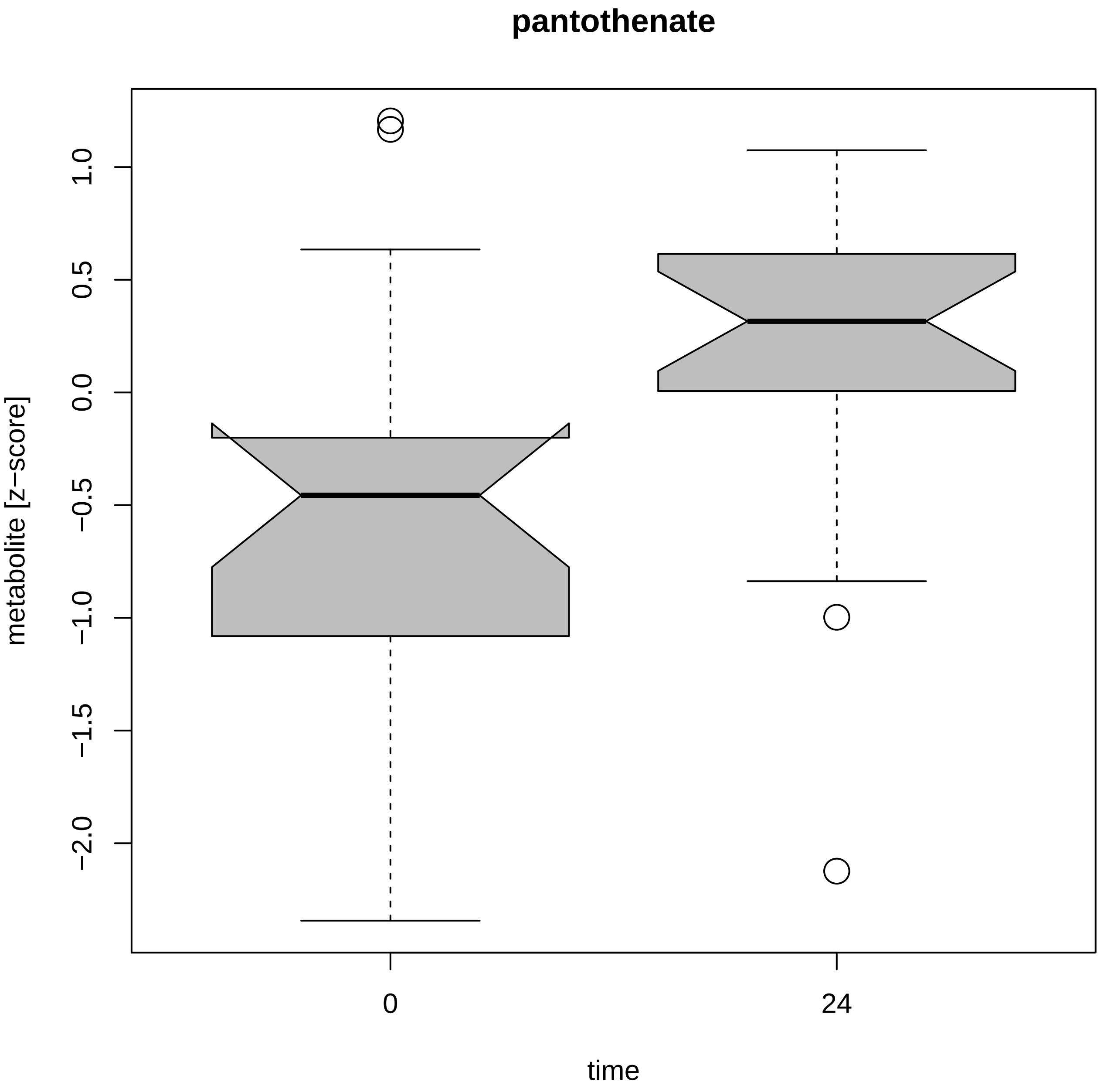
pantothenate
metabolite [z−score]
1.0
0.5
0.0
−0.5
−1.0
−1.5
−2.0
0
24
time

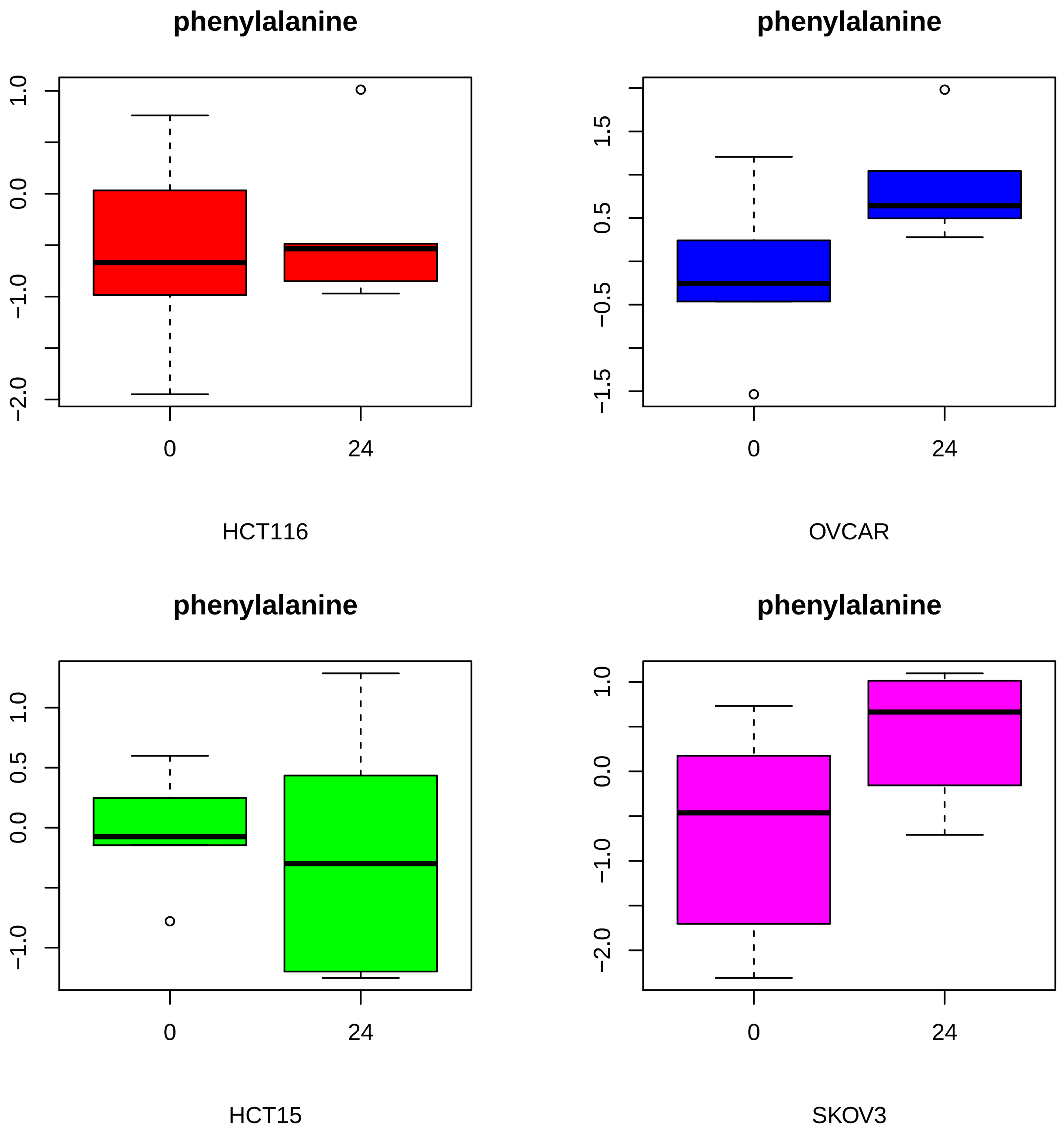
phenylalanine
1.0
0.0
−1.0
−2.0
0
24
HCT116
phenylalanine
1.5
0.5
−0.5
−1.5
0
24
OVCAR
phenylalanine
1.0
0.5
0.0
−1.0
0
24
HCT15
phenylalanine
1.0
0.0
−1.0
−2.0
0
24
SKOV3

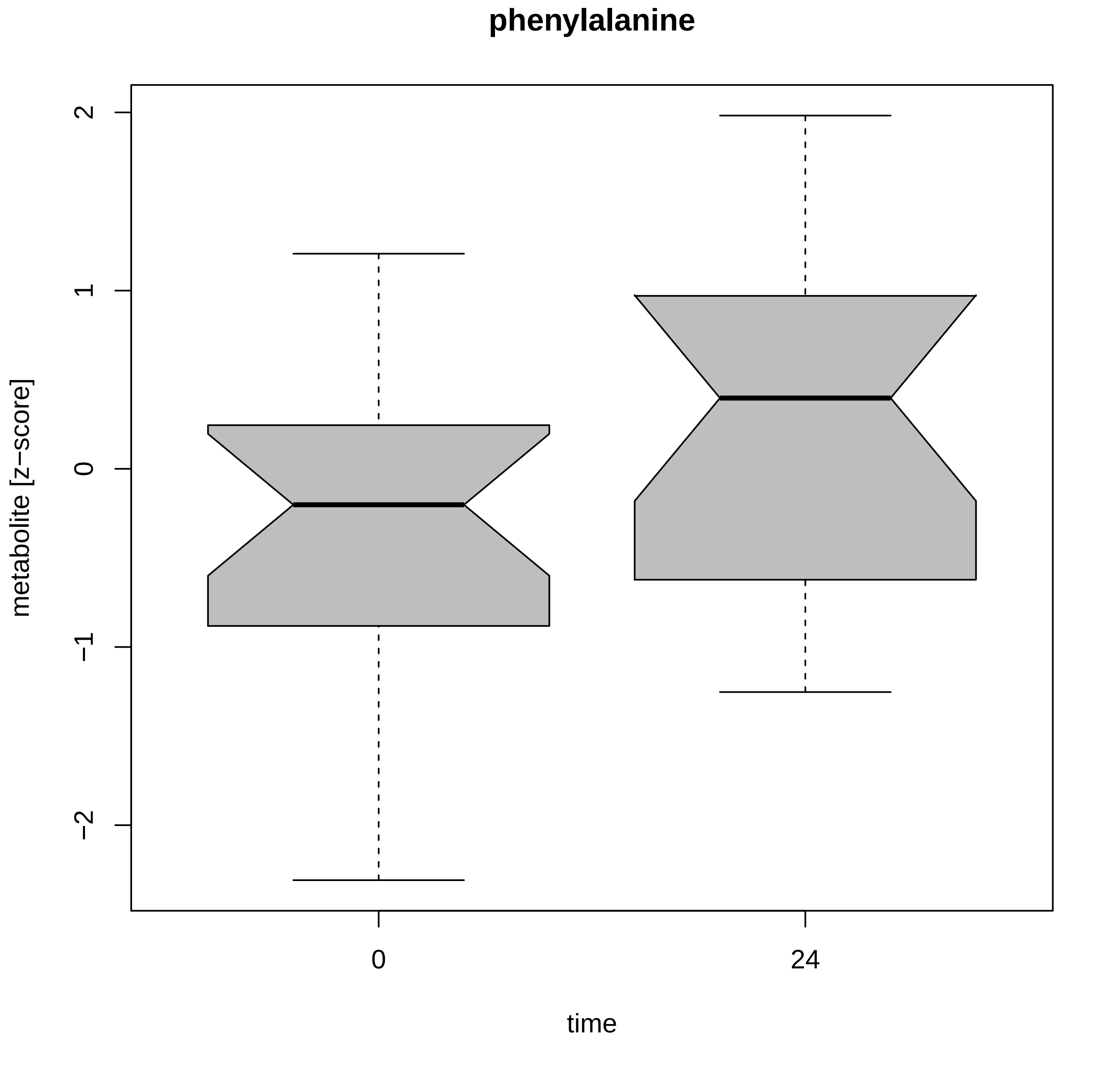
phenylalanine
metabolite [z−score]
2
1
0
−1
−2
0
24
time

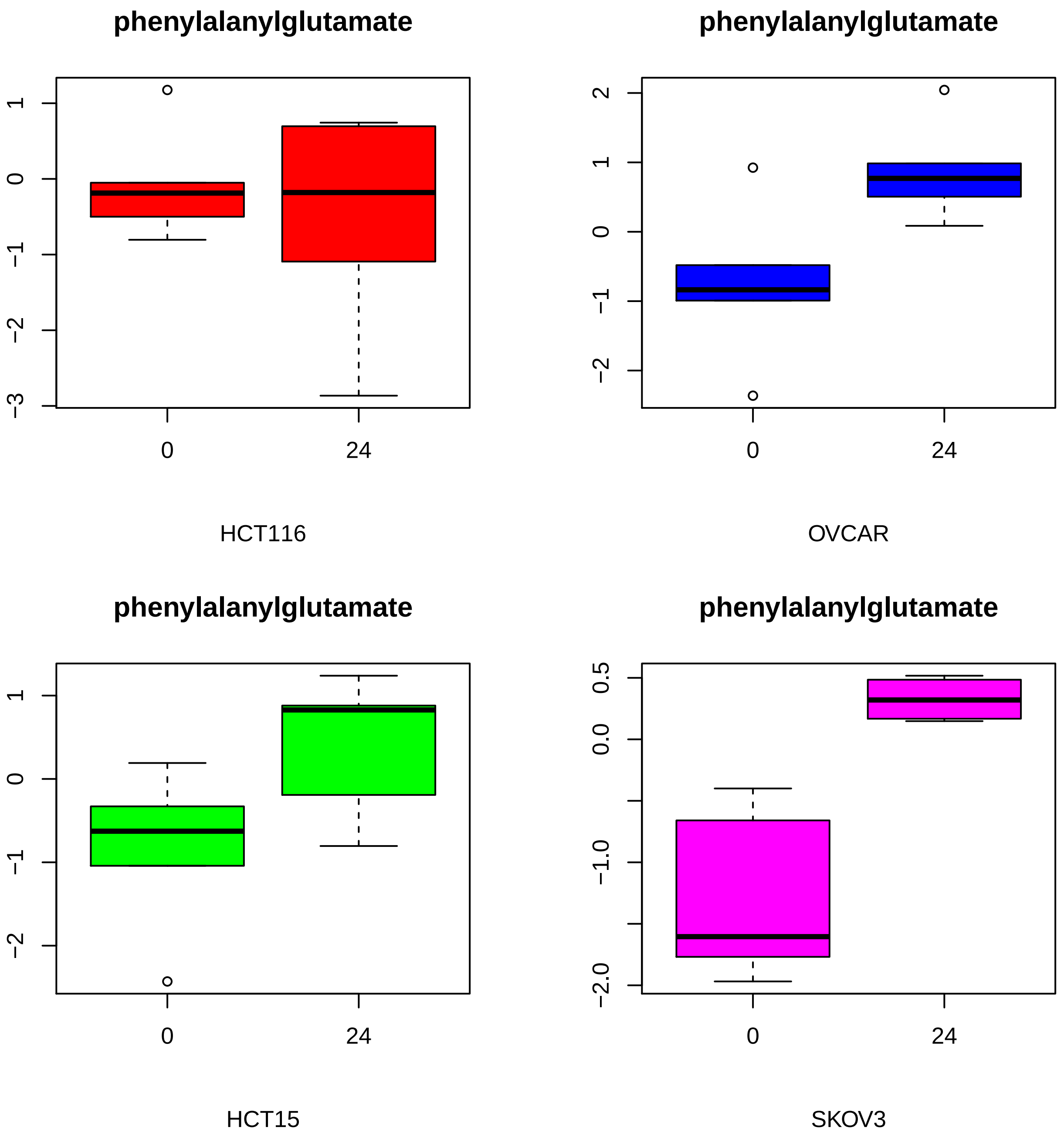
phenylalanylglutamate
1
0
−1
−2
−3
0
24
HCT116
phenylalanylglutamate
2
1
0
−1
−2
0
24
OVCAR
phenylalanylglutamate
1
0
−1
−2
0
24
HCT15
phenylalanylglutamate
0.5
0.0
−1.0
−2.0
0
24
SKOV3

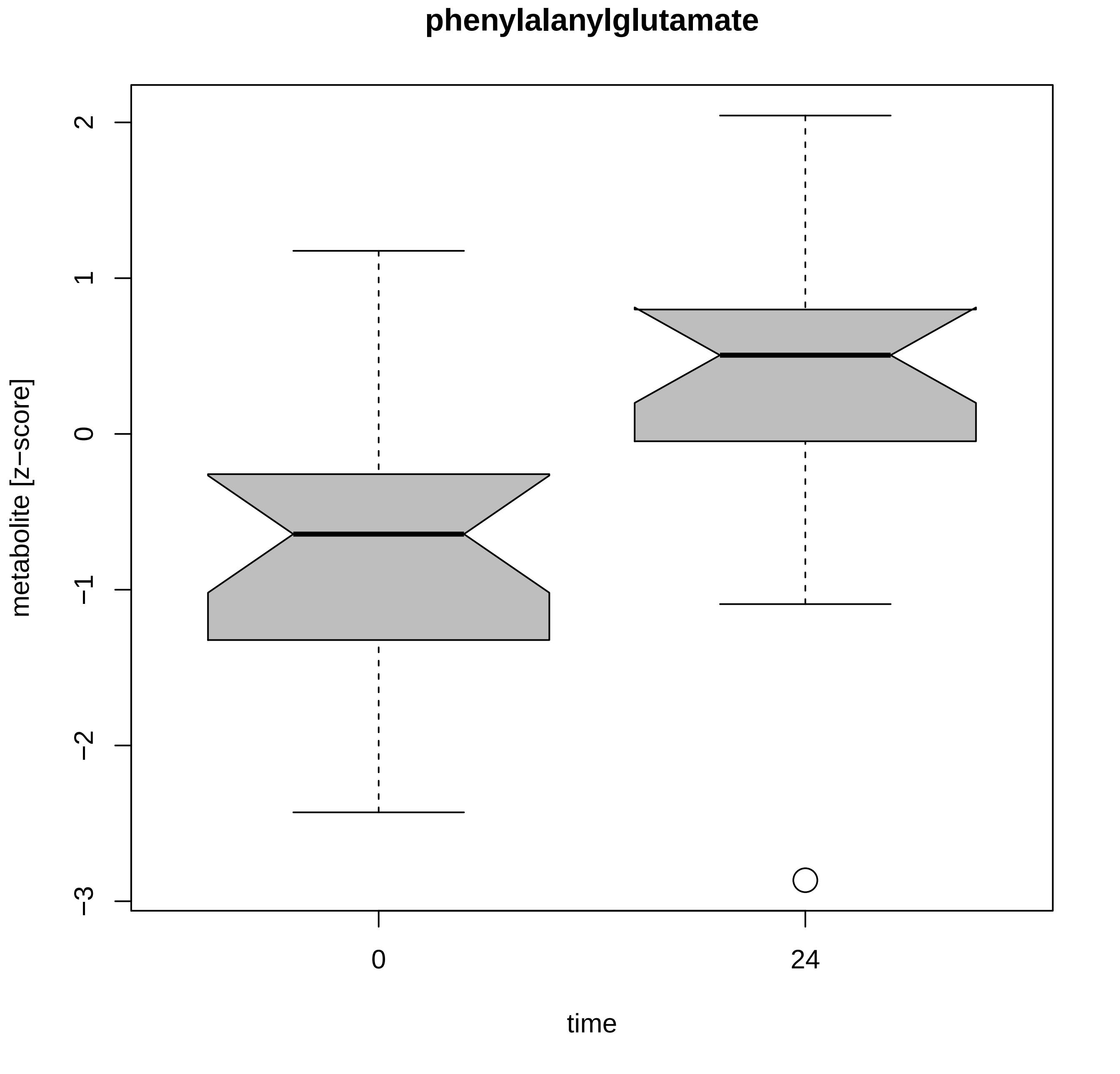

phenylalanylglutamate
metabolite [z−score]
2
1
0
−1
−2
−3
0
24
time

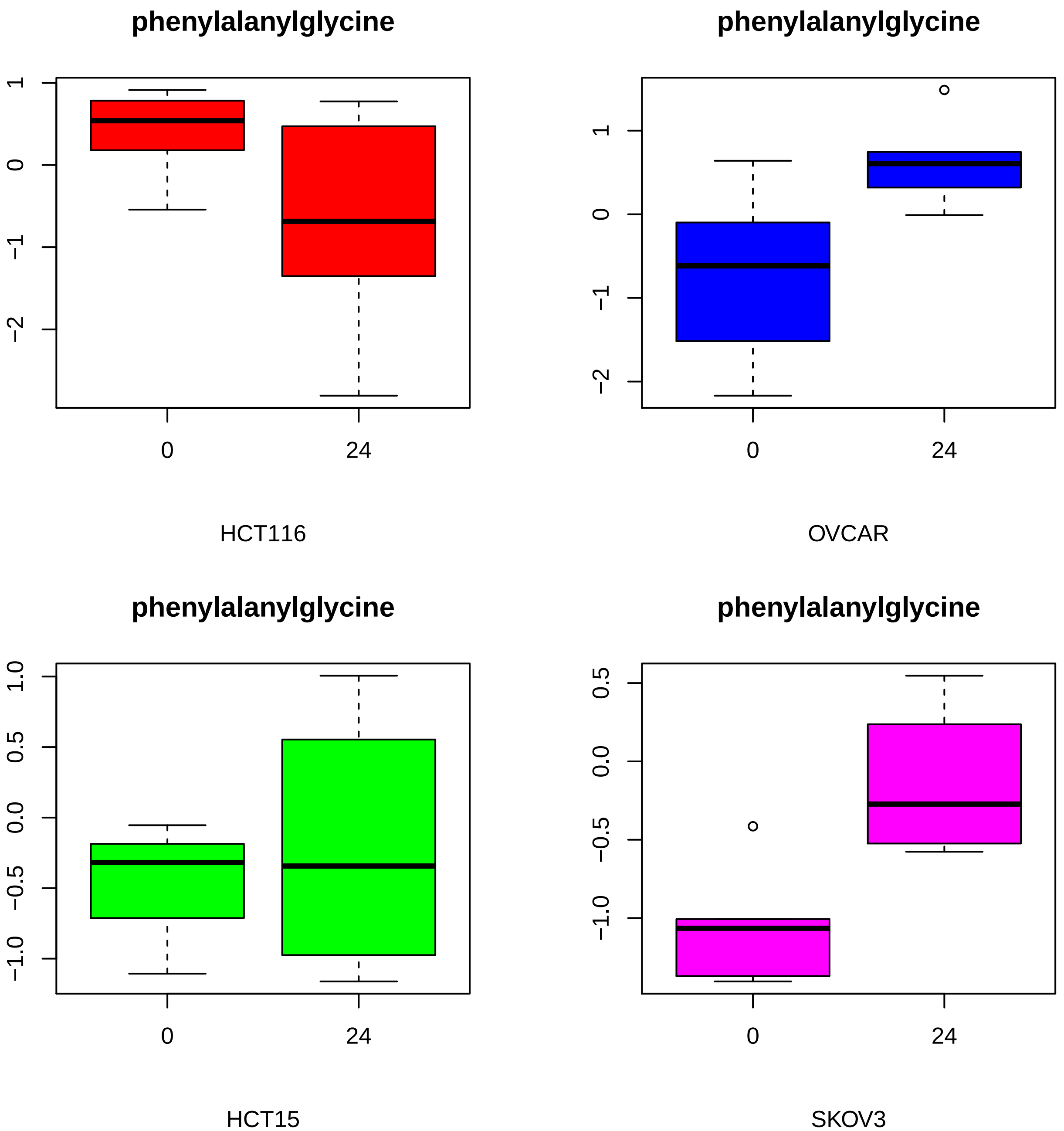

phenylalanylglycine
HCT116
phenylalanylglycine
OVCAR
phenylalanylglycine
HCT15
phenylalanylglycine
SKOV3
0
24

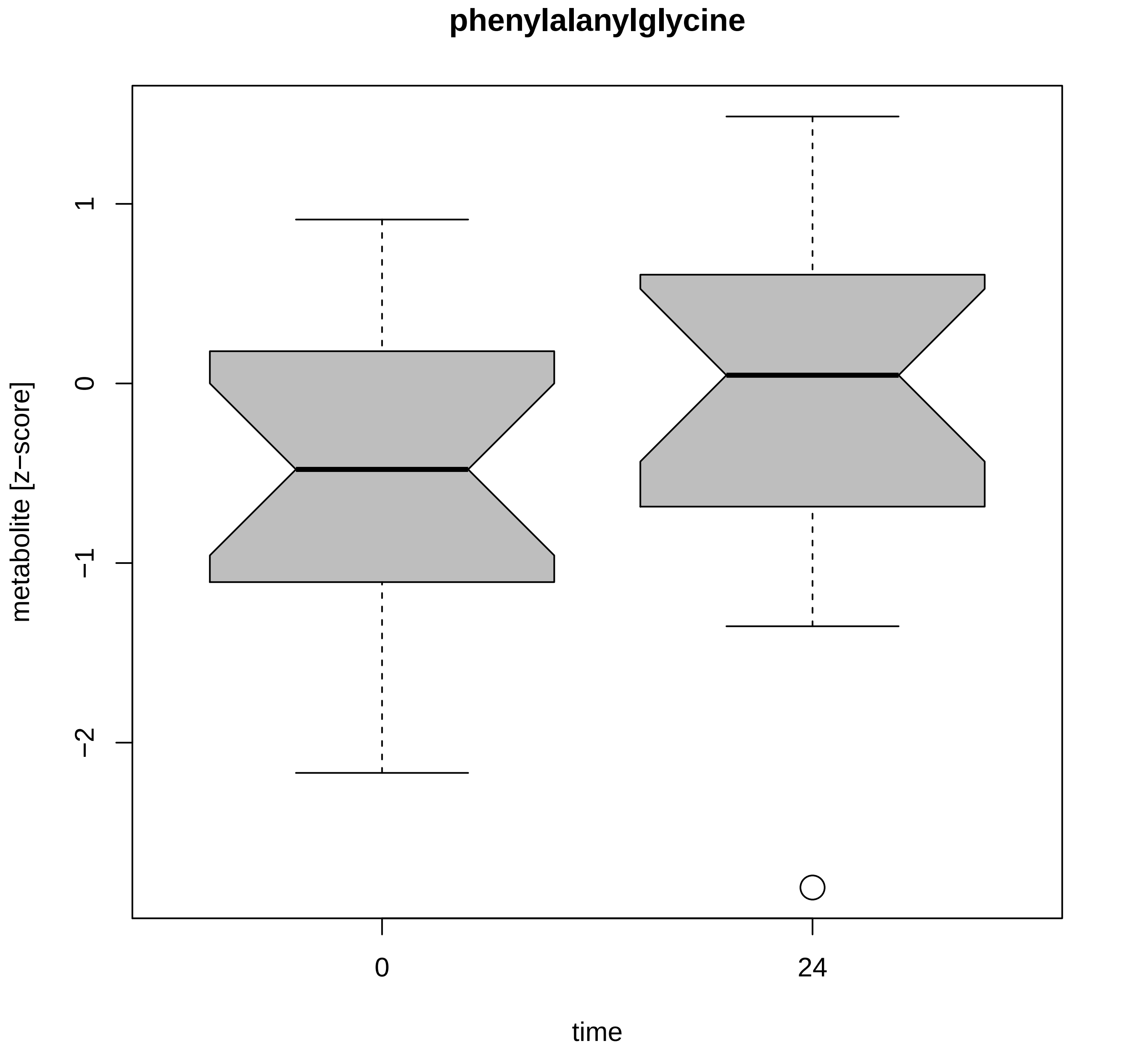

phenylalanylglycine
metabolite [z−score]
1
0
−1
−2
0
24
time

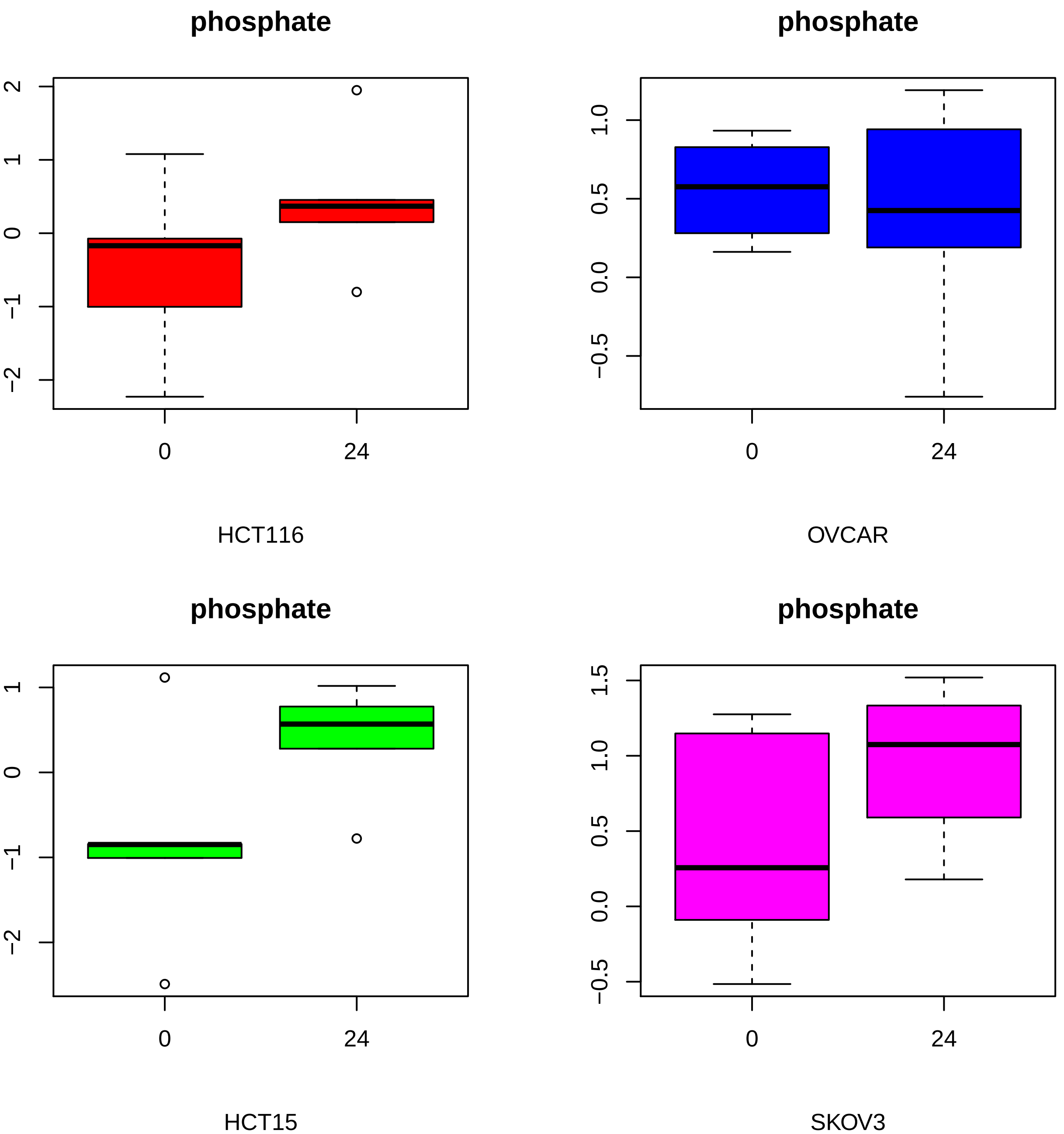
phosphate
2
1
0
−1
−2
0
24
HCT116
phosphate
1.0
0.5
0.0
−0.5
0
24
OVCAR
phosphate
1
0
−1
−2
0
24
HCT15
phosphate
1.5
1.0
0.5
0.0
−0.5
0
24
SKOV3

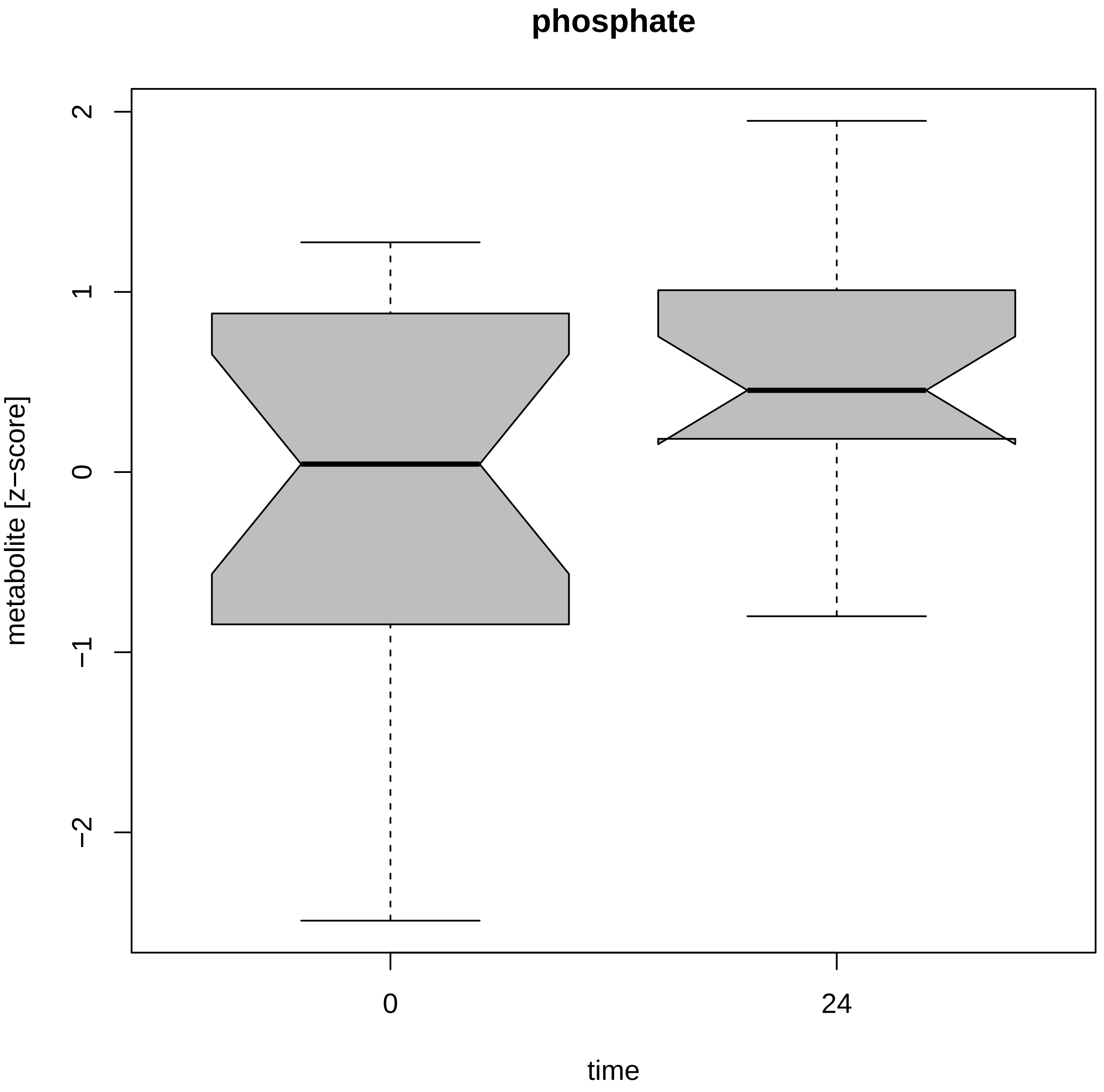

phosphate
metabolite [z−score]
2
1
0
−1
−2
0
24
time

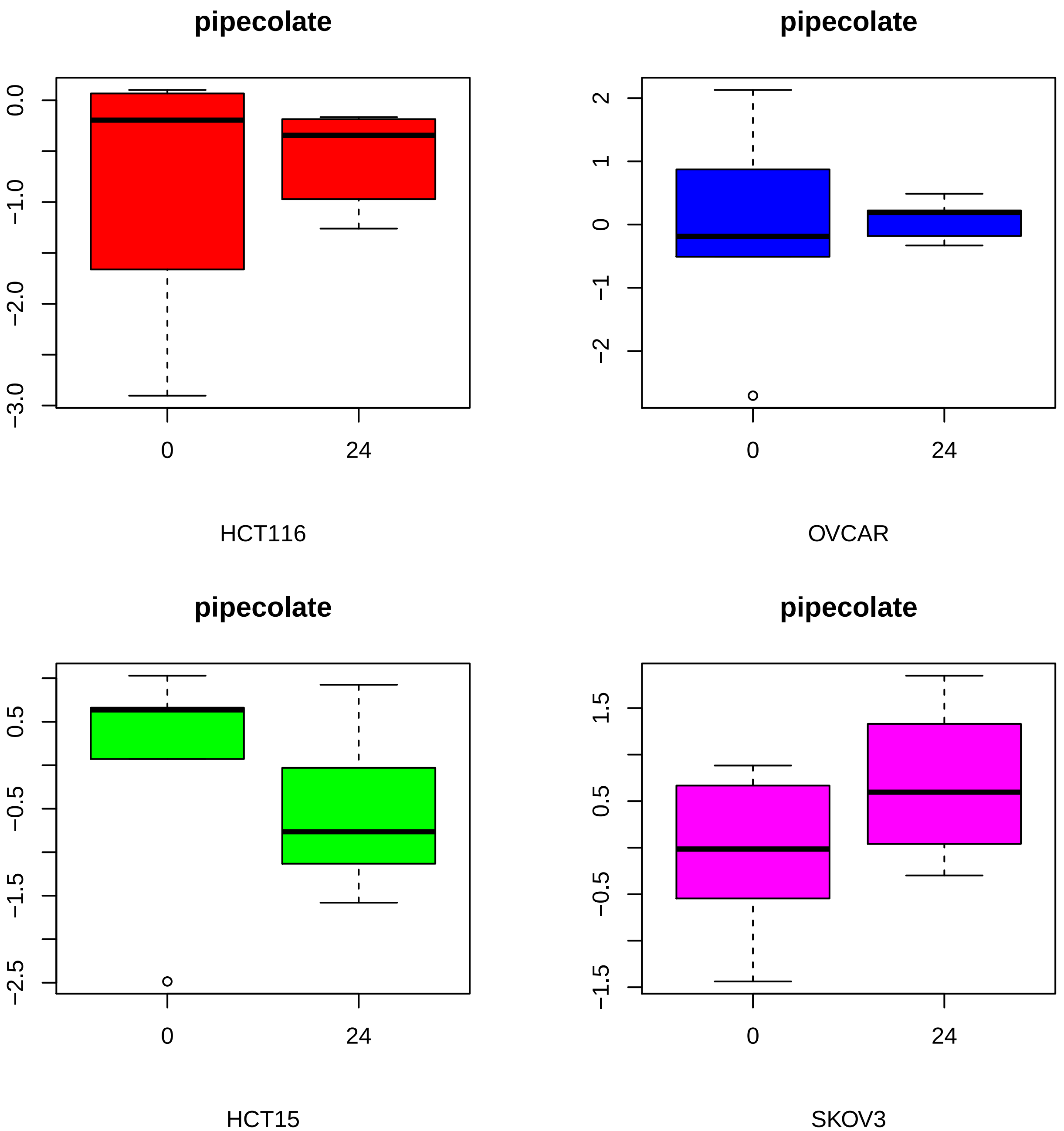
pipecolate
0.0
−1.0
−2.0
−3.0
0
24
HCT116
pipecolate
2
1
0
−1
−2
0
24
OVCAR
pipecolate
0.5
−0.5
−1.5
−2.5
0
24
HCT15
pipecolate
1.5
0.5
−0.5
−1.5
0
24
SKOV3

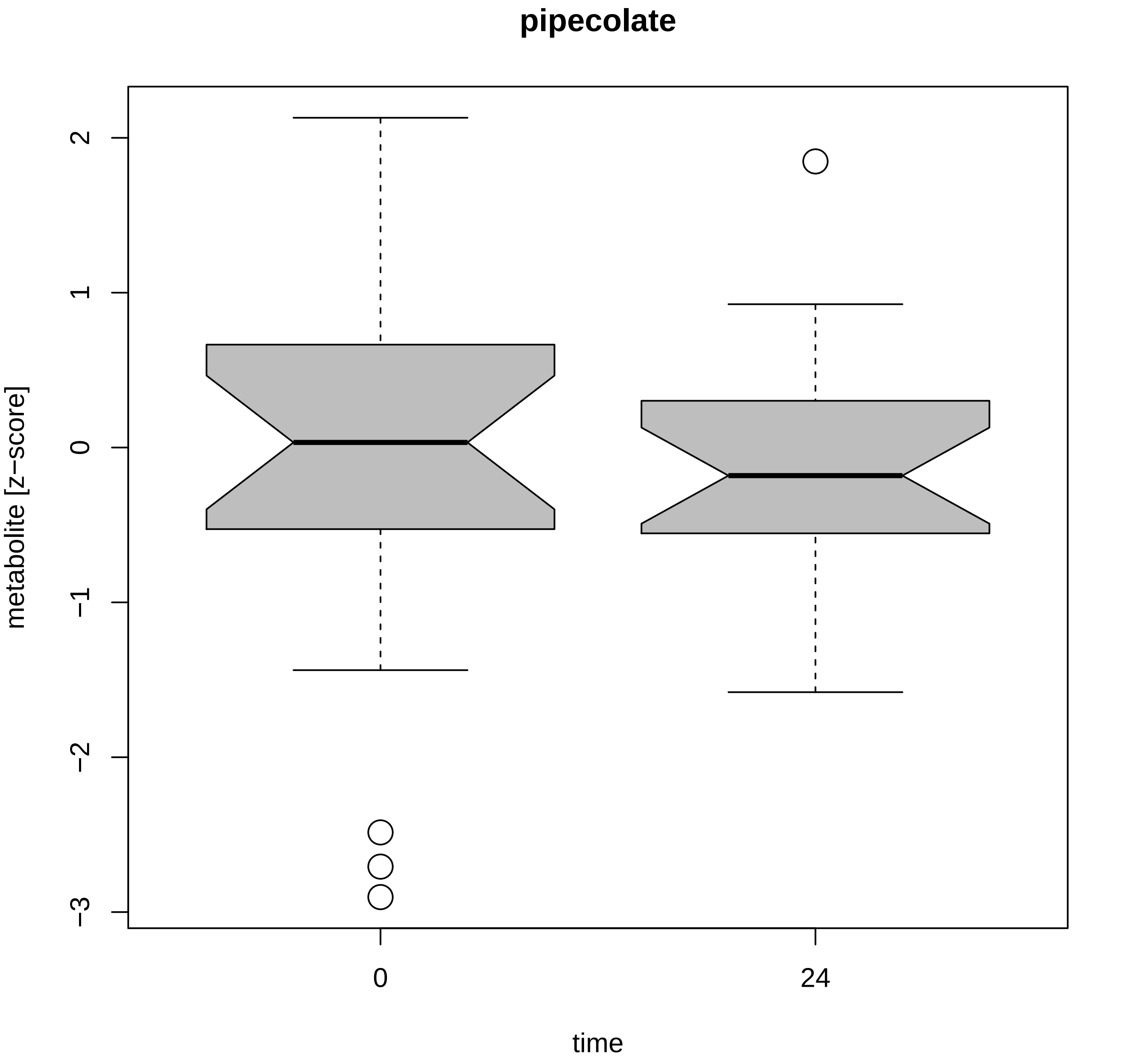
pipecolate
metabolite [z−score]
2
1
0
−1
−2
−3
0
24
time

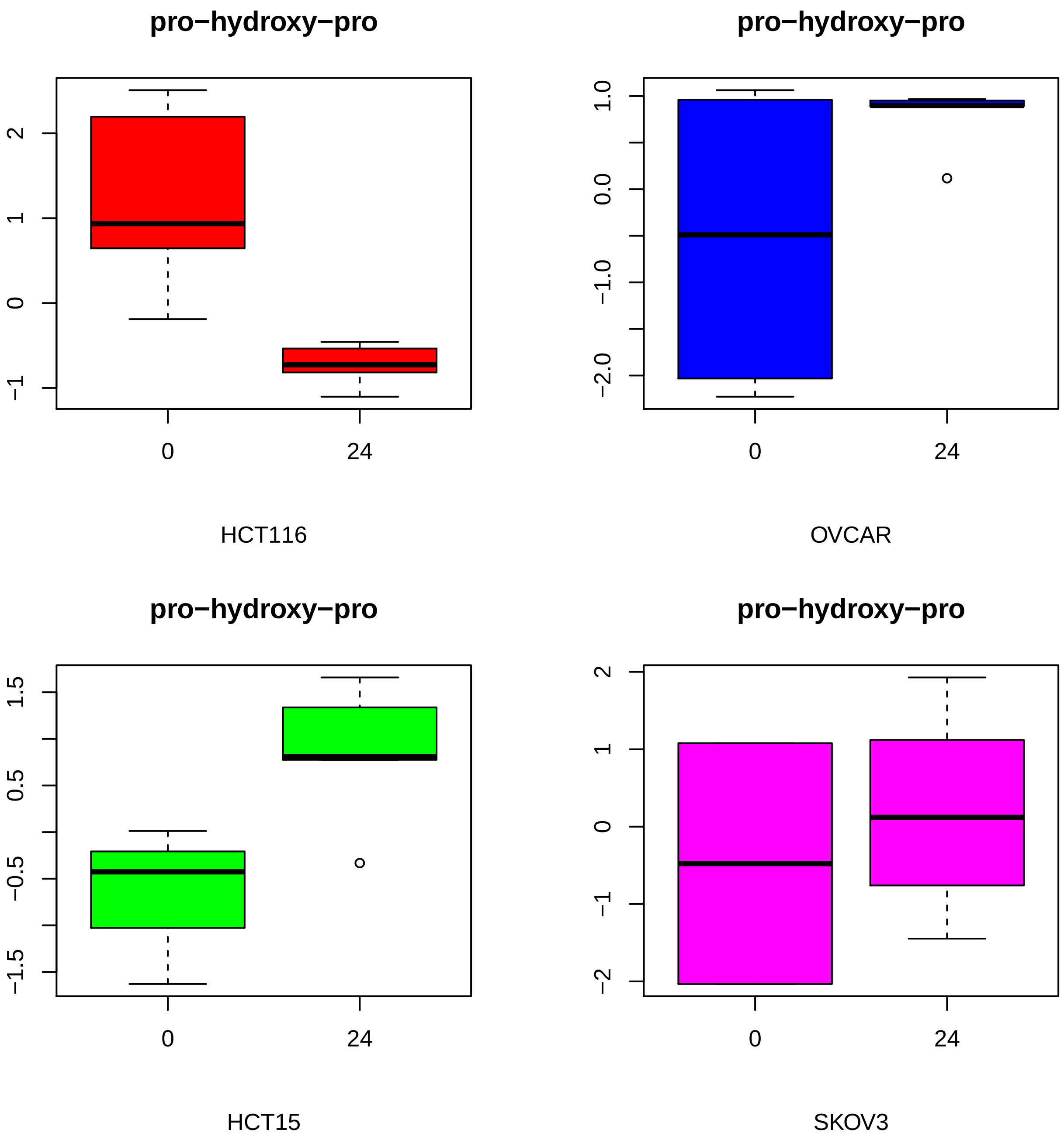
pro−hydroxy−pro
2
1
0
−1
0
24
HCT116
pro−hydroxy−pro
1.0
0.0
−1.0
−2.0
0
24
OVCAR
pro−hydroxy−pro
1.5
0.5
−0.5
−1.5
0
24
HCT15
pro−hydroxy−pro
2
1
0
−1
−2
0
24
SKOV3

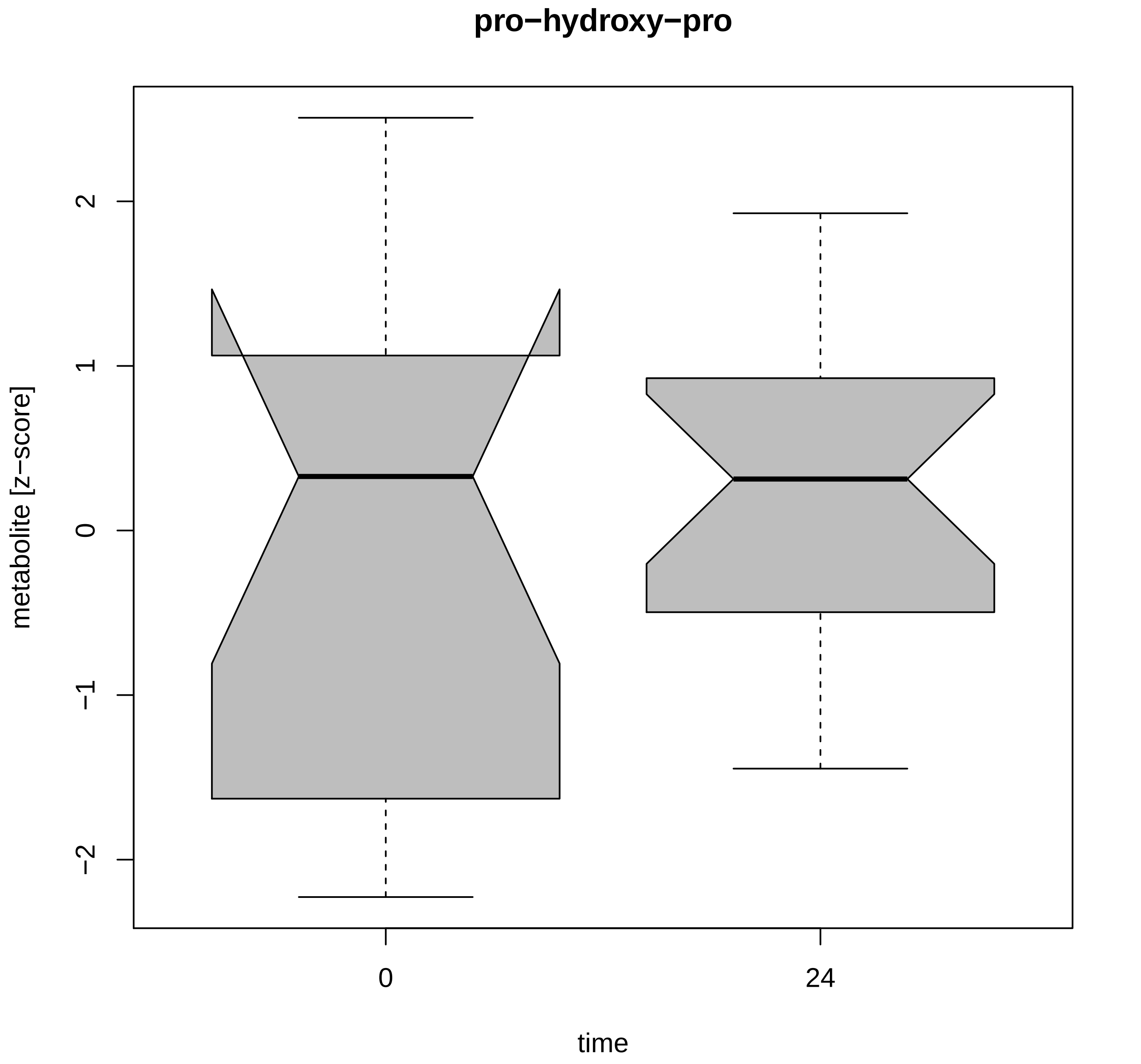
pro−hydroxy−pro
metabolite [z−score]
2
1
0
−1
−2
0
24
time

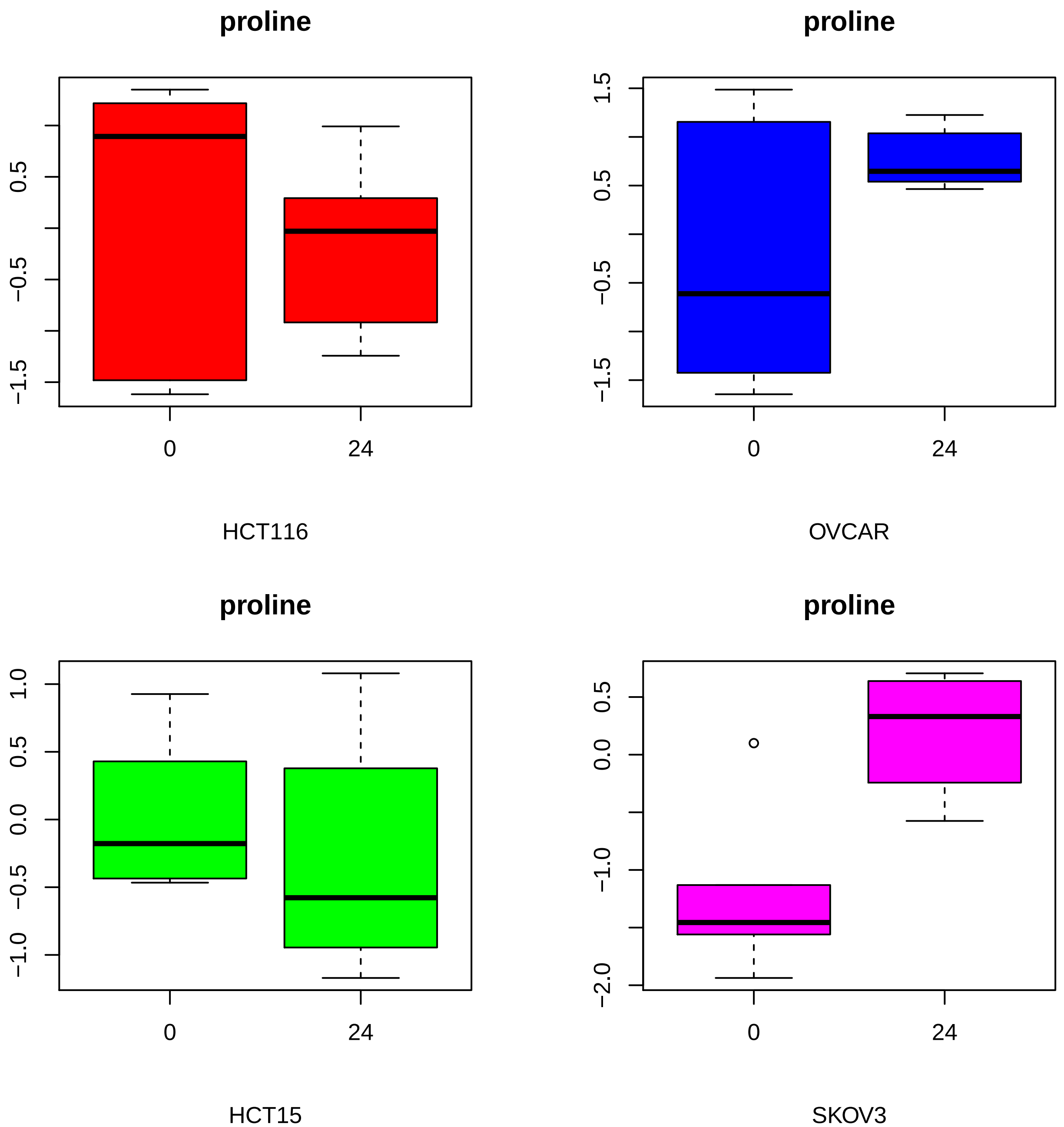
proline
0.5
−0.5
−1.5
0
24
HCT116
proline
1.5
0.5
−0.5
−1.5
0
24
OVCAR
proline
1.0
0.5
0.0
−0.5
−1.0
0
24
HCT15
proline
0.5
0.0
−1.0
−2.0
0
24
SKOV3

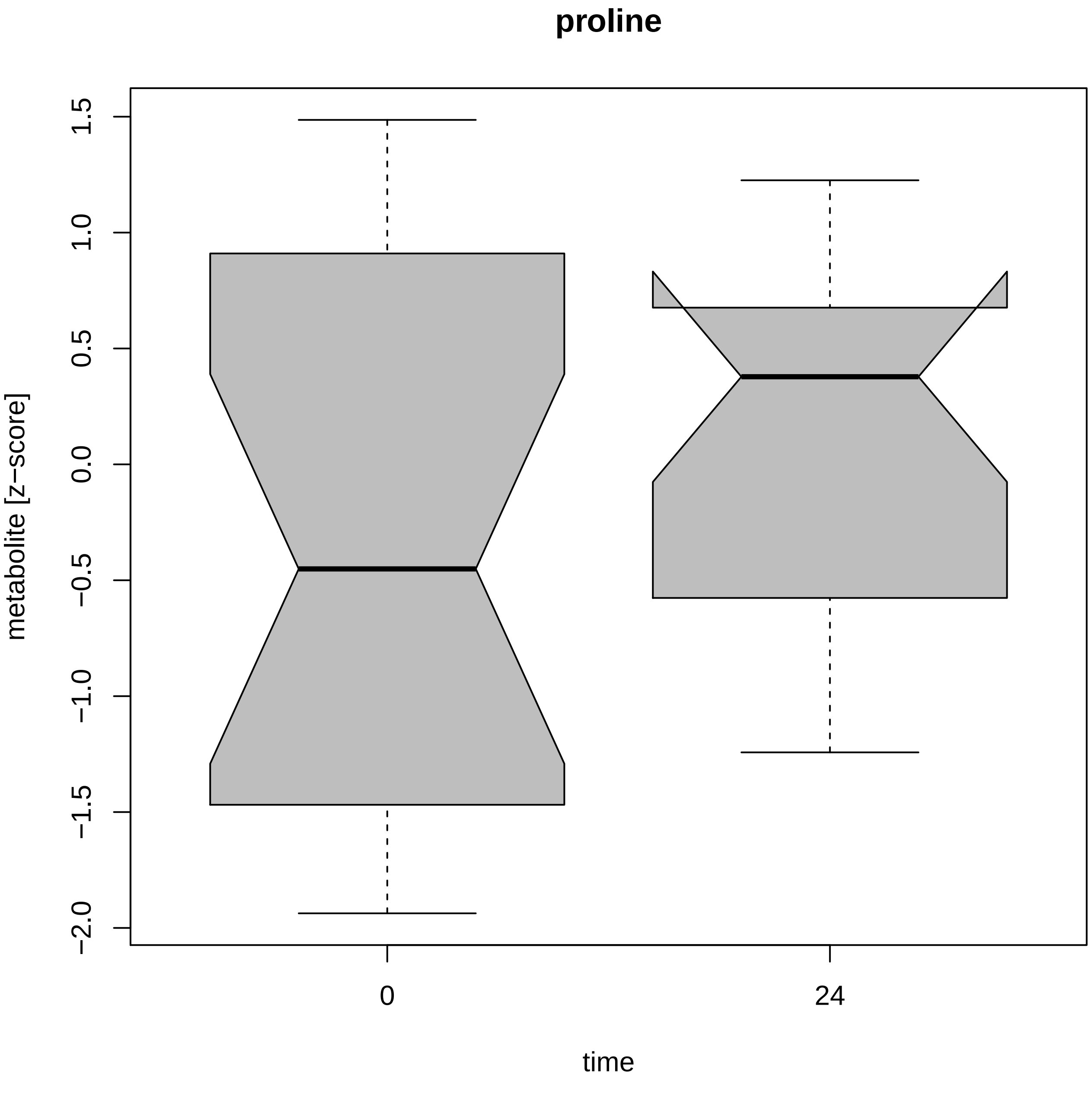

proline
1.5
1.0
0.5
0.0
−0.5
−1.0
−1.5
−2.0
metabolite [z−score]
0
24
time

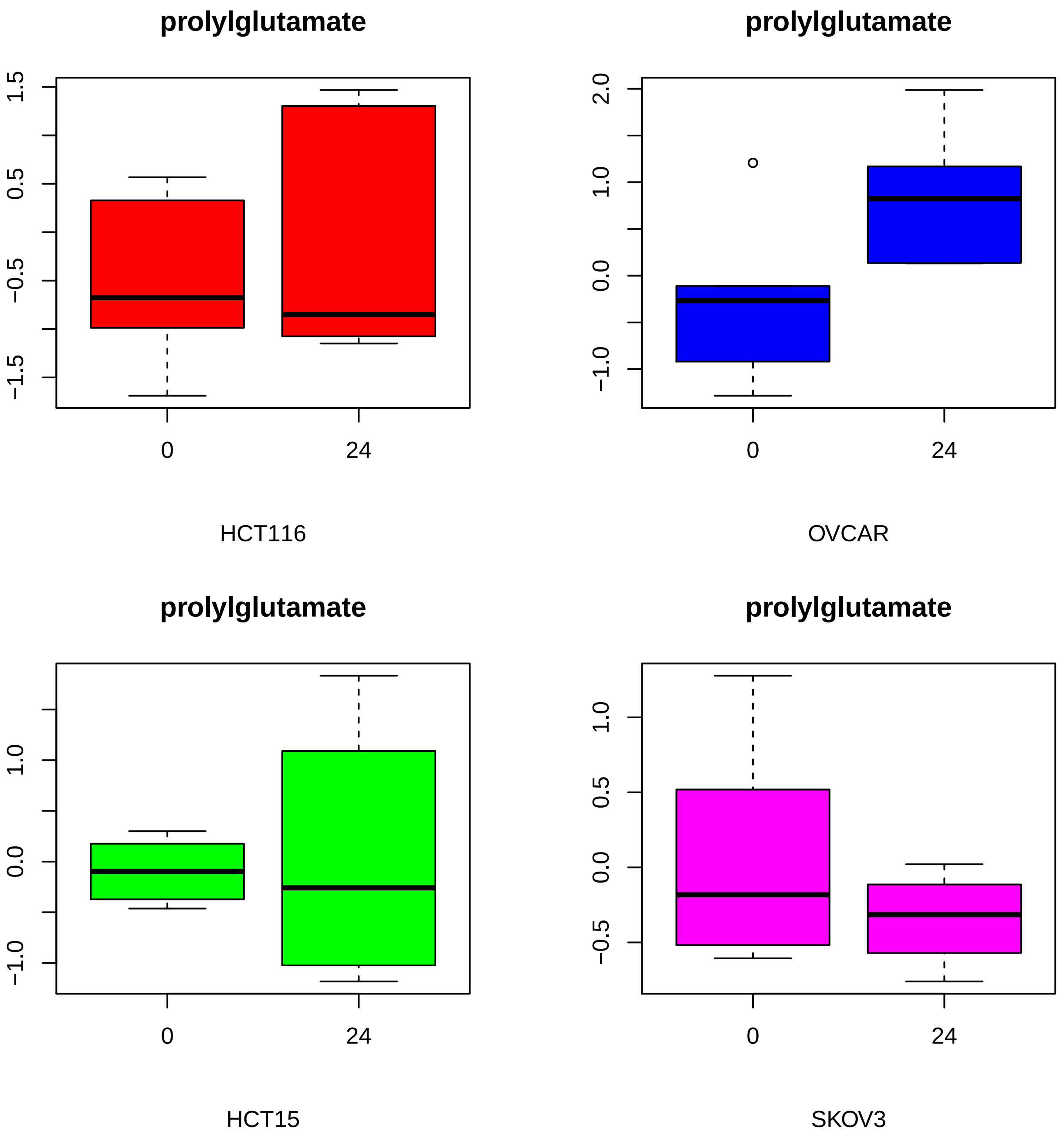
prolylglutamate
1.5
0.5
−0.5
−1.5
0
24
HCT116
prolylglutamate
2.0
1.0
0.0
−1.0
0
24
OVCAR
prolylglutamate
1.0
0.0
−1.0
0
24
HCT15
prolylglutamate
1.0
0.5
0.0
−0.5
0
24
SKOV3

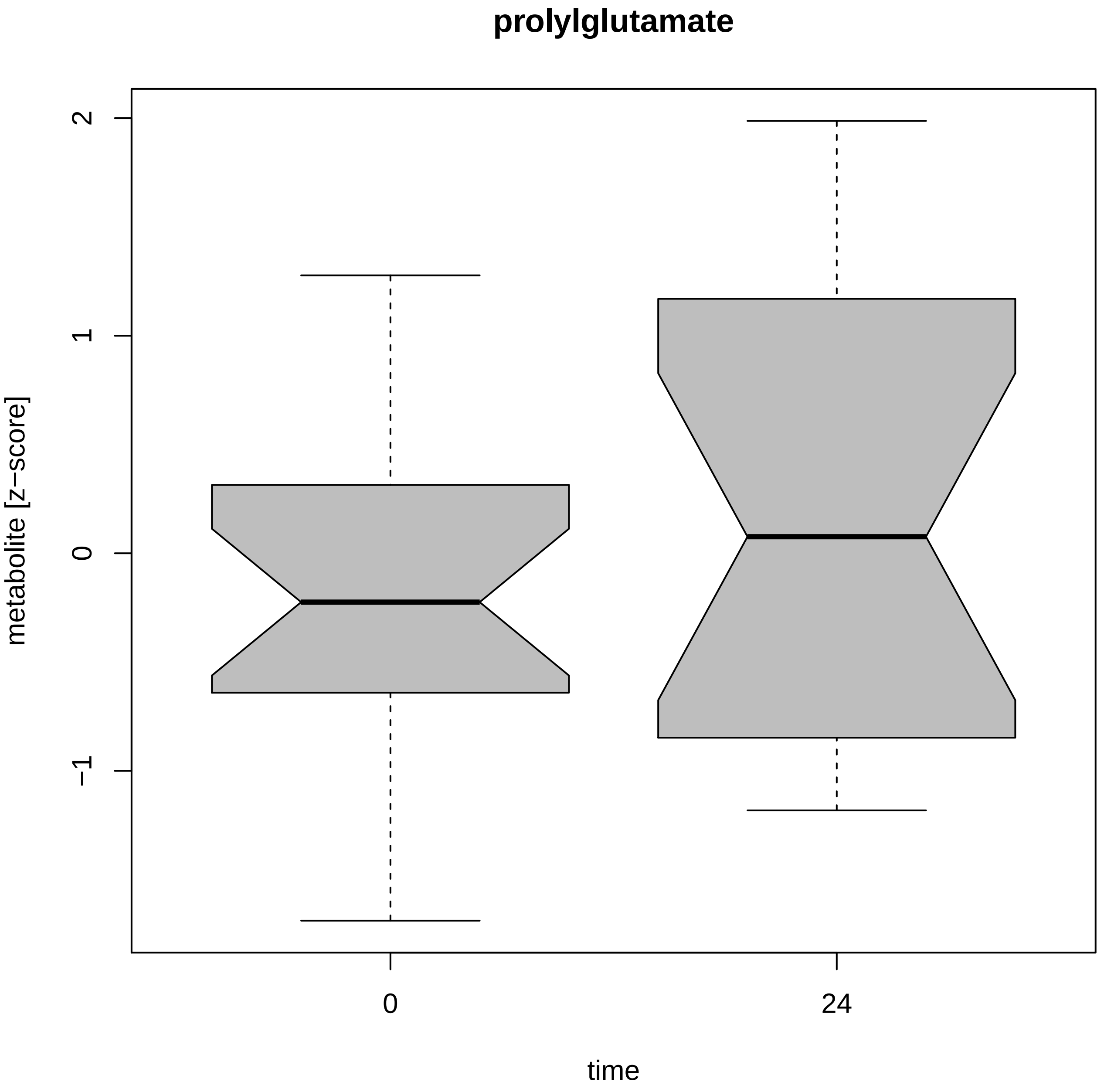
prolylglutamate
metabolite [z−score]
2
1
0
−1
0
24
time

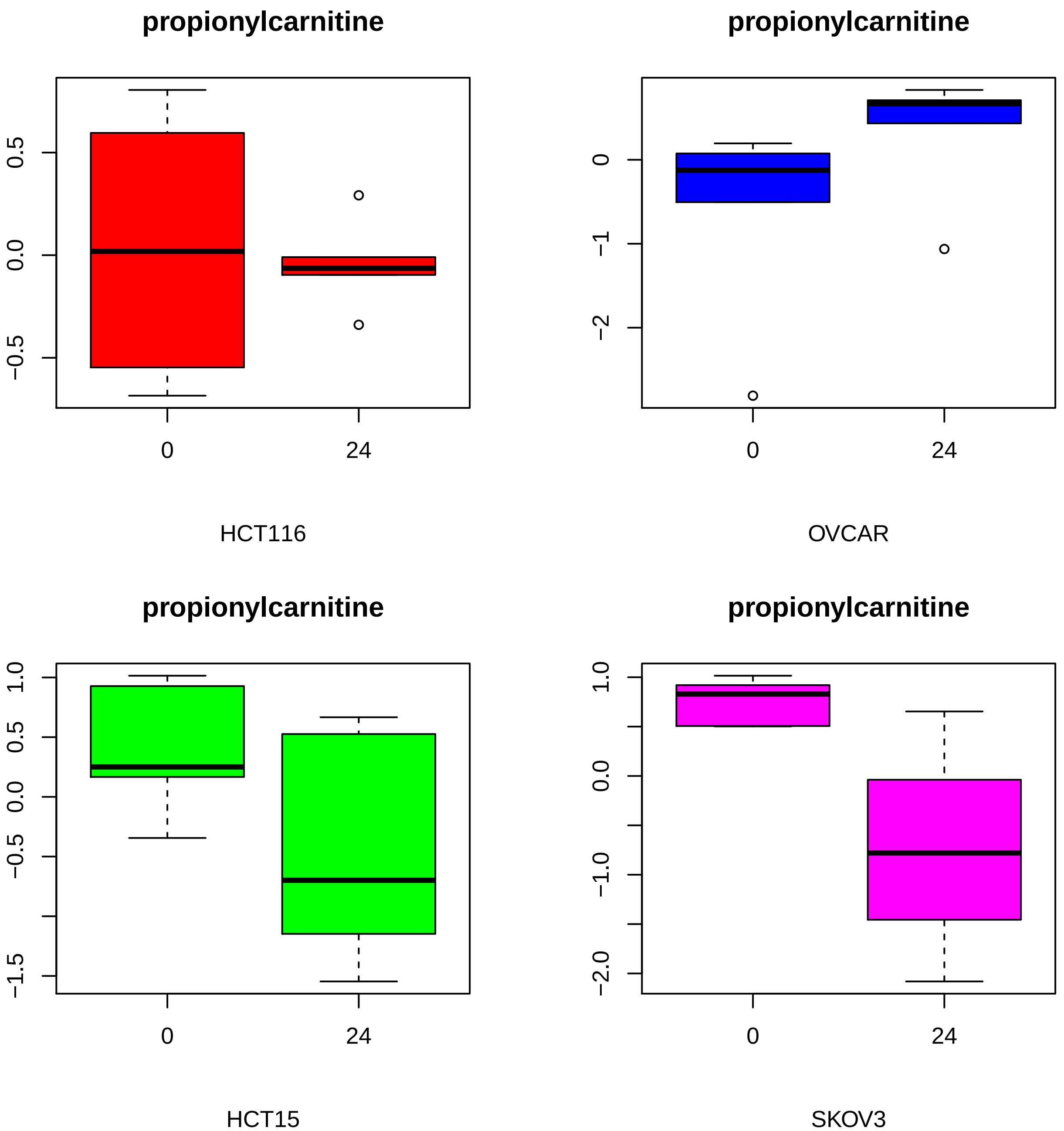
propionylcarnitine
0.5
0.0
−0.5
0
24
HCT116
propionylcarnitine
0
−1
−2
0
24
OVCAR
propionylcarnitine
1.0
0.5
0.0
−0.5
−1.5
0
24
HCT15
propionylcarnitine
1.0
0.0
−1.0
−2.0
0
24
SKOV3

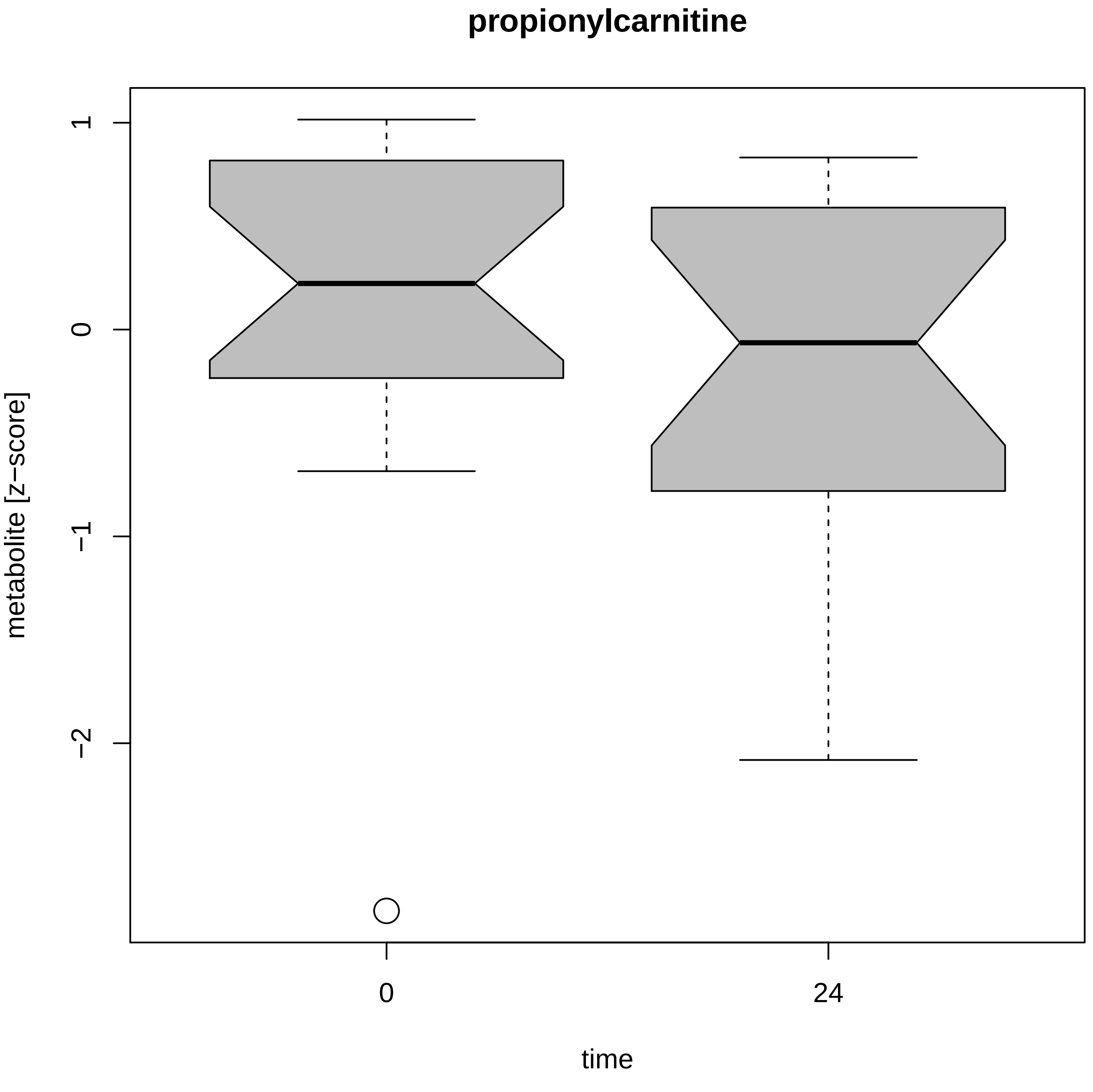
propionylcarnitine
metabolite [z−score]
1
0
−1
−2
0
24
time

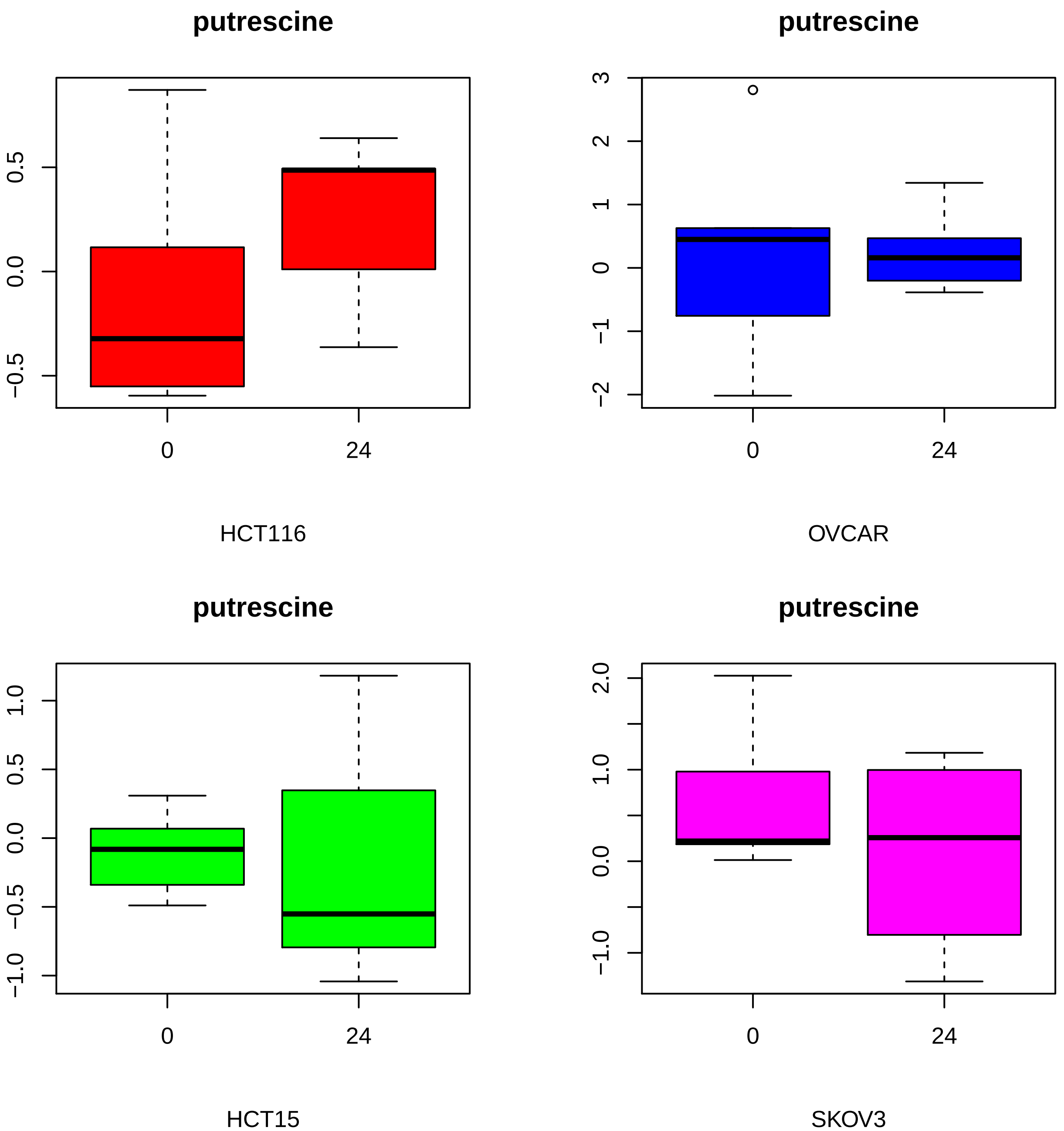
putrescine
0.5
0.0
−0.5
0
24
HCT116
putrescine
3
2
1
0
−1
−2
0
24
OVCAR
putrescine
1.0
0.5
0.0
−0.5
−1.0
0
24
HCT15
putrescine
2.0
1.0
0.0
−1.0
0
24
SKOV3

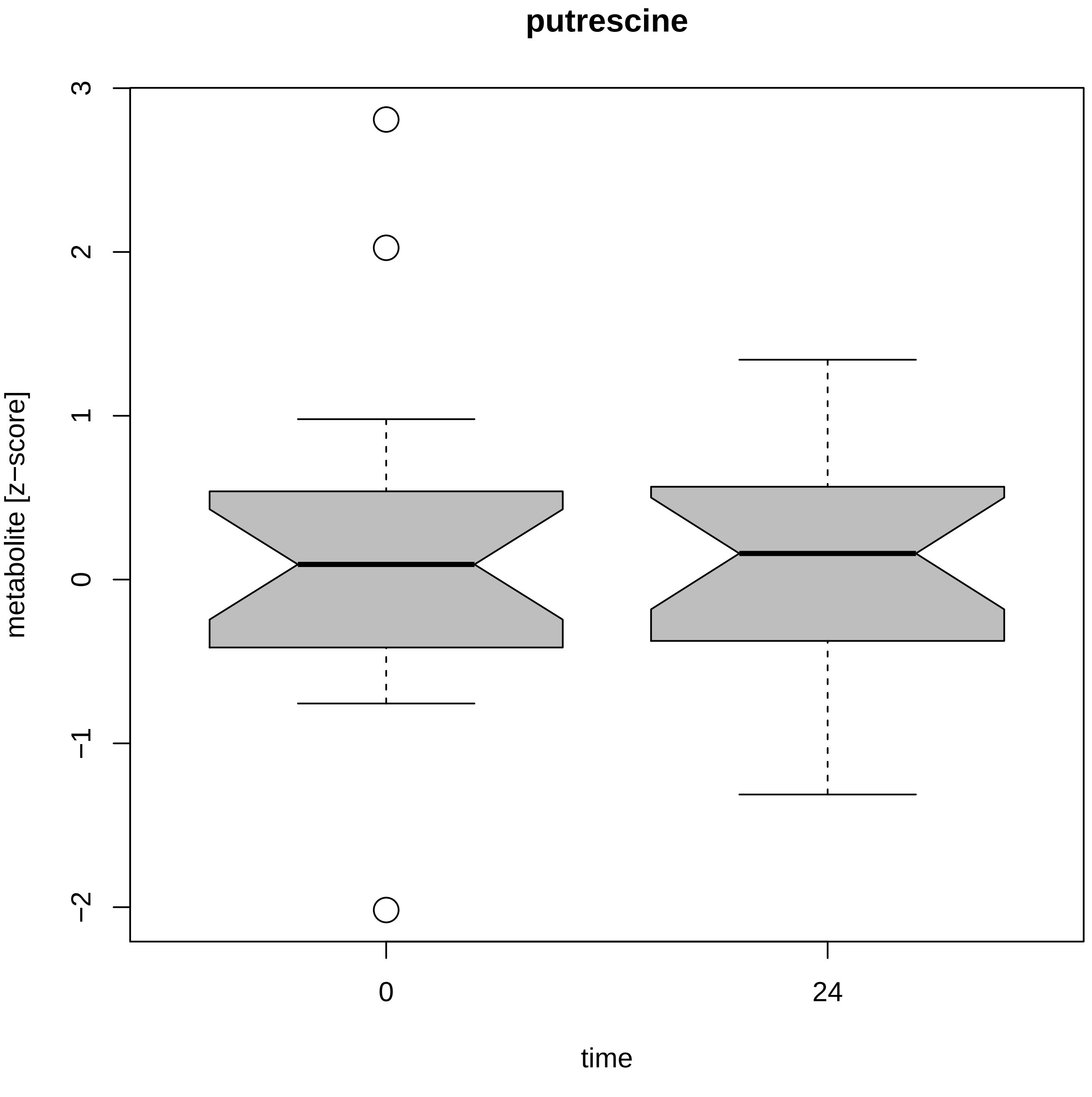
putrescine
metabolite [z−score]
3
2
1
0
−1
−2
0
24
time

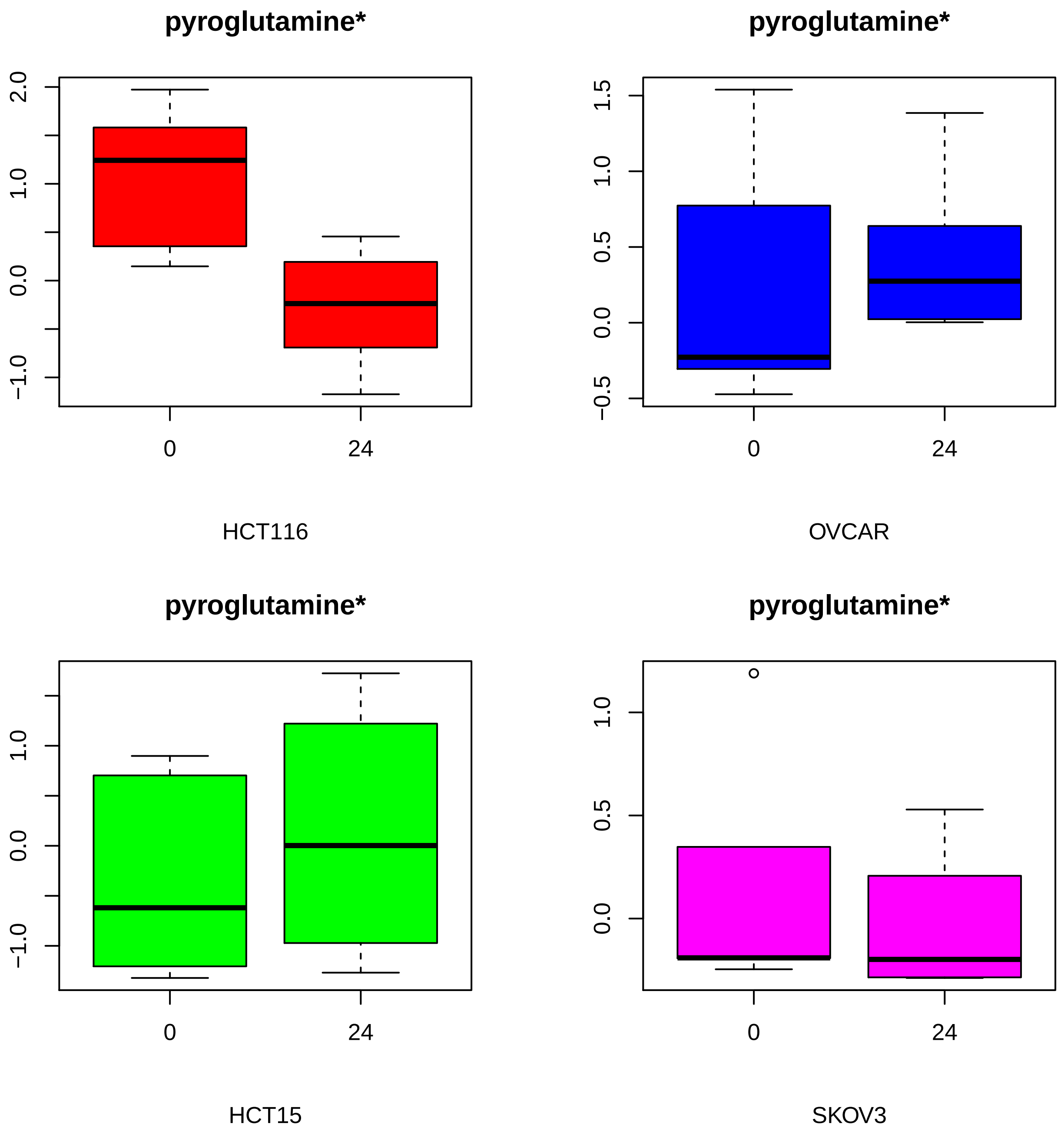
pyroglutamine*
2.0
1.0
0.0
−1.0
0
24
HCT116
pyroglutamine*
1.5
1.0
0.5
0.0
−0.5
0
24
OVCAR
pyroglutamine*
1.0
0.0
−1.0
0
24
HCT15
pyroglutamine*
1.0
0.5
0.0
0
24
SKOV3

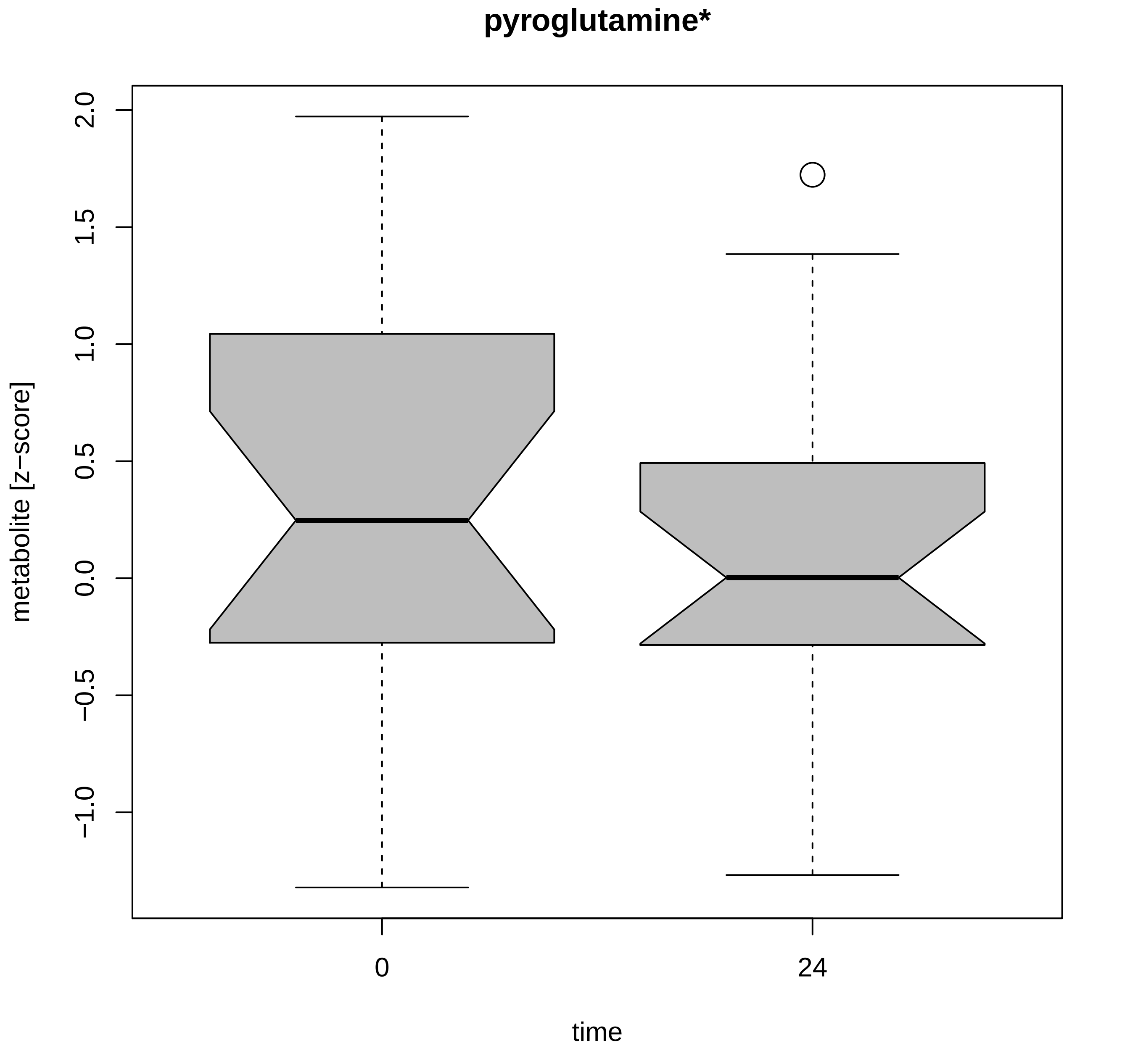
pyroglutamine*
metabolite [z−score]
2.0
1.5
1.0
0.5
0.0
−0.5
−1.0
0
24
time

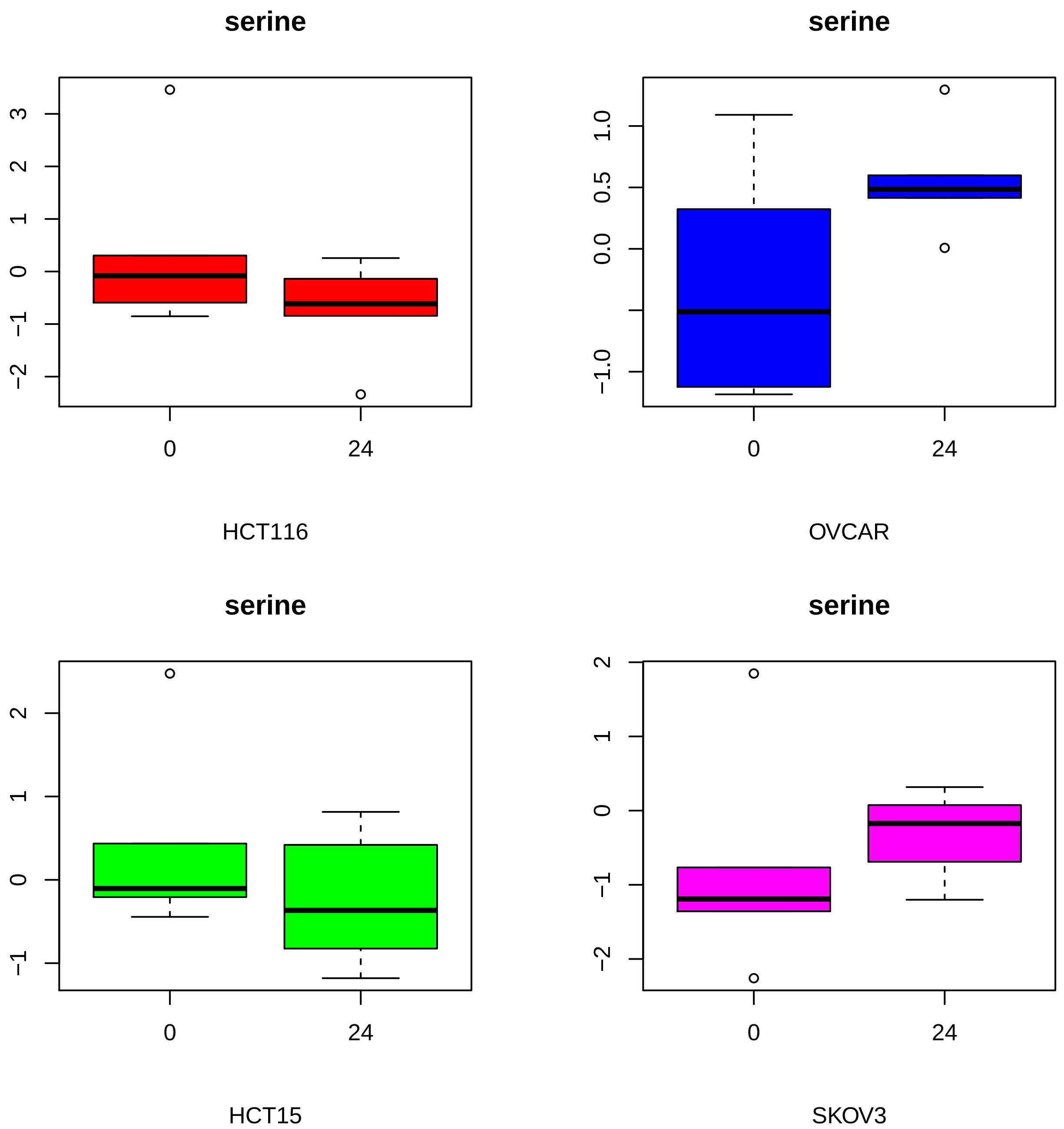
serine
HCT116
serine
OVCAR
serine
HCT15
serine
SKOV3
0
24

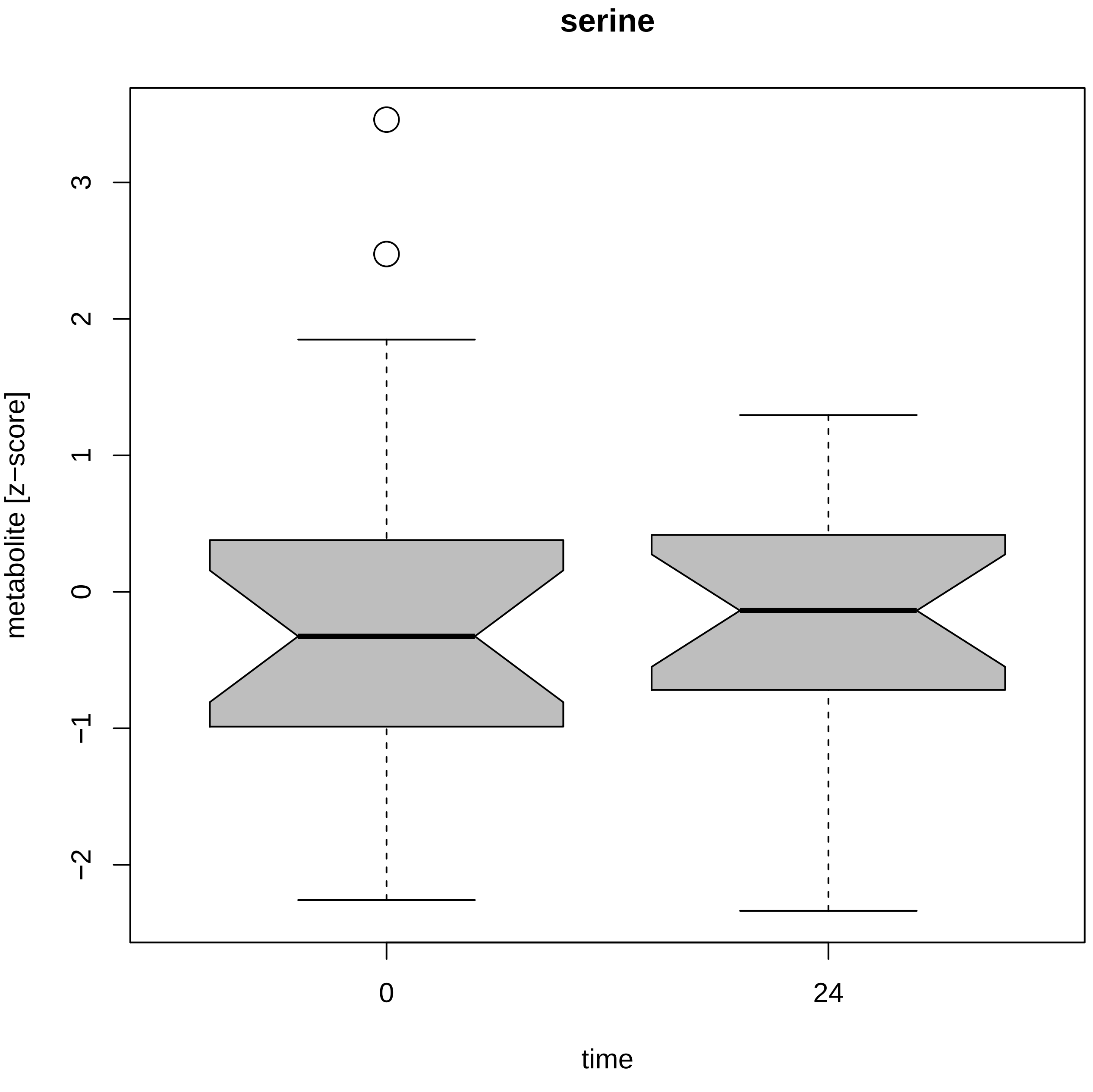
serine
metabolite [z−score]
3
2
1
0
−1
−2
0
24
time

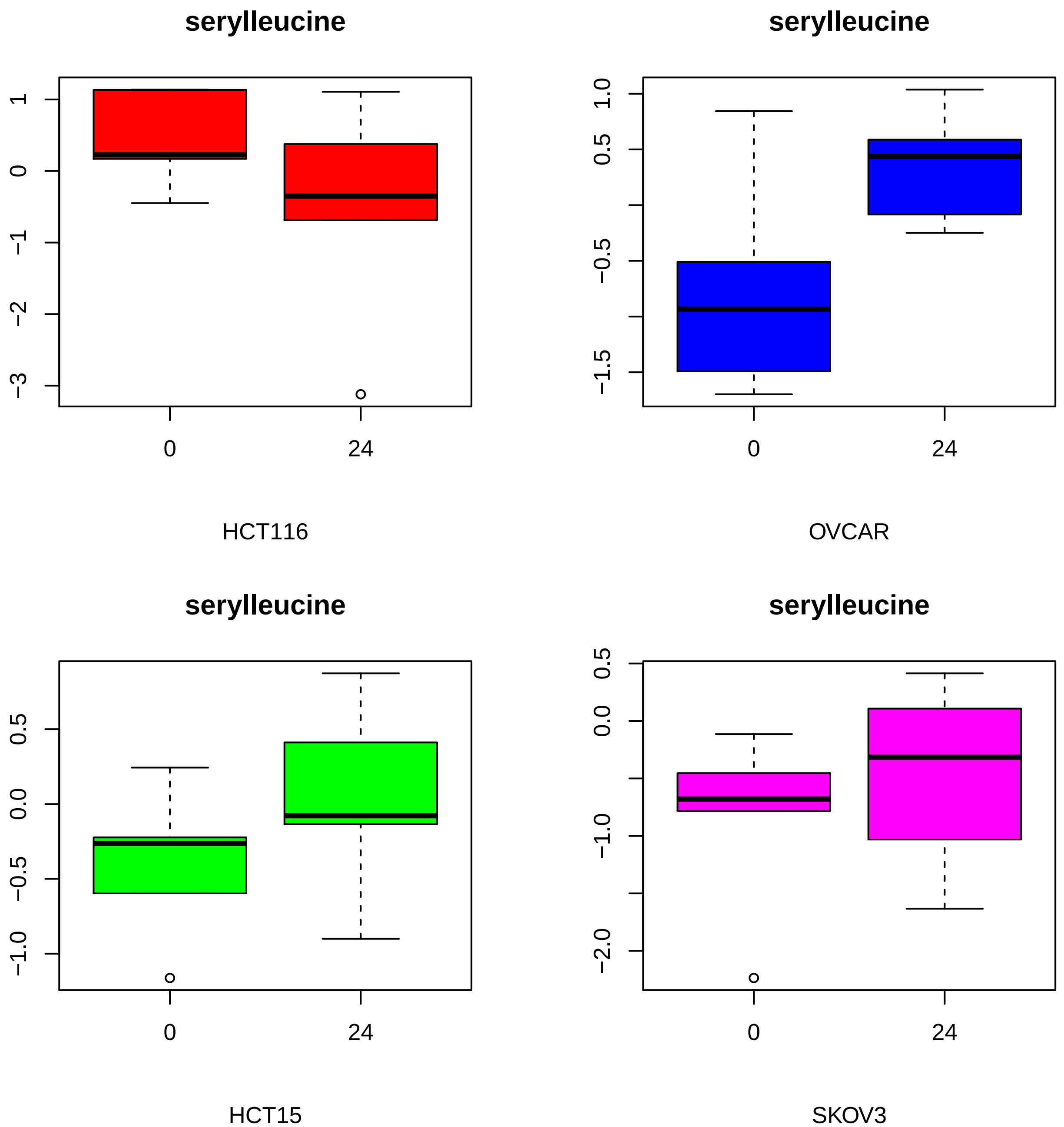
serylleucine
1
0
−1
−2
−3
0
24
HCT116
serylleucine
1.0
0.5
−0.5
−1.5
0
24
OVCAR
serylleucine
0.5
0.0
−0.5
−1.0
0
24
HCT15
serylleucine
0.5
0.0
−1.0
−2.0
0
24
SKOV3

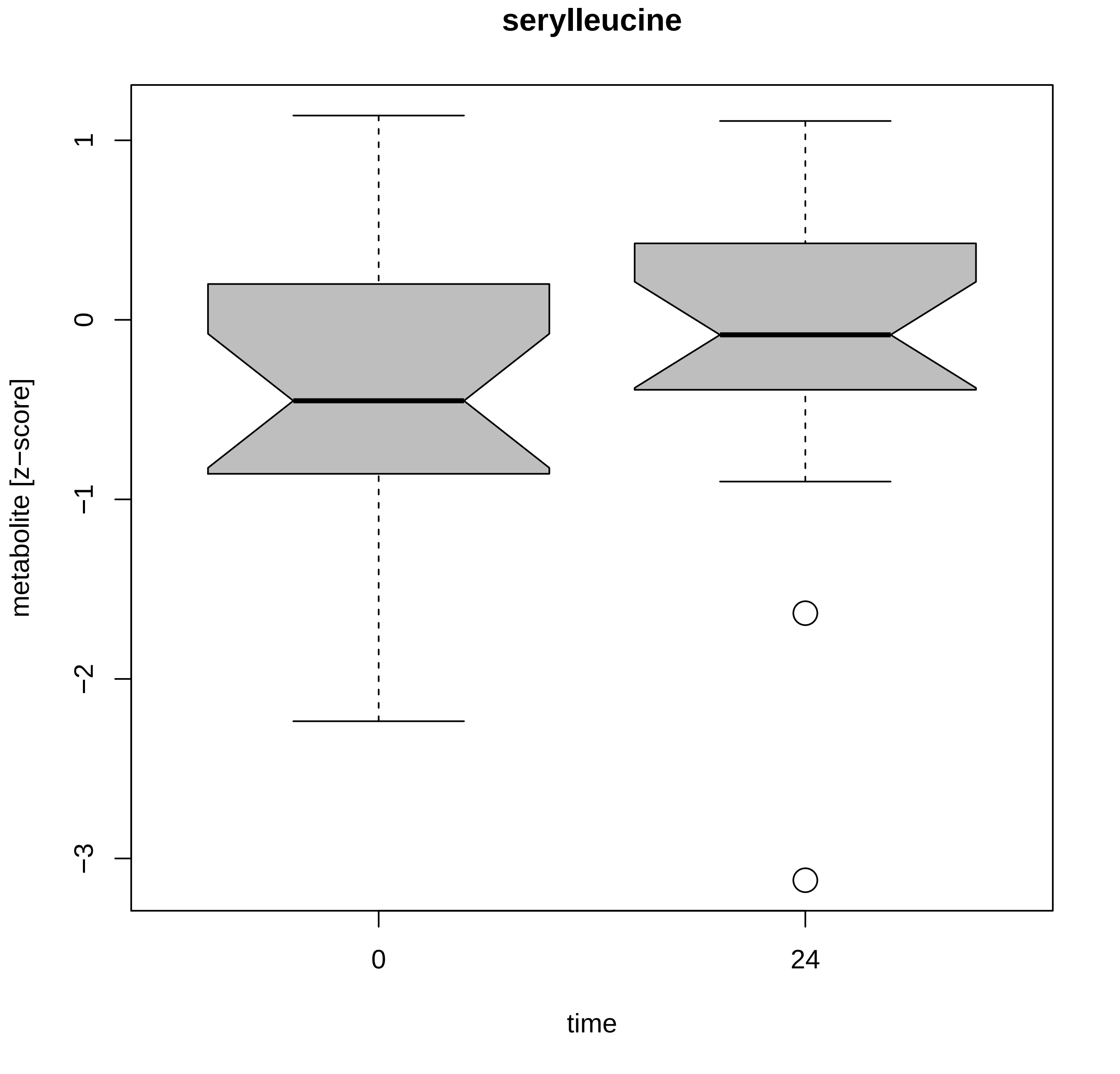

serylleucine
metabolite [z−score]
1
0
−1
−2
−3
0
24
time

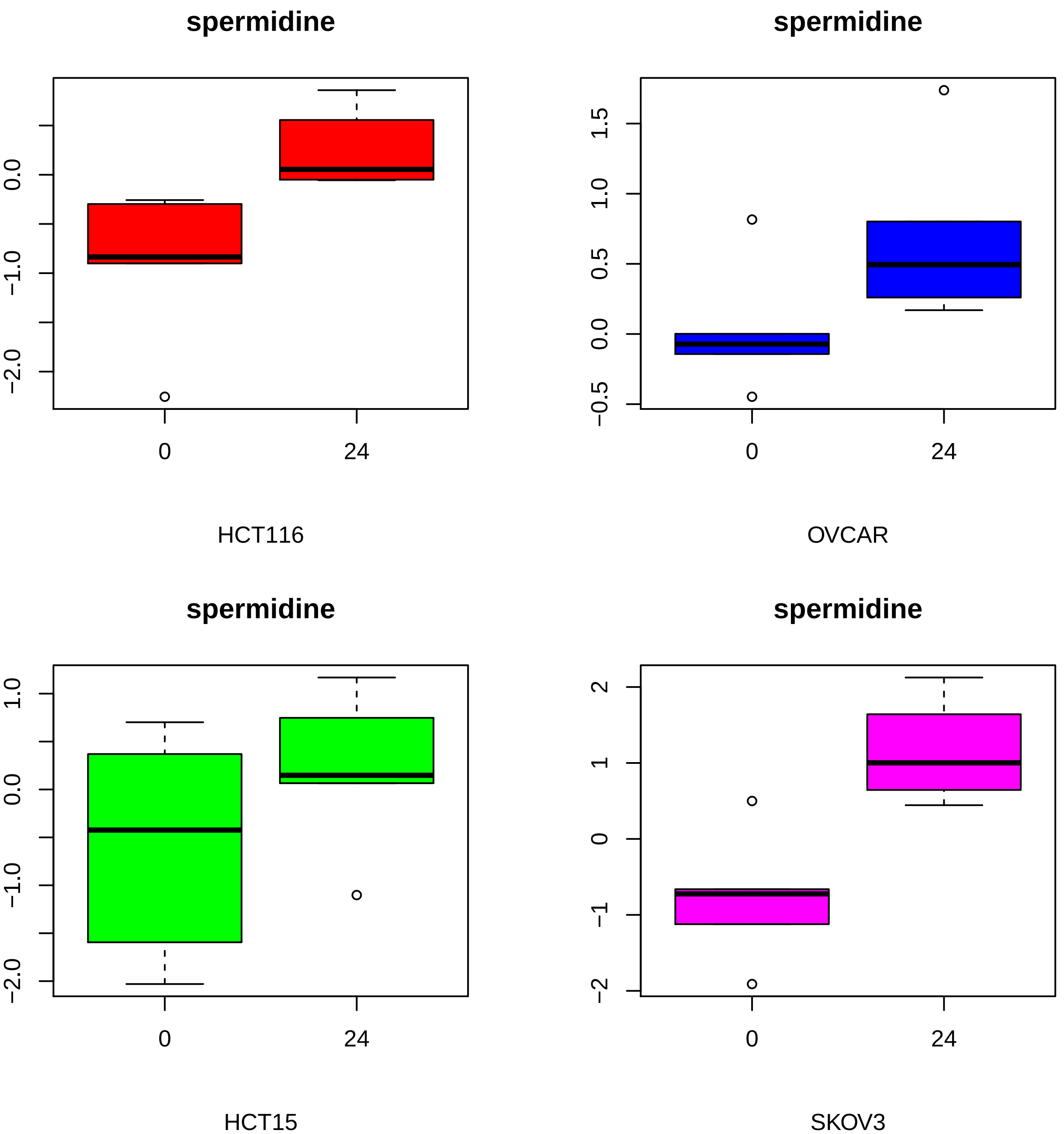
spermidine
0.0
−1.0
−2.0
0
24
HCT116
spermidine
1.5
1.0
0.5
0.0
−0.5
0
24
OVCAR
spermidine
1.0
0.0
−1.0
−2.0
0
24
HCT15
spermidine
2
1
0
−1
−2
0
24
SKOV3

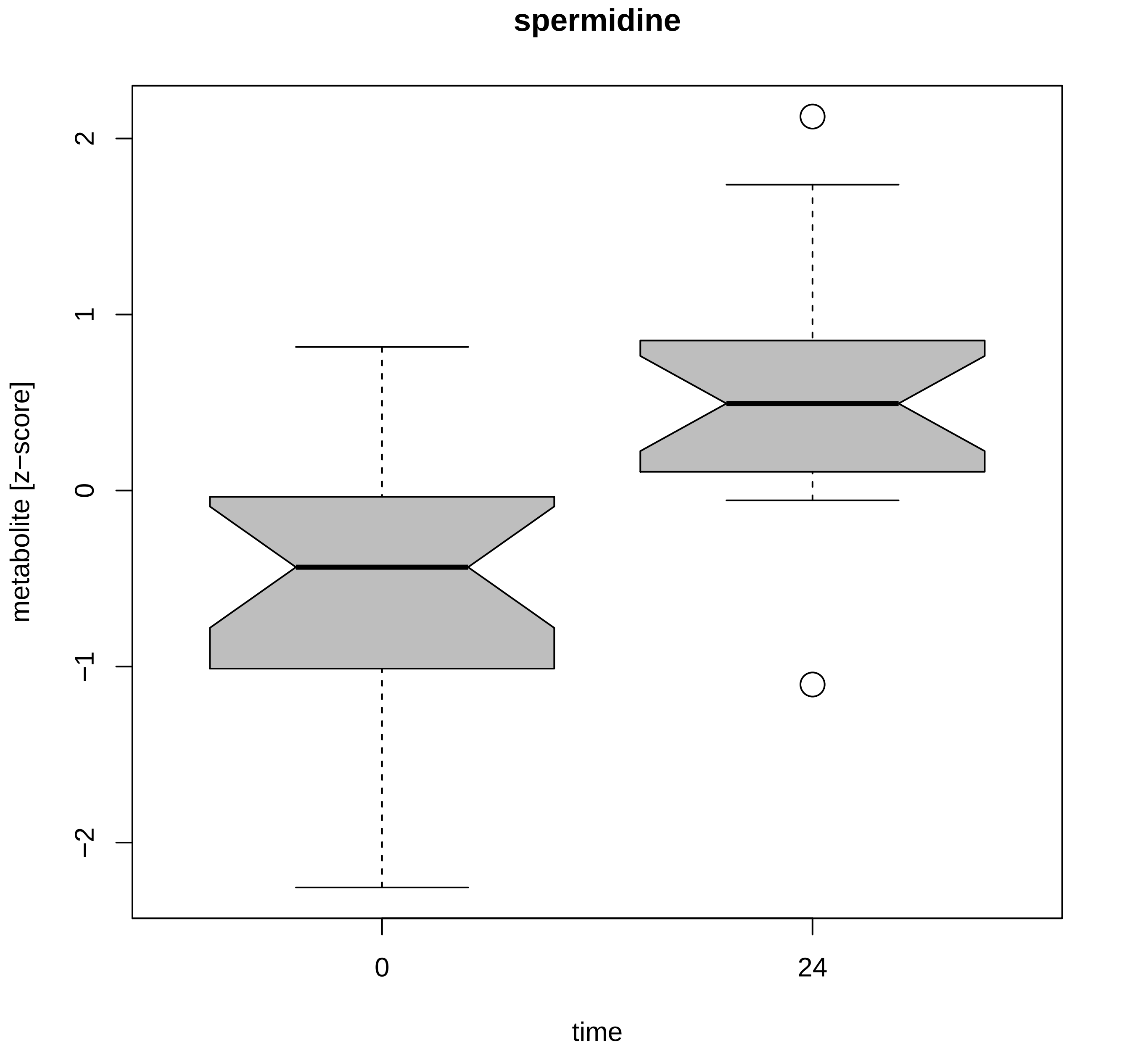
spermidine
metabolite [z−score]
2
1
0
−1
−2
0
24
time

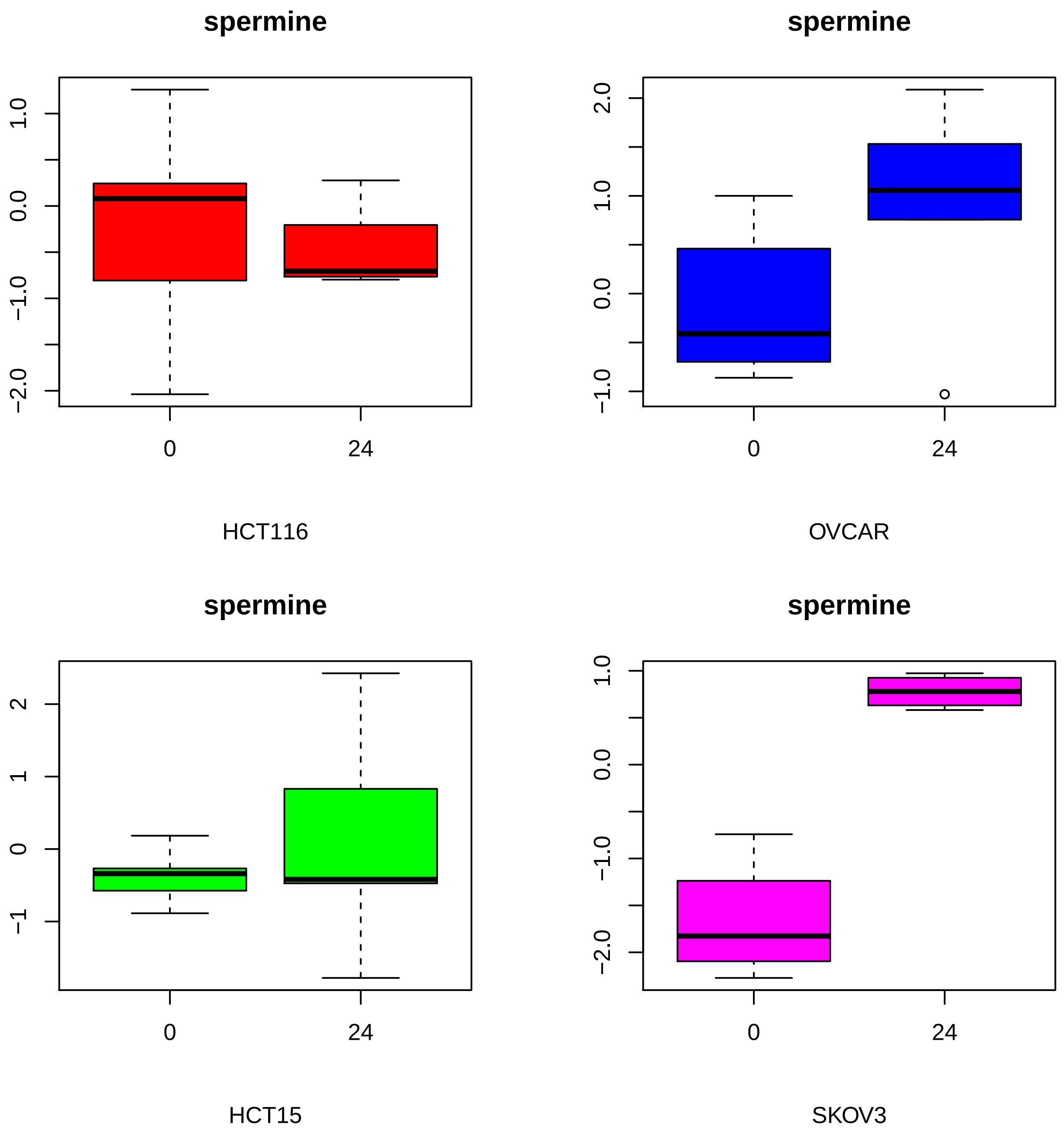
spermine
1.0
0.0
−1.0
−2.0
0
24
HCT116
spermine
2.0
1.0
0.0
−1.0
0
24
OVCAR
spermine
2
1
0
−1
0
24
HCT15
spermine
1.0
0.0
−1.0
−2.0
0
24
SKOV3

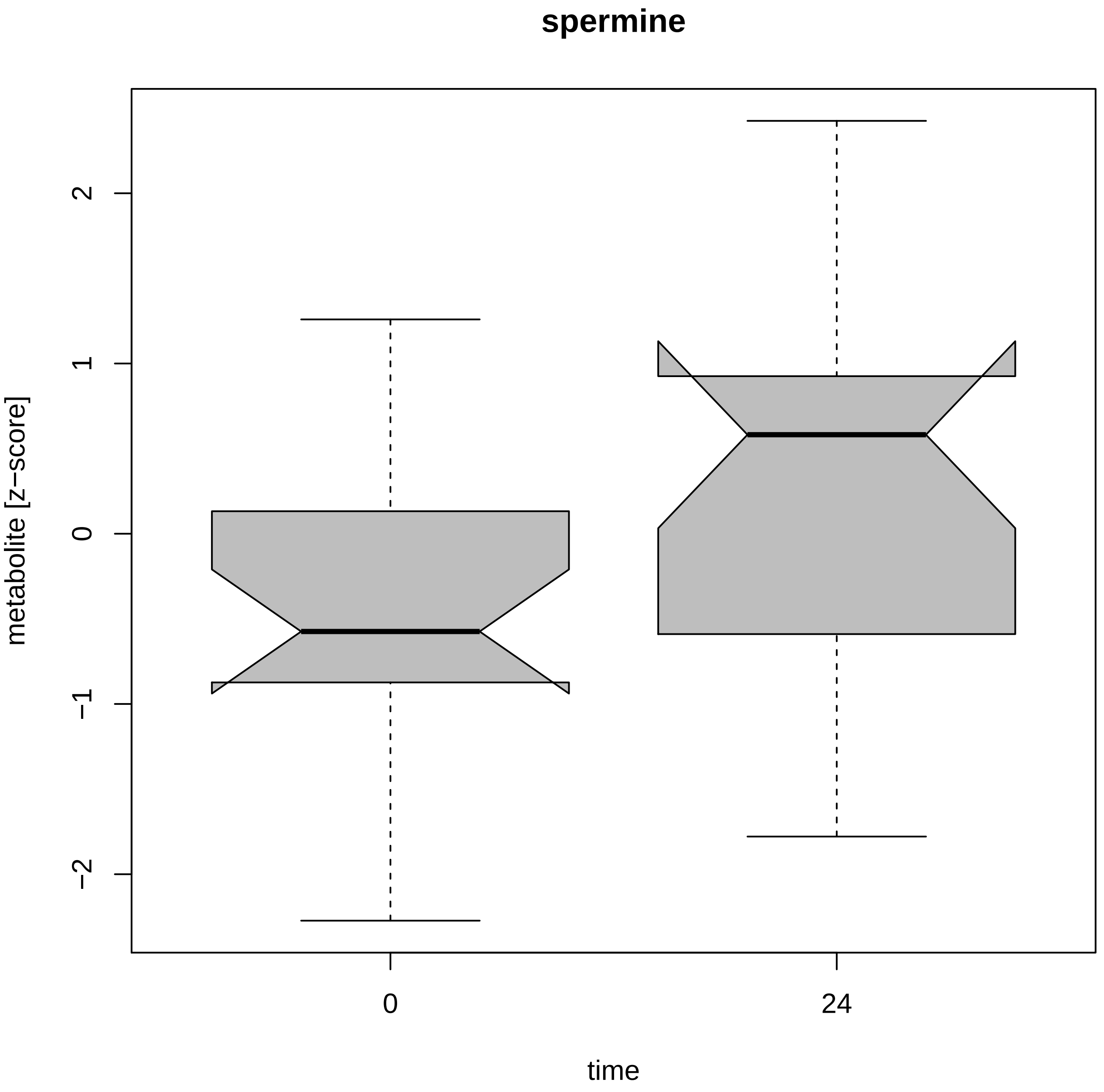
spermine
metabolite [z−score]
2
1
0
−1
−2
0
24
time

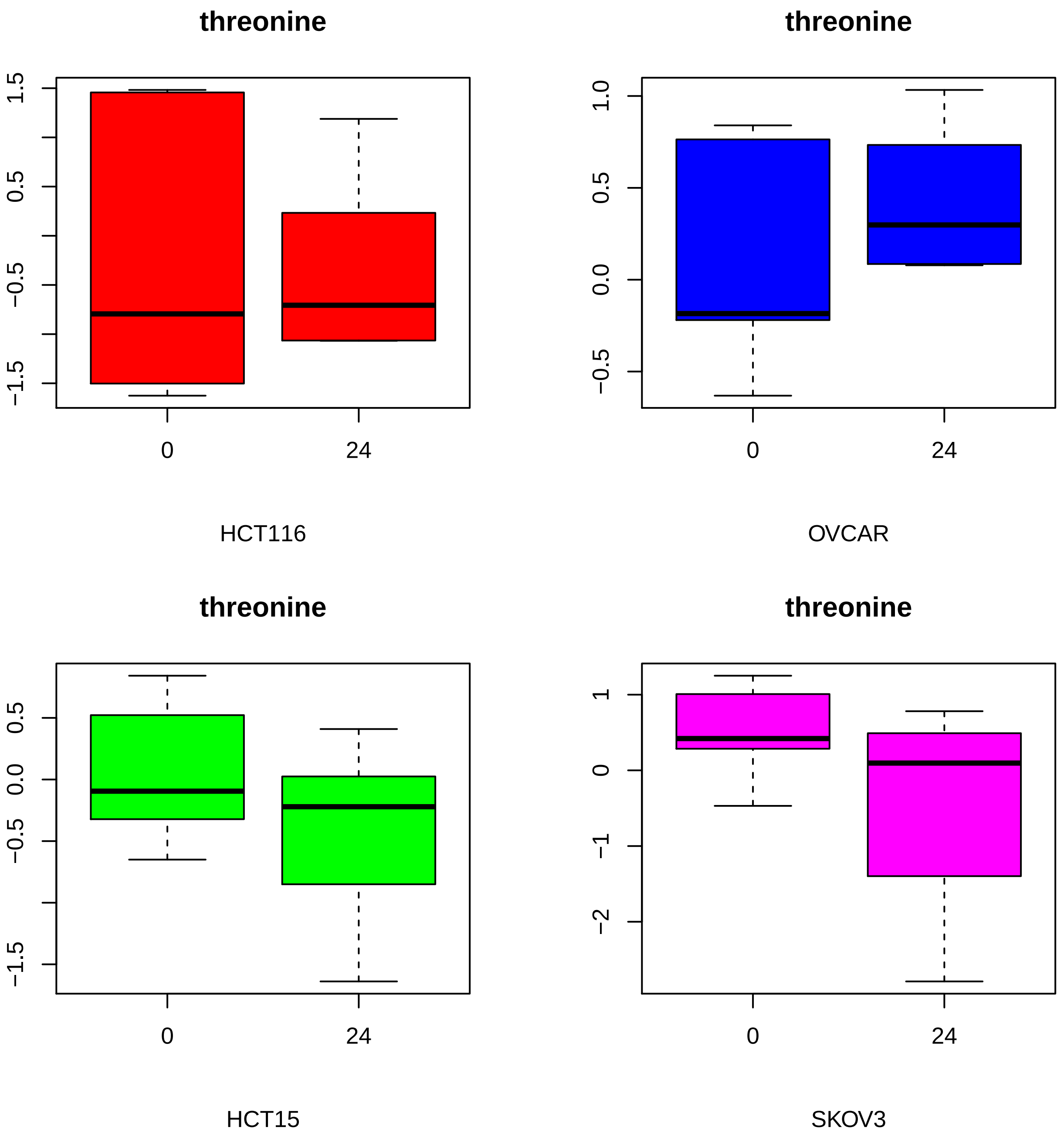
threonine
1.5
0.5
−0.5
−1.5
0
24
HCT116
threonine
1.0
0.5
0.0
−0.5
0
24
OVCAR
threonine
0.5
0.0
−0.5
−1.5
0
24
HCT15
threonine
1
0
−1
−2
0
24
SKOV3

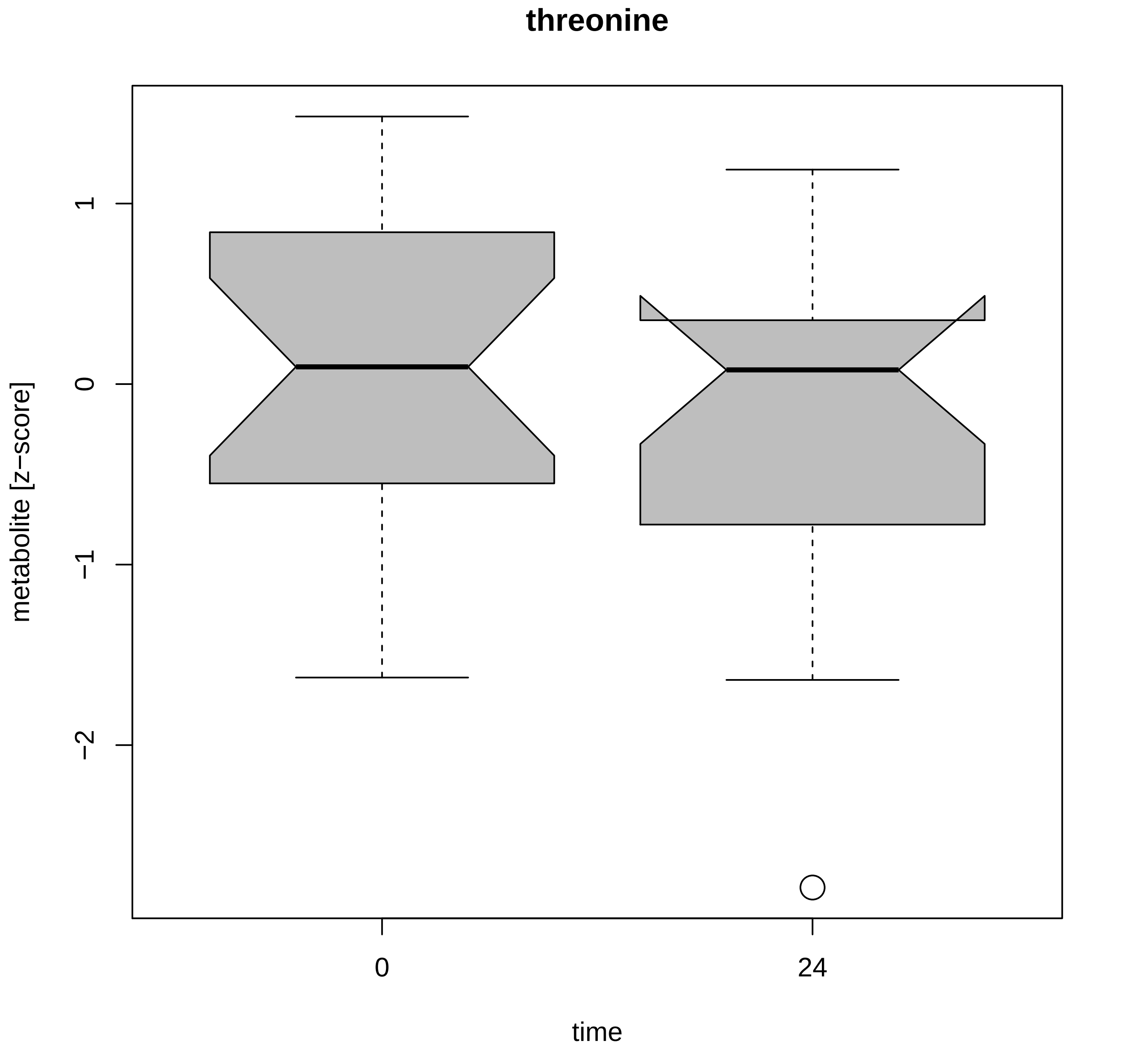
threonine
metabolite [z−score]
1
0
−1
−2
0
24
time

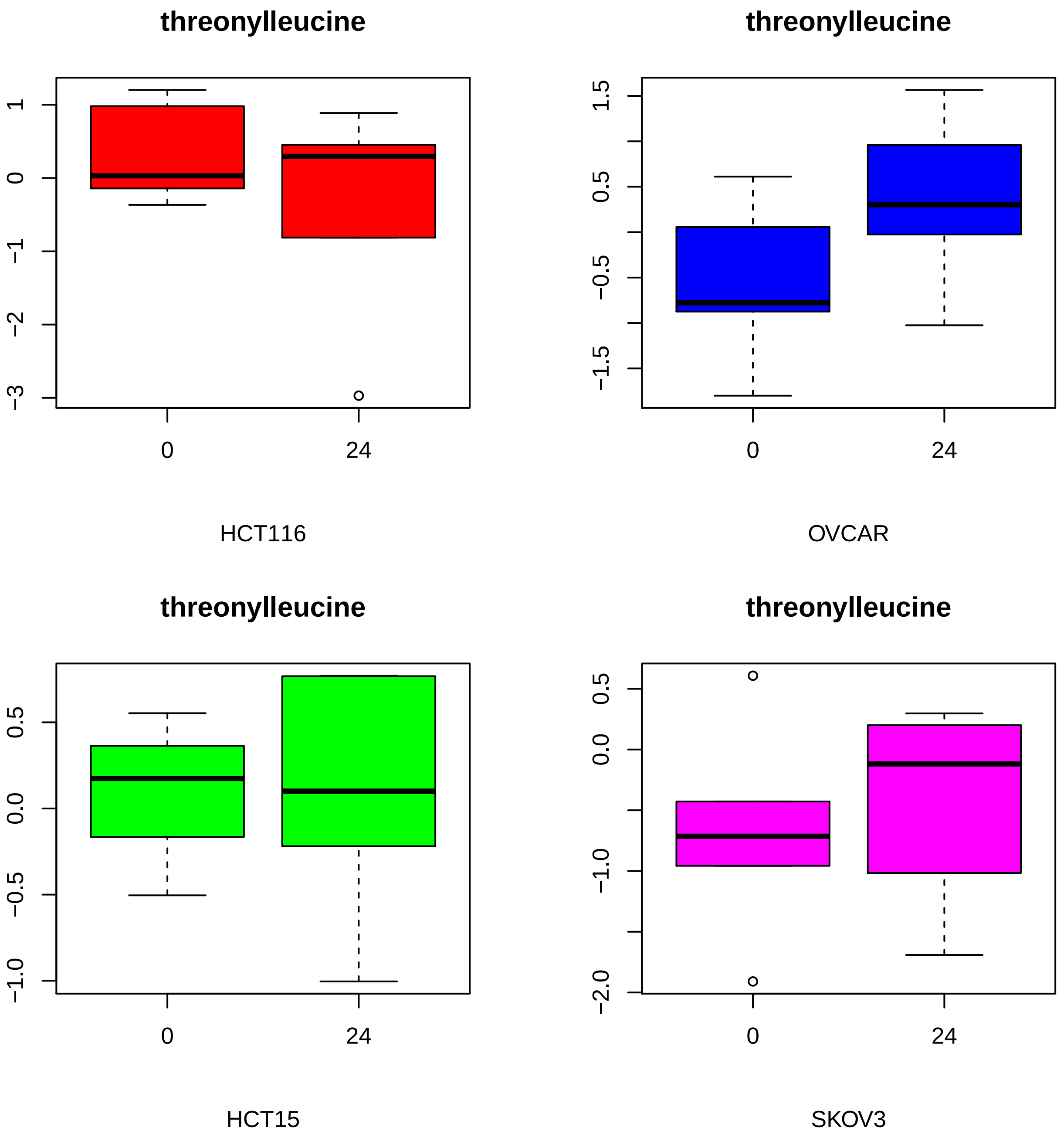
threonylleucine
1
0
−1
−2
−3
0
24
HCT116
threonylleucine
1.5
0.5
−0.5
−1.5
0
24
OVCAR
threonylleucine
0.5
0.0
−0.5
−1.0
0
24
HCT15
threonylleucine
0.5
0.0
−1.0
−2.0
0
24
SKOV3

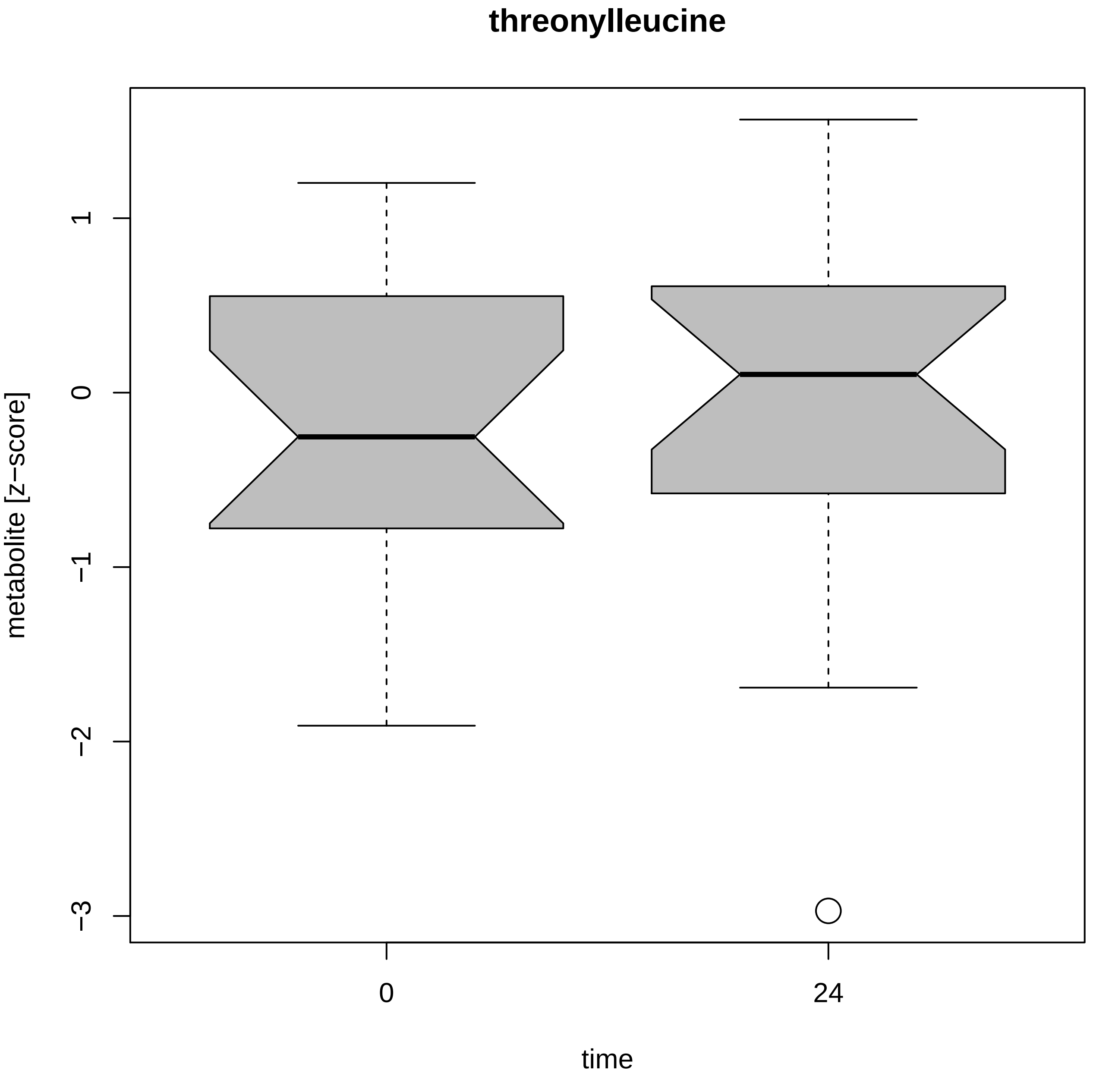
threonylleucine
1
0
−1
−2
−3
metabolite [z−score]
0
24
time

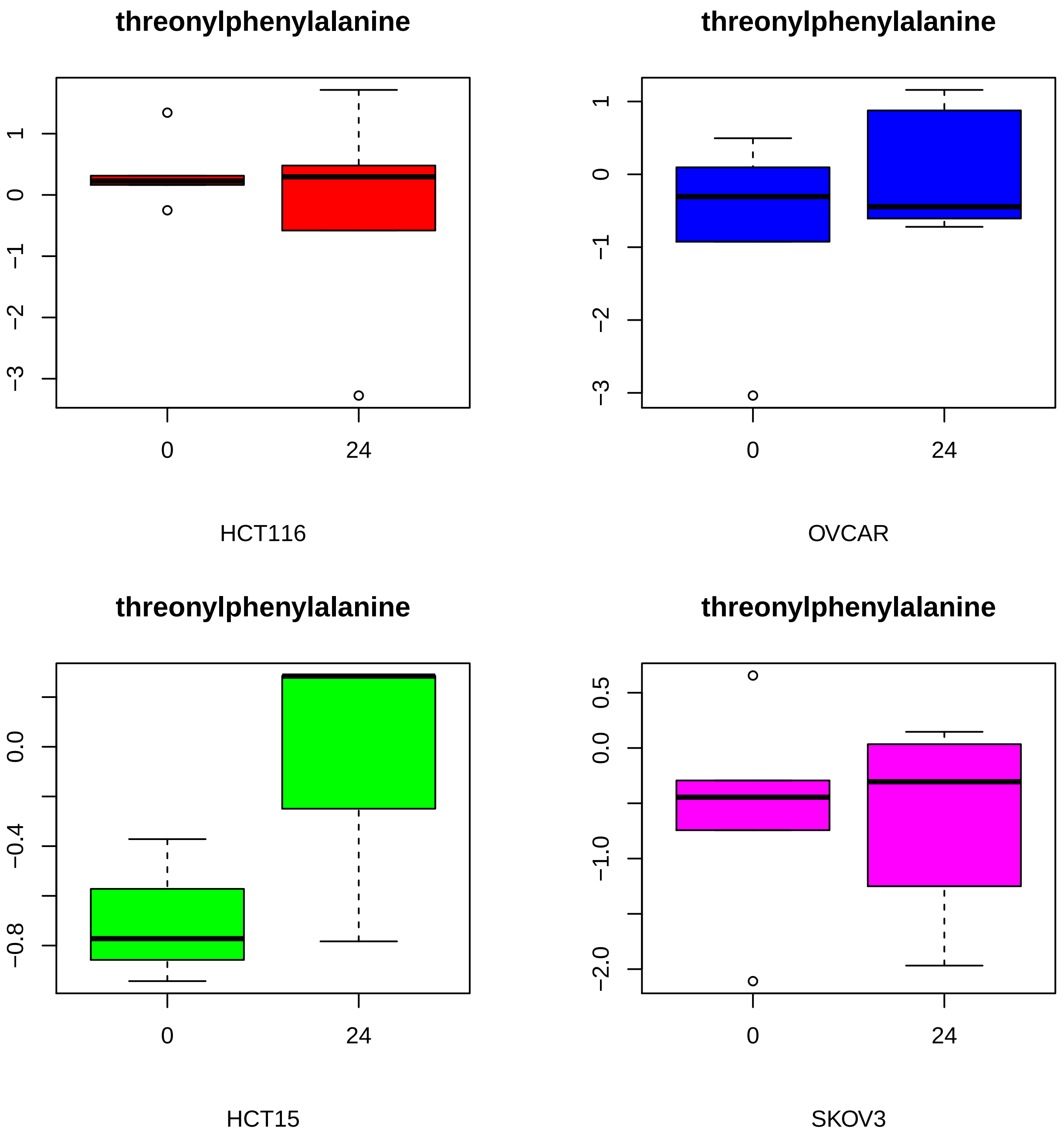
threonylphenylalanine
1
0
−1
−2
−3
0
24
HCT116
threonylphenylalanine
1
0
−1
−2
−3
0
24
OVCAR
threonylphenylalanine
0.0
−0.4
−0.8
0
24
HCT15
threonylphenylalanine
0.5
0.0
−1.0
−2.0
0
24
SKOV3

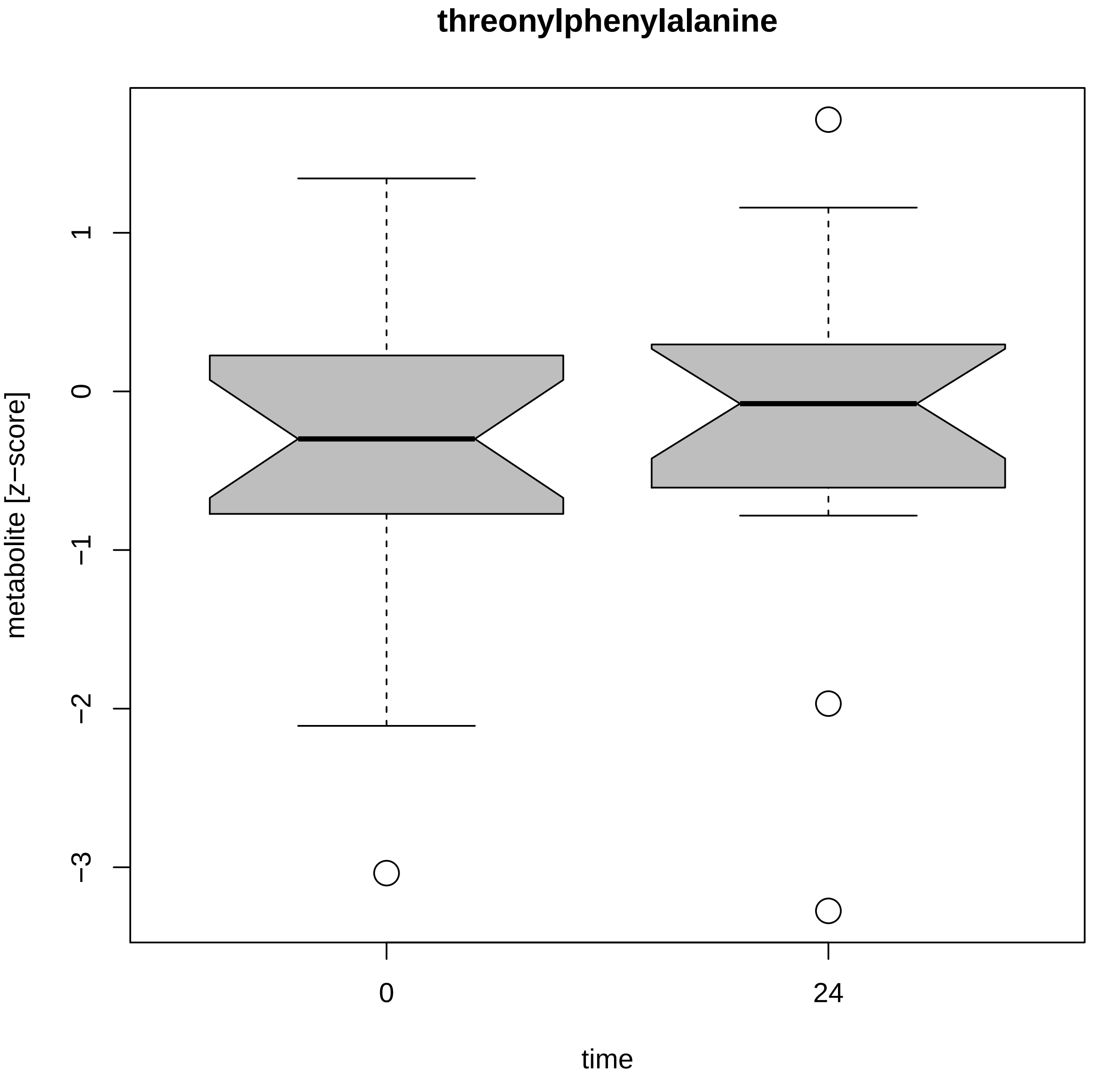

threonylphenylalanine
metabolite [z−score]
1
0
−1
−2
−3
0
24
time

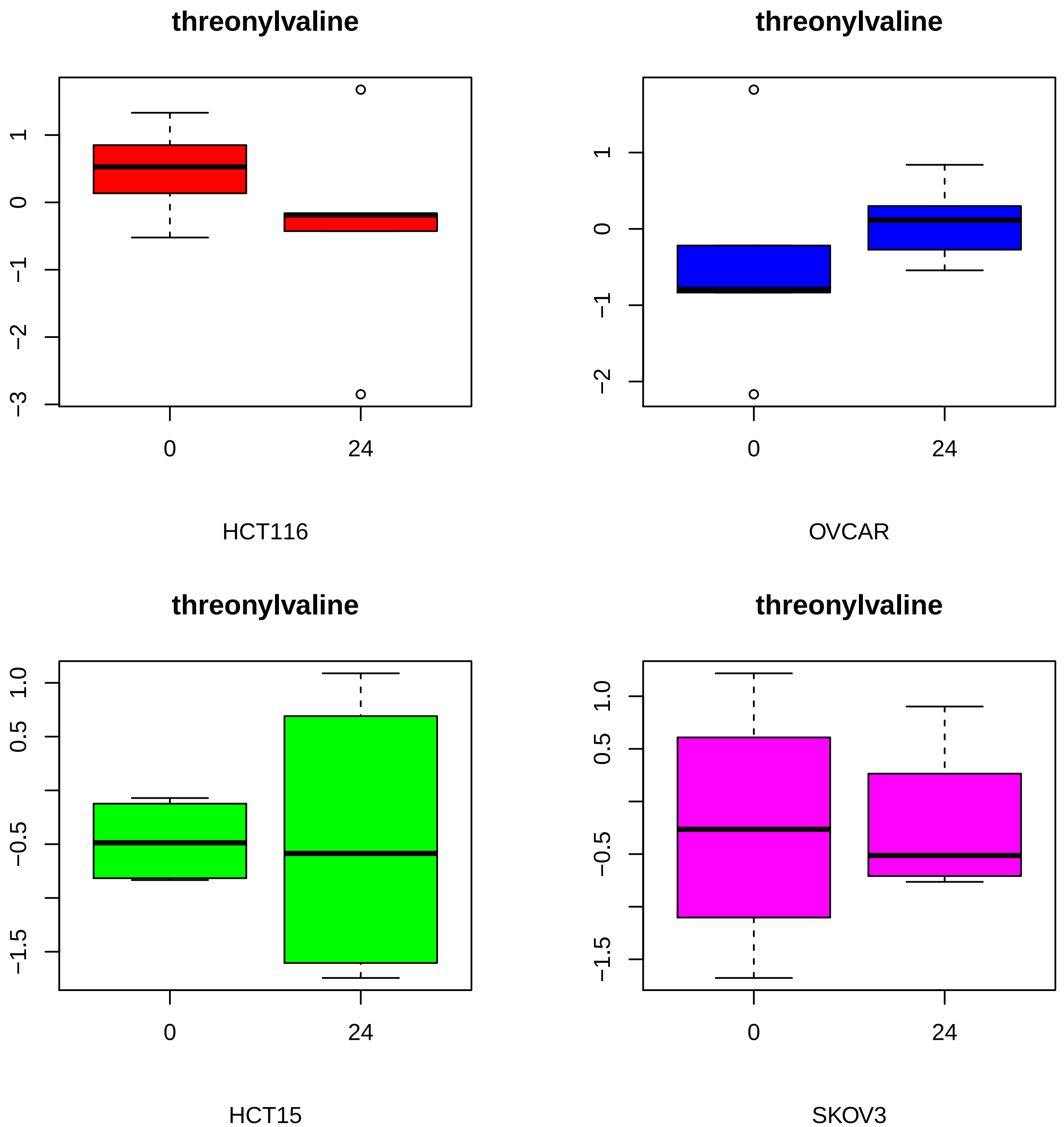
threonylvaline
HCT116
OVCAR
HCT15
SKOV3
0
24
1
0
−1
−2
−3
1.0
0.5
−0.5
−1.5

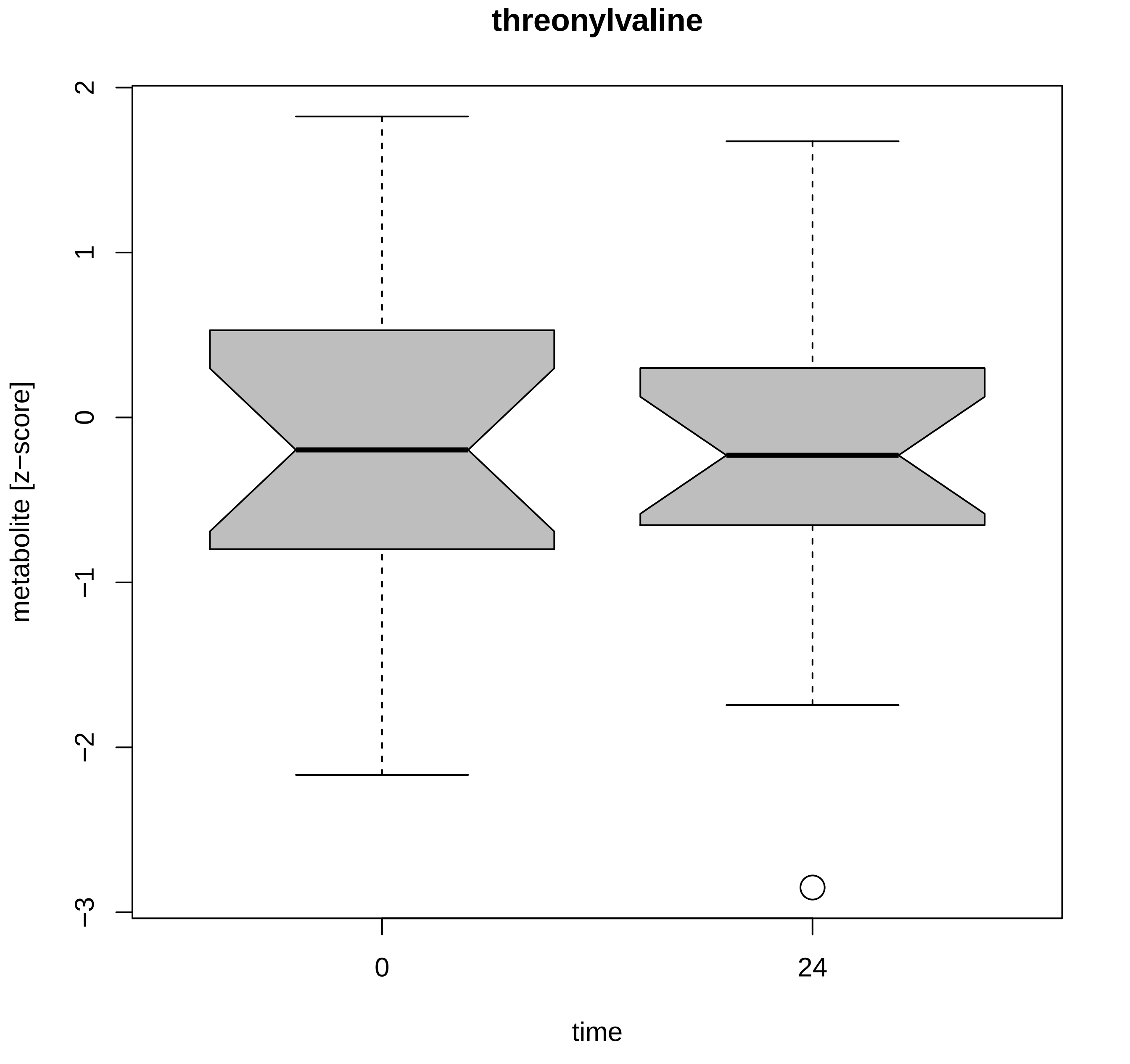
threonylvaline
metabolite [z−score]
2
1
0
−1
−2
−3
0
24
time

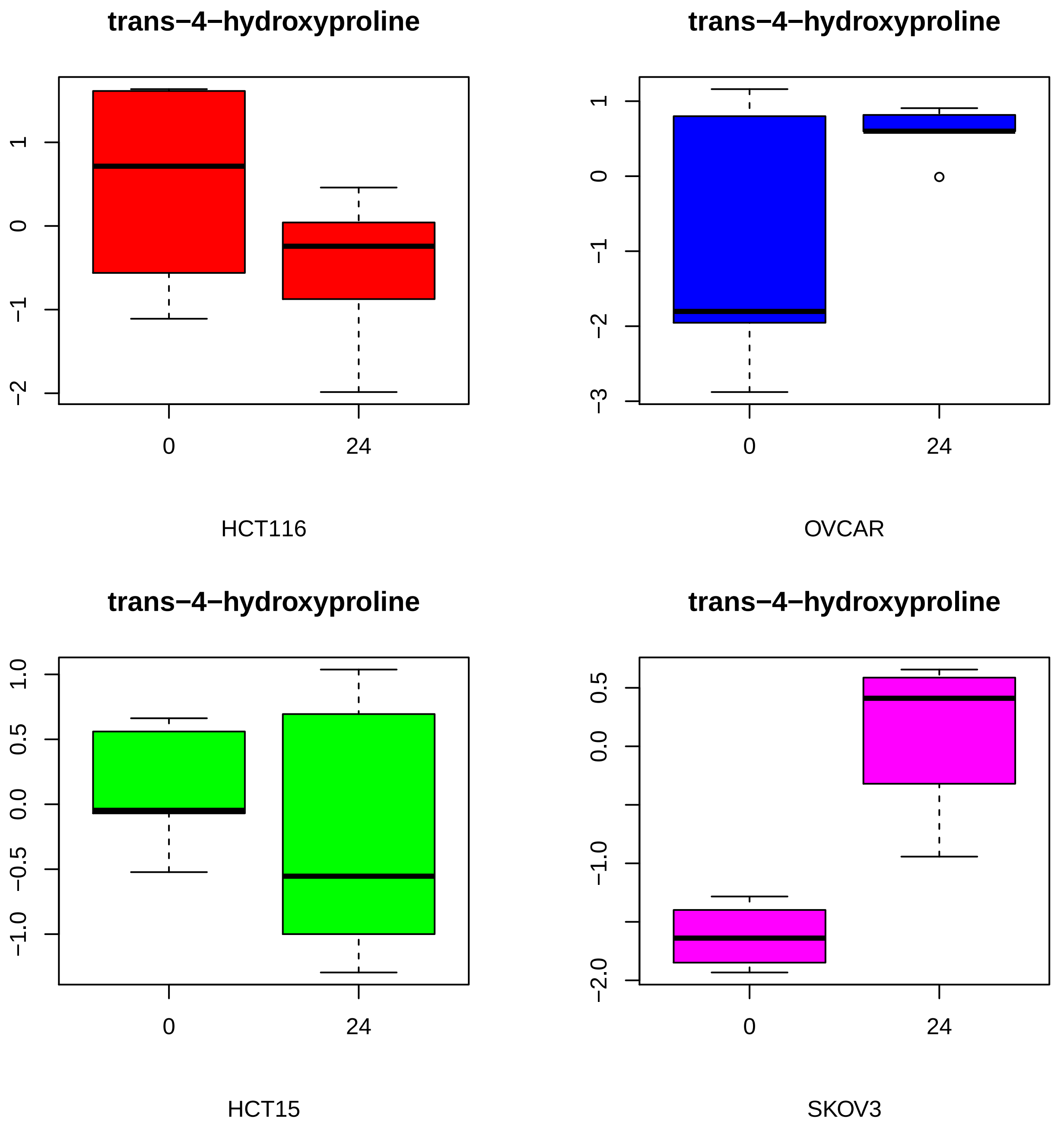
trans−4−hydroxyproline
1
0
−1
−2
0
24
HCT116
trans−4−hydroxyproline
1
0
−1
−2
−3
0
24
OVCAR
trans−4−hydroxyproline
1.0
0.5
0.0
−0.5
−1.0
0
24
HCT15
trans−4−hydroxyproline
0.5
0.0
−1.0
−2.0
0
24
SKOV3

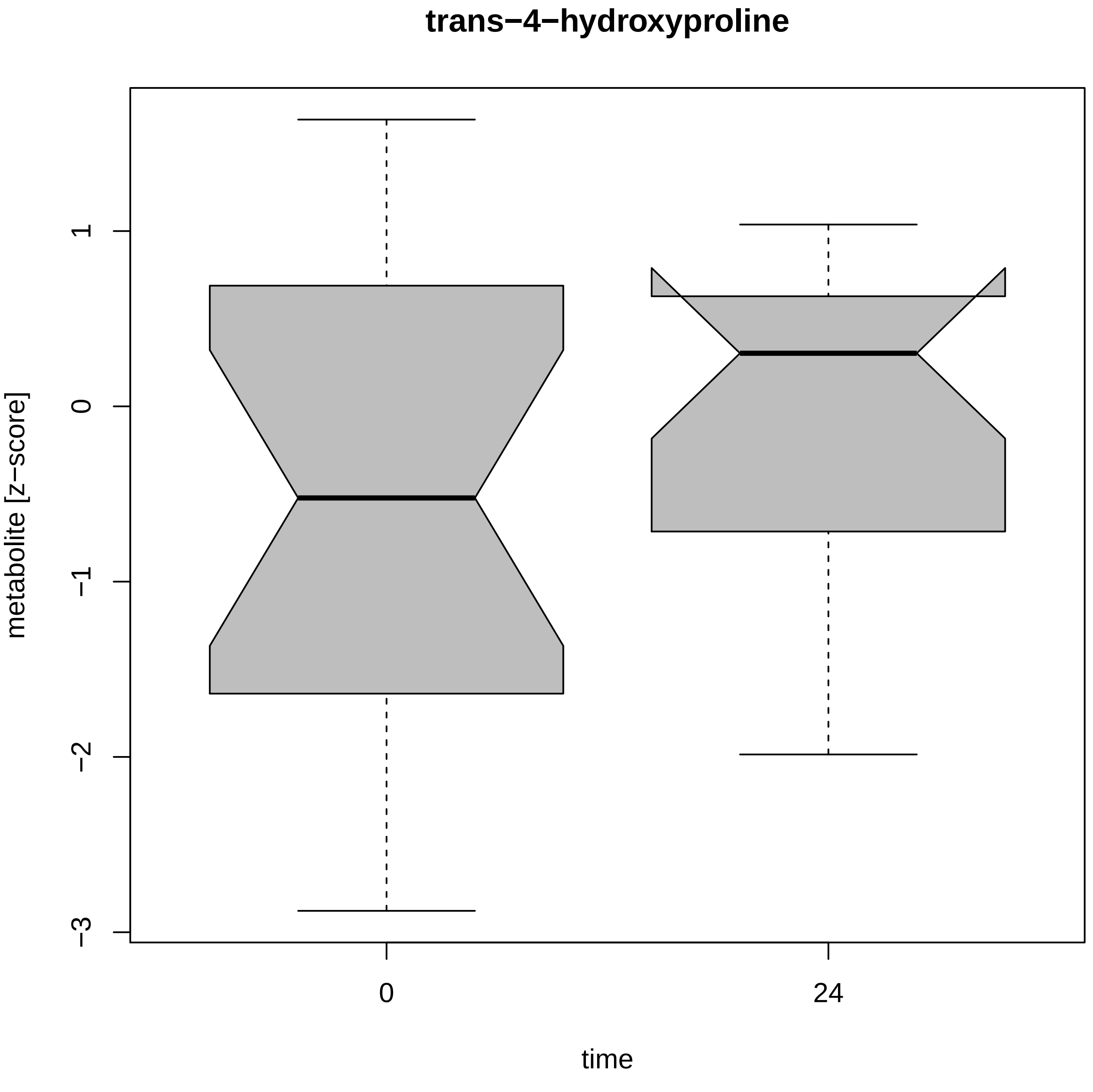

trans−4−hydroxyproline
metabolite [z−score]
1
0
−1
−2
−3
0
24
time

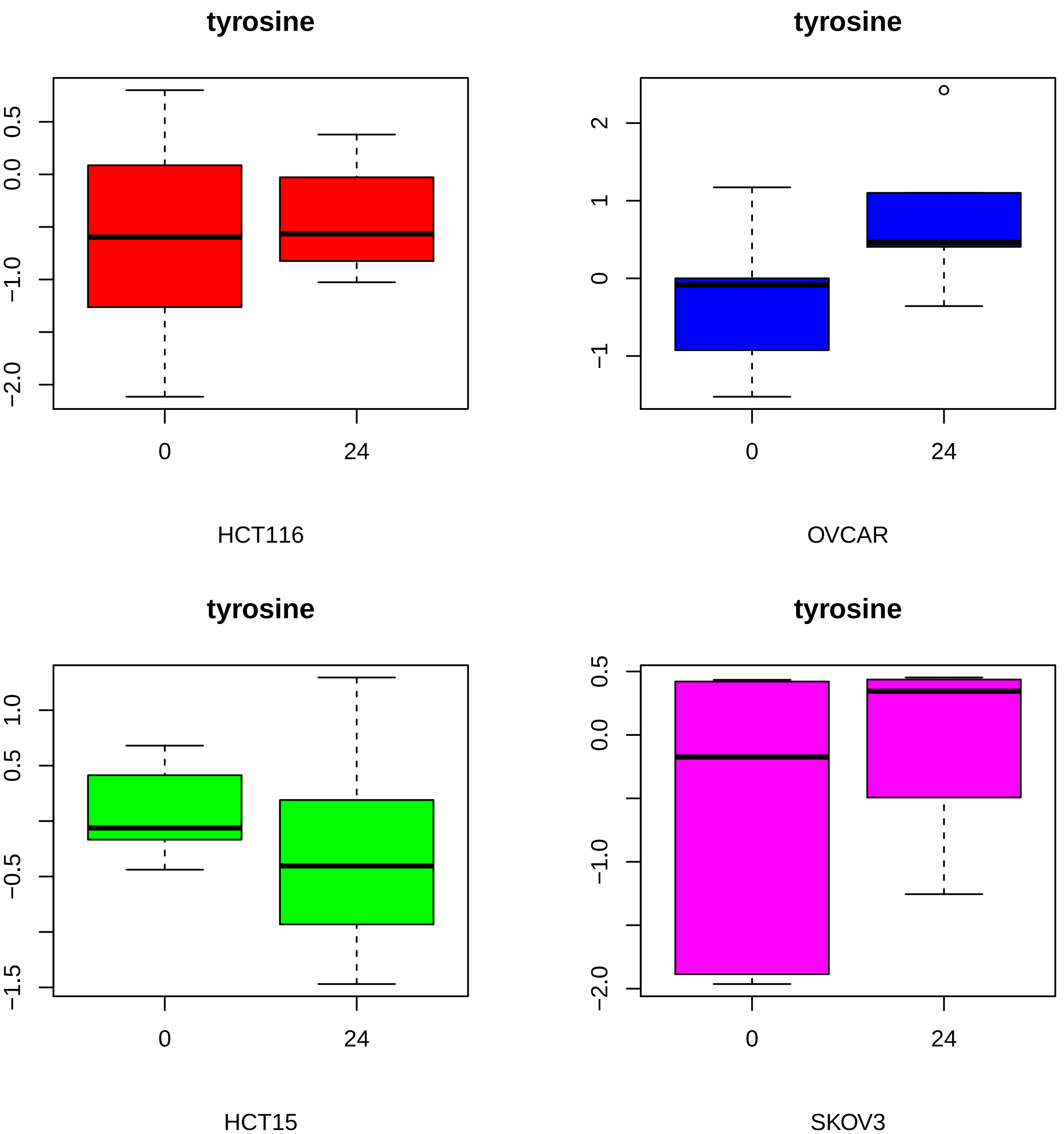

tyrosine
0.5
0.0
−1.0
−2.0
0
24
HCT116
tyrosine
2
1
0
−1
0
24
OVCAR
tyrosine
1.0
0.5
−0.5
−1.5
0
24
HCT15
tyrosine
0.5
0.0
−1.0
−2.0
0
24
SKOV3

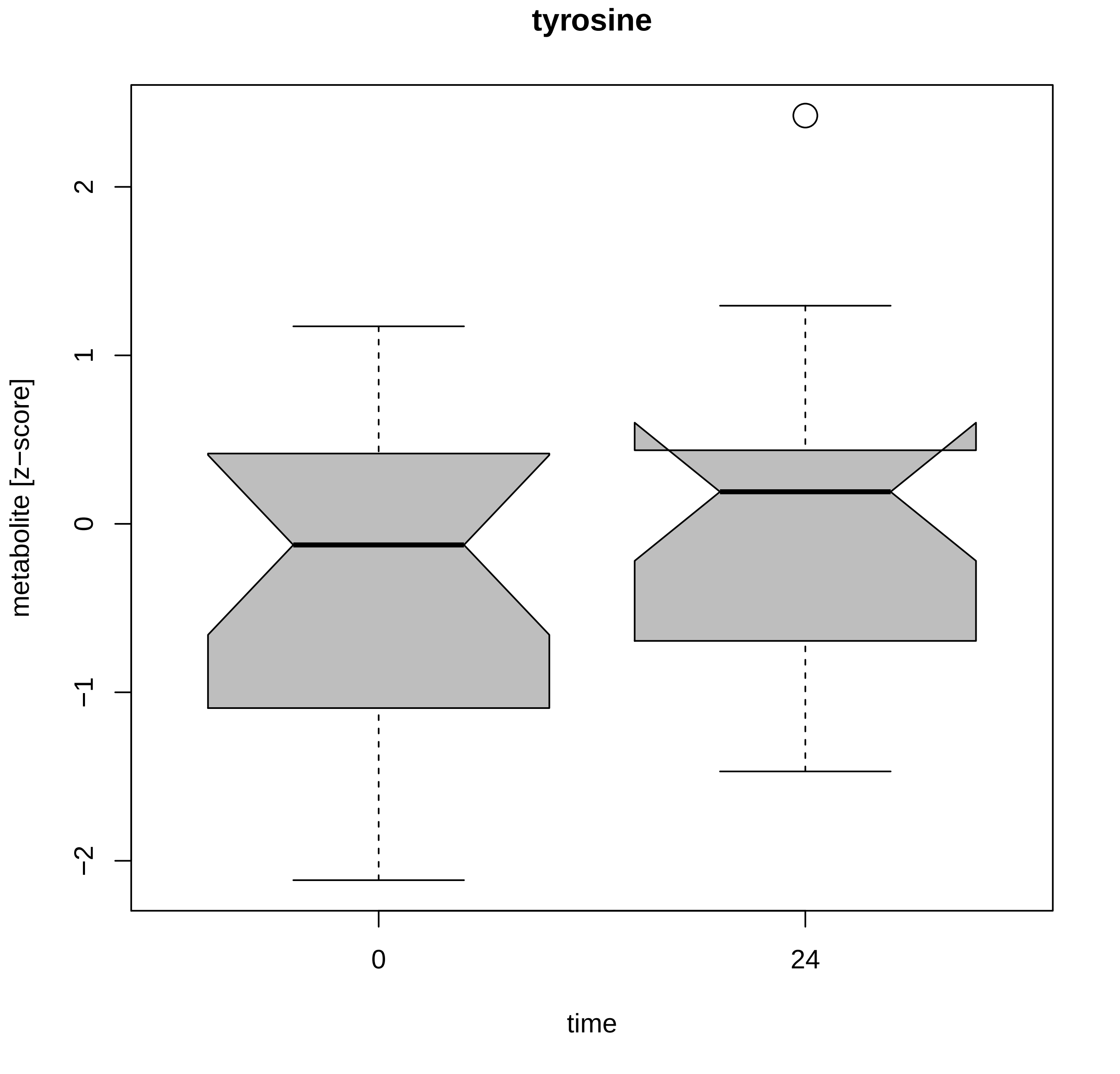
tyrosine
metabolite [z−score]
2
1
0
−1
−2
0
24
time

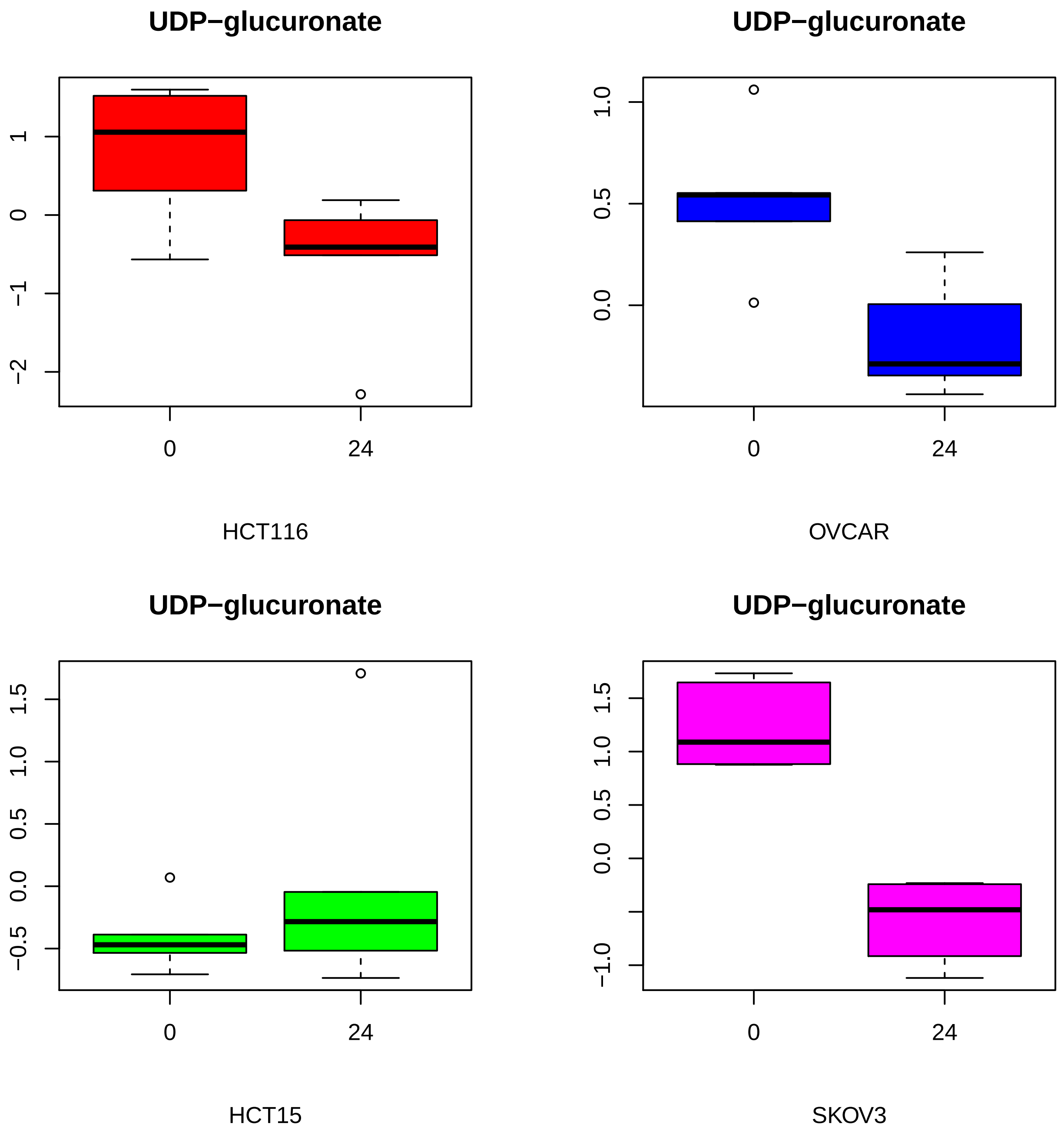
UDP−glucuronate
−2
−1
0
1
0
24
HCT116
UDP−glucuronate
0.0
0.5
1.0
0
24
OVCAR
UDP−glucuronate
−0.5
0.0
0.5
1.0
1.5
0
24
HCT15
UDP−glucuronate
−1.0
0.0
0.5
1.0
1.5
0
24
SKOV3

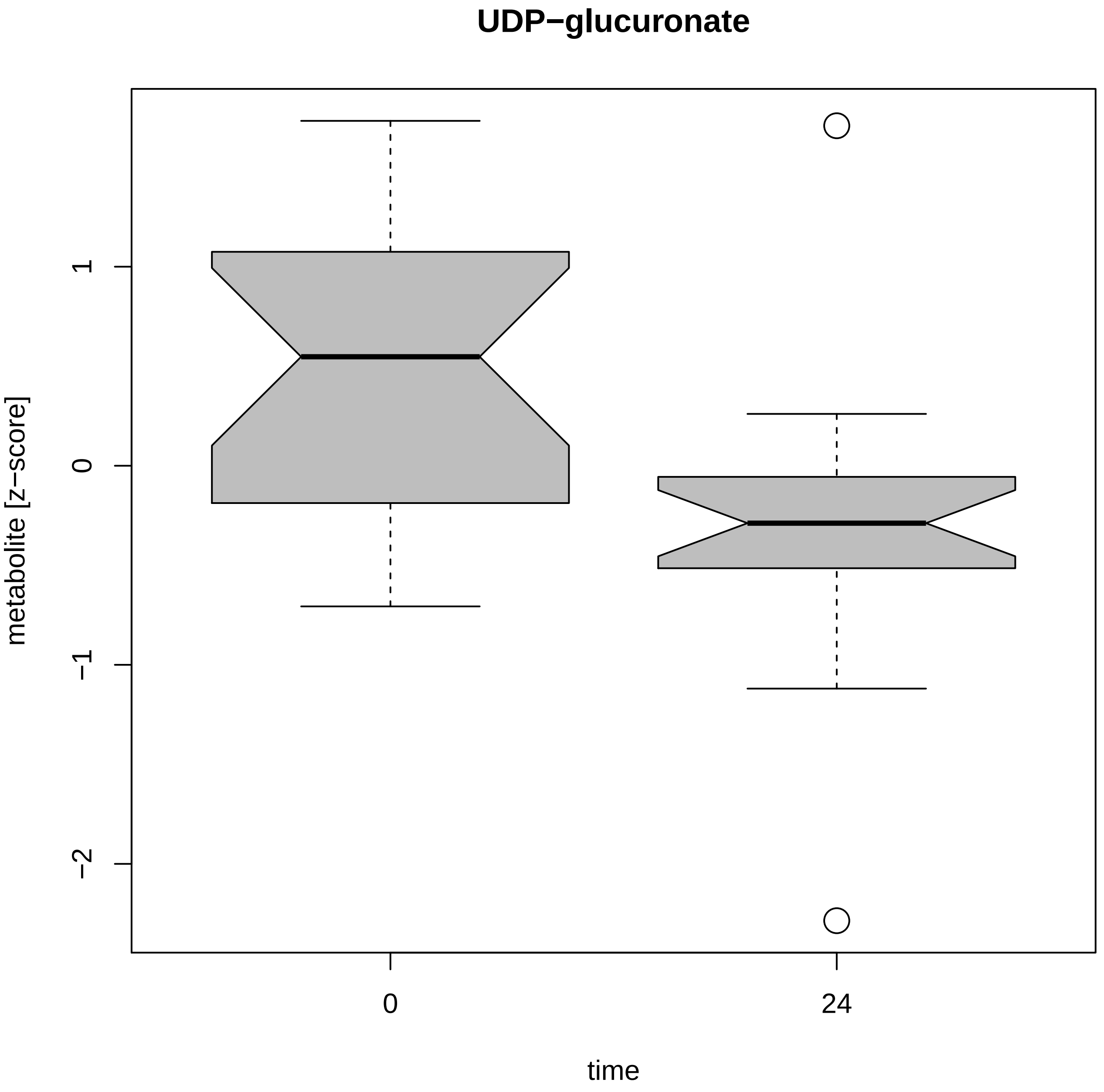
UDP−glucuronate
metabolite [z−score]
1
0
−1
−2
0
24
time

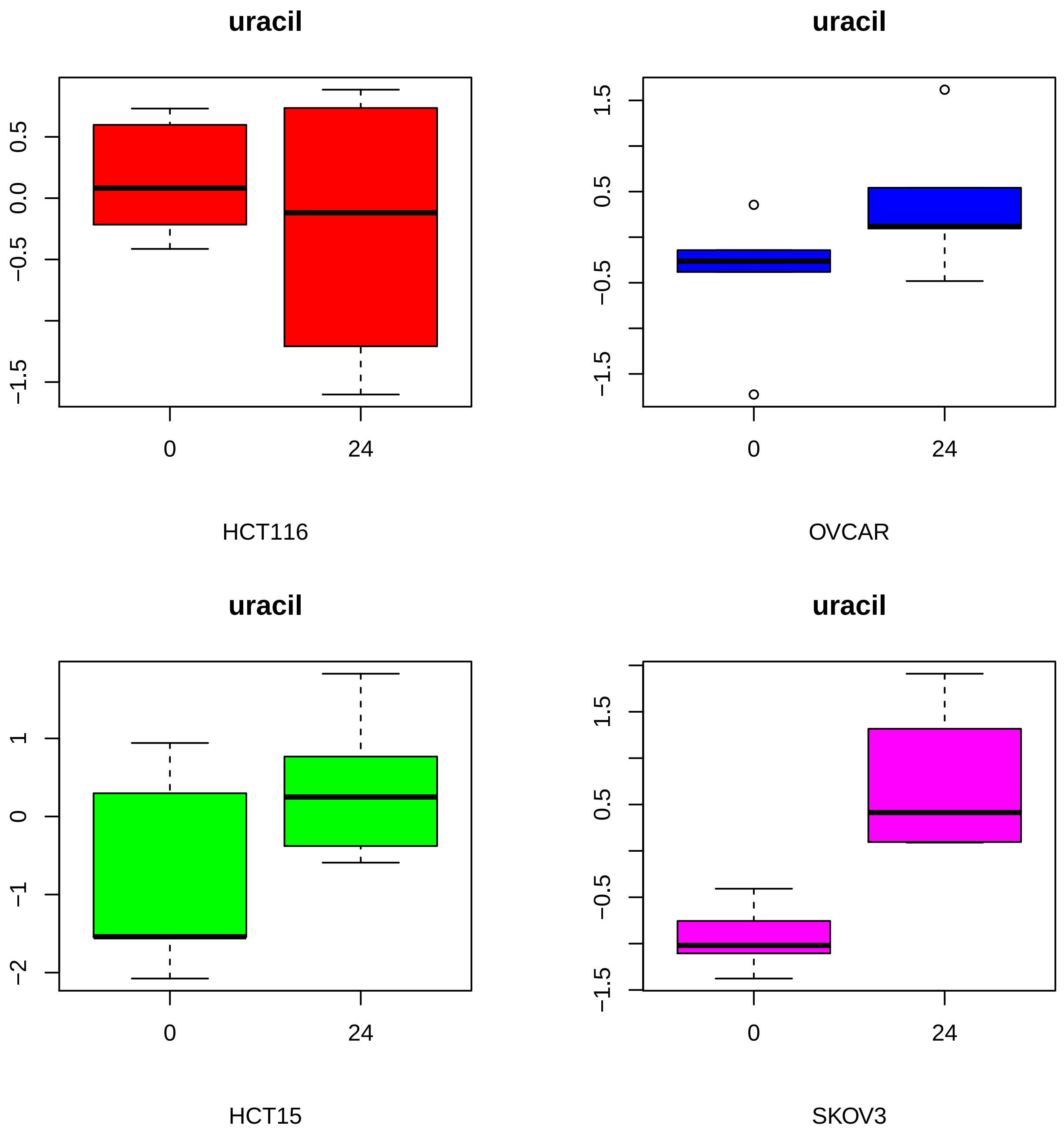
uracil
0.5
0.0
−0.5
−1.5
0
24
HCT116
uracil
1.5
0.5
−0.5
−1.5
0
24
OVCAR
uracil
1
0
−1
−2
0
24
HCT15
uracil
1.5
0.5
−0.5
−1.5
0
24
SKOV3

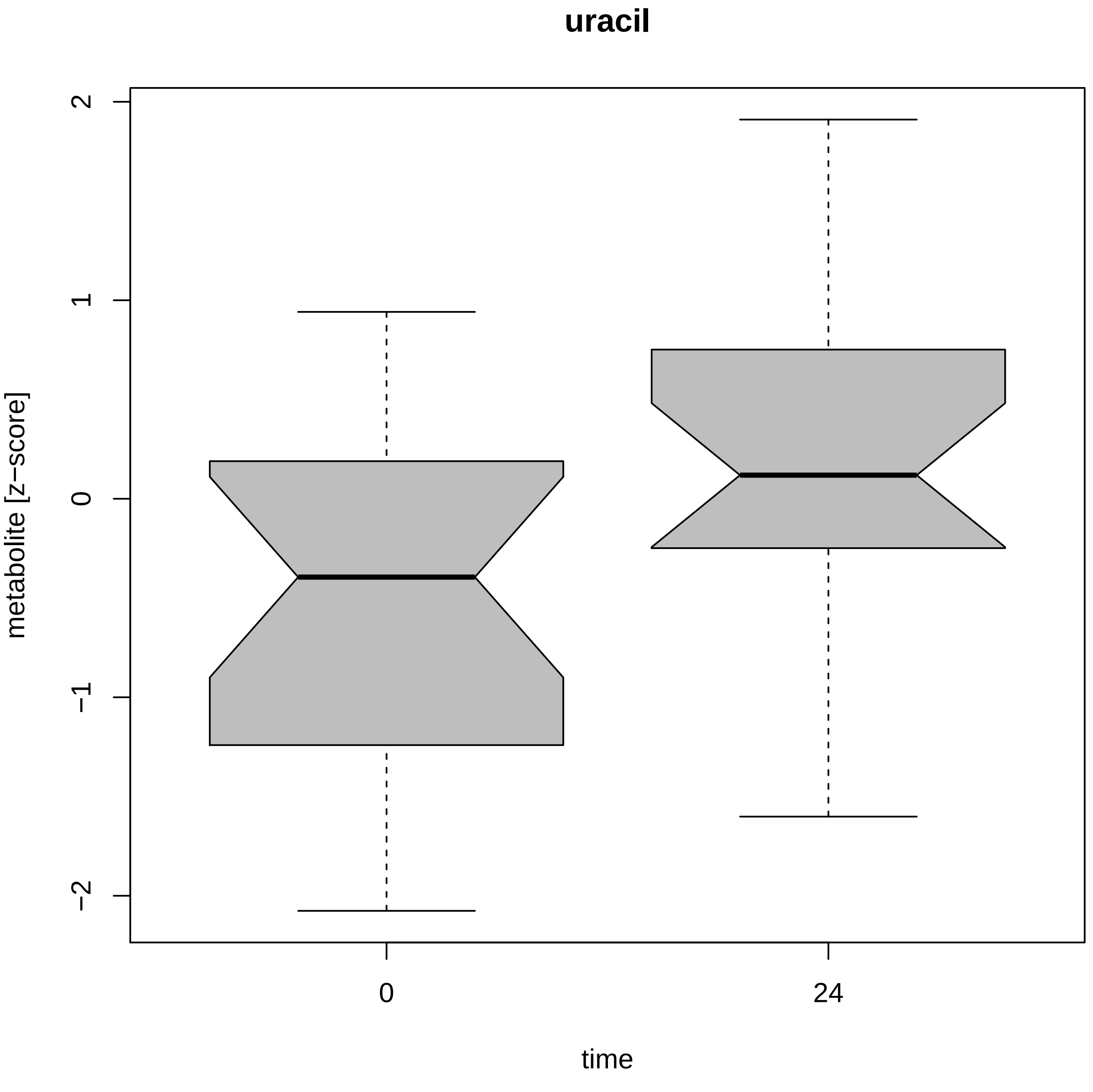
uracil
metabolite [z−score]
2
1
0
−1
−2
0
24
time

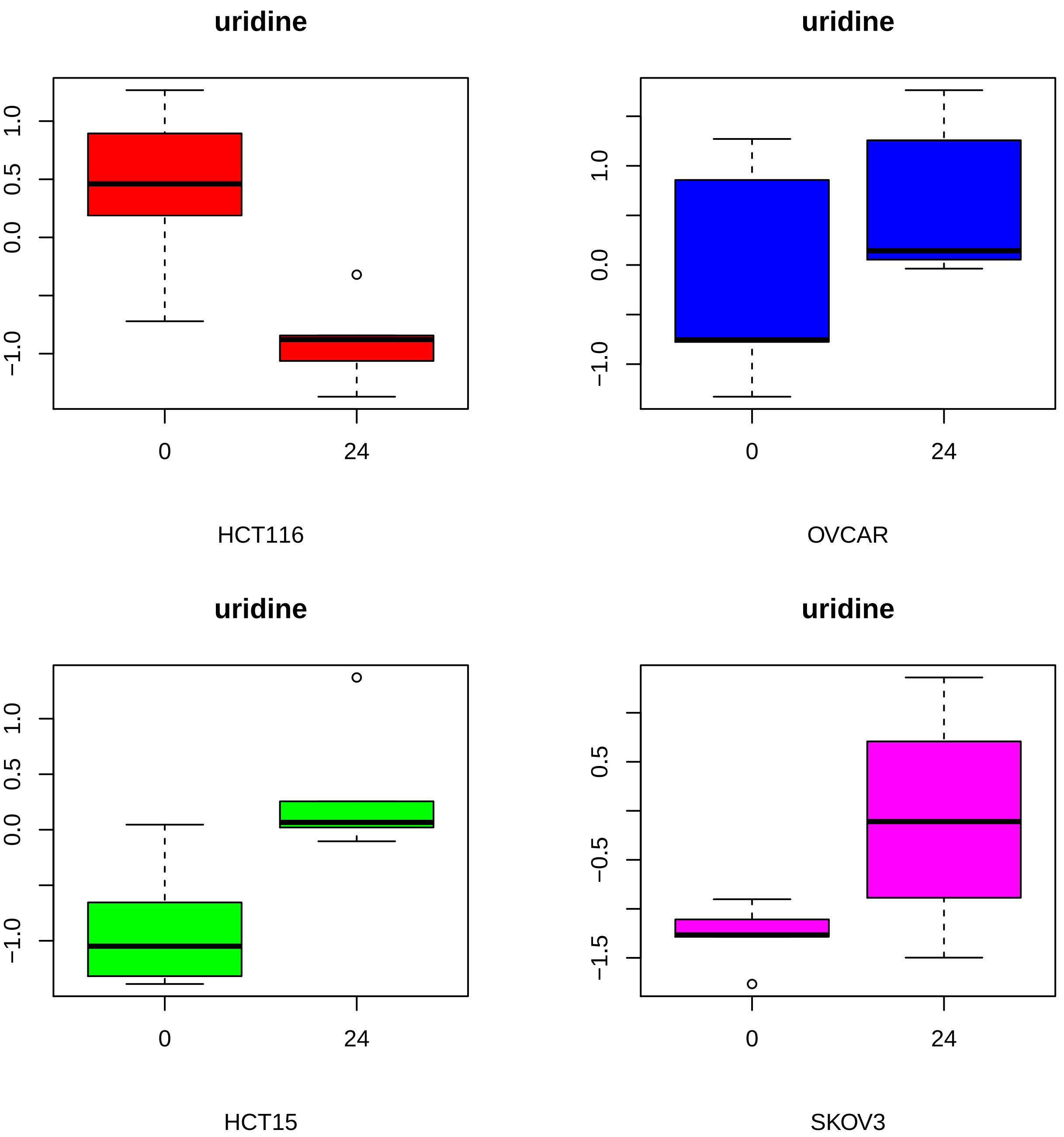
uridine
1.0
0.5
0.0
−1.0
0
24
HCT116
uridine
1.0
0.0
−1.0
0
24
OVCAR
uridine
1.0
0.5
0.0
−1.0
0
24
HCT15
uridine
0.5
−0.5
−1.5
0
24
SKOV3

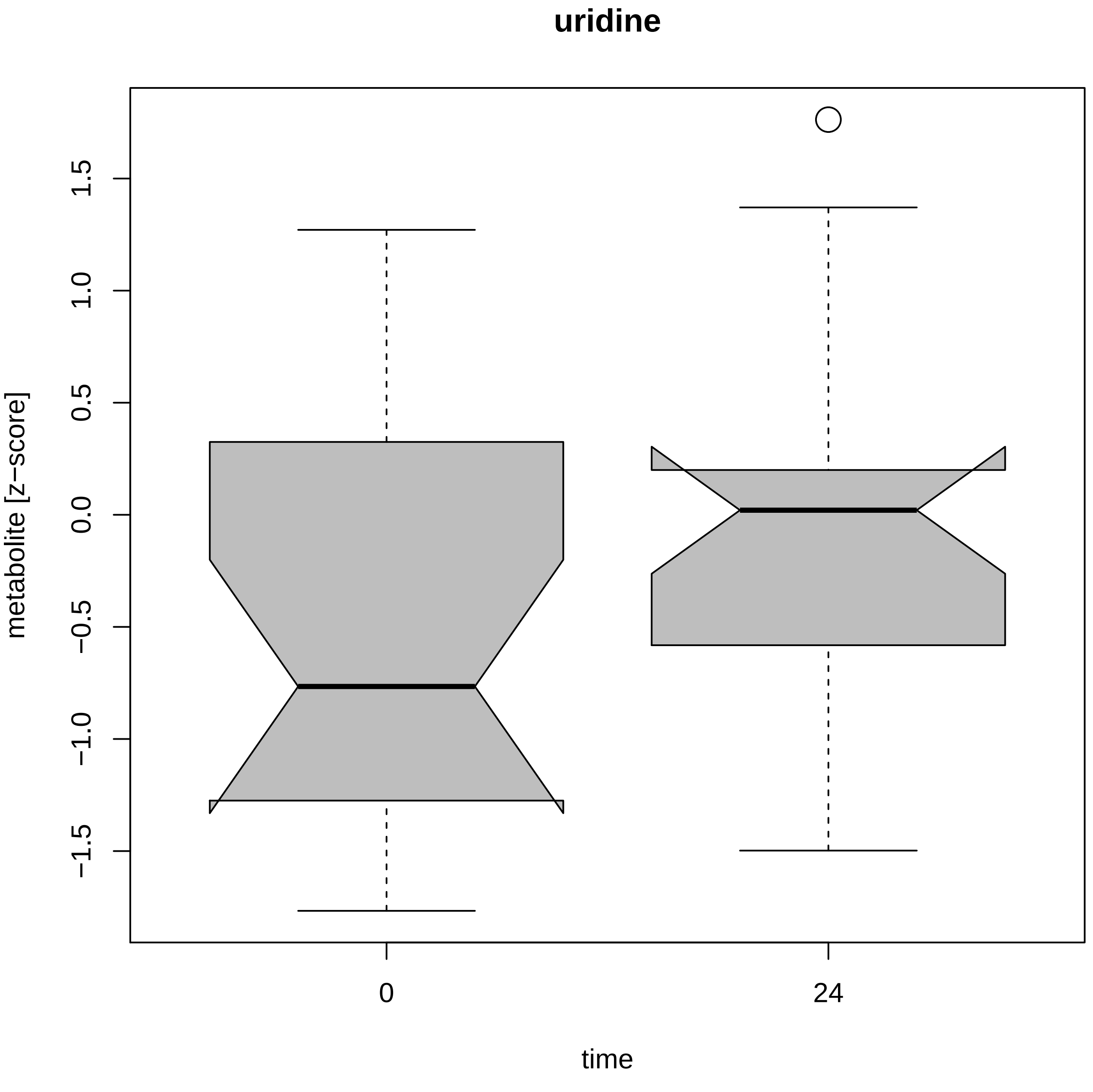

uridine
metabolite [z−score]
1.5
1.0
0.5
0.0
−0.5
−1.0
−1.5
0
24
time

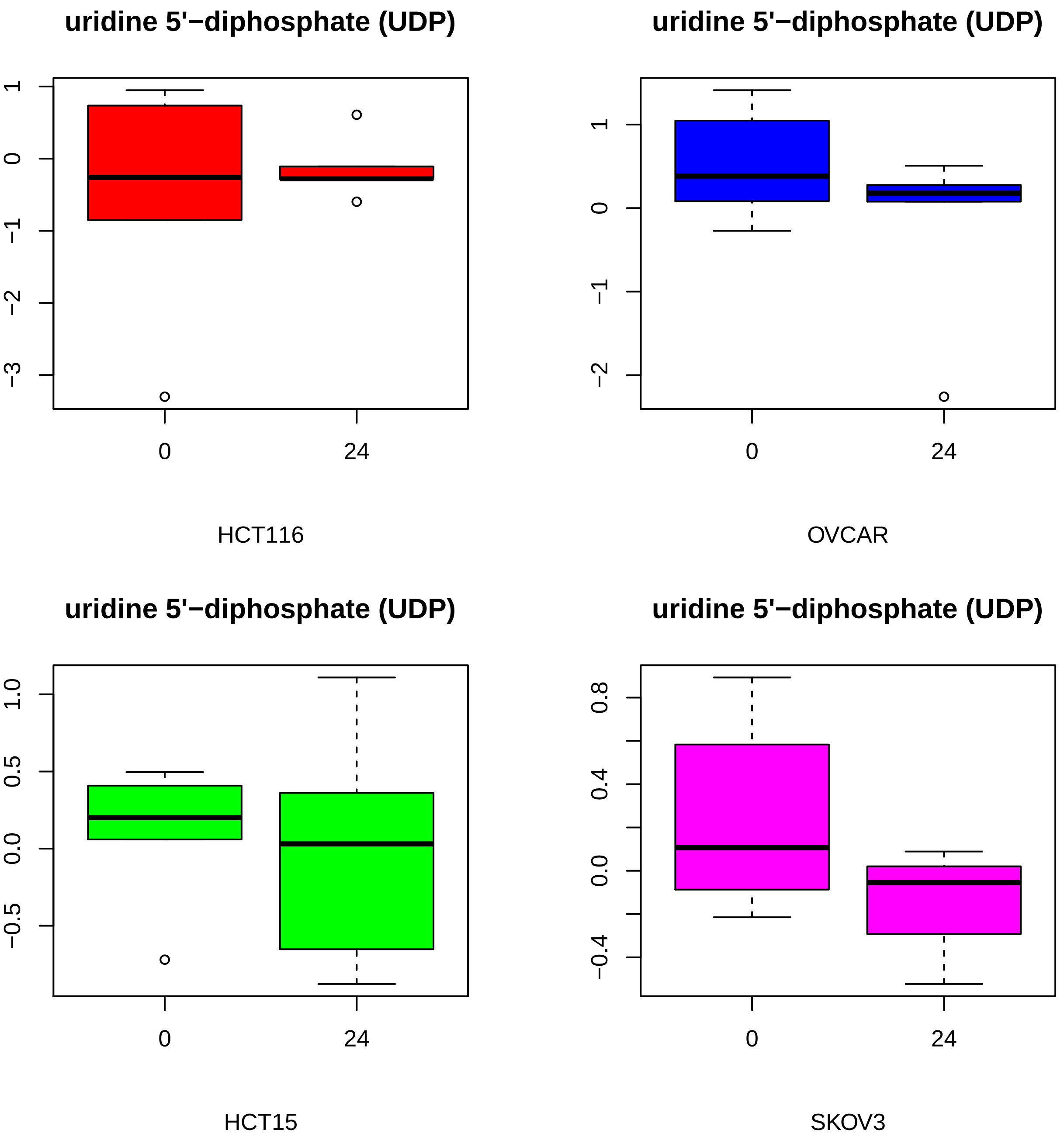

uridine 5'−diphosphate (UDP)
HCT116
OVCAR
HCT15
SKOV3
0
24

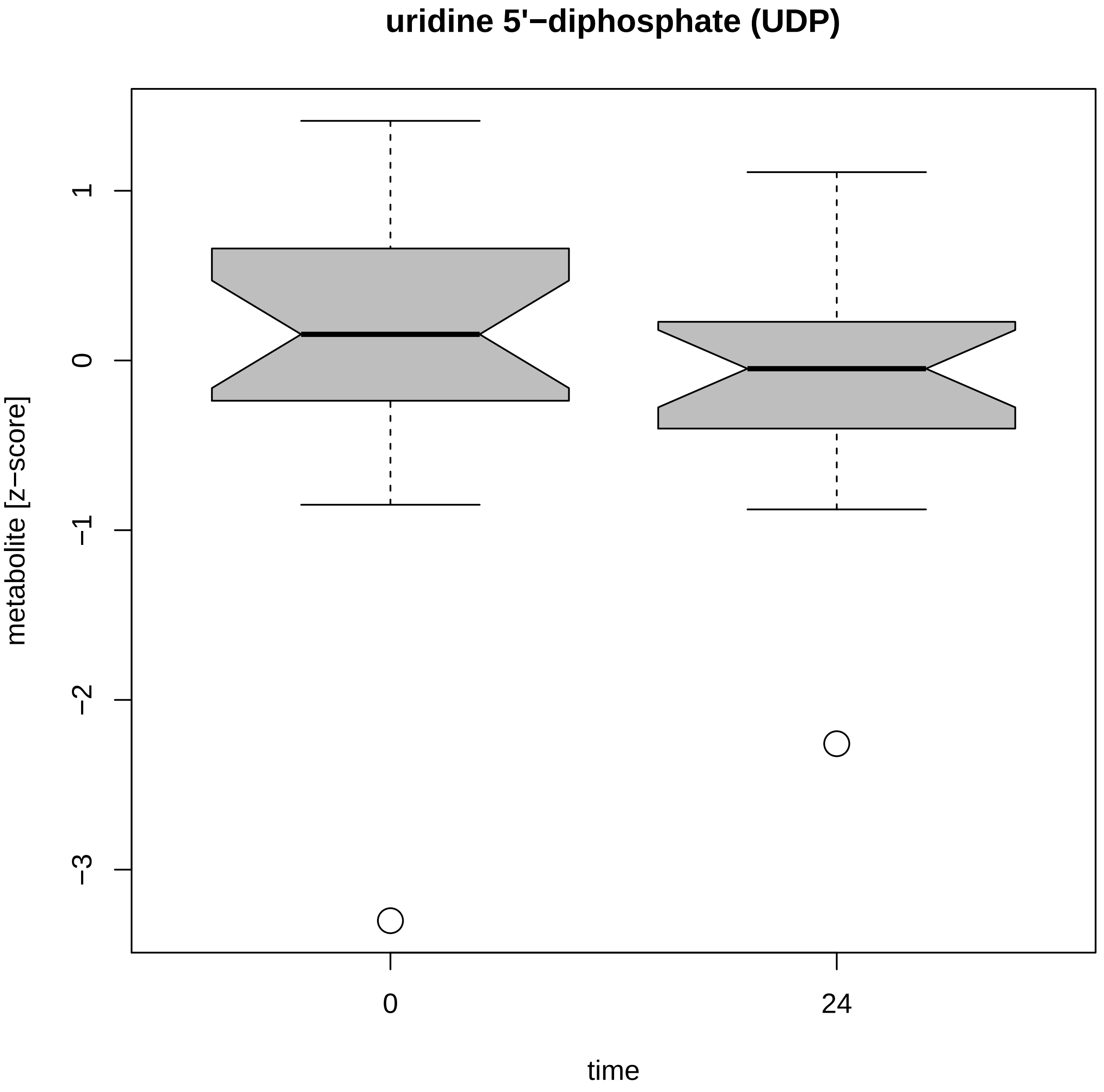
uridine 5'−diphosphate (UDP)
metabolite [z−score]
1
0
−1
−2
−3
0
24
time

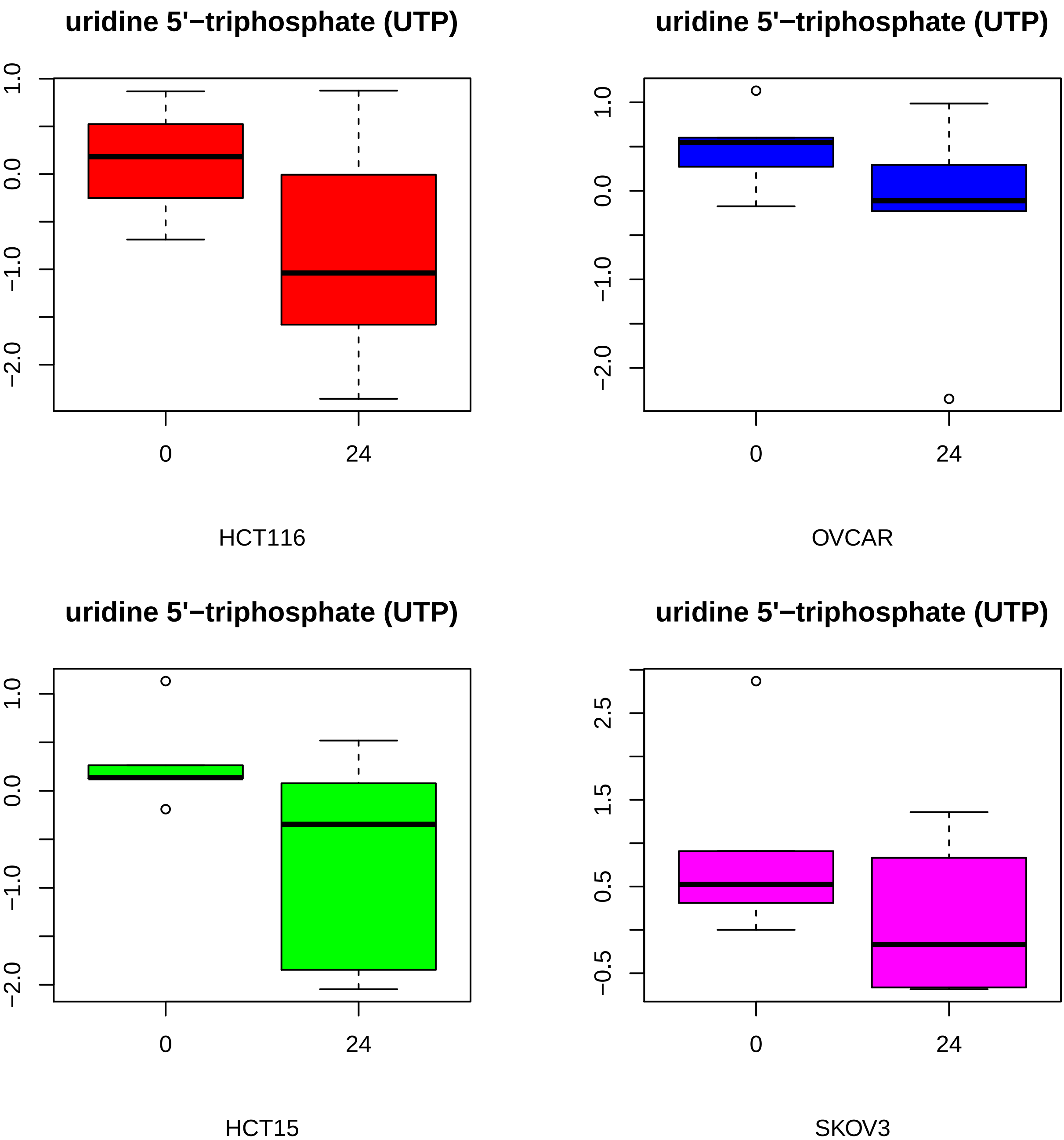
uridine 5'−triphosphate (UTP)
1.0
0.0
−1.0
−2.0
0
24
HCT116
uridine 5'−triphosphate (UTP)
1.0
0.0
−1.0
−2.0
0
24
OVCAR
uridine 5'−triphosphate (UTP)
1.0
0.0
−1.0
−2.0
0
24
HCT15
uridine 5'−triphosphate (UTP)
2.5
1.5
0.5
−0.5
0
24
SKOV3

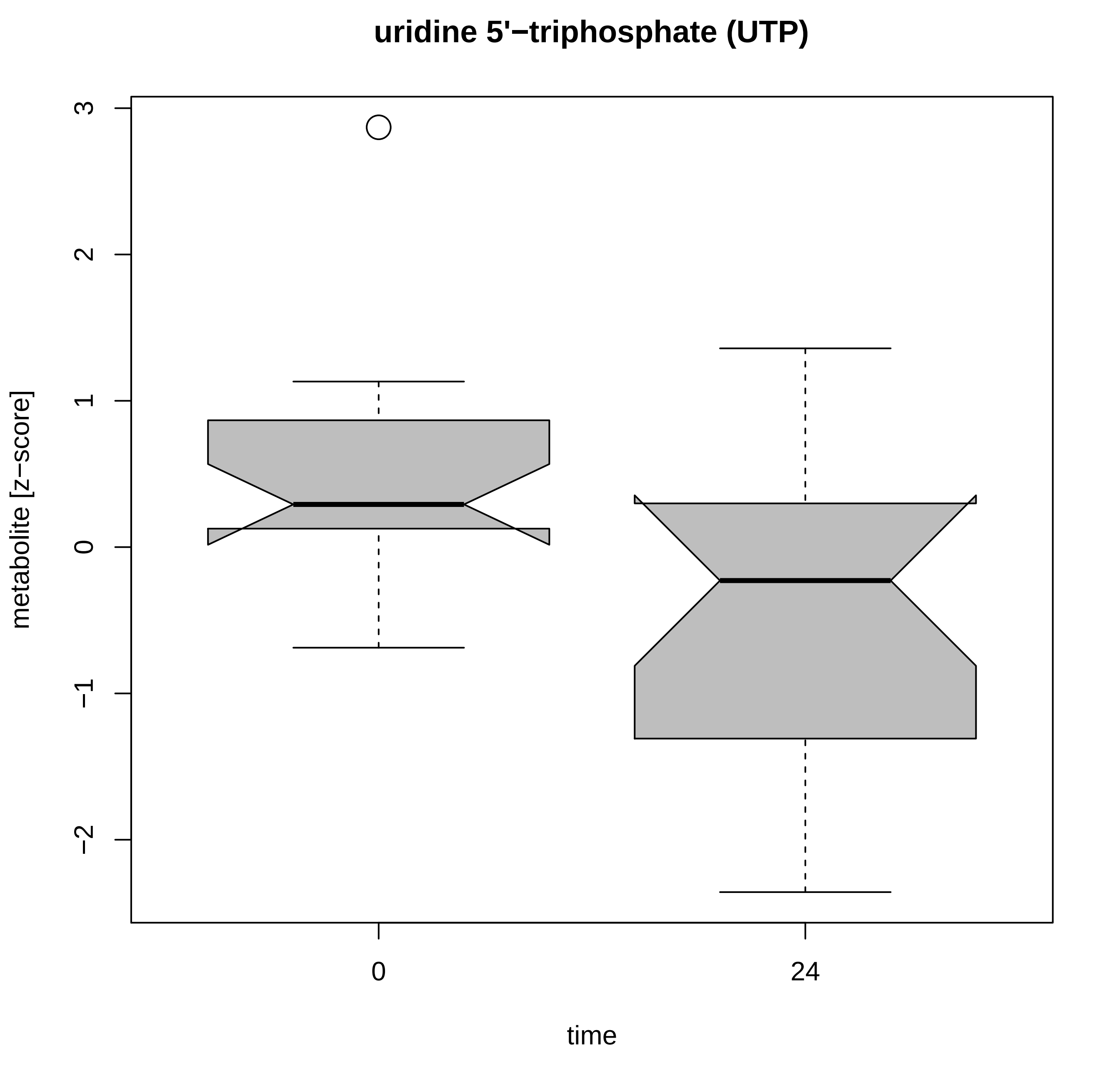
uridine 5'−triphosphate (UTP)
metabolite [z−score]
3
2
1
0
−1
−2
0
24
time

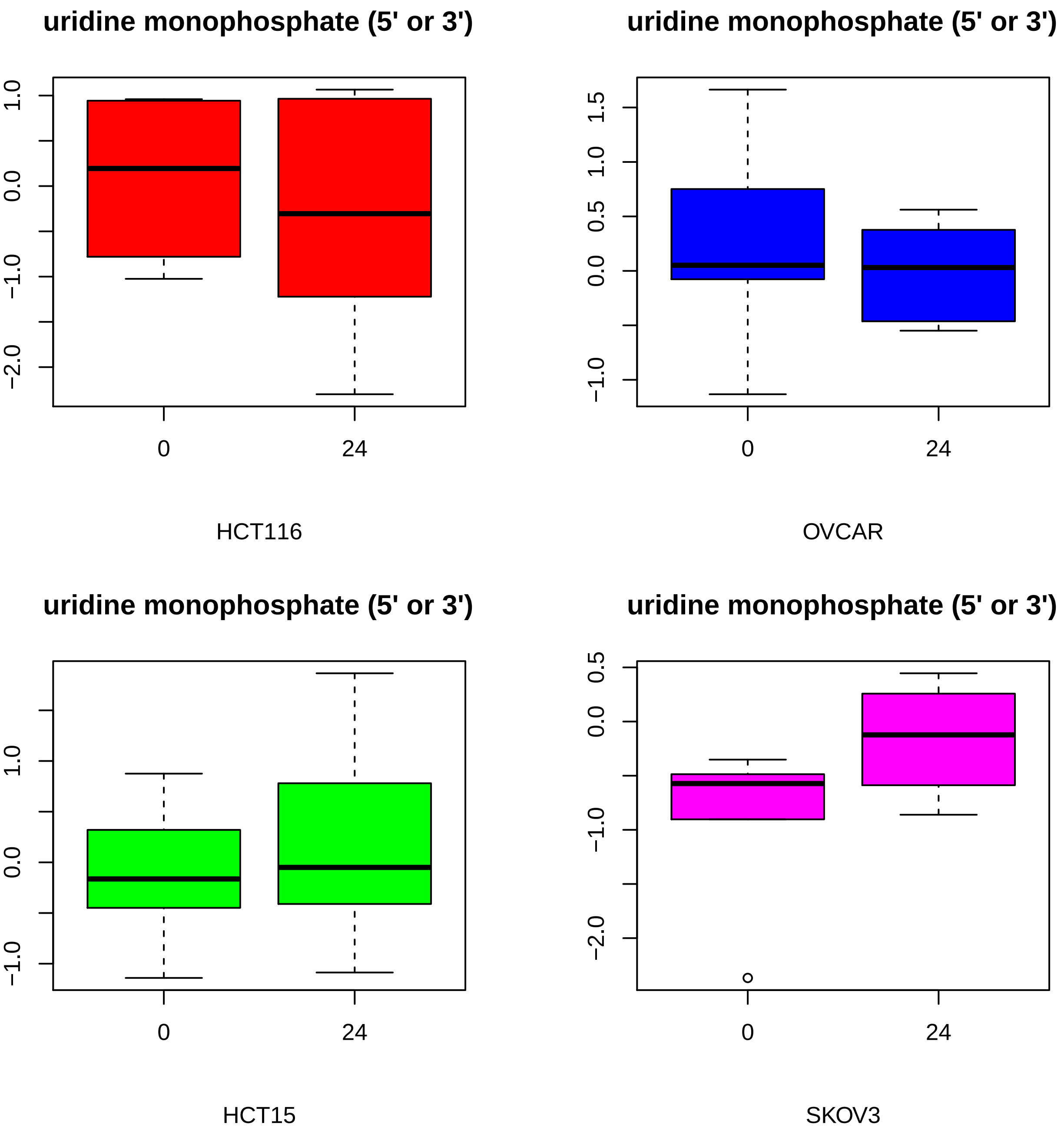
uridine monophosphate (5' or 3')
1.0
0.0
−1.0
−2.0
0
24
HCT116
uridine monophosphate (5' or 3')
1.5
1.0
0.5
0.0
−1.0
0
24
OVCAR
uridine monophosphate (5' or 3')
1.0
0.0
−1.0
0
24
HCT15
uridine monophosphate (5' or 3')
0.5
0.0
−1.0
−2.0
0
24
SKOV3

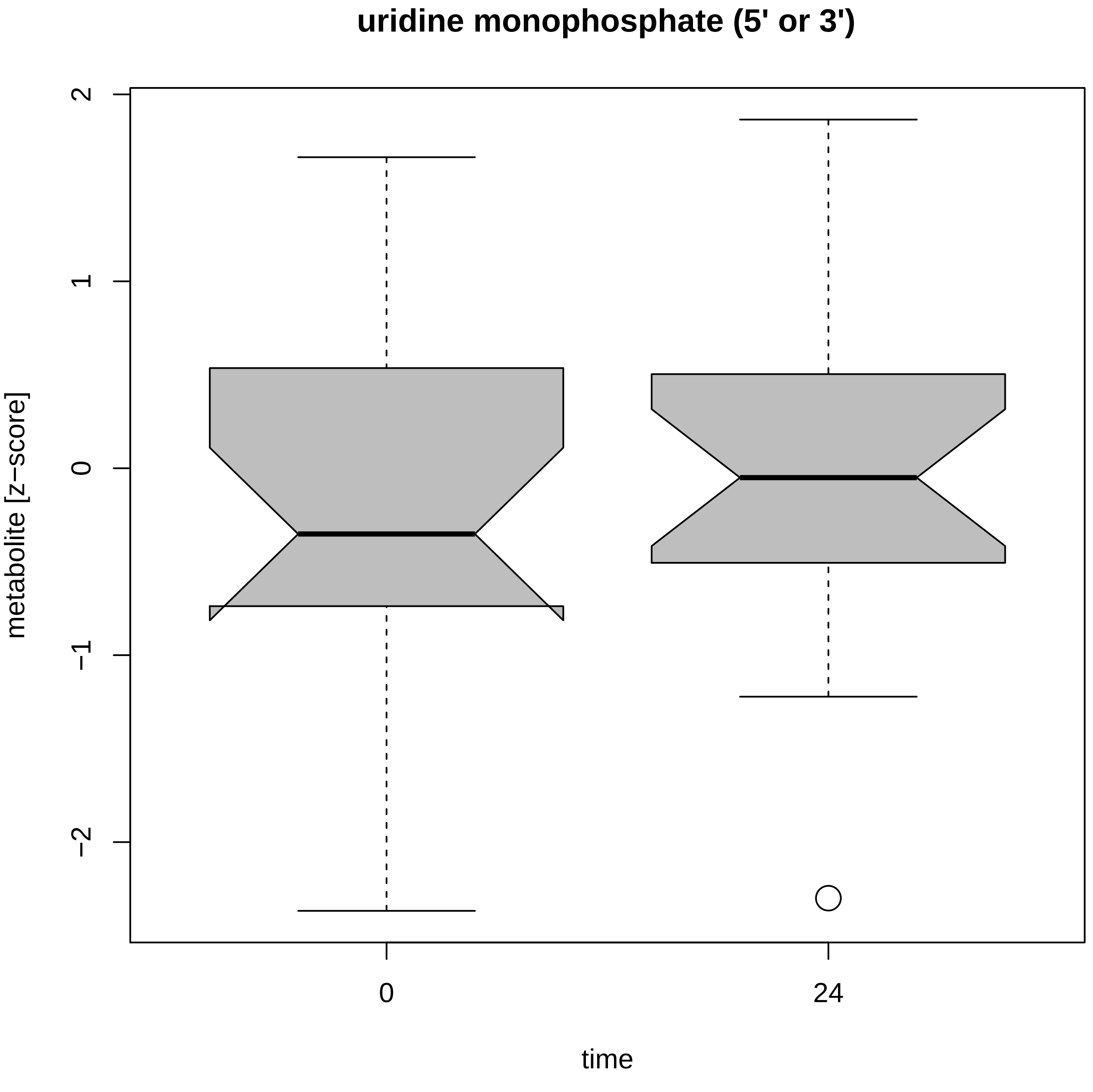

uridine monophosphate (5' or 3')
metabolite [z−score]
2
1
0
−1
−2
0
24
time

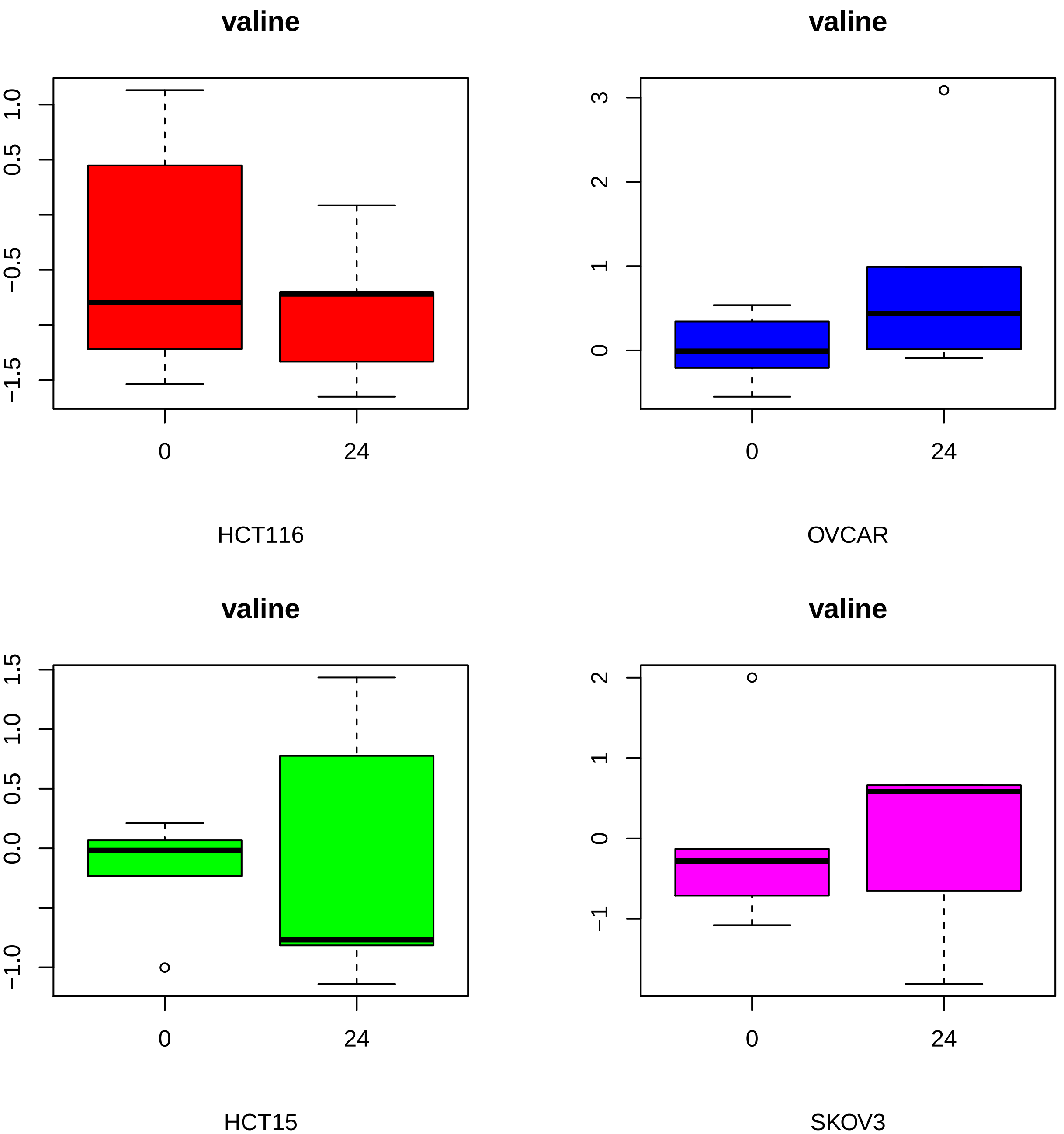

valine
1.0
0.5
−0.5
−1.5
0
24
HCT116
valine
3
2
1
0
0
24
OVCAR
valine
1.5
1.0
0.5
0.0
−1.0
0
24
HCT15
valine
2
1
0
−1
0
24
SKOV3

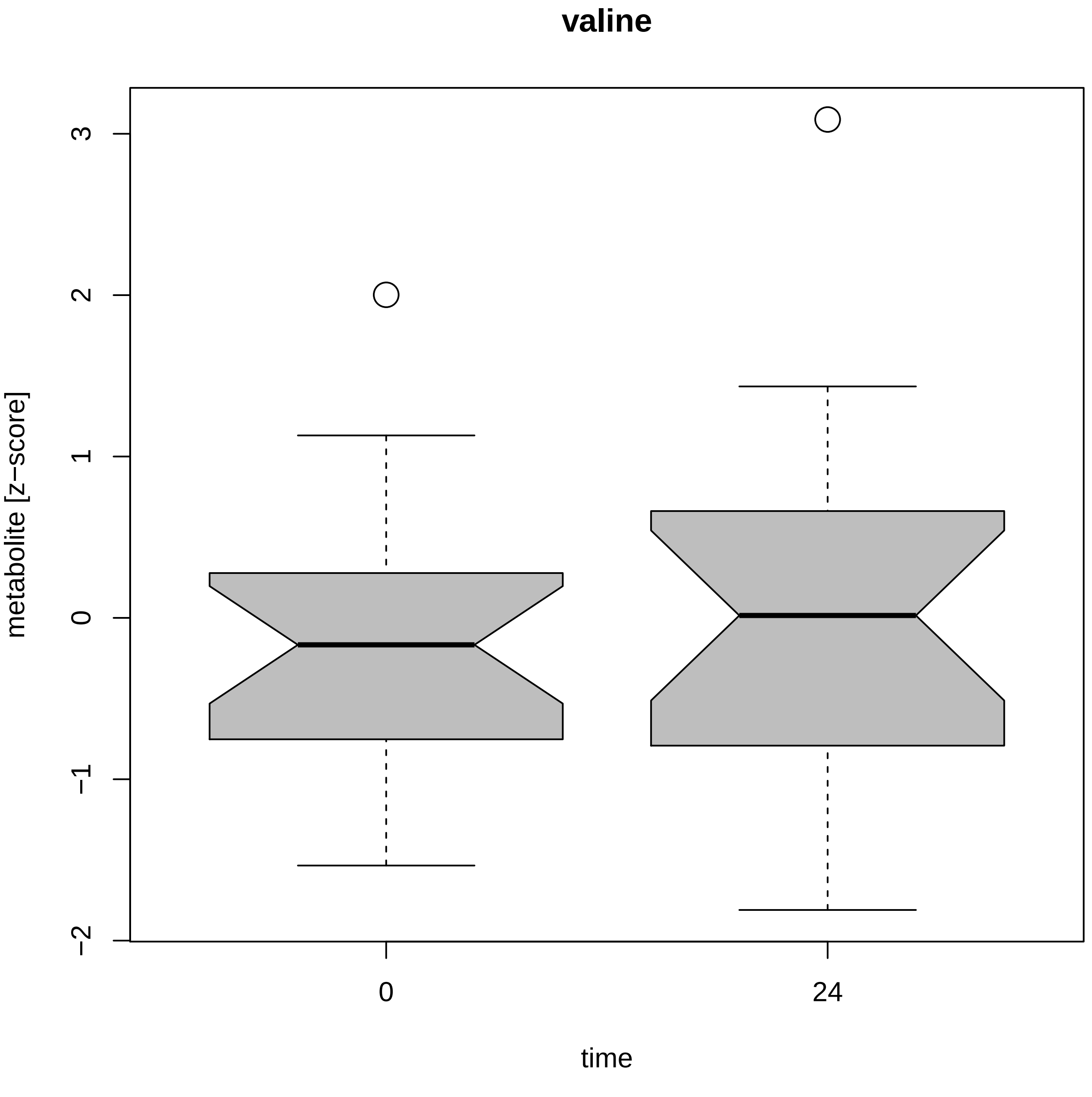
valine
metabolite [z−score]
3
2
1
0
−1
−2
0
24
time

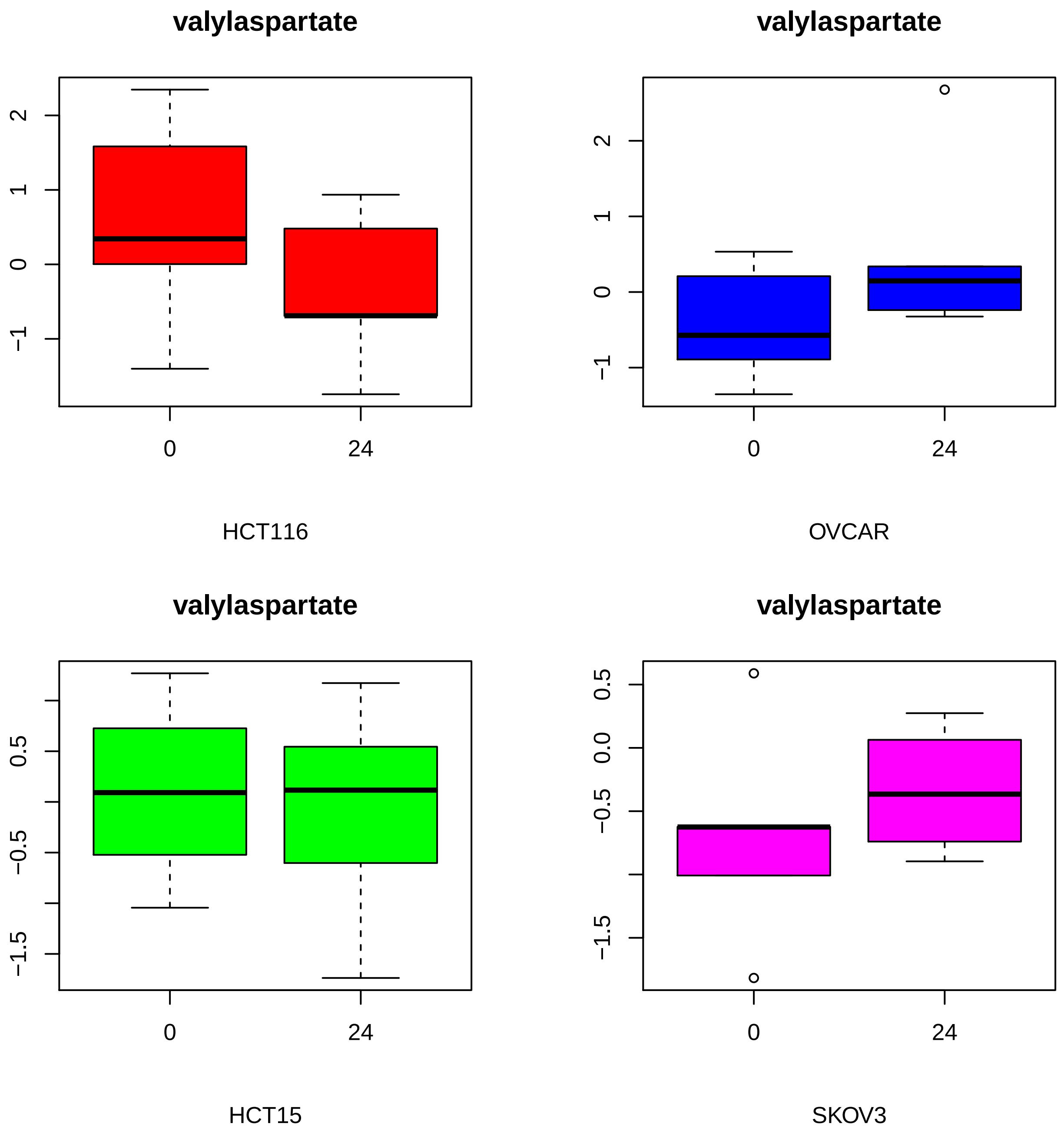
valylaspartate
2
1
0
−1
0
24
HCT116
valylaspartate
2
1
0
−1
0
24
OVCAR
valylaspartate
0.5
−0.5
−1.5
0
24
HCT15
valylaspartate
0.5
0.0
−0.5
−1.5
0
24
SKOV3

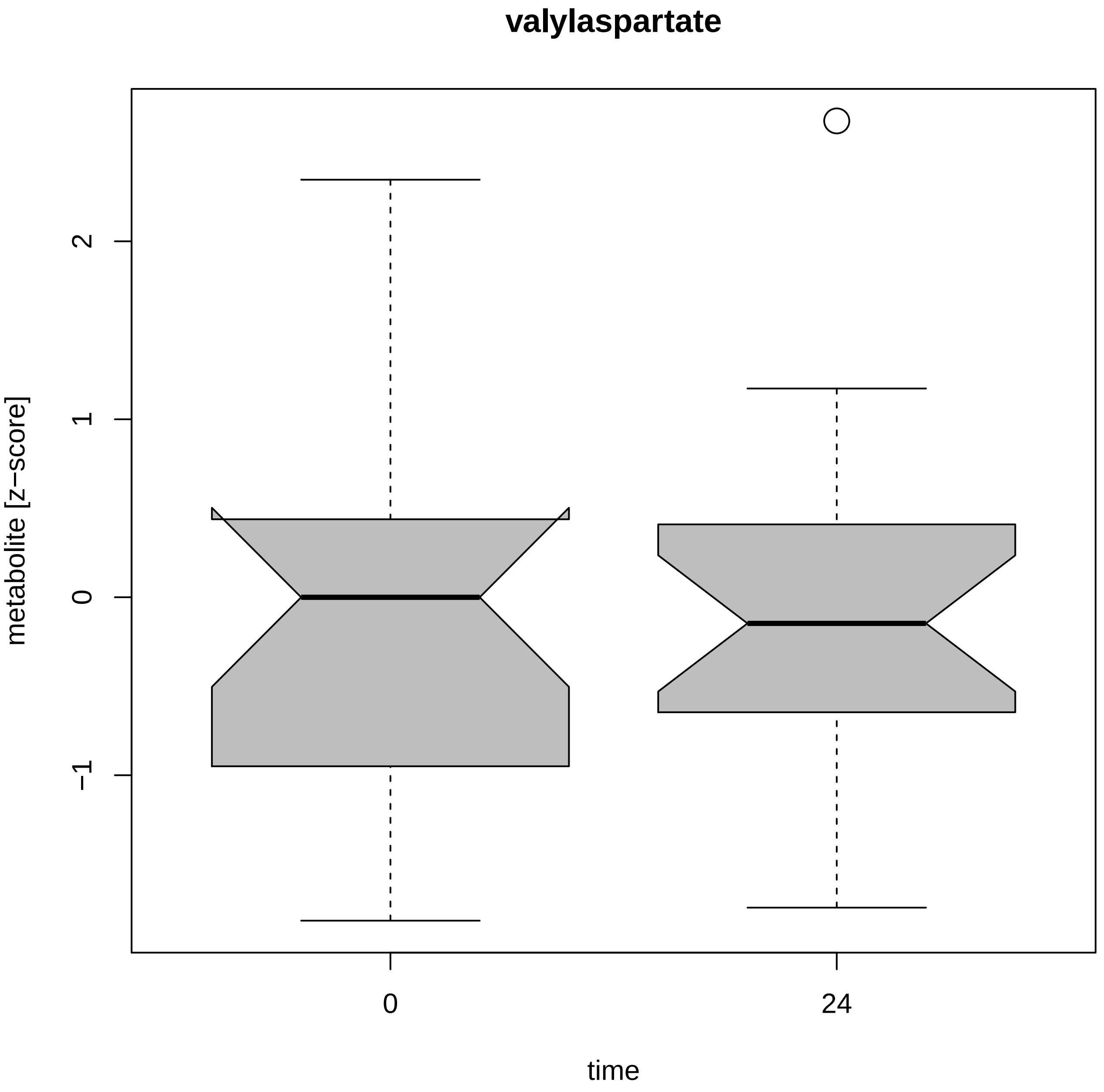

valylaspartate
metabolite [z−score]
2
1
0
−1
0
24
time

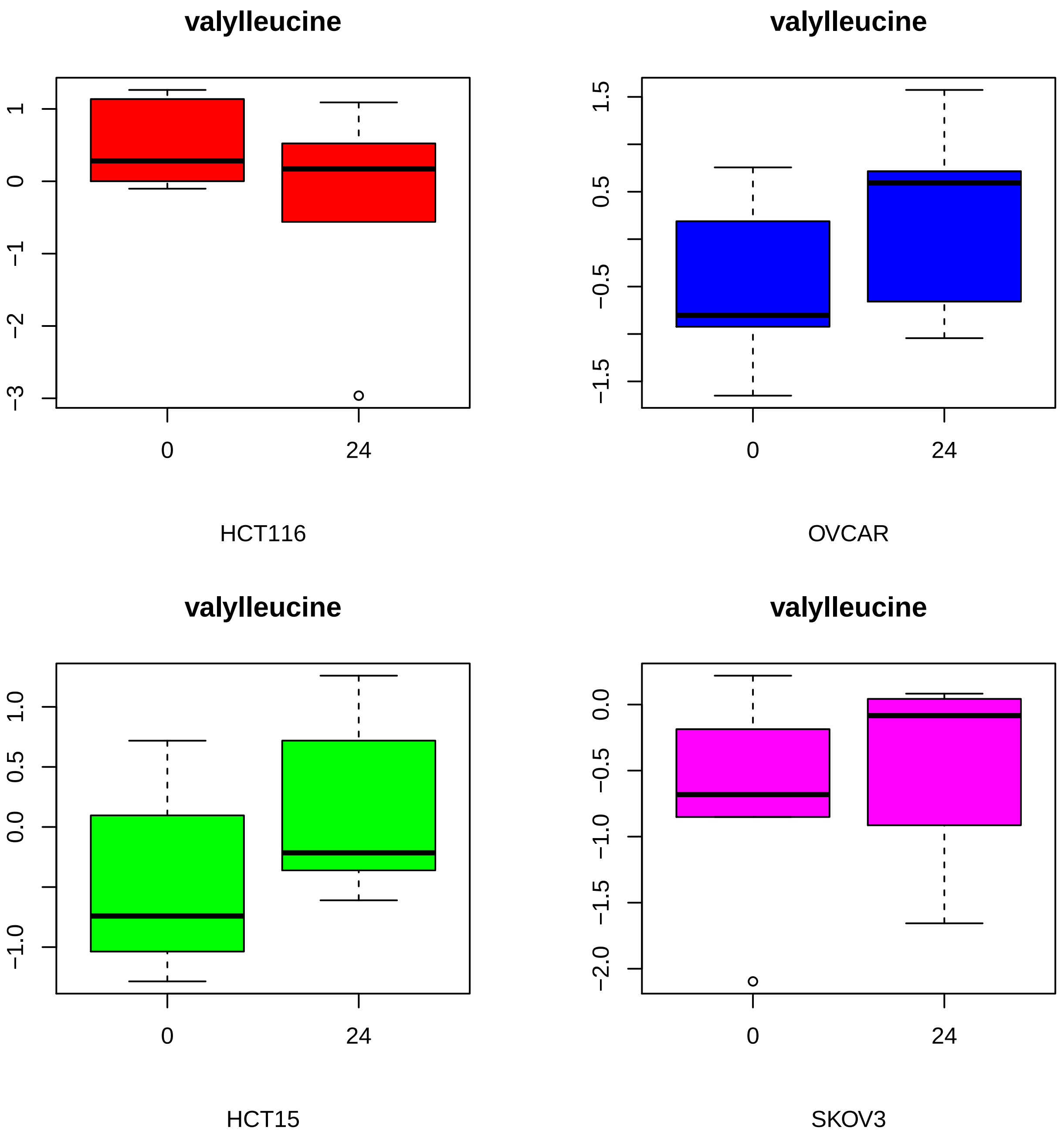
valylleucine
1
0
−1
−2
−3
0
24
HCT116
valylleucine
1.5
0.5
−0.5
−1.5
0
24
OVCAR
valylleucine
1.0
0.5
0.0
−1.0
0
24
HCT15
valylleucine
0.0
−0.5
−1.0
−1.5
−2.0
0
24
SKOV3

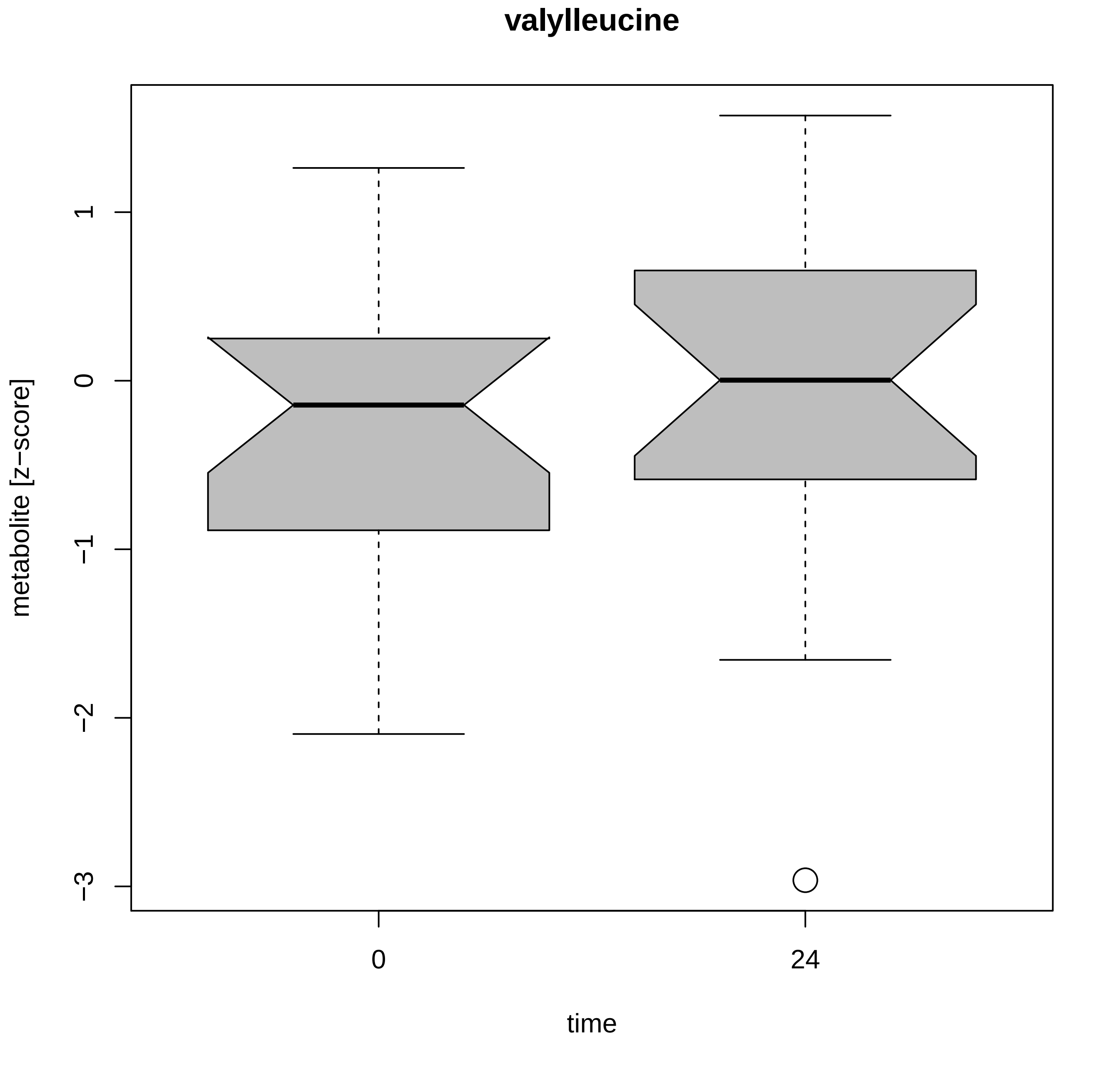
valylleucine
metabolite [z−score]
1
0
−1
−2
−3
0
24
time

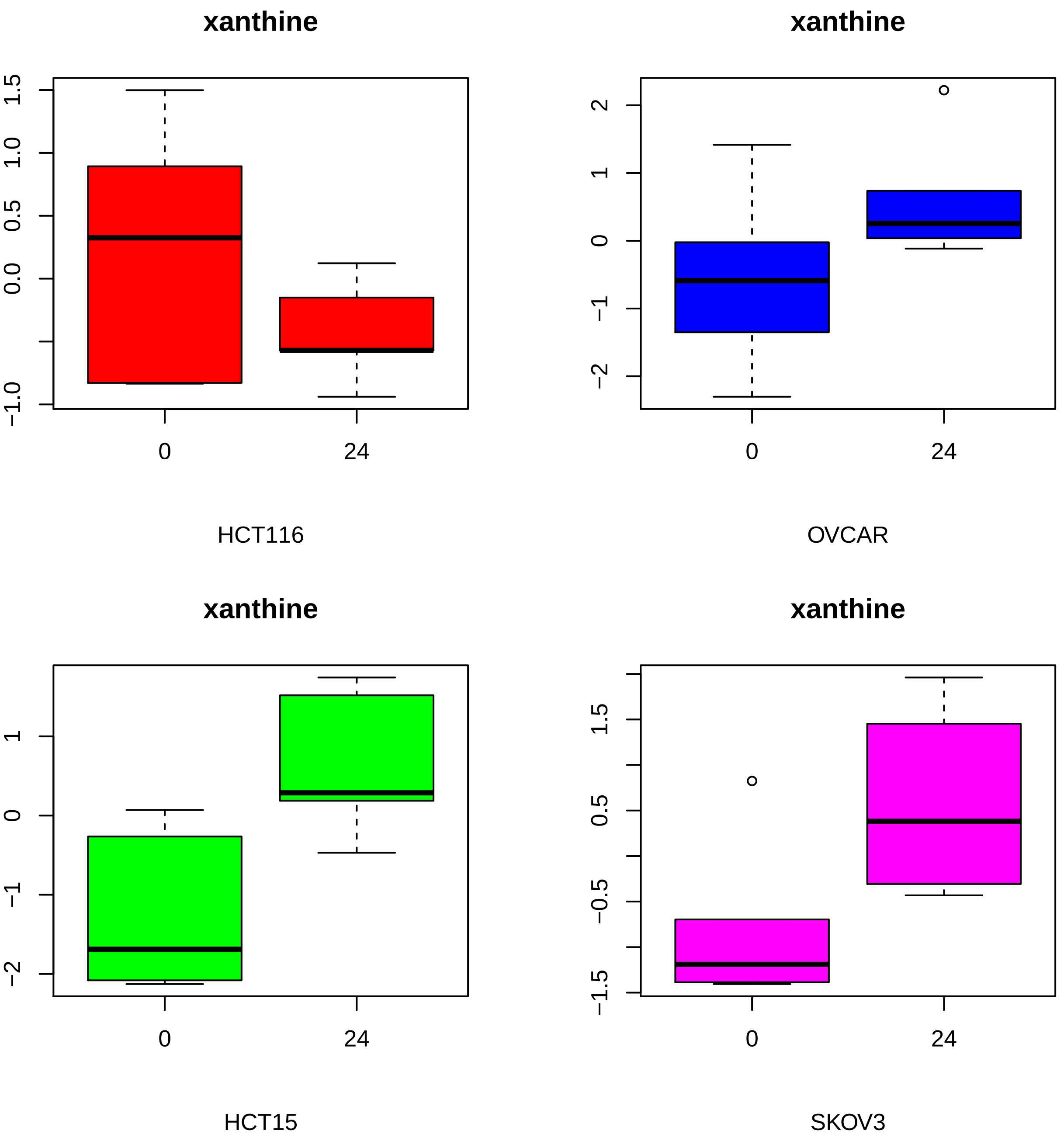
xanthine
1.5
1.0
0.5
0.0
−1.0
0
24
HCT116
xanthine
2
1
0
−1
−2
0
24
OVCAR
xanthine
1
0
−1
−2
0
24
HCT15
xanthine
1.5
0.5
−0.5
−1.5
0
24
SKOV3

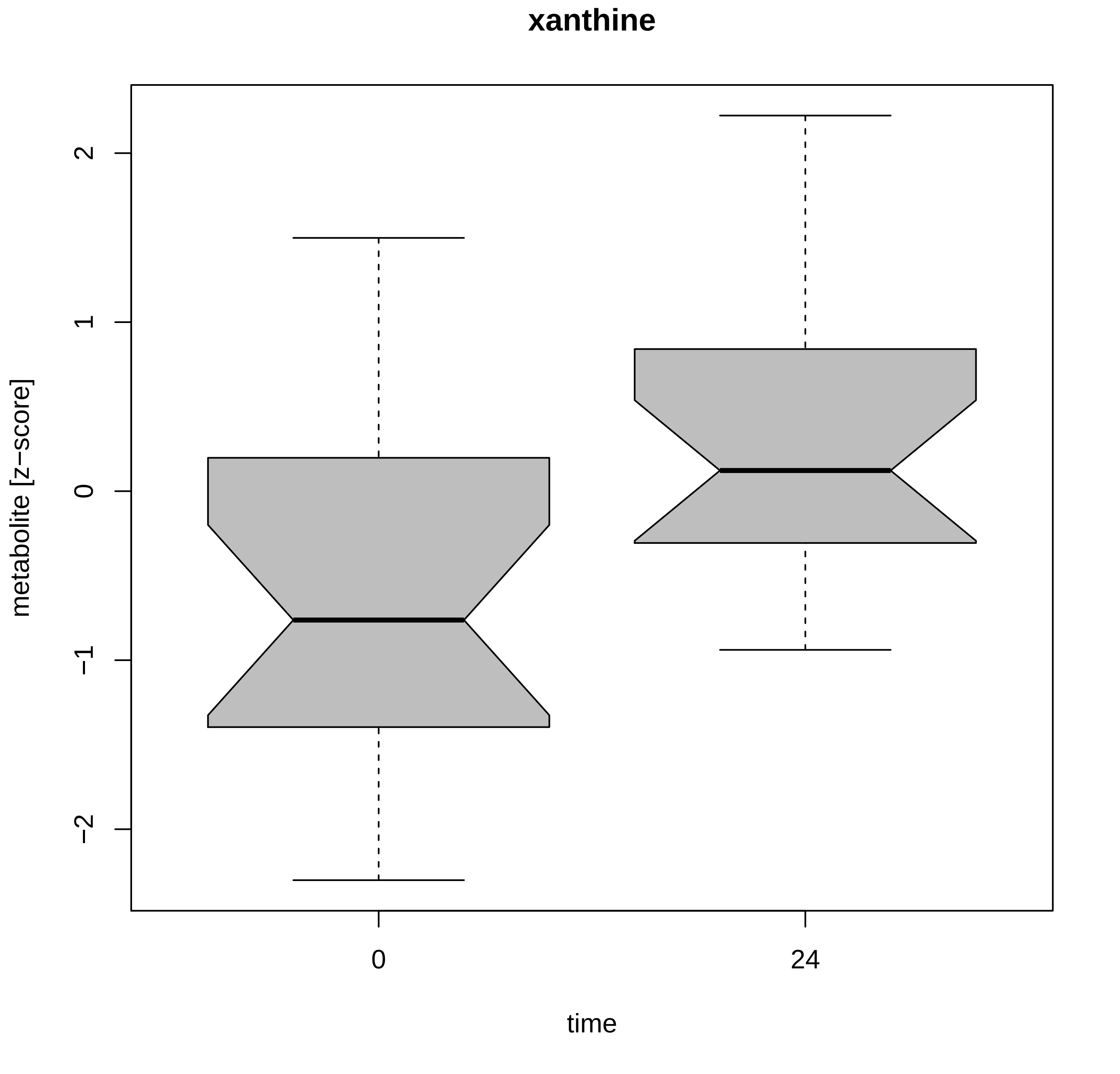
xanthine
metabolite [z−score]
2
1
0
−1
−2
0
24
time

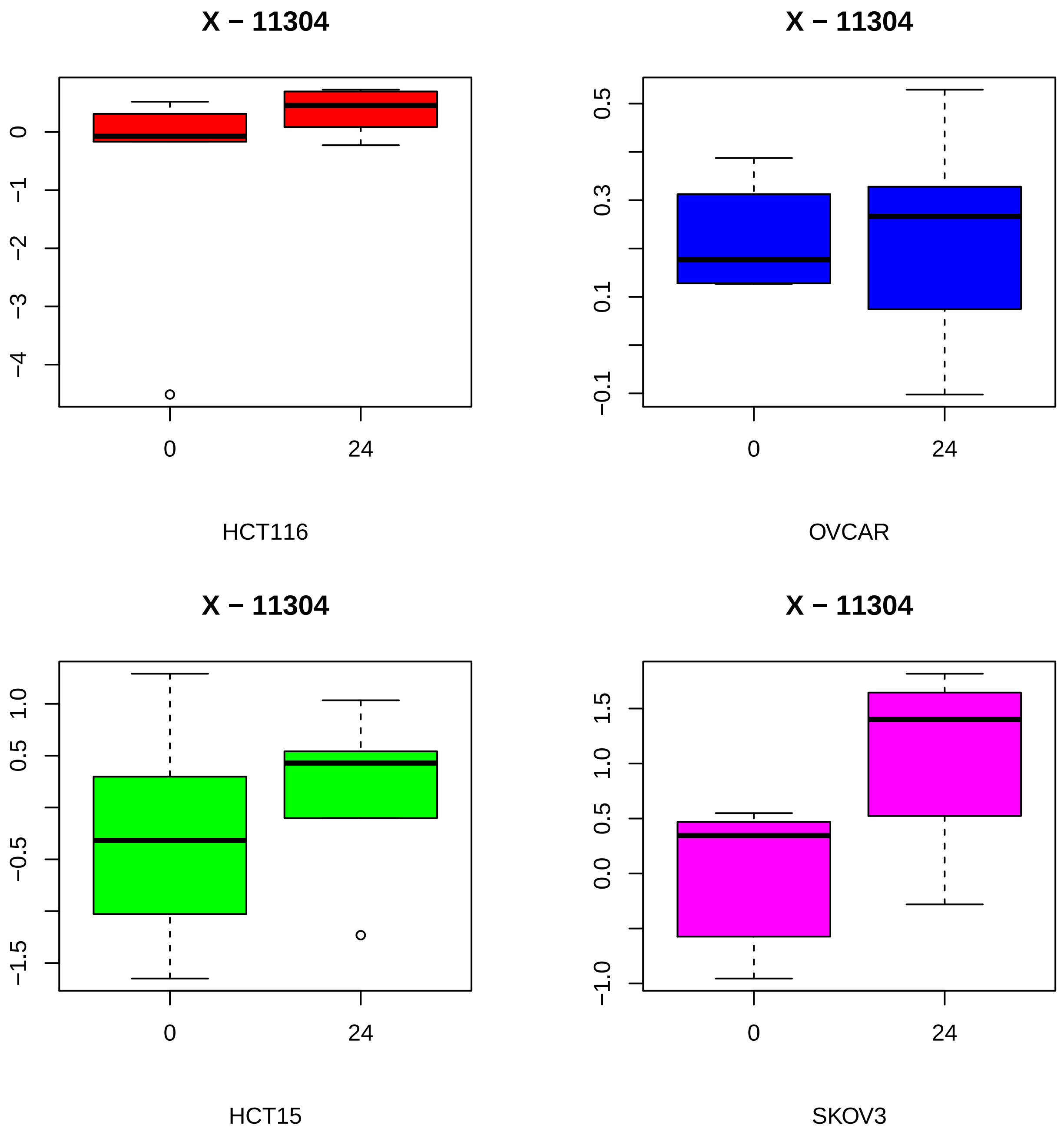

X − 11304
0
−1
−2
−3
−4
0
24
HCT116
X − 11304
0.5
0.3
0.1
−0.1
0
24
OVCAR
X − 11304
1.0
0.5
−0.5
−1.5
0
24
HCT15
X − 11304
1.5
1.0
0.5
0.0
−1.0
0
24
SKOV3

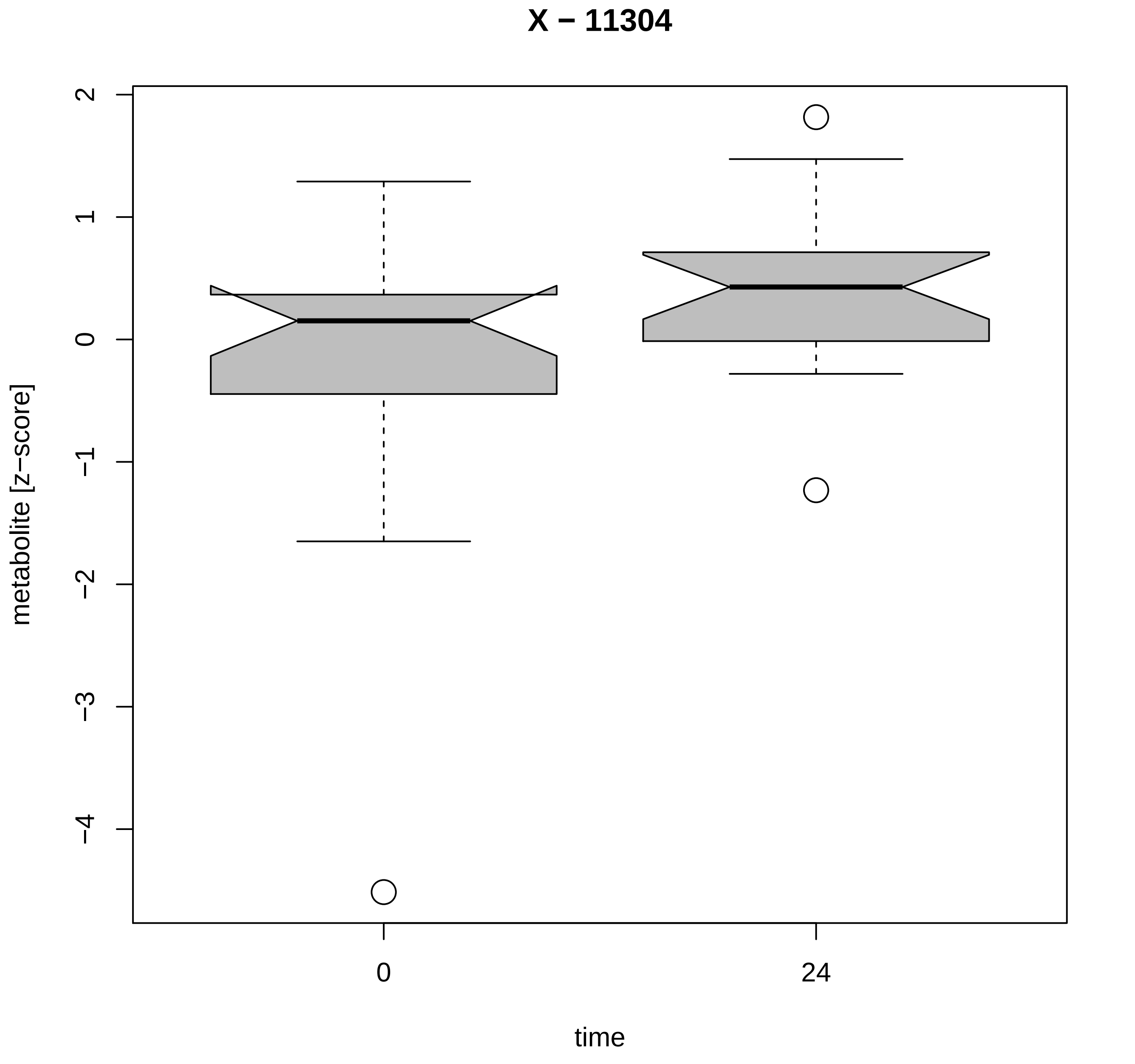
X − 11304
metabolite [z−score]
2
1
0
−1
−2
−3
−4
0
24
time

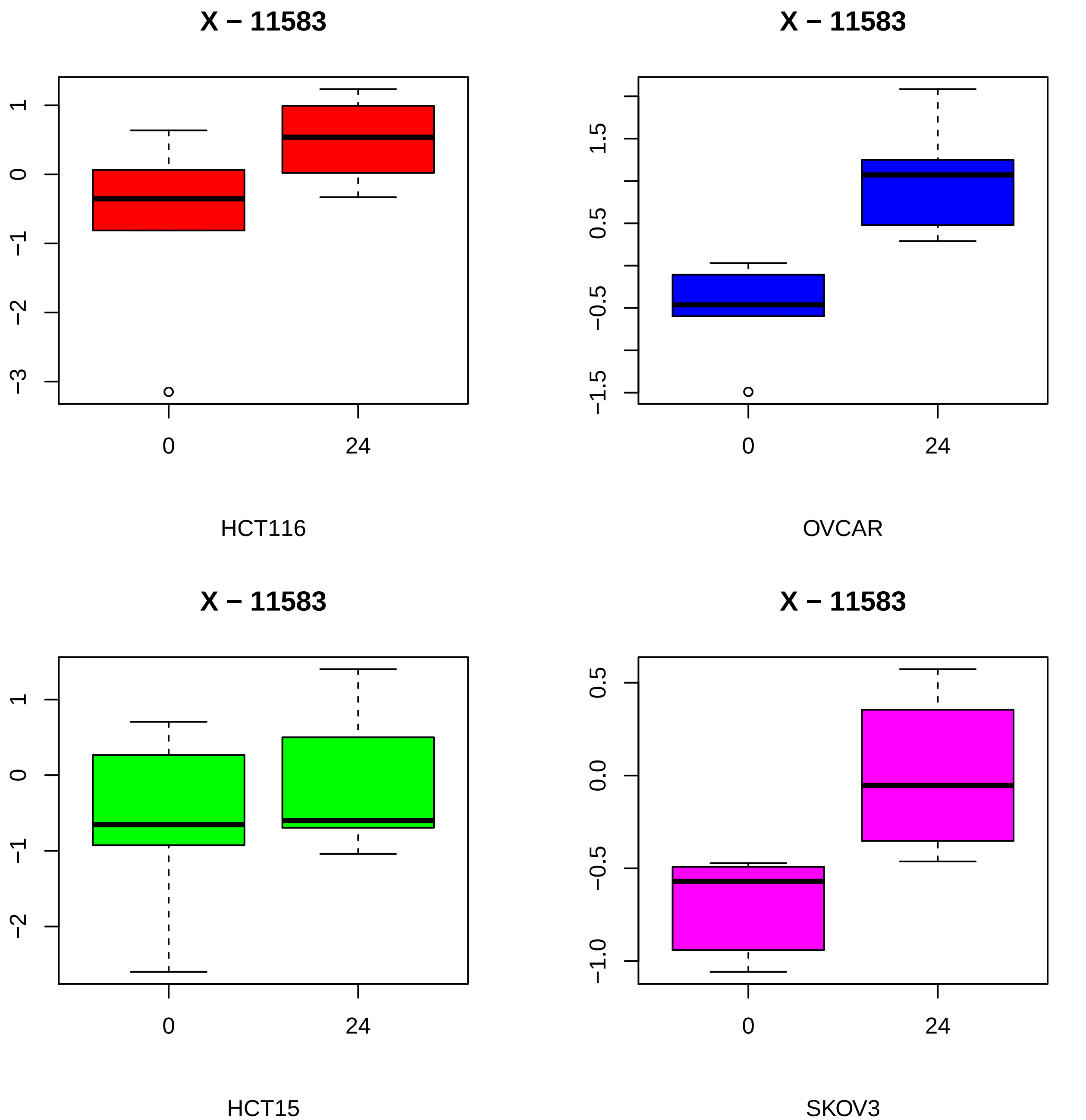
X − 11583
HCT116
OVCAR
HCT15
SKOV3
0
24

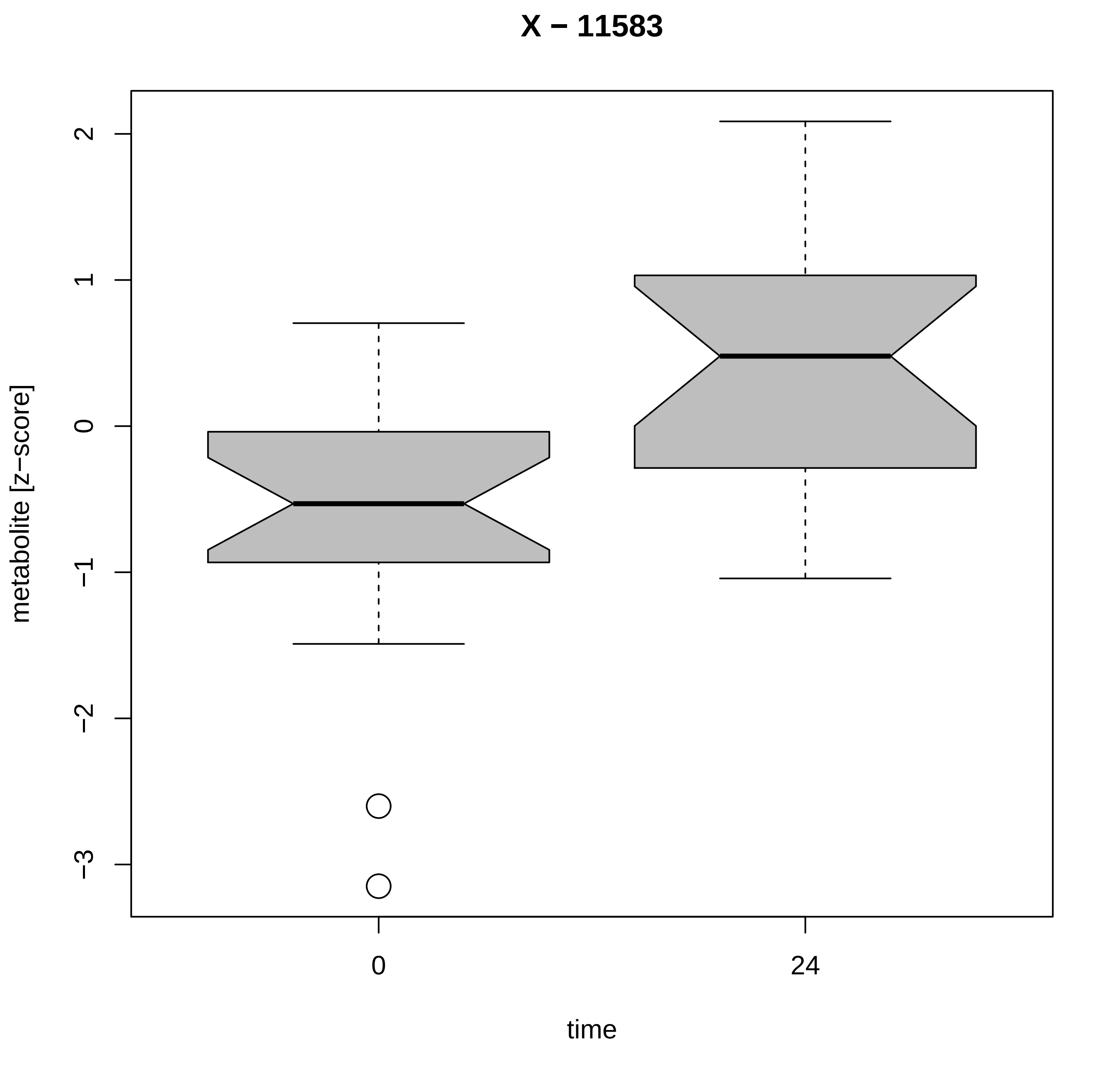

X − 11583
2
1
0
−1
−2
−3
metabolite [z−score]
0
24
time

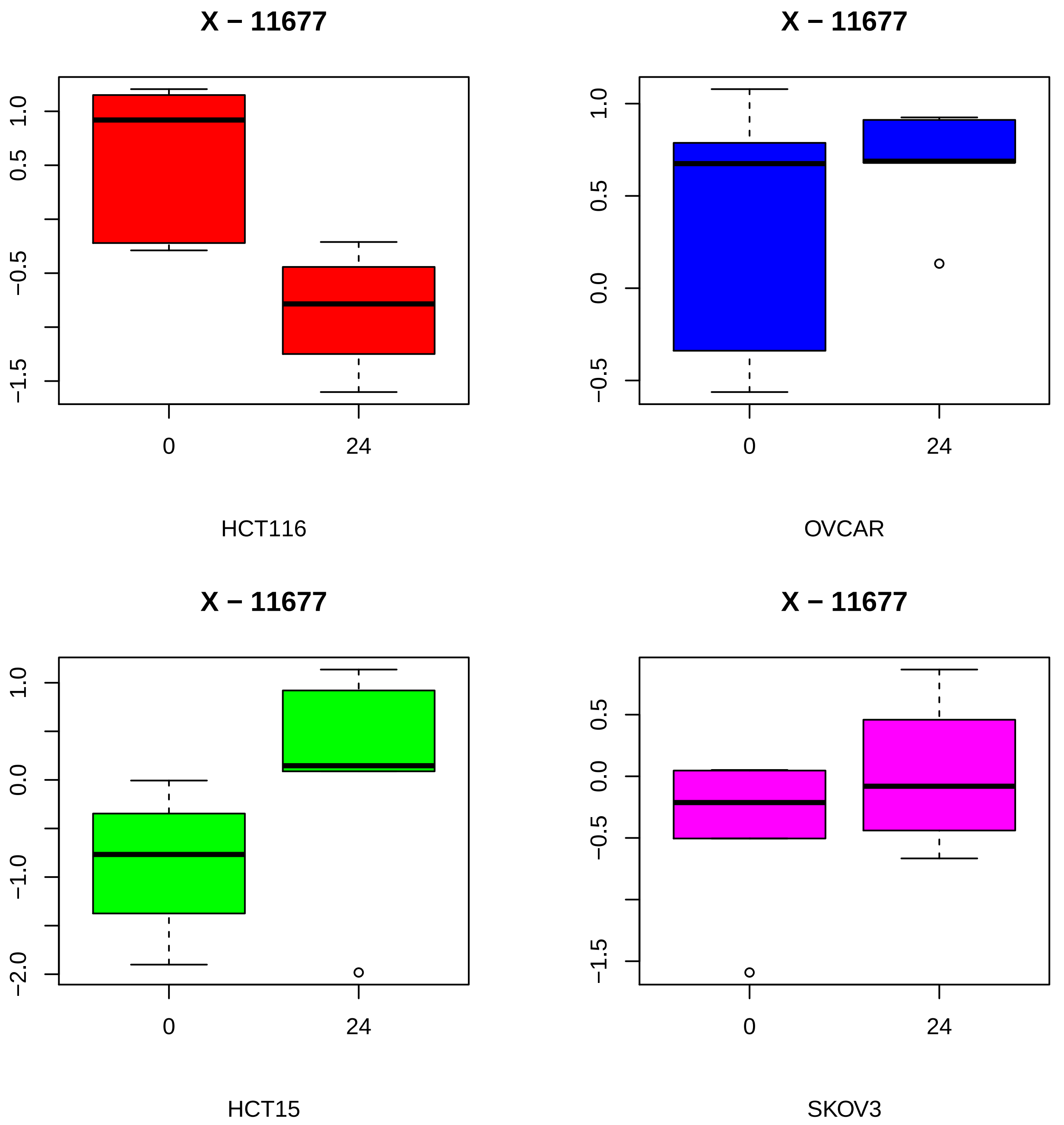
X − 11677
1.0
0.5
−0.5
−1.5
0
24
HCT116
X − 11677
1.0
0.5
0.0
−0.5
0
24
OVCAR
X − 11677
1.0
0.0
−1.0
−2.0
0
24
HCT15
X − 11677
0.5
0.0
−0.5
−1.5
0
24
SKOV3

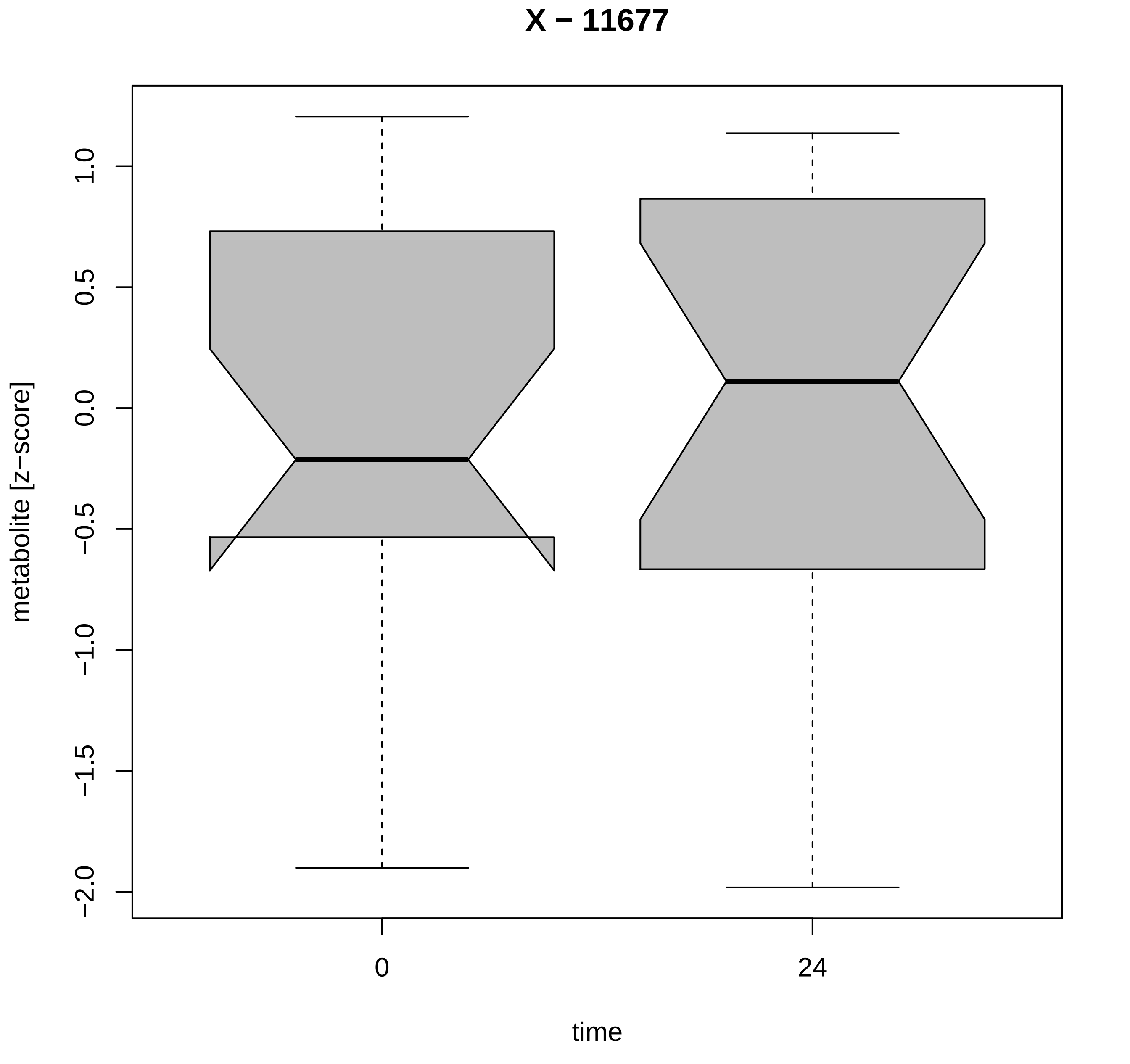

X − 11677
metabolite [z−score]
1.0
0.5
0.0
−0.5
−1.0
−1.5
−2.0
0
24
time

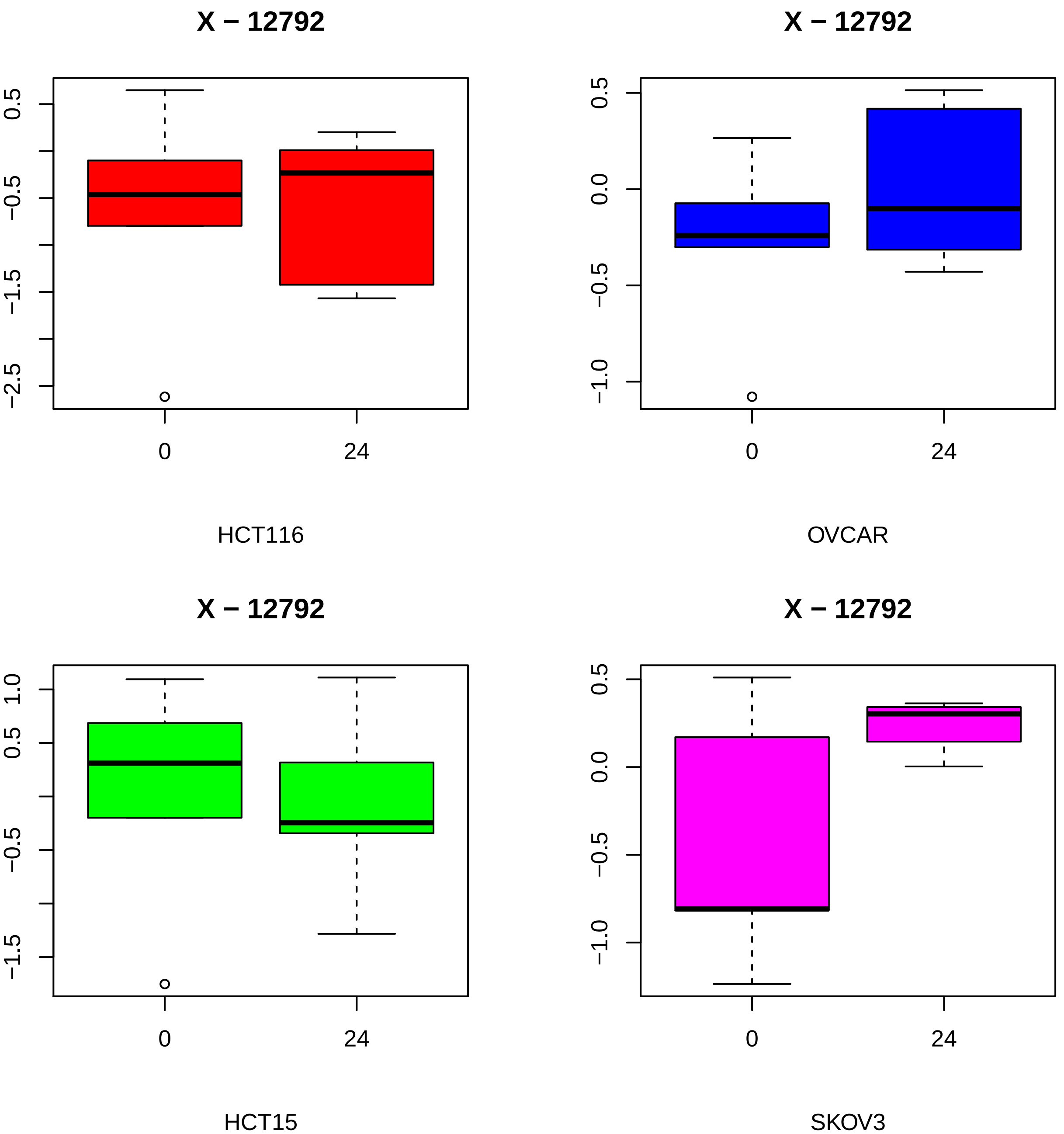
X − 12792
0.5
−0.5
−1.5
−2.5
0
24
HCT116
X − 12792
0.5
0.0
−0.5
−1.0
0
24
OVCAR
X − 12792
1.0
0.5
−0.5
−1.5
0
24
HCT15
X − 12792
0.5
0.0
−0.5
−1.0
0
24
SKOV3

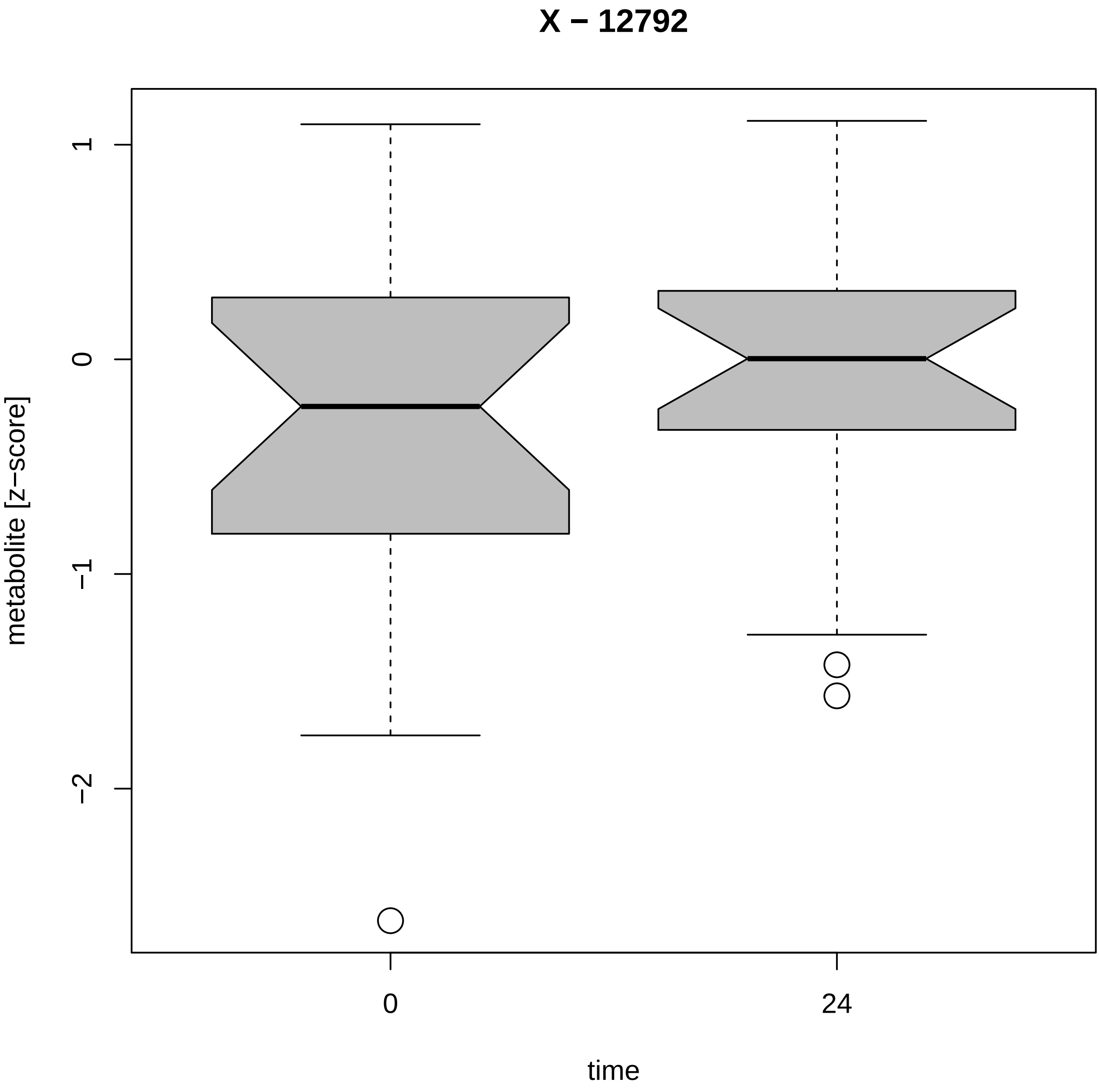

X − 12792
metabolite [z−score]
1
0
−1
−2
0
24
time

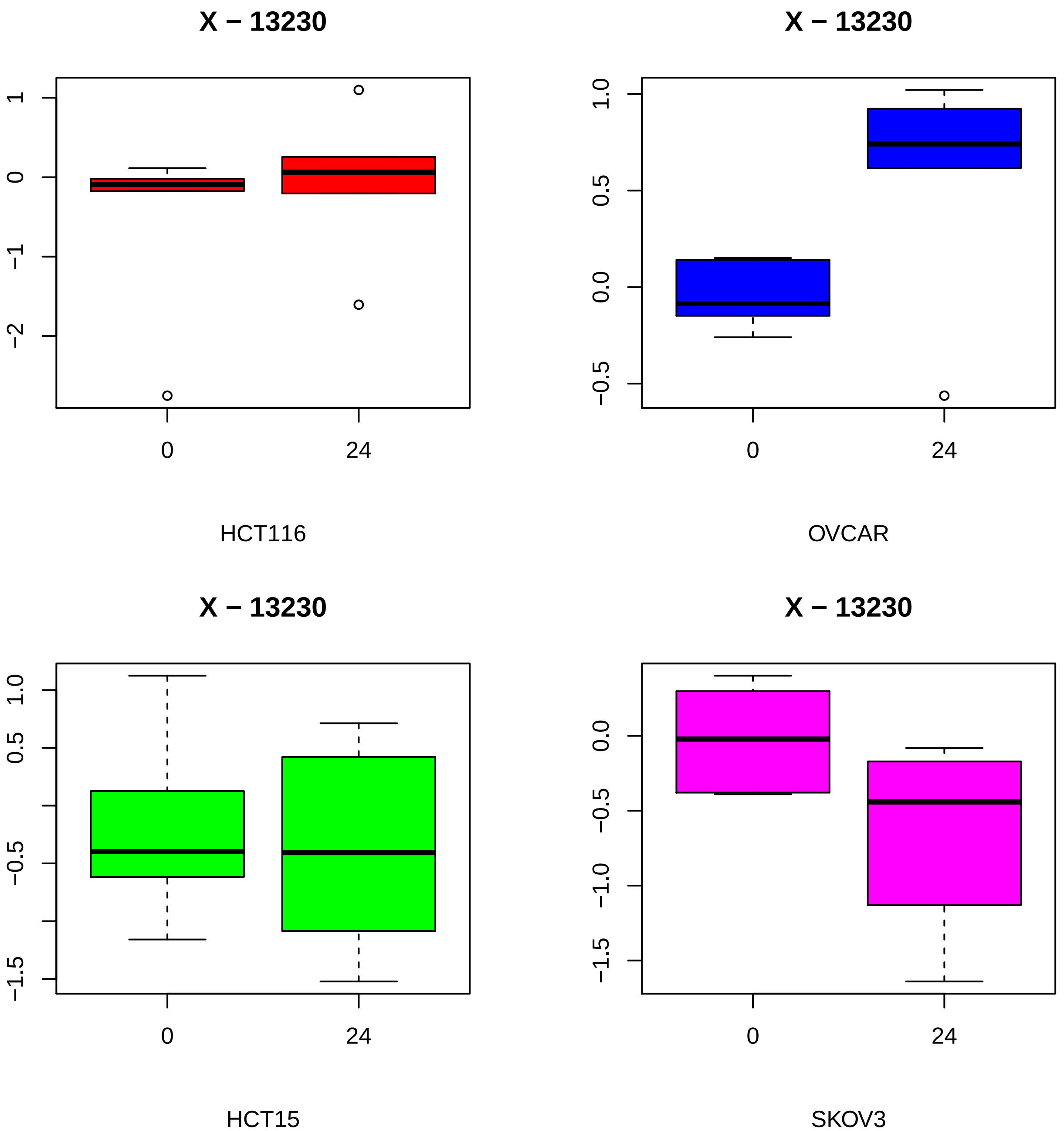
X − 13230
1
0
−1
−2
0
24
HCT116
X − 13230
1.0
0.5
0.0
−0.5
0
24
OVCAR
X − 13230
1.0
0.5
−0.5
−1.5
0
24
HCT15
X − 13230
0.0
−0.5
−1.0
−1.5
0
24
SKOV3

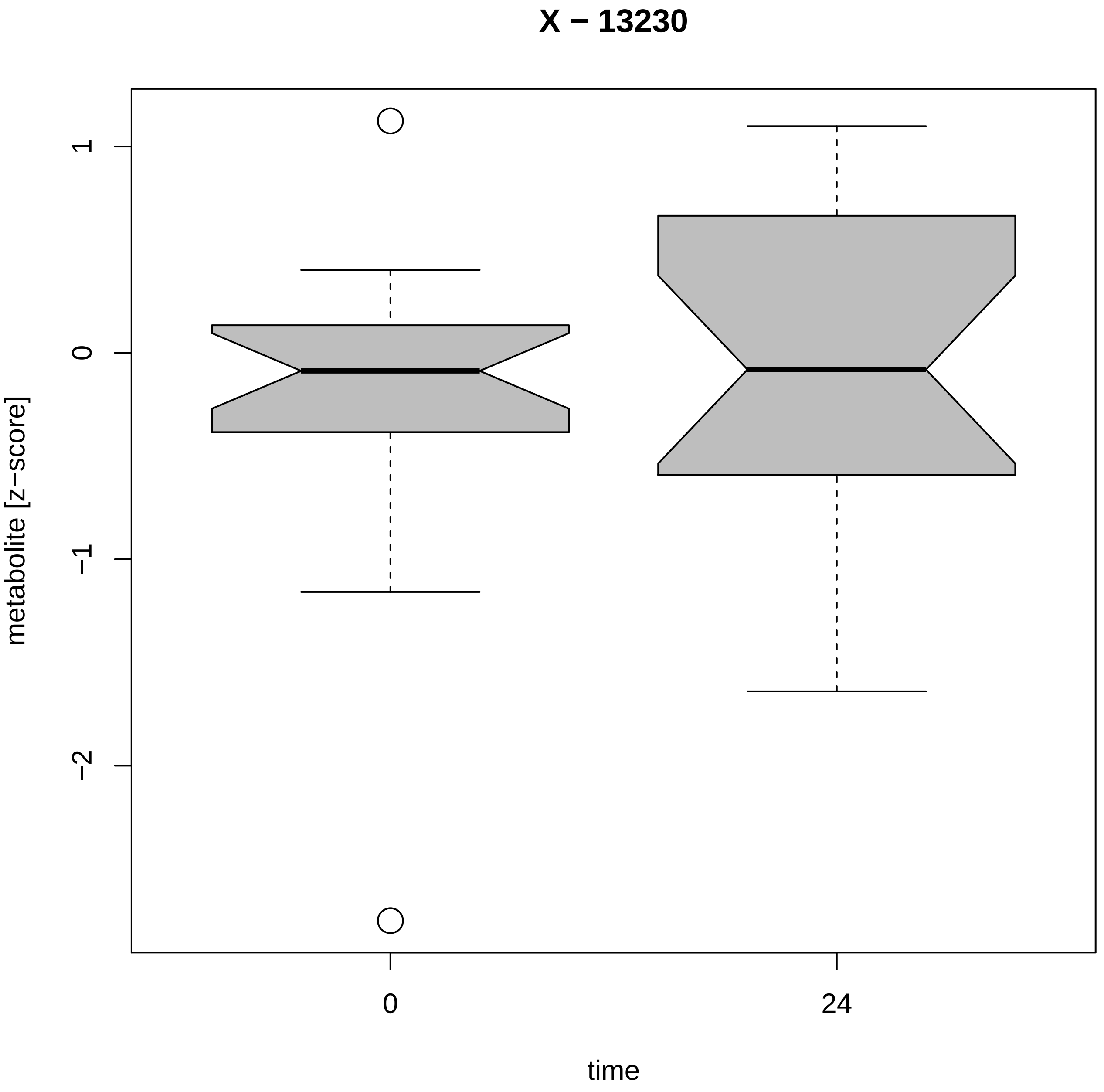

X − 13230
1
0
−1
−2
metabolite [z−score]
0
24
time

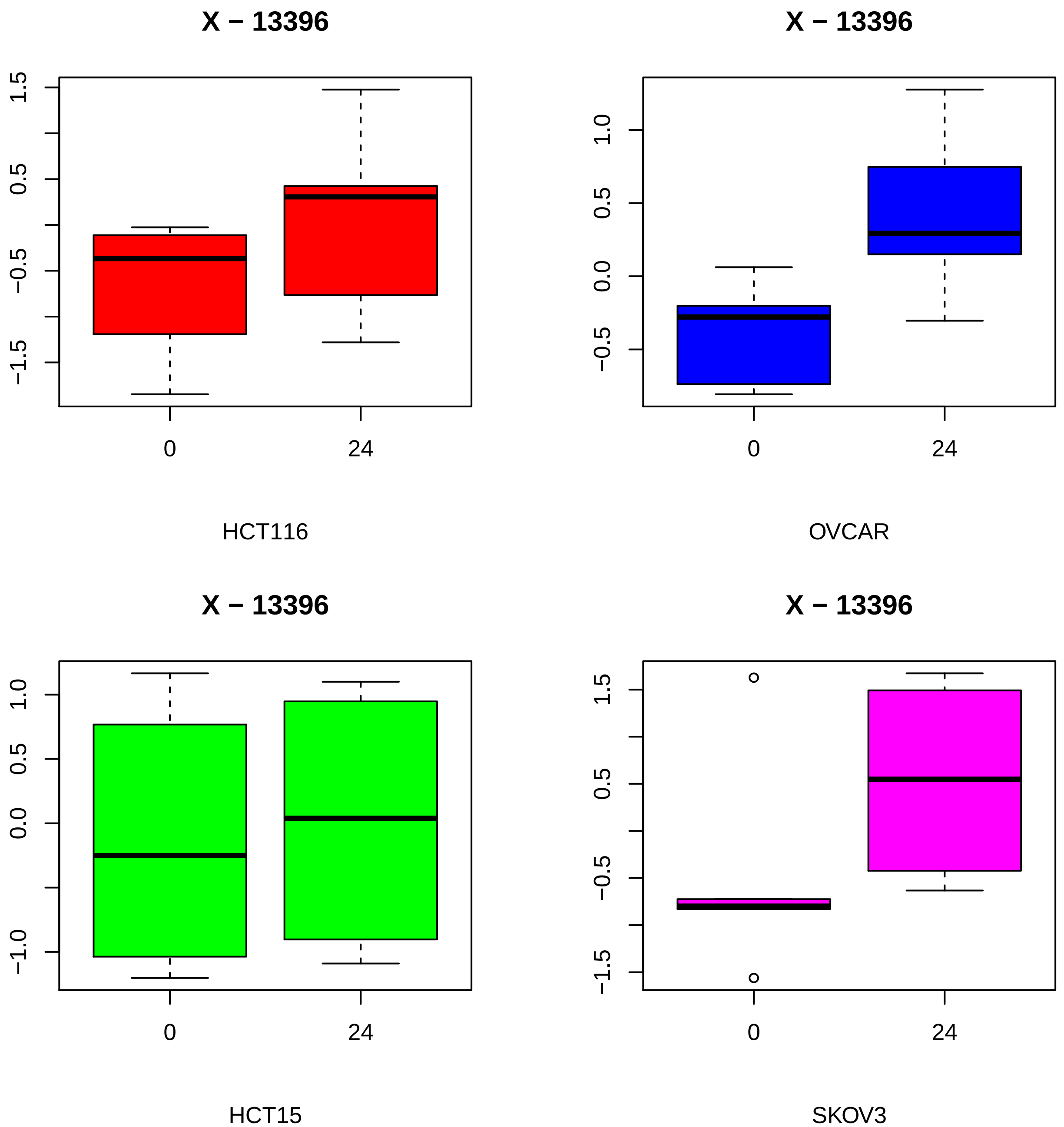
X − 13396
1.5
0.5
−0.5
−1.5
0
24
HCT116
X − 13396
1.0
0.5
0.0
−0.5
0
24
OVCAR
X − 13396
1.0
0.5
0.0
−1.0
0
24
HCT15
X − 13396
1.5
0.5
−0.5
−1.5
0
24
SKOV3

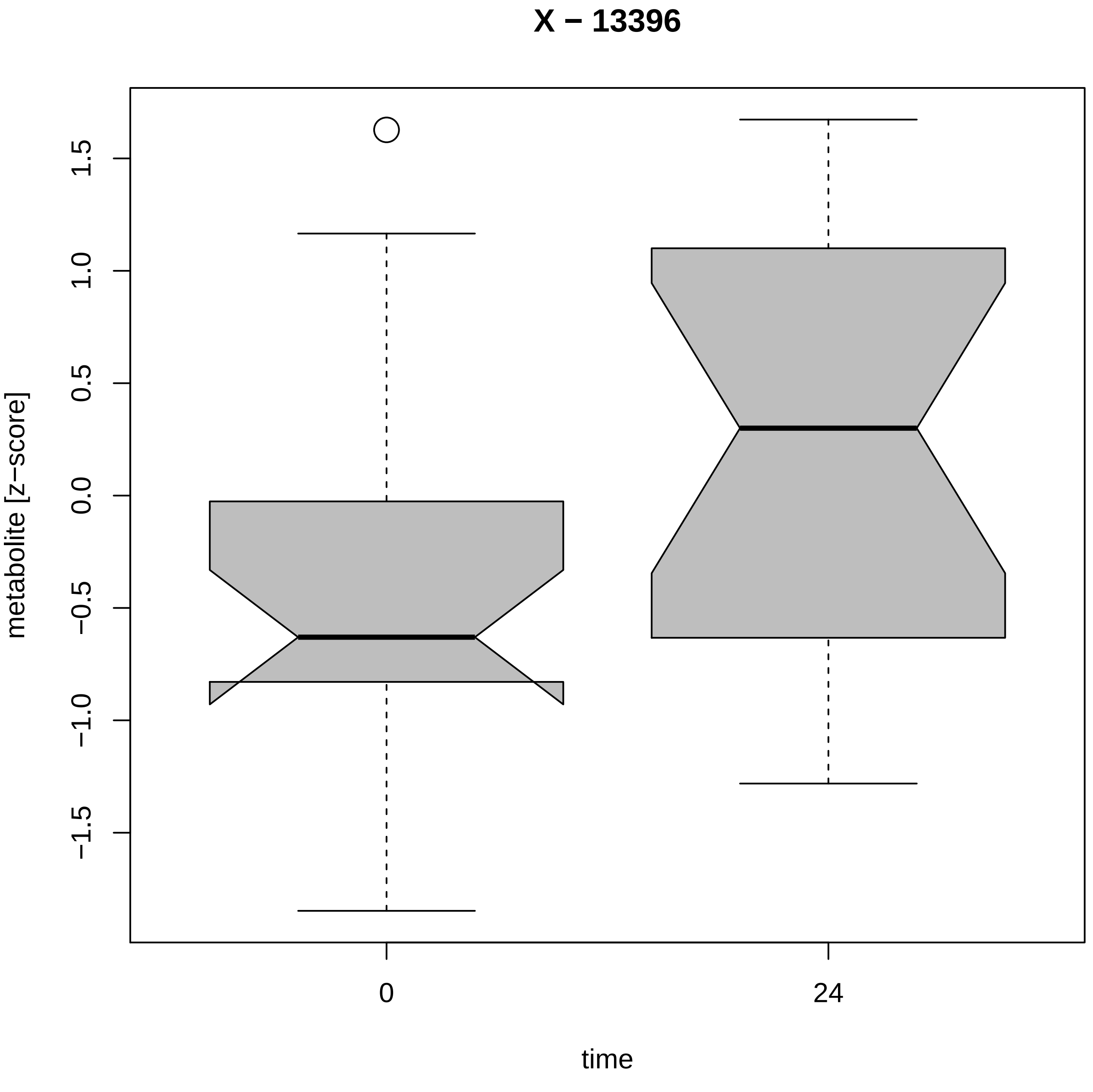
X − 13396
metabolite [z−score]
1.5
1.0
0.5
0.0
−0.5
−1.0
−1.5
0
24
time

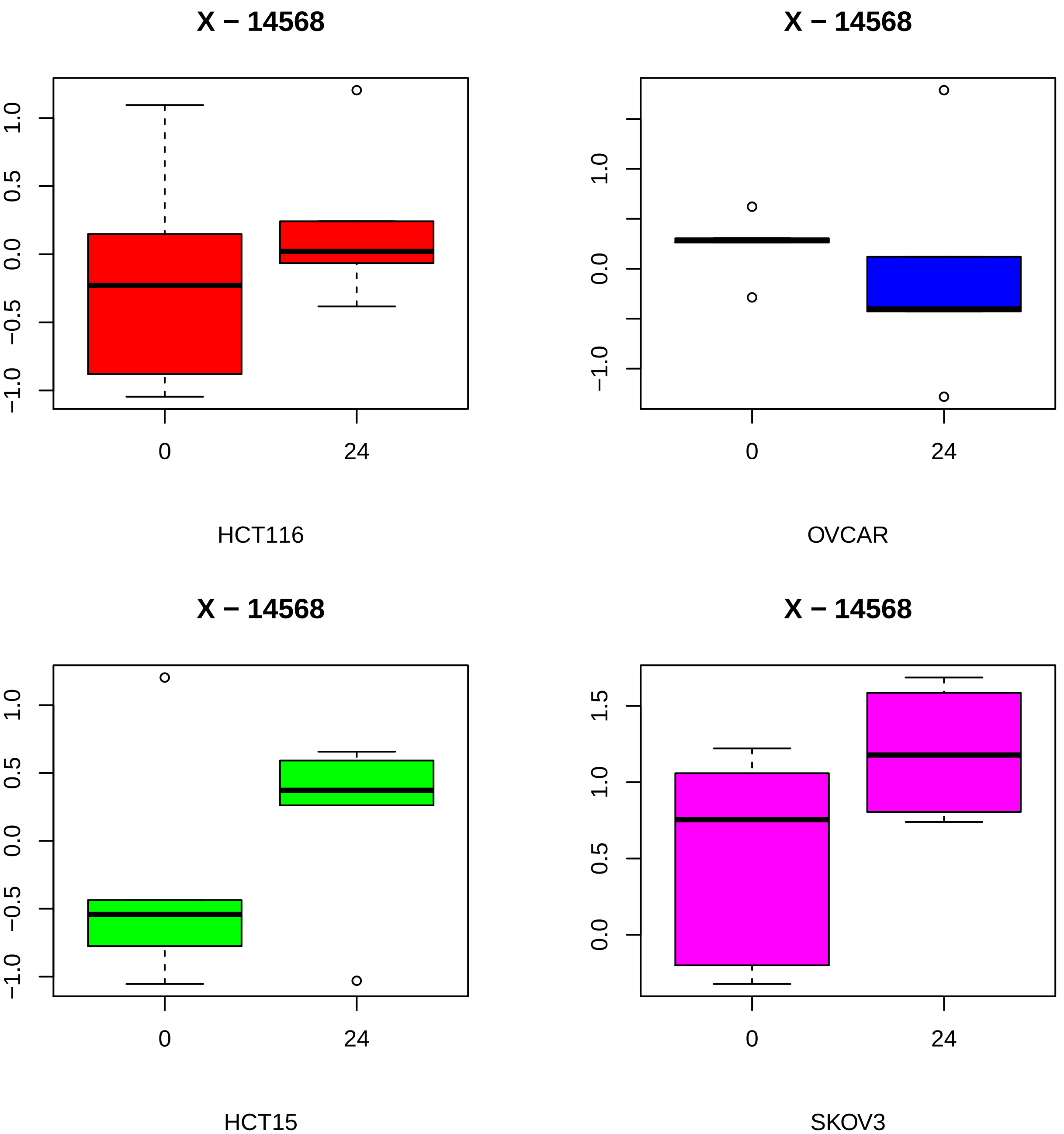

X − 14568
1.0
0.5
0.0
−0.5
−1.0
0
24
HCT116
X − 14568
1.0
0.0
−1.0
0
24
OVCAR
X − 14568
1.0
0.5
0.0
−0.5
−1.0
0
24
HCT15
X − 14568
1.5
1.0
0.5
0.0
0
24
SKOV3

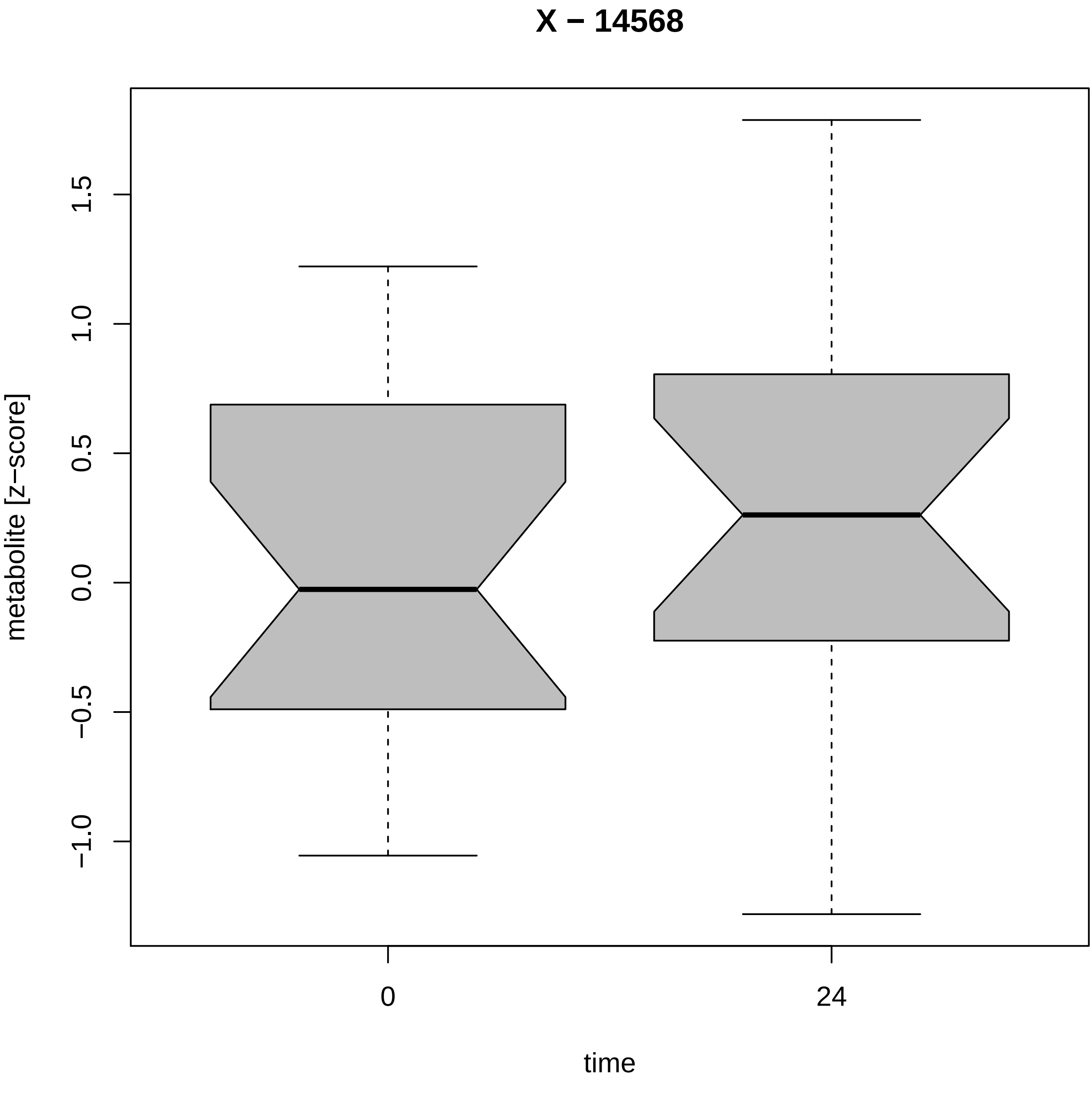

X − 14568
metabolite [z−score]
1.5
1.0
0.5
0.0
−0.5
−1.0
0
24
time

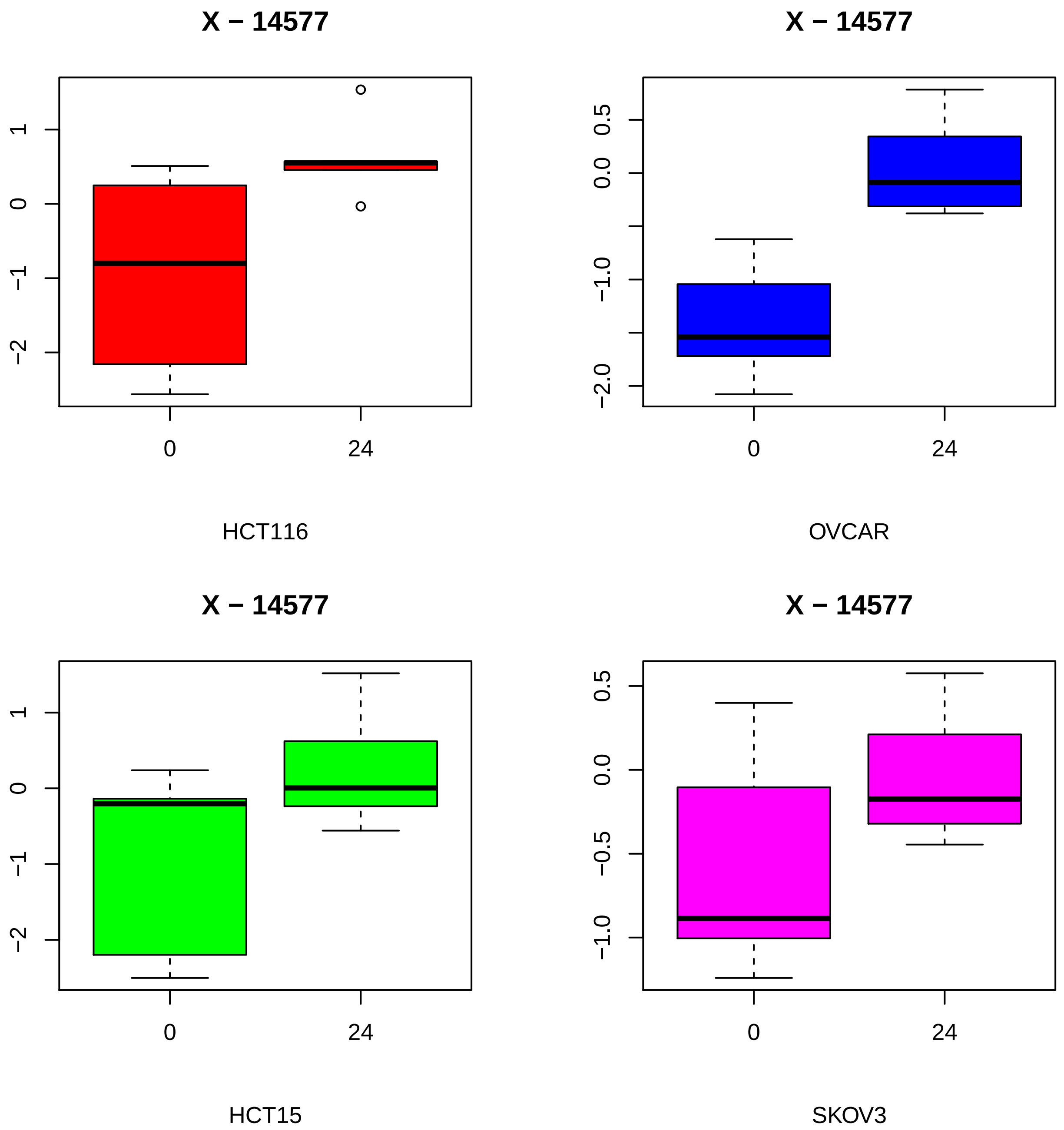
X − 14577
1
0
−1
−2
0
24
HCT116
X − 14577
0.5
0.0
−1.0
−2.0
0
24
OVCAR
X − 14577
1
0
−1
−2
0
24
HCT15
X − 14577
0.5
0.0
−0.5
−1.0
0
24
SKOV3

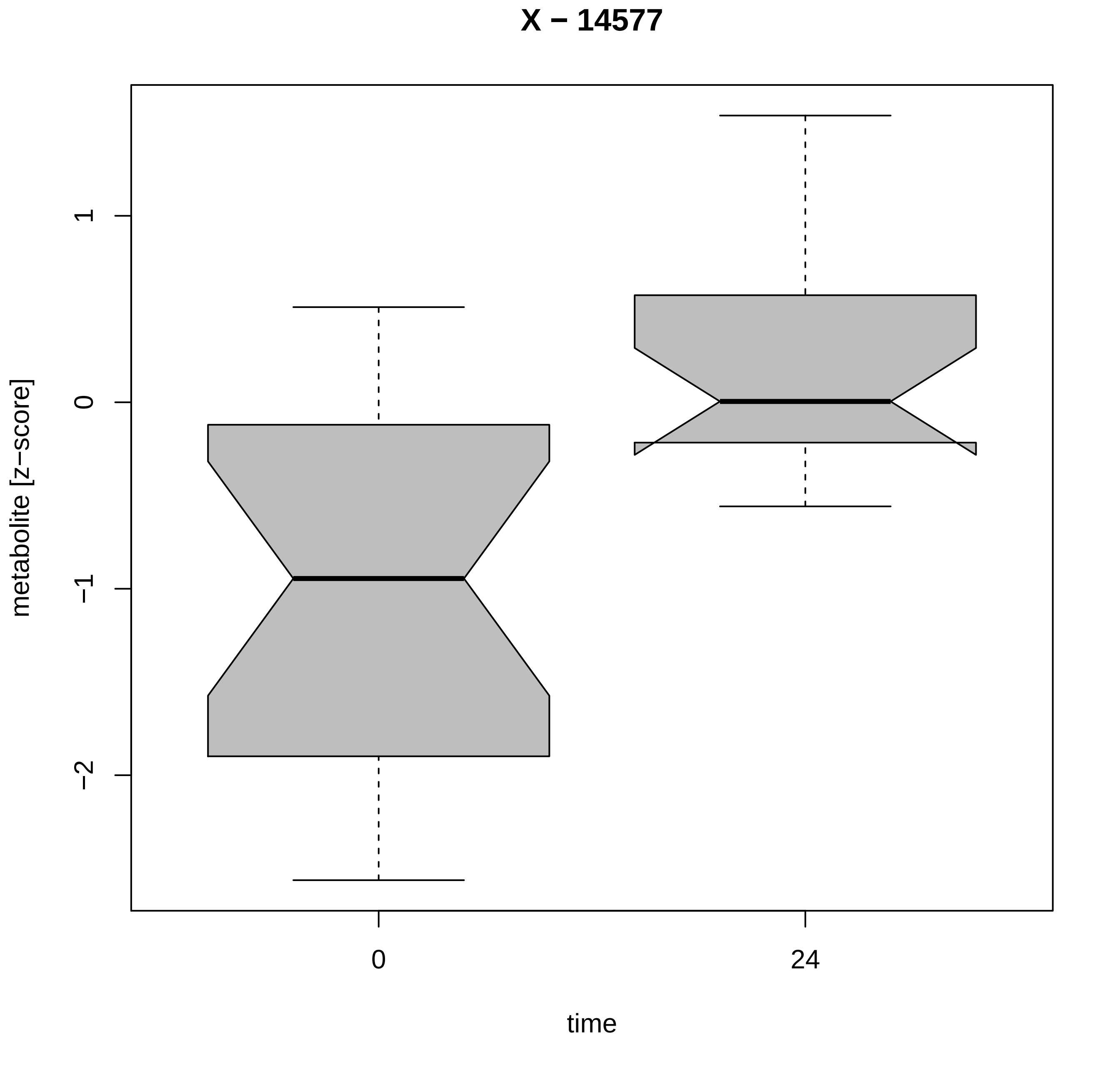

X − 14577
metabolite [z−score]
1
0
−1
−2
0
24
time

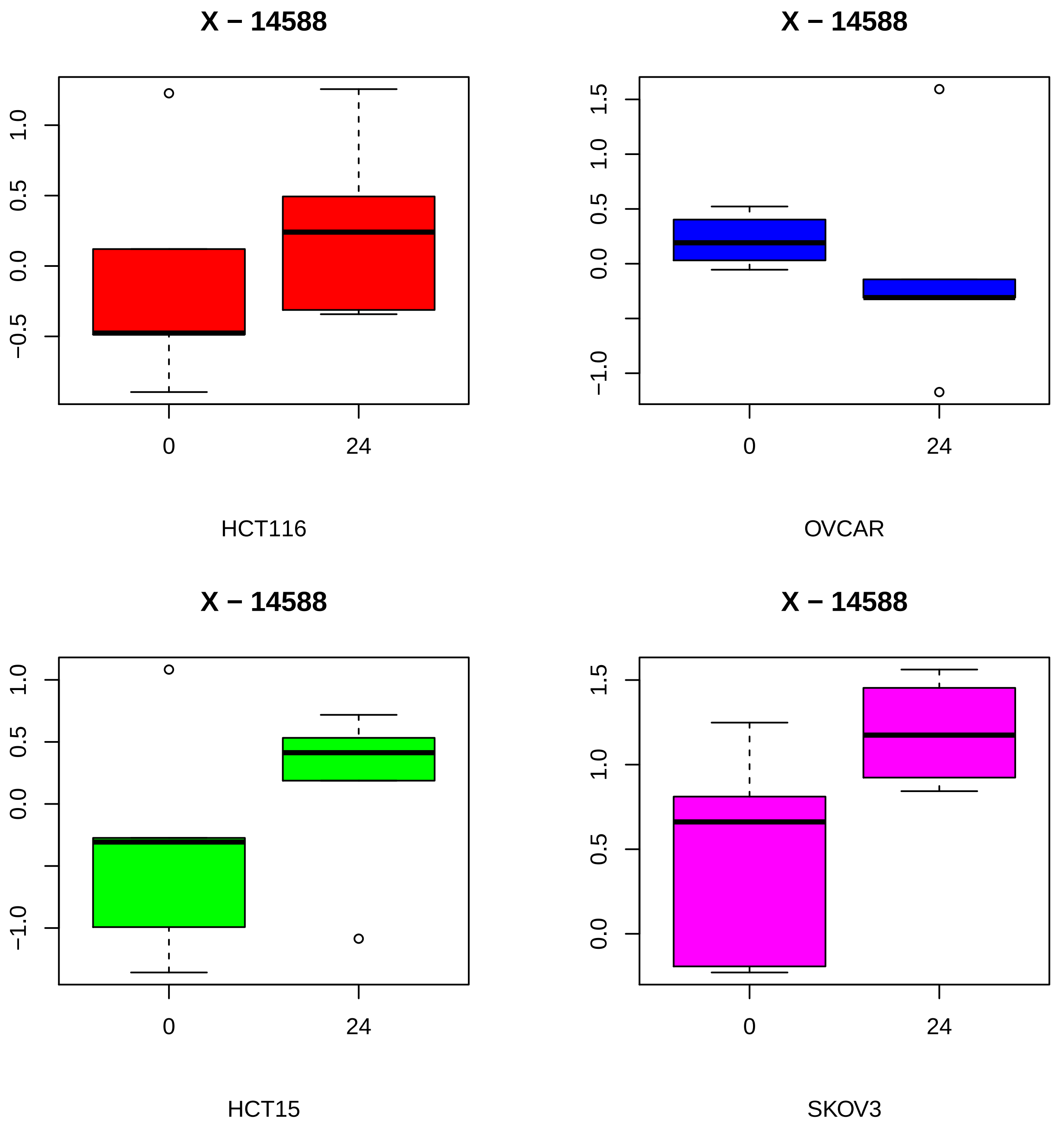
X − 14588
1.0
0.5
0.0
−0.5
0
24
HCT116
X − 14588
1.5
1.0
0.5
0.0
−1.0
0
24
OVCAR
X − 14588
1.0
0.5
0.0
−1.0
0
24
HCT15
X − 14588
1.5
1.0
0.5
0.0
0
24
SKOV3

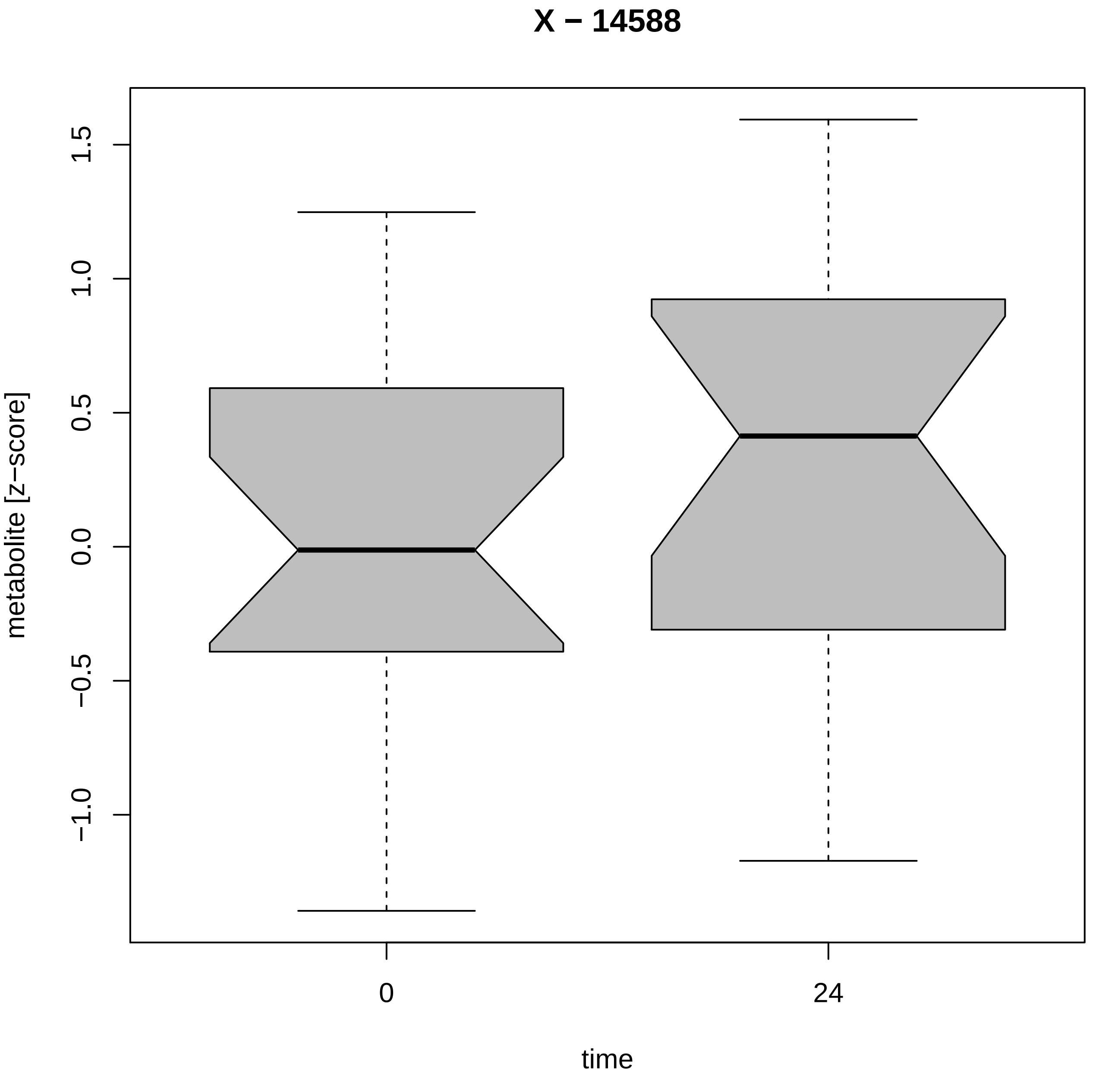

X − 14588
metabolite [z−score]
1.5
1.0
0.5
0.0
−0.5
−1.0
0
24
time

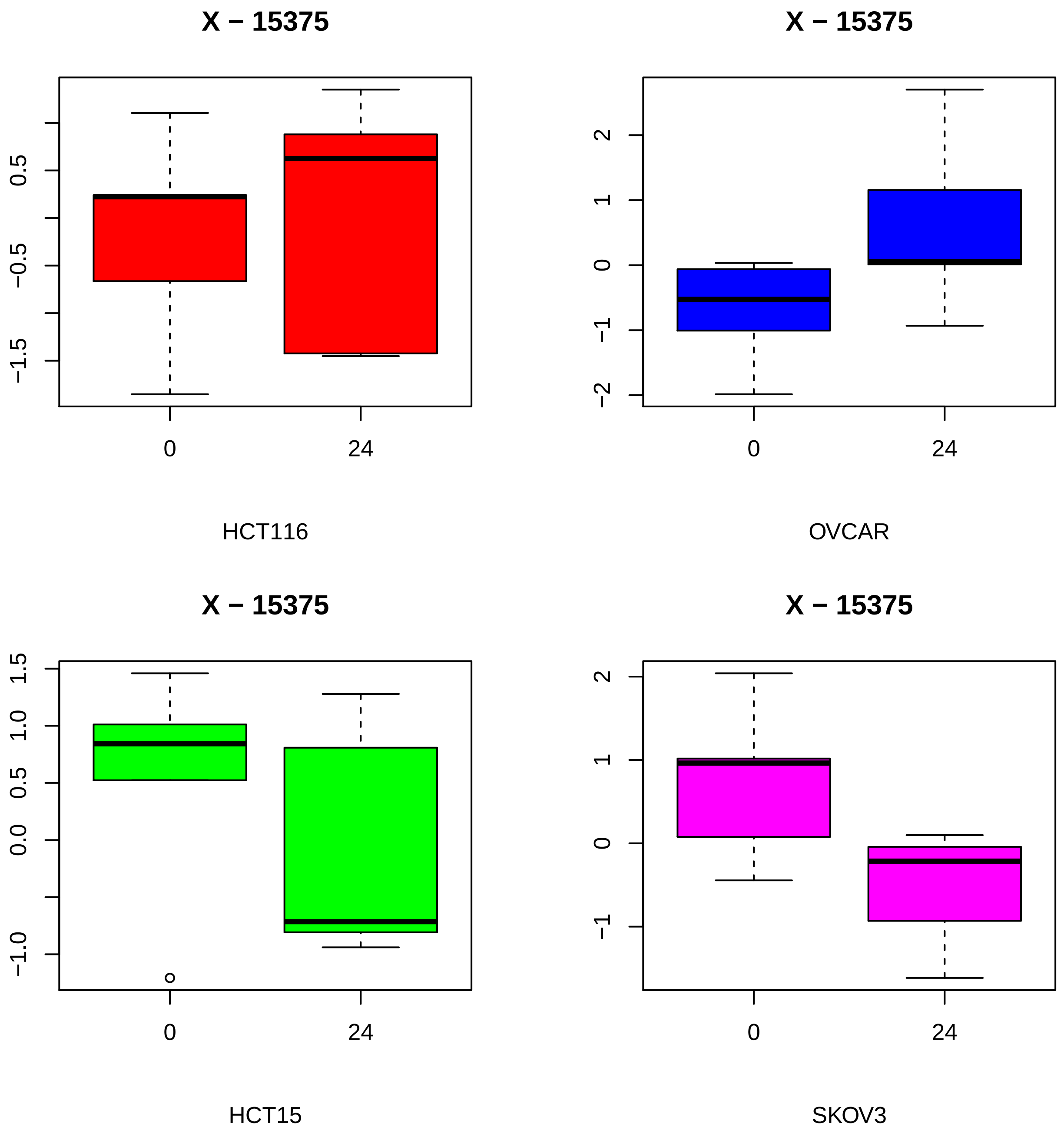

X − 15375
0.5
−0.5
−1.5
0
24
HCT116
X − 15375
2
1
0
−1
−2
0
24
OVCAR
X − 15375
1.5
1.0
0.5
0.0
−1.0
0
24
HCT15
X − 15375
2
1
0
−1
0
24
SKOV3

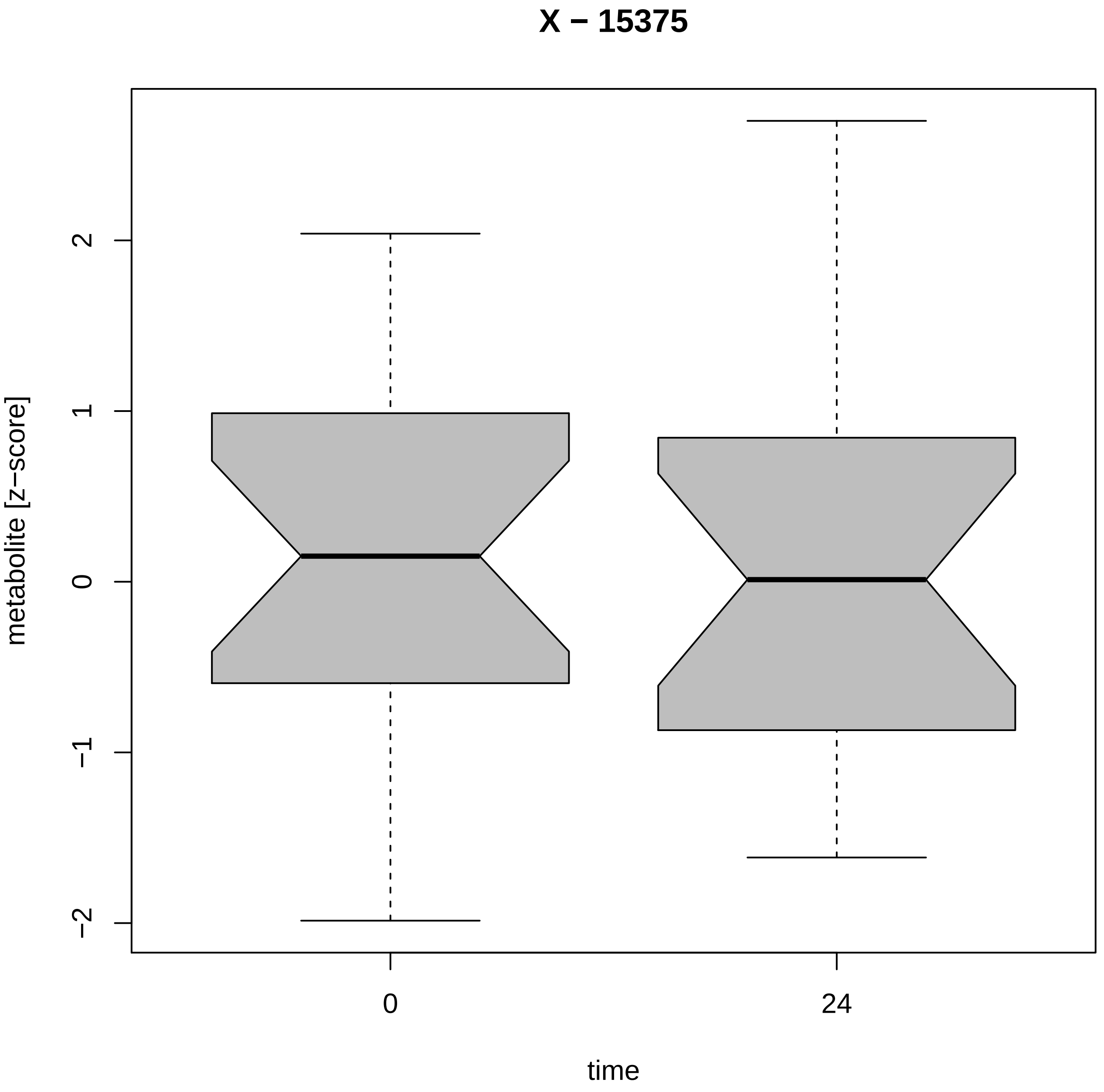

X − 15375
2
1
0
−1
−2
metabolite [z−score]
0
24
time

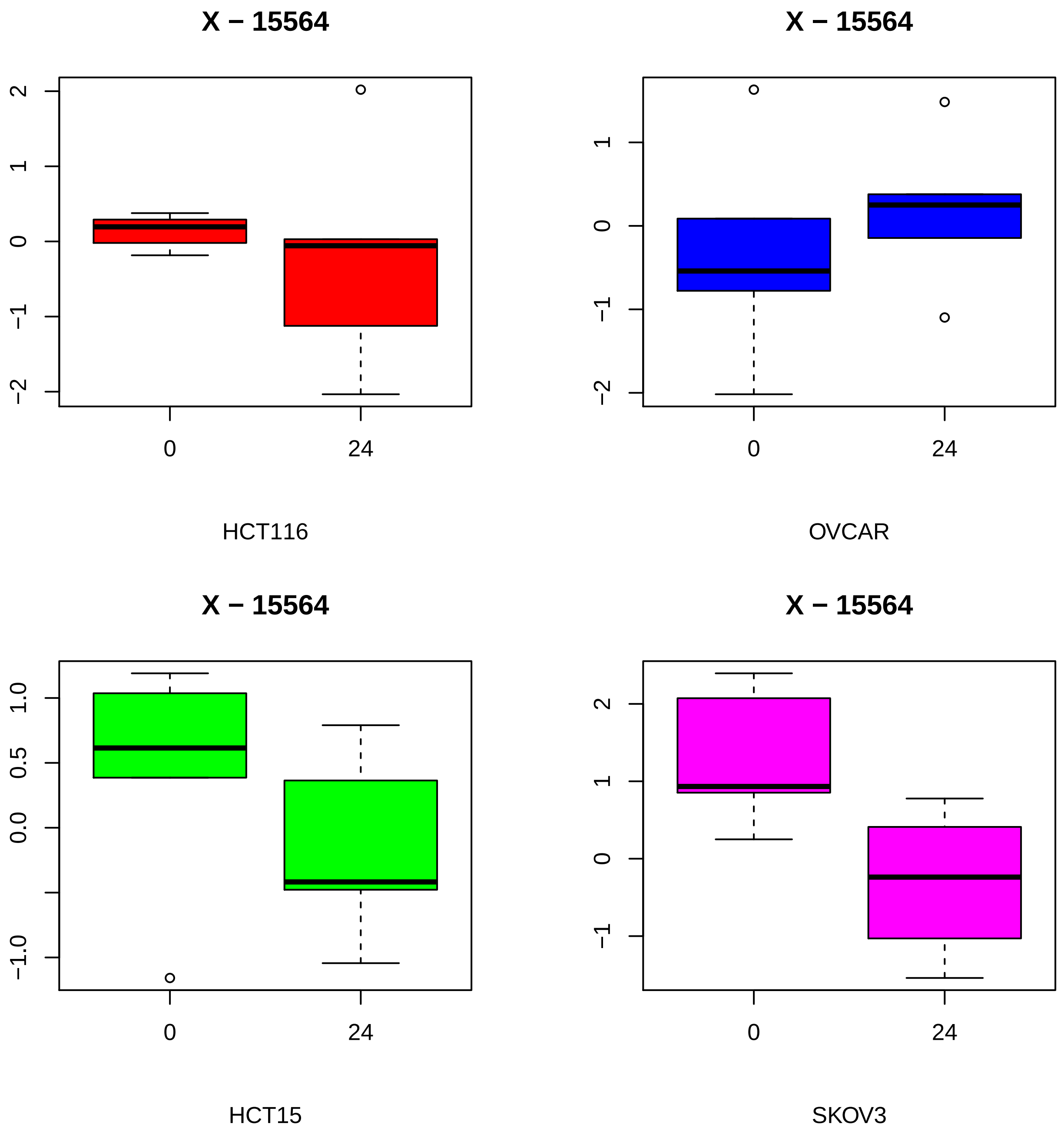

X − 15564
2
1
0
−1
−2
0
24
HCT116
X − 15564
1
0
−1
−2
0
24
OVCAR
X − 15564
1.0
0.5
0.0
−1.0
0
24
HCT15
X − 15564
2
1
0
−1
0
24
SKOV3

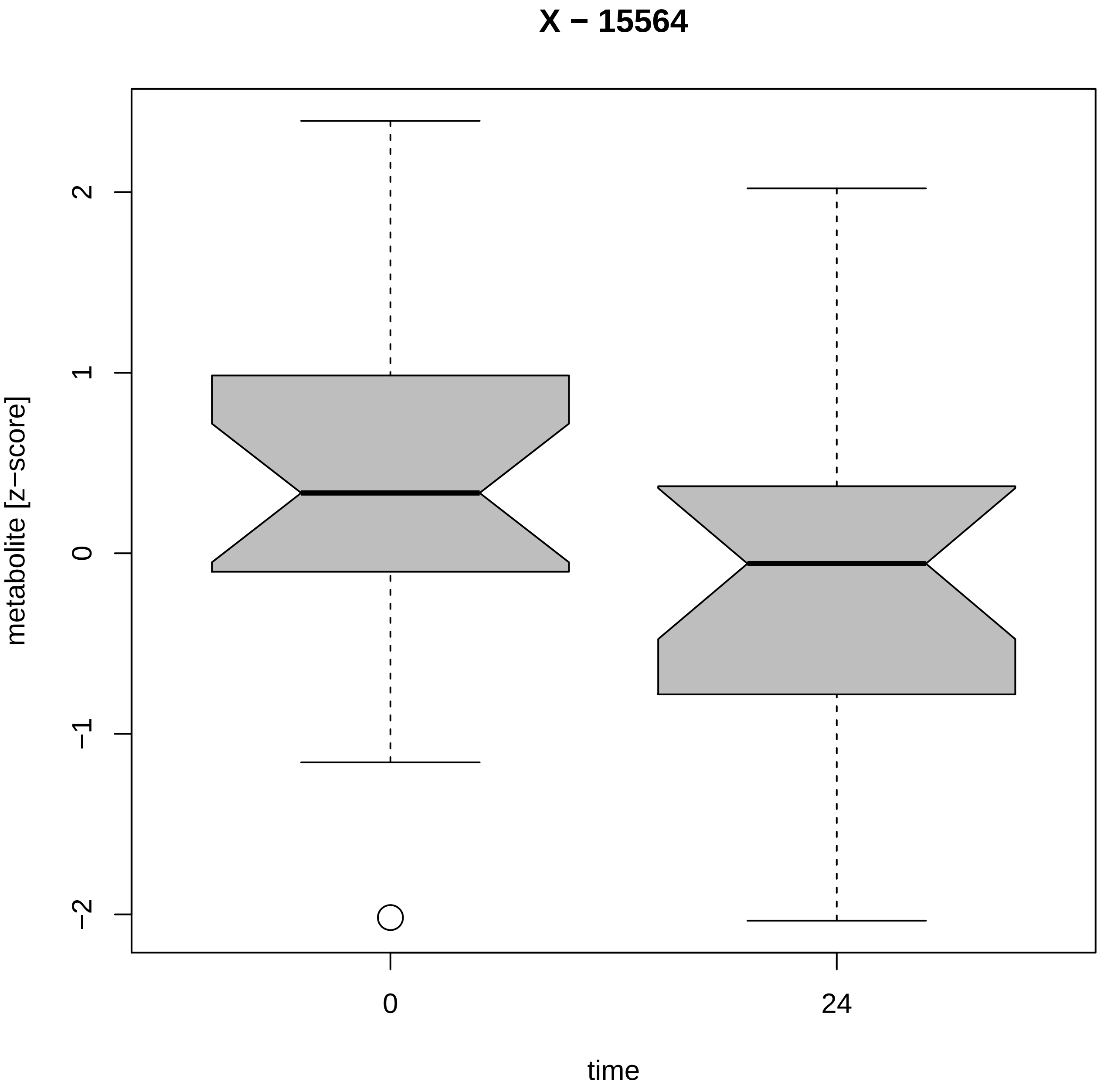

X − 15564
metabolite [z−score]
2
1
0
−1
−2
0
24
time
